# Direct cell-to-cell transfer in stressed tumor microenvironment aggravates tumorigenic or metastatic potential in pancreatic cancer

**DOI:** 10.1038/s41525-022-00333-w

**Published:** 2022-10-27

**Authors:** Giyong Jang, Jaeik Oh, Eunsung Jun, Jieun Lee, Jee Young Kwon, Jaesang Kim, Sang-Hyuk Lee, Song Cheol Kim, Sung-Yup Cho, Charles Lee

**Affiliations:** 1 Department of Life Science, Ewha Womans University, Seoul, 03760 Republic of Korea; 2 Ewha-JAX Cancer Immunotherapy Research Center, Ewha Womans University, Seoul, 03760 Republic of Korea; 3 Medical Research Center, Genomic Medicine Institute, Seoul National University College of Medicine, Seoul, 03080 Republic of Korea; 4 Department of Translational Medicine, Seoul National University College of Medicine, Seoul, 03080 Republic of Korea; 5 Department of Internal Medicine, Seoul National University Hospital, Seoul, 03080 Republic of Korea; 6 Department of Surgery, Asan Medical Center, University of Ulsan College of Medicine, Seoul, 05505 Republic of Korea; 7 Asan Medical Institute of Convergence Science and Technology (AMIST), Asan Medical Center, University of Ulsan College of Medicine, Seoul, 05505 Republic of Korea; 8 Department of Convergence Medicine, Asan Institute for Life Sciences, University of Ulsan College of Medicine and Asan Medical Center, Seoul, 05505 Republic of Korea; 9 Department of Surgery, Seoul National University Bundang Hospital, Gyeonggi-do, 13620 Republic of Korea; 10 The Jackson Laboratory for Genomic Medicine, Farmington, CT 06032 USA; 11 Department of Bio-Information Science, Ewha Womans University, Seoul, 03760 Republic of Korea; 12 Department of Biomedical Sciences, Seoul National University College of Medicine, Seoul, 03080 Republic of Korea; 13 Cancer Research Institute, Seoul National University, Seoul, 03080 Republic of Korea

**Keywords:** Cancer microenvironment, Metastasis

## Abstract

Pancreatic cancer exhibits a characteristic tumor microenvironment (TME) due to enhanced fibrosis and hypoxia and is particularly resistant to conventional chemotherapy. However, the molecular mechanisms underlying TME-associated treatment resistance in pancreatic cancer are not fully understood. Here, we developed an in vitro TME mimic system comprising pancreatic cancer cells, fibroblasts and immune cells, and a stress condition, including hypoxia and gemcitabine. Cells with high viability under stress showed evidence of increased direct cell-to-cell transfer of biomolecules. The resulting derivative cells (*CD44*^high^/*SLC16A1*^high^) were similar to cancer stem cell-like-cells (CSCs) with enhanced anchorage-independent growth or invasiveness and acquired metabolic reprogramming. Furthermore, CD24 was a determinant for transition between the tumorsphere formation or invasive properties. Pancreatic cancer patients with *CD44*^low^/*SLC16A1*^low^ expression exhibited better prognoses compared to other groups. Our results suggest that crosstalk via direct cell-to-cell transfer of cellular components foster chemotherapy-induced tumor evolution and that targeting of CD44 and MCT1(encoded by *SLC16A1*) may be useful strategy to prevent recurrence of gemcitabine-exposed pancreatic cancers.

## Introduction

Development of resistance to chemotherapy is a major hurdle for curative cancer treatment. Cancer cells show genetic heterogeneity due to genomic instability, and chemotherapy confers selective pressures on a subset of cancer cells, driving them toward more aggressive phenotypes^1–3^. Accumulating evidence indicates that responsiveness to chemotherapy is impacted not only by tumor-intrinsic factors but also by the TME^4–6^. Cells in the TME, including stromal cells, affect the tumorigenic and metastatic potential of cancer cells^7^. Intercellular communication, through secretory factors and receptor-ligand interactions, is one suggested mechanism by which these TME cells alter tumor cells to create subpopulations with survival advantages^8,9^. However, the exact mechanisms by which the TME induces resistance during chemotherapy have not been fully elucidated.

Direct cell-to-cell transfer of cellular components via cell fusion, exosomes, and tunneling nanotubes (TNTs) have been suggested as other ways of intercellular communication^10–13^. Direct cell-to-cell transfer of cellular components including proteins, DNA, mRNA, microRNA (miRNA), long noncoding RNA (lncRNA), and organelles, plays a crucial role in regulating the tumorigenic and metastatic potential of cancer cells in the TME^10–12^. For example, transfer of miRNA between a tumor and the surrounding cells via exosomes or TNTs induces reprogramming of gene expression in cancer cells, resulting in aggravation of metastatic activity^11,14^. Together, these findings indicate that an understanding of cancer evolution during chemotherapy requires consideration of the effects of direct cell-to-cell transfer.

Most cases of pancreatic cancer, one of the most aggressive tumor types, are diagnosed late, leaving few therapeutic options available to the patient^15,16^. Pancreatic cancer cells in the TME are surrounded by stromal cells (such as fibroblasts and macrophages), and cross-talk among these components is critical for the tumorigenic and metastatic potential of cancer cells^7^. In addition, deposition of abundant extracellular matrix from stromal cells results in persistent and severe hypoxia within the pancreatic cancer TME^17^, and both hypoxia and anticancer chemotherapy are associated with alteration of the TME that may in turn contribute to a poor therapeutic response^18,19^. Together, these studies indicate that investigation of the response of pancreatic cancer cells to anticancer chemotherapy requires investigation of the impact of both TME cells and hypoxia on cancer cells. In the present study, we examined the response of cancer cells to gemcitabine, one of the standard chemotherapeutic agents for pancreatic cancer^20^.

Here, we investigated the possible interplay between pancreatic tumor cells, stromal cells, and an anticancer chemotherapy, i.e., gemcitabine, by developing and implementing an in vitro TME mimic model system by incorporating cells (fibroblasts, macrophages, pancreatic cancer cells) of the TME, hypoxia or non-hypoxia and gemcitabine. Our data suggest that gemcitabine-induced direct cell-to-cell transfer in TME is strongly associated with tumor heterogeneity through enhancing tumorigenic or metastatic potential and inducing metabolic reprogramming of pancreatic cancer cells.

## Results

### Direct cell-to-cell transfer from macrophages to pancreatic cancer cells in a gemcitabine-treated TME mimic condition

**Fig. 1:**
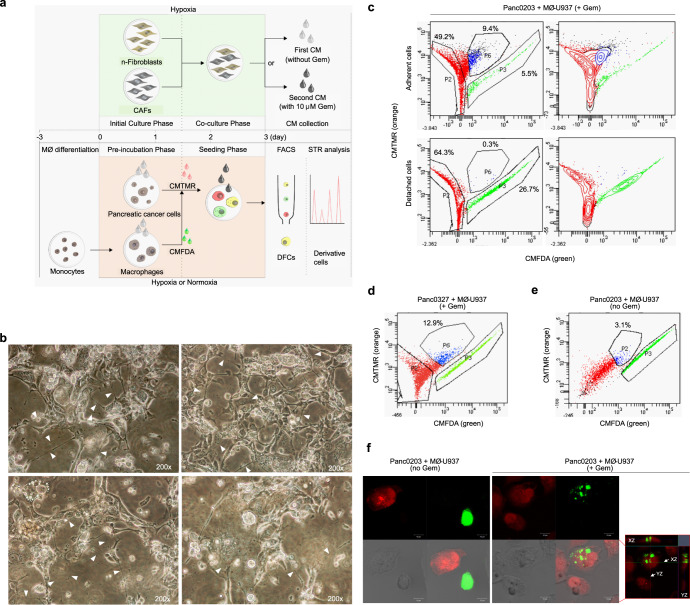
In vitro TME model development and isolation of pancreatic cancer cells exhibiting direct cell-to-cell transfer. **a** Schematic of the TME mimic model showing steps for collecting conditioned media (CM) from human pancreatic normal fibroblasts (n-fibroblasts) co-cultured with human pancreatic cancer-associated fibroblasts (CAFs) under hypoxic only or hypoxic/gemcitabine (gem)-induced stress conditions (upper panels); and for co-culturing pancreatic cancer cells with macrophages to isolate cells exhibiting direct cell-to-cell transfer (double fluorescent cells; DFCs) and to generate derivative cells. MØ: macrophage, CMTMR: 5-(and-6)-(((4-chloromethyl)benzoyl)amino)tetramethylrhodamine, CMFDA 5-chloromethylfluorescein diacetate, FACS fluorescence-activated cell sorting, STR short tandem repeat. **b** Morphological changes of total cultured cells with increased numbers of vacuoles, enlarged cytoplasm, and multiple TNTs at the end of the Seeding phase, prior to collection of the cells for FACS. Arrowheads denote TNTs. **c** Percentage of isolated Panc0203^CMTMR^ (red), MØ-U937^CMFDA^ (green), and DFCs (blue) detected via FACS following preparation of adherent and detached cells from total cultured cells. Detached cells represent dying cells. **d**, **e** Percentage of adherent double fluorescent dye-positive cells (DFCs) for FACS gating in the (**d**) gemcitabine-treated TME mimic model or (**e**) normal co-culture TME condition. The percentages in the graphs indicate the percentage of adherent DFCs. +Gem; gemcitabine-treated TME mimic model, no Gem; normal co-culture TME condition. **f** Confocal images of single-fluorescent dye-positive cells (SFCs) and DFCs (red box, a merged image of entire z-stack with orthogonal views in the same field). Green SFCs and red SFCs in the confocal images indicate MØ-U937^CMFDA^ and Panc0203^CMTMR^, respectively (scale bar, 10 μm).

To reconstruct the compositional heterogeneity of the TME during gemcitabine treatment in vitro, we developed a TME mimic condition (gemcitabine-treated TME model) that simplifies in vivo contexts (Fig. 1a). First, to enable determination of the effect of soluble factors from stromal fibroblasts on the interplay between pancreatic cancer cells and macrophages, we obtained conditioned media (CM) from normal fibroblasts co-cultured with cancer-associated fibroblasts (CAFs) in the presence of either hypoxia alone, i.e., first CM or hypoxia with gemcitabine, i.e., second CM. After the Pre-incubation phase in the presence of the first CM treatment, pancreatic cell lines (Panc0203 or Panc0327; labeled in red with the fluorescent dye CMTMR) were co-incubated with macrophages differentiated from U937 cells (MØ-U937; labeled in green with the fluorescent dye CMFDA) in hypoxic (for Panc0203^CMTMR^ + MØ-U937^CMFDA^) or normoxic (for Panc0327^CMTMR^ + MØ-U937^CMFDA^) conditions in the presence of the second CM. We used the normoxic condition for Panc0327 cells because they are highly susceptible to hypoxia.

**Fig. 2:**
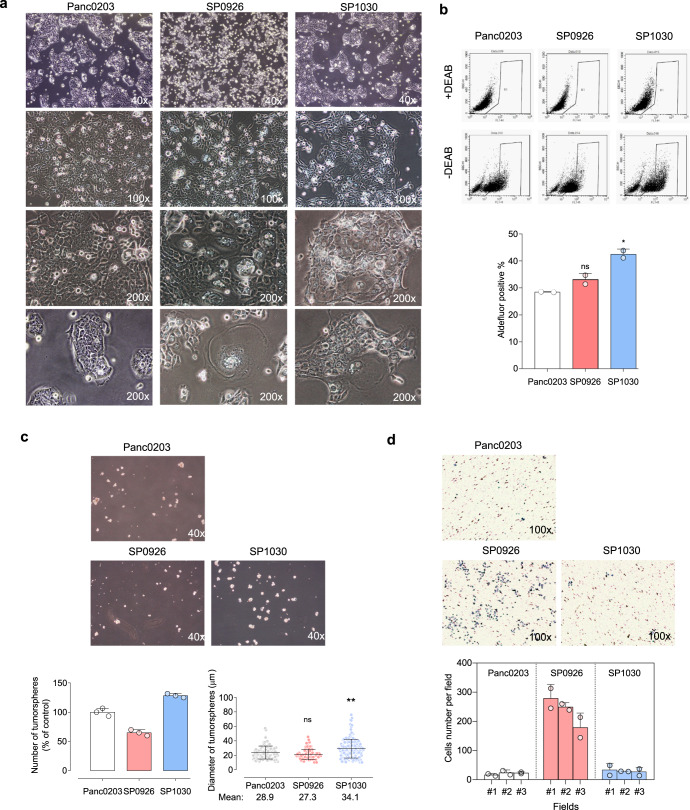
Enhanced anchorage-independent growth or invasive activity of pancreatic derivative cells from direct cell-to-cell transfer. **a** Representative morphologic and spatial patterns of Panc0203 cells and derivative cells. Representative images of confluent SP0926 and SP1030 cells show increased numbers of vacuoles or enlarged cytoplasm. Phase images of less confluent cells show a more dispersed growth pattern of SP0926 cells compared to that of SP1030 and maternal Panc0203 cells. **b** Basal levels of aldehyde dehydrogenase (ALDH) activity of the derivative cells compared to Panc0203 cells. DEAB: *N*,*N*-diethylaminobenzaldehyde. **c** Significantly different tumorsphere formation activities in the SP0926 and SP1030 clones. Spheroids were counted on day 7 after seeding. Upper panel shows representative images of tumorspheres, and lower panel shows relative number of tumorspheres compared to the Panc0203 cell group (left) and diameter of tumorspheres (right). **d** Increased invasion activity of SP0926 cells compared to the parent Panc0203 cell line. An invasion chamber assay system containing Matrigel was performed for 24 h. The initial seeding number of cells for each group was 1 × 10^5^. Upper chamber: serum-free medium, bottom chamber: normal culture medium. *P ≤ 0.05; **P ≤ 0.01; ***P ≤ 0.001. One-way ANOVA followed by Tukey’s multiple comparison test. Data are presented as the mean values ± SD.

Several cells that survived at the end of the Seeding phase in the gemcitabine-treated TME (stressed-TME; s-TME) model demonstrated increased numbers of vacuoles or enlarged cytoplasm (Fig. 1b), both of which were possibly associated with increased production of exosomes^21^. In addition, we detected a considerable number of cells exhibiting TNTs (Fig. 1b), which are involved in one of the mechanisms through which direct cell-to-cell transfer of cellular components takes place. To collect derivative cells resulting from direct cell-to-cell transfer between macrophages and pancreatic cancer cells, fluorescence-activated cell sorting (FACS) was performed after establishing a sorting area for CMFDA/CMTMR double-fluorescence positive cells (DFCs; Supplementary Fig. 1a). Most DFCs were grouped as a lateral population spanned out from the main population area of adherent CMTMR-positive pancreatic cancer cells toward that of adherent MØ-U937^CMFDA^ cells (Fig. 1c, d). DFCs comprised 9.4% and 12.9% of total adherent cells from Panc0203 and Panc0327 cells, respectively (Fig. 1c, d). In detached cells, which represent dying cells, only 0.3% of total cells showed double positivity (Fig. 1c). This result suggests that the survivability of DFCs was greater than that of single-fluorescent pancreatic cancer cells and macrophages. In contrast, a lower percentage of DFCs (3.1%) was detected after simple co-culture of Panc0203^CMTMR^ cells with MØ-U937^CMFDA^ cells under the normal co-culture TME condition without gemcitabine (Fig. 1e), compared to the percentage under the s-TME model (Fig. 1c, d). The enlarged cytoplasm with vacuoles and TNT-formed morphologies were maintained after seeding the DFCs (Supplementary Fig. 1b, c) and was observed as a mosaic pattern even after cell line generation (Fig. 2a and Supplementary Video 1). Most DFCs exhibited densely embedded spots of green fluorescent dye acquired from MØ-U937^CMFDA^ in CMTMR-positive pancreatic cancer cells (Fig. 1f and Supplementary Video 2), indicating that, under these conditions, pancreatic cancer cells acquired cellular components of macrophages by direct cell-to-cell transfer.

To investigate the roles of exosomes and TNTs in direct cell-to-cell transfer of biomolecules, we applied the exosome inhibitor (GW4869) and TNT inhibitors (ML141 and cytochalasin B) in the stressed tumor microenvironment (s-TME) condition^22–24^. Treatment of the exosome inhibitor reduced the number of cells with vacuoles and enlarged cytoplasm from 29.4 cells/field to 12.8 cells/field (which was statistically significant with a p-value of 0.014) compared to the vehicle treatment (DMSO) only (Supplementary Fig. 1d). When exosome or TNT-inhibitor-exposed adherent cells were subjected to flow cytometry analysis, the percentage of Panc0203^CMTMR+CMFDA^ double-fluorescent dye-positive cells (DFCs; which are indicative of cells that are Panc0203^CMTMR^ cells that uptake CMFDA fluorescent dye of MØ-U937^CMFDA^) decreased in cells treated with the exosome or TNT inhibitors (18.2% for GW4869, 8.1% for ML141, and 7.3% for cytochalasin B) compared to vehicle-treated cells (37.2% for DMSO; Supplementary Fig. 1e). MØ-U937^CMFDA^ single-fluorescent dye-positive cells (SFCs; which are indicative of MØ-U937^CMFDA^ cells that are stained by CMFDA only without uptake CMTMR fluorescent dye of Panc0203^CMTMR^) accounted for 43.3–63.6% of adherent cells in exosome or TNT inhibitor-treated s-TME (Supplementary Fig. 1f), indicating that the decrease of DFCs is not due to a toxic effect of the inhibitors and suggesting that the macrophages do indeed work as a biomolecule donor to the pancreatic cancer cells. Taken together, these data are consistent with our original suggestion that direct cell-to-cell transfer via exosomes or TNTs do play a role in the generation of DFCs.

Next, we investigated the direct effects of exosomes derived from macrophages subjected to the s-TME. Exosomes were purified from the vehicle (DMSO) or exosome inhibitor (GW4869)-treated conditioned media at the end of the Seeding phase of the s-TME model (Fig. 1a and Supplementary Fig. 1f), in which green fluorescent-dye-labeled exosomes were derived from MØ-U937^CMFDA^ cells (Supplementary Fig. 1f). The numbers and intensity of green fluorescence of the purified exosomes were significantly decreased in the exosome-inhibited (GW4869-treated) s-TME cells (blue line, s-TME CMFDA/GW4869-Exo; 6.56 × 10^8^ particles/ml and 31.68 RFU at 517 nm) compared to the vehicle (DMSO-treated) s-TME cells (red line, s-TME CMFDA/DMSO-Exo; 1.48 × 10^9^ particles/ml and 66.82 RFU at 517 nm; Supplementary Fig. 1f, g) showing the effectiveness of the exosome inhibitor. In order to test the function of exosomes generated in the s-TME, we collected exosomes from conditioned media of the s-TME and treated the purified exosomes on Panc0203 cells for 20 h. Higher levels of green fluorescence (which were derived from the exosomes) were detected in the cytoplasm of Panc0203 cells exposed to the s-TME CMFDA/DMSO-Exo (purified exosomes from conditioned media of CMFDA/DMSO-treated s-TME model) than in that of the Panc0203 cells exposed to the s-TME CMFDA/GW4869-Exo (purified exosomes from conditioned media of CMFDA/GW4869-treated s-TME model) (Supplementary Fig. 1h), indicating that the macrophages work as an exosome donor to the pancreatic cancer cells.

Moreover, treatment of the purified exosomes from the conditioned media of DMSO-treated s-TME model (s-TME DMSO-Exo) induced TNT formation and enlarged cytoplasm with vacuoles in the standard-cultured Panc0203 cells, and these effects were less induced by the purified exosomes from the conditioned media of GW4869-treated s-TME model (s-TME GW4869-Exo; Supplementary Fig. 1i). Gemcitabine induced a statistically significant reduction in viability of Panc0203 cells under the standard culture and s-TME GW4869-Exo-treated condition (Supplementary Fig. 1j). However, slightly decreased cell viability was observed in Panc0203 cells treated with the s-TME DMSO-Exo. Interestingly, no further reduction in cell viability was seen in the presence of gemcitabine treatment in Panc0203 cells treated with the s-TME DMSO-Exo. This indicates that the s-TME DMSO-Exo induces the resistance of Panc0203 cells to gemcitabine (Supplementary Fig. 1j). These data indicate that exosomes from the s-TME participate in the transfer of biomolecules and resulted in the change of cell morphology, proliferation, and responsiveness to gemcitabine.

### Increased anchorage-independent growth or invasiveness of pancreatic derivative cancer cells

To investigate the characteristics of pancreatic derivative cells generated via direct cell-to-cell transfer, we established five clonal cell lines from DFCs; i.e., two lines derived from Panc0203 cells (SP0926 (side population 0926) and SP1030), and three lines derived from Panc0327 cells (SP0913^1A^, SP0913^1B^, and SP0913^1C^; Fig. 2a and Supplementary Fig. 2a). Short tandem repeat (STR) profiling showed that the STR pattern of each SP cell line was identical to that of mother pancreatic cancer cell line (Supplementary Fig. 2b), demonstrating that the SP cell lines had expanded from pancreatic cancer cells that had acquired the cellular component of MØ-U937 cells by direct cell-to-cell transfer. However, there were some genomic structural variants (SVs) including a novel *BCL2L1-GLB1L2* fusion gene in the derivative cells (Supplementary Fig. 2c, d). The morphology of derivative cells during the exponential cell growth was examined with bright-field microscopy, which showed that the spatial patterns of derivative cells were diverse. For example, SP1030, SP0913^1A^, and SP0913^1C^ cells formed compact clusters, whereas SP0926 and SP0913^1B^ cells grew in more evenly distributed spatial patterns (Fig. 2a and Supplementary Fig. 2a). The growth rate of derivative cells was not significantly different from that of the mother pancreatic cancer cells in normoxic, hypoxic, or low oxygen and serum (LOS) conditions (Supplementary Fig. 3a). The sensitivity of Panc0203 derivative cells to gemcitabine differed based on the culture conditions; i.e., under normoxic conditions, SP9026 cells were significantly more resistant than SP1030 cells or mother cells, whereas under hypoxic and LOS conditions, SP1030 cells were significantly more resistant than SP0926 cells or mother cells (Supplementary Fig. 3b).

We next investigated the tumorsphere formation and invasive characteristics of the derivative cells. Compared to Panc0203 cells, SP1030 cells included larger numbers of aldefluor-positive cells (28.4% and 42.5%, respectively; Fig. 2b) representing aldehyde dehydrogenase (ALDH) activity, a marker for cancer stem cell-like cells (CSCs)^25^. However, the number of aldefluor-positive cells among SP0926 cells (33.1%) was not significantly different from that among Panc0203 cells (Fig. 2b). Results of an analysis of tumorsphere formation were consistent with the ALDH results, as both the number and diameter of spheroids were significantly greater in SP1030 cells than in Panc0203 cells, and the number of spheroids was significantly lower in SP0926 cells than in Panc0203 cells, with no significant difference in the diameter of spheroids between SP0926 and Panc0203 cells (Fig. 2c). An invasion assay to assess the metastatic capacity of derivative cells showed a dramatic increase in the invasive activity of SP0926 cells compared to Panc0203 cells, but the invasive activity of SP1030 cells was found to be similar to that of Panc0203 cells (Fig. 2d). Together, these data suggest that the anchorage-independent growth and invasive potential of derivative cells differed according to clones; for example, SP0926 cells showed increased invasive activity compared to mother Panc0203 cells, whereas SP1030 cells showed increased tumorsphere formation activity compared to the mother cells. The biased drift into either anchorage-independent growth or invasiveness was also observed in the derivative cell lines from Panc0327 cells; that is, compared to mother cells, SP0913^1A^ and SP0913^1C^ cells exhibited increased tumorsphere formation, whereas SP0913^1B^ cells showed increased invasiveness (Supplementary Fig. 3c, d).

### Metabolic reprogramming in pancreatic derivative cancer cells

**Fig. 3:**
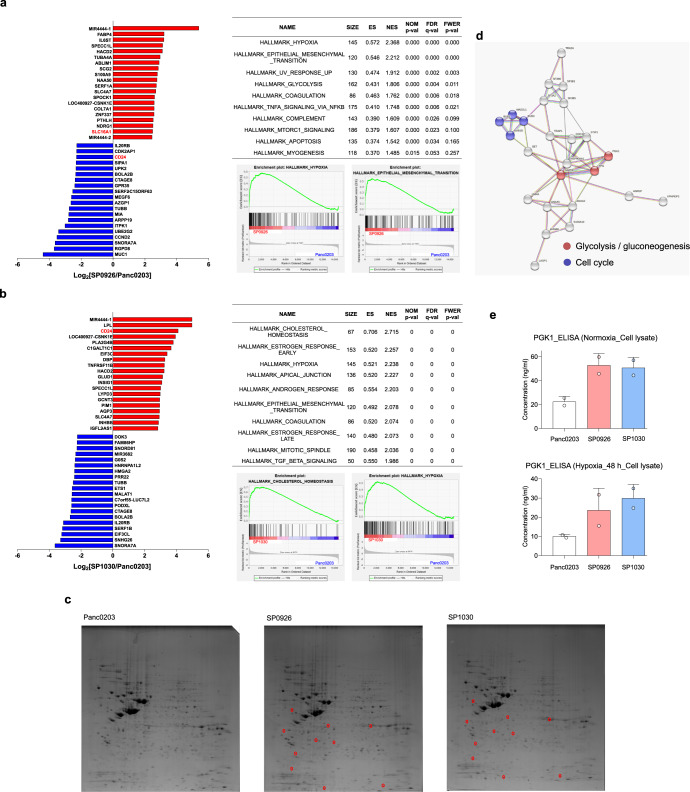
Multi-omic analyses showing metabolic reprogramming in pancreatic derivative cancer cells derived via direct cell-to-cell transfer. **a** Transcriptomic comparison between SP0926 and Panc0203 cells. Left panel shows genes of SP0926 cells differentially expressed compared to parent Panc0203 cells, sorted via RNA sequencing. The top 20 up-regulated (red) and down-regulated (blue) genes are depicted according to fold change. Right upper panel shows the list of the top 10 enriched gene sets in SP0926 cells compared to Panc0203 cells, determined via gene set enrichment analysis (GSEA) using hallmark gene sets (http://www.gsea-msigdb.org/gsea/msigdb/index.jsp). The table shows the number of genes in the gene sets (SIZE), enrichment score (ES), normalized enrichment score (NES), nominal *P*-value (NOM *P*-value), false discovery rate (FDR Q-value), and family-wise error rates (FWER Q-value). Right lower panel represents GSEA score curve for ‘HYPOXIA’ and ‘EPITHELIAL_MESENCHYMAL_TRANSITION’ gene sets. **b** Transcriptomic comparison between SP1030 cells and Panc0203 cells. Left panel shows genes of SP1030 cells differentially expressed compared to parent Panc0203 cells, determined via RNA sequencing. The top 20 up-regulated (red) and down-regulated (blue) genes are depicted according to fold change. Right upper panel shows the list of the top 10 enriched gene sets in SP1030 cells compared to Panc0203 cells determined via gene set enrichment analysis (GSEA) using hallmark gene sets. The table shows the number of genes in gene sets (SIZE), enrichment score (ES), normalized enrichment score (NES), nominal *P*-value (NOM *P*-value), false discovery rate (FDR Q-value), and family-wise error rates (FWER *Q*-value). Right lower panel represents GSEA score curve for ‘CHOLESTEROL_HOMEOSTASIS’ and ‘HYPOXIA’ gene sets. **c** Ten protein spots similarly upregulated (fold change ≥2; red circles) in both SP0926 and SP1030 cells compared to Panc0203 cells were analyzed using μLC-MS/MS. Among the 10 pairs of spots, two pairs have no significant hits to report. Sixteen proteins were identified in the remaining eight pairs of spots in which ~40% of the pairs were identified as containing multiple proteins. **d** STRING network analysis of interactions among 16 genes that were up-regulated in both SP0926 and SP1030 cells compared to Panc0203 cells. The analysis was performed by the option of ‘minimum required interaction score: median confidence (0.400)’ and ‘maximum number of interactors to show: no more than 10 interactions (1st shell)’. Functional enrichment analysis was performed for gene sets from the KEGG pathway, and red and blue dots indicate genes belonging to the KEGG pathway enriched in derivative cells. **e** PGK1 was detected by ELISA using lysates from normoxic (upper panel) and hypoxic (48 h; lower panel) Panc0203, SP0926, and SP1030 cells. Data are presented as the mean values ±SD.

Multi-omic analyses were undertaken to understand the molecular mechanisms underlying the altered anchorage-independent growth and invasive activities of the derivative cells. First, transcriptome analysis using RNA sequencing was conducted to compare gene-expression profiles of SP0926 and SP1030 cells with those of Panc0203 and MØ-U937 cells (Supplementary Table 1). The hierarchical clustering and multidimensional scaling data show that the transcriptomes of SP0926 and SP1030 cells were closer to that of Panc0203 cells than that of MØ-U937 cells (Supplementary Fig. 3e, f). Comparison of the transcriptome of SP0926 cells with that of Panc0203 cells revealed that metastasis-related gene sets (e.g., ‘EPITHELIAL_MESENCHYMAL_TRANSITION’), and metabolism-related gene sets (e.g., ‘HYPOXIA,’ ‘GLYCOLYSIS,’ and ‘MTORC1_SIGNALING’) were significantly enriched in SP0926 cells (Fig. 3a). These findings suggest that enrichment of these pathways was associated with increased metastatic potential of SP0926 cells. In addition, gene sets associated with responses to stress such as ‘UV_RESPONSE_UP’ and ‘APOPTOSIS’ and gene sets related with inflammation including ‘COAGULATION’, ‘TNFA_SIGNALING_VIA_NFKB’, and ‘COMPLEMENT’ were also enriched in SP0926 cells (Fig. 3a). For SP1030 cells, cancer stem cell-related gene sets (e.g., ‘CHOLESTEROL_HOMEOSTASIS’^26^, ‘HYPOXIA’^27^, and ‘EPITHELIAL_MESENCHYMAL_TRANSITION’^28^]) and cell cycle-related gene sets (e.g., ‘MITOTIC_SPINDLE’) were significantly enriched compared with Panc0203 cells (Fig. 3b), indicating that enrichment of these pathways was related to increased tumorigenic potential of SP1030 cells. In addition, hormone-related gene sets such as ‘ESTROGEN_RESPONSE_EARLY’, ‘ESTROGEN_RESPONSE_LATE’, and ‘ANDROGEN_RESPONSE’ and gene sets related with inflammation including ‘COAGULATION’ and ‘TGF_BETA_SIGNALING’ were also enriched in SP1030 cells (Fig. 3b).

In parallel, we performed two-dimensional gel electrophoresis (2DE) followed by proteomic peptide analysis of 10 protein spots that were shown via 2DE to be similarly increased in both SP0926 and SP1030 cells compared to Panc0203 cells (≥2-fold change; Fig. 3c). We identified 16 proteins from 10 spots and found that glycolysis/gluconeogenesis-related proteins, such as PGK1 and GAPDH, were enriched in SP0926 and SP1030 cells (Fig. 3d and Supplementary Table 2). The increased expression of the PGK1 protein in both normoxic and hypoxic conditions was validated by enzyme-linked immunosorbent assay (ELISA; Fig. 3e). These transcriptomic and proteomic results suggest that metabolic reprogramming events, including glycolysis and cholesterol metabolism, were highly associated with altered tumorigenic and metastatic potential of the pancreatic derivative cancer cells. We also performed proteomic analysis for exosomes from the normal co-culture TME condition and s-TME condition. We investigated proteins up-regulated in stressed TME condition and identified 21 proteins from 17 commonly found spots from both condition and 5 spots specific to stressed TME condition (Supplementary Fig. 4a and Supplementary Tables 3 and 4). Mass spectrometry analysis identified that several exosomal proteins including stratifin and LGALS3BP (https://www.ebi.ac.uk/QuickGO/GProteinSet?id=Exosome) were up-regulated in stressed TME condition. The increase of stratifin and LGALS3BP in stressed TME condition was confirmed in exosome and conditioned media by ELISA (Supplementary Fig. 4b, c). This suggests that the increased exosomal components in stressed TME may be involved in the formation of pancreatic cancer derivative cells.

### CD24 and CD44 expression-dependent tumorsphere formation or invasive activity in pancreatic derivative cancer cells

**Fig. 4:**
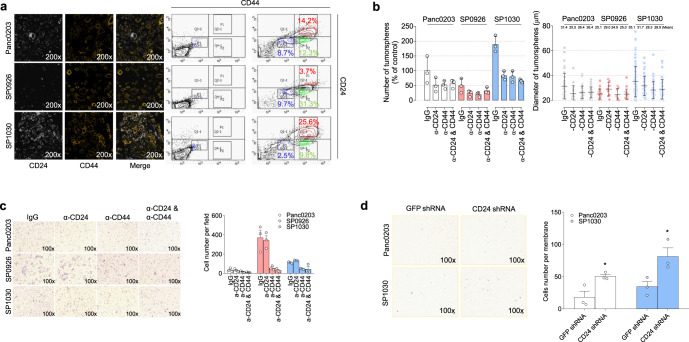
Regulation of CD24 and CD44 associated with anchorage-independent growth and invasive activities. **a** Confocal microscopy (left) and flow cytometry (right) were performed using specific antibodies for CD24 and CD44 labeled with PE and APC, respectively. **b**, **c** Suppression of tumorsphere formation activity (**b**) and invasion activity (**c**) by treatment with either or both of the anti-CD24 and CD44 antibodies. The tumorsphere assay was conducted for 7 days, and the invasion chamber assay system containing Matrigel was performed for 24 h. The relative number of tumorspheres compared to the percentage of Panc0203 cells group treated by IgG control (**b**, left). Diameter of tumorspheres (**b**, right). **d** Effect of *CD24* short hairpin RNA (shRNA) on the invasion activity of Panc0203 and SP1030 cells. An invasion chamber assay system containing Matrigel was performed for 24 h. Data are presented as the mean values ±SD.

Next, we investigated the specific molecular targets associated with altered anchorage-independent growth and invasive activities of the pancreatic derivative cancer cells. Because SP0926 and SP1030 cells showed opposing directional increases in tumorsphere formation and invasive activities, we chose transcripts that showed multiple-fold changes in terms of opposing expression levels between SP0926 and SP1030 cells. We found that expression of *CD24*, a well-known marker for inhibition of invasion and metastasis among CSCs^29–31^, was ~5-fold lower in SP0926 cells and ~18-fold higher in SP1030 cells compared to Panc0203 cells in transcriptomic analysis (Fig. 3a, b and Supplementary Table 1), which was confirmed by confocal microscopy and flow cytometry (Fig. 4a). For *CD44*, a well-known CSC marker regulating cancer stemness in various cancer types^32,33^, there is some discrepancy between the expression levels of mRNA and protein; the transcript levels of *CD44* were high only in SP1030 cells compared to mother cells (Supplementary Table 1 and Supplementary Fig. 5a), but, in image analysis for protein expression from confocal images, the CD44 density of ROIs increased in SP0926 (mean: 19.3) and SP1030 cells (mean: 24.1) compared to mother Panc0203 cells (mean: 15.3; Supplementary Fig. 5b). In addition, we examined the expression level of *CD44* and *CD24* genes in SFCs and DFCs to investigate the expression change of the markers. We isolated SFCs and DFCs by FACS sorting at the end of the Seeding phase of the s-TME model and examined the expression of *CD24* and *CD44* genes by qRT-PCR. The s-TME condition induced about a 2.5-fold and an 8.8-fold increase of *CD44* expression in SFCs and DFCs in the vehicle (NT, not treated) group, respectively, compared to standard-cultured Panc0203 cells (Supplementary Fig. 5c). The expression of *CD24* showed about a 4.3-fold and a 9-fold increase in SFCs and DFCs in the vehicle (NT, not treated) group, respectively, compared to standard-cultured Panc0203 cells (Supplementary Fig. 5c).

To validate the anchorage-independent growth and invasive effects of CD24 and CD44, we applied neutralizing antibodies to either or both CD markers. Treatment of the anti-CD44 antibody not only reduced tumorsphere formation of SP1030 and Panc0203 cells (Fig. 4b), but also the invasion capability of SP0926 and SP1030 cells (Fig. 4c). These results suggest that increased CD44 was involved in both the anchorage-independent growth and invasive potential of the derivative cells. Concordantly, CD44 neutralizing antibody suppressed tumorsphere formation of SP0913^1A^ and SP0913^1C^ cells, which are derived from Panc0327 cells (Supplementary Fig. 5d). Treatment of the anti-CD24 neutralizing antibody had little effect on the invasive activity of SP0926 cells (Fig. 4c), as CD24 expression levels are very low in this derivative cancer cells (Fig. 4a). However, overexpression of CD24 suppressed the invasive activity of SP0926 cells (Supplementary Fig. 5e). When the anti-CD24 antibody was treated for SP1030 cells, the invasive activity was slightly increased (cell number per field: control 112.3 ± 7.0, anti-CD24 133.3 ± 4.0), but there was no statistical significance in one-way ANOVA test (Fig. 4c). Knock-down of CD24 expression using short hairpin RNA (shRNA) aggravated the invasive activity of both SP1030 and Panc0203 cells (Fig. 4d). The knock-down effect of CD24 shRNA was validated using flow cytometric analysis for CD24 (Supplementary Fig. 5f). In addition, inhibition of CD24 using blocking antibody or shRNA resulted in a change in the spatial growth patterns of SP1030 and Panc0203 cells from cluster formations into dispersed single cells (Supplementary Fig. 5g). These data suggest that down-regulation of CD24 in SP0926 cells was associated with increased invasive potential of the pancreatic derivative cancer cells.

### MCT1-associated tumorsphere formation and invasive activities of pancreatic derivative cancer cells

**Fig. 5:**
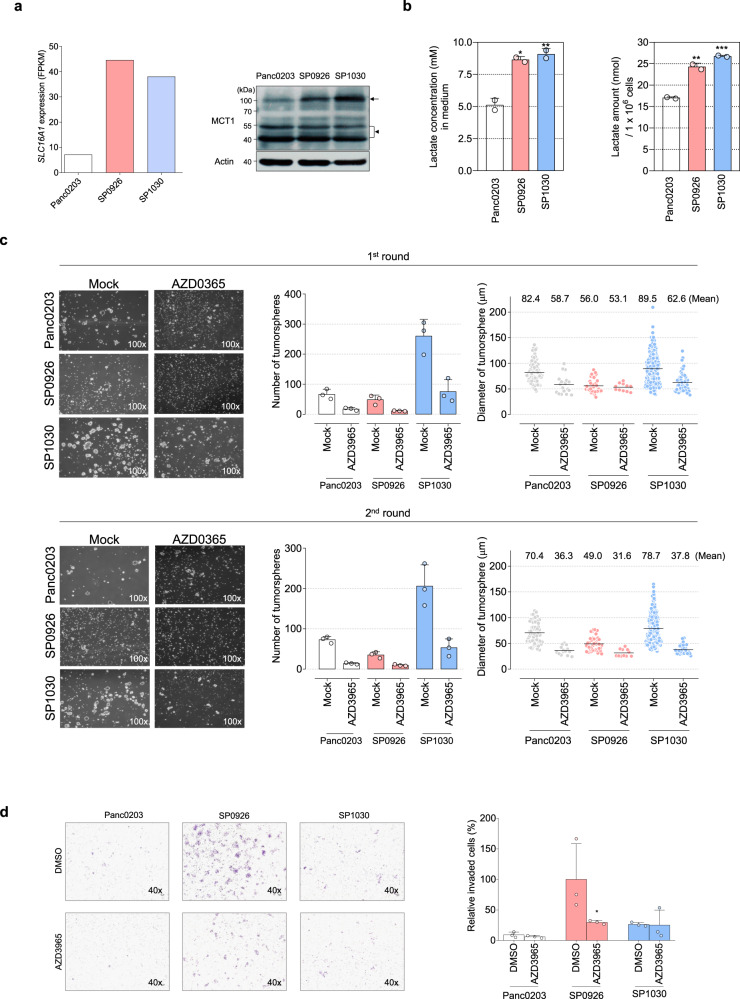
Roles of MCT1 in anchorage-independent growth and invasive activities of pancreatic derivative cells generated via direct cell-to-cell transfer. **a** Comparison of *SLC16A1* transcript (Fragments Per Kilobase of transcript per Million; FPKM, left) and MCT1 protein (right) expression levels among Panc0203, SP0926, and SP1030 cells. *SLC16A1* transcript levels were estimated by RNA sequencing and MCT1 protein levels were evaluated by western blotting. Arrow and arrow head indicate a dimer form (95–100 kDa) and monomer forms (43–48 kDa), respectively (right, upper). Uncut and unprocessed scans for MCT1 and beta-actin are shown in Supplementary Fig. 5i. **b** L-lactate concentration in the collected media (left) and l-lactate amount in the 1 × 10^6^ collected cells (right) at 72 h following 3.5 × 10^5^ cells seeding per six-well plates. *P ≤ 0.05; **P ≤ 0.005. One-way ANOVA followed by Tukey’s multiple comparison test. **c** Inhibition of tumorsphere formation activity by the MCT1 inhibitor AZD3965 (20 μM) compared with DMSO group (Mock). The first-round tumorspheres were observed on day 10 after seeding, and the second-round tumorsphere assay was conducted for 11 days followed by dissociation of the whole spheroids from the first round. **d** MCT1-dependent invasive activity. Cells were cultured for 24 h in the presence of DMSO or 20 μM AZD3965 in both the upper and bottom chambers. *P ≤ 0.05 as compared to DMSO control (unpaired *t* test). Data are presented as the mean values ±SD.

Because our transcriptomic and proteomic analyses suggest that alteration of metabolic reprogramming events, especially glycolysis, was highly associated with tumorsphere formation and invasive activities of the pancreatic derivative cancer cells (Fig. 3a–e), we investigated whether regulation of metabolic proteins would also confer changes in both anchorage-independent growth and invasiveness in vitro. Based on RNA sequencing data, *SLC16A1* mRNA, encoding MCT1 which transports lactate and pyruvate in aerobic glycolytic tumors^34^, was up-regulated to similar degrees in SP0926 (6.2-fold) and SP1030 cells (5.6-fold) (Fig. 5a (left) and Supplementary Table 1), which was confirmed by western blotting (Fig. 5a (right), Supplementary Fig. 5i). On the other hand, there was no change in the expression of *SLC16A1* in SFCs and DFCs compared to standard-cultured Panc0203 cells (Supplementary Fig. 5c). In the subsequent estimation of L-lactate, which is one of the final products of glycolysis, the increased level of lactate was detected in cell lysate and culture media of the derivative cells (Fig. 5b). Treatment of a known MCT1 inhibitor, AZD3965, significantly suppressed the tumorsphere formation of SP1030 cells (Fig. 5c) and invasive activity of SP0926 cells (Fig. 5d). In parental Panc0203 cells, AZD3965 slightly inhibited tumorsphere formation (Fig. 5c) and had little effect on invasiveness (Fig. 5d). Concordantly, AZD3965 treatment substantially abolished tumorsphere formation in SP0913^1A^ and SP0913^1C^ cells (Supplementary Fig. 5d) and the invasive activity of all derivative cells from Panc0327 cells (Supplementary Fig. 5h). These data support the idea that inhibition of aberrant glycolytic metabolism of derivative cells reduces both anchorage-independent growth and invasive activities.

### CD44 and MCT1 are candidate prognostic markers for pancreatic cancer patients

**Fig. 6:**
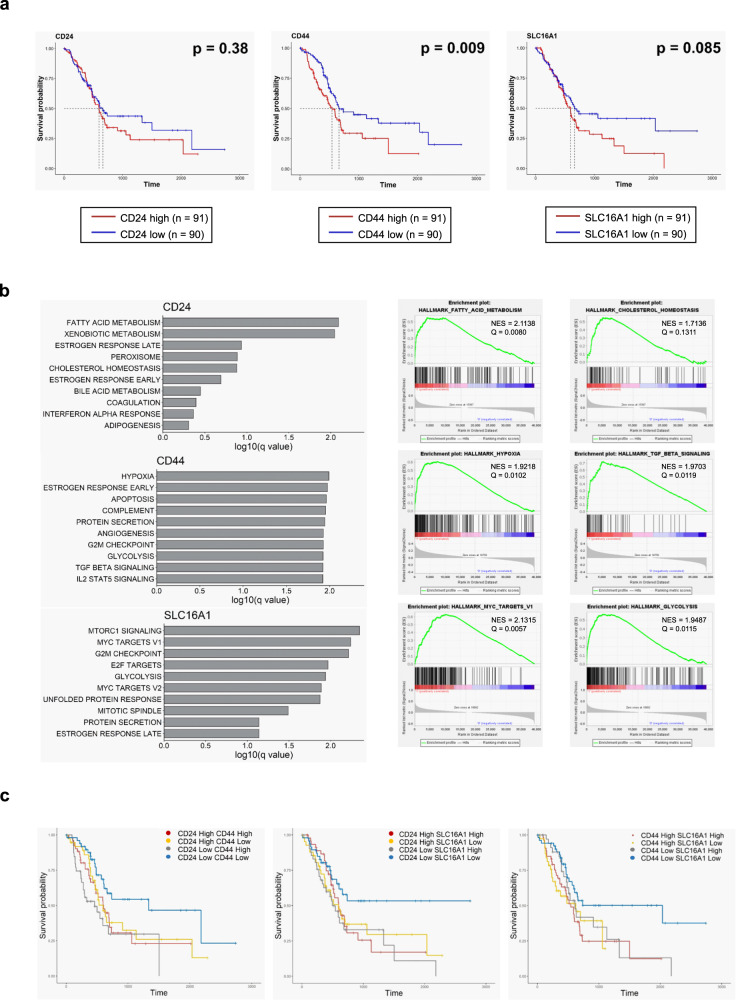
Clinical significance of *CD24*, *CD44*, and *SLC16A1* expression in pancreatic cancer-patient tissue samples. **a** Kaplan–Meier plot for overall survival of pancreatic cancer patients with high or low expression of *CD24*, *CD44*, or *SLC16A1*. Patients in the TCGA Pancreatic Cancer (PAAD) database (*n* = 182) were divided into two groups (median cutoff) with either high or low expression of CD24 (left), CD44 (middle), or MCT1 (right). Red and blue lines indicate samples with high or low expression, respectively, of each gene. Each median survival and P-value, determined by a log rank test, is indicated. **b** Gene set enrichment analysis (GSEA) using hallmark gene sets (http://www.gsea-msigdb.org/gsea/msigdb/index.jsp) between pancreatic cancer patients with high or low expression of *CD24*, *CD44*, or *SLC16A1*. Left panel shows the top 10 enriched gene sets in patients with high expression of genes [upper: *CD24*, middle: *CD44*, bottom: *SLC16A1*]. Right panel shows representative GSEA score curves enriched in patients with high expression of these genes (upper: *CD24*, middle: *CD44*, bottom: *SLC16A1*). **c** Effect of combined (in pairs) gene expression of *CD24*, *CD44*, and *SLC16A1* on overall survival of pancreatic cancer patients. Kaplan-Meier plots for overall survival according to patient groups based on the combined gene expression (median cutoff) effects are indicated (left: *CD24* and *CD44*, middle: *CD24* and *SLC16A1*, right: *CD44* and *SLC16A1*).

Because altered expression of *CD24*, *CD44*, and *SLC16A1* is highly associated with the anchorage-independent growth and invasive activities of pancreatic derivative cancer cells, we investigated the potential clinical significance of these findings by comparing survival rates of patients with high or low expression of these three genes using the TCGA Pancreatic Cancer (PAAD) database (*n* = 182, http://xena.ucsc.edu/). *CD24*, *CD44*, and *SLC16A1* expression were not associated with disease stage (Supplementary Fig. 6a). Although survival probability did not differ between patients with high *CD24* expression and those with low *CD24* expression, patients with high *CD44* or *SLC16A1* expression showed poorer prognosis than those with low expression of these genes (Fig. 6a). In gene set enrichment analyses using RNA sequencing data, patients with high *CD44* expression showed enriched pathways associated with metabolic reprogramming, including ‘HYPOXIA’ and ‘GLYCOLYSIS,’ and with CSC phenotype, including ‘TGF_BETA_SIGNALING’ (Fig. 6b). In addition, patients with high *SLC16A1* expression exhibited enrichment of metabolism-related gene sets, including ‘MTORC1_SIGNALING’ and ‘GLYCOLYSIS,’ and cell cycle-related gene sets, including ‘MYC_TARGETS,’ ‘E2F_TARGETS,’ ‘G2M_CHECKPOINT,’ and ‘MITOTIC_SPINDLE’ (Fig. 6b). These data suggest that increased *CD44* or *SLC16A1* expression is a probable prognostic marker in pancreatic cancer patients and is related to tumor aggressiveness mediated by metabolic reprogramming, CSC phenotype, and rapid cell proliferation. On the other hand, fatty acid and cholesterol metabolism pathways were highly enriched in patients with high *CD24* expression (Fig. 6b).

Next, we investigated the combined effect of *CD24*, *CD44*, and *SLC16A1* expression on the prognosis of the pancreatic cancer patients. *CD24* and *CD44* expression were positively correlated with one another (Pearson’s correlation coefficient *r* = 0.32, *P* < 0.001), as were *CD44* and *SLC16A1* expression (Pearson’s correlation coefficient *r* = 0.38, *P* < 0.001) (Supplementary Fig. 6b). In survival analyses considering the combined expression of two genes, patients with *CD24*^low^/*CD44*^low^, *CD24*^low^/*SLC16A1*^low^, and *CD44*^low^/*SLC16A1*^low^ expression exhibited better prognosis compared with other subgroups (Fig. 6c). Similar tendency was observed in another group of pancreatic patients (GSE84219 from Gene Expression Omnibus (https://www.ncbi.nlm.nih.gov/geo/), *n* = 30), although *p*-values are not statistically significant due to small sample size (Supplementary Fig. 6c). Previous reports have shown that MCT1 has tumorigenic and metastatic activity^35–38^, and that it is known to interact and co-localize with CD44 in breast cancer cells^39^ and in metastatic prostate cancer cells^40^. Considering the effects of single gene expression on patient survival, these data suggest that increased expression of either *CD44* or *SLC16A1* is a candidate prognostic marker for pancreatic cancer patients, and that targeting CD44 and MCT1 together is required for the proper treatment of aggressive pancreatic cancers.

## Discussion

Our study explored the dramatic impact of chemotherapy on a subset of pancreatic cancer cells within the TME. Although pancreatic tumors are heterogeneous in terms of histological type and stage, they generally show extremely aggressive phenotypes and have limited treatment options^15,16^. Due to enhanced fibrosis in the TME, pancreatic cancer cells are usually in a hypoxic environment^17^. In addition, after standard treatment that includes gemcitabine, a DNA-damaging deoxycytidine nucleoside analogue, pancreatic cancer cells in the TME are exposed to genotoxic stress^41^. This TME confers unusual selective pressure on the pancreatic cancer cells, which causes DNA changes and/or induces dysregulation of specific gene-expression patterns and thereby promotes survival of the cancer cells. In this study, we developed a TME mimic condition that simplifies in vivo contexts. This TME mimic condition comprises a cellular component, including cancer cells and stromal cells (fibroblasts and immune cells); and stress conditions, including a hypoxic and a genotoxic environment. In our TME mimic condition, pancreatic cancer cells showed enhanced direct cell-to-cell transfer of intracellular biomolecules from surrounding cells, which in turn resulted in increased anchorage-independent growth or invasive potential compared to the mother cells.

When we established the derivative cell lines resulting from direct cell-to-cell transfer, the derivative cells exhibited diverse potentials in terms of tumorigenicity and metastasis (Fig. 2 and Supplementary Fig. 3c, d) and genomic structural variants (SVs) including a novel *BCL2L1-GLB1L2* fusion gene (Supplementary Fig. 2c, d). These findings suggest that direct cell-to-cell transfer may represent a mean through which intratumoral heterogeneity is increased in response to stress conditions. Several studies have described the association between direct cell-to-cell transfer and cellular stresses. TNT generation was shown to be induced by metabolic stress (serum/nutrient starvation, hypoxia, and low pH), radiation, androgen receptor blockade, and pathogenic stress^42–44^. In addition, cell fusion was induced by toxins, cryo-damage, and hypoxic stress^45^, and exosome secretion was elicited by oxidative and cytostatic stress^46^. Direct cell-to-cell transfer via TNTs resulted in the transfer of mitochondria and mutant KRAS protein, which in turn resulted in alteration of the metabolic characteristics of cells and increased mutational heterogeneity^47,48^. In contrast, disruption of TNTs sensitized prostate cancer cells to hormone blockade therapy and metabolic stress^43^. Direct cell-to-cell transfer of biomolecules, such as DNA, mRNA, miRNA, lncRNA, proteins, and organelles, can induce remodeling of gene expression in cancer cells^11^.

The derivative cells are the result of the combined action of hypoxia, gemcitabine treatment, secretary factors from fibroblast, and direct cell-to-cell transfer from macrophage. We suggest that direct cell-to-cell transfer of biomolecules is one of the main factors that can aggravate the tumorigenic or metastatic potential of pancreatic cancer cells. First, the surviving cells in our TME mimic condition demonstrated the increase of cytoplasmic vacuoles (which is associated with production of exosome) and TNTs in bright field microscopy (Fig. 1b), suggesting the increase of direct cell-to-cell transfer in stressed TME conditions. The enlarged cytoplasm with vacuoles and TNT-formed morphologies were significantly maintained after seeding of the FACS-sorted DFCs (Supplementary Fig. 1a, b) and was partially observed as a mosaic pattern even after cell line generation (Fig. 2a and Supplementary Video 1). However, simple co-culture of pancreatic cancer cells and macrophage cells resulted in low percentage of DFCs (Fig. 1e). In addition, inhibition of exosomes or TNTs significantly decreased numbers of DFCs in the s-TME condition (Supplementary Fig. 1d). Second, the survivability of DFCs was greater than that of single-fluorescent pancreatic cancer cells and macrophages in our stressed TME conditions because DFCs were detected in 9.4–12.9% of adherent cells (which was viable) and 0.3% of detached cells (which was dying; Fig. 1c–e). Third, when we analyzed the proteome of exosomes in normal culture condition and stressed TME condition, we found increase of several exosomal proteins including stratifin and LGALS3BP (Supplementary Tables 3 and 4) in stressed TME condition, suggesting the increase of exosome in our experimental condition. Stratifin was reported to be externalized via exosome and stimulate the expression of matrix metalloproteinase-1, which is associated with the metastatic activity^49^. In addition, the protein expression of stratifin receptor^50^, CD13, was increased in both SP0926 and SP1030 cells compared to Panc0203 cells in proteomic analysis of cell lysates (Supplementary Table 2). LGALS3BP was reported to be up-regulated in various cancers and associated with cancer progression^51^ and exosomes containing high levels of LGALS3BP contributed to endometrial cancer cell proliferation and migration by activating PI3K/AKT/VEGFA signaling pathway^52^. Therefore, increased exosomal components in stressed TME are associated with tumorigenic and metastatic potential of pancreatic cancer cells. Forth, direct treatment of purified exosomes from the s-TME condition induced the characteristic alterations of pancreatic cancer cells in terms of cell morphology (increased TNT formation and enlarged cytoplasm with vacuoles; Supplementary Fig. 1h), cell proliferation (reduced cell proliferation with little cell death; Supplementary Fig. 1i), and drug responsiveness (increased resistance to gemcitabine; Supplementary Fig. 1i). Fifth, *CD44* and *CD24* levels were additionally increased in DFCs compared to the levels of SFCs, while there was no change in *SLC16A1* gene expression in both SFCs and DFCs. The additional up-regulations of *CD44* gene in DFCs were consistent (partly dependent on both exosomes and TNTs) whereas the upregulations of *CD24* genes in DFCs are transient (dependent on both exosomes and TNTs). This suggests those expressions are changeable rather than the *CD44/CD24/SLC16A1* expression is fixed when Panc0203 becomes SFCs and DFCs under the s-TME model and then DFCs becomes derivative cells under normal culture condition. It could be due to epigenetic reprogramming. Sixth, although the alterations of markers such as CD24 and CD44 are characteristics in the tumorsphere formation and invasiveness of the derivative cells, transcriptomic analysis demonstrated alterations of several other pathways associated with metabolism, response pathways to external stimuli, and inflammation (Fig. 3a, b). Therefore, cells from simple sorting for CD24 and CD44 levels would be quite different from the derivative cells. Taken together, we propose that direct cell-to-cell transfer in the s-TME condition is one of mechanisms resulting in the alterations of tumorigenic or metastatic potential in pancreatic cancer cells.

We included macrophages in our stressed TME mimic system because tumor-associated macrophages in pancreatic cancer play critical roles in tumorigenesis, metastasis, and chemotherapy resistance^53^. In addition, cell-to-cell transfer of biomolecules via exosomes or tunneling nanotubes has been suggested as a mode of tumor cell-macrophage communication^54^. Recently, pancreatic stellate cells (PSCs) have been suggested as one of the major contributors in aggressiveness and metastasis of pancreatic cancer^55^, and pancreatic cancer cell-derived exosomes promoted the pancreatic cancer recruitment of PSCs^56^, which reinforce the importance of cell-to-cell transfer of biomolecules in the aggressiveness of pancreatic cancer. Therefore, the roles of PSCs in cell-to-cell transfer of biomolecules in pancreatic cancer TME need to be further evaluated.

Established derivative cell lines showed changes in CD24/CD44/MCT1 expression, which were correlated with anchorage-independent growth and invasiveness in vitro. When we analyzed the expressions of CD44 and CD24 to understand the molecular characteristics of the derivative cells, protein expression of CD44 was increased in derivative cells compared to parental cells (Fig. 4a and Supplementary Fig. 5b), which is associated with anchorage-independent growth or invasiveness (Fig. 4b, c), but CD24 expression was substantially decreased in cells showing enhanced invasiveness (Fig. 4a, c). CD24 was previously shown to determine metastatic capacity in breast cancer, as well as in other tumor types^29–31^. Moreover, CD44 has been reported to be important for CSC genesis^34^, including promotion of both tumorigenic and metastatic potential, by creating a variety of repertoires in quantitative combination with other cell surface markers including CD24^57^. Together, these results suggest that the relative expression levels of CD24 may be one of the determinants for transition between anchorage-independent growth and invasive phenotypes; namely, cells with CD44^high^/CD24^low^ expression may exhibit relatively high invasiveness (i.e., metastatic trait), and cells with CD44^high^/CD24^high^ expression may exhibit increased ALDH activity and the ability to develop tumorspheres (i.e., tumorigenic trait).

Transcriptome gene set analysis results also suggest common metabolic alterations in derivative cells, including aerobic glycolysis. In particular, gene-expression analyses show that all derivative cells with high anchorage-independent growth or invasive activity show increased expression of *SLC16A1*, a phenomenon that contributes to the glycolytic phenotype of cancer cells^58^. MCT1 is barely detected in normal pancreatic tissue^59,60^, but its levels are abnormally increased in pancreatic cancer tissues and cell lines^61–63^, including the derivatives cells from Panc0203 and Panc0327 in the present study. In addition, MCT1 was reported to have tumorigenic and metastatic activity^35,37,38,64^ and to co-localize with CD44 in breast cancer and metastatic prostate cancer cells^39^. Considering the roles of CD24, CD44, and MCT1 in regulating a CSC-like phenotype, we suggest that the acquisition of metabostemness induces establishment of pancreatic cancer cells that exhibit different degrees of anchorage-independent growth and invasive properties based on the relative levels of *CD24*, *CD44*, and *SLC16A1* (Supplementary Fig. 6d).

In our experimental system, the characteristics of clonally derived cells were diverse, so it is difficult to generalize the proposed mechanisms mediated by direct cell-to-cell transfer of biomaterials. However, in our stressed TME mimic condition, derivative cells from two mother cells demonstrated the increased tumorsphere formation or invasiveness according to cell clones (Fig. 2c, d and Supplementary Fig. 3c, d). Therefore, it is obvious that derivative cells from our in vitro TME mimic condition showed the altered potential in tumorigenic or invasive phenotypes. Although we characterized the derivative cells with a few markers associated with tumorigenic or invasive activity, there was a significant transcriptomic reprogramming in the derivative cells (Fig. 3a, b). The specialized reprogramming of the derivative cells associated with tumorigenic or invasive phenotypes need to be further investigated.

One possible explanation on how the characteristics of derivative cells can persist throughout the generations is constitutive epigenetic reprogramming. Through direct cell-to-cell transfer of biomolecules, mRNAs, non-coding RNAs, proteins, and metabolites can all potentially be delivered into pancreatic cancer cells from stromal cells including macrophage and fibroblast, and these biomolecules can induce epigenetic reprogramming with or without the s-TME condition. Certain stressful conditions, such as hepatitis C viral infection^65^, nickel exposure^66^, and nerve injury^67^ have been reported to induce constitutive epigenetic reprogramming by altering histone modification or DNA methylation status. Moreover, it has been shown that non-DNA sequence-based epigenetic information can be inherited across generations via direct cell-to-cell transfer of non-coding RNAs^68^. In this study, we also found some genomic structural variants (SVs), including a novel *BCL2L1-GLB1L2* fusion gene in the derivative cells (Supplementary Fig. 2c, d), that could be another mechanism for the persistent characteristics of the derivative cells.

Because pancreatic cancer is usually resistant to standard chemotherapy and exhibits extremely poor prognosis in terms of overall survival, the development of procedures to overcome chemotherapy resistance is urgently required. Our data suggest that direct cell-to-cell transfer of biomolecules play roles in providing a survival advantage to cancer cells during chemotherapy and targeting CD44 and/or MCT1 may be a plausible strategy to overcome drug resistance and metastasis in pancreatic tumors. In this study, inhibition of CD44 with a monoclonal antibody or MCT1 with a chemical inhibitor suppressed the anchorage-independent growth or invasive activity of our CSC-like derivative cells. Moreover, a high expression level of *CD44* and/or *SLC16A1* was correlated with a relatively unfavorable survival in pancreatic cancer patients (Fig. 6a, c). Therefore, simultaneous targeting of CD44 and MCT1 may be a judicious strategy for pancreatic cancer patients with recurrence after conventional chemotherapy such as gemcitabine treatment.

## Methods

### Cell culture conditions

Panc0203 (ATCC, CRL-2553), Panc0327 (ATCC, CRL-2549), and pancreatic derivative cells (SP0926, SP1030 and SP0913^1A, 1B & 1C^) were grown in RPMI 1640 supplemented with 10 units/ml human recombinant insulin (Merck, 91077 C), 15% fetal bovine serum (FBS), and 1% penicillin-streptomycin (PS) solution. Human pancreatic normal fibroblasts (n-fibroblast; VitroBioPharma, SC00A5) and pancreatic CAFs (Vitro BioPharma, CAF08) were cultured in MSC-GRO low serum medium (Neuromics, PC00B1) with 1% PS solution as per the procedure recommended by provider. Confluent culturing adherent cells at greater than 80% were split 1:2 into new 100-mm culture dishes. Human monocytic cell line U937 cells (ATCC Cat# CRL-1593) were maintained in RPMI 1640 that included 10% FBS and 1% PS solution. To induce differentiation of monocytic U937 cells into macrophage-like cells (MØ-U937), phorbol 12-myristate 13-acetate (PMA; Sigma-Aldrich) dissolved in DMSO was added to 5 ×10^5^ cells/ml at a final concentration of 10 ng/ml for 72 h. All cells were maintained in a humidified atmosphere with 5% carbon dioxide at 37 °C as a standard culture condition.

### TME mimic model system

The following is a description of the gemcitabine-treated TME (s-TME) model. To collect the first conditioned medium (CM) as an Initial Culture Phase comprising exposure to hypoxia, n-fibroblasts and CAFs of greater than 90% confluence were cultured separately on 100-mm culture dishes for 1.5 days without gemcitabine treatment in RPMI 1640 medium that included 15% exosome-free (exo-free) FBS (System Biosciences), 10 units/ml human recombinant insulin, and 1% PS and using a GasPak EZ Anaerobe Pouch System (BD Biosciences). Then, as a Co-culture Phase giving the opportunity for interaction between n-fibroblasts and CAFs, cells of the two types were mixed (1:1) following trypsinization and allowed to continue to grow under hypoxia for an additional 1.5 days on a 100-mm culture dish until collection of the first CM. The culture dish contained a total of 20 ml of mixed medium obtained from the Initial Culture Phase after adding exo-free FBS (2.5% v/v) to provide fresh nutrient sources. For collection of the second CM, independently prepared culture dishes were treated with 10 μM of gemcitabine (TCI) at the beginning of the Initial Culture Phase for 1.5 days. Other than the Gemcitabine treatment, there is no difference between first CM and second CM acquisition process. Then, at the end of the Co-culture Phase for both the first CM and second CM collection, the co-culturing fibroblasts were scraped out from the culture dishes with cell lifters (Corning), and then the cell-containing media were centrifuged at 2500 rpm at room temperature for 5 min following gentle agitation for 30 min at 37 °C. Every supernatant was transferred to a new conical tube, and fresh exo-free FBS was added to a final concentration of 10% (v/v). The first CM and second CM samples collected were immediately used in the following the Pre-Incubation Phase and Seeding Phase, sequentially (Fig. 1a).

The first CM was added into 100-mm culture dishes paired with maternal pancreatic cancer cells (Panc0203 or Panc0327) and MØ-U937 cells, and then the paired dishes were cultured under hypoxic (Panc0203 and MØ-U937) or normoxic (Panc0327 and MØ-U937) condition for 1.5 days as a Pre-incubation Phase mimicking exposure of cancer cells and macrophages to biogenic substances derived from stromal fibroblasts without chemotherapy. Then, the first CM-exposed Panc0203 (or Panc0327) and MØ-U937 cells in the paired tissue-culture dishes were pre-stained with 5 μM of CellTracker Orange CMTMR and Green CMFDA (Invitrogen), respectively, for 45 min in serum-free medium under the standard culture condition. After trypsinization, the first CM-exposed MØ-U937^CMFDA^ cells were washed three times with phosphate buffered saline (PBS), re-suspended in the second CM, and transferred onto another dish with the first CM-exposed/PBS-washed (three times) Panc0203^CMTMR^ (or Panc0203^CMTMR^) cells. The mixed cells were co-cultured under hypoxic (Panc0203^CMTMR^ and MØ-U937^CMFDA^) or normoxic (Panc0327^CMTMR^ and MØ-U937^CMFDA^) condition for an additional 1.5 days, termed a Seeding Phase, mimicking a gemcitabine-exposed stromal environment.

To generate SP0926 and SP0913^1A, 1B & 1C^ cells following DFCs isolation, single pair of 100-mm culture dishes for each group was prepared by culturing MØ-U937 cells and maternal cancer cells (Panc0203 or Panc0327 cells) at the beginning the Pre-incubation Phase, and all sorted DFCs through FACS from each group were seeded on a 100-mm culture dish until grown to more than 60% confluency. To generate SP1030 cells, five pairs of 100-mm culture dishes were used in which Panc0203 and MØ-U937 cells are being cultured, and all isolated DFCs were seeded on a T-75 flask. It took 129, 92, 61, 68, and 72 days to generate SP0926, SP1030, SP0913^1A^, SP0913^1B^, and SP0913^1C^, respectively. STR analysis was then performed using each of the first stocks stored in a liquid nitrogen tank.

The following is a description of the normal co-culture TME condition. Compared with the s-TME model, the only difference of the normal co-culture TME condition was a normoxic/gemcitabine free condition in the process of obtaining the first and second CMs, which were then sequentially used in the Pre-incubation and Seeding Phases of the paired Panc0203 cells with MØ-U937 cells under normoxia. Note, fresh exo-free FBS was added to a final concentration of 2.5% (v/v) in first CM and 10% (v/v) in the second CM.

To observe the blockade effect of exosome biogenesis/release and TNTs formation, 10 μM GW4869 (Sigma), 10 μM ML-141 (Calbiochem) or 100 nM Cytocalasin B (abcam) was pre-treated to each n-fibroblasts and CAFs 24 h prior to the Initial Culture Phase, then the inhibitors were also applied to them at the beginning the Initial Culture Phase for the first CM and second CM generation. Each Panc0203 and MØ-U937 cells were also pre-exposed for 24 h to the inhibitors, and then the prepared first CM was treated to each Panc0203 and MØ-U937 cells and the second CM were treated to the mixed cell plate of Panc0203^CMTMR^ and MØ-U937^CMFDA^ cells, by sequence (Fig. 1a). Note, for every trypsinization step, take the trypsinized cell plates out of the incubator every few minutes and tap it to help the cells fall off sufficiently, avoiding the use of a cell lifter and exposure to light.

### Fluorescence-activated cell sorting

The whole cultured media, including ‘suspending (detached) cells’ considered as damaged cells in the s-TME model, from the culture plates at the end of the Seeding Phase (Fig. 1c, Supplementary fig. 1a) were collected into 50 ml conical tube(s) and the pelleting cells were washed 3 times by PBS following centrifugation at 1500 rpm for 5 min. The cells were resuspended in ice-cold FACS buffer (0.1% bovine serum albumin and 0.01% sodium azide in 1 x PBS). The ‘adherent cells’ considered as viable cells at the end of the Seeding Phase of the s-TME model (Fig. 1c and Supplementary fig. 1a) were trypsinized, and washed three times by PBS following centrifugation at 1500 rpm for 5 min. The pelleting cells were resuspended by ice-cold FACS buffer. Note, take the trypsinized cell plates out of the incubator every few minutes and tap it to help the cells fall off sufficiently, avoiding the use of a cell lifter and exposure to light. After passing the ‘suspending cells’ and ‘adherent cells’ through the strainer cap, stored on ice in a dark condition until the scheduled time for analysis.

To isolate DFC (CMFDA- and CMTMR-positive) populations, FACS was performed using a FACSAria IIu cell sorter (BD Biosciences) according to the manufacturer’s instructions. Briefly, FITC and PE channels were used for detecting CellTracker™ Green CMFDA (Ex/Em: 492/517 nm) and CellTracker™ Orange CMTMR (Ex/Em: 541/565 nm), respectively, following fluorescence compensation (Supplementary fig. 1a).

Flow cytometry for detecting CD24 and CD44 of Panc0203 and derivative cells was performed by the procedure for the ‘adherent cells’ described above. Briefly, after washing with PBS, the cells were incubated for 45 min with anti-human antibodies CD24 PE (BioLegend, 311106) and CD44 APC (BioLegend, 338806) diluted (1:50) in FACS buffer.

### Nanoparticle tracking analysis

NanoSight NS300 (NanoSight) was used for nanoparticle tracking analysis (NTA) measurements according to the manufacturer’s instructions using the purified s-TME CMFDA/DMSO-Exo and s-TME CMFDA/GW4869-Exo from total 20 ml volume of second CM. The data were collected following 3-time repeated running for taking exosome captures per each sample. Concentration (particles/ml) average and mean diameter (nM) within the range of 0–400 nm were used for data presentation. NTA Version: NTA 3.2 Dev Build 3.2.16, camera type: sCMOS, number of frames: 749, time: 30 seconds, temperature: 25°C, dilution factor: 5.

### Quantification of exosome fluorescence intensity

For exosome fluorescence quantification, Varioskan LUX Multimode Microplate Reader (Thermo Fisher) was used. CMFDA (Ex/Em: 492/517 nm) fluorescence signal quantification was performed according to the manufacturer’s instructions using the purified s-TME non-fluorescent(fluor)/DMSO-EXO, s-TME CMFDA/DMSO-Exo and s-TME CMFDA/GW4869-Exo from total 20 ml volume of second CM. Briefly, each diluted exosome samples (100 μl, dilution factor: 5) in PBS were loaded into a 96-well black plate (SPL) and fluorescence signals were read by operating Skanlt software 6.1.1 (Thermo Scientific) linked to Varioskan LUX Multimode Microplate Reader.

### Confocal microscopy

Plated cells at about 60% confluence were grown on Lab-Tek 2-chamber glasses (Nunc) in the normal culture medium under standard culture conditions. The cells were fixed with 3.7% formaldehyde solution for 15 min, followed by washing with PBS three times and incubation in PBS with 0.1% BSA for 45 min. After washing with PBS, the cells were incubated for 45 min with anti-human antibodies CD24 PE (BioLegend, 311106) and CD44 APC (BioLegend, 338806) diluted (1:20) in PBS with 0.1% BSA. The CD24 and CD44 specimens were mounted on microscope slides, and expression was observed with an LSM 880 AiryScan (Zeiss).

For the exosome treatment experiment, s-TME CMFDA/DMSO-Exo and s-TME CMFDA/GW4869-Exo were treated onto Panc0203 cells and maintained in the standard culture condition for 20 h.

### Short tandem repeat analysis

Purified genomic DNA of U937 cells, maternal PDAC cells (Panc0203, Panc0327), and pancreatic derivative cells (SP0926, SP1030, and SP0913^1A, 1B & 1C^) were used for STR analyses using an AmpFLSTR™ Identifier™ PCR Amplification Kit (Applied Biosystems). Amplified PCR products were separated by capillary electrophoresis on a 3530xL DNA analyzer (Applied Biosystems) according to the manufacturer’s instructions and analyzed using GeneMapper v5 software (Applied Biosystems).

### In vitro proliferation and viability assay

Five-thousand and 2.5 × 10^3^ cells were seeded into 96-well plates for either a cell proliferation or viability assays. The numbers of live cells were estimated using an EzCytox WST assay kit (Daeillab) at 0, 24, 48, and 72 h and measurement was performed by microplate spectrophotometer (Agilent) at OD 450 nm. The relative cell proliferation and viabilities were estimated at each time point via comparison to, respectively, the number of viable cells at the initial plating time (0 h), and the number of viable cells in the drug-untreated control cultures at the corresponding times. For hypoxic (15% FBS) and LOS (2% FBS, in vitro ischemic) conditions, cells were cultured in a GasPak EZ Anaerobe Pouch System (BD Biosciences).

For the exosome treatment experiment, 2.5 × 10^3^ Panc0203 cells were seeded into 96-well plates and s-TME CMFDA/DMSO-Exo and s-TME CMFDA/GW4869-Exo were treated for 24 h. Gemcitabine (250 μM) was applied for observing the effect on each exosome-treated group.

### Aldefluor assay

An ALDEFLUOR assay kit (STEMCELL technologies) was used in accordance with the manufacturer’s instructions. Briefly, trypsin-treated Panc0203, SP0926 and SP1030 cells were collected followed by wash in FACS buffer. Total 3 × 10^6^ cells per 1.5 ml Aldeflour buffer including 7.5 μl activated Aldefluor reagent. For the DEAB-treated control, total 1 × 10^6^ cells per 0.5 ml were relocated from the original tubes to an e-tube including 20 μl DEAB, immediately. Then, both e-tubes were incubated at 37 °C for 40 min followed by ice incubation. The incubated cells were washed two times with Aldefluor buffer through centrifugation all tubes for 5 min at 250×*g*. Resuspended cells in 200 μl Aldefluor buffer were used for Flow cytometry which was performed using an LSRFortessa cell analyzer (BD Biosciences, 520–540 nm green fluorescence channel) to detect ALDH activity in the cells.

### Tumorsphere assay

In the standard culture condition, 5 × 10^3^ cells were incubated in ultra-low attachment, 6-well plates (Corning) containing DMEM/F12, HEPES (Gibco), which included B-27 (Gibco), and 1% PS solution, without disturbing the plates for 6–11 days. For the second-round experiment, the first-round tumorspheres were trypsinized to generate single cells followed by re-suspension with a syringe with a 31-G needle (BD). For CD24 and/or CD44 neutralization, 1 μg of anti-human CD24 antibody (BioLegend, 311102) and 0.15 μg anti-human CD44 antibody (BioLegend, 338802) were added as treatment into each well. To observe the effect of the MCT1 inhibitor, 20 μM of AZD3965 was added as treatment to each cell line. Spheroids were observed microscopically, and the number and diameter of tumorspheres (per field or total) were analyzed on ImageJ.

### Invasion assay

Cells in serum-free RPMI 1640 (1 × 10^5^ cells / 200 μl) were added to the upper compartment of Millicell inserts of 8.0-μm pores and 12-mm diameter (Merck Millipore) that were pre-coated with 200 μl (0.4 mg) of Matrigel (BD Biosciences). The basal compartment were filled with 750 μl of RPMI 1640 including 15% FBS, 1% PS solution, and 10 units/ml of human recombinant insulin. In order to observe the effect of the MCT1 inhibitor, 20 μM of AZD3965 (MedChemExpress) was added to each cell line at the seeding step in the prepared inserts and the basal compartment of the 24-well plate, respectively. To observe the effect of CD24 overexpression, pRP-hCD24 plasmid (Vectorbuilder) was transiently transfected into SP0926 cells. In order to observe the CD24 and CD44 inhibition effect, anti-human CD24 antibody (BioLegend, 311102) was added into the insert (0.4 μg/200 μl) and basal compartment (0.6 μg/750 μl), and anti-human CD44 antibody (BioLegend, 338802) was added into the insert (60 ng/200 μl) and lower compartment (90 ng/750 μl) for CD44 inhibition. Mouse IgG was used as a control (Invitrogen, 31903). After overnight or 24 h of incubation, the remaining cells on the apical side of the insert membrane were removed by cotton swabs, fixed in 3.7% formaldehyde for 2 min, and then exposed to 100% methanol permeabilization for 10 min followed by trypan blue staining for 20 min. Cells were washed with PBS three times between every step.

### RNA sequencing

MØ-U937, Panc0203, SP0926, and SP1030 cells were prepared for RNA sequencing. A TruSeq Stranded mRNA LT Sample Prep Kit (Illumina) was used according to the manufacturer’s library protocol. Sequencing of the QC-passed libraries was performed on the NovaSeq 6000 sequencing system (Illumina) in paired-end with 101-nt read length. The pre-processed trimmed reads were mapped to the genomic DNA reference UCSC hg 19 (original GRCH3 7 from NCBI, Feb. 2009) using the HISAT2 program, which is capable of splice junction processing. After reading the mapping, transcript assembly was performed using the StringTie program. As a result, the expression profile values for each sample were obtained for the known transcripts, and the read count and FPKM (Fragment per Kilobase of transcript per Million mapped reads) were acquired based on the transcript/gene. From the original raw data (27,685 genes, 4 samples), processed data (14,657 genes, 4 samples) were obtained following exclusion of genes with an FPKM value of 0 in at least one of the four samples and by performing DEG (differentially expressed genes) analysis containing 7,360 significant genes that satisfy conditions of ≥2-fold change and <0.05 *P*-value conditions in at least one of the total comparison pairs, followed by hierarchical clustering (Euclidean distance, complete linkage). Fusion genes were analyzed by SOAPfuse, Defuse, FusionCatcher and STAR-Fusion based on RNAseq data, and compared with the results of DNA structural variants analyzed by Manta and Breakdancer following whole genome sequencing.

### Gene set enrichment analysis

To identify gene sets enriched in derivative cells compared to mother Panc0203 cells, gene set enrichment analysis (GSEA) was performed using the javaGSEA desktop application (GSEA v4.0.3)^69^. For this analysis, the hallmark gene sets were applied using pre-ranked options derived from the rank of fold changes of derivative cells compared to Panc0203 cells. *P*-values were calculated by permuting the data 1000 times to find enriched gene sets. The GSEA software produced enrichment score (ES), normalized ES (NES), nominal *P*-value, and false discovery rate (FDR; *Q*-value). Gene sets that were up- or down-regulated with a Q-value of <0.05 were considered significant. For RNA sequencing, GSEA was performed using data from the TCGA Pancreatic Cancer (PAAD) database (*n* = 182, http://xena.ucsc.edu/), using the same parameters by dividing samples into two groups based on the median expression level of CD24, CD44, or MCT1.

### Whole genome sequencing

For whole genome sequencing, libraries of Panc0203, SP0926 and SP1030 genomic DNAs were prepared according to the Illumina TruSeq DNA PCR-free library preparation guide and sequenced using Illumina HiSeq X sequencer following quality control and quantification with 151nt read length, which was performed by Macrogen. After generation of raw data through an integrated primary analysis software called RTA 2 (Real Time Analysis 2), next-generation sequencing data was aligned by Isaac Aligner 01.15.02.08., the mapping reference was the hg19 from UCSC, and analyzed by IVC (Isaac Variant Caller 2.0.13), SnpEff 3.3, Control-FREEC 6.4, Manta 0.20.2 and Breakdancer.

### Quantitative real-time PCR

Total RNA was purified using a RNeasy Plus Mini kit according to the manufacturer’s instructions (Qiagen). Total RNA (1 μg) was transcribed into complementary DNA using a Maxime RT PreMix (Intron Biotechnology) for 1 h at 45 °C. Quantitative real-time PCR was performed using SYBR Green PCR Master Mix (Applied Biosystems). Glyceraldehyde 3-phosphate dehydrogenase (GAPDH) was used as an internal control for normalization. The primer sequences for the total form h*CD44*, h*CD24* and h*SLC16A1* are listed below. Total form h*CD44* (forward; 5′- CCGCTATGTCCAGAAAGGA -3′, reverse; 5′- CTGTCTGTGCTGTCGGTGAT-3′), h*CD24* (forward; 5′-GACTCAGGCCAAGAAACGTC-3′, reverse; 5’-CCTGTTTTTCCTTGCCACAT-3′), and h*SLC16A1* (forward; 5′- GTGGCTCAGCTCCGTATTGT-3′, reverse; 5′- GAGCCGACCTAAAAGTGGTG-3′).

### Fusion gene PCR

Fusion genes were validated at both DNA (Supplementary Fig. 2d) and mRNA (data now shown) levels through fusion junction PCR and Sanger sequencing. PCR amplification of each fusion gene of genomic DNA was performed using primer sets listed below. BCL2L1/GLB1L2 fusion gene (forward; 5′- GGTAGTACCCACTGACAGAGTG-3′, reverse; 5′- CTCTTTCCCAGTCCCACCTC-3′), CABIN1/CSNK1E fusion gene (forward; 5′-CCTCCCCTAGCCCTGTGG-3′, reverse; 5′-CAGGGGAGCCTGCGTCATC-3′), TPTEP2-CSNK1E readthrough/ KIAA1671 fusion gene (forward; 5′-CTGGCTTGTGTCTGAACTG-3′, reverse; 5′-GATGGAGTGAGACTCCATCTC-3′).

### Mass spectrometry for peptide analysis

Exosome of CMs collected from in vitro TME models (s-TME model and normal co-culture condition using fibroblasts, CAFs, MØ-U937 and Panc0203 cells) by total exosome isolation reagent (Invitrogen). Whole cell lysates of Panc0203, SP0926, and SP1030 cells, which were prepared in sample buffer (7 M Urea, 2 M Thiourea, 100 mM DTT, 4.5% CHAPS, 40 mM Tris). Peptides were analyzed following two-dimensional gel electrophoresis (2-DE) and liquid chromatography-tandem mass spectrometry (LC-MS/MS) methodologies. Nano LC-MS/MS analysis was performed with an Easy n-LC (Thermo Fisher Scientific) and an LTQ Orbitrap XL mass spectrometer (Thermo Fisher Scientific) equipped with a nano-electrospray source. Samples were separated on a C18 nanobore column (150 mm × 0.1 mm, 3 μm pore size; Agilent). The mobile phase A for LC separation was 0.1% formic acid and 3% acetonitrile in deionized water, and the mobile phase B was 0.1% formic acid in acetonitrile. The chromatography gradient was designed for a linear increase from 0% B to 60% B in 9 min, 60% B to 90% B in 1 min, and 3% B in 5 min. The flow rate was maintained at 1,800 nl/min. Mass spectra were acquired using data-dependent acquisition with a full mass scan (380–1,700 m/z). For database searching, the Mascot algorithm (Matrixscience) was used to identify peptide sequences present in a protein sequence database. The peptides were filtered with a significance threshold *P*-value of <0.05. Database: nr_Human_20180410_curated (1249071 sequences; 414470512 residues).

### Enzyme-linked immunosorbent assay

Lysates from Panc0203, SP0926, and SP1030 cells were used for ELISA, which was performed using kits for PGK1 (BIOMATIK) according to the manufacturer’s instructions. Conditioned media and exosomes, which were collected from both the normal co-culture TME condition and the s-TME models and conducted ELISA for detecting stratifin (LifeSpan BioSciences) and LGALS3BP (LifeSpan BioSciences). Briefly, prepared samples (100 μl) were added into each well followed by incubation for 2 h at 37 °C. After aspiration, detection reagent A (first detection antibody) was added to each well followed by incubation for 1 h at 37 °C. Following 3 times wash, detection reagent B (horseradish peroxidase-linked second detection antibody) was added and incubated for 1 h at 37 °C. TMB substrate solution was treated following 5 times wash, and observation was conducted until the color change of the standard and sample solutions is observed. After adding stop solution, the colorimetric absorbance was read at 450 nm.

### Stable cell-line generation

Maternal pancreatic cells and pancreatic derivative cells were transfected with GFP shRNA, human CD24 shRNA plasmids (Santa Cruz) using Lipofectamine 2000 (Invitrogen) and were then selected by 0.4 μg/ml of puromycin (Gibco) until stable cell-line generation. CD24 and CD44 levels on the cells were analyzed by LSRFortessa cell analyzer using the anti-human antibodies CD24 PE and CD44 APC, following cell harvest and PBS rinse.

### l-lactate assay

The specific measurement and analysis of l-lactate in collected media and cell lysates were performed by using L-lactate assay kit (Colorimetric) (Abcam) according to the manufacturer’s instructions. Panc0203, SP0926 and SP1030 cells were cultured for 72 h in standard culture condition following 3.5 × 10^5^ cells seeding per six-well of plates. l-lactate concentration and amount were measured using the collected media and the lysates of 1 × 10^6^ collected cells. Particularly, cells were harvested in 200 μl of lactate assay buffer (Abcam) generating cell lysates after wash with cold PBS, then each 50 µL sample (of the collected media and the lysates) per well was used for assay. Plates were incubated at room temperature for 30 min and measurement was conducted by a microplate spectrophotometer (Agilent) at OD 450 nm.

### Western blotting

Panc0203, SP0926, and SP1030 cells were lysed in RIPA buffer (Thermo Scientific) containing protease inhibitor cocktail (Roche), and were centrifuged at 13,000 × *g* for 10 min at 4 °C. After protein quantification by the BCA method (Thermo Scientific), 20 μg of protein were resolved by SDS-PAGE and transferred to a nitrocellulose membrane followed by blocking with 5% skim milk in Tris-buffered saline for 1 h, and were incubated with anti-MCT1 Polyclonal Antibody (1:1000 dilution in 10 ml of Tris-buffered saline with 0.1% Tween® 20 detergent (TBST) with 5% BSA) (Thermo Fisher, A304–357A). The membranes were washed and incubated with horseradish peroxidase-conjugated secondary rabbit antibody, followed by enhanced chemiluminescence development according to the manufacturer’s instructions (Pierce). Western blot quantification was performed by ImageJ software. Note, MCT1 was detected as monomer forms (43–48 kDa) and a dimer form (95–100 kDa)^70–72^, when compared to ladder sizes of the used protein marker (PageRuler™ prestained protein ladder, Thermo Fisher, 26616). All blots were derived from the same experiment and were processed in parallel.

### Statistics

All statistical analysis was performed using Prism 8 (GraphPad). Error bars on graphs display the mean and SD. One-way ANOVA followed by Tukey’s or Dunnett’s multiple comparison test or unpaired *t* test were used; *P < 0.05; **P < 0.01; ***P < 0.001; ****P < 0.0001.

### Reporting summary

Further information on research design is available in the Nature Research Reporting Summary linked to this article.

## Supplementary information

Supplementary Information

Reporting Summary

Supplementary Video 1

Supplementary Video 2

## Data Availability

RNA sequencing data (.fastq.gz files) are available from the Gene Expression Omnibus (accession number GSE201876). The whole genome sequencing data for this study have been deposited in the European Nucleotide Archive (ENA) at EMBL-EBI under accession number PRJEB55995. Proteomics data are available via ProteomeXchange with identifier PXD037275.
