# Supplementary material for: Direct cell-to-cell transfer in stressed tumor microenvironment aggravates tumorigenic or metastatic potential in pancreatic cancer
Source: NPJ Genom Med. 2022 Oct 27;7:63. doi: 10.1038/s41525-022-00333-w (PMC9613679; doi:10.1038/s41525-022-00333-w)
Supplement: Supplementary file 1 — Supplementary Information [file 41525_2022_333_MOESM1_ESM.pdf]

Supplementary Information for

Direct cell-to-cell transfer in stressed tumor microenvironment aggravates tumorigenic or metastatic potential in pancreatic cancer

Giyong Jang, Jaeik Oh, Eunsung Jun, Jieun Lee, Jee Young Kwon, Jaesang Kim, Sang-Hyuk Lee, Song Cheol Kim, Sung-Yup Cho*, Charles Lee*

* Correspondence: csybio@snu.ac.kr (S.-Y.C.); Charles.Lee@jax.org (C.L.)

Supplementary appendix

Table of contents

Supplementary Figures 2

Supplementary Figure 1: Increased numbers of vacuoles or enlarged cytoplasm following DFCs isolation 2

Supplementary Figure 2: Genome characterization of pancreatic derivative cells from direct cell-to-cell transfer 5

Supplementary Figure 3: Enhanced tumorsphere formation or invasive activity of pancreatic derivative cells from direct cell-to-cell transfer 7

Supplementary Figure 4: Proteomic analysis of TME-derived exosomes 8

Supplementary Figure 5: Effect of CD24/CD44/MCT1 suppression on both tumorsphering and invasive activities of pancreatic derivative cancer cells 9

Supplementary Figure 6: Analysis of CD24, CD44, and SLC16A1 expression in pancreatic cancer-patient tissue samples 11

Supplementary Tables 12

Supplementary Table 1: Transcriptomic data of pancreatic derivative cells generated via direct cell-to-cell transfer 12

Supplementary Table 2: Proteomic analysis of the lysates of derivative cells (Panc0203, SP0926, SP1030) 172

Supplementary Table 3: Proteomic analysis of TME-derived exosomes compared to normal TME exosomes (commonly found spots) 173

Supplementary Table 4: Proteomic analysis of TME-derived exosomes compared to normal TME exosomes (Unpaired spots) 174

Supplementary Videos 175

Supplementary Video 1: Spontaneous TNTs formation from a derivative cell line. Bright-field light microscopy images of SP0926... 175

Supplementary Video 2: Densely embedded spots of green fluorescent dye acquired from MØ-U937^{CMFDA} in CMTMR-positive pancreatic cancer cells.. 175

Supplementary Figure 1

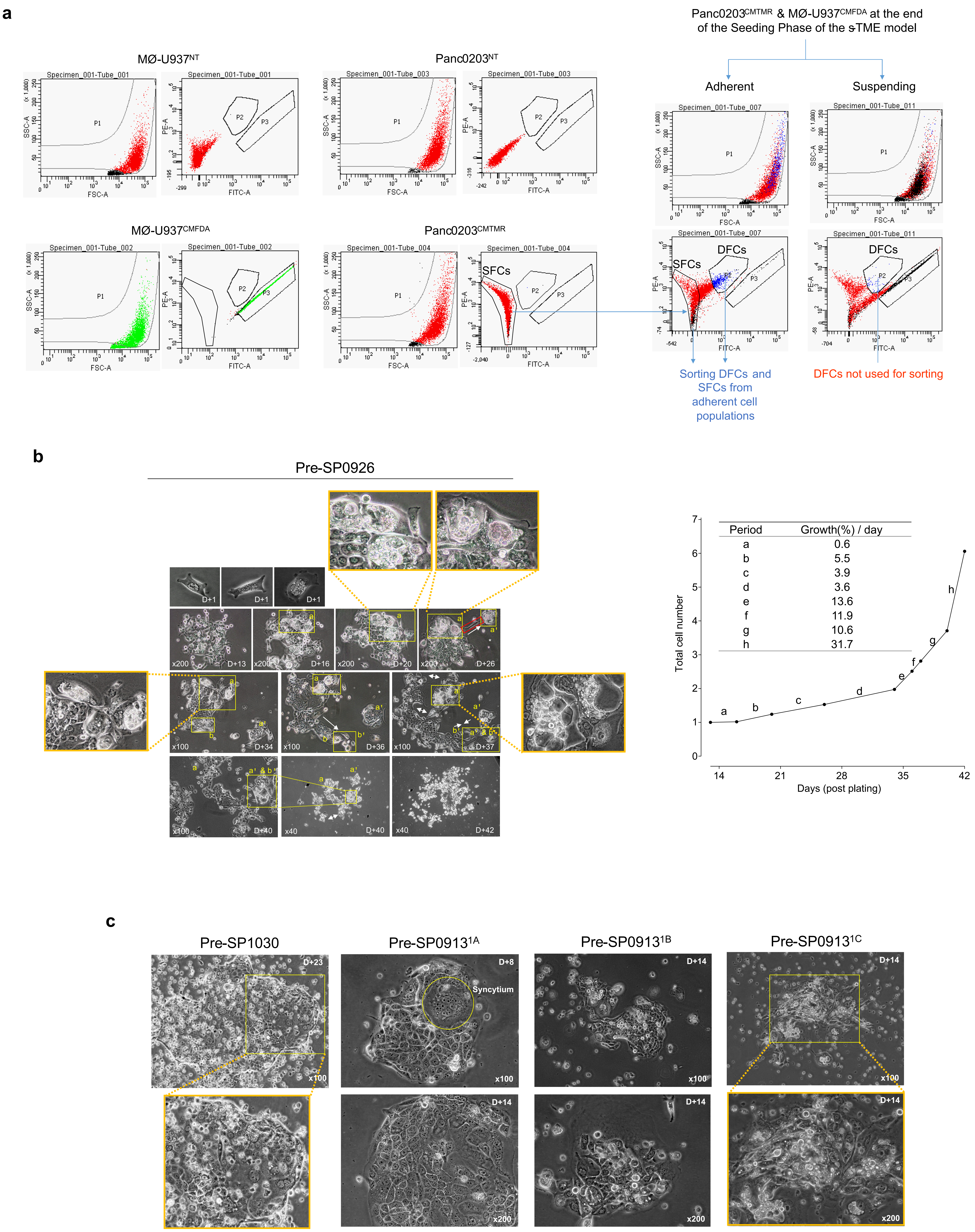

d

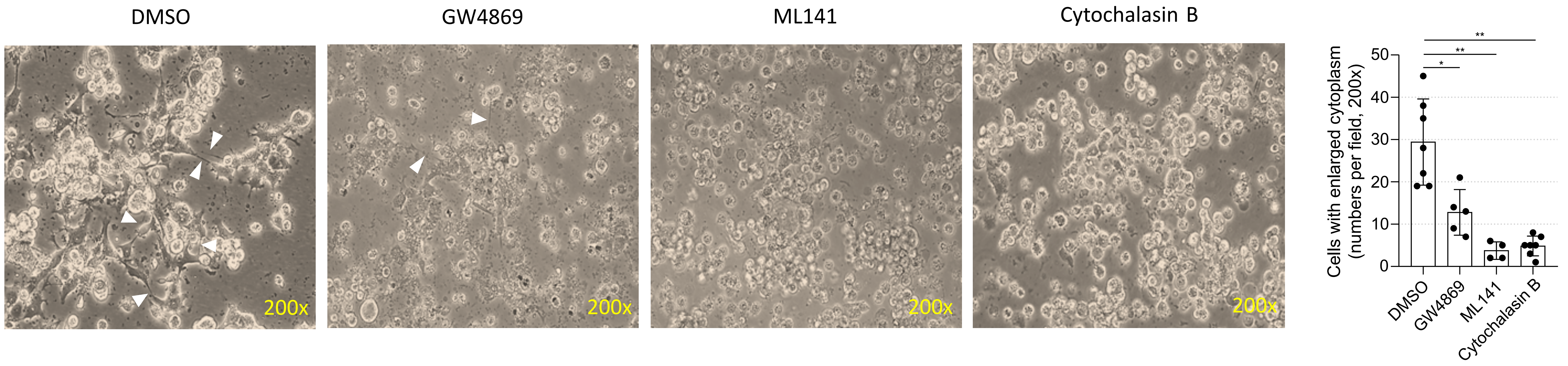

e

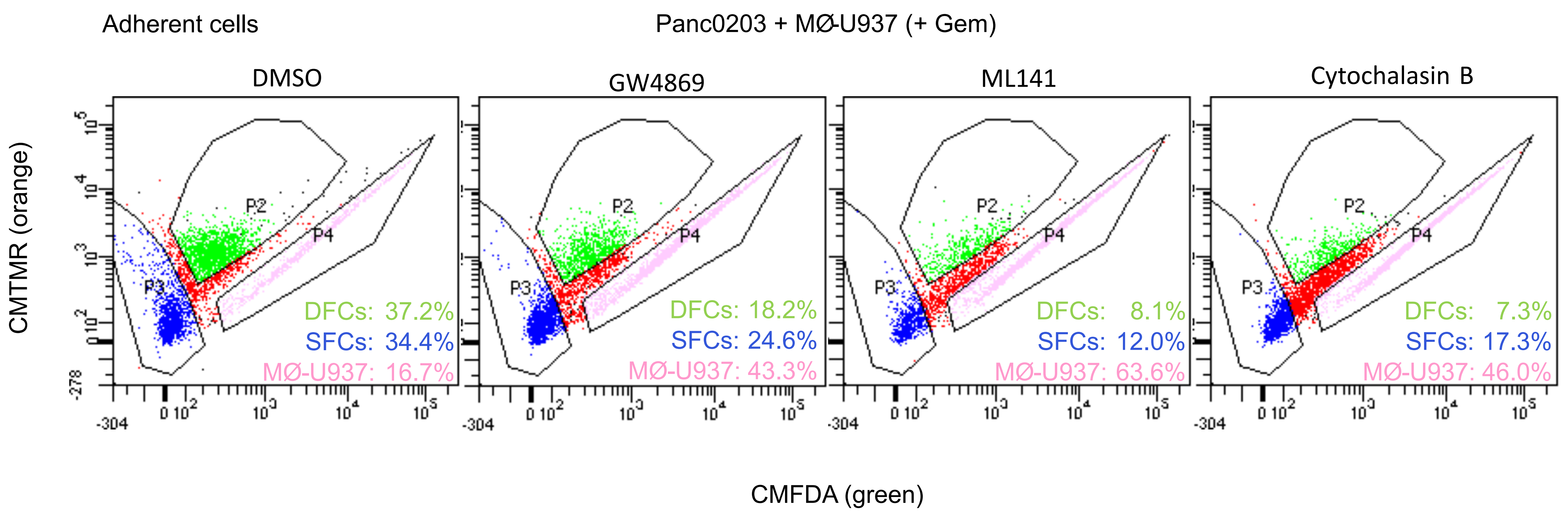

f

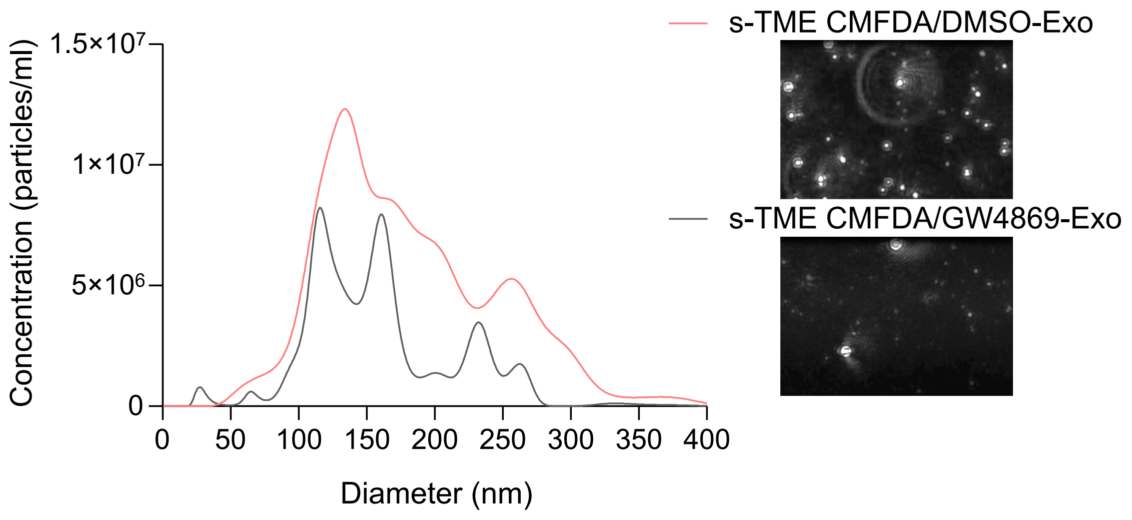

	Concentration (Mean +/- Standard Error; particles/ml)	Mean diameter (nm)
s-TME CMFDA /DMSO-Exo	$1.48*10^9 \pm 4.5*10^7$	181.4
s-TME CMFDA /GW4869-Exo	$6.56*10^8 \pm 8.75*10^7$	161.6

g

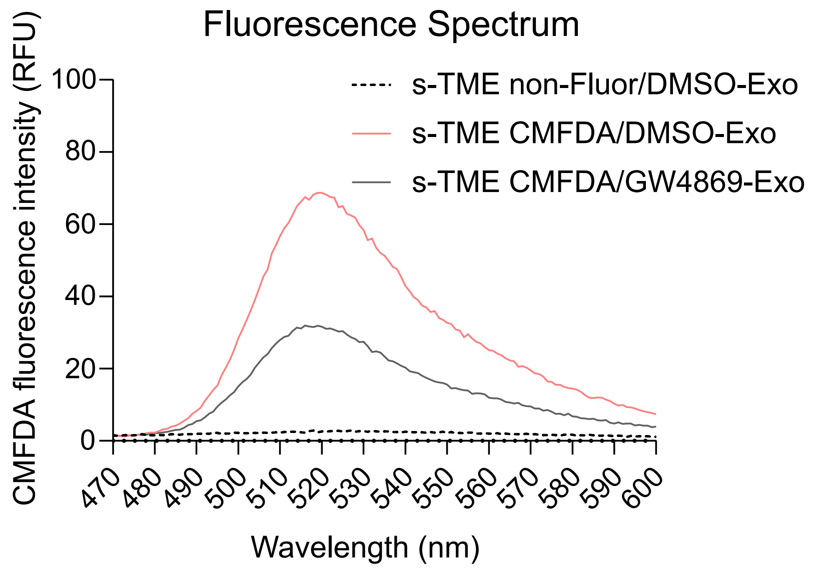

h

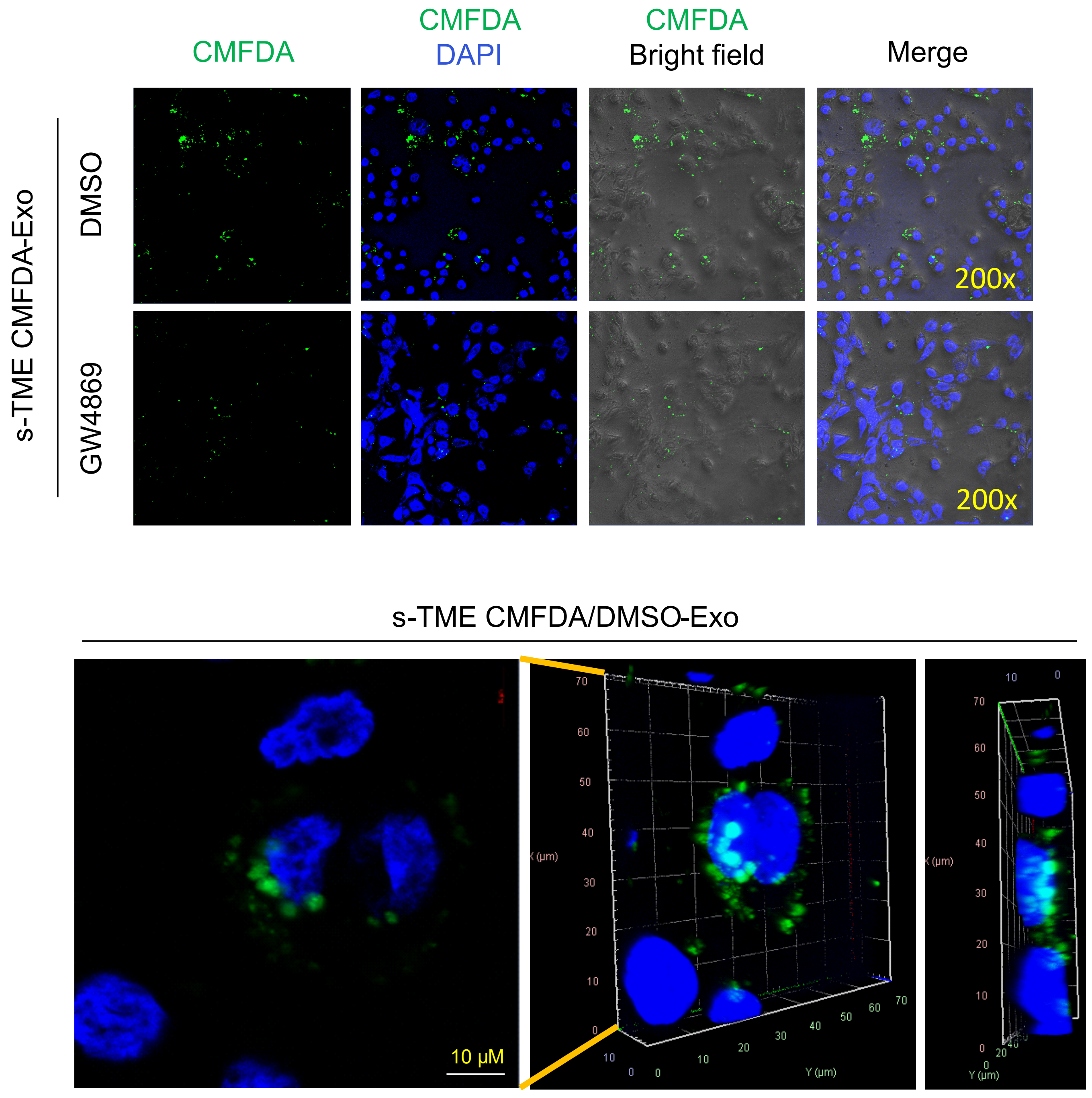

i

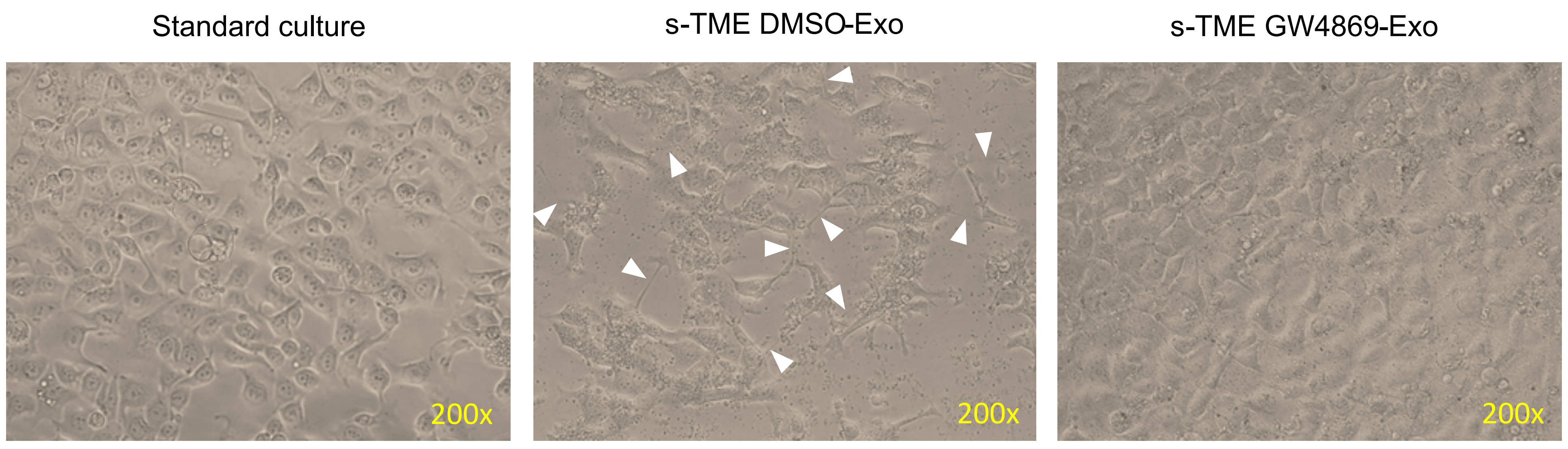

j

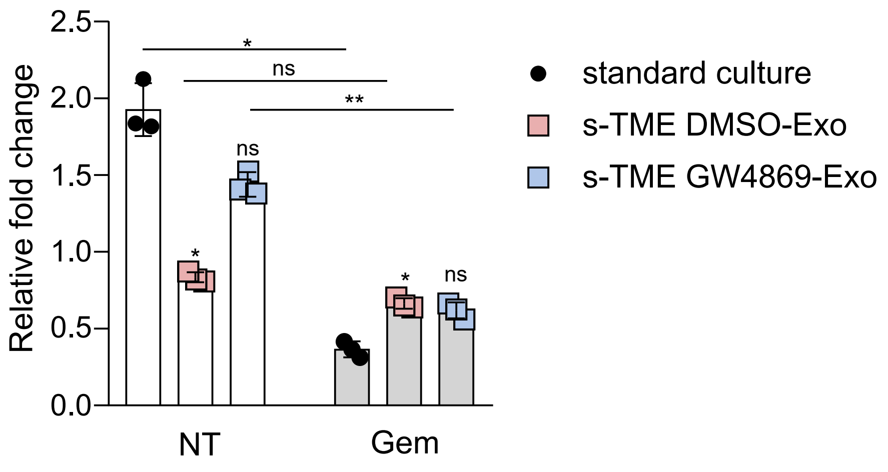

Supplementary Figure 1. Increased numbers of vacuoles or enlarged cytoplasm following DFCs isolation. a The gating strategy used for DFCs and SFCs sorting. Set up a sorting area for adherent CMFDA/CMTMR double-fluorescence positive cells (DFCs) and for adherent CMTMR single-fluorescence positive cells (SFCs) of the s-TME model. DFCs, which are exclusive against all the other cell populations (MØ-U937^{NT}, MØ-U937^{CMFDA}, Panc0203^{NT} and Panc0203^{CMTMR} cells) presented on Fig. 1c-e and Supplementary Fig. 1e. SFCs, which are exclusive against DFCs and MØ-U937^{CMFDA} cell populations presented on Fig. 1c, d and Supplementary Fig. 1e. **b** Multiple formation of bulk-bodied regions (yellow bounding boxes) and a stretching-body (a red border box) of pre-SP0926 (isolated DFCs) during a period of low growth rate (right chart) before the SP0926 cell line was established. A close-up view of one of the bulk-bodied areas is displayed in a orange border box. Arrow; migration route of proliferating cells. a', b'; a migrated cell population from a or b. **c** Multiple formation of bulk-bodied regions (yellow bounding boxes) of pre-SP colonies (sorted DFCs) during early period of time before establishment of each SP cell line (SP1030 and SP0913$^{1A, 1B, 1C}$). Close-up views of one of the bulk-bodied areas (orange border boxes). Syncytium (a yellow circle). **d** Observation of the blocking effect of exosome biosynthesis/release and TNT formation on the morphology of Panc0203^{CMTMR} and MØ-U937^{CMFDA} cells at the final point of the s-TME model before cell collection for flow cytometry. Left, bight-field light microscopy images. Arrow heads indicate TNTs. Right, One-way ANOVA test. * $P \leq 0.05$; ** $P \leq 0.01$. **e** The percentage of DFCs from the adherent cells exposed to the inhibitors (GW4869, ML141 or Cytochalasin B) in s-TME model. Flow cytometry data. 10 μM GW4869, 10 μM ML141 and 100 nM Cytochalasin B. **f** Nanoparticle Tracking Analysis (NTA) of purified exosomes. NTA was performed at 25˚C. Concentration (Mean +/- Standard Error; particles/ml). Mean diameter (nM). s-TME CMFDA/DMSO-Exo (purified exosomes from conditioned media of CMFDA/DMSO-treated s-TME model), s-TME CMFDA/GW4869-Exo (purified exosomes from conditioned media of CMFDA/GW4869-treated s-TME model). **g** Quantification of exosome fluorescence intensity. CMFDA (Ex/Em: 492/517 nm) fluorescence signal quantification was performed and the spectrum is presented within 470-600 nm range. s-TME non-flour/DMSO-Exo (purified exosomes from conditioned media of non-flurescent/DMSO-treated s-TME model), s-TME CMFDA/DMSO-Exo (purified exosomes from conditioned media of CMFDA/DMSO-treated s-TME model), s-TME CMFDA/GW4869-Exo (purified exosomes from conditioned media of CMFDA/GW4869-treated s-TME model). **h** Confocal images of exosome-transferred cells (Green spots: s-TME CMFDA/DMSO-Exo or s-TME CMFDA/GW4869-Exo). Confocal images of Panc0203 after s-TME CMFDA/DMSO-Exo treatment for 20 h. Z-stack views in the same field: scale bar, 10 μm. **i** Bright-field light microscopy images of normal Panc0203 cells and the Panc0203 cells exposed to s-TME DMSO-Exo (purified exosomes from conditioned media of DMSO-treated s-TME model) or s-TME GW4869-Exo (purified exosomes from conditioned media of GW4869-treated s-TME model) for 20 h. Arrow heads indicate TNTs. **j** Proliferation and viability was observed 24 h after s-TME DMSO-Exo or s-TME GW4869-Exo treatment. The reference value for calculating the relative fold change is the value obtained at 0 h control in Panc0203 cells. Gemcitabine (250 μM). * $P \leq 0.05$; ** $P \leq 0.01$. One-way ANOVA test. Data are presented as the mean values ± standard deviation (SD).

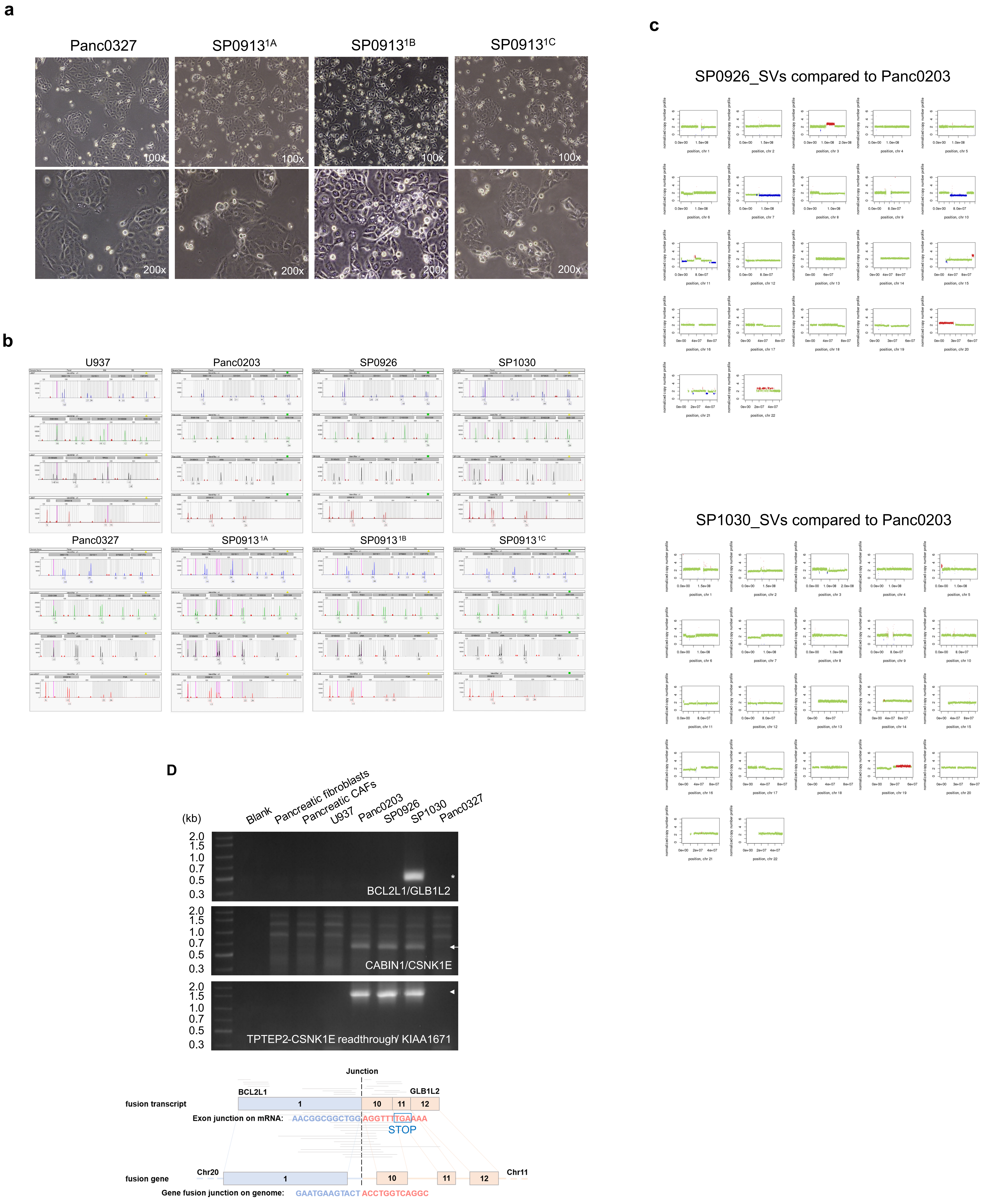
a
Panc0327
SP0913 1A
SP0913 1B
SP0913 1C
100x
200x
b
U937
Panc0203
SP0926
SP1030
Panc0327
SP0913 1A
SP0913 1B
SP0913 1C
c
SP0926_SVs compared to Panc0203
SP1030_SVs compared to Panc0203
D
(kb)
Blank
Pancreatic fibroblasts
Pancreatic CAFs
U937
Panc0203
SP0926
SP1030
Panc0327
2.0
1.5
1.0
0.7
0.5
0.3
BCL2L1/GLB1L2
CABIN1/CSNK1E
TPTEP2-CSNK1E readthrough/ KIAA1671
Junction
BCL2L1
GLB1L2
fusion transcript
Exon junction on mRNA:
AACGGCGGCTGG AGGTTTTGAAAA
STOP
fusion gene
Chr20
Chr11
Gene fusion junction on genome:
GAATGAAGTACT ACCTGGTCAGGC

Supplementary Figure 2. Genome characterization of pancreatic derivative cells from direct cell-to-cell transfer. a Representative morphologic and spatial patterns of Panc0327 cells and derivative cell lines. Phase images show a less dispersed growth pattern of $SP0913^{1B}$ cells compared to that of Panc0327, $SP0913^{1A}$, and $SP0913^{1C}$ cells. **b** STR profile was detected by primers that amplified the 15 STR loci and the gender marker Amelogenin. **c** Copy number alterations (Red: gains; Blue: losses) of derivative cells (SP0926 and SP1030 cells) compared to Panc0203. **d** A novel interchromosomal (chr20-chr11) BCL2L1/GLB1L2 (BG) fusion gene of SP1030 was identified (upper). Positive fusion gene controls for Panc0203, SP0926 and SP1030; CABIN1/CSNK1E and TPTEP2-CSNK1E readthrough/KIAA1671 fusion genes, asterisk; BCL2L1/GLB1L2 fusion gene, arrow; CABIN1/CSNK1E fusion gene, arrow head; TPTEP2-CSNK1E readthrough/KIAA1671 fusion gene. BCL2L1-GLB1L2 fusion junction on genomic DNA and mRNA of SP1030 (bottom).

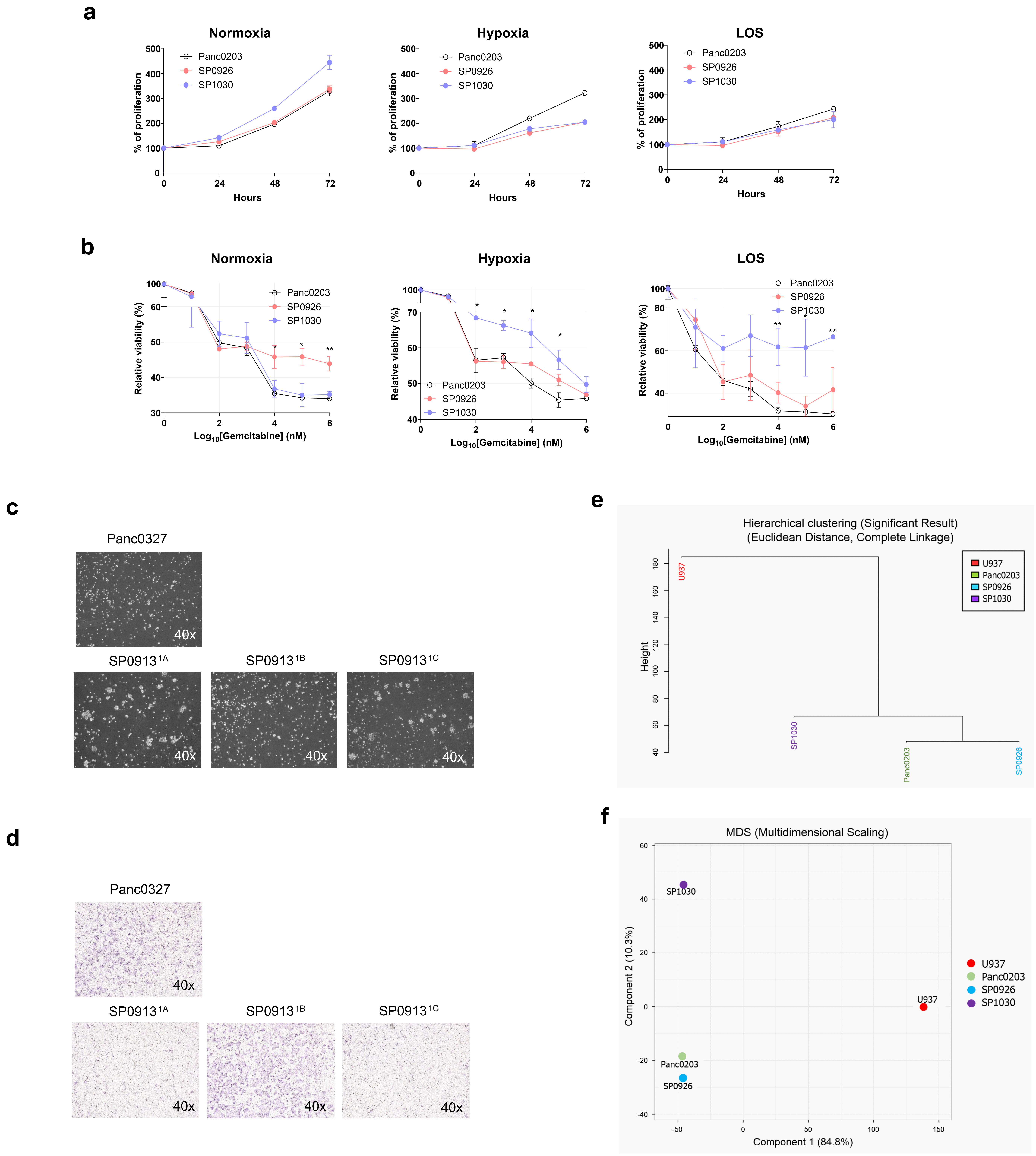

Supplementary Figure 3. Enhanced tumorsphere formation or invasive activity of pancreatic derivative cells from direct cell-to-cell transfer. a, b Proliferation under a gemcitabine free culture condition (**a**) and viability against gemcitabine at 48 h (**b**), together with normoxic, hypoxic, or low oxygen and serum (LOS) condition. * $P \leq 0.05$; ** $P \leq 0.01$; *** $P \leq 0.001$. One-way ANOVA followed by Tukey's multiple comparison test. Data are represented as the mean value ± SD. **c** Representative images of tumorsphere formation in Panc0327 and derivative cells. Spheroids were evaluated on day 7 after seeding. **d** Invasion activity of Panc0327 cells and derivative cells. An invasion chamber assay system containing Matrigel was performed for 24 h. The initial seeding number of cells for each group was 1×10^5. Upper chamber: serum-free medium, bottom chamber: normal culture medium. **e, f** Hierarchical clustering and multidimensional scaling of RNA sequencing data of pancreatic derivative cancer cells established via direct cell-to-cell transfer. In each sample, the Log2 (FPKM + 1) value was used to express the similarity between samples for (**e**) hierarchical clustering and (**f**) multidimensional scaling to represent the variability between each cell line in two-dimensional space comprising two components. Data are presented as the mean values ± SD.

a

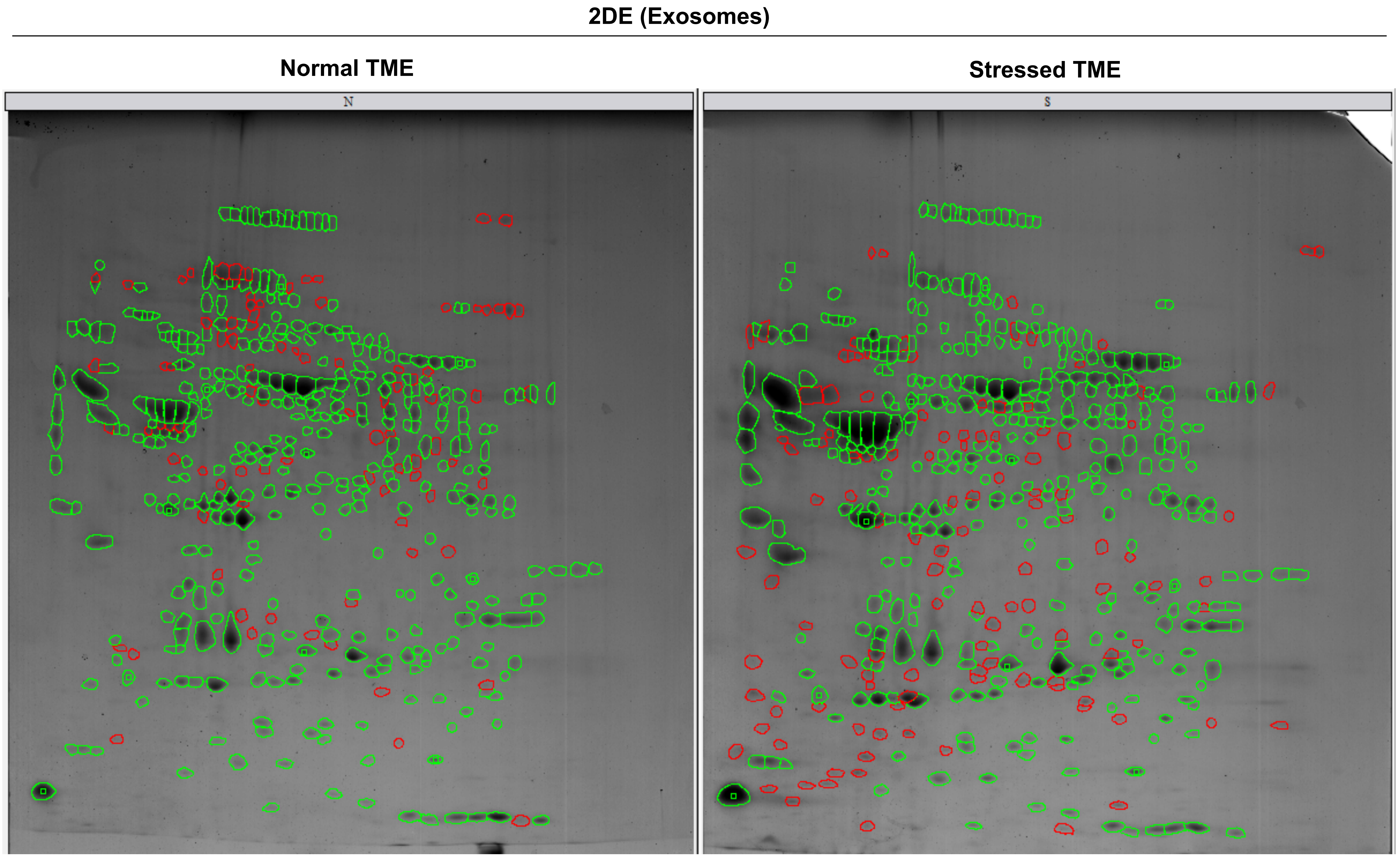

GelName	Sample Name	Spots
N	Normal co-culture TME Exosome	446
S	Stressed (gemcitabine-treated) TME Exosome	473

b

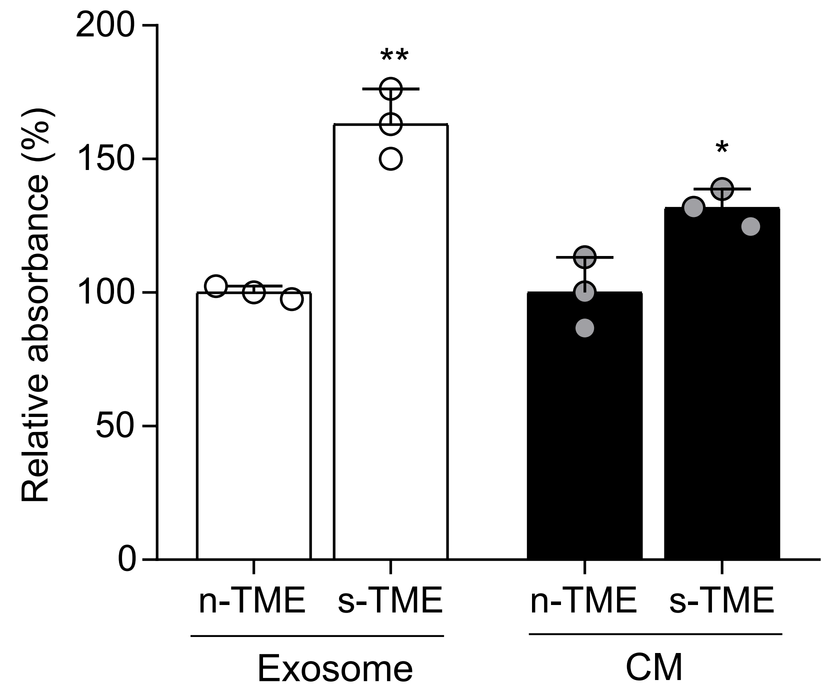

c

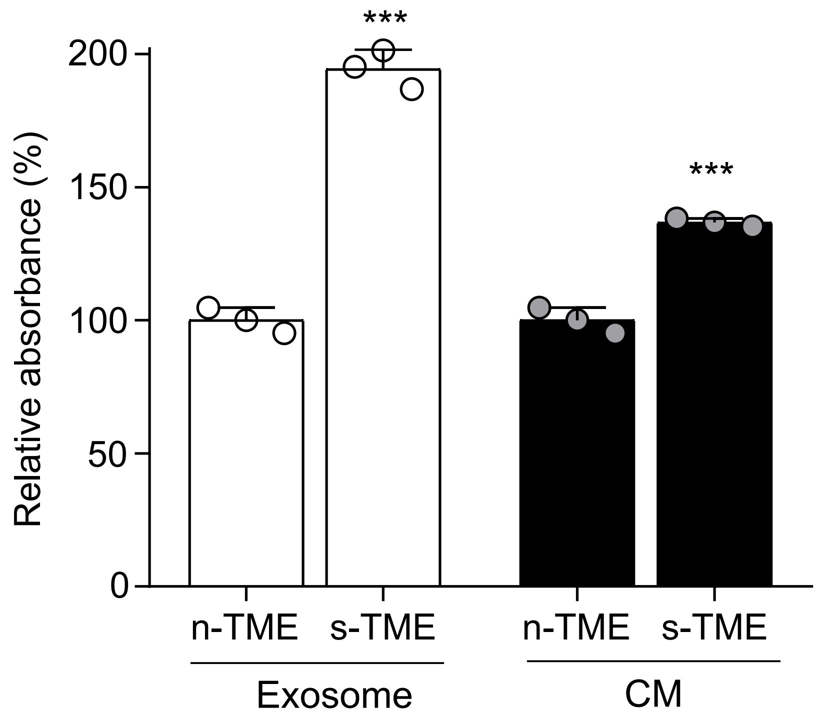

Supplementary Figure 4. Proteomic analysis of TME-derived exosomes. a A total of 27 spots (S/N ratio: >2x up in S-gel) were identified out of 356 commonly found spots (paired groups; green circles), then a total of 17 groups in descending order was selected as a MALDI candidate group. Among 117 non-paired S-gel spots (red circles), top 5 spots as MALDI candidates were selected by considering the volume descending order. **b, c** Stratifin (**b**) and LGALS3BP (**c**) levels were measured from exosomes and conditioned media (CMs) by ELISA. n-TME; normal co-culture TME condition, s-TME; Stressed (gemcitabine-treated) TME condition. *, $P < 0.05$; **, $P < 0.01$; ***, $P < 0.001$. Data are presented as the mean values ± SD.

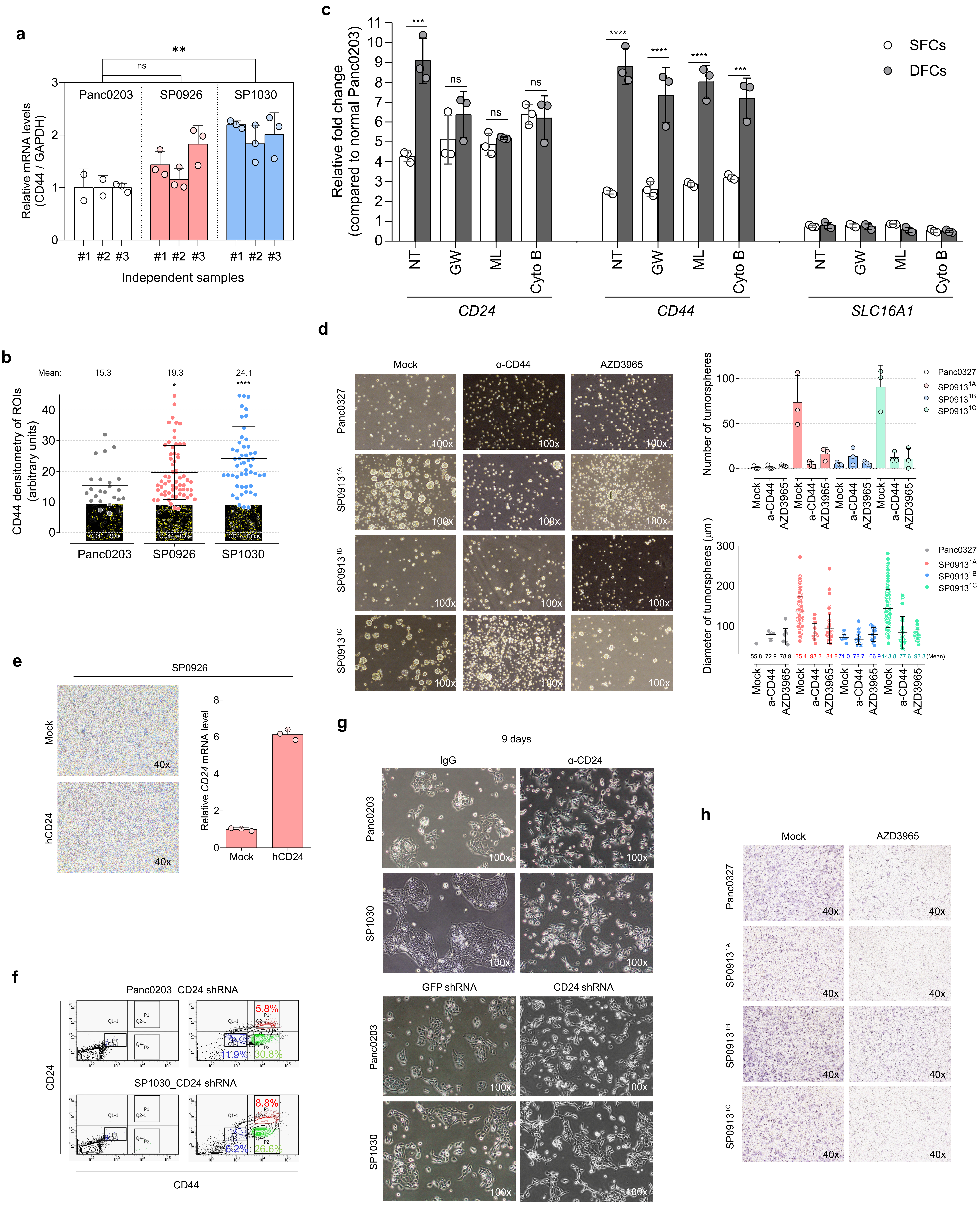
a
Relative mRNA levels (CD44 / GAPDH)
Panc0203
SP0926
SP1030
**
ns
#1 #2 #3
Independent samples
b
Mean: 15.3 19.3 24.1
*
****
CD44 densitometry of ROIs (arbitrary units)
CD44_ROIs
Panc0203
SP0926
SP1030
c
Relative fold change (compared to normal Panc0203)
SFCs
DFCs
***
ns
****
NT
GW
ML
Cyto B
CD24
CD44
SLC16A1
d
Mock
α-CD44
AZD3965
Panc0327
SP0913 1A
SP0913 1B
SP0913 1C
100x
Number of tumorspheres
Diameter of tumorspheres (µm)
a-CD44
55.8 72.9 78.9 135.4 93.2 84.8 71.0 78.7 66.9 143.8 77.6 93.3 (Mean)
e
SP0926
Mock
hCD24
40x
Relative CD24 mRNA level
f
Panc0203_CD24 shRNA
SP1030_CD24 shRNA
5.8%
11.9%
30.8%
8.8%
6.2%
26.6%
CD24
CD44
g
9 days
IgG
α-CD24
GFP shRNA
CD24 shRNA
Panc0203
SP1030
h
Mock
AZD3965
Panc0327
SP0913 1A
SP0913 1B
SP0913 1C

i

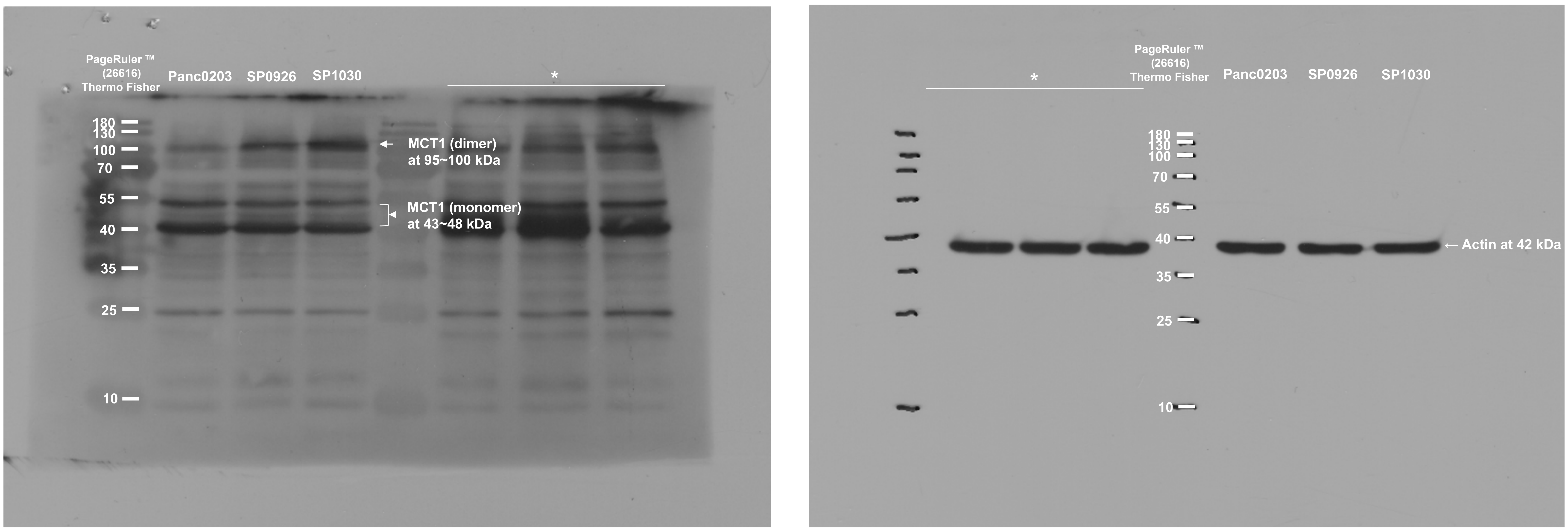

Supplementary Figure 5. Effect of CD24/CD44/MCT1 suppression on both tumorsphering and invasive activities of pancreatic derivative cancer cells. a Relative *CD44* level of derivative cells compared to that of parental Panc0203. Each independent sample represents a different passaged cell line collected on different days sequentially during the incubation process. Quantitative real-time PCR. ** $P \leq 0.01$. Ordinary one-way ANOVA followed by Dunnett's multiple comparisons test. **b** CD44 density measurement from confocal images of Panc0203, SP0926 and SP1030. ROIs (regions of interest). * $P \leq 0.05$; **** $P \leq 0.0001$. One-way ANOVA test. ImageJ was used for the image analysis. **c** Relative expression of CD24, CD44, and SLC16A1 in SFCs and DFCs compared to standard-cultured Panc0203. Quantitative real-time PCR. *** $P \leq 0.001$; **** $P \leq 0.0001$. One-way ANOVA followed by Tukey's multiple comparison test. GW (GW4869), ML (ML141), Cyto B (Cytochalasin) B). **d** Tumorsphere formation activity of pancreatic derivative cancer cells derived from Panc0327 cells. Spheroids were counted on day 7 after seeding. Left panel shows representative images of tumorspheres, and right panel shows number of tumorspheres (upper) and the diameter of tumorspheres (lower). **e** Human CD24 overexpression effect on SP0926 invasiveness. A 3D invasion assay was conducted for 10 h after seeding 1×10^5 cells at 48 h post-transfection (left). *CD24* level was checked by qPCR (60 h after transfection; right). Mock; empty vector, hCD24; human CD24 vector. Upper chamber (serum-free medium), bottom chamber (normal culture medium). **f** CD24 expression levels in *CD24* shRNA-transduced stable cells. Flow cytometry was performed following puromycin selection (0.4 μg/ml) until stable cells were generated. **g** Changes in spatial growth pattern via treatment with CD24 neutralizing antibody for 9 days (left) and via *CD24* shRNA stable transfection (right). **h** Effect of MCT1 inhibition on invasion activity of derivative cancer cells derived from Panc0327 cells. A 3D invasion assay was performed for 24 h after treatment with the MCT1 inhibitor AZD3965. Total 1×10^5 cells were seeded into upper chamber containing Matrigel. Upper chamber; serum-free medium, bottom chamber; normal culture medium for Panc0327. **i** Uncropped and unprocessed scans for MCT1 (left) and beta-actin (right). Left: arrow and arrow head indicate a dimer form (95~100 kDa) and monomer forms (43~48 kDa) of MCT1, respectively[70-72], when compared to ladder sizes of the used protein marker (PageRuler, Thermo Fisher). Right: arrow indicates β-actin bands (42 kDa). Asterisk: samples not relevant to this study. Cropped images (ROIs) presented on Fig. 5a. All graph data are presented as mean ± SD.

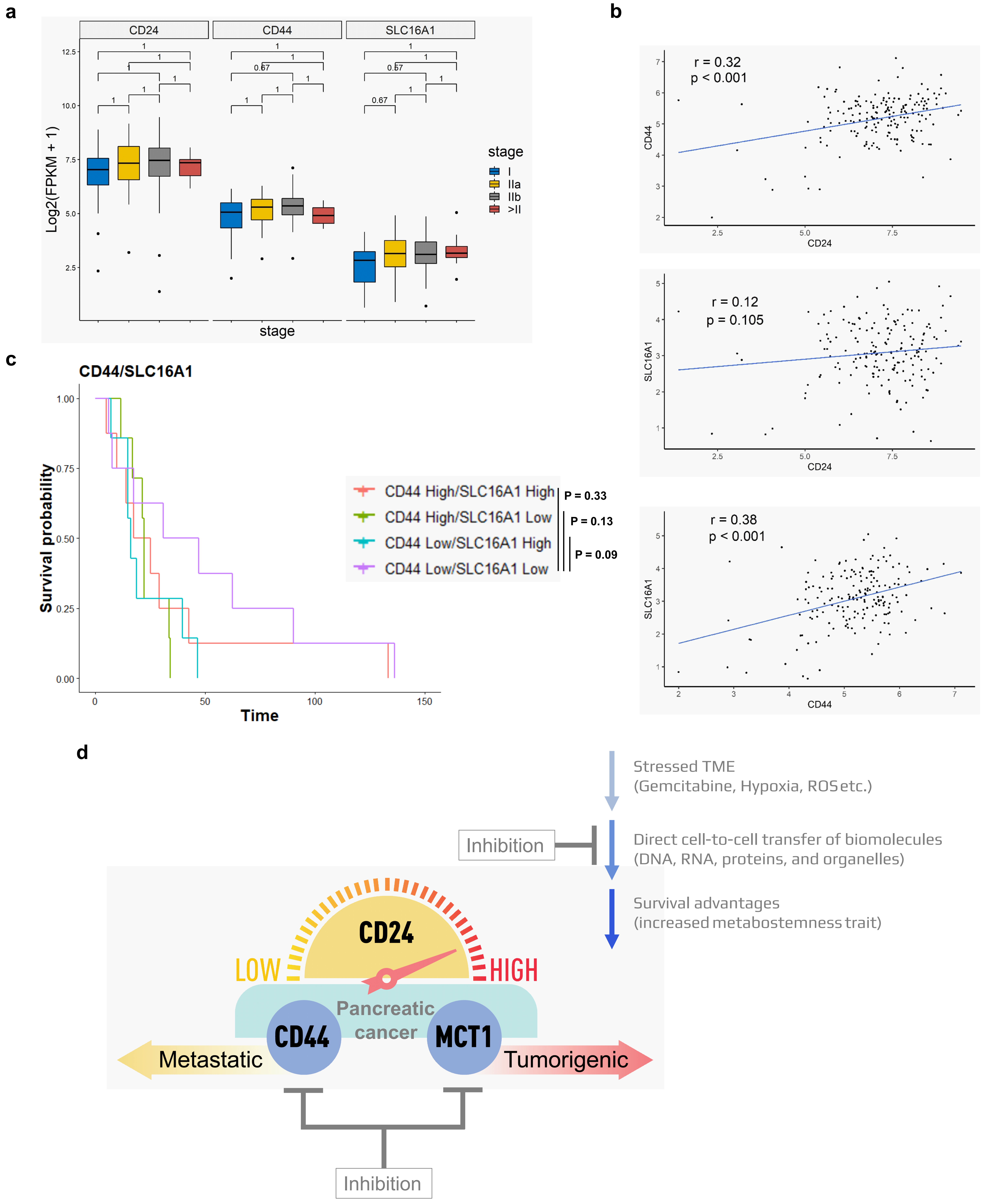

Supplementary Figure 6. Analysis of *CD24*, *CD44*, and *SLC16A1* expression in pancreatic cancer-patient tissue samples. **a** Expression of *CD24*, *CD44*, and *SLC16A1* in pancreatic cancer-patient tissue samples (TCGA Pancreatic Cancer (PAAD) database (n = 182)) according to stage. Numbers in graph indicate p-values between samples from different-stage tumors. **b** Correlation of *CD24*, *CD44*, and *SLC16A1* expression in pancreatic cancer-patient tissue samples. Pearson correlation coefficients and p-values from linear regression analysis are indicated. **c** Kaplan-Meier plot for overall survival of pancreatic cancer patients with high or low expression of *CD44*, or *SLC16A1*. Microarray gene-expression profiles (median cutoff) of 30 pancreatic tumors tissue from patients (GSE84219). P = 0.31. **d** Schematic showing CD44/MCT1/CD24 relationship after chemotherapy for pancreatic cancer. Combinations of relative levels of CD44/MCT1/CD24 expression involved in giving rise, post-gemcitabine-treatment, to cancer cells with a relatively high tumorigenic or a high metastatic phenotype compared to primary pancreatic cancer cells.

Supplementary Table 1. Transcriptomic data of pancreatic derivative cells generated via direct cell-to-cell transfer.

Gene_ID	Transcript_ID	Gene_Symbol	Description	SP0926/Panc0203.fc	SP1030/Panc0203.fc	Panc0203_FPKM	SP0926_FPKM	SP1030_FPKM	Panc0203.mean	SP0926.mean	SP1030.mean	Panc0203_Read_Count	SP0926_Read_Count	SP1030_Read_Count
4023	NM_000237	LPL	lipoprotein lipase	8.549289955	358.9989005	0.094222	0.839854	33.235188	0.43999062	2.02475093	7.02280826	14	161	6231
63915	NM_001199322,NM_001199323	BLOC1S5	biogenesis of lysosomal organell	169.7936083	320.3173479	0.006506	1.820737	3.247846	0.03628685	2.53229878	3.33208871	1	251	438
5671	NM_021016	PSG3	pregnancy specific beta-1-glycop	2.684182094	211.9128847	0.030165	0.103459	8.492898	0.10623215	0.27464693	4.15342015	3	11	811
100137049	NM_001114633	PLA2G4B	phospholipase A2 group IVB	24.99513712	171.7752473	0.082044	2.174733	14.064159	0.29776659	2.76453517	5.35280854	9	304	1925
729857	NM_001078170,NM_001078170	RGPD2	RANBP2-like and GRIP domain c	89.23627968	100.1846094	0.026826	2.592435	2.734242	0.20517559	3.8951276	4.05259921	6	728	753
100616394	NR_039646_chr2,NR_039646_c	MIR4444-1	microRNA 4444-1	105.5656834	84.17592771	0.031679	41.802429	31.395544	0.03628685	1.99055518	1.75769337	1	156	115
388692	NM_001013644	LOC388692	uncharacterized LOC388692	233.9341387	78.67342857	0.019636	5.612086	1.775174	0.10623215	4.28618731	2.85469418	3	971	301
27113	NM_001127240,NM_001127241	BBC3	BCL2 binding component 3	13.61255188	60.72840298	0.239956	3.292184	16.34935	0.5190475	2.79259185	4.7796009	17	311	1278
5673	NM_001130014,NM_002781	PSG5	pregnancy specific beta-1-glycop	1.23682069	53.77776723	0.400095	0.505744	21.17508	0.73313634	0.86372326	5.20026005	26	43	1727
445815	NM_007203,NM_147150	PALM2-AKAP2	PALM2-AKAP2 readthrough	2208.861203	53.47023221	0.001344	8.516644	0.192709	0.03628685	5.98368468	1.32870978	1	3267	73
3983	NM_001003407,NM_001003408	ABLIM1	actin binding LIM protein 1	50.50924608	48.13074502	0.139307	7.405021	6.619999	1.06712766	5.81904452	5.75087852	43	2909	2552
102800317	NM_001289912	LOC400927-CSNK1E	LOC400927-CSNK1E readthroug	26.4111313	41.58904556	0.251979	6.716522	9.925539	0.79797761	4.36304264	4.99259436	29	1027	1489
5669	NM_001184825,NM_001184826	PSG1	pregnancy specific beta-1-glycop	-4.077298965	39.20393425	0.50897	0.119044	18.658206	1.01352966	0.31941552	5.35937359	40	13	1934
440533	NM_001130167,NM_001130168	PSG8	pregnancy specific beta-1-glycop	-1.201549503	38.17946582	0.339267	0.282073	13.068906	0.7550755	0.65251278	4.77415178	27	30	1273
4128	NM_000240,NM_001270458	MAOA	monoamine oxidase A	4.735790347	34.99168791	0.16614	0.814625	5.661149	0.91952222	2.38738651	5.01309432	35	222	1511
6512	NM_001287595,NM_001287596	SLC1A7	solute carrier family 1 member 7	8.321942883	32.98843693	0.071765	0.610328	2.438447	0.26755841	1.44132992	2.96594348	8	90	329
3625	NM_002193	INHBB	inhibin beta B subunit	-2.905736914	31.17097204	0.282379	0.097674	8.388829	0.91952222	0.38406669	4.85173544	35	16	1346
54492	NM_001142651,NM_001308177	NEURL1B	neuralized E3 ubiquitin protein lig	1.271370539	30.27161321	0.122323	0.15356	3.492749	0.81895953	0.97983233	4.59783705	30	51	1121
255738	NM_174936,NR_110451	PCSK9	proprotein convertase subtilisin/k	1.068928627	25.86001533	0.197304	0.208984	4.819915	0.77668602	0.81765451	4.28644656	28	40	894
106865373	NM_001317744,NM_001350300	WRB-SH3BGR	WRB-SH3BGR readthrough	14.25922155	25.45958694	0.021027	0.544127	0.928773	0.07168335	0.81765451	1.24294651	2	40	66
100499483	NM_001348010,NM_020893	CCDC180	coiled-coil domain containing 18(	5.256273468	25.18854001	0.138099	0.351689	1.89328	0.5190475	1.71704117	3.58206614	17	120	530
100133005	NR_146066	RASA4DP	RAS p21 protein activator 4CD, p	25.32740701	24.59388461	0.011448	0.429248	0.388352	0.10623215	1.58576189	1.5590879	3	105	94
23384	NM_001145468,NM_001254732	SPECC1L	sperm antigen with calponin hom	24.66530884	24.33676022	0.475041	11.732961	10.88546	2.03825186	6.28005326	6.26101394	122	4024	3655
26149	NM_001317916,NM_033160,NR	ZNF658	zinc finger protein 658	21.52158928	21.15429325	0.062384	1.652818	1.561215	0.38477185	2.93245472	2.9113909	12	348	315
1795	NM_004947	DOCK3	dedicator of cytokinesis 3	6.018437558	21.09288541	0.091775	0.558473	1.842435	0.83964067	2.52753672	4.1466887	31	250	807
29057	NM_001242489,NM_001242490	FAM156A	family with sequence similarity 15	8.885754287	20.57127275	0.003171	0.115054	0.252592	0.03628685	0.31941552	0.65979727	1	13	28
8869	NM_001042437,NM_003896	ST3GAL5	ST3 beta-galactoside alpha-2,3-s	6.55430289	19.38154763	0.307304	2.019296	5.620147	0.77668602	2.50832973	3.89484064	28	246	670
645553	NR_135234	IGFL2-AS1	IGFL2 antisense RNA 1	8.448151298	18.13610851	0.592422	5.124465	10.325116	0.97665845	3.20120812	4.21745651	38	430	850
768239	NM_001085382	PSAPL1	prosaposin-like 1 (gene/pseudog	1.491030277	17.14398413	0.04472	0.074271	0.815887	0.29776659	0.42561204	2.3150407	9	18	192
100133941	NM_001291737,NM_001291738	CD24	CD24 molecule	-11.6513608	17.09317492	6.743779	0.57697	131.336774	4.0892716	1.24644988	8.10276991	629	72	13226
2702	NM_005266,NM_181703	GJA5	gap junction protein alpha 5	8.133905977	16.72812144	0.313221	2.565064	4.959818	0.97665845	3.15255179	4.10736121	38	414	784
3624	NM_002192	INHBA	inhibin beta A subunit	-2.058039831	16.49687813	0.145956	0.07105	2.280069	0.89996265	0.50527715	3.93836491	34	22	692
7593	NM_001267033,NM_003422,NM	MZF1	myeloid zinc finger 1	7.561658997	16.24273591	0.438357	3.383395	7.245323	1.16869613	3.38618649	4.41385336	49	496	981
81704	NM_001190458,NM_001193536	DOCK8	dedicator of cytokinesis 8	1.711987557	15.78542565	0.081718	0.143109	1.262726	0.68823103	1.03456085	3.41845388	24	55	468
93380	NM_001330000,NM_173470	MMGT1	membrane magnesium transport	-1.854911758	15.71539059	0.406459	0.220361	6.074769	1.62696745	1.08728891	5.08175167	82	59	1587
8972	NM_004668	MGAM	maltase-glucoamylase	3.670720492	15.44950592	0.016139	0.060461	0.239139	0.13997292	0.46599441	1.38699077	4	20	78
9245	NM_004751	GCNT3	glucosaminyl (N-acetyl) transfera	-2.139762856	15.21877658	0.994007	0.465362	14.321826	1.66222564	1.00745609	5.08703534	85	53	1593
149041	NM_001300850,NM_001300851	RC3H1	ring finger and CCCH-type doma	1.410229661	14.93240362	0.36376	0.515167	5.154098	2.3142594	2.72348335	5.91584555	156	294	2867
56102	NM_018924,NM_032097	PCDHGB3	protocadherin gamma subfamily	24.99200412	14.85634275	0.040623	0.568483	0.317269	0.13997292	1.85267538	1.35230545	4	137	75
55068	NM_001127615,NM_001242863	ENOX1	ecto-NOX disulfide-thiol exchang	5.023740936	14.68106885	0.014902	0.077608	0.21425	0.07168335	0.3412894	0.83750224	2	14	38
9615	NM_001242505,NM_001242506	GDA	guanine deaminase	14.47016182	14.54750979	1.291721	11.599675	9.515409	2.09955541	5.60236135	5.60994698	129	2496	2310
1852	NM_001318503,NM_001395	DUSP9	dual specificity phosphatase 9	1.173459454	14.07702632	0.06449	0.087071	1.022555	0.23670415	0.27464693	1.8266989	7	11	123
55802	NM_001290204,NM_001290205	DCP1A	decapping mRNA 1A	1.749369981	14.02808365	0.241381	0.421992	3.233833	1.27880673	1.80624674	4.39551594	56	131	968
140710	NM_080627,NM_199181	SOGA1	suppressor of glucose, autophag	14.23421451	13.7444649	0.574735	2.983307	2.688969	1.9554003	5.39250602	5.34327263	113	2151	1912
4982	NM_002546	TNFRSF11B	TNF receptor superfamily membe	1.582960113	13.56914846	2.601046	4.132582	33.282734	2.7991369	3.38355363	6.35598995	234	495	3907
9926	NM_001320808,NM_014873	LPGAT1	lysophosphatidylglycerol acyltran	1.159463922	13.45849739	1.046817	1.214661	13.359781	3.25290323	3.44545299	6.85641965	335	519	5547
9497	NM_001258379,NM_001258380	SLC4A7	solute carrier family 4 member 7	10.34663057	13.07730016	1.202266	13.082099	15.541273	3.38891307	6.63026582	6.96515172	372	5144	5985
130367	NM_001320833,NM_001320834	SGPP2	sphingosine-1-phosphate phosph	2.062512609	12.74008534	0.084485	0.177536	1.036397	0.5190475	0.92294555	2.71151569	17	47	268
163589	NM_001199085,NM_001199089	TDRD5	tudor domain containing 5	2.473352606	12.555501	0.125199	0.309399	1.667168	0.56944351	1.1381576	2.82969649	19	63	295
10715	NM_001290265,NM_021267,NM	CERS1	ceramide synthase 1	3.279644553	12.47655736	0.069924	0.279742	1.017755	0.23670415	0.669904	1.70368302	7	31	109
64359	NM_001205319,NM_022463	NXN	nucleoredoxin	4.094555	12.2677413	1.019045	4.184752	11.776834	2.00201503	3.73497962	5.24427765	118	646	1782
5650	NM_001207053,NM_001243126	KLK7	kallikrein related peptidase 7	-367.1182894	12.26679556	17.707227	0.046871	204.061806	5.20908974	0.13134883	8.78960678	1413	5	21320
79993	NM_001104558,NM_001297617	ELOVL7	ELOVL fatty acid elongase 7	3.973021655	12.20897914	1.010336	4.059593	11.732011	2.27683528	4.02581645	5.58464442	151	802	2269
26038	NM_015557	CHD5	chromodomain helicase DNA bin	-8.160754433	12.10504092	0.497146	0.060193	5.669664	2.5075876	0.65251278	5.85210402	184	30	2741
1525	NM_001207063,NM_001207064	CXADR	CXADR, Ig-like cell adhesion mo	10.7615657	12.00100396	1.322798	5.722368	6.030617	1.76310734	4.74377637	4.89555604	94	1353	1389
201562	NM_001329783,NM_001329784	HACD2	3-hydroxyacyl-CoA dehydratase	10.21247913	11.89026965	4.142653	42.719977	46.658197	4.19537543	7.47499454	7.69331111	680	9280	9946
1628	NM_001352	DBP	D-box binding PAR bZIP transcri	1.143346189	11.88577227	5.46293	6.262496	61.165939	3.41326233	3.58949269	6.85512987	379	579	5542
116211	NM_001204897,NM_001204898	TM4SF19	transmembrane 4 L six family me	36.16724872	11.79244666	0.053287	2.778875	0.826468	0.10623215	1.94842076	0.95003448	3	150	45
3851	NM_002272	KRT4	keratin 4	-10.8470125	11.75864398	0.799145	0.072378	8.894048	1.42282133	0.20477631	4.37837968	66	8	956
25984	NM_001282433,NM_015515	KRT23	keratin 23	-2.185049299	11.63810875	0.689984	0.311575	8.199189	1.32354034	0.75384785	4.21101796	59	36	846
1048	NM_001291484,NM_001308398	CEACAM5	carcinoembryonic antigen related	-1.770151862	11.19983145	2.019725	1.137141	22.999737	2.99161027	2.30073045	6.30210724	273	206	3762
4047	NM_001001438,NM_001145436	LSS	lanosterol synthase	4.615262955	11.15208325	1.466278	4.354696	9.760279	2.29192162	4.24761625	5.47539445	153	944	2100
1000	NM_001308176,NM_001792	CDH2	cadherin 2	6.225304037	11.12193158	0.287428	1.807431	3.029422	1.15225689	3.10858833	3.8705348	48	400	658
339400	NR_103778,NR_103779	FLG-AS1	FLG antisense RNA 1	7.315384361	11.0996427	0.025449	0.190504	0.268027	0.13997292	0.81765451	1.11084509	4	40	56
55808	NM_001289107,NM_018414,NR	ST6GALNAC1	ST6 N-acetylgalactosaminide alp	-1.330220287	11.03876803	0.07702	0.053846	0.842565	0.26755841	0.20477631	1.71282633	8	8	110
100528031	NR_037661	FKBP1A-SDCBP2	FKBP1A-SDCBP2 readthrough (	2.919339287	10.92195613	0.661532	1.947434	6.848739	1.06712766	2.07130288	3.69940604	43	168	579
148932	NM_145279,NM_201403	MOB3C	MOB kinase activator 3C	4.20315693	10.8276526	1.360164	5.726412	13.870132	2.24618091	4.06578025	5.37745464	147	826	1959
9073	NM_199328	CLDN8	claudin 8	4.035186879	10.70420058	0.048763	0.192357	0.498724	0.13997292	0.50527715	1.08290869	4	22	54
360	NM_001318144,NM_004925	AQP3	aquaporin 3 (Gill blood group)	-1.859980435	10.35014057	3.16683	1.69969	30.885445	2.76169834	2.03820547	5.92767386	227	163	2891
57717	NM_020957	PCDHB16	protocadherin beta 16	29.16825689	10.33604091	0.001905	0.185832	0.05941	0.03628685	0.86372326	0.36729312	1	43	14
692233	NR_003140	SNORD117	small nucleolar RNA, C/D box 11	4.829253767	10.33604091	0.177226	1.703267	3.502373	0.03628685	0.18071335	0.36729312	1	7	14
100846999	NR_049831_chr2,NR_049831_c	MIR4444-2	microRNA 4444-2	12.26617139	10.33604091	0.031715	4.628641	3.671579	0.03628685	0.42561204	0.36729312	1	18	14
5413	NM_001009939,NM_002688	SEPT5	septin 5	2.921400534	10.26059398	1.452407	4.689459	15.43533	2.0823043	3.38618649	5.09667211	127	496	1604
2877	NM_002083,NR_046320,NR_04	GPX2	glutathione peroxidase 2	-1.167963162	10.23957401	3.278484	2.936819	32.994764	2.23841445	2.06474386	5.28928581	146	167	1840
3638	NM_001346590,NM_001346591	INSIG1	insulin induced gene 1	-2.036099182	10.22656683	5.333297	2.677528	52.496629	4.09358292	3.15255179	7.3697324	631	414	7938
79937	NM_033655	CNTNAP3	contactin associated protein-like	1.531446981	10.19291012	0.099133	0.150866	0.960075	0.59399636	0.83317479	2.63662357	20	41	252
283106	NM_001256686	CSNK2A3	casein kinase 2 alpha 3	7.201918015	10.04170665	0.073902	0.676454	0.884417	0.17294258	0.95166931	1.20457043	5	49	63
2746	NM_001318900,NM_001318901	GLUD1	glutamate dehydrogenase 1	3.445952842	9.934084441	7.156771	24.526089	66.111956	4.67116511	6.41541706	7.93185674	961	4425	11743
9575	NM_001267843,NM_004898	CLOCK	clock circadian regulator	6.488201684	9.699838812	0.550212	3.590258	5.28064	2.78853894	5.29849962	5.86651418	232	2012	2769
284836	NR_026960	LINC00319	long intergenic non-protein codin	-1.328298723	9.595993445	0.052201	0.034458	0.475641	0.20517559	0.15624221	1.31676564	6	6	72
27076	NM_014400	LYPD3	LY6/PLAUR domain containing 3	1.381626963	9.468978875	6.370952	8.819398	56.771534	3.53555296	3.96723624	6.66330624	416	768	4846
56112	NM_018916,NM_032011	PCDHGA3	protocadherin gamma subfamily	-1.320831011	9.441162143	0.026266	0.022011	0.151277	0.10623215	0.08023518	0.80366934	3	3	36
23138	NM_015111	N4BP3	NEDD4 binding protein 3	-1.610444678	9.359150519	0.332058	0.204771	2.921483	1.55375576	1.1381576	4.25862092	76	63	876
1580	NM_000779,NM_001099772,NM	CYP4B1	cytochrome P450 family 4 subfar	6.2575974	9.202736341	0.102025	0.681333	0.9477	0.29776659	1.29208824	1.64757178	9	76	103
5292	NM_001243186,NM_002648	PIM1	Pim-1 proto-oncogene, serine/thr	3.066608324	9.123870144	7.295429	22.434877	62.681129	4.34210161	5.91012909	7.46716758	757	3102	8496
100289187	NM_001195541,NM_001195542	TMEM225B	transmembrane protein 225B	2.658859464	9.087442248	0.067688	0.228095	0.723999	0.17294258	0.42561204	1.12461302	5	18	57
6947	NM_001062	TCN1	transcobalamin 1	7.642125977	9.087179589	5.93917	45.481709	50.760822	3.3316643	6.13575486	6.38237404	356	3636	3980
9022	NM_004669	CLIC3	chloride intracellular channel 3	-2.056848361	8.972350472	18.079004	8.781378	152.490875	3.93685111	2.99243508	7.01636074	562	365	6203
6695	NM_004598	SPOCK1	SPARC/osteonectin, cwcv and ka	38.11063655	8.940897518	0.157066	6.016318	1.321038	0.79797761	4.87046532	2.9311116	29	1482	320
83693	NM_001146051,NM_031463	HSDL1	hydroxysteroid dehydrogenase lil	4.9597777	8.869666429	1.651127	8.324724	13.838174	2.78853894	4.92217849	5.7397392	232	1538	2532
113791	NM_001135911,NM_052880	PIK3IP1	phosphoinositide-3-kinase intera	1.496447252	8.833345509	1.325911	2.019578	11.137182	2.07360076	2.53704518	4.87635737	126	252	1370
23621	NM_001207048,NM_001207049	BACE1	beta-secretase 1	9.20471072	8.743093975	0.233756	2.187132	1.972494	1.23264156	3.74936649	3.68088855	53	653	571
284307	NM_001010879,NM_001321145	ZIK1	zinc finger protein interacting with	2.443296723	8.657400636	0.011267	0.042167	0.141822	0.10623215	0.25173066	0.75138414	3	10	33
134	NM_000674,NM_001048230	ADORA1	adenosine A1 receptor	1.227829369	8.592385399	0.119576	0.149175	1.014181	0.43999062	0.52452456	2.03057861	14	23	149
90338	NM_001102603,NM_001322125	ZNF160	zinc finger protein 160	5.524210586	8.555330118	1.981412	9.781928	14.301349	3.05488459	5.37140066	5.99011117	287	2119	3021
7052	NM_001323316,NM_001323317	TGM2	transglutaminase 2	1.511295999	8.511814882	76.111113	118.596303	417.113984	7.61813059	8.21143714	10.70112081	7674	15496	80340

2624	NM_001145661,NM_001145662	GATA2	GATA binding protein 2	2.866729439	8.499883445	1.072733	3.088278	8.634947	2.21486103	3.51686905	4.99914873	143	548	1496
3860	NM_002274,NM_153490	KRT13	keratin 13	3.444442925	8.415629333	1.734057	6.024698	13.794354	1.96484452	3.46052158	4.67002972	114	525	1181
1727	NM_000398,NM_001129819,NM	CYB5R3	cytochrome b5 reductase 3	4.260130242	8.339405158	2.889473	12.322786	22.286125	3.2058879	5.17223519	6.12165047	323	1839	3314
53947	NM_001318038,NM_017436,NR	A4GALT	alpha 1,4-galactosyltransferase (	-3.977952594	8.304594378	0.507392	0.126723	4.648537	1.04948198	0.3412894	3.30815729	42	14	430
100526835	NM_001112808,NM_001199327	FPGT-TNNI3K	FPGT-TNNI3K readthrough	2.658859464	8.292221915	0.037826	0.100145	0.309081	0.17294258	0.42561204	1.05442063	5	18	52
5314	NM_138694,NM_170724	PKHD1	PKHD1, fibrocystin/polyductin	-2.43097958	8.208060283	0.06766	0.027734	0.523118	1.04948198	0.52452456	3.29299602	42	23	425
100506243	NM_001205272	KRBOX1	KRAB box domain containing 1	5.023740936	8.136968582	0.052547	0.397138	0.584832	0.07168335	0.3412894	0.52094563	2	14	21
6707	NM_001097589,NM_005416	SPRR3	small proline rich protein 3	1.733219757	8.122602881	2.892037	5.002192	22.300059	1.83696709	2.4491251	4.45393896	101	234	1010
57537	NM_020777	SORCS2	sortilin related VPS10 domain co	6.413491115	8.045613533	0.949801	6.107076	7.189479	2.72326229	5.20764596	5.52684729	220	1886	2178
5802	NM_002850,NM_130853,NM_13	PTPRS	protein tyrosine phosphatase, rec	-2.564310583	8.042265599	1.111676	0.433458	8.553702	3.16550307	2.03820547	6.02539928	313	163	3097
51109	NM_001252650,NM_016026	RDH11	retinol dehydrogenase 11 (all-tra	3.447287909	7.878510076	12.70218	43.773179	94.407375	5.02835327	6.78217295	7.9672679	1242	5721	12036
9665	NM_001184998,NM_001184999	MARF1	meiosis regulator and mRNA stal	5.063685547	7.869370569	1.214092	6.168133	9.000184	3.34979977	5.57204137	6.19729591	361	2443	3495
257194	NM_173808	NEGR1	neuronal growth regulator 1	-38.72288841	7.816567385	0.284701	0.006252	2.101779	1.36692851	0.05398282	3.74022131	62	2	597
84983	NR_026661,NR_026662	FAM222A-AS1	FAM222A antisense RNA 1	11.772746	7.752021507	0.050598	0.732874	0.486053	0.07168335	0.70406985	0.49997203	2	33	20
255324	NM_001270989,NM_001270990	EPGN	epithelial mitogen	-2.609593906	7.634136887	0.47268	0.180758	3.466232	1.16869613	0.56226574	3.39875952	49	25	461
5655	NM_001077500,NM_002776,NM	KLK10	kallikrein related peptidase 10	1.076709285	7.605483589	11.162517	11.941462	80.154052	5.06942494	5.17299774	7.95876649	1279	1840	11965
2002	NM_001114123,NM_001257168	ELK1	ELK1, ETS transcription factor	6.490796943	7.560468515	0.839167	5.804157	6.253409	1.76310734	4.04926299	4.2570592	94	816	875
11018	NM_006858,NR_104015	TMED1	transmembrane p24 trafficking pr	5.887775447	7.547574979	4.272101	25.251807	30.604196	3.0370876	5.44142749	5.79244048	283	2227	2628
51479	NM_001257999,NM_001330063	ANKFY1	ankyrin repeat and FYVE domair	6.993296525	7.525293297	1.129503	8.006957	8.085646	3.22958708	5.89772745	6.00181463	329	3075	3046
23107	NM_001286748,NM_001286751	MRPS27	mitochondrial ribosomal protein S	1.261492163	7.438372322	4.299799	5.432001	30.144734	3.66286259	3.97425277	6.4560292	458	772	4191
6038	NM_001282192,NM_001282193	RNASE4	ribonuclease A family member 4	1.333777504	7.424516862	1.266584	1.78893	9.473644	1.74129091	2.04488597	4.20292915	92	164	841
9788	NM_001282971,NM_001282974	MTSS1	MTSS1, I-BAR domain containing	1.355744314	7.387232634	0.243515	0.33138	1.727745	1.15225689	1.41062362	3.3291189	48	87	437
8553	NM_003670	BHLHE40	basic helix-loop-helix family mem	1.771099821	7.345678815	4.748377	8.419765	32.801903	3.91021271	4.69258035	6.70198758	551	1304	4979
101928034	NR_125947	LOC101928034	uncharacterized LOC101928034	5.606274658	7.181042604	0.302679	1.79211	2.164788	0.5190475	1.78245981	2.04513029	17	128	151
23158	NM_015130	TBC1D9	TBC1 domain family member 9	7.812088293	7.078140286	0.303735	2.389981	2.031147	1.39514558	3.78172178	3.6502813	64	669	558
6367	NM_002990	CCL22	C-C motif chemokine ligand 22	-1.068408814	7.071651765	0.139364	0.130891	0.954386	0.49317359	0.46599441	1.96321628	16	20	140
10397	NM_001135242,NM_001258432	NDRG1	N-myc downstream regulated 1	7.53255262	7.065202698	2.505482	18.855081	16.611705	3.09414281	5.85322458	5.76248297	296	2980	2573
57476	NM_001286563,NM_001286564	GRAMD1B	GRAM domain containing 1B	-1.665395864	7.030239621	0.082474	0.048966	0.546226	0.71085839	0.46599441	2.45783582	25	20	217
11202	NM_001281431,NM_007196,NM	KLK8	kallikrein related peptidase 8	-8.839382876	6.983602031	5.496611	0.747661	30.772216	2.29192162	0.52452456	4.8192972	153	23	1315
56103	NM_018923,NM_032096	PCDHGB2	protocadherin gamma subfamily	11.464905	6.961048854	0.10867	0.76241	0.453066	0.35634914	2.08433212	1.56919173	11	170	95
4017	NM_002318	LOXL2	lysyl oxidase like 2	5.76376815	6.911450997	1.991006	11.490819	12.936691	3.06808971	5.44585108	5.702357	290	2234	2466
29799	NM_013313,NR_130910	YPEL1	yippee like 1	1.339431543	6.849729792	0.134805	0.180821	0.930206	0.68823103	0.86372326	2.37961802	24	43	203
5054	NM_000602	SERPINE1	serpin family E member 1	1.045953213	6.775530208	33.768219	35.360909	215.028992	6.72642285	6.7906441	9.4751295	4118	5755	34318
5046	NM_001291309,NM_002570,NM	PCSK6	proprotein convertase subtilisin/k	2.416977525	6.751418528	2.499882	6.253852	17.708722	3.55444243	4.75400498	6.20136181	422	1363	3505
79987	NM_153366	SVEP1	sushi, von Willebrand factor type	1.119455784	6.749779473	0.008478	0.008831	0.055027	0.13997292	0.15624221	0.76902392	4	6	34
1508	NM_001317237,NM_001908,NM	CTSB	cathepsin B	2.646789747	6.739450491	14.065413	37.096899	89.316106	5.73516237	7.1225018	8.46460831	2052	7257	17010
11254	NM_007231	SLC6A14	solute carrier family 6 member 14	-5.260091942	6.710980824	9.094278	1.728092	57.36898	5.37234789	3.11812251	8.0890104	1587	403	13100
7062	NM_007113	TCHH	trichohyalin	14.47880522	6.669488964	0.022292	0.330275	0.142508	0.20517559	1.70021226	1.02535867	6	118	50
347454	NM_001105576	SOWAHD	sosondowah ankyrin repeat dom	2.468588018	6.669488964	0.098396	0.239726	0.616986	0.20517559	0.46599441	1.02535867	6	20	50
54361	NM_030761	WNT4	Wnt family member 4	2.165921034	6.668376863	0.070576	0.155857	0.462552	0.35634914	0.68708806	1.52834475	11	32	91
23508	NM_015351	TTC9	tetratricopeptide repeat domain 9	-3.974778317	6.655887328	1.966128	0.49421	12.326963	3.46076162	1.81408935	6.07985916	393	132	3218
257019	NM_001244959,NM_001244960	FRMD3	FERM domain containing 3	6.198597553	6.628788894	0.039563	0.245083	0.24867	0.26755841	1.18729361	1.24294651	8	67	66
83716	NM_031476	CRISPLD2	cysteine rich secretory protein LC	1.72934806	6.619201837	0.693333	1.201521	4.327832	2.03825186	2.67262769	4.43196063	122	282	994
57512	NM_020752	GPR158	G protein-coupled receptor 158	7.86542728	6.597180283	0.007854	0.064701	0.050976	0.07168335	0.50527715	0.43515728	2	22	17
444	NM_001164750,NM_001164751	ASPH	aspartate beta-hydroxylase	1.423453222	6.508454271	41.270302	58.832428	245.707577	7.41866272	7.92555407	10.11384392	6678	12701	53458
10150	NM_001306070,NM_144778,NM	MBNL2	muscleblind like splicing regulato	2.618399368	6.501678637	4.604144	11.942474	28.248984	4.45531083	5.80287809	7.09953703	822	2876	6574
2200	NM_000138	FBN1	fibrillin 1	3.541945494	6.486831981	0.076854	0.277125	0.478374	0.91952222	2.05815489	2.76523898	35	166	280
101059953	NM_001282511,NM_001282511	NPIPA8	nuclear pore complex interacting	11.09963705	6.464563508	1.120254	12.921272	7.338375	1.79522562	4.83042646	4.08640889	97	1440	772
10420	NM_001320800,NM_007170	TESK2	testis-specific kinase 2	1.422606202	6.460519276	0.426596	0.607843	2.637632	1.2010231	1.51053416	3.23388481	51	97	406
124491	NM_001304996,NM_001304997	TMEM170A	transmembrane protein 170A	5.104713982	6.438503177	1.576113	8.122723	9.567031	3.08117507	5.28938617	5.61666065	293	1999	2321
3321	NM_001007237,NM_001542	IGSF3	immunoglobulin superfamily men	2.800516284	6.422975624	1.103457	3.09809	6.678221	3.14071816	4.51752196	5.67922052	307	1149	2426
105374119	NR_146711	LINC02004	long intergenic non-protein codin	-2.825824255	6.409770888	1.259871	0.451912	8.024856	0.46682716	0.18071335	1.79260867	15	7	119
558	NM_001278599,NM_001699,NM	AXL	AXL receptor tyrosine kinase	4.640606368	6.407083292	19.584011	91.343144	118.29959	6.51045123	8.71233037	9.17674051	3540	21950	27897
5918	NM_002888,NM_206963	RARRES1	retinoic acid receptor responder '	2.396452719	6.393557774	50.17902	119.804252	305.631485	6.20945718	7.45898336	8.86958264	2866	9177	22538
9143	NM_004209	SYNGR3	synaptogyrin 3	-1.511832526	6.390978081	0.422822	0.286524	2.607387	0.89996265	0.65251278	2.71151569	34	30	268
6307	NM_001017369,NM_006745	MSMO1	methylsterol monooxygenase 1	1.072220255	6.386070481	18.192117	20.288469	109.786806	5.34005262	5.43825945	7.98471176	1551	2222	12183
221711	NM_001040274	SYCP2L	synaptonemal complex protein 2	-2.171056089	6.383693115	0.033444	0.018701	0.250656	0.17294258	0.08023518	0.87055986	5	3	40
8854	NM_001206897,NM_003888,NM	ALDH1A2	aldehyde dehydrogenase 1 famil	-1.002019576	6.306116116	0.014141	0.021191	0.128905	0.10623215	0.10601836	0.58209675	3	4	24
81555	NM_001024947,NM_001271732	YIPF5	Yip1 domain family member 5	4.60925422	6.173703669	3.499919	16.282181	20.427337	3.58538547	5.6928694	6.10742084	432	2661	3281
5653	NM_001012964,NM_001012965	KLK6	kallikrein related peptidase 6	-1.388525543	6.123645732	194.122911	140.171544	1113.543208	8.32891675	7.85710041	10.93955947	12585	12110	94787
9456	NM_001277077,NM_001277078	HOMER1	homer scaffolding protein 1	1.962960039	6.103415346	0.705685	1.394991	4.070976	1.9928118	2.77662633	4.26329596	117	307	879
23471	NM_001317804,NM_001317805	TRAM1	translocation associated membra	-1.137393962	6.067649937	14.793134	12.681336	83.4332	5.50206729	5.3206905	8.07644721	1740	2044	12986
56113	NM_018915,NM_032009	PCDHGA2	protocadherin gamma subfamily	-2.171056089	6.065604981	0.040345	0.020039	0.156353	0.17294258	0.08023518	0.83750224	5	3	38
6713	NM_003129	SQLE	squalene epoxidase	1.192043883	6.043043041	16.369202	19.53721	92.993843	5.60455895	5.85322458	8.17494733	1871	2980	13907
54756	NM_001318864,NM_017563	IL17RD	interleukin 17 receptor D	1.715790206	6.031818886	0.080698	0.137935	0.45769	0.7550755	1.12560759	2.36808966	27	62	201
693199	NR_030345	MIR614	microRNA 614	-1.459211595	5.995991962	58.222504	40.039917	329.168732	2.61345921	2.17238371	4.98695254	201	184	1483
56145	NM_018906,NM_031497	PCDHA3	protocadherin alpha 3	2.967818323	5.983895882	0.390784	1.160356	1.342632	0.95786335	1.92688293	2.73414401	37	147	273
57563	NM_001292003,NM_001292006	KLHL8	kelch like family member 8	4.119393906	5.976005814	0.634022	2.719948	3.696686	2.17473011	3.95309996	4.46073909	138	760	1015
7042	NM_001135599,NM_003238,NR	TGFB2	transforming growth factor beta 2	4.360733749	5.955037437	2.453542	10.75691	13.819675	3.92238208	5.97190201	6.41530249	556	3240	4073
23306	NM_001130963,NM_015257	NEMP1	nuclear envelope integral membr	4.623520914	5.919491927	3.60452	16.313786	19.435152	4.32203244	6.47351845	6.82645897	746	4609	5432
56917	NM_001009813,NM_001301059	MEIS3	Meis homeobox 3	1.540034068	5.911023532	0.183246	0.303916	1.139882	0.49317359	0.70406985	1.77525664	16	33	117
768	NM_001216	CA9	carbonic anhydrase 9	-1.969526583	5.871351378	0.101009	0.037841	0.554105	0.20517559	0.10601836	0.93448595	6	4	44
6280	NM_002965	S100A9	S100 calcium binding protein A9	7.393418619	5.859876071	10.75774	79.878357	59.43008	2.82010209	5.51844844	5.19129304	238	2352	1716
55092	NM_001136216,NM_001136217	TMEM51	transmembrane protein 51	1.366495247	5.851548786	6.672874	9.067343	36.399371	3.76370129	4.18548887	6.22192148	494	902	3556
29986	NM_001256588,NM_014579	SLC39A2	solute carrier family 39 member 2	2.015933494	5.846043131	0.117768	0.261003	0.69589	0.23670415	0.44594451	1.03996283	7	19	51
654	NM_001718	BMP6	bone morphogenetic protein 6	-2.451716694	5.837267279	0.082964	0.03621	0.494498	0.41264541	0.18071335	1.5590879	13	7	94
100126270	NR_024499,NR_024501,NR_02	FMR1-AS1	FMR1 antisense RNA 1	-1.313695475	5.827286134	0.02192	0.016342	0.138199	0.07168335	0.05398282	0.39027104	2	2	15
100874246	NR_046748	ARHGAP31-AS1	ARHGAP31 antisense RNA 1	6.297462665	5.783608445	0.08385	0.793352	0.697744	0.10623215	0.58077243	0.54161868	3	26	22
10099	NM_001168412,NM_005724,NM	TSPAN3	tetraspanin 3	1.599291761	5.777724728	8.409212	13.406247	45.372048	5.01136367	5.67198336	7.50450249	1227	2622	8720
3481	NM_000612,NM_001007139,NM	IGF2	insulin like growth factor 2	-1.416866335	5.759985759	0.43902	0.309695	2.543937	1.6852613	1.3579479	3.78425716	87	82	617
2182	NM_001318509,NM_001318510	ACSL4	acyl-CoA synthetase long-chain f	3.736561829	5.748308779	1.642777	6.201255	8.950885	3.18986937	4.97122547	5.5765281	319	1593	2256
56111	NM_018917,NM_032053	PCDHGA4	protocadherin gamma subfamily	-1.647024248	5.747516848	0.038672	0.027397	0.145799	0.17294258	0.10601836	0.80366934	5	4	36
56110	NM_018918,NM_032054	PCDHGA5	protocadherin gamma subfamily	-2.596168628	5.738328447	0.064066	0.022968	0.189827	0.20517559	0.08023518	0.91876801	6	3	43
65061	NM_001261435,NM_001261436	CDK15	cyclin dependent kinase 15	-1.743397756	5.733723027	1.097743	0.593496	5.658888	2.19894236	1.61299082	4.43334413	141	108	995
5672	NM_001276495,NM_001316339	PSG4	pregnancy specific beta-1-glycop	-1.061016959	5.717327607	4.067406	3.855093	21.481207	3.2098649	3.13387411	5.59085682	324	408	2279
6319	NM_005063	SCD	stearoyl-CoA desaturase	-1.373016012	5.684779493	44.146286	32.190121	235.859589	7.88293269	7.42785606	10.38500594	9228	8980	64522
3732	NM_001024844,NM_002231	CD82	CD82 molecule	-1.134051212	5.682424846	10.758735	9.493948	57.54989	4.22711655	4.0558926	6.66889605	696	820	4865
126567	NM_001136263	C2CD4C	C2 calcium dependent domain cc	2.485947131	5.662317627	0.150571	0.374483	0.800673	0.54446555	1.10017522	1.85174813	18	60	126
6566	NM_001166496,NM_003051	SLC16A1	solute carrier family 16 member '	6.188488349	5.634254773	7.18995	44.645502	38.122454	4.82946313	7.41598785	7.28145967	1077	8906	7464
30817	NM_001271052,NM_013447	ADGRE2	adhesion G protein-coupled rece	2.373702116	5.614803769	0.111697	0.274103	0.598001	0.77668602	1.43116693	2.32699908	28	89	194
146330	NM_153350	FBXL16	F-box and leucine rich repeat pro	2.276219127	5.605707379	0.321384	0.738783	1.712036	1.08456011	1.82964782	2.86703245	44	134	304
441295	NR_002157,NR_002157_dup1	OR2A9P	olfactory receptor family 2 subfan	4.797099517	5.605305516	0.14664	0.763127	0.840063	0.20517559	0.80196545	0.90287695	6	39	42
387700	NM_213606	SLC16A12	solute carrier family 16 member '	4.919652754	5.605305516	0.032378	0.167797	0.17791	0.20517559	0.81765451	0.90287695	6	40	42
8702	NM_003778,NM_212543	B4GALT4	beta-1,4-galactosyltransferase 4	1.101003471	5.568628881	24.630831	27.093514	129.517731	5.79727387	5.93371747	8.25321487	2144	3154	14685
3311	NR_024151	HSPA7	heat shock protein family A (Hsp	1.483922171	5.563453139	0.036677	0.060527	0.220343	0.13997292	0.20477631	0.65979727	4	8	28
127534	NM_153212	GJB4	gap junction protein beta 4	1.213670505	5.539444988	3.478654	4.227701	18.128876	3.40980893	3.66515371	5.76413316	378	613	2576
29957	NM_013386,NM_213651	SLC25A24	solute carrier family 25 member 2	3.021920971	5.538169011	4.023449	12.138538	20.906769	3.90776647	5.43762501	6.29641656	550	2221	3747
23551	NM_014310	RASD2	RASD family member 2	6.644757684	5.52860212	1.210371	8.079565	6.311274	2.17473011	4.60714063	4.35374951	138	1226	939
4739	NM_001142393,NM_001271033	NEDD9	neural precursor cell expressed,	4.334238303	5.465387749	0.170704	0.719898	1.01929	0.83964067	2.14776967	2.41366048	31	180	209

84217	NM_001146192,NM_032257	ZMYND12	zinc finger MYND-type containing	1.244483702	5.455181203	0.117976	0.167992	0.68509	0.29776659	0.36283659	1.17840727	9	15	61
9912	NM_001321164,NM_001321166	ARHGAP44	Rho GTPase activating protein 4	2.671921329	5.432958501	0.408011	1.104925	2.220355	1.44997615	2.49375478	3.38166197	68	243	455
129049	NM_001039948,NM_001098497	SGSM1	small G protein signaling modula	1.251670471	5.43163231	0.217885	0.277645	1.135665	1.21691895	1.41062362	3.03682664	52	87	348
102723775	NR_120595,NR_120596,NR_12	LINC01315	long intergenic non-protein codin	-1.067926637	5.426067311	0.166828	0.236441	1.13896	0.26755841	0.25173066	1.08290869	8	10	54
6286	NM_005980	S100P	S100 calcium binding protein P	-1.869757496	5.404939262	152.376846	81.595787	774.087891	6.26067799	5.37405578	8.679584	2971	2123	19751
139886	NM_001012968	SPIN4	spindlin family member 4	1.195822577	5.371699287	5.19834	6.225706	26.271801	4.44859483	4.69576755	6.8193336	818	1307	5405
121551	NM_001017523,NM_001018072	BTBD11	BTB domain containing 11	2.026014849	5.34556057	0.35836	0.727952	1.832407	1.59082596	2.34470901	3.5569002	79	214	520
3037	NM_005328	HAS2	hyaluronan synthase 2	1.087777966	5.278184906	0.079882	0.095704	0.437503	0.35634914	0.38406669	1.31676564	11	16	72
60494	NM_001156474,NM_021827	CCDC81	coiled-coil domain containing 81	2.963716788	5.274447077	0.065664	0.192532	0.327562	0.23670415	0.61708849	0.96541723	7	28	46
8659	NM_001161504,NM_001319218	ALDH4A1	aldehyde dehydrogenase 4 famil	1.445286607	5.270315271	1.032442	1.306957	4.527497	1.9928118	2.40826098	4.0633618	117	226	759
10495	NM_001281736,NM_006375,NM	ENOX2	ecto-NOX disulfide-thiol exchang	6.287246153	5.264377143	0.508874	3.271654	2.601595	1.63881618	3.83857782	3.60188752	83	698	538
57348	NM_001005367,NM_001201461	TTYH1	tweety family member 1	-3.664933469	5.250790878	0.19209	0.04118	0.933177	0.43999062	0.13134883	1.52834475	14	5	91
345079	NM_001029870	SOWAHB	sosondowah ankyrin repeat dom	1.11290146	5.247454735	0.690963	0.771917	3.446748	1.57857476	1.68318474	3.51569869	78	116	504
4147	NM_001317748,NM_002380,NM	MATN2	matrilin 2	1.560775477	5.228139604	1.538251	2.417412	7.656017	2.85607368	3.42511354	5.07201438	245	511	1576
2192	NM_001996,NM_006485,NM_00	FBLN1	fibulin 1	1.962393327	5.227986012	16.414405	32.279321	80.850689	5.29583597	6.25037564	7.65216706	1503	3941	9665
106633805	NR_132757	SNORD136	small nucleolar RNA, C/D box 13	2.801003507	5.218424998	0.065196	0.514894	0.919511	0.03628685	0.10601836	0.19530369	1	4	7
595098	NR_002441	SNORD18A	small nucleolar RNA, C/D box 18	4.153170347	5.218424998	0.35312	1.458338	1.806578	0.03628685	0.15624221	0.19530369	1	6	7
5362	NM_025179	PLXNA2	plexin A2	-1.335507325	5.20581966	0.178118	0.133358	0.872951	1.57857476	1.31437719	3.5052118	78	78	500
79170	NM_024320	PRR15L	proline rich 15 like	-3.842074464	5.189771366	6.499468	1.692307	31.710238	3.40634723	1.80624674	5.66809934	377	131	2407
5284	NM_002644	PIGR	polymeric immunoglobulin recept	-2.059975106	5.178292026	1.215255	0.590307	5.925118	2.60139951	1.79043244	4.76868199	199	129	1268
407013	NR_029497	MIR24-2	microRNA 24-2	2.577321332	5.168011028	1.338325	3.614246	7.093835	0.13997292	0.3412894	0.62147005	4	14	26
145837	NR_026979	DRAIC	downregulated RNA in cancer, in	2.577321332	5.168011028	0.061375	0.151247	0.298258	0.13997292	0.3412894	0.62147005	4	14	26
11199	NM_007193	ANXA10	annexin A10	-1.00641791	5.136653751	1.048352	1.033013	5.060766	1.30878272	1.30327576	3.10438964	58	77	367
51301	NM_016591	GCNT4	glucosaminyl (N-acetyl) transfera	-1.330220287	5.125386915	0.060836	0.045638	0.295129	0.26755841	0.20477631	1.03996283	8	8	51
9459	NM_001306177,NM_004840	ARHGEF6	Rac/Cdc42 guanine nucleotide e	4.56401305	5.111340581	0.019861	0.114331	0.121319	0.17294258	0.669904	0.73352601	5	31	32
201625	NM_001291661,NM_198564	DNAH12	dynein axonemal heavy chain 12	7.34846829	5.111340581	0.010928	0.101792	0.06666	0.17294258	0.96581954	0.73352601	5	50	32
93273	NM_001001552,NM_001199050	LEMD1	LEM domain containing 1	-2.542521394	5.105742719	0.687189	0.233316	3.999359	0.66524311	0.29720487	2.0010274	23	12	145
9252	NM_001322227,NM_001322228	RPS6KA5	ribosomal protein S6 kinase A5	-1.082508635	5.077677706	1.000161	0.956014	4.70851	2.73434891	2.63769986	4.89354711	222	274	1387
6480	NM_003032,NM_173216,NM_17	ST6GAL1	ST6 beta-galactoside alpha-2,6-s	3.461686908	5.066390822	0.546682	1.907495	2.625146	1.79522562	3.25693382	3.75799726	97	449	605
677797	NR_002992	SNORA7B	small nucleolar RNA, H/ACA box	6.444584108	5.057391985	0.344742	2.41966	1.850897	0.07168335	0.42561204	0.34394329	2	18	13
100034743	NR_111936,NR_111936_dup1	PDZK1P1	PDZ domain containing 1 pseud	1.8268438	5.057391985	0.016384	0.044743	0.116582	0.07168335	0.13134883	0.34394329	2	5	13
56106	NM_018913,NM_032090	PCDHGA10	protocadherin gamma subfamily	13.06369279	5.053167882	0.154521	1.204835	0.458797	0.49317359	2.66830768	1.61867597	16	281	100
971	NM_001782	CD72	CD72 molecule	-1.03859314	5.045808655	0.1014	0.1013	0.563539	0.23670415	0.2284445	0.93448595	7	9	44
57822	NM_001195010,NM_021180,NM	GRHL3	grainyhead like transcription fact	-6.321471767	5.043349393	0.731187	0.115539	3.518275	1.61502061	0.40498891	3.51308411	81	17	503
8293	NM_021967,NM_021967_dup1,N	SERF1A	small EDRK-rich factor 1A	6.504701236	5.04136689	1.521323	17.019	13.114139	1.56621863	3.78372009	3.44613034	77	670	478
6275	NM_002961,NM_019554	S100A4	S100 calcium binding protein A4	4.005665612	5.037296254	262.884314	1054.487697	1244.978778	7.04826348	9.04212559	9.37217527	5157	27601	31951
8013	NM_006981,NM_173199,NM_17	NR4A3	nuclear receptor subfamily 4 grou	7.398749261	5.025160116	0.036588	0.272644	0.172689	0.26755841	1.33632702	1.02535867	8	80	50
339122	NM_001204883,NM_001204884	RAB43	RAB43, member RAS oncogene	-1.248410731	5.017251693	1.99067	1.595507	9.416756	3.27963753	2.99588686	5.48276272	342	366	2111
55289	NM_001142807	ACOXL	acyl-CoA oxidase like	-1.662261819	5.010937617	0.155124	0.091036	0.777729	0.46682716	0.29720487	1.5489128	15	12	93
125965	NM_144613	COX6B2	cytochrome c oxidase subunit 6B	-1.350795383	5.010786998	10.082799	7.472102	47.484921	4.11494833	3.70997765	6.37195038	641	634	3951
90249	NM_133369	UNC5A	unc-5 netrin receptor A	-1.039198952	5.007365195	4.168926	4.012989	19.62249	4.01393646	3.96195135	6.2649044	595	765	3665
643036	NR_003542	SLED1	proteoglycan 3 pseudogene	-1.937182024	4.999846938	0.104645	0.052271	0.498717	0.10623215	0.05398282	0.47868902	3	2	19
128178	NM_080738,NM_145861	EDARADD	EDAR associated death domain	1.617934183	4.98896908	0.158944	0.258387	0.778424	0.54446555	0.80196545	1.72191206	18	39	111
151887	NM_199511,NM_199512	CCDC80	coiled-coil domain containing 80	4.173405979	4.964955762	0.800516	3.622273	3.890055	2.23060596	4.03758735	4.27414582	145	809	886
55890	NM_018653,NM_022036	GPRC5C	G protein-coupled receptor class	-1.064789511	4.960672969	9.726355	9.150334	45.453976	4.55403551	4.46742999	6.81482914	883	1108	5388
3398	NM_002166	ID2	inhibitor of DNA binding 2, HLH p	1.412127389	4.954443206	2.545409	3.593384	11.878061	2.13345087	2.53229878	4.15342015	133	251	811
3853	NM_005554	KRT6A	keratin 6A	-1.647024248	4.952296514	0.042712	0.031765	0.25259	0.17294258	0.10601836	0.71544405	5	4	31
83483	NM_031310	PLVAP	plasmalemma vesicle associated	4.124362222	4.952296514	0.056774	0.23535	0.267307	0.17294258	0.61708849	0.71544405	5	28	31
23670	NM_001135820,NM_001349784	TMEM2	transmembrane protein 2	1.085730009	4.915572203	2.923764	3.24796	13.722276	4.29233333	4.40518902	6.52999085	730	1059	4414
148398	NM_152486	SAMD11	sterile alpha motif domain contai	-1.23825173	4.845145219	0.129106	0.103977	0.609906	0.41264541	0.3412894	1.38699077	13	14	78
57804	NM_001256870,NM_021173,NR	POLD4	DNA polymerase delta 4, access	-1.079282581	4.836501796	16.526307	15.606338	75.487934	4.83333524	4.72725823	7.06670597	1080	1337	6425
283875	NR_033861	LINC00514	long intergenic non-protein codin	-1.980810008	4.83058868	0.072938	0.036513	0.332095	0.29776659	0.15624221	1.08290869	9	6	54
4061	NM_001127213,NM_002346	LY6E	lymphocyte antigen 6 family mem	1.017946488	4.829715097	202.222977	206.090164	917.747986	7.85939016	7.88494265	10.12640778	9078	12347	53926
387646	NR_003525	LRRC37A6P	leucine rich repeat containing 37	2.858407533	4.817170233	0.028053	0.08113	0.127132	0.23670415	0.59904473	0.90287695	7	27	42
254100	NR_038923	SSSCA1-AS1	SSSCA1 antisense RNA 1 (head	2.193763055	4.805383315	0.360332	0.816937	1.679529	0.5190475	0.96581954	1.62837236	17	50	101
152110	NM_001031741,NM_001304384	NEK10	NIMA related kinase 10	15.11563291	4.793252448	0.02491	0.486955	0.146369	0.17294258	1.5673195	0.69713258	5	103	30
8459	NM_001008566,NM_003595	TPST2	tyrosylprotein sulfotransferase 2	2.399457367	4.787022989	0.875139	2.217061	4.158954	1.43646263	2.35011326	3.19852188	67	215	395
10846	NM_001130690,NM_006661	PDE10A	phosphodiesterase 10A	9.463762906	4.786387947	0.1236	1.192516	0.56664	1.01352966	3.41483526	2.5576298	40	507	236
647859	NR_026578	LOC647859	occludin pseudogene	-1.176759292	4.784674198	3.320462	2.844481	15.058135	1.5285023	1.37924953	3.32614297	74	84	436
7163	NM_001025252,NM_001025253	TPD52	tumor protein D52	-1.157690282	4.765338719	5.773055	5.002136	25.739469	4.56339093	4.36170552	6.76707475	889	1026	5211
4597	NM_002461	MVD	mevalonate diphosphate decarbo	1.005851161	4.754389605	11.50523	11.584016	51.422272	4.44691093	4.45494398	6.64312125	817	1098	4778
80352	NM_025236,NM_170769	RNF39	ring finger protein 39	-2.994591769	4.751788339	1.426056	0.473737	6.409615	2.01115992	1.00745609	3.94613924	119	53	696
353091	NM_001001788,NR_130110	RAET1G	retinoic acid early transcript 1G	1.342404334	4.74246244	0.916821	1.199944	4.065298	0.99521185	1.22307764	2.51644942	39	70	228
80853	NM_030647	KDM7A	lysine demethylase 7A	-1.269838379	4.734893573	0.491855	0.387516	2.195139	2.43469539	2.16012918	4.45120991	173	182	1008
3352	NM_000864	HTR1D	5-hydroxytryptamine receptor 1D	-2.265021151	4.730610826	1.747192	0.76795	7.795464	2.54584744	1.64851465	4.57920996	190	112	1106
7832	NM_006763	BTG2	BTG anti-proliferation factor 2	1.35455329	4.685379871	1.316419	1.789131	5.817461	2.16656821	2.51796519	4.11772436	137	248	790
91748	NM_001043318,NM_194278	ELMSAN1	ELM2 and Myb/SANT domain co	1.397168427	4.681797484	2.036533	2.92424	9.247551	4.09358292	4.55193121	6.25280965	631	1178	3634
1191	NM_001831,NR_038335,NR_04	CLU	clusterin	1.788727486	4.677646797	2.828689	5.072223	12.43755	3.15316384	3.91896052	5.24585326	310	741	1784
445577	NM_001098808	C9orf129	chromosome 9 open reading fran	2.4480728	4.674653892	0.316371	0.773779	1.402017	0.43999062	0.90836632	1.42086173	14	46	81
56120	NR_001297	PCDHGB8P	protocadherin gamma subfamily	3.602897765	4.67244491	0.026519	0.099251	0.116957	0.07168335	0.25173066	0.32020933	2	10	12
101929536	NR_135805	LOC101929536	uncharacterized LOC101929536	3.247686972	4.67244491	0.043568	0.131224	0.200553	0.07168335	0.2284445	0.32020933	2	9	12
5744	NM_002820,NM_198964,NM_1	PTHLH	parathyroid hormone like hormon	10.8869987	4.670415343	0.686059	8.639999	3.61758	1.10178443	3.75548863	2.66986823	45	656	259
93082	NM_001285485,NM_001285486	NEURL3	neuralized E3 ubiquitin protein li	1.144092858	4.655466545	1.778703	2.03632	7.064969	1.86749926	2.01116974	3.73798383	104	159	596
3157	NM_001098272,NM_001324219	HMGCS1	3-hydroxy-3-methylglutaryl-CoA	-1.029649203	4.654747482	2.650117	2.583182	11.748344	3.91021271	3.87089436	6.05176067	551	715	3155
26503	NM_012434	SLC17A5	solute carrier family 17 member	1.30833646	4.652289745	2.254137	2.953823	9.870309	3.05045588	3.39667017	5.1252021	286	500	1637
81539	NM_001077484,NM_001278387	SLC38A1	solute carrier family 38 member	1.280748118	4.651501433	4.243234	5.451295	17.689513	5.06833013	5.41588625	7.25196951	1278	2187	7312
10020	NM_001128227,NM_001190383	GNE	glucosamine (UDP-N-acetyl)-2-e	-1.122671704	4.635282291	7.19424	6.540603	31.944689	5.25215086	5.08983838	7.43489686	1457	1734	8307
23179	NM_001297669,NM_001297670	RGL1	ral guanine nucleotide dissociati	5.882965532	4.634208381	0.020288	0.150166	0.112035	0.17294258	0.81765451	0.6785857	5	40	29
110116772	NM_001350496	ZNF765-ZNF761	ZNF765-ZNF761 readthrough	2.825007878	4.632373934	0.393262	1.115014	1.716028	1.61502061	2.77260719	3.40158951	81	306	462
26152	NM_001290261,NM_015655	ZNF337	zinc finger protein 337	7.567904414	4.610109597	3.049152	23.001839	13.355105	3.85541331	6.6863532	5.98020636	529	5350	3000
102546175	NR_105016	LINC01338	long intergenic non-protein codin	1.213618985	4.598323757	1.853687	2.23821	8.07547	1.10178443	1.25799553	2.65096518	45	73	255
57016	NM_020299	AKR1B10	aldo-keto reductase family 1 men	1.7212194	4.597114704	0.196701	0.360669	0.918776	0.41264541	0.65251278	1.34055586	13	30	74
84720	NM_001201484,NM_032634,NM	PIGO	phosphatidylinositol glycan ancho	2.680309188	4.573756989	2.046469	5.883399	9.27083	3.15728865	4.47487005	5.21883106	311	1114	1750
105375355	NM_001347684,NM_030570,NM	UPK3B	uroplakin 3B	1.004792909	4.560297746	101.200313	102.235546	438.177259	7.23474357	7.24159619	9.41639548	5874	7886	32947
51666	NM_016116,NM_145872	ASB4	ankyrin repeat and SOCS box co	-1.142340232	4.541122068	0.040845	0.030756	0.177386	0.20517559	0.18071335	0.76902392	6	7	34
284729	NR_026567	ESPNP	espin pseudogene	4.347903262	4.535107075	0.15659	0.737965	0.714847	0.41264541	1.29208824	1.32870978	13	76	73
54946	NM_001008485,NM_001008486	SLC41A3	solute carrier family 41 member	3.515735531	4.503225083	3.020482	10.741072	12.775496	2.92543304	4.59694851	4.94100562	259	1217	1435
114299	NM_001037293,NM_053016	PALM2	paralemmin 2	1.963208792	4.500206932	0.198523	0.396775	0.844056	1.50279892	2.20257223	3.21148164	72	189	399
100113392	NR_003694	SNORD11B	small nucleolar RNA, C/D box 11	3.477086927	4.48733701	0.164202	0.816157	0.895867	0.03628685	0.13134883	0.16897038	1	5	6
407010	NR_029495	MIR23A	microRNA 23a	1.238869866	4.477339267	0.936739	1.201673	4.454187	0.10623215	0.13134883	0.43515728	3	5	17
9201	NM_001195415,NM_001195416	DCLK1	doublecortin like kinase 1	10.57257436	4.475164314	0.019655	0.202134	0.078877	0.17294258	1.24644988	0.65979727	5	72	28
23762	NM_001282738,NM_001282739	OSBP2	oxysterol binding protein 2	1.461389588	4.472813885	4.992743	7.151072	21.140072	4.42312766	4.94913788	6.53128351	803	1568	4418
9712	NM_001080491,NM_014688,NR	USP6NL	USP6 N-terminal like	2.856478048	4.458371158	3.897041	8.620695	12.327008	3.81681978	5.26312825	5.89189395	514	1962	2819
57493	NM_020733	HEG1	heart development protein with E	1.979260921	4.445898704	0.489303	0.973408	2.052202	2.4278825	3.27409343	4.3566691	172	455	941
168451	NM_001130475,NM_001287598	THAP5	THAP domain containing 5	-1.052434386	4.443093887	1.071841	1.028349	4.56036	2.19894236	2.14154997	4.08464905	141	179	771
55902	NM_001076552,NM_001242393	ACSS2	acyl-CoA synthetase short-chain	-1.211704206	4.440095626	13.508001	11.16403	56.386334	5.32912486	5.05964438	7.45171991	1539	1697	8405
3953	NM_001003679,NM_001003680	LEPR	leptin receptor	1.00437455	4.424445673	2.181325	2.196208	8.800768	3.43720745	3.44292618	5.47606585	386	518	2101
54800	NM_001349413,NM_001349414	KLHL24	kelch like family member 24	1.431939154	4.418254595	1.594777	2.754105	8.184248	3.91752664	4.40648646	5.98540307	554	1060	3011
65078	NM_023004	RTN4R	reticulon 4 receptor	-1.31580944	4.399348864	0.908941	0.692867	3.773472	1.43646263	1.19932051	3.09042641	67	68	363

4610	NM_001033081,NM_001033082	MYCL	MYCL proto-oncogene, bHLH tra	-2.780399894	4.39557723	0.145037	0.053834	0.856879	0.61813834	0.25173066	1.74883089	21	10	114
901	NM_004354	CCNG2	cyclin G2	1.002790384	4.379826453	5.130341	5.151721	21.125847	4.8255806	4.82945947	6.91676387	1074	1439	5786
57514	NM_020754	ARHGAP31	Rho GTPase activating protein 3	3.036369359	4.377851746	0.096536	0.295065	0.398823	0.81895953	1.73367602	2.12264217	30	122	162
57578	NM_001346218,NM_020818,NR	UNC79	unc-79 homolog (C. elegans)	1.848388558	4.377126806	0.058883	0.107706	0.225511	0.13997292	0.25173066	0.54161868	4	10	22
540	NM_000053,NM_001005918,NM	ATP7B	ATPase copper transporting beta	2.134049843	4.371169344	0.108046	0.236012	0.454018	0.77668602	1.33632702	2.04513029	28	80	151
23650	NM_001330382,NM_012101	TRIM29	tripartite motif containing 29	1.498579221	4.350918549	7.311059	10.968234	29.999393	4.49170017	5.05386023	6.5629131	844	1690	4517
54478	NM_001195228,NM_019013	PIMREG	PICALM interacting mitotic regula	1.757397697	4.350456608	4.172895	7.347073	17.088879	2.83562844	3.55948145	4.79259544	241	566	1290
90293	NM_001168299,NM_001168300	KLHL13	kelch like family member 13	3.498342783	4.333765766	0.267954	0.947125	1.310379	1.03161781	2.2203867	2.46867149	41	192	219
11237	NM_001134337,NM_001134338	RNF24	ring finger protein 24	2.515161884	4.333274647	1.391236	3.522577	5.710859	3.47073972	4.72101485	5.48276272	396	1331	2111
257000	NR_027064	TINCR	tissue differentiation-inducing nor	2.777296939	4.333063493	0.222247	0.622518	0.911162	0.86002952	1.70865125	2.18310266	32	119	171
57158	NM_020433,NM_175913	JPH2	junctophilin 2	-1.167609212	4.3289717	0.095393	0.065462	0.321856	0.43999062	0.38406669	1.35230545	14	16	75
3560	NM_000878,NM_001346222,NM	IL2RB	interleukin 2 receptor subunit bet	2.072658361	4.316120248	0.025455	0.066659	0.130796	0.17294258	0.3412894	0.64076093	5	14	27
51560	NM_016577	RAB6B	RAB6B, member RAS oncogene	3.47489894	4.316090962	0.465029	1.618994	1.890745	1.81624731	3.28823882	3.57205238	99	460	526
23193	NM_001278192,NM_001278193	GANAB	glucosidase II alpha subunit	1.430906564	4.30959993	35.283668	49.50462	139.181779	7.05629268	7.56996307	9.15552195	5186	9915	27489
3480	NM_000875,NM_001291858	IGF1R	insulin like growth factor 1 recept	-1.457706714	4.306081973	1.099857	0.755045	4.455131	3.81942505	3.3216324	5.84536435	515	472	2728
81537	NM_030791	SGPP1	sphingosine-1-phosphate phosph	1.193252315	4.297421139	7.990672	9.542398	32.274841	4.76470172	5.01100366	6.82698537	1028	1639	5434
79948	NM_001270366,NM_024888	PLPPR3	phospholipid phosphatase relatec	1.271370539	4.296849364	0.314988	0.431213	1.377972	0.81895953	0.97983233	2.10191219	30	51	159
10228	NM_001286210,NM_005819	STX6	syntaxin 6	1.362784949	4.295226539	2.393076	3.266961	9.598739	3.66286259	4.07885924	5.67571782	458	834	2420
11228	NM_001164746,NM_001164747	RASSF8	Ras association domain family m	1.989120878	4.288407295	0.235608	0.470342	1.017499	1.23264156	1.88281796	2.76523898	53	141	280
2199	NM_001004019,NM_001165035	FBLN2	fibulin 2	-1.313695475	4.287497836	0.011422	0.008558	0.043633	0.07168335	0.05398282	0.29607837	2	2	11
60598	NM_022358	KCNK15	potassium two pore domain chan	-1.442196925	4.28707656	0.134115	0.087763	0.544469	0.41264541	0.29720487	1.28032816	13	12	69
10484	NM_006364	SEC23A	Sec23 homolog A, coat complex	1.453926335	4.275345552	7.942242	11.559743	31.917768	5.04403172	5.57030594	7.10627477	1256	2440	6605
114898	NM_031908	C1QTNF2	C1q and TNF related 2	2.223481544	4.275076206	0.065269	0.146953	0.265358	0.20517559	0.42561204	0.73352601	6	18	32
7057	NM_003246	THBS1	thrombospondin 1	5.158409337	4.273002491	8.870279	45.809536	35.620831	5.98998006	8.33854101	8.06778787	2456	16928	12908
23474	NM_001320867,NM_001320868	ETHE1	ETHE1, persulfide dioxygenase	-1.756530893	4.265717615	17.877587	10.329294	71.088996	4.11070049	3.35963909	6.13828834	639	486	3353
5187	NM_002616	PER1	period circadian clock 1	1.854991853	4.252240583	5.326161	9.893996	21.289877	4.66827758	5.53337122	6.7125667	959	2377	5016
83850	NM_001322831,NM_001322834	ESYT3	extended synaptotagmin 3	1.430658782	4.238263528	0.049222	0.071918	0.199681	0.38477185	0.52452456	1.20457043	12	23	63
100506999	NR_146449,NR_146450	LINC02318	long intergenic non-protein codin	-1.099499183	4.213744303	0.377376	0.242524	1.160832	0.43999062	0.40498891	1.32870978	14	17	73
155382	NM_001077621	VPS37D	VPS37D, ESCRT-I subunit	-1.126786721	4.202521812	0.439895	0.388514	1.742291	0.7550755	0.68708806	1.96321628	27	32	140
3373	NM_033159,NM_153281,NM_15	HYAL1	hyaluronoglucosaminidase 1	-1.226390523	4.201820035	0.461738	0.373365	2.092144	1.10178443	0.95166931	2.54232458	45	49	233
4680	NM_002483	CEACAM6	carcinoembryonic antigen relatec	-4.732623548	4.197681173	35.664345	7.537007	140.692184	6.51286763	4.32786827	8.5704098	3546	1001	18308
440854	NM_001145122,NM_001321270	CAPN14	calpain 14	3.639086627	4.19207803	0.061561	0.24148	0.266528	0.3273552	0.95166931	1.05442063	10	49	52
100533970	NR_037928	P2RX5-TAX1BP3	P2RX5-TAX1BP3 readthrough (N	3.423121494	4.190826158	0.14885	0.512062	0.588888	0.93882015	2.05153568	2.27855908	36	165	186
54832	NM_001018088,NM_017684,NM	VPS13C	vacuolar protein sorting 13 homo	2.082681524	4.187438686	1.106512	2.327453	4.391838	3.97005831	4.97996669	5.96450295	576	1603	2967
1646	NM_001135241,NM_001321027	AKR1C2	aldo-keto reductase family 1 mer	3.422493825	4.187361888	0.407467	1.515723	2.129207	1.29387258	2.57907432	2.82548777	57	261	294
7107	NM_003272	GPR137B	G protein-coupled receptor 137B	-1.106999957	4.166014144	2.228837	2.011751	8.729435	2.44147625	2.32288731	4.28338119	174	210	892
25849	NM_015393	PARM1	prostate androgen-regulated muc	5.736415256	4.157076181	0.020705	0.150145	0.10228	0.17294258	0.80196545	0.62147005	5	39	26
11076	NM_007030	TPPP	tubulin polymerization promoting	-1.038897293	4.156130604	0.060777	0.058558	0.23665	0.43999062	0.42561204	1.31676564	14	18	72
101927550	NR_110102	LOC101927550	uncharacterized LOC101927550	2.468588018	4.142053275	0.042413	0.111659	0.181462	0.20517559	0.46599441	0.71544405	6	20	31
80823	NM_001142524,NM_001142525	BHLHB9	basic helix-loop-helix family mem	1.350799623	4.132607848	0.199979	0.269248	0.767682	0.83964067	1.04792463	2.09493548	31	56	158
3977	NM_001127671,NM_002310	LIFR	LIF receptor alpha	3.088562223	4.129728449	0.11282	0.355042	0.445805	1.10178443	2.18453503	2.52166171	45	186	229
6320	NM_002975	CLEC11A	C-type lectin domain containing 1	1.494224427	4.129019749	0.371505	0.556432	1.446651	0.59399636	0.81765451	1.63800401	20	40	102
687	NM_001206	KLF9	Kruppel like factor 9	1.512294813	4.125769496	0.711268	1.076582	2.763545	2.20692365	2.69403583	3.99380211	142	287	721
8935	NM_001303468,NM_003930	SKAP2	src kinase associated phosphopr	2.799423572	4.121773167	3.907854	10.963369	15.158452	4.02074421	5.44774276	5.99527239	598	2237	3032
79718	NM_001321193,NM_001321194	TBL1XR1	transducin beta like 1 X-linked re	3.753274924	4.120950964	7.169052	27.135872	27.835089	5.83252285	7.72203193	7.85625366	2198	11023	11141
8987	NM_003943	STBD1	starch binding domain 1	1.390575294	4.064005395	2.437521	3.390039	9.307775	3.00082115	3.42511354	4.88143447	275	511	1375
1185	NM_001256959,NM_001286,NR	CLCN6	chloride voltage-gated channel 6	1.263820577	4.052389777	2.154213	2.724092	8.199994	3.69442889	4.0088325	5.62697525	469	792	2338
728989	NR_024442	LOC728989	phosphodiesterase 4D interacting	1.581260973	4.045172343	0.413088	0.655494	1.578226	0.5190475	0.75384785	1.46482043	17	36	85
100653515	NM_001243540,NM_001243541	CEP295NL	CEP295 N-terminal like	1.295180463	4.042514584	0.177738	0.234307	0.689115	0.46682716	0.58077243	1.35230545	15	26	75
130535	NM_001321547,NM_001321548	KCTD18	potassium channel tetramerizatio	4.297193425	4.040474696	1.356161	5.945559	5.360165	2.23060596	4.07723084	3.99380211	145	833	721
634	NM_001024912,NM_001184813	CEACAM1	carcinoembryonic antigen relatec	-1.209992364	4.033908348	15.606588	12.986877	59.999887	5.72269098	5.45340297	7.71423404	2034	2246	10092
206338	NM_173800	LVRN	laeverin	-1.383577046	4.024108574	0.153306	0.11369	0.599256	0.77668602	0.59904473	1.95553366	28	27	139
1850	NM_004420	DUSP8	dual specificity phosphatase 8	1.560028771	4.021462535	0.476178	0.746624	1.812723	1.62696745	2.09080288	3.23388481	82	171	406
4501	NM_005952	MT1X	metallothionein 1X	1.495509233	4.014892873	2.504632	3.756368	9.515538	0.99521185	1.31437719	2.32103228	39	78	193
54885	NM_017752,NM_198881	TBC1D8B	TBC1 domain family member 8B	1.492674353	4.008150285	0.94378	1.709601	3.87316	2.55212659	3.04669644	4.35812668	191	381	942
100133669	NR_026913	LOC100133669	uncharacterized LOC100133669	-2.806963906	3.997659157	0.602541	0.202093	2.367795	0.5190475	0.20477631	1.45395569	17	8	84
2775	NM_020988,NM_138736	GNAO1	G protein subunit alpha o1	13.63377998	3.983563195	0.113131	1.540185	0.402316	0.68823103	3.22787299	1.78395874	24	439	118
85294	NM_033184	KRTAP2-4	keratin associated protein 2-4	1.301688978	3.981684695	0.119815	0.169312	0.515448	0.13997292	0.18071335	0.49997203	4	7	20
3310	NM_002155	HSPA6	heat shock protein family A (Hsp	1.775457177	3.975609471	0.435659	0.783997	1.659548	1.01352966	1.49109792	2.33885915	40	95	196
6774	NM_003150,NM_139276,NM_21	STAT3	signal transducer and activator o	1.015807952	3.958865	7.451716	7.546907	27.623659	5.19009033	5.21210456	7.14542046	1394	1892	6788
54805	NM_017649,NM_199076,NM_19	CNNM2	cyclin and CBS domain divalent r	-1.050414589	3.944807125	1.851281	1.639296	5.731664	2.82529613	2.76453517	4.64517113	239	304	1160
196913	NR_131171	LINC01599	long intergenic non-protein codin	1.655394661	3.938313647	0.234975	0.397822	0.887016	0.29776659	0.46599441	0.93448595	9	20	44
8682	NM_001297576,NM_001297577	PEA15	phosphoprotein enriched in astro	4.236737257	3.936567633	6.885123	28.87835	25.159476	4.12761734	6.14627712	6.04181814	647	3663	3133
93109	NM_001011655,NM_001166305	TMEM44	transmembrane protein 44	3.958067035	3.935511196	4.008867	16.104132	15.043001	3.3677101	5.2443866	5.2363737	366	1936	1772
2222	NM_001287742,NM_001287743	FDFT1	farnesyl-diphosphate farnesyltrar	-1.012349959	3.934367945	26.262851	26.597814	99.730578	5.86251679	5.84511405	7.82008905	2245	2963	10864
10529	NM_001173484,NM_006393,NM	NEBL	nebulette	1.031151657	3.924306239	0.436005	0.397229	1.768691	2.15010456	2.18453503	3.85822657	135	186	652
58476	NM_001329429,NM_001329430	TP53INP2	tumor protein p53 inducible nucle	1.179913774	3.913129778	1.555627	1.835901	5.734199	2.87121968	3.07960182	4.68520645	248	391	1194
345557	NM_001005473	PLCXD3	phosphatidylinositol specific phos	5.463165951	3.907742814	0.861545	4.71594	3.166483	2.90595399	5.18967248	4.72189817	255	1862	1226
5652	NM_002773	PRSS8	protease, serine 8	-1.068121911	3.906299711	77.079399	72.240685	282.970154	7.14464429	7.05026047	9.10286297	5516	6900	26502
102465523	NR_106927	MIR6867	microRNA 6867	-1.166137663	3.902616546	2.597957	2.175383	9.847919	0.23670415	0.20477631	0.76902392	7	8	34
2886	NM_001030002,NM_001242442	GRB7	growth factor receptor bound pro	1.009290694	3.901244901	13.80982	14.011028	50.964348	4.93533846	4.9482473	6.86387773	1162	1567	5576
10100	NM_001308315,NM_001308316	TSPAN2	tetraspanin 2	1.102181527	3.896465552	3.419165	3.788169	12.608468	3.55131131	3.68025029	5.41924838	421	620	2018
1001	NM_001317195,NM_001317196	CDH3	cadherin 3	-1.019960323	3.896337261	21.756089	21.238615	79.319396	6.30643285	6.27828263	8.25497637	3068	4019	14703
26471	NM_001042483,NM_012385	NUPR1	nuclear protein 1, transcriptional	-1.606975687	3.895785557	7.189358	4.411301	26.246976	2.81488929	2.24959721	4.61622671	237	197	1136
3486	NM_000598,NM_001013398	IGFBP3	insulin like growth factor binding	1.721051412	3.881303982	28.040427	48.354427	102.353002	6.17833593	6.95327869	8.1200553	2804	6448	13386
3156	NM_000859,NM_001130996	HMGCR	3-hydroxy-3-methylglutaryl-CoA r	-1.046044023	3.878963679	11.586868	11.121537	41.998735	5.70236863	5.63869677	7.63738734	2005	2561	9566
2099	NM_000125,NM_001122740,NM	ESR1	estrogen receptor 1	8.106036911	3.876007413	0.022517	0.197708	0.086692	0.20517559	1.1751656	0.6785857	6	66	29
57643	NM_020883	ZSWIM5	zinc finger SWIM-type containing	9.086462805	3.876007413	0.026896	0.247726	0.096307	0.20517559	1.26944952	0.6785857	6	74	29
785	NM_000726,NM_001005746,NM	CACNB4	calcium voltage-gated channel au	2.232117843	3.870463319	0.030734	0.069059	0.110486	0.3273552	0.65251278	0.99569924	10	30	48
50848	NM_001348091,NM_016946	F11R	F11 receptor	1.864567887	3.869242558	6.048324	11.206606	21.798479	4.86520493	5.74097317	6.78018209	1105	2753	5259
94009	NR_027786	SERHL	serine hydrolase-like (pseudogen	-1.41591317	3.8551196	0.316	0.220131	1.182196	0.5190475	0.38406669	1.42086173	17	16	81
1364	NM_001305	CLDN4	claudin 4	-1.844556529	3.831294926	159.748276	86.700951	575.163208	8.15882404	7.27979552	10.0929315	11181	8099	52688
140685	NM_025224	ZBTB46	zinc finger and BTB domain conta	1.057917735	3.830024043	0.13998	0.151092	0.519549	0.79797761	0.83317479	1.9400446	29	41	137
56262	NM_001127244,NM_001127245	LRRC8A	leucine rich repeat containing 8 fa	-1.513027364	3.819172929	37.584158	24.745272	134.356564	7.31816423	6.72534408	9.24475485	6226	5498	29246
79007	NM_001042610,NM_001288708	DBNDD1	dysbindin domain containing 1	-1.059242738	3.817678892	3.188219	3.006901	11.346318	2.94941774	2.87737723	4.73766195	264	333	1240
55680	NM_001042417,NM_001278225	RUFY2	RUN and FYVE domain containir	1.977100775	3.812590111	2.143564	4.281596	8.062031	3.31698997	4.22720295	5.13713733	352	930	1651
100132074	NM_001291281	FOXO6	forkhead box O6	1.19157151	3.803473678	0.590983	0.697442	2.10904	1.24819466	1.40024122	2.64141996	54	86	253
154761	NR_015421	LOC154761	family with sequence similarity 11	8.955483101	3.783963639	0.035397	0.375511	0.143905	0.13997292	0.95166931	0.47868902	4	49	19
1717	NM_001163817,NM_001360	DHCR7	7-dehydrocholesterol reductase	1.359685741	3.783451268	16.053786	21.900486	57.172612	5.4160359	5.85036724	7.31073549	1637	2974	7618
6720	NM_001005291,NM_001321096	SREBF1	sterol regulatory element binding	-1.009122876	3.783268097	20.003403	19.821105	72.395603	6.3951171	6.38217119	8.30212027	3265	4323	15193
100500842	NR_037425	MIR3652	microRNA 3652	-1.064258885	3.782328102	46.119556	43.24897	164.015244	2.78321062	2.70672986	4.53993458	231	290	1075
56605	NM_019891	ERO1B	endoplasmic reticulum oxidoredu	2.962120606	3.776570328	0.64569	1.919084	2.295425	2.0648444	3.38355363	3.70399851	125	495	581
100129617	NR_045112	LOC100129617	uncharacterized LOC100129617	1.104654034	3.76350864	0.856487	0.94613	3.037372	1.38110603	1.47139626	2.81703332	63	93	292
56204	NM_001286495,NM_019600,NR	FAM214A	family with sequence similarity 21	3.46798023	3.760626991	2.163818	7.431451	7.559446	3.36057263	5.05137419	5.16488576	364	1687	1684
145241	NR_003951	ADAM21P1	ADAM metallopeptidase domain	1.448836667	3.756249022	0.012705	0.019038	0.048099	0.03628685	0.05398282	0.14214747	1	2	5
106633810	NR_132762	SCARNA26A	small Cajal body-specific RNA 26	2.124920087	3.756249022	0.019973	0.314111	0.595985	0.03628685	0.08023518	0.14214747	1	3	5
80218	NM_001308445,NM_025146	NAA50	N(alpha)-acetyltransferase 50, Na	7.167095863	3.748534277	7.482587	53.698135	26.3622	5.51979639	8.33394575	7.40294735	1762	16874	8124
259217	NM_001330164,NM_025015	HSPA12A	heat shock protein family A (Hsp	3.648367754	3.741656954	0.048966	0.180041	0.168849	0.35634914	1.02107213	1.03996283	11	54	51
2272	NM_001166243,NM_001320899	FHIT	fragile histidine triad	4.450688488	3.734891266	0.214626	1.003033	0.803094	0.77668602	2.06474386	1.87636985	28	167	129

56977	NM_020225,NR_132761	STOX2	storkhead box 2	1.33486787	3.72916586	0.218716	0.364368	1.144827	1.69664266	1.99745948	3.22751932	88	157	404
100526842	NM_001199355,NM_001199356	RPL17-C18orf32	RPL17-C18orf32 readthrough	1.496517011	3.718631695	2.026445	3.027037	7.088388	2.27683528	2.75641772	3.93641476	151	302	691
5507	NM_005398	PPP1R3C	protein phosphatase 1 regulatory	2.80408886	3.708224237	0.76458	2.171125	2.690935	1.55375576	2.68551066	3.03318152	76	285	347
100131551	NR_024480	LINC00887	long intergenic non-protein codin	-1.216667477	3.700919912	0.355479	0.286636	1.236815	1.03161781	0.89363826	2.28470378	41	45	187
1288	NM_001287758,NM_001287759	COL4A6	collagen type IV alpha 6 chain	10.77756057	3.695221015	0.054214	0.586757	0.187849	0.43999062	2.28388661	1.21747621	14	203	64
403323	NR_122076,NR_122077	LOC403323	uncharacterized LOC403323	2.925067466	3.69357776	0.051084	0.19018	0.191753	0.10623215	0.29720487	0.36729312	3	12	14
100616424	NR_039856	MIR4707	microRNA 4707	1.214769507	3.690691261	5.150648	6.251258	18.22731	0.49317359	0.58077243	1.32870978	16	26	73
94241	NM_001135733,NM_033285	TP53INP1	tumor protein p53 inducible nucle	1.831406569	3.690064941	0.794941	1.460345	2.774546	2.4278825	3.17404113	4.10215159	172	421	781
154141	NM_001080480,NR_073465	MBOAT1	membrane bound O-acyltransfera	-1.556742567	3.68517998	1.79036	1.148008	6.210797	3.09414281	2.54649137	4.84759061	296	254	1342
5978	NM_001193508,NM_005612	REST	RE1 silencing transcription factor	2.969817129	3.678104008	2.040284	6.065433	7.053713	3.96064828	5.46838919	5.77071509	572	2270	2588
54566	NM_018424,NM_019114	EPB41L4B	erythrocyte membrane protein ba	-1.462666252	3.676699961	1.930067	1.333782	6.736832	3.11125378	2.63769986	4.86307268	300	274	1357
2644	NM_005258	GCHFR	GTP cyclohydrolase I feedback r	-1.878143293	3.676177396	2.78313	1.475028	9.631216	1.55375576	1.02107213	3.02219055	76	54	344
100750247	NR_045406	HIF1A-AS2	HIF1A antisense RNA 2	2.647789023	3.673978124	0.082973	0.232966	0.307904	0.23670415	0.56226574	0.73352601	7	25	32
50617	NM_020632,NM_130840,NM_13	ATP6V0A4	ATPase H+ transporting V0 subu	2.691950392	3.667000273	0.104032	0.28969	0.369996	0.41264541	0.92294555	1.15176086	13	47	59
5021	NM_000916	OXTR	oxytocin receptor	1.182470637	3.664844478	2.094989	2.48421	7.232106	3.30959645	3.52883263	5.07378969	350	553	1578
3643	NM_000208,NM_001079817	INSR	insulin receptor	-1.004415343	3.662826502	0.904075	0.90176	3.115507	3.2098649	3.20419534	4.96511882	324	431	1460
79784	NM_001077186,NM_001145809	MYH14	myosin heavy chain 14	-1.984329122	3.66281035	18.077114	9.119238	62.230619	6.91788493	5.94089851	8.78216087	4708	3170	21210
55753	NM_001143996,NM_001143997	OGDHL	oxoglutarate dehydrogenase-like	1.959337245	3.661651372	0.236891	0.46433	0.827072	0.95786335	1.51053416	2.15654364	37	97	167
100506827	NR_131958,NR_131959	LINC02475	long intergenic non-protein codin	-1.526548834	3.661409678	1.974809	1.283518	6.836374	2.55837852	2.07130288	4.24134834	192	168	865
5349	NM_001136007,NM_001136008	FXYD3	FXYD domain containing ion tran	-3.39532746	3.650311902	35.043068	10.463742	119.35993	5.52937575	3.83857782	7.37460163	1774	698	7965
9145	NM_004711,NM_145731,NM_14	SYNGR1	synaptogyrin 1	2.36654173	3.641237686	6.977472	15.357008	20.995755	3.79315817	4.97472832	5.58027977	505	1597	2262
3229	NM_017410	HOXC13	homeobox C13	2.172477755	3.634119609	0.131025	0.280097	0.450074	0.38477185	0.73744528	1.08290869	12	35	54
1032	NM_001800,NM_079421	CDKN2D	cyclin dependent kinase inhibitor	-1.115768515	3.630209422	11.086808	9.640549	38.128672	3.91265481	3.76563483	5.70178309	552	661	2465
148189	NR_027301	LINC00662	long intergenic non-protein codin	1.115358045	3.630154625	0.663725	0.745643	2.306721	1.23264156	1.32539385	2.56269567	53	79	237
51196	NM_001165979,NM_001288989	PLCE1	phospholipase C epsilon 1	-1.716166512	3.629802769	0.720408	0.419918	2.471962	2.72881625	2.09724475	4.42223881	221	172	987
23308	NM_001283050,NM_001283051	ICOSLG	inducible T-cell costimulator ligan	2.325835244	3.623170033	0.422185	0.994614	1.45351	1.24819466	2.07130288	2.58278312	54	168	241
8608	NM_001320108,NM_003708	RDH16	retinol dehydrogenase 16 (all-tra	-1.902600588	3.621829514	0.220013	0.117887	0.770247	0.81895953	0.48576948	1.91649468	30	21	134
5801	NM_001207015,NM_001207016	PTPRR	protein tyrosine phosphatase, rec	2.129280722	3.620771152	0.537144	1.15667	2.356296	1.50279892	2.29513766	2.9350236	72	205	321
11067	NM_007021	C10orf10	chromosome 10 open reading fra	-1.375054942	3.606994259	8.948097	6.515659	30.349192	4.24466875	3.81336732	6.03954888	705	685	3128
109729141	NR_146292,NR_146293	TDRKH-AS1	TDRKH antisense RNA 1	-1.412981023	3.605445714	0.849918	0.638374	3.797875	0.93882015	0.72085408	2.10885534	36	34	160
6721	NM_004599,NR_103834	SREBF2	sterol regulatory element binding	-1.070313945	3.603468062	7.423893	6.979657	25.180297	5.29863957	5.20317354	7.12137423	1506	1880	6675
93010	NM_145236	B3GNT7	UDP-GlcNAc:betaGal beta-1,3-N	-3.394350809	3.598238372	2.602301	0.762676	8.808457	3.34979977	1.89025622	5.09142367	361	142	1598
80223	NM_001002814,NM_025151	RAB11FIP1	RAB11 family interacting protein	-2.923830304	3.597350129	12.188992	4.458042	42.430178	6.37400578	4.85913858	8.20836255	3217	1470	14234
9542	NM_001184935,NM_004883,NM	NRG2	neuregulin 2	1.155161907	3.595320959	0.072158	0.080983	0.245197	0.35634914	0.40498891	1.01060517	11	17	49
57181	NM_001127257,NM_020342	SLC39A10	solute carrier family 39 member 1	1.438162404	3.589575535	2.447735	3.5243	8.258218	3.7744826	4.26631627	5.54039208	498	957	2199
113230	NM_001291291,NR_111941	MISP3	MISP family member 3	-2.556452947	3.584937331	0.918391	0.353434	3.131484	1.15225689	0.56226574	2.43038515	48	25	212
79689	NM_001205315,NM_001205316	STEAP4	STEAP4 metalloreductase	-4.751288635	3.567501665	1.943178	0.387328	6.349806	3.2098649	1.45142181	4.92830565	324	91	1422
6446	NM_001143676,NM_001143677	SGK1	serum/glucocorticoid regulated ki	-1.39146095	3.561844755	1.831002	1.378766	6.357614	2.44822539	2.07130288	4.07758814	175	168	767
64761	NM_022750,NR_130117	PARP12	poly(ADP-ribose) polymerase fan	2.434115816	3.543855476	3.162389	7.705244	10.559289	3.66576097	4.88077062	5.40732977	459	1493	2001
22891	NM_014951,NM_199450,NM_19	ZNF365	zinc finger protein 365	-1.931214258	3.527479676	1.168494	0.604187	3.871983	2.53320657	1.79836126	4.16179054	188	130	816
401089	NM_001040061	FOXL2NB	FOXL2 neighbor	4.677207436	3.522909301	0.242445	1.144272	0.805587	0.97665845	2.46913115	2.1430785	38	238	165
51268	NM_016518	PIPOX	pipecolic acid and sarcosine oxid	3.245060568	3.520899914	0.043385	0.17529	0.18104	0.17294258	0.50527715	0.54161868	5	22	22
760	NM_000067,NM_001293675	CA2	carbonic anhydrase 2	-1.249824891	3.520072366	5.458208	4.371416	18.168486	3.30216484	3.01642592	5.00938866	348	372	1507
574412	NR_029973	MIR452	microRNA 452	1.471633007	3.517603686	0.518045	0.707805	1.913844	0.07168335	0.10601836	0.24657076	2	4	9
677830	NR_002980	SNORA50A	small nucleolar RNA, H/ACA box	1.471633007	3.517603686	0.287591	0.566529	1.244174	0.07168335	0.10601836	0.24657076	2	4	9
102465503	NR_106895	MIR6836	microRNA 6836	-2.463050374	3.517603686	0.600275	0.237203	2.594736	0.07168335	0.02724389	0.24657076	2	1	9
102467003	NR_107005	MIR7851	microRNA 7851	1.471633007	3.517603686	0.194267	0.41241	1.025388	0.07168335	0.10601836	0.24657076	2	4	9
102466864	NR_106997	MIR6516	microRNA 6516	-1.313695475	3.517603686	0.46713	0.287816	2.155356	0.07168335	0.05398282	0.24657076	2	2	9
285521	NM_001297732,NM_001297733	COX18	COX18, cytochrome c oxidase as	-3.423648871	3.509269457	1.116731	0.324257	3.708363	2.57697339	1.29208824	4.20455055	195	76	842
10551	NM_006408	AGR2	anterior gradient 2, protein disulp	-2.2821103	3.501112429	30.994957	13.579753	102.006615	4.93893692	3.80748641	6.71285154	1165	682	5017
9732	NM_014705	DOCK4	dedicator of cytokinesis 4	1.183385792	3.499865405	0.037629	0.04413	0.124592	0.38477185	0.44594451	1.05442063	12	19	52
9423	NM_004822	NTN1	netrin 1	1.571798206	3.495893088	0.698282	1.097533	2.296101	2.33625658	2.8811152	3.92268941	159	334	684
3422	NM_001317955,NM_001317956	IDI1	isopentenyl-diphosphate delta isc	-1.127408789	3.484546863	14.322826	12.640568	47.341495	5.4065374	5.23784196	7.18312086	1626	1927	6969
55287	NM_001284406,NM_001284407	TMEM40	transmembrane protein 40	1.250624554	3.484187404	1.886753	2.349655	6.314692	2.23841445	2.49862948	3.80364308	146	244	626
92597	NM_001244766,NM_001244767	MOB1B	MOB kinase activator 1B	2.878450663	3.482487986	2.337092	6.672237	7.639178	4.02300634	5.48935666	5.75862518	599	2304	2566
3383	NM_000201	ICAM1	intercellular adhesion molecule 1	1.393699694	3.480058622	4.503529	6.286048	14.735223	3.92963473	4.38163336	5.65984958	559	1041	2393
8491	NM_001270425,NM_003618	MAP4K3	mitogen-activated protein kinase	1.085102366	3.471726338	3.967177	4.315895	12.99306	4.15055879	4.26202234	5.88735842	658	954	2810
57593	NM_001110514,NR_138537	EBF4	early B-cell factor 4	1.10836228	3.452809896	0.238468	0.269079	0.783867	0.7550755	0.81765451	1.75769337	27	40	115
400451	NM_207446	FAM174B	family with sequence similarity 17	1.774541276	3.440493558	0.585929	1.041836	1.896608	1.32354034	1.87534115	2.62698266	59	140	250
64114	NM_001321427,NM_001321428	TMBIM1	transmembrane BAX inhibitor mc	1.234110299	3.439690197	60.693409	75.229705	196.123979	7.14204496	7.44358266	8.91708144	5506	9079	23294
55790	NM_001130518,NM_018371,NR	CSGALNACT1	chondroitin sulfate N-acetylgalact	-1.408736109	3.433263318	1.529024	1.089986	4.952923	2.78853894	2.37683491	4.41245104	232	220	980
728463	NR_046268	TGFB2-AS1	TGFB2 antisense RNA 1 (head t	9.669857865	3.432323925	0.096993	1.390584	0.445599	0.10623215	0.81765451	0.34394329	3	40	13
102464831	NR_106728	MIR6080	microRNA 6080	-3.632026599	3.432323925	1.189149	0.232302	3.874113	0.10623215	0.02724389	0.34394329	3	1	13
648740	NR_024438	ACTG1P4	actin gamma 1 pseudogene 4	3.152058602	3.421531338	0.097182	0.333442	0.338223	0.26755841	0.72085408	0.76902392	8	34	34
388282	NM_001278081	LOC388282	uncharacterized LOC388282	3.336697327	3.421531338	0.150816	0.535971	0.525803	0.26755841	0.75384785	0.76902392	8	36	34
653583	NM_198850	PHLDB3	pleckstrin homology like domain f	-1.141081129	3.412705246	6.204677	5.442491	19.918781	4.00480921	3.82699692	5.71093841	591	692	2481
9787	NM_001146015,NM_014750	DLGAP5	DLG associated protein 5	1.542436983	3.4120267	15.439442	23.773909	49.361122	5.5412612	6.15556624	7.28988498	1789	3687	7508
1514	NM_001257971,NM_001257972	CTSL	cathepsin L	1.447547974	3.40882246	81.089929	117.465658	260.393675	7.01512412	7.54529217	8.77651705	5039	9746	21127
79646	NM_024594	PANK3	pantothenate kinase 3	1.872623799	3.404256498	3.286764	6.163714	10.525762	3.5668994	4.41424668	5.24585326	426	1066	1784
10501	NM_032108	SEMA6B	semaphorin 6B	1.509316618	3.403119885	1.165996	1.765043	3.732155	2.45494311	2.95745524	4.02350017	176	355	737
100526833	NR_037611,NR_037612	SEPT5-GP1BB	SEPT5-GP1BB readthrough	1.331049716	3.400132477	1.461835	1.954533	5.551711	2.93987166	3.30503223	4.56665693	262	466	1096
7779	NM_021194	SLC30A1	solute carrier family 30 member 1	1.345756562	3.397439696	5.772814	7.78908	18.453678	3.62762016	4.02581645	5.30750681	446	802	1864
6249	NM_001247997,NM_002956,NM	CLIP1	CAP-Gly domain containing linke	2.305035344	3.384749504	5.378134	12.415498	17.112961	4.96623093	6.1447231	6.69248626	1188	3659	4946
23057	NM_015039,NM_170706	NMNAT2	nicotinamide nucleotide adenylylt	2.854051327	3.380396155	0.256322	0.739232	0.820303	1.26358189	2.32288731	2.52166171	55	210	229
160851	NM_001204504,NM_001204505	DGKH	diacylglycerol kinase eta	4.001686685	3.380006526	0.690528	1.90985	1.513221	3.17773766	5.05386023	4.81823857	316	1690	1314
200407	NM_153836	CREG2	cellular repressor of E1A stimulat	1.122506006	3.375718821	0.67236	0.755436	2.141648	1.7191395	1.83736459	3.12850473	90	135	374
22998	NM_001112717,NM_001112718	LIMCH1	LIM and calponin homology dom	1.82910321	3.373323101	4.525883	8.129426	13.986388	4.78750079	5.6348274	6.50454979	1045	2554	4336
8754	NM_003816,NR_027638,NR_02	ADAM9	ADAM metallopeptidase domain	1.029018661	3.366987476	76.304569	78.575184	241.16256	8.25156967	8.29270568	9.9997059	11926	16397	49388
8744	NM_003811	TNFSF9	TNF superfamily member 9	-1.306187526	3.366186552	2.751862	2.114723	8.743061	2.45494311	2.14776967	4.00872756	176	180	729
8532	NM_001014447,NM_001014448	CPZ	carboxypeptidase Z	2.334852902	3.364574325	3.247776	8.484072	11.325757	3.17773766	4.30717831	4.81187039	316	986	1308
126282	NM_001167942,NM_152362	TNFAIP8L1	TNF alpha induced protein 8 like	-1.13019832	3.364503251	1.900824	1.683283	6.065691	3.02359429	2.86987209	4.64397663	280	331	1159
83696	NM_001160372,NM_001321646	TRAPPC9	trafficking protein particle comple	1.009864324	3.36363567	2.447592	2.476311	7.754047	4.12761734	4.14097459	5.81861581	647	873	2677
10276	NM_001047160,NM_005863,NR	NET1	neuroepithelial cell transforming	-1.079119422	3.361273842	35.44721	32.694276	112.372571	7.09282367	6.98380281	8.83440944	5320	6587	21994
120224	NM_001331210,NM_001331211	TMEM45B	transmembrane protein 45B	1.078237251	3.360418275	7.756546	8.106704	23.952804	3.86048198	3.96195135	5.5378219	531	765	2195
400221	NR_039985	FLJ22447	uncharacterized LOC400221	-1.328298723	3.343915689	0.114742	0.085972	0.354944	0.20517559	0.15624221	0.60191771	6	6	25
653145	NM_001040084,NM_001040084	ANXA8	annexin A8	1.709236563	3.341668554	2.49453	4.246758	7.837635	2.58923814	3.25980797	4.15174023	197	450	810
79137	NM_001321109,NM_001321110	RETREG2	reticulophagy regulator family me	1.672685303	3.332458087	4.940395	8.288452	15.509468	4.53037804	5.24728583	6.2227218	868	1940	3558
3589	NM_000641,NM_001267718	IL11	interleukin 11	-1.005311486	3.331002075	2.57428	2.560115	8.142564	2.7991369	2.79259185	4.38268287	234	311	959
26959	NM_001244262,NM_012257	HBP1	HMG-box transcription factor 1	-1.062037854	3.323092351	8.325783	7.746181	25.1256	4.53037804	4.44740017	6.21871574	868	1092	3548
388	NM_004040	RHOB	ras homolog family member B	2.476249122	3.320800805	9.880405	24.514517	30.8577	4.5757712	5.84750424	6.26451583	897	2968	3664
10610	NM_006456	ST6GALNAC2	ST6 N-acetylgalactosaminide alp	-2.914605154	3.31890306	1.459263	0.502568	4.576509	2.00201503	1.02107213	3.45705395	118	54	482
2049	NM_004443	EPHB3	EPH receptor B3	1.706653244	3.317805504	2.890626	4.946763	9.027523	3.6887407	4.41295621	5.33885009	467	1065	1906
2528	NM_000150,NM_001040701	FUT6	fucosyltransferase 6	-2.970056527	3.309577926	0.310664	0.102803	1.023059	0.97665845	0.40498891	2.07380058	38	17	155
64220	NM_001142617,NM_001142618	STRA6	stimulated by retinoic acid 6	1.750902563	3.308475458	5.135942	8.844977	15.396891	3.85794988	4.62285328	5.51317412	530	1240	2157
255394	NM_001286262,NM_152772	TCP11L2	t-complex 11 like 2	1.062251077	3.308234463	1.855031	2.025161	5.897779	2.30685184	2.37683491	3.81430154	155	220	631
283887	NR_135196,NR_135197	LINC02175	long intergenic non-protein codin	1.269454991	3.307637576	0.133826	0.170258	0.410008	0.3273552	0.40498891	0.88680889	10	17	41
5950	NM_001323517,NM_001323518	RBP4	retinol binding protein 4	1.34350598	3.307637576	0.28077	0.37875	0.802028	0.3273552	0.42561204	0.88680889	10	18	41
102724895	NR_146458	LIVAR	liver cell viability associated lncR	-1.798059653	3.302867513	0.420158	0.233354	1.302974	0.7550755	0.46599441	1.71282633	27	20	110
8654	NM_001083,NM_033430,NM_03	PDE5A	phosphodiesterase 5A	2.438873408	3.302422947	0.343078	0.839988	1.066269	1.74129091	2.74825434	3.12850473	92	300	374

30846	NM_014601	EHD2	EH domain containing 2	1.113512055	3.299399907	17.936443	19.998329	55.629322	5.99113594	6.14394546	7.69749126	2458	3657	9975
7113	NM_001135099,NM_005656	TMPRSS2	transmembrane protease, serine	-1.496238719	3.298402495	2.933378	1.962559	9.133634	3.36057263	2.84711955	4.98128857	364	325	1477
117583	NM_001302769,NM_057177,NM	PARD3B	par-3 family cell polarity regulator	1.619398475	3.297819986	0.094988	0.15433	0.297449	0.81895953	1.16293478	1.81825156	30	65	122
9871	NM_001318066,NM_014822	SEC24D	SEC24 homolog D, COPII coat c	1.05338376	3.294994989	4.449455	4.694343	13.80183	4.22907737	4.30021519	5.89490974	697	981	2825
643008	NM_001162995,NR_028439	SMIM5	small integral membrane protein	-6.366530392	3.292537211	3.599725	0.48869	8.817464	3.09414281	1.12560759	4.6910014	296	62	1199
728113	NM_001098845,NM_001278923	ANXA8L1	annexin A8 like 1	1.672618276	3.29080011	2.610334	4.254689	7.897468	2.63136183	3.27693362	4.17838702	204	456	826
112476	NM_001256442,NM_001256443	PRRT2	proline rich transmembrane prote	1.868504373	3.29024086	0.290756	0.571927	0.947838	0.93882015	1.44132992	2.00847214	36	90	146
25804	NM_001252129,NM_012321	LSM4	LSM4 homolog, U6 small nuclear	1.002609305	3.289697138	19.856321	19.866601	61.312166	5.17694504	5.18060106	6.86695259	1381	1850	5588
81849	NM_001320273,NM_001320274	ST6GALNAC5	ST6 N-acetylgalactosaminide alp	-1.772294266	3.286994484	0.072645	0.041783	0.225504	0.49317359	0.29720487	1.23026757	16	12	65
9536	NM_004878	PTGES	prostaglandin E synthase	1.572644213	3.278485909	57.82703	91.034752	178.159317	6.66691103	7.31493823	8.37006769	3950	8300	15928
7089	NM_001144761,NM_001144762	TLE2	transducin like enhancer of split 2	-1.483277844	3.274839587	3.13266	2.103291	9.219697	3.16140171	2.66830768	4.75657497	312	281	1257
79956	NM_024896	ERMP1	endoplasmic reticulum metallope	1.200642668	3.2725766	9.514941	11.436753	29.269426	5.65793614	5.9169731	7.34844041	1943	3117	7821
1820	NM_005224	ARID3A	AT-rich interaction domain 3A	1.004347742	3.271542574	1.43652	1.448151	4.435686	2.30685184	2.31185141	3.79935756	155	208	624
155185	NM_001284355,NM_001321766	AMZ1	archaelysin family metallopeptida	1.645954684	3.271199496	0.542837	0.93261	2.138602	1.97422731	2.541776	3.40441396	115	253	463
51084	NM_015974	CRYL1	crystallin lambda 1	1.177183933	3.268932025	1.412113	1.680091	4.384665	1.62696745	1.79043244	2.96976228	82	129	330
23186	NM_015156	RCOR1	REST corepressor 1	1.320180067	3.268801154	10.83597	14.326046	33.297901	5.94833435	6.343407	7.64083454	2385	4207	9589
3399	NM_002167	ID3	inhibitor of DNA binding 3, HLH p	1.144210548	3.268636321	0.716545	0.814991	2.200739	0.89996265	0.99371033	1.9400446	34	52	137
64746	NM_022735	ACBD3	acyl-CoA binding domain contain	-1.02011147	3.263048905	10.280832	10.088549	31.521965	5.19711926	5.16918099	6.87588368	1401	1835	5623
58480	NM_021205,NR_037962	RHOU	ras homolog family member U	1.099097395	3.260946959	0.382372	0.420556	1.170854	1.39514558	1.48128072	2.66044767	64	94	257
728975	NR_145677	LOC728975	uncharacterized LOC728975	1.139008156	3.250972596	2.951456	3.372007	9.035634	2.63728034	2.79655578	4.16845206	205	312	820
3887	NM_002281	KRT81	keratin 81	-10.02378369	3.250118796	1.652116	0.164151	5.064554	2.02927762	0.38406669	3.4624849	121	16	484
26525	NM_012275,NM_173170	IL36RN	interleukin 36 receptor antagonist	2.070384873	3.247358357	0.500676	1.054893	1.545694	1.21691895	1.90501868	2.40804223	52	144	208
9294	NM_004230	S1PR2	sphingosine-1-phosphate recepto	1.412690926	3.243686872	0.910945	1.287807	2.781211	2.0648444	2.45916281	3.50257812	125	236	499
157567	NM_001270377,NM_001270378	ANKRD46	ankyrin repeat domain 46	1.950241196	3.239688977	1.815735	2.912366	4.187223	2.26923253	3.07960182	3.74245532	150	391	598
80255	NM_001330314,NM_001330315	SLC35F5	solute carrier family 35 member F	2.945185555	3.23849276	3.775939	9.421399	9.36173	4.91477587	6.44128305	6.57675114	1145	4506	4561
122481	NM_001350888,NM_001350890	AK7	adenylate kinase 7	-1.112541313	3.235495389	0.103884	0.097286	0.336672	0.46682716	0.42561204	1.16514558	15	18	60
5337	NM_001130081,NM_002662	PLD1	phospholipase D1	-1.205970474	3.235442427	1.325593	1.11004	4.116468	3.13654562	2.89966118	4.71281235	306	339	1218
441168	NM_001010919,NM_001276460	FAM26F	family with sequence similarity 26	-1.199655349	3.22449362	0.190588	0.159905	0.618645	0.29776659	0.25173066	0.80366934	9	10	36
493	NM_001001396,NM_001684	ATP2B4	ATPase plasma membrane Ca2+	1.638505037	3.222900346	5.311891	8.891429	16.421552	5.55929747	6.25973808	7.22645993	1812	3967	7183
338596	NM_001004470,NM_001345961	ST8SIA6	ST8 alpha-N-acetyl-neuraminide	-1.763306246	3.221077741	0.030462	0.017139	0.09154	0.26755841	0.15624221	0.73352601	8	6	32
57221	NM_020340	ARFGEF3	ARFGEF family member 3	-3.576712034	3.218898096	0.484527	0.135117	1.467244	3.00540462	1.5673195	4.56286962	276	103	1093
83903	NM_031965	GSG2	germ cell associated 2, haspin	1.798299828	3.218540861	0.965405	1.751816	2.944701	1.86749926	2.52753672	3.25281446	104	250	412
100132356	NR_034127	LOC100132356	uncharacterized LOC100132356	1.594696474	3.216701281	0.088082	0.158087	0.309217	0.23670415	0.36283659	0.65979727	7	15	28
254559	NR_015411,NR_133001,NR_13	MIR9-3HG	MIR9-3 host gene	2.331861258	3.216701281	0.038502	0.089848	0.117094	0.23670415	0.50527715	0.65979727	7	22	28
143098	NM_001318170,NM_173496,NR	MPP7	membrane palmitoylated protein	-1.139784828	3.212152794	1.641008	1.451799	5.083061	3.23740108	3.06980877	4.81187039	331	388	1308
7477	NM_058238	WNT7B	Wnt family member 7B	-1.013824938	3.211899878	10.833259	10.694539	32.696434	5.40132993	5.38199194	7.06112346	1620	2135	6400
284611	NM_001010883	FAM102B	family with sequence similarity 10	-1.144268715	3.207614224	1.720915	1.506774	5.195896	3.31698997	3.14324318	4.89555604	352	411	1389
168507	NM_138295	PKD1L1	polycystin 1 like 1, transient rece	1.633007534	3.202811781	0.01151	0.021559	0.041771	0.17294258	0.27464693	0.49997203	5	11	20
5291	NM_001256045,NM_006219	PIK3CB	phosphatidylinositol-4,5-bisphosp	3.897994828	3.198270907	1.397324	5.047896	4.008943	3.0724647	4.9020831	4.62714903	291	1516	1145
808	NM_001329921,NM_001329922	CALM3	calmodulin 3	-1.049159824	3.189956522	44.778913	42.848378	134.376818	6.69909733	6.63054343	8.3630976	4040	5145	15851
2180	NM_001286708,NM_001286710	ACSL1	acyl-CoA synthetase long-chain f	-1.485532098	3.189554979	7.502814	4.863118	22.988669	4.90008261	4.35231081	6.53997876	1133	1019	4445
2568	NM_001291985,NM_014211	GABRP	gamma-aminobutyric acid type A	-6.351339207	3.179156497	4.707209	0.739982	14.049387	3.95828615	1.71704117	5.56205565	571	120	2233
5621	NM_000311,NM_001080121,NM	PRNP	prion protein	2.594687545	3.177633473	87.968069	228.358923	262.616216	7.90640356	9.278274	9.5702413	9380	32519	36660
3991	NM_005357	LIPE	lipase E, hormone sensitive type	-1.397936776	3.174061383	1.232453	0.883494	3.685002	2.48150547	2.09724475	3.95964434	180	172	703
412	NM_000351,NM_001320750,NM	STS	steroid sulfatase	1.107766476	3.163318054	2.295041	2.574111	6.85267	3.97707578	4.11581404	5.57464861	579	857	2253
23038	NM_001276252,NM_015023	WDTC1	WD and tetratricopeptide repeats	1.238322252	3.161922137	2.698935	3.349362	8.030031	3.7744826	4.06249189	5.36155532	498	824	1937
100130987	NR_024469	LOC100130987	uncharacterized LOC100130987	1.003573864	3.154322306	1.330767	1.35145	4.000796	1.08456011	1.08728891	2.18310266	44	59	171
80328	NM_025217	ULBP2	UL16 binding protein 2	1.251467152	3.149784776	3.272918	4.12068	9.725388	2.46162969	2.73178769	3.92662428	177	296	686
10090	NM_005715	UST	uronyl 2-sulfotransferase	6.15682024	3.146830221	0.05952	0.369286	0.174692	0.3273552	1.36863803	0.85412573	10	83	39
51364	NM_001308379,NM_015896	ZMYND10	zinc finger MYND-type containing	1.121353014	3.146830221	0.141892	0.154513	0.428473	0.3273552	0.36283659	0.85412573	10	15	39
81793	NM_001017388,NM_001195106	TLR10	toll like receptor 10	-1.334579138	3.139556157	0.203128	0.15205	0.599762	0.83964067	0.669904	1.80120704	31	31	120
154386	NR_131168	LINC01600	long intergenic non-protein codin	3.247686972	3.132656612	0.02139	0.072446	0.065616	0.07168335	0.2284445	0.22116494	2	9	8
26796	NR_002741	SNORD53	small nucleolar RNA, C/D box 53	1.471633007	3.132656612	0.365288	0.98033	1.996797	0.07168335	0.10601836	0.22116494	2	4	8
100422832	NR_036159	MIR3191	microRNA 3191	1.116422214	3.132656612	0.545311	0.523493	1.856648	0.07168335	0.08023518	0.22116494	2	3	8
102466659	NR_106770	MIR6515	microRNA 6515	1.116422214	3.132656612	0.827054	0.691181	2.652603	0.07168335	0.08023518	0.22116494	2	3	8
1545	NM_000104	CYP1B1	cytochrome P450 family 1 subfar	1.325185254	3.127157702	1.527751	2.025909	4.48949	3.1155	3.48037129	4.64278115	301	533	1158
8916	NM_001271602,NM_001318505	HERC3	HECT and RLD domain containir	-1.153506951	3.120001193	1.620822	1.50563	4.812071	3.09843961	2.91797176	4.62109129	297	344	1140
3077	NM_000410,NM_001300749,NM	HFE	hemochromatosis	1.826374558	3.114381131	1.939329	3.465408	5.702192	2.35073722	3.08609378	3.78425716	161	393	617
7020	NM_001032280,NM_001042425	TFAP2A	transcription factor AP-2 alpha	-1.889250509	3.113942408	10.105604	5.38155	30.017178	5.16162707	4.27912189	6.77282387	1366	966	5232
4854	NM_000435	NOTCH3	notch 3	2.147876163	3.112987338	1.745622	3.754715	5.109853	3.88058045	4.93031934	5.45101358	539	1547	2064
374	NM_001657,NM_001657_dup1	AREG	amphiregulin	1.156790135	3.110059195	114.595093	132.714172	334.944916	7.16373514	7.3725016	8.79385401	5590	8640	21383
1384	NM_000755,NM_001257363,NM	CRAT	carnitine O-acetyltransferase	1.270089322	3.109326258	8.140445	10.297186	24.108918	4.54619271	4.87796738	6.14040759	878	1490	3358
4616	NM_015675	GADD45B	growth arrest and DNA damage i	1.027236313	3.108481174	33.494152	34.449375	97.863976	5.51899523	5.55693117	7.1337666	1761	2417	6733
6374	NM_002994	CXCL5	C-X-C motif chemokine ligand 5	-5.011331615	3.103325241	19.366932	3.862476	56.481762	5.58248919	3.37297385	7.19581156	1842	491	7031
6745	NM_001292008,NM_003144,NR	SSR1	signal sequence receptor subunit	2.556270067	3.101233684	3.367617	8.609312	9.855219	5.03845181	6.36559004	6.64132658	1251	4273	4772
6398	NM_003004	SECTM1	secreted and transmembrane 1	-2.217189356	3.100107143	5.593388	2.517623	16.298429	3.74737673	2.72348335	5.30524173	488	294	1861
388403	NM_001005404	YPEL2	yippee like 2	-1.066067586	3.093635446	1.517252	1.425223	4.416821	3.12816418	3.04669644	4.64158467	304	381	1157
79875	NM_001286429,NM_024817	THSD4	thrombospondin type 1 domain c	-1.285326154	3.092578727	1.95426	1.535803	5.686445	4.20337642	3.86335533	5.77835625	684	711	2602
9851	NM_001351225,NM_014804,NR	KIAA0753	KIAA0753	4.003617419	3.088709783	1.468355	5.818423	4.376143	2.9541672	4.80900168	4.44984345	265	1418	1007
6792	NM_001037343,NM_001323289	CDKL5	cyclin dependent kinase like 5	3.243576786	3.086849266	0.067236	0.221915	0.198402	0.73313634	1.65726059	1.60891398	26	113	99
668	NM_023067	FOXL2	forkhead box L2	3.47292034	3.086849266	0.233094	0.812163	0.677838	0.73313634	1.72538257	1.60891398	26	121	99
100507291	NR_121608,NR_121609	LOC100507291	uncharacterized LOC100507291	3.446215928	3.083144975	0.396663	1.487778	1.262417	0.35634914	0.97983233	0.90287695	11	51	42
4148	NM_002381	MATN3	matrilin 3	1.495102784	3.081813634	0.302945	0.449311	0.881334	0.81895953	1.10017522	1.74883089	30	60	114
9982	NM_005130	FGFBP1	fibroblast growth factor binding pr	-2.560662776	3.078495966	34.558498	13.502392	99.972633	5.54520135	4.2359869	7.14647525	1794	936	6793
163175	NM_139284	LGI4	leucine rich repeat LGI family me	1.940895074	3.077945418	0.173587	0.349374	0.525289	0.59399636	0.99371033	1.36396013	20	52	76
204801	NM_001297743,NM_145007	NLRP11	NLR family pyrin domain containi	3.571567149	3.077869828	0.038716	0.138509	0.111905	0.20517559	0.63490936	0.56199967	6	29	23
390561	NR_072991	HERC2P10	hect domain and RLD 2 pseudog	2.100928307	3.077869828	0.216883	0.502528	0.697005	0.20517559	0.40498891	0.56199967	6	17	23
26579	NM_001293291,NM_001293294	MYEOV	myeloma overexpressed	1.56581016	3.07702413	30.623435	48.223416	87.512097	6.17630487	6.81600722	7.7843504	2800	5858	10597
3320	NM_001017963,NM_005348	HSP90AA1	heat shock protein 90 alpha famil	1.918233264	3.073957902	239.185898	460.465626	696.732981	9.63677076	10.57568353	11.25564844	31215	80004	118017
4327	NM_001272101,NM_002429,NR	MMP19	matrix metallopeptidase 19	3.431719619	3.070136261	0.284427	1.007707	0.826147	0.95786335	2.08433212	1.96321628	37	170	140
27010	NM_001042482,NM_001350879	TPK1	thiamin pyrophosphokinase 1	4.00934157	3.066426543	0.092935	0.373857	0.269613	0.3273552	1.02107213	0.83750224	10	54	38
56413	NM_001164692,NM_019839	LTB4R2	leukotriene B4 receptor 2	2.251638298	3.065413147	1.655529	3.746852	4.761464	1.79522562	2.71513092	3.10091148	97	292	366
147700	NM_177417	KLC3	kinesin light chain 3	-1.869839689	3.063341752	3.464083	1.853025	9.992476	2.82010209	2.08433212	4.29103243	238	170	897
25932	NM_013943	CLIC4	chloride intracellular channel 4	-1.603884768	3.06253152	5.899335	3.681787	16.978405	4.72634668	4.07723084	6.30399916	1000	833	3767
26234	NM_001193534,NM_001193535	FBXL5	F-box and leucine rich repeat pro	1.854550882	3.061062971	8.889145	16.564186	25.681778	4.98607799	5.85607627	6.56921958	1205	2986	4537
54894	NM_001305544,NM_001305545	RNF43	ring finger protein 43	2.188988148	3.060995964	0.372864	0.823866	1.30558	1.62696745	2.47903109	2.88736461	82	240	309
26575	NM_012419	RGS17	regulator of G protein signaling 1	1.427996637	3.055837368	0.97478	1.392551	2.815807	1.26358189	1.58576189	2.40240201	55	105	207
7105	NM_001278740,NM_001278741	TSPAN6	tetraspanin 6	1.334799579	3.053631282	9.415059	12.191857	26.183197	5.17694504	5.58355786	6.76047615	1381	2463	5187
256302	NM_152914	NATD1	N-acetyltransferase domain conta	-1.210547292	3.053303996	0.471004	0.391558	1.362302	1.6852613	1.50084877	2.95827541	87	96	327
1028	NM_000076,NM_001122630,NM	CDKN1C	cyclin dependent kinase inhibitor	-2.237367995	3.050490388	1.635899	0.711645	4.696911	1.96484452	1.19932051	3.30211189	114	68	428
5660	NM_001042465,NM_001042466	PSAP	prosaposin	1.171610995	3.047186108	88.229467	103.491447	252.742286	7.96125258	8.18890035	9.56484691	9745	15255	36523
6309	NM_001024956,NM_006918	SC5D	sterol-C5-desaturase	-1.15017435	3.04695587	2.719275	2.374833	7.81431	4.29233333	4.10146599	5.84951557	730	848	2736
6389	NM_001294332,NM_001330758	SDHA	succinate dehydrogenase comple	1.344716217	3.044769097	26.302017	35.356776	75.316025	6.11718378	6.53916241	7.70953136	2686	4826	10059
3920	NM_001122606,NM_002294,NM	LAMP2	lysosomal associated membrane	1.699709165	3.043110726	15.876941	26.562108	46.960864	6.24235022	6.9997934	7.83508261	2933	6661	10978
59084	NM_001290072,NM_001290073	ENPP5	ectonucleotide pyrophosphatase/	1.714765534	3.040156881	2.397995	4.11917	6.900538	3.30959645	4.02581645	4.81293371	350	802	1309
57708	NM_001077700,NM_001077701	MIER1	MIER1 transcriptional regulator	1.057561489	3.031524953	4.230411	4.559219	10.122175	4.21529532	4.29181484	5.76248297	690	975	2573
100130449	NR_024014	PP14571	uncharacterized LOC100130449	-3.436200863	3.026108954	2.427646	0.707745	6.93428	2.53320657	1.25799553	3.95387191	188	73	700
693228	NR_030373	MIR643	microRNA 643	1.448836667	3.025161034	0.159822	0.322083	0.835827	0.03628685	0.05398282	0.1148164	1	2	4
102466191	NR_106794	MIR6736	microRNA 6736	2.124920087	3.025161034	0.259023	0.687488	1.190776	0.03628685	0.08023518	0.1148164	1	3	4

4330	NM_002430	MN1	MN1 proto-oncogene, transcriptic	5.367723294	3.020624144	0.02571	0.150026	0.078496	0.26755841	1.07428646	0.69713258	8	58	30
102724593	NR_134489	LOC102724593	uncharacterized LOC102724593	1.054347812	3.020167785	0.856826	0.881607	2.421879	0.64188297	0.669904	1.43197761	22	31	82
3200	NM_030661,NM_153631	HOXA3	homeobox A3	-1.445292659	3.017001445	1.564876	1.055239	4.3228	2.59533164	2.16626946	4.01982118	198	183	735
4488	NM_002449	MSX2	msh homeobox 2	1.665903581	3.013453924	0.407655	0.687351	1.175501	0.91952222	1.31437719	1.88448459	35	78	130
643314	NM_015038	KIAA0754	KIAA0754	-1.1290886	3.010882157	2.022588	1.79366	5.7255	3.88307318	3.72044785	5.4066256	540	639	2000
8402	NM_001165417,NM_001165418	SLC25A11	solute carrier family 25 member '	2.887326217	3.008021201	3.472471	9.861111	9.607163	2.7991369	4.18699961	4.24292715	234	903	866
1509	NM_001909	CTSD	cathepsin D	2.422851834	3.007147278	247.393417	600.08075	699.128418	9.00108002	10.27613624	10.58760037	20077	64994	74257
337875	NR_027337	HIST2H2BA	histone cluster 2 H2B family men	1.758634431	3.006454952	1.691603	3.033222	4.866603	0.89996265	1.33632702	1.85174813	34	80	126
55796	NM_001170701,NM_001170702	MBNL3	muscleblind like splicing regulato	1.288187689	3.006218032	0.694551	0.894731	1.962189	3.13236099	3.46052158	4.60644807	305	525	1128
130733	NM_001167959,NM_152390	TMEM178A	transmembrane protein 178A	-1.369704185	3.005477893	1.234925	0.901352	3.503609	1.602974	1.31437719	2.83389297	80	78	296
6272	NM_001205228,NM_002959	SORT1	sortilin 1	-2.155838355	2.999763393	4.581875	2.120439	12.897197	5.01250255	3.95487459	6.56733053	1228	761	4531
7100	NM_003268	TLR5	toll like receptor 5	1.249760429	2.998984633	0.573164	0.720221	1.620834	1.76310734	1.99745948	3.03318152	94	157	347
27294	NM_014475	DHDH	dihydrodiol dehydrogenase	-1.971048762	2.993732559	3.751154	1.897487	10.547841	2.30685184	1.58576189	3.68088855	155	105	571
25987	NM_001258210,NM_001318477	TSKU	tsukushi, small leucine rich prote	2.102010638	2.993539608	9.879325	20.865146	27.654819	4.77413325	5.81806988	6.32054191	1035	2907	3811
85320	NM_032583,NM_033151,NM_14	ABCC11	ATP binding cassette subfamily (	6.2219852	2.993079417	0.021417	0.141101	0.060991	0.13997292	0.72085408	0.39027104	4	34	15
6698	NM_001199828,NM_005987	SPRR1A	small proline rich protein 1A	-1.019598581	2.992175933	1.11297	1.131563	3.259203	0.79797761	0.7861039	1.6852207	29	38	107
158046	NM_001161625,NM_145283	NXNL2	nucleoredoxin-like 2	1.575315469	2.990125073	2.07123	3.359868	6.030529	1.42282133	1.86782539	2.59272293	66	139	243
7476	NM_004625	WNT7A	Wnt family member 7A	-1.439834579	2.986700133	6.131156	4.264112	17.223156	3.49049124	3.01982084	4.98128857	402	373	1477
1259	NM_000087,NM_001142564	CNGA1	cyclic nucleotide gated channel a	-1.378329468	2.98483189	1.511818	1.065831	4.15467	2.3579234	1.99745948	3.73574288	162	157	595
1356	NM_000096,NR_046371	CP	ceruloplasmin	19.8477822	2.984221902	0.094179	1.874654	0.279875	0.49317359	3.19220918	1.15176086	16	427	59
8801	NM_001177599,NM_003848	SUCLG2	succinate-CoA ligase GDP-formi	1.467846266	2.980480269	7.754667	11.398798	21.720848	4.23103553	4.76010752	5.75475705	698	1369	2559
342908	NM_001033719	ZNF404	zinc finger protein 404	-1.029652827	2.979865428	2.383654	2.313101	6.676507	2.4003046	2.36620557	3.782087	168	218	616
10983	NM_001348132,NM_001348133	CCNI	cyclin I	1.61419395	2.973762923	14.702809	23.830439	41.195013	4.79678491	5.46776786	6.33430752	1052	2269	3848
100506658	NM_001205254,NM_001205255	OCLN	occludin	-2.670708122	2.973539391	8.586754	3.213561	23.731471	5.73309128	4.36036716	7.28720953	2049	1025	7494
80790	NM_030629,NM_198390	CMIP	c-Maf inducing protein	1.713390642	2.972697209	5.327417	9.381145	14.893016	4.5618359	5.31310082	6.09261172	888	2033	3247
147808	NM_203374	ZNF784	zinc finger protein 784	-1.196318003	2.972568334	1.569837	1.311954	4.389078	2.02024721	1.82964782	3.33505242	120	134	439
80232	NM_001115113,NM_025160	WDR26	WD repeat domain 26	2.707750591	2.97079549	14.428572	39.166536	40.302679	6.60294934	8.03067876	8.16394724	3777	13665	13801
106633803	NR_132755	SNORD134	small nucleolar RNA, C/D box 13	-1.645078408	2.970666021	2.926525	1.800291	8.440971	0.61813834	0.40498891	1.37552141	21	17	77
1316	NM_001160124,NM_001160125	KLF6	Kruppel like factor 6	4.525942714	2.966877218	4.382193	19.848467	12.188301	4.37080362	6.49380183	5.89289991	773	4675	2821
10239	NM_005829,NR_023361,NR_03	AP3S2	adaptor related protein complex :	1.760371562	2.962151339	4.114494	7.25311	11.473383	4.63612739	5.42679658	6.16393291	937	2204	3414
288	NM_001149,NM_001204403,NM	ANK3	ankyrin 3	1.306386309	2.956814504	0.974343	1.012878	2.73364	3.05488459	3.39927923	4.49955999	287	501	1044
100129858	NR_033939	SCOC-AS1	SCOC antisense RNA 1	2.395034387	2.956811111	0.103763	0.273649	0.324083	0.29776659	0.63490936	0.75138414	9	29	33
56138	NM_018902,NM_031861	PCDHA11	protocadherin alpha 11	1.444705715	2.950636756	0.353395	0.51875	0.931633	1.6852613	2.07130288	2.91535666	87	168	316
387755	NM_001031853,NM_001042536	INSC	inscuteable homolog (Drosophila)	-1.723018298	2.946628818	0.185366	0.106361	0.5335	0.64188297	0.40498891	1.40965954	22	17	80
593	NM_000709,NM_001164783	BCKDHA	branched chain keto acid dehydr	1.147392753	2.943465375	8.23218	9.460136	22.79795	3.92963473	4.11581404	5.42343154	559	857	2024
883	NM_001122671,NM_001122672	KYAT1	kynurenine aminotransferase 1	-1.601794483	2.942052231	11.750179	7.412437	32.734126	4.56804599	3.92440495	6.08426934	892	744	3228
793	NM_004929	CALB1	calbindin 1	1.896385267	2.936808979	0.110004	0.21467	0.310721	0.35634914	0.61708849	0.87055986	11	28	40
56300	NM_001278568,NM_019618	IL36G	interleukin 36, gamma	2.435456801	2.936808979	0.240471	0.602161	0.638507	0.35634914	0.75384785	0.87055986	11	36	40
8406	NM_001170750,NM_001170751	SRPX	sushi repeat containing protein, X	-1.002086032	2.933932372	0.53397	0.532152	1.476998	0.99521185	0.99371033	1.97085819	39	52	141
256987	NM_001174071,NM_001174072	SERINC5	serine incorporator 5	1.939085449	2.932747559	6.854788	13.253669	18.527323	5.43144524	6.37058443	6.96156042	1655	4288	5970
79917	NM_001099681,NM_001099682	MAGIX	MAGI family member, X-linked	-1.089657196	2.930492643	0.301319	0.282582	0.857887	0.83964067	0.7861039	1.73094093	31	38	112
57146	NM_001301769,NM_001301771	TMEM159	transmembrane protein 159	1.232754105	2.920647951	6.174747	7.704329	17.095955	3.76910202	4.05092325	5.24427765	496	817	1782
1490	NM_001901	CTGF	connective tissue growth factor	2.681531601	2.918195447	1.71333	4.617554	4.715393	2.29940604	3.52644784	3.63833419	154	552	553
286343	NM_203403	LURAP1L	leucine rich adaptor protein 1 like	-1.437245396	2.917319829	2.341458	1.626626	6.421825	2.83562844	2.3978615	4.24134834	241	224	865
2149	NM_001311313,NM_001992	F2R	coagulation factor II thrombin rec	7.280688465	2.915826362	1.129731	8.238808	3.095274	2.37926962	4.98257876	3.72899907	165	1606	592
148362	NM_001288579,NM_001288580	BROX	BRO1 domain and CAAX motif c	2.353571827	2.915478412	12.81186	22.457263	26.760455	5.11254966	6.32328377	6.62870105	1319	4148	4730
145483	NM_152445	FAM161B	family with sequence similarity 16	1.352816309	2.915131911	0.527051	0.715513	1.450981	1.51570785	1.81408935	2.68388473	73	132	262
6509	NM_001193493,NM_001348406	SLC1A4	solute carrier family 1 member 4	-1.113266596	2.914137669	0.4545	0.408104	1.242725	1.602974	1.50084877	2.79567786	80	96	287
440461	NR_027283	LOC440461	Rho GTPase activating protein 2	-1.320831011	2.909816254	0.031846	0.023861	0.122982	0.10623215	0.08023518	0.29607837	3	3	11
5629	NM_001270616,NM_002763	PROX1	prospero homeobox 1	3.165952837	2.909816254	0.009233	0.027696	0.025704	0.10623215	0.31941552	0.29607837	3	13	11
1366	NM_001185022,NM_001185023	CLDN7	claudin 7	1.040396431	2.906533288	61.647561	64.207472	169.524403	6.91118358	6.96785271	8.44262141	4686	6514	16752
56108	NM_018920,NM_032087	PCDHGA7	protocadherin gamma subfamily	-1.658385536	2.905619188	0.090775	0.054344	0.146166	0.3273552	0.20477631	0.80366934	10	8	36
534	NM_001204078,NM_130463,NM	ATP6V1G2	ATPase H+ transporting V1 subu	2.750474764	2.905619188	0.162879	0.452318	0.444131	0.3273552	0.77006602	0.80366934	10	37	36
7922	NM_001077516,NM_001288777	SLC39A7	solute carrier family 39 member 7	1.446304422	2.904930382	26.556659	38.124092	71.8773	5.94773912	6.47289933	7.47087749	2384	4607	8518
51454	NM_001252668,NM_001252669	GULP1	GULP, engulfment adaptor PTB d	1.356107746	2.902498816	4.723226	6.481545	13.00168	4.09788139	4.51511838	5.5790303	633	1147	2260
8324	NM_003507	FZD7	frizzled class receptor 7	-1.107256403	2.897334695	7.718212	6.979812	21.030355	4.90499701	4.76314913	6.40793246	1137	1372	4052
51533	NM_001278221,NM_001321126	PHF7	PHD finger protein 7	1.244070298	2.896992697	0.904252	1.158949	2.591758	1.63881618	1.86027027	2.83389297	83	138	296
5819	NM_001042724,NM_002856	NECTIN2	nectin cell adhesion molecule 2	-1.087367322	2.893940563	72.256602	68.371764	202.357841	7.49214496	7.37200361	9.01994138	7029	8637	25019
1318	NM_001860	SLC31A2	solute carrier family 31 member 2	1.913203939	2.893938921	4.151077	7.95764	11.307837	3.03260384	3.88212953	4.44573627	282	721	1004
8139	NM_022041	GAN	gigaxonin	1.312229879	2.891275079	0.789489	1.04196	2.156862	2.17473011	2.48886356	3.48133381	138	242	491
54629	NM_001040450,NM_001040453	MINDY2	MINDY lysine 48 deubiquitinase 2	-1.252330311	2.886053308	4.204579	3.363042	11.411857	5.28833296	4.97297796	6.79317142	1495	1595	5307
158800	NM_139282	RHOXF1	Rhox homeobox family member '	1.486457258	2.884723647	0.145336	0.232479	0.454226	0.17294258	0.25173066	0.45708734	5	10	18
60680	NM_001172673,NM_021938,NR	CELF5	CUGBP Elav-like family member	1.926108085	2.884723647	0.057466	0.050777	0.069723	0.17294258	0.31941552	0.45708734	5	13	18
60412	NM_001037126,NM_021807	EXOC4	exocyst complex component 4	-1.123795344	2.884473115	6.548218	5.751337	17.397569	4.74153657	4.5798	6.23427685	1011	1202	3587
7980	NM_001271003,NM_001271004	TFPI2	tissue factor pathway inhibitor 2	-1.516098425	2.884177533	11.378731	7.501131	31.024325	4.80863452	4.23452662	6.30286431	1061	935	3764
661	NM_001722	POLR3D	RNA polymerase III subunit D	1.338164022	2.882697787	2.29346	3.083928	6.239812	2.41415945	2.76453517	3.75357377	170	304	603
2861	NM_005302	GPR37	G protein-coupled receptor 37	-1.00207938	2.879143631	0.123367	0.121125	0.33501	0.54446555	0.54351856	1.21747621	18	24	64
24139	NM_001193268,NM_001193269	EML2	echinoderm microtubule associat	-1.297443105	2.877275779	23.153096	16.941401	60.889758	5.58555344	5.21876673	7.09057724	1846	1901	6533
9829	NM_001256864,NM_001256865	DNAJC6	DnaJ heat shock protein family (H	1.879927595	2.875797828	0.323691	0.609751	0.877037	1.5285023	2.18453503	2.68388473	74	186	262
1595	NM_000786,NM_001146152	CYP51A1	cytochrome P450 family 51 subfa	-1.460548299	2.87548597	14.0636	9.834417	38.301522	5.48985314	4.95801353	6.99262665	1725	1578	6101
59285	NM_031897,NM_145814,NM_14	CACNG6	calcium voltage-gated channel au	-2.690133954	2.873845685	17.525625	6.441608	47.76579	4.95205502	3.60087125	6.44441182	1176	584	4157
5602	NM_001318067,NM_001318068	MAPK10	mitogen-activated protein kinase	4.122118592	2.873743648	0.025553	0.107455	0.067921	0.23670415	0.80196545	0.60191771	7	39	25
8816	NM_001284206,NM_001284207	DCAF5	DDB1 and CUL4 associated fact	3.980784949	2.87355515	6.444226	24.867962	17.083635	5.20311692	7.16662401	6.70026472	1407	7484	4973
81607	NM_030916	NECTIN4	nectin cell adhesion molecule 4	-1.366966079	2.87039534	6.932662	5.081263	18.721048	4.62425646	4.19452969	6.10698744	929	908	3280
3949	NM_000527,NM_001195798,NM	LDLR	low density lipoprotein receptor	-1.070554682	2.869461092	13.413535	12.610755	36.249904	6.1293084	6.03239932	7.63663685	2709	3381	9561
10928	NM_006788	RALBP1	ralA binding protein 1	1.131411986	2.867594572	14.872796	16.846807	40.088772	6.00149745	6.17701106	7.50663987	2476	3743	8733
5654	NM_002775	HTRA1	HtrA serine peptidase 1	1.16230151	2.867583607	0.110818	0.129124	0.294895	0.29776659	0.3412894	0.73352601	9	14	32
10493	NM_006373	VAT1	vesicle amine transport 1	1.187644439	2.867516055	40.513638	48.178699	109.195015	6.77889944	7.02493086	8.29014391	4272	6779	15067
92689	NM_001330764,NM_001350631	FAM114A1	family with sequence similarity 1'	-1.051963021	2.865075145	10.587403	10.49276	29.176324	5.38647251	5.3151747	6.8824826	1603	2036	5649
149837	NR_015406	LINC00654	long intergenic non-protein codin	2.193008184	2.864827274	0.314138	0.695278	0.855277	1.5285023	2.36086138	2.67922768	74	217	261
2581	NM_000153,NM_001201401,NM	GALC	galactosylceramidase	4.117156163	2.864744542	0.354547	1.473084	0.962098	1.23264156	2.71513092	2.28470378	53	292	187
9620	NM_014246	CELSR1	cadherin EGF LAG seven-pass G	-1.017230009	2.861144735	3.047672	2.999453	8.195503	5.12102205	5.09708829	6.61047072	1327	1743	4670
4189	NM_012328	DNAJB9	DnaJ heat shock protein family (H	-1.066904422	2.85837032	4.448186	4.177592	11.962564	3.58538547	3.4999516	5.02047721	432	541	1519
831	NM_001042440,NM_001042441	CAST	calpastatin	-1.147685745	2.855950391	68.026573	59.508893	184.401059	8.22332184	8.02530499	9.73416125	11694	13614	41077
3988	NM_000235,NM_001127605,NM	LIPA	lipase A, lysosomal acid type	-1.102041223	2.852022613	21.650185	19.681718	57.9126	5.88071734	5.74302869	7.37676047	2274	2757	7977
7078	NM_000362	TIMP3	TIMP metallopeptidase inhibitor 3	4.784020031	2.848176183	38.406727	183.944031	102.795319	7.68751079	9.94020313	9.19301373	8054	51482	28214
151473	NM_152527	SLC16A14	solute carrier family 16 member '	-1.530008136	2.846733645	1.120075	0.734654	3.007118	2.53954085	2.05153568	3.87868233	189	165	662
100506696	NR_046325,NR_046326	PCAT6	prostate cancer associated trans	2.142201926	2.84170748	1.755047	3.900321	4.872154	1.21691895	1.94127715	2.2537152	52	149	182
115123	NM_178450	MARCH3	membrane associated ring-CH-ty	1.605892089	2.835389803	0.124178	0.199672	0.331663	0.59399636	0.86372326	1.29257653	20	43	70
79929	NM_024871	MAP6D1	MAP6 domain containing 1	1.503955669	2.835282182	1.193269	1.798329	3.189321	1.80577475	2.24959721	3.01481644	98	197	342
26261	NM_001163499,NM_012172,NM	FBXO24	F-box protein 24	-2.503311987	2.832657177	0.534924	0.217323	1.445908	1.13562816	0.56226574	2.1362985	47	25	164
11221	NM_007207,NR_111939,NR_11	DUSP10	dual specificity phosphatase 10	1.21539141	2.829126535	2.833366	3.224478	6.906686	2.92058788	3.16793391	4.29255782	258	419	898
3576	NM_000584	CXCL8	C-X-C motif chemokine ligand 8	3.69683713	2.825666419	3.894149	14.420317	10.342756	2.90104285	4.63950051	4.26950586	254	1255	883
6253	NM_005619,NM_206900,NM_20	RTN2	reticulon 2	1.602438583	2.82166315	2.463557	2.5051	4.132111	2.0823043	2.62883416	3.34096163	127	272	441
79974	NM_001105533,NM_024913	CPED1	cadherin like and PC-esterase dc	-1.000876884	2.821501411	2.52167	2.120576	6.088266	3.52282116	3.5216664	4.93613424	412	550	1430
5268	NM_002639	SERPINB5	serpin family B member 5	-1.138148043	2.820335083	19.358519	17.032644	51.321518	5.66229636	5.47952756	7.13971124	1949	2288	6761
56143	NM_018908,NM_031501	PCDHA5	protocadherin alpha 5	1.305671359	2.820170547	0.085073	0.111884	0.228263	0.26755841	0.3412894	0.65979727	8	14	28
9054	NM_001198989,NM_021100,NR	NFS1	NFS1, cysteine desulfurase	2.331586609	2.816794321	8.269241	19.070015	21.92858	4.33483599	5.51484389	5.7821617	753	2346	2609
8000	NM_005672,NR_033343	PSCA	prostate stem cell antigen	-1.353234482	2.81678869	499.657355	370.11119	1326.068123	8.96711948	8.53172278	10.45931562	19609	19361	67935

8974	NM_001017973,NM_001017974	P4HA2	prolyl 4-hydroxylase subunit alph	2.247649712	2.814496626	13.788575	33.002585	37.712624	5.00222007	6.14550032	6.46586474	1219	3661	4220
84808	NM_001291366,NM_001291367	PERM1	PPARGC1 and ESRR induced re	3.694120386	2.811823966	0.043508	0.169312	0.117836	0.20517559	0.65251278	0.52094563	6	30	21
100642175	NR_131221	SPRY4-IT1	SPRY4 intronic transcript 1	1.365608886	2.811823966	0.195865	0.298578	0.588253	0.20517559	0.27464693	0.52094563	6	11	21
9413	NM_001127608,NM_001347995	FAM189A2	family with sequence similarity 18	-1.586572606	2.811823966	0.063138	0.03159	0.167794	0.20517559	0.13134883	0.52094563	6	5	21
54842	NM_017694	MFSD6	major facilitator superfamily doma	1.173435885	2.808174185	5.701357	6.699865	15.052184	4.78883075	5.01185634	6.24455845	1046	1640	3613
81706	NM_030949	PPP1R14C	protein phosphatase 1 regulatory	-1.640409378	2.807791714	0.697404	0.420892	1.83985	1.32354034	0.93737891	2.38534784	59	48	204
6795	NM_001015878,NM_001015879	AURKC	aurora kinase C	-1.421513662	2.804050089	5.19902	3.657168	13.72525	2.87623322	2.4491251	4.23183903	249	234	859
8269	NM_003492	TMEM187	transmembrane protein 187	1.06353918	2.802126467	4.167202	4.434513	10.989171	2.95890107	3.03667667	4.32123795	266	378	917
11031	NM_006868	RAB31	RAB31, member RAS oncogene	1.975140655	2.800454517	2.56049	5.064062	6.741062	3.45407101	4.36970972	4.85276978	391	1032	1347
1362	NM_001199775,NM_001304	CPD	carboxypeptidase D	1.353067581	2.800029291	12.120038	16.418488	31.89363	6.75119512	7.18393971	8.22802646	4190	7575	14430
196996	NM_001012642	GRAMD2A	GRAM domain containing 2A	-1.309105261	2.797294291	1.161487	0.889096	3.071414	2.24618091	1.94842076	3.52091369	147	150	506
57501	NM_001348520,NM_001348521	KIAA1257	KIAA1257	8.226550328	2.795358361	0.025758	0.448377	0.087216	0.13997292	0.89363826	0.36729312	4	45	14
56098	NM_018928,NM_032406	PCDHGC4	protocadherin gamma subfamily	14.6047121	2.795358361	0.040002	0.364255	0.103718	0.13997292	1.33632702	0.36729312	4	80	14
9283	NM_004767	GPR37L1	G protein-coupled receptor 37 lik	-1.74594355	2.795358361	0.042556	0.023756	0.105672	0.13997292	0.08023518	0.36729312	4	3	14
100862728	NR_046420	UPK1A-AS1	UPK1A antisense RNA 1	2.941787718	2.795358361	0.131629	0.39401	0.351746	0.13997292	0.38406669	0.36729312	4	16	14
345651	NM_001017992	ACTBL2	actin, beta like 2	-1.74594355	2.795358361	0.037361	0.014031	0.100014	0.13997292	0.08023518	0.36729312	4	3	14
102466658	NR_106765	MIR6510	microRNA 6510	-1.74594355	2.795358361	1.800174	0.898132	5.128184	0.13997292	0.08023518	0.36729312	4	3	14
23604	NM_014326	DAPK2	death associated protein kinase 2	4.874097474	2.794918946	0.355121	1.749633	0.942075	0.93882015	2.45415268	1.83509706	36	235	124
147923	NM_001329515,NM_001329516	ZNF420	zinc finger protein 420	1.059509798	2.793174629	0.650749	0.873518	1.76351	1.70793493	1.76638114	2.87519973	89	126	306
3664	NM_001206696,NM_006147	IRF6	interferon regulatory factor 6	1.284371793	2.789709496	6.808152	8.831006	17.877707	4.94848917	5.29919828	6.39840103	1173	2013	4025
132864	NM_001177381,NM_001177382	CPEB2	cytoplasmic polyadenylation elen	2.106454389	2.789475301	0.53309	1.129486	1.408298	2.19894236	3.09899072	3.4624849	141	397	484
114783	NM_001080434	LMTK3	lemur tyrosine kinase 3	-4.121635791	2.784400845	2.805122	0.679311	7.351189	3.86805176	2.10365798	5.28085698	534	173	1829
79618	NM_001135726,NM_001324382	HMBOX1	homeobox containing 1	1.687879209	2.782287782	0.821801	1.417024	2.225364	2.0648444	2.67262769	3.30211189	125	282	428
10916	NM_014599,NM_177433,NM_20	MAGED2	MAGE family member D2	-1.101528558	2.781608961	5.819319	5.373644	15.380414	3.72253824	3.59405489	5.12691319	479	581	1639
1952	NM_001408	CELSR2	cadherin EGF LAG seven-pass G	-1.460221517	2.780276258	0.931629	0.638808	2.438529	3.40287721	2.91797176	4.78827692	376	344	1286
55057	NM_001039775	CRYBG2	crystallin beta-gamma domain co	-1.923840083	2.777752571	9.743026	5.068016	25.441097	5.66591984	4.74787652	7.1215888	1954	1357	6676
51347	NM_001346487,NM_001346488	TAOK3	TAO kinase 3	1.09001401	2.777501257	7.533575	9.175793	20.068766	5.06284359	5.18363121	6.50882125	1273	1854	4349
54785	NM_017622	BORCS6	BLOC-1 related complex subunit	1.658881682	2.775536597	0.75149	1.248512	1.954221	1.26358189	1.73367602	2.29082241	55	122	188
102	NM_001110,NM_001320570	ADAM10	ADAM metallopeptidase domain	1.299350698	2.774420062	33.541645	43.542992	87.309019	7.36617351	7.74195387	8.83277223	6438	11177	21969
7957	NM_001018041,NM_005670	EPM2A	EPM2A, laforin glucan phosphata	2.96281891	2.773695008	0.462696	0.694471	0.62172	0.83964067	1.74192207	1.67590018	31	123	106
8218	NM_001835,NM_007098	CLTCL1	clathrin heavy chain like 1	1.748464312	2.772052877	0.37234	0.66829	0.989547	1.602974	2.19057252	2.73414401	80	187	273
80298	NM_001033050,NM_025198	MTERF2	mitochondrial transcription termin	1.168811278	2.770226302	1.520579	1.79048	3.98483	1.87753473	2.04488597	3.07278016	105	164	358
8573	NM_001126054,NM_001126055	CASK	calcium/calmodulin dependent se	1.633940543	2.767347993	1.526319	2.493204	3.968057	3.72531928	4.39083965	5.12263165	480	1048	1634
114990	NM_138440	VASN	vasorin	1.629624558	2.76722844	5.71585	9.337338	14.883534	4.05651939	4.72725823	5.46866322	614	1337	2090
9841	NM_001164313,NM_014797	ZBTB24	zinc finger and BTB domain conta	4.232287648	2.764141694	1.831833	4.392573	2.838473	2.28439817	4.12056519	3.54926401	152	860	517
9124	NM_020992	PDLIM1	PDZ and LIM domain 1	-1.553423602	2.7617326	76.680336	49.421806	199.036484	6.89460161	6.26582699	8.35244181	4632	3984	15734
84986	NM_001204300,NM_001256423	ARHGAP19	Rho GTPase activating protein 19	-1.285667976	2.760920767	2.069533	1.613075	5.387062	3.59149522	3.2626764	4.97844822	434	451	1474
121506	NM_001300784,NM_152321	ERP27	endoplasmic reticulum protein 27	-1.306124671	2.759988319	0.83984	0.633239	2.067723	1.10178443	0.90836632	2.05953665	45	46	153
153910	NR_027311,NR_027312	LOC153910	uncharacterized LOC153910	3.806190828	2.759424437	0.068455	0.307575	0.206843	0.23670415	0.75384785	0.58209675	7	36	24
112574	NM_001102575,NM_001145427	SNX18	sorting nexin 18	2.145942069	2.758192683	1.538861	3.256955	3.960797	3.08983317	4.09825806	4.44161737	295	846	1001
6711	NM_003128,NM_178313	SPTBN1	spectrin beta, non-erythrocytic 1	1.522598083	2.757632322	35.079728	53.766105	90.892558	8.42623934	9.03133823	9.88700152	13466	27395	45673
320	NM_001163	APBA1	amyloid beta precursor protein bi	1.792004859	2.754537931	0.077306	0.141666	0.20623	0.59399636	0.93737891	1.26797492	20	48	68
6615	NM_005985	SNAI1	snail family transcriptional repres	1.662203418	2.75261019	1.094545	1.828587	2.848141	1.50279892	2.01797632	2.59766727	72	160	244
220004	NM_001170753,NM_145017	PPP1R32	protein phosphatase 1 regulatory	-1.039161837	2.752412605	1.668429	1.604635	4.310213	1.80577475	1.76638114	2.9773697	98	126	332
89944	NM_138342	GLB1L2	galactosidase beta 1 like 2	-1.073663685	2.751992794	11.56266	10.784372	29.915829	5.19109656	5.09145262	6.6262836	1395	1736	4722
22973	NR_004405	LAMB2P1	laminin subunit beta 2 pseudoger	-1.822115548	2.751651132	0.692293	0.38073	1.798063	1.03161781	0.65251278	1.95553366	41	30	139
7358	NM_001184700,NM_001184701	UGDH	UDP-glucose 6-dehydrogenase	-1.153847605	2.751321315	7.796147	6.927601	20.11783	4.63612739	4.43854891	6.0589488	937	1085	3171
56114	NM_018912,NM_031993	PCDHGA1	protocadherin gamma subfamily /	-1.990027621	2.751283873	0.156545	0.079216	0.240399	0.46682716	0.25173066	1.03996283	15	10	51
101927167	NR_125398	GATA2-AS1	GATA2 antisense RNA 1	3.157042755	2.750412442	0.533754	1.713757	1.404041	1.16869613	2.30630165	2.14982678	49	207	166
677771	NR_003005	SCARNA4	small Cajal body-specific RNA 4	2.892476179	2.747709537	0.27107	1.117814	1.014064	0.07168335	0.20477631	0.19530369	2	8	7
100616117	NR_039797	MIR4653	microRNA 4653	1.116422214	2.747709537	0.468113	0.52377	1.495007	0.07168335	0.08023518	0.19530369	2	3	7
94026	NM_033482	POM121L2	POM121 transmembrane nucleo	-1.313695475	2.747709537	0.016505	0.012367	0.041266	0.07168335	0.05398282	0.19530369	2	2	7
154881	NM_001167961,NM_153033	KCTD7	potassium channel tetramerizatio	1.352583486	2.746803931	0.96645	1.308994	2.497872	2.52684435	2.89597101	3.81642381	187	338	632
5529	NM_001282179,NM_001282180	PPP2R5E	protein phosphatase 2 regulatory	2.295798035	2.745909254	4.932322	11.27839	12.647708	4.36189543	5.52084648	5.77399483	768	2356	2594
5174	NM_001201325,NM_001201326	PDZK1	PDZ domain containing 1	2.750474764	2.744811832	0.106162	0.311055	0.290072	0.3273552	0.77006602	0.76902392	10	37	34
4534	NM_000252	MTM1	myotubularin 1	1.364815967	2.744083617	1.591489	2.184874	4.124095	2.66651408	3.05333783	3.97112035	210	383	709
5792	NM_001329137,NM_001329138	PTPRF	protein tyrosine phosphatase, rec	-1.081631009	2.736206606	39.176437	36.29182	99.920058	8.19726685	8.08445853	9.64633065	11484	14186	38648
9388	NM_001308006,NM_006033	LIPG	lipase G, endothelial type	-1.821158462	2.735814452	2.471501	1.354882	6.424275	3.46409533	2.70251092	4.83089121	394	289	1326
26586	NM_001098525,NM_001286686	CKAP2	cytoskeleton associated protein 2	1.149901605	2.734744852	15.243927	17.011308	39.391078	5.64257074	5.84032177	7.0755933	1922	2953	6465
813	NM_001130674,NM_001199671	CALU	calumenin	-1.31120406	2.734569745	28.261721	21.591756	72.626943	7.2141026	6.82622283	8.6592576	5790	5900	19474
284440	NR_026956	LINC00663	long intergenic non-protein codin	-1.083181726	2.734031311	0.659558	0.605882	1.69945	1.33814853	1.26944952	2.37386535	60	74	202
3892	NM_001320198	KRT86	keratin 86	-1.208163935	2.733583212	0.43148	0.360281	1.133471	0.97665845	0.84852988	1.86820921	38	42	128
968	NM_001040059,NM_001251	CD68	CD68 molecule	1.625952274	2.732791453	3.412929	5.570102	8.809745	2.84588727	3.46799726	4.16345881	243	528	817
9766	NM_014734	SUSD6	sushi domain containing 6	1.048380068	2.728781822	5.033048	5.281457	12.910851	4.77815653	4.84389659	6.19281015	1038	1454	3484
153642	NM_198150	ARSK	arylsulfatase family member K	1.726201676	2.727993987	1.574036	2.729878	4.048652	2.61945147	3.30503223	3.91081999	202	466	678
7153	NM_001067	TOP2A	topoisomerase (DNA) II alpha	1.33176646	2.725452525	62.355408	83.136093	159.710007	8.45238665	8.86470417	9.89628008	13713	24401	45968
25789	NM_012109	TMEM59L	transmembrane protein 59 like	1.554370615	2.724119333	0.403093	0.622264	1.033768	0.71085839	0.99371033	1.45395569	25	52	84
102465472	NR_106845	MIR6787	microRNA 6787	2.321184343	2.719943748	3.023345	7.852101	8.788867	0.26755841	0.56226574	0.64076093	8	25	27
55616	NM_001143778,NM_017707	ASAP3	ArfGAP with SH3 domain, ankyri	2.198630514	2.718477559	3.577268	7.864923	9.127339	3.94164205	5.0262753	5.32400962	564	1657	1886
200879	NM_139248	LIPH	lipase H	-1.793491027	2.718073527	37.811741	21.099606	96.586571	6.52488929	5.69446357	7.95756508	3576	2664	11955
9334	NM_004776	B4GALT5	beta-1,4-galactosyltransferase 5	-1.346678879	2.717621836	38.093765	28.319246	97.297356	7.46224138	7.03566124	8.89942086	6884	6830	23010
54855	NM_017709	FAM46C	family with sequence similarity 46	2.40586271	2.716525908	0.250464	0.605705	0.640419	1.26358189	2.12902957	2.26619062	55	177	184
93185	NM_001206665,NM_001320247	IGSF8	immunoglobulin superfamily men	1.261426219	2.716396563	7.894479	9.974091	20.326432	4.2524017	4.57172706	5.64559645	709	1195	2369
150771	NM_001008949,NM_001163523	ITPRIPL1	inositol 1,4,5-trisphosphate recep	-2.061547509	2.715788138	0.166437	0.078596	0.429479	0.43999062	0.2284445	0.98063768	14	9	47
7699	NM_001300776,NM_001300777	ZNF140	zinc finger protein 140	2.763174832	2.715539607	2.110127	5.765786	5.362325	2.96361946	4.30717831	4.28338119	267	986	892
2720	NM_000404,NM_001079811,NM	GLB1	galactosidase beta 1	1.579443941	2.715050958	9.714998	14.961248	24.596679	4.66972207	5.30825016	6.07454909	960	2026	3206
66005	NM_001142674,NM_001142675	CHID1	chitinase domain containing 1	1.957061581	2.714594888	4.47932	8.728039	11.367366	3.95355025	4.87609552	5.33441396	569	1488	1900
81533	NM_001305002,NM_030790	ITFG1	integrin alpha FG-GAP repeat co	1.141196178	2.713361716	11.960571	13.683174	30.506956	5.3418659	5.52801678	6.75937345	1553	2368	5183
79148	NM_001032278,NM_024302,NM	MMP28	matrix metallopeptidase 28	-1.426595304	2.713214655	21.780754	14.066657	58.08671	5.24538562	4.74889974	6.66124136	1450	1358	4839
79690	NM_024637	GAL3ST4	galactose-3-O-sulfotransferase 4	-3.36232483	2.712250075	0.389672	0.115376	0.99692	0.97665845	0.36283659	1.86000215	38	15	127
51474	NM_001113546,NM_001113547	LIMA1	LIM domain and actin binding 1	-1.272142469	2.711972716	42.164487	32.898346	105.651918	7.24252976	6.89785283	8.67586304	5906	6203	19700
58191	NM_001100812,NM_022059	CXCL16	C-X-C motif chemokine ligand 16	-1.420902855	2.709581774	9.407526	6.819886	23.375077	4.39545784	3.91714113	5.78974263	787	740	2623
133746	NM_152405	JMY	junction mediating and regulatory	1.267458659	2.70108542	1.596866	2.028767	4.057555	3.92722123	4.24906335	5.29994267	558	945	1854
359948	NM_001077397,NM_182972	IRF2BP2	interferon regulatory factor 2 binc	1.764380341	2.699160814	16.199936	28.667449	41.158025	6.21292906	7.0236632	7.6331796	2873	6773	9538
25818	NM_001077491,NM_001077492	KLK5	kallikrein related peptidase 5	-2.058039831	2.696604243	0.562127	0.26957	1.622962	0.89996265	0.50527715	1.73991364	34	22	113
374986	NM_001270384,NM_198549	MIGA1	mitoguardin 1	1.459060145	2.694116113	1.696553	2.486725	4.308376	3.53871843	4.04427071	4.88851253	417	813	1382
255928	NM_001146261,NM_001146262	SYT14	synaptotagmin 14	-1.675674326	2.691601199	0.266844	0.16163	0.714189	1.24819466	0.86372326	2.23479744	54	43	179
84197	NM_001277971,NM_032237	POMK	protein-O-mannose kinase	1.947201203	2.690916937	0.629803	1.173662	1.576786	0.97665845	1.52971203	1.85174813	38	99	126
7148	NM_019105,NM_032470,NM_03	TNXB	tenascin XB	1.433468476	2.687805569	0.713507	0.920076	1.142502	2.38631541	2.8201133	3.62870475	166	318	549
51735	NM_001164386,NM_001164387	RAPGEF6	Rap guanine nucleotide exchang	3.572064869	2.686173687	1.706831	4.549412	2.891879	3.33894587	5.06950644	4.67237502	358	1709	1183
201895	NM_001317896,NM_001317897	SMIM14	small integral membrane protein	-1.872301867	2.685113157	4.728522	2.525504	11.924988	4.6997269	3.84241755	6.08954382	981	700	3240
9258	NM_004225	MFHAS1	malignant fibrous histiocytoma ar	1.708915313	2.684405042	0.504884	0.865898	1.281647	1.84721644	2.44407994	2.99621456	102	233	337
6297	NM_001291446,NM_001291447	SALL2	spalt like transcription factor 2	2.379146135	2.682673498	0.347467	1.205015	0.883066	1.42282133	2.32288731	2.46326383	66	210	218
100532736	NM_001204088,NM_001204089	MINOS1-NBL1	MINOS1-NBL1 readthrough	1.299068763	2.68018266	2.298933	2.985363	5.790277	2.46828542	2.78463118	3.71768886	178	309	587
100132249	NR_135128	LOC100132249	uncharacterized LOC100132249	2.557769336	2.679824903	0.210365	0.536442	0.529377	0.88013425	1.65726059	1.70368302	33	113	109
7008	NM_001145398,NM_003216	TEF	TEF, PAR bZIP transcription fact	1.918273922	2.679380079	11.452956	21.99754	28.839766	5.64477584	6.57075261	7.04853969	1925	4934	6344
192668	NM_001037160	CYS1	cystin 1	-1.969526583	2.678801035	0.057254	0.028837	0.147027	0.20517559	0.10601836	0.49997203	6	4	20
146057	NM_173500	TTBK2	tau tubulin kinase 2	1.788816994	2.675723444	0.42617	0.766641	1.076367	1.74129091	2.37683491	2.86293141	92	220	303
3688	NM_002211,NM_033668,NM_13	ITGB1	integrin subunit beta 1	1.41395322	2.675430852	148.834312	211.339063	374.764452	9.13266278	9.63164656	10.55082818	21998	41559	72387

56606	NM_001001290,NM_020041	SLC2A9	solute carrier family 2 member 9	1.549913931	2.674878213	0.181857	0.282218	0.458183	0.41264541	0.59904473	0.91876801	13	27	43
7464	NM_003389,NM_052820	CORO2A	coronin 2A	-1.019754737	2.674018435	7.628716	7.486779	19.171772	5.38559376	5.35805128	6.78289786	1602	2099	5269
135114	NM_138571	HINT3	histidine triad nucleotide binding	1.116031997	2.673686574	1.762105	1.967232	4.432068	2.76710656	2.90334193	4.04718766	228	340	750
2181	NM_004457,NM_203372	ACSL3	acyl-CoA synthetase long-chain f	1.480780917	2.668034694	17.275921	25.510616	43.527349	6.19698895	6.7569656	7.60045466	2841	5621	9323
5033	NM_000917,NM_001017962,NM	P4HA1	prolyl 4-hydroxylase subunit alph	1.904440761	2.667470022	15.430124	29.436218	38.65529	5.45257769	6.36625696	6.84736687	1680	4275	5512
80271	NM_025194	ITPKC	inositol-trisphosphate 3-kinase C	-1.063841829	2.66628953	6.185091	5.823524	15.51196	4.41969781	4.33469951	5.79190132	801	1006	2627
79762	NM_024709	C1orf115	chromosome 1 open reading fran	-2.120301024	2.663707706	3.113761	1.464904	7.800678	3.32434579	2.39263351	4.64517113	354	223	1160
10406	NM_006103	WFDC2	WAP four-disulfide core domain 2	-1.978113479	2.662985365	30.154005	15.240751	75.532341	4.14017606	3.23373217	5.50135024	653	441	2139
91663	NM_001020818,NM_001020819	MYADM	myeloid associated differentiatior	-1.302809032	2.662801479	49.997462	38.221749	128.129644	7.2801335	6.90130813	8.6872872	6063	6218	19857
63874	NM_022060	ABHD4	abhydrolase domain containing 4	-1.109206034	2.662308204	12.788941	11.540526	32.003986	5.01136367	4.86669962	6.39591961	1227	1478	4018
4983	NM_002547	OPHN1	oligophrenin 1	-1.246204241	2.662296553	0.816955	0.656491	2.050667	2.80965758	2.541776	4.08816658	236	253	773
6907	NM_001139466,NM_001139467	TBL1X	transducin beta like 1X-linked	1.665563785	2.66174283	1.781942	2.975721	4.465921	3.45071403	4.13315895	4.77851272	390	868	1277
81571	NR_026677	MIR600HG	MIR600 host gene	1.233629037	2.661485149	1.351768	1.671596	3.388876	3.15316384	3.42511354	4.46073909	310	511	1015
220	NM_000693,NM_001293815	ALDH1A3	aldehyde dehydrogenase 1 famil	1.137226084	2.6608125	135.187637	153.990848	339.781042	8.89415522	9.07930937	10.30413358	18640	28323	61002
136319	NM_145808	MTPN	myotrophin	-1.627160486	2.660648394	15.246428	9.377161	38.123871	5.87446719	5.18740997	7.27085835	2264	1859	7409
439921	NM_001008528,NM_001008529	MXRA7	matrix remodeling associated 7	-1.231762698	2.660310358	3.665047	3.191417	9.833522	3.18986937	2.92523142	4.49955999	319	346	1044
9741	NM_014713	LAPTM4A	lysosomal protein transmembrane	1.670185279	2.659256961	46.512745	77.794281	116.263214	6.33902704	7.07188599	7.73891952	3139	7005	10267
3394	NM_002163	IRF8	interferon regulatory factor 8	4.251910915	2.65817444	0.134719	0.583525	0.338963	0.43999062	1.33632702	0.96541723	14	80	46
124590	NM_001282489,NM_173477	USH1G	USH1 protein network componer	-1.038897293	2.65817444	0.102651	0.098887	0.257197	0.43999062	0.42561204	0.96541723	14	18	46
79152	NM_024306	FA2H	fatty acid 2-hydroxylase	-2.37886529	2.654810042	7.78455	3.277972	19.444702	4.27914521	3.12759409	5.64081394	723	406	2361
57326	NM_001317734,NM_001317735	PBXIP1	PBX homeobox interacting protei	1.377108903	2.652453542	7.952906	10.997724	19.890011	4.71799337	5.16458753	6.09085944	994	1829	3243
23046	NM_001252100,NM_001252102	KIF21B	kinesin family member 21B	-1.472932037	2.65010391	0.450623	0.301465	1.123456	2.35073722	1.9196316	3.56953804	161	146	525
387104	NM_001012279	SOGA3	SOGA family member 3	-1.296735185	2.648934903	0.074805	0.057559	0.187019	0.89996265	0.73744528	1.72191206	34	35	111
283140	NR_126004	LOC283140	uncharacterized LOC283140	1.238869866	2.648562418	0.026177	0.032877	0.072639	0.10623215	0.13134883	0.27153692	3	5	10
376267	NM_001308154,NM_001330182	RAB15	RAB15, member RAS oncogene	1.349932044	2.648483209	8.291326	11.530579	21.107023	4.82687594	5.24656157	6.20014324	1075	1939	3502
112724	NM_001145971,NM_138412,NR	RDH13	retinol dehydrogenase 13	-1.388663657	2.647197604	13.472516	10.011069	32.423613	4.87023937	4.41553601	6.24377015	1109	1067	3611
10129	NM_023037	FRY	FRY microtubule binding protein	1.403176303	2.647035471	0.760656	1.06938	1.893936	3.16140171	3.60313623	4.46209528	312	585	1016
2317	NM_001164317,NM_001164318	FLNB	filamin B	1.184211888	2.645065449	125.578846	148.711804	312.043049	10.17713342	10.42086734	11.57966385	45415	71858	147747
6942	NM_005650,NM_181492	TCF20	transcription factor 20	1.638566682	2.643344824	3.983384	6.612036	10.033123	4.87650783	5.569727	6.24810046	1114	2439	3622
55964	NM_019106,NM_145733	SEPT3	septin 3	-1.137450403	2.643243144	0.129233	0.113377	0.400728	0.66524311	0.59904473	1.35230545	23	27	75
135228	NM_001159587,NM_001159588	CD109	CD109 molecule	2.698940692	2.637650579	3.156407	8.553779	7.828458	4.86520493	6.26618436	6.23348291	1105	3985	3585
5412	NM_007106	UBL3	ubiquitin like 3	1.175513279	2.635151338	2.340799	2.753451	5.79936	3.44734922	3.66081122	4.76098929	389	611	1261
102465493	NR_106879	MIR6821	microRNA 6821	1.307702239	2.630987222	5.327464	7.188659	13.984297	0.49317359	0.61708849	1.05442063	16	28	52
728806	NR_033799,NR_033799_dup1	NSFP1	N-ethylmaleimide-sensitive factor	-1.02531206	2.630715929	1.436979	1.425203	3.612528	0.88013425	0.86372326	1.6852207	33	43	107
10974	NM_006829	ADIRF	adipogenesis regulatory factor	-1.201851664	2.630590639	157.851791	131.466339	390.233948	6.70439259	6.44191589	8.09120323	4055	4508	13120
6692	NM_001032367,NM_003710,NM	SPINT1	serine peptidase inhibitor, Kunitz	1.580634946	2.627172491	66.048444	103.579745	161.998863	7.28084259	7.93794412	8.66861249	6066	12811	19601
5230	NM_000291	PGK1	phosphoglycerate kinase 1	2.57793131	2.626668708	96.707619	249.581467	238.710571	7.8401152	9.20248066	9.22945628	8957	30852	28937
64839	NM_001163315	FBXL17	F-box and leucine rich repeat pro	1.023014415	2.625984929	1.547241	1.586865	3.823498	3.14487866	3.17404113	4.43334413	308	421	995
2551	NM_001197297,NM_002040	GABPA	GA binding protein transcription f	1.537580956	2.624443417	4.661838	7.034292	11.625915	4.5509035	5.14994432	5.90441835	881	1810	2844
8814	NM_001282236,NM_004196	CDKL1	cyclin dependent kinase like 1	1.906083731	2.617555524	0.605297	1.161731	1.478065	1.01352966	1.55800914	1.87636985	40	102	129
9473	NM_001039477,NM_001105556	THEMIS2	thymocyte selection associated fa	-2.542369541	2.615875362	1.531409	0.599755	3.90929	2.34351507	1.37924953	3.54415067	160	84	515
5447	NM_000941	POR	cytochrome p450 oxidoreductase	-1.165037162	2.614373263	22.524179	19.356327	55.342968	5.8025506	5.58642267	7.17301154	2152	2468	6920
51232	NM_016441	CRIM1	cysteine rich transmembrane BM	1.409534533	2.612429017	8.591599	12.122396	21.093189	5.58325587	6.06972904	6.95000779	1843	3471	5922
9572	NM_021724	NR1D1	nuclear receptor subfamily 1 grou	1.687094856	2.612137411	15.050844	25.425701	36.953114	5.38119197	6.12160473	6.74496103	1597	3600	5131
151475	NR_040038	LINC01907	long intergenic non-protein codin	-1.800958187	2.608064578	0.221102	0.123993	0.561035	0.66524311	0.40498891	1.34055586	23	17	74
84665	NM_001256267,NM_001256268	MYPN	myopalladin	-1.649411664	2.607789559	0.500134	0.298408	1.272512	1.87753473	1.38978355	2.99621456	105	85	337
123036	NM_001128595,NM_001128596	TC2N	tandem C2 domains, nuclear	-1.001200868	2.605793017	4.424397	4.330296	10.745302	4.49006587	4.48841131	5.83178982	843	1125	2702
80320	NM_001258248,NM_199262	SP6	Sp6 transcription factor	1.028765296	2.603380362	1.995869	2.056231	4.895215	3.0724647	3.10858833	4.34348418	291	400	932
10298	NM_001014831,NM_001014832	PAK4	p21 (RAC1) activated kinase 4	1.089967008	2.601755935	23.895005	24.943201	55.528618	5.98011746	6.10251937	7.3455041	2439	3552	7805
25809	NM_012263,NR_027779	TTLL1	tubulin tyrosine ligase like 1	1.230059689	2.601475895	0.862693	1.069879	2.123555	1.32354034	1.51053416	2.29691521	59	97	189
4212	NM_001220482,NM_002399,NM	MEIS2	Meis homeobox 2	1.867349828	2.600327577	0.965501	1.931465	2.465339	2.0648444	2.79655578	3.21470347	125	312	400
10205	NM_005797,NM_144765	MPZL2	myelin protein zero like 2	-1.451060756	2.5948342	28.144457	19.116063	60.654047	6.03606811	5.50881622	7.39817301	2537	2336	8097
118663	NM_001318189,NM_144587	BTBD16	BTB domain containing 16	-1.586627617	2.594611956	0.34132	0.215045	0.840669	0.71085839	0.48576948	1.40965954	25	21	80
55002	NM_001349741,NM_001349742	TMCO3	transmembrane and coiled-coil d	1.325543694	2.593361279	3.136538	4.211068	7.811297	3.44734922	3.82117143	4.73878137	389	689	1241
27250	NM_001199492,NM_014456,NM	PDCD4	programmed cell death 4	1.381127598	2.592568618	3.982719	5.51234	9.747333	3.90776647	4.34691481	5.22203654	550	1015	1754
153562	NM_001038603,NM_001244734	MARVELD2	MARVEL domain containing 2	1.285361316	2.591680332	3.730232	4.802763	9.138973	3.14487866	3.47048058	4.41525433	308	529	982
3091	NM_001243084,NM_001530,NM	HIF1A	hypoxia inducible factor 1 alpha s	2.11685598	2.591533273	32.140184	68.103476	78.287042	6.99740199	8.07337174	8.36427674	4977	14077	15864
5878	NM_001252039,NM_004583,NM	RAB5C	RAB5C, member RAS oncogene	1.718936713	2.590462368	16.198457	28.624905	39.674801	4.85254141	5.61307745	6.19485086	1095	2515	3489
220213	NM_001145373	OTUD1	OTU deubiquitinase 1	1.605846747	2.589018511	3.226923	5.188292	7.849329	3.43040645	4.06249189	4.71849769	384	824	1223
79789	NM_024734	CLMN	calmin	-1.672798623	2.588348043	6.717512	4.020102	16.33905	6.39904131	5.66820237	7.76057433	3274	2615	10423
57482	NM_020722	KIAA1211	KIAA1211	-10.35458731	2.586043107	0.119373	0.01118	0.289363	0.86002952	0.10601836	1.63800401	32	4	102
351	NM_000484,NM_001136016,NM	APP	amyloid beta precursor protein	1.130515002	2.585957073	169.655844	192.420504	413.076171	9.21948907	9.39619055	10.58870664	23365	35293	74314
100529215	NM_001172650,NM_001202425	ZNF559-ZNF177	ZNF559-ZNF177 readthrough	2.168071441	2.585554884	0.202189	0.459826	0.501634	0.61813834	1.11294744	1.25551499	21	61	67
3797	NM_002254	KIF3C	kinesin family member 3C	1.250822093	2.585046332	1.35926	1.706656	3.310649	3.0370876	3.32438062	4.29560377	283	473	900
84649	NM_001253891,NM_032564	DGAT2	diacylglycerol O-acyltransferase 2	1.464925883	2.584475957	2.062857	2.9502	4.892087	2.53954085	3.009612	3.74913666	189	370	601
10499	NM_001321703,NM_001321707	NCOA2	nuclear receptor coactivator 2	5.885360743	2.579294617	0.688743	4.072312	1.670814	2.75082071	5.11862197	3.98061446	225	1770	714
60481	NM_001242828,NM_001242830	ELOVL5	ELOVL fatty acid elongase 5	1.457583923	2.579206711	12.922042	19.397174	29.43796	5.18807576	5.71921153	6.53063733	1392	2711	4416
4324	NM_002428	MMP15	matrix metallopeptidase 15	-1.186102914	2.576598335	6.324624	5.338514	15.324091	4.76470172	4.52828878	6.09741956	1028	1158	3258
57153	NM_001145056,NM_020428	SLC44A2	solute carrier family 44 member 2	-1.477270458	2.575742369	54.131817	36.714041	131.077035	7.50777635	6.94861046	8.86792094	7106	6427	22512
10538	NM_006399	BATF	basic leucine zipper ATF-like trar	1.889511884	2.574361963	3.804049	7.208458	9.22715	2.16656821	2.92523142	3.32017262	137	346	434
23002	NM_001270520,NM_014992	DAAM1	dishevelled associated activator c	1.229007059	2.572751708	3.558045	4.375519	8.600251	4.42141376	4.706341	5.74309004	802	1317	2538
5577	NM_002736	PRKAR2B	protein kinase cAMP-dependent	2.704992569	2.570968991	0.071459	0.212611	0.187525	0.35634914	0.81765451	0.78645063	11	40	35
78987	NM_001031717,NM_001077415	CRELD1	cysteine rich with EGF like doma	1.067993851	2.568577221	3.585903	3.770732	9.631847	3.35699061	3.44292618	4.62956502	363	518	1147
23345	NM_001347701,NM_001347702	SYNE1	spectrin repeat containing nuclea	-1.326633379	2.56709894	3.543272	2.713815	11.929697	5.19912125	4.80408772	6.53515454	1403	1413	4430
152098	NM_001040432,NM_001324169	ZCWPW2	zinc finger CW-type and PWWP c	2.072658361	2.566635514	0.044537	0.080599	0.108823	0.17294258	0.3412894	0.41288872	5	14	16
389692	NM_201589	MAFA	MAF bZIP transcription factor A	4.857113602	2.566635514	0.044098	0.27079	0.134688	0.17294258	0.70406985	0.41288872	5	33	16
100500865	NR_037500	MIR3936	microRNA 3936	1.193356706	2.566635514	1.153827	1.363821	2.741865	0.17294258	0.20477631	0.41288872	5	8	16
860	NM_001015051,NM_001024630	RUNX2	runt related transcription factor 2	1.704921799	2.565730146	0.234907	0.404141	0.600167	1.18495016	1.66595383	2.09493548	50	114	158
7739	NM_001178106,NM_001178107	ZNF185	zinc finger protein 185 with LIM d	1.035118762	2.565108391	9.182468	9.570361	22.267397	5.31350078	5.36206908	6.65027765	1522	2105	4802
2591	NM_004482	GALNT3	polypeptide N-acetylgalactosamir	-1.014569234	2.559821904	26.853191	26.498569	64.606766	6.43346186	6.41283705	7.77932727	3354	4417	10560
10371	NM_006080	SEMA3A	semaphorin 3A	-1.356825051	2.559313775	7.561636	5.579267	18.188524	5.41517498	4.98692172	6.75024381	1636	1611	5150
84902	NM_032816	CEP89	centrosomal protein 89	1.211984994	2.558366565	3.524442	4.27518	8.471397	3.34619087	3.5986027	4.61256748	360	583	1133
54854	NM_017708	FAM83E	family with sequence similarity 83	-7.880269554	2.558173258	1.308379	0.16026	3.156715	1.83696709	0.40498891	2.92325564	101	17	318
10076	NM_001195001,NM_005704,NM	PTPRU	protein tyrosine phosphatase, rec	-1.10939809	2.55681039	5.562758	5.03801	13.426545	4.973267	4.82849184	6.29945438	1194	1438	3755
148534	NM_001199679,NM_152487	TMEM56	transmembrane protein 56	1.031720554	2.555782475	2.288495	2.392613	5.57294	4.03650512	4.07885924	5.33589419	605	834	1902
64699	NM_001256317,NM_024022,NM	TMPRSS3	transmembrane protease, serine	-2.460894648	2.555125411	4.164359	1.654837	10.743783	3.22566415	2.13530335	4.48228657	328	178	1031
4329	NM_001278593,NM_001278594	ALDH6A1	aldehyde dehydrogenase 6 famil	1.002140556	2.552473283	2.546587	2.642197	6.319298	3.70291935	3.70576821	4.98601009	472	632	1482
653381	NR_146393,NR_146394	SORD2P	sorbitol dehydrogenase 2, pseud	1.328136358	2.551752464	0.466432	0.619888	1.122688	1.16869613	1.41062362	2.06668624	49	87	154
80326	NM_025216	WNT10A	Wnt family member 10A	1.156563126	2.550037346	8.620815	9.985529	20.662678	4.38845666	4.58897123	5.69660757	783	1210	2456
3339	NM_001291860,NM_005529	HSPG2	heparan sulfate proteoglycan 2	-1.691402746	2.549848448	18.39348	10.886507	44.073454	8.01024937	7.25588228	9.35726545	10083	7965	31622
79025	NM_001319152,NM_001319153	FNDC11	fibronectin type III domain contai	-5.369021074	2.548855025	2.687619	0.497524	6.455014	1.9928118	0.63490936	3.10438964	117	29	367
4864	NM_000271	NPC1	NPC intracellular cholesterol tran	1.178535144	2.548299212	12.661139	14.938189	30.319628	5.91522644	6.14860501	7.25020433	2330	3669	7303
5911	NM_021033	RAP2A	RAP2A, member of RAS oncoge	1.402277841	2.548242585	1.32911	1.867364	3.189663	2.73434891	3.15872433	3.94613924	222	416	696
144699	NM_152441	FBXL14	F-box and leucine rich repeat pro	1.150818187	2.547013024	1.469709	1.69073	3.526453	2.01115992	2.16626946	3.1250843	119	183	373
653677	NR_004401	SEC1P	secretory blood group 1, pseudog	1.24305565	2.545778104	0.056469	0.067622	0.140565	0.20517559	0.25173066	0.47868902	6	10	19
497661	NM_001035005,NM_001199346	C18orf32	chromosome 18 open reading fra	-1.514575997	2.54503061	6.358145	4.186592	15.223105	3.45071403	2.91797176	4.71622624	390	344	1221
10690	NM_006581	FUT9	fucosyltransferase 9	13.37490496	2.544673252	0.122488	1.644683	0.292974	1.33814853	4.42452914	2.29082241	60	1074	188
6856	NM_006754,NM_182715	SYPL1	synaptophysin like 1	-1.010214501	2.543601024	11.995819	11.876116	28.679127	4.80338004	4.7892446	6.11864377	1057	1398	3307
9500	NM_001005332,NM_001005333	MAGED1	MAGE family member D1	1.585106891	2.542354069	12.967297	20.147681	30.113256	5.10722904	5.75631865	6.42784991	1314	2783	4109

23764	NM_001161572,NM_001161573	MAFF	MAF bZIP transcription factor F	1.124723697	2.541995551	17.352306	19.542314	41.549202	5.40046018	5.56624847	6.72561184	1619	2433	5062
5734	NM_000958	PTGER4	prostaglandin E receptor 4	-1.886569513	2.538344281	1.176445	0.623818	2.812827	2.29940604	1.62195412	3.45433079	154	109	481
219790	NM_001282941,NM_145307	RTKN2	rhotekin 2	-2.811300071	2.536142047	5.61605	1.871054	11.777892	4.84361034	3.44039495	6.15557498	1088	517	3394
90993	NM_052854	CREB3L1	cAMP responsive element bindin	-1.301806159	2.53368195	1.475234	1.130149	3.511694	2.29192162	1.99745948	3.44338646	153	157	477
440952	NR_033965	EIF1B-AS1	EIF1B antisense RNA 1	-1.846367714	2.530786015	0.085728	0.037124	0.203245	0.23670415	0.13134883	0.54161868	7	5	22
148709	NR_002929	LOC148709	actin pseudogene	-1.329394012	2.530786015	0.075574	0.059117	0.189377	0.23670415	0.18071335	0.54161868	7	7	22
7494	NM_001079539,NM_005080	XBP1	X-box binding protein 1	1.492044809	2.529676024	78.289779	116.943536	186.289931	7.1250342	7.69892196	8.45774146	5441	10847	16929
79924	NM_001253845	ADM2	adrenomedullin 2	1.412236029	2.526750771	2.751665	3.897875	6.544733	3.56379523	4.02581645	4.82563275	425	802	1321
100302736	NM_001164468,NM_001164469	TMED7-TICAM2	TMED7-TICAM2 readthrough	-1.740935135	2.526077438	3.111935	1.815347	7.385124	3.63059014	2.91432819	4.89555604	447	343	1389
7280	NM_001069,NM_001310315	TUBB2A	tubulin beta 2A class IIa	1.3737229	2.525882695	8.271287	11.557529	19.81581	3.84009957	4.27059745	5.11489271	523	960	1625
146198	NM_001305203,NM_001305204	ZFP90	ZFP90 zinc finger protein	1.77972591	2.521759749	2.803408	4.459784	6.415153	3.58232086	4.36036716	4.84239278	431	1025	1337
6484	NM_001254757,NM_001254758	ST3GAL4	ST3 beta-galactoside alpha-2,3-s	-1.739637813	2.52116356	25.235072	14.700108	59.824746	5.57479997	4.79816878	6.89056312	1832	1407	5681
29994	NM_001289975,NM_001329857	BAZ2B	bromodomain adjacent to zinc fin	2.990374862	2.520113441	2.511116	7.067488	5.677015	4.29420754	5.8248786	5.58277547	731	2921	2266
29993	NM_001199583,NM_020804	PACSIN1	protein kinase C and casein kina	2.527281081	2.520024406	0.109366	0.277418	0.260206	0.54446555	1.11294744	1.11084509	18	61	56
340348	NM_178562	TSPAN33	tetraspanin 33	-5.548581184	2.513608607	0.901969	0.161834	2.128691	1.78459878	0.52452456	2.83807727	96	23	297
1605	NM_001165928,NM_001177634	DAG1	dystroglycan 1	1.557629482	2.513412518	8.25874	13.70874	20.781344	5.59546755	6.22412724	6.90708762	1859	3869	5747
113540	NM_052999,NM_181268,NM_18	CMTM1	CKLF like MARVEL transmembra	1.196443995	2.511982317	0.60044	0.668023	1.153281	0.59399636	0.68708806	1.19154816	20	32	62
133619	NM_001286808,NM_130809	PRRC1	proline rich coiled-coil 1	-1.02589058	2.509268736	12.058882	11.770069	28.440701	5.81173868	5.77552483	7.12351846	2166	2821	6685
253559	NM_001167674,NM_001167675	CADM2	cell adhesion molecule 2	1.047694804	2.507667769	0.127435	0.135146	0.290631	1.10178443	1.1381576	1.95553366	45	63	139
2517	NM_000147	FUCA1	fucosidase, alpha-L- 1, tissue	1.332402223	2.50731774	15.289059	20.402765	36.041595	5.02384223	5.42679658	6.32315631	1238	2204	3818
2743	NM_000824,NM_001166060,NM	GLRB	glycine receptor beta	-1.148749227	2.506295096	2.445386	2.099766	5.666105	3.00540462	2.83174937	4.21906166	276	321	851
8202	NM_001174087,NM_001174088	NCOA3	nuclear receptor coactivator 3	1.051888659	2.505983141	7.077944	7.45222	16.668725	5.80977473	5.88149093	7.11965656	2163	3040	6667
150372	NM_001318323,NM_145912	NFAM1	NFAT activating protein with ITAM	-1.477002155	2.503600799	0.042645	0.031411	0.110288	0.3273552	0.2284445	0.71544405	10	9	31
26032	NM_015551	SUSD5	sushi domain containing 5	1.491607957	2.503600799	0.05227	0.078328	0.121312	0.3273552	0.46599441	0.71544405	10	20	31
116441	NM_001184723,NM_138786	TM4SF18	transmembrane 4 L six family me	1.238859246	2.503083198	12.790252	15.877411	29.528046	5.56241133	5.86554139	6.86772028	1816	3006	5591
101928403	NR_135540	LOC101928403	uncharacterized LOC101928403	2.469622993	2.502228724	0.693537	1.748012	1.654355	0.93882015	1.70865125	1.72191206	36	119	111
10235	NM_001098670,NM_001098671	RASGRP2	RAS guanyl releasing protein 2	1.829001325	2.497800993	0.119868	0.221285	0.283759	0.35634914	0.59904473	0.76902392	11	27	34
6303	NM_002970,NR_027783	SAT1	spermidine/spermine N1-acetyltra	-1.505949218	2.494004795	69.350578	46.091912	164.845239	6.23456305	5.65352499	7.54153716	2917	2588	8948
2872	NM_017572,NM_199054	MKNK2	MAP kinase interacting serine/thr	1.331308796	2.49299822	42.505785	54.573982	89.897147	6.87125028	7.2810339	8.18175432	4557	8106	13973
100874278	NR_046817,NR_110056	ERI3-IT1	ERI3 intronic transcript 1	1.739750021	2.492958874	0.576868	0.962052	1.330049	0.38477185	0.61708849	0.82068496	12	28	37
361	NM_001317384,NM_001317387	AQP4	aquaporin 4	-3.145343253	2.492958874	0.057799	0.018059	0.135619	0.38477185	0.13134883	0.82068496	12	5	37
1831	NM_001015881,NM_001318468	TSC22D3	TSC22 domain family member 3	1.091878527	2.492279563	12.166024	13.990806	29.522002	4.67116511	4.79321772	5.95442013	961	1402	2946
5955	NM_001271837,NM_002902	RCN2	reticulocalbin 2	1.741960375	2.492144452	21.609848	37.857409	50.560577	5.57941842	6.36725676	6.87867922	1838	4278	5634
1821	NM_001171184,NM_001939	DRP2	dystrophin related protein 2	2.160677873	2.491256841	0.101646	0.222593	0.238698	0.77668602	1.34717796	1.47560397	28	81	86
57211	NM_001032394,NM_001032395	ADGRG6	adhesion G protein-coupled rece	-1.461997707	2.488748419	9.906645	6.787477	23.191381	6.09046901	5.55224986	7.3932051	2636	2409	8069
158135	NM_001139442,NM_194252	TTLL11	tubulin tyrosine ligase like 11	2.242533145	2.486496465	0.378715	1.099287	0.900251	1.15225689	1.90501868	2.01587867	48	144	147
79639	NM_001300746,NM_001300747	TMEM53	transmembrane protein 53	1.215588912	2.48559191	2.109939	2.560846	4.951374	2.53954085	2.77662633	3.69710431	189	307	578
9827	NM_001080496	RGP1	RGP1 homolog, RAB6A GEF cor	2.232544865	2.483875102	1.043403	2.342582	2.450454	1.46336426	2.30073045	2.42483178	69	206	211
11022	NM_001083963,NM_001083964	TDRKH	tudor and KH domain containing	1.053331049	2.483706132	2.672756	2.726363	5.954842	2.97768275	3.04336424	4.17673594	270	380	825
1027	NM_004064	CDKN1B	cyclin dependent kinase inhibitor	-1.497256523	2.4830378	19.937342	13.330029	46.543758	5.64330615	5.07522832	6.93811829	1923	1716	5873
114088	NM_015163,NM_052978	TRIM9	tripartite motif containing 9	1.790602259	2.482277475	0.075292	0.141089	0.186332	0.46682716	0.75384785	0.96541723	15	36	46
8819	NM_003864	SAP30	Sin3A associated protein 30	1.436845366	2.480854351	2.664732	3.833909	6.215762	1.9554003	2.36086138	3.02586351	113	217	345
8663	NM_001037808,NM_001037808	EIF3C	eukaryotic translation initiation fa	1.131203846	2.479521574	33.105391	37.477724	78.517079	6.69342755	6.86967318	7.99517141	4024	6082	12272
2589	NM_020474	GALNT1	polypeptide N-acetylgalactosamir	1.472081532	2.478360221	8.448669	12.452129	19.682472	5.02609951	5.569727	6.30890656	1240	2439	3780
9518	NM_004864	GDF15	growth differentiation factor 15	-1.679070287	2.475728023	55.187164	32.908344	128.420059	6.25154321	5.51664729	7.54809721	2952	2349	8989
26225	NM_001037174,NM_012097,NM	ARL5A	ADP ribosylation factor like GTPa	2.779959888	2.474532384	3.204485	8.911307	7.536235	3.43720745	4.82461482	4.66297087	386	1434	1175
219749	NM_001329647,NM_001329648	ZNF25	zinc finger protein 25	1.100023461	2.473485965	1.254699	1.381996	3.375968	2.78853894	2.90701332	3.96539376	232	341	706
92973	NR_024006	LINC00950	long intergenic non-protein codin	1.393851744	2.472358038	0.218626	0.303897	0.510097	1.08456011	1.3579479	1.91649468	44	82	134
89122	NM_033017,NM_033091	TRIM4	tripartite motif containing 4	1.071371601	2.472188937	3.381327	3.627352	7.839495	3.60363765	3.69519054	4.83717616	438	627	1332
54874	NM_001024948,NM_001164473	FNBP1L	formin binding protein 1 like	1.585093673	2.470345587	5.098023	8.057342	11.438991	4.59109862	5.23346232	5.85984164	907	1921	2756
10370	NM_001168388,NM_001168389	CITED2	Cbp/p300 interacting transactivat	1.996141994	2.469434585	6.088304	12.323515	14.150746	3.6424092	4.5809496	4.87635737	451	1203	1370
101927765	NR_125961,NR_125962	LOC101927765	uncharacterized LOC101927765	1.237856012	2.465730482	2.386801	2.960261	5.565699	1.26358189	1.45142181	2.15654364	55	91	167
342892	NM_001193552,NM_001267779	ZNF850	zinc finger protein 850	-1.190145813	2.464850398	1.047118	0.892788	2.437555	3.15316384	2.93245472	4.35521005	310	348	940
9710	NM_014686	KIAA0355	KIAA0355	1.227804349	2.46331277	1.947975	2.394826	4.510724	3.7744826	4.05092325	5.01124268	498	817	1509
5236	NM_001172818,NM_001172819	PGM1	phosphoglucomutase 1	1.501438356	2.463266436	10.261003	15.437895	23.774699	4.68551669	5.25306686	5.95249158	971	1948	2942
5159	NM_002609	PDGFRB	platelet derived growth factor rec	-1.294994713	2.461999518	0.288907	0.223954	0.676521	1.39514558	1.1751656	2.32699908	64	66	194
10758	NM_001164281,NM_001164283	TRAF3IP2	TRAF3 interacting protein 2	1.508817217	2.461622192	1.032404	1.606	5.428944	2.86114003	3.38618649	4.03812306	246	496	745
639	NM_001198,NM_182907	PRDM1	PR/SET domain 1	-1.63136666	2.461308365	0.331645	0.204146	0.790535	1.42282133	1.02107213	2.36229074	66	54	200
10874	NM_001292045,NM_001292046	NMU	neuromedin U	-2.583415439	2.46045148	33.130083	12.872717	76.711517	4.75792697	3.47048058	6.02494052	1023	529	3096
55803	NM_001346712,NM_001346714	ADAP2	ArfGAP with dual PH domains 2	-1.743150032	2.458257544	0.315043	0.179837	0.739716	0.89996265	0.58077243	1.64757178	34	26	103
11215	NM_016248	AKAP11	A-kinase anchoring protein 11	1.436584561	2.45820305	2.226354	3.204299	5.147239	4.49333262	4.99643048	5.75254203	845	1622	2555
11005	NM_001127698,NM_001127699	SPINK5	serine peptidase inhibitor, Kazal t	-1.901183901	2.45753841	1.189385	0.712836	2.823415	2.4003046	1.70021226	3.5261099	168	118	508
8444	NM_001004023,NM_003582	DYRK3	dual specificity tyrosine phosphor	4.076691187	2.453285791	0.531792	2.259499	1.278565	1.11880553	2.53229878	1.95553366	46	251	139
7466	NM_001145853,NM_006005	WFS1	wolframin ER transmembrane gly	1.592247489	2.452660949	13.753597	21.932743	31.718119	5.63667394	6.29694166	6.91379347	1914	4072	5774
4814	NM_004148	NINJ1	ninjurin 1	1.375304827	2.452581142	29.414122	40.51543	67.82795	5.23664058	5.68594093	6.50816492	1441	2648	4347
4070	NM_002353	TACSTD2	tumor associated calcium signal t	-1.395774694	2.452485092	401.771912	288.159698	925.951538	9.66447595	9.18411138	10.95767006	31821	30461	95985
51066	NM_001256748,NM_001256749	SSUH2	ssu-2 homolog (C. elegans)	-1.131987215	2.452157674	0.720714	0.627201	1.692196	1.18495016	1.08728891	2.04513029	50	59	151
5641	NM_001008530,NM_005606	LGMN	legumain	2.022329361	2.450475476	21.730753	43.700817	49.895477	5.47670971	6.47630122	6.75052132	1709	4618	5151
11004	NM_001297655,NM_001297656	KIF2C	kinesin family member 2C	1.231730065	2.447891079	30.280726	37.357346	69.617258	6.42364978	6.7211793	7.70524285	3331	5482	10029
5150	NM_001242318,NM_002603	PDE7A	phosphodiesterase 7A	1.804541329	2.447763249	0.667878	1.210006	1.540975	2.41415945	3.14012691	3.53644652	170	410	512
2052	NM_000120,NM_001136018,NM	EPHX1	epoxide hydrolase 1	1.833937839	2.44724393	47.478487	87.597751	109.30988	6.31891475	7.18564101	7.59937662	3095	7584	9316
51669	NM_001284239,NM_016127	SARAF	store-operated calcium entry ass	1.402572392	2.447041414	29.913813	41.977327	68.640234	5.98824449	6.46979973	7.26581957	2453	4597	7383
415116	NM_001001852	PIM3	Pim-3 proto-oncogene, serine/thr	1.006044318	2.444210173	36.771893	37.035984	84.46125	6.42920394	6.43779748	7.70867468	3344	4495	10053
148229	NM_001178002,NM_138813,NR	ATP8B3	ATPase phospholipid transportin	-1.090265384	2.442917358	0.859265	0.789678	2.026938	2.43469539	2.33383942	3.5569002	173	212	520
4751	NM_001204182,NM_001204183	NEK2	NIMA related kinase 2	1.289713907	2.438759032	30.61305	39.387633	69.841009	6.00092376	6.36291929	7.27375735	2475	4265	7424
686	NM_000060,NM_001281723,NM	BTD	biotinidase	1.321359611	2.438477944	2.972957	3.881959	7.767283	2.82529613	3.17708507	3.98628107	239	422	717
25800	NM_001099406,NM_012319	SLC39A6	solute carrier family 39 member 6	1.522729685	2.4378641	10.018412	15.379635	23.265178	5.18302698	5.77602682	6.44509779	1387	2822	4159
55812	NM_001040428,NM_018418	SPATA7	spermatogenesis associated 7	1.90327583	2.437362221	1.167918	2.334828	2.677499	1.7191395	2.42372099	2.72060965	90	229	270
11320	NM_001160154,NM_012214	MGAT4A	mannosyl (alpha-1,3-)-glycoprote	-1.053382508	2.436208013	2.945769	2.667716	6.755154	4.55403551	4.48227194	5.80211118	883	1120	2646
3485	NM_000597,NM_001313990,NM	IGFBP2	insulin like growth factor binding p	2.696924395	2.435653744	0.882616	2.269555	2.022304	1.11880553	2.05815489	1.94780992	46	166	138
817	NM_001221,NM_001321566,NM	CAMK2D	calcium/calmodulin dependent pr	-1.098883428	2.434355736	2.640285	2.401182	5.980873	3.88804577	3.76158491	5.1131673	542	659	1623
2796	NM_000825,NM_001083111	GNRH1	gonadotropin releasing hormone	-1.194984421	2.433682023	2.15567	1.927673	4.947406	2.46828542	2.2611178	3.5895311	178	199	533
1633	NM_000788	DCK	deoxycytidine kinase	1.266276046	2.432740145	6.615366	8.386816	15.124811	4.1526264	4.47610634	5.38676798	659	1115	1972
148268	NM_001300993,NM_001321991	ZNF570	zinc finger protein 570	-1.851133678	2.42979361	0.478895	0.253087	1.111317	1.86749926	1.28081328	2.89541791	104	75	311
2512	NM_000146	FTL	ferritin light chain	1.051687224	2.426924785	825.186829	868.812195	1881.990601	9.45284501	9.52544981	10.73076696	27474	38606	82009
80725	NM_025248	SRCIN1	SRC kinase signaling inhibitor 1	2.906533524	2.425450464	0.260704	0.765409	0.600218	1.48977343	2.64651142	2.43038515	71	276	212
153769	NM_152550	SH3RF2	SH3 domain containing ring finge	-1.67352376	2.425166267	6.08893	3.64513	13.887592	4.2524017	3.55948145	5.48543279	709	566	2115
652968	NM_001037666	CASTOR1	cytosolic arginine sensor for mTC	1.077233034	2.423054728	11.557882	12.469107	26.329782	4.26774435	4.36970972	5.50003047	717	1032	2137
57716	NM_020956,NM_181882	PRX	periaxin	-2.136495757	2.42209453	0.948448	0.440585	2.375328	2.60744196	1.75827411	3.73798383	200	125	596
102465466	NR_106835	MIR6777	microRNA 6777	-1.002067138	2.421446091	3.406538	3.425726	7.886159	0.29776659	0.29720487	0.64076093	9	12	27
2342	NM_002028	FNTB	farnesyltransferase, CAAX box, b	1.371731469	2.421049142	3.832123	5.258234	8.719518	3.52601466	3.94776294	4.72641971	413	757	1230
127262	NM_182752	TPRG1L	tumor protein p63 regulated 1 like	1.295419438	2.420873883	5.357295	6.95553	12.211884	3.7744826	4.12372395	4.98695254	498	862	1483
11127	NM_001300791,NM_001300792	KIF3A	kinesin family member 3A	2.032785473	2.419997732	2.687983	5.474961	6.115122	4.12340669	5.10430196	5.34914834	645	1752	1920
5106	NM_001018073,NM_001291556	PCK2	phosphoenolpyruvate carboxykin	1.134250245	2.415891307	31.2924	35.646505	66.382302	6.19548549	6.37489895	7.45649068	2838	4301	8433
7162	NM_001166392,NM_006670	TPBG	trophoblast glycoprotein	2.820049286	2.415550181	14.502735	40.301296	31.999603	5.54834575	7.02408587	6.80256961	1798	6775	5342
79885	NM_001136041,NM_001330636	HDAC11	histone deacetylase 11	1.630770581	2.412078582	1.461893	2.279356	3.193445	2.3142594	2.90334193	3.40441396	156	340	463
1365	NM_001306	CLDN3	claudin 3	-4.016230123	2.411455258	17.579782	4.372639	39.868568	4.5127793	2.68551066	5.74531961	857	285	2542
3304	NM_005346	HSPA1B	heat shock protein family A (Hsp7	2.263411727	2.411255477	34.243717	77.60376	77.60318	6.40988621	7.57890919	7.66973449	3299	9977	9784
10112	NM_005733	KIF20A	kinesin family member 20A	1.062485787	2.410836179	25.359953	26.974216	57.455906	6.43813111	6.52459849	7.69792299	3365	4777	9978

11165	NM_006703	NUDT3	nudix hydrolase 3	-1.155498514	2.410372754	3.139913	2.723775	7.12723	3.05929974	2.87737723	4.22386645	288	333	854
4137	NM_001123066,NM_001123067	MAPT	microtubule associated protein ta	-1.322731272	2.410051026	0.812788	0.674591	2.075268	2.61345921	2.28388661	3.73798383	201	203	596
8705	NM_003782	B3GALT4	beta-1,3-galactosyltransferase 4	1.026588444	2.408974259	1.748018	1.799054	3.967932	1.9554003	1.98361768	2.98870613	113	155	335
109616977	NR_145743	SNORA9B	small nucleolar RNA, H/ACA box	1.926108085	2.407591447	0.873954	1.863717	2.151258	0.17294258	0.31941552	0.39027104	5	13	15
57732	NM_001172656,NM_001172657	ZFYVE28	zinc finger FYVE-type containing	1.459759885	2.407482626	0.716681	0.960606	1.420721	1.62696745	2.01797632	2.59272293	82	160	243
995	NM_001287582,NM_001287583	CDC25C	cell division cycle 25C	1.07802454	2.403480893	15.276108	16.519308	34.450374	5.04625766	5.15149273	6.28573352	1258	1812	3719
165679	NM_001040100,NM_001320679	SPTSSB	serine palmitoyltransferase small	-3.024326792	2.400558293	3.356097	1.096951	7.502835	3.10699502	1.81408935	4.26950586	299	132	883
23554	NM_012338	TSPAN12	tetraspanin 12	-1.698249361	2.400223343	1.953681	1.15431	4.41501	2.55837852	1.95552917	3.67153991	192	151	567
546	NM_000489,NM_138270	ATRX	ATRX, chromatin remodeler	1.726556502	2.400101284	4.32884	7.528491	9.863641	5.59394673	6.36925428	6.83956187	1857	4284	5482
100996741	NM_001348152	LOC100996741	uncharacterized LOC100996741	11.50674781	2.39991625	0.024753	0.36288	0.067056	0.13997292	1.1381576	0.32020933	4	63	12
767811	NR_003238,NR_003238_dup1	H2BFXP	H2B histone family member X, ps	3.124020912	2.39991625	0.088359	0.33389	0.241835	0.13997292	0.40498891	0.32020933	4	17	12
105370578	NR_133913	LINC01629	long intergenic non-protein codin	5.12858604	2.39991625	0.165986	0.940621	0.38843	0.13997292	0.61708849	0.32020933	4	28	12
9214	NM_001142473,NM_001193338	FCMR	Fc fragment of IgM receptor	4.035186879	2.39991625	0.033921	0.135865	0.074799	0.13997292	0.50527715	0.32020933	4	22	12
2202	NM_001039348,NM_001039349	EFEMP1	EGF containing fibulin like extrac	1.076997623	2.399429001	56.498021	60.991694	127.161709	7.40506809	7.51147614	8.66280044	6615	9519	19522
1654	NM_001193416,NM_001193417	DDX3X	DEAD-box helicase 3, X-linked	1.277102653	2.398041735	38.335666	49.409042	85.363464	7.35139777	7.70235932	8.60810961	6372	10873	18794
100507056	NR_108049	CCAT1	colon cancer associated transcrip	1.267838295	2.396993186	0.214955	0.271352	0.4795	0.66524311	0.80196545	1.26797492	23	39	68
170394	NM_001098637,NM_138499	PWWP2B	PWWP domain containing 2B	-1.178890827	2.391992914	5.761056	4.717401	12.63334	3.9199564	3.69943092	5.12177381	555	629	1633
7764	NM_006526	ZNF217	zinc finger protein 217	1.206172533	2.391012587	20.008305	24.161514	44.959496	6.79155982	7.05977443	8.04160698	4310	6946	12675
4133	NM_001039538,NM_002374,NM	MAP2	microtubule associated protein 2	1.878876378	2.388903118	0.256032	0.545042	0.798675	1.65056839	2.32837375	2.61239943	84	211	247
124935	NM_001284498,NM_001284499	SLC43A2	solute carrier family 43 member 2	1.317738517	2.387410231	0.350833	0.461417	0.787001	1.9554003	2.2611178	2.9773697	113	199	332
7504	NM_021083	XK	X-linked Kx blood group	-2.068428717	2.387324565	1.271194	0.616949	2.861833	2.90104285	2.04488597	4.03994054	254	164	746
109623452	NR_145780	SNORD159	small nucleolar RNA, C/D box 15	1.479755237	2.387308582	0.836273	1.447248	2.323954	0.10623215	0.15624221	0.24657076	3	6	9
100616468	NR_039896	MIR4742	microRNA 4742	1.720640609	2.387308582	0.868487	1.580002	2.072167	0.10623215	0.18071335	0.24657076	3	7	9
100847035	NR_049839	MIR548AR	microRNA 548ar	1.238869866	2.387308582	0.958838	1.433434	3.13521	0.10623215	0.13134883	0.24657076	3	5	9
728279	NM_033032	KRTAP2-2	keratin associated protein 2-2	-1.002019576	2.387308582	0.109724	0.094571	0.234789	0.10623215	0.10601836	0.24657076	3	4	9
649	NM_001199,NM_006129,NR_03	BMP1	bone morphogenetic protein 1	1.905816514	2.387026115	9.366627	17.996042	19.912735	4.98491804	5.89356976	6.21349118	1204	3066	3535
135250	NM_001243325,NM_001243327	RAET1E	retinoic acid early transcript 1E	1.269131835	2.385727271	1.069844	1.37954	2.551075	1.59082596	1.82964782	2.53718654	79	134	232
134429	NM_001308056,NM_001308057	STARD4	StAR related lipid transfer domai	-17.61700393	2.38527861	3.213009	0.158121	7.584121	3.82202563	0.80196545	5.01586734	516	39	1514
6812	NM_001032221,NM_003165	STXBP1	syntaxin binding protein 1	1.145013427	2.385133523	2.271319	2.601674	5.095618	3.24904328	3.42511354	4.41245104	334	511	980
27092	NM_014405	CACNG4	calcium voltage-gated channel au	-1.046200113	2.381510766	4.424075	4.239219	9.914278	3.9817352	3.92077761	5.17979929	581	742	1702
84419	NM_032413,NM_197955	C15orf48	chromosome 15 open reading fra	-4.066023722	2.380953804	80.98644	19.636571	180.925019	6.10174952	4.14097459	7.34108841	2657	873	7781
101927895	NR_135114,NR_135115	LOC101927895	uncharacterized LOC101927895	2.144096823	2.378676737	0.680998	1.468572	1.603658	0.49317359	0.90836632	0.98063768	16	46	47
50853	NM_015873	VILL	villin like	-1.694575402	2.377983164	21.605675	12.762531	48.283573	5.89807507	5.15381223	7.1337666	2302	1815	6733
4681	NM_001204084,NM_001204085	NBL1	neuroblastoma 1, DAN family BM	-1.191883052	2.37763809	26.824301	22.599961	60.124241	5.84855467	5.60009515	7.08354535	2223	2492	6501
284069	NM_198475	FAM171A2	family with sequence similarity 17	2.378699764	2.377214449	0.543833	1.296636	1.215296	1.26358189	2.11639955	2.11576522	55	175	161
10603	NM_020979	SH2B2	SH2B adaptor protein 2	-1.800902257	2.37606982	0.720011	0.395169	1.616992	1.24819466	0.81765451	2.09493548	54	40	158
4286	NM_000248,NM_001184967,NM	MITF	melanogenesis associated transc	1.241269545	2.375868746	1.196529	1.466659	2.56052	2.65489157	2.92160616	3.76460718	208	345	608
57763	NM_023039	ANKRA2	ankyrin repeat family A member 2	1.044714482	2.373681803	5.19247	5.433097	11.595355	3.60061161	3.65863507	4.77742372	437	610	1276
54863	NM_017723	TOR4A	torsin family 4 member A	1.407231746	2.372815464	11.596048	16.335867	25.86268	5.55304953	6.03701399	6.78181217	1804	3392	5265
64207	NM_024496	IRF2BPL	interferon regulatory factor 2 bind	1.602539386	2.372723224	4.095014	6.572566	9.135654	4.13600195	4.78526051	5.33441396	651	1394	1900
55897	NM_018670	MESP1	mesoderm posterior bHLH transc	-3.945433847	2.372312603	14.563998	3.691776	32.473049	4.12761734	2.37153003	5.32550055	647	219	1888
9867	NM_014819	PJA2	praja ring finger ubiquitin ligase 2	1.184891005	2.372241444	11.106739	13.179551	24.769325	5.74615826	5.9867238	6.9768214	2068	3274	6034
84955	NM_001128211,NM_032869	NUDCD1	NudC domain containing 1	1.717211957	2.371112259	9.815384	16.880878	21.880037	5.27795218	6.04244873	6.5019149	1484	3405	4328
79845	NM_024787	RNF122	ring finger protein 122	1.077579562	2.37103448	0.687959	0.751213	1.552359	1.18495016	1.24644988	2.00847214	50	72	146
85409	NM_001271082,NM_033120	NKD2	naked cuticle homolog 2	1.03614508	2.370699933	0.322089	0.372962	0.785379	0.73313634	0.75384785	1.36396013	26	36	76
171177	NM_133639	RHOV	ras homolog family member V	-1.205350905	2.370211993	6.354028	5.285965	14.175702	3.53238053	3.28823882	4.70366895	415	460	1210
60370	NM_021732	AVPI1	arginine vasopressin induced 1	-1.338262552	2.369507855	23.068825	17.260235	51.38612	5.01250255	4.60714063	6.23109848	1228	1226	3579
2354	NM_001114171,NM_006732	FOSB	FosB proto-oncogene, AP-1 trans	1.249792933	2.367162477	4.247346	5.347479	9.680674	4.05209571	4.35634461	5.24427765	612	1022	1782
79589	NM_024539,NM_194463	RNF128	ring finger protein 128, E3 ubiqui	1.653836168	2.366792936	6.5632	10.958404	14.692961	4.25432848	4.9500279	5.45306113	710	1569	2067
105373383	NR_131982	LOC105373383	uncharacterized LOC105373383	1.093099346	2.36484007	0.611088	0.656143	1.358533	0.41264541	0.44594451	0.83750224	13	19	38
414778	NR_052012	HCG17	HLA complex group 17 (non-prot	1.116422214	2.362762462	0.037572	0.05978	0.147781	0.07168335	0.08023518	0.16897038	2	3	6
151888	NM_001085357,NM_181780	BTLA	B and T lymphocyte associated	3.958108558	2.362762462	0.010953	0.061002	0.037241	0.07168335	0.27464693	0.16897038	2	11	6
101929302	NR_121586	RFX3-AS1	RFX3 antisense RNA 1	3.247686972	2.362762462	0.038477	0.147963	0.107194	0.07168335	0.2284445	0.16897038	2	9	6
10744	NM_006607	PTTG2	pituitary tumor-transforming 2	1.8268438	2.362762462	0.078246	0.137807	0.17935	0.07168335	0.13134883	0.16897038	2	5	6
653653	NR_027408	LOC653653	adaptor related protein complex 1	1.471633007	2.362762462	0.043096	0.050778	0.086668	0.07168335	0.10601836	0.16897038	2	4	6
1141	NM_000748	CHRNB2	cholinergic receptor nicotinic beta	5.023740936	2.362762462	0.00915	0.046465	0.020939	0.07168335	0.3412894	0.16897038	2	14	6
100131496	NR_024594	LOC100131496	uncharacterized LOC100131496	2.537265386	2.362762462	0.023223	0.052288	0.050684	0.07168335	0.18071335	0.16897038	2	7	6
5465	NM_001001928,NM_005036	PPARA	peroxisome proliferator activated	1.06441822	2.361234816	0.819901	0.87387	1.828168	3.17367098	3.25405394	4.3182457	315	448	915
11182	NM_001145099,NM_017585	SLC2A6	solute carrier family 2 member 6	-1.43526752	2.355284033	5.489528	3.946807	12.244881	3.87056622	3.39143785	5.04873431	535	498	1550
10802	NM_001252231,NM_021982	SEC24A	SEC24 homolog A, COPII coat co	1.385555459	2.354932694	4.147749	5.597462	9.269158	4.51920371	4.97210198	5.71833457	861	1594	2494
728752	NR_036504	LOC728752	uncharacterized LOC728752	-1.59991567	2.352792195	0.543874	0.339289	1.226331	0.81895953	0.56226574	1.4863075	30	25	87
1998	NM_001276457,NM_001276458	ELF2	E74 like ETS transcription factor	-1.229272847	2.352703138	3.945638	3.21982	8.686261	3.70291935	3.43022535	4.87228279	472	513	1366
25777	NM_001199579,NM_001199580	SUN2	Sad1 and UNC84 domain contair	1.755677594	2.351349238	26.106968	45.992768	57.773849	6.68559504	7.49159429	7.91102953	4002	9388	11574
6464	NM_001130040,NM_001130041	SHC1	SHC adaptor protein 1	-1.053518862	2.351195389	72.027102	68.741068	159.767149	7.92391081	7.8490117	9.15389692	9495	12042	27458
3691	NM_000213,NM_001005619,NM	ITGB4	integrin subunit beta 4	1.034223169	2.350938353	252.707014	261.769719	558.570826	10.45963889	10.50815261	11.69228845	55247	76343	159747
162073	NM_001034841,NR_028028	ITPRIPL2	inositol 1,4,5-trisphosphate recep	2.442046119	2.348840721	3.705986	9.144545	8.181258	4.84744471	6.10571785	6.0504089	1091	3560	3152
374354	NM_198514	NHLRC2	NHL repeat containing 2	1.500945903	2.346856005	1.446547	2.175424	3.194447	3.32434579	3.86146441	4.47020574	354	710	1022
10687	NM_007257	PNMA2	paraneoplastic Ma antigen 2	-1.009061485	2.34480319	2.144339	2.127781	4.734821	3.47735365	3.46550967	4.63077151	398	527	1148
6342	NM_001007098,NM_001007099	SCP2	sterol carrier protein 2	1.145104435	2.343843427	34.622163	41.676569	77.810364	5.74478835	5.93686357	6.95820045	2066	3161	5956
2896	NM_002087	GRN	granulin precursor	1.694562618	2.342968234	86.446899	146.662399	190.354446	7.63283166	8.39077035	8.85700854	7753	17554	22342
101927590	NR_120626,NR_120627	LINC01166	long intergenic non-protein codin	3.565035638	2.342793444	0.093843	0.334205	0.206023	0.3273552	0.93737891	0.6785857	10	48	29
407009	NR_029638	MIR224	microRNA 224	1.195404003	2.342793444	3.095479	3.716185	7.065892	0.3273552	0.38406669	0.6785857	10	16	29
100128076	NR_015444	LOC100128076	protein tyrosine phosphatase pse	-1.211902248	2.342793444	0.129769	0.102541	0.286019	0.3273552	0.27464693	0.6785857	10	11	29
150160	NM_014406	CCT8L2	chaperonin containing TCP1 sub	1.573107208	2.340580007	1.488402	2.342613	3.277025	1.9928118	2.50832973	2.99621456	117	246	337
56616	NM_001278302,NM_001278303	DIABLO	diablo IAP-binding mitochondrial	2.197363411	2.340237359	11.371395	24.82105	24.750188	4.66248513	5.76696418	5.85623592	955	2804	2749
11223	NM_001271733	MST1L	macrophage stimulating 1 like	11.32793706	2.339563006	0.143685	1.656451	0.317017	0.73313634	3.09255667	1.35230545	26	395	75
3418	NM_001289910,NM_001290114	IDH2	isocitrate dehydrogenase (NADP	-1.104550055	2.339049927	20.669975	18.746558	45.522409	5.22686128	5.08741362	6.43062346	1431	1731	4117
9476	NM_004851	NAPSA	napsin A aspartic peptidase	-1.144825127	2.338787302	0.701784	0.611391	1.551934	1.04948198	0.95166931	1.80975447	42	49	121
2799	NM_002076	GNS	glucosamine (N-acetyl)-6-sulfatas	1.235085222	2.338362795	15.109279	18.683582	33.203209	6.2563582	6.557382	7.47104589	2962	4888	8519
8537	NM_001316361,NM_001323347	BCAS1	breast carcinoma amplified seque	-5.288085077	2.336938594	4.609855	0.857921	10.366587	4.03874269	1.96964178	5.21239866	606	153	1742
375387	NM_198565	NRROS	negative regulator of reactive oxy	1.375511969	2.335597202	0.25466	0.35113	0.561076	0.71085839	0.90836632	1.31676564	25	46	72
54869	NM_017729,NM_133180	EPS8L1	EPS8 like 1	-1.480074508	2.332455343	58.239866	39.350815	127.795999	7.203421	6.64243014	8.41968362	5747	5188	16487
22849	NM_001178137,NM_014912	CPEB3	cytoplasmic polyadenylation elen	-1.895383268	2.331804246	0.511474	0.267601	1.128774	1.97422731	1.34717796	2.96976228	115	81	330
7430	NM_001111077,NM_003379	EZR	ezrin	1.16904023	2.331645459	229.197594	268.436569	502.367142	9.45378855	9.67881643	10.6739649	27492	42942	78841
23394	NM_001282531,NM_001282532	ADNP	activity dependent neuroprotecto	-1.576349785	2.329095966	4.31293	2.734807	9.421713	4.78483717	4.1580214	5.97451578	1043	884	2988
79446	NM_001161476,NM_001350947	WDR25	WD repeat domain 25	3.793563573	2.327715255	0.77257	3.71805	1.851635	1.43646263	2.90334193	2.3150407	67	340	192
81	NM_001322033,NM_004924	ACTN4	actinin alpha 4	1.042871848	2.326914143	176.429567	184.201508	385.841315	9.41415722	9.4746324	10.63137229	26746	37268	76546
100507472	NR_045387	LOC100507472	uncharacterized LOC100507472	2.045196461	2.326636056	0.291751	0.596249	0.637589	0.66524311	1.1381576	1.24294651	23	63	66
10512	NM_001350120,NM_001350121	SEMA3C	semaphorin 3C	-1.750189879	2.32234248	13.582973	7.738835	29.683537	6.11824215	5.32618529	7.32198275	2688	2052	7678
537	NM_001183	ATP6AP1	ATPase H+ transporting accesso	1.462618185	2.321702189	50.367798	73.764992	109.908173	6.70051131	7.24468238	7.90779849	4044	7903	11548
1543	NM_000499,NM_001319216,NM	CYP1A1	cytochrome P450 family 1 subfar	-1.829753099	2.321547593	0.21426	0.115798	0.460913	0.64188297	0.38406669	1.20457043	22	16	63
6781	NM_003155	STC1	stanniocalcin 1	4.816793908	2.318137601	0.685872	3.320056	1.495788	1.84721644	3.75752359	2.81278744	102	657	291
566	NM_001700	AZU1	azurocidin 1	-3.702500878	2.317726457	0.787599	0.212484	1.731118	0.77668602	0.25173066	1.40965954	28	10	80
9043	NM_001130527,NM_001130528	SPAG9	sperm associated antigen 9	1.115293329	2.316105413	17.772824	19.834843	38.339871	7.15215597	7.30853313	8.35809827	5545	8263	15796
10487	NM_001105530,NM_001330502	CAP1	adenylate cyclase associated pro	1.36422189	2.312198199	48.520983	66.532929	105.828963	7.04992836	7.49510488	8.25301901	5163	9411	14683
127544	NM_001127361,NM_001300826	RNF19B	ring finger protein 19B	-1.151295602	2.310749869	6.800792	5.932403	14.7467	4.24272901	4.05092325	5.40732977	704	817	2001
8455	NM_001207047,NM_001323332	ATRN	attractin	1.965816988	2.309241778	6.284496	12.780226	14.623505	5.50287791	6.4623334	6.69219736	1741	4573	4945
2355	NM_005253	FOSL2	FOS like 2, AP-1 transcription fac	3.556878757	2.30921051	5.092898	18.137663	11.050667	4.37789078	6.1578792	5.54551873	777	3693	2207
254251	NM_001166139,NM_153686,NR	LCORL	ligand dependent nuclear recepto	1.050347995	2.308570177	3.990284	4.259016	8.614538	4.45698495	4.52470876	5.62637054	823	1155	2337

5909	NM_001145657,NM_001145658	RAP1GAP	RAP1 GTPase activating protein	1.101535324	2.308147329	3.783947	4.102716	8.128441	3.72253824	3.85197241	4.86614925	479	705	1360
2734	NM_001145666,NM_001145667	GLG1	golgi glycoprotein 1	1.293747204	2.307703484	13.30833	16.711477	28.357006	6.80048832	7.16911045	7.99961433	4337	7497	12310
1999	NM_001114309,NM_004433	ELF3	E74 like ETS transcription factor	-1.356801761	2.307146656	80.946028	59.62196	175.542236	7.95578656	7.51764283	9.15860968	9708	9560	27548
57546	NM_001329928,NM_001329929	PDP2	pyruvate dehyrogenase phospha	1.031668369	2.306584234	3.186353	3.279515	6.934105	4.53672473	4.5798	5.70694011	872	1202	2474
10252	NM_001258038,NM_001258039	SPRY1	sprouty RTK signaling antagonist	1.055356204	2.305812267	5.484337	5.582506	11.736779	3.76640418	3.83857782	4.91053445	495	698	1404
64343	NM_001134432,NM_001134433	AZI2	5-azacytidine induced 2	2.667685494	2.302897336	5.056504	11.143815	8.757118	3.40634723	4.7345082	4.53091599	377	1344	1068
3554	NM_000877,NM_001288706,NM	IL1R1	interleukin 1 receptor type 1	1.894086575	2.302485315	0.628181	1.199787	1.410862	2.07360076	2.82400243	3.06922486	126	319	357
552860	NR_038210	SAMD12-AS1	SAMD12 antisense RNA 1	-1.329394012	2.302147594	0.26087	0.182724	0.55334	0.23670415	0.18071335	0.49997203	7	7	20
693144	NR_030286	MIR559	microRNA 559	-1.329394012	2.302147594	1.729515	1.233188	4.143607	0.23670415	0.18071335	0.49997203	7	7	20
48	NM_001278352,NM_002197	ACO1	aconitase 1	-1.07963508	2.301946208	6.38326	6.038492	14.09376	4.55559896	4.44991916	5.72343286	884	1094	2503
9522	NM_001290229,NM_004866,NR	SCAMP1	secretory carrier membrane prote	1.358897053	2.300891874	6.396938	8.70144	13.834629	5.32087437	5.75377238	6.50257408	1530	2778	4330
728730	NR_037875	LOC728730	uncharacterized LOC728730	-1.483988918	2.300437993	1.346773	0.903912	2.917958	2.10810423	1.69172362	3.10785943	130	117	368
51167	NM_016230	CYB5R4	cytochrome b5 reductase 4	1.473460268	2.300098921	2.253047	3.331801	4.883603	2.57080169	3.05002095	3.62870475	194	382	549
81543	NR_030891	LRRC3	leucine rich repeat containing 3	-1.160704849	2.299712821	0.469579	0.40442	1.019535	1.88750088	1.73367602	2.85055786	106	122	300
55733	NM_001122834,NM_001170564	HHAT	hedgehog acyltransferase	-1.561440816	2.298722829	2.623588	1.719796	5.606104	3.34619087	2.78063431	4.46480383	360	308	1018
340277	NM_001127364,NM_001127365	FAM221A	family with sequence similarity 22	-1.820461479	2.298386238	1.886363	1.007688	4.23355	1.87753473	1.30327576	2.83807727	105	77	297
378707	NR_145670	RN7SL3	RNA, 7SL, cytoplasmic 3	1.792200191	2.296465521	5.019464	9.047151	10.928738	1.30878272	1.86782539	2.1362985	58	139	164
200942	NM_173546	KLHDC8B	kelch domain containing 8B	1.545985479	2.296286183	0.98338	1.525423	2.12689	1.54118428	1.98361768	2.43038515	75	155	212
97	NM_001107,NM_001302616,NM	ACYP1	acylphosphatase 1	1.647813395	2.295846942	20.764806	35.404825	47.89098	3.88307318	4.56477126	5.0259897	540	1189	1525
283673	NR_026949	EWSAT1	Ewing sarcoma associated transc	3.601963778	2.29557489	0.17903	0.670337	0.402398	0.54446555	1.41062362	1.03996283	18	87	51
1305	NM_001130103,NM_001320951	COL13A1	collagen type XIII alpha 1 chain	1.002731059	2.295545619	9.973173	10.013541	21.637124	4.90744794	4.91125208	6.07897551	1139	1526	3216
677	NM_001244698,NM_001244701	ZFP36L1	ZFP36 ring finger protein like 1	1.34541241	2.294896233	34.420251	46.015633	72.043044	6.98294965	7.40807509	8.17494733	4927	8857	13907
55088	NM_001321829,NM_018017,NM	CCDC186	coiled-coil domain containing 186	-1.155156109	2.294764757	2.476707	1.696146	5.023561	3.9817352	3.78770843	5.12776797	581	672	1640
100996259	NM_001348161	FAM106B	family with sequence similarity 10	6.181420607	2.294073046	0.017366	0.107546	0.028891	0.03628685	0.2284445	0.08695753	1	9	3
102466190	NR_106779	MIR6721	microRNA 6721	1.448836667	2.294073046	0.02048	0.38914	0.552976	0.03628685	0.05398282	0.08695753	1	2	3
100847010	NR_049844	MIR5581	microRNA 5581	2.801003507	2.294073046	0.388534	1.077119	0.728945	0.03628685	0.10601836	0.08695753	1	4	3
102466192	NR_106808	MIR6750	microRNA 6750	2.801003507	2.294073046	0.117423	0.869458	0.593711	0.03628685	0.10601836	0.08695753	1	4	3
100302128	NR_031632	MIR1302-3	microRNA 1302-3	2.801003507	2.294073046	0.127634	0.441592	0.372862	0.03628685	0.10601836	0.08695753	1	4	3
100616341	NR_039636,NR_039636_dup1	MIR4435-2	microRNA 4435-2	-1.294074149	2.294073046	0.164216	0.073253	0.720295	0.03628685	0.02724389	0.08695753	1	1	3
100616493	NR_039643	MIR4441	microRNA 4441	1.448836667	2.294073046	0.189086	0.33381	0.469032	0.03628685	0.05398282	0.08695753	1	2	3
102465534	NR_106946	MIR6886	microRNA 6886	2.124920087	2.294073046	0.2845	0.779483	0.859745	0.03628685	0.08023518	0.08695753	1	3	3
406888	NR_029483	MIRLET7F1	microRNA let-7f-1	2.124920087	2.294073046	0.238182	0.452843	0.543666	0.03628685	0.08023518	0.08695753	1	3	3
677882	NR_003022_chr1,NR_003022_c	SNORA59B	small nucleolar RNA, H/ACA box	1.448836667	2.294073046	0.147173	0.161409	0.376126	0.03628685	0.05398282	0.08695753	1	2	3
100847073	NR_049813	MIR5090	microRNA 5090	-1.294074149	2.294073046	0.20798	0.010149	0.602309	0.03628685	0.02724389	0.08695753	1	1	3
57731	NM_020971,NM_025213	SPTBN4	spectrin beta, non-erythrocytic 4	-1.047049217	2.293969174	0.1123	0.081385	0.187333	0.79797761	0.77006602	1.43197761	29	37	82
101410533	NR_102684	YTHDF3-AS1	YTHDF3 antisense RNA 1 (head	1.175440748	2.288804726	0.987749	1.193645	2.189894	0.77668602	0.8787583	1.39836968	28	44	79
347733	NM_178012	TUBB2B	tubulin beta 2B class IIb	1.328131768	2.288142003	4.197891	5.577896	9.028262	3.2098649	3.58032478	4.31374565	324	575	912
6782	NM_006948	HSPA13	heat shock protein family A (Hsp7	1.353176036	2.287205258	10.116955	13.708316	21.751474	5.33186457	5.75886043	6.50520776	1542	2788	4338
103689912	NR_125749,NR_125750,NR_125	TBX2-AS1	TBX2 antisense RNA 1	-1.95058483	2.284778109	0.644132	0.345151	1.490396	0.64188297	0.36283659	1.19154816	22	15	62
56132	NM_018937	PCDHB3	protocadherin beta 3	1.853842007	2.283259308	0.195005	0.365057	0.418932	0.7550755	1.18729361	1.36396013	27	67	76
29956	NM_022075,NM_181746	CERS2	ceramide synthase 2	1.330886162	2.282448758	11.802901	15.685452	25.289701	4.89885139	5.29919828	6.06208239	1132	2013	3178
538	NM_000052,NM_001282224,NR	ATP7A	ATPase copper transporting alph	1.238198205	2.280679243	1.337766	1.659551	2.905186	3.60061161	3.88585523	4.72189817	437	723	1226
219699	NM_001244889,NM_170744	UNC5B	unc-5 netrin receptor B	-2.167824899	2.280293125	1.827255	0.842905	3.919841	3.73086534	2.73592201	4.85793043	482	297	1352
728377	NR_033942	ARHGEF34P	Rho guanine nucleotide exchang	-1.043475651	2.279878016	3.369799	3.233381	7.224296	3.88804577	3.83086756	5.02139743	542	694	1520
102465449	NR_106809	MIR6751	microRNA 6751	1.488162123	2.279732242	2.179079	3.644305	5.233451	0.20517559	0.29720487	0.43515728	6	12	17
677778	NR_003011	SCARNA15	small Cajal body-specific RNA 15	3.326460675	2.279732242	1.201293	4.084754	2.58833	0.20517559	0.59904473	0.43515728	6	27	17
101926975	NR_110558	LINC01844	long intergenic non-protein codin	1.120502413	2.279732242	0.070326	0.079039	0.144197	0.20517559	0.2284445	0.43515728	6	9	17
84532	NM_001252675,NM_001252676	ACSS1	acyl-CoA synthetase short-chain	2.278686065	2.279731036	0.792032	1.748682	1.665313	1.90722898	2.86232771	2.86293141	108	329	303
5794	NM_001161440,NM_002842	PTPRH	protein tyrosine phosphatase, re	-1.741085793	2.279339696	6.234061	3.592686	13.498656	4.63612739	3.87839419	5.79190132	937	719	2627
55671	NM_001284280,NM_001284281	PPP4R3A	protein phosphatase 4 regulatory	2.337985787	2.278544229	12.894401	29.678423	27.530087	5.8357435	7.04652047	7.00965244	2203	6882	6174
85013	NM_001297551,NM_001297552	TMEM128	transmembrane protein 128	1.897383828	2.278237249	14.508133	29.590852	33.084396	4.38142134	5.27240856	5.53008379	779	1975	2183
92370	NM_001037172,NM_001282728	PXYLP1	2-phosphoxylose phosphatase 1	-1.364392127	2.276600533	2.555662	1.907835	5.718045	3.25675288	2.86232771	4.3566691	336	329	941
9723	NM_001178129,NM_012431	SEMA3E	semaphorin 3E	-1.749593007	2.274734708	0.663318	0.380885	1.426181	2.40724866	1.79043244	3.43235848	169	129	473
57337	NM_001077203,NM_001282801	SENP7	SUMO1/sentrin specific peptidas	1.354926252	2.270510158	2.173108	3.152811	4.751038	3.48721804	3.8914258	4.5966027	401	726	1120
100507475	NR_046452	IDH1-AS1	IDH1 antisense RNA 1	1.717559751	2.269426702	1.055081	1.833637	2.27045	0.59399636	0.90836632	1.11084509	20	46	56
1953	NM_001409	MEGF6	multiple EGF like domains 6	-9.626120046	2.268435717	13.885064	1.441346	29.603302	6.66654934	3.51686905	7.8403068	3949	548	11018
5532	NM_001142353,NM_001142354	PPP3CB	protein phosphatase 3 catalytic s	1.102346915	2.264578321	3.681753	3.702679	7.005354	3.50351028	3.63226192	4.61012283	406	598	1131
51741	NM_001291997,NM_016373,NM	WWOX	WW domain containing oxidored	1.505923285	2.26386205	2.906967	4.046407	5.943429	2.84076697	3.36231592	3.90285244	242	487	674
1495	NM_001290307,NM_001290309	CTNNA1	catenin alpha 1	1.411959449	2.263098592	79.363031	112.117715	168.652601	8.19639019	8.69265706	9.37194983	11477	21652	31946
10207	NM_001350145,NM_176877	PATJ	PATJ, crumbs cell polarity compl	1.009721445	2.262846871	4.129047	4.172221	8.777784	5.14718204	5.16074844	6.30248582	1352	1824	3763
6372	NM_002993	CXCL6	C-X-C motif chemokine ligand 6	1.935913889	2.262389767	0.153165	0.303923	0.336997	0.3273552	0.58077243	0.65979727	10	26	28
80108	NM_030613	ZFP2	ZFP2 zinc finger protein	1.047302025	2.262389767	0.107641	0.110626	0.222356	0.3273552	0.3412894	0.65979727	10	14	28
9821	NM_001083617,NM_014781	RB1CC1	RB1 inducible coiled-coil 1	1.790538981	2.260205561	5.619879	10.078603	11.941376	5.2278422	6.05118491	6.38273213	1432	3426	3981
6619	NM_001039697	SNAPC3	small nuclear RNA activating con	1.028507525	2.257993704	4.062218	4.18485	8.62198	3.72809498	3.76563483	4.84135097	481	661	1336
165215	NM_177454	FAM171B	family with sequence similarity 17	-1.130499829	2.257145732	1.777164	1.575601	3.774197	2.84076697	2.68977954	3.8988521	242	286	672
1777	NM_001375	DNASE2	deoxyribonuclease 2, lysosomal	1.205446844	2.25707981	15.651706	18.891138	33.212921	4.98259535	5.2443866	6.13148574	1202	1936	3337
4804	NM_002507	NGFR	nerve growth factor receptor	1.170668524	2.256278925	0.169501	0.203155	0.371445	0.66524311	0.75384785	1.21747621	23	36	64
83862	NM_001317803,NM_031925	TMEM120A	transmembrane protein 120A	-1.167576041	2.25560259	7.053211	6.0455	14.988245	3.46742135	3.26553914	4.56665693	395	452	1096
83648	NM_053279	FAM167A	family with sequence similarity 16	1.777761198	2.255347218	0.286994	0.511528	0.608942	1.10178443	1.60397149	1.84344662	45	107	125
10653	NM_001166103,NM_021102	SPINT2	serine peptidase inhibitor, Kunitz	1.389429675	2.255139449	399.969887	555.452896	845.966261	9.45032586	9.92424213	10.62239755	27426	50915	76071
119369	NR_002779	NUDT9P1	nudix hydrolase 9 pseudogene 1	-2.638770474	2.254878549	0.340072	0.112625	0.70819	0.43999062	0.18071335	0.85412573	14	7	39
283174	NR_024344	MIR4697HG	MIR4697 host gene	-1.269041462	2.254700133	0.654293	0.515084	1.389681	2.13345087	1.87534115	3.11132089	133	140	369
158055	NM_152571	C9orf163	chromosome 9 open reading fram	1.700813432	2.252705209	0.40449	0.690465	0.859608	1.01352966	1.45142181	1.72191206	40	91	111
10611	NM_001011513,NM_001011515	PDLIM5	PDZ and LIM domain 5	1.257564468	2.252542751	20.145387	26.270179	41.139421	6.74298487	7.07086352	7.90705184	4166	7000	11542
128077	NM_153713	LIX1L	limb and CNS expressed 1 like	1.118375816	2.251088958	1.159801	1.299792	2.460749	2.46828542	2.6019054	3.48667429	178	266	493
924	NM_006137	CD7	CD7 molecule	-3.316645489	2.249310751	0.710398	0.206068	1.504921	0.91952222	0.3412894	1.58918956	35	14	97
734	NM_001126111,NM_004337	OSGIN2	oxidative stress induced growth i	1.096550539	2.248707534	6.156993	6.823412	13.069024	4.73188879	4.86008588	5.87060506	1004	1471	2777
100506207	NR_038979,NR_038980	LOC100506207	uncharacterized LOC100506207	3.098510292	2.248547381	0.055216	0.221184	0.115785	0.17294258	0.48576948	0.36729312	5	21	14
56128	NM_019120	PCDHB8	protocadherin beta 8	6.32261636	2.248547381	0.038759	0.309607	0.10202	0.17294258	0.86372326	0.36729312	5	43	14
103695432	NR_125763	LINC01167	long intergenic non-protein codin	1.486457258	2.248547381	0.207123	0.321432	0.439137	0.17294258	0.25173066	0.36729312	5	10	14
57104	NM_020376	PNPLA2	patatin like phospholipase domai	-1.235180831	2.243748353	37.720596	30.573162	79.545441	6.49423763	6.19326394	7.65127561	3500	3786	9659
117247	NM_018593	SLC16A10	solute carrier family 16 member 1	-3.871506245	2.242991084	0.078934	0.016519	0.172575	0.29776659	0.08023518	0.60191771	9	3	25
6185	NM_001135771,NM_001324299	RPN2	ribophorin II	1.371270866	2.240213825	107.772006	147.952611	226.775085	8.04878255	8.50282431	9.20940829	10357	18976	28537
51604	NM_001184728,NM_001184729	PIGT	phosphatidylinositol glycan ancho	1.507140384	2.240068333	30.198361	45.626878	63.352234	6.04054113	6.62498118	7.19193432	2545	5125	7012
677885	NR_003025_chr1,NR_003025_c	SNORA59A	small nucleolar RNA, H/ACA box	-1.739107596	2.239671283	2.909122	1.624851	6.0616	0.5190475	0.31941552	0.98063768	17	13	47
150094	NM_173354	SIK1	salt inducible kinase 1	1.230535623	2.239262131	8.467906	10.434044	17.826912	5.31719229	5.60970201	6.46010721	1526	2509	4203
255057	NM_152769	CBARP	CACN beta subunit associated re	1.298013507	2.237542968	1.650327	2.141453	3.472884	2.3579234	2.66830768	3.35562963	162	281	446
26112	NM_015621	CCDC69	coiled-coil domain containing 69	-2.787900316	2.234385766	0.706593	0.250659	1.483113	1.74129091	0.8787583	2.64141996	92	44	253
6041	NM_021133	RNASEL	ribonuclease L	-1.14460693	2.233366099	3.315997	2.898776	6.963281	3.8780834	3.69731229	4.98223411	538	628	1478
25886	NM_001161580,NM_001161581	POC1A	POC1 centriolar protein A	1.563831207	2.233235793	5.35824	8.28621	11.060143	3.59454042	4.19603101	4.6863673	435	909	1195
100505715	NR_038334	LOC100505715	uncharacterized LOC100505715	-1.036911713	2.233231164	0.613331	0.5906	1.301443	1.21691895	1.18729361	1.98602166	52	67	143
340554	NM_001010888	ZC3H12B	zinc finger CCCH-type containing	1.588229712	2.231113259	0.409188	0.65356	0.860864	1.96484452	2.48886356	2.90342651	114	242	313
9569	NM_001199207,NM_005685,NM	GTF2IRD1	GTF2I repeat domain containing	-1.441674184	2.228827003	13.603517	9.445896	28.490737	5.53017117	5.01611219	6.66918965	1775	1645	4866
1317	NM_001859	SLC31A1	solute carrier family 31 member 1	1.362816338	2.228034243	6.327918	8.635586	13.250453	4.92811449	5.36206908	6.05760375	1156	2105	3168
1452	NM_001025105,NM_001271741	CSNK1A1	casein kinase 1 alpha 1	1.217150782	2.226597818	30.720924	37.225549	63.523843	6.24864648	6.52877463	7.39302736	2946	4791	8088
100532724	NR_037804	NPHP3-ACAD11	NPHP3-ACAD11 readthrough (N	-1.012187504	2.22501273	0.167417	0.16494	0.351578	1.18495016	1.1751656	1.9400446	50	66	137
84861	NM_032775,NR_033825	KLHL22	kelch like family member 22	3.114475307	2.224927063	6.819451	21.290719	14.214562	4.19537543	5.78003646	5.30524173	680	2830	1861
56172	NM_054027	ANKH	ANKH inorganic pyrophosphate t	-2.426664136	2.223295366	0.443624	0.181944	0.931596	2.19091668	1.30327576	3.15892909	140	77	383
54436	NM_001318480,NM_018986,NR	SH3TC1	SH3 domain and tetratricopeptide	1.081034921	2.22314295	1.423227	1.508644	2.914436	2.7991369	2.89597101	3.83329054	234	338	640

7763	NM_001102420,NM_001102421	ZFAND5	zinc finger AN1-type containing 5	-1.275776183	2.222279302	18.772141	14.812966	39.010544	6.72085965	6.37324105	7.86537915	4102	4296	11212
4856	NM_002514	NOV	nephroblastoma overexpressed	2.20072928	2.221801643	1.540114	3.406505	3.233263	2.29940604	3.26839621	3.28075123	154	453	421
10804	NM_001110219,NM_001110220	GJB6	gap junction protein beta 6	3.813032801	2.221587912	0.462851	1.775738	0.983957	0.97665845	2.23214197	1.65707652	38	194	104
374879	NM_198535	ZNF699	zinc finger protein 699	1.224990929	2.220776911	0.615407	0.757883	1.292131	2.32162912	2.5605457	3.30513776	157	257	429
100507602	NR_102759,NR_102760,NR_102	TRIM52-AS1	TRIM52 antisense RNA 1 (head t	1.276084237	2.22014986	2.745464	3.490688	5.935374	1.94589385	2.21447295	2.87519973	112	191	306
100996307	NR_073179,NR_073180	LIPE-AS1	LIPE antisense RNA 1	1.213351997	2.218809755	0.073925	0.102958	0.155917	0.26755841	0.31941552	0.54161868	8	13	22
192217	NR_132962	OXCT2P1	3-oxoacid CoA-transferase 2 pse	-2.106162822	2.218809755	0.113717	0.043463	0.242333	0.26755841	0.13134883	0.54161868	8	5	22
5097	NM_001278613,NM_001278615	PCDH1	protocadherin 1	1.221093853	2.218280942	33.896563	41.211223	70.752076	7.0122804	7.29843513	8.1555894	5029	8205	13721
339192	NR_147507	LOC339192	uncharacterized LOC339192	-1.152929914	2.218005019	0.58793	0.510943	1.228134	0.56944351	0.50527715	1.05442063	19	22	52
4303	NM_001170931,NM_005938	FOXO4	forkhead box O4	1.265617825	2.214513467	1.194223	1.491838	2.418526	2.26158951	2.53704518	3.23388481	149	252	406
29948	NM_182981	OSGIN1	oxidative stress induced growth i	-1.038197912	2.213753422	10.848771	10.462567	22.57757	4.42654937	4.37502129	5.53589126	805	1036	2192
9322	NM_001288962,NM_001288963	TRIP10	thyroid hormone receptor interact	1.026719747	2.21216274	107.42177	110.200898	224.092444	7.75829681	7.7961663	8.90010894	8461	11607	23021
29967	NM_001135703,NM_013437	LRP12	LDL receptor related protein 12	1.421973547	2.211903012	5.482873	7.806313	11.47191	4.53355487	5.02289554	5.6444023	870	1653	2367
57515	NM_020755	SERINC1	serine incorporator 1	1.301606064	2.211219385	27.774908	36.196171	57.719433	6.45288807	6.8293702	7.58870688	3400	5913	9247
890	NM_001237	CCNA2	cyclin A2	1.147736277	2.211213315	18.915152	21.740004	39.314045	5.74957736	5.9449222	6.87969443	2073	3179	5638
9650	NM_001145838,NM_001145839	MTFR1	mitochondrial fission regulator 1	-1.280849429	2.21112532	10.331165	7.309867	19.499032	4.68979447	4.3482657	5.80371668	974	1016	2649
954	NM_001246,NM_203468	ENTPD2	ectonucleoside triphosphate diph	1.389303688	2.208788244	2.862569	3.985385	5.964683	2.77786255	3.19220918	3.80150191	230	427	625
54541	NM_019058	DDIT4	DNA damage inducible transcript	1.365919796	2.208060602	113.363014	155.009567	235.22435	7.60025137	8.04813647	8.73896934	7579	13832	20583
8780	NM_001348193,NM_003831,NR	RIOK3	RIO kinase 3	-1.178232418	2.20673438	29.431458	24.99469	61.023468	6.71911675	6.48492454	7.85354347	4097	4646	11120
7048	NM_001024847,NM_003242	TGFBR2	transforming growth factor beta r	1.529330635	2.20616261	8.715946	13.349001	18.018125	5.33277665	5.93326746	6.45466729	1543	3153	4187
975	NM_001297649,NM_004356	CD81	CD81 molecule	1.992038689	2.204900238	61.504265	122.703144	127.762979	6.5023672	7.48868777	7.63438306	3520	9369	9546
2051	NM_001280794,NM_001280795	EPHB6	EPH receptor B6	1.490093624	2.203705267	5.345094	8.210264	11.910012	4.38494329	4.93751728	5.48676597	781	1555	2117
55246	NM_001304529,NM_001304530	CCDC25	coiled-coil domain containing 25	1.113961007	2.203582576	3.174161	3.534404	6.440683	3.58538547	3.72876963	4.6582457	432	643	1171
51014	NM_181836	TMED7	transmembrane p24 trafficking pr	1.439958167	2.203029649	22.524025	32.474224	46.64225	6.49382995	7.0149709	7.62457574	3499	6732	9481
112770	NM_001256604,NM_001256605	GLMP	glycosylated lysosomal membran	1.012929911	2.20254677	6.813532	6.545599	13.725799	3.42357323	3.44039495	4.48762353	382	517	1035
8898	NM_001243571,NM_016156,NM	MTMR2	myotubularin related protein 2	-1.037887164	2.20227107	4.437932	4.286334	9.230173	4.40068652	4.34961533	5.50200967	790	1017	2140
100505826	NR_038341	STK4-AS1	STK4 antisense RNA 1 (head to	-1.324521912	2.202195194	0.049454	0.037054	0.094463	0.13997292	0.10601836	0.29607837	4	4	11
102466722	NR_106788_chr1,NR_106788_c	MIR6730	microRNA 6730	2.759554525	2.202195194	1.407227	4.191451	3.122151	0.13997292	0.36283659	0.29607837	4	15	11
54814	NM_001163377,NM_017659	QPCTL	glutaminyl-peptide cyclotransfera	-1.246867022	2.201223876	7.269444	5.838975	15.247675	4.11918371	3.82117143	5.21159259	643	689	1741
284358	NM_001130915,NM_001297753	MAMSTR	MEF2 activating motif and SAP d	1.068841258	2.200443747	0.277188	0.289437	0.575992	0.61813834	0.65251278	1.12461302	21	30	57
100505648	NR_040058	RAD51-AS1	RAD51 antisense RNA 1 (head t	2.163244862	2.200254774	0.862191	1.884498	1.803975	0.97665845	1.63086207	1.64757178	38	110	103
16	NM_001605	AARS	alanyl-tRNA synthetase	1.305593324	2.200153418	79.097725	103.388916	163.542465	8.01423461	8.39763704	9.14880032	10111	17638	27361
47	NM_001096,NM_001303274,NM	ACLY	ATP citrate lyase	1.173244824	2.198158508	37.027413	43.589231	76.54301	7.30311857	7.53227678	8.4344403	6161	9658	16657
5768	NM_001004128,NM_002826	QSOX1	quiescin sulfhydryl oxidase 1	1.608725349	2.197030294	37.011713	59.072559	74.299408	6.75051272	7.43136571	7.87876967	4188	9002	11317
8497	NM_001304331,NM_001304332	PPFIA4	PTPRF interacting protein alpha	6.303865869	2.196592552	0.323635	2.064625	0.672762	1.61502061	3.80944938	2.46867149	81	683	219
84278	NR_002894	MFSD14C	major facilitator superfamily doma	1.545574634	2.196443822	3.022869	4.682123	6.246301	1.73025772	2.19658485	2.6075054	91	188	246
54521	NM_001184965,NM_001184966	WDR44	WD repeat domain 44	1.100537816	2.195890129	3.45645	3.766566	7.088053	3.88804577	4.01734947	4.9689398	542	797	1464
4008	NM_001306080,NM_001330583	LMO7	LIM domain 7	-1.979085665	2.194573278	9.795796	5.039391	20.429096	6.09799915	5.13358491	7.22046225	2650	1789	7153
26260	NM_012173,NM_183420,NM_18	FBXO25	F-box protein 25	1.335659623	2.192569297	1.838016	2.476043	3.866393	2.4278825	2.77662633	3.4072329	172	307	464
641649	NM_001042595,NM_001098821	TMEM91	transmembrane protein 91	1.29972489	2.191128233	4.711182	6.079482	9.122879	2.42103729	2.73592201	3.39875952	171	297	461
29785	NM_030622	CYP2S1	cytochrome P450 family 2 subfar	-1.180643167	2.19098587	4.307412	3.659772	8.886129	3.58844358	3.37031674	4.65350501	433	490	1167
5372	NM_002676	PMM1	phosphomannomutase 1	1.138129437	2.190131767	5.398574	6.150134	11.110059	2.9541672	3.11812251	3.98061446	265	403	714
3487	NM_001552	IGFBP4	insulin like growth factor binding	1.305785045	2.188016778	30.383713	39.712208	62.475235	6.06928257	6.44917363	7.18722684	2597	4531	6989
10044	NM_001142531,NM_001142532	SH2D3C	SH2 domain containing 3C	1.278768709	2.187828383	0.056441	0.07064	0.120286	0.23670415	0.29720487	0.47868902	7	12	19
101927151	NR_110687,NR_110688,NR_11	LOC101927151	uncharacterized LOC101927151	-1.157672113	2.186935994	0.928493	0.786507	2.063286	1.40904982	1.28081328	2.20918158	65	75	175
9139	NM_001032999,NM_001039709	CBFA2T2	CBFA2/RUNX1 translocation par	1.195533015	2.186323035	4.104289	4.937142	8.479029	4.92690697	5.17680441	6.02952154	1155	1845	3106
114780	NM_001076780,NM_001278423	PKD1L2	polycystin 1 like 2 (gene/pseudog	-1.236156162	2.1846609	9.269351	10.00396	27.44598	5.88942231	5.58928181	7.00361147	2288	2473	6148
5921	NM_002890,NM_022650	RASA1	RAS p21 protein activator 1	1.008710282	2.183991139	126.737057	128.082846	260.771108	9.07856811	9.091057	10.20409192	21187	28555	56912
57534	NM_020774	MIB1	mindbomb E3 ubiquitin protein lig	1.099913854	2.182637781	5.849258	6.4435	12.001291	5.79462825	5.92966236	6.90658965	2140	3145	5745
255809	NM_001136482	C19orf38	chromosome 19 open reading fra	2.824525752	2.181986089	0.230759	0.671019	0.4902	0.3273552	0.7861039	0.64076093	10	38	27
9722	NM_001126060,NM_001164757	NOS1AP	nitric oxide synthase 1 adaptor pr	1.368003251	2.181729786	0.344752	0.48702	0.742884	1.47662927	1.78245981	2.29082241	70	128	188
5595	NM_001040056,NM_001109891	MAPK3	mitogen-activated protein kinase	1.243618732	2.18102504	14.087057	17.461792	28.622684	4.72495781	5.02880493	5.82020306	999	1660	2680
54014	NM_001007246,NM_018963,NM	BRWD1	bromodomain and WD repeat do	-1.052787599	2.179197844	7.972461	7.350647	15.091384	6.23992124	6.16671138	7.35338191	2928	3716	7848
80344	NM_001163484,NM_025230,NM	DCAF11	DDB1 and CUL4 associated fact	-1.005587427	2.177988409	9.631349	9.714208	20.87832	5.36790544	5.36006158	6.47193601	1582	2102	4238
3337	NM_001300914,NM_001313964	DNAJB1	DnaJ heat shock protein family (H	-1.031854021	2.176094355	52.412345	50.917798	107.272411	6.95122956	6.90636099	8.06667393	4819	6240	12898
3728	NM_002230,NM_021991	JUP	junction plakoglobin	-1.082378348	2.175786918	86.938061	79.971035	177.125229	8.158181	8.04439095	9.27699433	11176	13796	29908
79412	NM_001253725,NM_001253726	KREMEN2	kringle containing transmembran	2.065354921	2.175460909	2.119779	4.593775	4.377247	2.39332696	3.29105133	3.35854542	167	461	447
51056	NM_015907	LAP3	leucine aminopeptidase 3	1.201322024	2.175263983	11.129478	13.391391	22.765686	4.57114104	4.82558505	5.6592585	894	1435	2392
85453	NM_033512	TSPYL5	TSPY like 5	13.51301208	2.174469657	0.12896	1.749506	0.262334	0.64188297	3.10539622	1.15176086	22	399	59
137695	NM_001286657,NM_001286660	TMEM68	transmembrane protein 68	1.165146911	2.173779981	9.431454	10.984021	19.257432	4.56339093	4.77525187	5.65036315	889	1384	2377
24149	NM_014345	ZNF318	zinc finger protein 318	-1.556859356	2.173746517	2.763565	1.776036	5.646161	4.49496323	3.8914258	5.58027977	846	726	2262
106635534	NR_132784	SNORA104	small nucleolar RNA, H/ACA box	1.576856794	2.172157854	82.602257	130.38797	168.549103	3.60665736	4.21984192	4.66061022	439	925	1173
163051	NM_152601	ZNF709	zinc finger protein 709	1.253400491	2.171909611	0.197532	0.247892	0.401624	0.95786335	1.12560759	1.60891398	37	62	99
100293534	NM_001242823,NM_001242823	C4B_2	complement component 4B (Chic	1.012571955	2.169781292	0.408896	0.414992	0.834262	1.66222564	1.67459501	2.51121823	85	115	227
64115	NM_022153	VSIR	V-set immunoregulatory receptor	-1.565169934	2.16951753	14.527919	9.29218	29.624327	6.09853551	5.46403427	7.20454806	2651	2263	7074
4924	NM_006184	NUCB1	nucleobindin 1	1.155037753	2.168703349	26.714319	30.893034	54.451656	6.08939006	6.29449105	7.19479224	2634	4065	7026
51493	NM_014306	RTCB	RNA 2',3'-cyclic phosphate and 5	1.92118995	2.167675872	33.450642	64.34211	68.147194	6.09370103	7.02556427	7.19845841	2642	6782	7044
7976	NM_017412,NM_145866	FZD3	frizzled class receptor 3	-1.05133486	2.167227	0.272681	0.258971	0.557286	2.22275497	2.16626946	3.16227031	144	183	384
1112	NM_001085471,NM_005197	FOXN3	forkhead box N3	1.377260706	2.167187521	0.454363	0.62962	0.929568	2.17473011	2.54649137	3.10785943	138	254	368
25938	NM_015473	HEATR5A	HEAT repeat containing 5A	-1.041951736	2.167135916	2.244841	2.156011	4.571854	4.18126603	4.12530075	5.25370559	673	863	1794
23607	NM_012120	CD2AP	CD2 associated protein	-1.109548355	2.166064025	11.058668	9.978991	22.51388	5.88880227	5.74148732	6.99074853	2287	2754	6093
51306	NM_001101800,NM_001101801	FAM13B	family with sequence similarity 13	2.064979509	2.165780381	4.599777	9.553374	9.351029	4.69263929	5.70978368	5.77726713	976	2693	2600
57715	NM_001203244,NM_017893	SEMA4G	semaphorin 4G	-2.250458234	2.165578222	2.187593	0.97455	4.681381	3.37835054	2.37153003	4.41665393	369	219	983
794	NM_001740,NM_007088,NR_02	CALB2	calbindin 2	-1.463491396	2.165373247	62.173024	42.478978	126.363766	6.48647187	5.94447568	7.59242705	3481	3178	9271
10755	NM_005716,NM_202468,NM_20	GIPC1	GIPC PDZ domain containing far	-1.203522496	2.165257325	96.184496	80.587433	196.468213	7.50797825	7.24232294	8.61825723	7107	7890	18927
6670	NM_001017371,NM_001172712	SP3	Sp3 transcription factor	1.028428902	2.162908356	7.237672	7.269642	14.677054	5.45593034	5.49546602	6.55117294	1684	2314	4480
399972	NR_033839	GSEC	G-quadruplex forming sequence	-1.303068225	2.162202745	5.524896	4.228883	11.226556	2.33625658	2.03820547	3.28688662	159	163	423
158219	NM_001168339,NM_001168340	TTC39B	tetratricopeptide repeat domain 3	-1.327142495	2.161970249	1.224459	0.921184	2.489058	2.29192162	1.97664665	3.23705705	153	154	407
5865	NM_002867	RAB3B	RAB3B, member RAS oncogene	-1.84050898	2.161563579	0.460186	0.248759	0.934819	2.75626978	2.04488597	3.74913666	226	164	601
1815	NM_000797	DRD4	dopamine receptor D4	-1.037182928	2.161335482	1.122262	1.093776	2.29859	1.32354034	1.29208824	2.08792485	59	76	157
51280	NM_016548,NM_177937	GOLM1	golgi membrane protein 1	-1.001123114	2.161258237	56.960129	56.979283	115.775532	7.42722822	7.4256182	8.53460174	6718	8966	17858
22894	NM_001128226,NM_001322348	DIS3	DIS3 homolog, exosome endorib	1.816138603	2.160049409	3.195449	5.807737	6.530451	4.61228705	5.44648192	5.69141343	921	2235	2447
1491	NM_001190463,NM_001902,NM	CTH	cystathionine gamma-lyase	-1.040859176	2.158657787	6.833367	6.569699	13.866323	3.92480368	3.87089436	4.98317903	557	715	1479
150696	NM_001165977,NM_001165978	PROM2	prominin 2	1.598387835	2.157987335	25.851328	41.560204	52.313853	6.86148689	7.53346487	7.9645194	4526	9666	12013
84719	NR_046213	LINC00260	long intergenic non-protein codin	1.739750021	2.157323364	0.243177	0.424987	0.495768	0.38477185	0.61708849	0.73352601	12	28	32
84501	NM_032451	SPIRE2	spire type actin nucleation factor	-2.556763577	2.157017918	9.136652	3.576755	18.517397	4.90008261	3.61889232	5.98304325	1133	592	3006
79990	NM_024927,NR_073573,NR_07	PLEKHH3	pleckstrin homology, MyTH4 and	1.352045096	2.156714717	8.895918	12.121506	18.356062	4.7374097	5.15844008	5.81702682	1008	1821	2674
124751	NM_001304947,NM_213597	KRBA2	KRAB-A domain containing 2	1.729859556	2.156687336	0.361248	0.659399	0.72024	0.91952222	1.34717796	1.5489128	35	81	93
2709	NM_005268	GJB5	gap junction protein beta 5	3.056274525	2.156619898	3.393673	10.394423	6.888506	2.45494311	3.87839419	3.4156568	176	719	467
104	NM_001112,NM_001160230,NM	ADARB1	adenosine deaminase, RNA spec	2.535805646	2.155286466	1.278814	2.829967	1.937609	2.72881625	3.93343361	3.71541614	221	749	586
3915	NM_002293	LAMC1	laminin subunit gamma 1	1.068751486	2.154784425	24.19787	25.89003	48.998299	7.54583461	7.64126565	8.64924646	7297	10420	19339
9651	NM_001303012,NM_001303013	PLCH2	phospholipase C eta 2	-1.215778796	2.154777609	0.777384	0.70666	1.874445	2.4278825	2.20257223	3.38452567	172	189	456
100506939	NR_038277	PRKG1-AS1	PRKG1 antisense RNA 1	1.080119318	2.153763581	0.104506	0.110346	0.211115	0.29776659	0.31941552	0.58209675	9	13	24
6725	NM_080823	SRMS	src-related kinase lacking C-term	-3.240707704	2.151503214	2.028914	0.623102	4.118233	2.52684435	1.30327576	3.49199507	187	77	495
394	NM_001030055,NM_001173	ARHGAP5	Rho GTPase activating protein 5	1.29912646	2.148831622	11.031001	14.347625	22.278814	6.70262967	7.07698749	7.79877911	4050	7030	10704
2906	NM_000836	GRIN2D	glutamate ionotropic receptor NM	-1.939380479	2.148514661	0.246113	0.124341	0.497678	1.15225689	0.70406985	1.86000215	48	33	127
154467	NM_138493	CCDC167	coiled-coil domain containing 167	1.104255327	2.148283261	6.028122	6.658039	12.160859	2.21486103	2.32837375	3.14210594	143	211	378
106633809	NR_132760	SNORD144	small nucleolar RNA, C/D box 14	1.233666549	2.148148894	5.89728	7.316193	12.196385	0.59399636	0.70406985	1.06873498	20	33	53
770	NM_001217,NR_136241	CA11	carbonic anhydrase 11	1.016289082	2.147714592	3.542062	3.716746	6.784808	3.04601354	3.06652959	4.05259921	285	387	753

51077	NM_001318508,NM_015962	FCF1	FCF1 rRNA-processing protein	1.69335249	2.14747421	10.617557	18.027735	21.443776	4.71239751	5.44963197	5.78541555	990	2240	2615
11015	NM_006855,NM_016657	KDELR3	KDEL endoplasmic reticulum pro	1.208476746	2.147009042	29.542719	35.367667	59.319532	5.61058821	5.87868909	6.69710078	1879	3034	4962
677808	NR_002962	SNORA23	small nucleolar RNA, H/ACA box	-1.586572606	2.146709311	0.763363	0.517535	1.62616	0.20517559	0.13134883	0.41288872	6	5	16
25831	NM_015382	HECTD1	HECT domain E3 ubiquitin protei	-1.081950208	2.146206551	20.555931	19.019159	41.459545	7.52882258	7.4158268	8.62644522	7211	8905	19035
10087	NM_001130105,NM_005713,NM	COL4A3BP	collagen type IV alpha 3 binding	1.462102672	2.145102944	4.137881	6.02714	8.314568	4.48679171	5.01441136	5.55317472	841	1643	2219
283651	NR_022014	HMGN2P46	high mobility group nucleosomal	-2.253485045	2.144644912	0.230212	0.091549	0.45971	0.5190475	0.25173066	0.95003448	17	10	45
135886	NM_182504	WBSCR28	Williams-Beuren syndrome chron	-2.05125153	2.144644912	0.520472	0.251571	1.046054	0.5190475	0.27464693	0.95003448	17	11	45
57787	NM_001199867,NM_031417	MARK4	microtubule affinity regulating kin	1.37679469	2.144138157	4.831081	6.659189	9.738079	4.66103338	5.10669852	5.7307654	954	1755	2516
9001	NM_001079870,NM_001079871	HAP1	huntingtin associated protein 1	-1.018601602	2.14183534	0.500861	0.498431	1.026412	1.56621863	1.5486383	2.37961802	77	101	203
27242	NM_014452	TNFRSF21	TNF receptor superfamily membe	1.288997832	2.141647432	79.111504	102.088913	159.222031	8.13718986	8.50229349	9.23318447	11014	18969	29012
653080	NR_146391,NR_146391_dup1	LOC653080	glucuronidase beta pseudogene	2.560291074	2.141558727	0.714089	1.856373	1.457404	1.42282133	2.40826098	2.20270591	66	226	174
83463	NM_001142935,NM_031300	MXD3	MAX dimerization protein 3	1.005453553	2.14110875	13.777773	14.082365	28.974802	4.4636621	4.47115481	5.52684729	827	1111	2178
9317	NM_001001484,NM_001261836	PTER	phosphotriesterase related	2.355269661	2.141000634	4.059984	9.55659	8.2084	3.98869623	5.17147225	5.03786164	584	1838	1538
51752	NM_001040458,NM_001198541	ERAP1	endoplasmic reticulum aminopep	1.407613441	2.140332805	7.760645	10.922464	15.638047	5.3254638	5.80828705	6.40405688	1535	2887	4041
255082	NR_026939,NR_026940,NR_02(	CASC2	cancer susceptibility 2 (non-prote	3.243883733	2.139651152	0.129402	0.397394	0.228105	0.43999062	1.11294744	0.82068496	14	61	37
219743	NM_001040273,NM_173555,NR	TYSND1	trypsin domain containing 1	-1.674716845	2.139445518	4.599335	2.764991	8.22117	3.84266317	3.16487058	4.88548333	524	418	1379
9781	NM_001349181,NM_001349182	RNF144A	ring finger protein 144A	-2.721756131	2.13704912	0.454081	0.165322	0.94142	1.82664439	0.95166931	2.68852679	100	49	263
107987295	NR_146322	LINC02246	long intergenic non-protein codin	-1.111279759	2.136968293	0.555447	0.500829	1.119975	2.46828542	2.34470901	3.41845388	178	214	468
27198	NM_032554	HCAR1	hydroxycarboxylic acid receptor 1	1.28078173	2.136048121	1.367416	1.752548	2.75197	2.3142594	2.60642858	3.24653213	156	267	410
147179	NM_133264	WIPF2	WAS/WASL interacting protein fa	1.205951326	2.135926675	6.272799	7.573245	12.59061	5.11890861	5.38199194	6.19158434	1325	2135	3481
55841	NM_015691	WWC3	WWC family member 3	-1.544497909	2.133561686	1.920447	1.24432	3.850627	3.70855202	3.14012691	4.74213443	474	410	1244
3303	NM_005345	HSPA1A	heat shock protein family A (Hsp	2.493145505	2.132998782	45.393665	113.305511	90.988815	6.75969808	8.06969125	7.84551213	4215	14041	11058
7345	NM_004181	UCHL1	ubiquitin C-terminal hydrolase L1	2.04247531	2.132922747	0.42287	0.898894	0.881308	0.56944351	0.99371033	1.02535867	19	52	50
4758	NM_000434	NEU1	neuraminidase 1	-1.942428584	2.132922747	0.22738	0.114421	0.473691	0.56944351	0.31941552	1.02535867	19	13	50
100133991	NR_024434,NR_024435,NR_11(	MAP3K14-AS1	MAP3K14 antisense RNA 1	1.757807245	2.132671311	1.887075	3.306494	4.202001	1.602974	2.19658485	2.41925694	80	188	210
9424	NM_004823	KCNK6	potassium two pore domain chan	1.652481195	2.132609989	11.078116	18.334244	22.211828	4.89020329	5.59555204	5.9568272	1125	2484	2951
400110	NR_073430	ANKRD20A19P	ankyrin repeat domain 20 family	3.311448045	2.131961005	0.051337	0.188363	0.11134	0.35634914	0.95166931	0.6785857	11	49	29
144811	NM_001128303,NM_001350638	LACC1	laccase domain containing 1	-1.00209073	2.129908038	1.231062	1.414555	2.770224	2.70083015	2.69827959	3.66919325	216	288	566
158747	NM_001177475,NM_001330241	MOSPD2	motile sperm domain containing	1.308569528	2.129727937	2.787839	4.161869	6.130853	3.27963753	3.63226192	4.28950542	342	598	896
374875	NM_001267868,NM_001267869	HSD11B1L	hydroxysteroid 11-beta dehydrog	2.557604846	2.129186943	0.572135	1.691885	1.116435	1.10178443	1.97664665	1.78395874	45	154	118
5346	NM_001145311,NM_002666	PLIN1	perilipin 1	-1.296794162	2.128927716	0.872791	0.678046	1.800829	1.82664439	1.5673195	2.68388473	100	103	262
552900	NM_001031827,NM_001031827	BOLA2	bolA family member 2	-1.093147777	2.128916795	7.339194	6.739637	13.564289	2.89117014	2.78063431	3.87461432	252	308	660
2817	NM_002081	GPC1	glypican 1	2.545763506	2.127895834	20.428133	52.073509	40.856567	6.21589832	7.55219576	7.29503081	2879	9793	7535
150290	NM_001304794,NM_001304795	DUSP18	dual specificity phosphatase 18	1.003526648	2.127643132	3.201252	2.972504	6.531409	3.15728865	3.16180073	4.15844821	311	417	814
2539	NM_000402,NM_001042351	G6PD	glucose-6-phosphate dehydroger	1.618010846	2.126603403	33.698719	54.385543	67.116744	6.30736115	6.99462666	7.38625688	3070	6637	8030
2261	NM_000142,NM_001163213,NM	FGFR3	fibroblast growth factor receptor	1.156367173	2.126419022	0.331577	0.382918	0.666308	1.26358189	1.38978355	1.99354404	55	85	144
100302743	NR_028374	SNORA80B	small nucleolar RNA, H/ACA box	1.720640609	2.126054747	0.575182	0.903307	1.053546	0.10623215	0.18071335	0.22116494	3	7	8
5649	NM_005045,NM_173054	RELN	reelin	6.056577294	2.126054747	0.006581	0.040658	0.01288	0.10623215	0.56226574	0.22116494	3	25	8
101593348	NR_103497	LOC101593348	uncharacterized LOC101593348	2.443296723	2.126054747	0.022255	0.083376	0.067848	0.10623215	0.25173066	0.22116494	3	10	8
10151	NR_002726	HNRNPA3P1	heterogeneous nuclear ribonucle	2.684182094	2.126054747	0.021456	0.068307	0.04966	0.10623215	0.27464693	0.22116494	3	11	8
100313886	NR_031750	MIR2116	microRNA 2116	-1.002019576	2.126054747	0.683172	0.742339	1.855346	0.10623215	0.10601836	0.22116494	3	4	8
102466745	NR_106890	MIR6832	microRNA 6832	-1.937182024	2.126054747	0.78786	0.41241	2.064245	0.10623215	0.05398282	0.22116494	3	2	8
51231	NM_001025778,NM_001308420	VRK3	vaccinia related kinase 3	1.033481534	2.124858482	7.311485	7.774314	14.679478	3.91752664	3.96195135	4.95359477	554	765	1448
610	NM_001194	HCN2	hyperpolarization activated cyclic	-1.14246819	2.124501303	0.496961	0.434733	0.994338	1.40904982	1.29208824	2.1765086	65	76	170
163183	NM_001039876,NM_001297735	SYNE4	spectrin repeat containing nuclea	-1.012196293	2.123094213	1.422062	1.414689	3.008492	1.54118428	1.52971203	2.33885915	75	99	196
9537	NM_001258320,NM_001258321	TP53I11	tumor protein p53 inducible prote	-1.368996484	2.122617296	14.516488	10.831173	29.021526	5.63667394	5.19418688	6.70714387	1914	1868	4997
57700	NM_001135051,NM_020940	FAM160B1	family with sequence similarity 16	-1.241895544	2.121697216	2.847062	2.295758	5.684414	4.09788139	3.80552077	5.13798608	633	681	1652
106635684	NR_132981_chr5,NR_132981_c	SNORD141B	small nucleolar RNA, C/D box 14	1.78151163	2.121604513	6.440764	11.461613	12.858667	0.73313634	1.12560759	1.26797492	26	62	68
10613	NM_001100626,NM_001347856	ERLIN1	ER lipid raft associated 1	-1.06369266	2.121342719	18.809218	17.882837	38.012163	5.91583524	5.82827094	6.98816212	2331	2928	6082
5934	NM_001323608,NM_001323609	RBL2	RB transcriptional corepressor lik	-1.087489714	2.120599019	11.770215	10.807481	23.197601	5.8025506	5.68380238	6.87333758	2152	2644	5613
7134	NM_003280	TNNC1	troponin C1, slow skeletal and ca	-1.926434989	2.119686565	6.606191	3.428109	13.162581	2.45494311	1.73367602	3.39308282	176	122	459
80315	NM_001308189,NM_001308191	CPEB4	cytoplasmic polyadenylation elen	1.320850867	2.119088072	1.990364	2.631358	3.96543	4.10857187	4.48963605	5.14728952	638	1126	1663
4335	NM_020310	MNT	MAX network transcriptional repr	-1.114736794	2.11897375	4.569485	4.101397	9.099328	4.49496323	4.34556265	5.54423878	846	1014	2205
2637	NM_001301687,NM_001485	GBX2	gastrulation brain homeobox 2	-1.184729479	2.118582956	0.115985	0.087118	0.230376	0.26755841	0.2284445	0.52094563	8	9	21
4987	NM_000913,NM_001200019,NM	OPRL1	opioid related nociceptin receptor	-2.633004277	2.118520099	0.599882	0.229626	1.203949	1.59082596	0.81765451	2.39673966	79	40	206
4851	NM_017617	NOTCH1	notch 1	1.283459469	2.117110047	1.902629	2.4449	3.784985	4.18934543	4.53185994	5.22922293	677	1161	1763
389384	NM_001010903	C6orf222	chromosome 6 open reading fran	2.235135808	2.116809555	0.084052	0.201142	0.178193	0.41264541	0.80196545	0.76902392	13	39	34
692247	NR_147607,NR_147608	LOC692247	uncharacterized LOC692247	-1.857687274	2.11674954	3.404977	1.781727	6.778867	1.48977343	0.97983233	2.27238811	71	51	185
339665	NM_001001479,NM_001318370	SLC35E4	solute carrier family 35 member E	1.920427627	2.116404192	6.871416	13.217779	13.687385	4.18126603	5.08417426	5.22043469	673	1727	1752
8566	NM_001331030,NM_003681	PDXK	pyridoxal kinase	-2.292848232	2.116059043	32.60335	14.226696	64.969404	7.87747369	6.6882213	8.95562343	9193	5357	23926
161145	NM_001348541,NM_001348542	TMEM229B	transmembrane protein 229B	-1.434710698	2.116016905	1.095252	0.76522	2.186373	2.44147625	2.03149389	3.37591747	174	162	453
2869	NM_005308	GRK5	G protein-coupled receptor kinas	-1.210247075	2.115820318	1.07609	0.89189	2.154078	1.88750088	1.69172362	2.74755257	106	117	276
23446	NM_001286730,NM_001330731	SLC44A1	solute carrier family 44 member	-1.016608926	2.114625232	10.178825	10.410934	21.767273	6.70051131	6.67697624	7.77360406	4044	5315	10518
27350	NM_014508	APOBEC3C	apolipoprotein B mRNA editing e	1.580808792	2.113412241	5.660425	8.954509	11.24419	2.83047153	3.41483526	3.79935756	240	507	624
1486	NM_004388	CTBS	chitobiase	1.107212998	2.113089633	4.500068	4.987977	8.944922	3.86805176	4.00541159	4.89455192	534	790	1388
84918	NM_032832	LRP11	LDL receptor related protein 11	-1.563157673	2.112168163	17.167034	10.997869	34.084728	5.94237097	5.31102396	7.00872471	2375	2030	6170
79369	NM_001330492,NM_030765	B3GNT4	UDP-GlcNAc:betaGal beta-1,3-N	1.383577516	2.110818976	0.358141	0.48896	0.75095	1.01352966	1.26944952	1.65707652	40	74	104
10673	NM_001145645,NM_006573	TNFSF13B	TNF superfamily member 13b	2.220188692	2.110375629	0.340191	0.755902	0.679173	0.91952222	1.57657016	1.52834475	35	104	91
2634	NM_004120	GBP2	guanylate binding protein 2	1.319073033	2.10971158	5.059371	6.686069	10.038338	4.41797983	4.80113128	5.45918641	800	1410	2076
56256	NM_019605	SERTAD4	SERTA domain containing 4	1.335359099	2.1082429	1.023707	1.372446	2.039846	1.59082596	1.88281796	2.39105499	79	141	205
286495	NR_030737	TTC3P1	tetratricopeptide repeat domain 3	-3.299211779	2.107722959	0.200534	0.061613	0.405539	0.59399636	0.20477631	1.05442063	20	8	52
1386	NM_001256090,NM_001256091	ATF2	activating transcription factor 2	2.882331944	2.107141847	3.735264	10.673474	7.317081	4.00938005	5.47705979	5.03695187	593	2284	1537
256691	NM_001347990,NM_153267,NR	MAMDC2	MAM domain containing 2	-2.071532244	2.106990448	1.039727	0.487955	2.011445	2.15010456	1.41062362	3.04408938	135	87	350
3655	NM_000210,NM_001079818,NM	ITGA6	integrin subunit alpha 6	1.379888615	2.105371621	58.26233	80.420771	115.223767	8.37214592	8.83550179	9.4439395	12969	23911	33583
100652853	NR_046368	LINC01023	long intergenic non-protein codin	1.798250867	2.104979523	1.889027	3.412086	3.771037	0.86002952	1.30327576	1.4430085	32	77	83
84552	NM_032510	PARD6G	par-6 family cell polarity regulator	1.359914537	2.103728704	0.299833	0.404096	0.592833	1.08456011	1.33632702	1.74883089	44	80	114
164091	NM_178422	PAQR7	progestin and adipoQ receptor fa	-1.730172614	2.102760869	20.242916	11.713092	40.01376	5.92130285	5.14761859	6.98108463	2340	1807	6052
90246	NR_026954	LOC90246	uncharacterized LOC90246	2.62796495	2.102549282	0.642568	1.697948	1.277384	1.44997615	2.47408961	2.21562831	68	239	176
382	NM_001663	ARF6	ADP ribosylation factor 6	1.141115021	2.102249518	45.084255	51.506413	89.076729	7.44230784	7.6317287	8.50989841	6789	10351	17554
647135	NM_001271870,NM_001330684	SRGAP2B	SLIT-ROBO Rho GTPase activat	-1.021854581	2.101703628	4.55994	4.468744	9.011993	4.77278965	4.74274951	5.81649676	1034	1352	2673
3222	NM_018953,NR_003084	HOXC5	homeobox C5	-1.890555353	2.101582411	0.161648	0.080741	0.317766	0.3273552	0.18071335	0.62147005	10	7	26
360132	NR_003949,NR_027339,NR_027	FKBP9P1	FK506 binding protein 9 pseudog	3.559622691	2.10093069	0.209184	0.831963	0.509046	0.64188297	1.58576189	1.12461302	22	105	57
81617	NM_001079670,NM_001287337	CAB39L	calcium binding protein 39 like	-1.118258646	2.099889859	0.804741	0.726911	1.602275	1.9554003	1.83736459	2.81703332	113	135	292
950	NM_001204255,NM_005506	SCARB2	scavenger receptor class B mem	1.105498892	2.097620775	23.984553	26.504783	47.208878	6.8093619	6.95283475	7.87138837	4364	6446	11259
9422	NM_003417	ZNF264	zinc finger protein 264	1.295281468	2.097141384	5.117202	6.63666	10.087529	5.94535574	6.31328884	7.00151446	2380	4119	6139
55320	NM_018353	MIS18BP1	MIS18 binding protein 1	1.215166391	2.096923019	8.987082	10.938225	17.717014	5.42033281	5.69552536	6.4709259	1642	2666	4235
5547	NM_001319214,NM_005040,NM	PRCP	prolylcarboxypeptidase	4.015251486	2.094590638	3.824821	15.285138	7.54129	3.8730763	5.80287809	4.88750351	536	2876	1381
3775	NM_002245	KCNK1	potassium two pore domain chan	1.298404614	2.09425189	20.175711	26.224163	39.712917	5.44162746	5.81073899	6.49066263	1667	2892	4294
9975	NM_001145425,NM_005126,NR	NR1D2	nuclear receptor subfamily 1 grou	1.119662494	2.09326407	5.519041	6.301327	11.064358	4.89761911	5.05551521	5.93794468	1131	1692	2912
64834	NM_001256399,NM_001256401	ELOVL1	ELOVL fatty acid elongase 1	1.008071136	2.091122978	104.741797	106.234473	206.030798	7.34327403	7.35480054	8.40292197	6336	8534	16296
7168	NM_000366,NM_001018004,NM	TPM1	tropomyosin 1 (alpha)	-1.725831968	2.090525198	19.001473	11.235859	36.486563	5.14094677	4.38295215	6.18338553	1346	1042	3461
54852	NM_001104554,NM_017705	PAQR5	progestin and adipoQ receptor fa	1.074691597	2.090522264	3.424381	3.681876	6.717284	4.19336823	4.29181484	5.21561844	679	975	1746
2781	NM_002073	GNAZ	G protein subunit alpha z	2.04247531	2.090381611	0.148356	0.304129	0.288856	0.56944351	0.99371033	1.01060517	19	52	49
100130519	NM_001190844	TMEM221	transmembrane protein 221	-2.264662914	2.090196262	0.162258	0.070591	0.319414	0.38477185	0.18071335	0.71544405	12	7	31
1995	NM_001420,NM_032281	ELAVL3	ELAV like RNA binding protein 3	1.193356706	2.089503314	0.022351	0.033451	0.051392	0.17294258	0.20477631	0.34394329	5	8	13
5307	NM_002653	PITX1	paired like homeodomain 1	1.109701011	2.089074067	14.918743	16.566778	29.286076	5.15442263	5.30059458	6.19607391	1359	2015	3492
1950	NM_001178130,NM_001178131	EGF	epidermal growth factor	-3.103434692	2.089032424	2.567986	0.82619	5.077106	3.9320442	2.48395571	4.94489092	560	241	1439
2316	NM_001110556,NM_001456	FLNA	filamin A	2.128750061	2.088881161	55.941481	119.311405	109.872516	8.85818228	9.94654399	9.91929587	18180	51709	46708
151742	NM_001317911,NM_001317912	PPM1L	protein phosphatase, Mg2+/Mn2+	-1.564726078	2.0886531	1.22331	0.781459	2.406453	3.8142098	3.22493445	4.82246845	513	438	1318

9911	NM_001242925,NM_001297611	TMCC2	transmembrane and coiled-coil d	1.264536583	2.087798654	0.274354	0.346161	0.536475	1.04948198	1.23481109	1.69448139	42	71	108
54443	NM_001284301,NM_001284302	ANLN	anillin actin binding protein	-1.164167364	2.087629937	78.310388	67.848907	154.737061	8.51188338	8.29323182	9.57169473	14292	16403	36697
57669	NM_001184937,NM_001184938	EPB41L5	erythrocyte membrane protein ba	1.079713374	2.087219352	3.38102	3.698911	6.674459	4.42141376	4.52709643	5.44759453	802	1157	2059
987	NM_001199282,NM_006726	LRBA	LPS responsive beige-like ancho	1.093599852	2.085901935	5.821182	6.372259	11.40883	5.8357435	5.96267072	6.88324207	2203	3219	5652
91875	NM_138376	TTC5	tetratricopeptide repeat domain 5	1.43270422	2.085068681	3.763396	5.405391	7.386845	2.9730102	3.43531911	3.93446197	269	515	690
147807	NM_153219	ZNF524	zinc finger protein 524	-1.152671867	2.085027825	7.090999	6.166905	13.927839	3.14487866	2.96451939	4.11772436	308	357	790
56978	NM_001099403,NM_020226	PRDM8	PR/SET domain 8	-1.101276421	2.084027008	15.19667	13.805374	29.327202	5.73654145	5.60009515	6.78181217	2054	2492	5265
9950	NM_005113	GOLGA5	golgin A5	1.276524557	2.0839793	15.542923	19.862545	30.440668	5.5205971	5.86601302	6.56354499	1763	3007	4519
10014	NM_001015053,NM_005474	HDAC5	histone deacetylase 5	1.160725043	2.083601736	4.099041	4.764586	8.02562	4.47692439	4.68297626	5.50200967	835	1295	2140
127343	NM_147192,NM_172225	DMBX1	diencephalon/mesencephalon ho	-2.289819921	2.079275497	4.251533	1.858576	8.318935	3.6915876	2.63327382	4.68868621	468	273	1197
4594	NM_000255	MUT	methylmalonyl-CoA mutase	1.110693814	2.078925744	3.588255	3.988485	7.009893	3.86553291	4.00712306	4.86921928	533	791	1363
128312	NM_175055	HIST3H2BB	histone cluster 3 H2B family men	1.682572788	2.077211596	3.005406	5.036515	5.861362	1.21691895	1.69172362	1.90855849	52	117	133
2523	NM_000148,NM_001329877	FUT1	fucosyltransferase 1 (H blood grc	-1.298296193	2.076629542	2.543497	1.957529	4.963018	3.52601466	3.18617855	4.51401614	413	425	1055
7045	NM_000358	TGFBI	transforming growth factor beta ir	1.930728133	2.076295407	84.364143	163.070618	164.61441	7.85431764	8.80046242	8.90510328	9046	23336	23101
9754	NM_001142503,NM_001142504	STARD8	StAR related lipid transfer domair	-1.693977173	2.076256613	0.723964	0.43104	1.413214	2.16656821	1.61299082	3.04408938	137	108	350
84301	NM_032341	DDI2	DNA damage inducible 1 homolo	1.647156022	2.074201338	1.299137	2.153301	2.549716	2.14180175	2.7276415	3.01481644	134	295	342
81693	NM_030943	AMN	amnion associated transmembra	-2.080069328	2.074201338	2.244098	1.080427	4.382058	2.14180175	1.40024122	3.01481644	134	86	342
400578	NR_029392	KRT16P2	keratin 16 pseudogene 2	-1.037827737	2.073766159	4.215489	4.064299	8.222257	2.90104285	2.85474367	3.84996236	254	327	648
23584	NM_001329920,NM_014312	VSIG2	V-set and immunoglobulin domai	-2.190036526	2.073611125	16.099867	7.29017	31.638235	4.31281697	3.26553914	5.32698995	741	452	1890
692235	NR_033295,NR_033295_dup1	SNORD103B	small nucleolar RNA, C/D box 10	2.121242749	2.073509172	2.074815	4.385242	4.027837	0.23670415	0.46599441	0.45708734	7	20	18
101929402	NR_126027	EXTL3-AS1	EXTL3 antisense RNA 1	1.700005729	2.073509172	0.201826	0.388127	0.450611	0.23670415	0.38406669	0.45708734	7	16	18
114569	NM_052886	MAL2	mal, T-cell differentiation protein	-1.511293059	2.073061275	272.702271	180.640121	531.250793	9.54972405	8.9549218	10.60049291	29385	25979	74924
3726	NM_002229	JUNB	JunB proto-oncogene, AP-1 trans	1.165551218	2.072234539	58.803658	68.607376	114.506973	6.71387535	6.93293968	7.7579542	4082	6357	10404
9414	NM_001170414,NM_001170415	TJP2	tight junction protein 2	-1.001360827	2.071778592	40.611856	39.132802	78.066292	7.45513887	7.45318811	8.50176141	6850	9140	17455
1371	NM_000097	CPOX	coproporphyrinogen oxidase	3.059526806	2.071120531	8.531871	26.146061	16.609802	4.55559896	6.12712406	5.57402156	884	3614	2252
51063	NM_015916,NR_024552,NR_04(	CALHM2	calcium homeostasis modulator 2	1.872345597	2.070371806	3.021748	5.749302	5.975675	2.73986043	3.54069782	3.67388275	223	558	568
80329	NM_001317089,NM_025218,NR	ULBP1	UL16 binding protein 1	1.96206654	2.070280052	0.231037	0.453534	0.449284	1.01352966	1.58576189	1.63800401	40	105	102
23266	NM_001297704,NM_001297705	ADGRL2	adhesion G protein-coupled rece	-1.550108012	2.070224101	0.565863	0.36233	1.10014	2.33625658	1.85267538	3.23070557	159	137	405
347734	NM_001286509,NM_001286510	SLC35B2	solute carrier family 35 member E	1.456149117	2.069335628	2.394844	3.655986	4.746582	2.51403512	2.97505117	3.42681271	185	360	471
3868	NM_005557	KRT16	keratin 16	-2.734888535	2.069272748	5.863684	2.14342	11.3994	3.43381096	2.19658485	4.41245104	385	188	980
604	NM_001130845,NM_001134738	BCL6	B-cell CLL/lymphoma 6	2.32391487	2.069136476	4.382861	10.497465	8.521348	4.02074421	5.18589965	5.02323609	598	1857	1522
5836	NM_001163940,NM_002863	PYGL	glycogen phosphorylase L	1.133955518	2.068864101	38.338157	43.705476	74.76547	6.7443565	6.92413441	7.78624649	4170	6318	10611
1120	NM_005198	CHKB	choline kinase beta	1.770884863	2.067906042	11.040123	19.578117	21.463673	4.21133328	5.00159078	5.21883106	688	1628	1750
100129550	NR_024618	LINC02035	long intergenic non-protein codin	1.597363036	2.067814921	0.854897	1.367272	1.660392	2.56460348	3.14635273	3.48133381	193	412	491
100288842	NR_027442	LOC100288842	UDP-GlcNAc:betaGal beta-1,3-N	-1.77411733	2.067297023	0.875256	0.476034	1.744471	0.59399636	0.36283659	1.03996283	20	15	51
644172	NR_026901	MAPK8IP1P2	mitogen-activated protein kinase	1.269197604	2.065962723	0.477213	0.61823	0.951062	0.88013425	1.04792463	1.45395569	33	56	84
771	NM_001218,NM_001293642,NM	CA12	carbonic anhydrase 12	21.46130288	2.064536078	0.032539	1.208805	0.071383	0.29776659	2.57907432	0.56199967	9	261	23
7012	NR_001566	TERC	telomerase RNA component	5.107046715	2.064536078	0.497369	2.686503	0.988202	0.29776659	1.12560759	0.56199967	9	62	23
10903	NM_001145862,NM_181873	MTMR11	myotubularin related protein 11	-1.175449693	2.06411335	8.254924	7.144939	16.028338	4.58038655	4.3576867	5.59457146	900	1023	2285
51092	NM_001040455	SIDT2	SID1 transmembrane family men	-1.283048096	2.063820315	4.843603	3.779321	9.397442	4.28481203	3.94597954	5.29157606	726	756	1843
81786	NM_033342,NM_203293,NM_20	TRIM7	tripartite motif containing 7	-1.386307543	2.062612515	7.011646	5.312617	12.666849	3.97707578	3.54069782	4.97370184	579	558	1469
421	NM_001670	ARVCF	armadillo repeat gene deleted in	-1.359536315	2.060298388	1.111176	0.816912	2.158799	2.4278825	2.07783221	3.32614297	172	169	436
9444	NM_001301085,NM_006775,NM	QKI	QKI, KH domain containing RNA	1.351185713	2.058898316	3.12402	4.102278	5.946902	4.96505491	5.38725856	5.98304325	1187	2143	3006
100188954	NR_024130	DNMBP-AS1	DNMBP antisense RNA 1	1.6268495	2.058793007	0.099588	0.175152	0.209482	0.35634914	0.54351856	0.65979727	11	24	28
100287177	NR_130728,NR_130729	EML2-AS1	EML2 antisense RNA 1	-1.93403858	2.058617246	1.391278	0.721457	2.721427	1.30878272	0.81765451	2.01587867	58	40	147
5226	NM_001304451,NM_001304452	PGD	phosphogluconate dehydrogenas	-1.005950917	2.057162458	35.679579	35.950414	69.253516	6.36333314	6.35487722	7.39498132	3193	4241	8079
128338	NM_001349881,NM_001349882	DRAM2	DNA damage regulated autophag	1.146638927	2.056673928	15.259328	17.445861	29.081447	5.05180751	5.24366088	6.0696643	1263	1935	3195
7789	NM_007156	ZXDA	zinc finger, X-linked, duplicated A	1.153373013	2.055844216	0.850534	0.985593	1.647898	2.41415945	2.58366954	3.30815729	170	262	430
102466723	NR_106792	MIR6734	microRNA 6734	1.093099346	2.054801926	4.734779	5.288556	9.560491	0.41264541	0.44594451	0.75138414	13	19	33
27248	NM_001127397,NM_001127398	ERLEC1	endoplasmic reticulum lectin 1	1.10772633	2.054621311	18.433556	20.541937	35.614285	5.55461405	5.69923552	6.57768983	1806	2673	4564
22949	NM_001146108,NM_001146109	PTGR1	prostaglandin reductase 1	1.20331609	2.054383067	14.345715	17.903587	28.873511	4.31466478	4.56941219	5.31578181	742	1193	1875
8736	NM_003803,NM_019856	MYOM1	myomesin 1	1.210041678	2.053068155	0.149025	0.183695	0.292471	0.89996265	1.03456085	1.47560397	34	55	86
83595	NM_031439	SOX7	SRY-box 7	6.717527666	2.05300629	0.582682	3.976386	1.134716	1.5285023	3.77370073	2.28470378	74	665	187
23089	NM_001040152,NM_001172437	PEG10	paternally expressed 10	-3.228539923	2.052390596	67.610428	20.950925	130.408952	8.77069598	7.0871367	9.8063114	17108	7080	43186
7077	NM_003255	TIMP2	TIMP metallopeptidase inhibitor 2	-1.110401829	2.052126735	139.328522	125.613113	268.687958	8.95658115	8.80581898	9.99221566	19466	23423	49132
7049	NM_001195683,NM_001195684	TGFBR3	transforming growth factor beta r	-1.233585455	2.052124904	1.054953	0.854654	2.035841	2.9541672	2.69403583	3.89283072	265	287	669
163	NM_001030006,NM_001282	AP2B1	adaptor related protein complex 2	-1.010994255	2.051935465	31.809835	31.530649	61.499734	7.48293069	7.46724431	8.51578755	6984	9230	17626
56109	NM_018919,NM_032086	PCDHGA6	protocadherin gamma subfamily	-1.054423872	2.051867237	0.151199	0.147172	0.164041	0.46682716	0.44594451	0.83750224	15	19	38
54877	NM_017742,NR_126534	ZCCHC2	zinc finger CCHC-type containing	-1.127680472	2.049875819	1.221581	1.084625	2.467841	3.00540462	2.85474367	3.94613924	276	327	696
55151	NM_018112	TMEM38B	transmembrane protein 38B	1.099308171	2.048701128	5.917747	6.514565	11.397555	4.42141376	4.55193121	5.42134148	802	1178	2021
199731	NM_145296	CADM4	cell adhesion molecule 4	-1.646412701	2.048664339	6.309865	3.836647	12.144243	3.84522223	3.18919702	4.82773844	525	426	1323
154091	NM_145176	SLC2A12	solute carrier family 2 member 12	-1.867295385	2.048122895	1.312137	0.70358	2.52662	3.0281061	2.27254712	3.96921401	281	201	708
549	NM_001306190,NM_001351431	AUH	AU RNA binding methylglutacony	-1.023633045	2.047905498	4.575338	4.320128	8.855334	3.04601354	3.01642592	3.98816501	285	372	718
6590	NM_003064	SLPI	secretory leukocyte peptidase inh	-2.112448077	2.047017842	194.305389	92.079185	373.776184	6.82985711	5.76494251	7.85689819	4427	2800	11146
10226	NM_001164189,NM_001164194	PLIN3	perilipin 3	1.248017145	2.046542023	21.828177	27.270635	41.936389	5.66229636	5.97627719	6.68088498	1949	3250	4906
677832	NR_003015	SNORA53	small nucleolar RNA, H/ACA box	1.49456776	2.045207533	2.377827	3.538383	4.604821	0.66524311	0.90836632	1.13825079	23	46	58
10123	NM_001282431,NM_005737	ARL4C	ADP ribosylation factor like GTPa	-1.034684613	2.044823247	22.061555	21.363418	42.09945	6.43473679	6.38612247	7.45819071	3357	4335	8443
1781	NM_001271785,NM_001271786	DYNC1I2	dynein cytoplasmic 1 intermediat	1.422548749	2.044017827	14.964438	21.308162	28.744914	6.008364	6.5101878	7.02831186	2488	4729	6255
152559	NM_001040202,NM_001350105	PAQR3	progestin and adipoQ receptor fa	-5.403117639	2.04355738	3.451269	0.624838	6.651643	3.80635139	1.76638114	4.78394543	510	126	1282
100128385	NR_024376	FAM225B	family with sequence similarity 22	1.394170531	2.043174223	0.296609	0.413756	0.568436	1.42282133	1.74192207	2.14982678	66	123	166
7421	NM_000376,NM_001017535,NM	VDR	vitamin D (1,25- dihydroxyvitamir	2.647794584	2.042637007	1.304184	3.454442	2.497504	2.78853894	4.05754526	3.70857641	232	821	583
115019	NM_052934,NM_134325	SLC26A9	solute carrier family 26 member 9	-2.644187277	2.042561437	0.601839	0.227997	1.158677	1.93632435	1.04792463	2.76083766	111	56	279
525	NM_001692	ATP6V1B1	ATPase H+ transporting V1 subu	-1.366301152	2.042283419	6.078146	4.454527	11.672876	3.64240927	3.23373217	4.61256748	451	441	1133
55321	NM_001304748,NM_001304749	TMEM74B	transmembrane protein 74B	1.122495615	2.040917172	1.255524	1.425499	2.48234	1.65056839	1.76638114	2.42483178	84	126	211
80321	NM_001288964,NM_001288965	CEP70	centrosomal protein 70	1.282121543	2.040720906	8.715738	11.37381	15.97215	4.30911424	4.65158753	5.30070087	739	1266	1855
481	NM_001677	ATP1B1	ATPase Na+/K+ transporting sub	1.421465336	2.040577359	66.09946	94.061615	126.754829	7.15576835	7.66015249	8.17959194	5559	10558	13952
50651	NM_001080397	SLC45A1	solute carrier family 45 member 1	1.349383921	2.038315865	0.527039	0.711252	1.009175	1.2010231	1.4614436	1.86820921	51	92	128
55625	NM_001145548,NM_017740	ZDHHC7	zinc finger DHHC-type containing	-1.370287346	2.036390621	35.216804	25.700619	67.517897	6.78491031	6.33525512	7.80426985	4290	4183	10745
100996335	NR_136559	LOC100996335	uncharacterized LOC100996335	2.666858399	2.035560872	0.905111	2.435784	1.742146	1.81624731	2.95035633	2.6172769	99	353	248
23357	NM_015305	ANGEL1	angel homolog 1	1.815025387	2.034439416	3.870469	7.040723	7.406727	4.04543465	4.86575665	5.02507241	609	1477	1524
5025	NM_001256796,NM_001261397	P2RX4	purinergic receptor P2X 4	-1.118644278	2.033370668	5.032341	4.485311	9.712211	3.45407101	3.30781222	4.40964229	391	467	978
26960	NM_001204197,NM_015678	NBEA	neurobeachin	1.770836578	2.031984499	0.423437	0.750803	0.80762	2.48150547	3.18919702	3.36725759	180	426	450
65108	NM_023009,NR_052852	MARCKSL1	MARCKS like 1	2.444000338	2.031155174	15.184325	37.352926	29.100789	4.59109862	5.84463554	5.58277547	907	2962	2266
6699	NM_003125	SPRR1B	small proline rich protein 1B	-1.003350453	2.030004142	8.368646	8.385062	16.019323	2.60139951	2.597368	3.4972963	199	265	497
196463	NM_001159727,NM_173542	PLBD2	phospholipase B domain containi	1.663226967	2.029632149	2.873944	4.784197	5.457338	3.15316384	3.82117143	4.08992214	310	689	774
1047	NM_001130675,NM_004362	CLGN	calmegin	4.179889721	2.028782667	0.276279	1.163077	0.531086	0.81895953	2.07130288	1.35230545	30	168	75
3292	NM_000413,NM_001330219,NR	HSD17B1	hydroxysteroid 17-beta dehydrog	-1.568829817	2.028314773	1.920424	1.258291	3.723541	2.02927762	1.5673195	2.85881867	121	103	302
4124	NM_002372	MAN2A1	mannosidase alpha class 2A mer	-1.217191624	2.028149481	9.108207	7.491192	17.358713	6.00207091	5.72338199	7.01081128	2477	2719	6179
55129	NM_001204831,NM_001204832	ANO10	anoctamin 10	1.153280398	2.027869506	3.363098	4.053211	6.607175	3.3677101	3.55480842	4.31524723	366	564	913
135112	NM_001122842,NM_001199619	NCOA7	nuclear receptor coactivator 7	1.408413132	2.027462544	3.962157	4.815451	7.150557	4.13809052	4.60826865	5.11575465	652	1227	1626
286103	NR_026974	ZNF252P-AS1	ZNF252P antisense RNA 1	-1.049557354	2.027391722	0.175536	0.166487	0.339604	0.64188297	0.61708849	1.09694451	22	28	55
158586	NM_007157	ZXDB	zinc finger, X-linked, duplicated E	1.473814035	2.026530627	2.865592	4.233267	5.465238	4.06751962	4.59921966	5.04240188	619	1219	1543
23499	NM_012090	MACF1	microtubule-actin crosslinking fac	-1.194453399	2.026042266	16.733978	14.025418	31.859968	8.17595161	7.9205681	9.1920946	11315	12657	28196
6934	NM_001146274,NM_001146283	TCF7L2	transcription factor 7 like 2	3.105004626	2.025924334	3.676528	11.406654	7.086256	3.93924856	5.50881622	4.90954071	563	2336	1403
10079	NM_006045	ATP9A	ATPase phospholipid transportin	-1.010458482	2.025609384	3.830811	3.797735	7.295703	4.88025587	4.86575665	5.87366563	1117	1477	2783
54491	NM_019018	FAM105A	family with sequence similarity 10	1.772554585	2.025483514	0.268725	0.479847	0.513937	1.51570785	2.10365798	2.2537152	73	173	182
79807	NM_001031720,NM_024751	GSTCD	glutathione S-transferase C-term	1.283205396	2.024939791	2.832865	3.58306	5.303019	3.60363765	3.9370293	4.56033921	438	751	1091
1718	NM_014762	DHCR24	24-dehydrocholesterol reductase	1.757044719	2.02379667	31.266905	55.000053	59.469074	7.03879266	7.84722131	8.0503117	5123	12027	12752
8938	NM_001199096,NM_001199097	BAIAP3	BAI1 associated protein 3	-1.193102421	2.023445288	7.885578	6.619076	15.025828	5.18100252	4.9339228	6.17761851	1385	1551	3447

55074	NM_001198532,NM_001198533	OXR1	oxidation resistance 1	-1.008897089	2.023131684	10.87898	12.133274	21.429381	5.59090027	5.57838681	6.59231674	1853	2454	4611
100132994	NM_001145139,NM_001145139	CXorf49B	chromosome X open reading fran	1.307022287	2.02306916	0.164977	0.216952	0.322272	0.38477185	0.48576948	0.69713258	12	21	30
8334	NM_003512	HIST1H2AC	histone cluster 1 H2A family men	1.800028568	2.022963339	4.555432	8.280325	8.723345	1.78459878	2.43393638	2.57277436	96	231	239
29887	NM_001199835,NM_001199837	SNX10	sorting nexin 10	1.49671615	2.022952293	3.52362	5.262272	6.667821	3.30216484	3.83472784	4.24292715	348	696	866
23376	NM_015323	UFL1	UFM1 specific ligase 1	1.1537459	2.022829802	7.559053	8.728041	14.368271	4.99762645	5.19793813	5.99105095	1215	1873	3023
154214	NM_001286398,NM_152553,NR	RNF217	ring finger protein 217	1.307342833	2.022181854	1.096587	1.446563	2.112799	3.71975182	4.08048581	4.67938813	478	835	1189
8542	NM_001136540,NM_001136541	APOL1	apolipoprotein L1	1.559547397	2.021786132	14.784612	23.114467	28.094435	5.40393602	6.03281945	6.40229181	1623	3382	4036
10585	NM_001077365,NM_001077366	POMT1	protein O-mannosyltransferase 1	-1.210299091	2.021286238	9.772219	8.233251	19.258565	4.9720967	4.706341	5.96402441	1193	1317	2966
5195	NM_004565	PEX14	peroxisomal biogenesis factor 14	1.452093551	2.018746702	13.630487	19.824377	25.871761	4.73188879	5.25306686	5.71776698	1004	1948	2493
26009	NM_001308237,NM_015534	ZZZ3	zinc finger ZZ-type containing 3	1.044979406	2.017946041	4.687533	5.222296	9.005418	4.80732269	4.8685837	5.79405678	1060	1480	2631
23646	NM_001031696,NM_001291311	PLD3	phospholipase D family member	1.232327101	2.017399992	31.156126	40.966927	58.854135	6.16815189	6.46576027	7.17052495	2784	4584	6908
374928	NM_001304334,NM_001304335	ZNF773	zinc finger protein 773	1.640563297	2.017245629	6.830805	11.319914	13.166695	3.8730763	4.54840945	4.83508422	536	1175	1330
284422	NM_001136503	SMIM24	small integral membrane protein	1.06573891	2.016853748	0.938244	0.993248	1.788107	0.88013425	0.92294555	1.43197761	33	47	82
53349	NM_001281734,NM_001281735	ZFYVE1	zinc finger FYVE-type containing	1.313883715	2.016663994	2.858513	3.759196	5.585425	3.7555621	4.12372395	4.71281235	491	862	1218
137075	NM_194284	CLDN23	claudin 23	-1.142219928	2.016408375	5.132547	4.498335	9.71806	3.42699388	3.25405394	4.36973462	383	448	950
3980	NM_002311,NM_013975	LIG3	DNA ligase 3	1.00132144	2.015820768	5.676019	5.810428	11.057131	4.42654937	4.42836625	5.40380548	805	1077	1996
102724827	NR_110543	MIATNB	MIAT neighbor (non-protein codir	1.006491339	2.01468647	0.440457	0.451347	0.851869	0.79797761	0.80196545	1.31676564	29	39	72
217	NM_000690,NM_001204889	ALDH2	aldehyde dehydrogenase 2 famil	-4.945039118	2.014670204	3.518306	0.712615	6.684867	3.01452812	1.28081328	3.93250653	278	75	689
80054	NR_026887	CEBPA-AS1	CEBPA antisense RNA 1 (head t	-1.002055038	2.01368638	0.071316	0.069924	0.130968	0.20517559	0.20477631	0.39027104	6	8	15
102724708	NR_136223,NR_136224	LOC102724708	uncharacterized LOC102724708	2.591141254	2.01368638	0.067817	0.19957	0.146757	0.20517559	0.48576948	0.39027104	6	21	15
56135	NM_018898,NM_031882	PCDHAC1	protocadherin alpha subfamily C,	1.13597264	2.013363018	0.2505	0.287157	0.476568	0.7550755	0.83317479	1.25551499	27	41	67
85377	NM_033386	MICALL1	MICAL like 1	1.226090453	2.012675767	10.226578	12.55717	19.349138	5.51658908	5.80484733	6.50980517	1758	2880	4352
11057	NM_007011,NM_152924	ABHD2	abhydrolase domain containing 2	-1.024579149	2.012357657	11.185861	10.929382	21.157049	6.58736588	6.55270215	7.58870688	3736	4872	9247
39	NM_001303253,NM_005891	ACAT2	acetyl-CoA acetyltransferase 2	1.014905573	2.012216377	18.546966	18.856755	35.098312	4.86015285	4.88077062	5.84380456	1101	1493	2725
9685	NM_001195555,NM_001195556	CLINT1	clathrin interactor 1	1.307533149	2.011982897	9.418633	12.348436	17.824391	5.23858851	5.61644502	6.2279131	1443	2521	3571
22901	NM_001267727,NM_014960	ARSG	arylsulfatase G	1.095799823	2.011918343	0.789975	0.864775	1.50046	1.65056839	1.74192207	2.40804223	84	123	208
1773	NM_001351825,NM_005223	DNASE1	deoxyribonuclease 1	1.422424235	2.010472139	2.39743	3.14671	4.641606	2.93987166	3.39143785	3.84996236	262	498	648
284207	NM_001004431	METRNL	meteorin like, glial cell differentia	-1.717008071	2.010358809	34.735691	20.248014	65.643135	5.79529011	5.03385092	6.78966501	2141	1666	5294
25829	NM_001195071,NM_001195072	TMEM184B	transmembrane protein 184B	1.519234215	2.010341562	16.950418	25.780016	32.016667	5.92130285	6.51651019	6.91676387	2340	4750	5786
84071	NM_001286609,NM_032131	ARMC2	armadillo repeat containing 2	1.332618142	2.010028967	0.154592	0.214039	0.306295	0.66524311	0.83317479	1.12461302	23	41	57
8994	NM_014240	LIMD1	LIM domains containing 1	1.443481105	2.00990577	3.445321	4.98092	6.509797	4.46532657	4.97472832	5.43935559	828	1597	2047
100130827	NM_001199824	SBK3	SH3 domain binding kinase famil	1.196968154	2.009471666	0.805853	0.987025	1.547229	1.01352966	1.15059938	1.60891398	40	64	99
9451	NM_001313915,NM_004836	EIF2AK3	eukaryotic translation initiation fa	1.232552263	2.009239207	2.371323	2.927444	4.482376	3.55444243	3.83279899	4.49823858	422	695	1043
100132406	NM_001039703,NM_001302371	NBPF10	NBPF member 10	1.155979774	2.008772882	1.180122	1.366986	2.230702	3.82202563	4.01734947	4.7763339	516	797	1275
1956	NM_001346897,NM_001346898	EGFR	epidermal growth factor receptor	-1.027147522	2.008472866	13.023065	12.055077	25.678224	6.20099052	6.16287794	7.19723739	2849	3706	7038
11338	NM_001012478,NM_007279	U2AF2	U2 small nuclear RNA auxiliary fa	1.264165184	2.008439311	47.179539	59.736553	89.087122	7.17522211	7.51132541	8.17629065	5635	9518	13920
966	NM_000611,NM_001127223,NM	CD59	CD59 molecule (CD59 blood gro	-1.767589804	2.007464328	28.500983	16.182596	53.939017	7.74053668	6.92390793	8.74253125	8357	6317	20634
90102	NM_001134437,NM_001134438	PHLDB2	pleckstrin homology like domain	1.226227788	2.006818537	17.328653	21.214905	33.098233	6.69236199	6.98402008	7.69027653	4021	6588	9925
83874	NM_001204240,NM_031937	TBC1D10A	TBC1 domain family member 10	1.934818205	2.005718163	21.817979	42.261115	41.11983	5.44078168	6.37688593	6.42819689	1666	4307	4110
253018	NR_026791	HCG27	HLA complex group 27 (non-prot	1.244440663	2.004588321	2.704603	3.369213	5.095545	2.46828542	2.73178769	3.33505242	178	296	439
4759	NM_005383	NEU2	neuraminidase 2	2.577321332	2.004474139	0.0744	0.23975	0.173526	0.13997292	0.3412894	0.27153692	4	14	10
100422860	NR_036251	MIR4292	microRNA 4292	1.666155364	2.004474139	1.438155	2.60988	2.94197	0.13997292	0.2284445	0.27153692	4	9	10
10125	NM_001128602,NM_001306086	RASGRP1	RAS guanyl releasing protein 1	2.941787718	2.004474139	0.020516	0.061834	0.038998	0.13997292	0.38406669	0.27153692	4	16	10
2735	NM_001160045,NM_001167609	GLI1	GLI family zinc finger 1	2.030621751	2.004474139	0.027011	0.054419	0.051092	0.13997292	0.27464693	0.27153692	4	11	10
81872	NM_001123387	KRTAP2-1	keratin associated protein 2-1	-1.324521912	2.004474139	0.117928	0.099282	0.2543	0.13997292	0.10601836	0.27153692	4	4	10
338699	NM_001300972,NM_001300973	ANKRD42	ankyrin repeat domain 42	1.932935669	2.002399465	1.418254	2.490952	2.420431	2.55837852	3.38618649	3.43235848	192	496	473
59352	NM_001017403,NM_001017404	LGR6	leucine rich repeat containing G	-2.867531651	2.001584573	0.422541	0.1471	0.840147	1.27880673	0.58077243	1.94780992	56	26	138
57136	NM_020531	APMAP	adipocyte plasma membrane ass	1.951630722	2.001297869	48.032063	93.846092	90.331116	6.71247443	7.67044931	7.70653074	4078	10634	10038
3162	NM_002133	HMOX1	heme oxygenase 1	2.258475321	2.000573968	19.463669	44.006672	36.591881	4.96034122	6.109706	5.93745724	1183	3570	2911
4494	NM_001301272,NM_005949	MT1F	metallothionein 1F	-1.864391547	1.999587418	1.607158	0.829738	3.035125	0.77668602	0.46599441	1.28032816	28	20	69
100129917	NR_036511,NR_036512	LOC100129917	uncharacterized LOC100129917	1.714653358	1.999394436	1.355754	2.845506	2.889797	1.70793493	2.2895231	2.46867149	89	204	219
2706	NM_004004	GJB2	gap junction protein beta 2	2.757248931	1.998232023	2.413064	6.689194	4.544739	2.70083015	4.01565009	3.58455875	216	796	531
124583	NM_001159772,NM_001159773	CANT1	calcium activated nucleotidase 1	-1.054509647	1.997698162	35.750239	33.498021	66.774809	6.89922686	6.82331145	7.89153384	4647	5888	11418
8453	NM_001198777,NM_001198778	CUL2	cullin 2	1.432182147	1.997424031	7.940096	11.387215	14.914725	5.0759764	5.5812619	6.05266115	1285	2459	3157
8140	NM_003486	SLC7A5	solute carrier family 7 member 5	-1.061671715	1.996785954	112.063889	105.671684	210.282074	8.95997951	8.87381994	9.95621643	19512	24556	47920
29903	NM_013301	CCDC106	coiled-coil domain containing 106	2.839233436	1.996021492	1.346475	3.8402	2.530424	1.8573935	3.07960182	2.64141996	103	391	253
90809	NM_001100814,NM_144568	TMEM55B	transmembrane protein 55B	1.049019362	1.994369021	15.751135	16.573933	29.550809	4.86898241	4.93572116	5.84015848	1108	1553	2718
50999	NM_001167830,NM_016040,NR	TMED5	transmembrane p24 trafficking pr	1.093670979	1.994212074	11.671677	12.784334	21.874861	6.13088238	6.25830165	7.11643039	2712	3963	6652
101929613	NR_110947,NR_110948,NR_11(	LOC101929613	uncharacterized LOC101929613	-1.45463322	1.994106381	1.459907	0.997668	3.844307	0.68823103	0.50527715	1.15176086	24	22	59
79365	NM_030762	BHLHE41	basic helix-loop-helix family mem	-1.088127678	1.993615759	31.241917	28.744167	58.540222	6.85800652	6.7372513	7.84719983	4515	5544	11071
9709	NM_001010989,NM_001272103	HERPUD1	homocysteine inducible ER prote	1.172214735	1.993098527	38.647626	45.293486	72.239996	6.19197132	6.41831407	7.17714635	2831	4434	6940
2081	NM_001433	ERN1	endoplasmic reticulum to nucleus	1.439642678	1.992854195	1.281914	1.853994	2.405925	2.58923814	3.04002433	3.45977198	197	379	483
4151	NM_005368,NM_203377,NM_20	MB	myoglobin	1.846488106	1.991396173	41.413693	72.779824	73.235396	5.5004447	6.37058443	6.47831697	1738	4288	4257
6576	NM_001256534,NM_001287387	SLC25A1	solute carrier family 25 member	1.301771125	1.990343415	21.252483	27.473581	39.437881	5.13363806	5.50458181	6.10612025	1339	2329	3278
57648	NM_001198972,NM_001198973	KIAA1522	KIAA1522	1.241329456	1.989334824	47.935575	59.135099	88.899325	7.96331541	8.27408027	8.95273205	9759	16186	23878
5165	NM_001142386,NM_005391	PDK3	pyruvate dehydrogenase kinase 3	-1.142886503	1.989293898	1.178251	0.767749	2.055465	3.04155747	2.87362954	3.94419958	284	332	695
153222	NM_001168393,NM_001168394	CREBRF	CREB3 regulatory factor	1.108746117	1.989110748	3.741834	3.728352	6.391555	4.52080535	4.66357413	5.48142583	862	1277	2109
6510	NM_001145144,NM_001145145	SLC1A5	solute carrier family 1 member 5	1.046751319	1.987917672	89.641756	93.961594	166.813824	7.9659633	8.031625	8.95435916	9777	13674	23905
9582	NM_001270411,NM_004900	APOBEC3B	apolipoprotein B mRNA editing e	1.466125133	1.987901132	26.977767	39.536663	50.385439	5.36968406	5.91058637	6.3435348	1584	3103	3873
1512	NM_001319137,NM_004390	CTSH	cathepsin H	1.998102103	1.987523931	65.254223	130.307414	122.527525	6.60710179	7.59834076	7.59072316	3788	10113	9260
3996	NM_004140	LLGL1	LLGL1, scribble cell polarity com	1.561479381	1.98739699	2.069298	3.242815	3.874817	3.25290323	3.84049897	4.16678956	335	699	819
202333	NM_153610	CMYA5	cardiomyopathy associated 5	1.955291722	1.987256346	0.204141	0.399949	0.382529	1.83696709	2.59281628	2.61239943	101	264	247
83983	NM_032037	TSSK6	testis specific serine kinase 6	-2.10892044	1.986923399	0.683841	0.31332	1.275669	0.97665845	0.54351856	1.5489128	38	24	93
64121	NM_001271851,NM_022157	RRAGC	Ras related GTP binding C	1.092370033	1.986705027	4.851673	5.332175	9.06135	3.79844995	3.91714113	4.73654166	507	740	1239
84448	NM_001130083,NM_001130084	ABLIM2	actin binding LIM protein family n	-1.567138519	1.986445151	0.136626	0.093931	0.30487	0.59399636	0.40498891	1.01060517	20	17	49
692093	NR_003050,NR_003050_dup1	SNORD62B	small nucleolar RNA, C/D box 62	2.435456801	1.985625009	3.17039	8.166646	6.176454	0.35634914	0.75384785	0.64076093	11	36	27
1829	NM_001943	DSG2	desmoglein 2	1.141024482	1.984850359	26.597584	30.382111	49.60841	7.25725678	7.44642371	8.2416137	5967	9097	14567
10848	NM_001142502,NM_006663	PPP1R13L	protein phosphatase 1 regulatory	-1.244546785	1.983804813	23.470209	18.877133	43.755282	6.17630487	5.86554139	7.15467624	2800	3006	6832
2625	NM_001002295,NM_002051	GATA3	GATA binding protein 3	-1.192386083	1.983555801	12.868897	10.802623	23.985048	5.30236925	5.05551521	6.27226751	1510	1692	3684
112	NM_015270	ADCY6	adenylate cyclase 6	-1.070127776	1.982754465	19.367516	18.118479	36.091351	6.85895655	6.76204243	7.8403068	4518	5641	11018
23224	NM_015180,NM_182910,NM_18	SYNE2	spectrin repeat containing nuclea	-1.17584173	1.980307128	9.418816	8.860761	10.59892	5.8344561	5.6051891	6.80764596	2201	2501	5361
254427	NM_153256	PROSER2	proline and serine rich 2	1.180989931	1.979817945	20.436861	24.163792	38.026508	6.07310824	6.3098262	7.04786245	2604	4109	6341
388581	NM_001014980	C1QTNF12	C1q and TNF related 12	1.029032639	1.979523758	1.180378	1.230289	2.227538	1.15225689	1.1751656	1.77525664	48	66	117
2822	NM_001503	GPLD1	glycosylphosphatidylinositol spec	1.137033775	1.979451915	0.111701	0.12732	0.205946	0.71085839	0.7861039	1.17840727	25	38	61
56980	NM_020228,NM_199437,NM_19	PRDM10	PR/SET domain 10	-1.498794131	1.979133736	3.63533	2.426473	6.548861	4.48186648	3.92982892	5.43452772	838	747	2040
26020	NM_001329226,NM_014045	LRP10	LDL receptor related protein 10	1.011026769	1.978508834	21.665305	21.932138	40.882921	7.19817553	7.21388986	8.17773589	5726	7735	13934
9925	NM_014872	ZBTB5	zinc finger and BTB domain conta	-1.458028242	1.978166148	4.167427	2.862164	7.751359	4.31281697	3.80158144	5.26073633	741	679	1803
2131	NM_000127	EXT1	exostosin glycosyltransferase 1	1.655623214	1.978018447	3.662092	6.071795	6.806401	3.70009475	4.38295215	4.62835753	471	1042	1146
441089	NR_003665	CRSP8P	mediator complex subunit 27 pse	3.247686972	1.977815388	0.019208	0.128386	0.070109	0.07168335	0.2284445	0.14214747	2	9	5
102465256	NR_106768	MIR6513	microRNA 6513	-1.313695475	1.977815388	0.573334	0.532795	1.465107	0.07168335	0.05398282	0.14214747	2	2	5
102465515	NR_106915	MIR6856	microRNA 6856	-1.313695475	1.977815388	0.460055	0.431601	1.346926	0.07168335	0.05398282	0.14214747	2	2	5
102465538	NR_106952	MIR6892	microRNA 6892	-1.313695475	1.977815388	0.34236	0.174668	0.868195	0.07168335	0.05398282	0.14214747	2	2	5
1604	NM_000574,NM_001114752,NM	CD55	CD55 molecule (Cromer blood gr	-1.027411274	1.977760806	24.672142	23.922758	45.487171	6.035508	5.99709531	7.00849268	2536	3298	6169
1942	NM_004428,NM_182685	EFNA1	ephrin A1	-1.01570663	1.977310608	10.731039	10.630032	19.833853	4.10002583	4.07885924	5.04149497	634	834	1542
9980	NM_001320714,NM_005128	DOPEY2	dopey family member 2	-1.11829854	1.97634594	1.533826	1.37278	2.849931	3.65995838	3.5120557	4.58544575	457	546	1111
283431	NM_001303130,NM_001303131	GAS2L3	growth arrest specific 2 like 3	-2.115624852	1.975694193	4.7364	2.147906	8.800765	4.75792697	3.73497962	5.71378758	1023	646	2486
2747	NM_012084	GLUD2	glutamate dehydrogenase 2	1.984123428	1.975308574	0.097619	0.199104	0.187267	0.29776659	0.54351856	0.54161868	9	24	22
5175	NM_000442	PECAM1	platelet and endothelial cell adhe	9.462702879	1.975308574	0.052758	0.50199	0.097552	0.29776659	1.67459501	0.54161868	9	115	22
5210	NM_001317134,NM_001317135	PFKFB4	6-phosphofructo-2-kinase/fructos	4.735845465	1.974584687	1.465973	7.316539	2.844256	2.6780437	4.7324405	3.54415067	212	1342	515

91694	NM_001329976,NM_152271,NR	LONRF1	LON peptidase N-terminal domai	-1.164946832	1.97415332	4.179662	3.591737	7.772816	3.94403156	3.73910482	4.87839036	565	648	1372
54836	NM_001317943,NM_001317944	BSPRY	B-box and SPRY domain contain	-1.358781961	1.973356549	14.004472	10.305926	26.022635	5.04514512	4.61838139	6.00414397	1257	1236	3051
54813	NM_001308112,NM_017658	KLHL28	kelch like family member 28	1.27482578	1.973186082	2.346766	2.997558	4.346086	4.00251834	4.33333585	4.93808477	590	1005	1432
10483	NM_001172745,NM_001172746	SEC23B	Sec23 homolog B, coat complex	1.662522867	1.972915719	15.118515	25.537634	28.249845	5.68675843	6.40895836	6.65324904	1983	4405	4812
10365	NM_016270	KLF2	Kruppel like factor 2	1.060596618	1.971808369	9.073567	9.642572	16.824072	4.68551669	4.76719467	5.63721662	971	1376	2355
10139	NM_001134758,NM_001267544	ARFRP1	ADP ribosylation factor related pr	2.644185012	1.97179228	8.115425	21.524967	15.116896	4.41281357	5.77301226	5.35864561	797	2816	1933
9619	NM_004915,NM_016818,NM_20	ABCG1	ATP binding cassette subfamily (	1.025777392	1.971263591	9.903887	10.191597	18.392388	4.89391597	4.92941706	5.84899732	1128	1546	2735
1890	NM_001113755,NM_001113756	TYMP	thymidine phosphorylase	-1.957360166	1.969458748	23.682112	11.84491	43.613154	5.35897928	4.42324784	6.31942	1572	1073	3808
2296	NM_001453	FOXC1	forkhead box C1	1.02086777	1.969237702	1.809853	1.84729	3.354951	2.82529613	2.85093665	3.69940604	239	326	579
51320	NM_016626	MEX3C	mex-3 RNA binding family memb	1.209986389	1.968760191	5.43397	6.583384	10.058969	4.51920371	4.78326433	5.46528578	861	1392	2085
286140	NR_003129_chr6,NR_003129_c	RNF5P1	ring finger protein 5 pseudogene	1.242655869	1.968585585	10.487678	13.049014	19.4116	3.56999691	3.859571	4.48629114	427	709	1034
55093	NM_001283024,NM_001283027	WDYHV1	WDYHV motif containing 1	1.105206954	1.967880049	12.665799	14.014158	22.765296	4.29233333	4.42964302	5.23240543	730	1078	1767
10497	NM_001330653,NM_006377	UNC13B	unc-13 homolog B	-1.103391588	1.967730818	2.825004	2.560708	5.29141	4.22907737	4.09504299	5.16738207	697	844	1687
152519	NM_207330	NIPAL1	NIPA like domain containing 1	1.291027649	1.967649499	2.812754	3.640852	5.209023	3.95828615	4.30578837	4.88851253	571	985	1382
100996437	NR_110037	LOC100996437	uncharacterized LOC100996437	1.174775307	1.966810056	0.415269	0.498931	0.786623	0.43999062	0.50527715	0.76902392	14	22	34
55914	NM_001006600,NM_001253697	ERBIN	erbb2 interacting protein	-1.171976335	1.966172423	32.011521	27.431137	59.180576	7.75948439	7.53168238	8.73160821	8468	9654	20478
246175	NM_001286790,NM_144571	CNOT6L	CCR4-NOT transcription complex	-1.159919715	1.965830936	1.959952	1.691532	3.641129	4.16497021	3.96371513	5.10016049	665	766	1608
699	NM_001278616,NM_001278617	BUB1	BUB1 mitotic checkpoint serine/tl	1.067487806	1.965719985	24.656723	26.369493	45.081505	6.44952833	6.54270679	7.41647691	3392	4838	8201
55959	NM_001161841,NM_018837,NM	SULF2	sulfatase 2	1.035885604	1.964542925	23.712201	24.600716	43.687038	6.6179922	6.66834936	7.58497709	3817	5283	9223
222584	NM_001010872	FAM83B	family with sequence similarity 83	1.151819244	1.962957161	1.717802	1.978115	3.16668	3.51641288	3.70365887	4.42641333	410	631	990
25943	NM_001009984	C20orf194	chromosome 20 open reading fra	2.009232417	1.962682416	2.291663	4.614678	4.231137	4.03874269	5.00073201	4.9679855	606	1627	1463
1629	NM_001918	DBT	dihydrolipoamide branched chain	1.191093549	1.962544488	1.307279	1.559297	2.412484	3.88556162	4.12214544	4.8097414	541	861	1306
157638	NM_174911	FAM84B	family with sequence similarity 84	-1.285620243	1.961998733	75.33815	58.664658	138.903091	8.65476479	8.29331949	9.62533773	15784	16404	38089
100130967	NM_001195032,NM_001302839	C6orf99	chromosome 6 open reading fran	1.401524212	1.960386774	0.629646	0.932318	1.267217	0.68823103	0.89363826	1.13825079	24	45	58
29760	NM_001114094,NM_001258440	BLNK	B-cell linker	1.308385209	1.960386774	0.338371	0.449637	0.642528	0.68823103	0.84852988	1.13825079	24	42	58
6653	NM_003105	SORL1	sortilin related receptor 1	-1.749070778	1.959707048	5.238827	2.998787	9.649995	5.83252285	5.04472373	6.79074481	2198	1679	5298
4069	NM_000239	LYZ	lysozyme	-2.365801991	1.959644571	3.480658	1.472976	6.418095	2.61945147	1.66595383	3.47059318	202	114	487
102466982	NR_106787	MIR6729	microRNA 6729	1.489387219	1.959189961	2.566315	4.090513	4.978037	0.23670415	0.3412894	0.43515728	7	14	17
55515	NM_018674,NM_182847	ASIC4	acid sensing ion channel subunit	1.700005729	1.959189961	0.058738	0.108043	0.11089	0.23670415	0.38406669	0.43515728	7	16	17
55259	NM_001082972,NM_001082973	CASC1	cancer susceptibility 1	1.384077964	1.959189961	0.06738	0.093387	0.143104	0.23670415	0.31941552	0.43515728	7	13	17
9249	NM_001319225,NM_001324370	DHRS3	dehydrogenase/reductase 3	1.279297701	1.959101573	68.625552	81.194593	115.895215	6.72155623	7.07392876	7.6850595	4104	7015	9889
339745	NM_001001664	SPOPL	speckle type BTB/POZ protein lik	1.267084257	1.958546832	4.95978	6.296612	9.134974	4.84361034	5.17452161	5.78866208	1088	1842	2621
8738	NM_001320099,NM_001320100	CRADD	CASP2 and RIPK1 domain conta	-1.289032888	1.957864983	2.081355	1.542196	3.501151	1.63881618	1.40024122	2.36229074	83	86	200
23348	NM_001130048,NM_001130049	DOCK9	dedicator of cytokinesis 9	-1.134090557	1.957785445	2.452906	2.152125	4.699141	4.26009342	4.08859126	5.19211053	713	840	1717
2319	NM_001330170,NM_004475	FLOT2	flotillin 2	1.401316539	1.95776251	14.627807	20.523462	26.919095	5.26749617	5.74354211	6.21831452	1473	2758	3547
643338	NM_001130448	C15orf62	chromosome 15 open reading fra	-2.589084942	1.956751311	1.365434	0.526576	2.507971	2.10810423	1.18729361	2.90342651	130	67	313
55775	NM_001008744,NM_001330205	TDP1	tyrosyl-DNA phosphodiesterase 1	1.189505445	1.956207858	4.876938	5.886755	8.98153	4.18934543	4.42708835	5.11833737	677	1076	1629
284802	NR_003579,NR_145491	FRG1BP	FSHD region gene 1 family mem	-1.146253431	1.95619011	3.716387	1.90142	5.944044	2.23060596	2.07783221	3.04046258	145	169	349
3799	NM_004521	KIF5B	kinesin family member 5B	1.125874617	1.956035332	28.86577	32.534515	53.059921	7.37947593	7.54955574	8.3431795	6498	9775	15633
101928123	NR_135248	LOC101928123	uncharacterized LOC101928123	1.739750021	1.955942057	0.182759	0.318944	0.338816	0.38477185	0.61708849	0.6785857	12	28	29
27238	NM_015698	GPKOW	G-patch domain and KOW motifs	1.222998502	1.955771791	6.747493	8.270217	12.413182	3.64240927	3.91166916	4.55272125	451	737	1085
3998	NM_005570	LMAN1	lectin, mannose binding 1	1.153903696	1.955212143	31.296686	36.150997	57.502628	7.21236912	7.41759738	8.17494733	5783	8916	13907
339988	NR_147207	LINC002481	long intergenic non-protein codin	1.542738532	1.954731302	0.907114	1.41981	1.689533	1.39514558	1.81408935	2.06668624	64	132	154
57519	NM_020759	STARD9	StAR related lipid transfer domai	-1.024321834	1.954484664	0.11545	0.112384	0.212787	1.46336426	1.44132992	2.14982678	69	90	166
25901	NM_015439	CCDC28A	coiled-coil domain containing 28/	-1.009067642	1.953470572	4.409097	4.388362	8.122779	2.89611494	2.88484352	3.76460718	253	335	608
57125	NM_020405	PLXDC1	plexin domain containing 1	-1.28693842	1.953386065	0.112732	0.086949	0.205931	0.7550755	0.61708849	1.23026757	27	28	65
51478	NM_001304512,NM_001304513	HSD17B7	hydroxysteroid 17-beta dehydrog	-1.31226985	1.953287749	8.009942	6.021665	15.002503	3.61567874	3.25980797	4.52314065	442	450	1062
347404	NM_001170331,NM_198511	LANCL3	LanC like 3	1.098932245	1.953064707	0.25239	0.270669	0.461295	0.86002952	0.92294555	1.37552141	32	47	77
100885776	NR_047679	UGDH-AS1	UGDH antisense RNA 1	1.511628101	1.952509901	0.413469	0.625704	0.756381	1.13562816	1.49109792	1.73991364	47	95	113
84858	NM_032772,NR_120651	ZNF503	zinc finger protein 503	-1.990988725	1.951657012	1.305132	0.65363	2.395253	2.39332696	1.64851465	3.21791812	167	112	401
51256	NM_001143964,NM_001143965	TBC1D7	TBC1 domain family member 7	1.103679225	1.951539803	5.207748	5.909396	10.225049	2.91572639	3.04002433	3.78425716	257	379	617
5238	NM_001199917,NM_001199918	PGM3	phosphoglucomutase 3	-1.056780486	1.950971615	3.284662	3.330734	6.026254	4.36189543	4.28618731	5.29157606	768	971	1843
55075	NM_001008224,NM_018003	UACA	uveal autoantigen with coiled-coil	1.262985744	1.950144593	26.88659	33.996942	49.281269	7.51321763	7.84841515	8.47295697	7133	12037	17109
401548	NM_001012994	SNX30	sorting nexin family member 30	1.241185518	1.950142546	1.256086	1.562759	2.305007	3.3677101	3.65208684	4.26173929	366	607	878
2537	NM_002038,NM_022872,NM_02	IFI6	interferon alpha inducible protein	-1.167163481	1.948532338	44.811788	38.360992	81.974297	5.23273681	5.01611219	6.17637972	1437	1645	3444
84307	NM_001135178,NM_032347	ZNF397	zinc finger protein 397	1.774764564	1.948336492	2.02708	3.621419	4.024638	2.60139951	3.3216324	3.44338646	199	472	477
29767	NM_001142885,NM_014548	TMOD2	tropomodulin 2	1.136434318	1.948178614	0.53697	0.611198	0.984777	2.53954085	2.69403583	3.37591747	189	287	453
55344	NM_018390_chrX,NM_018390_(	PLCXD1	phosphatidylinositol specific phos	1.089172161	1.946551256	4.128697	5.30761	7.387536	4.3072593	4.42452914	5.23240543	738	1074	1767
79143	NM_001146056,NM_001146082	MBOAT7	membrane bound O-acyltransfer:	1.24169801	1.945856672	49.860946	62.135984	93.577492	6.96600154	7.27607402	7.92080277	4869	8078	11653
22926	NM_007348	ATF6	activating transcription factor 6	1.238701972	1.945055636	5.184449	6.429257	9.479455	5.28927297	5.59099458	6.23109848	1496	2476	3579
122953	NM_001135047,NM_001135048	JDP2	Jun dimerization protein 2	-1.04127422	1.944590214	5.704869	5.392923	10.203922	4.480221	4.42452914	5.4080336	837	1074	2002
677770	NR_003004	SCARNA22	small Cajal body-specific RNA 22	2.335566235	1.944264678	3.015022	7.360894	5.639462	0.46682716	0.92294555	0.80366934	15	47	36
1810	NM_001938	DR1	down-regulator of transcription 1	1.15122605	1.943554491	9.495905	10.945539	17.35099	4.93893692	5.13593336	5.87468438	1165	1792	2785
127707	NM_152375	KLHDC7A	kelch domain containing 7A	1.019286611	1.943075354	1.081969	1.10562	1.983067	2.66651408	2.68977954	3.51046477	210	286	502
390980	NM_001023563,NM_001145078	ZNF805	zinc finger protein 805	1.049635799	1.942857214	4.674052	4.882425	8.49135	5.54913078	5.61756579	6.49232289	1799	2523	4299
50	NM_001098	ACO2	aconitase 2	1.452471389	1.942191446	19.313593	28.089499	35.257019	5.71571556	6.24567154	6.6600601	2024	3928	4835
10110	NM_001199264,NM_016276,NM	SGK2	SGK2, serine/threonine kinase 2	-2.039257247	1.941204177	1.812181	0.832831	3.317177	2.18284609	1.45142181	2.9773697	139	91	332
83729	NM_031479	INHBE	inhibin beta E subunit	7.341636058	1.940775056	0.103546	0.784966	0.19437	0.3273552	1.52971203	0.58209675	10	99	24
27147	NM_001318052,NM_001318053	DENND2A	DENN domain containing 2A	1.195404003	1.940775056	0.067759	0.079036	0.124786	0.3273552	0.38406669	0.58209675	10	16	24
7376	NM_001256647,NM_007121	NR1H2	nuclear receptor subfamily 1 grou	1.027320359	1.940227624	22.022037	22.835687	39.78389	5.50125623	5.53929737	6.44200835	1739	2387	4150
404636	NM_001303111,NM_001303112	FAM45A	family with sequence similarity 45	-1.32377934	1.940117945	1.178032	0.872956	2.07904	1.93632435	1.64851465	2.69776636	111	112	265
51130	NM_001201965,NM_016115,NM	ASB3	ankyrin repeat and SOCS box co	3.116223999	1.938875266	3.685643	12.378	6.705795	3.20189991	4.73140553	4.07935661	322	1341	768
54499	NM_001256164,NM_001256165	TMCO1	transmembrane and coiled-coil d	-1.028673209	1.938267195	12.494474	12.123405	22.797037	5.79330362	5.75326258	6.73545908	2138	2777	5097
7110	NM_007114	TMF1	TATA element modulatory factor	-1.02930076	1.93753502	4.96121	4.825973	9.035757	5.0976022	5.0571683	6.03134989	1305	1694	3110
9948	NM_005112,NM_017491	WDR1	WD repeat domain 1	1.485127174	1.936811654	72.97795	108.209437	133.014733	7.80439611	8.37287861	8.75496369	8737	17337	20813
80312	NM_030625	TET1	tet methylcytosine dioxygenase 1	-1.092589253	1.936455665	0.048999	0.044442	0.088875	0.54446555	0.50527715	0.91876801	18	22	43
830	NM_006136	CAPZA2	capping actin protein of muscle Z	-1.16373652	1.935538796	11.165454	9.602038	20.315737	4.73465189	4.52470876	5.66103101	1006	1155	2395
79586	NM_001195731,NM_024536	CHPF	chondroitin polymerizing factor	1.311244245	1.93525376	31.012708	40.793703	56.443126	6.49749496	6.88464666	7.44230257	3508	6146	8350
84614	NM_001122770,NM_001346115	ZBTB37	zinc finger and BTB domain conta	1.151981134	1.934965706	0.901529	0.966869	1.617641	1.55375576	1.69172362	2.2474368	76	117	181
9123	NM_001042422,NM_001042423	SLC16A3	solute carrier family 16 member 3	1.867360227	1.934473842	211.793548	396.535014	385.547726	8.72704906	9.62647137	9.67734939	16597	41410	39489
55361	NM_018425	PI4K2A	phosphatidylinositol 4-kinase type	-1.192313594	1.934176909	7.977424	6.695348	14.501628	5.06723449	4.8217002	5.99807984	1277	1431	3038
92154	NM_138383	MTSS1L	MTSS1L, I-BAR domain containi	-1.234778983	1.933347497	44.063725	35.724743	80.055283	7.74499725	7.44231817	8.69285724	8383	9071	19934
253650	NM_001331100,NM_147195	ANKRD18A	ankyrin repeat domain 18A	-4.19763606	1.933035908	0.915421	0.218035	1.663085	2.25390578	0.92294555	3.05131575	148	47	352
9567	NM_004286	GTPBP1	GTP binding protein 1	1.651820648	1.931508097	13.341378	22.062538	24.216057	6.05664057	6.7721428	6.99590748	2574	5681	6115
928	NM_001330312,NM_001769	CD9	CD9 molecule	-1.803936071	1.931465026	202.582993	112.430099	367.715851	8.02344385	7.17673423	8.97047061	10176	7537	24174
10735	NM_001042749,NM_001042750	STAG2	stromal antigen 2	1.26224765	1.930995217	16.858057	21.915258	31.6873	6.70086458	7.03398331	7.64352661	4045	6822	9607
100289495	NR_040022	LOC100289495	uncharacterized LOC100289495	1.035997523	1.930786668	0.086893	0.090234	0.154638	0.41264541	0.42561204	0.71544405	13	18	31
161176	NM_152592,NR_026779	SYNE3	spectrin repeat containing nuclea	-2.126274093	1.930682654	8.303926	3.513045	9.182648	4.70114026	3.67379966	5.62334321	982	617	2332
22974	NM_012112	TPX2	TPX2, microtubule nucleation fac	1.425089223	1.930643416	51.54998	73.54213	93.523949	7.52981716	8.03854519	8.47514157	7216	13740	17135
60468	NM_001170794,NM_021813	BACH2	BTB domain and CNC homolog 2	2.219208637	1.930459247	0.011528	0.029668	0.023693	0.17294258	0.36283659	0.32020933	5	15	12
163747	NM_001110533,NM_152607	LEXM	lymphocyte expansion molecule	4.417462774	1.930459247	0.068876	0.338891	0.133964	0.17294258	0.65251278	0.32020933	5	30	12
389337	NM_001001669	ARHGEF37	Rho guanine nucleotide exchang	-1.725236632	1.930117212	1.879514	1.089464	3.412566	3.30959645	2.6243808	4.18661421	350	271	831
54828	NM_001099432,NM_001320470	BCAS3	BCAS3, microtubule associated (	1.330834727	1.929885513	3.284014	4.411447	6.035975	3.66865353	4.05258161	4.56160497	460	818	1092
5826	NM_005050,NR_003256	ABCD4	ATP binding cassette subfamily [	1.490332156	1.928683403	7.949095	11.642824	14.113047	4.61678723	5.17299774	5.53589126	924	1840	2192
80055	NM_001321099,NM_001321100	PGAP1	post-GPI attachment to proteins	1.652576011	1.928492726	1.423537	2.377549	2.602875	4.04765842	4.73760421	4.95263026	610	1347	1447
2194	NM_004104	FASN	fatty acid synthase	1.029474916	1.927818603	50.55817	52.105312	91.593132	8.70511649	8.74692646	9.65042591	16346	22484	38758
100113407	NM_001100829	TMEM170B	transmembrane protein 170B	1.308727832	1.927745571	0.097384	0.128169	0.180284	0.86002952	1.04792463	1.36396013	32	56	76
391322	NM_001144931	LOC391322	D-dopachrome tautomerase-like	1.938114592	1.927745571	1.484664	2.855747	2.67539	0.86002952	1.36863803	1.36396013	32	83	76
286144	NM_001171795,NM_001171796	TRIQK	triple QxxK/R motif containing	-1.579819774	1.926580629	4.447066	2.778202	7.996725	4.07407972	3.46301778	4.97844822	622	526	1474

7873	NM_006010	MANF	mesencephalic astrocyte derived	1.327339673	1.926215281	50.237461	66.768997	90.970619	5.5886112	5.98975653	6.51993332	1850	3281	4383
3155	NM_000191,NM_001166059	HMGCL	3-hydroxymethyl-3-methylglutary	1.22175587	1.925721252	14.880814	18.402092	27.170191	4.58957318	4.86764197	5.50595994	906	1479	2146
90321	NM_001010851	ZNF766	zinc finger protein 766	-1.262883088	1.925556277	5.916564	4.69068	10.708282	4.18126603	3.86524379	5.08791408	673	712	1594
360023	NM_194314,NR_135153	ZBTB41	zinc finger and BTB domain conta	-1.045859579	1.924713014	2.74155	2.626871	4.967087	4.56494429	4.50304006	5.4800877	890	1137	2107
57604	NM_001099677,NM_020844	KIAA1456	KIAA1456	1.307702239	1.924517863	0.04363	0.058152	0.079095	0.49317359	0.61708849	0.83750224	16	28	38
169841	NM_001301275,NM_003448,NM	ZNF169	zinc finger protein 169	1.181483058	1.924283426	0.790138	0.949274	1.292579	1.55375576	1.71704117	2.24113096	76	120	180
10955	NM_006811,NM_198941	SERINC3	serine incorporator 3	1.088462835	1.924113731	32.40697	35.464098	60.209458	7.08391271	7.20534268	8.02300497	5287	7689	12512
5358	NM_001136025,NM_001172335	PLS3	plastin 3	-1.021847264	1.923973903	32.496435	33.108394	59.842156	6.84910932	6.81820237	7.78719361	4487	5867	10618
200958	NM_001282506,NM_001291833	MUC20	mucin 20, cell surface associated	-1.603536233	1.923820961	56.347473	34.952042	101.055939	6.86369731	6.18987729	7.80172795	4533	3777	10726
55353	NM_018407	LAPTM4B	lysosomal protein transmembran	1.544537629	1.923571155	55.040257	85.114662	99.501923	6.9181888	7.54116363	7.85625366	4709	9718	11141
8731	NM_001308263,NM_003799	RNMT	RNA guanine-7 methyltransferas	1.586768341	1.922530586	8.886506	11.485027	13.560492	5.45341658	6.10731444	6.38058222	1681	3564	3975
57185	NM_001322854,NM_001322855	NIPAL3	NIPA like domain containing 3	-1.173675336	1.922104188	5.708221	4.371345	9.650607	4.35831669	4.13941485	5.26695724	766	872	1811
26012	NM_001130969,NM_001130970	NSMF	NMDA receptor synaptonuclear s	2.323031155	1.921946287	10.148852	23.512828	18.301246	5.2001212	6.39366563	6.12379429	1404	4358	3319
11001	NM_001159629,NM_003645	SLC27A2	solute carrier family 27 member 2	-1.206989187	1.921840131	5.824161	4.853225	10.528788	3.86553291	3.61440811	4.75988697	533	590	1260
7259	NM_003309	TSPYL1	TSPY like 1	1.555871026	1.921668062	5.797248	9.032793	10.473209	4.94013441	5.5610149	5.85984164	1166	2424	2756
8976	NM_003941	WASL	Wiskott-Aldrich syndrome like	-1.374376239	1.921161869	7.598558	5.536016	13.721992	5.08358236	4.64060352	6.00507466	1292	1256	3053
7461	NM_003388,NM_032421	CLIP2	CAP-Gly domain containing linke	-2.176242783	1.920710107	2.836573	1.306436	5.125456	4.00938005	2.98897501	4.90755118	593	364	1401
27285	NM_014466	TEKT2	tektin 2	1.25321871	1.920655132	5.10168	6.405004	9.22337	3.08983317	3.38091595	3.9480763	295	494	697
80110	NM_025040	ZNF614	zinc finger protein 614	-7.838737256	1.920416567	0.425315	0.050428	0.777944	1.56621863	0.31941552	2.2537152	77	13	182
158405	NM_001287036,NM_001287038	KIAA1958	KIAA1958	-1.115654109	1.91987059	1.123779	1.018627	2.049371	3.22958708	3.08932884	4.09517603	329	394	777
54820	NM_001143979,NM_017668	NDE1	nudE neurodevelopment protein	1.101346022	1.91980439	4.42655	4.86237	7.975031	3.89547266	4.02581645	4.78935776	545	802	1287
65010	NM_001040454,NM_001281732	SLC26A6	solute carrier family 26 member 6	1.484119863	1.919727165	4.117024	6.025469	7.34308	3.53238053	4.0608449	4.41245104	415	823	980
4299	NM_001166693,NM_001313959	AFF1	AF4/FMR2 family member 1	1.176353365	1.919421637	3.511515	4.11202	6.371742	5.03397229	5.2616952	5.95345618	1247	1960	2944
100128966	NR_146628	LOC100128966	uncharacterized LOC100128966	2.690461792	1.918129359	0.351565	1.020397	0.661235	0.26755841	0.63490936	0.47868902	8	29	19
100820829	NM_001018100,NM_152451	MYZAP	myocardial zonula adherens prot	1.16987675	1.917292679	6.467107	7.547797	11.632585	3.99331833	4.20649667	4.88851253	586	916	1382
2524	NM_000511,NM_001097638	FUT2	fucosyltransferase 2	-5.205737137	1.916678412	3.003641	0.571095	5.405152	3.32800968	1.45142181	4.1964253	355	91	837
4199	NM_002395	ME1	malic enzyme 1	1.109342734	1.916549687	7.320899	8.136546	13.196405	4.70537203	4.84963122	5.61726943	985	1460	2322
729440	NM_001267723	CCDC61	coiled-coil domain containing 61	-1.333292546	1.915790714	6.995739	5.249619	12.605742	3.74737673	3.36765473	4.63318145	488	489	1150
373	NM_001656,NM_033227,NM_03	TRIM23	tripartite motif containing 23	1.676251934	1.915747744	2.036407	3.375768	3.619048	3.11125378	3.78770843	3.96730515	300	672	707
51195	NM_001303533,NM_001303534	RAPGEFL1	Rap guanine nucleotide exchang	-1.488007153	1.914960935	19.247081	12.820532	33.733909	6.28255717	5.71816702	7.21101548	3017	2709	7106
3171	NM_004497	FOXA3	forkhead box A3	2.132075795	1.914630978	1.134542	2.434383	2.057939	1.6852613	2.51796519	2.39105499	87	248	205
811	NM_004343	CALR	calreticulin	1.247612263	1.913823148	368.387421	460.11145	662.53125	9.42296357	9.74171716	10.35842006	26910	44858	63343
23492	NM_001346743,NM_001346744	CBX7	chromobox 7	-1.01456162	1.91238945	2.339583	2.313193	4.322833	3.3677101	3.34888182	4.23501577	366	482	861
51760	NM_001308157,NM_001330509	SYT17	synaptotagmin 17	1.000266076	1.912137783	2.827095	2.672453	4.793473	3.18583679	3.18617855	4.04356865	318	425	748
84787	NM_032701	KMT5C	lysine methyltransferase 5C	-1.327702095	1.911583616	14.353821	10.817962	25.783697	5.05623208	4.66140215	5.97023307	1267	1275	2979
84271	NM_001278657,NM_032311,NM	POLDIP3	DNA polymerase delta interacting	1.193024969	1.911535653	11.286013	13.449134	20.252531	5.2722583	5.52084648	6.18912959	1478	2356	3475
977	NM_001039490,NM_004357,NM	CD151	CD151 molecule (Raph blood gro	1.109152761	1.911491437	78.112258	86.956271	140.696659	6.87688916	7.02514203	7.80573942	4575	6780	10756
3707	NM_002221	ITPKB	inositol-trisphosphate 3-kinase B	1.465458513	1.911177742	1.521962	2.235647	2.73452	3.34257292	3.84815806	4.20778788	359	703	844
80129	NM_025059	CCDC170	coiled-coil domain containing 170	1.174775307	1.909196357	0.069229	0.079512	0.123207	0.43999062	0.50527715	0.75138414	14	22	33
4240	NM_001114614,NM_001310319	MFGE8	milk fat globule-EGF factor 8 prot	-1.456448339	1.908740248	5.382853	3.726258	10.021654	3.51962058	3.03332122	4.39125092	411	377	965
2224	NM_001135821,NM_001135822	FDPS	farnesyl diphosphate synthase	-1.17626181	1.906814012	87.579322	74.461483	155.880407	6.85895655	6.62693041	7.78421487	4518	5132	10596
100506746	NR_038841	AFF1-AS1	AFF1 antisense RNA 1	1.099523058	1.906107538	0.656817	0.724316	1.173913	1.16869613	1.24644988	1.75769337	49	72	115
80740	NM_025261	LY6G6C	lymphocyte antigen 6 family mem	-1.213270575	1.90559328	0.593606	0.488415	1.065669	0.59399636	0.50527715	0.98063768	20	22	47
11153	NM_007076	FICD	FIC domain containing	-1.010500091	1.905025304	1.416701	1.409662	2.549732	1.7191395	1.70865125	2.42483178	90	119	211
3161	NM_001142556,NM_001142557	HMMR	hyaluronan mediated motility rece	1.28476192	1.904618567	29.81589	38.403794	53.513065	6.52009264	6.87811488	7.44213077	3564	6118	8349
4247	NM_002408	MGAT2	mannosyl (alpha-1,6-)-glycoprote	1.532964456	1.904361621	15.387525	23.618858	27.543205	5.37942749	5.98368468	6.29222908	1595	3267	3736
8970	NM_021058	HIST1H2BJ	histone cluster 1 H2B family mem	1.666839853	1.903646021	1.404969	2.344686	2.500817	0.73313634	1.07428646	1.17840727	26	58	61
5597	NM_002748	MAPK6	mitogen-activated protein kinase	-1.174395429	1.901958627	12.005466	10.232116	21.45849	5.65502199	5.42807474	6.56890491	1939	2206	4536
51523	NM_001317199,NM_001317200	CXXC5	CXXC finger protein 5	-1.182301433	1.901047181	19.343169	15.236247	31.992912	5.52459402	5.28868274	6.43649962	1768	1998	4134
23612	NM_012396,NR_073080	PHLDA3	pleckstrin homology like domain f	-1.077593058	1.900811632	18.299371	16.444392	32.079341	5.49719403	5.39185113	6.40863599	1734	2150	4054
1457	NM_001895,NM_177559,NM_17	CSNK2A1	casein kinase 2 alpha 1	1.773515633	1.9007573	36.267849	64.364553	64.83044	6.60067932	7.42081106	7.52021206	3771	8936	8816
50814	NM_001129765,NM_015922	NSDHL	NAD(P) dependent steroid dehyd	1.246935082	1.899616892	19.089375	23.948186	34.381015	4.9244889	5.23346232	5.82758721	1153	1921	2694
79735	NM_001168222,NM_024682	TBC1D17	TBC1 domain family member 17	-1.207775553	1.897277602	11.854886	9.801525	21.103108	4.8242841	4.56244519	5.72399823	1073	1187	2504
10163	NM_001201404,NM_006990	WASF2	WAS protein family member 2	1.205667301	1.896417987	16.646164	20.274605	30.200707	6.54391798	6.8111171	7.45988874	3624	5838	8453
55751	NM_018241	TMEM184C	transmembrane protein 184C	1.467819085	1.89640018	4.544482	6.680947	8.106395	3.82462152	4.34556265	4.69907543	517	1014	1206
929	NM_000591,NM_001040021,NM	CD14	CD14 molecule	1.620817058	1.895923027	1.325918	2.170977	2.382047	1.65056839	2.16012918	2.33885915	84	182	196
84458	NM_001170765,NM_001170766	LCOR	ligand dependent nuclear recepto	-2.505151303	1.895860761	3.299125	1.703388	6.058535	4.929321	3.67379966	5.82969004	1157	617	2698
147	NM_000679	ADRA1B	adrenoceptor alpha 1B	1.960277796	1.894889289	0.521722	1.024105	0.930077	1.10178443	1.70021226	1.66651905	45	118	105
729877	NM_001123392,NM_001123392	TBC1D3H	TBC1 domain family member 3H	1.631720884	1.894881768	2.033124	3.317315	3.620631	2.38631541	2.98202989	3.17224778	166	362	387
56548	NM_019886	CHST7	carbohydrate sulfotransferase 7	2.399523684	1.894081312	0.341524	0.834665	0.617149	0.88013425	1.59489542	1.37552141	33	106	77
100270746	NR_026776	LOC100270746	uncharacterized LOC100270746	1.563082385	1.894081312	0.915525	1.425145	1.635386	0.88013425	1.21124797	1.37552141	33	69	77
54557	NM_019072	SGTB	small glutamine rich tetratricopep	1.252585058	1.893540999	1.583942	1.988837	2.824019	3.23740108	3.53121349	4.08464905	331	554	771
51201	NM_016353	ZDHHC2	zinc finger DHHC-type containing	-1.877309252	1.891632289	2.373006	1.262409	4.218129	3.36057263	2.56983975	4.21263029	364	259	847
5731	NM_000955	PTGER1	prostaglandin E receptor 1	-3.938343863	1.891565762	0.324779	0.075121	0.58	0.54446555	0.15624221	0.90287695	18	6	42
27134	NM_001267560,NM_001267561	TJP3	tight junction protein 3	-1.646325817	1.891483066	23.906102	14.580195	42.575186	6.20398443	5.49729379	7.1142756	2855	2317	6642
55342	NM_001171137,NM_018387,NR	STRBP	spermatid perinuclear RNA bindi	1.177145373	1.890490274	4.5657	5.373238	8.148068	4.88274916	5.11068396	5.77835625	1119	1760	2602
132014	NM_001193380,NM_153480,NM	IL17RE	interleukin 17 receptor E	1.071837593	1.889987003	2.709354	2.906857	4.887045	3.02359429	3.11177339	3.85616495	280	401	651
135154	NM_145267	SDHAF4	succinate dehydrogenase comple	-1.185386687	1.889874207	6.230849	5.265914	11.132738	2.07360076	1.89025622	2.82126674	126	142	293
54926	NM_017811	UBE2R2	ubiquitin conjugating enzyme E2	1.102342136	1.889204171	9.516912	10.504936	16.901865	5.40306784	5.54047969	6.30475523	1622	2389	3769
8362	NM_003541	HIST1H4K	histone cluster 1 H4 family memb	1.368840535	1.888814955	0.879872	1.174869	1.567576	0.38477185	0.50527715	0.65979727	12	22	28
100132159	NR_120496,NR_120497	CALML3-AS1	CALML3 antisense RNA 1	-2.633321293	1.888814955	0.084502	0.031657	0.150337	0.38477185	0.15624221	0.65979727	12	6	28
134353	NM_173491	LSM11	LSM11, U7 small nuclear RNA as	-1.246899289	1.887646267	3.109824	2.496289	5.517644	4.38845666	4.08697381	5.27237862	783	839	1818
10933	NM_001265603,NM_001265604	MORF4L1	mortality factor 4 like 1	1.368396599	1.88737456	66.149823	90.414819	117.461301	6.90168761	7.3509309	7.81240051	4655	8511	10806
7086	NM_001064,NM_001135055,NM	TKT	transketolase	1.30975553	1.887308712	83.622326	108.984525	148.19735	7.48046358	7.86785453	8.39300181	6972	12201	16184
10972	NM_006827	TMED10	transmembrane p24 trafficking pr	1.29036013	1.887278951	74.652557	96.44194	132.40358	8.23946164	8.60616376	9.15352973	11826	20389	27451
339483	NR_026850	MTMR9LP	myotubularin related protein 9-lik	1.475266328	1.887106039	0.415569	0.615388	0.73818	0.95786335	1.25799553	1.47560397	37	73	86
221400	NM_001010870,NM_001168359	TDRD6	tudor domain containing 6	4.038678222	1.886081071	0.023314	0.172897	0.098927	0.29776659	0.95166931	0.52094563	9	49	21
1174	NM_001283	AP1S1	adaptor related protein complex 1	-1.022903034	1.885843014	30.216364	29.583488	53.583736	5.29208934	5.26026072	6.1899483	1499	1958	3477
114793	NM_052905	FMNL2	formin like 2	-1.279843964	1.885575534	5.655101	4.42626	10.026295	4.987237	4.64390751	5.88078182	1206	1259	2797
11119	NM_001145008,NM_001145009	BTN3A1	butyrophilin subfamily 3 member	-1.336030489	1.88552862	3.57597	2.864896	6.643038	3.84009957	3.45551618	4.70710452	523	523	1213
11046	NM_001286990,NM_007001,NR	SLC35D2	solute carrier family 35 member D	-1.168331133	1.885525393	11.55262	10.212574	21.63119	4.38318339	4.17029377	5.26540453	780	892	1809
79709	NM_024656	COLGALT1	collagen beta(1-O)galactosyltrans	1.224761364	1.884479074	34.701683	42.554119	61.458767	6.98033279	7.27074087	7.88914127	4918	8048	11399
3673	NM_002203,NR_073103,NR_07	ITGA2	integrin subunit alpha 2	1.081009218	1.884138719	26.946018	29.365206	48.017057	7.69355889	7.80541678	8.60419941	8088	11682	18743
23767	NM_013281,NM_198391	FLRT3	fibronectin leucine rich transmem	1.962099884	1.883898817	0.978525	2.022996	1.82079	2.27683528	3.09577728	3.04408938	151	396	350
123096	NM_001039355,NM_001291813	SLC25A29	solute carrier family 25 member 2	-1.780642192	1.883282442	8.976105	4.936513	15.507989	4.53196733	3.74731998	5.41575311	869	652	2013
414	NM_001669,NM_009589	ARSD	arylsulfatase D	-1.400839544	1.883105397	4.234617	2.990915	7.344084	4.41797983	3.95841731	5.29918407	800	763	1853
2760	NM_000405,NM_001167607	GM2A	GM2 ganglioside activator	1.239726304	1.882753684	9.950594	12.396785	17.644868	5.19912125	5.50154961	6.09348707	1403	2324	3249
84518	NM_032488	CNFN	cornifelin	1.435131518	1.882321382	1.215149	1.749252	2.194181	0.71085839	0.93737891	1.13825079	25	48	58
51150	NM_016176,NM_016547	SDF4	stromal cell derived factor 4	1.226596021	1.882042371	64.042312	78.708391	113.477837	6.99481123	7.28738593	7.90181442	4968	8142	11500
340120	NM_001004441	ANKRD34B	ankyrin repeat domain 34B	1.823300959	1.881488083	0.267501	0.49597	0.479696	1.03161781	1.5392062	1.56919173	41	100	95
102725126	NR_136306	LOC102725126	FSHD region gene 1 pseudogene	1.331712598	1.881488083	1.06001	1.416631	1.87701	1.03161781	1.25799553	1.56919173	41	73	95
6256	NM_001291920,NM_001291921	RXRA	retinoid X receptor alpha	1.074328847	1.881251356	11.860337	12.66397	21.043589	6.02482449	6.12673052	6.92612999	2517	3613	5824
79772	NM_001002796,NM_001297777	MCTP1	multiple C2 and transmembrane	1.125310201	1.8809139	3.202967	3.604835	5.632219	3.92722123	4.08697381	4.79367305	558	839	1291
2145	NM_001321079,NM_001321081	EZH1	enhancer of zeste 1 polycomb re	1.178923811	1.880738399	11.278919	13.347224	19.997953	5.70448427	5.9377612	6.60280623	2008	3163	4645
55222	NM_001278211,NM_001278212	LRRC20	leucine rich repeat containing 20	-1.613680076	1.880737795	4.733848	2.618854	8.522898	3.91265481	3.27976824	4.77851272	552	457	1277
65065	NM_001114132	NBEAL1	neurobeachin like 1	1.517538204	1.880721468	0.826125	1.257587	1.463257	3.05488459	3.59633059	3.88273891	287	582	664
1747	NM_005220	DLX3	distal-less homeobox 3	3.571567149	1.880663449	0.056842	0.213617	0.106101	0.20517559	0.63490936	0.36729312	6	29	14
51754	NM_001042589,NM_001042590	TMEM8B	transmembrane protein 8B	1.287912276	1.879854869	1.470569	1.655877	2.227736	2.24618091	2.541776	3.00740444	147	253	340
93233	NM_144577	CCDC114	coiled-coil domain containing 114	-1.482465361	1.879666567	1.557594	1.048564	2.754093	2.55837852	2.10365798	3.34978031	192	173	444

171017	NM_001039920,NM_001135734	ZNF384	zinc finger protein 384	2.136851769	1.87873884	9.311323	20.07514	16.394086	4.89144191	5.96090566	5.77835625	1126	3215	2602
201232	NM_201566	SLC16A13	solute carrier family 16 member 1	1.041709681	1.878141619	2.211916	2.308476	3.906898	2.25390578	2.30073045	3.01481644	148	206	342
3694	NM_000888,NM_001282353,NM	ITGB6	integrin subunit beta 6	1.518496983	1.877737997	15.44719	23.071719	26.818351	6.10442241	6.69990858	7.00361147	2662	5401	6148
54971	NM_001173539,NM_001173540	BANP	BTG3 associated nuclear protein	-1.324700402	1.8775909	3.942432	2.948969	7.060786	3.31698997	2.95745524	4.15677414	352	355	813
222484	NM_153371	LNX2	ligand of numb-protein X 2	1.202525713	1.877515773	1.756975	2.115357	3.103428	3.2058879	3.44545299	4.03994054	323	519	746
10159	NM_005765	ATP6AP2	ATPase H+ transporting accesso	1.385416643	1.876664173	29.503632	40.929085	52.048748	5.89499076	6.35856873	6.7918238	2297	4252	5302
9695	NM_014674	EDEM1	ER degradation enhancing alpha	1.205962734	1.876618927	11.033875	13.325662	19.4634	6.06873521	6.33525512	6.96682461	2596	4183	5992
51704	NM_001304771,NM_016235	GPRC5B	G protein-coupled receptor class	1.751148092	1.876528833	2.858091	5.431659	5.463682	3.29843463	4.04260277	4.13653226	347	812	801
83464	NM_001145646,NM_031301	APH1B	aph-1 homolog B, gamma-secret	4.562045647	1.875747221	0.429776	1.958632	0.744086	1.44997615	3.15564136	2.08792485	68	415	157
80127	NM_025057	BBOF1	basal body orientation factor 1	2.526531666	1.875423645	0.347809	0.894456	0.619253	1.04948198	1.89025622	1.58918956	42	142	97
20	NM_001606,NM_212533	ABCA2	ATP binding cassette subfamily A	1.347651001	1.875422465	14.081431	18.995995	24.817943	6.8175298	7.24468238	7.71877963	4389	7903	10124
115290	NM_024907,NM_148169,NR_10	FBXO17	F-box protein 17	-1.328438557	1.875373654	2.006719	1.521782	3.588921	2.47491059	2.14776967	3.2559454	179	180	413
7056	NM_000361	THBD	thrombomodulin	2.662103177	1.874727055	9.60831	25.608812	16.926893	5.27510805	6.66428769	6.16434954	1481	5268	3415
8969	NM_021064	HIST1H2AG	histone cluster 1 H2A family men	1.40063497	1.874055766	0.82715	1.172129	1.476329	0.49317359	0.65251278	0.82068496	16	30	37
10794	NM_001330622,NM_006635	ZNF460	zinc finger protein 460	1.380818599	1.873985924	3.187984	4.518133	5.626738	3.5668994	3.99854533	4.41525433	426	786	982
10381	NM_001197181,NM_006086	TUBB3	tubulin beta 3 class III	-1.062436358	1.87383952	13.138192	12.381781	23.084949	4.56339093	4.47980886	5.44073202	889	1118	2049
50484	NM_001172477,NM_001172478	RRM2B	ribonucleotide reductase regulato	1.101659158	1.872960231	2.453389	2.710976	4.331433	3.68016614	3.80944938	4.53220782	464	683	1069
1163	NM_001826,NR_024163	CKS1B	CDC28 protein kinase regulatory	1.18196805	1.872851346	84.839101	100.528759	149.477445	6.10442241	6.34239053	6.99988134	2662	4204	6132
6310	NM_000332,NM_001128164	ATXN1	ataxin 1	1.315875852	1.872838641	1.189845	1.571231	2.103098	3.73639016	4.10626455	4.59041505	484	851	1115
55788	NM_018368	LMBRD1	LMBR1 domain containing 1	-1.005183786	1.872368308	7.359303	7.33393	12.95621	4.13391036	4.12687582	5.00008264	650	864	1497
83452	NM_031296	RAB33B	RAB33B, member RAS oncogen	-1.173974747	1.87215165	2.008362	1.710403	3.529826	3.09843961	2.89597101	3.92268941	297	338	684
352909	NM_001256714,NM_001256715	DNAAF3	dynein axonemal assembly facto	1.270047881	1.872034144	3.187987	4.069295	5.557214	2.99622306	3.30224686	3.81430154	274	465	631
55160	NM_001011722,NM_001319837	ARHGEF10L	Rho guanine nucleotide exchang	-1.123129955	1.872026466	7.786726	6.873912	14.400555	5.20411411	5.04138697	6.09042103	1408	1675	3242
389791	NR_024425	PTGES2-AS1	PTGES2 antisense RNA 1 (head	1.54441331	1.871428242	0.56347	0.883845	1.003144	1.11880553	1.49109792	1.67590018	46	95	106
376940	NM_198581	ZC3H6	zinc finger CCCH-type containing	-1.912617886	1.871097292	1.329816	0.696105	2.339221	3.99562384	3.14012691	4.85689977	587	410	1351
57191	NM_020633	VN1R1	vomeronasal 1 receptor 1	1.063769566	1.870937038	2.652582	2.820662	4.662993	2.33625658	2.40826098	3.10091148	159	226	366
78992	NM_001321439,NM_001321440	YIPF2	Yip1 domain family member 2	-1.016582846	1.870853829	22.527211	23.126442	41.362652	5.23371374	5.21061989	6.11950347	1438	1890	3309
81565	NM_001025579,NM_001330129	NDEL1	nudE neurodevelopment protein	-1.048083788	1.86983022	10.41676	9.930004	18.310226	4.66538426	4.60035389	5.54167545	957	1220	2201
25920	NM_015456	NELFB	negative elongation factor compl	1.295849297	1.869669094	24.223488	31.427059	42.564693	5.94714364	6.31570776	6.83904003	2383	4126	5480
8492	NM_003619	PRSS12	protease, serine 12	-1.083013832	1.869027427	5.259779	4.864577	9.24389	4.61078387	4.50061221	5.48543279	920	1135	2115
285172	NM_001321618,NM_001321619	FAM126B	family with sequence similarity 12	1.310442739	1.868837996	2.8879	3.793327	5.083339	4.79148701	5.16918099	5.66927404	1048	1835	2409
868	NM_001321786,NM_001321788	CBLB	Cbl proto-oncogene B	1.784863156	1.86853964	0.861322	1.603453	1.555381	2.78321062	3.5240591	3.58455875	231	551	531
29978	NM_013444	UBQLN2	ubiquilin 2	1.507449992	1.868159157	3.743929	5.657609	6.580961	3.7528388	4.30856691	4.60399302	490	987	1126
57720	NM_001136557,NM_001136558	GPR107	G protein-coupled receptor 107	1.206156828	1.867934004	16.287705	19.383146	29.172383	6.73576237	7.00387066	7.6309204	4145	6680	9523
55806	NM_005144,NM_018411	HR	HR, lysine demethylase and nucl	1.220922503	1.867737314	0.536369	0.660668	0.96306	1.96484452	2.18453503	2.68388473	114	186	262
22898	NM_014957	DENND3	DENN domain containing 3	-2.609632634	1.867722524	10.250425	3.929003	17.992449	5.78998672	4.44740017	6.67913671	2133	1092	4900
9993	NM_001173533,NM_001173534	DGCR2	DiGeorge syndrome critical regio	1.459945592	1.866929359	14.314153	21.062444	25.137557	5.98882325	6.52758268	6.87893309	2454	4787	5635
10882	NM_006688	C1QL1	complement C1q like 1	-3.312748522	1.866777903	0.514073	0.152543	0.918405	0.81895953	0.29720487	1.28032816	30	12	69
9580	NM_005686	SOX13	SRY-box 13	1.054458798	1.866317266	7.364017	7.772953	12.916916	4.91721027	4.99125166	5.7951333	1147	1616	2633
84959	NM_032873	UBASH3B	ubiquitin associated and SH3 dor	-2.542053477	1.865332871	1.786816	0.702276	3.135323	3.70291935	2.51796519	4.55017297	472	248	1083
64763	NM_001330519,NM_022752	ZNF574	zinc finger protein 574	1.234733904	1.865232629	6.270728	7.753782	10.994428	4.33118941	4.62173661	5.19700577	751	1239	1723
84868	NM_032782	HAVCR2	hepatitis A virus cellular receptor	1.457001873	1.865167344	0.21404	0.311398	0.370359	0.59399636	0.80196545	0.96541723	20	39	46
400579	NR_104343	LINC02076	long intergenic non-protein codin	1.084776333	1.865167344	0.267294	0.29793	0.479066	0.59399636	0.63490936	0.96541723	20	29	46
22875	NM_014936	ENPP4	ectonucleotide pyrophosphatase/	-1.193982527	1.865101747	4.085527	3.425349	7.159501	4.29420754	4.05258161	5.15904416	731	818	1677
728621	NM_001080850	CCDC30	coiled-coil domain containing 30	3.64772358	1.864800911	0.020735	0.090622	0.044053	0.10623215	0.36283659	0.19530369	3	15	7
100847046	NR_049808	MIR5010	microRNA 5010	1.720640609	1.864800911	0.488618	1.042045	1.03491	0.10623215	0.18071335	0.19530369	3	7	7
102466755	NR_106935	MIR6875	microRNA 6875	1.238869866	1.864800911	0.777068	1.199494	1.92956	0.10623215	0.13134883	0.19530369	3	5	7
100289410	NR_034002	MCF2L-AS1	MCF2L antisense RNA 1	1.96152598	1.864800911	0.045301	0.132072	0.11086	0.10623215	0.20477631	0.19530369	3	8	7
692076	NR_003037	SNORD7	small nucleolar RNA, C/D box 7	4.129494323	1.864800911	0.707639	3.271272	1.346561	0.10623215	0.40498891	0.19530369	3	17	7
377841	NM_001033113,NM_198585	ENTPD8	ectonucleoside triphosphate diph	-4.371216231	1.864077081	2.990304	0.682633	5.282352	2.87623322	1.29208824	3.68088855	249	76	571
22978	NM_001134373,NM_001351169	NT5C2	5'-nucleotidase, cytosolic II	-1.691269101	1.863493309	36.299823	21.689456	63.705875	7.00400175	6.25362336	7.89680865	5000	3950	11460
7483	NM_003395	WNT9A	Wnt family member 9A	-1.201549503	1.863420635	0.550704	0.472981	0.985701	0.7550755	0.65251278	1.19154816	27	30	62
2970	NR_002206,NR_002206_dup1	GTF2IP1	general transcription factor IIi pse	-1.13591566	1.863266932	11.034906	9.725157	19.324747	5.32271189	5.14373402	6.20379585	1532	1802	3511
310	NM_001156,NM_001320879,NM	ANXA7	annexin A7	1.120920002	1.86280523	34.678314	38.608349	60.30179	6.38811426	6.55094329	7.27761365	3249	4866	7444
115572	NM_052943	FAM46B	family with sequence similarity 46	1.030188504	1.86255868	3.744929	3.859028	6.554744	3.27204958	3.31058687	4.09866803	340	468	779
81844	NM_030961	TRIM56	tripartite motif containing 56	1.021791461	1.861852035	3.756458	3.84502	6.577163	4.19336823	4.22279084	5.05324052	679	927	1555
5218	NM_001287135,NM_001287136	CDK14	cyclin dependent kinase 14	-1.406733453	1.860939642	2.839654	2.040565	5.063063	3.92480368	3.47048058	4.7763339	557	529	1275
8897	NM_021090,NM_153050,NM_15	MTMR3	myotubularin related protein 3	1.024710287	1.860532194	4.479565	4.606374	7.832431	5.32637993	5.36073105	6.20541627	1536	2103	3515
56649	NM_001083947,NM_001173551	TMPRSS4	transmembrane protease, serine	-1.386520662	1.860408545	10.845	7.818664	18.934499	5.25215086	4.79520018	6.13020668	1457	1404	3334
6609	NM_000543,NM_001007593,NM	SMPD1	sphingomyelin phosphodiesteras	1.57432993	1.860053285	4.583259	7.30279	8.055639	3.59149522	4.20202068	4.4305758	434	913	993
114908	NM_052932	TMEM123	transmembrane protein 123	1.208252874	1.860050474	40.135181	48.547123	70.155251	7.1425652	7.41373148	8.03319161	5508	8892	12601
161436	NM_183387	EML5	echinoderm microtubule associat	1.155730254	1.859912006	0.327744	0.379272	0.572096	1.7191395	1.86782539	2.39673966	90	139	206
284297	NM_001144950,NM_001195267	SSC5D	scavenger receptor cysteine rich	1.143759485	1.859565797	0.086358	0.103243	0.155785	0.5190475	0.58077243	0.85412573	17	26	39
283518	NM_173605,NM_199464	KCNRG	potassium channel regulator	1.712511419	1.859565797	0.279295	0.48881	0.484419	0.5190475	0.80196545	0.85412573	17	39	39
653659	NM_001079809	TMEM183B	transmembrane protein 183B	1.197003336	1.859030301	14.807229	17.733944	25.859482	4.66393542	4.91399145	5.53202221	956	1529	2186
11252	NM_001184970,NM_001184971	PACSIN2	protein kinase C and casein kina	1.058686531	1.85886428	14.377781	15.356979	25.242127	5.5514833	5.6320572	6.43166217	1802	2549	4120
84440	NM_001303542,NM_001346747	RAB11FIP4	RAB11 family interacting protein	-1.62699535	1.858428058	2.400903	1.477466	4.197755	4.35831669	3.69943092	5.2196331	766	629	1751
23558	NM_001330499,NM_001348170	WBP2	WW domain binding protein 2	-1.461338991	1.857996813	75.989915	52.173305	133.390549	7.1873749	6.64463086	8.07655789	5683	5196	12987
101241892	NR_103844	NPTN-IT1	NPTN intronic transcript 1	1.780660713	1.856877219	0.631156	1.127142	1.102367	1.38110603	1.94842076	1.99354404	63	150	144
199692	NM_001290083,NM_001290084	ZNF627	zinc finger protein 627	1.53691177	1.856381884	3.010312	4.636181	5.307795	3.25290323	3.81922435	4.07404468	335	688	765
5874	NM_004163	RAB27B	RAB27B, member RAS oncogen	-1.459001592	1.855941975	2.614119	1.792932	4.560544	4.2349439	3.72461474	5.09142367	700	641	1598
5205	NM_005603	ATP8B1	ATPase phospholipid transportin	-1.240170627	1.855744024	7.858434	6.345662	13.706649	5.53731025	5.23419318	6.41495239	1784	1922	4072
100652736	NR_103767	LINC01311	long intergenic non-protein codin	1.729859556	1.855661238	0.807635	1.394174	1.403412	0.91952222	1.34717796	1.40965954	35	81	80
221416	NM_001171992,NM_001324369	C6orf223	chromosome 6 open reading fran	-19.34123007	1.854701623	1.370456	0.067142	2.404202	3.00997359	0.44594451	3.81642381	277	19	632
10628	NM_001313972,NM_006472	TXNIP	thioredoxin interacting protein	1.403506078	1.854676611	64.94166	88.71217	109.542657	7.5071705	7.99393027	8.39468951	7103	13320	16203
56675	NM_020645	NRIP3	nuclear receptor interacting prote	-8.095670806	1.854024362	0.877038	0.103534	1.528789	2.09095564	0.48576948	2.81703332	128	21	292
999	NM_001317184,NM_001317185	CDH1	cadherin 1	1.009325037	1.85354662	125.686154	126.973737	218.7896	9.20480534	9.21817366	10.09397069	23128	31190	52726
115361	NM_052941	GBP4	guanylate binding protein 4	-1.744647295	1.851940236	2.433777	1.396192	4.235852	3.96064828	3.22493445	4.80654202	572	438	1303
84734	NM_032648	FAM167B	family with sequence similarity 16	1.448591556	1.851788163	0.871407	1.257262	1.509119	0.86002952	1.12560759	1.32870978	32	62	73
11155	NM_001080114,NM_001080115	LDB3	LIM domain binding 3	-1.099499183	1.851582659	0.066356	0.059955	0.126794	0.43999062	0.40498891	0.73352601	14	17	32
101928042	NR_135833	LOC101928042	uncharacterized LOC101928042	-1.434126113	1.851582659	0.623862	0.422942	1.104382	0.43999062	0.31941552	0.73352601	14	13	32
79899	NM_001160167,NM_001160168	PRR5L	proline rich 5 like	1.049297005	1.851430876	0.642134	0.671296	1.110087	1.79522562	1.84504029	2.47942639	97	136	221
57402	NM_020672	S100A14	S100 calcium binding protein A14	1.11793242	1.851187632	35.28009	39.492523	61.379623	5.2258797	5.38265132	6.0965466	1430	2136	3256
79665	NM_001166301,NM_024612	DHX40	DEAH-box helicase 40	-1.276681044	1.8508741	8.11884	6.367269	14.130355	4.90744794	4.56825335	5.77344872	1139	1192	2593
10905	NM_006699	MAN1A2	mannosidase alpha class 1A mer	1.470497622	1.850551494	2.211739	3.258382	3.848285	3.65704831	4.17639102	4.49161333	456	896	1038
57544	NM_001160047,NM_020784	TXNDC16	thioredoxin domain containing 16	1.161931774	1.850442165	1.872597	2.180217	3.262254	3.24517298	3.44039495	4.0615736	333	517	758
440	NM_001178075,NM_001178076	ASNS	asparagine synthetase (glutamin	-1.07711873	1.850294154	47.850893	44.314532	83.575537	6.63065645	6.52459849	7.51172395	3851	4777	8764
9658	NM_014643	ZNF516	zinc finger protein 516	1.041617462	1.850108416	4.621767	4.818817	8.034273	5.31534771	5.37272883	6.18626042	1524	2121	3468
2549	NM_002039,NM_207123	GAB1	GRB2 associated binding protein	-1.12491806	1.849984421	3.51528	3.126481	6.112869	4.80732269	4.64390751	5.67103429	1060	1259	2412
339983	NM_178557	NAT8L	N-acetyltransferase 8 like	2.716951812	1.849944538	0.111224	0.302435	0.192207	0.71085839	1.45142181	1.12461302	25	91	57
157378	NM_194291	TMEM65	transmembrane protein 65	1.275541343	1.849348452	3.489888	4.456954	6.069258	3.96771155	4.29881852	4.81187039	575	980	1308
28227	NM_013239_chrX,NM_013239_(	PPP2R3B	protein phosphatase 2 regulatory	1.032482255	1.848504771	3.258788	3.370681	5.672897	3.12395512	3.16487058	3.93250653	303	418	689
100528064	NM_001199823,NR_137630,NR	NEDD8-MDP1	NEDD8-MDP1 readthrough	1.759161871	1.848422193	3.117321	5.334819	5.201301	1.73025772	2.34470901	2.40240201	91	214	207
23588	NM_014315	KLHDC2	kelch domain containing 2	-1.09685002	1.848237594	23.630142	21.566982	41.054096	5.41172615	5.28162956	6.28228282	1632	1988	3710
1152	NM_001823	CKB	creatine kinase B	-1.128893782	1.847793237	7.611496	6.758238	13.23676	3.56379523	3.40448323	4.39267399	425	503	966
55699	NM_018060	IARS2	isoleucyl-tRNA synthetase 2, mit	1.156066822	1.84714721	42.504005	49.195362	73.790894	7.20839903	7.4163099	8.08922983	5767	8908	13102
375316	NM_001080504	RBM44	RNA binding motif protein 44	1.865937883	1.846675859	0.10396	0.203059	0.18867	0.54446555	0.89363826	0.88680889	18	45	41
55108	NM_001143888,NM_001143889	BSDC1	BSD domain containing 1	1.179367917	1.846134783	10.179763	13.482741	19.276695	5.3571874	5.58985296	6.22551943	1570	2474	3565
23285	NM_015237	KIAA1107	KIAA1107	-1.38641176	1.845779616	2.652399	1.913291	4.597986	3.60967076	3.18315375	4.43886489	440	424	999

84837	NR_027263	ARHGAP5-AS1	ARHGAP5 antisense RNA 1 (hea	1.82862972	1.845473802	3.997965	7.315023	6.936513	2.5831188	3.3407609	3.35270793	196	479	445
259290	NM_176885	TAS2R31	taste 2 receptor member 31	1.594696474	1.84487075	0.161857	0.287121	0.296177	0.23670415	0.36283659	0.41288872	7	15	16
102465136	NR_106744	MIR378J	microRNA 378j	2.015933494	1.84487075	1.632555	3.308185	2.910454	0.23670415	0.44594451	0.41288872	7	19	16
101	NM_001109,NM_001164489,NM	ADAM8	ADAM metallopeptidase domain	1.447343522	1.844277644	56.124119	80.983264	96.923179	7.48887551	8.01980515	8.36826051	7013	13562	15908
148254	NM_001172775,NM_152791	ZNF555	zinc finger protein 555	-1.231777309	1.844062603	0.613404	0.497348	1.062574	2.61345921	2.36620557	3.38452567	201	218	456
5017	NM_004561	OVOL1	ovo like transcriptional repressor	-1.230153382	1.843047026	8.040927	6.538261	13.928629	4.62425646	4.33878278	5.47941817	929	1009	2106
375790	NM_001305275,NM_198576	AGRN	agrin	1.706067038	1.842853148	70.728127	120.804862	122.488029	8.98173046	9.75122652	9.86236954	19809	45155	44899
403	NM_004311	ARL3	ADP ribosylation factor like GTPa	-1.408187446	1.842673142	2.629138	1.870444	4.560008	3.45071403	3.009612	4.27105417	390	370	884
51272	NM_001098787,NM_016526	BET1L	Bet1 golgi vesicular membrane tr	1.213850293	1.842393413	7.370906	8.800943	12.665011	4.42483953	4.69258035	5.27546743	804	1304	1822
10000	NM_001206729,NM_005465,NM	AKT3	AKT serine/threonine kinase 3	1.33972269	1.841671501	10.199751	13.647547	17.59519	6.14237278	6.55913304	7.01405106	2734	4894	6193
54732	NM_017510	TMED9	transmembrane p24 trafficking pr	1.426687563	1.841427651	74.659836	106.636406	129.190201	7.57040247	8.08080625	8.44776463	7423	14150	16812
84612	NM_032521	PARD6B	par-6 family cell polarity regulator	-1.86600993	1.841035674	10.414839	5.588742	18.023703	5.57941842	4.70528714	6.44612614	1838	1316	4162
10438	NM_001190263,NM_001190265	C1D	C1D nuclear receptor corepressc	1.346422535	1.840622603	9.329636	12.724147	16.323676	3.61867338	4.01734947	4.44436461	443	797	1003
441425	NM_001012419,NM_001012419	ANKRD20A3	ankyrin repeat domain 20 family	-1.21373062	1.839777109	0.196768	0.159868	0.341631	0.81895953	0.70406985	1.26797492	30	33	68
6729	NM_001146282,NM_003136	SRP54	signal recognition particle 54	1.322531951	1.839073968	30.383468	40.24573	52.546768	6.09746258	6.49563171	6.96682461	2649	4681	5992
57761	NM_001301188,NM_001301190	TRIB3	tribbles pseudokinase 3	1.597656108	1.838506845	27.245648	43.740306	47.455151	6.06599532	6.73389617	6.93470322	2591	5531	5859
57231	NM_001297614,NM_001304479	SNX14	sorting nexin 14	1.039011254	1.83780257	11.630107	12.04787	20.599221	5.42803514	5.48199112	6.29070334	1651	2292	3732
56963	NM_001166283,NM_001166286	RGMA	repulsive guidance molecule fam	-1.593813712	1.837674453	0.893214	0.559553	1.63361	1.93632435	1.47139626	2.63181117	111	93	251
387	NM_001313941,NM_001313943	RHOA	ras homolog family member A	1.071502359	1.837351351	70.908209	75.308719	121.139522	7.05463513	7.15354777	7.92732258	5180	7416	11706
976	NM_001025160,NM_001784,NM	ADGRE5	adhesion G protein-coupled rece	1.594698988	1.837184693	26.208853	42.37939	45.71212	6.40555802	7.07249912	7.27530111	3289	7008	7432
1476	NM_000100	CSTB	cystatin B	1.01940012	1.836981533	395.496429	403.610901	682.740295	8.50370617	8.53135116	9.37923634	14211	19356	32108
10250	NM_001303448,NM_001303449	SRRM1	serine and arginine repetitive ma	1.118951087	1.836965124	35.502102	39.800658	61.374527	7.10435293	7.26538794	7.9769059	5363	8018	12117
285971	NM_173680	ZNF775	zinc finger protein 775	-1.020258951	1.836810007	0.472768	0.473032	0.833457	1.04948198	1.03456085	1.56919173	42	55	95
399687	NM_001346765,NM_001346766	MYO18A	myosin XVIIIA	-1.316723554	1.836420065	25.326952	19.589902	43.884241	7.5528968	7.158369	8.42629939	7333	7441	16563
11346	NM_001109974,NM_001166208	SYNPO	synaptopodin	-1.060995591	1.836314016	0.785494	0.7425	1.36218	2.3579234	2.2895231	3.10091148	162	204	366
25966	NM_015500,NM_199050	C2CD2	C2 calcium dependent domain cc	-1.184202435	1.835975101	4.883584	4.128667	8.425013	4.96269999	4.72725823	5.81808634	1185	1337	2676
4775	NM_004555,NM_173163,NM_17	NFATC3	nuclear factor of activated T-cells	1.235572636	1.835298726	1.472923	1.825525	2.555603	3.34979977	3.62781914	4.16012035	361	596	815
84449	NM_001300912,NM_032433	ZNF333	zinc finger protein 333	1.050354832	1.835069334	1.613939	1.692324	2.789822	2.79384765	2.85474367	3.57205238	233	327	526
3767	NM_000525,NM_001166290	KCNJ11	potassium voltage-gated channel	-1.842253977	1.834760045	0.443854	0.237203	0.763417	1.30878272	0.84852988	1.89255394	58	42	131
4668	NM_000262	NAGA	alpha-N-acetylgalactosaminidase	1.135943126	1.834261593	3.943316	4.487434	6.798037	3.93685111	4.10945474	4.76868199	562	853	1268
653645	NR_002924	TBC1D3P1-DHX40P1	TBC1D3P1-DHX40P1 readthrou	1.267838295	1.83413614	0.312861	0.395103	0.534954	0.66524311	0.80196545	1.05442063	23	39	52
166929	NM_001136257,NM_001136258	SGMS2	sphingomyelin synthase 2	1.322846206	1.83395718	2.348355	3.087447	4.059488	3.9199564	4.30021519	4.75103801	555	981	1252
5518	NM_014225,NR_033500	PPP2R1A	protein phosphatase 2 scaffold su	1.113578274	1.833491582	54.189869	60.445204	93.391085	7.05960209	7.21370459	7.92928505	5198	7734	11722
29969	NM_001166345,NM_001166346	MDFIC	MyoD family inhibitor domain cor	-1.41580212	1.833240959	3.686761	2.57253	6.440295	4.25432848	3.78372009	5.09405028	710	670	1601
5166	NM_002612	PDK4	pyruvate dehydrogenase kinase 4	-1.625440167	1.83265395	1.109527	0.67853	1.910706	2.32162912	1.79043244	3.05850609	157	129	354
10548	NM_001014842,NM_001289006	TM9SF1	transmembrane 9 superfamily me	1.482600202	1.832076513	25.536834	37.646387	43.551729	5.93578265	6.49624116	6.79854931	2364	4683	5327
81037	NM_030782	CLPTM1L	CLPTM1 like	-1.013865263	1.831560821	28.94569	28.576376	49.813557	6.14237278	6.12278922	7.00617036	2734	3603	6159
2801	NM_004486	GOLGA2	golgin A2	1.101271475	1.831545408	35.795605	39.461208	61.609547	7.22372265	7.36200736	8.09240786	5829	8577	13131
199745	NM_001331102,NM_001331103	THAP8	THAP domain containing 8	-1.168771898	1.83111193	0.935158	0.765029	1.546684	1.38110603	1.24644988	1.97845985	63	72	142
11030	NM_001008710,NM_001008711	RBPMS	RNA binding protein with multiple	1.032804606	1.830341277	7.419917	6.943229	11.77472	3.85541331	3.89881993	4.68171827	529	730	1191
6788	NM_001256312,NM_001256313	STK3	serine/threonine kinase 3	-1.358449418	1.830316961	17.798578	13.135195	31.191765	5.56784445	5.13671533	6.42611372	1823	1793	4104
4285	NM_005932	MIPEP	mitochondrial intermediate peptic	1.553341576	1.829886918	2.269566	3.53159	3.902588	2.67229041	3.22493445	3.43788301	211	438	475
100859930	NR_045680	HEIH	hepatocellular carcinoma up-regu	-1.049077892	1.828010742	8.147153	7.771061	14.005632	3.82979934	3.76563483	4.65350501	519	661	1167
3309	NM_005347	HSPA5	heat shock protein family A (Hsp7	1.212018048	1.827586946	342.439575	415.502625	588.117432	10.37137546	10.64859663	11.24082364	51966	84154	116810
27067	NM_001164380,NM_001164381	STAU2	staufen double-stranded RNA bir	1.189428508	1.827208988	7.098363	8.570815	12.148876	4.70677986	4.9482473	5.55126453	986	1567	2216
4883	NM_000908,NM_001204375,NM	NPR3	natriuretic peptide receptor 3	1.039375241	1.826789751	0.126208	0.130356	0.238091	0.93882015	0.96581954	1.42086173	36	50	81
23181	NM_001146115,NM_001146116	DIP2A	disco interacting protein 2 homol	1.005975889	1.826684063	10.508841	11.089338	19.134275	6.03606811	6.04453358	6.89533961	2537	3410	5700
401474	NM_001101676,NM_001349811	SAMD12	sterile alpha motif domain contair	1.09834426	1.82665789	4.43988	4.702504	7.362513	5.18201511	5.31379245	6.03317592	1386	2034	3114
338442	NM_177551	HCAR2	hydroxycarboxylic acid receptor 2	1.681697764	1.826469027	0.404527	0.675519	0.692902	0.86002952	1.24644988	1.31676564	32	72	72
2870	NM_001004105,NM_001004106	GRK6	G protein-coupled receptor kinas	1.059526858	1.826369984	14.841683	15.746703	25.252595	5.43569657	5.51724792	6.28955798	1660	2350	3729
11074	NM_007028,NR_134870,NR_13	TRIM31	tripartite motif containing 31	-5.160359976	1.826314001	1.233882	0.234923	2.18077	1.79522562	0.56226574	2.46326383	97	25	218
728743	NR_027237	LOC728743	zinc finger protein pseudogene	-1.518602488	1.825687272	1.48048	0.976368	2.540061	2.55212659	2.07130288	3.30513776	191	168	429
441394	NR_003667	SUGT1P1	SGT1 homolog, MIS12 kinetochc	1.010331226	1.824741408	0.679057	0.675605	1.152422	0.59399636	0.59904473	0.95003448	20	27	45
89796	NM_001167738,NM_020443	NAV1	neuron navigator 1	-1.214624249	1.824530005	1.784984	1.468067	3.286227	4.51760029	4.250509	5.35645948	860	946	1930
131583	NM_153690	FAM43A	family with sequence similarity 43	2.169634901	1.82451699	0.502644	1.098794	0.868448	1.35261029	2.12902957	1.9400446	61	177	137
3916	NM_005561	LAMP1	lysosomal associated membrane	1.527401676	1.824293915	37.340904	57.100437	64.024666	6.54470542	7.15045371	7.40506422	3626	7400	8136
51517	NM_016453,NM_184231	NCKIPSD	NCK interacting protein with SH3	1.693387206	1.824194746	4.622973	7.847342	7.929606	3.85541331	4.57403823	4.67705421	529	1197	1187
5522	NM_001206994,NM_001206995	PPP2R2C	protein phosphatase 2 regulatory	-1.647114739	1.824157535	1.713845	1.083737	2.938142	3.0724647	2.45916281	3.86028525	291	236	653
22927	NM_014282	HABP4	hyaluronan binding protein 4	-1.337786213	1.823213134	7.601315	5.685646	13.03071	4.37435155	3.97774828	5.20917167	775	774	1738
23328	NM_001346505,NM_001346506	SASH1	SAM and SH3 domain containing	-1.317585343	1.822675663	1.681888	1.275863	2.875462	3.77984319	3.41483526	4.59783705	500	507	1121
64374	NM_001037633,NM_022464	SIL1	SIL1 nucleotide exchange factor	2.063347694	1.822650747	12.444422	25.922666	21.37657	4.61378867	5.62816997	5.45306113	922	2542	2067
6744	NM_001130445,NM_001287503	SSFA2	sperm specific antigen 2	1.221814753	1.822313952	75.922568	95.418057	131.966323	8.64937537	8.93775028	9.51352931	15725	25671	35245
85441	NM_001037335,NM_033405	HELZ2	helicase with zinc finger 2	-1.004694806	1.821736876	11.123427	11.24255	18.967656	6.66907927	6.66238832	7.52800316	3956	5261	8864
57134	NM_001289010,NM_020379	MAN1C1	mannosidase alpha class 1C me	-1.143779286	1.821687853	0.067476	0.057288	0.113642	0.38477185	0.3412894	0.64076093	12	14	27
100506007	NR_110434,NR_110435	RTCA-AS1	RTCA antisense RNA 1	2.048841259	1.821687853	0.370933	0.80889	0.659948	0.38477185	0.70406985	0.64076093	12	33	27
27128	NM_001318024,NM_013385	CYTH4	cytohesin 4	1.863386516	1.821687853	0.093964	0.181991	0.166149	0.38477185	0.65251278	0.64076093	12	30	27
10608	NM_006454	MXD4	MAX dimerization protein 4	1.421197452	1.82132539	2.880049	4.100374	4.935505	3.53555296	4.00541159	4.34348418	416	790	932
55432	NM_001276320,NM_018566	YOD1	YOD1 deubiquitinase	-1.122248353	1.821183921	18.513647	16.512823	31.683906	6.83211648	6.66726737	7.69128877	4434	5279	9932
126298	NM_001007561	IRGQ	immunity related GTPase Q	-1.152901591	1.820556001	2.954894	2.566581	5.057667	4.85254141	4.65486649	5.69430137	1095	1269	2452
1894	NM_001258315,NM_001258316	ECT2	epithelial cell transforming 2	-1.032747644	1.820251912	36.708027	35.339512	63.376692	7.26229215	7.21611125	8.12220148	5988	7747	13406
6662	NM_000346	SOX9	SRY-box 9	-1.090676328	1.820152966	26.76689	24.569635	45.792301	6.6941375	6.57017385	7.55192345	4026	4932	9013
399664	NM_001174118,NM_203304	MEX3D	mex-3 RNA binding family memb	2.019788754	1.820099391	3.126193	6.345055	5.361624	3.26824058	4.20500622	4.0633618	339	915	759
4628	NM_001256012,NM_001256095	MYH10	myosin heavy chain 10	1.762203547	1.819782282	3.573521	6.309123	6.117852	4.79281331	5.58756701	5.63300839	1049	2470	2348
10769	NM_001252226,NM_006622	PLK2	polo like kinase 2	-1.239038191	1.819765041	78.957046	63.789093	135.005458	7.77567328	7.46802121	8.63646513	8564	9235	19168
203523	NM_152695	ZNF449	zinc finger protein 449	1.115210913	1.819074726	2.603499	2.906514	4.448077	3.49701545	3.64110665	4.30167645	404	602	904
8082	NM_001135823,NM_005086	SSPN	sarcospan	-1.244830857	1.818653141	2.675061	2.241187	4.640243	3.68016614	3.39143785	4.49161333	464	498	1038
100505532	NR_103854	LINC01301	long intergenic non-protein codin	1.397990722	1.81790256	0.056859	0.078744	0.102792	0.26755841	0.36283659	0.45708734	8	15	18
109729182	NR_147193	DM1-AS	DM1 locus antisense RNA	1.305671359	1.81790256	0.259835	0.364693	0.489981	0.26755841	0.3412894	0.45708734	8	14	18
54622	NM_019087	ARL15	ADP ribosylation factor like GTPa	1.028004806	1.81715626	1.82988	1.876622	3.125142	2.44147625	2.47408961	3.1788613	174	239	389
29841	NM_198182	GRHL1	grainyhead like transcription facto	1.369356863	1.816501284	5.325446	7.300547	9.092532	4.29981562	4.73347472	5.12776797	734	1343	1640
81619	NM_001128309,NM_001351266	TSPAN14	tetraspanin 14	-1.410961223	1.81640229	11.50798	8.056713	19.540012	5.99747681	5.51002377	6.84840435	2469	2338	5516
340719	NM_199461	NANOS1	nanos C2HC-type zinc finger 1	1.030160034	1.8163312	0.551898	0.56894	0.943158	1.66222564	1.69172362	2.30298238	85	117	190
55276	NM_018290	PGM2	phosphoglucomutase 2	1.154817786	1.816264206	8.076735	9.33579	13.78535	4.72634668	4.92670685	5.56268791	1000	1543	2234
140733	NM_001033087,NM_001351661	MACROD2	MACRO domain containing 2	1.318710559	1.816258298	0.201519	0.265865	0.341706	0.97665845	1.18729361	1.46482043	38	67	85
87	NM_001102,NM_001130004,NM	ACTN1	actinin alpha 1	1.599363465	1.815433125	52.16964	83.577742	89.006612	7.5723345	8.24699664	8.42924928	7433	15884	16597
81025	NM_030772	GJA9	gap junction protein alpha 9	-1.333849241	1.815332609	0.226014	0.169088	0.400303	0.61813834	0.48576948	0.98063768	21	21	47
5754	NM_001270398,NM_002821,NM	PTK7	protein tyrosine kinase 7 (inactive	1.606680352	1.815126217	3.734023	6.019615	6.344511	4.03874269	4.68938608	4.85896034	606	1301	1353
9289	NM_001145770,NM_001145771	ADGRG1	adhesion G protein-coupled rece	1.433866681	1.814836831	47.34452	67.867721	81.219883	7.48087506	7.99834682	8.33709462	6974	13361	15567
6990	NM_006520	DYNLT3	dynein light chain Tctex-type 3	1.245531967	1.814811727	6.494792	8.107772	11.087157	3.91021271	4.20798559	4.72641971	551	917	1230
51360	NM_015884	MBTPS2	membrane bound transcription fa	1.361212007	1.814612789	2.261472	3.087108	3.861458	3.45742019	3.86712977	4.2570592	392	713	875
5900	NM_001042368,NM_001271774	RALGDS	ral guanine nucleotide dissociatic	1.218799953	1.814572156	15.535674	18.960456	26.499752	5.82800183	6.10890925	6.67621822	2191	3568	4890
23556	NM_012327,NM_176787	PIGN	phosphatidylinositol glycan ancho	1.094466668	1.813901662	2.996784	3.279106	5.103215	3.92722123	4.04926299	4.74325038	558	816	1245
10840	NM_001270364,NM_001270365	ALDH1L1	aldehyde dehydrogenase 1 family	1.395710923	1.812852691	0.877897	1.181888	1.473786	1.86749926	2.23214197	2.53718654	104	194	232
3092	NM_001243198,NM_005338	HIP1	huntingtin interacting protein 1	-1.203382123	1.81243919	2.594704	2.167866	4.421214	4.41453771	4.16109929	5.24191101	798	886	1779
121456	NR_033801	SLC9A7P1	solute carrier family 9 member 7	-1.41591317	1.812052611	0.134253	0.094894	0.228682	0.5190475	0.38406669	0.83750224	17	16	38
284353	NM_198478	NKPD1	NTPase KAP family P-loop doma	-1.577178063	1.81197833	0.288699	0.183558	0.494456	1.21691895	0.8787583	1.76650173	52	44	116
55777	NM_018328	MBD5	methyl-CpG binding domain prote	1.118828884	1.811948383	1.289874	1.44653	2.196359	3.09843961	3.24247658	3.87868233	297	444	662
94005	NM_033198	PIGS	phosphatidylinositol glycan ancho	1.373743917	1.811474956	11.733008	16.142204	19.978485	5.08032757	5.5268242	5.91831779	1289	2366	2872
4326	NM_016155	MMP17	matrix metallopeptidase 17	-2.71704062	1.811002759	1.844958	0.680163	3.156444	2.42103729	1.37924953	3.15222336	171	84	381

648791	NM_001145115	PPP1R3G	protein phosphatase 1 regulatory	2.040283061	1.810532856	0.72201	1.469317	1.225042	1.15225689	1.80624674	1.6852207	48	131	107
54760	NM_017573	PCSK4	proprotein convertase subtilisin/k	1.267253666	1.810090611	2.89704	3.681373	4.935253	3.11125378	3.4174117	3.890818	300	508	668
158160	NR_003086	HSD17B7P2	hydroxysteroid 17-beta dehydrog	-1.355895199	1.809511027	1.218606	0.897289	2.092618	1.42282133	1.16293478	2.01587867	66	65	147
9748	NM_001304743,NM_014720	SLK	STE20 like kinase	-1.33530573	1.80851646	11.204305	8.401996	19.069417	6.42279339	6.01123547	7.2700843	3329	3331	7405
79842	NM_024784	ZBTB3	zinc finger and BTB domain conta	1.86407859	1.808283115	1.053161	1.966205	1.790648	2.02024721	2.74415527	2.70694712	120	299	267
51148	NM_001286760,NM_016174	CERCAM	cerebral endothelial cell adhesion	-1.266319158	1.808259444	13.057181	10.504796	22.200605	5.137819	4.80802022	5.97404055	1343	1417	2987
345757	NM_198507	FAM174A	family with sequence similarity 17	1.165540897	1.808074511	10.769698	12.562576	18.306448	3.92722123	4.13472547	4.73878137	558	869	1241
115703	NM_001172630,NM_052948	ARHGAP33	Rho GTPase activating protein 3	1.015613918	1.807075499	5.9693	6.049639	10.162731	4.60475543	4.62619813	5.43176167	916	1243	2036
7138	NM_001126132,NM_001126133	TNNT1	troponin T1, slow skeletal type	1.12927821	1.806886551	17.508264	19.895459	29.996859	4.49333262	4.66140215	5.31803041	845	1275	1878
100505702	NR_038303,NR_038304,NR_03	LINC01094	long intergenic non-protein codin	5.67528562	1.806753083	0.024415	0.138716	0.037309	0.13997292	0.669904	0.24657076	4	31	9
101929357	NR_110821	HP09053	uncharacterized LOC101929357	6.950917974	1.806753083	0.039369	0.361256	0.087282	0.13997292	0.7861039	0.24657076	4	38	9
1238	NM_001296	ACKR2	atypical chemokine receptor 2	3.670720492	1.806753083	0.035353	0.131329	0.054022	0.13997292	0.46599441	0.24657076	4	20	9
101927635	NR_135596	LOC101927635	uncharacterized LOC101927635	1.666155364	1.806753083	0.16124	0.267678	0.265904	0.13997292	0.2284445	0.24657076	4	9	9
7325	NM_152653	UBE2E2	ubiquitin conjugating enzyme E2	1.711015474	1.805971339	6.636716	11.371994	11.273421	3.63059014	4.35634461	4.4305758	447	1022	993
2195	NM_005245	FAT1	FAT atypical cadherin 1	-1.394554748	1.805929437	7.905906	5.675139	13.417648	6.84111877	6.36625696	7.68824992	4462	4275	9911
2166	NM_001441	FAAH	fatty acid amide hydrolase	1.955554475	1.805753235	2.193099	4.295366	3.72413	2.45494311	3.28823882	3.18544464	176	460	391
576	NM_001294335,NM_001294336	ADGRB2	adhesion G protein-coupled rece	-1.002090982	1.805511983	1.37986	1.385824	2.361486	3.04601354	3.04336424	3.81854297	285	380	633
64130	NM_001308419,NM_022165	LIN7B	lin-7 homolog B, crumbs cell pola	-1.102714592	1.805043313	3.743943	3.384509	6.28842	1.86749926	1.76638114	2.53203014	104	126	231
3098	NM_000188,NM_001322364,NM	HK1	hexokinase 1	-1.391849258	1.804798153	15.228346	10.955051	25.913442	5.76587717	5.29919828	6.60587691	2097	2013	4655
10981	NM_006834	RAB32	RAB32, member RAS oncogene	-1.114978226	1.804444087	5.011518	4.500174	8.512474	2.78853894	2.65526948	3.54415067	232	278	515
80817	NM_001040157,NM_001145314	CEP44	centrosomal protein 44	-1.025292911	1.804022278	5.520619	5.426886	9.298667	4.65521171	4.62061907	5.48075692	950	1238	2108
10691	NM_001319674,NM_006582,NM	GMEB1	glucocorticoid modulatory elemen	1.256386848	1.803490149	2.956299	3.714902	5.009016	3.11973375	3.41483526	3.89484064	302	507	670
11070	NM_007024	TMEM115	transmembrane protein 115	1.751477391	1.803438348	10.116963	17.752298	17.160559	4.43676607	5.21654942	5.25761579	811	1898	1799
96459	NM_001008738,NM_001346113	FNIP1	folliculin interacting protein 1	1.272270734	1.803185274	6.525621	8.147825	10.871969	5.32362978	5.66332648	6.15808745	1533	2606	3400
23324	NM_001292038,NM_015274	MAN2B2	mannosidase alpha class 2B mer	1.409084225	1.801987687	13.429261	18.942617	22.740431	5.80057409	6.28781833	6.6386304	2149	4046	4763
100499489	NR_036533	LOC100499489	uncharacterized LOC100499489	2.568615031	1.801785956	0.187778	0.479494	0.314276	0.54446555	1.12560759	0.87055986	18	62	40
411	NM_000046,NM_198709	ARSB	arylsulfatase B	1.343769927	1.800847133	1.309473	1.765312	2.073407	2.99622306	3.37562608	3.76240724	274	492	607
6844	NM_001330125,NM_014232	VAMP2	vesicle associated membrane pro	1.244692492	1.799890927	5.599156	7.057211	9.165665	3.70291935	3.99682365	4.50088019	472	785	1045
79085	NM_024103	SLC25A23	solute carrier family 25 member 2	1.113748445	1.79884118	6.366339	7.096628	10.759465	4.48842972	4.63729194	5.30675218	842	1253	1863
3652	NM_001145349,NM_005897	IPP	intracisternal A particle-promoted	-1.048230564	1.79835871	1.673495	1.571361	2.751992	2.56460348	2.50832973	3.29907966	193	246	427
195814	NM_001318049,NM_001318050	SDR16C5	short chain dehydrogenase/reduc	-1.731457091	1.798302413	1.817329	1.054017	3.124565	2.85607368	2.20257223	3.61169701	245	189	542
83737	NM_001257137,NM_001257138	ITCH	itchy E3 ubiquitin protein ligase	1.331821707	1.797844853	13.748256	18.190822	23.278612	6.52209318	6.93158852	7.36139864	3569	6351	7892
6415	NM_003009	SELENOW	selenoprotein W	1.245226766	1.797572272	58.074718	72.411118	98.127213	5.67888953	5.98975653	6.5124257	1972	3281	4360
440300	NR_033738	LOC440300	chondroitin sulfate proteoglycan 4	-1.133102858	1.797135302	0.093429	0.082077	0.157756	0.77668602	0.70406985	1.19154816	28	33	62
56261	NM_019593	GPCPD1	glycerophosphocholine phosphod	-1.17443235	1.797132007	6.055049	5.160917	10.229095	5.05291493	4.82849184	5.87925987	1264	1438	2794
100616396	NR_039862	MIR4712	microRNA 4712	1.080119318	1.796853568	2.745007	2.967934	4.727965	0.29776659	0.31941552	0.49997203	9	13	20
23200	NM_014616	ATP11B	ATPase phospholipid transporting	1.111997802	1.796820058	12.932283	14.398143	21.837957	6.54194749	6.69354544	7.38053068	3619	5377	7998
54865	NM_015590,NM_182679	GPATCH4	G-patch domain containing 4	1.130617204	1.796654641	6.812097	7.724976	11.514179	3.93685111	4.10306728	4.73989992	562	849	1242
9069	NM_001185072,NM_001185073	CLDN12	claudin 12	-1.523417674	1.79655454	17.747756	11.662183	29.83709	5.98998006	5.39446891	6.82514211	2456	2154	5427
56005	NM_019107	MYDGF	myeloid derived growth factor	1.092721135	1.79640629	72.822662	79.656418	122.935234	6.2389485	6.36525647	7.0755933	2926	4272	6465
644945	NR_029393	KRT16P3	keratin 16 pseudogene 3	-1.653598253	1.795705378	0.7615	0.455094	1.278721	1.29387258	0.90836632	1.85174813	57	46	126
10592	NM_001042550,NM_001042551	SMC2	structural maintenance of chromo	1.263190426	1.795505422	11.239181	14.158188	18.917422	6.05553602	6.38809405	6.89031129	2572	4341	5680
55872	NM_001278945,NM_018492	PBK	PDZ binding kinase	1.13040943	1.795228408	22.0245	24.940929	37.176125	5.53651876	5.70978368	6.36689119	1783	2693	3937
83641	NM_001282695,NM_001282696	FAM107B	family with sequence similarity 10	1.1155703	1.79522673	26.193938	28.681018	44.340589	6.42364978	6.57969385	7.26037344	3331	4965	7355
6655	NM_006939	SOS2	SOS Ras/Rho guanine nucleotide	1.242117273	1.79487897	5.076119	6.310053	8.562301	4.84999531	5.15303948	5.67162056	1093	1814	2413
79767	NM_024712	ELMO3	engulfment and cell motility 3	-1.021027752	1.794865537	20.211235	19.81497	34.093468	5.6564798	5.6270574	6.48766934	1941	2540	4285
644815	NM_001039999	FAM83G	family with sequence similarity 83	1.568381166	1.794011249	1.760567	2.774219	2.975444	2.59533164	3.15564136	3.3291189	198	415	437
9625	NM_001080395,NM_004920	AATK	apoptosis associated tyrosine kin	-1.099499183	1.79396896	0.065987	0.060039	0.115311	0.43999062	0.40498891	0.71544405	14	17	31
6709	NM_001130438,NM_001195532	SPTAN1	spectrin alpha, non-erythrocytic 1	1.385246135	1.793927813	32.808703	45.482687	55.307614	7.98349276	8.45205324	8.82409682	9897	18318	21837
22900	NM_001184900,NM_001184901	CARD8	caspase recruitment domain fam	-1.027356001	1.79388961	6.629494	4.960491	8.685302	4.61828417	4.5809496	5.4352184	925	1203	2041
250	NM_001632	ALPP	alkaline phosphatase, placental	1.273574871	1.793814943	9.384023	11.965262	15.82083	4.81517582	5.15303948	5.63541459	1066	1814	2352
8661	NM_003750	EIF3A	eukaryotic translation initiation fa	1.302790753	1.793737752	31.664093	41.29464	53.373478	7.36238208	7.74195387	8.20147727	6421	11177	14166
28231	NM_016354	SLCO4A1	solute carrier organic anion trans	-1.128156658	1.793250562	16.352322	14.51314	27.566895	5.49882028	5.32892486	6.3272551	1736	2056	3829
8546	NM_001271769,NM_003664	AP3B1	adaptor related protein complex 3	1.177119568	1.792769771	13.616787	16.052164	22.94714	5.76924991	6.00053598	6.59972899	2102	3306	4635
440145	NM_001071775	MZT1	mitotic spindle organizing protein	-1.027573306	1.7926812	8.193359	7.981151	13.81272	4.34028863	4.30300447	5.15065778	756	983	1667
54206	NM_018948	ERRFI1	ERBB receptor feedback inhibitor	1.776619845	1.792355911	65.084274	115.764671	109.629921	7.63874415	8.46471989	8.47740669	7785	18480	17162
128637	NM_144628,NR_111901	TBC1D20	TBC1 domain family member 20	1.398467225	1.792216963	12.343553	17.447777	21.185983	5.71011084	6.186105	6.53965765	2016	3767	4444
84667	NM_001165967,NM_032580	HES7	hes family bHLH transcription fac	-3.99191327	1.791743491	1.205139	0.292856	2.042455	1.57857476	0.58077243	2.18966672	78	26	172
23232	NM_015188	TBC1D12	TBC1 domain family member 12	-1.669664417	1.791668298	1.110574	0.667347	1.875386	2.85607368	2.24380223	3.6068006	245	196	540
4091	NM_005585,NR_027654	SMAD6	SMAD family member 6	-1.142825018	1.791522496	1.272558	1.115757	2.144599	2.20692365	2.05815489	2.90342651	142	166	313
154064	NM_130900	RAET1L	retinoic acid early transcript 1L	1.726460892	1.791260307	0.917762	1.620844	1.587054	0.79797761	1.18729361	1.21747621	29	67	64
100132273	NR_034118	NDUFA6-AS1	NDUFA6 antisense RNA 1 (head	1.006491339	1.791260307	0.449161	0.456456	0.767321	0.79797761	0.80196545	1.21747621	29	39	64
10866	NR_040662	HCP5	HLA complex P5 (non-protein co	-1.459149055	1.791052292	3.900947	2.671236	6.573143	3.41326233	2.92884759	4.19316234	379	347	835
81610	NM_030919	FAM83D	family with sequence similarity 83	1.433944544	1.790626415	25.115641	36.052128	42.257431	5.91644377	6.42920612	6.74635311	2332	4468	5136
29766	NM_014547	TMOD3	tropomodulin 3	-1.080547428	1.790260967	22.040422	20.421261	37.085785	6.66038669	6.54976952	7.49426505	3932	4862	8658
3106	NM_005514	HLA-B	major histocompatibility complex,	-1.085678256	1.789546682	235.547409	217.195541	396.105621	8.53316782	8.41490391	9.3710477	14505	17851	31926
51684	NM_001178133,NM_016169	SUFU	SUFU negative regulator of hedg	-1.003896335	1.787720303	2.43843	2.484209	4.296868	3.53871843	3.53359042	4.32123795	417	555	917
29988	NM_001271711,NM_001271712	SLC2A8	solute carrier family 2 member 8	1.225629144	1.787431937	2.760004	3.146491	4.821908	2.5831188	2.83174937	3.31117052	196	321	431
51762	NM_016530	RAB8B	RAB8B, member RAS oncogene	1.4112705	1.786654513	1.921177	2.71841	3.23206	3.34257292	3.79763131	4.11600233	359	677	789
80176	NM_025106	SPSB1	splA/ryanodine receptor domain a	1.149343422	1.786566193	3.523733	4.055598	5.915458	3.54817338	3.7329126	4.33017765	420	645	923
4035	NM_002332	LRP1	LDL receptor related protein 1	2.096005403	1.786130432	1.498578	3.144591	2.515489	4.50632616	5.54047969	5.3150315	853	2389	1874
83401	NM_152310	ELOVL3	ELOVL fatty acid elongase 3	-1.389473751	1.78519085	0.45653	0.330047	0.77181	0.71085839	0.54351856	1.09694451	25	24	55
10043	NM_001135729,NM_001135730	TOM1	target of myb1 membrane traffick	-1.14607237	1.785104538	10.658888	9.304608	17.823077	4.68551669	4.49696276	5.49672576	971	1132	2132
8519	NM_003641	IFITM1	interferon induced transmembran	-1.181091966	1.784736497	33.629154	28.510098	56.404137	4.61378867	4.38426973	5.42343154	922	1043	2024
7555	NM_001127192,NM_001127193	CNBP	CCHC-type zinc finger nucleic ac	1.739526698	1.784327768	15.976891	27.828265	26.802551	5.74067079	6.52788076	6.56417661	2060	4788	4521
163255	NM_001172225,NM_001172226	ZNF540	zinc finger protein 540	2.08978529	1.784315472	0.151013	0.338972	0.270558	0.59399636	1.04792463	0.93448595	20	56	44
79068	NM_001080432	FTO	FTO, alpha-ketoglutarate depend	1.416839785	1.784223163	5.415876	7.684294	9.0848	4.56339093	5.04805279	5.37169332	889	1683	1951
9368	NM_004252	SLC9A3R1	SLC9A3 regulator 1	1.152680068	1.784221564	23.272913	26.864466	39.029499	5.54520135	5.74610648	6.36689119	1794	2763	3937
647979	NR_027451	NORAD	non-coding RNA activated by DN	-1.078048357	1.783738938	62.523582	58.062599	104.804962	8.34990506	8.2418273	9.18287092	12770	15827	28016
54536	NM_001013848,NM_001319194	EXOC6	exocyst complex component 6	-2.113714504	1.783367943	4.494252	2.129367	7.535314	4.08061013	3.09255667	4.87737422	625	395	1371
79414	NM_024509	LRFN3	leucine rich repeat and fibronecti	1.099403172	1.783329424	2.676944	2.950641	4.49253	2.92058788	3.04002433	3.66919325	258	379	566
102465438	NR_106790	MIR6732	microRNA 6732	1.840144439	1.782860839	6.164736	11.770676	10.660408	0.46682716	0.77006602	0.75138414	15	37	33
109623451	NR_145781	SNORD157	small nucleolar RNA, C/D box 15	1.394264822	1.782860839	5.706114	7.925698	9.417883	0.46682716	0.61708849	0.75138414	15	28	33
10459	NM_001127325,NM_006341	MAD2L2	mitotic arrest deficient 2 like 2	1.720837778	1.782859735	9.427393	16.245414	15.822327	3.49701545	4.22573375	4.27414582	404	929	886
55283	NM_001253693,NM_018298	MCOLN3	mucolipin 3	-1.620734765	1.782859735	3.705186	2.286406	6.331879	3.49701545	2.87737723	4.27414582	404	333	886
388567	NM_001023561,NM_001321952	ZNF749	zinc finger protein 749	1.44966027	1.782835033	4.737993	6.904811	7.88214	4.35113248	4.86481307	5.15401818	762	1476	1671
5528	NM_001270476,NM_006245,NM	PPP2R5D	protein phosphatase 2 regulatory	-1.029383372	1.782752397	10.189815	10.004771	17.223176	4.96623093	4.92580232	5.77998839	1188	1542	2605
253832	NM_001286638,NM_001330059	ZDHHC20	zinc finger DHHC-type containing	1.125435786	1.782680954	12.434626	13.970833	20.784356	6.04277245	6.21082175	6.86720853	2549	3833	5589
26227	NM_006623	PHGDH	phosphoglycerate dehydrogenas	1.294586159	1.781506838	46.443195	60.196468	77.765213	6.52568718	6.89462039	7.35191952	3578	6189	7840
154043	NM_173515	CNKSR3	CNKSR family member 3	1.810477556	1.781469328	0.453369	0.826246	0.763637	1.29387258	1.86027027	1.84344662	57	138	125
6117	NM_002945	RPA1	replication protein A1	1.23468855	1.781344193	13.487295	16.678249	22.582829	5.85998825	6.15941912	6.68204931	2241	3697	4910
4001	NM_001198557,NM_005573,NR	LMNB1	lamin B1	1.301648901	1.78122697	52.645595	100.842823	114.996418	7.45618555	7.83462594	8.28545945	6855	11922	15018
9764	NM_001286565,NM_001286566	KIAA0513	KIAA0513	-1.8395782	1.780794963	2.00199	0.969445	3.961985	3.79050499	2.99588686	4.57670807	504	366	1104
729678	NR_027183,NR_045678,NR_04	LINC00847	long intergenic non-protein codin	1.292893195	1.780778695	3.564095	4.613433	5.926408	2.91572639	3.24247658	3.66213029	257	444	563
1382	NM_001199723,NM_001878	CRABP2	cellular retinoic acid binding prote	-2.195898379	1.780611048	287.713072	131.163588	481.455755	8.21332773	7.08430211	9.04357366	11613	7066	25433
11253	NM_016219,NR_045720,NR_04	MAN1B1	mannosidase alpha class 1B mer	1.233587041	1.780442194	30.790196	38.03959	51.520948	6.39074431	6.69035332	7.21544508	3255	5365	7128
64856	NM_022834,NM_199121	VWA1	von Willebrand factor A domain c	1.2191332	1.78037041	17.995113	22.291177	30.094194	6.3610997	6.64380598	7.18558585	3188	5193	6981
23769	NM_013280	FLRT1	fibronectin leucine rich transmem	1.047302025	1.779967701	0.080571	0.081766	0.133175	0.3273552	0.3412894	0.54161868	10	14	22
30837	NM_014598	SOCS7	suppressor of cytokine signaling	1.187955402	1.779886778	4.017155	4.780585	6.722347	5.01022388	5.25162377	5.82231668	1226	1946	2684

5869	NM_001252036,NM_001252037	RAB5B	RAB5B, member RAS oncogene	-1.207748758	1.779878337	14.710234	12.210503	24.700369	6.27070749	6.00225324	7.09429903	2992	3310	6550
3107	NM_001243042,NM_002117	HLA-C	major histocompatibility complex,	-1.029012006	1.779800742	342.991372	333.694489	573.661339	9.05160653	9.01042537	9.88213398	20794	27000	45519
49854	NM_001098402,NM_001098403	ZBTB21	zinc finger and BTB domain cont	-1.00893832	1.779260176	2.410841	2.388862	4.007558	4.16086733	4.14874812	4.95648442	663	878	1451
5833	NM_001184917,NM_001256433	PCYT2	phosphate cytidylyltransferase 2,	-1.242021514	1.779171175	13.956975	11.472556	23.509008	5.62703993	5.32137849	6.44544065	1901	2045	4160
57619	NM_020859	SHROOM3	shroom family member 3	-1.093880196	1.778736941	3.386647	3.100037	5.66171	5.22293093	5.09708829	6.03682106	1427	1743	3122
85440	NM_001271999,NM_001272000	DOCK7	dedicator of cytokinesis 7	1.202524743	1.778014552	3.325592	4.098256	5.445866	4.46698912	4.72205729	5.26850829	829	1332	1813
8692	NM_003773,NM_033158	HYAL2	hyaluronoglucosaminidase 2	1.142031322	1.777175951	18.192896	19.13179	28.741366	5.33368816	5.52084648	6.14758984	1544	2356	3375
59272	NM_021804	ACE2	angiotensin I converting enzyme	-3.463294403	1.776821496	0.149195	0.044714	0.260091	0.61813834	0.20477631	0.96541723	21	8	46
283554	NM_001099652	GPR137C	G protein-coupled receptor 137C	1.526879	1.776734676	0.321246	0.492476	0.53884	1.15225689	1.52015496	1.66651905	48	98	105
80325	NM_032548,NM_172027,NR_03	ABTB1	ankyrin repeat and BTB domain c	1.367071634	1.776321917	5.663119	7.816657	9.438684	3.56999691	3.98818428	4.34495514	427	780	933
5045	NM_001289823,NM_001289824	FURIN	furin, paired basic amino acid cle	1.189385673	1.775959391	12.450867	14.655739	20.699473	5.69600304	5.94179364	6.5124257	1996	3172	4360
602	NM_005178	BCL3	B-cell CLL/lymphoma 3	1.087049868	1.774762621	9.730227	10.590074	16.23728	4.22318689	4.33742297	5.01679049	694	1008	1515
9601	NM_004911	PDIA4	protein disulfide isomerase family	-1.272140162	1.774417418	103.078423	81.117905	171.89035	8.21567825	7.86973711	9.04091017	11632	12217	25386
25790	NM_012337	CFAP45	cilia and flagella associated prote	1.525006215	1.774394963	1.901327	2.90832	3.174726	2.11660268	2.60642858	2.79136857	131	267	286
89846	NM_001083536,NM_001286993	FGD3	FYVE, RhoGEF and PH domain c	1.178963228	1.774129499	8.12582	9.516671	13.52028	4.78483717	5.01441136	5.58899591	1043	1643	2276
257106	NM_001025598,NM_001287600	ARHGAP30	Rho GTPase activating protein 30	-3.08512449	1.77384247	0.869779	0.280542	1.45291	2.25390578	1.15059938	2.94281592	148	64	323
55103	NM_001286247,NM_152663	RALGPS2	Ral GEF with PH domain and SH	-1.015497448	1.773312656	12.475588	12.298659	20.780537	6.54115854	6.51921133	7.36085345	3617	4759	7889
54471	NM_001304564,NM_019008,NR	MIEF1	mitochondrial elongation factor 1	1.767459856	1.773047049	6.370672	11.406824	10.440119	5.15028961	5.95426739	5.95874997	1355	3200	2955
6596	NM_001318934,NM_001318935	HLTF	helicase like transcription factor	1.566698276	1.772580226	12.744769	20.431871	21.572658	6.00092376	6.64050176	6.81695064	2475	5181	5396
81618	NM_001012514,NM_001012516	ITM2C	integral membrane protein 2C	2.3157667	1.771851875	24.910503	60.309072	40.978861	5.67241926	6.86779052	6.48533693	1963	6074	4278
4191	NM_001282403,NM_001282404	MDH2	malate dehydrogenase 2	-1.328041371	1.771811556	50.384024	36.940957	78.679986	6.66691103	6.26224839	7.48595581	3950	3974	8608
4209	NM_001271629,NM_005920	MEF2D	myocyte enhancer factor 2D	1.003467849	1.771740901	8.459377	8.536646	14.277408	5.64917596	5.65407127	6.46180299	1931	2589	4208
55277	NM_001113411,NM_001244714	FGGY	FGGY carbohydrate kinase doma	1.189810795	1.771711557	1.902733	2.121091	3.020811	2.18284609	2.38212036	2.86293141	139	221	303
4293	NM_001284230,NM_001284231	MAP3K9	mitogen-activated protein kinase	1.018155489	1.771584365	3.972149	4.054445	6.52741	5.44921723	5.47458779	6.25984475	1676	2280	3652
100129424	NR_026660	RPL19P12	ribosomal protein L19 pseudoger	1.633007534	1.771415181	0.106784	0.187486	0.178532	0.17294258	0.27464693	0.29607837	5	11	11
8395	NM_001278253,NM_003558	PIP5K1B	phosphatidylinositol-4-phosphate	-3.184155122	1.771415181	0.035758	0.013264	0.074626	0.17294258	0.05398282	0.29607837	5	2	11
114814	NR_002328,NR_104033,NR_104	GNRHR2	gonadotropin releasing hormone	-1.99769917	1.771308876	1.088915	0.478378	1.168333	0.88013425	0.50527715	1.31676564	33	5	72
10135	NM_005746	NAMPT	nicotinamide phosphoribosyltrans	-1.011426039	1.771109572	19.334267	19.136213	32.179729	6.44700339	6.43080098	7.26445997	3386	4473	7376
10379	NM_006084	IRF9	interferon regulatory factor 9	-1.114634871	1.770881082	28.788393	25.861074	47.918167	5.58631849	5.43317608	6.39769249	1847	2214	4023
3718	NM_000215	JAK3	Janus kinase 3	-10.68685049	1.770665481	0.888759	0.079546	1.478885	2.51403512	0.52452456	3.22432601	185	23	403
28970	NM_001286067,NM_001286068	C11orf54	chromosome 11 open reading fra	-1.498241988	1.770616534	8.172434	4.685684	13.289921	4.93773843	4.37766975	5.74141559	1164	1038	2535
5738	NM_020440	PTGFRN	prostaglandin F2 receptor inhibito	1.431325458	1.770538979	6.247646	8.954988	10.397244	5.30050562	5.80681389	6.10872026	1508	2884	3284
102465451	NR_106811	MIR6753	microRNA 6753	2.374695384	1.770005335	3.392563	8.278974	5.741618	0.64188297	1.22307764	0.99569924	22	70	48
5663	NM_000021,NM_007318	PSEN1	presenilin 1	1.250254441	1.769256931	13.826315	17.298461	22.977277	6.37267601	6.69141814	7.18825152	3214	5369	6994
64112	NM_022151	MOAP1	modulator of apoptosis 1	1.452196126	1.76921377	8.163014	11.87337	13.579853	4.33847337	4.85439274	5.13032929	755	1465	1643
148898	NR_033690,NR_033691	ZNF436-AS1	ZNF436 antisense RNA 1	1.628615803	1.769158676	1.853542	3.333211	3.373537	2.63136183	3.24247658	3.34978031	204	444	444
84247	NM_032287	RTL6	retrotransposon Gag like 6	1.681085534	1.768941026	1.390762	2.348699	2.320015	3.04155747	3.71835988	3.78642406	284	638	618
387644	NR_026795	FAM238C	family with sequence similarity 23	3.145914998	1.768213571	0.196135	0.635234	0.332518	0.77668602	1.70021226	1.17840727	28	118	61
23467	NM_014293	NPTXR	neuronal pentraxin receptor	2.477007058	1.767893354	0.227129	0.569077	0.378945	1.2010231	2.07783221	1.72191206	51	169	111
847	NM_001752	CAT	catalase	1.208562195	1.767728425	15.199556	18.3916	25.257877	5.13154304	5.39773447	5.93550587	1337	2159	2907
389792	NM_203434	IER5L	immediate early response 5 like	1.350747524	1.767422374	10.433925	14.111005	17.329418	4.83462363	5.25522879	5.63421199	1081	1951	2350
6525	NM_001207017,NM_001207018	SMTN	smoothelin	1.197945291	1.767131171	15.422269	18.497869	25.586249	5.66157057	5.91742821	6.47058904	1948	3118	4234
84926	NM_032840	SPRYD3	SPRY domain containing 3	-1.149248598	1.766882138	5.813783	5.067259	9.657757	4.13809052	3.94954414	4.92339112	652	758	1417
9052	NM_003979	GPRC5A	G protein-coupled receptor class	-1.737124858	1.766233101	309.57843	178.41124	513.836426	9.74928267	8.95381295	10.56923311	33750	25959	73317
440093	NM_001013699	H3F3C	H3 histone family member 3C	1.896385267	1.766121016	0.251916	0.504927	0.44533	0.35634914	0.61708849	0.58209675	11	28	24
100616262	NR_039850	MIR4701	microRNA 4701	-1.049306843	1.766121016	4.304708	4.349754	7.382119	0.35634914	0.3412894	0.58209675	11	14	24
102465511	NR_106907	MIR6848	microRNA 6848	-3.582038262	1.766121016	3.782418	0.849986	6.698609	0.35634914	0.10601836	0.58209675	11	4	24
26286	NM_001142293,NM_014570	ARFGAP3	ADP ribosylation factor GTPase a	-1.201844271	1.765949109	7.026981	5.84672	11.658009	4.34933082	4.09825806	5.13883433	761	846	1653
220930	NR_024284	ZEB1-AS1	ZEB1 antisense RNA 1	-1.098051898	1.765860035	2.3983	2.186308	3.978104	2.62541894	2.5131555	3.34096163	203	247	441
442582	NR_040584	STAG3L2	stromal antigen 3-like 2 (pseudog	-1.127446771	1.765375719	8.171971	7.257976	13.560772	4.00022383	3.83857782	4.78068826	589	698	1279
8728	NM_033274	ADAM19	ADAM metallopeptidase domain	1.12969618	1.764859535	10.418941	11.783998	17.281937	6.07146991	6.24494647	6.88172272	2601	3926	5646
3703	NM_001278503,NM_001278504	STT3A	STT3A, catalytic subunit of the ol	-1.523061835	1.76468955	33.333393	21.910933	55.143263	7.11021619	6.50867837	7.9251116	5385	4724	11688
51341	NM_001317990,NM_015898	ZBTB7A	zinc finger and BTB domain cont	1.139415988	1.764552504	3.651883	4.159959	6.068498	4.56804599	4.74889974	5.36082844	892	1358	1936
100996712	NR_120535,NR_120535_dup1	SRGAP2D	SLIT-ROBO Rho GTPase activat	1.363387101	1.764006602	2.799921	3.827645	4.652636	2.86618866	3.25980797	3.59695765	247	450	536
51379	NM_015986,NR_073118	CRLF3	cytokine receptor like factor 3	-1.007086231	1.763647973	6.778941	6.742758	11.17001	4.34028863	4.33060465	5.12776797	756	1003	1640
57826	NM_001271186,NM_001271187	RAP2C	RAP2C, member of RAS oncoge	1.041717019	1.763534999	7.205843	7.497704	11.958795	4.83591088	4.89285546	5.63240621	1082	1506	2347
84078	NM_032138	KBTBD7	kelch repeat and BTB domain co	-1.220586606	1.763207114	1.101323	0.904875	1.832883	2.61345921	2.37683491	3.32614297	201	220	436
9908	NM_012297,NM_203504,NM_20	G3BP2	G3BP stress granule assembly fa	1.472524055	1.763143177	8.708435	12.976364	14.306345	5.24247649	5.78852012	6.04408383	1447	2847	3138
23654	NM_012401	PLXNB2	plexin B2	1.19314451	1.763141086	48.753242	58.232937	80.779076	8.23702779	8.49102443	9.05311049	11806	18821	25602
1040	NM_001263	CDS1	CDP-diacylglycerol synthase 1	-1.093021345	1.763126009	10.496676	9.61332	17.391932	5.56629422	5.44079444	6.37122872	1821	2226	3949
1728	NM_000903,NM_001025433,NM	NQO1	NAD(P)H quinone dehydrogenas	1.51269072	1.762944577	108.895269	161.568055	176.831873	8.0682177	8.66351676	8.88388337	10498	21218	22763
5550	NM_002726	PREP	prolyl endopeptidase	1.016292904	1.762924153	6.748184	6.87075	11.189477	4.34752691	4.36970972	5.13458808	760	1032	1648
10411	NM_001098531,NM_001098532	RAPGEF3	Rap guanine nucleotide exchang	-2.142975335	1.76263391	1.872748	0.881885	3.155134	3.62762016	2.65526948	4.39409566	446	278	967
11240	NM_007365	PADI2	peptidyl arginine deiminase 2	-1.868313921	1.762260301	0.458321	0.24354	0.763219	1.56621863	1.03456085	2.15654364	77	55	167
3134	NM_001098478,NM_001098479	HLA-F	major histocompatibility complex,	-1.465318234	1.761728069	31.944327	21.808764	52.801433	5.36255637	4.82752356	6.16434954	1576	1437	3415
4919	NM_001083592,NM_005012	ROR1	receptor tyrosine kinase like orph	1.044965788	1.760777083	1.183566	1.256974	2.191449	2.9541672	3.009612	3.68786049	265	370	574
3708	NM_001099952,NM_001168272	ITPR1	inositol 1,4,5-trisphosphate recep	3.352800181	1.760318775	0.166915	0.567167	0.279941	1.42282133	2.73178769	1.98602166	66	296	143
10217	NM_001008392,NM_005808	CTDSPL	CTD small phosphatase like	-1.065618557	1.76005709	11.105208	10.44404	18.397984	5.61659238	5.5268242	6.4194971	1887	2366	4085
10765	NM_001314042,NM_001347591	KDM5B	lysine demethylase 5B	1.888445509	1.759993329	5.589536	10.586642	9.259149	5.74341713	6.64792565	6.54734471	2064	5208	4468
84905	NM_001282933,NM_001282935	ZNF341	zinc finger protein 341	-1.216884088	1.759549105	4.719987	3.82571	7.687073	4.09788139	3.83279899	4.87635737	633	695	1370
23731	NM_032012	TMEM245	transmembrane protein 245	1.024203701	1.75889621	16.760111	17.185207	27.70649	7.03907211	7.07331623	7.84901515	5124	7012	11085
84236	NM_001167608,NM_001349069	RHBDD1	rhomboid domain containing 1	1.051826105	1.758896061	4.938365	4.941079	8.173092	4.62574568	4.69576755	5.41505304	930	1307	2012
162989	NM_001270614,NM_001270615	DEDD2	death effector domain containing	-1.069050526	1.758634162	18.972676	18.489935	30.273569	5.14406778	5.05054456	5.94086581	1349	1686	2918
79623	NM_001253826,NM_001253827	GALNT14	polypeptide N-acetylgalactosamir	1.249578822	1.7585496	13.015342	16.211767	21.474604	5.15132397	5.4646572	5.94814289	1356	2264	2933
55917	NM_018704	CTTNBP2NL	CTTNBP2 N-terminal like	1.083745722	1.758445732	3.152783	3.42507	5.214157	4.25817433	4.36837876	5.03967945	712	1031	1540
203328	NM_001287005,NM_001287006	SUSD3	sushi domain containing 3	1.079569121	1.757995732	1.289626	1.414228	2.149396	1.35261029	1.42093184	1.90057841	61	88	132
56925	NM_020169	LXN	latexin	-1.325986916	1.757915872	46.089645	34.797329	76.161766	5.67529852	5.27738104	6.47697595	1967	1982	4253
857	NM_001172895,NM_001172896	CAV1	caveolin 1	1.457487887	1.757806782	103.014368	147.53418	166.533745	8.07776997	8.61958137	8.88924918	10568	20580	22848
2309	NM_001455,NM_201559	FOXO3	forkhead box O3	1.016827211	1.757687938	8.694985	8.850644	14.368346	5.98069949	6.00439694	6.78452487	2440	3315	5275
10434	NM_001279356,NM_001279357	LYPLA1	lysophospholipase I	-1.249458564	1.757618115	20.321073	16.209529	33.402252	5.69174366	5.37736782	6.49331813	1990	2128	4302
23351	NM_001290256,NM_001290257	KHNYN	KH and NYN domain containing	1.114320021	1.757476264	16.429653	18.417613	27.180541	6.66835688	6.82306857	7.47575329	3954	5887	8547
83875	NM_001037290,NM_001256397	BCO2	beta-carotene oxygenase 2	-2.574796936	1.757453023	1.936786	0.752818	3.454254	2.70083015	1.64851465	3.4156568	216	112	467
57462	NM_020702	KIAA1161	KIAA1161	1.232579635	1.757433675	2.056843	2.53719	3.39688	3.79844995	4.08048581	4.56665693	507	835	1096
28999	NM_014079	KLF15	Kruppel like factor 15	-1.201072603	1.756896052	0.181643	0.155183	0.306222	0.54446555	0.46599441	0.85412573	18	20	39
26090	NM_001042472,NM_015600	ABHD12	abhydrolase domain containing 1	1.308768948	1.756508922	31.887861	41.586375	52.000899	6.00779304	6.39071865	6.8108429	2487	4349	5373
10126	NM_005740	DNAL4	dynein axonemal light chain 4	1.242819774	1.756392131	3.938118	4.892215	6.492622	2.74535098	3.01642592	3.4624849	224	372	484
152926	NM_152542	PPM1K	protein phosphatase, Mg2+/Mn2+	-2.026782124	1.75631974	1.383196	0.681779	2.286261	3.30588543	2.42883772	4.05439856	349	230	754
10026	NM_005482	PIGK	phosphatidylinositol glycan ancho	1.180508601	1.756217427	9.325119	11.022813	15.394186	5.42632706	5.66061052	6.22432111	1649	2601	3562
6560	NM_001145961,NM_001145962	SLC12A4	solute carrier family 12 member 4	1.345294436	1.755839208	7.961947	10.717164	13.147528	5.24247649	5.66061052	6.03818562	1447	2601	3125
900	NM_004060,NM_199246	CCNG1	cyclin G1	-1.071730401	1.75514465	27.241657	25.517646	45.032457	6.05443061	5.95604059	6.85667747	2570	3204	5548
64077	NM_001167880,NM_001318331	LHPP	phospholysine phosphohistidine i	1.205935524	1.755029358	2.823925	3.477172	4.356762	2.32162912	2.541776	3.00368412	157	253	339
83941	NM_032027,NR_135160,NR_13	TM2D1	TM2 domain containing 1	1.102161167	1.754479783	13.391474	14.775292	22.231094	4.15055879	4.28336529	4.92634185	658	969	1420
10447	NM_001040020,NM_014888	FAM3C	family with sequence similarity 3	1.131548441	1.753669347	53.075362	60.081814	87.494621	7.04074763	7.21777506	7.84642114	5130	7756	11065
255512	NR_029409	TOLLIP-AS1	TOLLIP antisense RNA 1 (head t	1.546569176	1.753530836	1.142548	1.768804	1.896708	1.08456011	1.45142181	1.56919173	44	91	95
441459	NM_001244752	ANKRD18B	ankyrin repeat domain 18B	-1.672376226	1.752890327	0.930834	0.555078	1.533881	2.14180175	1.60397149	2.80425801	134	107	289
8732	NM_001286426,NM_001286428	RNGTT	RNA guanylyltransferase and 5'-p	1.231548714	1.752855738	2.561222	3.164388	4.235579	3.70855202	3.98818428	4.47020574	474	780	1022
5796	NM_001135648,NM_001291981	PTPRK	protein tyrosine phosphatase, rec	1.450576847	1.752722304	8.697862	12.620038	14.309399	5.71431642	6.24240584	6.5120984	2022	3919	4359
84061	NM_032121	MAGT1	magnesium transporter 1	1.3048339	1.752494462	21.044596	27.491314	34.662518	6.38195877	6.76178901	7.18394299	3235	5640	6973
4248	NM_001098270,NM_002409	MGAT3	mannosyl (beta-1,4-)-glycoprotei	-1.624794179	1.752405981	2.600855	1.604138	4.289986	3.76640418	3.13073752	4.52962301	495	407	1067

23064	NM_001351527,NM_001351528	SETX	senataxin	1.791543707	1.752327962	6.745133	12.152422	11.152278	6.21886148	7.05150499	7.01981835	2885	6906	6218
5834	NM_002862	PYGB	glycogen phosphorylase B	1.388412565	1.751600792	34.083817	47.379395	56.108944	7.10968415	7.58020322	7.91388174	5383	9986	11597
100272217	NR_027440	LOC100272217	uncharacterized LOC100272217	1.280516484	1.750963377	1.178221	1.512703	1.939086	1.79522562	2.05815489	2.41366048	97	166	209
28983	NM_014058	TMPRSS11E	transmembrane protease, serine	-1.040547838	1.75068857	2.05775	1.989093	3.398735	2.40724866	2.36086138	3.09392991	169	217	364
162972	NM_001277090,NM_001277091	ZNF550	zinc finger protein 550	1.028388969	1.750484932	10.254068	10.413666	16.845054	5.25215086	5.29149439	6.04363098	1457	2002	3137
100507588	NM_001195259	TGFBR3L	transforming growth factor beta r	-1.775175879	1.750467453	2.426583	1.368031	4.002427	1.9928118	1.42093184	2.63662357	117	88	252
400961	NM_020459	PAIP2B	poly(A) binding protein interacting	1.551881001	1.74984817	0.839953	1.309799	1.383912	2.62541894	3.17404113	3.3291189	203	421	437
200081	NM_175852	TXLNA	taxilin alpha	1.563225741	1.749692991	9.900049	15.492608	16.27586	5.56163349	6.19514198	6.35562515	1815	3791	3906
389136	NM_001320493,NM_001320494	VGLL3	vestigial like family member 3	1.706651631	1.749628835	0.849816	1.441963	1.382418	3.25290323	3.96018541	3.99380211	335	764	721
529	NM_001039366,NM_001039367	ATP6V1E1	ATPase H+ transporting V1 subu	-1.012032302	1.749575136	28.51398	28.260335	46.855109	5.31811369	5.30129222	6.10958589	1527	2016	3286
114899	NM_030945,NM_181435,NR_14	C1QTNF3	C1q and TNF related 3	1.549008363	1.749462264	0.514121	0.802952	0.851134	1.66222564	2.12272838	2.25996639	85	176	183
10463	NM_006345	SLC30A9	solute carrier family 30 member 9	1.19034765	1.749217513	17.159031	20.444788	28.204689	5.79396609	6.04119638	6.5895273	2139	3402	4602
5351	NM_000302,NM_001316320	PLOD1	procollagen-lysine,2-oxoglutarate	1.949666616	1.74915219	38.536771	75.307673	63.71163	6.85292913	7.81008115	7.65424495	4499	11720	9679
3918	NM_005562,NM_018891	LAMC2	laminin subunit gamma 2	1.020278807	1.748867199	113.911829	116.219265	187.332382	9.27629956	9.30521689	10.08172654	24305	33133	52280
81490	NM_001329544,NM_001329545	PTDSS2	phosphatidylserine synthase 2	1.594638292	1.748504347	5.111003	8.145259	8.582002	3.64240927	4.27202169	4.39835229	451	961	970
55589	NM_017593,NM_198892	BMP2K	BMP2 inducible kinase	-1.096787353	1.748369474	3.197616	3.080935	5.097505	3.43381096	3.31335619	4.18168353	385	469	828
79594	NM_024544	MUL1	mitochondrial E3 ubiquitin proteir	1.95381679	1.748366937	9.755181	19.087418	16.034458	4.6062649	5.54343125	5.38676798	917	2394	1972
286336	NM_033387	FAM78A	family with sequence similarity 78	1.437496299	1.747961383	0.172748	0.247022	0.282475	0.73313634	0.96581954	1.11084509	26	50	56
127829	NM_001256129,NM_138795	ARL8A	ADP ribosylation factor like GTPa	1.0279662	1.747892776	10.944336	11.231504	17.875988	4.32386849	4.36170552	5.09841735	747	1026	1606
6400	NM_001244984,NM_005065	SEL1L	SEL1L ERAD E3 ligase adaptor s	1.111874786	1.746965514	10.590092	11.61481	17.144016	6.34897901	6.50019628	7.14626435	3161	4696	6792
55754	NM_001143958,NM_018247	TMEM30A	transmembrane protein 30A	-1.101224193	1.746515564	17.296442	15.606972	28.16902	6.26355069	6.12633688	7.06000436	2977	3612	6395
2058	NM_004446	EPRS	glutamyl-prolyl-tRNA synthetase	1.089578935	1.746312427	36.300865	39.596252	59.572048	7.47778611	7.60089309	8.27864467	6959	10131	14947
10390	NM_001007794,NM_001330743	CEPT1	choline/ethanolamine phosphotra	-1.028632586	1.745742348	20.109833	19.655612	33.442647	5.5285799	5.4887443	6.31904583	1773	2303	3807
54797	NM_001127350,NM_017638	MED18	mediator complex subunit 18	1.064256896	1.745579447	9.457978	10.023413	15.501208	4.18126603	4.26631627	4.95069932	673	957	1445
4646	NM_001300899,NM_004999	MYO6	myosin VI	-1.405617886	1.745484488	8.107297	5.768444	13.291773	6.10815615	5.62538694	6.90284938	2669	2537	5730
9813	NM_014774	EFCAB14	EF-hand calcium binding domain	-1.001241761	1.745125162	10.685564	10.685422	17.52437	5.93097218	5.92921109	6.72419959	2356	3144	5057
101928659	NR_135180	LOC101928659	uncharacterized LOC101928659	1.664117577	1.744763782	0.848121	1.347324	1.388147	0.41264541	0.63490936	0.65979727	13	29	28
2907	NM_000837,NM_001009184	GRINA	glutamate ionotropic receptor NM	1.623937733	1.743799763	46.860711	76.77217	77.19228	6.45204887	7.14521738	7.24725757	3398	7373	7288
64854	NM_001134223,NM_001286767	USP46	ubiquitin specific peptidase 46	1.048035145	1.743397474	2.088486	2.193223	3.41556	4.1064401	4.17029377	4.87228279	637	892	1366
221079	NM_178815	ARL5B	ADP ribosylation factor like GTPa	1.002121237	1.74319301	4.748939	4.76542	7.780937	4.13809052	4.14097459	4.90456173	652	873	1398
129804	NM_001128165,NM_153214	FBLN7	fibulin 7	1.508063479	1.742782196	0.701297	1.060845	1.154366	1.36692851	1.75827411	1.90855849	62	125	133
5271	NM_001031848,NM_001276490	SERPINB8	serpin family B member 8	2.836885843	1.742548353	1.022029	2.549138	1.625974	1.93632435	3.17404113	2.56774382	111	421	238
154791	NM_197964,NR_073058,NR_07	FMC1	formation of mitochondrial compl	-1.46753673	1.742449022	3.672622	2.473175	6.001622	2.39332696	1.96260273	3.07278016	167	152	358
10945	NM_006801	KDELR1	KDEL endoplasmic reticulum pro	1.010759143	1.741881727	119.749847	121.161606	196.007858	7.51201023	7.52736558	8.30929632	7127	9625	15269
7351	NM_003355	UCP2	uncoupling protein 2	1.159101279	1.741626789	2.103968	2.44186	3.450448	2.13345087	2.30073045	2.78704637	133	206	285
7812	NM_001007553,NM_001130523	CSDE1	cold shock domain containing E1	1.387596614	1.741547707	64.747792	89.14469	104.902971	8.00038134	8.47139924	8.79835723	10014	18566	21450
3136	NR_001434	HLA-H	major histocompatibility complex,	1.048792879	1.741500625	34.90316	36.648113	57.122898	5.88134087	5.9489347	6.67124319	2275	3188	4873
55789	NM_001145208,NM_018369	DEPDC1B	DEP domain containing 1B	1.262525205	1.741299649	12.64256	15.902143	20.543985	4.96269999	5.28938617	5.74309004	1185	1999	2538
54795	NM_001195227,NM_001321281	TRPM4	transient receptor potential cation	-1.312509239	1.741294594	16.132361	12.041826	25.782324	5.9524941	5.56740891	6.7427309	2392	2435	5123
49855	NM_001145923,NM_020843	SCAPER	S-phase cyclin A associated prot	-1.137504605	1.741105076	1.48224	1.341253	2.495127	3.00082115	2.83945493	3.72222359	275	323	589
4082	NM_002356	MARCKS	myristoylated alanine rich protein	1.693109303	1.741012609	10.661359	18.067482	17.442669	5.51337462	6.26009696	6.29983366	1754	3968	3756
91526	NM_001195144,NM_153697	ANKRD44	ankyrin repeat domain 44	2.166385123	1.740557557	0.209374	0.454131	0.395174	1.16869613	1.89025622	1.66651905	49	142	105
5066	NM_000919,NM_001177306,NM	PAM	peptidylglycine alpha-amidating r	1.515831226	1.740078821	16.521749	25.159836	27.112775	6.43261128	7.02704116	7.22466324	3352	6789	7174
10567	NM_006423	RABAC1	Rab acceptor 1	-1.03122018	1.739356301	51.635677	50.122925	84.393463	5.37766084	5.33438844	6.16143061	1593	2064	3408
388531	NM_207391	RGS9BP	regulator of G protein signaling 9	-2.326361014	1.73929184	0.253115	0.107902	0.413929	0.77668602	0.38406669	1.16514558	28	16	60
9767	NM_001077445,NM_014735	JADE3	jade family PHD finger 3	1.126734295	1.738565536	0.954038	1.076147	1.554507	2.48150547	2.6243808	3.16560381	180	271	385
8676	NM_003764	STX11	syntaxin 11	1.139759334	1.738310382	0.099157	0.110754	0.162199	0.61813834	0.68708806	0.95003448	21	32	45
105372476	NR_136528,NR_136529	LOC105372476	uncharacterized LOC105372476	1.742183719	1.738263737	2.717238	4.792852	4.617575	1.59082596	2.17238371	2.16988427	79	184	169
8826	NM_003870	IQGAP1	IQ motif containing GTPase activ	1.145143017	1.738112338	33.2593	38.131271	54.327541	7.87340503	8.06815494	8.66831877	9167	14026	19597
113278	NM_033409	SLC52A3	solute carrier family 52 member 3	1.125255186	1.738108075	9.710702	10.940886	15.87132	4.7277342	4.89192945	5.50200967	1001	1505	2140
27338	NM_014501	UBE2S	ubiquitin conjugating enzyme E2	1.184314482	1.737930517	155.675247	184.584732	254.267624	7.5041374	7.74695568	8.29813918	7088	11216	15151
64778	NM_001135095,NM_022763	FNDC3B	fibronectin type III domain contai	1.213321094	1.737916339	18.664255	22.742217	30.637175	7.00457422	7.2815643	7.7971681	5002	8109	10692
81624	NM_001042517,NM_001258366	DIAPH3	diaphanous related formin 3	1.010492731	1.737279297	3.85599	3.835267	6.325784	4.22318689	4.23744571	4.98695254	694	937	1483
197257	NM_153486,NM_194436	LDHD	lactate dehydrogenase D	-3.579961838	1.737156876	1.925901	0.536904	3.226304	2.29192162	1.06116576	2.95827541	153	57	327
6452	NM_001122681,NM_001145855	SH3BP2	SH3 domain binding protein 2	1.977091357	1.737095877	8.330266	16.474168	13.634476	6.23700106	7.21092267	7.02556268	2922	7719	6243
114876	NM_001242508,NM_018030,NM	OSBPL1A	oxysterol binding protein like 1A	-1.29398451	1.737068687	14.849418	13.075622	27.482162	5.8415226	5.47705979	6.62749283	2212	2284	4726
9917	NM_001324310,NM_001324311	FAM20B	FAM20B, glycosaminoglycan xylo	1.058287488	1.736774866	15.830767	16.793131	25.842501	6.53403852	6.61491476	7.3238488	3599	5089	7688
7702	NM_001282656,NM_001282657	ZNF143	zinc finger protein 143	1.065789826	1.736627344	7.847274	8.349695	12.7813	4.55247036	4.64060352	5.32251714	882	1256	1884
101241902	NR_077236	LOC101241902	chromosome 4 open reading fran	-5.997068941	1.736355262	0.236971	0.025673	0.382458	0.43999062	0.08023518	0.69713258	14	3	30
2117	NM_001145312,NM_005240	ETV3	ETS variant 3	1.013649274	1.736212912	3.296426	3.16141	4.927071	2.64317467	2.65962865	3.33801005	206	279	440
101927934	NR_105045	LINC01843	long intergenic non-protein codin	1.269405955	1.735896078	5.041355	6.420955	8.22711	2.68948191	2.98550663	3.3873837	214	363	457
160	NM_014203,NM_130787	AP2A1	adaptor related protein complex 2	1.105962013	1.735509313	17.935751	19.866277	29.261142	5.93036974	6.07340943	6.71569694	2355	3480	5027
221035	NM_001001330	REEP3	receptor accessory protein 3	-1.43389678	1.735190666	20.561188	14.352062	33.527039	6.71702247	6.20300317	7.50631125	4091	3812	8731
6237	NM_006270	RRAS	related RAS viral (r-ras) oncogen	-1.34803548	1.735142549	41.908844	31.115948	68.35553	5.36612462	4.94735617	6.14632498	1580	1566	3372
3895	NM_001079521,NM_001079522	KTN1	kinectin 1	1.492963818	1.734909969	54.51307	81.348607	88.924127	7.96757906	8.54385911	8.76000331	9788	19525	20886
23048	NM_015033	FNBP1	formin binding protein 1	1.250257687	1.734664571	7.50442	9.397205	12.236894	5.34548562	5.66061052	6.12507905	1557	2601	3322
729359	NM_001080400	PLIN4	perilipin 4	1.860606371	1.734219973	1.944891	3.626361	3.174419	3.70573844	4.54958432	4.45257508	473	1176	1009
286451	NM_001195214,NM_173834	YIPF6	Yip1 domain family member 6	1.066840167	1.733924241	9.853049	10.524869	16.114924	5.93758245	6.02945501	6.72165405	2367	3374	5048
54542	NM_001100588,NM_018835	RC3H2	ring finger and CCCH-type doma	1.275433706	1.733914817	4.061724	5.217095	6.556228	4.01393646	4.34556265	4.76977761	595	1014	1269
91442	NM_001300978,NM_152266	FAAP24	Fanconi anemia core complex as	1.233408671	1.732414984	1.721691	1.815166	2.414202	1.73025772	1.94842076	2.32699908	91	150	194
7984	NM_005435	ARHGEF5	Rho guanine nucleotide exchang	-1.366024811	1.732291344	5.650291	4.140467	9.202341	4.96387793	4.53067054	5.73694088	1186	1160	2527
4077	NM_001291571,NM_001291572	NBR1	NBR1, autophagy cargo receptor	-1.017154313	1.73164134	28.727425	28.389596	46.800184	7.08309989	7.05874334	7.87075028	5284	6941	11254
147184	NM_001195386,NM_001195387	TMEM99	transmembrane protein 99	1.356563479	1.731476979	4.183063	5.688268	6.817422	3.23740108	3.63669106	3.96347983	331	600	705
23097	NM_001300960,NM_001300963	CDK19	cyclin dependent kinase 19	1.020586361	1.731147049	1.517582	1.550547	2.473022	3.35699061	3.38355363	4.08816658	363	495	773
149466	NM_001164829,NM_182517	C1orf210	chromosome 1 open reading fran	1.40864629	1.730998775	4.495569	6.359524	7.337719	2.90595399	3.34347295	3.614139	255	480	543
2919	NM_001511,NR_046035	CXCL1	C-X-C motif chemokine ligand 1	2.214066735	1.730978917	2.215095	4.9462	3.632183	1.82664439	2.73178769	2.43591723	100	296	213
162962	NM_001102657	ZNF836	zinc finger protein 836	-1.260141881	1.730978917	0.780448	0.61792	1.272509	1.82664439	1.59489542	2.43591723	100	106	213
128344	NM_001300831,NM_181643,NR	PIFO	primary cilia formation	2.015933494	1.730551539	0.073787	0.150431	0.118437	0.23670415	0.44594451	0.39027104	7	19	15
125919	NM_213598	ZNF543	zinc finger protein 543	1.254671085	1.730123341	3.390366	4.260196	5.51192	3.71416279	4.01904684	4.45802288	476	798	1013
955	NM_001114089,NM_001247,NM	ENTPD6	ectonucleoside triphosphate diph	1.301901341	1.729965963	26.936784	35.26151	43.992712	6.76882532	7.14638265	7.55399176	4242	7379	9026
8609	NM_001270942,NM_001270943	KLF7	Kruppel like factor 7	-2.003860255	1.728526131	2.122702	1.049239	3.363117	4.16701728	3.24247658	4.92240621	666	444	1416
92565	NM_001350939,NM_145235	FANK1	fibronectin type III and ankyrin re	1.178486527	1.727890861	2.453771	3.010394	4.061244	2.0823043	2.26684378	2.72060965	127	200	270
153396	NM_001289007,NM_001289008	TMEM161B	transmembrane protein 161B	1.037277941	1.727837512	11.855112	12.500943	19.526456	4.94013441	4.99125166	5.70922621	1166	1616	2478
57107	NM_020381	PDSS2	decaprenyl diphosphate synthase	1.118992209	1.727341136	1.530543	1.712148	2.484047	2.65489157	2.79259185	3.34390718	208	311	442
259282	NM_148894	BOD1L1	biorientation of chromosomes in	1.083997679	1.727120487	7.326393	7.952313	11.892999	6.25202543	6.36692357	7.03242582	2953	4277	6273
25814	NM_001167621,NM_013236	ATXN10	ataxin 10	-1.045326775	1.727024815	26.989161	25.696453	43.536172	6.45916656	6.39595357	7.24055586	3415	4365	7254
5211	NM_001002021,NM_002626	PFKL	phosphofructokinase, liver type	1.315101475	1.726365459	22.790739	30.275576	37.382947	6.06325022	6.45325973	6.84190781	2586	4544	5491
9858	NM_014811	PPP1R26	protein phosphatase 1 regulatory	-1.12969149	1.726266735	20.145117	17.852459	32.678715	6.6108664	6.43684541	7.39231617	3798	4492	8064
10067	NM_005698,NM_052837	SCAMP3	secretory carrier membrane prote	1.14279866	1.725953696	20.017479	22.765051	32.341049	4.96623093	5.15303948	5.73413712	1188	1814	2522
51545	NM_016535	ZNF581	zinc finger protein 581	-1.148404134	1.725597642	10.948245	9.557979	17.780331	3.81681978	3.63226192	4.56033921	514	598	1091
10767	NM_001145158,NM_001145207	HBS1L	HBS1 like translational GTPase	-1.149749668	1.725474665	4.112285	3.776816	6.058438	4.6997269	4.50667419	5.46325552	981	1140	2082
84706	NM_001142466,NM_133443	GPT2	glutamic--pyruvic transaminase 2	1.349292894	1.725351002	12.01153	16.20074	19.447363	5.57016669	5.99450941	6.34427044	1826	3292	3875
85358	NM_033517	SHANK3	SH3 and multiple ankyrin repeat	2.531292822	1.725023122	0.665597	1.690504	1.080539	2.48150547	3.65645563	3.15558012	180	609	382
57623	NM_001029939,NM_001167583	ZFAT	zinc finger and AT-hook domain	1.502320508	1.725002835	2.197057	3.308455	3.693176	3.49049124	4.03423402	4.22226663	402	807	853
51765	NM_001042452,NM_001042453	STK26	serine/threonine kinase 26	1.103001052	1.724910474	11.79787	13.154966	19.299877	5.29770564	5.43572001	6.06877437	1505	2218	3193
51316	NM_001130715,NM_001130716	PLAC8	placenta specific 8	1.267264976	1.724857031	20.883826	25.529432	32.249444	4.79810635	5.12887652	5.56268791	1053	1783	2234
101930370	NR_134241,NR_134242	LOC101930370	uncharacterized LOC101930370	1.147829728	1.72443761	1.713539	2.030151	2.80953	2.25390578	2.41343275	2.9074142	148	227	314
64860	NM_001168478,NM_001168479	ARMCX5	armadillo repeat containing, X-lin	1.242726741	1.724310066	3.633921	4.503852	5.87325	3.41670749	3.70365887	4.14500092	380	631	806
57157	NM_001127357,NM_001127358	PHTF2	putative homeodomain transcript	-1.414483809	1.723485352	7.04389	4.985988	12.151805	5.12944497	4.64610598	5.89741808	1335	1261	2830

823	NM_001198868,NM_001198869	CAPN1	calpain 1	-1.071266481	1.723426743	97.840036	91.415604	158.702888	8.18595475	8.0869894	8.96915968	11394	14211	24152
5786	NM_002836,NM_080840,NM_08	PTPRA	protein tyrosine phosphatase, rec	1.478457405	1.72301061	15.424519	22.840811	24.831434	5.66591984	6.22081235	6.43891228	1954	3860	4141
7040	NM_000660	TGFB1	transforming growth factor beta 1	-1.142251131	1.723001216	15.124516	13.25342	24.488712	5.37057255	5.18363121	6.14083107	1585	1854	3359
318	NM_001161,NM_001244390,NM	NUDT2	nudix hydrolase 2	1.080729449	1.722880113	6.50279	7.977957	11.820153	2.96832247	3.06652959	3.67388275	268	387	568
101929243	NR_134625,NR_134626	LOC101929243	uncharacterized LOC101929243	2.329962286	1.722669475	1.107569	2.296592	1.318097	0.49317359	0.96581954	0.76902392	16	50	34
283981	NR_027231_chrX,NR_027231_c	LINC00685	long intergenic non-protein codin	1.121836775	1.722669475	0.552761	0.692094	0.987835	0.49317359	0.54351856	0.76902392	16	24	34
140609	NM_133494	NEK7	NIMA related kinase 7	-1.107683938	1.722510464	7.734569	6.991789	12.523749	5.00565571	4.86292405	5.77126223	1222	1474	2589
65983	NM_001146319,NM_001146320	GRAMD2B	GRAM domain containing 2B	-1.781015849	1.721826298	13.611743	7.629503	22.019293	5.33459909	4.52948015	6.10351555	1545	1159	3272
115548	NM_001146032,NM_138782	FCHO2	FCH domain only 2	-1.228627958	1.72150267	6.664253	5.427917	10.811691	5.05843928	4.7712289	5.82389986	1269	1380	2687
10140	NM_001243877,NM_001243885	TOB1	transducer of ERBB2, 1	-1.180631322	1.721303548	105.129354	89.134315	170.106992	7.8429989	7.60457183	8.62387253	8975	10157	19001
7132	NM_001065,NM_001346091,NM	TNFRSF1A	TNF receptor superfamily membe	1.479084831	1.720398148	47.260349	70.252543	76.614287	6.70015794	7.26037389	7.47709544	4043	7990	8555
348938	NM_001099287,NM_001172292	NIPAL4	NIPA like domain containing 4	-2.090659228	1.720386878	0.746734	0.35838	1.208184	1.76310734	1.10017522	2.35646843	94	60	199
29071	NM_001011551,NM_152692	C1GALT1C1	C1GALT1 specific chaperone 1	-7.638933371	1.720217239	20.970282	2.733778	33.929059	5.18605837	2.49375478	5.95200904	1390	243	2941
90952	NM_138961	ESAM	endothelial cell adhesion molecul	-2.050815077	1.719592763	4.844512	2.362341	7.848324	3.29843463	2.40826098	4.01797815	347	226	734
3716	NM_001320923,NM_001321852	JAK1	Janus kinase 1	1.360288552	1.719514257	12.17876	16.594732	19.686073	5.94773912	6.38546467	6.71995453	2384	4333	5042
726	NM_004055	CAPN5	calpain 5	1.040498878	1.719447976	3.419746	3.561925	5.523833	3.96536097	4.01904684	4.7082479	574	798	1214
7046	NM_001130916,NM_001306210	TGFBR1	transforming growth factor beta r	1.927447624	1.719041477	2.525182	4.869876	4.072135	4.0892716	4.99470627	4.83508422	629	1620	1330
5223	NM_001317079,NM_002629	PGAM1	phosphoglycerate mutase 1	1.350279542	1.719014964	68.854144	93.099978	111.318573	6.98004173	7.41034031	7.75684956	4917	8871	10396
389	NM_001042678,NM_001042679	RHOC	ras homolog family member C	1.150380503	1.718438288	142.65879	164.936949	231.711198	7.57020913	7.77133027	8.34813903	7422	11408	15687
9314	NM_001314052,NM_004235	KLF4	Kruppel like factor 4	2.657989466	1.718312718	11.299212	30.155029	18.20163	5.04848016	6.43143843	5.81118548	1260	4475	2663
21	NM_001089	ABCA3	ATP binding cassette subfamily A	-2.103102293	1.717795621	1.081926	0.513888	1.746993	2.99622306	2.11004283	3.69940604	274	174	579
654841	NR_102371	LOC654841	uncharacterized LOC654841	2.044226257	1.717675762	0.073193	0.166814	0.128618	0.26755841	0.50527715	0.43515728	8	22	17
100507316	NR_120682,NR_120683	MINCR	MYC-induced long noncoding RN	1.070121183	1.716534325	5.731011	5.23496	8.207467	1.89739865	1.96964178	2.50596801	107	153	226
5270	NM_001136528,NM_001136530	SERPINE2	serpin family E member 2	1.798368685	1.716341581	19.931017	35.960043	32.084594	5.48575866	6.31812263	6.2516338	1720	4133	3631
7357	NM_003358	UGCG	UDP-glucose ceramide glucosylt	-1.002949475	1.716287502	53.372147	53.271137	86.080948	7.71067572	7.70644704	8.48709805	8185	10904	17278
6645	NM_006750	SNTB2	syntrophin beta 2	-1.019327124	1.715954542	2.928842	2.875597	4.722404	4.85126892	4.82461482	5.6093351	1094	1434	2309
54407	NM_001307936,NM_018976	SLC38A2	solute carrier family 38 member 2	1.175667809	1.715864825	75.477669	88.815282	119.369744	8.45992018	8.69279	9.2371506	13785	21654	29092
284161	NM_001165993,NM_001165994	GDPD1	glycerophosphodiester phosphod	-1.26551468	1.715803896	1.27427	0.893212	2.013213	2.07360076	1.82188956	2.70236404	126	133	266
1806	NM_000110,NM_001160301	DPYD	dihydropyrimidine dehydrogenase	1.248157072	1.7157802	5.990952	7.402713	9.214849	4.64349742	4.95180631	5.39814864	942	1571	1988
203245	NM_197956	NAIF1	nuclear apoptosis inducing factor	1.160747637	1.715473222	2.788951	3.246737	4.502511	3.35699061	3.55246622	4.0758175	363	563	766
2335	NM_001306129,NM_001306130	FN1	fibronectin 1	2.852681949	1.714592105	1.591654	4.565563	2.57311	3.86300966	5.30963773	4.59907035	532	2028	1122
221584	NR_024063	ZSCAN12P1	zinc finger and SCAN domain co	-1.760428488	1.714422913	1.119186	0.636692	1.806804	2.44147625	1.81408935	3.10438964	174	132	367
79094	NM_001142776,NM_024111	CHAC1	ChaC glutathione specific gamma	1.169857762	1.712821646	10.211677	11.963501	16.462523	4.27535493	4.49085976	5.02047721	721	1127	1519
23204	NM_001313858,NM_015161	ARL6IP1	ADP ribosylation factor like GTPa	1.235983622	1.712794209	229.447051	283.995577	369.35056	9.07462061	9.37977066	9.84986165	21129	34893	44511
25837	NM_001308053,NM_014353	RAB26	RAB26, member RAS oncogene	-1.103246751	1.712379609	11.05535	9.991441	17.541735	4.20934818	4.07560059	4.95263026	687	832	1447
2696	NM_000164,NM_001308418	GIPR	gastric inhibitory polypeptide rece	-1.192084463	1.712320118	3.289266	2.79858	5.385113	3.5292011	3.29945612	4.25236388	414	464	872
79801	NM_001324318,NM_001324319	SHCBP1	SHC binding and spindle associa	1.157717479	1.711970577	13.174927	15.329526	21.396403	5.44837589	5.65516321	6.21026664	1675	2591	3527
79898	NM_001031721,NM_024840	ZNF613	zinc finger protein 613	-2.042044491	1.711763284	0.831741	0.402383	1.347156	1.54118428	0.95166931	2.09493548	75	49	158
339287	NM_001012241	MSL1	male specific lethal 1 homolog	1.015163267	1.711611955	23.170143	23.550343	37.273232	6.44616075	6.46762604	7.21464071	3384	4590	7124
113451	NM_001293562,NM_001301823	AZIN2	antizyme inhibitor 2	1.264311435	1.711266424	2.612469	3.194329	4.151761	2.54584744	2.83174937	3.21470347	190	321	400
11336	NM_007277	EXOC3	exocyst complex component 3	1.218206733	1.711229146	7.861036	9.585127	12.641756	4.46864976	4.74172192	5.21642227	830	1351	1747
57552	NM_001146276,NM_001146277	NCEH1	neutral cholesterol ester hydrolas	-1.212528767	1.710875066	41.474988	34.27803	66.601119	7.44441912	7.16815463	8.21571771	6799	7492	14307
5885	NM_006265	RAD21	RAD21 cohesin complex compor	1.090167846	1.710485189	134.693512	147.006119	216.510269	8.94484718	9.06915596	9.71803973	19308	28124	40620
84333	NM_001256549,NM_001257101	PCGF5	polycomb group ring finger 5	-1.704613941	1.709189913	14.442168	6.118171	12.183452	5.1647037	4.42324784	5.9212789	1369	1073	2878
26135	NM_001018067,NM_001018068	SERBP1	SERPINE1 mRNA binding protei	1.97089492	1.708768734	32.288616	63.711491	51.830019	7.73070921	8.70621946	8.50085445	8300	21857	17444
571	NM_001186,NM_206866,NR_02	BACH1	BTB domain and CNC homolog 1	1.451505652	1.70868876	3.803313	5.52229	6.089273	4.46199571	4.97909494	5.20755546	826	1602	1736
3146	NM_001313892,NM_001313893	HMGB1	high mobility group box 1	2.146050706	1.708483984	40.726631	88.333487	64.895399	7.42765516	8.52486962	8.1969027	6720	19269	14121
100616210	NR_039890	MIR4737	microRNA 4737	-1.002067138	1.707626065	2.780814	2.799718	4.676186	0.29776659	0.29720487	0.47868902	9	12	19
6513	NM_006516	SLC2A1	solute carrier family 2 member 1	1.312888319	1.707602089	109.932594	144.48877	176.408005	8.62137582	9.01324907	9.39183315	15422	27053	32390
51375	NM_015976,NM_152238,NR_03	SNX7	sorting nexin 7	1.317076109	1.707412164	5.579696	7.358211	8.941275	3.41326233	3.77771682	4.1280136	379	667	796
55898	NM_001039675,NM_001323619	UNC45A	unc-45 myosin chaperone A	1.042721382	1.707200608	16.737876	17.579549	27.128434	5.93036974	5.98975653	6.69219736	2355	3281	4945
51773	NM_016578	RSF1	remodeling and spacing factor 1	1.449803946	1.707034642	6.646697	9.650356	10.665669	5.0976022	5.62036393	5.8515867	1305	2528	2740
81788	NM_030952	NUAK2	NUAK family kinase 2	-1.482867792	1.706268702	1.068345	0.722659	1.71981	2.19894236	1.77444288	2.83389297	141	127	296
8099	NM_001270433,NM_001270434	CDK2AP1	cyclin dependent kinase 2 associ	-1.836926723	1.706262355	26.953058	15.917396	43.432803	5.41172615	4.56244519	6.16850922	1632	1187	3425
65977	NM_019091	PLEKHA3	pleckstrin homology domain cont	1.399540998	1.705618788	4.317357	6.055563	6.924783	3.509976	3.95841731	4.22706077	408	763	856
23401	NM_012083	FRAT2	FRAT2, WNT signaling pathway	-1.164466506	1.70560187	2.650488	2.284369	4.257216	2.74535098	2.5605457	3.42403181	224	257	470
10198	NM_022782,NR_103517	MPHOSPH9	M-phase phosphoprotein 9	1.116149595	1.705361771	3.891913	4.332187	6.218329	4.10216709	4.2519532	4.83717616	635	947	1332
79701	NM_024648,NM_175902,NR_03	OGFOD3	2-oxoglutarate and iron depende	1.024388838	1.705055034	8.706325	8.933664	13.773954	5.24635403	5.28021477	6.00041522	1451	1986	3043
100131347	NR_036551	LOC100131347	RAD52 motif containing 1 pseud	1.221652262	1.704773139	0.331841	0.408852	0.544151	0.81895953	0.95166931	1.20457043	30	49	63
2491	NM_001318521,NM_001318523	CENPI	centromere protein I	1.198949232	1.704174786	4.93204	6.007542	8.097591	4.03201954	4.27912189	4.76429118	603	966	1264
7317	NM_003334,NM_153280	UBA1	ubiquitin like modifier activating e	1.250585233	1.704088426	49.465672	61.81291	79.142538	7.44167385	7.76261798	8.20725098	6786	11339	14223
267	NM_001144,NM_001323511,NM	AMFR	autocrine motility factor receptor	1.240891542	1.703304181	23.375082	28.796608	37.074211	6.36199349	6.66997084	7.12308987	3190	5289	6683
442578	NR_040582	STAG3L3	stromal antigen 3-like 3 (pseudog	1.050064463	1.703088303	3.171535	3.332207	5.077492	2.94465259	3.00619294	3.6335275	263	369	551
2934	NM_000177,NM_001127662,NM	GSN	gelsolin	1.278611352	1.703057855	76.928369	98.176096	124.222678	7.65433256	8.00735333	8.41950911	7870	13445	16485
57579	NM_001105531,NM_001162529	FAM135A	family with sequence similarity 13	-1.480727044	1.702408241	1.307176	0.856567	1.991415	3.00540462	2.52275889	3.69710431	276	249	578
51667	NM_001243351,NM_016118	NUB1	negative regulator of ubiquitin like	-1.44316485	1.702165018	12.037335	8.329328	19.204808	5.23761487	4.72518011	5.98917078	1442	1335	3019
2651	NM_001491,NM_145649,NM_14	GCNT2	glucosaminyl (N-acetyl) transfera	-1.102327648	1.701664021	3.347682	3.043051	5.366923	3.97940737	3.84815806	4.7082479	580	703	1214
6564	NM_005073	SLC15A1	solute carrier family 15 member 1	-3.568466796	1.701568162	3.952792	1.103309	6.323643	3.70573844	2.12902957	4.42641333	473	177	990
160897	NM_180989	GPR180	G protein-coupled receptor 180	1.162275028	1.70151053	2.029277	2.362909	3.246411	4.21331566	4.41939707	4.94779804	689	1070	1442
1945	NM_005227,NM_182689,NM_18	EFNA4	ephrin A4	1.153223199	1.701051016	6.061587	6.951039	9.596011	3.0281061	3.21015128	3.71995801	281	433	588
51093	NM_001142560,NM_015997	RRNAD1	ribosomal RNA adenine dimethyl	-1.059492304	1.700606934	12.408714	11.887151	19.67971	4.71519816	4.63508	5.4585071	992	1251	2075
84803	NM_001256421,NM_001256422	GPAT3	glycerol-3-phosphate acyltransfe	1.14963368	1.700591584	32.093083	37.168403	51.526018	6.42236501	6.62135418	7.18147519	3328	5112	6961
25792	NM_001131015,NM_001131016	CIZ1	CDKN1A interacting zinc finger p	1.351333655	1.700375161	23.531188	31.816701	37.724108	6.10281927	6.53175021	6.86002492	2659	4801	5561
50804	NM_001301210,NM_016132,NR	MYEF2	myelin expression factor 2	1.266256628	1.700012493	7.852907	9.72172	12.964235	5.29863957	5.63150252	6.04905586	1506	2548	3149
168544	NM_001329856,NM_207336	ZNF467	zinc finger protein 467	1.016207009	1.699973681	1.482074	1.431362	2.184925	1.91699278	1.93409799	2.51644942	109	148	228
387522	NM_199203	TMEM189-UBE2V1	TMEM189-UBE2V1 readthrough	-1.268835746	1.699955539	0.678427	0.535719	1.088316	1.55375576	1.33632702	2.10191219	76	80	159
387082	NM_001002255	SUMO4	small ubiquitin-like modifier 4	-1.331384061	1.699564023	0.380251	0.261212	0.593423	0.3273552	0.25173066	0.52094563	10	10	21
65072	NR_040030	CFLAR-AS1	CFLAR antisense RNA 1	3.639086627	1.699564023	0.241458	0.909903	0.390724	0.3273552	0.95166931	0.52094563	10	49	21
406984	NR_029639	MIR200B	microRNA 200b	1.34350598	1.699564023	2.549327	3.573031	4.216389	0.3273552	0.42561204	0.52094563	10	18	21
651430	NR_046264	FLJ38576	uncharacterized LOC651430	1.195404003	1.699564023	0.104915	0.124265	0.166435	0.3273552	0.38406669	0.52094563	10	16	21
221184	NM_152727	CPNE2	copine 2	-1.187862681	1.699057994	6.36159	5.366419	10.169354	3.89547266	3.66515371	4.61987668	545	613	1139
64598	NM_001040097,NM_001040098	MOSPD3	motile sperm domain containing 3	-1.594946753	1.69691821	5.303367	3.327164	8.364592	2.83047153	2.27254712	3.50784068	240	201	501
100316904	NM_001168682,NM_001348677	SAP25	Sin3A associated protein 25	-3.021452398	1.69676589	4.554475	1.440369	7.297583	2.64317467	1.45142181	3.30815729	206	91	430
9318	NM_001143887,NM_004236	COPS2	COP9 signalosome subunit 2	1.892786831	1.696512521	11.845737	22.468939	18.906504	5.59470734	6.50110747	6.34500571	1858	4699	3877
128486	NM_001080472	FITM2	fat storage inducing transmembra	1.21727711	1.696443381	2.315982	2.824502	3.694582	3.53238053	3.79367035	4.24292715	415	675	866
64771	NM_022758,NM_024294	C6orf106	chromosome 6 open reading fran	-1.132309011	1.695999395	9.675483	8.590406	15.560402	5.4177562	5.2429348	6.16601485	1639	1934	3419
80830	NM_030641	APOL6	apolipoprotein L6	1.007047102	1.695882648	23.535872	23.729155	37.508484	7.86633587	7.87642389	8.62584029	9122	12274	19027
8518	NM_001318360,NM_001330749	IKBKAP	inhibitor of kappa light polypeptid	1.031214671	1.695843953	10.8235	11.220697	17.334667	6.02482449	6.06850015	6.77773352	2517	3468	5250
25861	NM_001083885,NM_001173425	WHRN	whirlin	3.508710725	1.695732106	0.89413	3.511367	1.632255	2.18284609	3.74731998	2.80852903	139	652	290
83660	NM_015059	TLN2	talin 2	3.426356399	1.695548106	0.076268	0.261533	0.121471	0.89996265	1.99055518	1.30472178	34	156	71
23321	NM_001130067,NM_001302692	TRIM2	tripartite motif containing 2	-1.57785688	1.695528664	2.406855	1.802847	4.174571	3.93444966	3.3298614	4.65706199	561	475	1170
84899	NM_001079669,NM_001286453	TMTC4	transmembrane and tetratricopep	-1.261704349	1.69525084	1.167464	0.920013	1.904868	2.52684435	2.255369	3.18215673	187	198	390
2069	NM_001432	EREG	epiregulin	1.431721466	1.694912202	70.658478	101.274033	112.542137	8.31917014	8.83556199	9.0785328	12500	23912	26058
10134	NM_001139441,NM_001139457	BCAP31	B-cell receptor-associated protei	1.326264734	1.694721414	107.328024	138.864749	170.432145	7.44463008	7.84996567	8.20228901	6800	12050	14174
10347	NM_019112	ABCA7	ATP binding cassette subfamily A	-1.060057256	1.694549988	13.803809	13.037103	21.983906	6.53364194	6.45043212	7.28816562	3598	4535	7499
4734	NM_001284338,NM_001284339	NEDD4	neural precursor cell expressed,	1.238717649	1.694279586	4.852939	5.178198	7.766281	4.85381277	5.15303948	5.59395302	1096	1814	2284
6495	NM_005982	SIX1	SIX homeobox 1	1.273360953	1.693803542	1.854223	2.365525	2.95515	2.55212659	2.84711955	3.20825259	191	325	398
729970	NR_033998	LOC729970	hCG2028352-like	-1.024244919	1.693421879	0.628033	0.599992	0.990131	0.66524311	0.65251278	0.99569924	23	30	48
26060	NM_012096	APPL1	adaptor protein, phosphotyrosine	1.199161505	1.693289908	2.658679	3.190331	4.231692	4.06092955	4.30856691	4.78502952	616	987	1283

3981	NM_001098268,NM_001330595	LIG4	DNA ligase 4	-1.363676613	1.693266961	3.801068	2.77574	6.06867	4.00709644	3.59177559	4.72980159	592	580	1233
401145	NM_001145065,NM_207491	CCSER1	coiled-coil serine rich protein 1	-1.555875738	1.693208413	0.514483	0.324439	0.825206	2.02024721	1.5673195	2.62698266	120	103	250
1902	NM_001351397,NM_001351398	LPAR1	lysophosphatidic acid receptor 1	1.003142365	1.693156537	15.149368	15.197334	24.672185	5.8606208	5.8650696	6.61016492	2242	3005	4669
85376	NM_015672	RIMBP3	RIMS binding protein 3	3.378831987	1.692953018	0.049013	0.168011	0.079297	0.35634914	0.96581954	0.56199967	11	50	23
57568	NM_020808	SIPA1L2	signal induced proliferation assoc	2.839760452	1.692953018	0.04353	0.123668	0.068437	0.35634914	0.84852988	0.56199967	11	42	23
4166	NM_021615	CHST6	carbohydrate sulfotransferase 6	-1.526342415	1.692568606	6.826005	4.477014	10.860264	5.54756029	4.95358253	6.29413399	1797	1573	3741
55647	NM_017817	RAB20	RAB20, member RAS oncogene	-1.093764533	1.692483946	8.602033	7.876504	13.695722	3.78251604	3.6629841	4.49823858	501	612	1043
128218	NM_001320244,NM_144626	TMEM125	transmembrane protein 125	-1.22090309	1.692480013	11.054932	9.060362	17.58462	4.40935908	4.13629029	5.14052934	795	870	1655
1847	NM_004419	DUSP5	dual specificity phosphatase 5	1.340071084	1.692287227	25.532162	34.257919	40.612827	5.99690152	6.41348249	6.74663137	2468	4419	5137
11160	NM_001003790,NM_001003791	ERLIN2	ER lipid raft associated 2	1.201567316	1.692049348	7.288638	8.61482	11.484706	5.1114871	5.36940611	5.85313811	1318	2116	2743
105447645	NR_131188	LOC105447645	uncharacterized LOC105447645	1.122288154	1.691701956	0.671163	0.750554	1.058904	0.93882015	1.02107213	1.35230545	36	54	75
2068	NM_000400,NM_001130867	ERCC2	ERCC excision repair 2, TFIIH cc	-1.143610788	1.691298717	14.690514	12.603428	22.992343	5.20411411	5.01611219	5.94620592	1408	1645	2929
100101440	NR_130940,NR_130940_dup1	PMS2P7	PMS1 homolog 2, mismatch repa	-1.66428338	1.690868522	6.313684	3.79687	10.063741	2.3579234	1.79836126	2.99621456	162	130	337
23473	NM_014296	CAPN7	calpain 7	1.244853708	1.690732479	3.003783	3.743839	4.773993	3.78518394	4.08048581	4.49955999	502	835	1044
51138	NM_001258006,NM_001330727	COPS4	COP9 signalosome subunit 4	1.405012636	1.690599514	12.800563	17.95164	20.299184	4.51116872	4.9834484	5.24270032	856	1607	1780
7716	NM_001330393,NM_007146	VEZF1	vascular endothelial zinc finger 1	-1.011286885	1.690586988	11.85218	11.597809	18.870258	5.77932107	5.76342439	6.52610593	2117	2797	4402
1744	NR_130749	DLSTP1	dihydrolipoamide S-succinyltrans	1.134491058	1.690421979	0.533353	0.608644	0.854954	1.36692851	1.48128072	1.87636985	62	94	129
7009	NM_001098576,NM_003217	TMBIM6	transmembrane BAX inhibitor mc	-1.082937199	1.690234108	283.148546	261.281809	448.619439	9.63718614	9.5223864	10.3936713	31224	38524	64911
51107	NM_001077628,NM_001243771	APH1A	aph-1 homolog A, gamma-secret	1.068162656	1.690044525	10.618289	11.313209	16.727949	4.48186648	4.57288311	5.21239866	838	1196	1742
79666	NM_024613	PLEKHF2	pleckstrin homology and FYVE d	-1.184225165	1.689192206	6.810764	5.752161	10.808073	4.36902637	4.13785342	5.09667211	772	871	1604
9631	NM_001278312,NM_004298,NM	NUP155	nucleoporin 155	1.165314237	1.688119802	5.625753	6.537629	8.905266	4.70114026	4.91399145	5.4338367	982	1529	2039
255187	NR_122044	LOC255187	uncharacterized LOC255187	1.121567544	1.687433649	0.379939	0.421571	0.623164	0.38477185	0.42561204	0.60191771	12	18	25
56142	NM_018909,NM_031848,NM_03	PCDHA6	protocadherin alpha 6	2.543387241	1.687433649	0.11667	0.203266	0.190921	0.38477185	0.83317479	0.60191771	12	41	25
9653	NM_001134492,NM_012262	HS2ST1	heparan sulfate 2-O-sulfotransfer	1.56733238	1.68718494	5.704981	8.638583	8.974776	4.99186377	5.62371455	5.7279496	1210	2534	2511
7386	NM_006003	UQCRFS1	ubiquinol-cytochrome c reductase	1.184553396	1.686666213	52.174625	61.886681	82.719505	5.98186283	6.22265489	6.72674065	2442	3865	5066
2979	NM_002098	GUCA1B	guanylate cyclase activator 1B	-1.107586497	1.686599529	1.83955	1.665982	2.915581	2.34351507	2.22627631	2.9773697	160	193	332
4179	NM_002389,NM_153826,NM_17	CD46	CD46 molecule	1.143644531	1.685796011	85.417242	97.948904	136.757192	8.09251942	8.28549591	8.84380377	10677	16315	22138
10479	NM_001042537,NM_001177651	SLC9A6	solute carrier family 9 member A(	1.645520958	1.685696842	4.484213	7.201762	7.073802	4.40068652	5.09225907	5.12605789	790	1737	1638
79673	NM_024620	ZNF329	zinc finger protein 329	-1.277548116	1.68556682	5.013199	3.933368	7.952065	4.14433812	3.81336732	4.86409893	655	685	1358
6890	NM_000593,NM_001292022	TAP1	transporter 1, ATP binding casse	-1.795986801	1.68542677	16.751334	9.574521	26.709182	5.61883752	4.79717993	6.35999664	1890	1406	3918
100996724	NR_144516,NR_144517	LOC100996724	phosphodiesterase 4D interacting	-1.115508228	1.685277791	3.62432	3.235347	5.773821	5.03621379	4.88356843	5.77126223	1249	1496	2589
152	NM_000683	ADRA2C	adrenoceptor alpha 2C	-1.211015311	1.684756011	0.903272	0.748747	1.436772	1.44997615	1.28081328	1.97085819	68	75	141
10867	NM_001168320,NM_006675	TSPAN9	tetraspanin 9	1.071646068	1.684427497	2.332989	2.505027	3.7204	3.44734922	3.53833258	4.14500092	389	557	806
29968	NM_021154,NM_058179	PSAT1	phosphoserine aminotransferase	-1.035565527	1.683660745	63.26214	61.188429	100.797479	7.1239808	7.07392876	7.87138837	5437	7015	11259
388341	NM_001113567,NM_207387	LRRC75A	leucine rich repeat containing 75/	1.771010926	1.683489776	0.250308	0.439999	0.395083	0.7550755	1.15059938	1.11084509	27	64	56
9095	NM_005149	TBX19	T-box 19	-1.384649792	1.682985321	1.042563	0.75438	1.657355	1.9554003	1.62195412	2.54744438	113	109	234
2969	NM_001163636,NM_001280800	GTF2I	general transcription factor IIi	-1.265062928	1.681630833	24.419299	19.72447	38.076371	6.72468665	6.38907884	7.46902373	4113	4344	8507
9846	NM_012296,NM_080491	GAB2	GRB2 associated binding protein	-2.52585881	1.68150251	0.306973	0.121301	0.486511	1.50279892	0.7861039	2.03057861	72	38	149
7538	NM_003407	ZFP36	ZFP36 ring finger protein	-1.485742243	1.681158586	58.412239	39.350014	92.276627	6.65310266	6.08884618	7.39675535	3912	3518	8089
55281	NM_018295	TMEM140	transmembrane protein 140	-1.376067762	1.681150747	1.292718	0.942045	2.048664	1.84721644	1.52971203	2.42483178	102	99	211
492311	NM_001007189	IGIP	IgA inducing protein	-1.013332588	1.681096493	3.484399	3.441307	5.512763	3.42699388	3.40966853	4.12116227	383	505	792
84443	NM_032428	FRMPD3	FERM and PDZ domain containir	2.02711012	1.680522179	0.5263	1.074852	0.834333	2.25390578	3.11177339	2.87519973	148	401	306
57447	NM_001282211,NM_001282212	NDRG2	NDRG family member 2	1.191748471	1.680440166	2.174844	3.354428	4.40473	2.82010209	3.04002433	3.48400652	238	379	492
6093	NM_005406	ROCK1	Rho associated coiled-coil contai	1.062635813	1.679945975	12.709714	13.520273	20.066355	6.379754	6.46638246	7.12115963	3230	4586	6674
4643	NM_004998	MYO1E	myosin IE	-1.690892302	1.679757037	35.133839	20.801399	55.460739	7.45785867	6.7057169	8.20279612	6863	5423	14179
6565	NM_001145998,NM_021082	SLC15A2	solute carrier family 15 member 2	2.215011661	1.679547226	0.136813	0.303373	0.217002	0.79797761	1.40024122	1.16514558	29	86	60
6459	NR_033420	SH3GL1P2	SH3 domain containing GRB2 lik	-1.756336525	1.679547226	0.302644	0.169758	0.4773	0.79797761	0.50527715	1.16514558	29	22	60
9706	NM_001142610,NM_014683	ULK2	unc-51 like autophagy activating	-2.570824236	1.679458391	1.243426	0.464967	2.283683	3.51641288	2.33928445	4.21263029	410	213	847
5300	NM_006221,NR_038422,NR_03	PIN1	peptidylprolyl cis/trans isomerase	1.491888829	1.679351652	17.928814	26.797031	28.319352	4.35113248	4.90483992	5.07023688	762	1519	1574
57551	NM_020791,NM_025142	TAOK1	TAO kinase 1	1.312938698	1.679351319	9.313333	12.032582	14.493723	6.76409988	7.15374093	7.50663987	4228	7417	8733
10966	NM_006822	RAB40B	RAB40B, member RAS oncogen	1.018076754	1.67934663	2.015343	2.053846	3.183559	2.15835987	2.17847216	2.7696269	136	185	281
286437	NR_039980	LOC286437	uncharacterized LOC286437	1.154339955	1.678745037	1.721751	1.989064	2.721644	2.29192162	2.45916281	2.91535666	153	236	316
100302650	NR_028308	BRE-AS1	BRE antisense RNA 1	2.501126862	1.678741563	0.210173	0.553483	0.347017	0.43999062	0.92294555	0.6785857	14	47	29
2632	NM_000158	GBE1	1,4-alpha-glucan branching enzy	2.055387298	1.678278838	7.088224	14.590792	11.182478	4.48515183	5.49119219	5.20593745	840	2307	1734
79730	NM_001330648,NM_024677	NSUN7	NOP2/Sun RNA methyltransferas	-1.245873665	1.677987201	3.204662	2.563984	5.043362	4.01166006	3.71626888	4.72189817	594	637	1226
6443	NM_000232	SGCB	sarcoglycan, beta (43kDa dystrop	1.308953787	1.677848615	4.503384	5.901181	7.103879	4.30540198	4.67653783	5.02231705	737	1289	1521
10025	NM_005481	MED16	mediator complex subunit 16	1.397800865	1.677113923	8.109115	11.354974	12.786678	4.59109862	5.0571683	5.31277821	907	1694	1871
10826	NM_032385	FAXDC2	fatty acid hydroxylase domain co	-3.845500254	1.676405998	5.564086	1.444129	8.770052	4.12340669	2.3978615	4.83508422	645	224	1330
23555	NM_001351263,NM_012339,NR	TSPAN15	tetraspanin 15	-1.802186053	1.676085946	42.711325	23.756927	67.260059	6.16610643	5.33234202	6.90309903	2780	2061	5731
29982	NM_001282405,NM_030759	NRBF2	nuclear receptor binding factor 2	-1.157322978	1.675861819	6.940877	6.0341	10.969369	3.79315817	3.5986027	4.49559213	505	583	1041
51528	NM_001098625,NM_001284201	JKAMP	JNK1/MAPK8-associated membr	1.335504771	1.675852318	27.598092	38.607256	45.377661	6.12194034	6.53412626	6.85848091	2695	4809	5555
23291	NM_012300,NM_033644,NM_03	FBXW11	F-box and WD repeat domain co	1.200295519	1.675740406	8.581362	10.356574	13.599932	5.28267998	5.53988865	6.0124986	1489	2388	3069
8273	NM_001142391,NM_001142392	SLC10A3	solute carrier family 10 member 3	1.667274592	1.675713857	8.112203	13.297145	12.574331	4.14225859	4.84676676	4.85380339	654	1457	1348
9375	NM_004800	TM9SF2	transmembrane 9 superfamily me	1.173585963	1.675535243	35.972519	42.264919	56.644352	6.95567706	7.18488512	7.6956189	4834	7580	9962
9425	NM_001143970,NM_001143971	CDYL	chromodomain Y like	1.413487933	1.675460163	4.346055	6.149853	6.835455	3.96771155	4.43981671	4.67471651	575	1086	1185
105378853	NR_135038,NR_135039,NR_14	LOC105378853	uncharacterized LOC105378853	1.993609964	1.675340134	0.817744	1.507655	1.269318	1.15225689	1.78245981	1.60891398	48	128	99
100268168	NR_026682,NR_026683	LOC100268168	uncharacterized LOC100268168	-1.105496358	1.675340134	0.935332	0.839012	1.351134	1.15225689	1.07428646	1.60891398	48	58	99
284339	NM_173633	TMEM145	transmembrane protein 145	-1.124842501	1.675340134	0.694551	0.621641	1.110153	1.15225689	1.06116576	1.60891398	48	57	99
8985	NM_001084	PLOD3	procollagen-lysine,2-oxoglutarate	-1.011489472	1.674989066	27.89921	27.613934	43.913464	6.35527652	6.33899711	7.09232986	3175	4194	6541
151188	NM_001350068,NM_152522,NR	ARL6IP6	ADP ribosylation factor like GTPa	1.171132022	1.674158316	15.138468	16.875684	19.852231	5.12944497	5.35132998	5.85623592	1335	2089	2749
26504	NM_020184	CNNM4	cyclin and CBS domain divalent r	1.348360937	1.674005906	8.126967	10.975491	12.789294	5.28550924	5.70715386	6.01388634	1492	2688	3072
5357	NM_001145319,NM_001172312	PLS1	plastin 1	-1.196367366	1.673285583	20.786803	17.391157	32.640214	6.24816313	5.99321473	6.98321152	2945	3289	6061
51062	NM_001127713,NM_015915,NM	ATL1	atlastin GTPase 1	3.597669329	1.673090927	0.298679	1.142974	0.508856	0.88013425	2.01116974	1.26797492	33	159	68
55370	NR_003505	PPP4R1L	protein phosphatase 4 regulatory	-1.043622346	1.672469721	3.423137	3.282972	5.382146	3.16959281	3.11495144	3.84581243	314	402	646
2782	NM_001282538,NM_001282539	GNB1	G protein subunit beta 1	1.140801553	1.672436938	59.809568	68.41869	94.142606	7.54603125	7.73512861	8.2848848	7298	11124	15012
337867	NM_001144072,NM_177967,NR	UBAC2	UBA domain containing 2	1.384498413	1.671828005	17.552198	24.507838	27.576392	5.55929747	6.02016213	6.28841171	1812	3352	3726
729041	NR_045483	FAAHP1	fatty acid amide hydrolase pseud	1.056435708	1.671713436	0.689417	0.731533	1.101356	0.89996265	0.93737891	1.29257653	34	48	70
54845	NM_001034915,NM_001122825	ESRP1	epithelial splicing regulatory prote	-1.008812841	1.671574133	56.333427	55.643679	87.65166	7.66975434	7.65715806	8.40812047	7955	10536	16355
79774	NM_001286732,NM_001286733	GRTP1	growth hormone regulated TBC p	-5.646794228	1.671355979	3.346242	0.528342	4.522096	2.46828542	0.84852988	3.10091148	178	42	366
113402	NM_145169,NR_130112,NR_13	SFT2D1	SFT2 domain containing 1	1.319197614	1.671222067	14.565135	19.257941	22.807736	4.04987877	4.42836625	4.75546927	611	1077	1256
440836	NM_001014440	ODF3B	outer dense fiber of sperm tails 3	-2.687343969	1.671039107	8.729489	3.243356	13.721148	3.2098649	2.02475093	3.88678411	324	161	666
409	NM_001257328,NM_001257329	ARRB2	arrestin beta 2	1.083319939	1.670606374	2.42464	2.651878	3.878728	2.48807034	2.58366954	3.12165574	181	262	372
9585	NM_001284259,NM_016195	KIF20B	kinesin family member 20B	-1.236032695	1.670401949	12.724494	10.337627	19.986514	6.32121441	6.0197383	7.05417101	3100	3351	6369
54867	NM_001083590,NM_017727	TMEM214	transmembrane protein 214	1.240129138	1.670095069	23.681053	29.46422	37.195097	6.15170641	6.45827295	6.88349514	2752	4560	5653
4037	NM_002333	LRP3	LDL receptor related protein 3	1.449715757	1.669978716	0.904548	1.313604	1.417917	2.09095564	2.51796519	2.68852679	128	248	263
8578	NM_003693,NM_145350,NR_02	SCARF1	scavenger receptor class F mem	-1.577980416	1.669949563	0.672649	0.424082	1.066654	1.73025772	1.30327576	2.28470378	91	77	187
653820	NM_001100910,NM_001320149	FAM72B	family with sequence similarity 72	-1.313143094	1.669701867	18.591913	13.682065	28.387852	5.38559376	5.00330679	6.11131559	1602	1630	3290
22933	NM_001193286,NM_012237,NM	SIRT2	sirtuin 2	1.081248097	1.669622892	11.45137	12.416969	18.007229	4.57268608	4.68083331	5.28775695	895	1293	1838
5042	NM_030979	PABPC3	poly(A) binding protein cytoplasm	-1.882326712	1.669513054	0.179204	0.094481	0.28606	0.5190475	0.29720487	0.78645063	17	12	35
643719	NR_027620	SCGB1B2P	secretoglobin family 1B member	1.48232253	1.669086186	0.696574	1.042123	1.094738	1.26358189	1.62195412	1.73991364	55	109	113
6884	NM_005645	TAF13	TATA-box binding protein associ	1.245768518	1.668704683	7.69234	9.584705	12.089785	2.41415945	2.67693481	3.04046258	170	283	349
8644	NM_001253908,NM_001253909	AKR1C3	aldo-keto reductase family 1 men	3.659523803	1.668551834	1.997536	7.357027	3.160036	1.76310734	3.28823882	2.32103228	94	460	193
55526	NM_018706	DHTKD1	dehydrogenase E1 and transketc	-1.293445672	1.668330879	7.905956	6.118063	12.397738	5.36344926	5.00244904	6.0877878	1577	1629	3236
127933	NM_001184763,NM_144624,NM	UHMK1	U2AF homology motif kinase 1	1.159446473	1.668233801	10.876367	12.626012	17.077451	6.51407431	6.72534408	7.24607717	3549	5498	7282
84878	NM_001316978,NM_001316979	ZBTB45	zinc finger and BTB domain cont	1.00737764	1.667676175	4.979756	5.058761	7.734158	3.63355401	3.64330938	4.32422401	448	603	919
2947	NM_000849,NR_024537	GSTM3	glutathione S-transferase mu 3	1.317462957	1.66738021	3.354716	4.429475	5.262668	3.85794988	4.23160161	4.55526505	530	933	1087
23645	NM_014330	PPP1R15A	protein phosphatase 1 regulatory	-1.040123638	1.667289703	80.160027	77.148369	125.59713	7.54681754	7.49037121	8.28124	7302	9380	14974
9630	NM_004297	GNA14	G protein subunit alpha 14	-1.584035663	1.666926336	0.392433	0.249704	0.62112	0.97665845	0.68708806	1.38699077	38	32	78
23370	NM_001130955,NM_015318	ARHGEF18	Rho/Rac guanine nucleotide excl	-1.023789495	1.666730658	6.94492	6.690755	10.717656	5.23469001	5.20167964	5.95634611	1439	1878	2950

9341	NM_004781	VAMP3	vesicle associated membrane pro	1.000727547	1.666585428	25.234903	25.276016	39.520229	5.78599633	5.78702662	6.5124257	2127	2844	4360
149175	NM_001031740,NM_001113482	MANEAL	mannosidase endo-alpha like	1.85846309	1.666339967	8.076097	15.330785	12.888391	4.528787	5.39381491	5.24033109	867	2153	1777
80115	NM_025045	BAIAP2L2	BAI1 associated protein 2 like 2	-6.255047348	1.665782009	2.969219	0.468742	4.65111	2.85098947	0.99371033	3.5052118	244	52	500
90317	NM_178523,NR_135823	ZNF616	zinc finger protein 616	-1.415807225	1.665142295	1.488056	1.049204	2.30998	2.87121968	2.4491251	3.5261099	248	234	508
4236	NM_005926	MFAP1	microfibrillar associated protein 1	1.305729767	1.664900229	12.455935	16.285749	19.503286	4.70255223	5.0744123	5.41575311	983	1715	2013
79739	NM_001350214,NM_001350215	TTLL7	tubulin tyrosine ligase like 7	2.145139041	1.66486597	0.196679	0.425739	0.308211	1.35261029	2.11639955	1.84344662	61	175	125
647946	NR_024391	MIR924HG	MIR924 host gene	-1.206938717	1.664846641	1.247051	1.040562	1.96438	1.8573935	1.66595383	2.42483178	103	114	211
80071	NM_025004	CCDC15	coiled-coil domain containing 15	-1.186686646	1.664565098	1.970549	1.666117	3.087561	3.08551061	2.86987209	3.75135692	294	331	602
642633	NR_108107	FAM230B	family with sequence similarity 23	2.377197821	1.663932396	0.657882	1.574412	1.033372	1.65056839	2.60642858	2.18966672	84	267	172
6714	NM_005417,NM_198291	SRC	SRC proto-oncogene, non-recept	1.245103648	1.663909631	13.365514	16.790567	20.944077	5.75707097	6.06809029	6.48099527	2084	3467	4265
10295	NM_001122957,NM_001271926	BCKDK	branched chain ketoacid dehydro	1.367633167	1.663867976	8.755935	12.108686	13.747814	4.29233333	4.72413992	4.99727909	730	1334	1494
83394	NM_001165966,NM_031220	PITPNM3	PITPNM family member 3	-1.117945515	1.66359832	1.495993	1.342462	2.342969	3.51641288	3.37031674	4.19968089	410	490	839
10810	NM_001291965,NM_006646	WASF3	WAS protein family member 3	2.082996969	1.66331917	1.525843	3.177181	2.380005	3.03260384	3.99682365	3.6947989	282	785	577
23400	NM_001141973,NM_001141974	ATP13A2	ATPase 13A2	1.260246186	1.662857451	7.356641	9.148119	11.208337	4.86394356	5.18740997	5.57777974	1104	1859	2258
10389	NM_006089,NR_033717	SCML2	sex comb on midleg like 2 (Drosc	-3.280106059	1.66243029	1.117521	0.348893	1.738873	2.46162969	1.24644988	3.08691439	177	72	362
5452	NM_001207025,NM_001207026	POU2F2	POU class 2 homeobox 2	1.808155765	1.662191642	0.159198	0.292981	0.251173	1.06712766	1.57657016	1.4969322	43	104	88
84433	NM_001324281,NM_032415	CARD11	caspase recruitment domain fam	1.091134598	1.661695421	8.427476	9.210705	13.215659	5.23273681	5.35536651	5.95007726	1437	2095	2937
440689	NM_001024599,NM_001161334	HIST2H2BF	histone cluster 2 H2B family men	2.208344713	1.661373503	2.321374	4.63453	3.608409	1.08456011	1.79836126	1.51794971	44	130	90
8507	NM_001256574,NM_001256575	ENC1	ectodermal-neural cortex 1	-3.659416704	1.661348247	27.309436	7.434449	42.967368	7.22495136	5.37869051	7.95347276	5834	2130	11921
390928	NM_001004318	ACP7	acid phosphatase 7, tartrate resis	-1.204499132	1.66128247	2.018703	1.679278	3.159206	2.77249458	2.54649137	3.41845388	229	254	468
10157	NM_005763	AASS	aminoadipate-semialdehyde synt	-1.374370191	1.661221628	1.432067	1.042907	2.239689	3.20189991	2.80050884	3.8705348	322	313	658
57690	NM_001142640,NM_018996	TNRC6C	trinucleotide repeat containing 6C	1.467492539	1.660978024	0.617606	0.907204	0.963625	2.78321062	3.26839621	3.42958826	231	453	472
1650	NM_005216	DDOST	dolichyl-diphosphooligosaccharid	1.450041311	1.660667112	125.880554	182.730392	196.443054	8.03105016	8.56543599	8.76062341	10230	19820	20895
83543	NM_001185095,NM_001185096	AIF1L	allograft inflammatory factor 1 like	1.10886948	1.660424992	9.711878	10.782186	15.151053	5.06723449	5.21210456	5.78161868	1277	1892	2608
10960	NM_006816	LMAN2	lectin, mannose binding 2	1.110162801	1.66034115	94.57299	105.109161	147.561371	7.40680151	7.55673119	8.13490568	6623	9824	13525
27044	NM_014390	SND1	staphylococcal nuclease and tudo	-1.18277684	1.660285737	28.982712	24.532026	45.221043	6.707912	6.46824743	7.43386048	4065	4592	8301
730338	NR_134575	LOC730338	uncharacterized LOC730338	1.426753538	1.659696884	0.29308	0.421471	0.461572	0.64188297	0.84852988	0.95003448	22	42	45
64129	NM_001204414,NM_001204415	TINAGL1	tubulointerstitial nephritis antigen	1.311757238	1.659545609	145.723923	191.330587	227.192875	8.31282824	8.70325326	9.04181744	12445	21812	25402
23112	NM_001024843,NM_001162501	TNRC6B	trinucleotide repeat containing 6E	1.313147135	1.659407902	1.919776	2.519693	2.992972	5.10935964	5.49241457	5.82337232	1316	2309	2686
23635	NM_001256732,NM_001256733	SSBP2	single stranded DNA binding prot	2.782665036	1.659127475	0.134475	0.377531	0.208884	1.13562816	2.11639955	1.57922529	47	175	96
84336	NM_001304813,NM_001304814	TMEM101	transmembrane protein 101	1.022571496	1.659091812	27.068126	27.699186	42.258251	5.42632706	5.45779006	6.14336932	1649	2253	3365
56957	NM_020205	OTUD7B	OTU deubiquitinase 7B	1.172328706	1.658262913	2.667307	3.133237	4.160958	4.14433812	4.36170552	4.84135097	655	1026	1336
23065	NM_001271427,NM_001271428	EMC1	ER membrane protein complex s	1.512404165	1.658202503	13.398633	20.307653	20.877982	6.46000163	7.05129764	7.18312086	3417	6905	6969
27336	NM_001163280,NM_014500	HTATSF1	HIV-1 Tat specific factor 1	1.13554486	1.658043556	35.04845	38.888738	53.831721	6.57893411	6.76052126	7.30243141	3714	5635	7574
11094	NM_001135775,NM_001242369	CACFD1	calcium channel flower domain c	1.418622147	1.657999942	4.080633	5.923993	6.352838	3.58232086	4.05092325	4.26329596	431	817	879
100652730	NR_046224,NR_046225	LINC00659	long intergenic non-protein codin	-1.039117497	1.657782566	1.799343	1.798976	2.864149	1.16869613	1.1381576	1.61867597	49	63	100
253260	NM_001285439,NM_001285440	RICTOR	RPTOR independent companion	-1.067336018	1.657642466	3.374112	3.156018	5.261479	5.02044968	4.92941706	5.73189018	1235	1546	2518
83752	NM_001300948,NM_001348078	LONP2	lon peptidase 2, peroxisomal	1.271282804	1.657586043	12.377933	15.648726	18.934185	5.8943731	6.23548722	6.61383023	2296	3900	4681
92815	NM_033445	HIST3H2A	histone cluster 3 H2A	1.390267756	1.657525749	28.527466	39.714642	44.418591	3.88556162	4.33333585	4.5754555	541	1005	1103
3959	NM_005567	LGALS3BP	galectin 3 binding protein	1.056096456	1.657444144	537.48645	568.260925	837.147888	10.20905667	10.28773371	10.93753476	46432	65519	94654
3831	NM_001130107,NM_005552,NM	KLC1	kinesin light chain 1	1.196435448	1.657226188	29.533052	35.34249	45.832641	6.19899112	6.45451467	6.91997492	2845	4548	5799
64710	NM_022731	NUCKS1	nuclear casein kinase and cyclin	1.183679429	1.657191576	50.476353	59.812107	78.605637	8.31594502	8.55852267	9.04289409	12472	19725	25421
23167	NM_001323553,NM_001323554	EFR3A	EFR3 homolog A	1.197375708	1.656063985	12.632246	15.171506	19.790967	6.11612463	6.37257735	6.83564348	2684	4294	5467
100507436	NM_000247,NM_001177519,NM	MICA	MHC class I polypeptide-related	-1.077155128	1.655840693	9.87414	9.780992	16.682145	4.02300634	3.92259242	4.71508918	599	743	1220
9024	NM_001256627,NM_001256629	BRSK2	BR serine/threonine kinase 2	1.196653324	1.655683472	0.142907	0.175689	0.223102	0.71085839	0.81765451	1.03996283	25	40	51
26270	NM_018438	FBXO6	F-box protein 6	-1.641147572	1.655570455	5.074099	3.096938	7.907969	3.1155	2.50348775	3.77555686	301	245	613
11100	NM_001301016,NM_001321208	HNRNPUL1	heterogeneous nuclear ribonucle	-1.021965507	1.65543109	54.4909	53.783705	85.732616	7.67946675	7.64827441	8.40389261	8009	10471	16307
100506023	NR_037845	LOC100506023	uncharacterized LOC100506023	-1.649822211	1.654550601	0.11801	0.073085	0.185612	0.73313634	0.48576948	1.06873498	26	21	53
8751	NM_001261464,NM_001261465	ADAM15	ADAM metallopeptidase domain	-1.079026007	1.654347078	35.936465	33.261765	55.480673	6.66365255	6.55504398	7.38429106	3941	4880	8019
79879	NM_001304797,NM_024821	CCDC134	coiled-coil domain containing 134	1.418929337	1.653296187	1.695925	2.488578	2.756357	1.6852613	2.05153568	2.22204636	87	165	177
118738	NM_001346932,NM_001346933	ZNF488	zinc finger protein 488	-1.540124407	1.652851583	2.539852	1.666486	4.038398	3.35339967	2.8044511	4.02166185	362	314	736
51246	NM_001272065,NM_001272066	SHISA5	shisa family member 5	1.040545931	1.652827989	44.839521	45.323127	68.963992	6.52927225	6.5860058	7.24804396	3587	4987	7292
10466	NM_001161520,NM_006348,NM	COG5	component of oligomeric golgi co	-1.571384046	1.652571757	4.611236	2.941758	7.105618	4.5618359	3.94419393	5.26229407	888	755	1805
102465454	NR_106816	MIR6758	microRNA 6758	1.175440748	1.652526648	11.446328	13.625328	17.820749	0.77668602	0.8787583	1.12461302	28	44	57
3795	NM_000221,NM_006488	KHK	ketohexokinase	1.594863996	1.6521023	0.975023	1.574101	1.516483	1.73025772	2.23214197	2.27238811	91	194	185
8611	NM_003711,NM_176895,NR_10	PLPP1	phospholipid phosphatase 1	1.134135471	1.651963842	13.246824	15.053599	20.525803	4.49496323	4.66898984	5.19374412	846	1282	1719
5799	NM_001308267,NM_001308268	PTPRN2	protein tyrosine phosphatase, rec	-2.152500781	1.651798233	0.986735	0.459402	1.593158	2.52684435	1.68318474	3.14885876	187	116	380
949	NM_001082959,NM_005505	SCARB1	scavenger receptor class B mem	1.600577932	1.65125866	24.36989	38.840565	37.563774	6.02538876	6.69566961	6.74021788	2518	5385	5114
71	NM_001199954,NM_001614,NR	ACTG1	actin gamma 1	1.396215776	1.650587392	1356.303341	1896.427647	2104.753424	11.35597675	11.83734289	12.07873993	102867	191900	208832
28962	NM_014028	OSTM1	osteopetrosis associated transme	1.180094015	1.650012013	2.170031	2.564016	3.367883	3.38540081	3.60313623	4.05259921	371	585	753
9040	NM_003969	UBE2M	ubiquitin conjugating enzyme E2	1.198225837	1.649533488	45.201813	54.235386	70.084625	6.08939006	6.34679007	6.80310481	2634	4217	5344
4763	NM_000267,NM_001042492,NM	NF1	neurofibromin 1	1.095030064	1.649451251	10.440883	11.414196	16.774077	6.95656491	7.08652976	7.67398341	4837	7077	9813
55234	NM_018225	SMU1	DNA replication regulator and spl	1.485895256	1.649248848	5.298401	7.886152	8.215929	5.23273681	5.79150248	5.93940598	1437	2853	2915
643414	NM_001080518	LIPK	lipase family member K	-1.1869219	1.649235075	0.676883	0.576839	1.081444	0.86002952	0.75384785	1.23026757	32	36	65
2664	NM_001493	GDI1	GDP dissociation inhibitor 1	1.030388716	1.649143063	57.261051	59.065964	88.747322	7.12239926	7.16528339	7.84004604	5431	7477	11016
3315	NM_001540	HSPB1	heat shock protein family B (sma	-1.238163459	1.649010933	300.068909	242.610062	464.990509	8.03931515	7.73241531	8.75876229	10289	11103	20868
84267	NM_001317997,NM_032307	C9orf64	chromosome 9 open reading fran	1.451759409	1.64888943	5.634239	8.26146	8.780844	3.86300966	4.36970972	4.54506286	532	1032	1079
133957	NM_145265	CCDC127	coiled-coil domain containing 127	1.234790989	1.648858282	9.146779	11.313231	14.178264	3.77179482	4.0558926	4.45120991	497	820	1008
163071	NM_001331097,NM_001331098	ZNF114	zinc finger protein 114	1.171197098	1.648614633	0.719784	0.847175	1.105373	1.46336426	1.61299082	1.96321628	69	108	140
149478	NM_001136537	BTBD19	BTB domain containing 19	-1.166691245	1.648614633	1.143865	0.98028	1.776866	1.46336426	1.32539385	1.96321628	69	79	140
10959	NM_001321445,NM_006815	TMED2	transmembrane p24 trafficking pr	1.075656388	1.648241762	100.72003	108.46938	155.994851	7.71085112	7.81558532	8.42907592	8186	11765	16595
284114	NM_001320444,NM_178518	TMEM102	transmembrane protein 102	1.089700712	1.648039181	2.255824	2.466909	3.502923	2.49460548	2.597368	3.11132089	182	265	369
29099	NM_001101653,NM_001307932	COMMD9	COMM domain containing 9	2.671746446	1.648005291	3.620231	9.525163	5.532194	3.50026652	4.83621478	4.17011265	405	1446	821
1997	NM_001145353,NM_172373	ELF1	E74 like ETS transcription factor	1.316361493	1.647823457	8.094632	10.688554	12.575854	4.929321	5.31448374	5.63120109	1157	2035	2345
10133	NM_001008211,NM_001008212	OPTN	optineurin	-1.032730409	1.647816384	15.371777	14.985845	23.867138	5.73791921	5.69233762	6.44783842	2056	2660	4167
6623	NM_001330120,NM_003087	SNCG	synuclein gamma	1.675444385	1.647571857	23.490929	39.216632	36.68623	4.33665583	5.05220335	5.02873806	754	1688	1528
126068	NM_152355	ZNF441	zinc finger protein 441	1.275464456	1.646996459	1.35224	1.724689	2.090891	2.77786255	3.08285145	3.4128543	230	392	466
89797	NM_001111018,NM_001111019	NAV2	neuron navigator 2	-1.26545278	1.646791692	3.029045	2.321219	4.617325	5.04180235	4.71369658	5.74420525	1254	1324	2540
26277	NM_001099274,NM_012461	TINF2	TERF1 interacting nuclear factor	1.225937302	1.646412899	9.567266	11.702915	14.505576	4.34210161	4.62285328	5.03330706	757	1240	1533
5525	NM_001199756,NM_006243	PPP2R5A	protein phosphatase 2 regulatory	-1.120131358	1.646315349	11.401298	10.191989	17.640222	5.15957233	5.00073201	5.86292511	1364	1627	2762
3964	NM_006499,NM_201543,NM_20	LGALS8	galectin 8	-1.089429679	1.645899594	19.508938	17.929419	30.179935	6.89614501	6.77365178	7.61027387	4637	5687	9387
1265	NM_001303499,NM_001303501	CNN2	calponin 2	-1.013924479	1.645869157	74.983816	74.083118	116.09683	7.5089873	7.48914709	8.22473441	7112	9372	14397
79177	NM_001145347,NM_024327	ZNF576	zinc finger protein 576	1.334775742	1.645298487	2.920712	3.881848	4.489563	2.65489157	3.01302298	3.28075123	208	371	421
5316	NM_001286258,NM_001320694	PKNOX1	PBX/knotted 1 homeobox 1	1.234406685	1.645163809	3.246526	4.048916	5.086801	4.10430518	4.39215006	4.78935776	636	1049	1287
4779	NM_001330261,NM_001330262	NFE2L1	nuclear factor, erythroid 2 like 1	1.146248925	1.645128647	59.481459	68.38669	92.040726	8.13810323	8.33437185	8.85429977	11021	16879	22300
7855	NM_003468	FZD5	frizzled class receptor 5	1.602499744	1.645068139	1.519133	2.442648	2.351931	3.42357323	4.05258161	4.08816658	382	818	773
161	NM_001242837,NM_001242837,NR	AP2A2	adaptor related protein complex 2	1.094012136	1.645064007	6.296827	6.960838	9.840793	4.87275002	4.99815263	5.57151066	1111	1624	2248
10723	NM_006598	SLC12A7	solute carrier family 12 member 7	1.068651677	1.645002849	10.799931	11.554404	16.698263	5.82735481	5.9215178	6.53547665	2190	3127	4431
6793	NM_005990	STK10	serine/threonine kinase 10	1.319542919	1.644800891	6.218522	8.214239	9.611329	5.22686128	5.61756579	5.92963585	1431	2523	2895
84947	NM_032861,NR_073096	SERAC1	serine active site containing 1	-1.146053381	1.643671094	1.186647	1.041231	1.912523	2.48807034	2.32837375	3.10091148	181	211	366
7272	NM_001166691,NM_003318	TTK	TTK protein kinase	1.123632372	1.643574277	19.764615	22.244845	30.540622	5.88196413	6.04744731	6.58921703	2276	3417	4601
54849	NM_001242816,NM_001242817	DEF8	differentially expressed in FDCP	-1.032426438	1.643431533	21.616884	19.828082	31.737779	5.37589203	5.33097613	6.07897551	1591	2059	3216
23424	NM_001302884,NM_014290	TDRD7	tudor domain containing 7	-1.073432744	1.643238933	7.556749	7.045105	11.667409	4.84232994	4.74377637	5.53910756	1087	1353	2197
55236	NM_018227	UBA6	ubiquitin like modifier activating e	1.038415589	1.642805429	22.89061	23.799999	35.343559	7.17700078	7.23101687	7.88926729	5642	7828	11400
2745	NM_001118890,NM_001243658	GLRX	glutaredoxin	-1.127784744	1.642653134	12.248356	10.718436	18.328619	3.8730763	3.71207778	4.55017297	536	635	1083
374868	NM_001306085,NM_198531	ATP9B	ATPase phospholipid transportin	-1.174898855	1.642466448	6.972112	5.939869	10.770852	4.96623093	4.74172192	5.66398036	1188	1351	2400
92359	NM_139161,NM_174881,NM_17	CRB3	crumbs 3, cell polarity complex co	1.105783596	1.642182835	11.93387	13.115949	17.306895	3.4839374	3.61665196	4.14837451	400	591	808
122830	NM_001011713	NAA30	N(alpha)-acetyltransferase 30, N	1.250961579	1.642033192	4.443438	5.56951	6.863031	4.34933082	4.65813802	5.03695187	761	1272	1537
9194	NM_001270622,NM_001270623	SLC16A7	solute carrier family 16 member 7	-1.563482294	1.641201622	0.932861	0.597453	1.438205	3.5668994	2.98897501	4.23342827	426	364	860

83742	NM_031484	MARVELD1	MARVEL domain containing 1	-1.460660148	1.640980383	17.624655	12.079622	27.178427	5.82151855	5.2865704	6.52610593	2181	1995	4402
255374	NM_203397	MBLAC1	metallo-beta-lactamase domain c	-1.51264311	1.640635702	0.887264	0.585586	1.360027	1.2010231	0.89363826	1.64757178	51	45	103
7638	NM_001297588,NM_001297589	ZNF221	zinc finger protein 221	-1.052588534	1.640356805	0.593489	0.556762	0.912947	1.21691895	1.1751656	1.66651905	52	66	105
253430	NM_152230	IPMK	inositol polyphosphate multikinas	-1.0711401	1.640213042	1.203873	1.127429	1.860013	3.0370876	2.95035633	3.68088855	283	353	571
22836	NM_014899	RHOBTB3	Rho related BTB domain containi	1.340099012	1.639858908	8.863494	11.890399	13.657146	5.61584323	6.03071759	6.31792276	1886	3377	3804
2132	NM_000401,NM_001178083,NM	EXT2	exostosin glycosyltransferase 2	1.035173907	1.639720147	27.32048	28.336406	41.810564	6.5949886	6.64435595	7.30262068	3756	5195	7575
1627	NM_004395,NM_080881	DBN1	drebrin 1	1.793900288	1.639279799	37.229379	66.800614	57.36901	6.75119512	7.58837191	7.45903998	4190	10043	8448
23420	NM_014287	NOMO1	NODAL modulator 1	1.368138809	1.639057876	24.319189	33.30661	37.456135	6.66618756	7.11458516	7.373521	3948	7217	7959
9358	NM_001271754,NM_001271755	ITGBL1	integrin subunit beta like 1	-1.076822104	1.638313976	47.060993	44.731462	75.979623	7.01937923	6.91345103	7.72726419	5054	6271	10184
54472	NM_001318512,NM_001318514	TOLLIP	toll interacting protein	1.234941252	1.637274591	3.64208	4.47466	5.573101	3.78784692	4.07233457	4.45802288	503	830	1013
161291	NM_001017970	TMEM30B	transmembrane protein 30B	1.071619901	1.637031948	20.989689	22.518391	32.28756	6.52009264	6.61883783	7.2250627	3564	5103	7176
375743	NM_001099666	PTAR1	protein prenyltransferase alpha s	1.544456394	1.636944299	6.42335	9.930838	9.880078	5.99690152	6.61603673	6.699115	2468	5093	4969
136306	NM_001139456,NM_001331192	SVOPL	SVOP like	-2.665440911	1.636697095	1.008594	0.377931	1.560356	1.47662927	0.73744528	1.97085819	70	35	141
283987	NM_030630	HID1	HID1 domain containing	-1.176143746	1.636602738	18.123051	15.425661	27.883032	5.90667624	5.67683012	6.60802251	2316	2631	4662
55653	NM_001010974,NM_017843,NM	BCAS4	breast carcinoma amplified seque	-2.670683391	1.636496726	6.232714	2.303141	9.721842	3.13654562	1.96964178	3.782087	306	153	616
126969	NM_001114106,NM_001258340	SLC44A3	solute carrier family 44 member 3	1.255880987	1.636042107	3.504893	4.462649	5.52574	3.19389071	3.49019467	3.84165053	320	537	644
9213	NM_001135669,NM_001328662	XPR1	xenotropic and polytropic retrovir	1.363385608	1.635396365	3.862097	5.257948	5.900298	4.99992509	5.43508445	5.69199148	1217	2217	2448
833	NM_001014437,NM_001194997	CARS	cysteinyl-tRNA synthetase	1.267507976	1.635032617	23.439118	29.38053	34.990274	5.8769705	6.21378915	6.57675114	2268	3841	4561
23534	NM_001191028,NM_012470,NR	TNPO3	transportin 3	-1.407533897	1.634939151	17.410008	12.406752	26.780554	6.24380564	5.75835243	6.9456516	2936	2787	5904
56137	NM_018903,NM_031864	PCDHA12	protocadherin alpha 12	-1.705724287	1.634938247	2.220961	1.307616	3.398281	3.64534899	2.95391015	4.30923152	452	354	909
80195	NM_001270367,NM_001270368	TMEM254	transmembrane protein 254	-1.379147045	1.634842614	9.296889	6.734508	14.199555	4.30354226	3.86712977	4.98412333	736	713	1480
8407	NM_001277223,NM_001277224	TAGLN2	transgelin 2	1.419514682	1.634564671	253.716086	360.700026	390.032486	8.45416895	8.95835383	9.16148215	13730	26041	27603
26269	NM_012180	FBXO8	F-box protein 8	-1.053069042	1.633995436	5.164786	4.905194	7.926143	3.79580649	3.72669368	4.46345019	506	642	1017
54826	NM_001317954,NM_017676,NR	GIN1	gypsy retrotransposon integrase	1.310271597	1.633969175	2.392546	2.997217	3.508664	3.1155	3.46550967	3.75799726	301	527	605
10156	NM_001079877,NM_006989	RASA4	RAS p21 protein activator 4	1.488614016	1.633751296	0.448916	0.670903	0.698924	1.79522562	2.22627631	2.3329413	97	193	195
56133	NM_018936	PCDHB2	protocadherin beta 2	1.170807637	1.633366056	3.073533	3.597261	4.7185	3.21778616	3.42255083	3.86439381	326	510	655
994	NM_001287516,NM_001287517	CDC25B	cell division cycle 25B	1.498404819	1.633188602	46.527303	68.803237	73.962006	7.33510429	7.91556484	8.03933739	6300	12613	12655
9525	NM_004869	VPS4B	vacuolar protein sorting 4 homolo	1.032165497	1.633133742	11.559362	11.946354	17.740114	5.28645109	5.33097613	5.979733	1493	2059	2999
23514	NM_001080394,NM_001282916	SPIDR	scaffolding protein involved in DN	-1.037786257	1.632702606	9.475605	9.139928	14.560176	5.00451141	4.95269469	5.69430137	1221	1572	2452
1263	NM_004073	PLK3	polo like kinase 3	1.186619298	1.63189081	7.228825	8.582198	11.084305	4.21529532	4.44991916	4.89153539	690	1094	1385
121512	NM_001304480,NM_001304481	FGD4	FYVE, RhoGEF and PH domain	-1.652952465	1.631535595	5.44139	2.650028	5.748708	4.96152109	4.26631627	5.64976818	1184	957	2376
139324	NM_001177478,NM_001177479	HDX	highly divergent homeobox	1.651236768	1.631221007	0.596424	1.006443	0.929053	2.23060596	2.8278811	2.81278744	145	320	291
6500	NM_006930,NM_170679	SKP1	S-phase kinase associated prote	1.267634598	1.631026745	80.501887	102.772995	124.363548	7.32574317	7.66598735	8.02805005	6259	10601	12556
10468	NM_006350,NM_013409	FST	follistatin	-7.316006922	1.630955117	6.796475	0.890398	10.489642	3.74737673	1.43116693	4.41104735	488	89	979
22925	NM_001007267,NM_001195641	PLA2R1	phospholipase A2 receptor 1	-2.035789512	1.630922051	0.731083	0.356798	1.095955	2.29940604	1.5486383	2.88736461	154	101	309
5494	NM_021003,NM_177951,NM_17	PPM1A	protein phosphatase, Mg2+/Mn2+	1.156927127	1.630796858	8.031903	9.489102	11.835443	5.74273104	5.94937984	6.43787878	2063	3189	4138
58493	NM_001329585,NM_001329586	INIP	INTS3 and NABP interacting prot	1.25620181	1.629991262	5.185402	6.570039	7.999683	4.44859483	4.76416158	5.12776797	818	1373	1640
8460	NM_003596	TPST1	tyrosylprotein sulfotransferase 1	1.526524545	1.629814016	2.548949	3.890497	3.907511	2.60744196	3.13387411	3.21791812	200	408	401
101867536	NR_109833	PRNCR1	prostate cancer associated non-c	1.067584274	1.629741267	0.419431	0.447432	0.642347	2.63136183	2.71093651	3.24338068	204	291	409
3133	NM_005516	HLA-E	major histocompatibility complex,	-1.373224089	1.629684605	42.179798	30.747612	64.593765	6.78557665	6.33286878	7.48512224	4292	4176	8603
57045	NM_020648	TWSG1	twisted gastrulation BMP signalin	1.31055144	1.629275272	19.47117	25.547222	29.816	6.17427094	6.55971624	6.87078697	2796	4896	5603
7392	NM_001321150,NM_003367,NM	USF2	upstream transcription factor 2, c	-1.175799035	1.629035635	33.633854	29.018395	52.332415	5.82929499	5.60009515	6.52351015	2193	2492	4394
22930	NM_001172435,NM_012233	RAB3GAP1	RAB3 GTPase activating protein	1.509108894	1.628799857	14.281921	21.585461	21.871443	6.11718378	6.70387133	6.81297027	2686	5416	5381
55229	NM_018216	PANK4	pantothenate kinase 4	1.177491178	1.628313489	10.452753	12.328391	16.003155	4.8216876	5.04971445	5.50530231	1071	1685	2145
10579	NM_001291876,NM_001291877	TACC2	transforming acidic coiled-coil co	-2.035002329	1.627861666	2.08675	0.6537	2.924505	3.55444243	2.65089709	4.20940382	422	277	845
7871	NM_001304420,NM_001304421	SLMAP	sarcolemma associated protein	-1.102937053	1.626759665	13.067204	12.489382	19.391172	5.93638283	5.79744877	6.62930477	2365	2865	4732
4598	NM_000431,NM_001114185,NM	MVK	mevalonate kinase	-1.160657481	1.625978469	7.165396	6.21741	10.984581	3.95355025	3.7534508	4.61866105	569	655	1138
81532	NM_001172223,NM_053005	MOB2	MOB kinase activator 2	1.148012597	1.625885237	5.187852	6.114125	8.311168	3.2098649	3.38881456	3.84996236	324	497	648
23522	NM_001256468,NM_001256469	KAT6B	lysine acetyltransferase 6B	-1.17430682	1.625316779	3.462289	2.980667	5.30331	4.89020329	4.666826	5.57213879	1125	1280	2249
100132707	NR_024476,NR_024477	PAXIP1-AS2	PAXIP1 antisense RNA 2	-1.195969777	1.625191537	1.364233	1.127392	1.96859	1.56621863	1.40024122	2.06668624	77	86	154
10558	NM_001281303,NM_006415,NM	SPTLC1	serine palmitoyltransferase long	1.243176972	1.624790546	66.110121	82.258782	99.940599	7.46015603	7.77258857	8.15726484	6874	11418	13737
79038	NM_001198953,NM_024071	ZFYVE21	zinc finger FYVE-type containing	-1.045001847	1.624592073	29.684693	28.540282	45.557543	5.4373936	5.37538151	6.12465092	1662	2125	3321
1943	NM_001405	EFNA2	ephrin A2	-1.708739921	1.624394455	0.49386	0.285316	0.759647	1.03161781	0.68708806	1.43197761	41	32	82
389203	NM_001145432	SMIM20	small integral membrane protein	1.313391804	1.624356652	13.029676	17.153191	19.909416	3.79580649	4.16417063	4.45530155	506	888	1011
3096	NM_002114	HIVEP1	human immunodeficiency virus ty	1.100491638	1.623728211	1.096453	1.20664	1.671729	3.38891307	3.51446439	4.03448121	372	547	743
7564	NM_001029976,NM_006958	ZNF16	zinc finger protein 16	1.474049312	1.623669379	3.622751	5.530082	5.720111	3.35699061	3.87089436	4.00128413	363	715	725
10906	NM_001143906,NM_006700	TRAFD1	TRAF-type zinc finger domain co	1.026971948	1.623631573	16.515068	16.991131	24.730437	5.44753406	5.48506467	6.13404048	1674	2297	3343
100506753	NR_126338,NR_126339	AZIN1-AS1	AZIN1 antisense RNA 1	-1.436500499	1.623604917	0.815398	0.590683	1.365944	0.77668602	0.58077243	1.11084509	28	26	56
54843	NM_001162951,NM_001162952	SYTL2	synaptotagmin like 2	-1.956260255	1.623439332	4.032145	2.770399	6.263548	4.1064401	3.21608274	4.77305948	637	435	1272
78998	NR_026785	RHPN1-AS1	RHPN1 antisense RNA 1 (head t	1.380160411	1.623413674	0.333525	0.466051	0.513783	0.73313634	0.93737891	1.05442063	26	48	52
54808	NM_017653	DYM	dymeclin	1.179715261	1.623351229	3.186135	3.771954	4.873414	3.19790087	3.41225421	3.83538509	321	506	641
55655	NM_001174081,NM_001174082	NLRP2	NLR family pyrin domain containi	1.342348036	1.623280225	16.895866	22.870791	25.909893	5.91522644	6.33389198	6.60495639	2330	4179	4652
8323	NM_001164615,NM_001164616	FZD6	frizzled class receptor 6	1.374891381	1.623158385	20.896984	27.496681	29.888576	6.16917354	6.62302931	6.86028209	2786	5118	5562
6845	NM_001145149_chrX,NM_0011	VAMP7	vesicle associated membrane pro	1.086898858	1.623046037	5.530627	5.974194	8.374283	3.9199564	4.03255443	4.58170751	555	806	1108
29966	NM_001083893,NM_014574	STRN3	striatin 3	1.147216436	1.622806453	12.734623	14.64239	19.482753	5.69529401	5.88986396	6.38309014	1995	3058	3982
100505812	NR_040599	CARD8-AS1	CARD8 antisense RNA 1	1.139759334	1.622777041	0.272746	0.317956	0.427262	0.61813834	0.68708806	0.90287695	21	32	42
6820	NM_004605,NM_177973	SULT2B1	sulfotransferase family 2B memb	-5.391977407	1.622673517	21.070503	3.871473	32.165839	4.68265777	2.47903109	5.35937359	969	240	1934
56995	NM_001007466,NM_020245	TULP4	tubby like protein 4	1.390708988	1.622235994	1.888758	2.628616	2.40847	4.13181574	4.5843929	4.79797547	649	1206	1295
10615	NM_006461	SPAG5	sperm associated antigen 5	1.024192451	1.621814651	41.801643	42.861977	63.710091	7.28532538	7.31959433	7.97939417	6085	8327	12138
29950	NM_013376	SERTAD1	SERTA domain containing 1	1.111472781	1.621793229	16.745459	18.627052	25.524305	4.36724694	4.5127108	5.03786164	771	1145	1538
285266	NR_040100	ENTPD3-AS1	ENTPD3 antisense RNA 1	1.679433165	1.621745281	0.201349	0.341513	0.308745	0.49317359	0.75384785	0.73352601	16	36	32
29117	NM_001173984,NM_013263	BRD7	bromodomain containing 7	1.126341013	1.62161718	30.958584	34.918418	47.193074	6.1428929	6.31225092	6.83250109	2735	4116	5455
2749	NR_048575	GLUD1P3	glutamate dehydrogenase 1 pseu	1.988770978	1.621457	0.417129	0.86186	0.657979	0.46682716	0.81765451	0.69713258	15	40	30
54933	NM_001304746,NM_017821	RHBDL2	rhomboid like 2	-1.407235103	1.621158589	9.31933	6.637228	14.213027	4.17923909	3.71835988	4.84551373	672	638	1340
91664	NM_001321522,NM_001321523	ZNF845	zinc finger protein 845	-1.788267732	1.621143724	2.299479	1.286954	3.578513	3.79580649	3.03667667	4.45257508	506	378	1009
26035	NM_001324091,NM_001324092	GLCE	glucuronic acid epimerase	1.210610228	1.620931567	5.153272	6.220557	7.989566	4.66538426	4.93122105	5.34032578	957	1548	1908
6016	NM_001256820,NM_001256821	RIT1	Ras like without CAAX 1	-1.096823173	1.620887604	6.121058	5.65507	9.242718	4.40589631	4.27912189	5.07644857	793	966	1581
221687	NM_001165032,NM_001165033	RNF182	ring finger protein 182	-1.442196925	1.620748525	0.090021	0.063674	0.144975	0.41264541	0.29720487	0.62147005	13	12	26
79745	NM_001287527,NM_001287528	CLIP4	CAP-Gly domain containing linke	-1.246469524	1.620639403	34.858061	27.964216	53.072311	7.14568271	6.83033724	7.83834994	5520	5917	11003
259232	NM_001350748,NM_001350749	NALCN	sodium leak channel, non-selecti	-2.476620042	1.62056039	1.129563	0.454137	1.725879	3.13654562	2.05153568	3.76899703	306	165	610
1050	NM_001285829,NM_001287424	CEBPA	CCAAT/enhancer binding protein	4.9542989	1.620306547	0.119322	0.593955	0.182107	0.38477185	1.33632702	0.58209675	12	80	24
10424	NM_006320	PGRMC2	progesterone receptor membrane	1.255528907	1.61989956	18.173655	22.84437	27.661913	6.08885029	6.41283705	6.77664393	2633	4417	5246
8027	NM_001324282,NM_001324283	STAM	signal transducing adaptor molec	1.143534607	1.619894647	12.518962	14.284092	18.955663	5.58478799	5.77452033	6.26917183	1845	2819	3676
6920	NM_003196	TCEA3	transcription elongation factor A3	-1.470249118	1.619886687	2.118952	1.437933	3.227978	2.20692365	1.79043244	2.77836293	142	129	283
10085	NM_001278642,NM_005711	EDIL3	EGF like repeats and discoidin do	1.6268495	1.619785021	0.054961	0.098709	0.090304	0.35634914	0.54351856	0.54161868	11	24	22
5583	NM_006255	PRKCH	protein kinase C eta	-1.106250518	1.619651955	3.486135	3.157598	5.309507	3.81942505	3.68453475	4.47558749	515	622	1026
6002	NM_002926,NM_198227,NM_19	RGS12	regulator of G protein signaling 1	-1.006757898	1.619548477	3.28154	3.269796	4.944768	4.33847337	4.32923711	5.00660315	755	1002	1504
84376	NM_032410	HOOK3	hook microtubule tethering protei	1.019408011	1.619289267	2.395473	2.446083	3.647217	5.11679207	5.14373402	5.79620902	1323	1802	2635
7111	NM_001166116,NM_003275	TMOD1	tropomodulin 1	-1.112099584	1.619160345	0.078141	0.070409	0.119584	0.3273552	0.29720487	0.49997203	10	12	20
4689	NM_000631,NM_013416	NCF4	neutrophil cytosolic factor 4	3.49098465	1.619160345	0.159229	0.625694	0.240972	0.3273552	0.92294555	0.49997203	10	47	20
151	NM_000682	ADRA2B	adrenoceptor alpha 2B	-1.112099584	1.619160345	0.077661	0.066847	0.11873	0.3273552	0.29720487	0.49997203	10	12	20
100506495	NR_103553,NR_103554	LIFR-AS1	LIFR antisense RNA 1	3.46340288	1.618398561	0.055105	0.216661	0.094679	0.29776659	0.84852988	0.45708734	9	42	18
29924	NM_001130071,NM_001130072	EPN1	epsin 1	1.176335733	1.618163206	28.886486	33.766503	44.414598	6.16303279	6.3943197	6.84970016	2774	4360	5521
51030	NM_001316919,NM_001316920	TVP23B	trans-golgi network vesicle protei	1.094788492	1.618059669	16.427381	18.927032	27.45089	5.21997612	5.34728212	5.89942163	1424	2083	2834
729252	NR_073414	KRT16P1	keratin 16 pseudogene 1	-1.403021204	1.617618223	1.493448	1.064692	2.278111	1.86749926	1.52971203	2.40240201	104	99	207
3667	NM_005544	IRS1	insulin receptor substrate 1	2.334722388	1.61747818	1.997521	4.674206	3.04057	4.17110273	5.34795755	4.83403711	668	2084	1329
84750	NM_032664	FUT10	fucosyltransferase 10	-1.464710042	1.617452061	0.747801	0.509295	1.143288	1.84721644	1.47139626	2.37961802	102	93	203
100507057	NR_038285	MELTF-AS1	MELTF antisense RNA 1	-1.01851227	1.617111349	7.670813	7.536628	11.653811	3.01906833	2.99588686	3.64312491	279	366	555
1456	NM_001031812,NM_001044723	CSNK1G3	casein kinase 1 gamma 3	-1.024454779	1.616891965	8.779264	8.425257	13.10767	5.21602693	5.18211693	5.89440754	1420	1852	2824

2548	NM_000152,NM_001079803,NM	GAA	glucosidase alpha, acid	1.144960206	1.616762243	41.722192	48.036478	63.70268	7.20491622	7.39897847	7.89429925	5753	8801	11440
57630	NM_020870	SH3RF1	SH3 domain containing ring finge	-1.082770099	1.616630845	6.43195	5.946874	9.773694	5.08682982	4.9756027	5.76358331	1295	1598	2575
101927043	NR_110207,NR_110208	LOC101927043	uncharacterized LOC101927043	2.331861258	1.616232328	0.192232	0.459851	0.289217	0.23670415	0.50527715	0.36729312	7	22	14
103344718	NR_001293171	HOTS	H19 opposite tumor suppressor	-1.545802484	1.616232328	0.047113	0.029908	0.06859	0.23670415	0.15624221	0.36729312	7	6	14
6916	NM_001061,NM_001130966,NM	TBXAS1	thromboxane A synthase 1	2.015933494	1.616232328	0.0761	0.151269	0.108757	0.23670415	0.44594451	0.36729312	7	19	14
283208	NM_001288748,NM_182904,NR	P4HA3	prolyl 4-hydroxylase subunit alph	1.700005729	1.616232328	0.068277	0.136418	0.121721	0.23670415	0.38406669	0.36729312	7	16	14
83861	NM_001346418,NM_031924,NR	RSPH3	radial spoke 3 homolog	-1.100618839	1.616163505	1.728473	1.562049	2.577963	3.53555296	3.40966853	4.18003621	416	505	827
8266	NM_014235	UBL4A	ubiquitin like 4A	1.245808516	1.615916018	8.839499	11.025066	13.424626	4.42654937	4.73036982	5.09317527	805	1340	1600
9652	NM_014639	TTC37	tetratricopeptide repeat domain 3	-1.25179741	1.615473715	15.115604	12.087309	22.946547	6.40858912	6.08884618	7.09408037	3296	3518	6549
10505	NM_001271661,NM_001271662	SEMA4F	ssemaphorin 4F	1.20297175	1.614773001	2.789614	3.469739	4.261927	4.13809052	4.39083965	4.79797547	652	1048	1295
1293	NM_004369,NM_057164,NM_05	COL6A3	collagen type VI alpha 3 chain	14.96901817	1.614617587	0.01481	0.224457	0.02265	0.20517559	1.73367602	0.32020933	6	122	12
105372383	NR_136525	LOC105372383	uncharacterized LOC105372383	1.733268597	1.614617587	0.064596	0.106978	0.098789	0.20517559	0.3412894	0.32020933	6	14	12
6305	NM_002972	SBF1	SET binding factor 1	1.252634788	1.614588334	10.801342	13.544137	16.387529	6.42107909	6.7426547	7.10584102	3325	5565	6603
55095	NM_001303614,NM_018028	SAMD4B	sterile alpha motif domain contair	1.205894651	1.614285386	10.446133	12.426709	15.436878	5.49067064	5.75530068	6.16933971	1726	2781	3427
56169	NM_031415	GSDMC	gasdermin C	-1.714472675	1.613872129	4.532227	2.649312	6.883385	3.70009475	2.99933041	4.34789256	471	367	935
9997	NM_001169109,NM_001169110	SCO2	SCO2, cytochrome c oxidase ass	-1.178115334	1.613861159	13.78317	11.926789	21.033323	3.87558202	3.65645563	4.52832887	537	609	1066
339451	NM_198317	KLHL17	kelch like family member 17	1.152837312	1.613203856	11.630811	13.424132	17.63044	4.90867185	5.10749649	5.58027977	1140	1756	2262
23385	NM_001290184,NM_001290186	NCSTN	nicastrin	1.008396589	1.61261969	28.361074	28.486359	42.618656	6.33993461	6.35184985	7.02257848	3141	4232	6230
56670	NM_033050	SUCNR1	succinate receptor 1	2.951960016	1.612371114	0.025029	0.091491	0.046482	0.17294258	0.46599441	0.27153692	5	20	10
93659	NM_033043	CGB5	chorionic gonadotropin beta subu	5.150214153	1.612371114	0.126777	0.779443	0.216041	0.17294258	0.73744528	0.27153692	5	35	10
100616359	NR_039883	MIR4730	microRNA 4730	-1.647024248	1.612371114	1.560952	0.812052	2.452963	0.17294258	0.10601836	0.27153692	5	4	10
619518	NR_103541	SSBP3-AS1	SSBP3 antisense RNA 1	1.779557809	1.612371114	0.049611	0.083746	0.076262	0.17294258	0.29720487	0.27153692	5	12	10
2005	NM_001973,NM_021795	ELK4	ELK4, ETS transcription factor	1.03164263	1.612043094	9.000845	8.945486	12.548345	6.46000163	6.50444356	7.14267439	3417	4710	6775
64327	NM_001350953,NM_001350954	LMBR1	limb development membrane pro	-1.362297995	1.611815184	6.159789	4.544827	9.369538	4.91233736	4.48350191	5.58277547	1143	1121	2266
4090	NM_001001419,NM_001001420	SMAD5	SMAD family member 5	1.103798136	1.611357834	7.625878	8.402167	11.5635	5.73032519	5.87025076	6.40828426	2045	3016	4053
64783	NM_001201545,NM_022768	RBM15	RNA binding motif protein 15	-1.206091263	1.611310545	8.480393	7.075005	13.049256	4.86394356	4.60375125	5.53331305	1104	1223	2188
100527978	NM_001199691	TMEM56-RWDD3	TMEM56-RWDD3 readthrough	-1.15894841	1.611159966	1.030702	0.888633	1.564933	1.32354034	1.19932051	1.77525664	59	68	117
4126	NM_005908	MANBA	mannosidase beta	1.001190724	1.611054213	6.010089	6.025805	9.098275	4.34933082	4.3509637	5.01031597	761	1018	1508
6790	NM_001323303,NM_001323304	AURKA	aurora kinase A	1.202253627	1.611049347	60.863725	69.998737	91.007346	7.11579079	7.3797859	7.79985211	5406	8684	10712
10066	NM_001320778,NM_005697	SCAMP2	secretory carrier membrane prote	1.200203058	1.610971905	25.114147	30.173898	38.020152	5.93878107	6.19814177	6.61779051	2369	3799	4694
3659	NM_002198	IRF1	interferon regulatory factor 1	1.117114317	1.610871513	16.926764	18.933865	25.628208	5.89684214	6.05408526	6.57549861	2300	3433	4557
2773	NM_006496	GNAI3	G protein subunit alpha i3	1.222121873	1.61021075	15.012633	18.368305	22.717087	6.13297837	6.4186356	6.81243872	2716	4435	5379
84958	NM_001193308,NM_032872	SYTL1	synaptotagmin like 1	1.251760338	1.609875565	17.513834	21.878889	26.410988	5.09974705	5.41524189	5.77071509	1307	2186	2588
4134	NM_001134364,NM_002375,NM	MAP4	microtubule associated protein 4	1.051991476	1.609327644	17.819659	18.648835	26.43902	6.76173135	6.83419894	7.44316123	4221	5933	8355
80124	NM_025054	VCPIP1	valosin containing protein interac	1.051293443	1.609120489	3.840478	4.042827	5.810066	4.9814326	5.05137419	5.65036315	1201	1687	2377
55770	NM_018303,NR_073064	EXOC2	exocyst complex component 2	-1.000705645	1.609115585	4.21855	4.071673	6.378079	4.27725131	4.276286	4.93515799	722	964	1429
126961	NM_021059,NM_021059_dup1	HIST2H3C	histone cluster 2 H3 family memb	2.212854945	1.609032028	0.170468	0.450929	0.308273	0.13997292	0.29720487	0.22116494	4	12	8
158131	NM_012364	OR1Q1	olfactory receptor family 1 subfan	4.035186879	1.609032028	0.086889	0.454486	0.169003	0.13997292	0.50527715	0.22116494	4	22	8
493913	NR_103711	PAPPA-AS1	PAPPA antisense RNA 1	-1.324521912	1.609032028	0.035749	0.032016	0.05988	0.13997292	0.10601836	0.22116494	4	4	8
101927586	NR_109950	LINC01271	long intergenic non-protein codin	2.030621751	1.609032028	0.04347	0.090835	0.066014	0.13997292	0.27464693	0.22116494	4	11	8
101928118	NR_125948,NR_125949	LOC101928118	uncharacterized LOC101928118	1.119455784	1.609032028	0.04221	0.041502	0.056759	0.13997292	0.15624221	0.22116494	4	6	8
349075	NM_182633	ZNF713	zinc finger protein 713	1.539761853	1.608821289	0.290994	0.449791	0.444923	1.2010231	1.58576189	1.62837236	51	105	101
284185	NR_038080	LINC00482	long intergenic non-protein codin	1.315580105	1.60880533	3.346227	4.406428	5.060922	3.41670749	3.77972069	4.05079762	380	668	752
92799	NM_138392	SHKBP1	SH3KBP1 binding protein 1	1.248783516	1.608705715	17.617916	22.029533	26.639503	5.37766084	5.69127348	6.0504089	1593	2658	3152
116832	NM_052969	RPL39L	ribosomal protein L39 like	1.944607095	1.608347815	14.447472	28.136816	21.820251	3.53555296	4.43346656	4.17342809	416	1081	823
1962	NM_001166415,NM_001966	EHHADH	enoyl-CoA hydratase and 3-hydr	1.125355706	1.608250343	4.662785	5.257378	7.041859	4.20934818	4.37103944	4.86512445	687	1033	1359
5002	NM_001315501,NM_001315502	SLC22A18	solute carrier family 22 member 1	-1.156729749	1.607569094	9.967218	8.542424	15.032309	3.96536097	3.76967342	4.615008	574	663	1135
9144	NM_001320523,NM_004710	SYNGR2	synaptogyrin 2	-1.218766406	1.60752062	135.58516	110.694133	199.99088	7.80735203	7.52333485	8.48976011	8755	9598	17310
374969	NM_199342	SVBP	small vasohibin binding protein	1.714126518	1.607391001	4.373521	7.527353	6.620595	2.14180175	2.77662633	2.69776636	134	307	265
4294	NM_002446	MAP3K10	mitogen-activated protein kinase	-1.100412624	1.607177225	5.254856	4.783033	7.940949	4.21727226	4.08697381	4.87228279	691	839	1366
55558	NM_017514	PLXNA3	plexin A3	1.261201182	1.606589732	11.960381	15.098981	18.055305	6.31522766	6.6462792	6.99239202	3087	5202	6100
79820	NM_024764	CATSPERB	cation channel sperm associated	-1.464854606	1.606522946	0.318012	0.219118	0.489925	1.10178443	0.83317479	1.50747923	45	41	89
10486	NM_006366	CAP2	CAP, adenylate cyclase-associat	1.496443882	1.606045031	1.074683	1.618781	1.626575	2.0648444	2.52753672	2.61239943	125	250	247
55290	NM_018310	BRF2	BRF2, RNA polymerase III transc	1.006788239	1.60560625	3.670867	3.697816	5.540015	3.0281061	3.03667667	3.64312491	281	378	555
9793	NM_001008938,NM_014756	CKAP5	cytoskeleton associated protein 5	-1.174584655	1.604655455	38.43881	32.809199	57.99676	7.97811052	7.74695568	8.65822263	9860	11216	19460
84951	NM_032865	TNS4	tensin 4	1.241788303	1.604564993	50.950146	63.341541	76.830627	7.66541663	7.97645529	8.34492647	7931	13159	15652
63931	NM_022100,NR_037606	MRPS14	mitochondrial ribosomal protein S	2.029416118	1.604480211	8.091968	16.771761	12.433266	4.19537543	5.17604388	4.84759061	680	1844	1342
26958	NM_001290033,NM_012133	COPG2	coatomer protein complex subun	-1.382382852	1.604431792	11.341604	8.472583	19.145064	4.26583542	3.82699692	4.91944741	716	692	1413
5289	NM_001308020,NM_002647	PIK3C3	phosphatidylinositol 3-kinase cata	1.666610493	1.603823493	2.556198	4.199112	3.771538	3.08983317	3.75752359	3.70628928	295	657	582
757	NM_006134,NR_040016	TMEM50B	transmembrane protein 50B	-1.96409354	1.603746415	10.172529	5.283258	15.556387	4.70537203	3.78372009	5.3659089	985	670	1943
677828	NR_003014	SNORA47	small nucleolar RNA, H/ACA box	1.238869866	1.603547076	0.396042	0.604728	0.828901	0.10623215	0.13134883	0.16897038	3	5	6
101929882	NR_135558	LOC101929882	uncharacterized LOC101929882	-1.002019576	1.603547076	0.109905	0.139071	0.211721	0.10623215	0.10601836	0.16897038	3	4	6
102465488	NR_106872	MIR6814	microRNA 6814	3.165952837	1.603547076	1.047191	3.543263	1.676526	0.10623215	0.31941552	0.16897038	3	13	6
105372273	NR_134909	LOC105372273	uncharacterized LOC105372273	1.238869866	1.603547076	0.108637	0.156835	0.182996	0.10623215	0.13134883	0.16897038	3	5	6
303	NR_001562	ANXA2P1	annexin A2 pseudogene 1	-1.937182024	1.603547076	0.03896	0.017004	0.083381	0.10623215	0.05398282	0.16897038	3	2	6
100500880	NR_037435	MIR3662	microRNA 3662	-1.937182024	1.603547076	0.809786	0.402451	1.147841	0.10623215	0.05398282	0.16897038	3	2	6
100616134	NR_039912	MIR499B	microRNA 499b	-1.002019576	1.603547076	0.780616	0.810863	1.504609	0.10623215	0.10601836	0.16897038	3	4	6
166863	NM_001277171,NM_001277173	RBM46	RNA binding motif protein 46	2.18448128	1.603519447	0.273085	0.601929	0.414878	0.99521185	1.66595383	1.37552141	39	114	77
55062	NM_001320772,NM_017983,NR	WIPI1	WD repeat domain, phosphoinos	-1.848661118	1.603447616	8.748124	4.759312	13.196047	4.13181574	3.31335619	4.7817748	649	469	1280
3619	NM_001040694,NM_020238	INCENP	inner centromere protein	1.326211879	1.603309326	15.793567	20.981673	23.817588	6.01519803	6.41702723	6.68785694	2500	4430	4930
84722	NM_001005290,NM_001032291	PSRC1	proline and serine rich coiled-coil	1.159906988	1.603274719	24.143767	27.651942	35.51583	5.45005808	5.6595227	6.11864377	1677	2599	3307
9578	NM_006035	CDC42BPB	CDC42 binding protein kinase be	1.200561269	1.6023095	17.884809	21.49987	26.932627	6.87907613	7.14074177	7.55462757	4582	7350	9030
664	NM_004052	BNIP3	BCL2 interacting protein 3	3.340518656	1.602206079	3.858294	12.913741	5.812888	2.83562844	4.42708835	3.43788301	241	1076	475
83443	NM_031287	SF3B5	splicing factor 3b subunit 5	1.328649897	1.602109129	30.159081	40.121212	45.408276	4.50146728	4.89562996	5.15737078	850	1509	1675
79699	NM_024646	ZYG11B	zyg-11 family member B, cell cyc	1.270804676	1.601683681	2.253865	2.868735	3.394217	4.23884171	4.56825335	4.88952086	702	1192	1383
23263	NM_001112732,NM_001320815	MCF2L	MCF.2 cell line derived transform	-1.261374274	1.601197357	0.265394	0.210664	0.410034	1.44997615	1.24644988	1.91649468	68	72	134
57584	NM_020824	ARHGAP21	Rho GTPase activating protein 2	-1.103806672	1.601187525	12.279223	11.134851	18.475063	6.43770726	6.29694166	7.11060505	3364	4072	6625
51227	NM_001320480,NM_016430,NM	PIGP	phosphatidylinositol glycan ancho	1.737235849	1.601040597	11.577109	20.136022	17.374639	3.45742019	4.19753076	4.08640889	392	910	772
9053	NM_001198608,NM_001198609	MAP7	microtubule associated protein 7	1.209721824	1.600909898	6.309194	7.610962	9.541917	4.85888706	5.12494108	5.51904993	1100	1778	2166
3069	NM_001243900,NM_001320965	HDLBP	high density lipoprotein binding p	1.52290586	1.600501775	38.759501	59.002649	57.907371	7.90026394	8.50502135	8.57652714	9340	19005	18386
10175	NM_005776	CNIH1	cornichon family AMPA receptor	1.368132567	1.600116408	50.650459	69.397179	76.179138	6.15377238	6.60053269	6.82435142	2756	5038	5424
54961	NM_017857	SSH3	slingshot protein phosphatase 3	-1.124540887	1.599620598	34.343437	30.580269	51.636028	6.60370522	6.43621034	7.27587959	3779	4490	7435
6786	NM_001277961,NM_001277962	STIM1	stromal interaction molecule 1	-1.051335654	1.599581085	32.173672	30.540857	48.370294	6.99509932	6.92345485	7.66856017	4969	6315	9776
2730	NM_001308253,NM_002061	GCLM	glutamate-cysteine ligase modifie	1.149366045	1.599353809	5.36628	6.175398	8.082688	4.77547559	4.96947085	5.43314535	1036	1591	2038
84280	NM_001297741,NM_001297742	BTBD10	BTB domain containing 10	2.417966477	1.599175917	7.420901	18.093877	11.224563	4.22907737	5.45716415	4.87737422	697	2252	1371
84725	NM_001197026,NM_001197027	PLEKHA8	pleckstrin homology domain cont	-1.262414508	1.599047303	3.441606	2.647809	5.194657	4.53988763	4.21984192	5.19374412	874	925	1719
9863	NM_001301128,NM_012301	MAGI2	membrane associated guanylate	1.569486325	1.598344712	0.228365	0.363581	0.3459	1.35261029	1.78245981	1.80120704	61	128	120
55783	NM_001099642,NM_001324374	CMTR2	cap methyltransferase 2	-1.343117728	1.5981765	18.193015	13.854577	26.121398	6.11453445	5.69605597	6.78316916	2681	2667	5270
6612	NM_001286416,NM_006936	SUMO3	small ubiquitin-like modifier 3	-1.309899746	1.59805947	26.038942	19.852404	39.105465	5.54991538	5.16994515	6.21469853	1800	1836	3538
254887	NM_001320466,NM_001320467	ZDHHC23	zinc finger DHHC-type containing	1.473134194	1.597953818	1.445092	2.381408	2.444296	3.30588543	3.81727465	3.92662428	349	687	686
80256	NM_001317991,NM_025182,NR	FAM214B	family with sequence similarity 2	-1.075582055	1.597852596	5.022236	4.839835	7.987519	3.97474042	3.87652288	4.61622671	578	718	1136
84224	NM_001256416,NM_001256417	NBPF3	NBPF member 3	-1.258458345	1.597774382	3.149719	2.50756	4.841773	3.82979934	3.5240591	4.46750731	519	551	1020
25934	NM_001329570,NM_015469	NIPSNAP3A	nipsnap homolog 3A	1.201875453	1.597754527	8.768281	10.468142	12.923941	3.97005831	4.21984192	4.61134568	576	925	1132
2074	NM_000124,NM_001277058,NM	ERCC6	ERCC excision repair 6, chromat	-1.219465794	1.597225204	3.542664	2.713138	4.757106	4.49659199	4.22426305	5.14813232	847	928	1664
90204	NM_080603	ZSWIM1	zinc finger SWIM-type containing	1.174803232	1.597111957	3.967031	4.666515	5.959689	3.55756677	3.77168848	4.18661421	423	664	831
7005	NM_003214	TEAD3	TEA domain transcription factor 3	1.002772348	1.596722698	12.120278	12.165565	18.186888	5.18201511	5.18589965	5.84224309	1386	1857	2722
8940	NM_001282112,NM_001282113	TOP3B	topoisomerase (DNA) III beta	1.125828935	1.596559577	7.330284	8.294076	10.824632	4.63760441	4.80211744	5.29081305	938	1411	1842
54441	NR_040583,NR_040583_dup1	STAG3L1	stromal antigen 3-like 1 (pseudog	-1.091354742	1.596333727	4.024166	3.686222	6.037367	3.23349937	3.12128662	3.84996236	330	404	648
10908	NM_001166111,NM_001166112	PNPLA6	patatin like phospholipase domai	-1.056224428	1.59620591	9.72644	9.21787	14.541854	5.41345159	5.33643196	6.07543546	1634	2067	3208

28952	NM_014008	CCDC22	coiled-coil domain containing 22	1.282936451	1.595594404	3.625848	4.664421	5.446785	3.20189991	3.52644784	3.81642381	322	552	632
7803	NM_003463	PTP4A1	protein tyrosine phosphatase type	1.333385722	1.595202459	9.881286	13.192325	14.819113	5.64330615	6.05118491	6.30626619	1923	3426	3773
4085	NM_002358	MAD2L1	mitotic arrest deficient 2 like 1	1.286978216	1.595200638	39.823639	51.299507	59.689857	5.82476383	6.18308005	6.48900046	2186	3759	4289
282809	NM_001199777,NM_172240,NR	POC1B	POC1 centriolar protein B	-1.072205477	1.595200174	4.732064	4.385683	7.088908	3.9817352	3.88771448	4.62109129	581	724	1140
50855	NM_001037281,NM_016948	PARD6A	par-6 family cell polarity regulator	-1.50214045	1.595165051	1.668639	1.114394	2.503005	1.61502061	1.24644988	2.10191219	81	72	159
25865	NM_001079880,NM_001079881	PRKD2	protein kinase D2	-1.0831522	1.595148762	15.520061	15.0206	22.888338	5.73447233	5.62148167	6.3980468	2051	2530	4024
55362	NM_001318792,NM_018426	TMEM63B	transmembrane protein 63B	1.11123152	1.59490072	10.18304	11.270857	15.269463	5.06942494	5.2172889	5.72682174	1279	1899	2509
114882	NM_001003712,NM_001319652	OSBPL8	oxysterol binding protein like 8	1.269387032	1.594851435	4.650364	6.015849	7.070778	5.09545415	5.43062766	5.7530961	1303	2210	2556
57613	NM_020853	FAM234B	family with sequence similarity 23	-1.041446804	1.594434583	4.003358	3.847316	5.996425	4.27535493	4.21984192	4.92043435	721	925	1414
54708	NM_017824	MARCH5	membrane associated ring-CH-ty	-1.472611572	1.593653376	8.689394	5.909686	13.018463	5.0976022	4.55894904	5.75420361	1305	1184	2558
54664	NM_001134232,NM_018374	TMEM106B	transmembrane protein 106B	-1.158904752	1.593432406	9.02179	7.827386	13.732538	5.84472325	5.63593399	6.5075083	2217	2556	4345
9878	NM_001303523,NM_014828	TOX4	TOX high mobility group box fam	1.369554171	1.593412356	13.519314	18.547649	20.250326	5.92130285	6.36858875	6.58455493	2340	4282	4586
8508	NM_001202502,NM_003634	NIPSNAP1	nipsnap homolog 1	1.125657476	1.593334583	21.790384	26.715907	35.400682	5.59850439	5.7659537	6.25945481	1863	2802	3651
2064	NM_001005862,NM_001289936	ERBB2	erb-b2 receptor tyrosine kinase 2	-1.149128669	1.593170188	37.187395	32.445453	55.715657	7.40050792	7.20123694	8.06923471	6594	7667	12921
6913	NM_001330677,NM_152380	TBX15	T-box 15	1.8268438	1.592868313	0.014714	0.022049	0.022373	0.07168335	0.13134883	0.1148164	2	5	4
162998	NM_175883	OR7D2	olfactory receptor family 7 subfan	1.471633007	1.592868313	0.016229	0.02432	0.024799	0.07168335	0.10601836	0.1148164	2	4	4
168448	NR_003595	CDC14C	cell division cycle 14C, pseudoge	1.116422214	1.592868313	0.009043	0.013551	0.027226	0.07168335	0.08023518	0.1148164	2	3	4
121952	NR_026965	METTL21EP	methyltransferase like 21E, pseu	4.313319351	1.592868313	0.015797	0.096575	0.034582	0.07168335	0.29720487	0.1148164	2	12	4
100302161	NR_031610	MIR1205	microRNA 1205	2.182054593	1.592868313	0.657835	1.626537	0.992659	0.07168335	0.15624221	0.1148164	2	6	4
9887	NM_001174061,NM_001331007	SMG7	SMG7, nonsense mediated mRN	1.428194747	1.592754094	13.678625	19.602088	20.215954	6.30921596	6.81795863	6.97397223	3074	5866	6022
113878	NM_001102594,NM_001102595	DTX2	deltex E3 ubiquitin ligase 2	-1.773667387	1.592709238	63.03576	35.089108	93.588251	7.1289776	6.31017284	7.7966307	5456	4110	10688
51495	NM_016395	HACD3	3-hydroxyacyl-CoA dehydratase	1.189367466	1.592636459	41.618332	49.559048	62.293346	7.03347295	7.2819178	7.70079795	5104	8111	9998
22837	NM_001278458,NM_001278460	COBLL1	cordon-bleu WH2 repeat protein	-1.892107277	1.592376788	1.865105	0.984945	2.819101	4.18934543	3.33804373	4.83089121	677	478	1326
5791	NM_001316676,NM_001316677	PTPRE	protein tyrosine phosphatase, rec	1.55135444	1.5921259	3.612246	5.170724	5.223612	4.17923909	4.78426276	4.82035506	672	1393	1316
51496	NM_016396	CTDSPL2	CTD small phosphatase like 2	1.12151488	1.591853066	5.358429	6.018754	8.017917	4.72495781	4.88449983	5.37529684	999	1497	1956
2805	NM_002079	GOT1	glutamic-oxaloacetic transaminas	-1.40357087	1.591815868	49.88966	35.58556	74.636093	6.70509716	6.22154965	7.37063535	4057	3862	7943
79812	NM_024756	MMRN2	multimerin 2	1.173041354	1.591758547	1.342655	1.577031	2.011799	2.69516719	2.89227137	3.28075123	215	337	421
108903150	NR_144447	ZNF8-ERVK3-1	ZNF8-ERVK3-1 readthrough	-1.340639607	1.591755816	1.594058	1.190467	2.386204	3.05045588	2.68551066	3.65503258	286	285	560
9397	NM_001308295,NM_004808	NMT2	N-myristoyltransferase 2	1.329928602	1.591654841	3.46448	5.70543	6.491276	4.00709644	4.39607413	4.64397663	592	1052	1159
8910	NM_001099400,NM_001099401	SGCE	sarcoglycan epsilon	-1.293298492	1.591082233	23.289639	18.113778	34.565424	5.30330016	4.94289226	5.95971039	1511	1561	2957
84243	NM_032283	ZDHHC18	zinc finger DHHC-type containing	1.059490806	1.591058705	14.517802	15.398919	21.706917	5.50368806	5.58527743	6.16184796	1742	2466	3409
79901	NM_001127383,NM_001256909	CYBRD1	cytochrome b reductase 1	-1.747829557	1.591045616	9.540089	5.448257	14.252085	5.36523338	4.58553885	6.02218491	1579	1207	3090
754	NM_001286822,NM_004339,NR	PTTG1IP	PTTG1 interacting protein	-1.632625618	1.590984271	100.111834	61.453181	149.710815	8.11084638	7.40694114	8.77883164	10814	8850	21161
55187	NM_015378,NM_018156	VPS13D	vacuolar protein sorting 13 homo	-1.058372813	1.590867511	2.994291	2.832193	4.476824	5.60455895	5.52443609	6.26334948	1871	2362	3661
646278	NR_037599	PDCD6IPP2	PDCD6IP pseudogene 2	-1.232703846	1.590844683	0.588656	0.482163	0.888628	1.15225689	0.99371033	1.5590879	48	52	94
8837	NM_001127183,NM_001127184	CFLAR	CASP8 and FADD like apoptosis	1.35384647	1.590823317	38.14362	29.733807	51.844658	7.00486037	7.4389936	7.67046796	5003	9050	9789
8225	NM_012227_chrX,NM_012227_c	GTPBP6	GTP binding protein 6 (putative)	1.360874692	1.590764646	7.62352	10.390503	11.408834	3.29843463	3.70365887	3.91280502	347	631	679
221037	NM_001282948,NM_001318153	JMJD1C	jumonji domain containing 1C	-1.232683893	1.590651544	8.425193	6.79881	12.561823	6.16457043	5.86742698	6.8267222	2777	3010	5433
587	NM_001164773,NM_001190,NM	BCAT2	branched chain amino acid trans	1.010993115	1.590533911	27.72267	26.793061	40.504663	5.38559376	5.40099266	6.04227156	1602	2164	3134
89890	NM_152903	KBTBD6	kelch repeat and BTB domain co	1.176007785	1.589999869	0.907546	1.069735	1.360253	2.49460548	2.68977954	3.06566078	182	286	356
79960	NM_001287437,NM_001287439	JADE1	jade family PHD finger 1	1.671851424	1.58994796	3.07741	5.010603	4.670884	4.14225859	4.85058478	4.78068826	654	1461	1279
285362	NM_001164674,NM_001164675	SUMF1	sulfatase modifying factor 1	1.721713499	1.589896228	11.463244	19.783459	17.167096	4.64790143	5.40748703	5.29538508	945	2174	1848
85403	NM_033083	EAF1	ELL associated factor 1	1.213965985	1.589737178	4.437319	5.395799	6.634566	4.35113248	4.61838139	4.99353253	762	1236	1490
148206	NM_182515,NR_117086,NR_11	ZNF714	zinc finger protein 714	1.26783974	1.589626069	3.174549	4.42893	5.09947	4.63760441	4.9677141	5.28469437	938	1589	1834
4901	NM_006177	NRL	neural retina leucine zipper	-1.391779045	1.589471097	0.30941	0.227562	0.471901	0.68823103	0.52452456	0.98063768	24	23	47
83449	NM_001160213,NM_031293	PMFBP1	polyamine modulated factor 1 bin	1.049097805	1.589215093	0.847984	0.873295	1.251798	1.94589385	1.99745948	2.46867149	112	157	219
219854	NM_001080546,NM_001258238	TMEM218	transmembrane protein 218	-1.057495451	1.589062558	2.321487	2.185462	3.484907	3.32067256	3.24827687	3.93446197	353	446	690
6879	NM_005642	TAF7	TATA-box binding protein associ	-1.025904909	1.588945055	45.906242	44.80386	68.559502	6.69484709	6.6583098	7.35776019	4028	5246	7872
84892	NM_032806	POMGNT2	protein O-linked mannose N-acet	1.953464008	1.588793296	5.144678	10.061588	7.682624	3.78784692	4.70212092	4.41665393	503	1313	983
7693	NM_003435	ZNF134	zinc finger protein 134	1.338957384	1.588734733	6.575606	8.815916	9.817889	5.02835327	5.43825945	5.67980348	1242	2222	2427
94103	NM_001320801,NM_001320802	ORMDL3	ORMDL sphingolipid biosynthesi	1.60041904	1.5887045	20.656452	33.097004	30.844177	5.57171277	6.2387686	6.22831165	1828	3909	3572
1738	NM_000108,NM_001289750,NM	DLD	dihydrolipoamide dehydrogenase	-1.195969668	1.588703718	12.644104	10.549716	19.080029	5.50287791	5.25090169	6.15892396	1741	1945	3402
3833	NM_002263	KIFC1	kinesin family member C1	1.023875424	1.588674126	25.652475	26.293201	38.302139	6.09907168	6.13262236	6.75909764	2652	3628	5182
284023	NR_024349	LOC284023	uncharacterized LOC284023	1.462099883	1.588145893	0.67728	0.996126	1.016677	1.42282133	1.79043244	1.87636985	66	129	129
10644	NM_001007225,NM_001291869	IGF2BP2	insulin like growth factor 2 mRNA	1.57745322	1.588110242	11.247424	17.367994	16.366364	5.44500562	6.09046153	6.10003528	1671	3522	3264
55248	NM_001198862,NM_018252	TMEM206	transmembrane protein 206	1.134563674	1.58762729	11.279076	12.826388	16.844452	4.49659199	4.67115044	5.13968208	847	1284	1654
23189	NM_001256876,NM_001256877	KANK1	KN motif and ankyrin repeat dom	-1.421109313	1.587421617	1.462726	1.025498	2.304757	3.18179291	2.74004451	3.78858771	317	298	619
26986	NM_002568	PABPC1	poly(A) binding protein cytoplasm	-1.210504783	1.587358936	415.614349	343.717896	619.959229	10.1777998	9.90245247	10.84396856	45436	50151	88707
160760	NM_139283,NR_138550	PPTC7	PTC7 protein phosphatase homo	-1.111446989	1.587091631	5.245621	4.724556	7.828438	4.33301385	4.18850877	4.97275069	752	904	1468
124944	NM_001142798,NM_001142799	C17orf49	chromosome 17 open reading fra	1.058345093	1.586558019	13.324858	14.09	19.888811	3.65121051	3.72669368	4.27414582	454	642	886
285753	NM_001083535,NM_001271852	CEP57L1	centrosomal protein 57 like 1	-1.358999019	1.586427566	2.129659	1.689554	3.411054	3.70573844	3.30224686	4.33017765	473	465	923
51599	NM_001260489,NM_001260490	LSR	lipolysis stimulated lipoprotein re	1.085820129	1.5855908	119.948694	130.642495	177.867895	7.98044044	8.09877524	8.6433557	9876	14328	19260
9099	NM_001243759,NM_004205,NM	USP2	ubiquitin specific peptidase 2	-3.371463349	1.585141259	0.56391	0.167353	0.841872	1.61502061	0.68708806	2.09493548	81	32	158
2803	NM_001172713,NM_002078	GOLGA4	golgin A4	-1.117042809	1.584924153	10.95297	9.815058	16.314273	6.40988621	6.25218083	7.06804256	3299	3946	6431
3417	NM_001282386,NM_001282387	IDH1	isocitrate dehydrogenase (NADP	1.075341348	1.584877107	22.232515	23.935105	33.089838	5.72199495	5.8248786	6.37627276	2033	2921	3963
116238	NM_001160407,NM_138463	TLCD1	TLC domain containing 1	-1.150235416	1.584812584	35.918234	31.049448	53.664784	5.17592889	4.97996669	5.82548131	1380	1603	2690
1266	NM_001286055,NM_001286056	CNN3	calponin 3	-1.173327884	1.584749147	27.102182	23.090882	40.323257	5.8234666	5.59725738	6.47831697	2184	2487	4257
79102	NM_032015	RNF26	ring finger protein 26	-1.411920447	1.58466609	20.071716	14.226385	29.886818	5.80780809	5.3206905	6.46248074	2160	2044	4210
57531	NM_001321080,NM_001321083	HACE1	HECT domain and ankyrin repea	-1.276148651	1.584558068	1.52696	1.124657	2.14217	2.91084846	2.61093762	3.50257812	256	268	499
55001	NM_001114108,NM_017904	TTC22	tetratricopeptide repeat domain 2	1.656077205	1.584497555	2.487634	4.191705	3.820898	2.58923814	3.21903935	3.16227031	197	436	384
55450	NM_018584	CAMK2N1	calcium/calmodulin dependent pr	1.930962846	1.58444558	29.114916	56.294472	43.353432	6.08018627	7.01921759	6.73630001	2617	6752	5100
51720	NM_001199297,NM_001199298	UIMC1	ubiquitin interaction motif contain	1.113653893	1.584204542	15.214223	16.734202	22.09095	5.25215086	5.40359392	5.90192215	1457	2168	2839
22920	NM_001204514,NM_001204516	KIFAP3	kinesin associated protein 3	1.383061946	1.584065259	9.126114	12.630342	13.457518	4.78883075	5.24220835	5.43314535	1046	1933	2038
8310	NM_001101667,NM_003501	ACOX3	acyl-CoA oxidase 3, pristanoyl	1.16687552	1.583997088	4.26103	4.962395	6.314804	3.68302999	3.88957134	4.30470323	465	725	906
327	NM_001640	APEH	acylaminoacyl-peptide hydrolase	1.278951335	1.583813573	19.706892	25.231234	29.334297	5.75298837	6.10211907	6.40652436	2078	3551	4048
5727	NM_000264,NM_001083602,NM	PTCH1	patched 1	-1.176555231	1.58371569	0.502071	0.426754	0.747108	2.30685184	2.12272838	2.85881867	155	176	302
729830	NM_001109977,NM_001348694	FAM160A1	family with sequence similarity 16	-1.403745289	1.583087212	1.98376	1.41463	2.966357	4.53514067	4.07069878	5.17484522	871	829	1696
90427	NM_001003940,NM_001003942	BMF	Bcl2 modifying factor	1.256406455	1.582870067	1.368629	1.725994	2.042301	2.86114003	3.14945559	3.44886901	246	413	479
7576	NM_006969,NR_036599,NR_03	ZNF28	zinc finger protein 28	-2.366046834	1.582738004	1.460723	0.617887	2.232038	2.94941774	1.94127715	3.54158719	264	149	514
101927974	NR_144536,NR_144537,NR_14	LOC101927974	uncharacterized LOC101927974	-1.67780182	1.582731745	1.292621	0.712222	1.929664	0.99521185	0.669904	1.36396013	39	31	76
100288069	NR_033908	LOC100288069	uncharacterized LOC100288069	1.352583486	1.582274061	3.550153	4.830175	5.301024	2.52684435	2.89597101	3.09392991	187	338	364
64064	NM_022120	OXCT2	3-oxoacid CoA-transferase 2	-3.299211779	1.582185793	0.284158	0.085866	0.418484	0.59399636	0.20477631	0.85412573	20	8	39
9121	NM_001271765,NM_004695	SLC16A5	solute carrier family 16 member 5	-1.583374389	1.581899786	59.440611	38.173854	89.518013	6.85578736	6.20001348	7.5128695	4508	3804	8771
6301	NM_001330669,NM_006513,NR	SARS	seryl-tRNA synthetase	1.236246881	1.581284418	48.579005	60.688474	75.619492	6.65602068	6.95925859	7.31186417	3920	6475	7624
5831	NM_001282279,NM_001282280	PYCR1	pyrroline-5-carboxylate reductase	1.06380143	1.581226187	19.744268	21.949922	30.540972	5.26367512	5.35065612	5.91088832	1469	2088	2857
8879	NM_003901	SGPL1	sphingosine-1-phosphate lyase 1	-1.396500977	1.580815787	11.319809	8.116989	16.821325	6.01349256	5.54047969	6.66595674	2497	2389	4855
11116	NM_001278690,NM_007045,NM	FGFR1OP	FGFR1 oncogene partner	1.323972102	1.580798958	5.882572	7.763688	8.664919	4.4636621	4.85249002	5.10016049	827	1463	1608
26256	NM_001308231,NM_012189,NM	CABYR	calcium binding tyrosine phospho	1.031167086	1.580751455	5.259375	5.536722	7.96229	3.34619087	3.38618649	3.95387191	360	496	700
4627	NM_002473	MYH9	myosin heavy chain 9	1.2766963	1.579979611	56.719975	72.493141	84.215149	8.70793129	9.05960079	9.36657384	16378	27938	31827
728609	NR_003263	SDHAP3	succinate dehydrogenase comple	-1.008388575	1.579961318	3.6307	3.598651	5.391273	2.34351507	2.33383942	2.89541791	160	212	311
29127	NM_001126103,NM_001126104	RACGAP1	Rac GTPase activating protein 1	-1.066631771	1.579781279	31.623381	29.598757	46.747044	6.63509978	6.54300176	7.28950308	3863	4839	7506
3910	NM_001105206,NM_001105207	LAMA4	laminin subunit alpha 4	1.377951298	1.579361711	17.180411	20.007217	22.432126	6.51447631	6.97267818	7.16803407	3550	6536	6896
23171	NM_015141	GPD1L	glycerol-3-phosphate dehydroger	-1.212455095	1.578887017	2.334774	1.925418	3.466723	3.35699061	3.10858833	3.96347983	363	400	705
5753	NM_001256358,NM_005975	PTK6	protein tyrosine kinase 6	-1.353067935	1.578813934	27.789188	20.526115	41.291254	6.08831031	5.6595227	6.73937923	2632	2599	5111
5787	NM_001109754,NM_001206971	PTPRB	protein tyrosine phosphatase, rec	4.066021817	1.578609774	0.80198	3.404384	1.189854	3.40980893	5.32755573	4.01797815	378	2054	734
55793	NM_001040217,NM_001163258	MINDY1	MINDY lysine 48 deubiquitinase	1.292876953	1.578216669	2.126581	4.097951	5.061344	3.06808971	3.39927923	3.66213029	290	501	563
283991	NM_182565	UBALD2	UBA like domain containing 2	-1.278655558	1.578092737	61.916809	48.47266	91.816376	6.596888	6.24639624	7.24961546	3761	3930	7300
376693	NR_026667	RPS10P7	ribosomal protein S10 pseudoger	1.204594684	1.57733644	0.651353	0.822609	0.989231	0.54446555	0.63490936	0.78645063	18	29	35
220972	NM_001002266,NM_001282866	MARCH8	membrane associated ring-CH-ty	-1.168749785	1.577319436	2.167547	1.860908	3.433853	3.6915876	3.48529134	4.30772367	468	535	908

9538	NM_001290135,NM_001330419	EI24	EI24, autophagy associated trans	-1.110264912	1.577095133	30.815249	27.756846	45.649309	6.11877104	5.97014822	6.76844567	2689	3236	5216
55734	NM_001319146,NM_018197,NM	ZFP64	ZFP64 zinc finger protein	1.296632372	1.576790206	3.651567	4.655133	5.303934	3.57924973	3.92621521	4.19152809	430	745	834
100506581	NM_001195124,NM_001195125	C16orf95	chromosome 16 open reading fra	1.038739659	1.576481236	2.962166	3.058436	4.537974	1.98354948	2.02475093	2.5006986	116	161	225
222950	NM_173564	NYAP1	neuronal tyrosine phosphorylatec	-8.860835627	1.576374757	0.243687	0.027354	0.364158	0.89996265	0.13134883	1.24294651	34	5	66
2821	NM_000175,NM_001184722,NM	GPI	glucose-6-phosphate isomerase	1.458313043	1.575744329	63.368607	92.351736	93.722504	8.0230201	8.56558118	8.67703144	10173	19822	19716
89958	NM_178448	SAPCD2	suppressor APC domain containi	1.096456855	1.575454792	15.892209	17.447752	23.533749	5.91461739	6.04536668	6.56164849	2329	3412	4513
1175	NM_001301076,NM_001301078	AP2S1	adaptor related protein complex 2	1.235309912	1.575251075	51.110345	63.018078	75.286332	5.58095462	5.88009069	6.22551943	1840	3037	3565
25894	NM_001129727,NM_001129728	PLEKHG4	pleckstrin homology and RhoGEI	-2.648063626	1.575180169	0.729346	0.273101	1.095373	2.09955541	1.16293478	2.62698266	129	65	250
115	NM_001116	ADCY9	adenylate cyclase 9	-1.301466502	1.575067364	1.784124	1.371882	2.64017	3.85032677	3.4999516	4.46885716	527	541	1021
55175	NM_018143	KLHL11	kelch like family member 11	1.083974232	1.574448127	4.338217	4.706794	6.419701	5.01364054	5.12651654	5.65214662	1229	1780	2380
4967	NM_001003941,NM_001165036	OGDH	oxoglutarate dehydrogenase	-1.129750976	1.574206301	23.025101	19.569744	34.319131	6.51125714	6.33729741	7.16011778	3542	4189	6858
7675	NM_001008727,NM_001308269	ZNF121	zinc finger protein 121	1.278799474	1.57399846	9.001352	11.605641	13.393617	3.93924856	4.27344453	4.55907234	563	962	1090
10492	NM_001159673,NM_001159674	SYNCRIP	synaptotagmin binding cytoplasm	1.095669195	1.573367869	18.82439	24.544309	29.399531	6.77889944	6.90956726	7.42797354	4272	6254	8267
6575	NM_001257180,NM_001257181	SLC20A2	solute carrier family 20 member 2	-1.306856001	1.573354227	11.415606	8.74368	16.875826	5.50449776	5.12809029	6.14674672	1743	1782	3373
5083	NM_006194	PAX9	paired box 9	-1.225709069	1.573159346	0.653883	0.536113	0.968878	1.57857476	1.38978355	2.04513029	78	85	151
22809	NM_001193646,NM_001290746	ATF5	activating transcription factor 5	1.698820872	1.572990037	6.702618	11.542604	9.793232	3.87558202	4.59921966	4.49294082	537	1219	1039
147686	NM_001317027,NM_001317028	ZNF418	zinc finger protein 418	1.654619756	1.572838527	0.310049	0.51445	0.461055	1.13562816	1.57657016	1.52834475	47	104	91
55254	NM_018266,NR_073506	TMEM39A	transmembrane protein 39A	1.137712477	1.572779553	7.797745	8.886525	11.534207	5.25984392	5.44142749	5.89942163	1465	2227	2834
64764	NM_001253775,NM_001318246	CREB3L2	cAMP responsive element bindin	-1.904268234	1.572343572	4.770415	2.661544	7.912355	5.11042377	4.2183652	5.74810174	1317	924	2547
4043	NM_002337,NR_110005	LRPAP1	LDL receptor related protein asso	1.165806898	1.572297287	12.42057	14.497278	18.352997	7.00486037	7.22459506	7.65365157	5003	7793	9675
147912	NM_175875	SIX5	SIX homeobox 5	-1.076684572	1.572223916	3.680437	3.420587	5.441249	3.69442889	3.59633059	4.30621424	469	582	907
147798	NM_001145303,NM_144686	TMC4	transmembrane channel like 4	-1.074191146	1.572211831	40.056613	37.318209	59.145144	6.60143639	6.49928452	7.24882993	3773	4693	7296
84859	NM_032773	LRCH3	leucine rich repeats and calponin	1.239547432	1.572179049	6.16313	7.651462	9.117424	3.89300127	4.18397654	4.5100879	544	901	1052
9762	NM_001282533	LZTS3	leucine zipper tumor suppressor	-1.574581606	1.571937192	7.283182	4.630235	10.758651	4.91111656	4.28336529	5.54615829	1142	969	2208
85315	NM_133367	PAQR8	progestin and adipoQ receptor fa	-1.097130082	1.571613595	1.241263	1.13342	1.835057	2.75082071	2.63769986	3.32316088	225	274	435
55101	NM_001167867,NM_001167868	ATP5SL	ATP5S like	-1.022954836	1.571282474	22.031407	21.620804	32.196916	5.13049438	5.09869445	5.76742788	1336	1745	2582
27173	NM_001271957,NM_001271958	SLC39A1	solute carrier family 39 member 1	-1.075866052	1.57113821	51.222292	47.863488	76.576535	6.78524352	6.68073434	7.43230451	4291	5329	8292
284439	NM_001321544,NM_178526	SLC25A42	solute carrier family 25 member 4	1.240869451	1.570817729	2.473311	3.080569	3.670475	3.15316384	3.43277448	3.74468587	310	514	599
3930	NM_002296,NM_194442	LBR	lamin B receptor	1.143102768	1.570354154	30.541451	34.998665	45.077789	6.84175964	7.03314362	7.48828722	4464	6818	8622
51727	NM_001136140,NM_016308,NR	CMPK1	cytidine/uridine monophosphate l	-1.153098559	1.569450799	38.293239	33.341643	56.571523	6.78090585	6.57739174	7.4264112	4278	4957	8258
4585	NM_001322468,NM_004532,NM	MUC4	mucin 4, cell surface associated	-1.905174565	1.569370083	15.16279	8.290171	21.973115	7.81716158	6.89300145	8.46503112	8815	6182	17015
11156	NM_007079,NM_032611	PTP4A3	protein tyrosine phosphatase typ	1.121156758	1.569349889	2.431545	2.736248	3.604268	2.92543304	3.06980877	3.5052118	259	388	500
10787	NM_013436,NM_205842	NCKAP1	NCK associated protein 1	1.289412712	1.568441883	36.540179	47.176876	53.864867	7.48231431	7.84722131	8.12872769	6981	12027	13467
283820	NM_001004060,NM_173614	NOMO2	NODAL modulator 2	1.271110405	1.568419738	22.704007	28.943136	33.392644	6.56580548	6.90865191	7.20960321	3680	6250	7099
983	NM_001170406,NM_001170407	CDK1	cyclin dependent kinase 1	-1.472900677	1.568248016	90.139139	61.648388	133.659757	7.38783779	6.83323448	8.03387577	6536	5929	12607
5480	NM_000943	PPIC	peptidylprolyl isomerase C	1.047198228	1.568124429	36.523117	38.28923	53.822094	5.49067064	5.55576227	6.12807238	1726	2415	3329
4660	NM_001167857,NM_001167858	PPP1R12B	protein phosphatase 1 regulatory	1.439540736	1.568023841	4.587717	7.119317	7.137258	5.65063969	6.16747684	6.28917599	1933	3718	3728
79944	NM_024884	L2HGDH	L-2-hydroxyglutarate dehydrogen	1.071067739	1.567900583	4.137995	4.441212	6.101537	4.67692288	4.77223569	5.30524173	965	1381	1861
50809	NM_016287	HP1BP3	heterochromatin protein 1 binding	1.085454609	1.567418372	49.733868	54.049271	73.260284	7.57657587	7.69428177	8.22223542	7455	10812	14372
4059	NM_001013257,NM_005581	BCAM	basal cell adhesion molecule (Lu	-1.449286889	1.56713722	60.749038	42.031283	87.648773	7.48457309	6.95283475	8.12979475	6992	6446	13477
554282	NM_001287385,NM_001287385	FAM72C	family with sequence similarity 72	-1.459410793	1.566721948	19.543103	13.165888	29.773419	5.34999754	4.82072735	5.98493142	1562	1430	3010
59269	NM_001127714,NM_024503,NR	HIVEP3	human immunodeficiency virus ty	-1.362220073	1.566212055	3.109312	2.455535	3.572361	2.92543304	2.54649137	3.50257812	259	254	499
9185	NM_001080975,NM_004726	REPS2	RALBP1 associated Eps domain	1.064389063	1.565406271	0.442269	0.47278	0.649737	2.15010456	2.2203867	2.67455554	135	192	260
56650	NM_001040181,NM_001040182	CLDND1	claudin domain containing 1	1.963565118	1.564613337	51.190712	100.379888	75.146023	6.77889944	7.74593108	7.41997042	4272	11208	8221
5152	NM_001001567,NM_001001568	PDE9A	phosphodiesterase 9A	1.791261038	1.564523437	8.643086	15.591843	12.385255	4.12971807	4.9339228	4.74547971	648	1551	1247
1832	NM_001008844,NM_001319034	DSP	desmoplakin	-1.074200565	1.564509564	12.399144	11.738105	17.574039	6.75868036	6.65640254	7.39958928	4212	5239	8105
11236	NM_007218	RNF139	ring finger protein 139	1.225319493	1.56425692	19.108465	23.443634	28.096991	5.96901418	6.25794232	6.60618362	2420	3962	4656
1785	NM_001005360,NM_001005361	DNM2	dynamin 2	1.114876264	1.564230663	30.601352	34.161073	44.980253	6.78324314	6.93878015	7.42397753	4285	6383	8244
23521	NM_001270491,NM_012423,NR	RPL13A	ribosomal protein L13a	-1.910305959	1.564148755	550.522691	288.64439	807.652976	9.30103568	8.36930675	9.94559063	24726	17294	47568
9208	NM_001137550,NM_001137551	LRRFIP1	LRR binding FLII interacting prote	1.377400096	1.563854953	28.694404	38.512128	41.44745	6.8346943	7.29318415	7.47524966	4442	8175	8544
51253	NM_001330602,NM_016491	MRPL37	mitochondrial ribosomal protein L	1.376799033	1.563559076	31.759747	43.900356	46.82743	5.55617688	6.00910191	6.1899483	1808	3326	3477
80217	NM_025145	CFAP43	cilia and flagella associated prote	1.605348765	1.563535869	0.130862	0.21053	0.18993	0.7550755	1.07428646	1.05442063	27	58	52
338761	NM_001008223	C1QL4	complement C1q like 4	1.068667182	1.563514166	0.172849	0.189846	0.260294	0.43999062	0.46599441	0.64076093	14	20	27
100421577	NR_038275	FAM21EP	family with sequence similarity 21	1.174775307	1.563514166	0.158703	0.193471	0.244902	0.43999062	0.50527715	0.64076093	14	22	27
4641	NM_001080779,NM_001080950	MYO1C	myosin IC	-1.052611738	1.563348457	28.947272	27.23952	42.056594	7.09792936	7.02450843	7.73877965	5339	6777	10266
388730	NM_203376	TMEM81	transmembrane protein 81	-1.031834337	1.563154713	3.054599	2.971889	4.500673	2.3142594	2.278228	2.85055786	156	202	300
170688	NR_104005	NUDT4P2	nudix hydrolase 4 pseudogene 2	1.448836667	1.562985058	0.006748	0.007336	0.009771	0.03628685	0.05398282	0.0585501	1	2	2
100616122	NR_039749	MIR4523	microRNA 4523	-1.294074149	1.562985058	0.247761	0.137134	0.338444	0.03628685	0.02724389	0.0585501	1	1	2
646862	NM_001195135	RNF225	ring finger protein 225	3.477086927	1.562985058	0.008111	0.085472	0.04038	0.03628685	0.13134883	0.0585501	1	5	2
106635683	NR_132980_chr5,NR_132980_c	SNORD141A	small nucleolar RNA, C/D box 14	1.448836667	1.562985058	0.129774	0.315224	0.316373	0.03628685	0.05398282	0.0585501	1	2	2
102466983	NR_106824	MIR6766	microRNA 6766	4.153170347	1.562985058	0.194267	1.609209	0.552481	0.03628685	0.15624221	0.0585501	1	6	2
692229	NR_004381	SNORD105	small nucleolar RNA, C/D box 10	2.124920087	1.562985058	0.274259	0.621041	0.265424	0.03628685	0.08023518	0.0585501	1	3	2
100129396	NM_001348162	FAM106CP	family with sequence similarity 1(	2.801003507	1.562985058	0.014799	0.037145	0.018939	0.03628685	0.10601836	0.0585501	1	4	2
100302274	NR_031589	MIR1178	microRNA 1178	-1.294074149	1.562985058	0.034525	0.021655	0.254352	0.03628685	0.02724389	0.0585501	1	1	2
100422846	NR_036122	MIR3164	microRNA 3164	2.124920087	1.562985058	0.280868	0.696801	0.252744	0.03628685	0.08023518	0.0585501	1	3	2
26810	NR_002751	SNORD41	small nucleolar RNA, C/D box 41	-1.294074149	1.562985058	0.351531	0.193935	0.314982	0.03628685	0.02724389	0.0585501	1	1	2
693215	NR_030359	MIR630	microRNA 630	-1.294074149	1.562985058	0.234989	0.030012	0.212185	0.03628685	0.02724389	0.0585501	1	1	2
728419	NM_001256867,NM_001256867	USP17L30	ubiquitin specific peptidase 17-lik	-1.294074149	1.562985058	0.008927	0.002461	0.018821	0.03628685	0.02724389	0.0585501	1	1	2
100302133	NR_031619	MIR1287	microRNA 1287	1.448836667	1.562985058	0.286186	0.23565	0.265909	0.03628685	0.05398282	0.0585501	1	2	2
100616480	NR_039697	MIR4479	microRNA 4479	2.124920087	1.562985058	0.259023	0.653296	0.359571	0.03628685	0.08023518	0.0585501	1	3	2
102465528	NR_106937	MIR6877	microRNA 6877	2.801003507	1.562985058	0.21855	1.16142	0.470021	0.03628685	0.10601836	0.0585501	1	4	2
406902	NR_029608	MIR10A	microRNA 10a	1.448836667	1.562985058	0.098899	0.282291	0.26627	0.03628685	0.05398282	0.0585501	1	2	2
407007	NR_029636	MIR222	microRNA 222	1.448836667	1.562985058	0.04945	0.224069	0.250078	0.03628685	0.05398282	0.0585501	1	2	2
442903	NR_029895	MIR331	microRNA 331	-1.294074149	1.562985058	0.090933	0.078456	0.305277	0.03628685	0.02724389	0.0585501	1	1	2
100422875	NR_036160	MIR3192	microRNA 3192	2.124920087	1.562985058	0.259023	0.564583	0.43693	0.03628685	0.08023518	0.0585501	1	3	2
100616115	NR_039679	MIR4469	microRNA 4469	-1.294074149	1.562985058	0.22077	0.014668	0.32376	0.03628685	0.02724389	0.0585501	1	1	2
100616417	NR_039949	MIR4786	microRNA 4786	2.801003507	1.562985058	0.20398	0.778728	0.460126	0.03628685	0.10601836	0.0585501	1	4	2
102465428	NR_106774	MIR6717	microRNA 6717	-1.294074149	1.562985058	0.215858	0.241827	0.384526	0.03628685	0.02724389	0.0585501	1	1	2
102465513	NR_106911	MIR6852	microRNA 6852	-1.294074149	1.562985058	0.023329	0.150051	0.326869	0.03628685	0.02724389	0.0585501	1	1	2
102466204	NR_106940	MIR6880	microRNA 6880	-1.294074149	1.562985058	0.317511	0.194075	0.395807	0.03628685	0.02724389	0.0585501	1	1	2
171586	NM_001308256,NM_001308257	ABHD3	abhydrolase domain containing 3	1.209411918	1.562858651	20.711306	25.022256	30.933167	5.30979981	5.57781111	5.94086581	1518	2453	2918
26091	NM_001278185,NM_001278186	HERC4	HECT and RLD domain containir	-1.13734759	1.562777271	23.112755	19.817934	32.290841	6.50196181	6.31846728	7.14034671	3519	4134	6764
7094	NM_006289	TLN1	talin 1	1.185037159	1.562715641	8.276076	9.820278	12.157132	6.10815615	6.34982807	6.74468245	2669	4226	5130
9403	NM_004261,NM_203341,NR_14	SELENOF	selenoprotein F	1.207199658	1.562498899	16.131023	19.204309	23.352176	4.80994515	5.07277886	5.4352184	1062	1713	2041
1183	NM_001256944,NM_001830	CLCN4	chloride voltage-gated channel 4	1.176965463	1.56234014	0.395784	0.467205	0.580279	1.8573935	2.03149389	2.35062252	103	162	198
8434	NM_001316345,NM_001316346	RECK	reversion inducing cysteine rich p	1.662400033	1.562164239	1.187467	1.655089	1.909034	1.7191395	2.26684378	2.19620105	90	200	173
55105	NM_001297754,NM_018040	GPATCH2	G-patch domain containing 2	1.803463371	1.562020737	3.584543	6.575872	5.627041	4.04765842	4.85913858	4.65942844	610	1470	1172
64793	NM_001281517,NM_001281518	CEP85	centrosomal protein 85	1.065987199	1.561938897	11.482286	12.253459	16.925994	5.49963272	5.58985296	6.13148574	1737	2474	3337
858	NM_001206747,NM_001206748	CAV2	caveolin 2	-1.573732438	1.561764932	58.860012	37.251861	85.851698	7.46577956	6.81625129	8.10602674	6901	5859	13256
54951	NM_001329668,NM_017845	COMMD8	COMM domain containing 8	1.487189538	1.561643785	6.886131	10.264303	10.121358	3.45742019	3.98645018	4.05259921	392	779	753
414919	NM_001001795	C8orf82	chromosome 8 open reading fran	-1.057265191	1.561634119	6.157605	5.827625	9.034415	3.65413237	3.58032478	4.25549579	455	575	874
54928	NM_017813	IMPAD1	inositol monophosphatase domai	1.313053213	1.561350745	11.268482	14.813913	16.53791	6.32809147	6.716741	6.96443417	3115	5465	5982
22888	NM_001267584,NM_014948,NM	UBOX5	U-box domain containing 5	1.285571398	1.560636307	3.239985	4.160662	4.778927	3.87056622	4.21095882	4.4769298	535	919	1027
3295	NM_000414,NM_001199291,NM	HSD17B4	hydroxysteroid 17-beta dehydrog	1.046110829	1.560451344	55.739392	58.464233	79.111861	7.20042592	7.26503037	7.83887203	5735	8016	11007
154810	NM_001301007,NM_130847	AMOTL1	angiomotin like 1	-1.288011025	1.560291951	2.334276	1.845359	3.44164	4.42483953	4.07885924	5.04240188	804	834	1543
9782	NM_001194954,NM_001194955	MATR3	matrin 3	1.388544693	1.559974131	48.486319	68.178367	71.169114	7.93100523	8.40292822	8.5704098	9542	17703	18308
5095	NM_000282,NM_001127692,NM	PCCA	propionyl-CoA carboxylase alpha	1.338399539	1.559933111	3.567491	4.839285	5.195359	3.29094516	3.67379966	3.87868233	345	617	662
90231	NM_138346	KIAA2013	KIAA2013	1.377760343	1.559810568	21.484188	29.641911	31.494122	5.9085127	6.36425528	6.5412625	2319	4269	4449
51527	NM_001271904,NM_001271905	GSKIP	GSK3B interacting protein	1.076318324	1.559790237	7.077731	7.256727	10.360149	4.05651939	4.15647998	4.66650461	614	883	1178

79098	NM_001083924,NM_023938	C1orf116	chromosome 1 open reading fran	-1.223382279	1.559134129	37.289818	30.432337	54.460213	7.6339421	7.34468396	8.27208752	7759	8474	14879
140735	NM_080677	DYNLL2	dynein light chain LC8-type 2	1.390825812	1.558802715	41.131653	57.269531	60.244797	5.95308737	6.42248839	6.58517742	2393	4447	4588
55204	NM_018178	GOLPH3L	golgi phosphoprotein 3 like	1.089034255	1.558762939	8.173627	8.909876	11.970673	4.71379852	4.83235848	5.33441396	991	1442	1900
353500	NM_181809	BMP8A	bone morphogenetic protein 8a	-8.172047514	1.558740896	0.057067	0.005192	0.087326	0.41264541	0.05398282	0.60191771	13	2	25
64122	NM_022158	FN3K	fructosamine 3 kinase	-2.14561122	1.558368954	4.383229	2.050523	6.44036	2.83562844	1.94842076	3.40158951	241	150	462
727956	NR_003265	SDHAP2	succinate dehydrogenase comple	1.173853078	1.558305158	16.760038	19.698402	24.544867	5.27035534	5.49607553	5.89691676	1476	2315	2829
8505	NM_001303486,NM_001303487	PARG	poly(ADP-ribose) glycohydrolase	-1.170751407	1.558202702	2.605181	2.222818	4.619134	3.58538547	3.37827344	4.18168353	432	493	828
11118	NM_001197246,NM_001197247	BTN3A2	butyrophilin subfamily 3 member	-1.025947199	1.558169273	5.204263	4.758048	7.410628	4.29420754	4.25915261	4.90755118	731	952	1401
92305	NM_001127266,NM_138385	TMEM129	transmembrane protein 129	-1.139396662	1.557366461	25.578941	22.646878	37.399528	6.15428841	5.96883147	6.78615005	2757	3233	5281
2783	NM_005273	GNB2	G protein subunit beta 2	-1.104052325	1.55725324	70.003403	63.469669	102.448936	6.8382313	6.69673052	7.47272887	4453	5389	8529
25853	NM_015397	DCAF12	DDB1 and CUL4 associated facto	1.208021422	1.5572251	5.551941	6.718122	8.13386	4.38318339	4.64390751	4.99727909	780	1259	1494
84222	NR_026815	TMEM191A	transmembrane protein 191A (ps	1.968473379	1.556977065	1.268409	2.499708	1.849572	1.77389308	2.52753672	2.2537152	95	250	182
84919	NM_032833	PPP1R15B	protein phosphatase 1 regulatory	1.276033766	1.556573901	23.657404	30.224215	34.611057	6.93720463	7.28632919	7.57137519	4772	8136	9136
54892	NM_001281932,NM_001281933	NCAPG2	non-SMC condensin II complex s	-1.182221629	1.55650889	14.938727	12.84765	22.26709	5.99575025	5.75835243	6.62598114	2466	2787	4721
100505783	NR_038337,NR_038338,NR_038	OSER1-AS1	OSER1 antisense RNA 1 (head t	-1.264207074	1.556480621	1.722825	1.362096	2.509155	1.88750088	1.64851465	2.37961802	106	112	203
54868	NM_001321264,NM_017728	TMEM104	transmembrane protein 104	1.051655719	1.556285842	7.522047	7.887743	10.331084	4.79281331	4.86292405	5.41224936	1049	1474	2008
5586	NM_001320707,NM_001320708	PKN2	protein kinase N2	-1.185981536	1.556279022	18.369669	15.453343	27.091548	6.79520403	6.55152982	7.42866737	4321	4868	8271
23060	NM_015042	ZNF609	zinc finger protein 609	1.206414687	1.55600087	5.950815	7.190318	8.704923	5.67097747	5.93686357	6.29869553	1961	3161	3753
9218	NM_003574,NM_194434	VAPA	VAMP associated protein A	1.153394017	1.555802144	30.952838	35.647331	45.352614	7.70328922	7.90825626	8.3384798	8143	12549	15582
5573	NM_001276289,NM_001276290	PRKAR1A	protein kinase cAMP-dependent	1.00796288	1.555694614	63.877422	61.709998	100.107156	7.9700726	7.98146978	8.60558069	9805	13205	18761
285367	NM_001142547,NM_001351736	RPUSD3	RNA pseudouridylate synthase d	1.084880242	1.555629705	6.945788	7.562812	10.156264	3.29843463	3.40448323	3.88273891	347	503	664
102238594	NR_108100	SPACA6P-AS	SPACA6P antisense RNA	1.034989251	1.555580737	0.304902	0.31992	0.45295	1.13562816	1.16293478	1.51794971	47	65	90
7572	NM_001308123,NM_006965	ZNF24	zinc finger protein 24	1.071517102	1.555254396	11.585047	12.594866	17.607201	5.92251507	6.02058584	6.55117294	2342	3353	4480
4213	NR_002211	MEIS3P1	Meis homeobox 3 pseudogene 1	1.408818984	1.555131135	2.102472	2.97127	3.074366	2.77786255	3.21015128	3.33801005	230	433	440
56892	NM_020130	C8orf4	chromosome 8 open reading fran	-2.148081618	1.554938545	0.81393	0.382142	1.204729	1.30878272	0.75384785	1.72191206	58	36	111
57547	NM_020787	ZNF624	zinc finger protein 624	1.190809431	1.554860807	0.708663	0.840689	1.035637	1.97422731	2.16626946	2.47405896	115	183	220
55607	NM_001166160,NM_001166161	PPP1R9A	protein phosphatase 1 regulatory	-1.646537171	1.554761773	2.533541	1.517939	3.6154	4.6997269	4.01565009	5.31653173	981	796	1876
5333	NM_001130964,NM_006225,NR	PLCD1	phospholipase C delta 1	-1.358372336	1.554454034	3.123983	2.331651	4.766833	3.36057263	2.96803853	3.94613924	364	358	696
91452	NM_001042473,NM_001271512	ACBD5	acyl-CoA binding domain contain	-1.324574009	1.553996419	7.366508	5.675083	10.946449	4.92206677	4.53185994	5.54103391	1151	1161	2200
7465	NM_001143976,NM_003390	WEE1	WEE1 G2 checkpoint kinase	-1.159775398	1.553704142	18.49029	15.801533	26.819008	5.92614562	5.71607575	6.55340139	2348	2705	4487
128869	NM_080476	PIGU	phosphatidylinositol glycan ancho	1.237309469	1.553556629	28.655216	35.49382	41.836689	5.7037794	6.00568164	6.32948591	2007	3318	3835
55845	NM_018462	BRK1	BRICK1, SCAR/WAVE actin nucl	1.332068462	1.55323342	23.838663	31.786617	34.797882	4.82298643	5.22392723	5.44004397	1072	1908	2048
8412	NM_001261408,NM_001261409	BCAR3	breast cancer anti-estrogen resis	1.214344619	1.553046374	25.105773	30.381656	36.662672	6.28774036	6.56466401	6.91626923	3028	4913	5784
51646	NM_001127399,NM_001127400	YPEL5	yippee like 5	1.142319701	1.552904108	22.422704	25.402003	32.603171	5.68318697	5.87166058	6.30815266	1978	3019	3778
9415	NM_001281501,NM_001281502	FADS2	fatty acid desaturase 2	-1.190694084	1.552885998	1.035472	0.891513	1.520195	2.04717061	1.86027027	2.55254608	123	138	235
334	NM_001142276,NM_001142277	APLP2	amyloid beta precursor like prote	-1.437311494	1.552652897	247.52874	173.287324	364.236616	9.80863184	9.28596051	10.4427961	35169	32693	67161
54768	NM_001198542,NM_001198543	HYDIN	HYDIN, axonemal central pair ap	2.896559199	1.552402078	0.096206	0.302728	0.14153	1.27880673	2.36086138	1.6852207	56	217	107
4318	NM_004994	MMP9	matrix metallopeptidase 9	1.909442895	1.552402078	0.618949	1.195774	0.914542	1.27880673	1.89765633	1.6852207	56	143	107
8450	NM_001079872,NM_001330624	CUL4B	cullin 4B	1.039377621	1.552192751	13.148906	13.611325	19.291121	6.05719254	6.11209361	6.68379406	2575	3576	4916
64065	NM_022121	PERP	PERP, TP53 apoptosis effector	-1.087023461	1.552099095	44.634724	41.103752	65.100258	7.55426599	7.4345489	8.18576158	7340	9022	14012
79887	NM_024829	PLBD1	phospholipase B domain containi	-2.303451696	1.551964654	26.146597	11.362655	38.133163	5.64917596	4.48227194	6.27304039	1931	1120	3686
79583	NM_001077416,NM_001077418	TMEM231	transmembrane protein 231	1.053335925	1.55180126	3.029411	3.286211	4.561852	3.31329794	3.38091595	3.89484064	351	494	670
64175	NM_001146289,NM_001243246	P3H1	prolyl 3-hydroxylase 1	1.357296249	1.551318423	7.035275	9.652189	10.383322	4.33118941	4.75298538	4.93905904	751	1362	1433
79694	NM_024641	MANEA	mannosidase endo-alpha	1.242297467	1.551054007	1.415559	1.760512	2.064912	2.88122939	3.15564136	3.44338646	250	415	477
23608	NM_001145125,NM_001291663	MKRN1	makorin ring finger protein 1	-1.377884795	1.550976738	31.221301	22.899693	49.400348	6.50601062	6.04952495	7.13355384	3529	3422	6732
8487	NM_001009182,NM_001009183	GEMIN2	gem nuclear organelle associate	1.229021338	1.550958185	10.447261	12.678167	15.185818	3.86048198	4.13941485	4.45802288	531	872	1013
8660	NM_003749	IRS2	insulin receptor substrate 2	2.169148553	1.550871091	2.061299	4.478905	3.007609	3.91265481	4.97734988	4.5113985	552	1600	1053
11247	NM_007224	NXPH4	neurexophilin 4	1.037511071	1.550784702	0.626737	0.660007	0.928523	1.08456011	1.11294744	1.45395569	44	61	84
65243	NM_023070	ZFP69B	ZFP69 zinc finger protein B	1.902180209	1.550318491	0.415178	0.803167	0.618948	0.88013425	1.37924953	1.20457043	33	84	63
65982	NM_001145542,NM_001145543	ZSCAN18	zinc finger and SCAN domain co	-1.570955097	1.549923922	8.643879	5.518985	12.79244	4.54304361	3.92621521	5.15317881	876	745	1670
54904	NM_017778,NM_023034	NSD3	nuclear receptor binding SET dor	-1.013884599	1.549749527	7.506371	7.414696	10.291154	5.0573361	5.03804247	5.67396328	1268	1671	2417
1398	NM_005206,NM_016823	CRK	CRK proto-oncogene, adaptor pr	1.248547897	1.549747615	8.877753	10.964256	12.802072	4.8007456	5.11068396	5.41435263	1055	1760	2011
57082	NM_144508,NM_170589	KNL1	kinetochore scaffold 1	-1.181151967	1.549739177	3.406809	2.884318	4.963559	4.71379852	4.48350191	5.32624544	991	1121	1889
200058	NR_026900	FLJ23867	uncharacterized protein FLJ2386	1.692409605	1.549676882	34.537273	58.522446	50.298462	6.52887435	7.2815643	7.15530516	3586	8109	6835
26140	NM_001025930,NR_037162	TTLL3	tubulin tyrosine ligase like 3	-1.022363278	1.549590521	3.225544	3.116947	4.652966	3.72253824	3.69306567	4.31524723	479	626	913
84627	NM_001127464	ZNF469	zinc finger protein 469	2.99174577	1.549539952	0.21041	0.634772	0.308525	1.89739865	3.19521507	2.38534784	107	428	204
63971	NM_001105566,NM_001105567	KIF13A	kinesin family member 13A	-1.258085234	1.549529482	1.901539	1.487721	2.691074	3.6915876	3.38881456	4.28338119	468	497	892
145249	NR_104113	LINC00618	long intergenic non-protein codin	1.088202878	1.549388432	1.524717	1.711197	2.292612	0.64188297	0.68708806	0.90287695	22	32	42
84342	NM_032382	COG8	component of oligomeric golgi co	1.333188573	1.549310371	9.108783	12.154842	13.262075	4.53196733	4.93122105	5.1413761	869	1548	1656
51060	NM_015913,NR_046405,NR_04	TXNDC12	thioredoxin domain containing 12	1.161135447	1.54920032	32.907529	38.201459	47.914586	6.28114034	6.49410697	6.90609151	3014	4676	5743
10073	NM_001042581,NM_001042588	SNUPN	snurportin 1	1.134602426	1.549121245	10.430004	11.921718	15.181885	4.27155466	4.44487678	4.87635737	719	1090	1370
656	NM_001720	BMP8B	bone morphogenetic protein 8b	-1.248212407	1.549037697	0.228662	0.18397	0.33802	1.06712766	0.90836632	1.43197761	43	46	82
145864	NM_001307952,NM_178232	HAPLN3	hyaluronan and proteoglycan link	1.63453334	1.549037697	0.518289	0.867212	0.768887	1.06712766	1.48128072	1.43197761	43	94	82
2770	NM_001256414,NM_002069	GNAI1	G protein subunit alpha i1	1.226580442	1.548762536	1.325715	1.630573	1.93479	2.40724866	2.65089709	2.93892502	169	277	322
51804	NM_017420	SIX4	SIX homeobox 4	1.24756188	1.548680605	5.43161	6.781925	7.903198	5.09545415	5.4061905	5.7115087	1303	2172	2482
11043	NM_012216,NM_052817	MID2	midline 2	-1.084133485	1.548588155	1.014885	0.938679	1.478287	3.04155747	2.93964205	3.60925088	284	350	541
10868	NM_001008563,NM_001110303	USP20	ubiquitin specific peptidase 20	1.068634629	1.548515771	6.835175	7.328324	9.992688	4.93773843	5.03048889	5.55190154	1164	1662	2217
57719	NM_020959	ANO8	anoctamin 8	-1.404613298	1.548510694	5.545091	3.953258	8.074152	4.5509035	4.08535454	5.15988012	881	838	1678
56889	NM_020123	TM9SF3	transmembrane 9 superfamily me	-1.313859511	1.548333181	47.530083	36.215145	69.160904	8.15560596	7.76337766	8.7845342	11156	11345	21245
10114	NM_001048200,NM_001278162	HIPK3	homeodomain interacting protein	-1.259139154	1.547717823	6.309866	4.947589	9.064872	5.56241133	5.23784196	6.18174016	1816	1927	3457
7019	NM_001270782,NM_003201,NR	TFAM	transcription factor A, mitochondr	1.566453129	1.547571338	5.709384	8.915833	8.268775	4.92085417	5.55107715	5.53395803	1150	2407	2189
375513	NR_132999,NR_133000	GUSBP4	glucuronidase, beta pseudogene	-1.002090108	1.547353465	0.708219	0.708013	1.030545	2.15010456	2.14776967	2.66044767	135	180	257
567	NM_004048	B2M	beta-2-microglobulin	-1.196607768	1.546890019	1153.287354	964.860352	1676.470459	10.11539924	9.85670389	10.7443113	43511	48584	82783
84056	NM_001014380,NM_032116	KATNAL1	katanin catalytic subunit A1 like 1	-7.957587764	1.546828461	1.279094	0.159957	1.8675	3.38187998	1.12560759	3.96156336	370	62	704
100462981	NM_001190470	MTRNR2L2	MT-RNR2-like 2	3.378831987	1.546617023	0.262939	0.937875	0.39135	0.35634914	0.96581954	0.52094563	11	50	21
322	NM_001164,NM_001257319,NM	APBB1	amyloid beta precursor protein bi	1.835509817	1.546342294	2.736994	5.325848	4.143728	3.00540462	3.79763131	3.56953804	276	677	525
57409	NM_001242498,NM_001242500	MIF4GD	MIF4G domain containing	-2.318591856	1.546088668	10.163207	4.707409	14.948334	3.82979934	2.74415527	4.42223881	519	299	987
384	NM_001172	ARG2	arginase 2	1.440690805	1.546030382	2.825701	4.078525	4.102802	2.68948191	3.14635273	3.23705705	214	412	407
728192	NR_034119	LINC00460	long intergenic non-protein codin	7.664222357	1.545754813	0.593278	4.62211	0.853494	0.61813834	2.35549733	0.87055986	21	216	40
23637	NM_012197	RABGAP1	RAB GTPase activating protein 1	1.19684401	1.545610781	15.807544	18.941643	22.963095	6.28444412	6.54064029	6.90609151	3021	4831	5743
55217	NM_001184797,NM_018196	TMLHE	trimethyllysine hydroxylase, epsil	1.564544499	1.545314505	3.087953	4.144817	3.639403	3.32067256	3.91349545	3.89684777	353	738	671
4649	NM_006901	MYO9A	myosin IXA	-1.065727618	1.545183173	1.422008	1.335679	2.06559	3.70573844	3.62112921	4.2940816	473	593	899
6397	NM_001039573,NM_001143998	SEC14L1	SEC14 like lipid binding 1	-1.077981028	1.545141754	10.446434	9.183977	13.621864	5.47588426	5.37007127	6.09217385	1708	2117	3246
55527	NM_018708	FEM1A	fem-1 homolog A	1.391138734	1.545102397	5.87996	8.185298	8.53678	4.52400331	4.98257876	5.12947602	864	1606	1642
113146	NM_001350929,NM_138420	AHNAK2	AHNAK nucleoprotein 2	-1.039771725	1.544992698	9.105909	8.767858	13.163907	7.34100929	7.28509536	7.965476	6326	8129	12021
7415	NM_007126	VCP	valosin containing protein	1.093723615	1.543985159	66.384415	72.690903	96.325615	7.9683129	8.09706873	8.59294653	9793	14311	18597
23616	NM_001350055,NM_018957	SH3BP1	SH3 domain binding protein 1	1.490333765	1.543800883	21.191795	30.947533	30.291422	5.8234666	6.39071865	6.44097707	2184	4349	4147
55346	NM_001145541,NM_018393	TCP11L1	t-complex 11 like 1	1.014725448	1.5436549	2.554796	2.507093	3.638978	2.96361946	2.98202989	3.52351414	267	362	507
84270	NM_001318010,NM_001318011	CARD19	caspase recruitment domain fam	1.057448477	1.543623846	41.102336	39.437921	49.478245	5.53810131	5.61700551	6.15347791	1785	2522	3389
118987	NM_173791	PDZD8	PDZ domain containing 8	1.004715783	1.543472504	2.659695	2.67626	3.861166	4.15469105	4.16109929	4.7521471	660	886	1253
55323	NM_001286679,NM_018357,NM	LARP6	La ribonucleoprotein domain fam	1.475394209	1.543236085	13.355197	17.464227	14.339143	3.9320442	4.46244854	4.52443946	560	1104	1063
4853	NM_001200001,NM_024408	NOTCH2	notch 2	1.393511987	1.542674605	9.023349	12.387046	12.796308	6.63399023	7.10861905	7.25431972	3860	7187	7324
55296	NM_001292054,NM_018317	TBC1D19	TBC1 domain family member 19	1.526841936	1.542516795	0.866331	1.251746	1.19864	1.80577475	2.26684378	2.27855908	98	200	186
6617	NM_003082	SNAPC1	small nuclear RNA activating con	1.837379462	1.542443281	12.163612	22.375086	17.628277	5.02044968	5.87775393	5.62999497	1235	3032	2343
55312	NM_018339	RFK	riboflavin kinase	-1.460406893	1.542438992	6.579288	4.507863	9.539365	4.19737984	3.68667221	4.79474986	681	623	1292
80014	NM_024949	WWC2	WW and C2 domain containing 2	1.032400002	1.542377054	3.864943	3.99624	5.603034	5.09437892	5.13905869	5.70465037	1302	1796	2470
201299	NM_001034836,NM_001163120	RDM1	RAD52 motif containing 1	-1.092607483	1.542136172	9.233173	8.571144	12.8117	3.34257292	3.22787299	3.9167669	359	439	681
167227	NM_001242377,NM_152624,NR	DCP2	decapping mRNA 2	-1.139112616	1.542087765	9.129664	8.002573	13.043762	6.49586721	6.31017284	7.1151379	3504	4110	6646
29899	NM_001321038,NM_001321039	GPSM2	G protein signaling modulator 2	1.013746857	1.5418392	13.839214	14.066778	20.185866	5.44416182	5.46341106	6.05715513	1670	2262	3167

55970	NM_018841	GNG12	G protein subunit gamma 12	1.155174326	1.541607333	10.849808	12.549098	15.719714	5.57248519	5.77652864	6.18626042	1829	2823	3468
65992	NM_023935	DDRGK1	DDRGK domain containing 1	1.478844468	1.541497491	58.571133	86.707054	84.845337	6.25106082	6.80940163	6.86874324	2951	5831	5595
79982	NM_001031723,NM_001278310	DNAJB14	DnaJ heat shock protein family (H	1.092678467	1.540925714	9.122206	9.747428	12.31104	5.66157057	5.78702662	6.27535655	1948	2844	3692
1612	NM_001288729,NM_001288730	DAPK1	death associated protein kinase 1	2.081593711	1.540734562	0.49154	1.027581	0.725089	1.97422731	2.8278811	2.46326383	115	320	218
2203	NM_000507,NM_001127628	FBP1	fructose-bisphosphatase 1	-1.203008566	1.540544865	15.014998	12.71373	21.849823	4.60021751	4.34556265	5.20269596	913	1014	1730
55830	NM_001010983,NM_001278280	GLT8D1	glycosyltransferase 8 domain cor	1.05719768	1.540323572	13.98789	14.649128	20.398882	4.97443634	5.05220335	5.58152816	1195	1688	2264
10594	NM_006445	PRPF8	pre-mRNA processing factor 8	1.06849203	1.540189532	23.587772	25.231289	34.140892	7.39702381	7.49205268	8.01713511	6578	9391	12461
29943	NM_013358	PADI1	peptidyl arginine deiminase 1	1.023513068	1.53991	176.476593	180.826126	255.374954	9.37068898	9.40416858	9.99277291	25951	35489	49151
8626	NM_001114978,NM_001114979	TP63	tumor protein p63	14.50592047	1.539905874	0.144433	2.303776	0.214757	0.79797761	3.55480842	1.09694451	29	564	55
100996541	NR_126161	FAM157C	family with sequence similarity 15	1.495042107	1.539905874	0.782861	1.163647	1.125241	0.79797761	1.07428646	1.09694451	29	58	55
399726	NM_001010911	CASC10	cancer susceptibility 10	1.057917735	1.539905874	0.371715	0.40347	0.550187	0.79797761	0.83317479	1.09694451	29	41	55
221981	NM_015204	THSD7A	thrombospondin type 1 domain c	-3.409067286	1.539875773	0.396375	0.115309	0.575594	2.35073722	1.1381576	2.87112187	161	63	305
81570	NM_001258392,NM_001258393	CLPB	ClpB homolog, mitochondrial AA/	1.594249441	1.539594159	5.532252	8.79696	7.949113	4.17110273	4.81389896	4.76539014	668	1423	1265
65018	NM_032409	PINK1	PTEN induced putative kinase 1	1.006886102	1.539488845	13.663034	13.777457	19.771978	5.18605837	5.19568855	5.79459514	1390	1870	2632
57020	NM_001300743,NM_020314	C16orf62	chromosome 16 open reading fra	1.791542761	1.539118594	4.395492	7.927586	6.361091	4.14433812	4.94913788	4.73766195	655	1568	1240
9197	NM_001190992,NM_004733	SLC33A1	solute carrier family 33 member 1	1.297088299	1.539028448	10.471339	14.007359	15.03514	5.21602693	5.58241034	5.8244272	1420	2461	2688
23382	NM_001130720,NM_001130722	AHCYL2	adenosylhomocysteinase like 2	-1.64229159	1.538983362	2.068402	1.260821	2.99612	3.49049124	2.85474367	4.06693155	402	327	761
11045	NM_001281443,NM_007000	UPK1A	uroplakin 1A	1.565658946	1.538756668	0.191237	0.297975	0.269658	0.3273552	0.48576948	0.47868902	10	21	19
9472	NM_004274	AKAP6	A-kinase anchoring protein 6	1.326757516	1.538571314	0.187603	0.250895	0.272579	1.54118428	1.82188956	1.97845985	75	133	142
8613	NM_003713	PLPP3	phospholipid phosphatase 3	1.224512352	1.538250521	0.955283	1.167677	1.382167	2.02927762	2.255369	2.52166171	121	198	229
5329	NM_001005376,NM_001005377	PLAUR	plasminogen activator, urokinase	-1.379312248	1.537898691	66.715	48.260778	95.953974	6.63509978	6.17663089	7.25098912	3863	3742	7307
636	NM_001003398,NM_001714	BICD1	BICD cargo adaptor 1	1.369735372	1.537828075	2.147044	2.866815	2.962582	3.00997359	3.41483526	3.56701931	277	507	524
4690	NM_001190796,NM_001291999	NCK1	NCK adaptor protein 1	1.639389041	1.537508974	5.021233	8.21283	7.276158	4.49333262	5.1813592	5.09142367	845	1851	1598
286480	NR_110506	UBE2E4P	ubiquitin conjugating enzyme E2	2.943400565	1.537346847	0.427197	1.253607	0.606005	0.56944351	1.28081328	0.80366934	19	75	36
100147773	NR_125374	LOC100147773	uncharacterized LOC100147773	1.964133983	1.537346847	0.227185	0.449304	0.334554	0.56944351	0.96581954	0.80366934	19	50	36
196383	NM_145058,NR_130703	RILPL2	Rab interacting lysosomal proteir	-1.02214278	1.537098927	4.328923	4.190729	6.239999	3.08117507	3.05333783	3.64073154	293	383	554
2137	NM_001440,NR_073468,NR_07	EXTL3	exostosin like glycosyltransferase	-1.023639403	1.537030753	3.915939	3.831153	5.662072	4.65521171	4.62285328	5.25527094	950	1240	1796
6235	NM_001030001,NM_001032,NM	RPS29	ribosomal protein S29	1.257950689	1.536904863	431.564107	542.886051	593.281227	7.23620668	7.56532392	7.85289744	5880	9883	11115
200312	NM_001017981	RNF215	ring finger protein 215	1.455972846	1.536896043	29.683578	43.272503	42.873703	5.82021839	6.35420502	6.43131602	2179	4239	4119
55007	NM_001104595,NM_001349913	FAM118A	family with sequence similarity 11	1.124036664	1.536702743	7.031946	8.009846	9.749624	4.41969781	4.5809496	5.01586734	801	1203	1514
7341	NM_001005781,NM_001005782	SUMO1	small ubiquitin-like modifier 1	3.742895837	1.536595809	31.805427	107.618415	44.64032	5.43824136	7.3178716	6.04634597	1663	8317	3143
10095	NM_005720	ARPC1B	actin related protein 2/3 complex	-1.290365679	1.536451606	74.698479	57.952816	107.856834	6.81198066	6.44791403	7.42710578	4372	4527	8262
5896	NM_000448	RAG1	recombination activating 1	-1.395569725	1.536419351	0.182694	0.129895	0.264222	1.11880553	0.8787583	1.4863075	46	44	87
7568	NM_001203250,NM_021143	ZNF20	zinc finger protein 20	1.005768557	1.536316334	3.255635	3.280079	4.702819	3.42014445	3.42767171	3.99192552	381	512	720
3109	NM_002118	HLA-DMB	major histocompatibility complex,	1.577392843	1.536312136	1.019217	1.615397	1.48534	1.26358189	1.68318474	1.65707652	55	116	104
79729	NM_001162530,NM_024676	SH3D21	SH3 domain containing 21	1.8336333	1.536219313	4.957315	9.481626	7.329501	3.67442132	4.49696276	4.25393069	462	1132	873
57678	NM_001244949,NM_020918	GPAM	glycerol-3-phosphate acyltransfe	-4.80648861	1.536191874	1.150312	0.237163	1.658791	3.0281061	1.31437719	3.58455875	281	78	531
9455	NM_004839,NM_199330	HOMER2	homer scaffolding protein 2	1.119574578	1.535600089	4.911573	5.503815	7.08867	3.37835054	3.52644784	3.9480763	369	552	697
23542	NM_012324	MAPK8IP2	mitogen-activated protein kinase	1.419722487	1.535249934	0.904739	1.282618	1.303236	1.9928118	2.38738651	2.47942639	117	222	221
53635	NM_001305105,NM_001305108	PTOV1	prostate tumor overexpressed 1	1.033764219	1.535232059	52.824468	54.753304	76.573103	6.43048263	6.47784488	7.04311286	3347	4623	6320
84989	NR_027182	JMJD1C-AS1	JMJD1C antisense RNA 1	-1.518180238	1.535207238	2.098976	1.386897	3.055786	1.90722898	1.49109792	2.38534784	108	95	204
23624	NM_001130852,NM_012116	CBLC	Cbl proto-oncogene C	-1.34865896	1.535201999	34.238707	25.568503	49.050307	5.7268601	5.30477538	6.33578786	2040	2021	3852
6487	NM_001270459,NM_001270460	ST3GAL3	ST3 beta-galactoside alpha-2,3-s	2.002301693	1.535156788	0.966038	2.195732	1.611985	1.81624731	2.597368	2.28470378	99	265	187
55640	NM_001195283,NM_017791	FLVCR2	feline leukemia virus subgroup C	-1.04149748	1.535156788	0.704266	0.680751	1.018989	1.81624731	1.77444288	2.28470378	99	127	187
730755	NM_001165252	KRTAP2-3	keratin associated protein 2-3	-1.226720353	1.53509626	2.761579	2.239849	3.977965	1.7191395	1.52015496	2.1765086	90	98	170
23294	NM_015245	ANKS1A	ankyrin repeat and sterile alpha r	1.184790356	1.535089741	2.578602	3.058606	3.718163	4.08278037	4.31410799	4.67120285	626	991	1182
23233	NM_001321729,NM_001321730	EXOC6B	exocyst complex component 6B	1.594709929	1.534838472	3.30398	5.279707	4.799668	4.32753359	4.9738534	4.92043435	749	1596	1414
26130	NM_001282679,NM_001282680	GAPVD1	GTPase activating protein and VI	1.166486515	1.534650986	10.175172	12.770396	15.496849	6.26450698	6.48400307	6.87588368	2979	4643	5623
647121	NR_003955	EMBP1	embigin pseudogene 1	1.101608519	1.534560665	0.770425	0.848214	1.111822	2.0648444	2.17238371	2.5576298	125	184	236
84447	NM_032431,NM_172230	SYVN1	synoviolin 1	1.12164333	1.534182278	28.756377	32.285493	41.465115	6.43303663	6.59684362	7.04469779	3353	5025	6327
51087	NM_015982	YBX2	Y-box binding protein 2	-2.198952917	1.534075013	1.463989	0.664123	2.118994	1.70793493	1.02107213	2.16322937	89	54	168
9516	NM_001136472,NM_001136473	LITAF	lipopolysaccharide induced TNF	-1.065148678	1.533949126	66.415744	62.493019	95.871578	7.33715107	7.24667581	7.95130153	6309	7914	11903
1291	NM_001848	COL6A1	collagen type VI alpha 1 chain	2.33053023	1.533634078	74.534088	173.905991	107.422592	8.26501111	9.48299667	8.8803373	12038	37485	22707
408050	NM_001004067	NOMO3	NODAL modulator 3	1.268481964	1.533516849	16.979977	21.56708	24.472094	6.17732075	6.51620975	6.78723249	2802	4749	5285
2050	NM_004444	EPHB4	EPH receptor B4	-1.350529442	1.533245246	16.322079	12.09751	23.516716	6.13245466	5.70610059	6.74189371	2715	2686	5120
9133	NM_004701	CCNB2	cyclin B2	1.217576407	1.533200156	51.590965	62.888962	74.336823	6.30038432	6.58113081	6.91056862	3055	4970	5761
27074	NM_014398	LAMP3	lysosomal associated membrane	1.060824476	1.53314173	2.601467	2.768349	3.756851	3.24517298	3.3216324	3.80791592	333	472	628
90550	NM_001270679,NM_001270680	MCU	mitochondrial calcium uniporter	-1.625959626	1.533109606	21.249457	13.301455	30.611412	5.98069949	5.29359953	6.58921703	2440	2005	4601
58985	NM_021258	IL22RA1	interleukin 22 receptor subunit al	-1.809425761	1.532664445	2.999484	1.656587	4.322944	3.27204958	2.53229878	3.83538509	340	251	641
440519	NR_045525	ZNF724	zinc finger protein 724	1.039258885	1.532446537	0.790292	0.846347	1.166168	0.54446555	0.56226574	0.76902392	18	25	34
646450	NM_001123040	ARIH2OS	ariadne homolog 2 opposite strar	1.393851744	1.532353236	0.709638	1.000497	1.03757	1.08456011	1.3579479	1.4430085	44	82	83
400673	NM_001017921	VMAC	vimentin-type intermediate filame	1.432056851	1.532329133	1.090997	1.567246	1.577185	1.47662927	1.82964782	1.90057841	70	134	132
114781	NM_001099272,NM_001172418	BTBD9	BTB domain containing 9	1.152286782	1.532299613	0.736082	0.847117	1.084274	2.89117014	3.06980877	3.43788301	252	388	475
55650	NM_001202554,NM_017837	PIGV	phosphatidylinositol glycan ancho	1.159286792	1.531579269	9.538737	10.391939	13.708733	4.54619271	4.750944	5.13968208	878	1360	1654
645958	NR_077247	RPS18P9	ribosomal protein S18 pseudoge	-1.531493358	1.531524271	2.104525	1.37385	3.014507	1.57857476	1.19932051	2.01587867	78	68	147
389072	NM_001080475	PLEKHM3	pleckstrin homology domain cont	1.145970512	1.531463832	0.595168	0.68438	0.85954	2.74535098	2.91432819	3.28382219	224	343	422
6296	NM_005622,NM_202000	ACSM3	acyl-CoA synthetase medium-cha	-1.55260268	1.530765646	3.471879	2.164988	5.122821	2.9541672	2.41858604	3.50257812	265	228	499
1613	NM_001348	DAPK3	death associated protein kinase 3	1.079700488	1.530391418	11.741962	12.692786	16.885071	4.77681668	4.88356843	5.37241474	1037	1496	1952
1397	NM_001270837,NM_001270841	CRIP2	cysteine rich protein 2	1.48108554	1.530152574	27.365983	39.378119	41.414872	5.12523965	5.6784421	5.72456337	1331	2634	2505
5533	NM_001243974,NM_001243975	PPP3CC	protein phosphatase 3 catalytic s	-1.00595756	1.529898841	2.200432	2.186268	3.165597	2.57697339	2.56983975	3.10438964	195	259	367
57549	NM_001135050,NM_020789	IGSF9	immunoglobulin superfamily men	1.28164103	1.529872487	7.104033	9.164023	10.219979	4.85762016	5.20466589	5.45374301	1099	1882	2068
4642	NM_001303279,NM_001303280	MYO1D	myosin ID	-1.209195915	1.529697543	14.422217	11.860432	20.454487	6.24767961	5.97758716	6.85435545	2944	3253	5539
6520	NM_001012662,NM_001012664	SLC3A2	solute carrier family 3 member 2	1.215222268	1.52919465	181.037375	216.976492	258.850378	8.42270981	8.70318727	9.03403037	13433	21811	25265
8363	NM_021968	HIST1H4J	histone cluster 1 H4 family memb	1.655394661	1.529171058	0.652286	1.06796	0.930036	0.29776659	0.46599441	0.43515728	9	20	17
646576	NR_037595	HHIP-AS1	HHIP antisense RNA 1	1.326665894	1.529171058	0.184885	0.263341	0.282858	0.29776659	0.38406669	0.43515728	9	16	17
353189	NM_180991	SLCO4C1	solute carrier organic anion trans	-1.779540991	1.529039054	0.369734	0.206997	0.533038	1.55375576	1.06116576	1.98602166	76	57	143
4957	NM_001242352,NM_001242353	ODF2	outer dense fiber of sperm tails 2	1.275139421	1.52902836	12.441506	15.972782	17.842231	5.59622736	5.94045074	6.19851688	1860	3169	3498
6768	NM_021978	ST14	suppression of tumorigenicity 14	-2.169919937	1.529025819	104.248604	48.093704	149.787613	8.39208155	7.27944151	9.00321197	13150	8097	24730
5034	NM_000918	P4HB	prolyl 4-hydroxylase subunit beta	1.049544851	1.528899645	414.56427	435.590179	595.630859	10.02454571	10.09424432	10.63656155	40853	57289	76822
152006	NM_022781,NM_194328,NM_19	RNF38	ring finger protein 38	-1.099953881	1.52840609	3.747142	3.309122	5.260545	4.25047234	4.12056519	4.83613057	708	860	1331
2026	NM_001975	ENO2	enolase 2	1.264568243	1.528317136	10.957038	13.867291	15.732561	4.74702075	5.0744123	5.34032578	1015	1715	1908
5429	NM_001291969,NM_001291970	POLH	DNA polymerase eta	-1.072121905	1.527635275	1.437741	1.343555	2.066554	3.67729659	3.58491601	4.24922516	463	577	870
9592	NM_004907	IER2	immediate early response 2	1.137348439	1.52760816	73.886009	84.126183	106.074402	7.21137762	7.39587904	7.81929558	5779	8782	10858
1213	NM_001288653,NM_004859	CLTC	clathrin heavy chain	1.080042575	1.527309401	72.236183	78.106461	103.670697	9.23865527	9.34956693	9.84882522	23678	34169	44479
729082	NR_026757	OIP5-AS1	OIP5 antisense RNA 1	1.026446266	1.527277093	11.704836	12.033958	16.804358	4.49821892	4.53423581	5.08703534	848	1163	1593
55143	NM_001256875,NM_018101	CDCA8	cell division cycle associated 8	1.049179248	1.527212088	31.046782	32.666666	44.959678	6.24767961	6.31605299	6.85202968	2944	4127	5530
4288	NM_001145966,NM_002417	MKI67	marker of proliferation Ki-67	-1.090132335	1.527149241	21.292987	19.79731	30.210947	7.98653864	7.86254658	8.59542044	9918	12156	18629
23191	NM_001033028,NM_001287810	CYFIP1	cytoplasmic FMR1 interacting pro	-1.105336988	1.52657521	16.120084	14.351932	23.051948	6.69484709	6.55182299	7.30034791	4028	4869	7563
3142	NM_021958	HLX	H2.0 like homeobox	2.836190909	1.526176095	0.286315	0.810124	0.404321	0.71085839	1.49109792	0.98063768	25	95	47
101926918	NR_135112	GTF2IP12	general transcription factor IIi pse	1.613990164	1.526176095	0.671959	1.117226	0.983406	0.71085839	1.02107213	0.98063768	25	54	47
10743	NM_030665	RAI1	retinoic acid induced 1	1.239980029	1.526072221	7.024057	8.720689	10.074924	5.73860759	6.0437	6.33911307	2057	3408	3861
9830	NM_014788,NM_033219	TRIM14	tripartite motif containing 14	1.077341564	1.525601383	6.837273	7.376089	9.806314	4.987237	5.09145262	5.5809041	1206	1736	2263
1743	NM_001244883,NM_001933,NR	DLST	dihydrolipoamide S-succinyltrans	1.231789279	1.525531329	45.369341	54.381978	67.051645	6.8363031	7.13468736	7.44127149	4447	7319	8344
84890	NM_032804	ADO	2-aminoethanethiol dioxygenase	-1.19058546	1.52544289	4.711442	3.963434	6.756676	4.1772093	3.94061606	4.75878381	671	753	1259
23530	NM_001331026,NM_012343,NM	NNT	nicotinamide nucleotide transhyd	1.060945104	1.52530536	6.068911	6.107419	8.273875	4.76064069	4.8429386	5.35134557	1025	1453	1923
222236	NM_001122838,NM_198990	NAPEPLD	N-acyl phosphatidylethanolamine	-1.403911225	1.524950654	3.631777	2.583092	5.211624	4.28103662	3.82117143	4.86409893	724	689	1358
23548	NM_012382	TTC33	tetratricopeptide repeat domain 3	1.108909037	1.524782834	0.648121	0.719606	0.930751	2.16656821	2.28388661	2.66044767	137	203	257
4601	NM_001008541,NM_005962,NM	MXI1	MAX interactor 1, dimerization pr	1.360689649	1.524581664	2.898101	4.122902	4.333534	3.43381096	3.84241755	3.99567625	385	700	722
7082	NM_001301025,NM_001301026	TJP1	tight junction protein 1	-1.280145136	1.524486047	9.099461	7.143796	13.059873	6.14808384	5.79744877	6.74941097	2745	2865	5147

25874	NM_001143674,NM_015415,NR	MPC2	mitochondrial pyruvate carrier 2	1.908176953	1.523253884	7.118631	13.489826	9.935877	4.09788139	4.98952124	4.67588584	633	1614	1186
11345	NM_007285	GABARAPL2	GABA type A receptor associated	1.170701351	1.523033613	61.997372	72.674179	88.732895	5.94237097	6.16632849	6.5412625	2375	3715	4449
5516	NM_001009552	PPP2CB	protein phosphatase 2 catalytic s	1.2350147	1.522899805	21.97563	27.167213	31.444626	5.43144524	5.72961516	6.02677467	1655	2731	3100
493869	NM_001008397,NM_001306197	GPX8	glutathione peroxidase 8 (putative	1.18608534	1.522561367	12.054689	13.831461	16.677169	5.45341658	5.69446357	6.04860456	1681	2664	3148
57511	NM_001145079,NM_020751,NR	COG6	component of oligomeric golgi co	1.088507907	1.521937667	4.522694	4.804135	6.362081	4.08061013	4.19603101	4.65706199	625	909	1170
8850	NM_003884	KAT2B	lysine acetyltransferase 2B	-1.49685097	1.521904033	0.5494	0.368981	0.787368	1.84721644	1.45142181	2.30902414	102	91	191
5638	NM_000950,NM_001142395,NM	PRRG1	proline rich and Gla domain 1	-1.243452233	1.521729802	8.128181	6.709264	10.785315	4.98955224	4.68618473	5.57965517	1208	1298	2261
2707	NM_001005752,NM_024009	GJB3	gap junction protein beta 3	-1.009058771	1.521548128	40.422471	36.912588	53.271844	6.3440116	6.33116183	6.94346856	3150	4171	5895
6102	NM_006915	RP2	RP2, ARL3 GTPase activating pr	-1.348340135	1.521494457	2.231287	1.653569	3.192249	3.22173053	2.84329233	3.77337356	327	324	612
84343	NM_001308258,NM_032383	HPS3	HPS3, biogenesis of lysosomal o	1.117805864	1.521372487	11.75724	13.16917	17.06501	5.59318572	5.75071091	6.18831041	1856	2772	3473
167691	NM_001122769,NM_181714	LCA5	LCA5, lebercilin	-1.329308523	1.521162443	1.117663	0.839719	1.645624	2.61945147	2.28388661	3.14210594	202	203	378
57606	NM_020846	SLAIN2	SLAIN motif family member 2	-1.013892139	1.520727884	6.448652	6.368199	9.216494	5.27605672	5.25666827	5.86804962	1482	1953	2772
101060226	NM_001351365,NM_001351365	NBPF19	NBPF member 19	1.051497474	1.52051535	2.168877	2.283597	3.10027	4.85126892	4.92127111	5.43866688	1094	1537	2046
101929780	NR_104217,NR_104217_dup1,N	NBPF25P	NBPF member 25, pseudogene	-1.067202121	1.520354371	3.66981	3.442584	5.244829	3.80108857	3.71417485	4.36973462	508	636	950
55534	NM_018717	MAML3	mastermind like transcriptional co	1.454838515	1.520166353	0.39727	0.57898	0.568052	1.90722898	2.32288731	2.37386535	108	210	202
151313	NM_001320848,NM_001320849	FAHD2B	fumarylacetoacetate hydrolase d	-1.212360087	1.520166353	2.138305	1.686513	2.972548	1.90722898	1.70865125	2.37386535	108	119	202
3709	NM_002223	ITPR2	inositol 1,4,5-trisphosphate recep	-1.464305568	1.520000121	0.924122	0.631821	1.320511	3.62464406	3.12759409	4.18825403	445	406	832
1209	NM_001282175,NM_001282176	CLPTM1	CLPTM1, transmembrane protein	1.228669784	1.519888026	48.900394	60.306039	70.238331	6.97274618	7.26770998	7.57278825	4892	8031	9145
8720	NM_003791	MBTPS1	membrane bound transcription fa	1.218928582	1.519851214	28.064356	34.24889	40.085579	6.91514723	7.19861809	7.51499452	4699	7653	8784
6251	NM_012425,NM_152724	RSU1	Ras suppressor protein 1	1.004787577	1.519783127	8.992632	9.013786	12.78225	5.05954162	5.06622657	5.64857748	1270	1705	2374
64431	NM_022496,NR_048568,NR_04	ACTR6	ARP6 actin related protein 6 hom	-1.192849681	1.519764365	10.418386	8.795588	14.879289	4.64202642	4.39868427	5.2260334	941	1054	1759
4502	NM_005953	MT2A	metallothionein 2A	-1.465774236	1.519366369	105.129463	71.801201	150.123581	5.45593034	4.91945464	6.04815313	1684	1535	3147
85415	NM_033103	RHPN2	rhophilin Rho GTPase binding pr	-1.057015442	1.519316466	26.233011	24.847099	37.454647	6.51085424	6.43175704	7.10887449	3541	4476	6617
51276	NM_001290314,NM_001321272	ZNF571	zinc finger protein 571	-1.135629416	1.51914851	1.98945	2.214455	3.280549	2.72881625	2.57446443	3.2559454	221	260	413
149371	NM_175876	EXOC8	exocyst complex component 8	-1.171940372	1.519126085	6.536919	5.583349	9.331336	5.0759764	4.85439274	5.66456951	1285	1465	2401
54793	NM_017634	KCTD9	potassium channel tetramerizatio	-1.019649898	1.519076382	9.93703	9.755014	14.186504	5.08358236	5.05634199	5.6722066	1292	1693	2414
10455	NM_001166010,NM_006117,NM	ECI2	enoyl-CoA delta isomerase 2	1.504759135	1.518987147	14.023464	21.136887	20.017986	4.33301385	4.89839912	4.9115275	752	1512	1405
391059	NM_001013660	FRRS1	ferric chelate reductase 1	1.281569581	1.518645126	1.628947	2.087496	2.321938	2.39332696	2.68977954	2.89942777	167	286	312
375287	NM_198557	RBM43	RNA binding motif protein 43	1.092042933	1.51846737	2.77665	3.035342	3.967376	3.53555296	3.65208684	4.09517603	416	607	777
729852	NM_001302348,NM_001302349	UMAD1	UBAP1-MVB12-associated (UMA	-1.366063683	1.518278508	3.018049	2.193177	4.325452	2.98698269	2.6019054	3.5261099	272	266	508
2879	NM_001039847,NM_001039848	GPX4	glutathione peroxidase 4	-1.031645228	1.518203593	247.387772	242.745904	354.299106	7.83818952	7.79344154	8.43840747	8945	11585	16703
57684	NM_001304363,NM_001304364	ZBTB26	zinc finger and BTB domain cont	1.197042138	1.518116046	4.75782	5.603184	6.782787	3.40634723	3.64330938	3.96156336	377	603	704
10948	NM_001165937,NM_001165938	STARD3	StAR related lipid transfer domai	1.446263094	1.518042133	20.065871	29.153112	28.649589	5.81761456	6.34205155	6.41109565	2175	4203	4061
6197	NM_004586	RPS6KA3	ribosomal protein S6 kinase A3	1.123400792	1.517912008	5.022484	5.649282	7.166101	5.27510805	5.43889362	5.86446437	1481	2223	2765
100506385	NR_038885,NR_038886	LINC01426	long intergenic non-protein codin	5.025069432	1.517529052	0.319644	1.517108	0.495971	0.66524311	1.98361768	0.91876801	23	155	43
2683	NM_001497	B4GALT1	beta-1,4-galactosyltransferase 1	1.598314627	1.517321318	17.230522	27.574709	24.568657	6.15892439	6.82791842	6.75357037	2766	5907	5162
100506465	NR_110025,NR_110026	LINC01234	long intergenic non-protein codin	1.859587532	1.517222165	0.090926	0.175646	0.125939	0.26755841	0.46599441	0.39027104	8	20	15
100129924	NM_001242750	TMEM269	transmembrane protein 269	-1.067926637	1.517222165	0.208979	0.180135	0.299278	0.26755841	0.25173066	0.39027104	8	10	15
9228	NM_001346810,NR_073397	DLGAP2	DLG associated protein 2	1.490310084	1.517222165	0.020241	0.03037	0.027098	0.26755841	0.38406669	0.39027104	8	16	15
253782	NM_001256126,NM_203463	CERS6	ceramide synthase 6	1.167227201	1.516725956	9.229845	10.781573	13.153893	5.96254669	6.18232282	6.55562641	2409	3757	4494
6171	NM_001035267,NM_021104	RPL41	ribosomal protein L41	1.064151117	1.516533616	722.162674	773.206504	1046.080681	8.42890749	8.51835883	9.02826251	13491	19182	25164
3845	NM_004985,NM_033360	KRAS	KRAS proto-oncogene, GTPase	-1.034356441	1.516041694	7.687525	7.450028	10.995195	5.47505833	5.4274358	6.0643165	1707	2205	3183
23338	NM_001289984,NM_001289985	JADE2	jade family PHD finger 2	-1.053021858	1.515885607	5.458283	5.263757	7.872868	5.17592889	5.10350222	5.76248297	1380	1751	2573
130940	NM_001301684,NM_001301685	CCDC148	coiled-coil domain containing 148	1.007492492	1.515541151	0.731157	0.534785	1.03711	1.21691895	1.22307764	1.58918956	52	70	97
10103	NM_005727	TSPAN1	tetraspanin 1	-1.426252313	1.515447447	94.52533	66.34137	134.607529	7.23766831	6.72949688	7.83416643	5886	5514	10971
54585	NM_001276378,NM_001276379	LZTFL1	leucine zipper transcription factor	1.567084256	1.51512236	1.22693	1.931164	1.754233	2.55212659	3.10858833	3.06566078	191	400	356
83699	NM_031469	SH3BGRL2	SH3 domain binding glutamate ri	-2.116577509	1.51466862	2.326388	1.100843	3.312004	3.58232086	2.62883416	4.13992569	431	272	803
80709	NM_001317950,NM_001317952	AKNA	AT-hook transcription factor	-1.336026837	1.514455309	2.40436	1.802728	3.452214	3.78784692	3.40448323	4.35082401	503	503	937
60484	NM_021817	HAPLN2	hyaluronan and proteoglycan link	1.443807002	1.513854441	0.209053	0.312511	0.307338	0.46682716	0.63490936	0.65979727	15	29	28
54918	NM_017801	CMTM6	CKLF like MARVEL transmembra	-1.133796061	1.513808911	31.006828	27.37726	44.111679	6.68130478	6.50201808	7.2747224	3990	4702	7429
254065	NM_153252	BRWD3	bromodomain and WD repeat do	-1.038080174	1.513779246	3.567858	3.440971	5.075531	5.50611581	5.45340297	6.09348707	1745	2246	3249
405	NM_001197325,NM_001286035	ARNT	aryl hydrocarbon receptor nuclea	1.113905062	1.51369366	5.745575	6.402459	8.153857	4.8216876	4.97210198	5.40239335	1071	1594	1994
780	NM_001202521,NM_001202522	DDR1	discoidin domain receptor tyrosin	1.05978543	1.513280747	53.811812	57.550139	75.392292	7.6411392	7.72450476	8.23637248	7798	11042	14514
58533	NM_021249,NM_152233	SNX6	sorting nexin 6	-1.713505324	1.513198853	20.47495	12.019013	29.267996	6.10602377	5.3439002	6.69652477	2665	2078	4960
114823	NM_052925	LENG8	leukocyte receptor cluster memb	1.109254628	1.513168879	78.475029	87.14669	111.590324	8.1579237	8.30701803	8.75378755	11174	16561	20796
27063	NM_014391	ANKRD1	ankyrin repeat domain 1	-27.03247606	1.512752079	45.340187	1.67127	64.467674	6.4620872	2.07783221	7.05372131	3422	169	6367
375346	NM_198563	TMEM110	transmembrane protein 110	1.31196605	1.512651011	2.259036	2.96465	3.207723	3.51319803	3.87464915	4.06693155	409	717	761
6696	NM_000582,NM_001040058,NM	SPP1	secreted phosphoprotein 1	-1.276312319	1.512618949	0.317138	0.248425	0.494197	0.64188297	0.52452456	0.88680889	22	23	41
84162	NM_015312	KIAA1109	KIAA1109	-1.244360755	1.512609031	5.102715	4.104856	7.253279	6.29243623	5.98150995	6.88324207	3038	3262	5652
6738	NM_001042369,NM_001042370	TROVE2	TROVE domain family member 2	1.089095038	1.512148714	6.171956	7.031404	8.700708	4.26200996	4.37899217	4.83298924	714	1039	1328
84276	NM_032316	NICN1	nicolin 1	1.365168668	1.512027631	2.943131	4.030048	4.187854	3.36057263	3.77168848	3.90883222	364	664	677
92255	NM_001007527	LMBRD2	LMBR1 domain containing 2	-1.035452584	1.511832519	0.748021	0.722442	1.0622	1.75224037	1.71704117	2.19620105	93	120	173
1808	NM_001197293,NM_001244604	DPYSL2	dihydropyrimidinase like 2	1.138495774	1.511674845	6.652376	7.611048	10.003986	4.95679582	5.13827799	5.53717864	1180	1795	2194
2961	NM_001348353,NM_002095	GTF2E2	general transcription factor IIE su	1.151905856	1.511522309	10.552859	12.151176	14.970896	4.24078667	4.43473883	4.81080629	703	1082	1307
5926	NM_002892,NM_023000,NM_02	ARID4A	AT-rich interaction domain 4A	-1.088112107	1.511280488	5.77944	5.317151	8.178745	5.0573361	4.93931117	5.63841672	1268	1557	2357
2041	NM_005232	EPHA1	EPH receptor A1	-1.19780692	1.511241762	8.756743	7.316485	12.438092	4.89144191	4.64060352	5.47068589	1126	1256	2093
9682	NM_014663	KDM4A	lysine demethylase 4A	1.133218878	1.51116372	8.620297	9.780021	12.240088	5.27984516	5.45591151	5.86292511	1486	2250	2762
5119	NM_001083314,NM_002768,NR	CHMP1A	charged multivesicular body prot	1.27815456	1.510954422	42.666829	55.059107	61.238082	6.68380898	7.03482252	7.27452944	3997	6826	7428
23380	NM_001170637,NM_001300952	SRGAP2	SLIT-ROBO Rho GTPase activat	1.107574027	1.510891729	14.50826	16.210516	21.523853	6.28774036	6.43334909	6.87690087	3028	4481	5627
57222	NM_001031711	ERGIC1	endoplasmic reticulum-golgi inter	1.436976659	1.510885519	23.832275	34.288288	33.839134	6.11134883	6.62804307	6.69968997	2675	5136	4971
29888	NM_001039877,NM_013403	STRN4	striatin 4	-1.039876322	1.510202024	43.695889	42.003664	61.918465	7.09792936	7.04193615	7.68911882	5339	6860	9917
89782	NM_001136049,NM_033029,NR	LMLN	leishmanolysin like peptidase	2.097836126	1.510058116	2.028445	4.253534	2.898921	3.91752664	4.93572116	4.47961067	554	1553	1029
1889	NM_001113347,NM_001113348	ECE1	endothelin converting enzyme 1	1.564845209	1.509924344	32.262811	50.635813	45.898691	7.34440507	7.98722581	7.93588874	6341	13258	11776
3551	NM_001190720,NM_001242778	IKBKB	inhibitor of nuclear factor kappa E	1.118282867	1.509863836	12.551294	13.994339	17.869405	5.67960666	5.83791964	6.26451583	1973	2948	3664
7846	NM_001270399,NM_001270400	TUBA1A	tubulin alpha 1a	-2.683919869	1.509389449	41.243488	16.008179	61.497538	6.45582143	5.05881949	7.04424513	3407	1696	6325
23567	NM_001308213,NM_001308214	ZNF346	zinc finger protein 346	1.535630785	1.509235363	2.798819	4.145183	3.628029	3.49701545	4.07069878	4.04718766	404	829	750
6138	NM_001253379,NM_001253380	RPL15	ribosomal protein L15	1.86511821	1.50900578	147.230306	271.746693	197.095047	8.47559305	9.37298412	9.06782754	13936	34729	25865
6772	NM_007315,NM_139266	STAT1	signal transducer and activator o	1.199176303	1.508799817	27.23937	31.605358	37.56181	6.71422537	6.97399142	7.30299912	4083	6542	7577
79042	NM_001077446,NM_001282332	TSEN34	tRNA splicing endonuclease subu	1.182953741	1.50864681	24.163855	28.200864	33.717475	5.76181945	6.00010634	6.3461079	2091	3305	3880
100996432	NR_136571	LINC01663	long intergenic non-protein codin	2.236223666	1.508166923	0.787777	1.778669	1.126337	1.79522562	2.70672986	2.24113096	97	290	180
9991	NM_001163788,NM_001163790	PTBP3	polypyrimidine tract binding prote	-6.574283229	1.508096014	18.599142	2.850726	26.99707	7.18257666	4.51992154	7.77196467	5664	1151	10506
840	NM_001227,NM_001267056,NM	CASP7	caspase 7	1.149905207	1.506553358	8.347466	9.886622	12.065967	4.480221	4.67330781	5.04963668	837	1286	1551
221914	NM_152742	GPC2	glypican 2	1.169051928	1.506349232	0.476357	0.5635	0.684375	1.15225689	1.28081328	1.50747923	48	75	89
91833	NM_001242414,NM_001242415	WDR20	WD repeat domain 20	1.199938971	1.50631545	9.585177	11.593653	13.838822	4.59566527	4.84867703	5.16655045	910	1459	1686
80824	NM_030640	DUSP16	dual specificity phosphatase 16	-1.366015592	1.506221653	2.642252	1.935162	3.740604	4.10857187	3.68880651	4.67120285	638	624	1182
8239	NM_001039590,NM_001039591	USP9X	ubiquitin specific peptidase 9, X-l	1.351512482	1.506043284	7.948228	10.758036	11.252898	6.59612854	7.02683026	7.18188678	3759	6788	6963
127255	NM_001105659,NM_001322315	LRRIQ3	leucine rich repeats and IQ motif	1.121721245	1.505900467	0.120328	0.1377	0.181446	0.43999062	0.48576948	0.62147005	14	21	26
1132	NM_000741	CHRM4	cholinergic receptor muscarinic 4	-2.314716033	1.505900467	0.195728	0.086303	0.275562	0.43999062	0.20477631	0.62147005	14	8	26
653483	NR_027906	AFDN-AS1	AFDN antisense RNA 1 (head to	-1.763607491	1.505735857	0.456512	0.261719	0.656824	1.03161781	0.669904	1.36396013	41	31	76
9780	NM_001142864	PIEZO1	piezo type mechanosensitive ion	1.224947804	1.505610472	44.258575	54.274693	62.620972	8.44944629	8.74141055	9.03841222	13685	22398	25342
89848	NM_033449	FCHSD1	FCH and double SH3 domains 1	1.398639054	1.505189668	11.444456	16.021961	16.184986	5.61883752	6.09449202	6.19892364	1890	3532	3499
23125	NM_001171166,NM_001171167	CAMTA2	calmodulin binding transcription a	-1.392889626	1.505083941	7.833776	5.587198	10.960482	5.11785073	4.65595783	5.69372425	1324	1270	2451
54623	NM_001256826,NM_019088,NR	PAF1	PAF1 homolog, Paf1/RNA polym	-1.339289377	1.504540841	28.905054	21.697389	40.842991	6.02764362	5.61363926	6.60955312	2522	2516	4667
643977	NR_104643	FLJ32255	uncharacterized LOC643977	1.375810229	1.504467506	3.105642	4.279156	4.392578	3.22566415	3.64330938	3.76240724	328	603	607
8878	NM_001142298,NM_001142299	SQSTM1	sequestosome 1	1.281237457	1.504428366	200.727017	257.483565	283.931397	9.14964905	9.50663078	9.73801496	22259	38105	41187
10768	NM_001242673,NM_001242674	AHCYL1	adenosylhomocysteinase like 1	1.032099667	1.504359212	24.561854	25.305278	34.405495	6.57624091	6.62135418	7.16032666	3707	5112	6859
10367	NM_001195518,NM_001195519	MICU1	mitochondrial calcium uptake 1	-1.53860977	1.504130338	12.072359	7.855221	17.078596	4.91721027	4.32100453	5.49009354	1147	996	2122
3714	NM_002226,NM_145159	JAG2	jagged 2	-1.042631413	1.504077299	6.553559	6.306658	9.270603	5.2482889	5.18967248	5.8244272	1453	1862	2688
51780	NM_016604	KDM3B	lysine demethylase 3B	1.05428538	1.504038696	16.855909	17.789408	23.823593	6.81785555	6.89346419	7.40241765	4390	6184	8121

3801	NM_001130099,NM_001130100	KIFC3	kinesin family member C3	1.13956858	1.50381524	31.294673	35.136432	43.479507	6.68595199	6.87272728	7.26989072	4003	6095	7404
157	NM_005160	GRK3	G protein-coupled receptor kinas	1.37872607	1.503813092	18.227959	25.162027	25.760908	7.33737831	7.79826828	7.92302035	6310	11624	11671
1740	NM_001142699,NM_001142700	DLG2	discs large MAGUK scaffold prot	-2.56086594	1.503558916	0.074965	0.027719	0.103979	0.7550755	0.3412894	1.02535867	27	14	50
401331	NR_024116	RASA4CP	RAS p21 protein activator 4C, ps	-1.385392244	1.503558916	0.452721	0.333586	0.64394	0.7550755	0.58077243	1.02535867	27	26	50
8645	NM_003740	KCNK5	potassium two pore domain chan	1.492313723	1.50342713	9.166303	13.694914	12.954919	5.11996571	5.68380238	5.69430137	1326	2644	2452
79710	NM_001085354,NM_024657	MORC4	MORC family CW-type zinc finge	-1.12597825	1.503253066	4.800564	4.277554	6.803633	4.23884171	4.07723084	4.80119389	702	833	1298
6548	NM_003047,NR_046474	SLC9A1	solute carrier family 9 member A	1.720066353	1.503145519	6.97192	11.748011	9.723312	5.02271227	5.78652845	5.59580755	1237	2843	2287
64855	NM_001035534,NM_022833	FAM129B	family with sequence similarity 12	1.112496359	1.503042773	95.050879	105.841597	134.400799	8.53098391	8.68439115	9.11756777	14483	21528	26774
10550	NM_006407	ARL6IP5	ADP ribosylation factor like GTPa	1.013203474	1.502751693	43.91576	44.549889	62.025105	6.52369162	6.54241176	7.10605791	3573	4837	6604
728340	NM_001098728	GTF2H2C	GTF2H2 family member C	1.178307218	1.502735119	0.485141	0.579641	0.702395	1.01352966	1.1381576	1.34055586	40	63	74
80864	NM_030652,NR_037860	EGFL8	EGF like domain multiple 8	1.308933772	1.502735119	0.77558	1.031591	1.109051	1.01352966	1.22307764	1.34055586	40	70	74
3631	NM_001134224,NM_001134225	INPP4A	inositol polyphosphate-4-phospha	1.154144133	1.502656355	6.307068	7.129156	8.671678	5.35539329	5.55751527	5.9311056	1568	2418	2898
201181	NM_001242704	ZNF385C	zinc finger protein 385C	1.070347778	1.502543639	0.864557	0.925888	1.223328	1.67378945	1.74192207	2.10191219	86	123	159
729987	NR_046088	LINC01776	long intergenic non-protein codin	1.489387219	1.501913117	0.069301	0.120987	0.109788	0.23670415	0.3412894	0.34394329	7	14	13
5624	NM_000312	PROC	protein C, inactivator of coagulati	-2.292028919	1.501913117	0.102736	0.043939	0.145982	0.23670415	0.10601836	0.34394329	7	4	13
6248	NM_006511	RSC1A1	regulator of solute carriers 1	-2.157229137	1.501681477	4.347663	2.015526	6.147809	3.43720745	2.47408961	3.97872063	386	239	713
1519	NM_001334	CTSO	cathepsin O	1.307785987	1.50166133	2.681717	3.511554	3.783447	3.1155	3.46301778	3.64551432	301	526	556
1408	NM_001127457,NM_021117	CRY2	cryptochrome circadian clock 2	1.442090502	1.501543353	4.510344	6.50979	6.365397	4.28292556	4.78824961	4.84447416	725	1397	1339
55224	NM_001297760,NM_001297761	ETNK2	ethanolamine kinase 2	-1.023016598	1.501484686	2.799753	2.755741	3.992951	3.0281061	2.99933041	3.55435929	281	367	519
83547	NM_031430	RILP	Rab interacting lysosomal protein	-1.557554608	1.501484686	4.09209	2.633943	5.791747	3.0281061	2.48395571	3.55435929	281	241	519
9498	NM_001039960,NM_001258401	SLC4A8	solute carrier family 4 member 8	-1.160845593	1.501333922	0.043444	0.043843	0.090491	0.59399636	0.52452456	0.82068496	20	23	37
10142	NM_005751,NM_147185	AKAP9	A-kinase anchoring protein 9	-1.585927195	1.501303048	4.635921	2.924783	6.545855	5.83959881	5.1889187	6.41740132	2209	1861	4079
29925	NM_013334,NM_021971	GMPPB	GDP-mannose pyrophosphorylas	1.417491974	1.501268465	4.407107	6.250276	6.205967	2.99622306	3.44545299	3.52091369	274	519	506
56141	NM_018910,NM_031852	PCDHA7	protocadherin alpha 7	1.653508472	1.501209517	0.356597	0.602371	0.512473	0.88013425	1.25799553	1.17840727	33	73	61
122553	NM_001079537,NM_177452	TRAPPC6B	trafficking protein particle comple	1.478607505	1.501107956	16.974183	25.180612	23.645218	5.79065071	6.34645212	6.3679768	2134	4216	3940
5881	NM_001316307,NM_005052	RAC3	ras-related C3 botulinum toxin su	-1.230178865	1.501030108	12.03365	9.763193	16.936045	3.74737673	3.47295962	4.29712434	488	530	901
51132	NM_016120,NM_183353	RLIM	ring finger protein, LIM domain in	5.40310772	1.501025017	1.971234	10.705668	2.785799	4.41797983	6.79585153	4.98128857	800	5776	1477
1454	NM_001894,NM_152221	CSNK1E	casein kinase 1 epsilon	1.256749145	1.500521856	11.703544	14.731215	16.509221	5.03733324	5.35805128	5.60811055	1250	2099	2307
83594	NM_001300741,NM_031438	NUDT12	nudix hydrolase 12	-1.068884666	1.500503157	4.452154	4.179997	6.300879	4.01620928	3.92621521	4.57169125	596	745	1100
2932	NM_001146156,NM_002093	GSK3B	glycogen synthase kinase 3 beta	1.506898608	1.500449937	7.69438	11.614235	10.857097	5.75707097	6.33967644	6.33356678	2084	4196	3846
23133	NM_001184896,NM_001184897	PHF8	PHD finger protein 8	1.049707115	1.50011748	2.842844	2.933549	3.597216	3.90531607	3.97074877	4.45802288	549	770	1013
84002	NM_032047	B3GNT5	UDP-GlcNAc:betaGal beta-1,3-N	1.22899285	1.50004526	16.700201	20.54538	23.540689	6.08831031	6.38184143	6.66625094	2632	4322	4856
5887	NM_001244713,NM_001244724	RAD23B	RAD23 homolog B, nucleotide ex	1.238067376	1.499954397	50.638447	62.805758	71.451576	7.68804546	7.9947931	8.27063707	8057	13328	14864
3913	NM_002292	LAMB2	laminin subunit beta 2	1.03203261	1.499891182	21.238291	21.946114	29.939318	6.92031409	6.9654339	7.50120802	4716	6503	8700
9562	NM_001178117,NM_001178118	MINPP1	multiple inositol-polyphosphate p	-1.028692949	1.499885055	10.731088	9.961449	13.332337	4.78616959	4.74685257	5.35353947	1044	1356	1926
51024	NM_016068	FIS1	fission, mitochondrial 1	-1.062694205	1.499842586	41.24995	38.871685	58.175354	4.94013441	4.85534316	5.50924359	1166	1466	2151
79649	NM_001173516,NM_001173517	MAP7D3	MAP7 domain containing 3	1.662761514	1.499835154	5.422265	9.033366	7.647427	4.65082999	5.36140022	5.21642227	947	2104	1747
55313	NM_001099455,NM_018340	CPPED1	calcineurin like phosphoesterase	-1.017434462	1.499667738	5.562673	5.482258	7.825049	5.08466566	5.0604688	5.65511417	1293	1698	2385
51809	NM_017423	GALNT7	polypeptide N-acetylgalactosami	1.096513134	1.498752058	22.959551	25.202885	32.333183	6.60408301	6.73570375	7.18291525	3780	5538	6968
148103	NM_001007248	ZNF599	zinc finger protein 599	1.272931234	1.498666523	0.643349	0.823978	0.910384	1.56621863	1.80624674	1.97845985	77	131	142
8763	NM_001142401,NM_001142402	CD164	CD164 molecule	1.269786233	1.498196823	54.516808	69.522985	77.690845	7.47819835	7.82106856	8.05874013	6961	11810	12827
6448	NM_000199	SGSH	N-sulfoglucosamine sulfohydrola	-1.367794219	1.497832357	10.181933	7.453254	14.337001	4.83075499	4.39737979	5.39673096	1078	1053	1986
60672	NM_021933	MIIP	migration and invasion inhibitory	1.060626397	1.49679417	12.87454	13.672943	18.122885	4.56027919	4.64170569	5.12177381	887	1257	1633
728175	NR_040108	LINC02363	long intergenic non-protein codin	1.607015754	1.496733267	0.168335	0.284062	0.253512	0.41264541	0.61708849	0.58209675	13	28	24
23250	NM_015205,NM_032189	ATP11A	ATPase phospholipid transportin	1.096743194	1.49636287	4.506822	4.9596	6.364116	5.30887308	5.43889362	5.87824435	1517	2223	2792
58491	NM_021216	ZNF71	zinc finger protein 71	-2.011163269	1.496097079	0.945387	0.466787	1.326973	1.9554003	1.28081328	2.40804223	113	75	208
90007	NM_177401	MIDN	midnolin	1.111915594	1.495945174	25.248209	28.10745	35.49696	6.55841652	6.7099265	7.1344047	3661	5439	6736
578	NM_001188	BAK1	BCL2 antagonist/killer 1	1.067564552	1.495602015	6.31363	6.755693	8.880168	3.85794988	3.94597954	4.40541889	530	756	975
84793	NR_026878	FOXD2-AS1	FOXD2 antisense RNA 1 (head t	1.31648117	1.495269408	1.685868	2.220604	2.369475	2.3579234	2.68551066	2.84224948	162	285	298
23564	NM_001303007,NM_001303008	DDAH2	dimethylarginine dimethylaminoh	-2.286306497	1.495230656	11.831462	5.411259	19.333977	4.37789078	3.27124763	4.93515799	777	454	1429
6948	NM_000355,NM_001184726	TCN2	transcobalamin 2	1.11708451	1.495007502	6.871688	7.624578	9.572632	3.88307318	4.03255443	4.4305758	540	806	993
10580	NM_001034954,NM_001034955	SORBS1	sorbin and SH3 domain containin	-1.935426365	1.494890873	1.417379	0.88407	2.42047	3.47405048	2.63769986	4.01058243	397	274	730
646074	NR_024593	POM121L10P	POM121 transmembrane nucleo	2.081645973	1.494805711	0.051437	0.107537	0.073113	0.56944351	1.00745609	0.78645063	19	53	35
57488	NM_020728	ESYT2	extended synaptotagmin 2	-1.287197481	1.494780897	13.865596	10.785686	19.480145	6.34762596	5.98845757	6.92170099	3158	3278	5806
2553	NM_001320910,NM_001320915	GABPB1	GA binding protein transcription f	-1.020566928	1.494668154	10.073893	9.538328	11.640951	4.26965076	4.24181329	4.82457876	718	940	1320
55336	NM_018378	FBXL8	F-box and leucine rich repeat pro	1.290485048	1.49464298	4.13996	5.361063	5.830784	2.90104285	3.22493445	3.4156568	254	438	467
10053	NM_001300887,NM_005498	AP1M2	adaptor related protein complex	1.041333464	1.494281823	56.240328	58.656092	79.015884	6.59992185	6.65776512	7.17446007	3769	5244	6927
10013	NM_001321225,NM_001321226	HDAC6	histone deacetylase 6	-1.052511012	1.493903693	13.246379	12.528896	18.372095	5.71990486	5.64750225	6.28993987	2030	2577	3730
3075	NM_000186,NM_001014975	CFH	complement factor H	1.036702921	1.493838932	14.866925	16.075403	22.626984	5.56551847	5.61644502	6.13446583	1820	2521	3344
140699	NM_152503,NM_213631,NM_21	MROH8	maestro heat like repeat family m	1.733229261	1.493799251	0.204663	0.372093	0.287382	0.71085839	1.07428646	0.96541723	25	58	46
50649	NM_015320,NM_032995	ARHGEF4	Rho guanine nucleotide exchang	1.089930747	1.493732219	9.797021	10.782691	13.766204	5.20311692	5.3241272	5.76907242	1407	2049	2585
30850	NM_014603	CDR2L	cerebellar degeneration related p	-1.437203092	1.493732219	10.416768	7.256544	14.620782	5.20311692	4.69682839	5.76907242	1407	1308	2585
10130	NM_001282704,NM_001282705	PDIA6	protein disulfide isomerase family	1.479830944	1.493683988	135.972278	201.431564	190.860006	8.27560367	8.83952982	8.85294348	12127	23978	22279
23527	NM_012287	ACAP2	ArfGAP with coiled-coil, ankyrin r	1.337164226	1.492852141	12.824162	17.166836	17.989624	6.5003391	6.91550292	7.07315474	3515	6280	6454
4776	NM_001136022,NM_001198965	NFATC4	nuclear factor of activated T-cells	-1.524771131	1.49260984	6.101501	3.997481	8.416233	4.87775825	4.2946204	5.43935559	1115	977	2047
101928505	NR_104668	LOC101928505	uncharacterized LOC101928505	-1.099904058	1.49257808	2.785505	2.528586	3.898296	2.46162969	2.35011326	2.95056637	177	215	325
84514	NM_001142623,NM_032484	GHDC	GH3 domain containing	-2.163416884	1.492476883	8.846259	4.114494	12.34705	4.47527325	3.43531911	5.0314812	834	515	1531
51164	NM_001135643,NM_001135644	DCTN4	dynactin subunit 4	-1.005421473	1.49236333	14.724943	14.661199	20.65674	5.8267075	5.81904452	6.39591961	2189	2909	4018
23344	NM_001184796,NM_015292	ESYT1	extended synaptotagmin 1	1.070766965	1.492203257	15.806624	16.95924	22.200357	6.05719254	6.15440836	6.62749283	2575	3684	4726
55832	NM_001329674,NM_001329675	CAND1	cullin associated and neddylation	1.195228139	1.491955892	17.61883	21.040806	24.706633	6.69803595	6.95305674	7.27066488	4037	6447	7408
100129480	NM_001195279	MKRN2OS	MKRN2 opposite strand	-1.335744355	1.491226682	3.042472	2.286652	4.286877	2.0823043	1.77444288	2.54232458	127	127	233
148137	NM_001039887	PROSER3	proline and serine rich 3	1.513987192	1.491090927	5.283355	8.016579	7.410546	3.59149522	4.14874812	4.1280136	434	878	796
144110	NM_153347	TMEM86A	transmembrane protein 86A	-1.135299535	1.491037841	1.289828	1.141755	1.816583	2.48807034	2.33928445	2.9773697	181	213	332
55286	NM_001104629,NM_018302	C4orf19	chromosome 4 open reading fran	-1.09726684	1.491010218	1.386089	1.263678	1.937237	2.56460348	2.45415268	3.05850609	193	235	354
23216	NM_001253912,NM_001253913	TBC1D1	TBC1 domain family member 1	-1.022926387	1.490989565	9.168582	7.424264	10.178778	5.35808361	5.32618529	5.92275718	1571	2052	2881
5925	NM_000321	RB1	RB transcriptional corepressor 1	1.307739033	1.490534942	5.162342	6.758497	7.232274	4.64349742	5.01696186	5.20026005	942	1646	1727
64145	NM_001302378,NM_022340,NR	RBSN	rabenosyn, RAB effector	-1.010830284	1.490306891	2.741018	3.016092	4.054011	4.16497021	4.15029781	4.71395121	665	879	1219
4599	NM_001144925,NM_001178046	MX1	MX dynamin like GTPase 1	-1.333383644	1.490189405	12.887547	8.816179	17.933485	5.2722583	4.86952482	5.83545708	1478	1481	2709
84138	NM_032178	SLC7A6OS	solute carrier family 7 member 6	1.357164809	1.490180171	7.394941	10.053919	10.356955	3.67154031	4.08211055	4.20940382	461	836	845
55061	NM_001037175,NM_017982	SUSD4	sushi domain containing 4	-3.496167606	1.489939423	0.72119	0.207155	1.011486	1.65056839	0.68708806	2.06668624	84	32	154
64840	NM_001282167,NM_022825,NM	PORCN	porcupine homolog (Drosophila)	1.415139539	1.489726512	4.859944	7.21955	7.171578	3.40287721	3.86335533	3.93250653	376	711	689
376497	NM_198580	SLC27A1	solute carrier family 27 member	1.162632399	1.48960221	5.376936	6.25959	7.525898	4.30911424	4.51632067	4.85998953	739	1148	1354
210	NM_000031,NM_001003945,NM	ALAD	aminolevulinate dehydratase	-1.069069338	1.48930652	9.693861	9.006298	13.631647	4.9814326	4.88821942	5.54103391	1201	1501	2200
59274	NM_022566	MESDC1	mesoderm development candida	-1.194191627	1.489253418	7.28608	6.10832	10.19875	4.50146728	4.2577156	5.05503907	850	951	1557
57662	NM_001080429,NM_020902	CAMSAP3	calmodulin regulated spectrin ass	1.118958223	1.489194168	5.086188	5.808344	7.257132	4.44184742	4.59694851	4.99447008	814	1217	1491
57106	NM_020378	NAT14	N-acetyltransferase 14 (putative)	1.648533773	1.488830364	12.49966	20.650345	17.501863	4.12551355	4.81389896	4.67237502	646	1423	1183
643853	NM_001039770,NM_001136238	TMPPE	transmembrane protein with meta	1.619398475	1.488766787	0.193468	0.316992	0.2755	0.81895953	1.16293478	1.09694451	30	65	55
118490	NM_001024593	MSS51	MSS51 mitochondrial translationa	-1.91401962	1.488699105	1.854849	0.965011	2.597414	2.4278825	1.71704117	2.9113909	172	120	315
9055	NM_001267580,NM_003981,NM	PRC1	protein regulator of cytokinesis 1	-1.104672578	1.488690062	58.537628	53.265759	82.003426	7.47283	7.33005869	8.04421259	6935	8388	12698
10007	NM_005471	GNPDA1	glucosamine-6-phosphate deami	1.164000977	1.488595978	17.310984	20.169735	24.214678	5.31165148	5.52563064	5.87366563	1520	2364	2783
150384	NR_024009	GTSE1-AS1	GTSE1 antisense RNA 1 (head t	-1.45463322	1.488312276	0.408157	0.282803	0.567245	0.68823103	0.50527715	0.93448595	24	22	44
55454	NM_001319654,NM_001319656	CSGALNACT2	chondroitin sulfate N-acetylgalact	-1.158252426	1.487668514	3.205071	2.884959	4.880372	3.73086534	3.53596345	4.26795589	482	556	882
10956	NM_001017956,NM_001017957	OS9	OS9, endoplasmic reticulum lecti	1.086642589	1.487589636	52.111763	56.822595	72.299021	7.13448031	7.25354111	7.70409709	5477	7952	10021
118429	NM_001145794,NM_001286780	ANTXR2	anthrax toxin receptor 2	-1.252070404	1.487555037	4.740437	3.823374	7.281561	5.15132397	4.83717724	5.71093841	1356	1447	2481
10055	NM_001145713,NM_001145714	SAE1	SUMO1 activating enzyme subur	-1.069194914	1.487050951	36.993785	34.478919	51.84191	6.52369162	6.42824835	7.09101559	3573	4465	6535
402569	NM_001145715	KPNA7	karyopherin subunit alpha 7	-4.15642858	1.486549578	0.706157	0.1701	0.996818	1.13562816	0.36283659	1.47560397	47	15	86
3214	NM_024015	HOXB4	homeobox B4	-1.627449758	1.486423612	19.404776	11.931105	27.105297	5.30050562	4.62061907	5.86035601	1508	1238	2757
8725	NM_001252641,NM_003796,NR	URI1	URI1, prefoldin like chaperone	-1.313870756	1.486252236	20.304993	14.841715	27.787644	6.03830635	5.65133781	6.60280623	2541	2584	4645
3748	NM_004977,NR_110912	KCNC3	potassium voltage-gated channel	1.02391609	1.486102281	1.271431	1.303652	1.784287	2.36507395	2.39263351	2.84224948	163	223	298

9023	NM_003956	CH25H	cholesterol 25-hydroxylase	-1.002073241	1.486052343	0.227444	0.223933	0.318972	0.38477185	0.38406669	0.54161868	12	16	22
629	NM_001710	CFB	complement factor B	1.311964013	1.485997598	7.453725	9.795232	10.409904	4.32753359	4.70212092	4.8753398	749	1313	1369
526	NM_001693	ATP6V1B2	ATPase H+ transporting V1 subu	1.494136218	1.485855848	7.224337	10.811258	10.090991	4.48679171	5.04472373	5.03695187	841	1679	1537
26175	NM_001098621,NM_015676	TMEM251	transmembrane protein 251	1.437348913	1.485732837	7.591467	10.529369	10.643641	3.06808971	3.53833258	3.58206614	290	557	530
126375	NM_175872	ZNF792	zinc finger protein 792	1.537170001	1.48517126	0.737921	1.139045	1.032714	1.92669094	2.40826098	2.36808966	110	226	201
10075	NM_031407	HUWE1	HECT, UBA and WWE domain co	1.158556547	1.485062892	12.410212	14.393686	17.319685	7.48477826	7.69600701	8.05267663	6993	10825	12773
84225	NM_001136046,NM_001267822	ZMYND15	zinc finger MYND-type containing	-3.087862545	1.48498317	0.609872	0.19832	0.86926	1.30878272	0.56226574	1.67590018	58	25	106
821	NM_001024649,NM_001746	CANX	calnexin	1.070513342	1.484980872	132.860878	142.332664	185.26757	9.31685222	9.41500639	9.88655969	24999	35757	45659
7262	NM_003311	PHLDA2	pleckstrin homology like domain f	-1.614116899	1.484839672	39.75489	24.666567	55.509392	5.19511448	4.52828878	5.75254203	1399	1158	2555
4245	NM_001114617,NM_001114618	MGAT1	mannosyl (alpha-1,3-)-glycoprote	1.214646243	1.484662553	19.199394	23.625132	28.247008	5.893137	6.16938873	6.4553484	2294	3723	4189
79890	NM_001319987,NM_024832	RIN3	Ras and Rab interactor 3	1.417702836	1.484532949	2.18299	3.266639	3.046185	3.20189991	3.65863507	3.71995801	322	610	588
337876	NM_175856	CHSY3	chondroitin sulfate synthase 3	2.802918811	1.484337237	0.389424	1.094425	0.539963	1.21691895	2.23798388	1.56919173	52	195	95
25898	NM_001009922,NM_001278536	RCHY1	ring finger and CHY zinc finger do	1.186646891	1.484326279	2.011311	2.35412	2.732756	3.24517298	3.46799726	3.76460718	333	528	608
706	NM_000714,NM_001256530,NM	TSPO	translocator protein	1.077854544	1.484309568	63.041443	67.735857	88.451721	5.78266256	5.88893602	6.34390267	2122	3056	3874
10640	NM_006544	EXOC5	exocyst complex component 5	1.081315889	1.484094274	6.175668	6.687928	8.614923	5.70589297	5.81660668	6.26645766	2010	2904	3669
56900	NM_001322248,NM_020141,NR	TMEM167B	transmembrane protein 167B	-1.242220847	1.48402396	9.801413	7.837685	13.496389	4.75248416	4.45243375	5.30448591	1019	1096	1860
92558	NM_207311,NR_147892,NR_14	BICDL1	BICD family like cargo adaptor 1	-1.666121309	1.483806245	9.816048	5.897703	13.569007	4.94013441	4.23452662	5.49407653	1166	935	2128
79642	NM_024590	ARSJ	arylsulfatase family member J	1.068834927	1.483648454	1.788997	1.911165	2.491056	3.19389071	3.27976824	3.71085992	320	457	584
729515	NM_018452	TMEM242	transmembrane protein 242	1.041598128	1.483600957	2.740985	2.857143	3.822919	3.86048198	3.91531944	4.39693481	531	739	969
79598	NM_001303401,NM_024548	CEP97	centrosomal protein 97	1.491800246	1.483532338	3.565305	5.300824	4.949082	4.78616959	5.3459303	5.33811168	1044	2081	1905
400618	NR_103737,NR_103738	SOX9-AS1	SOX9 antisense RNA 1	-2.043746278	1.48352321	0.402948	0.192747	0.598095	1.11880553	0.65251278	1.45395569	46	30	84
100288152	NR_125375	SLC9A3-AS1	SLC9A3 antisense RNA 1	-1.103978903	1.483507426	5.448566	4.940858	7.595417	3.38540081	3.25693382	3.90883222	371	449	677
57636	NM_001199417	ARHGAP23	Rho GTPase activating protein 2:	-1.24704496	1.48335351	56.739941	45.550781	79.096207	8.3544032	8.03697534	8.92183267	12810	13725	23371
50650	NM_001128615,NM_001128616	ARHGEF3	Rho guanine nucleotide exchang	1.22571563	1.48324581	3.23319	3.985215	4.541648	3.63946355	3.91166916	4.17011265	450	737	821
85455	NM_033510	DISP2	dispatched RND transporter fami	1.153948731	1.482939108	0.677907	0.784345	0.947819	2.11660268	2.278228	2.57277436	131	202	239
643641	NM_001099220	ZNF862	zinc finger protein 862	-1.563200061	1.482932658	2.822241	1.805909	3.93179	4.32936266	3.72461474	4.87432152	750	641	1368
388559	NM_001310127	ZNF888	zinc finger protein 888	-3.134771041	1.48289798	3.402687	1.086455	4.743519	3.2098649	1.86027027	3.72674411	324	138	591
4677	NM_004539	NARS	asparaginyl-tRNA synthetase	-1.006651366	1.482295252	54.927074	54.62735	76.515404	7.26897876	7.25947669	7.8337736	6016	7985	10968
26224	NM_012158	FBXL3	F-box and leucine rich repeat pro	1.153042549	1.482220728	3.620331	4.181508	5.048857	3.75828027	3.94954414	4.29103243	492	758	897
26093	NM_015603	CCDC9	coiled-coil domain containing 9	-1.010655153	1.481865893	9.651145	9.562464	13.44116	4.36010717	4.34556265	4.90456173	767	1014	1398
5007	NM_002556	OSBP	oxysterol binding protein	1.124648376	1.481788958	15.395711	17.335793	21.442717	6.27070749	6.4381147	6.83197669	2992	4496	5453
3004	NM_001258351,NM_005317	GZMM	granzyme M	1.120502413	1.481594656	0.166259	0.179432	0.217122	0.20517559	0.2284445	0.29607837	6	9	11
5916	NM_000966,NM_001042728,NM	RARG	retinoic acid receptor gamma	-1.481942163	1.481058507	16.647736	11.465542	22.945498	5.58018673	5.027119	6.13701529	1839	1658	3350
80144	NM_001166133,NM_025074	FRAS1	Fraser extracellular matrix compl	-1.425722865	1.480407523	3.757476	2.633962	6.3526	5.85110331	5.34998195	6.40898763	2227	2087	4055
79854	NR_024321	LINC00115	long intergenic non-protein codin	1.149096554	1.479953288	1.103353	1.27595	1.538073	1.27880673	1.40024122	1.63800401	56	86	102
1203	NM_006493	CLN5	ceroid-lipofuscinosis, neuronal 5	1.391577639	1.479853495	4.05213	5.644268	5.63573	3.59149522	4.03423402	4.11772436	434	807	790
6193	NM_001009	RPS5	ribosomal protein S5	-1.044564963	1.479692374	526.795166	504.899231	732.544067	8.5739496	8.51121563	9.13802256	14922	19087	27157
727910	NM_001164407	TLCD2	TLC domain containing 2	-1.129893566	1.479685421	3.545427	3.141253	4.929212	4.41797983	4.250509	4.96128769	800	946	1456
4071	NM_014220	TM4SF1	transmembrane 4 L six family me	-2.437036071	1.479406164	485.229126	199.324203	674.594727	9.64367819	8.36113303	10.20811347	31365	17196	57071
84717	NM_001001520,NM_001348169	HDGFL2	HDGF like 2	1.370035091	1.479351599	34.514272	47.584296	48.410216	6.35527652	6.80473506	6.91453664	3175	5812	5777
79065	NM_001077198,NM_024085,NR	ATG9A	autophagy related 9A	1.365058996	1.479245826	13.80591	18.850995	19.103784	5.69812802	6.13966094	6.25398456	1999	3646	3637
1130	NM_000081,NM_001301365,NR	LYST	lysosomal trafficking regulator	-1.719432431	1.47863545	4.735501	3.146841	5.552623	5.28267998	4.52709643	5.83493376	1489	1157	2708
644961	NR_033926	ACTG1P20	actin gamma 1 pseudogene 20	1.581734256	1.478606447	0.59414	0.95318	0.832508	1.35261029	1.79043244	1.72191206	61	129	111
204851	NM_152696,NM_181358,NM_19	HIPK1	homeodomain interacting protein	1.025908294	1.477794216	5.639812	5.715001	8.63083	5.29021237	5.32618529	5.84172222	1497	2052	2721
340485	NM_001010887	ACER2	alkaline ceramidase 2	-1.058037367	1.477344229	1.249429	1.186197	1.738179	2.16656821	2.10365798	2.62213795	137	173	249
100287902	NR_034040	LGALS8-AS1	LGALS8 antisense RNA 1	-1.335033612	1.477058836	0.798113	0.597725	1.105924	1.08456011	0.8787583	1.40965954	44	44	80
57171	NM_001135917,NM_020438,NR	DOLPP1	dolichyldiphosphatase 1	1.122216522	1.476934487	21.214292	23.720606	29.44564	5.51015299	5.67306183	6.06252949	1750	2624	3179
51170	NM_016245	HSD17B11	hydroxysteroid 17-beta dehydrog	-1.198341327	1.476861502	33.154747	27.695555	46.017883	5.9524941	5.69605597	6.5075083	2392	2667	4345
1062	NM_001286734,NM_001813	CENPE	centromere protein E	-1.126923147	1.476833333	11.993899	10.734323	16.760828	6.64284282	6.47227994	7.20069427	3884	4605	7055
26001	NM_001320356,NM_001320357	RNF167	ring finger protein 167	1.251391502	1.476728738	13.077045	16.449036	19.01447	4.71379852	5.0262753	5.25839655	991	1657	1800
100996246	NR_146452	LINC02361	long intergenic non-protein codin	1.20137804	1.47665503	0.570757	0.697835	0.803089	0.88013425	1.00745609	1.16514558	33	53	60
26137	NM_001164342,NM_001164343	ZBTB20	zinc finger and BTB domain conta	1.694674561	1.476221135	0.092046	0.156462	0.125812	1.80577475	2.38738651	2.22843599	98	222	178
115353	NM_001256409,NM_052940	LRRC42	leucine rich repeat containing 42	1.607620423	1.476197368	20.20671	32.59768	28.32	5.17389443	5.84367804	5.72286727	1378	2960	2502
2627	NM_005257	GATA6	GATA binding protein 6	1.659795927	1.476128598	1.839705	3.059052	2.553827	2.95890107	3.61440811	3.45977198	266	590	483
85439	NM_001256430,NM_033104	STON2	stonin 2	1.207092761	1.47515078	0.75179	1.0491	1.042213	3.38891307	3.63669106	3.90484845	372	600	675
5780	NM_002833	PTPN9	protein tyrosine phosphatase, no	1.227076019	1.475070194	5.463077	6.708982	7.574191	4.45865714	4.74172192	4.99821421	824	1351	1495
63935	NM_022104	PCIF1	PDX1 C-terminal inhibiting factor	1.289493338	1.474857771	10.218282	13.192021	14.165446	4.79942658	5.15458457	5.34327263	1054	1816	1912
9779	NM_001134381,NM_001349073	TBC1D5	TBC1 domain family member 5	1.01487552	1.474821106	3.331739	3.381705	5.022584	4.72634668	4.74685257	5.26928318	1000	1356	1814
23335	NM_015285,NM_052834	WDR7	WD repeat domain 7	1.065059193	1.474399462	1.212951	1.292246	1.681936	3.26442151	3.34617992	3.77555686	338	481	613
147968	NM_144691	CAPN12	calpain 12	1.192191657	1.474339372	1.204651	1.444474	1.675483	2.23060596	2.43393638	2.68852679	145	231	263
79571	NM_024523	GCC1	GRIP and coiled-coil domain con	-1.082881728	1.474280936	2.542521	2.350041	3.522081	3.49049124	3.38618649	4.00872756	402	496	729
4051	NM_000896,NM_001199208,NM	CYP4F3	cytochrome P450 family 4 subfar	-3.225040668	1.47394348	10.719916	3.32312	14.847926	5.00794161	3.41483526	5.55317472	1224	507	2219
2108	NM_000126,NM_001127716	ETFA	electron transfer flavoprotein alph	1.046169483	1.473892238	62.236666	65.548804	86.500827	6.36645416	6.43080098	6.92046829	3200	4473	5801
389765	NR_029410	LOC389765	kinesin family member 27 pseudo	-1.589284412	1.473624325	1.099808	0.690995	1.537736	1.54118428	1.1381576	1.93223726	75	63	136
100130361	NM_001145140,NM_001145140	CXorf49	chromosome X open reading fran	-1.28693842	1.473570439	0.38137	0.288682	0.518569	0.7550755	0.61708849	1.01060517	27	28	49
11037	NM_001198595,NM_006873	STON1	stonin 1	1.087777966	1.473449025	0.046989	0.055452	0.070863	0.35634914	0.38406669	0.49997203	11	16	20
2207	NM_004106	FCER1G	Fc fragment of IgE receptor Ig	-1.331809288	1.473449025	0.449235	0.340379	0.671901	0.35634914	0.27464693	0.49997203	11	11	20
101927911	NR_110818	LOC101927911	uncharacterized LOC101927911	2.300688918	1.473449025	0.108855	0.261129	0.155483	0.35634914	0.72085408	0.49997203	11	34	20
3911	NM_005560	LAMA5	laminin subunit alpha 5	-1.094061791	1.473361378	68.197235	62.403645	94.423607	9.56988132	9.44036516	10.1283844	29799	36392	54000
7184	NM_003299	HSP90B1	heat shock protein 90 beta family	-1.060512208	1.472868628	387.03595	365.357452	535.701416	10.07614439	9.99146381	10.63434524	42342	53346	76704
91179	NM_153334,NM_182895	SCARF2	scavenger receptor class F mem	-1.030210978	1.472826496	0.466606	0.458446	0.654883	1.39514558	1.36863803	1.76650173	64	83	116
55667	NM_001330640,NM_017925,NR	DENND4C	DENN domain containing 4C	-1.05398781	1.472754666	3.768401	3.540531	5.389226	4.96623093	4.89285546	5.50989942	1188	1506	2152
80228	NM_001126340,NM_001271818	ORAI2	ORAI calcium release-activated c	-1.023110821	1.472568637	1.599857	1.555821	2.204017	4.14848821	4.1173995	4.68055367	657	858	1190
143684	NM_001330357,NM_144664	FAM76B	family with sequence similarity 76	-1.099438812	1.472321166	5.805064	5.288435	8.039182	4.54619271	4.41553601	5.08439592	878	1067	1590
8863	NM_001289861,NM_001289862	PER3	period circadian clock 3	1.421032892	1.471991158	14.620401	20.775602	20.215043	6.49993314	7.00215532	7.05259645	3514	6672	6362
501	NM_001182,NM_001201377,NM	ALDH7A1	aldehyde dehydrogenase 7 family	1.026593966	1.471991158	18.61802	19.134834	25.837548	6.49993314	6.53738695	7.05259645	3514	4820	6362
51439	NM_016255	FAM8A1	family with sequence similarity 8	-1.045321844	1.471943698	2.295065	2.198104	3.176968	3.53871843	3.48037129	4.05619567	417	533	755
8939	NM_003934	FUBP3	far upstream element binding pro	1.237993763	1.471742042	19.378078	24.019018	26.806976	5.92069636	6.22412724	6.47058904	2339	3869	4234
8803	NM_003850	SUCLA2	succinate-CoA ligase ADP-formir	1.224498295	1.471627499	9.097447	11.151133	12.582335	4.33483599	4.61389559	4.86921928	753	1232	1363
101927668	NR_110114	LOC101927668	uncharacterized LOC101927668	-5.457538505	1.471566936	2.323721	0.41946	3.258234	1.5285023	0.42561204	1.91649468	74	18	134
1789	NM_001207055,NM_001207056	DNMT3B	DNA methyltransferase 3 beta	1.1133065	1.471514467	3.902705	4.428318	5.486004	4.11706758	4.26345507	4.64755715	642	955	1162
64853	NM_022831	AIDA	axin interactor, dorsalization asso	-1.056834014	1.471272995	14.484934	13.722517	20.03289	5.4399354	5.36206908	5.98634592	1665	2105	3013
22932	NM_012230,NM_152992	POMZP3	POM121 and ZP3 fusion	1.057445616	1.471189456	5.223867	5.563674	7.232932	3.15316384	3.22493445	3.65740237	310	438	561
54584	NM_053004	GNB1L	G protein subunit beta 1 like	1.691890014	1.471015829	13.262016	22.490007	18.35092	4.36724694	5.09708829	4.90156607	771	1743	1395
9600	NM_001130848,NM_004910	PITPNM1	phosphatidylinositol transfer prote	1.231603574	1.470730026	8.711138	10.745301	12.047853	5.20411411	5.49729379	5.74810174	1408	2317	2547
2125	NM_001320747,NM_001988	EVPL	envoplakin	-1.444254179	1.470672197	61.528338	42.690165	85.047462	8.63539732	8.10667909	9.1907148	15573	14407	28169
101927279	NR_110514	EP300-AS1	EP300 antisense RNA 1	-2.121756333	1.47035899	0.303416	0.142209	0.419952	0.49317359	0.25173066	0.6785857	16	10	29
92749	NM_145038	DRC1	dynein regulatory complex subun	-3.503336339	1.47035899	0.160916	0.039282	0.227649	0.49317359	0.15624221	0.6785857	16	6	29
55207	NM_018184	ARL8B	ADP ribosylation factor like GTPa	1.269421308	1.470123846	11.098053	14.110261	15.338791	5.05623208	5.39119595	5.59827656	1267	2149	2291
7108	NM_001277233,NM_003273,NR	TM7SF2	transmembrane 7 superfamily me	-1.477068946	1.470026758	17.030355	11.648347	23.434553	4.77681668	4.23890304	5.31578181	1037	938	1875
153527	NM_144723	ZMAT2	zinc finger matrin-type 2	1.268504502	1.469537224	30.578279	38.837051	42.234226	5.62703993	5.9639931	6.17307105	1901	3222	3436
57414	NM_001040456,NM_001040457	RHBDD2	rhomboid domain containing 2	-1.248447265	1.4694221	17.752044	14.438214	25.050944	5.00908319	4.70000624	5.54998966	1225	1311	2214
2314	NM_001256264,NM_001256265	FLII	FLII, actin remodeling protein	-1.417496238	1.469102833	47.894559	33.800502	66.08679	7.67065639	7.17025659	8.2233355	7960	7503	14383
26608	NM_012453	TBL2	transducin beta like 2	-1.137691609	1.46907862	15.207557	13.379196	20.99485	5.52299658	5.34118895	6.0678839	1766	2074	3191
115817	NM_001136050,NM_138452	DHRS1	dehydrogenase/reductase 1	1.080354223	1.468960046	14.514418	15.731977	20.068747	4.45698495	4.5636087	4.99071621	823	1188	1487
116068	NM_001286812,NM_198273	LYSMD3	LysM domain containing 3	1.072050561	1.468883512	5.493011	5.940797	7.594802	4.57268608	4.66898984	5.10797864	895	1282	1617
116254	NM_138785	GINM1	glycoprotein integral membrane 1	1.355457335	1.468804689	19.200092	26.054605	26.50157	5.34005262	5.7694873	5.88331483	1551	2809	2802
26789	NR_002737	SNORD59A	small nucleolar RNA, C/D box 59	-1.121647032	1.468732586	7.124846	6.357911	9.924208	0.61813834	0.56226574	0.83750224	21	25	38
22824	NM_001317381,NM_001317382	HSPA4L	heat shock protein family A (Hsp7	-1.173086837	1.468643567	5.423875	4.626675	7.483592	4.48842972	4.2691718	5.02231705	842	959	1521

90956	NM_052853	ADCK2	aarF domain containing kinase 2	-1.732105221	1.468561847	6.623482	3.825683	9.146944	4.02300634	3.29385837	4.54889714	599	462	1082
51012	NM_001256403,NM_016045	PRELID3B	PRELI domain containing 3B	1.368302433	1.46850373	26.854559	36.259913	36.712873	6.06270057	6.50928233	6.61016492	2585	4726	4669
10009	NM_001184742,NM_006777	ZBTB33	zinc finger and BTB domain cont	1.209875278	1.468445894	5.875789	7.067864	8.110592	4.94610703	5.21284632	5.48543279	1171	1893	2115
2875	NM_005309	GPT	glutamic--pyruvic transaminase	1.179484801	1.467729036	0.34953	0.422727	0.488078	0.73313634	0.83317479	0.98063768	26	41	47
27237	NM_014448	ARHGEF16	Rho guanine nucleotide exchang	-1.124791813	1.467548235	16.489586	14.67749	22.742308	5.57402879	5.40813487	6.11778356	1831	2175	3305
3275	NM_001242864,NM_001242865	PRMT2	protein arginine methyltransferas	1.116921528	1.467508507	18.962353	21.42473	24.432063	5.76249653	5.91924724	6.30739837	2092	3122	3776
5662	NM_001270965,NM_001270966	PSD	pleckstrin and Sec7 domain cont	-2.593862466	1.467280974	0.449703	0.168659	0.616778	1.50279892	0.77006602	1.88448459	72	37	130
401409	NM_001008749	RAB19	RAB19, member RAS oncogene	-2.424477302	1.467048046	0.842095	0.33637	1.168504	0.83964067	0.40498891	1.11084509	31	17	56
64072	NM_001171930,NM_001171931	CDH23	cadherin related 23	1.398919602	1.467048046	0.107119	0.172459	0.116971	0.83964067	1.07428646	1.11084509	31	58	56
5184	NM_000285,NM_001166056,NM	PEPD	peptidase D	1.50659824	1.466836716	46.021305	69.439533	63.500887	6.50762697	7.09359492	7.05529464	3533	7112	6374
79660	NM_001201329,NM_024607	PPP1R3B	protein phosphatase 1 regulatory	1.311374976	1.466812532	1.536535	1.965105	2.101949	3.22173053	3.57571888	3.72448562	327	573	590
10749	NM_006612	KIF1C	kinesin family member 1C	-1.084584767	1.466321752	8.85944	8.179066	12.209694	6.11506471	5.99967658	6.66065085	2682	3304	4837
55752	NM_001306147,NM_018243	SEPT11	septin 11	1.067588175	1.46627253	12.509565	13.369696	17.239971	6.10922116	6.20225632	6.65473245	2671	3810	4817
5783	NM_006264,NM_080683,NM_08	PTPN13	protein tyrosine phosphatase, no	1.433428946	1.466265489	1.908815	2.745051	2.695742	4.08278037	4.5763457	4.60767403	626	1199	1129
3516	NM_005349,NM_015874,NM_20	RBPJ	recombination signal binding prot	1.194284999	1.4662315	12.257405	14.39327	16.490636	6.13035791	6.38316002	6.67592604	2711	4326	4889
30818	NM_001034914,NM_013434	KCNIP3	potassium voltage-gated channel	-1.279617652	1.465891163	0.405423	0.320498	0.578837	1.11880553	0.93737891	1.4430085	46	48	83
23086	NM_001144763,NM_001144764	EXPH5	exophilin 5	-1.856146607	1.465851566	5.297619	2.831707	7.232628	5.73032519	4.86103256	6.27342667	2045	1472	3687
55737	NM_018206	VPS35	VPS35, retromer complex compo	1.231143879	1.465641336	28.462639	35.082344	39.206238	7.1200237	7.41807989	7.66826644	5422	8919	9774
93653	NR_002330	ST7-AS1	ST7 antisense RNA 1	-1.737613589	1.46546353	0.774461	0.439445	1.062684	1.27880673	0.86372326	1.62837236	56	43	101
389541	NM_001008395,NM_001318236	LAMTOR4	late endosomal/lysosomal adapto	-1.447287919	1.465271532	44.444008	30.871873	61.542769	4.81648053	4.30578837	5.35134557	1067	985	1923
3608	NM_001267809,NM_004515	ILF2	interleukin enhancer binding facto	-1.679589896	1.465010995	78.141682	46.473954	107.322499	7.17598467	6.43462146	7.72373502	5638	4485	10159
79443	NM_024513	FYCO1	FYVE and coiled-coil domain con	1.082924757	1.464964213	2.602602	2.821453	3.584814	4.49821892	4.60826865	5.02873806	848	1227	1528
157506	NM_172037	RDH10	retinol dehydrogenase 10	1.317272897	1.464676114	6.046866	7.979343	8.332144	4.6062649	4.98952124	5.13798608	917	1614	1652
4720	NM_001166159,NM_004550	NDUFS2	NADH:ubiquinone oxidoreducta	1.059058704	1.464610547	36.442003	38.659893	50.073019	6.20647461	6.28817029	6.75079877	2860	4047	5152
22820	NM_016128	COPG1	coatomer protein complex subun	1.289485039	1.46394631	53.811592	69.461815	74.027496	7.34032918	7.70512969	7.88737578	6323	10894	11385
2931	NM_019884	GSK3A	glycogen synthase kinase 3 alph	1.048820674	1.463829919	21.238598	22.294138	29.214216	5.52539208	5.59270531	6.06520918	1769	2479	3185
5191	NM_000288	PEX7	peroxisomal biogenesis factor 7	-1.103850867	1.463793849	6.453367	5.849239	8.885231	3.3677101	3.23956766	3.872576	366	443	659
24142	NM_001200016,NM_001200018	NAT6	N-acetyltransferase 6	-1.171196617	1.463299252	4.849963	4.598635	6.596843	2.97768275	2.78063431	3.46789548	270	308	486
84823	NM_032737	LMNB2	lamin B2	1.225284498	1.46313202	49.209	60.366322	67.662033	7.80423171	8.09616447	8.35125296	8736	14302	15721
3073	NM_000520,NM_001318825,NR	HEXA	hexosaminidase subunit alpha	1.062234097	1.463042358	36.161131	38.351328	49.39777	6.58698369	6.67320833	7.13121138	3735	5301	6721
51292	NM_001002000,NM_001002001	GMPR2	guanosine monophosphate reduc	1.212534724	1.463008408	24.086947	29.661841	33.134068	5.54205009	5.81465343	6.08118363	1790	2900	3221
9306	NM_004232	SOCS6	suppressor of cytokine signaling	-1.021110409	1.46296668	11.529161	11.304181	15.85155	6.05829583	6.02861267	6.60034496	2577	3372	4637
22822	NM_007350	PHLDA1	pleckstrin homology like domain	1.14371035	1.462839348	95.206398	109.010231	130.877731	9.10095866	9.29435112	9.64890085	21519	32884	38717
11212	NM_001349346,NM_001349347	PLPBP	pyridoxal phosphate binding prot	1.228622282	1.462784114	5.924333	7.135835	7.810264	3.86553291	4.14408903	4.38268287	533	875	959
51776	NM_016653,NM_133646	ZAK	sterile alpha motif and leucine zip	1.181518245	1.462694481	8.543089	9.343018	10.136904	5.46677296	5.70240807	6.00507466	1697	2679	3053
201595	NM_178862	STT3B	STT3B, catalytic subunit of the ol	1.070636342	1.462661455	31.982374	34.279507	43.963364	7.15267258	7.25047383	7.69806688	5547	7935	9979
206358	NM_001308150,NM_001308151	SLC36A1	solute carrier family 36 member	1.705267366	1.462408116	1.564869	2.658551	2.141617	3.30216484	4.01053992	3.80364308	348	793	626
25939	NM_015474	SAMHD1	SAM and HD domain containing	-1.445103533	1.462256718	3.579368	2.47962	4.920657	3.60061161	3.12128662	4.11082387	437	404	786
10253	NM_001318536,NM_001318537	SPRY2	sprouty RTK signaling antagonist	-1.412103007	1.4621354	3.171198	2.066643	4.221576	3.01452812	2.58825016	3.5052118	278	263	500
4891	NM_000617,NM_001174125,NM	SLC11A2	solute carrier family 11 member 2	-1.278605186	1.462110638	14.152186	11.130641	19.255981	5.80583878	5.45841569	6.3457406	2157	2254	3879
11059	NM_007013	WWP1	WW domain containing E3 ubiqu	1.100508861	1.46198451	9.152613	10.088284	12.580545	5.23273681	5.3674088	5.76852445	1437	2113	2584
23381	NM_001323614,NM_001323615	SMG5	SMG5, nonsense mediated mRN	1.22435076	1.461786508	21.983348	27.053702	30.340877	6.62842965	6.91777938	7.17156155	3845	6290	6913
3127	NM_002125	HLA-DRB5	major histocompatibility complex,	2.738059699	1.461765993	0.660862	1.844049	0.911322	0.81895953	1.63086207	1.08290869	30	110	54
59338	NM_001001974,NM_001195608	PLEKHA1	pleckstrin homology domain cont	-1.25201125	1.461284879	5.559942	4.616216	7.821209	4.47527325	4.16723545	5.00194865	834	890	1499
79541	NM_030908	OR2A4	olfactory receptor family 2 subfan	2.08978529	1.460907986	0.295857	0.620315	0.404644	0.59399636	1.04792463	0.80366934	20	56	36
100527943	NM_001199535	TGIF2-C20orf24	TGIF2-C20orf24 readthrough	1.457001873	1.460907986	0.466149	0.695074	0.651458	0.59399636	0.80196545	0.80366934	20	39	36
100996419	NR_134283	LOC100996419	uncharacterized LOC100996419	1.079911082	1.460779365	1.409309	1.523287	1.939807	1.59082596	1.66595383	1.97845985	79	114	142
9253	NM_001289979,NM_001289980	NUMBL	NUMB like, endocytic adaptor pro	-2.596754112	1.460778686	7.611	2.931349	10.442861	4.77815653	3.48283341	5.30826105	1038	534	1865
100287042	NR_036520	LOC100287042	uncharacterized LOC100287042	1.093337916	1.460547464	1.280551	1.408373	1.764748	2.09095564	2.19057252	2.52685524	128	187	230
5098	NM_002588,NM_032402,NM_03	PCDHGC3	protocadherin gamma subfamily	1.155558502	1.460295716	5.639263	5.994867	7.512331	4.62871952	4.82945947	5.15653336	932	1439	1674
4520	NM_005955	MTF1	metal regulatory transcription fac	1.224164909	1.460244953	2.185738	2.681805	3.002155	4.16906145	4.44613902	4.68984427	667	1091	1198
9372	NM_004799,NM_007324	ZFYVE9	zinc finger FYVE-type containing	1.178783062	1.460120929	1.774915	2.135192	2.438767	3.32067256	3.53596345	3.82065902	353	556	634
649446	NR_024101	DLGAP1-AS1	DLGAP1 antisense RNA 1	1.153144274	1.45999764	7.731549	8.92532	10.615346	3.08551061	3.26839621	3.57706795	294	453	528
4217	NM_005923	MAP3K5	mitogen-activated protein kinase	-1.298978885	1.459489626	7.222366	5.565835	9.908095	5.23078096	4.86481307	5.76413316	1435	1476	2576
51719	NM_001130849,NM_001130850	CAB39	calcium binding protein 39	1.04413774	1.45945809	30.922276	32.321119	42.548841	6.86211879	6.92390793	7.40365332	4528	6317	8128
23463	NM_012405	ICMT	isoprenylcysteine carboxyl methy	1.037614427	1.459368	21.062243	21.879498	28.884554	6.64026642	6.69301391	7.18106348	3877	5375	6959
10549	NM_006406	PRDX4	peroxiredoxin 4	1.330026882	1.45931383	33.51572	44.621124	45.950165	4.95679582	5.35670952	5.4874321	1180	2097	2118
83478	NM_001025616,NM_001042669	ARHGAP24	Rho GTPase activating protein 2	1.511205634	1.459274355	1.153821	1.775593	1.58195	2.63728034	3.15255179	3.10785943	205	414	368
80224	NM_001201573,NM_001201574	NUBPL	nucleotide binding protein like	1.293619268	1.459262288	4.419136	5.670138	6.057862	3.81159508	4.15956116	4.32422401	512	885	919
22803	NM_001317960,NM_012255	XRN2	5'-3' exoribonuclease 2	1.637503924	1.459141123	20.299643	33.340313	27.898759	6.12615526	6.82961202	6.66477934	2703	5914	4851
85027	NM_032947	SMIM3	small integral membrane protein	-1.169520908	1.459013672	3.37484	2.89209	4.634624	3.11973375	2.92160616	3.61169701	302	345	542
57085	NM_001040194,NM_001040195	AGTRAP	angiotensin II receptor associate	1.371408146	1.458951855	27.743715	37.97805	38.180728	5.05402149	5.49790253	5.58526687	1265	2318	2270
55784	NM_001159643,NM_001159644	MCTP2	multiple C2 and transmembrane	-1.505427243	1.458703187	2.46474	1.629759	3.21126	4.07407972	3.52644784	4.59165471	622	552	1116
6337	NM_001038,NM_001159575,NM	SCNN1A	sodium channel epithelial 1 alpha	-1.218573198	1.458432846	78.008787	63.497537	106.738989	8.0313311	7.74733971	8.5740206	10232	11219	18354
23035	NM_001289003,NM_015020	PHLPP2	PH domain and leucine rich repe	1.28261467	1.4584177З	1.846824	2.370177	2.52912	3.99562384	4.33469951	4.5113985	587	1006	1053
101927851	NR_125945	LOC101927851	uncharacterized LOC101927851	1.047302025	1.45835299	0.276998	0.287781	0.388207	0.3273552	0.3412894	0.45708734	10	14	18
100507557	NR_038244,NR_038245,NR_03	LOC100507557	uncharacterized LOC100507557	-6.301587867	1.45835299	0.063572	0.009555	0.08629	0.3273552	0.05398282	0.45708734	10	2	18
208	NM_001243027,NM_001243028	AKT2	AKT serine/threonine kinase 2	-1.090501404	1.458174679	20.738663	19.091861	28.347683	6.74401371	6.62023634	7.28395409	4169	5108	7477
7026	NM_001145155,NM_001145156	NR2F2	nuclear receptor subfamily 2 grou	1.44909641	1.458054954	22.778623	32.395566	29.453599	6.61462121	7.14521738	7.15404705	3808	7373	6829
9692	NM_001256678,NM_001256679	KIAA0391	KIAA0391	1.255755079	1.45780348	9.47151	16.903036	16.087419	4.6997269	5.01696186	5.2260334	981	1646	1759
140564	NM_152426	APOBEC3D	apolipoprotein B mRNA editing e	1.175812632	1.457515232	0.656911	0.769039	0.894841	1.38110603	1.52971203	1.73991364	63	99	113
2935	NM_001130006,NM_001130007	GSPT1	G1 to S phase transition 1	2.131762457	1.457463805	14.832801	31.650438	20.304941	6.70439259	7.78909605	7.24351633	4055	11550	7269
10730	NM_001253866,NM_014263,NM	YME1L1	YME1 like 1 ATPase	1.186911728	1.457412214	31.003829	37.283692	42.728489	7.01455582	7.26001507	7.55446864	5037	7988	9029
93	NM_001106	ACVR2B	activin A receptor type 2B	-1.200862515	1.457276088	1.040011	0.867331	1.426566	3.64828273	3.40707821	4.15509812	453	504	812
100129716	NR_027435	ARRDC3-AS1	ARRDC3 antisense RNA 1	1.45142249	1.457201407	0.355095	0.514122	0.486533	0.89996265	1.1751656	1.17840727	34	66	61
57724	NM_020964	EPG5	ectopic P-granules autophagy pro	-1.249863753	1.457149176	2.656455	2.127974	3.637902	5.08141332	4.7702214	5.61116998	1290	1379	2312
80700	NM_001171091,NM_025241	UBXN6	UBX domain protein 6	1.109060842	1.457082887	23.321937	25.902423	31.949822	5.47588426	5.62204021	6.0087914	1708	2531	3061
9546	NM_004886	APBA3	amyloid beta precursor protein bi	1.143973784	1.456857627	7.224184	8.266553	9.891512	4.01620928	4.19902895	4.53091599	596	911	1068
146664	NM_001199172,NM_144677,NM	MGAT5B	mannosyl (alpha-1,6-)-glycoprote	-2.254605049	1.456139054	1.302355	0.562076	1.772505	2.64317467	1.73367602	3.11132089	206	122	369
7384	NM_003365	UQCRC1	ubiquinol-cytochrome c reductas	1.306063999	1.4560793	45.185741	59.080074	61.822853	6.17274361	6.55328797	6.70857291	2793	4874	5002
3588	NM_000628	IL10RB	interleukin 10 receptor subunit be	-1.018398298	1.455329496	11.234526	11.052998	15.372296	4.49006587	4.46494141	5.01124268	843	1106	1509
78991	NM_001301054,NM_001301057	PCYOX1L	prenylcysteine oxidase 1 like	1.171000991	1.45530675	12.615637	14.948624	17.40952	5.02384223	5.24511195	5.55126453	1238	1937	2216
29940	NM_001080976,NM_001322937	DSE	dermatan sulfate epimerase	1.314237879	1.455056835	10.773309	14.043291	14.681896	5.43908863	5.82536371	5.96975643	1664	2922	2978
157769	NM_001317917,NM_001317918	FAM91A1	family with sequence similarity 9	1.181140063	1.454970433	22.262645	27.183598	30.443998	6.81328826	7.05150499	7.35027257	4376	6906	7831
114659	NM_001321350,NM_052888	LRRC37B	leucine rich repeat containing 37	-1.131955118	1.454613403	3.670098	3.126212	4.922478	3.56999691	3.40707821	4.07226969	427	504	764
284454	NR_036515	LOC284454	uncharacterized LOC284454	-1.121233675	1.454434039	20.463827	18.271595	27.96859	5.18403815	5.02374123	5.71207875	1388	1654	2483
100505894	NR_039993,NR_039994,NR_03	TMEM161B-AS1	TMEM161B antisense RNA 1	1.140369353	1.454295619	4.030142	5.102007	6.944267	2.24618091	2.3978615	2.68852679	147	224	263
26205	NM_012384	GMEB2	glucocorticoid modulatory elemer	1.174241788	1.454184424	7.382655	8.675209	10.087205	4.99301815	5.218028	5.51904993	1211	1900	2166
55654	NM_001193304,NM_017849	TMEM127	transmembrane protein 127	1.459551179	1.453913158	9.645112	14.05403	13.139883	5.45760375	5.9927829	5.98728816	1686	3288	3015
105376306	NR_135119	LOC105376306	uncharacterized LOC105376306	1.318710559	1.45359496	1.757935	2.3454	2.433892	0.97665845	1.18729361	1.26797492	38	67	68
64089	NM_001348189,NM_022133,NM	SNX16	sorting nexin 16	-1.18612343	1.453466088	3.543825	3.063737	4.886876	3.61867338	3.39405638	4.12116227	443	499	792
84418	NM_032412	CYSTM1	cysteine rich transmembrane mo	-1.524836446	1.453441561	61.996189	40.690575	84.681221	5.80583878	5.21061989	6.33726668	2157	1890	3856
26052	NM_001136127,NM_001278252	DNM3	dynamin 3	-1.335715559	1.453414473	0.411443	0.394527	0.829234	2.00201503	1.70021226	2.42483178	118	118	211
285598	NM_001317948,NM_173664	ARL10	ADP ribosylation factor like GTPa	3.538161119	1.453327047	0.051219	0.220211	0.098026	0.17294258	0.54351856	0.24657076	5	24	9
105372483	NR_136527	LOC105372483	uncharacterized LOC105372483	2.365758913	1.453327047	0.143213	0.409891	0.23691	0.17294258	0.38406669	0.24657076	5	16	9
407018	NR_029501	MIR27A	microRNA 27a	-1.110794961	1.453327047	1.401378	1.28886	2.288578	0.17294258	0.15624221	0.24657076	5	6	9
4886	NM_000909	NPY1R	neuropeptide Y receptor Y1	-1.110794961	1.453327047	0.035552	0.03976	0.054059	0.17294258	0.15624221	0.24657076	5	6	9
55201	NM_001308363,NM_018174	MAP1S	microtubule associated protein 1	-1.002422648	1.453147253	11.928735	11.916874	16.283302	5.30608931	5.3026865	5.83388654	1514	2018	2706
139322	NM_198450	APOOL	apolipoprotein O like	-1.264551834	1.453085562	5.073549	4.016765	6.930665	5.04291747	4.71579131	5.56836587	1255	1326	2243

23245	NM_001184734,NM_001184735	ASTN2	astrotactin 2	1.456696507	1.452969024	0.658697	1.727682	2.158781	1.9554003	2.37683491	2.37386535	113	220	202
6603	NM_001098426,NM_001330439	SMARCD2	SWI/SNF related, matrix associat	-1.035882332	1.452737861	51.025224	49.308742	69.775193	7.11499574	7.06450799	7.65053232	5403	6969	9654
89953	NM_001289034,NM_001289035	KLC4	kinesin light chain 4	-1.118529075	1.45238329	4.421329	4.804089	7.187744	3.70573844	3.55714683	4.20940382	473	565	845
63939	NM_001190826,NM_001190827	FAM217B	family with sequence similarity 21	1.036554959	1.452302886	2.274528	2.313955	3.04641	3.5668994	3.61440811	4.06693155	426	590	761
10565	NM_006421	ARFGEF1	ADP ribosylation factor guanine r	-1.125220369	1.452018216	9.098251	8.093029	12.415994	6.02256521	5.85512633	6.55371946	2513	2984	4488
113177	NM_001031735,NM_001039846	IZUMO4	IZUMO family member 4	1.316792609	1.451975329	1.941328	2.589856	2.701624	1.54118428	1.81408935	1.91649468	75	132	134
3074	NM_000521,NM_001292004	HEXB	hexosaminidase subunit beta	1.175985782	1.451955277	72.401802	85.198363	98.733732	7.07712516	7.30940035	7.6118021	5262	8268	9397
498	NM_001001935,NM_001001937	ATP5A1	ATP synthase, H+ transporting, r	1.279708294	1.451783175	98.652628	127.529987	135.693626	7.56187068	7.9160204	8.09732548	7379	12617	13176
105376805	NR_135056,NR_135057,NR_135	LOC105376805	uncharacterized LOC105376805	-1.229319358	1.451380358	13.120034	9.855807	16.825475	2.71768687	2.46913115	3.18544464	219	238	391
127428	NM_153035,NR_130900	TCEANC2	transcription elongation factor A N	1.223325864	1.451342156	1.402252	1.685981	1.863619	3.16140171	3.42255083	3.64789978	312	510	557
1429	NM_001130042,NM_001130043	CRYZ	crystallin zeta	-1.243289189	1.451233708	21.001322	16.755249	28.824493	5.5459881	5.2392989	6.07366218	1795	1929	3204
55771	NM_018304	PRR11	proline rich 11	-1.0452789	1.45088702	11.589245	11.098998	15.80252	6.21045	6.14744153	6.74133532	2868	3666	5118
83667	NM_031459	SESN2	sestrin 2	1.3310212	1.450880932	7.293379	9.718954	9.943759	4.70396282	5.10270203	5.22363661	984	1750	1756
23255	NM_015210	MTCL1	microtubule crosslinking factor 1	-2.55654778	1.450638339	13.869834	5.42975	18.907417	6.379754	5.05220335	6.91106522	3230	1688	5763
10994	NM_006844	ILVBL	ilvB acetolactate synthase like	1.338436491	1.450569015	21.36458	28.632914	29.124683	5.62926888	6.04244873	6.15683176	1904	3405	3397
1164	NM_001827	CKS2	CDC28 protein kinase regulatory	-1.016541074	1.450484295	230.048187	226.55481	313.564819	7.11870225	7.09520497	7.65201852	5417	7120	9664
80318	NM_001135953,NM_025211	GKAP1	G kinase anchoring protein 1	1.18855812	1.450407849	2.251941	2.633049	3.013375	2.29192162	2.49375478	2.73414401	153	243	273
10520	NM_001265597,NM_001265598	ZNF211	zinc finger protein 211	1.086799993	1.450369357	9.099129	9.637283	11.93736	5.0976022	5.2143287	5.62091677	1305	1895	2328
5570	NM_001270393,NM_001270394	PKIB	cAMP-dependent protein kinase	1.481688341	1.450243012	1.400379	2.185315	1.929529	1.84721644	2.278228	2.2537152	102	202	182
79577	NM_024529	CDC73	cell division cycle 73	1.358014664	1.450118073	7.833779	10.651875	10.67771	5.53334846	5.96663422	6.05984481	1779	3228	3173
100128640	NR_028389	ACVR2B-AS1	ACVR2B antisense RNA 1	2.320446056	1.450074532	0.284499	0.670162	0.395329	0.77668602	1.41062362	1.02535867	28	87	50
4935	NM_000273	GPR143	G protein-coupled receptor 143	-7.30206498	1.450074532	0.426966	0.04623	0.577841	0.77668602	0.13134883	1.02535867	28	5	50
1856	NM_004422	DVL2	dishevelled segment polarity prot	1.233620986	1.449996355	6.923184	8.557851	9.441629	4.4162598	4.706341	4.93124635	799	1317	1425
79991	NM_024928	STN1	STN1, CST complex subunit	-1.688749331	1.449824339	4.096601	2.427894	5.580179	4.74702075	4.02750389	5.26618109	1015	803	1810
2948	NM_000850,NM_147148,NR_02	GSTM4	glutathione S-transferase mu 4	1.385058276	1.449625828	1.361178	1.928005	1.882366	1.5285023	1.85267538	1.90057841	74	137	132
51566	NM_016607,NM_177947,NM_17	ARMCX3	armadillo repeat containing, X-lin	1.146000318	1.449625655	7.581981	8.682945	10.294115	4.6940596	4.88356843	5.21239866	977	1496	1742
10682	NM_006579	EBP	emopamil binding protein (sterol	1.005416436	1.449343082	39.821747	40.097858	54.248848	5.51979639	5.52742062	6.04544154	1762	2367	3141
768211	NM_001085399,NM_001085400	RELL1	RELT like 1	1.076997156	1.449318299	3.435415	3.695188	4.933011	3.6420927	3.741163	4.14161942	451	649	804
54101	NM_020639	RIPK4	receptor interacting serine/threon	1.330011388	1.449122601	19.214993	25.582573	26.166414	6.19849084	6.60506016	6.72758668	2844	5054	5069
122416	NM_001348651,NM_001348652	ANKRD9	ankyrin repeat domain 9	-1.095818909	1.448886351	3.895667	3.575132	5.292054	4.26583542	4.14097459	4.77742372	716	873	1276
54681	NM_177938,NM_177939	P4HTM	prolyl 4-hydroxylase, transmembr	-1.165740608	1.44873736	16.273617	13.983509	22.422826	5.14302819	4.92851423	5.66515841	1348	1545	2402
10773	NM_006626	ZBTB6	zinc finger and BTB domain cont	1.102363251	1.448656881	4.352134	4.804218	5.925873	4.20138033	4.33469951	4.71167259	683	1006	1217
8559	NM_003675	PRPF18	pre-mRNA processing factor 18	1.241489919	1.448431349	8.485837	10.563623	11.566102	3.91752664	4.21095882	4.42223881	554	919	987
5962	NM_001260492,NM_001260493	RDX	radixin	-1.00173445	1.448337731	16.389105	16.396235	22.24062	6.17833593	6.17587027	6.70657186	2804	3740	4995
2306	NM_004474	FOXD2	forkhead box D2	-1.332724173	1.448286768	0.07502	0.056375	0.102719	0.43999062	0.3412894	0.60191771	14	14	25
4056	NM_145867	LTC4S	leukotriene C4 synthase	1.440045618	1.448286768	0.539792	0.791254	0.729782	0.43999062	0.59904473	0.60191771	14	27	25
26994	NM_014372	RNF11	ring finger protein 11	1.377455744	1.448029351	14.88023	20.523634	20.250483	5.50934646	5.96267072	6.03363206	1749	3219	3115
489	NM_005173,NM_174953,NM_17	ATP2A3	ATPase sarcoplasmic/endoplasm	-2.036743969	1.447961708	0.461362	0.225302	0.6324	1.6852613	1.06116576	2.07380058	87	57	155
57532	NM_020772	NUFIP2	NUFIP2, FMR1 interacting protei	1.044491156	1.447553083	27.996826	29.275393	38.084625	8.21716082	8.27975514	8.74928168	11644	16250	20731
205327	NM_153689	C2orf69	chromosome 2 open reading fran	-1.061505851	1.447529572	6.449646	6.08844	8.777884	4.61078387	4.52828878	5.12605789	920	1158	1638
26470	NM_001114099,NM_001114100	SEZ6L2	seizure related 6 homolog like 2	1.625518814	1.447346769	15.417452	25.749297	21.149217	5.7597863	6.45043212	6.28496741	2088	4535	3717
80148	NM_001146343,NM_001146345	PQLC1	PQ loop repeat containing 1	-1.147334235	1.447209964	17.250248	15.081072	23.68036	5.41947445	5.22613324	5.94232417	1641	1911	2921
100093631	NR_003580,NR_003580_dup1	GTF2IP4	general transcription factor IIi pse	-1.367814222	1.446864924	30.420156	22.263088	41.357071	6.76409988	6.31708818	7.29293658	4228	4130	7524
51271	NM_001171201,NM_001171202	UBAP1	ubiquitin associated protein 1	1.411959926	1.446816918	12.038358	17.026833	16.321641	5.04959013	5.5345584	5.56899537	1261	2379	2244
10285	NM_005871	SMNDC1	survival motor neuron domain co	1.107841178	1.446642916	18.576982	20.603561	25.251289	5.23664058	5.38067227	5.75752106	1441	2133	2564
201931	NM_001100389	TMEM192	transmembrane protein 192	1.308064119	1.446601837	3.322576	4.347679	4.517354	3.95828615	4.32375395	4.46209528	571	998	1016
27020	NM_001161363,NM_001161364	NPTN	neuroplastin	1.380623738	1.446363541	45.858426	62.983807	61.984717	6.57199855	7.03314362	7.09975487	3696	6818	6575
51199	NM_016350,NM_020921,NM_18	NIN	ninein	1.131666296	1.446249862	5.268278	5.776338	9.620043	5.63223546	5.80730511	6.15557498	1908	2885	3394
90780	NM_138300	PYGO2	pygopus family PHD finger 2	-1.014336245	1.446117358	12.261703	12.104173	16.669271	5.28456677	5.26455989	5.8053204	1491	1964	2652
7841	NM_001146158,NM_006302	MOGS	mannosyl-oligosaccharide glucos	1.349185992	1.446108003	31.548319	42.949352	42.991187	6.49097293	6.91891626	7.01820583	3492	6295	6211
4041	NM_001291902,NM_002335	LRP5	LDL receptor related protein 5	1.337223693	1.44597625	25.238364	33.794855	34.304446	6.99797708	7.41437652	7.52654553	4979	8896	8855
5782	NM_001131008,NM_001131009	PTPN12	protein tyrosine phosphatase, no	-2.025170762	1.445777661	46.531249	23.033043	63.194077	7.19942618	6.19138345	7.72825082	5731	3781	10191
54823	NM_001105518,NM_017673	SWT1	SWT1, RNA endoribonuclease ho	1.030604199	1.445754689	0.826906	0.85477	1.138915	2.03825186	2.07130288	2.45783582	122	168	217
8743	NM_001190942,NM_001190943	TNFSF10	TNF superfamily member 10	2.352842212	1.445566033	3.844954	8.99683	5.245399	3.04155747	4.1718205	3.51830855	284	893	505
7150	NM_003286	TOP1	topoisomerase (DNA) I	1.422724859	1.445022033	51.233433	72.973015	69.573601	7.5501545	8.0565285	8.07888022	7319	13913	13008
9328	NM_001122823,NM_001286709	GTF3C5	general transcription factor IIIC su	1.107755562	1.444890247	34.00018	37.636775	46.073986	6.33993461	6.48584542	6.86541598	3141	4649	5582
9528	NM_001305043,NM_001305049	TMEM59	transmembrane protein 59	1.143872874	1.444467276	17.48663	21.784852	24.765885	6.7889037	6.98119295	7.31543251	4302	6575	7643
56949	NM_020196	XAB2	XPA binding protein 2	1.042391089	1.444288661	30.22447	31.539959	41.02713	6.30643285	6.36559004	6.83118973	3068	4273	5450
25836	NM_015384,NM_133433	NIPBL	NIPBL, cohesin loading factor	1.14904847	1.444128692	4.040563	4.670092	5.520836	5.35987438	5.55576227	5.87925987	1573	2415	2794
10380	NM_001286149,NM_001286150	BPNT1	3'(2'), 5'-bisphosphate nucleotida	1.060205078	1.443951804	9.510156	10.382801	12.915348	4.56959435	4.65049289	5.08086917	893	1265	1586
3783	NM_002250	KCNN4	potassium calcium-activated chai	-1.470771295	1.443902182	70.406776	47.920124	95.535179	7.27207275	6.71987533	7.79918158	6029	5477	10707
2771	NM_001166425,NM_001282617	GNAI2	G protein subunit alpha i2	1.384957844	1.443265176	39.038511	54.163499	52.834595	6.44067162	6.90590237	6.96491258	3371	6238	5984
727758	NR_033770	ROCK1P1	Rho associated coiled-coil contai	1.681183577	1.443215087	2.411672	4.060842	3.268843	2.76169834	3.42255083	3.22432601	227	510	403
25960	NM_032777	ADGRA2	adhesion G protein-coupled rece	-1.333473374	1.44266673	0.07787	0.058441	0.105905	0.54446555	0.42561204	0.73352601	18	18	32
103724388	NR_125772	LINC01068	long intergenic non-protein codin	1.039258885	1.44266673	0.727963	0.747525	0.988585	0.54446555	0.56226574	0.73352601	18	25	32
64215	NM_022365	DNAJC1	DnaJ heat shock protein family (H	1.329250636	1.442623296	14.740993	19.621479	19.991293	4.95561208	5.35469454	5.47001198	1179	2094	2092
5127	NM_001170460,NM_006201,NM	CDK16	cyclin dependent kinase 16	1.270652345	1.442598303	24.434501	31.263476	33.183282	6.27023148	6.61182485	6.79317142	2991	5078	5307
65056	NM_001127235,NM_001127236	GPBP1	GC-rich promoter binding protein	1.001927038	1.442296661	29.45698	29.389639	40.951553	7.04854109	7.05129764	7.57357268	5158	6905	9150
26207	NM_012417,NM_181671	PITPNC1	phosphatidylinositol transfer prot	-1.153846939	1.441565956	1.014524	0.885834	1.378421	2.86618866	2.68977954	3.33208871	247	286	438
51026	NM_016072	GOLT1B	golgi transport 1B	-1.393174798	1.441263156	18.147728	13.038939	24.582024	5.87822053	5.40942966	6.3980468	2270	2177	4024
163259	NM_001256404,NM_198459	DENND2C	DENN domain containing 2C	1.257394536	1.441188591	0.560294	0.710052	0.759638	1.79522562	2.03820547	2.18966672	97	163	172
5784	NM_005401	PTPN14	protein tyrosine phosphatase, no	1.054464939	1.440469221	3.672531	3.878055	4.972683	5.61734115	5.69233762	6.13489105	1888	2660	3345
257364	NM_001318146,NM_153271	SNX33	sorting nexin 33	1.059637571	1.440406033	6.336478	6.729249	8.609793	4.39021014	4.46991427	4.89555644	784	1110	1389
100505641	NR_046251,NR_046252,NR_046	FGD5-AS1	FGD5 antisense RNA 1	1.083566454	1.439836791	13.996374	15.357661	18.967084	5.71990486	5.83358571	6.23744823	2030	2939	3595
3693	NM_002213	ITGB5	integrin subunit beta 5	2.165980375	1.43977791	33.595047	72.848274	45.453453	7.17267734	8.28231923	7.69547477	5625	16279	9961
2643	NM_000161,NM_001024024,NM	GCH1	GTP cyclohydrolase 1	1.151819244	1.439574816	3.97792	4.332748	5.193672	3.51641288	3.70365887	4.00314859	410	631	726
5257	NM_000293,NM_001031835	PHKB	phosphorylase kinase regulatory	-1.325059348	1.439544902	12.886264	9.787693	17.45926	6.15531992	5.75580975	6.67475675	2759	2782	4885
9454	NM_001145721,NM_001145722	HOMER3	homer scaffolding protein 3	-1.146216062	1.439247011	12.051492	10.844257	16.866391	4.4636621	4.276286	4.9689398	827	964	1464
374920	NM_199341	C19orf68	chromosome 19 open reading fra	1.142850119	1.439224781	3.771526	4.317801	5.102972	3.82721276	4.00712306	4.32123795	518	791	917
2012	NM_001423	EMP1	epithelial membrane protein 1	-1.453530639	1.438983804	313.12973	215.660995	423.428802	9.74123325	9.20243398	10.26577074	33562	30851	59400
60313	NM_021639	GPBP1L1	GC-rich promoter binding protein	-1.019336996	1.438943307	31.95051	31.381968	43.206398	6.87407247	6.84667854	7.3953363	4566	5985	8081
9517	NM_004863	SPTLC2	serine palmitoyltransferase long c	-1.135476545	1.438818498	17.025904	15.013245	23.022104	7.09120758	6.90933848	7.6128709	5314	6253	9404
12	NM_001085	SERPINA3	serpin family A member 3	3.787324545	1.438580807	2.066967	7.854319	2.794424	2.07360076	3.71835988	2.4901017	126	638	223
10238	NM_005828,NR_073585	DCAF7	DDB1 and CUL4 associated facto	-1.069650127	1.43851687	30.899369	28.715861	37.982996	7.32276228	7.22624911	7.84460255	6246	7802	11051
84313	NM_032353	VPS25	vacuolar protein sorting 25 homo	1.303127544	1.437989783	48.31934	63.042816	65.304329	5.72408203	6.09971488	6.2398222	2036	3545	3601
6892	NM_003190,NM_172208,NM_17	TAPBP	TAP binding protein	-1.007800163	1.437930276	43.90058	43.814398	59.9189	7.30730079	7.29616205	7.82852566	6179	8192	10928
84669	NM_032582	USP32	ubiquitin specific peptidase 32	1.155098566	1.437927152	8.413856	9.728488	11.370461	5.87195953	6.07667303	6.38844962	2260	3488	3997
57048	NM_001201576,NM_020360	PLSCR3	phospholipid scramblase 3	1.485967864	1.437751759	9.702815	14.29378	12.999123	4.34752691	4.89562996	4.84966451	760	1509	1344
441263	NR_023383	DTX2P1-UPK3BP1-PMS2P11	DTX2P1-UPK3BP1-PMS2P11 re	-2.198886003	1.437742536	9.870119	4.493092	13.346922	4.16497021	3.12128662	4.66414974	665	404	1176
11163	NM_001301022,NM_001301023	NUDT4	nudix hydrolase 4	1.077699385	1.437656158	11.531499	12.444482	15.583731	5.78332993	5.88940006	6.29907501	2123	3057	3754
79873	NM_024815	NUDT18	nudix hydrolase 18	1.144877298	1.437641788	2.296944	2.632715	3.117144	2.14180175	2.29513766	2.56269567	134	205	237
3574	NM_000880,NM_001199886,NM	IL7	interleukin 7	-4.312792673	1.437531367	0.655167	0.147333	0.879423	1.21691895	0.38406669	1.53866543	52	16	92
56254	NM_019592	RNF20	ring finger protein 20	1.364435475	1.437528733	12.1849	16.65085	16.46777	5.5786497	6.01889027	6.09304946	1837	3349	3248
113452	NM_001329722,NM_001329723	TMEM54	transmembrane protein 54	-1.178425666	1.437371796	40.959813	34.833651	55.207075	5.46011023	5.22906934	5.97356517	1689	1915	2986
1954	NM_001271938,NM_001410	MEGF8	multiple EGF like domains 8	1.289114549	1.437338727	4.228386	5.456899	5.71022	5.553832	5.913327	6.0678839	1805	3109	3191
11238	NM_007220	CA5B	carbonic anhydrase 5B	-1.028153541	1.437331406	0.893926	0.873684	1.21111	2.64904502	2.61543261	3.10091148	207	269	366
84168	NM_018153,NM_032208,NM_05	ANTXR1	anthrax toxin receptor 1	2.327004871	1.43730313	5.945807	12.549122	6.862105	4.57422946	5.75784426	5.07910255	896	2786	1584
9227	NM_001301645,NM_004744	LRAT	lecithin retinol acyltransferase (ph	1.027606516	1.437260581	2.779577	2.864383	3.611089	3.79050499	3.82699692	4.28184606	504	692	891
8357	NM_003536	HIST1H3H	histone cluster 1 H3 family memb	1.878271481	1.437217832	2.106132	4.044401	2.884874	0.99521185	1.52015496	1.28032816	39	98	69

55553	NM_001145811,NM_001145819	SOX6	SRY-box 6	2.0051132	1.436858458	0.199428	0.406537	0.27306	1.46336426	2.17847216	1.81825156	69	185	122
8697	NM_004661	CDC23	cell division cycle 23	1.13939116	1.436596391	18.708654	21.345438	25.265139	5.87007592	6.05532648	6.38523632	2257	3436	3988
1060	NM_001812	CENPC	centromere protein C	-1.129480148	1.436569796	11.991955	10.630397	16.196503	5.32637993	5.1553565	5.83807086	1536	1817	2714
145873	NM_001039958	MESP2	mesoderm posterior bHLH transc	-1.737613589	1.436484014	0.904408	0.521028	1.225872	1.27880673	0.86372326	1.60891398	56	43	99
84911	NM_001256838,NM_032825	ZNF382	zinc finger protein 382	-1.78021419	1.436435396	1.074482	0.601744	1.452294	1.98354948	1.41062362	2.39105499	116	87	205
5538	NM_000310,NM_001142604	PPT1	palmitoyl-protein thioesterase 1	1.387313131	1.435962234	54.814404	76.129379	74.053169	7.07303724	7.54234442	7.59180769	5247	9726	9267
4582	NM_001018016,NM_001018017	MUC1	mucin 1, cell surface associated	-20.63644048	1.435636128	153.607482	6.378678	204.227313	7.87246447	3.62112921	8.39229061	9161	593	16176
5509	NM_006242	PPP1R3D	protein phosphatase 1 regulatory	-1.253168518	1.435583458	2.735505	2.186681	3.696065	3.35699061	3.06652959	3.83538509	363	387	641
165918	NM_152617	RNF168	ring finger protein 168	1.13010537	1.435311705	11.785694	13.336304	15.896854	5.96195729	6.13575486	6.47630497	2408	3636	4251
51265	NM_001113575,NM_001300853	CDKL3	cyclin dependent kinase like 3	1.329290573	1.435294135	1.263846	1.984674	2.10343	1.76310734	2.06474386	2.14982678	94	167	166
55132	NM_001278604,NM_001350531	LARP1B	La ribonucleoprotein domain fam	-1.241227022	1.435147142	11.006281	9.251147	15.035125	4.84104841	4.54134004	5.34694775	1086	1169	1917
5567	NM_001242857,NM_001242858	PRKACB	protein kinase cAMP-activated ca	1.286935306	1.434892406	2.922816	3.80423	4.059311	3.73914466	4.07885924	4.22706077	485	834	856
2525	NM_000149,NM_001097639,NM	FUT3	fucosyltransferase 3 (Lewis blooc	-2.076325512	1.434847842	38.125713	19.407597	55.839329	6.55490324	5.51724792	7.07115648	3652	2350	6445
3975	NM_005568	LHX1	LIM homeobox 1	1.359552315	1.434792053	1.913803	2.604661	2.580188	2.88620833	3.27693362	3.34684674	251	456	443
3005	NM_005318	H1F0	H1 histone family member 0	-1.062529242	1.434745586	225.176056	212.15213	303.596497	8.99950137	8.91217458	9.51944438	20055	25219	35390
5317	NM_000299,NM_001005337	PKP1	plakophilin 1	-1.021559294	1.434725638	0.059679	0.059118	0.084146	0.41264541	0.40498891	0.56199967	13	17	23
91450	NR_026998	LOC91450	uncharacterized LOC91450	-1.23825173	1.434725638	0.34119	0.259353	0.45759	0.41264541	0.3412894	0.56199967	13	14	23
9786	NM_001244189,NM_001244190	KIAA0586	KIAA0586	1.230670802	1.434503626	2.880894	3.52187	3.863242	4.15055879	4.43473883	4.64636463	658	1082	1161
642475	NM_001100878	MROH6	maestro heat like repeat family m	-1.238693699	1.434388067	49.125896	39.702908	66.218506	7.28955943	6.98293338	7.8072075	6103	6583	10767
4430	NM_001130158,NM_001161819	MYO1B	myosin IB	1.288115979	1.434324586	22.514313	28.862704	30.535108	6.81034449	7.17273677	7.32682947	4367	7516	7704
1031	NM_001262,NM_078626	CDKN2C	cyclin dependent kinase inhibitor	-1.123279179	1.43386001	7.072199	6.072258	8.85545	3.81159508	3.65645563	4.30016067	512	609	903
9684	NM_001272036,NM_014665	LRRC14	leucine rich repeat containing 14	-1.100609314	1.433810863	10.053636	9.145903	13.550844	5.76046434	5.62482969	6.27226751	2089	2536	3684
9693	NM_001351724,NM_001351725	RAPGEF2	Rap guanine nucleotide exchang	-1.056706748	1.433701471	3.682043	3.467828	4.937851	4.73465189	4.65813802	5.23795796	1006	1272	1774
123720	NM_001080435	WHAMM	WAS protein homolog associatec	-1.504705265	1.433590014	10.2326	6.8082	13.789466	5.30050562	4.72933337	5.80905548	1508	1339	2659
583	NM_031885	BBS2	Bardet-Biedl syndrome 2	-1.156041408	1.433242406	19.749735	17.105089	26.605198	5.78466375	5.57953754	6.29603638	2125	2456	3746
9663	NM_014646	LPIN2	lipin 2	-1.024550636	1.433102151	5.218734	5.0995	7.029502	5.02947882	4.99556864	5.53524714	1243	1621	2191
60684	NM_021942,NM_199053	TRAPPC11	trafficking protein particle comple	-1.102779147	1.433102151	7.139529	6.481859	9.627117	5.02947882	4.89285546	5.53524714	1243	1506	2191
55030	NM_017943,NM_152231	FBXO34	F-box protein 34	1.052314147	1.433011136	10.984368	11.077735	14.170836	5.19009033	5.2616952	5.69718355	1394	1960	2457
340385	NM_001317936,NM_213605,NR	ZNF517	zinc finger protein 517	1.503745394	1.4324216	0.98708	1.753288	1.412432	1.83696709	2.28388661	2.22843599	101	203	178
1363	NM_001873	CPE	carboxypeptidase E	-1.079113596	1.432188645	1.447905	1.345042	1.948618	2.24618091	2.16012918	2.66986823	147	182	259
657	NM_004329	BMPR1A	bone morphogenetic protein rece	-1.20651159	1.432126701	5.187095	4.30103	6.977207	4.26774435	4.01224533	4.76319139	717	794	1263
6324	NM_001037,NM_001321605,NM	SCN1B	sodium voltage-gated channel be	1.012509039	1.431947125	0.222001	0.181902	0.235722	0.5190475	0.52452456	0.69713258	17	23	30
2764	NM_004124	GMFB	glia maturation factor beta	1.004477612	1.431479191	15.179821	15.266235	20.422743	5.9536804	5.96002232	6.46417373	2394	3213	4215
23019	NM_001265612,NM_016284,NM	CNOT1	CCR4-NOT transcription complex	1.0951089	1.431411109	25.631914	27.959754	33.435859	7.63634513	7.76679129	8.15160239	7772	11372	13683
51421	NM_001278683,NM_001278685	AMOTL2	angiomotin like 2	-1.338534106	1.431280671	34.692541	25.947787	46.662521	7.41047812	6.99268435	7.92523453	6640	6628	11689
9802	NM_001136264,NM_001136266	DAZAP2	DAZ associated protein 2	-1.125460377	1.431107075	56.512781	50.21359	75.732543	6.88965161	6.72065785	7.40312387	4616	5480	8125
140688	NM_001256798,NM_001351680	NOL4L	nucleolar protein 4 like	1.126582901	1.430772432	15.271303	16.545721	21.024483	6.57701091	6.74727013	7.08926137	3709	5583	6527
80381	NM_001024736,NM_001329628	CD276	CD276 molecule	1.606469682	1.430697384	16.710468	27.139488	22.13988	5.76046434	6.43430347	6.26917183	2089	4484	3676
83986	NM_001284497,NM_032039,NR	FAM234A	family with sequence similarity 23	1.490957355	1.430579424	20.708108	30.887089	27.818609	6.03830635	6.60731859	6.54830272	2541	5062	4471
147040	NM_001002914	KCTD11	potassium channel tetramerizatio	1.816159684	1.43039557	3.627333	6.5992	4.87549	3.56068436	4.36571317	4.03994054	424	1029	746
55090	NM_018019	MED9	mediator complex subunit 9	-1.039194069	1.430105736	4.196702	4.042831	5.646132	3.33530968	3.28542082	3.80791592	357	459	628
4097	NM_002359,NM_032711	MAFG	MAF bZIP transcription factor G	1.002340772	1.429941226	20.700766	20.74007	27.822867	6.68488089	6.6882213	7.19662649	4000	5357	7035
158358	NM_001017969	KIAA2026	KIAA2026	1.011358046	1.429550181	1.952952	1.975696	2.622195	3.8780834	3.89327789	4.36394232	538	727	946
1201	NM_000086,NM_001042432,NM	CLN3	CLN3, battenin	1.003232949	1.429544355	18.448776	18.576812	23.942685	5.05512721	5.05964438	5.55762202	1266	1697	2226
23243	NM_001195098,NM_001195099	ANKRD28	ankyrin repeat domain 28	-1.191490707	1.429518635	4.005755	3.171534	5.229514	4.72217607	4.47980886	5.22123584	997	1118	1753
23708	NM_018094	GSPT2	G1 to S phase transition 2	1.657722715	1.429410225	2.772433	4.605439	3.726929	3.11973375	3.78172178	3.58455875	302	669	531
9725	NM_014698	TMEM63A	transmembrane protein 63A	-1.137864454	1.42940068	21.610641	19.015995	29.030739	6.56541753	6.38118168	7.07625765	3679	4320	6468
10393	NM_001256706,NM_001256707	ANAPC10	anaphase promoting complex sul	-1.23888082	1.429376778	8.11432	6.381041	11.18962	3.66576097	3.38355363	4.1466887	459	495	807
84248	NM_001011537,NM_032288,NR	FYTTD1	forty-two-three domain containing	1.329592872	1.429035815	23.427205	31.248795	31.556183	6.42664313	6.83347566	6.93665568	3338	5930	5867
83591	NM_031435	THAP2	THAP domain containing 2	-1.171768993	1.42900607	0.84375	0.722209	1.139663	2.32162912	2.14154997	2.74755257	157	179	276
27075	NM_014399	TSPAN13	tetraspanin 13	-1.47049896	1.42893178	25.12532	17.104658	33.742462	5.57093993	5.02880493	6.076764	1827	1660	3211
4824	NM_001256339,NM_006167,NR	NKX3-1	NK3 homeobox 1	-107.9298953	1.428436082	1.378322	0.007656	1.962223	2.43469539	0.05398282	2.86703245	173	2	304
116115	NM_001314033,NM_133444	ZNF526	zinc finger protein 526	1.082492643	1.428002503	3.129081	3.392314	4.206641	3.29094516	3.39405638	3.76020394	345	499	606
26092	NM_001267578,NM_015602	TOR1AIP1	torsin 1A interacting protein 1	1.152481769	1.427978499	14.49401	16.722021	19.451372	5.86251679	6.06398526	6.36906159	2245	3457	3943
100652772	NR_073113	NNT-AS1	NNT antisense RNA 1	1.219939044	1.427560543	0.936466	1.146185	1.263433	2.01115992	2.23214197	2.41366048	119	194	209
57446	NM_022477,NM_032013,NR_03	NDRG3	NDRG family member 3	-1.0466658	1.427296888	7.003887	6.778563	9.403427	4.4350683	4.37236795	4.92830565	810	1034	1422
56648	NM_020390	EIF5A2	eukaryotic translation initiation fa	1.24670277	1.427281628	1.891327	2.362834	2.537863	3.48721804	3.77972069	3.96156336	401	668	704
10078	NM_001297658,NM_001297659	TSSC4	tumor suppressing subtransferab	2.951137262	1.426935976	4.231316	12.483237	5.655073	3.05045588	4.49208242	3.51046477	286	1128	502
22872	NM_001077206,NM_001077207	SEC31A	SEC31 homolog A, COPII coat co	1.145302624	1.426720344	19.98053	23.077603	27.036607	6.40469083	6.59826361	6.91230597	3287	5030	5768
90488	NM_001319661,NM_001319662	TMEM263	transmembrane protein 263	-1.096384789	1.42662472	9.667993	8.851574	13.043113	5.06613802	4.93751728	5.56584509	1276	1555	2239
3052	NM_001122608,NM_001171991	HCCS	holocytochrome c synthase	1.686326544	1.426585713	4.081588	6.875877	5.476992	3.37126564	4.06742162	3.84165053	367	827	644
23275	NM_015227,NM_133635,NR_00	POFUT2	protein O-fucosyltransferase 2	1.306177622	1.426374756	18.09958	24.499714	25.676937	6.02426	6.40442001	6.52999085	2516	4391	4414
51203	NM_001243142,NM_001243143	NUSAP1	nucleolar and spindle associated	-1.01475154	1.426058264	62.037692	60.839064	83.874268	7.19942618	7.17844405	7.70853185	5731	7546	10052
404201	NR_015359	WDFY3-AS2	WDFY3 antisense RNA 2	1.069041285	1.426012592	0.802161	0.862145	1.087272	1.16869613	1.22307764	1.47560397	49	70	86
6049	NM_005977,NM_183043,NM_18	RNF6	ring finger protein 6	1.109328036	1.426005527	7.57567	8.320016	10.349364	4.74290957	4.88729042	5.23874944	1012	1500	1775
80267	NM_001319960,NM_025191,NR	EDEM3	ER degradation enhancing alpha	-1.086409145	1.425937284	8.219552	7.574209	11.012648	5.75707097	5.6398004	6.26101394	2084	2563	3655
10473	NM_006353	HMGN4	high mobility group nucleosomal	-1.228996489	1.425904272	18.291754	14.9018	24.524059	5.17287613	4.88449983	5.6727924	1377	1497	2415
284385	NR_122038	ZNF561-AS1	ZNF561 antisense RNA 1 (head t	2.3414446	1.425773588	0.754611	1.768795	1.007647	1.23264156	2.05815489	1.5489128	53	166	93
54467	NM_019004	ANKIB1	ankyrin repeat and IBR domain c	-1.656479142	1.425745931	14.365083	8.6819	19.25074	6.42749724	5.71030906	6.93421469	3340	2694	5857
55334	NM_001252148,NM_001252150	SLC39A9	solute carrier family 39 member 9	1.217276888	1.425688865	12.553794	15.712976	15.851385	5.91948261	6.19889074	6.42402754	2337	3801	4098
100419583	NR_125376	LOC100419583	ring finger protein 4 pseudogene	-1.466327914	1.425617106	6.298258	4.301785	8.446554	3.76640418	3.2626764	4.24607959	495	451	868
152189	NM_001320308,NM_178868	CMTM8	CKLF like MARVEL transmembra	-1.285270376	1.425607913	4.522966	3.504955	6.251935	2.62541894	2.32837375	3.06566078	203	211	356
23236	NM_015192,NM_182734	PLCB1	phospholipase C beta 1	-2.302205409	1.425568062	0.204393	0.088084	0.276517	1.29387258	0.70406985	1.61867597	57	33	100
51510	NM_001195536,NM_016410	CHMP5	charged multivesicular body prote	-1.30206029	1.425551545	35.853101	27.537355	48.017961	6.1914686	5.81660668	6.69710078	2830	2904	4962
64787	NM_022772	EPS8L2	EPS8 like 2	-1.011056415	1.425369124	90.17041	89.289848	120.785606	8.1125734	8.09676738	8.62235703	10827	14308	18981
23254	NM_001017999,NM_001018000	KAZN	kazrin, periplakin interacting prote	1.27858167	1.425359712	1.441478	1.932094	2.008025	2.73986043	3.04669644	3.18544464	223	381	391
7549	NM_001017396,NM_001282398	ZNF2	zinc finger protein 2	1.229698098	1.425315317	1.477002	1.841746	1.989624	2.76169834	3.01982084	3.20825259	227	373	398
200197	NR_027136	TMEM51-AS1	TMEM51 antisense RNA 1	-2.540548173	1.425292193	1.168212	0.459659	1.5663	3.16140171	2.04488597	3.62386582	312	164	547
51477	NM_001170938,NM_001253389	ISYNA1	inositol-3-phosphate synthase 1	-5.196738636	1.425224521	18.823964	3.756047	25.309164	5.3829543	3.14324318	5.8838209	1599	411	2803
159	NM_001126	ADSS	adenylosuccinate synthase	1.03740586	1.425155217	28.685066	29.794136	38.422928	6.29851812	6.35083931	6.8041746	3051	4229	5348
81603	NM_001345950,NM_030912,NR	TRIM8	tripartite motif containing 8	-1.280536635	1.4250192	37.120301	29.037716	49.730322	6.64651543	6.29379011	7.1532077	3894	4063	6825
3376	NM_002161,NM_013417,NR_07	IARS	isoleucyl-tRNA synthetase	1.082062931	1.424596295	41.81952	45.250115	56.093607	7.56128712	7.6744937	8.06956839	7376	10664	12924
652995	NR_015379	UCA1	urothelial cancer associated 1 (nc	-1.785630944	1.424213546	121.660103	68.204979	162.818527	8.10378397	7.2714531	8.612391	10761	8052	18850
90693	NM_138771	CCDC126	coiled-coil domain containing 126	1.005890855	1.424084826	1.313652	1.329844	1.765928	2.0648444	2.07130288	2.46867149	125	168	219
54764	NM_017580	ZRANB1	zinc finger RANBP2-type contain	-1.004501337	1.423963884	5.515891	5.496435	7.378457	4.78350351	4.77725916	5.2777797	1042	1386	1825
6667	NM_001251825,NM_003109,NM	SP1	Sp1 transcription factor	-1.375632694	1.423659713	19.608552	14.168681	25.959855	7.17623876	6.71987533	7.68288015	5639	5477	9874
64132	NM_022167,NR_110010	XYLT2	xylosyltransferase 2	1.268596694	1.423240978	29.029745	36.526734	38.303832	6.65893282	6.99914857	7.16387302	3928	6658	6876
476	NM_000701,NM_001160233,NM	ATP1A1	ATPase Na+/K+ transporting sub	1.441751954	1.423234069	107.116089	154.766795	143.257324	8.61163876	9.13833432	9.11971765	15318	29508	26814
84617	NM_001303524,NM_001303525	TUBB6	tubulin beta 6 class V	1.269751431	1.423169415	37.811744	48.341311	50.992501	6.24235022	6.58285328	6.74579644	2933	4976	5134
57184	NM_001321920,NM_001321921	FAM219B	family with sequence similarity 21	1.1367424	1.423032352	15.490609	17.669508	20.622928	5.59470734	5.77602682	6.09479909	1858	2822	3252
23161	NM_001350862,NM_001350863	SNX13	sorting nexin 13	-1.222318354	1.422625038	5.169268	4.041363	6.931506	4.94610703	4.666826	5.44073202	1171	1280	2049
55697	NM_001351157,NM_018052	VAC14	Vac14, PIKFYVE complex compo	1.486694824	1.422596916	8.691486	12.936613	11.622982	4.77413325	5.32892486	5.26695724	1035	2056	1811
54887	NM_017754	UHRF1BP1	UHRF1 binding protein 1	-1.121013851	1.422520616	2.135804	1.907462	2.85593	4.39545784	4.23890304	4.88346032	787	938	1377
51108	NM_001077180,NM_001288659	METTL9	methyltransferase like 9	1.50997197	1.422305918	17.087863	24.23507	21.439249	5.69529401	6.28040713	6.19525866	1995	4025	3490
100506826	NR_038266,NR_121654	MYLK-AS1	MYLK antisense RNA 1	2.629771009	1.421994256	1.851843	4.821541	2.478712	1.27880673	2.24959721	1.59908548	56	197	98
144108	NM_194285	SPTY2D1	SPT2 chromatin protein domain c	-1.086752568	1.42186923	8.823999	8.132503	11.79606	5.65210193	5.5345584	6.15137779	1935	2379	3384
284338	NM_199285	PRR19	proline rich 19	1.682318004	1.421764436	0.738129	1.260349	0.998443	1.08456011	1.52971203	1.37552141	44	99	77
6867	NM_001122824,NM_001146216	TACC1	transforming acidic coiled-coil cor	-1.174723444	1.421677199	7.194334	6.141161	10.352508	5.79462825	5.56682881	6.29451467	2140	2434	3742
64423	NM_001031714,NM_022489,NM	INF2	inverted formin, FH2 and WH2 dc	1.010819961	1.421641754	75.237724	79.064428	109.079095	8.3308582	8.34633642	8.83709045	12602	17020	22035

145482	NM_001146154,NM_001146155	PTGR2	prostaglandin reductase 2	-1.001225505	1.421612344	8.829495	8.90906	11.835459	4.528787	4.52709643	5.01771306	867	1157	1516
118	NM_001119,NM_001286645,NM	ADD1	adducin 1	1.221938131	1.42155141	33.065294	40.90029	44.78369	7.02897792	7.31614681	7.53317395	5088	8307	8896
27352	NM_001301849,NM_001350039	SGSM3	small G protein signaling modula	1.106731295	1.421537187	29.592293	32.610352	39.069092	6.41678441	6.56146445	6.91923454	3315	4902	5796
7433	NM_001251882,NM_001251883	VIPR1	vasoactive intestinal peptide rece	1.148581745	1.421499415	1.923361	2.029247	2.290167	2.52684435	2.69403583	2.95827541	187	287	327
1026	NM_000389,NM_001220777,NM	CDKN1A	cyclin dependent kinase inhibitor	1.40366227	1.421281915	8.261868	11.480382	11.037135	4.20138033	4.66790832	4.68520645	683	1281	1194
733	NM_000606	C8G	complement C8 gamma chain	-1.695995864	1.421152802	0.81758	0.473595	1.107068	0.77668602	0.50527715	1.01060517	28	22	49
7155	NM_001068,NM_001330700	TOP2B	topoisomerase (DNA) II beta	-1.225322082	1.420964618	14.448848	11.809484	19.294165	6.25202543	5.96311165	6.75329345	2953	3220	5161
55768	NM_001145293,NM_001145294	NGLY1	N-glycanase 1	1.297322605	1.420707859	5.334198	6.771162	6.847835	3.77716539	4.12844917	4.25236388	499	865	872
125058	NM_001271844,NM_001271845	TBC1D16	TBC1 domain family member 16	1.331671635	1.420704811	2.051605	2.583209	2.591213	4.39021014	4.78625756	4.87635737	784	1395	1370
100306951	NR_028514	PITPNA-AS1	PITPNA antisense RNA 1	1.07201235	1.420583093	5.198685	5.602614	6.982711	1.93632435	2.01116974	2.32699908	111	159	194
6899	NM_005992,NM_080646,NM_08	TBX1	T-box 1	1.121998887	1.42048205	0.279752	0.282777	0.335257	0.59399636	0.65251278	0.78645063	20	30	35
103091865	NR_111942	BRWD1-IT2	BRWD1 intronic transcript 2 (non	1.270889103	1.42048205	0.504696	0.660318	0.6755	0.59399636	0.72085408	0.78645063	20	34	35
79089	NM_001076674,NM_001330235	TMUB2	transmembrane and ubiquitin like	1.202078339	1.4204414	32.732802	39.758906	43.301541	6.22722422	6.48952306	6.72786857	2902	4661	5070
11322	NM_001127198,NM_001321185	TMC6	transmembrane channel like 6	-1.208551334	1.420126325	51.112501	42.346921	68.116354	7.17573053	6.90452564	7.67880321	5637	6232	9846
100861412	NM_001256141	FSBP	fibrinogen silencer binding protei	-1.065825778	1.419767254	1.353538	1.273376	1.806289	3.02359429	2.94322232	3.47597349	280	351	489
4524	NM_001330358,NM_005957	MTHFR	methylenetetrahydrofolate reduct	-1.026012618	1.419663798	3.071947	2.9983	4.101495	4.49659199	4.46120049	4.98317903	847	1103	1479
54507	NM_001288607,NM_001288608	ADAMTSL4	ADAMTS like 4	-2.313441387	1.419350318	0.781509	0.337751	1.045932	2.10810423	1.28081328	2.51121823	130	75	227
6241	NM_001034,NM_001165931	RRM2	ribonucleotide reductase regulato	1.243762437	1.419293974	66.690742	83.048741	88.952529	7.73537267	8.04875978	8.23855039	8327	13838	14536
51008	NM_001198798,NM_001198799	ASCC1	activating signal cointegrator 1 cc	-1.332038692	1.419094897	6.294985	4.699022	8.396155	4.16292023	3.77571017	4.64397663	664	666	1159
26811	NR_000015	SNORD55	small nucleolar RNA, C/D box 55	1.430658782	1.418925241	4.105857	6.034698	5.455721	0.38477185	0.52452456	0.52094563	12	23	21
693222	NR_030367	MIR637	microRNA 637	-1.230805124	1.418925241	3.02193	2.513179	4.169969	0.38477185	0.31941552	0.52094563	12	13	21
619279	NM_001033723	ZNF704	zinc finger protein 704	2.172477755	1.418925241	0.021084	0.047595	0.028352	0.38477185	0.73744528	0.52094563	12	35	21
4300	NM_001286691,NM_004529	MLLT3	MLLT3, super elongation comple	1.210592311	1.418623477	0.784373	0.954787	1.049657	2.63136183	2.86610484	3.06566078	204	330	356
121260	NM_145648	SLC15A4	solute carrier family 15 member 4	-1.411976505	1.41846358	11.857919	8.407941	15.811177	5.05402149	4.57403823	5.54551873	1265	1197	2207
3169	NM_004496	FOXA1	forkhead box A1	-2.303902025	1.418421372	12.539755	5.44996	16.718697	5.40219915	4.24181329	5.89641527	1621	940	2828
63905	NM_001003897,NM_022077	MANBAL	mannosidase beta like	-1.011937701	1.418185867	33.404555	33.242125	44.699272	5.44416182	5.4274358	5.93843194	1670	2205	2913
10210	NM_001195622,NM_005802	TOPORS	TOP1 binding arginine/serine rich	1.25702135	1.418012651	5.544466	6.721964	7.266436	4.50470835	4.8217002	4.98977621	852	1431	1486
79959	NM_001271989,NM_024899,NR	CEP76	centrosomal protein 76	1.105798784	1.417834717	5.125262	5.679752	6.875814	3.97474042	4.1110472	4.45120991	578	854	1008
333	NM_001024807,NM_005166	APLP1	amyloid beta precursor like prote	1.758186886	1.417691097	2.559196	4.514592	3.414773	2.86114003	3.58720616	3.30513776	246	578	429
2118	NM_001079675,NM_001261437	ETV4	ETS variant 4	-1.636269485	1.41766766	28.051004	16.486322	35.285629	5.65210193	4.95978213	6.14716835	1935	1580	3374
9513	NM_004860	FXR2	FMR1 autosomal homolog 2	1.026248038	1.417431748	8.63383	8.870488	11.498748	4.70677986	4.74274951	5.19374412	986	1352	1719
822	NM_001256139,NM_001256140	CAPG	capping actin protein, gelsolin like	-1.348128142	1.417339173	74.53655	56.086842	99.4783	6.65820534	6.23219837	7.15719027	3926	3891	6844
494470	NM_001256758,NM_152470,NR	RNF165	ring finger protein 165	1.705902248	1.417271387	3.120294	6.192143	3.965438	3.7336304	4.45870114	4.20455055	483	1101	842
9070	NM_001105214,NM_001261832	ASH2L	ASH2 like histone lysine methyltr	1.218826127	1.417031228	7.469835	9.121278	9.939369	4.49821892	4.77223569	4.98223411	848	1381	1478
2925	NM_005314	GRPR	gastrin releasing peptide receptor	-1.301196206	1.417028977	1.099566	0.842791	1.46431	1.9554003	1.68318474	2.34475282	113	116	197
11063	NM_001308165,NM_007017,NM	SOX30	SRY-box 30	3.152058602	1.416995366	0.063308	0.200172	0.083647	0.26755841	0.72085408	0.36729312	8	34	14
3685	NM_001144999,NM_001145000	ITGAV	integrin subunit alpha V	1.510543609	1.41677783	13.622676	20.601612	18.137051	6.55919609	7.14909797	7.05731497	3663	7393	6383
6789	NM_006282	STK4	serine/threonine kinase 4	1.042921807	1.416574437	8.571324	8.949655	11.411227	5.75707097	5.81660668	6.2516338	2084	2904	3631
10427	NM_001042734,NM_001300813	SEC24B	SEC24 homolog B, COPII coat co	1.074428652	1.416550184	10.970591	11.925825	14.59001	5.68390197	5.78553158	6.17803121	1979	2841	3448
5998	NM_001276260,NM_001276261	RGS3	regulator of G protein signaling 3	1.005655912	1.416508976	21.799132	21.815692	28.049261	5.84855467	5.856551	6.3435348	2223	2987	3873
59341	NM_001177428,NM_001177431	TRPV4	transient receptor potential cation	-1.64825043	1.416235558	3.529817	2.297727	4.973434	3.60363765	2.95745524	4.0704925	438	355	763
1287	NM_000495,NM_033380	COL4A5	collagen type IV alpha 5 chain	2.160497777	1.41612455	7.201025	15.590137	9.590833	5.54441418	6.63912276	6.03727606	1793	5176	3123
58497	NM_001303229,NM_001303242	PRUNE1	prune exopolyphosphatase 1	-1.338094456	1.415930196	5.611446	4.230222	7.73146	4.14225859	3.74936649	4.61987668	654	653	1139
253558	NM_001002257,NM_001304445	LCLAT1	lysocardiolipin acyltransferase 1	2.106065756	1.415700436	2.899169	6.480574	4.078244	3.97005831	4.99556864	4.44436461	576	1621	1003
2923	NM_005313	PDIA3	protein disulfide isomerase family	1.314016326	1.415580297	164.125092	215.900497	218.332428	8.93376863	9.32704857	9.43429834	19160	33639	33359
54910	NM_017789	SEMA4C	semaphorin 4C	1.847892242	1.415547701	10.721434	19.836294	14.261401	5.25118634	6.11962841	5.74141559	1456	3595	2535
112611	NM_001322335,NM_001322336	RWDD2A	RWD domain containing 2A	-1.179258432	1.415406317	0.954351	0.827451	1.291441	2.21486103	2.03149389	2.62213795	143	162	249
10724	NM_001142434,NM_012215	MGEA5	meningioma expressed antigen 5	-1.624325085	1.415078588	46.097677	28.349995	61.220279	7.87026746	7.17426092	8.36934509	9147	7524	15920
23362	NM_015310,NM_206909	PSD3	pleckstrin and Sec7 domain conta	1.534649417	1.414849074	3.145288	4.83156	4.180871	5.20112046	5.80533922	5.69025663	1405	2881	2445
196527	NM_001025356,NM_001142678	ANO6	anoctamin 6	1.011741542	1.414821963	13.728249	13.882913	18.091817	6.35527652	6.37191335	6.85073596	3175	4292	5525
2957	NM_001278940,NM_015859,NM	GTF2A1	general transcription factor IIA su	-6.575892671	1.414495225	4.045183	0.615653	5.341345	4.69831216	2.2611178	5.18226995	980	199	1705
3908	NM_000426,NM_001079823	LAMA2	laminin subunit alpha 2	1.260733933	1.41445038	0.145223	0.183643	0.192347	1.24819466	1.45142181	1.5590879	54	91	94
11016	NM_001130060,NM_001206682	ATF7	activating transcription factor 7	1.011091456	1.414357935	9.366862	7.923	7.869487	4.80732269	4.82267239	5.29233867	1060	1432	1844
5108	NM_001315507,NM_001315508	PCM1	pericentriolar material 1	1.013768216	1.414347133	7.327581	7.406694	9.731426	5.97077302	5.99018926	6.46417373	2423	3282	4215
79692	NM_001242797,NM_001242798	ZNF322	zinc finger protein 322	-1.03682042	1.414337392	2.105385	2.036902	2.798814	3.44397655	3.39667017	3.90484845	388	500	675
148867	NM_001144884,NM_133496	SLC30A7	solute carrier family 30 member 7	1.168314152	1.414245974	4.514405	5.359074	6.037103	5.19711926	5.41588625	5.68562013	1401	2187	2437
8528	NM_003649,NM_004032	DDO	D-aspartate oxidase	1.430167411	1.41414518	2.594604	3.799425	3.559831	2.40724866	2.83945493	2.82548777	169	323	294
26043	NM_015562	UBXN7	UBX domain protein 7	1.934708238	1.41391232	5.062653	9.808172	6.72567	4.60475543	5.52801678	5.08703534	916	2368	1593
142940	NM_139169	TRUB1	TruB pseudouridine synthase fan	1.131436092	1.413599495	3.579931	4.053969	4.755254	3.6887407	3.85387581	4.15509812	467	706	812
4291	NM_001130156,NM_001130157	MLF1	myeloid leukemia factor 1	1.982215093	1.413573435	4.208015	8.220424	5.558267	3.3316643	4.2461677	3.78858771	356	943	619
54438	NM_001242628,NM_001242629	GFOD1	glucose-fructose oxidoreductase	1.033126412	1.413497684	0.889016	0.922528	1.057348	1.74129091	1.77444288	2.10885534	92	127	160
51230	NM_016436	PHF20	PHD finger protein 20	1.116701283	1.413460799	5.316185	5.944018	7.060982	4.9767722	5.13123263	5.46257813	1197	1786	2081
387263	NM_001029863,NM_001317342	C6orf120	chromosome 6 open reading fran	1.237618391	1.413052819	4.970501	6.059761	6.494137	4.40762874	4.70212092	4.88649377	794	1313	1380
2530	NM_004480,NM_178155,NM_17	FUT8	fucosyltransferase 8	1.388341945	1.412724227	21.148927	31.678009	28.55771	6.64247505	7.11180405	7.13674198	3883	7203	6747
11135	NM_152243	CDC42EP1	CDC42 effector protein 1	-1.10252762	1.412677532	34.65834	31.472359	46.01506	6.20896051	6.07013843	6.70170058	2865	3472	4978
112616	NM_138410,NM_181472	CMTM7	CKLF like MARVEL transmembra	-1.111398799	1.41250215	60.296342	54.036105	79.02909	6.36199349	6.21156417	6.85512987	3190	3835	5542
1063	NM_016343	CENPF	centromere protein F	-1.059440551	1.41238487	32.961536	31.147749	43.750088	8.37258948	8.28954485	8.86945489	12973	16361	22536
23596	NM_014322	OPN3	opsin 3	-1.26441859	1.412138155	11.740649	9.298368	15.585361	4.95916039	4.63286465	5.44348094	1182	1249	2053
55156	NM_001286702,NM_018120	ARMC1	armadillo repeat containing 1	-1.060772959	1.4120495	16.39854	15.476336	21.759514	5.63149439	5.54814121	6.12079205	1907	2402	3312
152641	NR_024008	WWC2-AS2	WWC2 antisense RNA 2	-1.097033567	1.411993356	0.264847	0.249564	0.364836	0.66524311	0.61708849	0.87055986	23	28	40
51283	NM_001330500,NM_016561	BFAR	bifunctional apoptosis regulator	1.276989828	1.411968608	17.229469	22.140355	23.058543	5.70236863	6.04910966	6.19199306	2005	3421	3482
4200	NM_001168335,NM_002396	ME2	malic enzyme 2	1.276175909	1.411877479	5.583835	7.146468	7.606218	4.70818633	5.04805279	5.18965667	987	1683	1714
55165	NM_001127182,NM_018131	CEP55	centrosomal protein 55	-1.634735107	1.411844742	26.133616	15.858022	34.538734	6.0769238	5.38133225	6.56827536	2611	2134	4534
81857	NM_030973	MED25	mediator complex subunit 25	-1.019889649	1.411815461	19.189819	18.837801	25.460485	5.48493837	5.45716415	5.97308963	1719	2252	2985
24137	NM_012310	KIF4A	kinesin family member 4A	1.233326848	1.411657694	16.503838	20.380054	21.894707	6.19648797	6.49532689	6.6881467	2840	4680	4931
148479	NM_153812	PHF13	PHD finger protein 13	1.219253351	1.411591061	8.912915	10.884142	11.828758	5.03845181	5.31655563	5.52295387	1251	2038	2172
11173	NM_014272	ADAMTS7	ADAM metallopeptidase with thro	-2.532397424	1.411540162	0.696589	0.275052	0.929075	2.25390578	1.31437719	2.66044767	148	78	257
51094	NM_001290553,NM_001290557	ADIPOR1	adiponectin receptor 1	1.044511123	1.411462657	48.449155	50.742432	64.480361	6.69980449	6.76204243	7.19295566	4042	5641	7017
102465666	NR_106959	MIR7109	microRNA 7109	1.119455784	1.411310972	1.49911	1.641783	1.896868	0.13997292	0.15624221	0.19530369	4	6	7
729652	NR_135198	LOC729652	uncharacterized LOC729652	3.488487299	1.411310972	0.031426	0.112866	0.036743	0.13997292	0.44594451	0.19530369	4	19	7
109623460	NR_145802	SNORD138	small nucleolar RNA, C/D box 13	2.577321332	1.411310972	1.607584	4.05402	2.186364	0.13997292	0.3412894	0.19530369	4	14	7
100616116	NR_039791	MIR4648	microRNA 4648	-1.324521912	1.411310972	1.439014	0.843689	1.902073	0.13997292	0.10601836	0.19530369	4	4	7
79674	NM_001167911,NM_001167912	VEPH1	ventricular zone expressed PH do	-1.138835473	1.411200485	2.901261	2.198159	2.453683	2.78321062	2.6243808	3.21791812	231	271	401
100507373	NR_045214	LOC100507373	uncharacterized LOC100507373	-1.518911724	1.41116425	0.824619	0.537102	1.088573	1.73025772	1.33632702	2.09493548	91	80	158
6388	NM_006923,NR_045585	SDF2	stromal cell derived factor 2	1.2462607	1.410701032	38.428635	47.716982	50.482869	5.85364745	6.16632849	6.34279879	2231	3715	3871
5252	NM_002636,NM_024165,NR_02	PHF1	PHD finger protein 1	-1.179027644	1.410697423	22.107558	18.74546	29.247144	5.64771074	5.41524189	6.13574112	1929	2186	3347
11123	NM_001251977,NM_001251978	RCAN3	RCAN family member 3	-1.136366177	1.410650111	3.7967	3.582082	5.140666	3.49701545	3.3298614	3.95579862	404	475	701
3663	NM_001098627,NM_001098629	IRF5	interferon regulatory factor 5	-2.724983766	1.410650111	3.646561	1.405667	4.837818	3.49701545	2.255369	3.95579862	404	198	701
64924	NM_001251969,NM_022902,NM	SLC30A5	solute carrier family 30 member 5	-1.037995567	1.410632537	17.681665	17.122573	22.082986	5.94833435	5.8954191	6.43787878	2385	3070	4138
51382	NM_015994	ATP6V1D	ATPase H+ transporting V1 subu	1.549820197	1.410609535	21.472263	33.321762	28.476913	5.27700476	5.89588107	5.76248297	1483	3071	2573
6815	NM_001130701,NM_145251	STYX	serine/threonine/tyrosine interact	1.188397802	1.410466502	5.232915	6.224541	6.954506	4.68836995	4.92851423	5.16821322	973	1545	1688
55024	NM_001083907,NM_001127507	BANK1	B-cell scaffold protein with ankyri	-2.827774227	1.409556434	1.764925	0.660423	2.378255	2.79384765	1.63086207	3.22751932	233	110	404
148022	NM_182919	TICAM1	toll like receptor adaptor molecule	-1.130117438	1.409475286	17.129271	15.178594	22.694424	5.53651876	5.36407379	6.02264454	1783	2108	3091
387119	NM_001042475,NM_001178035	CEP85L	centrosomal protein 85 like	2.173175938	1.409417969	0.215824	0.681997	0.288647	1.39514558	2.18453503	1.72191206	64	186	111
286410	NM_001010986,NM_173694	ATP11C	ATPase phospholipid transporting	-1.061634948	1.409359432	3.750712	3.537667	4.971349	4.54619271	4.46369552	5.02323609	878	1105	1522
10001	NM_001284209,NM_001284210	MED6	mediator complex subunit 6	1.268658762	1.40930048	7.478194	9.447557	9.910226	4.19336823	4.51992154	4.66532766	679	1151	1177
79629	NM_024578	OCEL1	occludin/ELL domain containing 1	-1.589555296	1.408785834	7.067909	4.462022	9.383245	3.14071816	2.56520021	3.58704707	307	258	532
59	NM_001141945,NM_001320855	ACTA2	actin, alpha 2, smooth muscle, ac	-3.626897021	1.408524897	0.8864	0.243446	1.231152	1.33814853	0.50527715	1.65707652	60	22	104
26119	NM_015627	LDLRAP1	low density lipoprotein receptor a	1.067747186	1.408447879	7.95411	8.507977	10.532923	4.56959435	4.66031492	5.04602381	893	1274	1547

957	NM_001249,NM_001321984,NM	ENTPD5	ectonucleoside triphosphate diph	-1.003529881	1.407868277	4.656889	4.032708	5.25856	4.53672473	4.53185994	5.0121688	872	1161	1510
652965	NR_002918	SNORA48	small nucleolar RNA, H/ACA box	1.282490649	1.407504499	10.807956	13.89724	14.216808	1.27880673	1.50084877	1.58918956	56	96	97
55288	NM_001033566,NM_001033567	RHOT1	ras homolog family member T1	-1.151133907	1.407265441	10.389338	9.036198	13.780983	5.07270439	4.87609552	5.55317472	1282	1488	2219
23096	NM_001111125,NM_001243197	IQSEC2	IQ motif and Sec7 domain 2	1.083559521	1.407170129	3.920163	5.121593	3.833301	3.58232086	3.68880651	4.03994054	431	624	746
84954	NM_001159846,NM_001300862	MPND	MPN domain containing	1.142445877	1.406889658	3.955889	4.450221	5.157752	2.85607368	3.0232078	3.28994455	245	374	424
25862	NM_001286554,NM_018561	USP49	ubiquitin specific peptidase 49	1.095605114	1.406778923	2.033169	2.370202	2.775885	4.07625981	4.20052559	4.5437825	623	912	1078
1917	NM_001958	EEF1A2	eukaryotic translation elongation	2.656432931	1.406748867	94.552773	251.459183	124.990395	7.53735367	8.94200644	8.02747764	7254	25747	12551
60343	NM_001171132,NM_001171133	FAM3A	family with sequence similarity 3	1.21268366	1.406493967	16.308188	19.90566	21.573297	4.88648104	5.15612801	5.36445917	1122	1818	1941
5154	NM_002607,NM_033023	PDGFA	platelet derived growth factor sub	-2.248649429	1.40647806	5.066359	2.252441	6.689036	3.87558202	2.82400243	4.33906229	537	319	929
57522	NM_001346201,NM_020762	SRGAP1	SLIT-ROBO Rho GTPase activat	-1.088106654	1.406390271	4.068466	3.74348	5.395491	5.11890861	5.00073201	5.59889315	1325	1627	2292
9963	NM_005847,NM_152685	SLC23A1	solute carrier family 23 member 1	1.136763187	1.406327453	0.567316	0.644126	0.757986	1.21691895	1.32539385	1.51794971	52	79	90
100505681	NR_038452	NAPA-AS1	NAPA antisense RNA 1	1.295180463	1.406251882	0.589187	0.758551	0.766398	0.46682716	0.58077243	0.62147005	15	26	26
2116	NM_001300974,NM_001304549	ETV2	ETS variant 2	-1.054423872	1.406251882	0.248107	0.242497	0.335178	0.46682716	0.44594451	0.62147005	15	19	26
23576	NM_001134445,NM_001330655	DDAH1	dimethylarginine dimethylaminoh	-1.682967158	1.406011304	8.335972	5.053719	11.090231	5.06064312	4.33878278	5.53974996	1271	1009	2198
10376	NM_006082	TUBA1B	tubulin alpha 1b	1.116284867	1.405930539	761.384338	850.865662	1005.93335	10.34322251	10.50181237	10.83442805	50961	76008	88122
55194	NM_001304762,NM_018166,NR	EVA1B	eva-1 homolog B	-1.174394106	1.405847205	5.836064	4.979354	7.661467	2.71208982	2.51796519	3.13871764	218	248	377
57475	NM_020715	PLEKHH1	pleckstrin homology, MyTH4 and	1.073350471	1.405826889	4.310245	4.633121	5.696386	4.83075499	4.92941706	5.30750681	1078	1546	1864
10444	NM_006336	ZER1	zyg-11 related cell cycle regulato	1.343799514	1.405576487	9.758393	13.134261	12.894932	5.38735072	5.80484733	5.86856107	1604	2880	2773
29946	NM_013368,NM_203344	SERTAD3	SERTA domain containing 3	-1.458754719	1.40519275	22.038097	14.82598	29.096206	4.967406	4.44361342	5.44485344	1189	1089	2055
100506710	NR_036592	EBLN3P	endogenous Bornavirus-like nucl	-1.023469376	1.404873793	8.9427	8.748721	11.807992	5.28927297	5.25666827	5.76907242	1496	1953	2585
149428	NM_001159642,NM_138278	BNIPL	BCL2 interacting protein like	-1.312700322	1.404611111	1.233634	0.940059	1.677675	1.96484452	1.68318474	2.34475282	114	116	197
2036	NM_001258329,NM_001258330	EPB41L1	erythrocyte membrane protein ba	1.167471982	1.403801826	17.898165	20.05692	23.371927	6.70227682	6.92368141	7.1876368	4049	6316	6991
10193	NM_001242826,NM_005785,NM	RNF41	ring finger protein 41	1.016546329	1.403781834	2.940655	2.957695	3.820383	4.00022383	4.02243562	4.46345019	589	800	1017
84498	NM_001286379,NM_001286380	FAM120B	family with sequence similarity 12	1.3465833	1.403758744	4.137534	5.257808	5.406209	4.43846184	4.85058478	4.90854629	812	1461	1402
54834	NM_001135589,NM_017686	GDAP2	ganglioside induced differentiatio	1.264845958	1.403675183	3.296701	3.665704	3.810441	4.26009342	4.58324605	4.72754788	713	1205	1231
85458	NM_001037954,NM_001278542	DIXDC1	DIX domain containing 1	-2.315682964	1.403674949	0.392239	0.172112	0.380581	1.42282133	0.7861039	1.74883089	66	38	114
5519	NM_001177562,NM_001177563	PPP2R1B	protein phosphatase 2 scaffold su	1.218526813	1.403604981	7.516117	6.064871	7.140005	4.77815653	5.05386023	5.25213854	1038	1690	1792
51147	NM_001127582,NM_001127583	ING4	inhibitor of growth family member	1.036360043	1.403484623	14.675596	15.272466	19.355558	4.43676607	4.4859587	4.90655538	811	1123	1400
55972	NM_018843	SLC25A40	solute carrier family 25 member 4	-1.810406312	1.403434376	6.383933	3.525075	8.419185	4.36010717	3.55948145	4.82879013	767	566	1324
23047	NM_015032	PDS5B	PDS5 cohesin associated factor I	1.327051125	1.403378014	2.521631	3.348992	3.324896	4.27914521	4.66898984	4.74659308	723	1282	1248
339761	NM_001001665	CYP27C1	cytochrome P450 family 27 subfa	1.787920633	1.403344615	1.057097	1.897756	1.39597	2.54584744	3.27124763	2.96211454	190	454	328
80146	NM_001253875,NM_001253876	UXS1	UDP-glucuronate decarboxylase	1.570921176	1.403061129	28.271442	44.423595	37.206165	5.86756061	6.5101878	6.34904298	2253	4729	3888
133022	NM_152402	TRAM1L1	translocation associated membra	1.388932287	1.403038648	1.879223	2.614322	2.479604	2.23841445	2.6243808	2.63662357	146	271	252
4216	NM_001291958,NM_001301072	MAP3K4	mitogen-activated protein kinase	-1.002091603	1.403019123	6.374127	6.392007	8.488266	5.15132397	5.14839425	5.62818389	1356	1808	2340
10912	NM_006705	GADD45G	growth arrest and DNA damage i	-1.130088132	1.402991568	0.7992	0.713614	1.067379	0.88013425	0.80196545	1.12461302	33	39	57
5981	NM_001204747,NM_002913	RFC1	replication factor C subunit 1	1.166346758	1.402976804	18.428922	21.521323	24.302185	6.47412439	6.69381113	6.95796015	3451	5378	5955
5082	NM_005388	PDCL	phosducin like	1.053224223	1.402931775	7.722775	8.143745	10.182349	4.59718428	4.66898984	5.06845719	911	1282	1572
143384	NM_153810	CACUL1	CDK2 associated cullin domain 1	1.06221807	1.402632882	7.736166	8.229978	10.199302	5.69458464	5.78003646	6.17472633	1994	2830	3440
26118	NM_001348350,NM_015626,NM	WSB1	WD repeat and SOCS box contai	-1.301540501	1.402536951	42.207596	34.790284	55.144358	7.3909063	7.01326872	7.87648296	6550	6724	11299
55968	NM_001206736,NM_016143,NM	NSFL1C	NSFL1 cofactor	1.319400873	1.402464847	19.604224	26.147615	25.490279	6.09046901	6.48523156	6.57236251	2636	4647	4547
357	NM_001320663,NM_001320664	SHROOM2	shroom family member 2	-2.282934827	1.402367875	0.755343	0.260938	0.791694	2.42103729	1.5392062	2.82969649	171	100	295
548645	NM_001015882,NR_037148	DNAJC25	DnaJ heat shock protein family (H	1.503174559	1.402270475	10.091503	15.35175	13.290891	4.64202642	5.21061989	5.1131673	941	1890	1623
84876	NM_032790	ORAI1	ORAI calcium release-activated c	1.318280752	1.402243584	9.487088	12.521483	12.501131	3.89547266	4.27059745	4.35521005	545	960	940
389114	NM_001134656,NM_207404	ZNF662	zinc finger protein 662	2.704109421	1.402193628	1.839894	5.024603	2.44806	3.47073972	4.8217002	3.92071794	396	1431	683
23303	NM_015254	KIF13B	kinesin family member 13B	-1.577065586	1.402109052	5.741608	3.643019	7.565084	5.64404118	5.00330679	6.12336578	1924	1630	3318
3796	NM_001098511,NM_001243952	KIF2A	kinesin family member 2A	1.360351875	1.401989894	12.669966	17.249764	16.636334	5.66447154	6.10091747	6.14379193	1952	3548	3366
147727	NR_024333	ILF3-AS1	ILF3 antisense RNA 1 (head to h	1.03508842	1.401919411	8.112776	8.411061	10.690594	4.07843661	4.12530075	4.54121836	624	863	1076
23625	NM_001098784,NM_001098785	FAM89B	family with sequence similarity 89	1.269420751	1.401125173	16.023779	20.249022	20.98271	4.55247036	4.88356843	5.02139743	882	1496	1520
10430	NM_001242597,NM_001242598	TMEM147	transmembrane protein 147	-1.190765979	1.400895988	65.388056	55.616022	86.885651	5.86756061	5.62036393	6.34684223	2253	2528	3882
147525	NR_026849	LINC00526	long intergenic non-protein codin	-1.429350823	1.400851691	1.514072	1.05523	1.993432	1.55375576	1.23481109	1.89255394	76	71	131
2590	NM_001291866,NM_004481,NR	GALNT2	polypeptide N-acetylgalactosami	1.598400673	1.400804328	44.20904	70.954895	58.584666	7.58022887	8.2540406	8.06433186	7474	15962	12877
7267	NM_001001894,NM_001320703	TTC3	tetratricopeptide repeat domain 3	1.098216786	1.400709044	10.684524	11.846696	15.046541	6.56425305	6.69805556	7.04605492	3676	5394	6333
22858	NM_014920,NM_016513	ICK	intestinal cell kinase	-1.1781121	1.400659875	0.468383	0.39764	0.616426	1.94589385	1.77444288	2.32103228	112	127	193
5580	NM_001316327,NM_006254,NM	PRKCD	protein kinase C delta	-1.061675113	1.400516238	24.858707	22.704555	32.255739	6.06434888	5.97933194	6.54414675	2588	3257	4458
101927111	NR_109992,NR_109993	SUCLG2-AS1	SUCLG2 antisense RNA 1 (head	1.6268495	1.400281028	0.183784	0.41721	0.28768	0.35634914	0.54351856	0.47868902	11	24	19
203429	NR_026595	FAM226A	family with sequence similarity 22	1.896385267	1.400281028	0.139503	0.265918	0.18655	0.35634914	0.61708849	0.47868902	11	28	19
9480	NM_004852	ONECUT2	one cut homeobox 2	-2.302002062	1.400254533	2.412435	1.049257	3.175149	5.28079072	4.12530075	5.75586329	1487	863	2561
9021	NM_003955	SOCS3	suppressor of cytokine signaling	-1.853926818	1.399448302	33.734253	18.215723	44.367142	6.50641488	5.62928167	6.9867494	3530	2544	6076
9649	NM_001190728,NM_001190729	RALGPS1	Ral GEF with PH domain and SH	-1.042606854	1.399432464	4.363177	4.352279	5.646362	4.26200996	4.20500622	4.72529065	714	915	1229
100507002	NR_110801	LOC100507002	uncharacterized LOC100507002	-1.558144365	1.399289307	5.219489	3.358476	6.861742	2.19091668	1.71704117	2.58278312	140	120	241
100381270	NM_001174108	ZBED6	zinc finger BED-type containing 6	1.236326712	1.399058023	4.168803	5.159564	5.481317	3.94641713	4.23452662	4.40400834	566	935	974
7013	NM_003218,NM_017489	TERF1	telomeric repeat binding factor 1	-1.085542053	1.398822304	12.103677	11.167597	15.906135	5.15132397	5.03636731	5.62394919	1356	1669	2333
55958	NM_018847	KLHL9	kelch like family member 9	-1.136773583	1.398814772	4.268342	3.761469	5.614227	4.64202642	4.46494141	5.10971027	941	1106	1619
30815	NM_001286999,NM_001287000	ST6GALNAC6	ST6 N-acetylgalactosaminide alp	1.522258652	1.398804566	11.434589	17.520363	15.008152	4.80600967	5.39446891	5.27546743	1059	2154	1822
8880	NM_001303433,NM_003902,NR	FUBP1	far upstream element binding pro	1.534708192	1.398470061	53.926796	82.691768	71.450183	7.07112558	7.68535727	7.55192345	5240	10745	9013
6558	NM_001046,NM_001256461,NR	SLC12A2	solute carrier family 12 member 2	1.481889158	1.398157792	2.297654	3.412805	3.016476	4.03201954	4.57057009	4.49028463	603	1194	1037
51614	NM_015966,NM_198398	ERGIC3	ERGIC and golgi 3	1.282883456	1.398098012	144.778945	185.929851	190.230757	7.58099675	7.93872899	8.06232133	7478	12818	12859
1730	NM_006729,NM_007309	DIAPH2	diaphanous related formin 2	-1.141482459	1.397813924	2.747557	2.329205	3.701025	3.72809498	3.55246622	4.18003621	481	563	827
57706	NM_020946,NM_024820	DENND1A	DENN domain containing 1A	-1.002091503	1.397318522	5.796463	6.455164	8.581048	4.49496323	4.49208242	4.9593683	846	1128	1454
3164	NM_001202233,NM_001202234	NR4A1	nuclear receptor subfamily 4 grou	-1.098220301	1.397238223	4.682072	4.50985	6.547613	3.76370129	3.63890054	4.21584956	494	601	849
79786	NM_001303451,NM_024731	KLHL36	kelch like family member 36	-1.083729143	1.397182384	8.070519	7.433308	10.651717	5.91095768	5.79695418	6.38666534	2323	2864	3992
64403	NM_022478,NM_144985	CDH24	cadherin 24	1.384898472	1.396998671	5.590279	7.875864	7.343222	4.32753359	4.77725916	4.78935776	749	1386	1287
891	NM_031966	CCNB1	cyclin B1	1.31498547	1.396981644	136.962906	180.303391	179.801941	8.17975844	8.57361629	8.66066101	11345	19933	19493
11161	NM_007176	C14orf1	chromosome 14 open reading fra	1.109809766	1.396959583	39.112228	43.463947	51.35284	5.89869115	6.04661542	6.37411319	2303	3415	3957
1762	NM_004943	DMWD	dystrophia myotonica, WD repea	1.553979676	1.396874889	4.961283	7.726761	6.518195	4.0871111	4.69258035	4.54506286	628	1304	1079
146223	NM_178818,NM_181521	CMTM4	CKLF like MARVEL transmembra	-1.011540774	1.39685529	13.88716	13.139564	18.653024	6.62172853	6.60534266	7.09975487	3827	5055	6575
84273	NM_032313	NOA1	nitric oxide associated 1	1.016752356	1.396720317	12.610351	12.838499	16.563784	4.85762016	4.88077062	5.32550055	1099	1493	1888
6505	NM_004170	SLC1A1	solute carrier family 1 member 1	1.240685774	1.396692429	5.226796	6.498205	6.864593	4.3257022	4.62285328	4.78719527	748	1240	1285
5293	NM_001350234,NM_001350235	PIK3CD	phosphatidylinositol-4,5-bisphosp	1.429112966	1.396210276	0.382843	0.548105	0.501266	1.57857476	1.94127715	1.91649468	78	149	134
5634	NM_001039091,NM_002765	PRPS2	phosphoribosyl pyrophosphate sy	1.422444654	1.396096476	15.475222	22.048253	20.312145	5.29208934	5.78951493	5.76303325	1499	2849	2574
8227	NM_005088_chrX,NM_005088_c	AKAP17A	A-kinase anchoring protein 17A	-1.180358258	1.395981157	9.333701	7.880569	12.283187	4.92327834	4.69258035	5.39104626	1152	1304	1978
252839	NM_001288564,NM_001288565	TMEM9	transmembrane protein 9	1.730880943	1.395764703	37.46982	61.672175	47.835114	5.92372626	6.70518984	6.3980468	2344	5421	4024
56241	NM_019601	SUSD2	sushi domain containing 2	-3.531662512	1.395752946	5.193064	1.466769	6.812846	4.09573376	2.47408961	4.55272125	632	239	1085
10924	NM_001286138,NM_006714	SMPDL3A	sphingomyelin phosphodiesteras	1.330193205	1.395699912	1.300078	1.827202	1.783873	1.81624731	2.12272838	2.1765086	99	176	170
4086	NM_001003688,NM_005900	SMAD1	SMAD family member 1	1.184974079	1.395631778	3.591954	4.266227	4.715205	3.54817338	3.77370073	3.99380211	420	665	721
8295	NM_001244580,NM_003496	TRRAP	transformation/transcription doma	-1.271803664	1.395521626	6.801225	5.347632	8.943943	6.40295487	6.0606928	6.87893309	3283	3449	5635
23091	NM_001076788,NM_001330564	ZC3H13	zinc finger CCCH-type containing	1.732563824	1.395494511	5.644307	10.036353	7.519162	5.46011023	6.23913274	5.93159518	1689	3910	2899
7050	NM_001278682,NM_001278684	TGIF1	TGFB induced factor homeobox	-1.136971839	1.395414077	55.861136	49.131917	72.480772	6.52409095	6.34103412	7.00034813	3574	4200	6134
54920	NM_001271762,NM_001271763	DUS2	dihydrouridine synthase 2	1.330412158	1.395102137	9.689855	12.978717	12.838486	4.51599508	4.91216578	4.97844822	859	1527	1474
100506100	NR_046240	LOC100506100	uncharacterized LOC100506100	1.228683098	1.395071875	6.679273	8.267749	8.788501	2.25390578	2.49375478	2.64620047	148	243	254
22990	NM_001308160,NM_014982	PCNX1	pecanex homolog 1 (Drosophila)	1.239409238	1.394650701	4.878722	6.053162	6.473298	5.96313584	6.26832673	6.43649962	2410	3991	4134
23052	NM_015036	ENDOD1	endonuclease domain containing	-1.219274112	1.394505646	4.246689	3.48641	5.564789	4.35113248	4.08048581	4.81080629	762	835	1307
25897	NM_001280539,NM_015435,NM	RNF19A	ring finger protein 19A, RBR E3 u	-1.287851685	1.394195933	9.894545	7.711509	12.921534	5.43144524	5.07604389	5.90142239	1655	1717	2838
8784	NM_004195,NM_148901,NM_14	TNFRSF18	TNF receptor superfamily membe	-1.220047841	1.394127178	4.804649	3.971006	6.318708	2.72881625	2.48886356	3.1454863	221	242	379
100507639	NR_121625	LINC02432	long intergenic non-protein codin	-2.081671237	1.393947266	12.599995	6.056668	16.510439	3.76370129	2.81621366	4.21263029	494	317	847
54897	NM_001079843,NM_017766	CASZ1	castor zinc finger 1	-3.469218995	1.393672875	1.265936	0.324453	2.147583	3.2834166	1.81408935	3.71995801	343	132	588
8408	NM_003565	ULK1	unc-51 like autophagy activating	-1.066454873	1.393234334	12.698849	11.920699	16.624649	6.03494768	5.94358222	6.50717988	2535	3176	4344
144717	NM_001177996,NM_001177997	FAM109A	family with sequence similarity 10	-1.221437803	1.393166389	5.097292	4.112794	6.560941	4.06532627	3.79565219	4.51923721	618	676	1059
9463	NM_001039583,NM_001039584	PICK1	protein interacting with PRKCA 1	1.328667724	1.393163804	16.049711	21.449316	21.328196	5.13886234	5.53870585	5.60565833	1344	2386	2303

1314	NM_001098398,NM_004371	COPA	coatomer protein complex subuni	1.219578821	1.3930367	23.452249	28.699724	30.776618	7.02362182	7.30801255	7.49873223	5069	8260	8685
2185	NM_004103,NM_173174,NM_17	PTK2B	protein tyrosine kinase 2 beta	1.079599275	1.392992531	13.992579	15.032422	18.78145	5.83895697	5.94759844	6.31003667	2208	3185	3783
79753	NM_024700	SNIP1	Smad nuclear interacting protein	1.131720419	1.392944954	4.914003	5.567331	6.429439	4.52559963	4.69682839	4.98601009	865	1308	1482
317	NM_001160,NM_013229,NM_18	APAF1	apoptotic peptidase activating fac	-1.166904657	1.392482846	2.679916	2.400255	3.54667	4.3072593	4.09665142	4.76429118	738	845	1264
8242	NM_001146702,NM_001282622	KDM5C	lysine demethylase 5C	1.323417746	1.392297986	17.641086	23.371442	23.287325	6.88063621	7.2819178	7.35466028	4587	8111	7855
497258	NR_002832,NR_033312,NR_03	BDNF-AS	BDNF antisense RNA	1.068928627	1.392231071	0.311124	0.331865	0.445152	0.77668602	0.81765451	0.99569924	28	40	48
79734	NM_001282684,NM_001282685	KCTD17	potassium channel tetramerizatio	2.105980009	1.392211925	6.229954	12.766607	8.01474	3.46076162	4.46494141	3.90085366	393	1106	673
5338	NM_001243108,NM_002663	PLD2	phospholipase D2	1.00859903	1.392183899	6.129631	6.180156	8.022657	4.44691093	4.45870114	4.9055589	817	1101	1399
2310	NR_026718	FOXO3B	forkhead box O3B pseudogene	1.010381876	1.392166479	0.864703	0.873105	1.130374	2.83047153	2.84329233	3.24967672	240	324	411
64863	NM_001308401,NM_022840	METTL4	methyltransferase like 4	-1.122637962	1.392156162	7.310621	6.438558	9.506696	4.74702075	4.58668388	5.20917167	1015	1208	1738
79132	NM_024119	DHX58	DExH-box helicase 58	-1.353462481	1.39210154	13.229301	9.784941	17.314447	5.11785073	4.69576755	5.58339873	1324	1307	2267
79083	NM_001042467,NM_001281473	MLPH	melanophilin	1.212171693	1.392076426	47.779727	58.878134	61.846361	7.46411561	7.74028273	7.93905755	6893	11164	11802
10038	NM_001042618,NM_005484	PARP2	poly(ADP-ribose) polymerase 2	-1.118308694	1.39201001	41.09119	36.910073	53.842325	6.24671209	6.08763347	6.71853673	2942	3515	5037
79867	NM_001143850,NM_024809	TCTN2	tectonic family member 2	1.41945943	1.391918056	4.078644	5.808335	5.339407	3.65413237	4.12530075	4.09866803	455	863	779
149473	NM_001349127,NM_001349128	CCDC24	coiled-coil domain containing 24	1.56057533	1.391826005	7.167384	11.749293	9.432907	3.39939883	3.99164624	3.83747659	375	782	642
5383	NR_027775,NR_027776,NR_02	PMS2P5	PMS1 homolog 2, mismatch repa	-1.830913133	1.391615438	8.285908	4.56666	10.862693	3.45742019	2.68977954	3.89684777	392	286	671
729614	NR_024279	FLJ37453	uncharacterized LOC729614	-1.050365366	1.391591932	1.19522	1.135151	1.559141	2.0648444	2.01116974	2.44142817	125	159	214
9563	NM_001282587,NM_004285	H6PD	hexose-6-phosphate dehydrogen	1.284806623	1.391422336	2.713726	3.491312	3.548999	4.64936645	4.99815263	5.10971027	946	1624	1619
414149	NM_001039844	ACBD7	acyl-CoA binding domain contain	-1.690866917	1.391080678	3.437185	2.031861	4.495384	3.61867338	2.93964205	4.0615736	443	350	758
3191	NM_001005335,NM_001533	HNRNPL	heterogeneous nuclear ribonucle	1.072401753	1.390782374	165.76442	177.747391	213.481262	8.32559883	8.4261415	8.80023502	12556	17991	21478
79883	NM_001146254,NM_001146255	PODNL1	podocan like 1	1.165490008	1.390725496	0.395175	0.473176	0.531936	1.21691895	1.34717796	1.50747923	52	81	89
90499	NR_038409	FAM95A	family with sequence similarity 95	2.023640302	1.39067307	0.141888	0.307947	0.194025	0.43999062	0.7861039	0.58209675	14	38	24
51309	NM_016608	ARMCX1	armadillo repeat containing, X-lin	1.174775307	1.39067307	0.171467	0.200445	0.218501	0.43999062	0.50527715	0.58209675	14	22	24
4584	NM_005960	MUC3A	mucin 3A, cell surface associated	-49.61413382	1.390552925	1.017928	0.020149	1.334628	2.76710656	0.15624221	3.18215673	228	6	390
51011	NM_016044	FAHD2A	fumarylacetoacetate hydrolase d	-1.016814226	1.390547757	13.214888	13.007341	17.265388	4.14641466	4.12372395	4.59907035	656	862	1122
22911	NM_001142550,NM_001142551	WDR47	WD repeat domain 47	1.475027797	1.390308044	2.068113	3.060173	2.703774	3.29094516	3.80355245	3.72448562	345	680	590
6019	NM_001329191,NM_005059,NM	RLN2	relaxin 2	1.396135583	1.390248188	1.788976	2.09096	2.361146	1.10178443	1.37924953	1.37552141	45	84	77
55212	NM_018190,NM_176824	BBS7	Bardet-Biedl syndrome 7	1.233232386	1.39011854	4.262586	5.692331	5.963229	3.90286151	4.18699961	4.35082401	548	903	937
92806	NM_145039	CENPBD1	CENPB DNA-binding domains cc	1.385909374	1.390106366	1.438685	2.005108	1.88552	2.40724866	2.80050884	2.80425801	169	313	289
388558	NM_001039886,NM_001321424	ZNF808	zinc finger protein 808	-1.112259247	1.390106366	1.271973	1.150058	1.739358	2.40724866	2.28388661	2.80425801	169	203	289
26013	NM_015478,NM_032107	L3MBTL1	l(3)mbt-like 1 (Drosophila)	-1.018436771	1.389760729	2.664821	2.496237	3.318871	3.22173053	3.19821471	3.6526589	327	429	559
340075	NM_001012301	ARSI	arylsulfatase family member I	-1.414831878	1.389664194	1.472854	1.040205	1.927035	2.47491059	2.07783221	2.87519973	179	169	306
84179	NM_001294341,NM_001294342	MFSD7	major facilitator superfamily doma	-1.068844677	1.389582446	1.808244	1.707054	2.374449	2.25390578	2.17847216	2.64141996	148	185	253
26168	NM_015670	SENP3	SUMO1/sentrin/SMT3 specific pe	1.550266644	1.389485456	9.979548	15.498332	13.037326	4.65812548	5.27027225	5.11661607	952	1972	1627
79744	NM_001098491,NM_001098492	ZNF419	zinc finger protein 419	1.041680652	1.389442152	22.580776	23.632232	30.892247	5.66664345	5.72442272	6.1331894	1955	2721	3341
101752400	NR_103792	CAPN10-AS1	CAPN10 antisense RNA 1 (head	1.662215591	1.389338433	1.789195	2.977891	2.334009	2.85098947	3.50238056	3.26840163	244	542	417
64969	NM_001321995,NM_001321996	MRPS5	mitochondrial ribosomal protein S	1.175630663	1.389048071	29.274346	34.467651	38.497006	6.6194879	6.85073511	7.08948076	3821	6002	6528
23028	NM_001009999,NM_015013	KDM1A	lysine demethylase 1A	1.363935132	1.388886479	39.767184	54.299463	51.881561	6.88996148	7.33449594	7.36048988	4617	8414	7887
79022	NM_001143841,NM_001143842	TMEM106C	transmembrane protein 106C	1.027880992	1.388878886	37.83581	38.355186	48.946181	5.80518174	5.84415687	6.27188091	2156	2961	3683
54707	NM_018066	GPN2	GPN-loop GTPase 2	1.420233526	1.388768787	6.940346	9.867643	9.064116	3.48721804	3.95487459	3.92465818	401	761	685
92400	NM_033117,NR_027125,NR_02	RBM18	RNA binding motif protein 18	1.207644002	1.388574196	8.361322	10.122365	10.937258	4.70677986	4.96947085	5.16488576	986	1591	1684
6811	NM_001244666,NM_001330294	STX5	syntaxin 5	1.508964759	1.388076735	10.472589	16.151791	13.781387	4.3072593	4.87609552	4.75988697	738	1488	1260
5774	NM_001145368,NM_001145369	PTPN3	protein tyrosine phosphatase, no	-3.363503004	1.388034543	10.444854	3.638826	15.772634	6.44067162	4.72933337	6.90907779	3371	1339	5755
9967	NM_001321471,NM_001321473	THRAP3	thyroid hormone receptor associa	1.534832409	1.387878869	31.988736	49.145383	41.722739	7.11949526	7.7339664	7.58948271	5420	11115	9252
574036	NR_024337	SERTAD4-AS1	SERTAD4 antisense RNA 1	-1.470858095	1.387771951	2.117509	1.432277	2.759024	1.40904982	1.08728891	1.72191206	65	59	111
11040	NM_006875	PIM2	Pim-2 proto-oncogene, serine/thr	1.102143969	1.387733498	3.053315	3.370408	3.991553	2.91084846	3.03332122	3.3291189	256	377	437
106633800	NR_132753	SNORD133	small nucleolar RNA, C/D box 13	1.068150199	1.387593906	0.779466	0.868847	1.020669	0.23670415	0.25173066	0.32020933	7	10	12
220202	NM_145178	ATOH7	atonal bHLH transcription factor 7	-1.03859314	1.387593906	0.103336	0.116139	0.157776	0.23670415	0.2284445	0.32020933	7	9	12
406900	NR_029831	MIR106B	microRNA 106b	-1.03859314	1.387593906	2.043751	2.092228	2.777891	0.23670415	0.2284445	0.32020933	7	9	12
100289511	NR_029378	LOC100289511	uncharacterized LOC100289511	2.121242749	1.387593906	0.115845	0.271614	0.16734	0.23670415	0.46599441	0.32020933	7	20	12
8330	NM_003510	HIST1H2AK	histone cluster 1 H2A family men	1.805314984	1.387593906	0.361505	0.691921	0.521003	0.23670415	0.40498891	0.32020933	7	17	12
100288730	NR_029383	PAN3-AS1	PAN3 antisense RNA 1	2.301867911	1.387153455	0.280132	0.659724	0.373045	0.68823103	1.26944952	0.88680889	24	74	41
100505869	NR_108036	CFAP58-AS1	CFAP58 antisense RNA 1 (head	-1.682596604	1.387153455	0.554571	0.331679	0.749115	0.68823103	0.44594451	0.88680889	24	19	41
113201	NM_138423,NM_177974	CASC4	cancer susceptibility 4	-1.159557512	1.387097015	22.635411	19.625981	29.531903	6.45372678	6.24276906	6.92120803	3402	3920	5804
79144	NM_024299	PPDPF	pancreatic progenitor cell differer	-1.015874344	1.387011511	155.71579	153.451599	202.959061	6.89243808	6.86990834	7.3610352	4625	6083	7890
55181	NM_018149	SMG8	SMG8, nonsense mediated mRN	1.130524995	1.38689528	9.876721	11.186495	12.87943	5.01931705	5.19117885	5.47874833	1234	1864	2105
133418	NM_198449	EMB	embigin	-1.213095562	1.38680232	18.977411	15.6588	24.733349	6.3331139	6.05821852	6.79989065	3126	3443	5332
29894	NM_013291	CPSF1	cleavage and polyadenylation sp	-1.048337218	1.386486998	44.402386	42.404331	57.8526	7.60950095	7.54175415	8.07888022	7628	9722	13008
6595	NM_001289396,NM_001289397	SMARCA2	SWI/SNF related, matrix associat	1.068560446	1.38630555	3.954501	4.380269	5.206087	4.51438809	4.60601172	4.9679855	858	1225	1463
5590	NM_001033581,NM_001033582	PRKCZ	protein kinase C zeta	1.17292282	1.386105508	13.590259	15.967634	17.742297	4.99532414	5.21876673	5.45374301	1213	1901	2068
84804	NM_001322080,NM_001322081	MFSD9	major facilitator superfamily doma	-1.137532417	1.385962354	3.205943	3.231152	4.060004	4.05430925	3.88026307	4.50088019	613	720	1045
132949	NM_001286668,NM_001286669	AASDH	aminoadipate-semialdehyde deh	1.079972615	1.385865893	2.987247	3.115852	3.833643	3.43720745	3.53833258	3.8705348	386	557	658
51241	NM_001204090,NM_016468	COX16	COX16, cytochrome c oxidase as	1.353575806	1.385410541	16.359563	22.213154	21.306375	4.8216876	5.24511195	5.2777797	1071	1937	1825
56990	NM_001038702,NM_020240	CDC42SE2	CDC42 small effector 2	-1.090674454	1.385314801	23.189019	21.242631	30.527162	6.38195877	6.25830165	6.84736687	3235	3963	5512
728613	NR_003713	LOC728613	programmed cell death 6 pseudo	1.917139746	1.385014014	0.714524	1.380603	0.93355	1.48977343	2.16012918	1.80975447	71	182	121
27430	NM_013283,NM_182796	MAT2B	methionine adenosyltransferase 2	1.098165709	1.385000407	27.851415	31.039953	36.564585	5.92976705	6.06275147	6.39307846	2354	3454	4010
2217	NM_001136019,NM_004107	FCGRT	Fc fragment of IgG receptor and	-1.158914216	1.38483865	18.812973	16.325562	24.588128	4.99186377	4.78625756	5.44896312	1210	1395	2061
79624	NM_001286562,NM_024573	ARMT1	acidic residue methyltransferase	1.409037993	1.384714803	4.028233	5.69665	5.270361	3.48721804	3.94419393	3.92071794	401	755	683
54932	NM_001286823,NM_017820,NR	EXD3	exonuclease 3'-5' domain contair	1.063080878	1.384594728	5.837989	5.852336	7.594059	3.55131131	3.63226192	3.98628107	421	598	717
63917	NM_001304514,NM_022087	GALNT11	polypeptide N-acetylgalactosamir	-1.634570021	1.384527342	11.731523	7.332408	15.267298	5.01704913	4.33606188	5.47405072	1232	1007	2098
84807	NM_001321831,NM_139239	NFKBID	NFKB inhibitor delta	-1.036407691	1.384362618	12.293396	11.842542	15.899972	4.71099515	4.66140215	5.16488576	989	1275	1684
10116	NM_015322	FEM1B	fem-1 homolog B	-1.017507495	1.384261145	6.543538	6.43907	8.514356	5.53572684	5.51123031	5.99620882	1782	2340	3034
6950	NM_001008897,NM_030752	TCP1	t-complex 1	1.59564153	1.383414236	49.876451	79.598344	64.738072	6.90537089	7.57502011	7.37027424	4667	9950	7941
3149	NM_001301228,NM_001301229	HMGB3	high mobility group box 3	-1.050772191	1.383078051	13.648291	12.965844	17.707289	5.60153485	5.53158861	6.06118777	1867	2374	3176
25825	NM_012105,NM_138991,NM_13	BACE2	beta-site APP-cleaving enzyme 2	-1.602252802	1.383045575	4.601775	2.869861	5.967252	5.34729208	4.68831976	5.8053204	1559	1300	2652
4034	NM_001289934,NM_002319	LRCH4	leucine rich repeats and calponin	-1.3081965	1.382911167	18.780078	14.206237	24.379771	5.89499076	5.51484389	6.35598995	2297	2346	3907
147929	NM_001042474,NM_152477	ZNF565	zinc finger protein 565	-1.151305284	1.382392069	2.416733	1.956791	3.093811	2.52684435	2.36086138	2.92325564	187	217	318
25913	NM_001042594,NM_015450,NR	POT1	protection of telomeres 1	-2.142955705	1.382042693	5.56575	2.628909	7.245606	4.52240522	3.49264009	4.97179891	863	538	1467
79139	NM_001134671,NM_001330601	DERL1	derlin 1	3.318661713	1.381868096	23.272111	77.332244	30.297298	6.24719593	7.96450308	6.70857291	2943	13050	5002
10518	NM_001271888,NM_001271889	CIB2	calcium and integrin binding fami	1.197332675	1.381857225	2.206088	2.557651	2.80542	2.10810423	2.31185141	2.47942639	130	208	221
7321	NM_001204880,NM_003338	UBE2D1	ubiquitin conjugating enzyme E2	-1.167905546	1.381467668	6.158346	5.279061	8.000731	4.08061013	3.87089436	4.52314065	625	715	1062
201475	NM_001025300	RAB12	RAB12, member RAS oncogene	-1.088174293	1.380955687	33.552727	30.870371	43.549385	6.14704714	6.02692652	6.60710335	2743	3368	4659
2021	NM_004435	ENDOG	endonuclease G	-1.032942714	1.380563032	10.892406	10.54889	14.127208	3.71696001	3.67379966	4.15174023	477	617	810
84775	NM_001172677,NM_032689	ZNF607	zinc finger protein 607	-1.283475366	1.380517907	4.291445	3.164715	5.135104	4.1526264	3.81532231	4.59536729	659	686	1119
6625	NM_001301069,NM_003089	SNRNP70	small nuclear ribonucleoprotein U	1.031565009	1.380436565	123.617897	127.586521	160.304119	7.90379741	7.94844831	8.3672656	9363	12905	15897
5607	NM_001206804,NM_002757,NM	MAP2K5	mitogen-activated protein kinase	-1.011523007	1.380207222	2.969609	2.929375	3.849005	2.96832247	2.95391015	3.38166197	268	354	455
2150	NM_005242	F2RL1	F2R like trypsin receptor 1	1.339543629	1.379978846	29.596745	39.687695	38.380127	6.40902161	6.82646518	6.86899886	3297	5901	5596
404093	NM_001271875,NM_001292025	CUEDC1	CUE domain containing 1	2.063994269	1.379872611	24.446538	51.322574	31.843843	6.49301423	7.53019529	6.95314577	3497	9644	5935
118812	NM_001098831,NM_178832	MORN4	MORN repeat containing 4	-1.47776668	1.379842078	5.139192	3.42775	6.66711	3.69442889	3.18315375	4.1280136	469	424	796
6541	NM_003045	SLC7A1	solute carrier family 7 member 1	1.277840685	1.379756741	11.028898	14.109756	14.299799	6.31937498	6.66916033	6.77882228	3096	5286	5254
10970	NM_006825	CKAP4	cytoskeleton associated protein 4	-1.074581776	1.379522574	19.853962	18.49847	25.746828	5.8999225	5.79794319	6.35744822	2305	2866	3911
9755	NM_014726	TBKBP1	TBK1 binding protein 1	1.639970358	1.3794723	2.939593	4.828202	3.811799	3.68302999	4.35231081	4.11600233	465	1019	789
51552	NM_016322	RAB14	RAB14, member RAS oncogene	-1.068687583	1.379271639	30.692616	28.751123	39.784008	7.03767435	6.94258617	7.49856702	5119	6400	8684
6746	NM_003145	SSR2	signal sequence receptor subunit	1.121964057	1.379218033	114.429779	128.534424	148.327484	6.98004173	7.14482875	7.44075568	4917	7371	8341
54930	NM_001166269,NM_001166270	HAUS4	HAUS augmin like complex subu	-1.297859266	1.378545327	27.779865	21.417022	36.022383	5.49475122	5.12809029	5.9491104	1731	1782	2935
4861	NM_001321086,NM_002517	NPAS1	neuronal PAS domain protein 1	-1.130088132	1.378437081	0.48967	0.360378	0.817142	0.88013425	0.80196545	1.11084509	33	39	56
10766	NM_016272	TOB2	transducer of ERBB2, 2	1.281908567	1.378260907	9.434718	12.111753	12.222726	5.42461696	5.77552483	5.87824435	1647	2821	2792
27072	NM_014396,NM_080631	VPS41	VPS41, HOPS complex subunit	-1.238745266	1.378191708	7.886268	6.374726	10.215436	5.27035534	4.97034843	5.72286727	1476	1592	2502
11069	NM_001100397,NM_001282899	RAPGEF4	Rap guanine nucleotide exchang	1.269454991	1.377949313	0.05862	0.076282	0.07605	0.3273552	0.40498891	0.43515728	10	17	17

57587	NM_001292033,NM_020827	CFAP97	cilia and flagella associated prote	1.247656828	1.377672961	4.511288	5.680778	5.926158	4.48186648	4.78824961	4.92634185	838	1397	1420
169200	NM_001008495,NM_001146273	TMEM64	transmembrane protein 64	1.018215721	1.377524247	3.265651	3.355409	4.233531	4.02977152	4.05423805	4.46750731	602	819	1020
1520	NM_001199739,NM_004079	CTSS	cathepsin S	-2.316500243	1.377338264	6.891658	2.97571	8.91672	4.83591088	3.68880651	5.28392771	1082	624	1833
10670	NM_006570	RRAGA	Ras related GTP binding A	1.596142948	1.377266326	20.309095	32.45435	26.286278	5.07161207	5.73013338	5.52165373	1281	2732	2170
7414	NM_003373,NM_014000	VCL	vinculin	-1.502415665	1.377154252	34.020784	22.676973	43.965591	7.4603647	6.87717935	7.91981608	6875	6114	11645
29934	NM_001256185,NM_001256186	SNX12	sorting nexin 12	1.150682355	1.377107752	13.559045	16.147435	17.377349	5.02722683	5.22392723	5.47673694	1241	1908	2102
7534	NM_001135699,NM_001135700	YWHAZ	tyrosine 3-monooxygenase/tryptc	-1.06164019	1.377059049	349.544323	329.650547	452.767944	10.02518062	9.93897083	10.48639298	40871	51438	69223
1955	NM_001080497	MEGF9	multiple EGF like domains 9	-1.160834884	1.376897487	2.817257	2.428539	3.646398	4.17314113	3.97074877	4.61256748	669	770	1133
84548	NM_001174092,NM_001282302	TMEM185A	transmembrane protein 185A	1.400768307	1.376871835	5.08046	7.750072	5.931004	3.55131131	4.00198255	3.97872063	421	788	713
387521	NM_001162505,NM_199129,NR	TMEM189	transmembrane protein 189	1.205725981	1.376741711	19.568738	23.511842	25.326211	5.48985314	5.75428199	5.94232417	1725	2779	2921
79810	NM_001284403,NM_001284404	PTCD2	pentatricopeptide repeat domain	1.510476192	1.376570187	4.042053	6.14321	5.257497	3.21778616	3.75955568	3.63593285	326	658	552
54899	NM_001289095,NM_001289096	PXK	PX domain containing serine/thre	1.501939841	1.376537194	1.119471	1.689082	1.441819	2.16656821	2.64211237	2.53718654	137	275	232
100129083	NM_001256795	LOC100129083	uncharacterized LOC100129083	1.329570437	1.376497102	0.550384	0.738917	0.722258	0.93882015	1.15059938	1.17840727	36	64	61
645676	NR_027023	ASH1L-AS1	ASH1L antisense RNA 1	1.129993047	1.376342657	2.677995	3.041456	3.472214	2.61945147	2.76857683	3.01481644	202	305	342
25758	NM_012194	KIAA1549L	KIAA1549 like	-2.175565733	1.376260676	0.138135	0.064018	0.179871	1.36692851	0.7861039	1.66651905	62	38	105
9266	NM_004228,NM_017457	CYTH2	cytohesin 2	-1.125163074	1.375893765	33.864395	30.133236	43.792643	7.25629567	7.08733896	7.71409176	5963	7081	10091
11041	NM_006876	B4GAT1	beta-1,4-glucuronyltransferase 1	1.5527618	1.375844696	6.073891	9.447536	7.853714	3.72809498	4.32375395	4.15844821	481	998	814
23768	NM_001346143,NM_001346144	FLRT2	fibronectin leucine rich transmem	2.326768757	1.375700868	1.788211	3.550544	1.921421	2.49460548	3.55948145	2.88332104	182	566	308
57639	NM_020879	CCDC146	coiled-coil domain containing 146	-1.7555321	1.375553146	2.162329	1.231127	2.796899	3.00540462	2.32288731	3.4156568	276	210	467
214	NM_001243280,NM_001243281	ALCAM	activated leukocyte cell adhesion	1.678854274	1.375545392	7.551429	12.875187	9.981597	5.1981206	5.92966236	5.64738581	1402	3145	2372
6311	NM_001310121,NM_001310123	ATXN2	ataxin 2	-1.03964187	1.375487529	17.455571	16.425178	22.562464	6.22967464	6.17434782	6.68437517	2907	3736	4918
8760	NM_003818	CDS2	CDP-diacylglycerol synthase 2	1.432092718	1.375344086	8.851108	12.6928	11.440943	6.34627164	6.85905112	6.80123075	3155	6037	5337
8509	NM_001330107,NM_003635	NDST2	N-deacetylase and N-sulfotransfe	-1.417213302	1.374773548	3.766841	2.660669	4.872651	3.97005831	3.50480544	4.40400834	576	543	974
11337	NM_007278	GABARAP	GABA type A receptor-associate	1.103282961	1.374523233	55.473457	61.273945	71.656944	5.62182561	5.76089064	6.07277472	1894	2792	3202
105371730	NR_136413	LOC105371730	uncharacterized LOC105371730	1.208152745	1.374318254	0.417611	0.511345	0.549526	0.73313634	0.84852988	0.93448595	26	42	44
7485	NM_001146218,NM_001350293	WRB	tryptophan rich basic protein	-1.046077097	1.374214864	13.938257	13.862457	18.009952	4.52080535	4.45870114	4.96224642	862	1101	1457
57536	NM_001322327,NM_020776,NR	KIAA1328	KIAA1328	-1.019295619	1.37311416	0.770168	0.747997	1.129753	2.25390578	2.23214197	2.62698266	148	194	250
54994	NM_017896	GID8	GID complex subunit 8 homolog	1.197821972	1.372746479	15.850459	19.012123	20.450436	6.11559477	6.37257735	6.56701545	2683	4294	4530
677844	NR_003020	SNORA78	small nucleolar RNA, H/ACA box	2.063830339	1.37271801	2.520881	5.553308	3.359686	0.41264541	0.75384785	0.54161868	13	36	22
114799	NM_052911	ESCO1	establishment of sister chromatid	-1.167637387	1.372578664	7.008903	6.008817	9.045217	4.987237	4.7712289	5.43176167	1206	1380	2036
290	NM_001150	ANPEP	alanyl aminopeptidase, membran	-2.137532625	1.372547279	146.8125	68.756271	189.367493	9.02997783	7.93715882	9.48608653	20484	12804	34580
54682	NM_018050	MANSC1	MANSC domain containing 1	-1.244166282	1.372452884	8.323903	6.695306	10.736681	4.29420754	3.99682365	4.73092712	731	785	1234
343637	NM_001029871,NM_001040007	RSPO4	R-spondin 4	2.761765834	1.37205212	3.059398	8.543933	4.075906	3.18179291	4.54252068	3.59448638	317	1170	535
8417	NM_001326578,NM_001326579	STX7	syntaxin 7	1.985976242	1.371923167	3.195799	5.444031	4.363894	3.53555296	4.46244854	3.95772276	416	1104	702
1192	NM_001287593,NM_001287594	CLIC1	chloride intracellular channel 1	1.140930082	1.371876756	194.943111	224.594725	253.547673	7.89317092	8.08263354	8.34768053	9294	14168	15682
7030	NM_001282142,NM_006521	TFE3	transcription factor binding to IGH	1.288527779	1.371755261	8.413205	10.829847	10.80056	4.8626811	5.2172889	5.30524173	1103	1899	1861
56927	NM_001080452	GPR108	G protein-coupled receptor 108	1.119309468	1.371700339	35.316166	39.571754	45.527031	6.17782843	6.33831747	6.62839909	2803	4192	4729
9777	NM_014742	TM9SF4	transmembrane 9 superfamily me	-1.004142978	1.371655076	29.775738	29.684694	38.379402	6.86211879	6.85620532	7.31468202	4528	6025	7639
55602	NM_001317343,NM_017632	CDKN2AIP	CDKN2A interacting protein	1.247354879	1.37155382	5.391322	6.756844	6.959449	4.3072593	4.61164745	4.74325038	738	1230	1245
60	NM_001101	ACTB	actin beta	-1.280155232	1.371553594	2309.370605	1805.968506	2976.508057	11.99265713	11.63643728	12.4483725	159954	166947	269830
728229	NM_001242313	TMEM191B	transmembrane protein 191B	1.757807245	1.37150278	1.567398	2.754851	2.020353	1.602974	2.19658485	1.92438744	80	188	135
10314	NM_001136574,NM_001136575	LANCL1	LanC like 1	1.304372865	1.371330336	13.261053	17.314716	17.239195	5.91705205	6.29484139	6.366167	2333	4066	3935
29109	NM_001318202,NM_013241	FHOD1	formin homology 2 domain conta	-1.174874493	1.371300595	15.173897	12.933509	19.562473	5.85745527	5.62928167	6.30626619	2237	2544	3773
9945	NM_005110	GFPT2	glutamine-fructose-6-phosphate t	1.762899953	1.371161446	3.400162	6.000903	4.379344	3.49049124	4.2519532	3.91081999	402	947	678
374378	NM_198516	GALNT18	polypeptide N-acetylgalactosamir	1.965782804	1.371096495	3.308073	6.529809	4.270272	3.18986937	4.08535454	3.60188752	319	838	538
80351	NM_025235	TNKS2	tankyrase 2	-1.26106223	1.371087207	10.309951	8.231658	13.361332	6.00779304	5.67897902	6.45704978	2487	2635	4194
6341	NM_004589	SCO1	SCO1, cytochrome c oxidase ass	1.294575249	1.370895817	11.967609	15.521388	15.428981	4.42312766	4.78026487	4.85998953	803	1389	1354
57722	NM_020962	IGDCC4	immunoglobulin superfamily DCC	1.226690182	1.370237823	0.781222	0.959629	1.006229	2.57080169	2.8201133	2.95827541	194	318	327
57533	NM_001113361,NM_001113363	TBC1D14	TBC1 domain family member 14	-1.081810853	1.36989177	22.12006	19.625653	28.552119	6.60634769	6.49410697	7.0564174	3786	4676	6379
6302	NM_001330168,NM_001330169	TSPAN31	tetraspanin 31	-1.027491443	1.369824709	16.305531	15.898197	20.96965	4.83591088	4.79816878	5.2762386	1082	1407	1823
145567	NM_001010854,NM_001320421	TTC7B	tetratricopeptide repeat domain 7	1.133435195	1.369765976	2.363561	2.683187	3.04941	3.15728865	3.31887893	3.56701931	311	471	524
51293	NM_001165895,NM_016579	CD320	CD320 molecule	1.748985131	1.369742596	25.224251	43.990667	32.311532	4.97910428	5.7659537	5.42064411	1199	2802	2020
10489	NM_006369	LRRC41	leucine rich repeat containing 41	1.471603315	1.369540307	13.947238	20.550304	17.952906	5.35897928	5.90508943	5.80318171	1572	3091	2648
65997	NM_023940	RASL11B	RAS like family 11 member B	1.679433165	1.369434795	0.207933	0.352053	0.271321	0.49317359	0.75384785	0.64076093	16	36	27
7060	NM_001306212,NM_001306213	THBS4	thrombospondin 4	3.179528306	1.36938831	0.273067	0.890265	0.372554	0.91952222	1.94127715	1.15176086	35	149	59
342945	NM_001321116,NM_001321117	ZSCAN22	zinc finger and SCAN domain co	-1.020548389	1.369212729	1.709448	1.679773	2.203379	3.12816418	3.10219703	3.53644652	304	398	512
1665	NM_001358	DHX15	DEAH-box helicase 15	1.131970615	1.369194704	103.46711	117.255928	133.131104	8.25144909	8.42973507	8.70350327	11925	18036	20082
10428	NM_006324	CFDP1	craniofacial development protein	-1.010441984	1.369162344	42.069523	41.677414	54.12233	5.73860759	5.72390245	6.18461832	2057	2720	3464
51588	NM_015897	PIAS4	protein inhibitor of activated STA	1.251296434	1.369112242	8.796711	11.024871	11.323805	4.83204569	5.14528911	5.27160538	1079	1804	1817
23176	NM_001098811,NM_001098812	SEPT8	septin 8	-1.090634568	1.369101995	25.452408	23.234259	32.600633	6.73886216	6.61491476	7.18845636	4154	5089	6995
55218	NM_001193360,NM_001193361	EXD2	exonuclease 3'-5' domain contair	1.015088156	1.369006588	7.659648	7.880701	9.928203	5.28927297	5.31033101	5.73245224	1496	2029	2519
51337	NM_016647	THEM6	thioesterase superfamily membe	1.132250682	1.368973189	15.744201	17.849968	20.254438	5.15854386	5.33302449	5.60074135	1363	2062	2295
43	NM_000665,NM_001282449,NM	ACHE	acetylcholinesterase (Cartwright	-2.412935015	1.36880485	0.852378	0.356465	1.239446	1.55375576	0.84852988	1.86820921	76	42	128
2908	NM_000176,NM_001018074,NM	NR3C1	nuclear receptor subfamily 3 grou	1.427400826	1.368485529	8.05783	11.532112	10.387577	5.75435052	6.25973808	6.19973682	2080	3967	3501
8800	NM_001271572,NM_001271573	PEX11A	peroxisomal biogenesis factor 11	-1.031850429	1.36841113	5.233766	5.070666	6.722418	3.89793981	3.85577671	4.32422401	546	707	919
10507	NM_001142287,NM_006378	SEMA4D	semaphorin 4D	1.193036695	1.368358798	6.119201	7.303851	7.851777	4.83204569	5.07848781	5.27083173	1079	1720	1816
105377621	NR_136202	LOC105377621	uncharacterized LOC105377621	-1.491247311	1.36826241	1.288874	0.848322	1.660478	0.97665845	0.72085408	1.21747621	38	34	64
8658	NM_003747	TNKS	tankyrase	1.263462791	1.367828205	3.294422	4.167558	4.233733	4.99070847	5.31862454	5.43037665	1209	2041	2034
23515	NM_001320445,NM_001320446	MORC3	MORC family CW-type zinc finge	1.042729866	1.367796978	10.350049	10.808923	13.310541	5.45341658	5.51243584	5.89641527	1681	2342	2828
90864	NM_001324081,NM_080861	SPSB3	splA/ryanodine receptor domain a	-1.04049758	1.36776287	24.203016	23.921764	31.923838	5.22489744	5.16918099	5.6633355	1429	1835	2404
6009	NM_005614	RHEB	Ras homolog enriched in brain	-1.59640633	1.367666994	56.438244	35.389545	72.545067	6.84591841	6.1785307	7.29426962	4477	3747	7531
10160	NM_001001715,NM_001286839	FARP1	FERM, ARH/RhoGEF and plecks	1.119269537	1.367661539	2.989601	3.385137	3.955276	3.92480368	4.07723084	4.35082401	557	833	937
9700	NM_012291	ESPL1	extra spindle pole bodies like 1, s	-1.071496292	1.367612478	10.409612	9.726921	13.379111	6.09046901	5.99235095	6.53644257	2636	3287	4434
57508	NM_001330417,NM_001351695	INTS2	integrator complex subunit 2	-1.027940875	1.367492816	8.643907	8.430641	11.106706	5.71850978	5.67951575	6.1626823	2028	2636	3411
8623	NM_001173473_chrX,NM_0011	ASMTL	acetylserotonin O-methyltransfer	1.626219685	1.36738884	3.469335	5.64964	4.466669	3.0370876	3.66948316	3.44063736	283	615	476
654466	NR_003670	FGF7P3	fibroblast growth factor 7 pseudo	1.337403371	1.367182304	0.103776	0.138977	0.134753	0.56944351	0.72085408	0.73352601	19	34	32
6414	NM_001085486,NM_001093726	SELENOP	selenoprotein P	-1.581449141	1.367182304	0.207285	0.131968	0.273138	0.56944351	0.38406669	0.73352601	19	16	32
219402	NM_001166261,NM_001166262	MTIF3	mitochondrial translational initiati	-1.036170659	1.366911409	16.669829	16.391144	21.773783	4.20337642	4.15493692	4.63318145	684	882	1150
51278	NM_016545	IER5	immediate early response 5	1.177345483	1.366575905	24.986938	29.446516	32.084721	5.85745527	6.08925019	6.30134978	2237	3519	3760
80306	NM_025205	MED28	mediator complex subunit 28	1.196028371	1.366573929	14.651111	17.538967	18.817196	5.13573004	5.38725856	5.57527538	1341	2143	2254
140825	NM_001278535,NM_080749	NEURL2	neuralized E3 ubiquitin protein lig	1.546569176	1.366470035	0.785718	1.22917	1.014331	1.08456011	1.45142181	1.34055586	44	91	74
5613	NM_005044	PRKX	protein kinase, X-linked	-1.038940716	1.366411321	1.937078	1.866557	2.488856	3.64240927	3.59177559	4.0615736	451	580	758
8703	NM_001199873,NM_001199874	B4GALT3	beta-1,4-galactosyltransferase 3	1.104156171	1.366245601	17.439195	19.236504	22.348695	5.3829543	5.5226424	5.82389986	1599	2359	2687
91862	NM_001017967,NM_001271329	MARVELD3	MARVEL domain containing 3	-1.072804523	1.365968479	14.562645	13.427229	18.135475	5.10935964	5.01100366	5.54807524	1316	1639	2211
574029	NR_002834	DUSP5P1	dual specificity phosphatase 5 ps	-2.976908687	1.36574505	0.490425	0.159022	0.635926	1.13562816	0.48576948	1.39836968	47	21	79
4907	NM_001204813,NM_002526	NT5E	5'-nucleotidase ecto	-1.081403963	1.365558164	37.063832	34.389905	47.703621	7.18888683	7.07678378	7.63573576	5689	7029	9555
64769	NM_001270875,NM_001270876	MEAF6	MYST/Esa1 associated factor 6	-1.008758421	1.365447042	8.244574	8.196043	10.603548	5.30516019	5.29289816	5.74476254	1513	2004	2541
684	NM_004335	BST2	bone marrow stromal cell antigen	-1.066136897	1.365234304	115.060379	108.044266	147.626266	6.84751475	6.75594809	7.29331757	4482	5617	7526
55	NM_001099,NM_001134194,NM	ACPP	acid phosphatase, prostate	2.362137476	1.365106868	0.570755	1.144296	0.800736	1.18495016	2.0043309	1.45395569	50	158	84
11179	NM_021994	ZNF277	zinc finger protein 277	-1.973850328	1.364691006	5.451331	2.762342	6.991882	3.9320442	3.04002433	4.35521005	560	379	940
55113	NM_018053	XKR8	XK related 8	-1.60178387	1.364678124	4.624302	2.888278	5.931153	3.71416279	3.09899072	4.13313083	476	397	799
25816	NM_001077654,NM_001286813	TNFAIP8	TNF alpha induced protein 8	1.413386987	1.364587024	5.145441	7.227242	6.556585	3.46742135	3.9280232	3.88071204	395	746	663
4905	NM_006178,NR_040116	NSF	N-ethylmaleimide sensitive factor	-1.195017091	1.364580664	12.907673	10.812114	16.552863	5.67385961	5.42231408	6.11476878	1965	2197	3298
4012	NM_005575,NM_175920	LNPEP	leucyl and cystinyl aminopeptidas	-1.055896707	1.364539093	3.327015	3.154204	4.26967	3.95355025	3.88026307	4.37694243	569	720	955
478	NM_001256213,NM_001256214	ATP1A3	ATPase Na+/K+ transporting sub	2.608561349	1.364277831	0.597333	1.578841	0.772377	1.65056839	2.71931318	1.97085819	84	293	141
10533	NM_001136031,NM_001144912	ATG7	autophagy related 7	1.232975868	1.364182442	4.887287	5.910328	6.843796	4.60927912	4.90024229	5.04149497	919	1514	1542
2232	NM_001258012,NM_001258013	FDXR	ferredoxin reductase	1.042411092	1.363889329	8.307729	8.862676	10.863018	4.04987877	4.10626455	4.47424393	611	851	1025
64395	NM_178439	GMCL1	germ cell-less, spermatogenesis	-1.147000682	1.363841358	5.143926	4.487868	6.588748	4.47030848	4.28195221	4.90056614	831	968	1394
85457	NM_033426	CIPC	CLOCK interacting pacemaker	1.240885357	1.3636037	5.329383	6.621597	6.829541	4.5587208	4.85819066	4.98977621	886	1469	1486

256126	NM_001105578	SYCE2	synaptonemal complex central el	2.719866512	1.36330934	0.75806	2.126509	1.003942	0.77668602	1.55800914	0.98063768	28	102	47
26782	NR_002444	SNORA66	small nucleolar RNA, H/ACA box	1.441721052	1.36330934	5.474529	7.86954	7.060546	0.77668602	1.02107213	0.98063768	28	54	47
57178	NM_020338	ZMIZ1	zinc finger MIZ-type containing 1	-1.343329793	1.362943873	6.427573	4.791313	8.233627	5.59394673	5.17832427	6.03271963	1857	1847	3113
100506394	NR_044993	GAS6-AS2	GAS6 antisense RNA 2 (head to	-1.240194415	1.362798074	0.87152	0.700051	1.113641	1.40904982	1.22307764	1.70368302	65	70	109
1603	NM_001344	DAD1	defender against cell death 1	1.246714316	1.362287044	153.702194	191.858322	196.804611	6.71876791	7.03419316	7.16116188	4096	6823	6863
8895	NM_003909	CPNE3	copine 3	-1.245354842	1.362152046	14.609477	11.745076	18.704292	6.13350189	5.82196451	6.57393141	2717	2915	4552
6001	NM_001005339,NM_002925	RGS10	regulator of G protein signaling 1	-1.137090656	1.361775765	13.936459	12.21677	17.767324	3.72531928	3.55480842	4.14161942	480	564	804
129790	NR_026865	C7orf13	chromosome 7 open reading fran	1.12448363	1.361757569	2.241693	2.530781	2.876216	2.58923814	2.73178769	2.96976228	197	296	330
29091	NM_001304476,NM_001304477	STXBP6	syntaxin binding protein 6	-1.475146932	1.361576385	1.727795	1.189111	2.167821	3.05045588	2.56983975	3.44886901	286	259	479
51603	NM_001007239,NM_014955,NM	METTL13	methyltransferase like 13	1.145309189	1.361573599	9.247036	10.691202	12.068342	4.98607799	5.17604388	5.41924838	1205	1844	2018
23169	NM_015139	SLC35D1	solute carrier family 35 member [	-1.049441126	1.36142605	1.701389	1.623444	2.17867	3.55131131	3.48774509	3.96347983	421	536	705
10869	NM_001199160,NM_001199161	USP19	ubiquitin specific peptidase 19	1.244477921	1.361347	8.053239	10.03777	10.295331	5.25984392	5.56798878	5.69487827	1465	2436	2453
11169	NM_001008396,NM_007086	WDHD1	WD repeat and HMG-box DNA b	1.285993026	1.361332749	9.198479	11.832498	11.789777	5.7806586	6.13770922	6.21871574	2119	3641	3548
57677	NM_001297619,NM_020917	ZFP14	ZFP14 zinc finger protein	-1.262674471	1.360953669	0.778255	0.618965	0.996643	2.74535098	2.46415559	3.13191707	224	237	375
4810	NM_001136024,NM_001291867	NHS	NHS actin remodeling regulator	1.02177066	1.360888556	1.362207	1.393833	1.743876	3.66865353	3.69731229	4.08288706	460	628	770
55521	NM_001017397,NM_001017398	TRIM36	tripartite motif containing 36	1.815444675	1.360720717	0.52417	1.004098	0.702743	1.70793493	2.35549733	2.03057861	89	216	149
10272	NM_005860	FSTL3	follistatin like 3	-2.034598368	1.360530621	21.172796	10.415382	27.078012	5.72199495	4.72518011	6.15892396	2033	1335	3402
63894	NM_001193314,NM_001193315	VIPAS39	VPS33B interacting protein, apic	1.283602177	1.360486023	7.061009	8.794902	8.921426	4.22515306	4.56825335	4.64874869	695	1192	1163
8894	NM_001316363,NM_001316364	EIF2S2	eukaryotic translation initiation fa	1.118515875	1.360410041	105.489485	118.017992	134.406521	8.09292324	8.25395051	8.53556825	10680	15961	17870
4485	NM_020998,NR_146060	MST1	macrophage stimulating 1	-1.029846786	1.360266904	3.473127	3.376091	4.49441	3.13654562	3.09899072	3.53644652	306	397	512
64786	NM_001146213,NM_001146214	TBC1D15	TBC1 domain family member 15	-1.180541608	1.360015338	6.229065	5.304298	7.964991	5.16162707	4.92941706	5.59457146	1366	1546	2285
27125	NM_014423	AFF4	AF4/FMR2 family member 4	1.213645715	1.35997097	12.39781	15.063131	15.844216	6.86590435	7.1430786	7.30621192	4540	7362	7594
9321	NM_001321851,NM_004239	TRIP11	thyroid hormone receptor interact	1.133512583	1.359559151	4.41485	5.00827	5.63937	5.45843972	5.6353808	5.89289991	1687	2555	2821
133686	NM_001085411,NM_001287340	NADK2	NAD kinase 2, mitochondrial	1.138702328	1.359240817	3.442181	3.967325	4.396807	3.81159508	3.98645018	4.22706077	512	779	856
57683	NM_001285549,NM_020923	ZDBF2	zinc finger DBF-type containing 2	1.842736811	1.35919187	2.840111	5.24458	3.639337	4.87400371	5.73323878	5.3037297	1112	2738	1859
7059	NM_001252607,NM_001252608	THBS3	thrombospondin 3	-1.258819484	1.359099854	17.42529	13.942823	22.310677	5.80123322	5.47582432	6.23705219	2150	2282	3594
80762	NM_030571	NDFIP1	Nedd4 family interacting protein 1	1.146368266	1.359072451	26.612604	30.545837	33.992214	6.55802658	6.75314622	6.99660954	3660	5606	6118
87769	NM_001195087,NM_033110	GGACT	gamma-glutamylamine cyclotrans	1.019383571	1.359063495	0.55363	0.571402	0.718122	1.30878272	1.32539385	1.58918956	58	79	97
123	NM_001122,NR_038064	PLIN2	perilipin 2	4.021708776	1.358917234	20.943007	84.342181	26.782634	5.39784781	7.3797859	5.83126516	1616	8684	2701
201229	NM_001076680	LYRM9	LYR motif containing 9	1.125405848	1.35891534	2.298147	2.602852	2.943499	2.11660268	2.24959721	2.46867149	131	197	219
26133	NM_015638,NM_199368	TRPC4AP	transient receptor potential catior	1.105901482	1.358833509	31.592369	34.978491	40.408566	6.6194879	6.76330885	7.05798779	3821	5646	6386
211	NM_000688,NM_001104443,NM	ALAS1	5'-aminolevulinate synthase 1	1.45044907	1.358831434	16.509819	23.67178	20.883719	5.23175921	5.75631865	5.66398036	1436	2783	2400
84232	NM_032272	MAF1	MAF1 homolog, negative regulat	-1.048138364	1.358451604	33.030914	31.553604	42.182007	5.8606208	5.79398308	6.29603638	2242	2858	3746
23143	NM_001164211,NM_001164213	LRCH1	leucine rich repeats and calponin	1.239061288	1.358299289	3.634565	4.512515	4.650954	4.1588115	4.45243375	4.57920996	662	1096	1106
2535	NM_001466	FZD2	frizzled class receptor 2	1.979876725	1.358174628	4.112958	8.160286	5.252169	4.02751998	4.96859275	4.44573627	601	1590	1004
284309	NM_001348007,NM_173632,NR	ZNF776	zinc finger protein 776	1.316344497	1.357947025	9.916768	13.150365	15.467613	5.29396387	5.68166066	5.725693	1501	2640	2507
253635	NM_001278505,NM_001322249	GPATCH11	G-patch domain containing 11	1.984596557	1.357885303	1.841104	3.673832	2.377115	3.00540462	3.90250283	3.39875952	276	732	461
137492	NM_001145152,NM_152415	VPS37A	VPS37A, ESCRT-I subunit	-1.016728177	1.357870198	4.354968	4.287883	5.570089	4.35472906	4.33197089	4.77742372	764	1004	1276
4439	NM_002441,NM_025259,NM_17	MSH5	mutS homolog 5	1.047119756	1.357202367	8.156179	8.54863	10.440279	4.54461902	4.60826865	4.9689398	877	1227	1464
54531	NM_001346105,NM_017550	MIER2	MIER family member 2	1.212567464	1.357035271	12.845679	15.576647	16.303282	5.1647037	5.43572001	5.59457146	1369	2218	2285
57205	NM_020453	ATP10D	ATPase phospholipid transportin	1.082975767	1.35694194	3.993336	4.330075	5.092499	4.72634668	4.83717724	5.15233896	1000	1447	1669
10015	NM_001162429,NM_001256192	PDCD6IP	programmed cell death 6 interact	-1.003915539	1.356832936	17.405471	17.467688	22.418715	6.63139796	6.62581688	7.06781988	3853	5128	6430
29916	NM_001330320,NM_013323,NM	SNX11	sorting nexin 11	-1.125992358	1.356812936	7.644618	7.349953	10.523525	4.34933082	4.18699961	4.7708724	761	903	1270
3612	NM_001144878,NM_001144879	IMPA1	inositol monophosphatase 1	-1.2382232	1.356528433	7.058509	5.717184	8.954184	4.59718428	4.30300447	5.02139743	911	983	1520
105369306	NR_147887	LOC105369306	uncharacterized LOC105369306	-1.203087877	1.356502318	1.8197	1.510648	2.32613	2.36507395	2.15396266	2.72964665	163	181	272
1308	NM_000494	COL17A1	collagen type XVII alpha 1 chain	1.213626389	1.355901318	26.084932	31.691744	33.23843	7.16475992	7.44231817	7.60137806	5594	9071	9329
25914	NM_001318520,NM_173630	RTTN	rotatin	-1.180419203	1.355455678	7.703123	6.522485	9.794019	5.75775028	5.52324054	6.189539	2085	2360	3476
57655	NM_001136199,NM_001320034	GRAMD1A	GRAM domain containing 1A	-1.211225032	1.35542324	14.740632	12.221954	18.652437	5.27795218	5.00929679	5.70694011	1484	1637	2474
2776	NM_002072	GNAQ	G protein subunit alpha q	1.068443342	1.355334812	8.283759	8.8623	10.553429	5.70518879	5.79893152	6.1365907	2009	2868	3349
4000	NM_001257374,NM_001282624	LMNA	lamin A/C	1.270081788	1.355097934	193.79959	233.179842	233.216878	8.81608088	9.16032331	9.25364137	17656	29962	29427
5970	NM_001145138,NM_001243984	RELA	RELA proto-oncogene, NF-kB su	1.186298616	1.355067739	24.057684	28.545805	30.471335	5.91400808	6.15672319	6.3461079	2328	3690	3880
8635	NM_003730	RNASET2	ribonuclease T2	1.002803673	1.35499813	9.596887	9.635845	12.219518	3.66576097	3.66948316	4.07404468	459	615	765
25999	NM_001199570,NM_015526	CLIP3	CAP-Gly domain containing linke	-1.223270745	1.354627854	0.658049	0.53601	0.829367	1.6852613	1.49109792	2.0010274	87	95	145
6426	NM_001078166,NM_006924,NR	SRSF1	serine and arginine rich splicing f	-1.120022847	1.35458485	91.028821	79.944797	116.004343	8.79712343	8.63398342	9.23412741	17425	20787	29031
53630	NM_017429	BCO1	beta-carotene oxygenase 1	-1.819322514	1.354422595	0.636378	0.355183	0.822601	1.33814853	0.8787583	1.61867597	60	44	100
55664	NM_017913	CDC37L1	cell division cycle 37 like 1	1.163983924	1.354321349	3.023526	3.524415	3.854589	3.50674676	3.70787447	3.91081999	407	633	678
9820	NM_001168370,NM_014780	CUL7	cullin 7	-1.055556225	1.354219061	6.168549	5.851963	7.84879	5.09006999	5.01441136	5.51644141	1298	1643	2162
103504738	NR_128720_chr1,NR_128720_c	MIR6859-4	microRNA 6859-4	1.246657524	1.35398247	13.582571	17.035292	17.518246	0.93882015	1.10017522	1.16514558	36	60	60
326625	NM_052845,NR_038118	MMAB	methylmalonic aciduria (cobalam	1.384010683	1.353946453	3.214487	4.454259	4.076966	3.80897562	4.24906335	4.21906166	511	945	851
101927481	NR_126336	LINC01909	long intergenic non-protein codin	1.122215709	1.353762817	0.957589	1.065839	1.209033	0.81895953	0.89363826	1.02535867	30	45	50
100126351	NR_030635	MIR939	microRNA 939	-1.144656771	1.353762817	9.436645	8.267582	12.036478	0.81895953	0.73744528	1.02535867	30	35	50
642273	NM_001077710	FAM110C	family with sequence similarity 1	1.13772114	1.353746313	6.873803	7.831187	8.747992	4.78350351	4.96331284	5.20674668	1042	1584	1735
85012	NM_001006933,NM_032926	TCEAL3	transcription elongation factor A	1.34151136	1.353537846	8.774952	11.802491	11.16694	3.40287721	3.79168578	3.80364308	376	674	626
65991	NM_023934	FUNDC2	FUN14 domain containing 2	1.189247837	1.353417643	5.948605	7.078254	7.565507	4.18329013	4.4206818	4.59907035	674	1071	1122
25778	NM_015375,NM_199462	DSTYK	dual serine/threonine and tyrosin	-1.125958576	1.353326079	2.637619	2.340522	3.35154	4.40589631	4.24326622	4.82457876	793	941	1320
259230	NM_147156	SGMS1	sphingomyelin synthase 1	-1.477519505	1.353226581	2.176498	1.473875	2.772444	3.16550307	2.67693481	3.55943664	313	283	521
114827	NM_052929	FHAD1	forkhead associated phosphopep	2.522661822	1.353199244	0.107051	0.271374	0.13382	0.61813834	1.23481109	0.78645063	21	71	35
4357	NM_001013436,NM_001130517	MPST	mercaptopyruvate sulfurtransfera	1.131888478	1.353019324	58.42086	66.291876	73.775035	6.29196733	6.46855802	6.72335158	3037	4593	5054
55147	NM_001077351,NM_001077352	RBM23	RNA binding motif protein 23	1.263620176	1.352670868	7.043927	8.956074	9.030169	4.21529532	4.53660777	4.63077151	690	1165	1148
1376	NM_000098,NM_001330589	CPT2	carnitine palmitoyltransferase 2	1.325650182	1.352595105	5.654753	7.519374	7.221189	4.17314113	4.56011536	4.58793254	669	1185	1113
1778	NM_001376	DYNC1H1	dynein cytoplasmic 1 heavy chair	-1.005473565	1.352451811	32.136715	31.997622	40.843613	8.81281599	8.80495835	9.24755928	17616	23409	29303
100131655	NR_040024	LOC100131655	uncharacterized LOC100131655	1.394264822	1.352450602	0.295626	0.409629	0.379608	0.46682716	0.61708849	0.60191771	15	28	25
84054	NR_001282	PCDHB19P	protocadherin beta 19 pseudoger	-1.427196617	1.352450602	0.083913	0.059306	0.103788	0.46682716	0.3412894	0.60191771	15	14	25
3660	NM_002199	IRF2	interferon regulatory factor 2	-1.080791827	1.352402161	12.884614	11.931904	16.368429	4.89144191	4.78326433	5.31428079	1126	1392	1873
7165	NM_001243891,NM_001243892	TPD52L2	tumor protein D52 like 2	1.06911102	1.352401641	59.68232	63.908703	76.204096	7.10568758	7.20142381	7.53848699	5368	7668	8929
22893	NM_001301132,NM_014952,NR	BAHD1	bromo adjacent homology domai	1.086768883	1.352384774	5.932151	5.72469	7.704832	4.6077728	4.72309898	5.02782252	918	1333	1527
26084	NM_001251962,NM_001251963	ARHGEF26	Rho guanine nucleotide exchang	1.092758915	1.352336894	0.561286	0.623862	0.711685	1.9554003	2.05153568	2.29082241	113	165	188
83860	NM_031923	TAF3	TATA-box binding protein associ	1.690090557	1.352185061	2.32704	3.942003	2.960033	3.08551061	3.77168848	3.47597349	294	664	489
168455	NM_175884	CCDC71L	coiled-coil domain containing 71	-1.768806146	1.352159461	2.536501	1.434061	3.221391	4.1588115	3.39667017	4.5729471	662	500	1101
100128822	NR_027387	LINC01003	long intergenic non-protein codin	-1.36181364	1.352099347	1.899863	1.402611	2.427172	1.86749926	1.55800914	2.19620105	104	102	173
51322	NM_016628,NM_100264,NM_10	WAC	WW domain containing adaptor v	1.118251954	1.352059816	22.903343	25.895865	29.282495	6.96070103	7.12072434	7.3928496	4851	7248	8067
114879	NM_001144063,NM_020896,NM	OSBPL5	oxysterol binding protein like 5	1.385519446	1.351865146	5.809913	8.061422	7.35612	4.50794215	4.96066562	4.92634185	854	1581	1420
100288428	NR_033378	LMCD1-AS1	LMCD1 antisense RNA 1 (head t	-1.451716328	1.351798139	0.141246	0.099028	0.183747	0.38477185	0.27464693	0.49997203	12	11	20
56171	NM_018897	DNAH7	dynein axonemal heavy chain 7	2.419750745	1.351798139	0.023516	0.060258	0.031025	0.38477185	0.80196545	0.49997203	12	39	20
3008	NM_005321	HIST1H1E	histone cluster 1 H1 family memb	2.172477755	1.351798139	0.399588	0.866541	0.49186	0.38477185	0.73744528	0.49997203	12	35	20
8411	NM_003566	EEA1	early endosome antigen 1	-1.19454522	1.351439652	2.949388	2.472091	3.748782	4.58498718	4.3401413	5.00381225	903	1010	1501
51397	NM_001308080,NM_016144,NR	COMMD10	COMM domain containing 10	-1.002615856	1.351358257	16.574255	16.424786	21.281116	4.66393542	4.66031492	5.08351504	956	1274	1589
23633	NM_012316	KPNA6	karyopherin subunit alpha 6	1.118346593	1.351239218	12.039909	13.478674	15.287772	6.44994873	6.60957348	6.87994812	3393	5070	5639
56101	NM_018925,NM_032099	PCDHGB5	protocadherin gamma subfamily	1.144588559	1.351232079	4.566518	5.248386	5.830045	4.39021014	4.5763457	4.80654202	784	1199	1303
157570	NM_001017420	ESCO2	establishment of sister chromatid	1.468008748	1.351170421	2.235332	3.282753	2.836098	3.05488459	3.55246622	3.44338646	287	563	477
54553	NR_033797	LINC02486	long intergenic non-protein codin	1.380957208	1.351043746	2.417047	3.346709	3.074259	3.01452812	3.43022535	3.40158951	278	513	462
7126	NM_021137	TNFAIP1	TNF alpha induced protein 1	-1.559579093	1.35079621	13.078803	8.393852	16.607798	5.6300111	5.00502076	6.05625745	1905	1632	3165
106144532	NR_132369,NR_132370	ARF4-AS1	ARF4 antisense RNA 1	1.244483702	1.350716051	0.416124	0.586444	0.526778	0.29776659	0.36283659	0.39027104	9	15	15
166968	NM_001297598,NM_001297599	MIER3	MIER family member 3	-1.219258126	1.350705805	8.43265	6.929137	10.710455	5.45257769	5.17375987	5.87773632	1680	1841	2791
10541	NM_006401	ANP32B	acidic nuclear phosphoprotein 32	1.201691515	1.350538819	127.525078	153.423416	161.85994	7.65579107	7.91965971	8.08747343	7878	12649	13086
4645	NM_001080467	MYO5B	myosin VB	-1.537060683	1.350498541	6.029975	3.92688	7.653355	5.830587	5.22392723	6.25750353	2195	1908	3646
83541	NM_001042353,NM_001289145	FAM110A	family with sequence similarity 1	1.923703376	1.350347474	11.606282	20.884766	13.846517	4.33301385	5.24220835	4.7477056	752	1933	1249
9179	NM_004722	AP4M1	adaptor related protein complex 4	-1.283904232	1.350257681	4.828869	3.758549	6.129167	3.29094516	2.97154911	3.68554025	345	359	573
65996	NR_026052	CENPBD1P1	CENPB DNA-binding domains cc	-1.656889201	1.349919501	1.285965	0.777115	1.634052	2.37926962	1.82188956	2.73862739	165	133	274

8192	NM_006012	CLPP	caseinolytic mitochondrial matrix	1.144513134	1.349916089	28.764139	32.95348	36.500664	5.05512721	5.2443866	5.47673694	1266	1936	2102
23423	NM_001301203,NM_001330376	TMED3	transmembrane p24 trafficking pr	1.007851727	1.349829183	61.565518	62.505504	78.557264	6.49993314	6.5110927	6.92858469	3514	4732	5834
1522	NM_001336	CTSZ	cathepsin Z	1.046841506	1.349786859	205.317978	215.180664	260.434387	8.23300294	8.29883199	8.66449497	11773	16467	19545
5315	NM_001206796,NM_001206797	PKM	pyruvate kinase, muscle	1.547195358	1.349523029	524.309688	812.298933	664.458695	10.44989874	11.07919028	10.88208194	54875	113440	91083
3099	NM_000189	HK2	hexokinase 2	1.244130463	1.349506338	20.632061	25.695475	26.163498	7.16475992	7.47792875	7.59459273	5594	9299	9285
4942	NM_000274,NM_001171814,NM	OAT	ornithine aminotransferase	1.129096688	1.349430239	24.842661	28.038775	31.277056	5.67888953	5.85084386	6.10394999	1972	2975	3273
81544	NM_001351167,NM_001351168	GDPD5	glycerophosphodiester phosphoc	1.680996882	1.349403353	0.940844	1.924974	1.219757	2.26923253	2.89227137	2.62213795	150	337	249
1385	NM_001320793,NM_004379,NM	CREB1	cAMP responsive element bindin	1.27557873	1.349326219	5.117901	6.308693	6.07697	5.43059347	5.77452033	5.85417145	1654	2819	2745
90580	NM_138358	TIMM29	translocase of inner mitochondria	1.211638269	1.349242787	9.684584	11.749907	12.276972	3.82462152	4.08373346	4.23024804	517	837	858
2936	NM_000637,NM_001195102,NM	GSR	glutathione-disulfide reductase	1.101249996	1.349214423	9.953008	11.141629	12.796201	4.98839509	5.1233639	5.40873708	1207	1776	2003
1464	NM_001897	CSPG4	chondroitin sulfate proteoglycan 4	-2.019783358	1.34919643	7.717341	3.8253	9.786203	5.9841867	4.99298	6.41039332	2446	1618	4059
9618	NM_004295	TRAF4	TNF receptor associated factor 4	-1.090190658	1.349152001	60.182709	55.259144	76.301254	7.41823311	7.29441109	7.84810778	6676	8182	11078
51696	NM_016217	HECA	hdc homolog, cell cycle regulator	-1.288721432	1.349137655	2.784089	2.165033	3.531493	4.02300634	3.68239411	4.43196063	599	621	994
255520	NM_153702	ELMOD2	ELMO domain containing 2	1.159439945	1.34900937	5.063364	5.877748	6.421902	4.50794215	4.71264808	4.92339112	854	1323	1417
5164	NM_001199898,NM_001199899	PDK2	pyruvate dehydrogenase kinase 2	1.078864702	1.348899404	9.598519	9.468127	11.546781	4.40935908	4.51391509	4.82352399	795	1146	1319
4784	NM_001271043,NM_001271044	NFIX	nuclear factor I X	1.006451348	1.348742027	3.823733	3.860276	4.854166	4.44859483	4.45744984	4.86307268	818	1100	1357
101928994	NR_120648	LOC101928994	uncharacterized LOC101928994	-1.002055038	1.348571725	0.081204	0.080522	0.101288	0.20517559	0.20477631	0.27153692	6	8	10
10578	NM_001302758,NM_006433,NM	GNLY	granulysin	3.694120386	1.348571725	0.144166	0.693653	0.175328	0.20517559	0.65251278	0.27153692	6	30	10
973	NM_001783,NM_021601	CD79A	CD79a molecule	1.24305565	1.348571725	0.112833	0.155507	0.152734	0.20517559	0.25173066	0.27153692	6	10	10
8355	NM_003534	HIST1H3G	histone cluster 1 H3 family memb	1.855821834	1.348571725	0.331127	0.598434	0.421658	0.20517559	0.36283659	0.27153692	6	15	10
340156	NM_001012418,NM_001347872	MYLK4	myosin light chain kinase family r	2.346034781	1.348571725	0.026841	0.060333	0.032623	0.20517559	0.44594451	0.27153692	6	19	10
9896	NM_014845	FIG4	FIG4 phosphoinositide 5-phospha	1.10488966	1.348570033	3.549026	3.932012	4.499996	3.55444243	3.68667221	3.95387191	422	623	700
25942	NM_001145357,NM_001145358	SIN3A	SIN3 transcription regulator famil	1.241508004	1.347891741	7.868998	9.832492	10.109958	5.7268601	6.03365933	6.15053688	2040	3384	3382
57609	NM_173602	DIP2B	disco interacting protein 2 homolo	-1.236820221	1.347779985	7.364295	5.96071	9.329525	5.98882325	5.68754276	6.41355113	2454	2651	4068
196264	NM_001286152,NM_198275,NR	MPZL3	myelin protein zero like 3	-1.583516077	1.347704992	7.261922	4.670874	9.293698	4.86772434	4.23306486	5.28546063	1107	934	1835
9371	NM_004798	KIF3B	kinesin family member 3B	1.240133563	1.347676214	7.220258	8.965536	9.147854	5.45927522	5.76342439	5.88128877	1688	2797	2798
79176	NM_024326	FBXL15	F-box and leucine rich repeat pro	-1.223098286	1.347638328	2.868877	2.348954	3.647141	2.93026199	2.6812291	3.31117052	260	284	431
5604	NM_002755	MAP2K1	mitogen-activated protein kinase	1.203361839	1.347630743	21.381927	25.762238	27.081038	5.77731246	6.03994294	6.20095573	2114	3399	3504
64979	NM_032479	MRPL36	mitochondrial ribosomal protein L	1.333877754	1.347554669	29.343163	39.187908	37.148022	4.21331566	4.6093958	4.62351744	689	1228	1142
155435	NM_053043	RBM33	RNA binding motif protein 33	-1.231626381	1.34730252	8.570033	6.967326	10.851577	6.44573925	6.14899263	6.87155263	3383	3670	5606
29763	NM_001184974,NM_001184975	PACSIN3	protein kinase C and casein kina	1.037311602	1.347269374	16.024045	16.781598	20.596219	4.96152109	5.01270852	5.37960921	1184	1641	1962
6396	NM_001136026,NM_001136232	SEC13	SEC13 homolog, nuclear pore ar	1.055346192	1.347170555	43.703117	46.414044	55.363194	5.95841587	6.03491824	6.38237404	2402	3387	3980
842	NM_001229,NM_001278054,NM	CASP9	caspase 9	1.000150034	1.347097991	3.968297	3.839483	5.00494	3.62762016	3.62781914	4.02716981	446	596	739
18	NM_000663,NM_001127448,NM	ABAT	4-aminobutyrate aminotransferas	1.538812851	1.34678609	0.323662	0.503047	0.413415	1.47662927	1.90501868	1.76650173	70	144	116
4772	NM_001278669,NM_001278670	NFATC1	nuclear factor of activated T-cells	1.217193121	1.346687536	0.997825	1.149174	1.684258	2.38631541	2.61991364	2.74309688	166	270	275
84914	NM_001204817,NM_032828	ZNF587	zinc finger protein 587	1.164566167	1.346463452	11.035175	12.874856	13.971798	6.23992124	6.45702128	6.66419028	2928	4556	4849
7182	NM_001291694,NM_003298	NR2C2	nuclear receptor subfamily 2 grou	1.141616165	1.346452143	5.058031	5.782603	6.415996	5.4160359	5.60292734	5.83650316	1637	2497	2711
23710	NM_031412	GABARAPL1	GABA type A receptor associatec	-1.517310926	1.346392397	13.602221	8.966554	17.244645	4.68551669	4.1126379	5.10016049	971	855	1608
60673	NM_001098673,NM_021934	ATG101	autophagy related 101	1.337331662	1.346161613	18.872357	25.754557	24.096198	4.77681668	5.18287427	5.19211053	1037	1853	1717
139231	NM_207318	FAM199X	family with sequence similarity 19	1.337107271	1.346100884	13.511222	18.08576	17.092484	6.65820534	7.07372461	7.08332505	3926	7014	6500
88455	NM_033121	ANKRD13A	ankyrin repeat domain 13A	-1.376792825	1.345972395	11.769155	8.558522	14.893944	5.51739157	5.06786744	5.93794468	1759	1707	2912
304	NR_003573	ANXA2P2	annexin A2 pseudogene 2	-1.071113888	1.345925345	1.84252	1.721444	2.344075	1.75224037	1.68318474	2.06668624	93	116	154
6334	NM_001177984,NM_001330260	SCN8A	sodium voltage-gated channel alp	1.761566078	1.345925345	0.209017	0.370481	0.26483	1.75224037	2.37153003	2.06668624	93	219	154
8694	NM_012079	DGAT1	diacylglycerol O-acyltransferase	-1.307081442	1.345788147	18.67411	14.305667	23.619785	6.10174952	5.72181948	6.52480862	2657	2716	4398
474343	NM_001006681,NM_001006682	SPIN2B	spindlin family member 2B	1.338463617	1.345620408	1.977555	2.620333	2.384488	1.7191395	2.02475093	2.03057861	90	161	149
199964	NM_182532	TMEM61	transmembrane protein 61	-1.036601541	1.345620408	1.910846	1.851084	2.425733	1.7191395	1.68318474	2.03057861	90	116	149
126231	NM_001172689,NM_001172690	ZNF573	zinc finger protein 573	-1.290891727	1.34507242	0.613735	0.463462	0.847935	1.30878272	1.10017522	1.57922529	58	60	96
221656	NM_153042	KDM1B	lysine demethylase 1B	-1.65710483	1.344633102	1.77149	1.068615	2.241975	2.94465259	2.33383942	3.32316088	263	212	435
1609	NM_001347	DGKQ	diacylglycerol kinase theta	1.153762061	1.344613843	8.328218	9.623545	10.526719	5.27320884	5.47458779	5.69083514	1479	2280	2446
9703	NM_001321560,NM_001321561	KIAA0100	KIAA0100	1.165237218	1.34461376	19.553742	22.554677	24.390789	7.12450759	7.34366839	7.54905472	5439	8468	8995
27236	NM_001025593,NM_001025595	ARFIP1	ADP ribosylation factor interactin	1.026047485	1.344362611	13.576172	14.097761	17.209467	5.38031	5.41653033	5.79835805	1596	2188	2639
4289	NM_001145354,NM_001321316	MKLN1	muskelin 1	-1.151053584	1.344333293	4.958933	4.313944	6.266245	5.78199488	5.58298421	6.20217361	2121	2462	3507
64400	NM_001012398,NM_001308325	AKTIP	AKT interacting protein	-1.248010986	1.344308115	13.279355	10.623782	16.776529	5.04959013	4.74069359	5.46528578	1261	1350	2085
2065	NM_001005915,NM_001982	ERBB3	erb-b2 receptor tyrosine kinase 3	-1.406087172	1.344298222	19.299832	13.69108	25.12027	6.64798186	6.16211003	7.07115648	3898	3704	6445
54811	NM_001130031,NM_001130032	ZNF562	zinc finger protein 562	1.206618082	1.344201284	4.063948	4.960428	5.160552	4.58038655	4.8410207	4.99165559	900	1451	1488
6227	NM_001024	RPS21	ribosomal protein S21	-1.182578673	1.343960797	676.167969	572.440063	853.99469	8.03189283	7.79096	8.45697646	10236	11565	16920
2533	NM_001243093,NM_001349333	FYB	FYN binding protein	-1.508817593	1.343919626	0.257806	0.170468	0.326017	1.21691895	0.90836632	1.47560397	52	46	86
23259	NM_001164232,NM_001164234	DDHD2	DDHD domain containing 2	-1.280503517	1.343710071	7.393155	5.797106	9.216514	5.08141332	4.73657294	5.49672576	1290	1346	2132
3621	NM_001267728,NM_005537,NM	ING1	inhibitor of growth family member	-1.07308082	1.343615668	6.560209	6.662993	7.671439	4.16497021	4.06906113	4.57043432	665	828	1099
11113	NM_001206999,NM_007174	CIT	citron rho-interacting serine/threc	-1.309784697	1.343300855	14.836591	11.308572	18.685788	6.98236854	6.59655946	7.40524048	4925	5024	8137
5991	NM_001282116,NM_001282117	RFX3	regulatory factor X3	1.293547576	1.343291106	1.051741	1.268718	1.297348	3.11973375	3.45300696	3.50257812	302	522	499
22997	NM_001277285	IGSF9B	immunoglobulin superfamily men	-1.97969436	1.343237602	0.264926	0.133426	0.336536	1.16869613	0.70406985	1.42086173	49	33	81
79670	NM_001185059,NM_001185074	ZCCHC6	zinc finger CCHC-type containing	-1.109955201	1.343228042	11.264606	10.275505	14.30235	5.98592715	5.83791964	6.4058198	2449	2948	4046
9326	NM_001281432,NM_001281433	ZNHIT3	zinc finger HIT-type containing 3	-1.055074032	1.343091106	35.974197	34.627869	46.577466	5.15442263	5.07930153	5.56962461	1359	1721	2245
389677	NM_203390	RBM12B	RNA binding motif protein 12B	1.281395607	1.343018696	6.463021	8.294336	8.161091	5.04959013	5.39773447	5.46393259	1261	2159	2083
57572	NM_020812	DOCK6	dedicator of cytokinesis 6	-1.196632519	1.34300696	10.832503	9.062869	13.675363	6.09853551	5.84367804	6.51863045	2651	2960	4379
51742	NM_001206794,NM_016374,NM	ARID4B	AT-rich interaction domain 4B	1.258467098	1.34287464	8.400358	10.677006	10.555807	5.63519596	5.96090566	6.05311118	1912	3215	3158
54882	NM_001197030,NM_017747,NM	ANKHD1	ankyrin repeat and KH domain co	-1.018457731	1.342711244	28.538012	25.830909	46.32218	7.29214081	7.26592413	7.71494524	6114	8021	10097
26123	NM_001143973,NM_015631	TCTN3	tectonic family member 3	-1.153946807	1.342595789	13.939153	12.070727	17.555884	5.25215086	5.05137419	5.66751164	1457	1687	2406
100616318	NR_039810	MIR4664	microRNA 4664	2.443296723	1.34229324	0.744234	2.760792	1.335153	0.10623215	0.25173066	0.14214747	3	10	5
100616378	NR_039945	MIR4784	microRNA 4784	2.202411351	1.34229324	0.692969	2.091982	1.241395	0.10623215	0.2284445	0.14214747	3	9	5
100128573	NR_024491	LOC100128573	uncharacterized LOC100128573	2.925067466	1.34229324	0.101142	0.445449	0.18471	0.10623215	0.29720487	0.14214747	3	12	5
338069	NR_002329	ST7-OT4	ST7 overlapping transcript 4	1.479755237	1.34229324	0.049267	0.073832	0.06066	0.10623215	0.15624221	0.14214747	3	6	5
8302	NM_013431	KLRC4	killer cell lectin like receptor C4	3.165952837	1.34229324	0.057549	0.2725	0.086156	0.10623215	0.31941552	0.14214747	3	13	5
137196	NR_130917,NR_130918,NR_13(	CCDC26	CCDC26 long non-coding RNA	1.238869866	1.34229324	0.041644	0.055775	0.057712	0.10623215	0.13134883	0.14214747	3	5	5
102465480	NR_106858	MIR6800	microRNA 6800	2.202411351	1.34229324	0.730089	2.146195	1.011961	0.10623215	0.2284445	0.14214747	3	9	5
407011	NR_029664	MIR23B	microRNA 23b	1.238869866	1.34229324	0.761046	0.982382	0.971156	0.10623215	0.13134883	0.14214747	3	5	5
441501	NR_034005	LINC00891	long intergenic non-protein codin	3.64772358	1.34229324	0.068827	0.265009	0.088271	0.10623215	0.36283659	0.14214747	3	15	5
100423011	NR_036090	MIR3138	microRNA 3138	1.479755237	1.34229324	0.773909	1.207054	0.977451	0.10623215	0.15624221	0.14214747	3	6	5
83955	NR_002182	NACAP1	nascent polypeptide associated c	3.165952837	1.34229324	0.103965	0.334594	0.125273	0.10623215	0.31941552	0.14214747	3	13	5
102465481	NR_106860	MIR6802	microRNA 6802	1.238869866	1.34229324	1.159624	1.355542	1.402071	0.10623215	0.13134883	0.14214747	3	5	5
153090	NM_032552,NM_138709	DAB2IP	DAB2 interacting protein	-1.702447292	1.342107787	12.028261	7.159838	15.284309	5.92251507	5.17147225	6.34095711	2342	1838	3866
117177	NM_001024647,NM_001278402	RAB3IP	RAB3A interacting protein	-1.89003301	1.342085741	2.672949	1.403323	3.329887	4.67260671	3.80355245	5.08263362	962	680	1588
6624	NM_003088	FSCN1	fascin actin-bundling protein 1	1.843965954	1.341843403	11.132874	20.548548	14.037709	4.9685801	5.83020585	5.38104381	1190	2932	1964
102723099	NR_130107	GHET1	gastric carcinoma proliferation en	1.170668524	1.341636225	0.309107	0.370666	0.389759	0.66524311	0.75384785	0.83750224	23	36	38
150468	NM_001304361,NM_152515,NR	CKAP2L	cytoskeleton associated protein 2	1.323032101	1.341508801	8.612725	11.156481	10.919895	5.31165148	5.70662732	5.72625748	1520	2687	2508
11101	NM_001001976,NM_001288734	ATE1	arginyltransferase 1	1.041290496	1.341450772	2.367345	2.499838	3.014579	3.66865353	3.7225328	4.0633618	460	640	759
1642	NM_001923	DDB1	damage specific DNA binding pro	1.401536176	1.340955418	37.106083	52.064701	46.762138	7.30730079	7.79170491	7.72825082	6179	11571	10191
283358	NM_173593	B4GALNT3	beta-1,4-N-acetyl-galactosaminyl	-1.978152671	1.340943984	4.679996	2.367517	5.90211	4.06092955	3.15872433	4.46209528	616	416	1016
60485	NM_021818	SAV1	salvador family WW domain cont	-1.129090992	1.340920606	9.901117	8.77915	12.475204	4.967406	4.79816878	5.37889138	1189	1407	1961
5364	NM_001130082,NM_002673	PLXNB1	plexin B1	-1.139395739	1.340810148	12.912375	11.369689	16.390205	6.53086276	6.34476118	6.95000779	3591	4211	5922
255252	NM_153260	LRRC57	leucine rich repeat containing 57	-1.053052297	1.34079874	4.746923	4.513961	5.991358	3.60665736	3.53833258	3.99941726	439	557	724
55624	NM_001243766,NM_001290129	POMGNT1	protein O-linked mannose N-acet	-1.043574617	1.340673961	27.566254	25.597789	33.769123	6.16917354	6.10851071	6.58704326	2786	3567	4594
51449	NM_016297	PCYOX1	prenylcysteine oxidase 1	1.365670857	1.340575428	7.826363	10.702934	9.860951	5.39173379	5.83213817	5.80585457	1609	2936	2653
55171	NM_001134415,NM_001286749	TBCCD1	TBCC domain containing 1	1.429164559	1.340574034	6.177062	9.303205	7.723124	4.18531139	4.67653783	4.58793254	675	1289	1113
381	NM_001662	ARF5	ADP ribosylation factor 5	-1.210051977	1.340437711	56.590275	46.813583	71.287514	5.93997869	5.669824	6.35671927	2371	2618	3909
665	NM_001330491,NM_004331	BNIP3L	BCL2 interacting protein 3 like	1.462536543	1.34000987	21.507377	31.511184	27.096277	6.21589832	6.75823649	6.63322286	2879	5626	4745
5050	NM_001145939,NM_001145940	PAFAH1B3	platelet activating factor acetylhy	-1.018871596	1.339750477	31.064067	30.479509	39.060986	5.06504071	5.03887932	5.47606585	1275	1672	2101
128153	NM_138796	SPATA17	spermatogenesis associated 17	2.564425892	1.339406862	0.414697	1.08614	0.534253	0.95786335	1.77444288	1.17840727	37	127	61

90326	NM_001195752,NM_001195753	THAP3	THAP domain containing 3	-1.08582239	1.339160045	10.819263	9.505793	12.99056	3.94164205	3.83086756	4.33906229	564	694	929
162394	NM_001330183,NM_144975	SLFN5	schlafen family member 5	1.439850893	1.338826831	6.408112	8.902375	7.960252	5.53889193	6.05532648	5.95200904	1786	3436	2941
55139	NM_001042410,NM_001282792	ANKZF1	ankyrin repeat and zinc finger do	1.574369788	1.338591757	19.211106	30.356217	24.383989	5.62033234	6.26439661	6.03363206	1892	3980	3115
4320	NM_005940,NR_133013	MMP11	matrix metallopeptidase 11	-1.322300293	1.338276492	1.126826	0.864435	1.421261	1.81624731	1.5392062	2.1294865	99	100	163
116039	NM_001142462,NM_001286841	OSR2	odd-skipped related transciption f	-1.733626084	1.338086888	5.021248	2.90545	6.230226	3.32067256	2.62883416	3.70399851	353	272	581
1645	NM_001353	AKR1C1	aldo-keto reductase family 1 mem	2.34671552	1.337824198	1.230961	2.890373	1.541921	1.40904982	2.2895231	1.6852207	65	204	107
56985	NM_020233	ADPRM	ADP-ribose/CDP-alcohol diphosp	1.196151196	1.337714782	2.172513	2.619891	2.746402	2.0823043	2.28388661	2.41366048	127	203	209
57659	NM_001128833,NM_020899	ZBTB4	zinc finger and BTB domain conta	1.4018411	1.337573333	6.275826	8.858393	7.889187	5.22194666	5.69817645	5.63180377	1426	2671	2346
102724562	NR_135824	LOC102724562	macrophage stimulating 1 pseudo	-40.71005012	1.337447327	3.608077	0.079112	4.535214	2.87121968	0.20477631	3.24022233	248	8	408
146923	NM_001321381,NM_173079	RUNDC1	RUN domain containing 1	1.269913706	1.337413583	4.482417	5.703362	5.639965	4.59109862	4.9230853	4.99540703	907	1539	1492
50856	NM_016184,NM_194447,NM_19	CLEC4A	C-type lectin domain family 4 me	-1.30795592	1.337358329	0.969532	0.741305	1.221131	1.15225689	0.95166931	1.39836968	48	49	79
2909	NM_004491	ARHGAP35	Rho GTPase activating protein 35	1.28178265	1.337354825	7.382986	9.475414	9.279527	6.02030239	6.37357278	6.43408293	2509	4297	4127
5290	NM_006218	PIK3CA	phosphatidylinositol-4,5-bisphosp	-1.017265315	1.337327569	4.000492	3.937567	5.027444	5.18706741	5.16305312	5.5964252	1391	1827	2288
126433	NM_178820	FBXO27	F-box protein 27	-1.725667047	1.337104549	4.360898	2.529292	5.4859	3.46409533	2.76857683	3.84996236	394	305	648
2762	NM_001253846,NM_001500	GMDS	GDP-mannose 4,6-dehydratase	1.009663102	1.337013513	7.171725	7.258021	9.021147	3.6887407	3.70154644	4.07935661	467	630	768
57121	NM_001142961,NM_020400	LPAR5	lysophosphatidic acid receptor 5	-1.24887343	1.336951565	2.753078	2.201846	3.478174	3.1155	2.83560729	3.49199507	301	322	495
10403	NM_006101	NDC80	NDC80, kinetochore complex com	1.076123001	1.336815777	33.613659	36.212132	42.226631	6.17172449	6.276155	6.58548856	2791	4013	4589
84749	NM_001301175,NM_032663	USP30	ubiquitin specific peptidase 30	-1.308785243	1.336782804	3.948763	3.167121	5.454067	4.07843661	3.71626888	4.47558749	624	637	1026
1759	NM_001005336,NM_001288737	DNM1	dynamin 1	1.289161142	1.336730498	10.629196	14.089876	13.816069	5.36790544	5.72650194	5.77781179	1582	2725	2601
4354	NM_001166460,NM_001166461	MPP1	membrane palmitoylated protein 1	1.199345805	1.336722318	3.947588	4.778851	5.00847	3.16140171	3.39667017	3.53901915	312	500	513
10618	NM_001206840,NM_001206841	TGOLN2	trans-golgi network protein 2	1.481463128	1.336455288	18.830477	27.917078	23.696381	6.87532501	7.43835948	7.29064849	4570	9046	7512
9276	NM_004766,NR_023350	COPB2	coatomer protein complex subun	1.624139534	1.336227876	54.667015	88.88889	68.5995	7.38586173	8.08222768	7.80186185	6527	14164	10727
8767	NM_003821	RIPK2	receptor interacting serine/threon	-1.250674354	1.336225843	16.889671	13.513658	21.203936	5.44331753	5.12887652	5.85313811	1669	1783	2743
23151	NM_015124	GRAMD4	GRAM domain containing 4	-1.234858567	1.336151025	4.261901	3.454954	5.353116	4.29607932	4.0088325	4.69562066	732	792	1203
55707	NM_001145277,NM_001145278	NECAP2	NECAP endocytosis associated 2	1.096508852	1.335920432	16.373995	18.021894	20.591822	5.09545415	5.22466294	5.5026688	1303	1909	2141
84885	NM_001318015,NM_001318016	ZDHHC12	zinc finger DHHC-type containing	1.029583166	1.33564164	56.669626	57.839516	70.595954	6.01349256	6.05491286	6.42541866	2497	3435	4102
1535	NM_000101	CYBA	cytochrome b-245 alpha chain	1.404627317	1.335599652	259.258789	364.555298	325.370544	7.51964009	8.00756709	7.93515649	7165	13447	11770
100131187	NM_001113205,NM_001113206	TSTD1	thiosulfate sulfurtransferase like d	-1.180387513	1.335490652	29.773151	26.207652	37.387831	4.13809052	3.91349545	4.53478801	652	738	1071
55193	NM_001350074,NM_001350075	PBRM1	polybromo 1	1.350996428	1.335453151	2.614219	3.523691	3.286848	4.37435155	4.79023891	4.77415178	775	1399	1273
3188	NM_001032393,NM_019597	HNRNPH2	heterogeneous nuclear ribonucle	1.28353737	1.33485937	38.565814	49.565664	48.393088	6.50479717	6.86141835	6.91750551	3526	6047	5789
488	NM_001681,NM_170665	ATP2A2	ATPase sarcoplasmic/endoplasm	-1.002618424	1.334852936	34.847224	34.74936	43.480307	8.12828695	8.12452776	8.54367745	10946	14587	17971
10712	NM_001267608,NM_006589,NM	FAM189B	family with sequence similarity 18	-1.117044888	1.334848938	10.570605	9.443935	13.028858	5.01931705	4.86481307	5.42482324	1234	1476	2026
9727	NM_001142272,NM_014700	RAB11FIP3	RAB11 family interacting protein	1.10214123	1.33466711	6.621492	7.308608	8.308169	4.82946313	4.96507495	5.23319995	1077	1586	1768
284252	NM_001136205,NM_001142730	KCTD1	potassium channel tetramerizatic	1.135268464	1.334533491	8.287466	8.977958	9.861589	4.51599508	4.69151638	4.91648254	859	1303	1410
94161	NR_000024	SNORD46	small nucleolar RNA, C/D box 46	1.415093022	1.334387609	7.464078	10.575132	9.182323	0.77668602	1.00745609	0.96541723	28	53	46
100528017	NM_001199744	SAA2-SAA4	SAA2-SAA4 readthrough	-1.812776157	1.333924378	1.607746	0.892943	2.039596	1.23264156	0.80196545	1.4863075	53	39	87
1353	NM_001162861,NM_001162862	COX11	COX11, cytochrome c oxidase co	1.024167537	1.333913186	8.487135	7.442393	9.967968	3.60061161	3.63226192	3.98628107	437	598	717
400986	NM_001310154	ANKRD36C	ankyrin repeat domain 36C	-1.356043837	1.333514506	1.754597	1.294725	2.20015	3.88804577	3.48283341	4.27877089	542	534	889
231	NM_001346142,NM_001628,NR	AKR1B1	aldo-keto reductase family 1 mem	1.609227119	1.333454588	60.998263	100.864996	76.964693	6.58238943	7.26306216	6.99379923	3723	8005	6106
23155	NM_001048210,NM_001278202	CLCC1	chloride channel CLIC like 1	1.237202468	1.333400841	9.945656	12.406551	12.687899	5.58018673	5.88149093	5.98775904	1839	3040	3016
55209	NM_001080517,NM_001292043	SETD5	SET domain containing 5	1.083087445	1.333301066	16.68987	18.043689	21.128693	6.85102049	6.96521381	7.26290456	4493	6502	7368
100130717	NR_024482,NR_024483	HDHD5-AS1	HDHD5 antisense RNA 1	1.652261867	1.333059371	0.898248	1.52821	1.168108	0.43999062	0.669904	0.56199967	14	31	23
400242	NR_015415	DICER1-AS1	DICER1 antisense RNA 1	-1.180225717	1.332883867	6.416216	5.439279	8.015959	2.58923814	2.39263351	2.94281592	197	223	323
7360	NM_001001521,NM_006759	UGP2	UDP-glucose pyrophosphorylase	1.298863904	1.332858338	38.268947	49.816105	47.874896	6.32626079	6.69937939	6.73630001	3111	5399	5100
9924	NM_001127460,NM_001166279	PAN2	PAN2 poly(A) specific ribonuclea	-1.072756505	1.33253545	13.672474	12.766115	17.127619	6.16508261	6.06521799	6.57424499	2778	3460	4553
7328	NM_001202498,NM_003344,NM	UBE2H	ubiquitin conjugating enzyme E2	-1.82531326	1.332501378	31.122379	17.07033	39.037486	7.29682241	6.43621034	7.70867468	6134	4490	10053
2110	NM_001281737,NM_001281738	ETFDH	electron transfer flavoprotein deh	1.118332131	1.331667882	8.034328	9.260727	9.708447	4.22711655	4.38031337	4.62109129	696	1040	1140
100132403	NR_146178_chr16,NR_146178_	FAM157B	family with sequence similarity 15	1.601346245	1.331537761	3.596135	5.768729	4.499855	3.80108857	4.44108339	4.18825403	508	1087	832
84895	NM_001329990,NM_032809,NR	MIGA2	mitoguardin 2	1.123986889	1.331348844	19.468618	22.025717	23.942068	6.30875248	6.47537422	6.71711753	3073	4615	5032
79363	NM_030907	RSG1	REM2 and RAB like small GTPas	1.448076254	1.331229471	0.784276	1.141811	0.979496	1.13562816	1.45142181	1.37552141	47	91	77
55635	NM_001114120,NM_017779	DEPDC1	DEP domain containing 1	1.745265452	1.331197919	10.18548	17.708094	12.581549	5.64036226	6.43143843	6.04589382	1919	4475	3142
84193	NM_032233,NM_199123	SETD3	SET domain containing 3	-1.017221359	1.331193897	24.303108	24.267241	30.353346	6.08289937	6.05863119	6.49033035	2622	3444	4293
1824	NM_004949,NM_024422	DSC2	desmocollin 2	1.171880269	1.331037687	4.065123	4.772641	5.090226	4.44015563	4.65922688	4.83613057	813	1273	1331
55582	NM_001271927,NM_001271928	KIF27	kinesin family member 27	1.067185455	1.330857061	1.534254	1.615492	1.880928	2.97768275	3.05994879	3.34390718	270	385	442
11033	NM_001284308,NM_001284309	ADAP1	ArfGAP with dual PH domains 1	-1.465250926	1.330600624	59.794694	39.035497	79.008635	7.01796225	6.47197015	7.42727937	5049	4604	8263
65117	NM_023012,NR_036434,NR_03	RSRC2	arginine and serine rich coiled-co	-1.066296532	1.330515454	41.002497	38.601456	51.744627	6.60181478	6.5101878	7.01011609	3774	4729	6176
688	NM_001286818,NM_001730	KLF5	Kruppel like factor 5	1.047226214	1.330465632	43.458618	45.677737	54.714361	7.24689109	7.31303698	7.65646795	5924	8289	9694
22841	NM_001330167,NM_014904	RAB11FIP2	RAB11 family interacting protein	1.284130299	1.330306664	1.078735	1.386654	1.422794	2.94941774	3.26839621	3.31417746	264	453	432
5824	NM_001193644,NM_002857,NR	PEX19	peroxisomal biogenesis factor 19	1.126667416	1.33029235	12.003794	13.50221	14.992092	5.44753406	5.6158843	5.8510692	1674	2520	2739
8482	NM_001146029,NM_001146030	SEMA7A	semaphorin 7A (John Milton Hag	1.056668198	1.330173391	17.850898	18.990154	22.415225	5.89499076	5.97321596	6.30059192	2297	3243	3758
348262	NM_001093767,NM_001288798	MCRIP1	MAPK regulated corepressor inte	1.426784599	1.330132581	12.913934	18.016526	15.822897	3.99792567	4.48350191	4.38697325	588	1121	962
6239	NM_001003698,NM_001003699	RREB1	ras responsive element binding p	1.012823399	1.330124237	4.556926	4.553077	5.670072	5.28267998	5.30059458	5.68503952	1489	2015	2436
80776	NM_030578	B9D2	B9 protein domain 2	1.020559094	1.329886571	1.655382	1.704137	2.085762	1.42282133	1.44132992	1.69448139	66	90	108
644246	NR_034172	KANSL1-AS1	KANSL1 antisense RNA 1	1.111131564	1.329886571	3.283433	3.672562	4.117305	1.42282133	1.52015496	1.69448139	66	98	108
54545	NM_001040446,NM_001294343	MTMR12	myotubularin related protein 12	-1.013893293	1.329738142	1.847024	1.816664	2.300452	3.36414578	3.34617992	3.74022131	365	481	597
3476	NM_001551	IGBP1	immunoglobulin binding protein 1	1.153844464	1.329661472	28.916431	33.39793	36.131001	5.57711104	5.77953587	5.98067956	1835	2829	3001
100508120	NR_046229	GMDS-AS1	GMDS antisense RNA 1 (head to	-1.398390948	1.329607102	0.720468	0.516071	0.896137	1.08456011	0.84852988	1.31676564	44	42	72
63826	NM_001304803,NM_021947	SRR	serine racemase	1.421829182	1.329001109	2.405388	3.42855	3.054939	2.77786255	3.22198991	3.13532137	230	437	376
79056	NM_024081	PRRG4	proline rich and Gla domain 4	-1.786773896	1.328466666	5.725857	3.209779	7.152154	4.99532414	4.19302682	5.39389141	1213	907	1982
90678	NM_001005373,NM_001005374	LRSAM1	leucine rich repeat and sterile alp	1.035173246	1.328295142	9.610796	9.659066	11.680845	4.96269999	5.01100366	5.36082844	1185	1639	1936
401152	NM_001001701,NM_001170330	C4orf3	chromosome 4 open reading fran	1.438908093	1.328266455	12.016766	17.317011	15.00589	5.0976022	5.60970201	5.49672576	1305	2509	2132
127943	NM_001002901,NM_001288829	FCRLB	Fc receptor like B	-1.115957615	1.328105812	1.848332	1.669185	2.315609	2.20692365	2.08433212	2.53718654	142	170	232
3713	NM_005547	IVL	involucrin	-1.082278843	1.328099792	7.454267	6.893645	9.307079	4.05651939	3.94954414	4.44436461	614	758	1003
90522	NM_001039671,NM_001039672	YIF1B	Yip1 interacting factor homolog E	-1.146879291	1.327816172	11.196309	9.765606	13.960635	4.973267	4.7822652	5.37097154	1194	1391	1950
57617	NM_020857	VPS18	VPS18, CORVET/HOPS core su	1.140852068	1.327747507	4.135549	4.722336	5.158349	4.06092955	4.2403589	4.44847569	616	939	1006
3858	NM_000421	KRT10	keratin 10	-1.02180918	1.327682013	10.290336	10.079443	12.839492	4.48842972	4.45870114	4.88143447	842	1101	1375
51068	NM_001320227,NM_015938	NMD3	NMD3 ribosome export adaptor	1.136791634	1.327665439	28.705748	32.773448	35.810292	6.28208505	6.46482647	6.68640722	3016	4581	4925
9805	NM_001145513,NM_001145514	SCRN1	secernin 1	-1.138409735	1.32764986	23.160345	20.366288	28.962679	6.90445095	6.71909239	7.31035907	4664	5474	7616
156	NM_001619	GRK2	G protein-coupled receptor kinas	-1.048457729	1.327639121	46.284489	44.195976	57.751163	7.28744396	7.21962148	7.69403269	6094	7766	9951
643224	NR_027156	TUBBP5	tubulin beta pseudogene 5	1.138981473	1.327585731	2.911202	3.329709	3.637533	3.09843961	3.26553914	3.46519272	297	452	485
51343	NM_001136197,NM_001136198	FZR1	fizzy and cell division cycle 20 rel	1.266514986	1.327390082	15.751384	20.019874	19.649017	5.81042968	6.14588877	6.21268572	2164	3662	3533
6047	NM_001185009,NM_001185010	RNF4	ring finger protein 4	1.153481907	1.327226301	26.039392	30.067939	32.468518	6.25009557	6.45357357	6.65384259	2949	4545	4814
102723471	NR_135668,NR_135669,NR_13	LOC102723471	uncharacterized LOC102723471	6.074189659	1.32711303	0.292687	1.249208	0.237722	0.35634914	1.44132992	0.45708734	11	90	18
105375800	NR_136279	LOC105375800	chromosome 12 open reading fra	1.6268495	1.32711303	0.197877	0.31776	0.247482	0.35634914	0.54351856	0.45708734	11	24	18
10627	NM_001303047,NM_001303048	MYL12A	myosin light chain 12A	-1.02857601	1.326912627	384.731777	375.163517	478.512996	8.83848976	8.79793116	9.24578869	17933	23295	29267
406	NM_001030272,NM_001030273	ARNTL	aryl hydrocarbon receptor nuclea	1.390143067	1.326828949	2.93146	4.27803	3.81219	3.26059229	3.69306567	3.63111814	337	626	550
389289	NM_001014279	ANXA2R	annexin A2 receptor	-1.308517643	1.326682604	1.009083	0.772929	1.256828	1.16869613	0.96581954	1.40965954	49	50	80
51594	NM_015909,NR_052013	NBAS	neuroblastoma amplified sequen	1.426704067	1.326596292	4.438975	6.340542	5.546342	5.02384223	5.52324054	5.42064411	1238	2360	2020
9367	NM_001195328,NM_004251	RAB9A	RAB9A, member RAS oncogene	-1.444139121	1.326323741	13.98549	9.659733	17.687524	4.25047234	3.7534508	4.63918874	708	655	1155
100500849	NR_037480	MIR3916	microRNA 3916	-1.208163935	1.325596135	10.40499	8.572353	13.065849	0.97665845	0.84852988	1.19154816	38	42	62
2010	NM_000117	EMD	emerin	1.189614452	1.325560156	36.775883	43.80677	45.824444	5.61359342	5.85939613	6.01296133	1883	2993	3070
2077	NM_001301035,NM_001308402	ERF	ETS2 repressor factor	1.569047538	1.325503	7.142875	11.149649	8.844453	4.28292556	4.90575769	4.67120285	725	1520	1182
2218	NM_001079802,NM_001198963	FKTN	fukutin	1.145552335	1.325192861	5.078278	5.916018	6.43917	5.26463133	5.45591151	5.66162136	1470	2250	2396
80164	NM_001190467,NM_104345	PRR36	proline rich 36	-4.661031992	1.325171596	0.685833	0.144407	0.857509	2.01115992	0.72085408	2.32699908	119	34	194
57597	NM_001291324	BAHCC1	BAH domain and coiled-coil conta	1.370889009	1.324864441	4.426054	6.073605	5.511189	5.50287791	5.94937984	5.90092246	1741	3189	2837
51430	NM_001282750,NM_001282751	SUCO	SUN domain containing ossificati	1.073923735	1.324838431	15.797066	17.342454	19.942398	6.46666486	6.56843617	6.86848757	3433	4926	5594
24144	NM_001008697,NM_001346857	TFIP11	tuftelin interacting protein 11	1.687337858	1.324781886	15.315255	26.126535	18.878821	5.76384974	6.50777196	6.16309929	2094	4721	3412
27165	NM_001280796,NM_001280797	GLS2	glutaminase 2	-1.581449141	1.324641168	0.174703	0.115261	0.221349	0.56944351	0.38406669	0.71544405	19	16	31
91523	NM_001281429,NM_138371	PCED1B	PC-esterase domain containing 1	1.039429808	1.324347088	8.578293	8.800388	10.740737	4.29794868	4.3509637	4.68520645	733	1018	1194

22883	NM_001009566,NM_001302883	CLSTN1	calsyntenin 1	1.777714674	1.324338184	44.03406	78.449041	54.917227	7.80718797	8.63439873	8.21088567	8754	20793	14259
25797	NM_012413	QPCT	glutaminyl-peptide cyclotransfera	1.244321984	1.324303741	6.360578	7.930768	7.924861	3.53238053	3.82311587	3.90684171	415	690	676
6598	NM_001007468,NM_001317946	SMARCB1	SWI/SNF related, matrix associat	1.193943434	1.324211299	40.41704	48.3103	50.438412	6.10388823	6.35622068	6.50389152	2661	4245	4334
22931	NM_001256410,NM_001256411	RAB18	RAB18, member RAS oncogene	-1.011824427	1.323899668	12.406043	12.228193	15.44931	5.93638283	5.91970164	6.33541791	2365	3123	3851
9267	NM_001292018,NM_001292019	CYTH1	cytohesin 1	-1.355688788	1.323852385	19.282555	14.232545	24.727321	6.04944579	5.61812585	6.44886481	2561	2524	4170
79572	NM_024524	ATP13A3	ATPase 13A3	-1.025706957	1.323653055	50.392265	49.184914	62.683636	8.49405617	8.45754014	8.89760527	14116	18388	22981
145773	NM_152450	FAM81A	family with sequence similarity 81	1.037059651	1.323593209	0.520842	0.543068	0.656677	1.47662927	1.51053416	1.74883089	70	97	114
151963	NM_178496	MB21D2	Mab-21 domain containing 2	1.040702629	1.323190673	3.440071	3.582754	4.277174	3.61867338	3.67164303	3.99380211	443	616	721
5208	NM_001018053,NM_006212	PFKFB2	6-phosphofructo-2-kinase/fructos	1.036209399	1.323183162	8.968456	9.039965	10.897146	5.76181945	5.81220815	6.15934204	2091	2895	3403
79932	NM_024874	KIAA0319L	KIAA0319 like	1.170397149	1.323124497	11.711458	13.725511	14.565633	5.80452441	6.02776984	6.20217361	2155	3370	3507
57140	NM_018226	RNPEPL1	arginyl aminopeptidase like 1	-1.105027291	1.322816967	22.789948	20.649677	28.339134	6.08722974	5.94536859	6.48567036	2630	3180	4279
55763	NM_001024924,NM_018261,NM	EXOC1	exocyst complex component 1	1.066004865	1.32265245	14.84949	15.921944	19.093449	5.70940872	5.79991918	6.10612025	2015	2870	3278
64921	NM_022900	CASD1	CAS1 domain containing 1	-1.493163364	1.322637511	7.499432	5.025773	9.320627	4.87400371	4.31962786	5.26540453	1112	995	1809
7913	NM_001134709,NM_003472	DEK	DEK proto-oncogene	1.070128311	1.322626191	41.917412	44.532467	51.704536	6.87219162	6.96917034	7.27259845	4560	6520	7418
100128927	NM_001137601	ZBTB42	zinc finger and BTB domain conta	-1.498774201	1.322549617	2.04071	1.365458	2.538692	3.00540462	2.50832973	3.36435937	276	246	449
55762	NM_001172655,NM_018260	ZNF701	zinc finger protein 701	-3.099437607	1.32249355	0.98386	0.335122	1.292615	2.66071453	1.44132992	3.00740444	209	90	340
467	NM_001030287,NM_001040619	ATF3	activating transcription factor 3	3.59252006	1.322436993	7.969062	28.261217	9.97313	4.09573376	5.87868909	4.47827086	632	3034	1028
9632	NM_004922,NM_198597	SEC24C	SEC24 homolog C, COPII coat c	-1.485308069	1.322346641	25.797129	17.475001	32.025909	6.83082584	6.26618436	7.23084242	4430	3985	7205
100130027	NR_147501	LOC100130027	uncharacterized LOC100130027	1.047774144	1.322321897	0.89133	0.935837	1.109403	2.26923253	2.32288731	2.59766727	150	210	244
57801	NM_001142467,NM_021170	HES4	hes family bHLH transcription fac	1.066976993	1.321902958	17.598228	18.771112	21.601555	4.19336823	4.28195221	4.57670807	679	968	1104
1153	NM_001280,NM_001300815,NM	CIRBP	cold inducible RNA binding prote	1.000378276	1.321863529	81.969634	82.598886	93.048408	7.02079481	7.02133625	7.42066811	5059	6762	8225
4125	NM_000528,NM_001173498	MAN2B1	mannosidase alpha class 2B mer	-1.178886917	1.321683105	16.725504	14.201735	20.778563	5.74204461	5.50942012	6.13786412	2062	2337	3352
286676	NM_001199799,NM_001199800	ILDR1	immunoglobulin like domain cont	1.484584613	1.321558419	1.707202	2.559829	2.101698	2.52684435	3.01302298	2.86703245	187	371	304
4287	NM_001127696,NM_001127697	ATXN3	ataxin 3	1.177068914	1.321180439	4.985148	5.881059	6.212409	5.11361143	5.34254521	5.50530231	1320	2076	2145
647024	NM_001164446	C6orf132	chromosome 6 open reading fran	-1.524440623	1.321087096	4.424936	2.904496	5.494613	4.90989472	4.32649813	5.29994267	1141	1000	1854
10092	NM_001270439,NM_005717	ARPC5	actin related protein 2/3 complex	2.699349106	1.320996646	20.316718	54.934831	25.239406	5.39697596	6.80792961	5.79028261	1615	5825	2624
81542	NM_030755	TMX1	thioredoxin related transmembra	1.238983817	1.320302889	21.021292	26.072023	26.079239	6.40902161	6.71490949	6.80577781	3297	5458	5354
135	NM_000675,NM_001278497,NM	ADORA2A	adenosine A2a receptor	-10.7316709	1.320219442	2.490853	0.210606	2.862876	2.84076697	0.65251278	3.19199808	242	30	393
90933	NM_033549,NM_201627,NR_04	TRIM41	tripartite motif containing 41	1.087247529	1.320200126	20.945861	22.675373	26.117266	6.15531992	6.27437957	6.55117294	2759	4008	4480
100526820	NR_037593	CAHM	colon adenocarcinoma hypermet	-1.294358382	1.320086307	0.936106	0.727016	1.167095	0.86002952	0.70406985	1.05442063	32	33	52
285313	NM_001178145,NM_001178146	IGSF10	immunoglobulin superfamily men	3.20221161	1.319714241	0.073225	0.234969	0.19623	0.68823103	1.5673195	0.85412573	24	103	39
1746	NM_004405	DLX2	distal-less homeobox 2	5.064991677	1.319714241	0.266667	1.383967	0.337742	0.68823103	2.03820547	0.85412573	24	163	39
3768	NM_021012	KCNJ12	potassium voltage-gated channel	1.308385209	1.319714241	0.119606	0.157376	0.148724	0.68823103	0.84852988	0.85412573	24	42	39
26805	NR_002749	SNORD45A	small nucleolar RNA, C/D box 45	1.153153536	1.319714241	7.351306	8.553183	9.235501	0.68823103	0.77006602	0.85412573	24	37	39
388695	NM_001136543,NM_212551	LYSMD1	LysM domain containing 1	1.051263802	1.31962294	2.364627	2.470798	2.885255	2.69516719	2.75641772	3.04046258	215	302	349
51538	NM_001282566,NM_001282567	ZCCHC17	zinc finger CCHC-type containing	1.299675715	1.31951355	13.061604	16.454755	16.004279	4.41969781	4.7822652	4.80333552	801	1391	1300
54439	NM_018989	RBM27	RNA binding motif protein 27	1.126541227	1.319484605	9.989913	11.26783	12.387864	6.01406127	6.18345852	6.40863599	2498	3760	4054
23395	NM_015340	LARS2	leucyl-tRNA synthetase 2, mitoch	1.36464704	1.319013065	3.556244	4.864145	4.409347	3.96064828	4.38426973	4.33758531	572	1043	928
161725	NM_001329907,NM_130901	OTUD7A	OTU deubiquitinase 7A	1.586500434	1.318972698	0.037782	0.06002	0.045429	0.49317359	0.72085408	0.62147005	16	34	26
30061	NM_014585	SLC40A1	solute carrier family 40 member 1	1.772365897	1.318972698	0.124454	0.221842	0.154902	0.49317359	0.7861039	0.62147005	16	38	26
401052	NM_001008737	LOC401052	uncharacterized LOC401052	1.028904044	1.318972698	0.215734	0.218377	0.269783	0.49317359	0.50527715	0.62147005	16	22	26
692090	NR_003046	SNORD59B	small nucleolar RNA, C/D box 59	-1.255378962	1.318972698	5.304782	4.308475	6.69442	0.49317359	0.40498891	0.62147005	16	17	26
10291	NM_005877	SF3A1	splicing factor 3a subunit 1	1.614862348	1.3189291	31.840281	51.476337	39.466187	7.32597221	8.01396512	7.72316955	6260	13507	10155
100506190	NR_038955	LINC00963	long intergenic non-protein codin	1.307210062	1.318873357	2.867397	3.758526	3.563787	2.79384765	3.13073752	3.14210594	233	407	378
56063	NM_019118,NR_133634,NR_13	TMEM234	transmembrane protein 234	2.110588579	1.31874389	5.082442	10.893372	6.539367	2.93987166	3.91531944	3.29299602	262	739	425
9400	NM_001003715,NM_001003716	RECQL5	RecQ like helicase 5	-1.092804771	1.318705397	26.55608	24.316769	32.547126	6.54588578	6.41927845	6.94128221	3629	4437	5886
5910	NM_001100426,NM_001100427	RAP1GDS1	Rap1 GTPase-GDP dissociation	1.409252862	1.318400404	9.202234	13.009985	11.631534	5.10935964	5.59213529	5.49804855	1316	2478	2134
57479	NM_020719	PRR12	proline rich 12	1.182214346	1.318335033	7.631536	9.030237	9.454984	5.71920749	5.95648354	6.11131559	2029	3205	3290
53981	NM_001322270,NM_001322271	CPSF2	cleavage and polyadenylation sp	1.202485522	1.31819789	20.122148	23.321803	24.90939	6.64688218	6.91048203	7.04197969	3895	6258	6315
27286	NM_014467	SRPX2	sushi repeat containing protein, X	-1.140684586	1.318044004	8.570512	7.522151	10.619653	4.26965076	4.0902069	4.64993924	718	841	1164
8045	NM_001143993,NM_001143994	RASSF7	Ras association domain family m	-1.018486928	1.317902531	16.762761	16.489442	20.634618	4.84232994	4.81682937	5.22842621	1087	1426	1762
63027	NM_001286455,NM_001286456	SLC22A23	solute carrier family 22 member 2	-1.958123932	1.317858015	0.557929	0.2856	0.695385	2.12505137	1.44132992	2.44142817	132	90	214
55630	NM_001280557,NM_017767,NM	SLC39A4	solute carrier family 39 member 4	-1.933305176	1.317825921	57.597456	16.090362	47.836598	5.69458464	4.7692132	6.08602965	1994	1378	3232
8650	NM_001005743,NM_001005744	NUMB	NUMB, endocytic adaptor protein	1.080372807	1.317701515	33.125188	35.687432	41.151302	6.86338174	6.97399142	7.25842341	4532	6542	7345
9308	NM_001040280,NM_001251901	CD83	CD83 molecule	3.054049648	1.317591572	3.353211	10.302976	4.163313	3.18986937	4.69045162	3.54926401	319	1302	517
4355	NM_001278370,NM_001278371	MPP2	membrane palmitoylated protein	1.735254526	1.317549736	1.226628	2.481865	1.766594	2.78853894	3.49264009	3.13532137	232	538	376
55957	NM_019104	LIN37	lin-37 DREAM MuvB core comple	1.063121831	1.317002839	8.438504	8.974471	10.444923	3.38540081	3.46550967	3.74913666	371	527	601
64216	NM_022366	TFB2M	transcription factor B2, mitochon	1.454516661	1.316964081	9.582238	13.965225	11.868787	4.14433812	4.65922688	4.52184068	655	1273	1061
3912	NM_002291	LAMB1	laminin subunit beta 1	-1.799189655	1.316943821	26.806019	14.915757	33.177715	7.26301006	6.42312952	7.65794805	5991	4449	9704
100506299	NR_110153,NR_110154	ID2-AS1	ID2 antisense RNA 1 (head to he	1.305671359	1.316768567	0.256159	0.336652	0.301809	0.26755841	0.3412894	0.34394329	8	14	13
102725509	NR_110731	LINC01232	long intergenic non-protein codin	1.49580772	1.316432952	0.709608	1.072599	0.888741	1.18495016	1.5392062	1.42086173	50	100	81
7621	NM_021916	ZNF70	zinc finger protein 70	1.276897568	1.316403395	0.595919	0.762101	0.740932	1.74129091	1.99745948	2.03057861	92	157	149
1944	NM_004952	EFNA3	ephrin A3	-1.235598204	1.316384609	2.890529	2.34023	3.576584	2.58923814	2.33928445	2.92718897	197	213	319
54461	NM_018998	FBXW5	F-box and WD repeat domain co	1.008522018	1.316284243	45.22979	45.660648	55.947037	6.72538138	6.73750906	7.11858197	4115	5545	6662
403341	NM_001099270	ZBTB34	zinc finger and BTB domain conta	1.047665303	1.31601367	4.314464	4.526249	5.335979	4.83719698	4.9020831	5.22123584	1083	1516	1753
5193	NM_000286	PEX12	peroxisomal biogenesis factor 12	1.252139417	1.315929014	2.505461	3.134503	3.096752	2.91572639	3.20120812	3.26529764	257	430	416
157922	NM_015447	CAMSAP1	calmodulin regulated spectrin ass	1.093422743	1.315623756	6.757565	7.399944	8.358129	5.67888953	5.80533922	6.0678839	1972	2881	3191
149603	NM_001010858	RNF187	ring finger protein 187	1.169442044	1.315422752	18.092857	21.17996	22.367727	5.81239274	6.03449873	6.20176777	2167	3386	3506
3068	NM_001126050,NM_001126051	HDGF	heparin binding growth factor	1.280584116	1.31515122	129.836314	168.365309	162.667416	8.23251432	8.58826784	8.62659641	11769	20137	19037
553115	NM_012392,NR_033686,NR_03	PEF1	penta-EF-hand domain containin	1.183840805	1.315092723	28.841133	34.327592	35.801219	5.82281754	6.06233997	6.21187981	2183	3453	3531
2631	NM_001202469,NM_001483	NIPSNAP2	nipsnap homolog 2	-1.273276214	1.314948472	22.745527	17.878374	28.18643	5.50449776	5.16458753	5.89189395	1743	1829	2819
3269	NM_000861,NM_001098211,NM	HRH1	histamine receptor H1	1.558020191	1.314094695	1.747541	2.730617	2.272802	3.13236099	3.71207778	3.48667429	305	635	493
57089	NM_001349962,NM_001349963	ENTPD7	ectonucleoside triphosphate diph	-1.058175635	1.313843958	0.583979	0.550796	0.729816	2.59533164	2.52753672	2.9311116	198	250	320
5253	NM_005392	PHF2	PHD finger protein 2	-1.048774295	1.313820244	13.816594	13.187895	17.05669	6.18895235	6.12120968	6.57800259	2825	3599	4565
282974	NM_001318878,NM_001318879	STK32C	serine/threonine kinase 32C	-1.297319742	1.313687659	6.707955	4.876682	10.264224	3.11973375	2.79259185	3.47328584	302	311	488
6314	NM_000333,NM_001128149,NM	ATXN7	ataxin 7	1.204644336	1.313609095	3.549288	4.28174	4.40078	4.6997269	4.9588981	5.07998613	981	1579	1585
4724	NM_001318051,NM_002495,NR	NDUFS4	NADH:ubiquinone oxidoreductas	1.093218128	1.313577283	81.953702	89.390141	101.40609	5.80057409	5.92695261	6.18790064	2149	3139	3472
64398	NM_001256550,NM_022474	MPP5	membrane palmitoylated protein	-1.060241622	1.313433101	9.757821	9.282464	12.195708	5.75639134	5.67360076	6.14336932	2083	2625	3365
153241	NM_001166226,NM_153223	CEP120	centrosomal protein 120	-1.0777002	1.313420202	5.024382	4.664938	6.199663	4.58804613	4.48473082	4.96703057	905	1122	1462
22838	NM_014901	RNF44	ring finger protein 44	1.117548449	1.313400589	16.179646	18.101614	19.971039	6.05387759	6.21193524	6.44200835	2569	3836	4150
120	NM_001121,NM_001320591,NM	ADD3	adducin 3	1.302324582	1.313198327	8.75429	11.430795	10.80356	5.28267998	5.65516321	5.66692369	1489	2591	2405
84976	NM_001350630,NM_032890	DISP1	dispatched RND transporter fami	1.778330397	1.313119889	0.688679	1.224869	0.845409	2.10810423	2.78463118	2.41925694	130	309	210
79644	NM_024592	SRD5A3	steroid 5 alpha-reductase 3	-1.022186597	1.313101995	7.737798	7.582877	9.554026	4.98955224	4.9588981	5.37169332	1208	1579	1951
6289	NM_001127380,NM_030754	SAA2	serum amyloid A2	-2.428150272	1.312952371	14.007849	5.369894	20.932554	3.80372238	2.66397469	4.17177132	509	280	822
100129722	NR_038389	STPG3-AS1	STPG3 antisense RNA 1	1.725961209	1.312850322	0.992577	1.723679	1.222982	1.40904982	1.94842076	1.66651905	65	150	105
64866	NM_022842,NM_178181	CDCP1	CUB domain containing protein 1	1.530169495	1.312752051	27.374326	41.76772	39.186328	7.29682241	7.90734007	7.68723555	6134	12541	9904
25994	NM_001099668,NM_001099669	HIGD1A	HIG1 hypoxia inducible domain fa	-1.131270145	1.312382974	6.651847	5.871032	8.293626	4.26965076	4.10146599	4.64397663	718	848	1159
5832	NM_001017423,NM_001323412	ALDH18A1	aldehyde dehydrogenase 18 fam	-1.091641684	1.312227464	18.921823	17.353724	23.28198	6.01803602	5.89356976	6.40476231	2505	3066	4043
91754	NM_001329237,NM_001329238	NEK9	NIMA related kinase 9	1.113885133	1.312142998	5.32163	5.947373	6.625953	5.07270439	5.22392723	5.45442456	1282	1908	2069
162	NM_001127,NM_001166019,NM	AP1B1	adaptor related protein complex 1	1.21954715	1.312109413	12.501805	15.266113	15.411511	5.68533091	5.96663422	6.07055367	1981	3228	3197
9469	NM_004273	CHST3	carbohydrate sulfotransferase 3	-2.070797446	1.311812079	2.60796	1.261755	3.217402	4.22711655	3.25693382	4.60030259	696	449	1123
7296	NM_001093771,NM_001261445	TXNRD1	thioredoxin reductase 1	-1.27283758	1.31147924	52.593151	42.658311	65.264315	7.72498887	7.37879475	8.11456803	8267	8678	13335
29028	NM_014109	ATAD2	ATPase family, AAA domain cont	1.01860769	1.311295868	68.185043	69.529671	84.02285	8.53287021	8.55939776	8.9229411	14502	19737	23389
9064	NM_001297609,NM_004672	MAP3K6	mitogen-activated protein kinase	1.065224712	1.311168985	5.689106	6.070556	7.006412	4.64790143	4.73554094	5.02507241	945	1345	1524
80829	NM_001197051,NM_053023	ZFP91	ZFP91 zinc finger protein	-1.044253709	1.31116007	17.115133	16.413467	21.094936	6.59118228	6.52937024	6.97848082	3746	4793	6041
374900	NM_001204835,NM_001204836	ZNF568	zinc finger protein 568	-3.436620808	1.310481983	0.649709	0.198265	0.797713	1.80577475	0.7861039	2.09493548	98	38	158
2672	NM_001127215,NM_001127216	GFI1	growth factor independent 1 trans	-1.457234826	1.310403016	5.477697	3.836527	6.846608	3.97240126	3.47048058	4.34053776	577	529	930
84085	NM_001348092,NM_032145	FBXO30	F-box protein 30	1.349548287	1.309939751	2.530274	3.424526	3.116073	3.56999691	3.97074877	3.93054845	427	770	688
9559	NM_001035260,NM_001318944	VPS26A	VPS26, retromer complex compo	-1.382414147	1.30990061	14.492143	10.618217	18.021435	5.9321763	5.47396912	6.31604903	2358	2279	3799

6483	NM_006927	ST3GAL2	ST3 beta-galactoside alpha-2,3-s	-1.162980817	1.309706751	2.24622	1.937011	2.767048	3.42699388	3.23080555	3.78425716	383	440	617
441531	NM_001029891	PGAM4	phosphoglycerate mutase family	1.233376685	1.309701018	2.512595	3.111846	3.099755	2.3579234	2.60642858	2.67922768	162	267	261
5986	NM_002917	RFNG	RFNG O-fucosylpeptide 3-beta-N	1.079108252	1.309565689	29.144571	31.491484	35.869972	5.72477705	5.83262085	6.10742084	2037	2937	3281
60526	NM_001282719,NM_001282720	LDAH	lipid droplet associated hydrolase	-3.585206961	1.309517561	4.470193	1.231131	5.455796	4.14433812	2.49862948	4.51401614	655	244	1055
6536	NM_001024845,NM_001261380	SLC6A9	solute carrier family 6 member 9	1.8558121	1.309374233	3.193803	5.963494	3.988481	3.47735365	4.30856691	3.83538509	398	987	641
23464	NM_001171690,NM_014291	GCAT	glycine C-acetyltransferase	-1.227292632	1.309027162	6.582641	5.444674	8.287212	3.46742135	3.20120812	3.82488183	395	430	636
6526	NM_006933	SLC5A3	solute carrier family 5 member 3	1.085293109	1.30892602	3.673914	3.992148	4.520983	5.41258913	5.52801678	5.79297945	1633	2368	2629
55013	NM_017918	MCUB	mitochondrial calcium uniporter d	-1.65779262	1.308859189	11.196218	6.755624	13.78809	3.89793981	3.23080555	4.26329596	546	440	879
81839	NM_001172411,NM_001172412	VANGL1	VANGL planar cell polarity protei	1.011013952	1.308698282	5.559015	5.625263	6.834055	5.58631849	5.60179513	5.96737086	1847	2495	2973
11064	NM_001330762,NM_007018	CNTRL	centriolin	1.063726239	1.308493634	9.417799	10.059086	11.266515	5.80320883	5.8907913	6.18502902	2153	3060	3465
7532	NM_012479	YWHAG	tyrosine 3-monooxygenase/trypto	-1.187724406	1.308457268	58.56287	49.358612	72.006699	7.74585348	7.49891108	8.1321395	8388	9436	13499
284119	NM_012232	CAVIN1	caveolae associated protein 1	-1.081729224	1.308330846	34.221981	31.670561	42.073303	6.93179706	6.81942046	7.31674494	4754	5872	7650
125061	NM_001010982,NM_001145526	AFMID	arylformamidase	1.112731772	1.308306275	25.485465	27.523693	28.803184	5.19310692	5.34322286	5.57151066	1397	2077	2248
644096	NM_001042631	SDHAF1	succinate dehydrogenase comple	1.024386229	1.30805247	8.27269	8.485781	10.176025	3.33894587	3.37031674	3.6924898	358	490	576
22846	NM_014909	VASH1	vasohibin 1	2.441100236	1.307957653	0.576121	1.40922	0.707928	2.13345087	3.21312006	2.44142817	133	434	214
6391	NM_001035511,NM_001035512	SDHC	succinate dehydrogenase comple	1.153220769	1.307886194	20.312317	22.934125	24.530737	5.80386677	6.00610962	6.18502902	2154	3319	3465
9833	NM_001256685,NM_001256687	MELK	maternal embryonic leucine zippe	1.049784142	1.307883082	27.702957	29.176202	35.080841	6.0560884	6.12515529	6.43822336	2573	3609	4139
10023	NM_005479	FRAT1	FRAT1, WNT signaling pathway	-1.279617652	1.307202741	0.452972	0.351181	0.551831	1.11880553	0.93737891	1.34055586	46	48	74
91137	NM_001303249,NM_001303250	SLC25A46	solute carrier family 25 member 4	1.937297291	1.307020207	5.173269	10.033333	6.362039	4.65229204	5.57838681	5.02507241	948	2454	1524
90874	NM_001080470	ZNF697	zinc finger protein 697	2.011158374	1.306911333	1.218188	2.455809	1.496471	2.81488929	3.71626888	3.15222336	237	637	381
56181	NM_001099625,NM_001099626	MTFR1L	mitochondrial fission regulator 1 l	1.006482775	1.306824889	10.81123	10.952117	13.172671	4.44353723	4.45243375	4.81399624	815	1096	1310
55974	NM_001122837,NM_001122839	SLC50A1	solute carrier family 50 member 1	1.011097262	1.306821086	43.814403	44.962568	53.240864	5.80583878	5.82147825	6.18585007	2157	2914	3467
112950	NM_001001653,NM_052877,NM	MED8	mediator complex subunit 8	-1.034582268	1.306810176	18.737605	17.994263	23.039338	5.18908339	5.14139825	5.56584509	1393	1799	2239
10016	NM_001267556,NM_001267557	PDCD6	programmed cell death 6	-1.112807677	1.306448689	62.263809	55.761711	75.889741	6.11453445	5.96267072	6.49530656	2681	3219	4308
23507	NM_001134476,NM_015350	LRRC8B	leucine rich repeat containing 8 fa	-1.078011166	1.306383805	3.248154	3.019283	3.989756	4.68265777	4.57864948	5.05503907	969	1201	1557
400720	NM_001024596,NM_001144068	ZNF772	zinc finger protein 772	1.027508374	1.306354846	4.40976	4.502788	5.402994	4.55716073	4.59467379	4.92830565	885	1215	1422
192669	NM_024852,NM_177422	AGO3	argonaute 3, RISC catalytic comp	1.363044392	1.306071671	3.778151	5.148965	4.630347	3.84777676	4.26774474	4.20940382	526	958	845
51520	NM_001317964,NM_001317965	LARS	leucyl-tRNA synthetase	1.068034107	1.305957686	40.114364	42.22951	48.432383	7.56459082	7.65906432	7.94791756	7393	10550	11875
128866	NM_176812	CHMP4B	charged multivesicular body prot	-1.051221185	1.305840924	105.606331	100.575096	129.59549	7.41242078	7.34078705	7.79542081	6649	8451	10679
55717	NM_018117	WDR11	WD repeat domain 11	1.081124283	1.305767651	7.866346	8.517161	9.653193	5.21997612	5.32960894	5.59580755	1424	2057	2287
84996	NR_026845	URB1-AS1	URB1 antisense RNA 1 (head to	1.039470956	1.30563597	6.268973	6.517407	7.683458	2.59533164	2.64211237	2.92325564	198	275	318
51526	NM_016470	OSER1	oxidative stress responsive serin	1.028472429	1.305614668	22.758358	23.434389	27.928864	5.66374685	5.70346404	6.04181814	1951	2681	3133
2987	NM_000858,NM_001159390,NM	GUK1	guanylate kinase 1	1.081345548	1.305519152	77.93251	83.985928	94.025557	6.27830249	6.38973499	6.65858217	3008	4346	4830
26280	NM_017416	IL1RAPL2	interleukin 1 receptor accessory p	2.347074086	1.305465878	0.244965	0.57637	0.296403	0.77668602	1.42093184	0.95003448	28	88	45
109729181	NR_146321	ZNF710-AS1	ZNF710 antisense RNA 1	-1.961784485	1.305465878	0.608573	0.312948	0.76239	0.77668602	0.44594451	0.95003448	28	19	45
51643	NM_001282606,NM_001282609	TMBIM4	transmembrane BAX inhibitor mo	-1.227821235	1.305285154	15.530768	12.782206	19.216828	4.85888706	4.57403823	5.23161046	1100	1197	1766
201163	NM_144606,NM_144997	FLCN	folliculin	1.170246194	1.305254046	4.385324	5.202301	6.133645	3.90776647	4.12056519	4.26950586	550	860	883
7088	NM_001303103,NM_001303104	TLE1	transducin like enhancer of split 1	-1.125092347	1.305149736	7.407813	6.605893	9.117978	4.94848917	4.78426276	5.32177032	1173	1393	1883
79792	NM_001166237,NM_024736	GSDMD	gasdermin D	1.014820437	1.304924034	35.687972	36.258183	43.809017	5.96018767	5.98107461	6.33874398	2405	3261	3860
23210	NM_001081461,NM_015167	JMJD6	arginine demethylase and lysine	1.073757187	1.304845609	16.13924	17.719482	20.476712	5.05180751	5.15149273	5.42551859	1263	1812	2027
84790	NM_001303114,NM_001303115	TUBA1C	tubulin alpha 1c	1.065186472	1.30474189	531.404114	566.582206	651.515494	9.73400195	9.82500456	10.11737177	33394	47527	53589
1267	NM_001330216,NM_033133	CNP	2',3'-cyclic nucleotide 3' phospho	1.21565132	1.304670835	14.568528	17.73396	17.863764	6.23065365	6.50898038	6.60985905	2909	4725	4668
286205	NM_001144877,NM_173690	SCAI	suppressor of cancer cell invasio	1.372425066	1.304268827	1.520405	2.096118	1.870408	4.25432848	4.69045162	4.61987668	710	1302	1139
100506548	NR_037665	LOC100506548	uncharacterized LOC100506548	1.402907831	1.304268742	3.811074	5.358386	4.673092	3.94641713	4.40778273	4.30772367	566	1061	908
7916	NM_004638,NM_080686	PRRC2A	proline rich coiled-coil 2A	1.160306594	1.304252083	29.999267	34.851957	36.796589	7.66885172	7.88238064	8.0504244	7950	12325	12753
79628	NM_024577	SH3TC2	SH3 domain and tetratricopeptide	-1.008058057	1.304065157	2.452362	2.435771	3.005173	6.01064561	5.99924669	6.38844962	2492	3303	3997
388564	NM_001190764	TMEM238	transmembrane protein 238	-1.443422875	1.303936254	10.038493	6.963849	12.331308	2.90595399	2.45916281	3.24338068	255	236	409
26297	NM_012139,NR_104040,NR_10	SERGEF	secretion regulating guanine nucl	-1.066451297	1.303767153	7.595271	7.046988	9.323043	3.60665736	3.5216664	3.96156336	439	550	704
83744	NM_001007101,NM_001261458	ZNF484	zinc finger protein 484	1.145696806	1.303671834	3.023142	3.445554	3.698679	3.65121051	3.83279899	4.0068703	454	695	728
55297	NM_001330367,NM_001352078	CCDC91	coiled-coil domain containing 91	-1.018352973	1.303436622	8.288524	8.072378	10.046842	4.44184742	4.41682418	4.80867573	814	1068	1305
9479	NM_005456	MAPK8IP1	mitogen-activated protein kinase	1.560355205	1.303099194	0.468746	0.738209	0.577903	1.30878272	1.72538257	1.5489128	58	121	93
9611	NM_001190438,NM_001190440	NCOR1	nuclear receptor corepressor 1	-1.027315748	1.302945914	6.090829	5.833129	7.335801	5.8267075	5.78852012	6.20257934	2189	2847	3508
5214	NM_001242339,NM_001323067	PFKP	phosphofructokinase, platelet	1.600467354	1.302878061	115.4168	183.705201	140.48874	8.18481865	8.86145565	8.56537099	11385	24346	18244
94025	NM_024690	MUC16	mucin 16, cell surface associated	-1.299426355	1.302765945	6.068361	4.675014	7.429351	8.02174811	7.64552993	8.40203901	10164	10451	16286
1725	NM_001206974,NM_001930,NM	DHPS	deoxyhypusine synthase	-1.201189732	1.302634783	39.635111	33.309085	48.33326	5.71361634	5.45465779	6.08866608	2021	2248	3238
55502	NM_001142853,NM_001282434	HES6	hes family bHLH transcription fac	1.117990901	1.302432099	1.489773	1.660902	1.827818	1.63881618	1.75012126	1.90855849	83	124	133
9361	NM_001276479,NM_001276480	LONP1	lon peptidase 1, mitochondrial	1.350498765	1.302078065	37.49202	55.358555	45.947558	6.88219462	7.31251802	7.26017856	4592	8286	7354
122525	NM_001017923	C14orf28	chromosome 14 open reading fra	-1.262028994	1.302019573	2.304329	1.824082	2.815348	2.91084846	2.6243808	3.24653213	256	271	410
10184	NM_005779	LHFPL2	lipoma HMGIC fusion partner-like	-1.245100908	1.301847434	12.909794	10.3809	15.795277	6.00950526	5.69870608	6.38487885	2490	2672	3987
29979	NM_013438,NM_053067	UBQLN1	ubiquilin 1	1.21779662	1.301701043	34.685703	42.275045	42.405492	7.1441248	7.42657771	7.52216379	5514	8972	8828
60312	NM_001134647,NM_198595	AFAP1	actin filament associated protein	-1.127506393	1.301685107	18.862131	16.743797	23.017376	7.11975951	6.94794234	7.49774071	5421	6424	8679
2992	NM_001184720,NM_001184721	GYG1	glycogenin 1	1.384600996	1.301332548	18.397489	25.292211	22.310829	5.16265334	5.62092291	5.53331305	1367	2529	2188
10713	NM_001256725,NM_001256726	USP39	ubiquitin specific peptidase 39	1.33237373	1.300686185	31.134927	41.446682	37.955898	6.10922116	6.51801145	6.4836686	2671	4755	4273
100131827	NM_001128223,NM_001290208	ZNF717	zinc finger protein 717	1.730266228	1.300670767	1.361109	4.21357	2.574223	2.34351507	3.009612	2.65571422	160	370	256
94121	NM_001129896,NM_001174068	SYTL4	synaptotagmin like 4	1.104217207	1.300627153	5.270158	5.7987	6.349722	4.36902637	4.50546383	4.73205178	772	1139	1235
100289341	NR_027447	MAN1B1-AS1	MAN1B1 antisense RNA 1 (head	1.604555789	1.300269382	0.757558	1.227797	0.93095	1.26358189	1.70021226	1.4969322	55	118	88
7454	NM_000377	WAS	Wiskott-Aldrich syndrome	1.279988657	1.300133706	0.638228	0.81451	0.781665	1.10178443	1.30327576	1.31676564	45	77	72
5912	NM_002886	RAP2B	RAP2B, member of RAS oncoge	-1.038189651	1.300117994	7.422059	7.157173	9.066846	5.94952407	5.89634288	6.32278311	2387	3072	3817
54602	NM_001161407,NM_019080	NDFIP2	Nedd4 family interacting protein 2	-1.088775326	1.30005794	10.212805	9.391726	12.482041	5.56396574	5.44395692	5.93550587	1818	2231	2907
100130992	NR_038921	LOC100130992	uncharacterized LOC100130992	1.943405604	1.3000405	0.157725	0.31462	0.19714	1.01352966	1.57657016	1.21747621	40	104	64
56034	NM_016205,NR_036641	PDGFC	platelet derived growth factor C	2.027614388	1.299807098	2.897799	5.863506	3.526543	3.24904328	4.19001636	3.59201087	334	905	534
4015	NM_001178102,NM_001317073	LOX	lysyl oxidase	5.149396115	1.299761229	0.145828	0.792168	0.180339	0.81895953	2.30630165	0.99569924	30	207	48
90407	NM_080652	TMEM41A	transmembrane protein 41A	1.104015636	1.299672325	26.387403	29.170086	32.228466	6.20248825	6.343407	6.57612501	2852	4207	4559
4752	NM_001146099,NM_002498,NM	NEK3	NIMA related kinase 3	-1.362676115	1.299590672	1.676851	1.218933	2.030159	2.28439817	1.94127715	2.59272293	152	149	243
23085	NM_001301248,NM_178039,NM	ERC1	ELKS/RAB6-interacting/CAST fa	1.31505542	1.299371504	1.268201	1.65568	1.551628	3.64828273	4.01565009	3.99941726	453	796	724
100507127	NR_038291	LINC00707	long intergenic non-protein codin	-2.296190882	1.299204913	0.991513	0.430272	1.217075	1.9928118	1.19932051	2.28470378	117	68	187
100289388	NR_102280,NR_102281	KCTD21-AS1	KCTD21 antisense RNA 1	1.978117628	1.299204243	0.48053	0.949571	0.584962	0.59399636	1.00745609	0.73352601	20	53	32
10046	NM_001177465,NM_001177466	MAMLD1	mastermind like domain containin	1.121998887	1.299204243	0.111906	0.126042	0.138709	0.59399636	0.65251278	0.73352601	20	30	32
57003	NM_020198	CCDC47	coiled-coil domain containing 47	1.160013853	1.299002516	55.095947	63.987274	67.260513	7.52683135	7.73989681	7.90243892	7201	11161	11505
10672	NM_001282425,NM_006572	GNA13	G protein subunit alpha 13	-1.284034773	1.298897089	11.958645	9.321033	14.596297	6.21886148	5.86365332	6.59169733	2885	3002	4609
9857	NM_014810	CEP350	centrosomal protein 350	1.054695904	1.29887587	4.601975	4.860511	5.618722	5.93818188	6.01379159	6.31003667	2368	3337	3783
55145	NM_018105,NM_199003	THAP1	THAP domain containing 1	1.110610535	1.298791201	4.895595	5.562678	6.040537	3.50026652	3.63890054	3.84788889	405	601	647
57190	NM_020451,NM_206926	SELENON	selenoprotein N	1.165440603	1.298783283	13.432081	15.640469	16.344578	5.82476383	6.0420314	6.19607391	2186	3404	3492
100996255	NR_102756	LINC02256	long intergenic non-protein codin	-1.990027621	1.298649323	0.316851	0.156333	0.380879	0.46682716	0.25173066	0.58209675	15	10	24
5623	NM_004158	PSPN	persephin	-1.00207692	1.298649323	0.560543	0.566144	0.677006	0.46682716	0.46599441	0.58209675	15	20	24
301	NM_000700	ANXA1	annexin A1	-1.263585711	1.298646516	800.78656	634.443237	977.254211	10.22710457	9.88989742	10.60383732	47017	49716	75098
293	NM_001636_chrX,NM_001636_c	SLC25A6	solute carrier family 25 member 6	1.085483131	1.298642442	51.460133	55.927589	62.812683	6.23602635	6.35285968	6.60863495	2920	4235	4664
64802	NM_001297778,NM_001297779	NMNAT1	nicotinamide nucleotide adenylylt	1.078127568	1.298593536	3.065033	3.403687	4.274816	3.38187998	3.48037129	3.72674411	370	533	591
29800	NM_001323627,NM_013304	ZDHHC1	zinc finger DHHC-type containing	-1.483435229	1.298410897	2.745253	1.851233	3.354426	2.67229041	2.20853486	2.99621456	211	190	337
57062	NM_020414	DDX24	DEAD-box helicase 24	1.319787016	1.298406016	46.167473	61.000164	56.33493	7.06097877	7.45867069	7.43524216	5203	9175	8309
339344	NM_001012643	MYPOP	Myb related transcription factor, p	1.091895653	1.298329629	3.71071	4.057792	4.53301	2.96832247	3.07960182	3.30211189	268	391	428
3675	NM_002204	ITGA3	integrin subunit alpha 3	1.393844037	1.298328288	241.309937	336.722595	294.414825	10.20939813	10.68812386	10.58577413	46443	86493	74163
203197	NM_001330760,NM_153045	TMEM268	transmembrane protein 268	1.029640402	1.29827025	2.999857	3.137991	3.739976	3.80897562	3.84815806	4.16179054	511	703	816
149420	NM_001243532,NM_001243533	PDIK1L	PDLIM1 interacting kinase 1 like	-1.381549336	1.298170343	6.108329	4.424022	7.441332	4.68694402	4.24181329	5.05053848	972	940	1552
5707	NM_001191037,NM_002807,NR	PSMD1	proteasome 26S subunit, non-AT	1.305348992	1.298046398	29.444987	38.484283	35.931153	6.58889365	6.96982871	6.96180011	3740	6523	5971
1435	NM_000757,NM_172210,NM_17	CSF1	colony stimulating factor 1	1.153573351	1.298001667	1.208483	1.488773	1.566302	2.48807034	2.65962865	2.80425801	181	279	289
27090	NM_175039,NM_175040	ST6GALNAC4	ST6 N-acetylgalactosaminide alp	1.625340746	1.297928264	9.593741	15.677907	11.683047	4.06312959	4.73036982	4.41944907	617	1340	985
80851	NM_001322462,NM_001322463	SH3BP5L	SH3 binding domain protein 5 lik	-1.022047476	1.297830244	2.917762	2.927844	3.694529	3.43040645	3.40188358	3.77555686	384	502	613
192670	NM_017629,NR_146062	AGO4	argonaute 4, RISC catalytic comp	-1.416419684	1.297722232	4.276672	2.803832	5.403333	4.7808325	4.30021519	5.14475817	1040	981	1660

55668	NM_001322026,NM_001322027	GPATCH2L	G-patch domain containing 2 like	1.156087284	1.297658639	21.292425	23.459467	23.055863	6.40599143	6.61294922	6.77800579	3290	5082	5251
102724571	NR_132738	LINC01759	long intergenic non-protein coding	2.454270809	1.297545635	0.260871	0.653051	0.319855	0.3273552	0.70406985	0.41288872	10	33	16
149647	NM_153606	FAM71A	family with sequence similarity 71	-1.890555353	1.297545635	0.109407	0.050433	0.137141	0.3273552	0.18071335	0.41288872	10	7	16
105377590	NR_133944	LOC105377590	uncharacterized LOC105377590	-6.301587867	1.297545635	0.546765	0.082015	0.652088	0.3273552	0.05398282	0.41288872	10	2	16
284613	NM_001134400,NM_001134402	CYB561D1	cytochrome b561 family member	1.067473794	1.297251889	2.601004	2.777232	3.152233	3.78518394	3.87277297	4.13653226	502	716	801
4301	NM_001040000,NM_001207008	AFDN	afadin, adherens junction formati	-1.126983551	1.297241732	17.979476	15.951531	21.952923	7.08228661	6.91116773	7.45529946	5281	6261	8426
55858	NM_018475,NR_073070	TMEM165	transmembrane protein 165	1.293311729	1.297238493	36.155066	46.820506	44.052441	6.14133197	6.50777196	6.5120984	2732	4721	4359
4500	NR_001447	MT1L	metallothionein 1L, pseudogene	-1.12034122	1.297113755	3.169986	2.850085	3.870734	1.21691895	1.12560759	1.4430085	52	62	83
64083	NM_022130	GOLPH3	golgi phosphoprotein 3	-1.244650794	1.297100179	19.417593	15.618214	23.671646	5.68960924	5.38067227	6.05850059	1987	2133	3170
63977	NM_001040424,NM_001282934	PRDM15	PR/SET domain 15	1.047800108	1.296956929	3.133134	2.849219	3.364487	4.09142887	4.15493692	4.44710663	630	882	1005
51755	NM_015083,NM_016507	CDK12	cyclin dependent kinase 12	1.204673202	1.296909193	8.953352	10.802377	10.916335	6.19297625	6.45827295	6.56354499	2833	4560	4519
23396	NM_001195733,NM_001300849	PIP5K1C	phosphatidylinositol-4-phosphate	1.316749959	1.296796987	7.971902	10.57968	9.815686	5.31257643	5.70082267	5.67922052	1521	2676	2426
150084	NM_001080444	IGSF5	immunoglobulin superfamily men	-3.124697541	1.296713891	0.590887	0.189097	0.719197	1.13562816	0.46599441	1.35230545	47	20	75
104355426	NR_126418	LENG8-AS1	LENG8 antisense RNA 1	1.064200585	1.296624416	6.942594	7.403152	8.452947	2.81488929	2.89227137	3.14210594	237	337	378
55054	NM_001190266,NM_001190267	ATG16L1	autophagy related 16 like 1	1.911120288	1.296483931	33.045473	63.301424	40.390442	6.77487823	7.70301942	7.14647525	4260	10878	6793
92521	NM_001033553,NM_001033554	SPECC1	sperm antigen with calponin hom	1.242977996	1.296474927	6.082533	7.579002	7.656085	4.67979317	4.98257876	5.04149497	967	1606	1542
8892	NM_014239	EIF2B2	eukaryotic translation initiation fa	1.320741435	1.296074386	23.086679	30.524906	28.110725	5.15545404	5.54696516	5.52035242	1360	2400	2168
8797	NM_003844	TNFRSF10A	TNF receptor superfamily membe	1.168877264	1.295983878	5.551371	6.504855	6.767605	3.38891307	3.59405489	3.73125051	372	581	593
284352	NM_019121	PPP1R37	protein phosphatase 1 regulatory	1.065977093	1.295977234	13.570691	14.487615	16.530682	5.32271189	5.41266157	5.68851969	1532	2182	2442
2790	NM_001017998,NM_001198664	GNG10	G protein subunit gamma 10	1.322667053	1.295877632	31.975292	42.348287	38.972561	5.32454708	5.71921153	5.69025663	1534	2711	2445
92840	NM_001329556,NM_138393	REEP6	receptor accessory protein 6	1.48444898	1.295841306	7.245089	10.783669	8.833946	3.5668994	4.09665142	3.91280502	426	845	679
10569	NM_006425	SLU7	SLU7 homolog, splicing factor	1.147470431	1.295807106	17.939278	20.6134	21.848057	5.98186283	6.17739112	6.35050828	2442	3744	3892
9410	NM_004814	SNRNP40	small nuclear ribonucleoprotein U	1.399645371	1.295607792	21.942163	30.758448	26.7297	5.15854386	5.6320572	5.52295387	1363	2549	2172
11274	NM_017414	USP18	ubiquitin specific peptidase 18	1.097496984	1.295166243	9.939101	10.927744	12.10502	4.37257867	4.50061221	4.72980159	774	1135	1233
100132062	NR_028325_chr1,NR_028325_c	LOC100132062	uncharacterized LOC100132062	1.725739658	1.295080773	1.542101	2.664961	1.876482	2.92058788	3.62559261	3.24967672	258	595	411
79567	NM_001193522,NM_001193523	RIPOR1	RHO family interacting cell polari	1.492356998	1.294805979	14.815389	22.065861	17.993976	5.91766008	6.48737891	6.28496741	2334	4654	3717
7881	NM_001308217,NM_001308222	KCNAB1	potassium voltage-gated channel	9.400172152	1.29428298	0.023219	0.27511	0.035393	0.17294258	1.15059938	0.22116494	5	64	8
146	NM_000678	ADRA1D	adrenoceptor alpha 1D	4.857113602	1.29428298	0.039252	0.237025	0.056788	0.17294258	0.70406985	0.22116494	5	33	8
9935	NM_005461	MAFB	MAF bZIP transcription factor B	5.882965532	1.29428298	0.030842	0.228166	0.047128	0.17294258	0.81765451	0.22116494	5	40	8
54661	NR_001280	PCDHB17P	protocadherin beta 17 pseudoger	1.486457258	1.29428298	0.046574	0.074558	0.064942	0.17294258	0.25173066	0.22116494	5	10	8
164153	NM_203412	UBL4B	ubiquitin like 4B	-1.110794961	1.29428298	0.086226	0.077862	0.094752	0.17294258	0.15624221	0.22116494	5	6	8
189	NM_000030	AGXT	alanine-glyoxylate aminotransfera	6.469166635	1.29428298	0.071158	0.538626	0.100066	0.17294258	0.8787583	0.22116494	5	44	8
692234	NR_004054,NR_004054_dup1	SNORD103A	small nucleolar RNA, C/D box 10	1.04680643	1.29428298	1.212837	1.518775	1.735771	0.17294258	0.18071335	0.22116494	5	7	8
6887	NM_005421	TAL2	TAL bHLH transcription factor 2	1.04680643	1.29428298	0.161489	0.194674	0.241271	0.17294258	0.18071335	0.22116494	5	7	8
79856	NM_024798,NR_073534	SNX22	sorting nexin 22	2.072658361	1.29428298	0.034689	0.072043	0.043624	0.17294258	0.3412894	0.22116494	5	14	8
7732	NM_007148	RNF112	ring finger protein 112	1.926108085	1.29428298	0.032418	0.072869	0.047453	0.17294258	0.31941552	0.22116494	5	13	8
3177	NM_001300868,NM_001300869	SLC29A2	solute carrier family 29 member 2	1.247581169	1.29418779	11.962401	15.302928	14.857118	4.94610703	5.25594871	5.30750681	1171	1952	1864
5096	NM_000532,NM_001178014	PCCB	propionyl-CoA carboxylase beta s	1.165444653	1.29395522	26.435249	30.850613	32.114752	5.57788058	5.79447869	5.94280996	1836	2859	2922
148266	NM_001330482,NM_152484	ZNF569	zinc finger protein 569	-1.981812221	1.293908067	0.6278	0.312091	0.750774	1.80577475	1.1751656	2.08088	98	66	156
27013	NM_001321389,NM_001321390	CNPPD1	cyclin Pas1/PHO80 domain conta	1.123983442	1.293863842	17.478127	19.769365	21.130983	5.19109656	5.35536651	5.55381089	1395	2095	2220
29775	NM_014550	CARD10	caspase recruitment domain fam	1.122301064	1.293679096	14.571163	16.373964	17.713896	5.81565859	5.97933194	6.18132852	2172	3257	3456
10232	NM_001177355,NM_005823,NM	MSLN	mesothelin	-1.965579101	1.29361874	24.816776	12.648238	30.284795	5.72824713	4.77926366	6.09348707	2042	1388	3249
55709	NM_001318716,NM_001318717	KBTBD4	kelch repeat and BTB domain co	-1.153185925	1.293586673	8.755013	7.568047	10.594184	4.42996298	4.23452662	4.7861128	807	935	1284
9604	NM_001201365,NM_004290,NM	RNF14	ring finger protein 14	-1.168362386	1.293432103	7.388297	6.325352	8.988931	4.98607799	4.7692132	5.34694775	1205	1378	1917
3927	NM_001271608,NM_006148,NR	LASP1	LIM and SH3 protein 1	1.431978304	1.293395873	132.103523	189.134734	159.933843	9.04855632	9.56574608	9.41910384	20750	39701	33009
29959	NM_001321357,NM_001321358	NRBP1	nuclear receptor binding protein 1	1.28245341	1.293071263	28.550793	36.655413	34.693854	5.99286804	6.34679007	6.35854095	2461	4217	3914
2677	NM_000821,NM_001142269,NM	GGCX	gamma-glutamyl carboxylase	1.218332226	1.292824254	13.285154	15.535361	12.454409	5.6120916	5.89171805	5.97594053	1881	3062	2991
23061	NM_015043,NM_198868	TBC1D9B	TBC1 domain family member 9B	1.388542329	1.292766605	21.071763	29.363344	25.651661	6.73127311	7.20105003	7.09866535	4132	7666	6570
54947	NM_017839	LPCAT2	lysophosphatidylcholine acyltrans	1.243818141	1.29246351	18.679115	23.262613	22.691772	6.62954348	6.94146779	6.99637556	3848	6395	6117
11198	NM_007192	SUPT16H	SPT16 homolog, facilitates chron	1.111949108	1.292429663	51.169209	56.95969	62.14621	7.87262127	8.02509384	8.24131753	9162	13612	14564
8303	NM_003498	SNN	stannin	-1.051519822	1.292228243	3.46365	3.29445	4.202192	3.59757921	3.53121349	3.94031242	436	554	693
55200	NM_001144856,NM_001144857	PLEKHG6	pleckstrin homology and RhoGEF	-1.312831676	1.292213947	10.954208	8.391905	13.305631	5.02835327	4.64939741	5.38819549	1242	1264	1974
84144	NM_032184	SYDE2	synapse defective Rho GTPase h	-1.626097205	1.292024029	1.239688	0.762625	1.507739	2.74535098	2.17238371	3.06566078	224	184	356
642	NM_000386	BLMH	bleomycin hydrolase	1.184365513	1.291849747	16.000488	18.968826	19.426378	5.34095955	5.57953754	5.702357	1552	2456	2466
115749	NM_001099676,NM_001170633	C12orf56	chromosome 12 open reading fra	-2.078184309	1.291703918	0.484744	0.230658	0.597393	0.99521185	0.56226574	1.19154816	39	25	62
79595	NM_001145928,NM_001330299	SAP130	Sin3A associated protein 130	1.204244651	1.291701891	9.635598	11.752757	11.778599	5.31811369	5.58011256	5.67922052	1527	2457	2426
155066	NM_001100592,NM_001289990	ATP6V0E2	ATPase H+ transporting V0 subu	-4.168613503	1.29165105	8.650668	2.072362	10.485772	4.58345526	2.70251092	4.93905904	902	289	1433
4600	NM_002463	MX2	MX dynamin like GTPase 2	-1.062904053	1.291610348	1.882201	1.778751	2.294157	2.68948191	2.61543261	3.00740444	214	269	340
27333	NM_001308155,NM_014498	GOLIM4	golgi integral membrane protein 4	-1.270904581	1.291599605	5.417953	4.297267	6.579094	4.5757712	4.2461677	4.93124635	897	943	1425
25827	NM_001171713,NM_001349316	FBXL2	F-box and leucine rich repeat pro	-1.056828224	1.291554308	6.083314	5.931626	7.091142	4.16701728	4.09182073	4.51793371	666	842	1058
5048	NM_000430	PAFAH1B1	platelet activating factor acetylhy	1.211265426	1.291529057	11.845276	14.362999	14.376687	6.03886537	6.31155856	6.4029981	2542	4114	4038
51763	NM_001135642,NM_016532,NM	INPP5K	inositol polyphosphate-5-phospha	-1.102896492	1.291496064	2.712377	2.781716	3.721339	3.30959645	3.18315375	3.64551432	350	424	556
257397	NM_152787	TAB3	TGF-beta activated kinase 1 and	1.133669431	1.291479564	2.611263	2.965977	3.171036	4.19135823	4.36304264	4.542501	678	1027	1077
54498	NM_001270691,NM_175839,NM	SMOX	spermine oxidase	3.146937369	1.291435364	29.87633	93.595496	36.348362	6.03438714	7.67328158	6.39840103	2534	10655	4025
53616	NM_001324417,NM_001324418	ADAM22	ADAM metallopeptidase domain	-1.63962664	1.291337742	1.947287	1.169328	2.655341	4.23884171	3.57341041	4.59041505	702	572	1115
201158	NM_001135036,NM_145301	TVP23C	trans-golgi network vesicle protei	1.217238242	1.291220114	3.05444	4.328702	4.026176	3.27204958	3.52883263	3.6068006	340	553	540
80854	NM_001306199,NM_001306200	SETD7	SET domain containing lysine me	1.051432391	1.291178949	8.182009	8.532591	9.3455	5.68533091	5.75631865	6.04770155	1981	2783	3146
7920	NM_001177515,NM_021160,NR	ABHD16A	abhydrolase domain containing 1	1.03265718	1.291163407	10.850649	11.22937	13.15252	4.48515183	4.52948015	4.83926507	840	1159	1334
2686	NM_001351702,NM_178026	GGT7	gamma-glutamyltransferase 7	1.319325553	1.291100841	8.102306	10.702428	9.838587	4.44859483	4.83235848	4.8022651	818	1442	1299
23152	NM_001304815,NM_015125	CIC	capicua transcriptional repressor	1.134868235	1.291097359	6.820554	8.484429	9.37142	5.62480753	5.80386305	5.98681712	1898	2878	3014
22924	NM_001303050,NM_012326	MAPRE3	microtubule associated protein R	1.038057386	1.291018851	13.043275	13.548689	15.816407	4.82687594	4.8789024	5.18391472	1075	1491	1707
642402	NR_111962	GOLGA6L17P	golgin A6 family-like 17, pseudog	-1.33555789	1.290932415	1.199101	0.896857	1.453426	1.66222564	1.38978355	1.92438744	85	85	135
10811	NM_001256067,NM_001256068	NOXA1	NADPH oxidase activator 1	-1.530317322	1.290835261	12.580291	8.164251	15.148255	4.35472906	3.77771682	4.70710452	764	667	1213
3371	NM_002160	TNC	tenascin C	2.007671355	1.290831352	6.505696	13.077718	7.892868	5.79396609	6.78641474	6.15641295	2139	5738	3396
4214	NM_005921	MAP3K1	mitogen-activated protein kinase	1.073751629	1.290517221	2.850041	3.065393	3.459137	4.45027676	4.54840945	4.80333552	819	1175	1300
114826	NM_052928	SMYD4	SET and MYND domain containi	1.246884512	1.290378856	3.284339	4.10402	3.987297	3.89547266	4.19452969	4.24134834	545	908	865
84079	NM_032139	ANKRD27	ankyrin repeat domain 27	-1.025080821	1.290358969	17.811306	17.394114	21.59638	6.28962054	6.25434409	6.65324904	3032	3952	4812
151477	NR_024079	LINC00471	long intergenic non-protein coding	1.144210548	1.290358718	0.685541	0.782054	0.829127	0.89996265	0.99371033	1.08290869	34	52	54
8470	NM_001145670,NM_001145671	SORBS2	sorbin and SH3 domain containir	-1.166128284	1.29030407	3.746641	3.069684	4.310265	4.31281697	4.10306728	4.66414974	741	849	1176
25839	NM_001195139,NM_015386	COG4	component of oligomeric golgi co	1.145214724	1.290265023	26.488795	30.345175	32.07516	6.20248825	6.39562695	6.56575443	2852	4364	4526
27252	NM_014458	KLHL20	kelch like family member 20	-1.051905123	1.289844358	4.582994	4.357388	5.556384	4.04097679	3.97250183	4.38840055	607	771	963
79178	NM_001126339,NM_001256062	THTPA	thiamine triphosphatase	1.952226076	1.289480433	2.313385	4.198922	2.883779	2.44147625	3.27124763	2.74755257	174	454	276
9715	NM_001031690,NM_001278297	FAM131B	family with sequence similarity 13	-1.259829354	1.289407568	0.10084	0.080566	0.122712	0.5190475	0.42561204	0.64076093	17	18	27
1262	NM_001037329	CNGA4	cyclic nucleotide gated channel a	1.843761865	1.289407568	0.221858	0.421055	0.278536	0.5190475	0.84852988	0.64076093	17	42	27
100287015	NR_040040	LOC100287015	uncharacterized LOC100287015	1.264352261	1.289387265	1.258325	1.591692	1.524384	1.98354948	2.24380223	2.26619062	116	196	184
3842	NM_002270,NM_153188	TNPO1	transportin 1	1.166289571	1.289380223	20.979903	24.508853	25.436134	7.45723148	7.6779897	7.82207081	6860	10690	10879
10362	NM_006339	HMG20B	high mobility group 20B	1.220322367	1.289175103	41.554501	50.781822	50.357513	6.02482449	6.30809176	6.38630822	2517	4104	3991
9319	NM_001166260,NM_004237	TRIP13	thyroid hormone receptor interact	1.092474403	1.289128508	15.58981	17.132148	18.866999	5.21008282	5.33438844	5.56773609	1414	2064	2242
79894	NM_024836	ZNF672	zinc finger protein 672	-1.07762539	1.28912529	7.510816	6.980438	9.100429	4.5618359	4.45870114	4.91450256	888	1101	1408
79627	NM_001324266,NM_024576	OGFRL1	opioid growth factor receptor like	1.129430439	1.288996342	1.620674	1.833896	1.964702	3.90776647	4.07233457	4.25236388	550	830	872
5576	NM_001321982,NM_001321983	PRKAR2A	protein kinase cAMP-dependent	1.070533534	1.288994629	2.551704	2.742557	3.522176	4.09573376	4.18850877	4.44299165	632	904	1002
56477	NM_001301873,NM_001301874	CCL28	C-C motif chemokine ligand 28	-1.888453008	1.288884398	3.398145	1.887687	4.179183	3.22566415	2.43901707	3.5569002	328	232	520
255104	NM_001349112,NM_001349113	TMCO4	transmembrane and coiled-coil d	-1.204475148	1.288870434	11.644463	9.766737	15.502456	5.31072594	5.04971445	5.66868681	1519	1685	2408
29922	NM_013330,NM_197972,NR_10	NME7	NME/NM23 family member 7	1.666090444	1.288814661	10.552308	17.653455	12.911498	4.16086733	4.86481307	4.5087761	663	1476	1051
441172	NR_033896	FLJ46906	uncharacterized LOC441172	1.250571651	1.288803888	1.806431	2.275409	2.20274	1.47662927	1.69172362	1.72191206	70	117	111
321	NM_001130414,NM_005503	APBA2	amyloid beta precursor protein bi	1.13654643	1.2886861	2.727901	3.104022	3.305391	3.40287721	3.57109823	3.73798383	376	571	596
254170	NM_203301	FBXO33	F-box protein 33	1.148244125	1.288492364	3.412014	3.924817	4.133683	4.17923909	4.36837876	4.52703356	672	1031	1065
8727	NM_001286974,NM_003798	CTNNAL1	catenin alpha like 1	-1.095391485	1.288402637	83.664039	76.398421	101.135765	7.71085112	7.5800595	8.07489679	8186	9985	12972
59349	NM_001303051,NM_001303109	KLHL12	kelch like family member 12	-1.12846917	1.288395502	19.514711	17.054256	23.146002	5.98708629	5.81563038	6.34757619	2451	2902	3884

3454	NM_000629	IFNAR1	interferon alpha and beta recepto	-1.142036113	1.28834041	5.908214	5.180229	7.156778	5.17287613	4.98692172	5.52943707	1377	1611	2182
201627	NM_152678	DENND6A	DENN domain containing 6A	-1.142360711	1.28806793	5.428907	4.75839	6.573624	4.67979317	4.49574422	5.03239442	967	1131	1532
10269	NM_005857	ZMPSTE24	zinc metallopeptidase STE24	1.300782996	1.287959167	23.624821	30.768896	28.596542	6.20597692	6.58084354	6.5667003	2859	4969	4529
26505	NM_017623,NM_199078	CNNM3	cyclin and CBS domain divalent r	-1.02441247	1.287841906	6.732538	6.570652	8.156997	5.05069925	5.01696186	5.40592109	1262	1646	1999
283337	NM_001004304	ZNF740	zinc finger protein 740	1.270022779	1.287740255	6.630636	8.428534	8.023095	4.83204569	5.16612031	5.18555761	1079	1831	1709
653082	NM_001351303,NR_147095,NR_	ZDHHC11B	zinc finger DHHC-type containing	-1.357432417	1.287614673	0.690388	0.511542	0.8555	2.0648444	1.74192207	2.35062252	125	123	198
10797	NM_006636,NR_027405	MTHFD2	methylenetetrahydrofolate dehyd	-1.007711486	1.28744306	68.281998	67.558464	82.38808	7.20117528	7.19016803	7.56349959	5738	7608	9086
26578	NM_012383	OSTF1	osteoclast stimulating factor 1	-1.201855575	1.287357418	26.398205	21.987875	31.92638	5.11890861	4.86197862	5.47405072	1325	1473	2098
84872	NM_001303124,NM_001303125	ZC3H10	zinc finger CCCH-type containing	1.665310045	1.286947621	0.95551	1.685226	1.184106	1.65056839	2.19057252	1.90855849	84	187	133
55652	NM_017842	SLC48A1	solute carrier family 48 member 1	-1.767613479	1.286693538	9.599998	5.439614	11.611736	4.87650783	4.09182073	5.22922293	1114	842	1763
4074	NM_001207024,NM_002355	M6PR	mannose-6-phosphate receptor,	1.02304907	1.286080504	52.354009	53.196904	62.360825	7.00743316	7.04005655	7.3679248	5012	6851	7928
388650	NM_001006605,NM_001252269	FAM69A	family with sequence similarity 69	1.296668845	1.28607068	6.218695	8.042759	7.492435	4.06312959	4.4181112	4.40682806	617	1069	976
25793	NM_001033024,NM_001257990	FBXO7	F-box protein 7	1.478180184	1.285921998	54.276326	81.015239	66.182167	6.83662465	7.39636887	7.19662649	4448	8785	7035
9278	NM_001145338,NM_005453	ZBTB22	zinc finger and BTB domain conta	1.034288458	1.28586341	5.652151	5.849949	6.827522	3.97005831	4.01565009	4.31224251	576	796	911
8795	NM_003842,NM_147187,NR_02	TNFRSF10B	TNF receptor superfamily membe	1.401104295	1.28584352	30.186356	42.349979	36.57078	6.93360184	7.41679284	7.29369847	4760	8911	7528
84250	NM_032290	SLF1	SMC5-SMC6 complex localizatio	1.170670643	1.285761151	6.353908	7.452259	7.682532	4.62127341	4.8400608	4.9708465	927	1450	1466
10498	NM_199141	CARM1	coactivator associated arginine m	-1.052759311	1.285722655	13.911987	13.22904	16.816071	5.36523338	5.29289816	5.720036	1579	2004	2497
7586	NM_001287054,NM_001287055	ZKSCAN1	zinc finger with KRAB and SCAN	-1.182507104	1.285647247	7.503227	6.356848	9.094615	6.15377238	5.91560689	6.51177102	2756	3114	4358
114787	NM_052899	GPRIN1	G protein regulated inducer of ne	1.459150573	1.285633073	2.596453	3.792823	3.136765	3.54187697	4.04760082	3.87664976	418	815	661
9188	NM_001256910,NM_004728	DDX21	DExD-box helicase 21	-1.260995613	1.285393273	32.708401	25.964971	39.502212	7.23912847	6.90704865	7.59922255	5892	6243	9315
53918	NM_015946	PELO	pelota homolog (Drosophila)	1.099438886	1.285327808	12.565601	13.827267	15.176122	5.19912125	5.33234202	5.55253827	1403	2061	2218
8467	NM_003601	SMARCA5	SWI/SNF related, matrix associat	1.264935993	1.285143648	14.260848	18.059645	17.22257	6.79520403	7.13155254	7.15425681	4321	7303	6830
10564	NM_006420	ARFGEF2	ADP ribosylation factor guanine n	-1.107900221	1.285094783	10.957438	9.902635	13.23421	6.60105791	6.45482824	6.95964139	3772	4549	5962
23450	NM_012426	SF3B3	splicing factor 3b subunit 3	1.354288359	1.284902637	22.490099	30.494497	27.157953	7.74053668	8.17631061	8.10070345	8357	15122	13207
55855	NR_027141_chr10,NR_027141_	FAM45BP	family with sequence similarity 45	-1.287648853	1.284859286	5.398629	4.197479	6.527977	3.72253824	3.38881456	4.05978318	479	497	757
1977	NM_001130678,NM_001130679	EIF4E	eukaryotic translation initiation fa	-1.025925875	1.284833525	5.202386	5.10166	6.287035	5.89189983	5.85560138	6.24810046	2292	2985	3622
114881	NM_145798	OSBPL7	oxysterol binding protein like 7	1.072806575	1.28442376	20.090153	21.578661	24.252014	6.18036414	6.28040713	6.53708616	2808	4025	4436
2564	NM_004961	GABRE	gamma-aminobutyric acid type A	1.467511489	1.284303363	41.696545	61.257195	50.32114	7.01085643	7.56066981	7.36937106	5024	9851	7936
163050	NM_144976	ZNF564	zinc finger protein 564	1.022309419	1.28426929	2.443581	2.504416	2.949959	3.00540462	3.03332122	3.32614297	276	377	436
58495	NM_001303461,NM_001303462	OVOL2	ovo like zinc finger 2	-1.212858223	1.284153914	2.3053	1.897834	2.78524	2.17473011	1.96260273	2.46326383	138	152	218
255812	NR_003264	SDHAP1	succinate dehydrogenase comple	1.059320034	1.28408086	25.934052	27.504463	31.301767	5.99805187	6.07992926	6.35379978	2470	3496	3901
10146	NM_005754,NM_198395	G3BP1	G3BP stress granule assembly fa	1.508835509	1.283921087	53.783198	81.212091	64.923786	7.21953718	7.80971346	7.57795764	5812	11717	9178
333926	NM_005167	PPM1J	protein phosphatase, Mg2+/Mn2+	-1.175536684	1.283889187	1.329115	1.135906	1.615443	1.69664266	1.5392062	1.95553366	88	100	139
11188	NM_001276293,NM_001276294	NISCH	nischarin	1.320402655	1.283768752	15.772488	20.332249	18.895689	6.08939006	6.48523156	6.44509779	2634	4647	4159
5966	NM_001291746,NM_002908	REL	REL proto-oncogene, NF-kB sub	1.470460828	1.283671594	1.140467	1.700593	1.395175	3.27963753	3.78770843	3.6068006	342	672	540
10892	NM_006785,NM_173844	MALT1	MALT1 paracaspase	-1.294105875	1.283435662	3.221853	2.481128	3.869665	4.06312959	3.71626888	4.40400834	617	637	974
6385	NM_002999	SDC4	syndecan 4	1.294602728	1.283433843	112.400116	145.683746	135.567932	8.16421429	8.53558195	8.52311531	11223	19413	17716
50488	NM_001024937,NM_001321236	MINK1	misshapen like kinase 1	1.103277177	1.283415419	17.868415	19.734028	21.485998	6.45288807	6.5931451	6.80924532	3400	5012	5367
10227	NM_001120,NM_001146069	MFSD10	major facilitator superfamily doma	1.460842647	1.283405642	66.728397	97.803341	82.175093	6.92697326	7.47003919	7.28433747	4738	9248	7479
113178	NM_001329539,NM_001329540	SCAMP4	secretory carrier membrane prote	1.20554771	1.283364395	36.198708	43.738672	43.609113	6.57931844	6.84643957	6.93592381	3715	5984	5864
374882	NM_001145416,NM_001321112	TMEM205	transmembrane protein 205	-1.191164949	1.283301577	77.313319	63.72989	96.593381	6.05110931	5.80287809	6.40617212	2564	2876	4047
642280	NR_027481	ZNF876P	zinc finger protein 876, pseudoge	-1.435786104	1.283207555	0.568518	0.397013	0.688112	1.29387258	1.00745609	1.51794971	57	53	90
23509	NM_015352,NM_172236	POFUT1	protein O-fucosyltransferase 1	1.343338194	1.283060973	15.553941	21.078762	18.987851	6.31476611	6.73596179	6.67036346	3086	5539	4870
57111	NM_020387,NR_133653	RAB25	RAB25, member RAS oncogene	-1.103219583	1.282808943	70.36432	63.722245	82.818558	6.22575196	6.08601495	6.58081438	2899	3511	4574
11276	NM_001163544,NM_001163545	SYNRG	synergin gamma	1.123724951	1.282732709	5.564326	6.325403	6.771513	5.49475122	5.6595227	5.84692246	1731	2599	2731
10121	NM_005736	ACTR1A	ARP1 actin related protein 1 hom	-1.371354205	1.282699124	25.504253	18.61689	30.740362	6.1768129	5.72857817	6.53160649	2801	2729	4419
3416	NM_001165946,NM_001322793	IDE	insulin degrading enzyme	-1.750217273	1.282628096	6.716882	3.870409	8.193785	5.32454708	4.54370036	5.67571782	1534	1171	2420
5976	NM_001297549,NM_002911	UPF1	UPF1, RNA helicase and ATPase	1.110845725	1.282564294	35.347236	39.331547	42.589647	7.53220137	7.68308403	7.88951931	7228	10728	11402
253959	NM_001283043,NM_001283044	RALGAPA1	Ral GTPase activating protein ca	1.09904284	1.282540951	3.637846	3.484642	4.034452	4.87149524	5.00330679	5.2196331	1110	1630	1751
493812	NR_026790	HCG11	HLA complex group 11 (non-prot	-1.025557604	1.282484829	1.959659	1.915124	2.363657	3.5668994	3.53359042	3.8988521	426	555	672
23185	NM_001351277,NM_015155	LARP4B	La ribonucleoprotein domain fam	1.134115195	1.282398414	13.66186	15.499394	16.468094	6.84400047	7.02408587	7.20008483	4471	6775	7052
6453	NM_001001132,NM_001331008	ITSN1	intersectin 1	-2.141287725	1.282361477	4.987876	2.331609	6.038679	4.76605286	3.72669368	5.1131673	1029	642	1623
221477	NM_001286635,NM_001286636	C6orf89	chromosome 6 open reading fran	1.043056348	1.282136894	6.590786	6.847232	7.913843	5.47092159	5.53039898	5.82231668	1702	2372	2684
54464	NM_001282857,NM_001282859	XRN1	5'-3' exoribonuclease 1	1.043820794	1.282136621	4.989054	5.701697	6.460635	5.49230426	5.55283585	5.84380456	1728	2410	2725
254013	NM_001135863,NM_001135864	ETFBKMT	electron transfer flavoprotein beta	-1.464854606	1.28211081	0.540034	0.367581	0.655097	1.10178443	0.83317479	1.30472178	45	41	71
5797	NM_001105244,NM_002845	PTPRM	protein tyrosine phosphatase, rec	1.171843529	1.282079496	3.764016	4.416736	4.53605	4.42825718	4.64720396	4.77196635	806	1262	1271
1121	NM_000390,NM_001145414,NM	CHM	CHM, Rab escort protein 1	1.870933829	1.281964886	3.660111	6.138397	3.759388	4.05209571	4.91490342	4.39125092	612	1530	965
23366	NM_001100425,NM_001199706	KIAA0895	KIAA0895	-1.285176017	1.281795241	3.521313	2.655424	4.3195	3.9817352	3.64550876	4.3197426	581	604	916
51362	NM_015891	CDC40	cell division cycle 40	1.003611771	1.281678525	3.863787	3.878435	4.650632	3.95355025	3.95841731	4.29103243	569	763	897
5906	NM_001010935,NM_001291896	RAP1A	RAP1A, member of RAS oncoge	-1.004234675	1.281527453	8.492012	8.37359	10.258445	5.41861558	5.41266157	5.76907242	1640	2182	2585
9439	NM_001270521,NM_001270522	MED23	mediator complex subunit 23	1.044970157	1.281435253	7.012414	7.33596	8.302444	5.1687957	5.23053515	5.51774626	1373	1917	2164
79888	NM_024830	LPCAT1	lysophosphatidylcholine acyltrans	1.771401776	1.281301356	13.373949	23.722101	16.107115	5.71850978	6.53145293	6.07010905	2028	4800	3196
26145	NM_015649	IRF2BP1	interferon regulatory factor 2 binc	1.114364774	1.281297713	7.380727	8.238745	8.888913	4.28103662	4.42964302	4.62230487	724	1078	1141
80222	NM_001271895,NM_001271896	TARS2	threonyl-tRNA synthetase 2, mito	1.038928173	1.281275607	10.987914	11.487559	13.236469	4.87525631	4.92851423	5.22203654	1113	1545	1754
2332	NM_001185075,NM_001185076	FMR1	fragile X mental retardation 1	1.196574033	1.281125241	13.852798	16.75522	16.65354	5.89560815	6.15054206	6.24770733	2298	3674	3621
126119	NM_001270639,NM_001270640	JOSD2	Josephin domain containing 2	-1.265621707	1.281030629	15.490291	12.872929	19.859078	3.96771155	3.65208684	4.30470323	575	607	906
128239	NM_178229	IQGAP3	IQ motif containing GTPase activ	-1.595243603	1.280894927	41.75079	26.199368	50.254711	7.94851577	7.27820177	8.30439025	9659	8090	15217
991	NM_001255	CDC20	cell division cycle 20	1.185231155	1.280819619	147.089279	174.52655	177.033981	7.91129651	8.15553063	8.26705304	9412	14905	14827
103	NM_001025107,NM_001111,NM	ADAR	adenosine deaminase, RNA spec	1.003445553	1.280646111	25.975437	26.114723	31.523441	7.3981135	7.40304655	7.75311516	6583	8826	10369
80216	NM_001102406,NM_001253884	ALPK1	alpha kinase 1	-1.482729747	1.280645347	11.570534	7.827847	13.959277	5.93097218	5.37405578	6.28266664	2356	2123	3711
284186	NM_178520	TMEM105	transmembrane protein 105	1.036562148	1.280397994	1.575363	1.63199	1.891742	2.60744196	2.65089709	2.9113909	200	277	315
6478	NM_005067	SIAH2	siah E3 ubiquitin protein ligase 2	1.205360112	1.28035351	10.607961	12.803202	12.76637	4.8112546	5.07196145	5.15653336	1063	1712	1674
10211	NM_001318875,NM_005803	FLOT1	flotillin 1	-1.406468867	1.280239623	46.333278	33.139127	55.918316	6.52568718	6.03994294	6.87867922	3578	3399	5634
55356	NM_018420	SLC22A15	solute carrier family 22 member 1	1.131380743	1.280147819	0.698887	0.794236	0.845283	2.0823043	2.2203867	2.36229074	127	192	200
10054	NM_005499	UBA2	ubiquitin like modifier activating e	-1.008538356	1.27985788	55.668652	55.258381	66.951271	7.18434629	7.1721648	7.53816554	5671	7513	8927
116236	NM_198147	ABHD15	abhydrolase domain containing 1	-1.244421742	1.279746525	10.684413	8.596415	12.855988	5.16675115	4.86103256	5.51382817	1371	1472	2158
101928483	NR_121577	NALT1	NOTCH1 associated lncRNA in T	-1.800902257	1.279222646	2.559389	1.434239	3.076972	1.24819466	0.81765451	1.46482043	54	40	85
23456	NM_012089	ABCB10	ATP binding cassette subfamily E	-1.085845407	1.278823943	7.204285	6.642811	8.661375	4.8112546	4.69682839	5.15485706	1063	1308	1672
440918	NR_147695	FLJ46875	uncharacterized LOC440918	-1.07801766	1.278612676	1.552548	1.442817	1.869296	3.23349937	3.1370039	3.55435929	330	409	519
246184	NM_139286	CDC26	cell division cycle 26	1.228434354	1.277969171	19.004961	23.402933	22.848198	4.09788139	4.37899217	4.43334413	633	1039	995
123606	NM_001142275,NM_144599	NIPA1	non imprinted in Prader-Willi/Ang	-1.316146786	1.277963426	9.983711	7.593538	11.992114	6.01860294	5.62928167	6.36761502	2506	2544	3939
92283	NM_001297623,NM_001322821	ZNF461	zinc finger protein 461	-1.028892204	1.277890843	2.331242	2.26763	2.809744	3.32800968	3.29105133	3.6502813	355	461	558
23770	NM_001308373,NM_012181	FKBP8	FK506 binding protein 8	1.007879549	1.277886075	84.699135	85.454525	101.706982	7.21830385	7.22955154	7.56996076	5807	7820	9127
149986	NM_144703	LSM14B	LSM family member 14B	1.138489959	1.277823679	18.574833	21.16745	22.301369	5.58248919	5.7659537	5.92963585	1842	2802	2895
4609	NM_002467	MYC	MYC proto-oncogene, bHLH tran	2.234033063	1.277756399	45.645168	102.107101	54.816284	6.73023514	7.88238064	7.08089953	4129	12325	6489
51161	NM_001171740,NM_001171741	C3orf18	chromosome 3 open reading fran	1.641775872	1.277247751	0.741073	1.22208	0.889066	1.56621863	2.07783221	1.80975447	77	169	121
5162	NM_000925,NM_001173468,NM	PDHB	pyruvate dehydrogenase (lipoam	-1.053360525	1.277182917	18.152676	16.874843	21.367647	4.80469545	4.7324405	5.14644623	1058	1342	1662
79669	NM_001171747,NM_024616	C3orf52	chromosome 3 open reading fran	1.380303317	1.277054703	7.296158	10.160967	8.884373	4.10430518	4.54605682	4.43886489	636	1173	999
117178	NM_001166293,NM_001166294	SSX2IP	SSX family member 2 interacting	1.356689236	1.276914947	3.008485	4.087042	3.595104	4.18126603	4.60035389	4.51662904	673	1220	1057
3190	NM_001318186,NM_001318187	HNRNPK	heterogeneous nuclear ribonucle	1.291431253	1.276840805	218.760873	283.332705	264.851343	9.33977462	9.70824431	9.69187184	25400	43828	39889
267004	NM_170753	PGBD3	piggyBac transposable element c	2.092276743	1.276764895	0.400341	0.840449	0.478432	0.91952222	1.52015496	1.09694451	35	98	55
55069	NM_017994	TMEM248	transmembrane protein 248	-1.170093021	1.276746182	20.010742	17.11841	24.008327	6.3850398	6.16134171	6.73377575	3242	3702	5091
221496	NM_001143944,NM_001348709	LEMD2	LEM domain containing 2	-1.093483628	1.276649137	20.387045	18.041136	22.769866	5.69954294	5.57319717	6.04589382	2001	2445	3142
3992	NM_013402	FADS1	fatty acid desaturase 1	-1.932439784	1.276588438	11.811898	6.117329	14.172114	5.69600304	4.7712289	6.04227156	1996	1380	3134
8565	NM_003680	YARS	tyrosyl-tRNA synthetase	1.073507004	1.276574567	50.929344	54.741257	61.106136	7.28155132	7.38324958	7.6318245	6069	8705	9529
117289	NM_001278733,NM_054114,NM	TAGAP	T-cell activation RhoGTPase acti	1.441721052	1.276544148	0.187168	0.268389	0.225786	0.77668602	1.02107213	0.93448595	28	54	44
79009	NM_024045	DDX50	DExD-box helicase 50	-1.315816197	1.27652086	14.040846	10.685697	16.851164	5.15854386	4.77525187	5.50200967	1363	1384	2140
388574	NR_029406	RPL23AP87	ribosomal protein L23a pseudoge	-1.333849241	1.276177017	0.235746	0.176097	0.291393	0.61813834	0.48576948	0.75138414	21	21	33
677811	NR_002964	SNORA28	small nucleolar RNA, H/ACA box	1.458890677	1.276177017	4.325265	6.250461	5.085809	0.61813834	0.83317479	0.75138414	21	41	33

125170	NM_001144900,NM_139162,NM	MIEF2	mitochondrial elongation factor 2	-1.045280776	1.276079975	6.146516	5.896781	7.404436	4.04987877	3.9899163	4.38268287	611	781	959
22859	NM_001008701,NM_014921	ADGRL1	adhesion G protein-coupled rece	1.596026325	1.275970511	3.740107	5.978342	4.494938	4.88896361	5.54519929	5.23001921	1124	2397	1764
10856	NM_001321190,NM_001321191	RUVBL2	RuvB like AAA ATPase 2	1.064623237	1.275700718	111.669116	119.099573	134.037813	7.78002669	7.86997226	8.12990141	8590	12219	13478
283635	NM_001079519,NM_001289022	FAM177A1	family with sequence similarity 17	-1.363960536	1.275690452	13.763713	10.051307	16.5279	5.39697596	4.96154856	5.74085701	1615	1582	2534
23365	NM_001198665,NM_001301084	ARHGEF12	Rho guanine nucleotide exchang	-3.403376185	1.27569004	8.893327	2.614473	10.647348	6.42407778	4.69682839	6.77173056	3332	1308	5228
122622	NM_001320424,NM_152328,NM	ADSSL1	adenylosuccinate synthase like 1	-1.11428319	1.275376363	6.223736	5.541173	7.453084	3.55444243	3.41225421	3.87868233	422	506	662
23253	NM_001083625,NM_001204056	ANKRD12	ankyrin repeat domain 12	-1.026813783	1.275374484	4.530449	4.412014	5.4092	5.66447154	5.6270574	6.00925532	1952	2540	3062
9493	NM_001281301,NM_004856,NM	KIF23	kinesin family member 23	-1.132723459	1.275170866	42.002983	37.799627	49.996429	7.19792526	7.0194296	7.54649995	5725	6753	8979
163081	NM_001300979,NM_001322911	ZNF567	zinc finger protein 567	-1.428248213	1.275112944	7.314978	5.094358	8.759897	4.40068652	3.91531944	4.73654166	790	739	1239
9581	NM_001042385,NM_001042386	PREPL	prolyl endopeptidase-like	1.252029501	1.274956953	9.406788	12.075988	11.435424	5.86756061	6.18686025	6.21268572	2253	3769	3533
255783	NM_178511,NR_037675	INAFM1	InaF motif containing 1	-1.126198837	1.274941589	14.037971	12.431327	17.038537	3.60967076	3.45300696	3.93446197	440	522	690
146691	NM_001033551,NM_001082968	TOM1L2	target of myb1 like 2 membrane t	-1.188810678	1.274878182	7.036371	5.599502	7.972466	5.27130713	5.02880493	5.61361286	1477	1660	2316
64748	NM_001170635,NM_022737	PLPPR2	phospholipid phosphatase relatec	1.077927946	1.27474953	5.913974	6.391069	7.089268	4.04987877	4.15184584	4.3812499	611	880	958
10616	NM_001323956,NM_001323958	RBCK1	RANBP2-type and C3HC4-type z	1.239943247	1.27459122	107.267321	132.313489	135.130931	7.76692685	8.07592212	8.11553789	8512	14102	13344
23196	NM_001286722,NM_001286723	FAM120A	family with sequence similarity 12	1.040399021	1.274473364	45.031454	46.685589	54.004857	7.79962102	7.85650719	8.14813103	8708	12105	13650
284361	NM_175063,NM_206538	EMC10	ER membrane protein complex s	1.521017381	1.274459188	37.674252	57.395715	45.10914	6.30550396	6.90429605	6.65146694	3066	6231	4806
55300	NM_018323,NR_144633	PI4K2B	phosphatidylinositol 4-kinase type	-1.131783109	1.274340387	8.718867	7.701561	10.450714	4.98259535	4.80998247	5.32251714	1202	1419	1884
53834	NM_001004356,NM_001004358	FGFRL1	fibroblast growth factor receptor-l	-1.155831754	1.274255828	35.141426	29.339524	40.52859	6.73679637	6.5299656	7.08354535	4148	4795	6501
163859	NM_152608	SDE2	SDE2 telomere maintenance hon	1.039341646	1.274135661	7.382497	7.68319	8.840351	4.89885139	4.95269469	5.23795796	1132	1572	1774
9337	NM_001301073,NM_001301074	CNOT8	CCR4-NOT transcription complex	-1.098259921	1.273915178	28.417371	26.218812	34.813541	6.16354552	6.03029685	6.50849312	2775	3376	4348
79654	NM_024602	HECTD3	HECT domain E3 ubiquitin protei	-1.01461009	1.273722701	7.297503	7.205371	8.741059	4.74428126	4.72413992	5.08175167	1013	1334	1587
54344	NM_018973,NM_153741	DPM3	dolichyl-phosphate mannosyltran	-1.159125822	1.27347208	28.623069	23.237084	33.444318	3.64534899	3.45049336	3.96921401	452	521	708
10419	NM_001039619,NM_001282953	PRMT5	protein arginine methyltransferas	1.294763602	1.273303144	36.719628	48.110494	43.913401	6.50762697	6.87671136	6.85280535	3533	6112	5533
391123	NM_001013661	VSIG8	V-set and immunoglobulin domai	2.437170513	1.273274696	0.093322	0.241452	0.120506	0.23670415	0.52452456	0.29607837	7	23	11
659	NM_001204	BMPR2	bone morphogenetic protein rece	1.184735452	1.273268424	3.459624	4.105219	4.14076	5.38119197	5.62036393	5.72230146	1597	2528	2501
388272	NM_001001436,NM_001348660	C16orf87	chromosome 16 open reading fra	1.166605181	1.273185271	2.053049	2.39484	2.441004	3.91509278	4.12372395	4.24292715	553	862	866
158584	NM_174912	FAAH2	fatty acid amide hydrolase 2	-1.081553451	1.273156019	1.766425	1.634353	2.108382	2.15835987	2.07130288	2.43591723	136	168	213
10474	NM_001278270,NM_006354,NM	TADA3	transcriptional adaptor 3	1.384690684	1.272973447	12.932944	18.011074	15.571819	5.03509348	5.49241457	5.37385651	1248	2309	1954
6814	NM_007269	STXBP3	syntaxin binding protein 3	-1.09118925	1.272840997	20.027269	18.373411	23.960373	5.65429253	5.53099392	5.99620882	1938	2373	3034
9114	NM_004691	ATP6V0D1	ATPase H+ transporting V0 subu	1.709450891	1.272729068	22.26671	38.116585	26.642687	5.21800288	5.97540322	5.55762202	1422	3248	2226
26258	NM_001311255,NM_001311256	BLOC1S6	biogenesis of lysosomal organelle	-1.055395994	1.272652762	9.720292	9.135195	11.534079	5.26463133	5.1889187	5.60443065	1470	1861	2301
10279	NM_005865	PRSS16	protease, serine 16	-1.293024861	1.27241889	3.293041	2.551625	3.937073	3.34979977	3.01982084	3.66684277	361	373	565
2997	NM_001161587,NM_002103,NR	GYS1	glycogen synthase 1	1.367116203	1.272362114	14.942039	20.31862	17.81423	5.73240025	6.17625063	6.0741057	2048	3741	3205
54432	NM_018982,NR_036639,NR_03	YIPF1	Yip1 domain family member 1	1.064427336	1.272217583	7.724535	8.846296	9.289196	3.88307318	3.96723624	4.20940382	540	768	845
1184	NM_000084,NM_001127898,NM	CLCN5	chloride voltage-gated channel 5	-1.469949534	1.272095811	0.879108	0.609378	1.085481	3.27204958	2.78463118	3.58704707	340	309	532
23313	NM_001009880,NM_015264	KIAA0930	KIAA0930	-1.077245932	1.271943856	3.331028	3.123037	4.017729	4.4162598	4.31410799	4.74881726	799	991	1250
2802	NM_001172557,NM_005895	GOLGA3	golgin A3	-1.360114474	1.271884278	17.501178	13.803409	22.349217	7.26683287	6.82646518	7.6118021	6007	5901	9397
6787	NM_001193533,NM_001348412	NEK4	NIMA related kinase 4	1.112107419	1.27175941	2.390901	2.625728	2.638166	3.7744826	3.91714113	4.09866803	498	740	779
56288	NM_001184785,NM_001184786	PARD3	par-3 family cell polarity regulator	1.026787762	1.271754849	8.795614	8.899456	9.259899	5.3670153	5.4042435	5.70636801	1581	2169	2473
9131	NM_001130846,NM_001130847	AIFM1	apoptosis inducing factor mitoch	1.312253271	1.271582477	22.488099	29.383571	26.72794	5.65429253	6.03952488	5.99480395	1938	3398	3031
3993	NM_001015002,NM_001031803	LLGL2	LLGL2, scribble cell polarity com	-2.104871469	1.271523642	87.829801	42.132575	107.40319	8.00296212	6.93541353	8.34832239	10032	6368	15689
1054	NM_001252296,NM_001806	CEBPG	CCAAT/enhancer binding protein	-1.109823077	1.271462429	19.60729	17.667873	23.387682	6.18643171	6.03826998	6.52869704	2820	3395	4410
10536	NM_014262	P3H3	prolyl 3-hydroxylase 3	-11.07580502	1.270893876	1.387725	0.117492	1.659064	2.20692365	0.40498891	2.48477392	142	17	222
126731	NM_145257	CCSAP	centriole, cilia and spindle associ	-1.132740736	1.270638493	5.687214	5.027262	6.792991	4.87650783	4.7031771	5.21159259	1114	1314	1741
113829	NM_080670	SLC35A4	solute carrier family 35 member /	1.181621753	1.270633531	19.523861	23.096369	23.311892	5.8267075	6.06357411	6.16684678	2189	3456	3421
9223	NM_001033057,NM_004742,NM	MAGI1	membrane associated guanylate	-1.095664163	1.27053105	4.641002	4.230891	5.504514	5.0306035	4.90300262	5.36663322	1244	1517	1944
5875	NM_004581,NM_182836	RABGGTA	Rab geranylgeranyltransferase a	-1.00694763	1.270522924	11.587334	11.354491	13.691137	4.63020414	4.62061907	4.96320452	933	1238	1458
64859	NM_001031716,NM_001254736	NABP1	nucleic acid binding protein 1	1.133880329	1.270517847	25.367243	29.927651	30.495454	6.6108664	6.79039565	6.95314577	3798	5754	5935
55684	NM_001173988,NM_001173989	RABL6	RAB, member RAS oncogene far	1.158005833	1.270456899	50.234071	59.613344	60.013727	7.2165754	7.42689741	7.55986232	5800	8974	9063
7626	NM_001185063,NM_007131,NR	ZNF75D	zinc finger protein 75D	1.069752868	1.270309687	2.048147	2.216741	2.506844	3.37481244	3.46301778	3.69017701	368	526	575
51633	NM_001286745,NM_016023	OTUD6B	OTU domain containing 6B	-1.053834647	1.269792075	5.732077	5.449621	6.847478	4.28857758	4.21688696	4.61744439	728	923	1137
10384	NM_001242803,NM_006994,NM	BTN3A3	butyrophilin subfamily 3 member	1.001887943	1.26972444	4.407804	4.402853	5.270546	3.78518394	3.78770843	4.10736121	502	672	784
140576	NM_001317007,NM_001317008	S100A16	S100 calcium binding protein A16	1.013457175	1.269644572	131.362905	127.142863	158.745114	7.31839448	7.33755991	7.6609037	6227	8432	9724
1540	NM_001042355,NM_001042412	CYLD	CYLD lysine 63 deubiquitinase	1.82964965	1.269549878	3.415458	6.257624	4.075787	4.88648104	5.73582152	5.22043469	1122	2743	1752
8019	NM_007371	BRD3	bromodomain containing 3	1.141384614	1.269253841	8.619933	9.850215	10.283173	5.59394673	5.78103713	5.93159518	1857	2832	2899
57189	NM_001080392	KIAA1147	KIAA1147	-1.294368359	1.269147071	6.670647	5.160901	7.95853	5.59698677	5.23346232	5.93452919	1861	1921	2905
57182	NM_001167882,NM_020337	ANKRD50	ankyrin repeat domain 50	1.607579002	1.268996475	6.992667	11.721812	8.340949	5.92856092	6.60449501	6.26723366	2352	5052	3671
26523	NM_001317122,NM_001317123	AGO1	argonaute 1, RISC catalytic comp	1.271508991	1.268976551	3.017236	3.762168	3.524551	4.49659199	4.82945947	4.82668598	847	1439	1322
64167	NM_001130140,NM_001329229	ERAP2	endoplasmic reticulum aminopep	-1.000044121	1.268866986	17.591041	17.542763	21.166008	6.60597049	6.60590749	6.94637855	3785	5057	5907
14	NM_001087,NM_001302545	AAMP	angio associated migratory cell p	1.434582585	1.268748491	66.885346	96.082859	79.77482	6.91148886	7.42849482	7.25236148	4687	8984	7314
10282	NM_001317739,NM_005868,NR	BET1	Bet1 golgi vesicular membrane tr	-1.524088354	1.268655112	12.871216	8.288897	15.385557	4.37965714	3.80748641	4.7082479	778	682	1214
6539	NM_001122847,NM_001122848	SLC6A12	solute carrier family 6 member 12	2.004697726	1.268510601	0.1148	0.235279	0.141127	0.49317359	0.86372326	0.60191771	16	43	25
22835	NM_001320666,NM_001320667	ZFP30	ZFP30 zinc finger protein	-1.384851502	1.268431919	2.033264	1.468354	2.43951	3.73086534	3.30224686	4.05079762	482	465	752
323	NM_001166050,NM_001166051	APBB2	amyloid beta precursor protein bi	-1.222290682	1.268306815	7.397191	5.987747	8.641419	5.97311481	5.68860967	6.3111659	2427	2653	3786
10975	NM_006830	UQCR11	ubiquinol-cytochrome c reductase	-1.023311555	1.268272923	51.010075	49.918621	60.811028	6.07310824	6.04036087	6.41144669	2604	3400	4062
130355	NM_001017927,NM_001322329	C2orf76	chromosome 2 open reading fran	-1.426959367	1.268118937	4.680508	3.12331	4.781706	2.0648444	1.69172362	2.3329413	125	117	195
7114	NM_021109	TMSB4X	thymosin beta 4, X-linked	1.121897022	1.268066422	1093.822388	1228.498901	1303.424072	9.38987796	9.55558517	9.73205489	26299	39422	41017
53827	NM_001164605,NM_001320912	FXYD5	FXYD domain containing ion tran	-1.446047771	1.268058444	161.946182	113.579796	199.514601	7.22323087	6.69540426	7.56381544	5827	5384	9088
55357	NM_001267571,NM_001267572	TBC1D2	TBC1 domain family member 2	1.67621533	1.268048271	19.044743	24.821184	17.741888	5.52539208	6.25794232	5.8613842	1769	3962	2759
8576	NM_001008910,NM_001330213	STK16	serine/threonine kinase 16	1.125379527	1.267885501	6.223106	6.741709	7.266669	4.19737984	4.35902755	4.52314065	681	1024	1062
51361	NM_015888	HOOK1	hook microtubule tethering protei	-1.017502358	1.267780253	7.00294	6.890375	8.341079	5.35539329	5.33097613	5.69025663	1568	2059	2445
50807	NM_001247996,NM_018482	ASAP1	ArfGAP with SH3 domain, ankyri	1.180287793	1.267618186	22.796831	27.093464	27.251665	7.14697969	7.38456689	7.48695545	5525	8713	8614
257160	NM_001077239,NM_001278249	RNF214	ring finger protein 214	1.002207935	1.267458183	7.408954	7.367673	8.64237	4.33847337	4.34149855	4.66532766	755	1011	1177
25870	NM_001042469,NM_001042470	SUMF2	sulfatase modifying factor 2	-1.217827903	1.267456394	56.172483	46.001178	66.68326	6.8064101	6.52489718	7.14563148	4355	4778	6789
677772	NR_003006	SCARNA6	small Cajal body-specific RNA 6	2.269805198	1.267161341	2.391576	5.566901	2.87109	0.71085839	1.29208824	0.85412573	25	76	39
101928687	NR_120641,NR_120642	LINC01468	long intergenic non-protein codin	-1.752393574	1.267161341	0.632925	0.354227	0.798741	0.71085839	0.44594451	0.85412573	25	19	39
7205	NM_003302	TRIP6	thyroid hormone receptor interact	-1.47799709	1.266625768	37.79245	25.596083	44.98365	6.03270421	5.47952756	6.36906159	2531	2288	3943
728554	NR_003615	LOC728554	THO complex 3 pseudogene	1.185906887	1.26643218	12.776388	15.180233	15.208978	4.56959435	4.80605531	4.89756216	893	1415	1391
55552	NM_001080493,NM_001297610	ZNF823	zinc finger protein 823	-1.774624861	1.266369505	5.719984	3.22462	6.813178	3.8780834	3.1244438	4.19805401	538	405	838
6734	NM_001177842,NM_003139	SRPRA	SRP receptor alpha subunit	-1.868673991	1.266241623	59.15874	31.694893	70.380075	7.48334147	6.58829422	7.82220283	6986	4995	10880
7403	NM_001291415,NM_001291416	KDM6A	lysine demethylase 6A	1.095375219	1.26619335	5.874348	6.334489	6.989844	5.08141332	5.20913369	5.41295079	1290	1888	2009
90141	NM_001284266,NM_001284267	EFCAB11	EF-hand calcium binding domain	-1.077122096	1.266172462	5.730754	5.201203	6.408361	3.90531607	3.80552077	4.22546449	549	681	855
91050	NM_001130726,NM_001330643	CCDC149	coiled-coil domain containing 149	1.320819641	1.266063818	1.172664	1.555085	1.427756	2.50111114	2.83945493	2.78704637	183	323	285
3030	NM_000182	HADHA	hydroxyacyl-CoA dehydrogenase	1.329353008	1.266032787	43.3377	57.670773	51.557865	7.01284959	7.42081106	7.35082176	5031	8936	7834
1058	NM_001042426,NM_001809	CENPA	centromere protein A	1.160773504	1.265900841	18.245322	21.444086	21.743565	4.71379852	4.92127111	5.04240188	991	1537	1543
7006	NM_003215	TEC	tec protein tyrosine kinase	-1.526801332	1.265704728	3.173491	2.084542	3.777007	3.60967076	3.05994879	3.92465818	440	385	685
23020	NM_014014	SNRNP200	small nuclear ribonucleoprotein U	1.157128647	1.265603041	45.704388	52.944927	54.355392	8.331999	8.54194185	8.67088678	12612	19499	19632
9044	NM_003972	BTAF1	B-TFIID TATA-box binding protei	-1.646326704	1.265575343	13.628297	8.288332	16.208685	6.56075397	5.85132032	6.89734604	3667	2976	5708
4302	NM_005937	MLLT6	MLLT6, PHD finger domain conta	-1.089297508	1.265268322	19.702324	18.106928	23.427935	7.17877726	7.05626571	7.51613747	5649	6929	8791
23780	NM_030882,NM_145637	APOL2	apolipoprotein L2	1.702150584	1.265235459	35.404831	59.666229	42.103291	6.47164217	7.2322978	6.80764596	3445	7835	5361
56180	NM_001306188,NM_019556	MOSPD1	motile sperm domain containing '	1.291579132	1.264979949	2.937121	3.677266	3.344651	2.92543304	3.2511683	3.22432601	259	447	403
57496	NM_001308142,NM_014048	MKL2	MKL1/myocardin like 2	1.740590648	1.264887469	5.087784	8.812037	6.030462	5.47257771	6.25830165	5.80478602	1704	3963	2651
3226	NM_017409	HOXC10	homeobox C10	-1.218842414	1.264838441	8.73768	7.178463	10.390192	4.14433812	3.87652288	4.46615621	655	718	1019
85019	NM_001318834,NM_032933,NR	TMEM241	transmembrane protein 241	-1.084417855	1.264826286	6.654708	6.065892	7.836037	4.33665583	4.22573375	4.66061022	754	929	1173
11042	NR_034021	SMA5	glucuronidase beta pseudogene	1.350992186	1.264471311	1.600911	2.172836	1.905858	1.90722898	2.23798388	2.16322937	108	195	168
85442	NM_001347864,NM_001347865	KNDC1	kinase non-catalytic C-lobe doma	1.306270061	1.264275561	0.553702	0.759639	0.677183	1.38110603	1.63086207	1.59908548	63	110	98
1737	NM_001931	DLAT	dihydrolipoamide S-acetyltransfe	-1.68135946	1.264219836	9.643345	5.737171	11.452386	5.38559376	4.65922688	5.71663112	1602	1273	2491
27111	NM_001199784,NM_015685,NM	SDCBP2	syndecan binding protein 2	-2.204121051	1.264158886	13.645822	6.485908	16.655296	4.45195674	3.38881456	4.7763339	820	497	1275
10449	NM_006111	ACAA2	acetyl-CoA acyltransferase 2	1.197075742	1.264114591	19.988194	23.957331	23.74613	5.2722583	5.52563064	5.60258718	1478	2364	2298

10005	NM_005469	ACOT8	acyl-CoA thioesterase 8	1.266278337	1.264111616	10.576791	13.422195	12.574654	3.70855202	4.02581645	4.02350017	474	802	737
401232	NR_026856	LINC01011	long intergenic non-protein codin	1.064287222	1.264087913	0.734043	0.791046	0.887616	1.10178443	1.15059938	1.29257653	45	64	70
348487	NM_182623	FAM131C	family with sequence similarity 13	-1.194679862	1.26365587	1.68654	1.408388	2.005408	1.92669094	1.74192207	2.18310266	110	123	171
11235	NM_007217,NM_145859,NM_14	PDCD10	programmed cell death 10	1.400124068	1.263244863	44.029756	62.295092	52.553369	5.74478835	6.22265489	6.07632129	2066	3865	3210
2235	NM_000140,NM_001012515	FECH	ferrochelatase	-1.10791279	1.263231241	1.572581	1.418857	1.867572	3.60061161	3.46550967	3.91280502	437	527	679
10683	NM_016941,NM_203486	DLL3	delta like canonical Notch ligand	-1.00207938	1.263107118	0.195109	0.194515	0.265116	0.54446555	0.54351856	0.65979727	18	24	28
2946	NM_000848,NM_001142368	GSTM2	glutathione S-transferase mu 2	1.328596534	1.263107118	0.346551	0.505372	0.453454	0.54446555	0.68708806	0.65979727	18	32	28
7533	NM_003405	YWHAH	tyrosine 3-monooxygenase/trypto	1.054553759	1.26277704	52.436245	55.352413	62.223743	6.53205449	6.60788264	6.86541598	3594	5064	5582
11328	NM_001284341,NM_001284343	FKBP9	FK506 binding protein 9	-1.073751023	1.262649586	17.027099	16.037436	20.437208	5.86881881	5.76797396	6.20014324	2255	2806	3502
25957	NM_001322405,NM_001322406	PNISR	PNN interacting serine and argini	1.033896469	1.262594434	39.584555	40.766643	46.870594	7.57484227	7.62268657	7.90966344	7446	10286	11563
55038	NM_017955,NM_145701	CDCA4	cell division cycle associated 4	1.272161187	1.262583671	19.748964	25.152193	23.501595	5.59013765	5.93101533	5.92029254	1852	3148	2876
64420	NM_001282640,NM_001282643	SUSD1	sushi domain containing 1	1.144145751	1.262512385	4.666829	5.307791	5.553248	3.88058045	4.06249189	4.1964253	539	824	837
30001	NM_014584	ERO1A	endoplasmic reticulum oxidoredu	1.455763351	1.262480422	43.211933	62.976391	51.266727	7.86365626	8.40349688	8.19863258	9105	17710	14138
2810	NM_006142	SFN	stratifin	-1.109585988	1.262353072	148.200073	133.714874	175.811981	7.57561302	7.42641784	7.91016035	7450	8971	11567
28996	NM_001113239,NM_022740	HIPK2	homeodomain interacting protein	-1.412367948	1.262315297	3.021878	2.142096	3.59116	5.51739157	5.03217088	5.84692246	1759	1664	2731
899	NM_001323538,NM_001761	CCNF	cyclin F	1.048997008	1.262123246	14.56694	15.520353	17.490961	5.95011857	6.01804174	6.28113075	2388	3347	3707
7528	NM_003403	YY1	YY1 transcription factor	1.415609929	1.262116421	38.215004	54.160294	45.328888	6.88934167	7.38719791	7.2226643	4615	8729	7164
81848	NM_001127496,NM_001293289	SPRY4	sprouty RTK signaling antagonist	1.478402305	1.261960537	5.371898	7.395273	6.370884	4.74838854	5.29500126	5.07290231	1016	2007	1577
23582	NM_012142,NR_027513,NR_02	CCNDBP1	cyclin D1 binding protein 1	-1.223143805	1.26177643	6.969881	5.708508	8.238981	4.65958016	4.38163336	4.98317903	953	1041	1479
9765	NM_001105251,NM_001284236	ZFYVE16	zinc finger FYVE-type containing	1.185555083	1.261721821	4.715398	5.282372	5.466055	5.57479997	5.81563038	5.90391945	1832	2902	2843
653188	NR_027386,NR_027386_dup1	GUSBP3	glucuronidase, beta pseudogene	1.161587898	1.261596722	0.625579	0.743095	0.74777	0.97665845	1.08728891	1.15176086	38	59	59
106635525	NR_132774	SNORA92	small nucleolar RNA, H/ACA box	1.819759044	1.261488548	0.888383	1.745075	1.13263	0.29776659	0.50527715	0.36729312	9	22	14
100009667	NR_131184	POU5F1P5	POU class 5 homeobox 1 pseud	-2.937033591	1.261488548	0.230826	0.083678	0.28809	0.29776659	0.10601836	0.36729312	9	4	14
7292	NM_001297562,NM_003326	TNFSF4	TNF superfamily member 4	-1.494302911	1.261488548	0.060245	0.040985	0.080324	0.29776659	0.20477631	0.36729312	9	8	14
102466748	NR_106904	MIR6845	microRNA 6845	-1.199655349	1.261488548	3.834383	3.019305	4.574493	0.29776659	0.25173066	0.36729312	9	10	14
407050	NR_029510	MIR93	microRNA 93	1.737576852	1.261488548	2.820108	5.089629	3.265409	0.29776659	0.48576948	0.36729312	9	21	14
7798	NM_001142546,NM_033631	LUZP1	leucine zipper protein 1	1.216906694	1.261392872	4.042619	4.888851	4.790459	5.09437892	5.37007127	5.42064411	1302	2117	2020
9061	NM_005443	PAPSS1	3'-phosphoadenosine 5'-phospho	1.03688399	1.261369226	21.010983	21.808369	24.906734	5.72824713	5.77953587	6.05760375	2042	2829	3168
10855	NM_001098540,NM_001166498	HPSE	heparanase	1.045662863	1.261348544	3.967531	4.149016	4.699897	4.2349439	4.29602113	4.55399371	700	978	1086
55614	NM_001199865,NM_001199866	KIF16B	kinesin family member 16B	1.016095831	1.261214174	4.501216	4.596824	5.261616	4.54619271	4.56825335	4.86819666	878	1192	1362
80727	NM_025250	TTYH3	tweety family member 3	1.11456689	1.261153904	15.324685	17.101078	18.161371	6.18895235	6.343407	6.51960771	2825	4207	4382
153768	NM_138492,NM_182960,NM_20	PRELID2	PRELI domain containing 2	-1.204852467	1.26111976	3.258504	2.662733	3.84246	4.03874269	3.78770843	4.35521005	606	672	940
285282	NM_173825	RABL3	RAB, member of RAS oncogene	1.445926696	1.260446134	2.870728	4.161657	3.405262	3.56999691	4.06413701	3.87868233	427	825	662
11231	NM_007214	SEC63	SEC63 homolog, protein transloc	1.209085848	1.260322687	10.958646	13.265689	12.979437	6.13663902	6.4070151	6.4662027	2723	4399	4221
51319	NM_001271834,NM_001271838	RSRC1	arginine and serine rich coiled-co	1.025544389	1.260174817	19.504067	20.051038	23.0727	5.08249824	5.11783013	5.40732977	1291	1769	2001
100132287	NR_028322_chr1,NR_028322_c	LOC100132287	uncharacterized LOC100132287	1.539430411	1.260124747	1.673784	2.581099	1.983816	3.02359429	3.58262222	3.32017262	280	576	434
731275	NR_029401	LINC01347	long intergenic non-protein codin	1.243452418	1.260116739	1.768067	2.206489	2.098831	2.93026199	3.20717639	3.22432601	260	432	403
55213	NM_018191	RCBTB1	RCC1 and BTB domain containin	1.007445103	1.260080682	3.049183	3.080943	3.614673	3.70009475	3.70997765	4.01058243	471	634	730
51631	NM_001244585,NM_001270643	LUC7L2	LUC7 like 2, pre-mRNA splicing f	-1.427158813	1.260045019	39.864674	27.949009	47.229863	6.73334681	6.22596555	7.06402906	4138	3874	6413
140838	NM_152667	NANP	N-acetylneuraminic acid phospha	1.304019905	1.259989825	3.18027	4.152469	3.76616	3.67442132	4.03087288	3.98439467	462	805	716
466	NM_005171	ATF1	activating transcription factor 1	-1.003191259	1.259948559	7.141473	7.121212	8.453422	4.20337642	4.19902895	4.52053953	684	911	1060
3717	NM_001322194,NM_001322195	JAK2	Janus kinase 2	-1.559296503	1.259905966	1.693387	1.065541	2.028111	3.27963753	2.71931318	3.58206614	342	293	530
4719	NM_001199981,NM_001199982	NDUFS1	NADH:ubiquinone oxidoreductas	1.28860448	1.259840049	19.48785	25.16913	23.072581	6.06215071	6.42312952	6.39094392	2584	4449	4004
54470	NM_001009584,NM_001184768	ARMCX6	armadillo repeat containing, X-lin	1.18957415	1.259739674	9.362486	11.719324	10.846147	3.71136013	3.94419393	4.02166185	475	755	736
7813	NM_001308248,NM_001350197	EVI5	ecotropic viral integration site 5	-1.22808699	1.259718175	1.593638	1.255286	1.861776	3.65413237	3.38355363	3.96347983	455	495	705
222171	NM_001329996,NM_001329997	PRR15	proline rich 15	-1.363369991	1.259587186	21.953744	16.074597	25.51362	5.03621379	4.60488193	5.3601012	1249	1224	1935
349667	NM_178570	RTN4RL2	reticulon 4 receptor like 2	1.738135305	1.259501576	0.469508	0.8201	0.554665	1.01352966	1.47139626	1.19154816	40	93	62
10896	NM_022375	OCLM	oculomedin	-1.986169758	1.259324161	3.581485	1.801345	4.264779	2.03825186	1.3579479	2.29691521	122	82	189
54718	NR_027795	BTN2A3P	butyrophilin subfamily 2 member	1.694255509	1.259146956	1.068607	1.817923	1.267509	1.84721644	2.43393638	2.09493548	102	231	158
6386	NM_001007067,NM_001007068	SDCBP	syndecan binding protein	-1.032954669	1.258989526	78.366197	75.870176	91.402821	7.38388295	7.33738986	7.71437631	6518	8431	10093
57118	NM_001351032,NM_020397,NM	CAMK1D	calcium/calmodulin dependent pr	6.691972671	1.258883427	0.264455	1.846472	0.765046	1.62696745	3.90617635	1.86000215	82	734	127
4884	NM_002522	NPTX1	neuronal pentraxin 1	2.722568707	1.258778307	0.096139	0.260647	0.113821	0.59399636	1.25799553	0.71544405	20	73	31
283455	NM_173598	KSR2	kinase suppressor of ras 2	-1.333736392	1.258778307	0.030718	0.022559	0.036051	0.59399636	0.46599441	0.71544405	20	20	31
10771	NM_001202464,NM_001202465	ZMYND11	zinc finger MYND-type containing	1.19357713	1.258607025	16.420329	20.625052	19.580936	5.94177327	6.19326394	6.2687844	2374	3786	3675
100271927	NM_001277335,NM_001277335	RASA4B	RAS p21 protein activator 4B	-1.969071985	1.258450188	0.573554	0.289539	0.686717	1.36692851	0.84852988	1.57922529	62	42	96
541578	NM_001013845	CXorf40B	chromosome X open reading fran	1.091452842	1.25808461	13.578063	14.833927	16.059649	4.22711655	4.34691481	4.542501	696	1015	1077
28987	NM_014062,NR_074074	NOB1	NIN1/PSMD8 binding protein 1 h	1.218408541	1.258019249	34.957099	42.615379	41.307493	5.93818188	6.21896745	6.26451583	2368	3855	3664
23347	NM_015295	SMCHD1	structural maintenance of chromo	1.074105456	1.257458058	14.37155	15.451747	16.981987	6.93480377	7.03712783	7.26290456	4764	6837	7368
1182	NM_001243372,NM_001243374	CLCN3	chloride voltage-gated channel 3	-1.048363668	1.257303018	11.624147	11.218685	13.821519	6.14911979	6.08196067	6.47529792	2747	3501	4248
10554	NM_006411,NM_032741	AGPAT1	1-acylglycerol-3-phosphate O-ac	-1.025048575	1.257261149	18.363508	17.940538	21.701084	5.37057255	5.33575111	5.69372425	1585	2066	2451
79712	NM_001006636,NM_001164629	GTDC1	glycosyltransferase like domain c	1.544506017	1.257149546	2.894296	5.047381	3.914945	3.16550307	3.73497962	3.4624849	313	646	484
147081	NR_026680,NR_027295,NR_13	LINC02210	long intergenic non-protein codin	1.268468019	1.257085877	15.140467	19.823214	15.687315	5.21503795	5.5499035	5.53717864	1419	2405	2194
123775	NM_001100873,NM_152337	C16orf46	chromosome 16 open reading fra	-1.223708769	1.256740234	3.165454	2.455999	3.73417	2.71208982	2.46913115	2.99621456	218	238	337
26065	NM_001114093,NM_015578	LSM14A	LSM14A, mRNA processing body	-1.08652526	1.25660714	32.196496	29.689195	38.097235	6.88779097	6.76912011	7.21484184	4610	5669	7125
91949	NM_153603	COG7	component of oligomeric golgi co	1.072714667	1.25630051	12.416015	13.333767	14.654468	5.19009033	5.28868274	5.51121019	1394	1998	2154
5536	NM_001204284,NM_006247	PPP5C	protein phosphatase 5 catalytic s	1.02535701	1.256240917	30.781145	31.620391	36.461866	6.08560737	6.12120968	6.41039332	2627	3599	4059
56006	NM_019108	SMG9	SMG9, nonsense mediated mRN	-1.130237353	1.256139468	12.725982	11.275012	15.022902	6.12510269	5.95115905	6.44989048	2701	3193	4173
440434	NR_036750	LOC440434	aminopeptidase puromycin sensi	-1.143564236	1.255882927	60.085228	52.600174	70.917656	6.60973804	6.41831407	6.9354357	3795	4434	5862
207063	NM_145177_chrX,NM_145177_c	DHRSX	dehydrogenase/reductase X-linke	1.362756815	1.255846402	2.614512	3.564227	3.087463	2.91572639	3.31058687	3.20501631	257	468	397
114803	NM_001085487	MYSM1	Myb like, SWIRM and MPN doma	1.262158777	1.255838462	9.09559	11.491648	10.733111	6.12510269	6.45670819	6.44954867	2701	4555	4172
57132	NM_020412	CHMP1B	charged multivesicular body prot	1.061679468	1.255794106	43.079769	45.788044	50.836586	7.01512412	7.10082603	7.3414569	5039	7148	7783
80035	NR_026808	ANP32A-IT1	ANP32A intronic transcript 1	1.472656299	1.255664645	0.26297	0.386081	0.308708	0.88013425	1.16293478	1.03996283	33	65	51
8971	NM_006026	H1FX	H1 histone family member X	1.234678167	1.255626275	69.870773	86.354424	82.443863	6.69378257	6.99527351	7.01935782	4025	6640	6216
2665	NM_001115156,NM_001494	GDI2	GDP dissociation inhibitor 2	1.131041086	1.255543357	71.668245	81.103134	84.537568	7.4018123	7.57847759	7.72839171	6600	9974	10192
201626	NM_001322176,NM_001322177	PDE12	phosphodiesterase 12	1.206166399	1.255518969	3.663727	4.329913	4.239886	4.34210161	4.60035389	4.6558773	757	1220	1169
6671	NM_001326542,NM_001326543	SP4	Sp4 transcription factor	1.27554083	1.255281049	1.826002	1.85242	1.739389	3.18583679	3.50238056	3.48133381	318	542	491
647310	NM_001195082	TEX22	testis expressed 22	1.189768076	1.255232562	0.220903	0.264672	0.262796	0.64188297	0.73744528	0.76902392	22	35	34
4130	NM_002373	MAP1A	microtubule associated protein 1/	1.223623142	1.255232562	0.055082	0.068639	0.066122	0.64188297	0.75384785	0.76902392	22	36	34
23568	NM_012106	ARL2BP	ADP ribosylation factor like GTPa	1.105393645	1.255137796	10.807345	11.959114	12.750741	4.53037804	4.66898984	4.84551373	868	1282	1340
285636	NM_175921	C5orf51	chromosome 5 open reading fran	1.040555095	1.254589076	3.291847	3.431318	3.882887	4.15675275	4.21095882	4.46750731	661	919	1020
10641	NM_006545	NPRL2	NPR2-like, GATOR1 complex su	1.206120628	1.254410037	7.31212	8.845129	8.634933	3.68588817	3.9370293	3.99004649	466	751	719
8664	NM_003753	EIF3D	eukaryotic translation initiation fa	1.580844631	1.254283874	213.641296	338.119293	251.821457	8.66684046	9.32623474	8.99298659	15917	33620	24555
51759	NM_016520	C9orf78	chromosome 9 open reading fran	-1.014570888	1.254266295	19.878983	19.620569	23.439913	5.17796048	5.1576698	5.49672576	1382	1820	2132
2124	NM_006495	EVI2B	ecotropic viral integration site 2B	1.680656492	1.254104063	6.935182	11.663788	8.168756	3.85541331	4.5636087	4.16179054	529	1188	816
90507	NM_001145023,NM_138355	SCRN2	secernin 2	1.069549691	1.254093726	31.562208	33.694895	37.405507	5.74889418	5.84415687	6.07010905	2072	2961	3196
90529	NM_001199012,NM_001199013	STPG1	sperm tail PG-rich repeat contain	-1.151638759	1.254024665	2.932716	2.54171	3.468328	3.13654562	2.95745524	3.42958826	306	355	472
7068	NM_000461,NM_001128176,NM	THRB	thyroid hormone receptor beta	1.080867041	1.253994863	1.531849	1.704281	1.848177	3.63355401	3.73704369	3.93641476	448	647	691
441478	NM_001004354	NRARP	NOTCH-regulated ankyrin repeat	1.150854113	1.25377872	2.238405	2.583625	2.645988	2.75082071	2.92523142	3.03318152	225	346	347
2769	NM_002068	GNA15	G protein subunit alpha 15	-1.026575243	1.253657809	5.97039	5.825816	7.033602	3.84266317	3.80748641	4.14837451	524	682	808
399512	NM_001320870,NM_001320871	SLC25A35	solute carrier family 25 member 3	-1.876108202	1.253514362	7.263794	4.008585	8.887551	4.12551355	3.28823882	4.4347263	646	460	996
10664	NM_001191022,NM_006565	CTCF	CCCTC-binding factor	-1.015946729	1.253070065	16.917612	16.670805	20.087823	6.04221495	6.0197383	6.36326659	2548	3351	3927
91445	NM_001135825,NM_152267,NR	RNF185	ring finger protein 185	1.230863252	1.252918883	4.329152	5.761751	5.421734	3.9320442	4.21392593	4.23818553	560	921	863
10686	NM_006580	CLDN16	claudin 16	-1.30795592	1.252862878	0.377019	0.29231	0.451375	1.15225689	0.95166931	1.34055586	48	49	74
284615	NM_001039888	ANKRD34A	ankyrin repeat domain 34A	1.169051928	1.252862878	0.345177	0.40672	0.405724	1.15225689	1.28081328	1.34055586	48	75	74
8717	NM_001323552,NM_003789	TRADD	TNFRSF1A associated via death	-1.105622177	1.252670675	16.650691	15.076108	19.604812	4.64790143	4.50909186	4.96128769	945	1142	1456
9343	NM_001142605,NM_001258353	EFTUD2	elongation factor Tu GTP binding	1.234816544	1.252484942	32.111016	39.700925	37.64099	7.03263119	7.3348367	7.35520781	5101	8416	7858
100037417	NM_001084393	DDTL	D-dopachrome tautomerase like	1.521206021	1.25228476	7.339764	11.176681	8.631462	3.66576097	4.23160161	3.96730515	459	933	707
283537	NM_001135919,NM_001347960	SLC46A3	solute carrier family 46 member 3	-2.066953281	1.252225756	1.259055	0.681833	1.480904	2.35073722	1.57657016	2.6172769	161	104	248
55604	NM_001173977,NM_017640	CARMIL1	capping protein regulator and my	-1.153900039	1.252144476	3.842617	3.335627	4.520298	4.41453771	4.2183652	4.72529065	798	924	1229

4122	NM_001320977,NM_006122,NR	MAN2A2	mannosidase alpha class 2A mer	-2.030800706	1.252039264	8.541743	4.302082	10.32459	5.78666216	4.79123253	6.10568646	2128	1400	3277
9647	NM_014634	PPM1F	protein phosphatase, Mg2+/Mn2+	1.650820723	1.252024811	4.163944	6.885112	4.903162	4.46199571	5.15920994	4.77305948	826	1822	1272
55746	NM_018230	NUP133	nucleoporin 133	1.03531405	1.25180525	16.611696	17.21389	19.542149	6.09692582	6.14627712	6.41670205	2648	3663	4077
57596	NM_001159531,NM_020836	BEGAIN	brain enriched guanylate kinase a	1.273570112	1.251774994	0.344673	0.445738	0.410632	0.95786335	1.1381576	1.12461302	37	63	57
662	NM_001205,NM_013978,NM_01	BNIP1	BCL2 interacting protein 1	-1.032542012	1.251760723	10.579344	10.330836	12.494575	3.82462152	3.78172178	4.1280136	517	669	796
347688	NM_177987	TUBB8	tubulin beta 8 class VIII	1.45099756	1.251534396	0.852616	1.256049	1.022402	1.18495016	1.51053416	1.37552141	50	97	77
80737	NM_025258	VWA7	von Willebrand factor A domain c	-1.650304251	1.251233794	0.794052	0.47956	0.937201	1.70793493	1.24644988	1.9400446	89	72	137
3033	NM_001184705,NM_001331027	HADH	hydroxyacyl-CoA dehydrogenase	1.259413247	1.251017679	17.231154	21.705455	20.247234	5.0976022	5.42167258	5.41224936	1305	2196	2008
254048	NM_173569	UBN2	ubinuclein 2	-1.233224619	1.250761614	1.909057	1.551042	2.245803	4.8007456	4.51029918	5.1131673	1055	1143	1623
55293	NM_001040697,NM_001261382	UEVLD	UEV and lactate/malate dehyrog	-1.690621033	1.250645453	8.533632	5.084569	9.925273	4.34391231	3.63447819	4.6523174	758	599	1166
25912	NM_001098616,NM_001297717	C1orf43	chromosome 1 open reading fran	-1.065545647	1.250557787	59.21148	56.049369	70.403973	6.75392153	6.66320264	7.07382021	4198	5264	6457
6234	NM_001031	RPS28	ribosomal protein S28	1.008818832	1.250320439	336.768951	340.138428	395.701965	6.98033279	6.99290029	7.30034791	4918	6629	7563
147699	NM_001080401	PPM1N	protein phosphatase, Mg2+/Mn2+	-1.37364763	1.25022275	2.208039	1.604349	2.597975	2.23060596	1.88281796	2.4901017	145	141	223
3632	NM_001321042,NM_005539	INPP5A	inositol polyphosphate-5-phospha	1.314927578	1.249905321	3.719359	4.892083	4.3724	3.57308778	3.9388238	3.8705348	428	752	658
4898	NM_001101662,NM_001242361	NRDC	nardilysin convertase	-1.128251001	1.249877147	43.507737	38.542927	51.012661	7.34530926	7.17235548	7.66532585	6345	7514	9754
63898	NM_001174159,NM_001174160	SH2D4A	SH2 domain containing 4A	1.207819941	1.249804303	17.451913	21.540025	20.775126	5.86251679	6.13066108	6.17926858	2245	3623	3451
5189	NM_000466,NM_001282677,NM	PEX1	peroxisomal biogenesis factor 1	-1.694881795	1.249783406	6.818129	4.012823	7.987116	4.91233736	4.18397654	5.22443598	1143	901	1757
100049076	NR_033968,NR_033968_dup1	GUSBP9	glucuronidase, beta pseudogene	1.380160411	1.249770544	1.083252	1.519013	1.306278	0.73313634	0.93737891	0.87055986	26	48	40
51673	NM_015964,NM_016140	TPPP3	tubulin polymerization promoting	-1.120064962	1.249770544	0.573743	0.563368	0.75166	0.73313634	0.669904	0.87055986	26	31	40
10120	NM_005735	ACTR1B	ARP1 actin related protein 1 hom	1.172663925	1.249612229	15.503211	18.209784	18.21125	5.13363806	5.35738055	5.44690975	1339	2098	2058
1173	NM_001025205,NM_001311198	AP2M1	adaptor related protein complex 2	1.546491275	1.249546062	89.318726	138.287385	104.887618	7.40289838	8.02888973	7.72260386	6605	13648	10151
440482	NR_040113	ANKRD20A5P	ankyrin repeat domain 20 family r	1.067465872	1.249383711	1.340628	1.432006	1.571373	2.54584744	2.6243808	2.81703332	190	271	292
5905	NM_001278651,NM_001317930	RANGAP1	Ran GTPase activating protein 1	1.438756456	1.249178878	111.706285	161.092231	129.607265	8.7452319	9.26903147	9.0655431	16808	32311	25824
100529251	NM_001202509,NM_001204098	CKLF-CMTM1	CKLF-CMTM1 readthrough	1.150074057	1.249172038	1.919232	2.2523	2.234851	1.24819466	1.36863803	1.4430085	54	83	83
79581	NM_001253815,NM_001253816	SLC52A2	solute carrier family 52 member 2	1.034500094	1.249148	36.763216	36.519745	42.787083	6.10655716	6.15479443	6.42333147	2666	3685	4096
10042	NM_001003681,NR_027780	HMGXB4	HMG-box containing 4	1.024833638	1.249136587	15.606184	15.992874	18.180403	6.0163339	6.05118491	6.33282566	2502	3426	3844
9443	NM_001100816,NM_004270	MED7	mediator complex subunit 7	1.080870631	1.249016155	19.521223	20.267584	23.439695	4.53196733	4.63950051	4.8403084	869	1255	1335
7791	NM_001010972,NM_003461	ZYX	zyxin	1.126414974	1.249010346	34.915401	39.348948	40.990935	6.31153111	6.48123514	6.62870105	3079	4634	4730
8445	NM_003583,NM_006482	DYRK2	dual specificity tyrosine phosphor	-1.274218331	1.24891148	6.644508	5.280572	7.879109	5.35269794	5.01270852	5.6663355	1565	1641	2404
8204	NM_003489	NRIP1	nuclear receptor interacting prote	1.117165978	1.248905672	12.75967	14.271607	14.976427	6.568131	6.7263834	6.88577077	3686	5502	5662
49856	NM_017818	WRAP73	WD repeat containing, antisense	-1.018468721	1.24886971	15.795571	15.522091	18.536371	4.76199564	4.73657294	5.07201438	1026	1346	1576
728769	NR_105014	SCAMP1-AS1	SCAMP1 antisense RNA 1	1.136921045	1.248865853	3.964048	4.514069	4.648692	2.80440683	2.96451939	3.08339379	235	357	361
7542	NM_006782	ZFPL1	zinc finger protein like 1	1.032033982	1.248856471	20.409145	21.087433	23.967541	4.83591088	4.87983681	5.14644623	1082	1492	1662
23077	NM_015057	MYCBP2	MYC binding protein 2, E3 ubiqui	-1.053388184	1.248765725	2.010867	1.910436	2.360008	4.92690697	4.85439274	5.23795796	1155	1465	1774
9390	NM_004256	SLC22A13	solute carrier family 22 member 1	1.264404815	1.248702752	0.131604	0.166632	0.148654	0.41264541	0.50527715	0.49997203	13	22	20
101340251	NR_102369	SNORD124	small nucleolar RNA, C/D box 12	-1.084841227	1.248702752	3.153536	2.856489	3.754404	0.41264541	0.38406669	0.49997203	13	16	20
7067	NM_001190918,NM_001190919	THRA	thyroid hormone receptor, alpha	-1.090271042	1.248616977	3.948269	3.635751	4.718441	3.40634723	3.29385837	3.69940604	377	462	579
5481	NM_005038	PPID	peptidylprolyl isomerase D	1.323349881	1.248527634	19.013794	25.19009	22.30851	5.12734384	5.52144537	5.43935559	1333	2357	2047
84771	NR_024004,NR_024005	DDX11L2	DEAD/H-box helicase 11 like 2	2.009309581	1.248414958	2.170212	3.523824	2.016678	2.19091668	3.02995795	2.44691815	140	376	215
7402	NM_007124	UTRN	utrophin	-1.100135464	1.248409342	1.479615	1.34745	1.737318	4.24272901	4.1126379	4.54762018	704	855	1081
79269	NM_001286810,NM_024345	DCAF10	DDB1 and CUL4 associated facto	1.078913729	1.248260445	2.42924	2.623167	2.849702	4.30354226	4.40778273	4.60889895	736	1061	1130
124056	NM_001267721,NM_144603,NM	NOXO1	NADPH oxidase organizer 1	-6.433544177	1.248180055	3.177137	0.486787	3.717087	2.59533164	0.83317479	2.86703245	198	41	304
11080	NM_001317099,NM_001317100	DNAJB4	DnaJ heat shock protein family (H	1.139558638	1.248072195	5.607815	6.400357	6.652163	4.11494833	4.2932183	4.41805218	641	976	984
6184	NM_002950	RPN1	ribophorin I	1.700291573	1.248053793	136.696472	232.698441	160.327881	8.28811888	9.05200409	8.60688402	12233	27791	18778
140901	NM_080836	STK35	serine/threonine kinase 35	1.520271257	1.247969763	11.210949	17.066286	13.150496	6.14963749	6.74701411	6.46518858	2748	5582	4218
51435	NM_016240,NM_182826	SCARA3	scavenger receptor class A mem	-15.03117011	1.247860703	0.818443	0.053931	1.086565	1.96484452	0.25173066	2.20918158	114	10	175
79018	NM_024052	GID4	GID complex subunit 4 homolog	-1.136855867	1.247740436	2.041149	1.797307	2.398085	3.23740108	3.07308051	3.5261099	331	389	508
283229	NM_001286606,NM_173584	CRACR2B	calcium release activated channe	-1.883196022	1.247692454	12.748279	6.760701	15.09507	4.5509035	3.69093766	4.85793043	881	625	1352
22845	NM_014908	DOLK	dolichol kinase	1.530794916	1.247656272	11.496299	17.625731	13.479445	4.68265777	5.27738104	4.99071621	969	1982	1487
653160	NR_037869	LOC653160	uncharacterized LOC653160	1.374231513	1.247526226	2.192995	3.023651	2.575701	2.33625658	2.71513092	2.59766727	159	292	244
10935	NM_001302272,NM_006793,NR	PRDX3	peroxiredoxin 3	-1.062359447	1.247511718	75.27383	69.456763	86.611368	6.87938828	6.79287816	7.19601533	4583	5764	7032
9633	NM_001039656,NM_004923	TESMIN	testis expressed metallothionein l	-1.607650557	1.247450074	7.043404	4.721427	8.498898	4.65082999	4.00026496	4.95840765	947	787	1453
55814	NM_018429	BDP1	B double prime 1, subunit of RNA	1.054036135	1.247362668	4.76558	5.028753	5.58618	5.71151407	5.7860301	6.02494052	2018	2842	3096
57591	NM_001282660,NM_001282661	MKL1	megakaryoblastic leukemia (trans	1.228621056	1.247213596	7.416038	9.193416	8.648988	5.05291493	5.34186724	5.36300797	1264	2075	1939
317760	NR_037933	ADAM20P1	ADAM metallopeptidase domain	1.36715105	1.247134894	0.499542	0.681075	0.5817	1.30878272	1.59489542	1.50747923	58	106	89
5468	NM_001330615,NM_005037,NM	PPARG	peroxisome proliferator activated	1.118020156	1.247108964	71.58986	78.818253	82.959271	7.03038412	7.19016803	7.34678947	5093	7608	7812
9170	NM_004720	LPAR2	lysophosphatidic acid receptor 2	-1.135288131	1.246922609	14.496308	12.785072	16.996433	4.71938895	4.54370036	5.0269064	995	1171	1526
375	NM_001024226,NM_001024227	ARF1	ADP ribosylation factor 1	-1.022634126	1.246830311	214.358056	209.736867	250.884842	8.6269636	8.59475589	8.9445082	15482	20228	23742
57561	NM_001329670,NM_001329671	ARRDC3	arrestin domain containing 3	1.730594463	1.246629873	17.158337	29.854145	20.221258	6.18844858	6.97136374	6.50257408	2824	6530	4330
1845	NM_004090	DUSP3	dual specificity phosphatase 3	-1.14062646	1.246366218	22.62237	19.85417	26.497475	6.52049297	6.33286878	6.83512022	3565	4176	5465
10845	NM_006660,NR_133680	CLPX	caseinolytic mitochondrial matrix	-1.153101229	1.246140036	7.469049	6.480401	8.740909	5.15648472	4.95712842	5.4659619	1361	1577	2086
53354	NM_138316,NM_148977,NM_14	PANK1	pantothenate kinase 1	-1.759040688	1.246081031	0.512937	0.291501	0.600757	2.16656821	1.57657016	2.41925694	137	104	210
124540	NM_001322250,NM_001322251	MSI2	musashi RNA binding protein 2	1.296826227	1.246053985	12.293313	15.49998	13.166051	6.05055502	6.42056328	6.36362946	2563	4441	3928
23199	NM_001134473,NM_001278184	GSE1	Gse1 coiled-coil protein	-1.466006316	1.246047958	15.11421	10.573258	18.035876	6.80312322	6.25722339	7.11793683	4345	3960	6659
10454	NM_006116,NM_153497	TAB1	TGF-beta activated kinase 1 (MA	1.282758409	1.246027385	33.086342	42.362904	38.644154	6.69945096	7.05564563	7.01405106	4041	6926	6193
25845	NR_024158	PP7080	uncharacterized LOC25845	1.629384067	1.245692735	3.712724	6.065264	4.353061	2.87121968	3.49751855	3.14885876	248	540	380
3201	NM_002141	HOXA4	homeobox A4	-4.872746509	1.245616497	1.158256	0.238102	1.355386	1.56621863	0.48576948	1.78395874	77	21	118
100526694	NM_001198812	MSANTD3-TMEFF1	MSANTD3-TMEFF1 readthrough	1.050179227	1.245581768	1.332524	1.400834	1.552192	2.05603457	2.11004283	2.30298238	124	174	190
55601	NM_017631	DDX60	DExD/H-box helicase 60	-1.982146745	1.245365049	11.470778	5.791072	13.423897	6.10495639	5.13827799	6.41740132	2663	1795	4079
5019	NM_000436	OXCT1	3-oxoacid CoA-transferase 1	1.034852511	1.245299643	8.235175	8.534765	9.641295	4.88523815	4.93302278	5.19211053	1121	1550	1717
414918	NM_001001794	DENND6B	DENN domain containing 6B	1.488452871	1.2447906	3.548776	5.302923	4.157998	2.99161027	3.50480544	3.27149895	273	543	418
23511	NM_015354	NUP188	nucleoporin 188	1.023240077	1.244183255	22.303976	22.847464	26.081263	6.96217535	6.99505793	7.27510823	4856	6639	7431
55023	NM_017934	PHIP	pleckstrin homology domain inter	1.168187046	1.244129225	5.1349	6.006295	6.003548	5.92735377	6.14821729	6.23784416	2350	3668	3596
81491	NM_001143957,NM_030784	GPR63	G protein-coupled receptor 63	-1.776868122	1.244128898	0.137255	0.077241	0.162854	0.86002952	0.54351856	1.01060517	32	24	49
100506639	NR_102752	LOC100506639	uncharacterized LOC100506639	1.465304337	1.243907613	0.819488	1.205449	0.957927	1.16869613	1.50084877	1.35230545	49	96	75
6840	NM_001323599,NM_001323600	SVIL	supervillin	-1.097525737	1.243886356	13.191315	11.577952	15.38648	6.46416976	6.33150339	6.7758262	3427	4172	5243
64342	NM_022460	HS1BP3	HCLS1 binding protein 3	-1.176459897	1.243534526	4.972414	4.228713	5.813079	3.65995838	3.44545299	3.95194263	457	519	699
427	NM_001127505,NM_004315,NM	ASAH1	N-acylsphingosine amidohydrolas	-1.071996329	1.2434373	22.690296	21.046192	26.425688	5.92735377	5.82875491	6.23705219	2350	2929	3594
23299	NM_001003800,NM_015250	BICD2	BICD cargo adaptor 2	1.239049608	1.243210193	11.737619	15.613915	15.626497	6.10602377	6.4112222	6.41600244	2665	4412	4075
55700	NM_001286365,NM_001286366	MAP7D1	MAP7 domain containing 1	1.318754339	1.243154274	60.499154	79.812368	70.864793	7.69000425	8.08749504	8.00264636	8068	14216	12336
339942	NR_026991	H1FX-AS1	H1FX antisense RNA 1	2.287303009	1.243088839	0.802745	1.850854	0.942063	1.44997615	2.31185141	1.65707652	68	208	104
80207	NM_001017989,NM_025136	OPA3	OPA3, outer mitochondrial memb	-1.051691345	1.242925401	4.857251	4.760851	5.317956	5.02044968	4.9500279	5.32550055	1235	1569	1888
163732	NM_133467	CITED4	Cbp/p300 interacting transactivat	-1.182557755	1.242886832	28.240679	23.906006	32.992569	5.19009033	4.95535656	5.49606391	1394	1575	2131
10311	NM_001331018,NM_001331021	DSCR3	DSCR3 arrestin fold containing	-1.357520335	1.242870642	9.668646	7.041907	11.130191	4.98375716	4.55894904	5.28852158	1203	1184	1839
440026	NM_001165030,NM_015012,NR	TMEM41B	transmembrane protein 41B	-1.595668522	1.242759904	43.558329	27.640126	51.284215	7.02785197	6.36024357	7.33924454	5084	4257	7771
83787	NM_001161009,NM_001161010	ARMC10	armadillo repeat containing 10	-1.33358273	1.242748327	11.192839	8.354362	12.954031	4.81908641	4.4206818	5.12263165	1069	1071	1634
6118	NM_001286076,NM_001297558	RPA2	replication protein A2	1.131639294	1.242539658	22.294785	24.85425	25.463182	5.32912486	5.50336969	5.63541459	1539	2327	2352
9811	NM_001142397,NM_014772	CTIF	cap binding complex dependent t	-1.029169067	1.242504329	2.302729	2.251321	2.725797	3.8142098	3.77571017	4.10736121	513	666	784
221294	NM_152729	NT5DC1	5'-nucleotidase domain containin	1.101624936	1.242366324	6.246424	6.896811	7.296343	4.3257022	4.45870114	4.62472899	748	1101	1143
11078	NM_001039141,NM_007032,NM	TRIOBP	TRIO and F-actin binding protein	1.244324198	1.242276787	43.470837	46.148353	49.110326	7.17191304	7.485315	7.48295272	5622	9347	8590
5920	NM_004585	RARRES3	retinoic acid receptor responder 3	-1.329128196	1.242187692	52.209118	39.312077	60.947594	5.31811369	4.91945464	5.62394919	1527	1535	2333
60528	NM_001165962,NM_018127,NM	ELAC2	elaC ribonuclease Z 2	1.097305009	1.242137538	7.922768	8.809743	9.327438	4.92327834	5.05303203	5.22683144	1152	1689	1760
10327	NM_001202413,NM_001202414	AKR1A1	aldo-keto reductase family 1 mer	-1.119861434	1.241986635	25.52083	22.993121	29.89035	5.24538562	5.08660446	5.55062723	1450	1730	2215
5608	NM_001330450,NM_002758	MAP2K6	mitogen-activated protein kinase	11.46879459	1.241894382	0.230396	1.45424	0.277254	0.5190475	2.58366954	0.62147005	17	262	26
158572	NR_026742	USP27X-AS1	USP27X antisense RNA 1 (head	2.893765435	1.241894382	0.189443	0.563924	0.223657	0.5190475	1.1751656	0.62147005	17	66	26
101927056	NR_125395,NR_125396	LOC101927056	uncharacterized LOC101927056	3.76876841	1.241894382	0.564276	2.47843	0.775792	0.5190475	1.40024122	0.62147005	17	86	26
55741	NM_001145025,NM_018217,NR	EDEM2	ER degradation enhancing alpha	1.139364007	1.241893051	17.869259	20.819398	21.569418	5.16060006	5.3439002	5.46528578	1365	2078	2085
80235	NM_025163	PIGZ	phosphatidylinositol glycan ancho	1.301370996	1.241795484	3.650836	4.761793	4.265407	3.42699388	3.77571017	3.71313983	383	666	585
25821	NM_001123226,NM_012123,NM	MTO1	mitochondrial tRNA translation op	1.125531754	1.241593883	5.671758	6.385984	6.649011	4.12340669	4.28477699	4.41944907	645	970	985

4292	NM_000249,NM_001167617,NM	MLH1	mutL homolog 1	1.271022385	1.241381652	8.635407	11.154239	10.091211	4.54146649	4.87422123	4.84135097	875	1486	1336
55738	NM_001281482,NM_001281483	ARFGAP1	ADP ribosylation factor GTPase a	-1.022632474	1.241322807	46.306809	45.26752	53.726933	7.18232368	7.15026011	7.49227519	5663	7399	8646
3661	NM_001197122,NM_001197123	IRF3	interferon regulatory factor 3	1.143256697	1.241314856	61.723426	71.133103	71.598156	6.60973804	6.80104024	6.91874074	3795	5797	5794
126353	NM_173481,NR_135168	MISP	mitotic spindle positioning	-1.439662629	1.241288804	35.787381	24.191856	41.042999	6.62545522	6.10611716	6.93445897	3837	3561	5858
154743	NM_152556	BMT2	base methyltransferase of 25S rF	1.10632639	1.241048605	1.367589	1.517845	1.59776	2.64904502	2.77260719	2.91535666	207	306	316
27433	NM_001085347,NM_001134430	TOR2A	torsin family 2 member A	-1.001316537	1.240930619	10.592963	9.790707	11.912973	4.12551355	4.12372395	4.42084462	646	862	986
57192	NM_020533	MCOLN1	mucolipin 1	1.106097189	1.240746378	6.202837	6.86794	7.240368	3.7744826	3.90984055	4.06514778	498	736	760
146540	NM_152458	ZNF785	zinc finger protein 785	1.524586083	1.240712063	2.145881	3.273247	2.498435	2.96832247	3.5120557	3.24338068	268	546	409
4893	NM_002524	NRAS	NRAS proto-oncogene, GTPase	1.170947365	1.240682935	10.96606	12.8536	12.781712	5.60153485	5.8248786	5.90691023	1867	2921	2849
90441	NM_033414	ZNF622	zinc finger protein 622	1.382866404	1.240673089	8.011481	11.101068	9.355625	3.82979934	4.2691718	4.12116227	519	959	792
6891	NM_000544,NM_001290043,NM	TAP2	transporter 2, ATP binding casse	-1.012906171	1.240175587	13.572683	13.464929	15.453312	6.2163926	6.19814177	6.52318535	2880	3799	4393
163049	NM_153358	ZNF791	zinc finger protein 791	1.223468201	1.240167637	5.364933	6.572662	6.255579	3.81159508	4.08373346	4.10215159	512	837	781
161424	NM_001286367,NM_174913	NOP9	NOP9 nucleolar protein	1.453003453	1.23961037	7.020568	10.22076	8.178597	5.40306784	5.93146604	5.70636801	1622	3149	2473
153684	NR_015447	LOC153684	uncharacterized LOC153684	2.316669953	1.239558897	0.210427	0.507945	0.254735	0.56944351	1.08728891	0.6785857	19	59	29
6794	NM_000455	STK11	serine/threonine kinase 11	1.157589551	1.239489904	24.115318	27.942934	28.090954	6.28397262	6.49258062	6.59014764	3020	4671	4604
57589	NM_001135920,NM_001206557	RIC1	RIC1 homolog, RAB6A GEF com	1.199220822	1.239170084	5.007665	6.101059	5.722902	4.98259535	5.23711295	5.28316064	1202	1926	1832
54929	NM_001256766,NM_017814	TMEM161A	transmembrane protein 161A	1.037342711	1.239015359	22.734886	23.598417	26.644925	5.37323474	5.42487721	5.67571782	1588	2201	2420
3141	NM_000411,NM_001242784,NM	HLCS	holocarboxylase synthetase	-1.047020551	1.238975715	4.665261	4.479181	6.204391	5.02044968	4.95624277	5.32102311	1235	1576	1882
53916	NM_016154	RAB4B	RAB4B, member RAS oncogene	-1.445297829	1.238950529	16.576506	11.481027	19.311481	4.33665583	3.83665412	4.63197698	754	697	1149
83548	NM_031431	COG3	component of oligomeric golgi co	-1.072009226	1.238871182	5.867599	5.478852	6.829742	4.76875136	4.67222953	5.06756652	1031	1285	1571
8291	NM_001130455,NM_001130976	DYSF	dysferlin	1.302555247	1.238870948	1.570679	2.041904	1.882392	3.54817338	3.90066256	3.83329054	420	731	640
4849	NM_014516	CNOT3	CCR4-NOT transcription complex	-1.033266737	1.238707606	27.326067	26.474558	31.812202	6.28208505	6.23548722	6.587354	3016	3900	4595
55355	NM_001282962,NM_001282963	HJURP	Holliday junction recognition prot	1.073793534	1.238684212	58.799853	63.20616	68.579346	7.49886488	7.60103475	7.80613996	7062	10132	10759
29919	NM_001276342,NM_001318707	C18orf8	chromosome 18 open reading fra	-1.112152171	1.238277724	18.085502	16.21096	20.174186	5.25022117	5.10110034	5.55126453	1455	1748	2216
65266	NM_001321299,NM_032387	WNK4	WNK lysine deficient protein kina	-1.691574178	1.238107327	3.420362	2.034367	3.981946	3.90040276	3.20717639	4.18989199	547	432	833
84818	NM_001203263,NM_001203264	IL17RC	interleukin 17 receptor C	-1.216080677	1.238097114	7.195832	5.949137	8.412051	4.19336823	3.9280232	4.48629114	679	746	1034
10954	NM_006810,NR_028444	PDIA5	protein disulfide isomerase family	1.324383806	1.238064135	15.582363	20.609006	18.050506	4.85635215	5.24945643	5.15485706	1098	1943	1672
7375	NM_001251877,NM_003363,NM	USP4	ubiquitin specific peptidase 4	1.011782502	1.237960209	8.213516	8.204059	9.509313	5.04736934	5.06376177	5.34694775	1259	1702	1917
79946	NM_024886	C10orf95	chromosome 10 open reading fra	1.175218372	1.237665903	0.364642	0.436769	0.422078	0.61813834	0.70406985	0.73352601	21	33	32
100128893	NR_102763	GATA6-AS1	GATA6 antisense RNA 1 (head t	1.104300296	1.237665903	0.292805	0.334986	0.346637	0.61813834	0.669904	0.73352601	21	31	32
1657	NM_001290321,NM_001290322	DMXL1	Dmx like 1	1.123487721	1.237630884	2.728246	3.157383	3.56398	5.12102205	5.28445497	5.42064411	1327	1992	2020
5504	NM_001291504,NM_001291505	PPP1R2	protein phosphatase 1 regulatory	1.239470148	1.237615667	21.414146	26.58452	24.899517	6.19548549	6.50141107	6.4992752	2838	4700	4320
122773	NM_172193	KLHDC1	kelch domain containing 1	-1.023069128	1.237580216	1.300551	1.273895	1.50987	2.14180175	2.11639955	2.38534784	134	175	204
51102	NM_001024732,NM_001349711	MECR	mitochondrial trans-2-enoyl-CoA	1.005680052	1.237558162	8.547023	8.349675	9.904483	4.47692439	4.48473082	4.77196635	835	1122	1271
197320	NM_001201407,NM_182531,NR	ZNF778	zinc finger protein 778	1.292351513	1.23742841	1.81152	2.362127	2.130949	2.87623322	3.20120812	3.1454863	249	430	379
9364	NM_001017979,NM_001159601	RAB28	RAB28, member RAS oncogene	1.155519409	1.23739686	7.972599	9.37629	9.498716	3.89793981	4.09343276	4.18661421	546	843	831
91147	NM_001142301,NM_153704,NR	TMEM67	transmembrane protein 67	2.075645872	1.237353199	2.673321	5.292474	3.033876	3.58844358	4.57864948	3.872576	433	1201	659
3312	NM_006597,NM_153201	HSPA8	heat shock protein family A (Hsp7	1.016155134	1.237318987	207.510348	211.180132	241.337853	8.95717274	8.98024739	9.26383497	19474	26440	29636
25819	NM_012118	NOCT	nocturnin	1.749575156	1.237122949	2.72016	4.78126	3.172144	2.64904502	3.35427048	2.9113909	207	484	315
23358	NM_015306	USP24	ubiquitin specific peptidase 24	1.008037408	1.237093186	9.606129	9.693834	11.169046	6.67304593	6.68448268	6.97729571	3967	5343	6036
9688	NM_001242795,NM_001242796	NUP93	nucleoporin 93	1.192640298	1.23674601	31.77253	37.952685	36.947501	6.51447631	6.76609107	6.81801022	3550	5657	5400
6195	NM_001006665,NM_001330441	RPS6KA1	ribosomal protein S6 kinase A1	-1.100554004	1.23672233	18.981533	17.30145	22.063555	5.91034682	5.77452033	6.21228282	2322	2819	3532
22862	NM_001079673,NM_001278438	FNDC3A	fibronectin type III domain contai	-1.235718463	1.236455555	6.822468	5.314797	7.926376	5.37323474	5.07604389	5.6727924	1588	1717	2415
90161	NM_001077188,NM_147175	HS6ST2	heparan sulfate 6-O-sulfotransfer	1.671944408	1.236333356	4.575573	7.661008	5.391256	4.4162598	5.13044769	4.70939036	799	1785	1215
6183	NM_021107,NM_033362,NM_03	MRPS12	mitochondrial ribosomal protein S	1.005019012	1.236322499	25.770076	26.122844	30.016273	4.75112025	4.75807621	5.04692788	1018	1367	1548
26524	NM_014572	LATS2	large tumor suppressor kinase 2	-1.733819063	1.236112744	5.678165	3.27715	6.595464	4.98375716	4.22279084	5.28085698	1203	927	1829
5500	NM_002709,NM_206876	PPP1CB	protein phosphatase 1 catalytic s	1.169042316	1.236099563	31.357077	37.820685	36.741591	7.25965677	7.48362566	7.56365752	5977	9336	9087
25798	NM_001159491,NM_015379	BRI3	brain protein I3	-1.79800369	1.236033185	66.996117	37.318614	77.801749	5.71571556	4.89100283	6.01619629	2024	1504	3077
57001	NM_020186	SDHAF3	succinate dehydrogenase comple	-1.005440644	1.235719921	2.8529	2.83971	3.316737	2.75082071	2.74415527	3.01481644	225	299	342
5441	NM_021128	POLR2L	RNA polymerase II subunit L	1.329185025	1.235627568	40.767365	54.266682	47.361317	5.16162707	5.56217955	5.45918641	1366	2426	2076
101928673	NR_125960	LOC101928673	uncharacterized LOC101928673	-1.494603819	1.235485367	1.073718	0.719288	1.244758	1.54118428	1.18729361	1.74883089	75	67	114
113730	NM_138433	KLHDC7B	kelch domain containing 7B	-1.08218088	1.23511057	2.273094	2.097585	2.634348	2.93026199	2.83174937	3.19852188	260	321	395
65264	NM_023079	UBE2Z	ubiquitin conjugating enzyme E2	1.164031536	1.234832074	49.486061	57.670944	57.431484	7.24446976	7.46226223	7.54697931	5914	9198	8982
91404	NM_178123	SESTD1	SEC14 and spectrin domain cont	1.45430122	1.234798292	2.333942	3.399914	2.710804	4.63020414	5.15226631	4.92339112	933	1813	1417
108783654	NR_144402	LOC108783654	uncharacterized LOC108783654	1.196322873	1.234717421	4.4259	5.305467	5.142568	3.48721804	3.72461474	3.76680378	401	641	609
101927599	NR_110703,NR_110704,NR_110	ZNF529-AS1	ZNF529 antisense RNA 1	-1.27126361	1.234680988	1.85685	1.380707	2.056186	1.59082596	1.36863803	1.80120704	79	83	120
112398	NM_053046,NM_080732	EGLN2	egl-9 family hypoxia inducible fac	1.063874713	1.234524351	19.785305	21.066525	22.946192	5.40999864	5.49729379	5.70751197	1630	2317	2475
8911	NM_001003406,NM_021096	CACNA1I	calcium voltage-gated channel su	2.610204256	1.23401083	0.363441	0.95	0.419813	2.18284609	3.35695728	2.42483178	139	485	211
29985	NM_144564,NM_213568	SLC39A3	solute carrier family 39 member 3	1.323943141	1.233965575	15.422352	20.397719	17.947624	4.70818633	5.09949686	5.00101595	987	1746	1498
1326	NM_001244134,NM_001320961	MAP3K8	mitogen-activated protein kinase	-2.618183553	1.233882253	3.718296	1.389615	4.290458	3.47073972	2.278228	3.74913666	396	202	601
5255	NM_001122670,NM_001172436	PHKA1	phosphorylase kinase regulatory	-1.1972216	1.233478222	6.217585	5.134366	7.115558	5.22686128	4.97472832	5.52230395	1431	1597	2171
2067	NM_001166049,NM_001983,NM	ERCC1	ERCC excision repair 1, endonuc	1.203841834	1.233432386	33.259993	43.62392	43.554763	6.15377238	6.41799247	6.45262202	2756	4433	4181
8724	NM_001300928,NM_001300929	SNX3	sorting nexin 3	1.249878144	1.233331822	24.140875	30.02927	27.524207	5.37854443	5.69340099	5.67454836	1594	2662	2418
55798	NM_018396	METTL2B	methyltransferase like 2B	-1.286928288	1.233267887	8.160812	6.342896	9.461256	4.19737984	3.85577671	4.48495752	681	707	1033
4773	NM_001136021,NM_001258292	NFATC2	nuclear factor of activated T-cells	-1.64317937	1.233235271	1.335352	0.814756	1.547384	3.43381096	2.80050884	3.71085992	385	313	584
254531	NM_153613	LPCAT4	lysophosphatidylcholine acyltrans	-1.443332372	1.233231353	46.495781	32.251339	53.89106	6.44952833	5.92740459	6.74885548	3392	3140	5145
10460	NM_006342	TACC3	transforming acidic coiled-coil co	1.135879353	1.233214555	123.120865	140.008728	142.680145	8.41400964	8.59731456	8.71563565	13352	20264	20252
1974	NM_001967	EIF4A2	eukaryotic translation initiation fa	-1.109242346	1.232943576	512.698059	462.719177	594.02301	9.88333037	9.73392218	10.18514933	37040	44616	56169
83481	NM_031308	EPPK1	epiplakin 1	-1.08426834	1.232939213	0.68884	0.63582	0.797321	2.67229041	2.57446443	2.9311116	211	260	320
58499	NM_001347997,NM_021224	ZNF462	zinc finger protein 462	1.615639935	1.232763259	2.893296	4.606826	3.310783	4.82946313	5.50215656	5.12177381	1077	2325	1633
1961	NM_001965	EGR4	early growth response 4	7.023379751	1.2326949	0.319385	2.253543	0.36887	0.79797761	2.63327382	0.93448595	29	273	44
142678	NM_001170686,NM_001170687	MIB2	mindbomb E3 ubiquitin protein lig	1.547971026	1.232493771	9.029561	13.965549	10.475447	4.81778406	5.42998986	5.10971027	1068	2209	1619
23239	NM_194449	PHLPP1	PH domain and leucine rich repe	1.122195496	1.232474028	3.461109	3.892014	4.012032	4.49984402	4.65922688	4.78935776	849	1273	1287
5347	NM_005030	PLK1	polo like kinase 1	1.365080572	1.232315165	45.801373	62.593166	53.043335	6.68023021	7.12545937	6.97895459	3987	7272	6043
9261	NM_004759,NM_032960	MAPKAPK2	mitogen-activated protein kinase-	1.158270018	1.232135309	19.900843	23.603633	22.945323	5.99113594	6.20001348	6.28802941	2458	3804	3725
5129	NM_002596,NM_212502,NM_21	CDK18	cyclin dependent kinase 18	1.052963825	1.231990413	14.918555	15.675343	17.254018	5.54913078	5.62204021	5.84432468	1799	2531	2726
6482	NM_003033,NM_173344	ST3GAL1	ST3 beta-galactoside alpha-2,3-s	1.052777853	1.231797257	18.380443	19.358392	21.171197	6.9419944	7.0156087	7.24055586	4788	6735	7254
7164	NM_001003395,NM_001003396	TPD52L1	tumor protein D52 like 1	1.115286146	1.231741047	11.28591	12.686402	13.187278	4.67979317	4.83139279	4.96989347	967	1441	1465
94101	NM_001128150,NM_016467	ORMDL1	ORMDL sphingolipid biosynthesis	1.148186488	1.231450732	16.2627	18.847113	19.378263	5.12734384	5.32137849	5.41994641	1333	2045	2019
101927204	NR_110184	LOC101927204	uncharacterized LOC101927204	-1.345694677	1.231265761	3.20895	2.390774	3.712763	3.00540462	2.63769986	3.27149895	276	274	418
257218	NM_001042683,NM_173082	SHPRH	SNF2 histone linker PHD RING h	1.00557297	1.231230785	1.856817	1.870957	2.152525	3.83495865	3.84241755	4.11600233	521	700	789
641808	NR_146190	FAM185BP	family with sequence similarity 18	-1.29558159	1.231110157	0.245538	0.188163	0.284682	0.88013425	0.72085408	1.02535867	33	34	50
100506046	NR_037605	GAS5-AS1	GAS5 antisense RNA 1	1.314410648	1.231110157	1.220466	1.618041	1.422111	0.88013425	1.07428646	1.02535867	33	58	50
100913187	NM_001193289	APOBEC3A_B	APOBEC3A and APOBEC3B del	1.608295428	1.231110157	0.764135	1.245458	0.90493	0.88013425	1.23481109	1.02535867	33	71	50
9520	NM_001330257,NM_006310	NPEPPS	aminopeptidase puromycin sensi	-1.405487764	1.231061662	114.699075	81.802287	133.140209	8.92207415	8.43220545	9.22142044	19005	18067	28776
646962	NM_001039792	HRCT1	histidine rich carboxyl terminus 1	-2.14819489	1.23100638	3.847984	1.793666	4.444915	2.19091668	1.41062362	2.43038515	140	87	212
26509	NM_013451,NM_133337	MYOF	myoferlin	-2.070050773	1.230976433	102.156773	49.347696	118.107616	9.40627192	8.35887293	9.7056772	26600	17169	40273
26128	NM_015634	KIF1BP	KIF1 binding protein	-1.444069825	1.230904695	18.413702	12.764444	21.301001	5.54362658	5.027119	5.83754849	1792	1658	2713
129685	NM_138572	TAF8	TATA-box binding protein associ	1.031982097	1.230771575	3.716701	3.8429	4.300112	4.05872616	4.10146599	4.34201172	615	848	931
7531	NM_006761,NR_024058	YWHAE	tyrosine 3-monooxygenase/trypto	1.198337709	1.230683671	180.112705	216.102945	208.350254	8.32925955	8.58955351	8.6278809	12588	20155	19054
1523	NM_001202543,NM_001202544	CUX1	cut like homeobox 1	-1.24684154	1.230561345	6.72413	5.218639	9.826843	5.52937575	5.21876673	5.8228446	1774	1901	2685
134553	NM_001135586,NM_001300894	C5orf24	chromosome 5 open reading fran	1.327970335	1.230476241	7.94685	9.28734	8.910323	5.09545415	5.49424621	5.38676798	1303	2312	1972
10999	NM_005094	SLC27A4	solute carrier family 27 member 4	1.066529752	1.230398257	8.892999	9.499129	10.283557	4.84232994	4.93212219	5.13203431	1087	1549	1645
11034	NM_001011546,NM_006870	DSTN	destrin, actin depolymerizing fact	1.155505787	1.230393685	132.683014	154.731953	155.050329	7.73122812	7.93884108	8.02907983	8303	12819	12565
5524	NM_001193397,NM_001271832	PTPA	protein phosphatase 2 phosphata	1.0777267	1.230050731	34.279909	37.176858	39.787385	6.53720732	6.64408099	6.83302529	3607	5194	5457
10559	NM_001168398,NM_006416	SLC35A1	solute carrier family 35 member A	-4.476454684	1.229975617	73.493448	16.552563	84.644398	7.09120758	4.96507495	7.38786329	5314	1586	8039
10383	NM_006088	TUBB4B	tubulin beta 4B class IVb	1.131748951	1.229946474	1069.345459	1211.567993	1235.959473	10.69476057	10.87321349	10.99319383	65033	98340	98379
105376382	NR_134491	LOC105376382	uncharacterized LOC105376382	-2.893558532	1.229935206	2.798033	0.952985	3.255979	1.55375576	0.73744528	1.75769337	76	35	115
57492	NM_001346813,NM_017519,NM	ARID1B	AT-rich interaction domain 1B	1.096314303	1.22990277	3.582893	3.951514	4.198745	5.13049438	5.25954295	5.42134148	1336	1957	2021

94059	NM_001301782,NM_198988	LENG9	leukocyte receptor cluster memb	-1.795079121	1.229442911	1.640927	0.917199	1.891446	2.02927762	1.44132992	2.25996639	121	90	183
57026	NM_020315	PDXP	pyridoxal phosphatase	1.587948845	1.228753071	9.210092	14.641226	10.632506	4.24660587	4.88543063	4.52962301	706	1498	1067
91252	NM_001128225,NM_001330245	SLC39A13	solute carrier family 39 member 1	-1.232373718	1.228744173	6.84781	5.505846	8.067019	4.06532627	3.78372009	4.34642459	618	670	934
3139	NR_027822	HLA-L	major histocompatibility complex,	-1.1722642	1.228732256	0.486629	0.411957	0.558293	1.67378945	1.52015496	1.88448459	86	98	130
23595	NM_001197259,NM_012381,NM	ORC3	origin recognition complex subun	1.201992898	1.228666032	11.846789	14.220723	13.625916	4.93293448	5.19042586	5.22123584	1160	1863	1753
64419	NM_001077525,NM_001077526	MTMR14	myotubularin related protein 14	1.280773805	1.228524956	7.874518	10.090258	9.010729	4.28103662	4.62173661	4.56413316	724	1239	1094
23322	NM_001127897,NM_001308334	RPGRIP1L	RPGRIP1 like	2.139494336	1.228208128	3.030395	5.734199	2.971548	3.6915876	4.72829618	3.96730515	468	1338	707
1352	NM_001303	COX10	COX10, heme A:farnesyltransfera	1.133832782	1.22819028	2.027033	2.304921	2.343332	2.79384765	2.95035633	3.05131575	233	353	352
5890	NM_001321809,NM_001321810	RAD51B	RAD51 paralog B	1.329423106	1.228098891	6.3091	8.500139	8.258188	3.70855202	4.09182073	3.98439467	474	842	716
100507459	NR_109990	NRSN2-AS1	NRSN2 antisense RNA 1	1.575834021	1.227953959	1.759365	2.774416	2.033765	1.76310734	2.255369	1.97845985	94	198	142
3209	NM_000522	HOXA13	homeobox A13	1.472953101	1.227779629	0.996423	1.47031	1.147589	1.78459878	2.20257223	2.0010274	96	189	145
23403	NM_001080469,NM_001329632	FBXO46	F-box protein 46	-1.027421589	1.227637625	10.071602	9.776748	11.642684	5.10081829	5.06293923	5.38890871	1308	1701	1975
55114	NM_001006634,NM_018054	ARHGAP17	Rho GTPase activating protein 17	2.82478505	1.227596692	3.445531	9.840167	4.071248	3.64828273	5.07032524	3.92268941	453	1710	684
4323	NM_004995	MMP14	matrix metallopeptidase 14	-4.165023078	1.227576149	25.867729	6.212952	29.841703	6.51608321	4.50667419	6.80897888	3554	1140	5366
9392	NM_001142621,NM_001328646	TGFBRAP1	transforming growth factor beta re	1.727132909	1.227573357	4.080139	6.91037	4.657032	4.51920371	5.28092234	4.80333552	861	1987	1300
64393	NM_022470,NM_152240	ZMAT3	zinc finger matrin-type 3	1.158229016	1.227558883	1.604887	1.876358	1.844422	3.92480368	4.12372395	4.20292915	557	862	841
140700	NM_080621	SAMD10	sterile alpha motif domain contair	1.106128171	1.227516183	9.787987	10.835093	11.284318	4.44522507	4.5843929	4.72867518	816	1206	1232
5870	NM_001243718,NM_001243719	RAB6A	RAB6A, member RAS oncogene	1.051347612	1.227495228	30.842123	31.924476	34.244173	6.6291723	6.700702	6.92219377	3847	5404	5808
144245	NM_001013620,NM_001308340	ALG10B	ALG10B, alpha-1,2-glucosyltrans	-1.099578972	1.227392579	3.551095	3.373461	4.138852	5.08899074	4.95624277	5.37673573	1297	1576	1958
84838	NM_001329733,NM_032752	ZNF496	zinc finger protein 496	-1.195831667	1.227180547	11.184752	9.346194	13.107303	5.8606208	5.60744732	6.15137779	2242	2505	3384
5690	NM_001199779,NM_001199780	PSMB2	proteasome subunit beta 2	1.255286541	1.227156079	36.84138	45.296775	41.044834	7.35297208	7.67919788	7.64666103	6379	10699	9628
100302692	NR_028379	FTX	FTX transcript, XIST regulator (no	-1.135381379	1.227048332	0.555589	0.495568	0.652537	1.2010231	1.10017522	1.37552141	51	60	77
22879	NM_001286639,NM_001286640	MON1B	MON1 homolog B, secretory traff	-1.066877301	1.227019317	14.459798	13.249316	16.28159	5.54913078	5.45779006	5.83859305	1799	2253	2715
283596	NR_001459,NR_003138	SNHG10	small nucleolar RNA host gene 1	1.218671854	1.226909149	16.525239	19.535775	16.408658	4.63907992	4.91399145	4.92339112	939	1529	1417
9798	NM_001270975,NM_001270976	IST1	IST1, ESCRT-III associated facto	-1.166982345	1.226898701	59.751093	51.037783	66.749995	7.83658282	7.61485073	8.13043461	8935	10230	13483
6497	NM_003036	SKI	SKI proto-oncogene	1.297092397	1.226848237	6.646808	8.62845	7.661821	5.24538562	5.61195318	5.53331305	1450	2513	2188
54540	NM_001190946,NR_024019	FAM193B	family with sequence similarity 19	-1.410854227	1.22677464	34.073191	25.641163	38.994875	7.17750857	6.68501736	7.47054062	5644	5345	8516
2033	NM_001429	EP300	E1A binding protein p300	1.226146862	1.226640913	6.173174	7.579863	7.118069	5.74615826	6.03533764	6.03591064	2068	3388	3120
3185	NM_001098204,NM_001098205	HNRNPF	heterogeneous nuclear ribonucle	-1.178014978	1.226612071	113.717872	97.003351	131.717213	8.22012139	7.98462215	8.51390891	11668	13234	17603
60682	NM_001044305,NM_001281439	SMAP1	small ArfGAP 1	1.200523759	1.226470322	4.672752	5.616116	5.383499	3.99331833	4.24181329	4.27105417	586	940	884
81558	NM_030802	FAM117A	family with sequence similarity 11	1.055180702	1.226446877	6.509243	6.877799	7.508896	3.99792567	4.07069878	4.27568916	588	829	887
57614	NM_001346229,NM_001346230	KIAA1468	KIAA1468	1.060929186	1.226424022	5.855339	6.183828	6.742879	5.02947882	5.11227506	5.31578181	1243	1762	1875
2584	NM_000154	GALK1	galactokinase 1	-1.194106803	1.226155996	11.791138	9.890813	13.59304	4.05651939	3.81727465	4.33462681	614	687	926
2937	NM_000178,NM_001322494,NM	GSS	glutathione synthetase	1.120866891	1.225987221	53.369864	60.197645	62.658758	6.68166279	6.84476562	6.97302126	3991	5977	6018
79794	NM_024738	C12orf49	chromosome 12 open reading fra	-1.339886556	1.225937513	3.461617	2.58766	3.991219	4.23299104	3.83665412	4.51270791	699	697	1054
4778	NM_001136023,NM_001261461	NFE2	nuclear factor, erythroid 2	-3.381787658	1.225825665	0.879561	0.259513	1.081763	1.35261029	0.54351856	1.53866543	61	24	92
55876	NM_001042471,NM_001165958	GSDMB	gasdermin B	-1.041720534	1.225457254	38.556433	36.909993	44.328025	5.92493645	5.86695581	6.21389375	2346	3009	3536
5471	NM_002703	PPAT	phosphoribosyl pyrophosphate ai	1.512972133	1.225290608	8.510939	12.893148	9.800693	4.98607799	5.56798878	5.27083173	1205	2436	1816
23139	NM_001319245,NM_001324320	MAST2	microtubule associated serine/thr	-1.329386053	1.225268176	23.423258	17.55713	26.960738	7.04297863	6.63580778	7.33406914	5138	5164	7743
4791	NM_001077494,NM_001261403	NFKB2	nuclear factor kappa B subunit 2	-1.29741921	1.224886458	35.443094	27.511911	39.237934	6.80772276	6.43589271	7.09801125	4359	4489	6567
2800	NM_002077	GOLGA1	golgin A1	1.08243312	1.224699817	8.477429	9.1826	9.755043	5.36790544	5.47952756	5.65392788	1582	2288	2383
5713	NM_002811	PSMD7	proteasome 26S subunit, non-AT	-1.000651969	1.22468995	87.084549	87.138718	100.236099	7.15602603	7.15509232	7.4465908	5560	7424	8375
79679	NM_001253849,NM_001253850	VTCN1	V-set domain containing T-cell ac	1.257003164	1.224660868	0.714867	0.898612	0.96409	1.54118428	1.76638114	1.73991364	75	126	113
23093	NM_015072	TTLL5	tubulin tyrosine ligase like 5	-1.040168755	1.224652839	5.255511	5.058506	6.048476	4.66393542	4.6093958	4.94586061	956	1228	1440
55632	NM_001308097,NM_017769	G2E3	G2/M-phase specific E3 ubiquitin	-1.026547253	1.224472571	14.956905	14.59621	17.209537	6.41721445	6.37986127	6.70628577	3316	4316	4994
440288	NR_038448,NR_038449,NR_12(	UBL7-AS1	UBL7 antisense RNA 1 (head to	-1.011449462	1.224439866	2.954126	2.903433	3.277642	2.3579234	2.34470901	2.59766727	162	214	244
9686	NM_001128219,NM_001128220	VGLL4	vestigial like family member 4	1.010832238	1.224401814	5.128161	5.211539	6.043448	4.33483599	4.34961533	4.61378826	753	1017	1134
51442	NM_016267	VGLL1	vestigial like family member 1	-1.774173666	1.224270379	59.548744	33.597095	68.511215	6.15892439	5.34728212	6.44715375	2766	2083	4165
100302143	NR_031650	MIR1248	microRNA 1248	-1.01529584	1.224016733	66.283791	65.404274	76.258125	2.9730102	2.95391015	3.23070557	269	354	405
645212	NR_034170,NR_034171	EIF3J-AS1	EIF3J antisense RNA 1 (head to	-1.043546899	1.224007952	7.027485	7.259667	8.533474	3.64828273	3.59177559	3.91874377	453	580	682
1690	NM_001135058,NM_001347720	COCH	cochlin	-2.42563167	1.223727535	25.807122	10.553135	29.009199	6.14341284	4.89378089	6.43096978	2736	1507	4118
10189	NM_005782	ALYREF	Aly/REF export factor	1.3248583	1.223671472	70.872147	93.990059	81.490913	6.26115717	6.66238832	6.54894104	2972	5261	4473
5888	NM_001164269,NM_001164270	RAD51	RAD51 recombinase	1.04544603	1.223520497	5.792009	6.054069	6.687472	3.80372238	3.86335533	4.0758175	509	711	766
4010	NM_001174146,NM_001174147	LMX1B	LIM homeobox transcription facto	-2.277473342	1.223429653	0.441336	0.192973	0.508087	1.81624731	1.07428646	2.03057861	99	58	149
1846	NM_001394,NM_057158	DUSP4	dual specificity phosphatase 4	1.339516089	1.223423955	7.82441	10.437716	8.998294	5.45593034	5.86931011	5.74085701	1684	3014	2534
81631	NM_022818	MAP1LC3B	microtubule associated protein 1	1.295020394	1.22340982	39.939281	51.771725	45.909569	6.49138142	6.86070859	6.77936636	3493	6044	5256
5591	NM_001081640,NM_006904	PRKDC	protein kinase, DNA-activated, ca	-1.759383398	1.223263773	29.250865	16.650769	33.625225	8.5907267	7.77848791	8.88078104	15097	11465	22714
9060	NM_001015880,NM_004670	PAPSS2	3'-phosphoadenosine 5'-phospho	1.642841114	1.22315832	1.486786	2.455473	1.71614	2.72326229	3.35157866	2.973571	220	483	331
6491	NM_001048166,NM_001282936	STIL	SCL/TAL1 interrupting locus	-1.278655969	1.222960863	13.101138	10.258458	14.743812	6.02369529	5.67521634	6.31003667	2515	2628	3783
10147	NM_001017392,NM_001321697	SUGP2	SURP and G-patch domain conta	-1.100149111	1.222801854	29.755362	28.02337	34.052091	7.33373816	7.19693203	7.62230301	6294	7644	9466
27163	NM_001042402,NM_014435	NAAA	N-acylethanolamine acid amidas	1.188211382	1.222765851	7.107373	8.473497	8.16496	3.78518394	4.01734947	4.05619567	502	797	755
22847	NM_001136156,NM_014910	ZNF507	zinc finger protein 507	-1.133287196	1.22265843	4.014476	3.551046	4.634358	4.95561208	4.78126538	5.23716605	1179	1390	1773
11014	NM_001100603,NM_006854	KDELR2	KDEL endoplasmic reticulum pro	-1.108934562	1.222316835	76.431676	68.75433	87.409874	7.73036317	7.58192679	8.01874882	8298	9998	12475
55284	NM_001001481,NM_001271015	UBE2W	ubiquitin conjugating enzyme E2	1.03058923	1.222154069	4.544504	4.289141	4.789023	5.03956952	5.08173995	5.32102311	1252	1724	1882
54838	NM_001083913,NM_017787	WBP1L	WW domain binding protein 1 like	-1.237626363	1.222093753	8.160628	6.565061	9.371645	5.07270439	4.77525187	5.35427002	1282	1384	1927
998	NM_001039802,NM_001791,NM	CDC42	cell division cycle 42	1.111536716	1.222033174	85.971276	95.820262	98.152852	7.50757443	7.65933644	7.79542081	7105	10552	10679
1741	NM_001166278,NM_020730,NM	DLG3	discs large MAGUK scaffold prot	-1.066829696	1.221988685	14.689612	13.88021	16.425578	6.11718378	6.02523839	6.402645	2686	3364	4037
10979	NM_001134999,NM_001135000	FERMT2	fermitin family member 2	-1.956655329	1.221471118	3.235852	1.663986	3.743817	3.53555296	2.6812291	3.80150191	416	284	625
23258	NM_001243254,NM_001348748	DENND5A	DENN domain containing 5A	-1.186292056	1.221348996	7.197771	6.08356	8.39279	5.15751465	4.91854554	5.43866688	1362	1534	2046
3909	NM_000227,NM_001127717,NM	LAMA3	laminin subunit alpha 3	1.033377535	1.221322626	110.953027	119.8409	130.90908	9.3108473	9.35814156	9.59888113	24895	34373	37396
284370	NM_001199324,NM_001321317	ZNF615	zinc finger protein 615	-2.202321844	1.221303572	1.488901	0.63938	1.618856	2.72881625	1.82964782	2.9773697	221	134	332
84272	NM_032312	YIPF4	Yip1 domain family member 4	1.281178735	1.221298425	18.654631	23.936958	21.415993	5.1981206	5.54696516	5.47941817	1402	2400	2106
7090	NM_001105192,NM_001282979	TLE3	transducin like enhancer of split 3	1.480921144	1.220910482	7.645561	10.192678	7.874644	5.29770564	5.85227276	5.5790303	1505	2978	2260
2806	NM_001286220,NM_002080	GOT2	glutamic-oxaloacetic transaminas	1.013103086	1.220778421	68.121375	69.07637	78.154727	7.35880455	7.37747216	7.64502004	6405	8670	9617
6129	NM_000971	RPL7	ribosomal protein L7	1.163493949	1.22041836	829.053955	965.658691	950.804932	9.40220444	9.62036932	9.68919618	26525	41235	39815
378	NM_001660	ARF4	ADP ribosylation factor 4	-1.019012082	1.220399125	81.261253	79.831871	93.194534	7.11552582	7.08855191	7.40100416	5405	7087	8113
64645	NM_033055	MFSD14A	major facilitator superfamily dom	-1.234214998	1.220323619	15.858607	12.860827	18.186193	5.3829543	5.08741362	5.66398036	1599	1731	2400
10179	NM_001286045,NM_001286046	RBM7	RNA binding motif protein 7	-1.032925841	1.220125775	4.869427	4.703896	5.55812	4.22515306	4.18094712	4.49823858	695	899	1043
81572	NM_030815	PDRG1	p53 and DNA damage regulated	1.265989803	1.219549716	17.605801	22.307795	20.174564	4.61678723	4.94467948	4.8925416	924	1563	1386
6549	NM_003048	SLC9A2	solute carrier family 9 member A2	-1.275477358	1.219470299	1.079835	0.845367	1.23554	2.78853894	2.49375478	3.03682664	232	243	348
80313	NM_001143757,NM_001143758	LRRC27	leucine rich repeat containing 27	1.362873166	1.219356781	0.70928	1.109239	1.099019	1.94589385	2.2895231	2.16322937	112	204	168
647042	NM_001164465,NM_001322400	GOLGA6L10	golgin A6 family-like 10	-1.214347109	1.219303174	0.985523	0.814661	1.233364	1.7191395	1.52971203	1.92438744	90	99	135
23237	NM_015193	ARC	activity regulated cytoskeleton as	4.625861132	1.219152744	0.514003	2.383941	0.584648	1.30878272	2.97154911	1.4863075	58	359	87
27245	NM_001029882	AHDC1	AT-hook DNA binding motif conta	1.297645583	1.219125052	7.634776	9.920138	8.746465	5.59318572	5.96222966	5.87366563	1856	3218	2783
54331	NM_001243773,NM_001243774	GNG2	G protein subunit gamma 2	3.363091823	1.219103971	0.345474	1.175939	0.404866	1.21691895	2.4491251	1.38699077	52	234	78
56131	NM_018938	PCDHB4	protocadherin beta 4	-1.253578989	1.219042506	0.306897	0.247021	0.352698	1.11880553	0.95166931	1.28032816	46	49	69
3363	NM_000872,NM_019859,NM_01	HTR7	5-hydroxytryptamine receptor 7	-1.334925126	1.218962652	0.307269	0.228935	0.358131	1.01352966	0.81765451	1.16514558	40	40	60
284325	NM_198476	C19orf54	chromosome 19 open reading fra	-1.037522619	1.218813529	10.425868	10.063021	11.946644	4.85635215	4.80507185	5.13288607	1098	1414	1646
22839	NM_001042486,NM_014902,NM	DLGAP4	DLG associated protein 4	1.284879049	1.218800244	11.328991	15.294188	14.020551	5.38647251	5.74045883	5.66574708	1603	2752	2403
92714	NM_001317968,NM_152285	ARRDC1	arrestin domain containing 1	-1.258524393	1.218760357	80.256191	63.893912	91.767514	7.00743316	6.67858806	7.2908393	5012	5321	7513
101928820	NR_134633,NR_134634	LOC101928820	uncharacterized LOC101928820	1.175440748	1.218700686	0.263071	0.33728	0.308529	0.77668602	0.8787583	0.90287695	28	44	42
80213	NM_001307960,NM_001308026	TM2D3	TM2 domain containing 3	1.449708163	1.218559119	23.605269	34.154144	27.185828	5.00679911	5.5286127	5.28392771	1223	2369	1833
10426	NM_001286277,NM_001286278	TUBGCP3	tubulin gamma complex associat	-1.046450882	1.218542209	8.736017	8.319167	11.737403	4.98375716	4.92036316	5.26073633	1203	1536	1803
84253	NM_001286779,NM_032293,NR	GARNL3	GTPase activating Rap/RanGAP	2.205420054	1.218463078	0.151967	0.338575	0.173233	0.64188297	1.16293478	0.75138414	22	65	33
266971	NR_002319	PIPSL	PIP5K1A and PSMD4-like, pseuc	1.568669535	1.218352371	0.136028	0.215919	0.157835	0.59399636	0.84852988	0.69713258	20	42	30
79935	NM_024877	CNTD2	cyclin N-terminal domain contain	-1.112763486	1.218352371	0.285435	0.252745	0.322651	0.59399636	0.54351856	0.69713258	20	24	30
3832	NM_004523	KIF11	kinesin family member 11	-1.37212638	1.218338903	20.410803	14.888677	23.367607	6.67124425	6.22007467	6.95362794	3962	3858	5937
55870	NM_018489	ASH1L	ASH1 like histone lysine methyltr	1.504442305	1.218251875	4.814643	7.250488	5.511492	5.81239274	6.39301127	6.09261172	2167	4356	3247
26807	NR_002439	SNORD43	small nucleolar RNA, C/D box 43	2.527281081	1.218217215	7.524188	19.279203	8.675838	0.54446555	1.11294744	0.64076093	18	61	27

100287098	NR_109770	TONSL-AS1	TONSL antisense RNA 1	1.493932334	1.218217215	0.382591	0.58394	0.447864	0.54446555	0.75384785	0.64076093	18	36	27
54567	NM_019074	DLL4	delta like canonical Notch ligand	3.119890505	1.218048504	0.116741	0.382571	0.138417	0.49317359	1.18729361	0.58209675	16	67	24
400	NM_001177,NM_001301068	ARL1	ADP ribosylation factor like GTPa	-1.417480976	1.217870464	23.195906	16.566396	26.544395	6.21342436	5.71816702	6.49431269	2874	2709	4305
101928837	NR_120561,NR_120562	LOC101928837	uncharacterized LOC101928837	1.546153742	1.217831974	0.941689	1.585704	1.131747	0.43999062	0.63490936	0.52094563	14	29	21
7257	NM_005999	TSNAX	translin associated factor X	1.133446486	1.217704349	12.996435	14.741469	14.868413	5.10935964	5.28516046	5.3860537	1316	1993	1971
826	NM_001003962,NM_001302632	CAPNS1	calpain small subunit 1	-1.440590106	1.217621125	298.832104	201.143505	352.003772	8.69486655	8.16973555	8.97831127	16230	15053	24306
643911	NM_001308963,NR_034105,NR	CRNDE	colorectal neoplasia differentially	1.925204764	1.217543935	0.297454	0.627095	0.361313	0.38477185	0.669904	0.45708734	12	31	18
59335	NM_021619	PRDM12	PR/SET domain 12	-1.986550784	1.217543935	0.12525	0.060824	0.144298	0.38477185	0.20477631	0.45708734	12	8	18
10914	NM_001252006,NM_001252007	PAPOLA	poly(A) polymerase alpha	1.093083215	1.217253209	46.261156	50.487165	52.773786	7.65706606	7.78486215	7.93942273	7885	11516	11805
123688	NM_001013619,NM_001083612	HYKK	hydroxylysine kinase	-1.306352782	1.217142064	2.273787	1.985857	1.725348	2.46162969	2.15396266	2.69776636	177	181	265
109623476	NR_145793	SNORD160	small nucleolar RNA, C/D box 16	1.121353014	1.217141957	3.523416	3.788459	3.857765	0.3273552	0.36283659	0.39027104	10	15	15
1981	NM_001194946,NM_001194947	EIF4G1	eukaryotic translation initiation fa	1.374883457	1.216877393	129.763211	176.15472	148.986116	9.45802689	9.91677833	9.74084612	27573	50652	41268
79816	NM_001143986,NM_024760,NR	TLE6	transducin like enhancer of split 6	-1.874327569	1.216760494	3.23485	1.527115	3.591081	2.29192162	1.62195412	2.52166171	153	109	229
51104	NM_001025780,NM_016014	ABHD17B	abhydrolase domain containing 1	1.278418239	1.216759967	5.186631	6.767802	5.632862	3.99331833	4.32786827	4.26018095	586	1001	877
55773	NM_001199198,NM_018309	TBC1D23	TBC1 domain family member 23	1.277781867	1.216759856	9.8124	12.583142	11.238593	5.24732179	5.59270531	5.52360351	1452	2479	2173
23244	NM_001100399,NM_001100400	PDS5A	PDS5 cohesin associated factor	-1.190765201	1.216705469	29.010137	23.4643	32.773906	7.47531018	7.22496279	7.75684956	6947	7795	10396
100506392	NR_103743,NR_103744	SLC16A1-AS1	SLC16A1 antisense RNA 1	1.125140123	1.216605256	2.940299	3.497127	3.227786	2.23060596	2.36620557	2.45783582	145	218	217
81577	NM_001243650,NM_030819,NR	GFOD2	glucose-fructose oxidoreductase	1.261063705	1.216602301	9.312877	11.745616	11.00912	4.67692288	4.99987273	4.94973287	965	1626	1444
80818	NM_001077195,NM_030634	ZNF436	zinc finger protein 436	1.370243396	1.21657566	3.196779	4.570241	3.807959	3.90776647	4.33606188	4.17342809	550	1007	823
100996455	NR_073177	LOC100996455	uncharacterized LOC100996455	-1.067926637	1.216541769	0.077729	0.083116	0.10013	0.26755841	0.25173066	0.32020933	8	10	12
101060264	NR_126415,NR_126416,NR_12	FOXP4-AS1	FOXP4 antisense RNA 1	1.582629446	1.216541769	0.343712	0.660096	0.492316	0.26755841	0.40498891	0.32020933	8	17	12
3785	NM_004518,NM_172106,NM_17	KCNQ2	potassium voltage-gated channel	2.782781154	1.216541769	0.021016	0.062415	0.023921	0.26755841	0.65251278	0.32020933	8	30	12
4066	NM_005583	LYL1	LYL1, basic helix-loop-helix famil	2.413503705	1.216541769	0.102421	0.279695	0.133485	0.26755841	0.58077243	0.32020933	8	26	12
317772	NM_175065	HIST2H2AB	histone cluster 2 H2A family men	1.490310084	1.216541769	0.420333	0.693127	0.499176	0.26755841	0.38406669	0.32020933	8	16	12
5864	NM_002866	RAB3A	RAB3A, member RAS oncogene	1.151730765	1.216521037	2.440783	2.82127	2.804535	2.19894236	2.36086138	2.42483178	141	217	211
79939	NM_024881	SLC35E1	solute carrier family 35 member E	1.041200619	1.216430801	11.989086	12.494412	13.701435	5.91766008	5.97496603	6.19607391	2334	3247	3492
167153	NM_001114393,NM_001114394	PAPD4	poly(A) RNA polymerase D4, nor	1.05629481	1.216339573	13.761267	14.314585	15.632199	6.09531432	6.17320493	6.37411319	2645	3733	3957
5725	NM_002819,NM_031990,NM_03	PTBP1	polypyrimidine tract binding prote	1.171983547	1.216326181	135.47359	159.075318	155.363472	8.75249247	8.98095516	9.03442927	16893	26453	25272
100	NM_000022,NM_001322050,NM	ADA	adenosine deaminase	1.048584414	1.216294494	6.495669	6.824188	7.326833	3.41326233	3.47543441	3.67153991	379	531	567
389333	NM_001161546	PROB1	proline rich basic protein 1	1.24041631	1.215696109	0.886142	1.108636	1.018251	1.93632435	2.17238371	2.14982678	111	184	166
5770	NM_001278618,NM_002827	PTPN1	protein tyrosine phosphatase, no	1.080593499	1.215695472	19.272287	20.843232	22.046686	6.08452478	6.19476657	6.36254057	2625	3790	3925
84752	NM_033309	B3GNT9	UDP-GlcNAc:betaGal beta-1,3-N	1.264805079	1.215639656	7.045605	8.922519	8.054201	4.29045667	4.61389559	4.55907234	729	1232	1090
7018	NM_001063	TF	transferrin	5.042205991	1.215548794	0.056241	0.284194	0.059279	0.20517559	0.83317479	0.24657076	6	41	9
105369486	NR_135100	LOC105369486	uncharacterized LOC105369486	1.488162123	1.215548794	0.107958	0.174033	0.130312	0.20517559	0.29720487	0.24657076	6	12	9
4046	NM_001013253,NM_001013254	LSP1	lymphocyte-specific protein 1	-1.586572606	1.215548794	0.07754	0.046738	0.088448	0.20517559	0.13134883	0.24657076	6	5	9
79165	NM_024316	LENG1	leukocyte receptor cluster memb	-1.040872991	1.215318038	13.649421	13.14924	15.613182	3.7336304	3.68025029	3.99567625	483	620	722
23252	NM_015207	OTUD3	OTU deubiquitinase 3	1.503014897	1.215185159	4.854083	7.305566	5.542335	4.99070847	5.56334327	5.26385014	1209	2428	1807
84641	NM_032558	MFSD14B	major facilitator superfamily dom	1.103221381	1.215171819	43.23579	47.754177	49.373707	7.12766433	7.26842371	7.40700195	5451	8035	8147
114819	NR_023386	CROCCP3	ciliary rootlet coiled-coil, rootletin	1.380725022	1.214987134	1.317879	1.822434	1.507095	2.98234021	3.39667017	3.23070557	271	500	405
1198	NM_001130028,NM_003992	CLK3	CDC like kinase 3	-1.108116039	1.214919937	28.713072	25.760173	30.899429	6.03606811	5.89032771	6.31304598	2537	3059	3791
55716	NM_001300750,NM_001300751	LMBR1L	limb development membrane pro	1.021390446	1.214767951	10.608624	10.825016	11.962635	4.6331688	4.66248855	4.90356387	935	1276	1397
285605	NM_001308081,NM_173666	DTWD2	DTW domain containing 2	1.191642623	1.21466514	1.504231	1.793754	1.71919	3.25675288	3.48529134	3.51046477	336	535	502
7270	NM_001205296,NM_007344,NR	TTF1	transcription termination factor 1	1.29488093	1.214619406	8.893184	12.52077	10.53542	4.8216876	5.18287427	5.09317527	1071	1853	1600
11092	NM_001316897,NM_001316898	SPACA9	sperm acrosome associated 9	1.292067413	1.214500338	3.219097	4.125192	3.245215	2.85607368	3.1801226	3.10091148	245	423	366
284406	NM_001321917,NM_001321918	ZFP82	ZFP82 zinc finger protein	-1.380993676	1.214500338	1.315975	0.986945	1.678551	2.85607368	2.46415559	3.10091148	245	237	366
259266	NM_001206846,NM_018136	ASPM	abnormal spindle microtubule as	-1.191651564	1.214457394	22.558168	19.430869	26.966894	7.79184836	7.54012965	8.07101344	8661	9711	12937
575	NM_001702	ADGRB1	adhesion G protein-coupled rece	-1.319271463	1.214260967	0.380053	0.290565	0.434917	1.61502061	1.3579479	1.80975447	81	82	121
256051	NM_001199295,NM_153263	ZNF549	zinc finger protein 549	1.318045326	1.214086352	6.138539	8.097523	7.005839	4.67979317	5.06458384	4.94973287	967	1703	1444
399474	NM_001003682,NM_001171868	TMEM200B	transmembrane protein 200B	-1.773073359	1.213901917	6.772081	3.735005	7.562805	4.13181574	3.36765473	4.39693481	649	489	969
387921	NM_001012754,NM_001017370	NHLRC3	NHL repeat containing 3	1.152942499	1.213865138	5.429402	6.146457	6.136014	4.26583542	4.46120049	4.53220782	716	1103	1069
79919	NM_001085437,NM_001282921	C2orf54	chromosome 2 open reading fran	-1.405316918	1.213816882	7.368849	5.240128	8.691084	4.25432848	3.79367035	4.52053953	710	675	1060
23304	NM_001184801,NM_015255	UBR2	ubiquitin protein ligase E3 compo	-1.038887283	1.213716673	5.699161	4.690607	5.601394	5.25600252	5.20242678	5.52879006	1461	1879	2181
114294	NM_001288585,NM_032857,NM	LACTB	lactamase beta	1.15358253	1.213683981	6.569251	7.668877	7.563505	3.92238208	4.11581404	4.18497252	556	857	830
93587	NM_001134665,NM_001134666	TRMT10A	tRNA methyltransferase 10A	1.093001559	1.213640911	2.03932	2.225223	2.317987	3.01452812	3.12759409	3.26218696	278	406	415
9823	NM_001282231,NM_014782,NM	ARMCX2	armadillo repeat containing, X-lin	7.315384361	1.213589917	0.036602	0.273023	0.041948	0.13997292	0.81765451	0.16897038	4	40	6
390205	NM_001145077	LRRC10B	leucine rich repeat containing 10	-1.74594355	1.213589917	0.046925	0.017667	0.051906	0.13997292	0.08023518	0.16897038	4	3	6
51655	NM_001199989,NM_016084	RASD1	ras related dexamethasone induc	3.124020912	1.213589917	0.059934	0.183377	0.068688	0.13997292	0.40498891	0.16897038	4	17	6
5817	NM_001135768,NM_001135769	PVR	poliovirus receptor	-1.002618905	1.213587395	28.97957	29.089147	36.053662	7.30776474	7.30401518	7.58544384	6181	8237	9226
10299	NM_001270660,NM_001270661	MARCH6	membrane associated ring-CH-ty	1.04269925	1.213539746	12.769016	13.358147	14.538341	6.92092074	6.98075752	7.19805152	4718	6573	7042
51097	NM_016002	SCCPDH	saccharopine dehydrogenase (pu	-1.079515431	1.213505065	33.301399	30.884579	37.976585	6.34446389	6.23548722	6.62052589	3151	3900	4703
1399	NM_005207	CRKL	CRK like proto-oncogene, adapto	1.408901695	1.213474171	41.257866	58.190521	47.047691	7.7475644	8.24019102	8.02552972	8398	15809	12534
5989	NM_002918	RFX1	regulatory factor X1	1.160244192	1.213259069	4.632365	5.385405	5.287955	4.37612225	4.5809496	4.64278115	776	1203	1158
7096	NM_003263	TLR1	toll like receptor 1	-1.906237583	1.213127344	4.657055	2.446829	5.308229	3.80108857	2.96099164	4.0615736	508	356	758
7185	NM_001190945,NM_001190947	TRAF1	TNF receptor associated factor 1	1.159336157	1.212994131	4.008384	4.685185	4.576398	4.20138033	4.40389042	4.46615621	683	1058	1019
285527	NM_015030	FRYL	FRY like transcription coactivator	-1.029786594	1.212969745	9.908566	9.631843	11.294065	6.83211648	6.79014717	7.10844153	4434	5753	6615
143884	NM_152434	CWF19L2	CWF19 like 2, cell cycle control (	-1.22189342	1.21287288	4.862656	3.981802	5.546642	4.04765842	3.77771682	4.3107378	610	667	910
79803	NM_024747	HPS6	HPS6, biogenesis of lysosomal o	-1.601408694	1.212868533	5.371712	3.358479	6.123384	3.90531607	3.2825973	4.16678956	549	458	819
27244	NM_001199933,NM_001199934	SESN1	sestrin 1	-1.181084537	1.212781647	1.496575	1.26559	1.805308	2.50111114	2.30630165	2.73414401	183	207	273
57530	NM_020770	CGN	cingulin	-1.530116669	1.21268351	19.980839	13.071893	22.7684	6.64724883	6.04119638	6.92293263	3896	3402	5811
22929	NM_001195602,NM_001195604	SEPHS1	selenophosphate synthetase 1	1.144783725	1.212511682	22.690985	26.051021	25.84184	6.18794463	6.38052162	6.46248074	2823	4318	4210
4087	NM_001003652,NM_001135937	SMAD2	SMAD family member 2	-1.150600292	1.212130862	3.827132	3.331901	4.376151	5.32271189	5.12572903	5.59395302	1532	1779	2284
25806	NM_012476	VAX2	ventral anterior homeobox 2	1.653207676	1.212027302	1.296469	2.139064	1.482205	1.29387258	1.76638114	1.46482043	57	126	85
9929	NM_014876	JOSD1	Josephin domain containing 1	1.411820984	1.211830474	36.396549	51.439255	41.447926	6.93930211	7.43343558	7.21443955	4779	9015	7123
9246	NM_004223,NM_198183	UBE2L6	ubiquitin conjugating enzyme E2	1.316835783	1.211643447	56.408062	73.535356	63.608739	6.13454836	6.52668807	6.40793246	2719	4784	4052
9965	NM_005117	FGF19	fibroblast growth factor 19	1.074652345	1.211401487	4.289326	4.613946	4.889988	3.31329794	3.40707821	3.56449618	351	504	523
26058	NM_001103146,NM_001103147	GIGYF2	GRB10 interacting GYF protein 2	1.202333307	1.211378828	10.861449	13.293842	10.946235	6.16610643	6.42856768	6.43925662	2780	4466	4142
55651	NM_001034833,NM_017838	NHP2	NHP2 ribonucleoprotein	1.077469978	1.211298386	71.243757	76.891993	80.93428	5.90361028	6.00952888	6.17596655	2311	3327	3443
5217	NM_002628,NM_053024	PFN2	profilin 2	1.112613164	1.210994313	104.444622	112.726044	119.935756	7.5552432	7.70842082	7.83010205	7345	10919	10940
57486	NM_020726	NLN	neurolysin	1.018237296	1.21094264	5.410224	5.515878	6.159199	5.54362658	5.56914783	5.8143746	1792	2438	2669
4173	NM_005914,NM_182746	MCM4	minichromosome maintenance co	1.316321011	1.210918326	37.092698	48.906532	42.231068	7.44694859	7.84147706	7.72161336	6811	11979	10144
65249	NM_023072	ZSWIM4	zinc finger SWIM-type containing	-1.11190421	1.210814155	5.886633	5.299279	6.701174	4.6940596	4.54723362	4.96032831	977	1174	1455
2954	NM_001312660,NM_145870,NM	GSTZ1	glutathione S-transferase zeta 1	1.098483019	1.210802157	17.410131	19.235113	18.980784	4.55403551	4.68404654	4.8192972	883	1296	1315
200933	NM_001105573	FBXO45	F-box protein 45	-1.093661022	1.210549964	7.612474	6.971516	8.661469	5.44331753	5.3172456	5.71321819	1669	2039	2485
441381	NM_001024678	LRRC24	leucine rich repeat containing 24	-1.298410578	1.210350436	4.581145	3.532086	5.216698	3.15728865	2.8278811	3.40441396	311	320	463
126272	NM_152361	EID2B	EP300 interacting inhibitor of diff	1.512890918	1.210280514	1.75301	2.669725	1.999508	1.7191395	2.16012918	1.91649468	90	182	134
101669762	NR_103783	BLACAT1	bladder cancer associated transc	-1.558133839	1.209796487	12.909851	8.292599	14.673865	5.08249824	4.46618624	5.34988112	1291	1107	1921
10423	NM_001286585,NM_001286586	CDIPT	CDP-diacylglycerol--inositol 3-ph	1.115096645	1.209539886	18.367226	20.575617	20.985717	5.13363806	5.2865704	5.40097983	1339	1995	1992
151011	NM_001321496,NM_001321498	SEPT10	septin 10	1.111627024	1.209531979	8.823309	9.707905	9.765004	5.04514512	5.19343546	5.31202633	1257	1867	1870
3459	NM_000416	IFNGR1	interferon gamma receptor 1	1.48276367	1.209488631	14.439649	21.450552	16.417505	4.94610703	5.49911925	5.21239866	1171	2320	1742
11332	NM_007274,NM_181864,NM_18	ACOT7	acyl-CoA thioesterase 7	-1.190230359	1.209477982	38.828027	32.327672	46.590141	6.08127212	5.8340679	6.3519721	2619	2940	3896
27343	NM_001174084,NM_001174085	POLL	DNA polymerase lambda	-1.247580706	1.209316599	8.865428	7.084012	10.517055	4.53988763	4.2359869	4.80333552	874	936	1300
22919	NM_012325	MAPRE1	microtubule associated protein R	1.312755894	1.209193143	60.932602	80.084641	69.239548	7.29120266	7.6816112	7.56365752	6110	10717	9087
23203	NM_001282944,NM_001282946	PMPCA	peptidase, mitochondrial process	1.243582667	1.209126942	40.423677	50.300645	45.851437	6.43090861	6.74214096	6.70198758	3348	5563	4979
10333	NM_006068	TLR6	toll like receptor 6	-1.799883523	1.209046142	5.324039	2.962668	6.052231	4.97910428	4.16723545	5.24506567	1199	890	1783
80346	NM_001316964,NM_001316965	REEP4	receptor accessory protein 4	1.176868031	1.208948488	29.904634	35.611669	34.023065	5.66519587	5.89588107	5.9340406	1953	3071	2904
155060	NR_036573	LOC155060	AI894139 pseudogene	-1.002091095	1.208518978	2.761046	2.756488	3.136339	3.24517298	3.24247658	3.49199507	333	444	495
55831	NM_018447	EMC3	ER membrane protein complex s	1.1585731	1.208407368	14.427576	16.743082	16.394287	5.25311474	5.46029098	5.51970132	1458	2257	2167
101927365	NR_125843	LOC101927365	uncharacterized LOC101927365	-1.086381354	1.208273643	1.902073	1.761862	2.169212	1.5285023	1.45142181	1.71282633	74	91	110
10605	NM_006451,NM_182789,NM_18	PAIP1	poly(A) binding protein interacting	1.114815516	1.208141787	16.552884	19.470753	19.561282	5.44669174	5.60009515	5.71378758	1673	2492	2486

26019	NM_015542,NM_080599	UPF2	UPF2 regulator of nonsense tran	1.13529798	1.208079652	10.785643	12.311971	12.281682	5.80715195	5.98715744	6.07543546	2159	3275	3208
677768	NR_003002	SCARNA13	small Cajal body-specific RNA 13	1.116422214	1.207921239	0.180394	0.146266	0.196358	0.07168335	0.08023518	0.08695753	2	3	3
152015	NM_001012337,NM_001308313	ROPN1B	rhophilin associated tail protein 1	5.378951729	1.207921239	0.044345	0.25501	0.037461	0.07168335	0.36283659	0.08695753	2	15	3
406972	NR_029582	MIR196A1	microRNA 196a-1	1.8268438	1.207921239	0.732664	1.352983	0.723762	0.07168335	0.13134883	0.08695753	2	5	3
100616150	NR_039871	MIR4720	microRNA 4720	1.471633007	1.207921239	0.541902	0.957609	0.742139	0.07168335	0.10601836	0.08695753	2	4	3
4629	NM_001040113,NM_001040114	MYH11	myosin heavy chain 11	1.471633007	1.207921239	0.007537	0.011266	0.008609	0.07168335	0.10601836	0.08695753	2	4	3
619570	NR_002591	SNORD95	small nucleolar RNA, C/D box 95	2.182054593	1.207921239	0.818182	1.734357	0.722505	0.07168335	0.15624221	0.08695753	2	6	3
102465446	NR_106804	MIR6746	microRNA 6746	-1.313695475	1.207921239	0.68456	0.522155	0.744495	0.07168335	0.05398282	0.08695753	2	2	3
151651	NM_001330688,NM_144715	EFHB	EF-hand domain family member	12.48316759	1.207921239	0.017649	0.237105	0.020349	0.07168335	0.73744528	0.08695753	2	35	3
100287238	NM_001256855	USP17L13	ubiquitin specific peptidase 17-lik	1.116422214	1.207921239	0.018195	0.032714	0.032326	0.07168335	0.08023518	0.08695753	2	3	3
100302233	NR_031672_chr15,NR_031672_	MIR1268A	microRNA 1268a	-2.463050374	1.207921239	0.697138	0.216008	0.978624	0.07168335	0.02724389	0.08695753	2	1	3
56623	NM_001318502,NM_019892	INPP5E	inositol polyphosphate-5-phospha	-1.193318244	1.207914585	15.306912	12.846087	17.384777	5.69103253	5.44142749	5.95874997	1989	2227	2955
9731	NM_014704	CEP104	centrosomal protein 104	1.057060216	1.207908033	3.535489	3.740805	4.013968	4.53830704	4.61501835	4.80012188	873	1233	1297
1994	NM_001419	ELAVL1	ELAV like RNA binding protein 1	1.130184137	1.20772468	18.227293	20.623734	20.685143	6.76173135	6.93676112	7.03174097	4221	6374	6270
10241	NM_001261390,NM_001261391	CALCOCO2	calcium binding and coiled-coil d	-1.305144933	1.207718426	35.370053	27.097055	40.100626	6.99625111	6.61547585	7.26659592	4973	5091	7387
55829	NM_018445,NM_203472	SELENOS	selenoprotein S	1.488326437	1.207671882	37.613366	56.007788	43.013528	5.58555344	6.14938014	5.85262116	1846	3671	2742
8945	NM_001256856,NM_003939,NM	BTRC	beta-transducin repeat containing	-1.224435462	1.207585499	2.883832	2.396102	3.310825	4.19537543	3.92077761	4.45393896	680	742	1010
10522	NM_001293634,NM_021008	DEAF1	DEAF1, transcription factor	1.315272989	1.207407102	4.809777	6.390211	5.460297	3.78784692	4.1580214	4.04175574	503	884	747
64285	NM_022450	RHBDF1	rhomboid 5 homolog 1	-1.292238583	1.207331355	40.940449	31.71797	46.454632	6.91026734	6.54388631	7.18003369	4683	4842	6954
7054	NM_000360,NM_199292,NM_19	TH	tyrosine hydroxylase	4.437329134	1.207297334	0.472091	2.132509	0.536414	0.91952222	2.31185141	1.05442063	35	208	52
81031	NM_030777	SLC2A10	solute carrier family 2 member 1(	-1.036504932	1.207283795	11.857727	11.453476	13.454501	5.6410988	5.59042388	5.90790578	1920	2475	2851
6777	NM_012448	STAT5B	signal transducer and activator o	1.021576372	1.207184303	10.286634	10.518077	11.66893	5.70095648	5.73116925	5.96784829	2003	2734	2974
51144	NM_016142	HSD17B12	hydroxysteroid 17-beta dehydrog	-1.054674127	1.207162591	61.280762	58.172249	69.520493	7.25605529	7.1797725	7.52605933	5962	7553	8852
440503	NM_001013706	PLIN5	perilipin 5	1.047777241	1.207116674	1.76278	1.847961	2.006108	2.37926962	2.43393638	2.60259472	165	231	245
84864	NM_001042533,NM_001261829	RIOX2	ribosomal oxygenase 2	2.89514055	1.207069609	5.429954	15.758828	6.118113	4.78350351	6.2825285	5.04602381	1042	4031	1547
5264	NM_001037537,NM_001323080	PHYH	phytanoyl-CoA 2-hydroxylase	-1.035379652	1.206915652	15.128106	14.763628	17.25216	4.63020414	4.58209828	4.89153539	933	1204	1385
372	NM_001142281,NM_001655	ARCN1	archain 1	-1.731626877	1.206900941	38.732247	22.13892	43.729244	7.23401145	6.44885883	7.50367958	5871	4530	8715
445328	NM_001003702	ARHGEF35	Rho guanine nucleotide exchang	-1.284056793	1.206875884	6.778581	5.287687	7.693641	4.11706758	3.77972069	4.37406362	642	668	953
63951	NM_022160	DMRTA1	DMRT like family A1	1.143475281	1.206807332	1.820697	2.087618	2.069047	2.35073722	2.50832973	2.57277436	161	246	239
10725	NM_001113178,NM_006599,NM	NFAT5	nuclear factor of activated T-cells	1.253001231	1.2067983	14.924433	18.931172	17.054397	7.58712508	7.91100132	7.85702706	7510	12573	11147
1659	NM_001302623,NM_001322216	DHX8	DEAH-box helicase 8	-1.015836026	1.206777196	17.228409	17.950529	20.061263	6.35662242	6.33423288	6.62477064	3178	4180	4717
29082	NM_014169	CHMP4A	charged multivesicular body prot	1.15760746	1.206419513	22.081484	25.593485	25.043831	4.91477587	5.11941338	5.17732438	1145	1771	1699
285704	NM_001012761	RGMB	repulsive guidance molecule fam	-1.100399726	1.206340109	4.745334	4.314073	5.378109	4.47030848	4.33878278	4.72980159	831	1009	1233
3656	NM_001570	IRAK2	interleukin 1 receptor associated	1.071107646	1.206209752	4.595055	4.928364	5.212356	4.04765842	4.14097459	4.30319063	610	873	905
22983	NM_014975	MAST1	microtubule associated serine/thr	2.001169316	1.205833205	0.633324	1.2758	0.721646	2.02024721	2.83174937	2.22843599	120	321	178
28981	NM_001143779,NM_001347946	IFT81	intraflagellar transport 81	1.303970865	1.205823847	5.621383	7.671097	6.715169	4.13809052	4.50182664	4.39409566	652	1136	967
100130522	NR_028339,NR_028340	PARD6G-AS1	PARD6G antisense RNA 1	2.024479325	1.205718653	0.238437	0.492353	0.273496	0.83964067	1.37924953	0.96541723	31	84	46
283385	NM_173855	MORN3	MORN repeat containing 3	1.08613974	1.205718653	0.686253	0.74823	0.778113	0.83964067	0.89363826	0.96541723	31	45	46
10524	NM_001206833,NM_006388,NM	KAT5	lysine acetyltransferase 5	1.094350851	1.205710172	22.403744	24.268147	25.226686	5.60983593	5.73736895	5.87468438	1878	2746	2785
163059	NM_001080411,NM_001308346	ZNF433	zinc finger protein 433	1.225646804	1.205682814	2.944321	3.568461	3.318232	2.9541672	3.21312006	3.19199808	265	434	393
8427	NM_001303481,NM_003575	ZNF282	zinc finger protein 282	-1.342501671	1.205629225	8.830167	6.587263	10.151565	5.04291747	4.63286465	5.30524173	1255	1249	1861
115950	NM_138783	ZNF653	zinc finger protein 653	1.128061171	1.205582568	2.38957	2.700248	2.711766	2.63728034	2.78463118	2.86703245	205	309	304
56255	NM_021156	TMX4	thioredoxin related transmembra	1.228697832	1.205484232	4.533315	5.579953	5.136729	4.81908641	5.10669852	5.07998613	1069	1755	1585
56951	NM_020199	C5orf15	chromosome 5 open reading fran	1.258388653	1.205387069	52.264751	65.841148	59.199898	6.87125028	7.20030218	7.13865149	4557	7662	6756
3029	NM_001040427,NM_001286249	HAGH	hydroxyacylglutathione hydrolase	-1.19212674	1.205252125	25.133057	20.937766	27.416782	5.24441656	4.99815263	5.50727429	1449	1624	2148
283578	NM_001346131,NM_001346133	TMED8	transmembrane p24 trafficking pr	1.213268597	1.205172607	4.813184	5.868035	5.475416	5.23664058	5.50881622	5.49937013	1441	2336	2136
387338	NM_001256127,NM_001256128	NSUN4	NOP2/Sun RNA methyltransferas	1.098523737	1.205096568	4.642613	5.07661	5.230465	4.58038655	4.71054878	4.83926507	900	1321	1334
5062	NM_002577	PAK2	p21 (RAC1) activated kinase 2	1.21665652	1.205096168	26.326982	32.065678	29.814125	7.30706876	7.58837191	7.57467017	6178	10043	9157
26271	NM_001142522,NM_012177	FBXO5	F-box protein 5	1.179058376	1.20464841	15.014911	17.846667	17.114378	5.01136367	5.24220835	5.27237862	1227	1933	1818
10097	NM_001005386,NM_005722	ACTR2	ARP2 actin related protein 2 hon	1.181121311	1.204601744	71.710931	84.801082	81.18055	8.10258125	8.3419355	8.37024828	10752	16968	15930
5051	NM_000437	PAFAH2	platelet activating factor acetylhy	-1.188033716	1.204530907	4.748336	3.998653	5.374262	4.13391036	3.90066256	4.38840055	650	731	963
7428	NM_000551,NM_198156	VHL	von Hippel-Lindau tumor suppres	1.197764404	1.204467529	9.127096	10.932984	10.321277	5.37412105	5.62872593	5.6366162	1589	2543	2354
22853	NM_014916	LMTK2	lemur tyrosine kinase 2	-1.446036802	1.204429201	7.537007	5.219512	8.53188	6.05884715	5.53633734	6.3235294	2578	2382	3819
102724428	NM_001320643	LOC102724428	serine/threonine-protein kinase S	-1.087248124	1.204196111	10.512437	9.680408	11.896945	5.64917596	5.53099392	5.91237727	1931	2373	2860
56905	NM_015492	C15orf39	chromosome 15 open reading fra	1.279909019	1.204193808	10.784482	13.817693	12.202767	5.56939302	5.9187927	5.83231428	1825	3121	2703
203414	NR_126059	LINC01560	long intergenic non-protein codin	1.108576603	1.204096127	0.556197	0.620392	0.63066	1.24819466	1.33632702	1.40965954	54	80	80
90268	NM_138348	OTULIN	OTU deubiquitinase with linear li	-1.210065854	1.20408385	1.968017	1.626329	2.224878	4.03650512	3.77972069	4.28950542	605	668	896
161882	NM_153813	ZFPM1	zinc finger protein, FOG family m	1.22197679	1.203993933	1.273549	1.556795	1.438969	2.32896139	2.56520021	2.54744438	158	258	234
23130	NM_015104	ATG2A	autophagy related 2A	1.552525041	1.203786494	5.18413	8.057291	5.862702	5.05180751	5.67090409	5.31202633	1263	2620	1870
203260	NM_001195200,NM_001195201	CCDC107	coiled-coil domain containing 107	-1.163382357	1.203694318	5.938677	5.153764	6.799774	3.0370876	2.84711955	3.27458965	283	325	419
1583	NM_000781,NM_001099773	CYP11A1	cytochrome P450 family 11 subfa	1.08075192	1.20367415	0.350146	0.377883	0.389289	0.7550755	0.80196545	0.87055986	27	39	40
222962	NM_001040661,NM_001300847	SLC29A4	solute carrier family 29 member 4	-1.891987515	1.20367415	0.24173	0.128824	0.271017	0.7550755	0.44594451	0.87055986	27	19	40
54386	NM_018975,NR_144545	TERF2IP	TERF2 interacting protein	-1.093605374	1.203365636	25.342785	23.042644	28.911917	5.78998672	5.66332648	6.05266115	2133	2606	3157
10166	NM_014252	SLC25A15	solute carrier family 25 member 1	1.087858474	1.203336854	2.939713	3.202231	3.323982	3.64534899	3.75752359	3.89283072	452	657	669
79400	NM_001184779,NM_001184780	NOX5	NADPH oxidase 5	4.048104842	1.203204286	4.287677	17.398853	4.847968	3.7744826	5.71030906	4.02350017	498	2694	737
79090	NM_001270891,NM_001270892	TRAPPC6A	trafficking protein particle comple	-1.202448533	1.203184601	14.256997	11.841265	16.063657	3.55756677	3.3161202	3.80364308	423	470	626
23326	NM_015276	USP22	ubiquitin specific peptidase 22	1.249367167	1.203121863	20.804026	26.02656	23.524065	6.73817389	7.05667894	7.00267984	4152	6931	6144
27348	NM_001317893,NM_001317894	TOR1B	torsin family 1 member B	1.22635195	1.203092196	16.99523	21.892367	20.329732	5.58937463	5.87822158	5.8510692	1851	3033	2739
254359	NM_001348571,NM_207340	ZDHHC24	zinc finger DHHC-type containing	1.118481963	1.202993135	6.16273	6.721875	7.096027	4.83333524	4.98952124	5.09142367	1080	1614	1598
8189	NM_004819	SYMPK	symplekin	-1.074560003	1.202923291	35.078186	32.679615	39.654179	7.17038321	7.06738169	7.43524216	5616	6983	8309
24148	NM_012469	PRPF6	pre-mRNA processing factor 6	1.160070211	1.202646165	45.37952	52.709698	51.293003	7.10888572	7.3216589	7.37334081	5380	8339	7958
388327	NM_001105520	C17orf100	chromosome 17 open reading fra	-1.036318028	1.202590075	2.018847	1.956938	2.283825	2.02927762	1.99055518	2.23479744	121	156	179
9901	NM_001033117,NM_014850	SRGAP3	SLIT-ROBO Rho GTPase activat	-1.364181185	1.202480154	0.418719	0.306847	0.473101	2.22275497	1.88281796	2.43591723	144	141	213
130617	NM_001270998,NM_001270999	ZFAND2B	zinc finger AN1-type containing 2	1.328447288	1.202432765	14.090509	19.006259	16.407287	4.21331566	4.60375125	4.46615621	689	1223	1019
5571	NM_001206709,NM_001206710	PRKAG1	protein kinase AMP-activated no	-1.067595988	1.202274132	24.888643	23.312688	28.127935	5.42376114	5.33165924	5.6838776	1646	2060	2434
54020	NM_001320537,NM_018964	SLC37A1	solute carrier family 37 member 1	-1.100500359	1.202262322	10.566407	9.607499	11.929361	5.29677111	5.1622853	5.55635276	1504	1826	2224
63035	NM_001184772,NM_021946	BCORL1	BCL6 corepressor like 1	1.055476022	1.202171185	2.515176	2.640194	2.82156	4.23884171	4.31272471	4.49161333	702	990	1038
80012	NM_001308116,NM_024947	PHC3	polyhomeotic homolog 3	1.158679191	1.202085852	8.443011	9.796892	9.537665	6.71737173	6.92797922	6.98061156	4092	6335	6050
57828	NM_001330496,NM_021185	CATSPERG	cation channel sperm associated	1.485595651	1.202080133	0.495897	0.756851	0.562529	1.48977343	1.88281796	1.66651905	71	141	105
84886	NM_001136494,NM_001136495	C1orf198	chromosome 1 open reading fran	1.036351698	1.202029299	6.451252	6.694132	7.288967	4.62276571	4.67222953	4.87839036	928	1285	1372
23526	NM_001258328,NM_001282334	ARHGAP45	Rho GTPase activating protein 4	-1.005276694	1.201949888	12.001077	11.914483	13.551535	5.9841867	5.97671398	6.24574008	2446	3251	3616
55159	NM_018124	RFWD3	ring finger and WD repeat domai	1.08304664	1.201608353	28.452696	30.847778	32.129742	7.11048214	7.22477893	7.37370116	5386	7794	7960
123355	NM_001284400,NM_001321675	LRRC28	leucine rich repeat containing 28	1.364823575	1.201536904	0.700402	0.956952	0.790884	2.33625658	2.70672986	2.55254608	159	290	235
6747	NM_001308197,NM_001308204	SSR3	signal sequence receptor subuni	1.062858869	1.201414424	18.257203	19.432487	20.669585	6.28538667	6.37224539	6.54702524	3023	4293	4467
51451	NM_001032391,NM_016309	LCMT1	leucine carboxyl methyltransfera	1.278895844	1.201375886	15.097086	19.289306	17.213611	4.38845666	4.72829618	4.64158467	783	1338	1157
60626	NM_001286134,NM_021932	RIC8A	RIC8 guanine nucleotide exchan	1.130454777	1.201296492	17.296905	19.557557	19.519099	6.01064561	6.18497139	6.27149421	2492	3764	3682
9445	NM_021999	ITM2B	integral membrane protein 2B	1.157308357	1.20128564	104.273781	120.818214	117.719955	7.59626904	7.80603138	7.85960209	7558	11687	11167
9474	NM_001286106,NM_001286107	ATG5	autophagy related 5	-1.179729638	1.201153272	7.539778	6.514774	8.63704	4.62276571	4.39476729	4.87737422	928	1051	1371
339327	NM_001297763,NM_178544	ZNF546	zinc finger protein 546	-1.091550094	1.201037327	0.216718	0.19959	0.24492	1.43646263	1.3579479	1.60891398	67	82	99
142891	NM_001174156,NM_144660	SAMD8	sterile alpha motif domain contai	-1.409910168	1.200851065	6.68958	4.739425	7.531745	5.5004447	5.01781102	5.75917693	1738	1647	2567
27291	NM_001256619,NM_001256620	R3HCC1L	R3H domain and coiled-coil cont	-1.336060911	1.200832836	5.977953	5.18	6.667574	4.38670105	3.99164624	4.63918874	782	782	1155
79004	NM_024040	CUEDC2	CUE domain containing 2	-1.162694234	1.200672974	26.831247	23.093353	30.26993	5.01136367	4.80113128	5.26773297	1227	1410	1812
23333	NM_015283	DPY19L1	dpy-19 like 1	-1.206936209	1.200545953	5.975259	4.956708	6.742398	4.87400371	4.61277196	5.12947602	1112	1231	1642
4790	NM_001165412,NM_001319226	NFKB1	nuclear factor kappa B subunit 1	1.701592422	1.200323509	5.684774	9.684866	6.413214	4.5618359	5.30338313	4.81505799	888	2019	1311
1951	NM_001407	CELSR3	cadherin EGF LAG seven-pass G	-2.598608038	1.20013211	1.556031	0.598483	1.75569	4.25817433	2.99588686	4.5087761	712	366	1051
685	NM_001316963,NM_001729	BTC	betacellulin	1.451265351	1.199927509	4.563166	6.642487	5.142182	3.71696001	4.21984192	3.96156336	477	925	704
2114	NM_001256295,NM_005239	ETS2	ETS proto-oncogene 2, transcrip	1.228661356	1.199905223	34.886034	42.922642	39.343987	7.01057147	7.30558068	7.27163199	5023	8246	7413
5625	NM_001195226,NM_016335	PRODH	proline dehydrogenase 1	2.610844353	1.199862337	0.680415	1.785432	0.768823	1.38110603	2.37683491	1.5489128	63	220	93
9545	NM_004283	RAB3D	RAB3D, member RAS oncogene	-1.447909215	1.199832085	4.037964	2.793178	4.553172	4.15469105	3.65645563	4.40400834	660	609	974

222389	NM_001100912,NM_152751	BEND7	BEN domain containing 7	-2.976401666	1.19973892	4.993212	1.660118	5.566021	3.38187998	2.05815489	3.62144026	370	166	546
23286	NM_001161661,NM_001161662	WWC1	WW and C2 domain containing 1	-1.235924882	1.199535924	20.424031	16.536951	23.019208	7.07521891	6.7721428	7.33591963	5255	5681	7753
9241	NM_005450	NOG	noggin	-1.530225545	1.199491639	3.372632	2.198222	3.792908	2.85098947	2.33928445	3.07986458	244	213	360
79001	NM_001311311,NM_024006,NM	VKORC1	vitamin K epoxide reductase com	1.360221431	1.199319327	28.581718	38.865518	32.179339	4.91477587	5.3459303	5.16904389	1145	2081	1689
9928	NM_001305792,NM_014875	KIF14	kinesin family member 14	-1.433437984	1.199297201	15.800765	10.91618	17.846766	6.77856477	6.26475434	7.03857485	4271	3981	6300
100130894	NR_034083	VAC14-AS1	VAC14 antisense RNA 1	-1.471295814	1.199235825	1.64509	1.121224	1.864109	2.19894236	1.78245981	2.40804223	141	128	208
1672	NM_005218	DEFB1	defensin beta 1	-1.596434952	1.199217162	3.40086	2.150339	3.881561	1.35261029	0.97983233	1.51794971	61	51	90
643246	NM_001085481	MAP1LC3B2	microtubule associated protein 1	-1.002081141	1.19915479	0.640863	0.637043	0.734765	0.61813834	0.61708849	0.71544405	21	28	31
22870	NM_014931	PPP6R1	protein phosphatase 6 regulatory	1.031178479	1.19907406	44.930012	46.382584	50.631786	7.32299179	7.36701414	7.58342016	6247	8607	9213
10472	NM_001278196,NM_006352,NM	ZBTB18	zinc finger and BTB domain cont	-1.36184982	1.199056586	9.972228	7.235736	11.242778	5.37146049	4.9384145	5.6275797	1586	1556	2339
55852	NM_001288732,NM_001288733	TEX2	testis expressed 2	1.142990415	1.198873923	5.842145	6.687898	6.585425	4.90867185	5.09548035	5.16238511	1140	1741	1681
6494	NM_006747,NM_153253	SIPA1	signal-induced proliferation-assoc	-9.197543926	1.19885155	8.58585	0.922015	9.898985	4.95442736	2.09080288	5.20836379	1178	171	1737
51635	NM_001322280,NM_001322281	DHRS7	dehydrogenase/reductase 7	-1.078391065	1.198832967	57.98585	52.549081	62.337744	6.7859097	6.67805099	7.04537652	4293	5319	6330
54821	NM_001009954,NM_017669	ERCC6L	ERCC excision repair 6 like, spin	1.085898848	1.198801459	7.838298	8.48924	8.750858	5.06284359	5.17832427	5.31728127	1273	1847	1877
9203	NM_001171162,NM_001171163	ZMYM3	zinc finger MYM-type containing	1.034049851	1.198751077	11.143588	12.181671	12.327771	5.88756138	5.93506664	6.14505901	2285	3157	3369
9429	NM_001257386,NM_001348985	ABCG2	ATP binding cassette subfamily G	1.889869785	1.198633729	1.492932	2.674826	1.574732	2.7991369	3.61665196	3.02586351	234	591	345
54480	NM_001284295,NM_019015	CHPF2	chondroitin polymerizing factor 2	1.566775267	1.198553565	8.628233	13.539907	10.063488	5.10935964	5.74200129	5.36373375	1316	2755	1940
23411	NM_001142498,NM_001314049	SIRT1	sirtuin 1	-1.110044194	1.198394443	7.442748	6.805748	8.352696	4.85254141	4.7073941	5.10537729	1095	1318	1614
375757	NM_001040011,NM_001318089	SWI5	SWI5 homologous recombination	-1.001840609	1.198372384	29.020676	29.226071	32.278639	4.72356761	4.72101485	4.97560227	998	1331	1471
7850	NM_001261419,NM_004633,NR	IL1R2	interleukin 1 receptor type 2	-1.942939997	1.198345246	6.915211	3.574862	7.912177	3.43040645	2.59281628	3.66919325	384	264	566
5495	NM_001033557,NM_002706,NM	PPM1B	protein phosphatase, Mg2+/Mn2+	-1.020925487	1.198340972	16.650247	17.497538	20.287613	5.69316485	5.66386906	5.94959391	1992	2607	2936
163033	NM_152600	ZNF579	zinc finger protein 579	-1.155173714	1.198334275	15.021753	13.016124	16.909988	5.03172731	4.83042646	5.28546063	1245	1440	1835
7511	NM_001167604,NM_001324128	XPNPEP1	X-prolyl aminopeptidase 1	-1.011785698	1.198313886	14.682478	14.577892	16.755601	5.23078096	5.2143287	5.48543279	1435	1895	2115
599	NM_001199839,NM_004050	BCL2L2	BCL2 like 2	1.319532132	1.198307103	18.306345	24.39362	20.850176	6.02876973	6.42344998	6.28611642	2524	4450	3720
28511	NM_001001349,NM_001144927	NKIRAS2	NFKB inhibitor interacting Ras lik	1.326614699	1.198293196	14.143647	18.672871	15.87082	5.11254966	5.51002377	5.36663322	1319	2338	1944
9066	NM_001252065,NM_001300773	SYT7	synaptotagmin 7	-1.19690105	1.197930168	2.531824	2.125803	2.867775	4.02977152	3.78770843	4.27568916	602	672	887
2958	NM_001320929,NM_001320930	GTF2A2	general transcription factor IIA su	1.698331575	1.197871624	19.304799	33.053968	21.921096	4.95797859	5.70293615	5.21078606	1181	2680	1740
8087	NM_001013438,NM_001013439	FXR1	FMR1 autosomal homolog 1	1.12118198	1.197807374	26.572649	29.69163	29.650678	7.79267723	7.95699619	8.05200133	8666	12982	12767
6776	NM_001288718,NM_001288719	STAT5A	signal transducer and activator of	-2.590688483	1.197756202	2.158119	0.813727	2.452444	3.32800968	2.16626946	3.56449618	355	183	523
63932	NM_001170569,NM_001170570	CXorf56	chromosome X open reading fran	1.036591318	1.197645078	8.557878	8.877817	9.620723	4.34933082	4.39868427	4.59783705	761	1054	1121
5432	NM_032940	POLR2C	RNA polymerase II subunit C	1.196394842	1.19760996	39.724781	47.585709	44.709702	6.1454907	6.40084412	6.40229181	2740	4380	4036
51274	NM_016531	KLF3	Kruppel like factor 3	-1.005754159	1.197361881	12.791007	12.732248	14.395503	6.14860191	6.14044089	6.40511489	2746	3648	4044
29761	NM_001283041,NM_001283042	USP25	ubiquitin specific peptidase 25	1.193529318	1.197290064	7.709885	9.214396	8.659181	5.30701783	5.55634684	5.56079029	1515	2416	2231
84875	NM_001317895,NM_032789,NR	PARP10	poly(ADP-ribose) polymerase fan	-1.20511301	1.197129437	21.96292	18.203165	24.658787	6.25202543	5.9867238	6.50849312	2953	3274	4348
406983	NR_029834	MIR200A	microRNA 200a	1.024038065	1.197017761	5.439473	5.520368	6.027264	0.56944351	0.58077243	0.65979727	19	26	28
57343	NM_001290318,NM_001290319	ZNF304	zinc finger protein 304	1.161369323	1.196948108	9.081788	10.48789	10.144875	5.33550944	5.54637678	5.58899591	1546	2399	2276
3622	NM_001291959,NM_001564	ING2	inhibitor of growth family member	1.089962813	1.196947798	5.40865	5.857206	6.047106	3.79050499	3.90617635	4.03265683	504	734	742
10671	NM_006571	DCTN6	dynactin subunit 6	-1.070382696	1.196893687	26.653212	24.938522	29.998917	4.91842592	4.82364393	5.16987408	1148	1433	1690
4194	NM_001204171,NM_001204172	MDM4	MDM4, p53 regulator	1.231229456	1.196814093	8.711375	10.993261	9.970444	6.4124769	6.70940097	6.66889605	3305	5437	4865
283	NM_001097577,NM_001145	ANG	angiogenin	-1.158772382	1.19668535	1.525669	1.40554	1.785347	1.50279892	1.36863803	1.67590018	72	83	106
58489	NM_021214	ABHD17C	abhydrolase domain containing 1	-1.597945342	1.196450591	19.881075	12.45734	22.352121	5.54520135	4.88729042	5.79889481	1794	1500	2640
51422	NM_001040633,NM_001304527	PRKAG2	protein kinase AMP-activated nor	-1.571141211	1.196188441	23.970544	15.288757	26.859173	5.73516237	5.09869445	5.98917078	2052	1745	3019
92140	NM_178812	MTDH	metadherin	1.148975431	1.19600086	17.652534	20.306248	19.840298	7.05297567	7.25191807	7.30941758	5174	7943	7611
166815	NM_145715	TIGD2	tigger transposable element deriv	1.162961259	1.19594392	7.565073	8.804603	8.499984	4.03201954	4.23744571	4.27568916	603	937	887
55225	NM_018211	RAVER2	ribonucleoprotein, PTB binding 2	-1.087483085	1.195733899	6.241581	5.746497	7.015753	4.78216862	4.66574286	5.0314812	1041	1279	1531
347735	NM_001199037,NM_001199038	SERINC2	serine incorporator 2	-1.077525425	1.195654577	106.654362	99.142306	120.072518	7.71190313	7.60471313	7.96858055	8192	10158	12047
3976	NM_001257135,NM_002309	LIF	LIF, interleukin 6 family cytokine	-1.079368698	1.195577694	238.937736	221.624628	267.992425	9.84624496	9.73618126	10.10369716	36099	44686	53083
93129	NM_152288	ORAI3	ORAI calcium release-activated c	1.251896081	1.195529486	10.520008	13.186665	11.814157	4.54933495	4.86103256	4.79690107	880	1472	1294
57150	NM_001042493,NM_001287445	SMIM8	small integral membrane protein	-1.062594682	1.195501932	1.602266	1.571028	1.746609	2.15835987	2.09080288	2.36229074	136	171	200
788	NM_000387	SLC25A20	solute carrier family 25 member 2	1.003455187	1.195404821	3.728993	3.743132	4.192586	2.97768275	2.98202989	3.20501631	270	362	397
2000	NM_001127197,NM_001421	ELF4	E74 like ETS transcription factor	1.150174381	1.195396851	10.638585	12.317997	12.051269	5.46843384	5.66603734	5.7206027	1699	2611	2498
1488	NM_001083914,NM_001290214	CTBP2	C-terminal binding protein 2	-1.011587838	1.195392405	30.136962	29.658563	33.713964	6.67340599	6.65694773	6.92858469	3968	5241	5834
101927809	NR_134605	LOC101927809	uncharacterized LOC101927809	-1.227348822	1.195341036	3.00133	2.424137	3.359338	1.44997615	1.26944952	1.61867597	68	74	100
8677	NM_001271609,NM_001271610	STX10	syntaxin 10	-1.036604679	1.195299075	26.950004	26.075159	30.211704	5.16981689	5.11941338	5.42064411	1374	1771	2020
51088	NM_001007075,NM_001171654	KLHL5	kelch like family member 5	-1.337242778	1.195286484	8.142595	6.152861	9.26149	5.92372626	5.51243584	6.1772057	2344	2342	3446
90416	NM_001080791,NM_001080792	C15orf57	chromosome 15 open reading fra	-1.32013401	1.195248117	2.521858	1.948283	2.804012	2.4003046	2.08433212	2.61239943	168	170	247
55613	NM_017677	MTMR8	myotubularin related protein 8	-1.659705177	1.195233919	0.49628	0.29881	0.55694	1.2010231	0.83317479	1.35230545	51	41	75
84340	NM_001281302,NM_032380,NM	GFM2	G elongation factor mitochondrial	1.050225172	1.195051222	21.038581	22.160947	23.79004	6.03606811	6.10571785	6.28955798	2537	3560	3729
4162	NM_006500	MCAM	melanoma cell adhesion molecul	-1.642595338	1.195021014	2.599631	1.585629	2.921573	3.24129226	2.61991364	3.47328584	332	270	488
147166	NM_001037330	TRIM16L	tripartite motif containing 16 like	1.726323264	1.194879234	7.615158	13.161879	8.541958	3.97240126	4.72101485	4.21424082	577	1331	848
1734	NM_000793,NM_001007023,NM	DIO2	iodothyronine deiodinase 2	18.62501565	1.194823715	0.339714	6.551042	0.395066	1.63881618	5.33711249	1.81825156	83	2068	122
218	NM_000691,NM_001135167,NM	ALDH3A1	aldehyde dehydrogenase 3 family	-3.825308139	1.194773023	1.778225	0.461433	2.227881	2.12505137	0.90836632	2.32699908	132	46	194
162993	NM_001077624	ZNF846	zinc finger protein 846	-1.024348869	1.194736405	2.819618	2.754588	3.163025	2.77786255	2.74825434	2.99995417	230	300	338
2274	NM_001039492,NM_001318894	FHL2	four and a half LIM domains 2	-1.073459636	1.194586155	69.487676	64.009553	77.830573	6.79751829	6.69620016	7.05192112	4328	5387	6359
960	NM_000610,NM_001001389,NM	CD44	CD44 molecule (Indian blood gro	-1.390420525	1.194411709	133.309712	93.620511	156.567164	9.40930152	8.93460611	9.66525732	26656	25615	39159
57644	NM_020884	MYH7B	myosin heavy chain 7B	1.756261568	1.194381196	0.06592	0.117969	0.075715	0.5190475	0.81765451	0.60191771	17	40	25
10010	NM_001199135,NM_004180,NM	TANK	TRAF family member associated	1.002549569	1.194375403	21.84774	21.641515	24.085104	5.39610359	5.39969027	5.6467896	1614	2162	2371
8226	NM_001135565,NM_001178135	PUDP	pseudouridine 5'-phosphatase	-1.113412586	1.194260018	7.228105	6.530088	8.169427	4.01393646	3.86901329	4.25549579	595	714	874
23355	NM_001009921,NM_001349292	VPS8	VPS8, CORVET complex subuni	1.23295503	1.193779749	19.269666	22.585337	19.841831	6.44826641	6.74727013	6.70112641	3389	5583	4976
745	NM_001127392,NM_013279	MYRF	myelin regulatory factor	-3.292620867	1.193758087	17.837754	5.349189	19.661622	6.66799555	4.9808379	6.92120803	3953	1604	5804
54890	NM_017758	ALKBH5	alkB homolog 5, RNA demethylas	1.346565911	1.193732997	9.713081	13.094834	10.894068	5.06504071	5.48322133	5.31352969	1275	2294	1872
51317	NM_001101802,NM_001352025	PHF21A	PHD finger protein 21A	1.142784672	1.19369487	4.366417	5.014686	4.895821	5.01022388	5.19718866	5.25839655	1226	1872	1800
29855	NM_001079514,NM_001288656	UBN1	ubinuclein 1	1.206460403	1.193519268	8.108583	9.794537	9.318906	5.66374685	5.92966236	5.91436016	1951	3145	2864
4750	NM_001199397,NM_001199398	NEK1	NIMA related kinase 1	-1.054198191	1.19347113	1.650445	1.56326	1.871222	3.34257292	3.27409343	3.57456234	359	455	527
22982	NM_014974	DIP2C	disco interacting protein 2 homol	1.093276595	1.193448918	2.322329	2.540813	2.604527	4.23689412	4.35902755	4.47961067	701	1024	1029
7411	NM_001303543,NM_001303544	VBP1	VHL binding protein 1	1.070700539	1.193440749	36.273932	38.994909	41.254613	5.91766008	6.01464262	6.16892453	2334	3339	3426
9184	NM_001007793,NM_004725	BUB3	BUB3, mitotic checkpoint protein	-1.00237976	1.193405045	55.137081	56.291168	64.382668	7.06564968	7.06224605	7.31899205	5220	6958	7662
51124	NM_016097	IER3IP1	immediate early response 3 inter	-1.206939225	1.193366614	30.218489	25.074574	33.894287	5.5004447	5.2356538	5.75032359	1738	1924	2551
6938	NM_001306219,NM_001306220	TCF12	transcription factor 12	1.044612082	1.193228211	7.033285	7.558069	8.09708	5.05512721	5.11624515	5.30297309	1266	1767	1858
55010	NM_001319988,NM_001319993	PARPBP	PARP1 binding protein	-1.145223618	1.19310634	7.171948	6.084459	8.26212	4.3072593	4.12214544	4.55017297	738	861	1083
93474	NM_001204220,NM_033213	ZNF670	zinc finger protein 670	1.350300246	1.192985435	1.718211	2.319869	1.925979	3.00540462	3.39143785	3.23070557	276	498	405
29883	NM_001322087,NM_001322088	CNOT7	CCR4-NOT transcription complex	1.068812608	1.192937477	17.774273	18.548885	19.67783	5.47753469	5.57146313	5.72682174	1710	2442	2509
131965	NM_001301790,NM_001301791	METTL6	methyltransferase like 6	1.443426406	1.192876329	5.184112	6.32605	6.001095	3.31329794	3.79763131	3.54415067	351	677	515
10283	NM_001297644,NM_001297645	CWC27	CWC27 spliceosome associated	1.193421779	1.192827482	18.480799	22.075116	20.759047	5.26940291	5.51844844	5.51774626	1475	2352	2164
22936	NM_012081	ELL2	elongation factor for RNA polyme	2.123855083	1.192772894	7.549546	16.052141	8.462638	5.50611581	6.57595105	5.75531028	1745	4952	2560
23075	NM_001297714,NM_015055	SWAP70	SWAP switching B-cell complex s	-1.603111346	1.192252016	9.646008	6.023176	10.86281	5.55069956	4.88821942	5.79943137	1801	1501	2641
29	NM_001092,NM_001159746,NM	ABR	active BCR-related	1.086675136	1.192153289	10.796146	11.961957	12.10228	5.76181945	5.87962364	6.01110952	2091	3036	3066
3749	NM_001039574,NM_004978,NR	KCNC4	potassium voltage-gated channel	-1.357901135	1.192115687	0.354113	0.259026	0.405292	1.33814853	1.08728891	1.4969322	60	59	88
79800	NM_001104586,NM_001282910	CARF	calcium responsive transcription	1.517361365	1.192076067	1.946472	2.650447	1.824541	3.21778616	3.76563483	3.44613034	326	661	478
55071	NM_017998	C9orf40	chromosome 9 open reading fran	-1.46240501	1.19205953	13.804506	9.445811	15.46291	5.01136367	4.48350191	5.25761579	1227	1121	1799
6904	NM_005993	TBCD	tubulin folding cofactor D	-1.088112134	1.191918443	19.782494	18.2015	22.15979	6.43261128	6.31225092	6.68321271	3352	4116	4914
55182	NM_001319956,NM_001319957	RNF220	ring finger protein 220	1.165406731	1.191910766	23.000474	27.325703	22.429	5.8650409	6.08236661	6.11433759	2249	3502	3297
340706	NM_001272046,NM_001320804	VWA2	von Willebrand factor A domain c	-14.57602291	1.191736575	0.785724	0.053547	0.878782	2.45494311	0.38406669	2.66516564	176	16	258
11133	NM_001291296,NM_007059,NR	KPTN	kaptin, actin binding protein	1.30227159	1.191653153	8.728474	11.207182	9.574556	3.91752664	4.276286	4.15509812	554	964	812
10313	NM_001265589,NM_001265590	RTN3	reticulon 3	1.382246058	1.191414491	88.014664	119.390409	97.348505	7.80866383	8.27390257	8.060308	8763	16184	12841
56061	NM_019116	UBFD1	ubiquitin family domain containing	1.192489612	1.191163507	10.175156	12.145477	11.389491	5.63962535	5.88893602	5.88735842	1918	3056	2810
6240	NM_001033,NM_001318064,NM	RRM1	ribonucleotide reductase catalytic	-1.180892287	1.191149089	52.081338	45.001734	59.403905	7.36572797	7.12742772	7.61668159	6436	7282	9429
8906	NM_001282474,NM_001282475	AP1G2	adaptor related protein complex 1	1.002139139	1.191066111	66.149814	66.00488	74.643352	7.44209654	7.44516171	7.69302238	6788	9089	9944
253724	NR_027249	TTC41P	tetratricopeptide repeat domain 4	1.592433541	1.191046763	0.086264	0.136908	0.095008	0.46682716	0.68708806	0.54161868	15	32	22

11167	NM_007085	FSTL1	follistatin like 1	-1.413264278	1.190986902	18.216419	12.903186	20.385698	6.1108172	5.62036393	6.35963286	2674	2528	3917
147965	NM_001351675,NM_174905	FAM98C	family with sequence similarity 98	-1.188110631	1.190822897	11.129375	9.403271	12.46561	3.8780834	3.64770479	4.11427824	538	605	788
26235	NM_001278716,NM_012160,NR	FBXL4	F-box and leucine rich repeat pro	1.069799036	1.190817521	2.966238	3.220093	3.334431	3.27204958	3.35963909	3.49993963	340	486	498
26267	NM_012166	FBXO10	F-box protein 10	1.633428822	1.190797165	0.809597	1.325737	0.90761	2.20692365	2.78861701	2.40804223	142	310	208
29072	NM_001349370,NM_014159,NR	SETD2	SET domain containing 2	1.163397695	1.190782466	7.360091	8.568786	8.356447	5.966078	6.18118623	6.21429619	2415	3754	3537
84266	NM_032306	ALKBH7	alkB homolog 7	-1.183233454	1.190736253	9.429119	7.978673	10.545393	3.72531928	3.50238056	3.95964434	480	542	703
7408	NM_003370	VASP	vasodilator-stimulated phosphopr	1.017888194	1.190612609	77.610046	79.083374	86.836266	7.44336387	7.46879769	7.6937441	6794	9240	9949
23659	NM_012320	PLA2G15	phospholipase A2 group XV	1.236439742	1.190514216	8.329909	10.32028	9.328591	4.53037804	4.82461482	4.77196635	868	1434	1271
387254	NR_002594	SLC7A5P2	solute carrier family 7 member 5	1.456338167	1.190348286	1.153907	1.683926	1.287162	1.94589385	2.36620557	2.1362985	112	218	164
9698	NM_001020658,NM_014676	PUM1	pumilio RNA binding family meml	1.141121469	1.190329028	20.674565	23.619729	23.127935	6.77286341	6.96168776	7.02211883	4254	6486	6228
80221	NM_001288968,NM_001288969	ACSF2	acyl-CoA synthetase family mem	1.139349537	1.190230932	66.724539	77.730916	73.587884	7.12608683	7.31303698	7.37568145	5445	8289	7971
619567	NR_002587	SNORD2	small nucleolar RNA, C/D box 2	1.361002999	1.189963663	51.449276	70.188774	57.621052	2.17473011	2.53229878	2.37386535	138	251	202
23007	NM_001130960,NM_001130961	PLCH1	phospholipase C eta 1	-3.673594124	1.189933201	0.745563	0.188725	0.800456	2.47491059	1.16293478	2.68388473	179	65	262
5686	NM_001199772,NM_001199773	PSMA5	proteasome subunit alpha 5	1.350632131	1.189727009	23.739633	32.143504	26.501247	6.47783974	6.9072778	6.72589413	3460	6244	5063
219	NM_000692	ALDH1B1	aldehyde dehydrogenase 1 family	1.953484649	1.189612885	3.929633	7.699485	4.395393	3.67442132	4.5843929	3.90684171	462	1206	676
5929	NM_001193272,NM_001193273	RBBP5	RB binding protein 5, histone lysi	1.039829613	1.189569352	7.330547	7.605744	8.177007	5.00336619	5.05799413	5.24664042	1220	1695	1785
23271	NM_001297707,NM_001297708	CAMSAP2	calmodulin regulated spectrin ass	1.079617066	1.189191137	7.955308	8.607597	8.898454	5.85618712	5.96487401	6.10221143	2235	3224	3269
5451	NM_001198783,NM_001198786	POU2F1	POU class 2 homeobox 1	1.023418275	1.188992523	2.835832	2.921696	3.184779	5.30794575	5.34051034	5.55190154	1516	2073	2217
84542	NM_001129993,NM_001330432	KIAA1841	KIAA1841	1.110548347	1.188920729	2.150601	2.389481	2.426149	3.38891307	3.52644784	3.61657686	372	552	544
113828	NM_138435	FAM83F	family with sequence similarity 83	2.26232561	1.188822333	1.000945	2.281488	1.121581	1.602974	2.48886356	1.77525664	80	242	117
55819	NM_001280801,NM_018434	RNF130	ring finger protein 130	1.125468602	1.188817182	12.235988	13.791698	13.65964	4.80469545	4.96947085	5.04602381	1058	1591	1547
11145	NM_001128203,NM_007069	PLA2G16	phospholipase A2 group XVI	1.202042743	1.18881698	41.000603	49.302116	44.598055	5.72408203	5.98498794	5.96927963	2036	3270	2977
79073	NM_024092	TMEM109	transmembrane protein 109	1.317110065	1.188756433	22.257387	29.350498	24.862429	5.72129859	6.11209361	5.96641552	2032	3576	2971
80227	NM_001267803,NM_001267804	PAAF1	proteasomal ATPase associated	-1.041753237	1.188597319	16.238973	15.610084	18.148469	4.72356761	4.666826	4.96416199	998	1280	1459
121441	NM_001135175,NM_001135176	NEDD1	neural precursor cell expressed,	-1.123011877	1.188545729	13.273166	12.119101	14.838983	5.69316485	5.52920837	5.93794468	1992	2370	2912
55628	NM_001146189,NM_001146190	ZNF407	zinc finger protein 407	1.233325725	1.188410828	1.297544	1.597486	1.436582	3.45742019	3.73497962	3.68554025	392	646	573
5272	NM_004155	SERPINB9	serpin family B member 9	1.256680538	1.188379004	3.533067	4.445169	3.946865	3.93444966	4.24471769	4.16845206	561	942	820
55048	NM_017966	VPS37C	VPS37C, ESCRT-I subunit	1.205975006	1.188139056	8.544182	10.323956	9.545982	4.54933495	4.80900168	4.78827692	880	1418	1286
55146	NM_001134387,NM_001134388	ZDHHC4	zinc finger DHHC-type containing	1.334790527	1.188116797	14.419114	18.79934	15.874163	4.37612225	4.77525187	4.61378826	776	1384	1134
23397	NM_001281710,NM_001281711	NCAPH	non-SMC condensin I complex su	1.283728503	1.188070381	13.082085	16.874527	14.588729	6.28397262	6.64022606	6.52966751	3020	5180	4413
5867	NM_001271998,NM_004578,NR	RAB4A	RAB4A, member RAS oncogene	-1.146379979	1.188060201	7.381833	6.452957	8.304126	4.50470835	4.31687056	4.74325038	852	993	1245
27042	NM_014388	DIEXF	digestive organ expansion factor	1.097695753	1.188046411	3.973886	4.368161	4.438419	5.08141332	5.21210456	5.32326357	1290	1892	1885
3674	NM_000419	ITGA2B	integrin subunit alpha 2b	-1.76454619	1.1880349	0.593548	0.331404	0.66264	1.57857476	1.08728891	1.74883089	78	59	114
79934	NM_001142555,NM_024876	COQ8B	coenzyme Q8B	-1.165624354	1.187947801	8.118644	7.088412	9.358706	4.34572074	4.13629029	4.58295467	759	870	1109
2100	NM_001040275,NM_001214902	ESR2	estrogen receptor 2	1.801719032	1.187765409	0.246338	0.454267	0.281786	0.99521185	1.48128072	1.12461302	39	94	57
388552	NM_212550	BLOC1S3	biogenesis of lysosomal organelle	-1.278255324	1.187744231	8.227259	6.441424	9.177067	4.44859483	4.1126379	4.6863673	818	855	1195
10626	NM_001348119,NM_001348120	TRIM16	tripartite motif containing 16	-1.175115348	1.187689759	26.124814	16.834626	27.881904	5.87822053	5.64969525	6.12250838	2270	2581	3316
9681	NM_001007188,NM_001136029	DEPDC5	DEP domain containing 5	1.192473571	1.187676649	1.562069	1.945212	1.853435	3.24129226	3.47048058	3.46519272	332	529	485
4943	NM_001348262,NM_001348263	TBC1D25	TBC1 domain family member 25	-1.207919586	1.187483383	2.21828	1.86673	2.491865	3.23349937	2.99243508	3.45705395	330	365	482
7027	NM_007111,NR_026580	TFDP1	transcription factor Dp-1	1.213150915	1.187452717	29.65793	35.512843	32.511019	6.21935475	6.49471706	6.46417373	2886	4678	4215
9738	NM_001199022,NM_001323569	CCP110	centriolar coiled-coil protein 110	1.740962265	1.187303387	4.239378	7.482661	4.785941	4.57268608	5.34660637	4.81080629	895	2082	1307
85414	NM_033102	SLC45A3	solute carrier family 45 member 3	1.457474961	1.187260155	27.931162	40.753948	31.161236	6.53878911	7.07739484	6.78398274	3611	7032	5273
91749	NM_153369	MFSD4B	major facilitator superfamily doma	1.46083776	1.187145913	0.841261	1.225974	0.935572	2.07360076	2.50832973	2.26619062	126	246	184
2353	NM_005252	FOS	Fos proto-oncogene, AP-1 transc	1.263561993	1.186922381	57.207016	72.364243	63.802048	6.92122397	7.25624213	7.16657906	4719	7967	6889
143872	NM_152432	ARHGAP42	Rho GTPase activating protein 42	1.067637586	1.186723907	1.160242	1.238885	1.292634	2.67229041	2.75234181	2.88332104	211	301	308
388407	NM_203425	C17orf82	chromosome 17 open reading fra	1.264404815	1.186695124	0.205187	0.274243	0.239036	0.41264541	0.50527715	0.47868902	13	22	19
101929351	NR_121597,NR_121598	CDC37L1-AS1	CDC37L1 antisense RNA 1 (head	-1.021559294	1.186695124	1.111906	1.126187	1.316045	0.41264541	0.40498891	0.47868902	13	17	19
677837	NR_002986	SNORA60	small nucleolar RNA, H/ACA box	1.035997523	1.186695124	2.394054	2.535824	2.731361	0.41264541	0.42561204	0.47868902	13	18	19
29089	NM_001310326,NM_014176	UBE2T	ubiquitin conjugating enzyme E2	-1.240868227	1.18662448	134.304589	108.369274	149.797208	6.90445095	6.59599096	7.14942456	4664	5022	6807
5609	NM_001297555,NM_001297556	MAP2K7	mitogen-activated protein kinase	-1.000620203	1.186077336	7.609906	7.61122	8.489636	4.75384679	4.75298538	4.99165559	1020	1362	1488
373156	NM_001143679,NM_001143680	GSTK1	glutathione S-transferase kappa	-1.560174877	1.186061427	134.32333	86.672349	154.549299	7.22519698	6.58886576	7.46986664	5835	4997	8512
151613	NM_001042601,NM_001288582	TTC14	tetratricopeptide repeat domain 1	1.221393475	1.186054298	19.824609	24.139833	19.567113	6.22575196	6.51079113	6.46890357	2899	4731	4229
23008	NM_014997	KLHDC10	kelch domain containing 10	-1.307479005	1.18595687	5.011241	3.837391	5.587103	5.01931705	4.64610598	5.25839655	1234	1261	1800
9482	NM_004853,NR_033656	STX8	syntaxin 8	1.060588496	1.185820793	10.537325	11.19319	11.771082	3.43720745	3.51446439	3.66213029	386	547	563
84064	NM_001318765,NM_032124	HDHD2	haloacid dehalogenase like hydro	1.021321652	1.18577143	7.683897	7.938609	8.608495	4.13391036	4.16263578	4.36684138	650	887	948
9240	NM_006029	PNMA1	paraneoplastic Ma antigen 1	1.198696368	1.185608738	13.390866	16.069931	14.919222	5.14821864	5.40294404	5.38748191	1353	2167	1973
254102	NM_001099409,NM_001351087	EHBP1L1	EH domain binding protein 1 like	1.352283964	1.185360338	18.651334	25.505245	21.094192	6.55802658	6.98944134	6.80096283	3660	6613	5336
51185	NM_001173482,NM_016302	CRBN	cereblon	-1.005900934	1.185322063	5.992303	5.967163	6.67327	4.01166006	4.00369809	4.24292715	594	789	866
10491	NM_006371	CRTAP	cartilage associated protein	1.079715565	1.185110262	9.186324	9.92974	10.230702	5.91887536	6.02776984	6.16017783	2336	3370	3405
51663	NM_016107,NR_144318	ZFR	zinc finger RNA binding protein	1.174604033	1.185041052	14.978895	17.615662	16.750388	6.12878335	6.35789825	6.37050671	2708	4250	3947
5462	NM_001159542	POU5F1B	POU class 5 homeobox 1B	-1.068571065	1.184835813	0.381425	0.365407	0.42952	0.68823103	0.65251278	0.78645063	24	30	35
503693	NR_024061	LOH12CR2	loss of heterozygosity, 12, chrom	-1.068571065	1.184835813	0.399144	0.373261	0.445541	0.68823103	0.65251278	0.78645063	24	30	35
8729	NM_001199378,NM_001199379	GBF1	golgi brefeldin A resistant guanin	-1.170409337	1.184646745	17.748516	15.184917	19.764722	6.80968951	6.58486022	7.05214626	4365	4983	6360
132	NM_001123,NM_001202449,NM	ADK	adenosine kinase	-1.384070312	1.18463998	34.236232	24.448143	38.727653	6.16098005	5.69976476	6.40229181	2770	2674	4036
9048	NM_001136215,NM_057090,NM	ARTN	artemin	1.026397482	1.184534285	1.419606	1.447795	1.562992	1.87753473	1.90501868	2.05953665	105	144	153
494514	NM_001012716	TYMSOS	TYMS opposite strand	-1.762632453	1.184435926	2.159647	1.223779	2.414706	1.47662927	1.00745609	1.63800401	70	53	102
57685	NM_001293274,NM_020925	CACHD1	cache domain containing 1	1.211919181	1.184338283	1.431312	1.723249	1.574547	3.06370142	3.31058687	3.28075123	289	468	421
100129794	NR_033240	SLC25A21-AS1	SLC25A21 antisense RNA 1	1.353416497	1.18431697	2.383213	3.248239	2.666997	2.3579234	2.71931318	2.5576298	162	293	236
9918	NM_014865	NCAPD2	non-SMC condensin I complex su	-1.1006015	1.184086665	44.897186	40.838402	49.954174	7.72168503	7.58407836	7.96439979	8248	10013	12012
54751	NM_001024215,NM_001024216	FBLIM1	filamin binding LIM protein 1	-1.412558973	1.183894522	12.857935	9.800064	15.41109	5.38998216	4.90575769	5.62818389	1607	1520	2340
65124	NM_023016	SOWAHC	sosondowah ankyrin repeat doma	1.399887754	1.183866044	3.904372	5.474687	4.346055	4.22515306	4.68831976	4.45666286	695	1300	1012
9639	NM_001308152,NM_001308153	ARHGEF10	Rho guanine nucleotide exchang	-1.104071752	1.183735355	8.364792	7.634349	9.274913	5.52618969	5.38660128	5.7646828	1770	2142	2577
201725	NM_001008393,NR_077234,NR	C4orf46	chromosome 4 open reading fran	-1.111296988	1.183522926	7.24608	6.44871	7.918766	4.66827758	4.52231714	4.90256532	959	1153	1396
7730	NM_001172651,NM_003451	ZNF177	zinc finger protein 177	-1.464353624	1.183404086	0.719412	0.495242	0.806874	1.44997615	1.12560759	1.60891398	68	62	99
56252	NM_019589	YLPM1	YLP motif containing 1	1.407483339	1.183349667	8.694906	12.253394	9.670073	6.12720707	6.61435345	6.36689119	2705	5087	3937
8370	NM_003548,NM_003548_dup1	HIST2H4A	histone cluster 2 H4 family memb	1.969248623	1.183294994	5.214502	10.294545	5.763483	1.59082596	2.31185141	1.75769337	79	208	115
54058	NM_001286462,NM_001286463	C21orf58	chromosome 21 open reading fra	-1.031516347	1.183204033	9.2659	9.040999	9.958123	4.50146728	4.45870114	4.73429846	850	1101	1237
56997	NM_020247	COQ8A	coenzyme Q8A	1.037466666	1.18316035	6.994185	7.26024	7.778918	4.38670105	4.43728	4.61866105	782	1084	1138
694	NM_001731	BTG1	BTG anti-proliferation factor 1	1.184645712	1.182641967	13.292782	15.762989	14.772149	5.94416257	6.18497139	6.18256308	2378	3764	3459
6837	NM_133640,NM_181491	MED22	mediator complex subunit 22	1.449018005	1.182602729	8.433475	12.133666	9.39764	5.00451141	5.52563064	5.23954048	1221	2364	1776
254295	NM_001100876,NM_001100877	PHYHD1	phytanoyl-CoA dioxygenase dom	-9.298919168	1.182486647	1.882489	0.193898	2.088218	2.29940604	0.50527715	2.49540988	154	22	224
58506	NM_021228	SCAF1	SR-related CTD associated facto	-1.031665093	1.182481679	26.924259	26.124912	29.917213	6.83372816	6.7891528	7.07359842	4439	5749	6456
118788	NM_152309	PIK3AP1	phosphoinositide-3-kinase adapto	-7.82546016	1.182321849	1.72233	0.219772	1.911162	3.18179291	1.02107213	3.39875952	317	54	461
9554	NM_004892	SEC22B	SEC22 homolog B, vesicle traffic	1.044744475	1.182259948	26.482065	27.705427	29.427889	5.63149439	5.69340099	5.86856107	1907	2662	2773
80019	NM_024954	UBTD1	ubiquitin domain containing 1	-1.555896645	1.182191639	8.746028	5.626354	9.710252	3.94641713	3.35963909	4.17342809	566	486	823
10181	NM_005778,NR_036627	RBM5	RNA binding motif protein 5	1.137095464	1.182068875	28.393649	32.400084	31.835032	6.44994873	6.63331653	6.68872607	3393	5155	4933
9672	NM_014654	SDC3	syndecan 3	1.558219287	1.18197051	2.485408	3.877605	2.763115	3.74463792	4.34556265	3.96921401	487	1014	708
81573	NM_030816	ANKRD13C	ankyrin repeat domain 13C	-1.098172077	1.181916225	4.981487	4.544317	5.537285	4.83333524	4.7031771	5.0666753	1080	1314	1570
10209	NM_005801	EIF1	eukaryotic translation initiation fa	-1.166043162	1.181820428	506.018097	434.438141	561.968079	9.35377093	9.13251481	9.59444346	25648	29389	37281
692085	NR_003042	SNORD45C	small nucleolar RNA, C/D box 45	1.020492746	1.181693594	7.136403	7.496786	8.032857	0.64188297	0.65251278	0.73352601	22	30	32
54462	NM_001284240,NM_001284241	CCSER2	coiled-coil serine rich protein 2	-1.338788824	1.181624323	4.063772	3.010466	5.591176	4.70677986	4.30439709	4.93905904	986	984	1433
260425	NM_001142782,NM_152900	MAGI3	membrane associated guanylate	-1.04755947	1.181398644	1.977669	1.815665	2.224069	3.78251604	3.72044785	4.0068703	501	639	728
29803	NM_001099695,NM_001099696	REPIN1	replication initiator 1	-1.587839741	1.181376566	29.120113	18.573585	31.81824	6.45540274	5.79794319	6.69335261	3406	2866	4949
55111	NM_001300836,NM_018049	PLEKHJ1	pleckstrin homology domain cont	1.006571955	1.181130591	28.574523	28.812323	31.645507	5.1492545	5.15844008	5.38319304	1354	1821	1967
25902	NM_001242767,NM_001242768	MTHFD1L	methylenetetrahydrofolate dehyd	1.270513382	1.18097979	9.872839	11.040966	9.496318	4.66827758	5.00159078	4.89956551	959	1628	1393
27309	NM_001292002,NM_014487	ZNF330	zinc finger protein 330	-1.172866719	1.180820136	27.318574	23.342828	30.340704	5.68675843	5.46153982	5.92226458	1983	2259	2880
55124	NM_001135721,NM_001330480	PIWIL2	piwi like RNA-mediated gene sile	1.424697675	1.180777034	0.060279	0.092587	0.076838	0.35634914	0.48576948	0.41288872	11	21	16
682	NM_001322243,NM_001728,NM	BSG	basigin (Ok blood group)	1.145199835	1.180739146	335.627021	384.827168	372.511971	9.15475131	9.35003059	9.39405519	22338	34180	32440
132789	NM_001270880,NM_001270881	GNPDA2	glucosamine-6-phosphate deami	-1.349317974	1.180722434	8.224337	6.19097	9.224046	4.25817433	3.85197241	4.48629114	712	705	1034

342371	NM_001137675	ATXN1L	ataxin 1 like	1.169347771	1.180714025	8.397079	9.832064	9.318309	6.03326541	6.25578446	6.26955915	2532	3956	3677
55435	NM_001128426,NM_018569,NR	AP1AR	adaptor related protein complex 1	-1.001205631	1.180704666	4.978074	4.998573	5.503381	4.89638579	4.89470572	5.12862225	1130	1508	1641
10254	NM_005843	STAM2	signal transducing adaptor molec	1.100714357	1.18065082	4.694619	5.177332	5.213488	4.75928446	4.89285546	4.99071621	1024	1506	1487
389538	NR_002822	MGC72080	MGC72080 pseudogene	1.268773315	1.180265729	7.371102	9.370424	8.166501	2.58923814	2.8811152	2.79136857	197	334	286
84852	NR_024124,NR_024125,NR_02	ATP1A1-AS1	ATP1A1 antisense RNA 1	1.038107159	1.180018171	1.631224	1.955224	1.72503	1.75224037	1.79043244	1.92438744	93	129	135
25962	NM_015496,NM_183009	KIAA1429	KIAA1429	-1.079362158	1.179859475	13.093806	11.856056	13.804875	6.19949123	6.09086509	6.43511915	2846	3523	4130
56904	NM_001287045,NM_001287046	SH3GLB2	SH3 domain containing GRB2 lik	-1.062669725	1.179624785	31.15508	29.372296	34.24484	6.12299522	6.03659509	6.3581768	2697	3391	3913
100506084	NM_001093083,NM_001103154	ARL17B	ADP ribosylation factor like GTPa	5.152277941	1.179585714	1.231856	3.441233	1.265617	0.83964067	2.34470901	0.95003448	31	214	45
26046	NM_001320766,NM_015565	LTN1	listerin E3 ubiquitin protein ligase	1.064436541	1.17943864	6.526226	6.958696	7.264534	5.65210193	5.74045883	5.8858434	1935	2752	2807
57692	NM_020932	MAGEE1	MAGE family member E1	1.517561509	1.179354778	0.998857	1.52371	1.110581	2.20692365	2.69827959	2.39673966	142	288	206
55670	NM_001127649,NM_001199319	PEX26	peroxisomal biogenesis factor 26	1.557626802	1.1793014	17.059967	26.795655	19.303875	6.14860191	6.78067287	6.38344806	2746	5715	3983
55197	NM_001303411,NM_001303412	RPRD1A	regulation of nuclear pre-mRNA d	1.08079939	1.179297395	9.520663	9.177864	9.145391	5.03956952	5.14839425	5.27083173	1252	1808	1816
1652	NM_001084392,NM_001355	DDT	D-dopachrome tautomerase	1.27677555	1.179079668	69.646252	91.495384	77.437816	5.46677296	5.81220815	5.69948515	1697	2895	2461
55294	NM_001013415,NM_001257069	FBXW7	F-box and WD repeat domain co	-1.04275304	1.178796612	7.987539	7.624239	9.065141	5.23175921	5.17299774	5.46325552	1436	1840	2082
6929	NM_001136139,NM_001351778	TCF3	transcription factor 3	1.215455988	1.178594858	21.665943	26.269027	22.913825	6.39904131	6.67751372	6.63352381	3274	5317	4746
2308	NM_002015	FOXO1	forkhead box O1	1.430260501	1.178282718	0.715991	1.029619	0.7966	2.32896139	2.75641772	2.52166171	158	302	229
11168	NM_001128217,NM_001317898	PSIP1	PC4 and SFRS1 interacting prote	1.25297999	1.178190194	33.305411	39.223437	36.142927	6.30967929	6.63137592	6.5435063	3075	5148	4456
1938	NM_001961	EEF2	eukaryotic translation elongation	1.166593389	1.178157702	399.798767	466.914032	442.63092	10.26474887	10.48688366	10.5011047	48261	75225	69933
222658	NM_001286579,NM_001286580	KCTD20	potassium channel tetramerizatic	-1.2809051	1.178132609	5.765599	4.456563	6.418066	4.98839509	4.64390751	5.21802858	1207	1259	1749
388324	NM_001167985,NM_001167986	INCA1	inhibitor of CDK, cyclin A1 interac	-2.41097265	1.177926435	0.332534	0.130776	0.398524	0.59399636	0.27464693	0.6785857	20	11	29
286148	NM_181787	DPY19L4	dpy-19 like 4 (C. elegans)	-1.125354293	1.177904174	5.934956	5.280073	6.570089	5.2110752	5.04555672	5.44141974	1415	1680	2050
7840	NM_015120	ALMS1	ALMS1, centrosome and basal b	1.695396478	1.177855873	1.57809	2.679268	1.747056	4.38318339	5.11624515	4.60889895	780	1767	1130
9019	NM_001146191,NM_003953,NM	MPZL1	myelin protein zero like 1	1.150537128	1.177782346	14.882565	17.032643	16.421986	6.19197132	6.39170165	6.42507101	2831	4352	4101
57799	NM_001172663,NM_001172664	RAB40C	RAB40C, member RAS oncogen	1.20069946	1.177749616	10.199342	12.34529	11.291757	4.75520813	5.01015048	4.98317903	1021	1638	1479
23512	NM_001321207,NM_015355	SUZ12	SUZ12 polycomb repressive com	1.108867581	1.177676381	20.508453	22.756911	22.741953	6.49545999	6.64298064	6.72899562	3503	5190	5074
3958	NM_001177388,NM_002306,NR	LGALS3	galectin 3	1.278725075	1.177481149	363.988648	485.484066	409.975739	8.6986746	9.05262573	8.93385659	16273	27803	23567
1969	NM_001329090,NM_004431	EPHA2	EPH receptor A2	-1.582009293	1.177477119	175.538849	111.294757	195.166115	9.41065196	8.75012372	9.64603236	26681	22534	38640
84937	NM_032268	ZNRF1	zinc and ring finger 1	-1.034378847	1.177450003	10.352524	10.019579	11.454894	5.5763411	5.5286127	5.80745591	1834	2369	2656
5883	NM_001243224,NM_004584	RAD9A	RAD9 checkpoint clamp compon	-1.036159468	1.177394469	19.269071	18.413268	21.087513	5.26940291	5.21950507	5.49937013	1475	1902	2136
22887	NM_001198850,NM_001198851	FOXJ3	forkhead box J3	1.189561733	1.177205005	17.813593	21.363288	19.860821	6.49912087	6.74701411	6.73209045	3512	5582	5085
6843	NM_001297438,NM_014231,NM	VAMP1	vesicle associated membrane pr	1.232674876	1.177031459	5.411557	6.565821	5.857961	4.05209571	4.33742297	4.27414582	612	1008	886
284365	NR_038359	LINC01869	long intergenic non-protein codin	-1.106637167	1.17683836	0.78537	0.714454	0.882954	0.79797761	0.73744528	0.90287695	29	35	42
5090	NM_001134778,NM_001330782	PBX3	PBX homeobox 3	-1.195814295	1.176587257	8.166167	6.871955	9.082339	4.51920371	4.27344453	4.74436548	861	962	1246
51101	NM_016010	ZC2HC1A	zinc finger C2HC-type containing	-1.151001821	1.176552092	3.539846	3.082838	3.915998	3.65121051	3.46550967	3.8684907	454	527	657
64062	NM_001286631,NM_001286632	RBM26	RNA binding motif protein 26	1.072852985	1.176337583	8.741368	9.34923	9.630821	5.48657849	5.58585016	5.71606286	1721	2467	2490
2592	NM_000155,NM_001258332	GALT	galactose-1-phosphate uridylyltra	1.193701857	1.1762516	9.018863	10.761887	9.947739	3.72253824	3.96018541	3.94031242	479	764	693
114883	NM_001330580,NM_001350208	OSBPL9	oxysterol binding protein like 9	1.194081122	1.176141427	23.164223	29.656214	27.248208	6.46375348	6.71700246	6.69537206	3426	5466	4956
254428	NM_173854	SLC41A1	solute carrier family 41 member 1	1.414277673	1.176045081	7.985171	11.307961	8.827104	5.32271189	5.81220815	5.55126453	1532	2895	2216
9711	NM_001145642,NM_001346873	RUBCN	RUN and cysteine rich domain co	-1.335588984	1.176001256	7.308985	5.47754	8.074531	6.06763988	5.6573446	6.29831595	2594	2595	3752
7037	NM_001128148,NM_001313965	TFRC	transferrin receptor	1.769940081	1.175988054	42.905114	75.945734	47.488706	7.73571752	8.55647872	7.96858055	8329	19697	12047
115209	NM_145243	OMA1	OMA1 zinc metallopeptidase	-1.13650872	1.175879697	13.083507	11.52471	14.459143	4.68122618	4.50425245	4.90655538	968	1138	1400
10331	NM_014256	B3GNT3	UDP-GlcNAc:betaGal beta-1,3-N	1.061040929	1.17585363	34.694546	36.857212	38.341675	6.53760293	6.62219199	6.76899367	3608	5115	5218
23215	NM_015172	PRRC2C	proline rich coiled-coil 2C	-1.013628618	1.175821681	28.276674	27.927898	31.245083	8.16434239	8.14488157	8.39726166	11224	14795	16232
22884	NM_014023	WDR37	WD repeat domain 37	1.126313036	1.175687422	7.516625	8.472733	8.304806	5.11890861	5.2858656	5.34621347	1325	1994	1916
26574	NM_012138	AATF	apoptosis antagonizing transcript	1.259250546	1.175463847	45.7183	57.633293	50.502396	6.58965693	6.91914353	6.82065577	3742	6296	5410
6993	NM_001291602,NM_001291603	DYNLT1	dynein light chain Tctex-type 1	1.074177833	1.175449515	58.196187	62.56544	64.290783	5.48082991	5.58183624	5.70922621	1714	2460	2478
23580	NM_012121	CDC42EP4	CDC42 effector protein 4	1.000752939	1.175431241	40.009838	40.087975	44.198174	6.92848243	6.9295594	7.15990887	4743	6342	6857
55100	NM_001345998,NM_001345999	WDR70	WD repeat domain 70	1.125974758	1.175179768	6.765551	7.60684	7.425334	3.92963473	4.0902069	4.14837451	559	841	808
54469	NM_001242911,NM_001242912	ZFAND6	zinc finger AN1-type containing 6	-1.147145788	1.175112365	33.992005	29.880343	37.06545	5.80057409	5.60631865	6.02952154	2149	2503	3106
142	NM_001618	PARP1	poly(ADP-ribose) polymerase 1	1.21749205	1.175026143	33.304123	40.597004	36.781036	7.02503325	7.30697082	7.25607987	5074	8254	7333
3110	NM_001165255,NM_005515	MNX1	motor neuron and pancreas hom	-3.238336977	1.175001141	1.444069	0.448073	1.611039	2.01115992	0.95166931	2.18966672	119	49	172
25949	NM_015484,NM_207170	SYF2	SYF2 pre-mRNA splicing factor	-1.014820785	1.174895631	29.349201	29.000658	32.469574	5.69387492	5.67306183	5.92226458	1993	2624	2880
7158	NM_001141979,NM_001141980	TP53BP1	tumor protein p53 binding protein	1.223739341	1.174770466	13.366554	16.374967	14.754054	6.35572529	6.64380598	6.58548856	3176	5193	4589
81609	NM_001330723,NM_030918	SNX27	sorting nexin family member 27	-1.023332075	1.174729799	9.8732	9.637609	10.700658	5.99055812	5.9578116	6.21951784	2457	3208	3550
100505573	NM_001301268	INAFM2	InaF motif containing 2	1.387796299	1.174583317	8.9763	12.474712	9.910284	4.60021751	5.05634199	4.82352399	913	1693	1319
11104	NM_001204076,NM_007044	KATNA1	katanin catalytic subunit A1	1.20151258	1.174554604	6.404971	7.674674	6.967986	3.72253824	3.96899357	3.93836491	479	769	692
933	NM_001185099,NM_001185100	CD22	CD22 molecule	-1.537474761	1.174486118	7.514265	4.912077	8.296655	4.56959435	3.98123534	4.79259544	893	776	1290
57466	NM_001145444,NM_001145445	SCAF4	SR-related CTD associated facto	-1.139806404	1.174449784	10.181332	8.95419	11.234891	5.42632706	5.24220835	5.65333437	1649	1933	2382
115294	NM_001286782,NM_001286783	PCMTD1	protein-L-isoaspartate (D-asparta	-1.158132244	1.174437675	14.547393	12.430221	15.891526	5.89499076	5.68700902	6.12336578	2297	2650	3318
54812	NM_001002243,NM_017657,NM	AFTPH	aftiphilin	1.182503717	1.174334521	9.806546	11.621576	10.825788	5.31072594	5.54696516	5.53717864	1519	2400	2194
166379	NM_001178007,NM_152618	BBS12	Bardet-Biedl syndrome 12	1.039466219	1.174286247	1.292429	1.357331	1.436509	2.3579234	2.40307061	2.54744438	162	225	234
2300	NM_005250	FOXL1	forkhead box L1	-1.425401186	1.174082698	16.487961	11.582532	18.193048	5.7065968	5.20690152	5.9340406	2011	1885	2904
1798	NM_001382	DPAGT1	dolichyl-phosphate N-acetylgluco	-2.042847694	1.173919666	32.192959	15.768855	35.510487	6.09046901	5.08173995	6.31867157	2636	1724	3806
693130	NR_030382	MIR548D1	microRNA 548d-1	-1.334792626	1.17386541	9.666614	7.102761	10.670473	0.93882015	0.75384785	1.05442063	36	36	52
865	NM_001755,NM_022845	CBFB	core-binding factor beta subunit	-1.233141724	1.173852789	24.943396	20.202359	27.451295	6.26019865	5.96222966	6.4886678	2970	3218	4288
10137	NM_001198838,NM_001198840	RBM12	RNA binding motif protein 12	1.291435648	1.173839719	14.482998	18.708352	15.958223	6.57277081	6.93833172	6.80176645	3698	6381	5339
84445	NM_001318099,NM_001318100	LZTS2	leucine zipper tumor suppressor	-1.437282001	1.173712494	23.187112	16.180218	25.407264	6.01519803	5.50154961	6.24298142	2500	2324	3609
55247	NM_018248	NEIL3	nei like DNA glycosylase 3	1.13475643	1.173678516	11.088041	12.602056	12.232906	4.75248416	4.92851423	4.97560227	1019	1545	1471
51257	NM_001005415,NM_001005416	MARCH2	membrane associated ring-CH-ty	1.014607523	1.173569467	4.186746	4.477112	4.870725	2.82529613	2.84329233	3.02586351	239	324	345
79893	NM_024835	GGNBP2	gametogenetin binding protein 2	1.043367143	1.173557555	58.896221	61.515453	64.949554	7.35880455	7.41968708	7.58839643	6405	8929	9245
2444	NM_002031	FRK	fyn related Src family tyrosine kin	-1.408103435	1.173275262	0.887441	0.626955	0.975834	1.79522562	1.4614436	1.96321628	97	92	140
8714	NM_001144070,NM_003786	ABCC3	ATP binding cassette subfamily C	1.346760375	1.173261325	102.857067	138.95604	112.827609	8.99331363	9.42207973	9.2234211	19969	35933	28816
8733	NM_003801	GPAA1	glycosylphosphatidylinositol anch	1.285041369	1.173259729	32.104511	41.30352	35.400093	6.03606811	6.39301127	6.26334948	2537	4356	3661
5527	NM_001161725,NM_001161726	PPP2R5C	protein phosphatase 2 regulatory	1.069555708	1.17299806	64.311008	68.341342	62.947378	7.07984401	7.17616385	7.30847548	5272	7534	7606
22794	NM_007359	CASC3	cancer susceptibility 3	1.130128507	1.172942468	17.839525	20.185354	19.666624	6.20198918	6.37622391	6.42923735	2851	4305	4113
64418	NM_001287497,NM_022484,NM	TMEM168	transmembrane protein 168	-5.035295193	1.172706165	4.227235	0.839033	4.655346	4.95679582	2.80050884	5.17979929	1180	313	1702
27069	NM_014394	GHITM	growth hormone inducible transm	-1.235673985	1.172503172	63.466122	51.420639	69.930832	7.25123928	6.94816508	7.47944121	5942	6425	8569
1022	NM_001324069,NM_001324070	CDK7	cyclin dependent kinase 7	-1.011790638	1.172452408	41.884126	41.067163	45.008972	5.90973571	5.89310706	6.13574112	2321	3065	3347
60559	NM_021928	SPCS3	signal peptidase complex subuni	1.059436568	1.172435709	29.013006	30.768091	31.962374	7.03150809	7.11418819	7.25939875	5097	7215	7350
55148	NM_175748,NR_038150	UBR7	ubiquitin protein ligase E3 compo	-1.042893259	1.172397564	13.116399	12.65442	14.473911	5.59090027	5.53158861	5.81596651	1853	2374	2672
100506621	NR_109779	LINC01279	long intergenic non-protein codin	2.970309729	1.172261045	0.043588	0.134158	0.046341	0.29776659	0.75384785	0.34394329	9	36	13
100616314	NR_039905	MIR4750	microRNA 4750	1.244483702	1.172261045	4.144361	5.028633	4.597725	0.29776659	0.36283659	0.34394329	9	15	13
105374546	NR_134673,NR_134674,NR_13	LOC105374546	uncharacterized LOC105374546	-1.980810008	1.172261045	0.445643	0.237595	0.428609	0.29776659	0.15624221	0.34394329	9	6	13
100422943	NR_036156	MIR3189	microRNA 3189	-2.937033591	1.172261045	3.012468	1.05811	3.394182	0.29776659	0.10601836	0.34394329	9	4	13
22864	NM_001330121,NM_001330122	R3HDM2	R3H domain containing 2	-1.228115244	1.172182057	14.799257	12.123045	16.332912	5.98360608	5.69233762	6.20945938	2445	2660	3525
9553	NM_004891,NM_145330	MRPL33	mitochondrial ribosomal protein L	-1.035804049	1.172135329	105.774054	102.783445	117.066746	5.69671172	5.64695348	5.92177183	1997	2576	2879
55364	NM_018439	IMPACT	impact RWD domain protein	1.116699984	1.172005354	14.456774	16.163183	15.92113	5.75230681	5.90875638	5.97736388	2077	3099	2994
5476	NM_000308,NM_001127695,NM	CTSA	cathepsin A	1.282843931	1.171995446	65.30024	83.841623	71.631166	7.60667574	7.96439297	7.83455915	7613	13049	10974
27342	NM_001287060,NM_001287061	RABGEF1	RAB guanine nucleotide exchang	-1.536941255	1.171815554	13.882352	8.897892	15.59844	5.61883752	5.01441136	5.84328426	1890	1643	2724
83692	NM_001184808,NM_001242614	CD99L2	CD99 molecule like 2	1.563707941	1.171332379	3.141723	4.980275	3.464912	3.63059014	4.23306486	3.84165053	447	934	644
10984	NR_002728	KCNQ1OT1	KCNQ1 opposite strand/antisens	1.619650457	1.171264259	0.264133	0.428422	0.29078	4.61977956	5.29289816	4.83926507	926	2004	1334
91782	NM_001317899,NM_152272	CHMP7	charged multivesicular body prote	-1.087490003	1.171024905	10.78959	10.048054	10.702864	4.95797859	4.8410207	5.17897479	1181	1451	1701
56894	NM_001037553,NM_020132	AGPAT3	1-acylglycerol-3-phosphate O-ac	-1.162790798	1.170976013	13.788377	11.870696	15.17089	6.47577684	6.26081446	6.70112641	3455	3970	4976
151246	NM_001160033,NM_001160046	SGO2	shugoshin 2	1.329538317	1.170818202	10.626705	14.276552	11.791968	5.58325587	5.9867238	5.80638855	1843	3274	2654
22938	NM_001318844,NM_012245	SNW1	SNW domain containing 1	1.135071972	1.17069417	54.291733	61.845987	60.217228	6.83983616	7.02112453	7.06536813	4458	6761	6419
55719	NM_001136123,NM_001243770	SLF2	SMC5-SMC6 complex localizatio	-1.511849326	1.170570907	11.754984	8.273714	12.830656	6.05553602	5.47025157	6.27959322	2572	2273	3703
93517	NM_145168	SDR42E1	short chain dehydrogenase/redu	1.33093583	1.170465875	5.230202	6.967554	5.748833	3.9199564	4.30856691	4.13313083	555	987	799
8848	NM_001243797,NM_001243798	TSC22D1	TSC22 domain family member 1	2.655265013	1.170174268	12.404903	32.456272	13.62895	5.47918324	6.86779052	5.70120895	1712	6074	2464
57459	NM_020699	GATAD2B	GATA zinc finger domain contain	-1.037768424	1.170165095	6.048225	5.834952	6.650739	5.45843972	5.4061905	5.6803862	1687	2172	2428

9477	NM_001305455,NM_001305456	MED20	mediator complex subunit 20	-1.180713069	1.170162176	5.379971	4.478749	5.807659	3.76370129	3.54305919	3.9749255	494	559	711
8698	NM_003775	S1PR4	sphingosine-1-phosphate recepto	1.077414227	1.170030808	0.409077	0.430251	0.450153	0.71085839	0.75384785	0.80366934	25	36	36
8228	NM_001142389,NM_001172672	PNPLA4	patatin like phospholipase domai	1.540864202	1.169828615	4.36335	6.631639	4.639387	3.66286259	4.2461677	3.872576	458	943	659
56654	NM_015392	NPDC1	neural proliferation, differentiatior	-1.026864102	1.169571277	70.423691	68.664093	77.414055	6.72503406	6.68715411	6.94904089	4114	5353	5918
339324	NM_001012756,NM_001166036	ZNF260	zinc finger protein 260	-1.057182525	1.169527833	6.340978	5.945656	6.965262	5.09222606	5.01441136	5.31202633	1300	1643	1870
90196	NM_001099791,NM_001197129	SYS1	SYS1, golgi trafficking protein	1.053017649	1.169478487	10.920775	11.511907	12.03377	4.95324168	5.0254311	5.17236179	1177	1656	1693
5714	NM_002812	PSMD8	proteasome 26S subunit, non-AT	-1.164907044	1.169346638	82.000854	70.460976	90.104652	6.95271359	6.73441285	7.1767334	4824	5533	6938
100134869	NR_004847,NR_004847_dup1	UBE2Q2P2	ubiquitin conjugating enzyme E2	-1.247926633	1.16931915	0.975206	0.773099	1.061437	1.29387258	1.11294744	1.43197761	57	61	82
1915	NM_001402	EEF1A1	eukaryotic translation elongation	1.16214943	1.16927405	920.657349	1071.121582	1011.605225	11.62070125	11.83743308	11.84624821	123593	191912	177743
7398	NM_001017415,NM_001017416	USP1	ubiquitin specific peptidase 1	1.064780724	1.169143047	19.964788	21.286142	21.940924	6.27782897	6.36725676	6.50059565	3007	4278	4324
55112	NM_001350914,NM_001350915	WDR60	WD repeat domain 60	1.099758285	1.169109204	7.618632	8.244377	8.311927	4.87400371	5.00673269	5.09229974	1112	1634	1599
11039	NR_024054,NR_029426	SMA4	glucuronidase beta pseudogene	1.398223379	1.168993299	1.929581	2.722683	2.218315	2.16656821	2.55587612	2.34475282	137	256	197
369	NM_001256196,NM_001256197	ARAF	A-Raf proto-oncogene, serine/thr	1.17666967	1.168880417	12.407456	14.912556	13.422374	4.87775825	5.10510125	5.09579869	1115	1753	1603
51076	NM_015960	CUTC	cutC copper transporter	-1.841268412	1.16882188	9.956368	5.414108	10.938514	3.83495865	3.03667667	4.04537929	521	378	749
100533496	NM_001204478,NR_037924	TVP23C-CDRT4	TVP23C-CDRT4 readthrough	1.1585933	1.168715205	3.735546	4.331577	4.151812	3.53555296	3.7308426	3.74245532	416	644	598
153830	NM_001199380,NM_001199381	RNF145	ring finger protein 145	1.374318031	1.168665493	24.598661	33.870311	27.018012	6.45372678	6.90796502	6.67621822	3402	6247	4890
56906	NM_020147	THAP10	THAP domain containing 10	-1.181565698	1.168653242	2.246803	1.896858	2.461903	2.45494311	2.2611178	2.64141996	176	199	253
3105	NM_001242758,NM_002116	HLA-A	major histocompatibility complex,	-1.12048613	1.168636847	395.640266	353.497834	434.501037	9.27985092	9.11600498	9.50434361	24365	29054	35021
8887	NM_001079864,NM_001206901	TAX1BP1	Tax1 binding protein 1	-1.313661034	1.168572017	46.708047	35.570851	51.306689	7.27135934	6.88068447	7.49476209	6026	6129	8661
26049	NM_015566,NR_046462	FAM169A	family with sequence similarity 16	1.061393506	1.168511621	3.912097	4.159186	4.30072	4.56804599	4.65049289	4.78394543	892	1265	1282
10819	NR_045002	OR7E14P	olfactory receptor family 7 subfan	-1.439055442	1.168510825	6.292762	4.369627	6.911343	3.01906833	2.56983975	3.21791812	279	259	401
7320	NM_003337	UBE2B	ubiquitin conjugating enzyme E2	-1.089236885	1.168468972	19.674437	18.079409	21.604063	5.68533091	5.56450605	5.90591399	1981	2430	2847
1434	NM_001256135,NM_001316,NR	CSE1L	chromosome segregation 1 like	1.152688192	1.168466417	74.371308	85.774392	82.781223	8.03427774	8.23855288	8.25810262	10253	15791	14735
2323	NM_001204502,NM_001204503	FLT3LG	fms related tyrosine kinase 3 liga	-1.195932049	1.168217924	5.36497	4.466792	5.877753	2.73434891	2.51796519	2.92718897	222	248	319
100861541	NR_047482	DOCK9-AS2	DOCK9 antisense RNA 2 (head t	-1.85364595	1.16817149	0.439236	0.233237	0.480876	0.86002952	0.52452456	0.96541723	32	23	46
283209	NM_173582	PGM2L1	phosphoglucomutase 2 like 1	1.508334861	1.168104235	1.990488	3.006573	2.185773	4.12971807	4.69470593	4.34201172	648	1306	931
64397	NM_001284306,NM_001284307	ZNF106	zinc finger protein 106	-1.001846241	1.167979626	4.908964	4.903718	5.970912	5.72755378	5.72494281	5.94765889	2041	2722	2932
56850	NM_020137	GRIPAP1	GRIP1 associated protein 1	1.181753895	1.167810988	11.232662	13.295555	12.329981	5.09652857	5.33097613	5.31428079	1304	2059	1873
401647	NM_001010917	GOLGA7B	golgin A7 family member B	1.44896793	1.167804768	0.511014	0.741749	0.558783	2.07360076	2.49862948	2.2474368	126	244	181
54902	NM_001271420,NM_017775	TTC19	tetratricopeptide repeat domain 1	1.103498444	1.167665854	14.904915	16.315593	16.382064	5.70800345	5.84750424	5.92767386	2013	2968	2891
25844	NM_015388	YIPF3	Yip1 domain family member 3	1.158314161	1.167640833	26.636095	30.886635	29.233536	5.3882284	5.59555204	5.60688496	1605	2484	2305
100271722	NR_027036	LINC00899	long intergenic non-protein codin	2.157357645	1.167217362	1.254248	2.728026	1.380571	1.57857476	2.40307061	1.73094093	78	225	112
55764	NM_001280541,NM_001280545	IFT122	intraflagellar transport 122	1.651177949	1.167152503	6.980233	11.580566	7.61931	4.84104841	5.54461018	5.05683538	1086	2396	1559
23293	NM_001256827,NM_001256828	SMG6	SMG6, nonsense mediated mRN	1.228531445	1.166883486	3.520814	5.512429	4.094465	4.40935908	4.69364353	4.62230487	795	1305	1141
55544	NM_001291780,NM_017495,NM	RBM38	RNA binding motif protein 38	1.60012219	1.166633865	6.821076	11.101902	7.57652	4.09142887	4.73760421	4.30167645	630	1347	904
673	NM_004333	BRAF	B-Raf proto-oncogene, serine/thr	-1.013098621	1.16648367	2.442075	2.417774	2.680801	3.00540462	2.98897501	3.20177275	276	364	396
93594	NM_001145088,NM_001330606	TBC1D31	TBC1 domain family member 31	1.03741492	1.166358887	10.491973	10.852665	11.532788	5.17897521	5.23053515	5.39531188	1383	1917	1984
9679	NM_014661	FAM53B	family with sequence similarity 53	-1.219458263	1.166091692	2.284497	1.875741	2.506256	3.78518394	3.5216664	3.99192552	502	550	720
22905	NM_001102664,NM_014964,NM	EPN2	epsin 2	1.462242055	1.166039962	4.824846	7.577469	5.361902	4.52719419	5.05551521	4.73989992	866	1692	1242
3866	NM_002275	KRT15	keratin 15	-1.698032078	1.16584383	28.080111	16.551798	30.762726	5.68103985	4.9366195	5.8984202	1975	1554	2832
113622	NM_138430,NM_199162	ADPRHL1	ADP-ribosylhydrolase like 1	-1.128077622	1.165841524	1.044421	0.867401	1.14276	1.55375576	1.44132992	1.70368302	76	90	109
54872	NM_001127178,NM_001289051	PIGG	phosphatidylinositol glycan ancho	1.227084972	1.165674415	22.107221	27.240119	24.518459	6.31430441	6.60618982	6.5328977	3085	5058	4423
166378	NM_001317799,NM_001345856	SPATA5	spermatogenesis associated 5	-1.223016792	1.16556951	1.93674	1.563228	2.271818	4.01393646	3.74321825	4.22226663	595	650	853
6597	NM_001128844,NM_001128845	SMARCA4	SWI/SNF related, matrix associat	1.148034989	1.165261857	30.797865	35.391565	33.707353	7.32711689	7.52512768	7.54649995	6265	9610	8979
285343	NM_001029839,NM_001029840	TCAIM	T-cell activation inhibitor, mitocho	-1.131475317	1.16495104	3.702866	3.199986	4.130886	3.36414578	3.20419534	3.56449618	365	431	523
669	NM_001293085,NM_001724,NM	BPGM	bisphosphoglycerate mutase	-2.034628899	1.164811994	9.674358	4.759635	10.594118	4.15675275	3.21312006	4.36539258	661	434	947
5277	NM_002641,NM_020473,NR_03	PIGA	phosphatidylinositol glycan ancho	1.005528746	1.164705326	2.83024	3.000565	3.428465	3.46076162	3.46799726	3.66213029	393	528	563
101410538	NR_102705,NR_102706	MMP24-AS1	MMP24 antisense RNA 1	-1.171997294	1.164560661	15.827079	13.612434	17.493075	4.80338004	4.58324605	5.01586734	1057	1205	1514
197258	NM_145059	FUK	fucokinase	-1.02786266	1.164511781	6.669814	6.49974	7.304206	4.72217607	4.68404654	4.93418107	997	1296	1428
4076	NM_005898,NM_203364	CAPRIN1	cell cycle associated protein 1	-1.037185829	1.164279667	58.564724	56.788052	67.263287	8.24431706	8.19181908	8.46308516	11866	15286	16992
10194	NM_001308210,NM_005786	TSHZ1	teashirt zinc finger homeobox 1	-1.493005826	1.164038949	2.839234	1.939715	3.106595	3.88804577	3.35695728	4.09342686	542	485	776
2531	NM_002035	KDSR	3-ketodihydrosphingosine reducta	1.006477083	1.164033148	6.886919	6.941969	7.535287	5.1647037	5.17375987	5.37817319	1369	1841	1960
79983	NM_001307940,NM_024921	POF1B	premature ovarian failure, 1B	-1.227880561	1.163991594	7.386548	6.171207	8.26717	4.87400371	4.58897123	5.08615607	1112	1210	1592
4218	NM_005370	RAB8A	RAB8A, member RAS oncogene	-1.006318182	1.163988257	46.485447	46.250797	50.850853	6.63731632	6.6283211	6.85435545	3869	5137	5539
26507	NM_001345887,NM_001345888	CNNM1	cyclin and CBS domain divalent r	-1.016254045	1.163975064	2.430226	2.375246	2.686719	3.92238208	3.90066256	4.1280136	556	731	796
284677	NR_147122,NR_147123,NR_147	SNHG28	small nucleolar RNA host gene 2	-1.175536684	1.163932218	0.978219	0.832089	1.118262	1.69664266	1.5392062	1.85174813	88	100	126
10540	NM_001261412,NM_001261413	DCTN2	dynactin subunit 2	-1.172258456	1.163777977	39.067087	33.378019	42.719817	6.34627164	6.12002389	6.56259705	3155	3596	4516
5286	NM_001321378,NM_001321380	PIK3C2A	phosphatidylinositol-4-phosphate	-1.298544664	1.163755488	13.075257	10.079693	14.299219	6.76037615	6.38743716	6.97729571	4217	4339	6036
81628	NM_001303043,NM_030935,NR	TSC22D4	TSC22 domain family member 4	-1.41436449	1.163459318	15.87148	11.602535	17.498623	5.15028961	4.666826	5.36300797	1355	1280	1939
26099	NM_001114600,NM_001271869	SZRD1	SUZ RNA binding domain contair	1.154842254	1.163305903	22.946063	26.519026	25.128607	6.37001279	6.57537437	6.58579963	3208	4950	4590
25963	NM_001110503,NM_001286487	TMEM87A	transmembrane protein 87A	1.003153661	1.163115437	38.123333	38.346594	41.619258	6.86022227	6.86472595	7.07647903	4522	6061	6469
196074	NM_001113528,NM_001297775	METTL15	methyltransferase like 15	-1.284384139	1.163037111	4.926086	3.829022	5.386741	4.45195674	4.10945474	4.66061022	820	853	1173
10509	NM_001324029,NM_001324030	SEMA4B	semaphorin 4B	1.785370587	1.162826768	61.971817	111.385386	68.257591	7.84890848	8.6823842	8.06567065	9012	21498	12889
55629	NM_017761	PNRC2	proline rich nuclear receptor coac	-1.06185767	1.162798	47.405899	44.696484	51.800659	6.81296147	6.7271624	7.02876954	4375	5505	6257
8678	NM_001313998,NM_001313999	BECN1	beclin 1	1.159529234	1.162742042	27.538206	31.873614	29.95533	5.85428279	6.06439629	6.06832921	2232	3458	3192
8028	NM_001195626,NM_001195627	MLLT10	myeloid/lymphoid or mixed-lineag	1.105315412	1.162580154	11.467252	11.865394	10.091732	5.3820734	5.52324054	5.59457146	1598	2360	2285
3329	NM_002156,NM_199440	HSPD1	heat shock protein family D (Hsp	1.565487518	1.162525872	245.139865	384.188444	267.801543	9.11620269	9.76187788	9.33310306	21748	45490	31096
55610	NM_001257998,NM_017667,NM	VPS50	VPS50, EARP/GARPII complex s	-1.355691547	1.162515746	4.820767	3.304594	5.147612	4.57731129	4.15956116	4.7861128	898	885	1284
27018	NM_001282674,NM_014380,NM	BEX3	brain expressed X-linked 3	1.670161523	1.162293338	62.665228	105.39471	68.749612	5.99805187	6.72897843	6.21187981	2470	5512	3531
90313	NM_001346077,NM_001346078	TP53I13	tumor protein p53 inducible prote	-1.161303372	1.162292505	35.926733	31.041089	39.409591	5.82021839	5.60857511	6.03363206	2179	2507	3115
55501	NM_001243794,NM_001243795	CHST12	carbohydrate sulfotransferase 12	-1.023458709	1.162289491	5.193207	5.059097	5.649198	3.54502861	3.51446439	3.74468587	419	547	599
4140	NM_001128918,NM_001128919	MARK3	microtubule affinity regulating kin	1.208359126	1.162279337	37.161604	44.944391	40.60854	6.97596077	7.24703796	7.19132117	4903	7916	7009
55662	NM_017902	HIF1AN	hypoxia inducible factor 1 alpha s	-1.046831023	1.16225441	8.077215	7.725646	8.824893	5.79131439	5.72650194	6.00460939	2135	2725	3052
26230	NM_001010927,NM_012454	TIAM2	T-cell lymphoma invasion and me	-1.335766731	1.16222694	0.596505	0.444888	0.649493	2.15010456	1.83736459	2.32103228	135	135	193
5074	NM_002583	PAWR	pro-apoptotic WT1 regulator	-1.23344954	1.162185157	20.136845	16.341066	21.990866	5.29490023	5.00073201	5.50661727	1502	1627	2147
9824	NM_001286479,NM_001286480	ARHGAP11A	Rho GTPase activating protein 1	-1.198224343	1.162087579	27.008472	21.60827	31.361554	7.09228517	6.83347566	7.30753276	5318	5930	7601
4238	NM_001135037,NM_001242336	MFAP3	microfibrillar associated protein 3	1.530598409	1.161801937	7.681641	11.782224	8.399674	5.27889898	5.88009069	5.49009354	1485	3037	2122
89887	NM_033113	ZNF628	zinc finger protein 628	1.237396148	1.161779736	2.388335	2.960408	2.610528	3.31698997	3.59633059	3.51308411	352	582	503
79033	NM_001301698,NM_001301699	ERI3	ERI1 exoribonuclease family mer	1.345559332	1.161676556	19.691267	26.004263	21.30235	5.00565571	5.42231408	5.21561844	1222	2197	1746
201266	NM_001159770,NM_139177	SLC39A11	solute carrier family 39 member 1	-1.48190526	1.161529009	33.423401	22.602579	36.507903	6.50924151	5.94937984	6.7230688	3537	3189	5053
253943	NM_001277813,NM_001277814	YTHDF3	YTH N6-methyladenosine RNA b	-1.015452395	1.161453548	14.676262	14.20516	16.099029	6.1823895	6.16057299	6.39556477	2812	3700	4017
222643	NM_173561	UNC5CL	unc-5 family C-terminal like	-2.093633815	1.161302567	0.753107	0.358371	0.830253	1.73025772	1.07428646	1.88448459	91	58	130
9778	NM_001100590,NM_014743	KIAA0232	KIAA0232	-1.149103101	1.161220908	8.577123	7.476242	9.363657	6.04332975	5.8460706	6.2559406	2550	2965	3642
6924	NM_003198	ELOA	elongin A	1.346026104	1.161216678	11.131367	15.003017	12.14743	5.7705968	6.19251204	5.98257082	2104	3784	3005
147011	NM_001304949,NM_001304951	PROCA1	protein interacting with cyclin A1	-2.349394622	1.161030401	4.478867	1.862413	4.648495	3.04601354	2.03149389	3.23705705	285	162	407
100507144	NR_120528	LOC100507144	uncharacterized LOC100507144	-1.695995864	1.160857224	0.652821	0.378508	0.721713	0.77668602	0.50527715	0.87055986	28	22	40
91746	NM_001031732,NM_001330698	YTHDC1	YTH domain containing 1	1.160952032	1.160749583	22.712457	26.353725	24.79331	7.12266297	7.33653931	7.33628944	5432	8426	7755
23207	NM_015164	PLEKHM2	pleckstrin homology and RUN do	1.108203122	1.160729241	14.366329	15.941117	15.673047	5.88445448	6.03029685	6.09610992	2280	3376	3255
158067	NM_001317958,NM_001317959	AK8	adenylate kinase 8	1.813481059	1.160643676	0.227279	0.473669	0.231604	0.61813834	0.97983233	0.69713258	21	51	30
3297	NM_005526	HSF1	heat shock transcription factor 1	-1.032612782	1.160501664	43.540253	42.204979	47.479263	6.54234181	6.49654578	6.75495417	3620	4684	5167
1477	NM_001033521,NM_001033522	CSTF1	cleavage stimulation factor subur	1.19473662	1.160358784	19.832624	23.663804	21.599099	5.50530701	5.75682736	5.71549438	1744	2784	2489
51310	NM_001289050,NM_016609,NM	SLC22A17	solute carrier family 22 member 1	10.98977682	1.160218275	0.152353	1.818196	0.165505	0.43999062	2.30630165	0.49997203	14	207	20
9931	NM_001330447,NM_014877	HELZ	helicase with zinc finger	1.123809594	1.160185707	4.868504	5.476915	5.306333	6.05664057	6.22265489	6.26800924	2574	3865	3673
27289	NM_014470	RND1	Rho family GTPase 1	1.023521072	1.160148562	2.904631	2.970345	3.159623	2.51403512	2.541776	2.69315397	185	253	264
1729	NM_001079812,NM_001314007	DIAPH1	diaphanous related formin 1	1.139362261	1.159924035	55.919672	63.766667	60.935268	8.3056456	8.49331582	8.51904893	12383	18851	17666
4092	NM_001190821,NM_001190822	SMAD7	SMAD family member 7	-1.026412617	1.159922069	2.12397	1.935443	2.265717	2.78853894	2.75641772	2.973571	232	302	331
5523	NM_001190447,NM_002718,NM	PPP2R3A	protein phosphatase 2 regulatory	2.069772085	1.159895229	3.376272	7.293876	3.75203	4.54776468	5.56508709	4.75325534	879	2431	1254
84874	NM_001318005,NM_032788	ZNF514	zinc finger protein 514	1.006236732	1.159882205	8.402276	8.5267	9.121658	5.66157057	5.67036415	5.87162597	1948	2619	2779
64762	NM_001242409,NM_022751	GAREM1	GRB2 associated regulator of MA	-1.085253937	1.159872962	0.831802	0.767601	0.909592	2.75082071	2.65089709	2.9350236	225	277	321

165324	NM_181713	UBXN2A	UBX domain protein 2A	1.088742383	1.159714296	7.312017	7.974983	7.981171	3.85794988	3.97250183	4.05799054	530	771	756
605	NM_001024808,NM_020993	BCL7A	BCL tumor suppressor 7A	-2.103403243	1.159635943	3.987106	1.88988	4.352958	3.95592014	2.98202989	4.15677414	570	362	813
403150	NR_103831	FLJ31356	uncharacterized protein FLJ3135	1.251040143	1.159304407	0.558852	0.699284	0.605585	1.35261029	1.55800914	1.4863075	61	102	87
84836	NM_001146314,NM_001254753	ABHD14B	abhydrolase domain containing 1	-1.857788738	1.159199724	13.109911	6.805284	13.873068	4.88150305	4.02918937	5.08791408	1118	804	1594
85456	NM_033396	TNKS1BP1	tankyrase 1 binding protein 1	1.36198128	1.1589813	38.358055	52.303635	41.776646	7.77063374	8.21458845	7.98258708	8534	15530	12165
25992	NM_001080437	SNED1	sushi, nidogen and EGF like dom	6.228303691	1.158955485	0.024219	0.166512	0.028705	0.23670415	1.08728891	0.27153692	7	59	10
100616459	NR_039900	MIR4745	microRNA 4745	2.015933494	1.158955485	2.832537	5.819133	3.013242	0.23670415	0.44594451	0.27153692	7	19	10
102466749	NR_106908	MIR6849	microRNA 6849	-4.43112822	1.158955485	2.567701	0.531598	2.862432	0.23670415	0.05398282	0.27153692	7	2	10
56129	NM_018940	PCDHB7	protocadherin beta 7	1.068150199	1.158955485	0.047455	0.047369	0.049392	0.23670415	0.25173066	0.27153692	7	10	10
3758	NM_000220,NM_153764,NM_15	KCNJ1	potassium voltage-gated channel	-1.166137663	1.158955485	0.066471	0.056779	0.072553	0.23670415	0.20477631	0.27153692	7	8	10
102465464	NR_106833	MIR6775	microRNA 6775	-1.03859314	1.158955485	2.316187	2.427349	2.727628	0.23670415	0.2284445	0.27153692	7	9	10
5818	NM_002855,NM_203285,NM_20	NECTIN1	nectin cell adhesion molecule 1	1.120092642	1.158696473	13.147436	13.982624	14.993857	6.01007555	6.17129809	6.21951784	2491	3728	3550
11142	NM_001281444,NM_001281445	PKIG	cAMP-dependent protein kinase	1.103894485	1.158523486	5.238417	5.794576	5.548627	2.83047153	2.95391015	3.01481644	240	354	342
200316	NM_001006666,NM_145298	APOBEC3F	apolipoprotein B mRNA editing e	1.363526898	1.158404946	8.992168	11.845693	8.169648	4.47030848	4.90024229	4.67354624	831	1514	1184
55727	NM_001002860,NM_001289133	BTBD7	BTB domain containing 7	1.018389478	1.158169728	9.133104	9.177866	9.622397	5.78932242	5.81514199	5.99761231	2132	2901	3037
4664	NM_001321312,NM_001321313	NAB1	NGFI-A binding protein 1	1.058689073	1.158125209	19.266531	21.682245	20.876862	6.33766462	6.41895706	6.54702524	3136	4436	4467
9175	NM_001242314,NM_001242317	MAP3K13	mitogen-activated protein kinase	-1.35568154	1.158089191	27.619421	20.871907	24.04288	5.92976705	5.49911925	6.13828834	2354	2320	3353
4053	NM_000428	LTBP2	latent transforming growth factor	2.232311993	1.158071435	7.783243	17.392767	8.468499	6.04054113	7.18696286	6.2492792	2545	7591	3625
1453	NM_001893,NM_139062,NR_11	CSNK1D	casein kinase 1 delta	1.014366329	1.157694485	63.754764	64.805452	69.359428	7.87199396	7.89248574	8.08241183	9158	12412	13040
57449	NM_001042663,NM_001042664	PLEKHG5	pleckstrin homology and RhoGEI	-1.401306989	1.157653784	5.45807	4.076705	6.151351	4.76875136	4.30300447	4.97275069	1031	983	1468
166	NM_001130,NM_198969,NM_19	AES	amino-terminal enhancer of split	-1.13208874	1.157624711	129.739297	114.588476	141.095077	7.73657926	7.55848301	7.94682816	8334	9836	11866
27152	NM_015693	INTU	inturned planar cell polarity prote	1.190249251	1.157514113	2.822204	3.364557	3.074425	3.32434579	3.55246622	3.51569869	354	563	504
6418	NM_001122821,NM_001248000	SET	SET nuclear proto-oncogene	1.121218768	1.157318328	191.633443	214.195349	207.319651	9.07693597	9.24171571	9.28735957	21163	31704	30124
83715	NM_031475	ESPN	espin	-4.039135846	1.157310836	4.543018	1.124659	4.942153	4.05651939	2.28388661	4.25549579	614	203	874
90871	NM_001318058,NM_001318059	TMEM261	transmembrane protein 261	1.02291636	1.157234449	8.884974	9.111976	9.679775	4.49821892	4.52948015	4.70022518	848	1159	1207
5445	NM_000305,NM_001018161	PON2	paraoxonase 2	1.03755154	1.157229412	54.592781	56.657754	59.233694	6.46541785	6.51801145	6.67387916	3430	4755	4882
5971	NM_006509	RELB	RELB proto-oncogene, NF-kB su	1.292420214	1.157184939	10.558265	13.666914	11.490727	4.60927912	4.96595521	4.81187039	919	1587	1308
729096	NR_026592	BMS1P4	BMS1, ribosome biogenesis facto	-1.350109307	1.156972445	3.808866	2.826653	4.149194	3.05045588	2.67693481	3.23705705	286	283	407
3032	NM_000183,NM_001281512,NM	HADHB	hydroxyacyl-CoA dehydrogenase	1.076108089	1.156941455	42.676296	45.952481	46.436474	6.52249295	6.62720865	6.73068453	3570	5133	5080
8520	NM_003642,NR_027862	HAT1	histone acetyltransferase 1	1.590939072	1.156816238	35.502868	56.513146	38.125055	5.85618712	6.51681057	6.06297645	2235	4751	3180
6731	NM_001267722,NM_006947	SRP72	signal recognition particle 72	1.008630578	1.156782077	25.982987	25.864577	28.080171	6.60936173	6.6216335	6.81748053	3794	5113	5398
10006	NM_001012750,NM_001012751	ABI1	abl interactor 1	-1.212236875	1.156557168	12.797393	10.571708	14.008097	5.50368806	5.23273108	5.70922621	1742	1920	2478
11183	NM_006575,NM_198794	MAP4K5	mitogen-activated protein kinase	1.089926916	1.156362213	18.656206	20.400588	20.32542	6.34174803	6.46451508	6.54894104	3145	4580	4473
692225	NR_004378	SNORD94	small nucleolar RNA, C/D box 94	1.265488634	1.156359761	4.836348	6.208998	5.343398	0.73313634	0.8787583	0.82068496	26	44	37
109617010	NR_145765	SNORA77B	small nucleolar RNA, H/ACA box	1.408828355	1.156359761	5.327576	7.660546	5.86903	0.73313634	0.95166931	0.82068496	26	49	37
116840	NM_001037144,NM_001330124	CNTROB	centrobin, centriole duplication ar	1.052748535	1.15621815	12.369133	13.026117	13.441508	5.65429253	5.72702128	5.85984164	1938	2726	2756
22904	NM_001100122,NM_014963	SBNO2	strawberry notch homolog 2	1.066877558	1.156155981	35.102711	37.588177	38.168634	7.40376665	7.49662857	7.61195484	6609	9421	9398
22954	NM_001099679,NM_012210	TRIM32	tripartite motif containing 32	1.534895403	1.156005318	7.599744	11.686697	8.261841	4.83462363	5.43508445	5.03695187	1081	2217	1537
4215	NM_001330431,NM_002401,NM	MAP3K3	mitogen-activated protein kinase	-1.107085096	1.155765316	8.398934	7.651625	9.251436	5.33186457	5.1889187	5.53589126	1542	1861	2192
9742	NM_014714	IFT140	intraflagellar transport 140	1.042859025	1.155670501	4.959878	5.178673	5.384887	4.72495781	4.78326433	4.92634185	999	1392	1420
4794	NM_004556	NFKBIE	NFKB inhibitor epsilon	1.251754721	1.15556108	3.993122	5.004191	4.33333	3.46409533	3.76158491	3.65503258	394	659	560
55157	NM_018122	DARS2	aspartyl-tRNA synthetase 2, mito	1.015078743	1.155520115	24.298912	24.693232	26.384069	6.32580276	6.34712794	6.5319294	3110	4218	4420
84219	NM_032259	WDR24	WD repeat domain 24	1.087447989	1.155478395	6.041746	6.575946	6.557335	4.33847337	4.45368941	4.53736359	755	1097	1073
11343	NM_001003794,NM_001256585	MGLL	monoglyceride lipase	-1.033553722	1.155320388	15.987585	16.374184	17.913316	6.16815189	6.12120968	6.37375294	2784	3599	3956
23469	NM_001290259,NM_001290260	PHF3	PHD finger protein 3	-1.163955285	1.155311498	8.705454	7.331735	9.371419	5.99286804	5.77753174	6.19811001	2461	2825	3497
3300	NM_001039550,NM_006736	DNAJB2	DnaJ heat shock protein family (I	1.362226954	1.155250855	11.618953	15.094868	12.599894	4.80994515	5.24220835	5.01124268	1062	1933	1509
125144	NR_027158,NR_027159,NR_02	LRRC75A-AS1	LRRC75A antisense RNA 1	-1.039104092	1.155234229	101.430949	99.917906	115.790036	6.87281885	6.81795863	7.0793539	4562	5866	6482
4316	NM_002423	MMP7	matrix metallopeptidase 7	-1.584902959	1.155083099	1.306855	0.834265	1.426841	1.29387258	0.93737891	1.42086173	57	48	81
58513	NM_001258374,NM_001258375	EPS15L1	epidermal growth factor receptor	1.19973861	1.155007684	8.891222	10.580209	9.527509	4.80469545	5.05881949	5.00567345	1058	1696	1503
10678	NM_001319075,NM_006577	B3GNT2	UDP-GlcNAc:betaGal beta-1,3-N	1.202310797	1.154881403	12.374379	14.893528	13.433185	5.09330289	5.35200352	5.29538508	1301	2090	1848
170958	NM_001348156,NM_001348157	ZNF525	zinc finger protein 525	-14.40090659	1.154787737	1.947912	0.174664	2.144791	3.16959281	0.63490936	3.35562963	314	29	446
5446	NM_000940	PON3	paraoxonase 3	-1.541865673	1.154732511	3.441346	2.229749	3.730674	2.32162912	1.84504029	2.4901017	157	136	223
8237	NM_004651	USP11	ubiquitin specific peptidase 11	1.125107089	1.154609491	13.281795	14.961388	14.414868	5.44500562	5.61139072	5.64798177	1671	2512	2373
27347	NM_013233	STK39	serine/threonine kinase 39	-1.07853116	1.154470452	23.245937	21.581287	25.226397	6.23114291	6.12357834	6.43580955	2910	3605	4132
81579	NM_030821	PLA2G12A	phospholipase A2 group XIIA	1.018548795	1.154091088	6.824044	6.959635	7.403334	5.16162707	5.18740997	5.36300797	1366	1859	1939
4780	NM_001145412,NM_001145413	NFE2L2	nuclear factor, erythroid 2 like 2	1.173959218	1.1540348	29.450605	34.802654	32.034307	6.38328002	6.61210602	6.58766467	3238	5079	4596
55837	NM_001318916,NM_018453	EAPP	E2F associated phosphoprotein	1.349294561	1.153992264	21.745459	29.435455	23.820391	4.8790076	5.29849962	5.07910255	1116	2012	1584
3839	NM_002267	KPNA3	karyopherin subunit alpha 3	1.259253472	1.153932205	5.396892	6.800731	5.848265	4.61528873	4.93572116	4.81399624	923	1553	1310
93627	NM_001163435,NM_001163436	TBCK	TBC1 domain containing kinase	-1.418870974	1.153824866	3.294439	2.301739	3.59213	4.17110273	3.69943092	4.36684138	668	629	948
9130	NM_004699	FAM50A	family with sequence similarity 5(	1.035612512	1.153640689	76.938889	79.774727	83.425926	6.67664254	6.72664312	6.88096245	3977	5503	5643
56992	NM_020242	KIF15	kinesin family member 15	1.039590981	1.153306287	6.600309	6.871603	7.154868	5.00451141	5.05881949	5.20431761	1221	1696	1732
6839	NM_001282166,NM_003173	SUV39H1	suppressor of variegation 3-9 hor	1.260056892	1.153101783	4.98499	6.860028	5.691207	3.95355025	4.26774474	4.1466887	569	958	807
85364	NM_033089	ZCCHC3	zinc finger CCHC-type containing	1.521603972	1.15306685	9.155976	13.942562	9.921711	4.67835874	5.26455989	4.87635737	966	1964	1370
59339	NM_021623	PLEKHA2	pleckstrin homology domain cont	1.192637136	1.152985482	13.167033	15.722397	14.266515	6.19598682	6.44696861	6.39875517	2839	4524	4026
8555	NM_001077181,NM_001351567	CDC14B	cell division cycle 14B	-1.006909122	1.152785555	9.197721	9.092633	10.222912	5.6120916	5.60236135	5.81331234	1881	2496	2667
57217	NM_001288951,NM_001288953	TTC7A	tetratricopeptide repeat domain 7	1.032092546	1.152732269	14.233547	14.760217	15.458864	6.18137717	6.22633294	6.38380589	2810	3875	3984
51160	NM_016208,NM_183057	VPS28	VPS28, ESCRT-I subunit	-1.31120167	1.152724267	54.954446	41.583462	59.905428	5.80715195	5.42423685	6.0087914	2159	2200	3061
4241	NM_005929,NM_033316	MELTF	melanotransferrin	-2.010242648	1.152624726	107.923328	55.295459	119.073635	8.41927897	7.41614888	8.6236453	13401	8907	18998
54856	NM_001282856,NM_001282858	GON4L	gon-4 like	-1.027288176	1.15258197	9.572159	9.106384	10.150648	5.94833435	5.91012909	6.15011624	2385	3102	3381
23516	NM_001128431,NM_001135153	SLC39A14	solute carrier family 39 member 1	1.352736462	1.152557169	15.128675	20.298309	16.345985	6.14652852	6.57710371	6.34867642	2742	4956	3887
9236	NM_001204450,NM_001204451	CCPG1	cell cycle progression 1	-1.627921618	1.152492109	5.289204	4.030958	6.262588	4.96623093	4.29181484	5.16488576	1188	975	1684
56478	NM_001164501,NM_001164502	EIF4ENIF1	eukaryotic translation initiation fa	1.076564577	1.152406856	4.755773	5.101812	5.104318	4.16701728	4.26774474	4.36103743	666	958	944
6780	NM_001037328,NM_001319134	STAU1	staufen double-stranded RNA bir	1.021109212	1.152094571	42.226686	43.909016	46.570935	7.18080485	7.21073702	7.38375446	5657	7718	8016
283237	NM_001318812,NM_001318813	TTC9C	tetratricopeptide repeat domain 9	1.298351251	1.152011356	14.847856	19.193035	16.466957	4.08278037	4.43981671	4.27568916	626	1086	887
119504	NM_001242546,NM_001242547	ANAPC16	anaphase promoting complex sul	-1.426709771	1.151972031	20.868971	14.476631	22.308055	6.06982971	5.56624847	6.27110741	2598	2433	3681
2119	NM_004454	ETV5	ETS variant 5	1.781310781	1.151956185	8.858071	15.795513	9.585985	5.18504861	6.00053598	5.38390874	1389	3306	1968
5434	NM_001316323,NM_001316324	POLR2E	RNA polymerase II subunit E	1.165660052	1.151817222	31.808843	37.128986	34.552263	6.48606198	6.70492623	6.68785694	3480	5420	4930
345778	NM_001010891,NM_001167741	MTX3	metaxin 3	1.116461582	1.151764816	8.87344	9.917689	9.603894	6.10442241	6.26117308	6.30551091	2662	3971	3771
8476	NM_003607,NM_014826	CDC42BPA	CDC42 binding protein kinase alp	-1.355557708	1.151643849	7.80746	5.799928	8.450048	6.33766462	5.90508943	6.5390152	3136	3091	4442
6676	NM_001317931,NM_003116	SPAG4	sperm associated antigen 4	-1.258388987	1.151604245	6.961303	5.482528	7.402586	3.44734922	3.14945559	3.6335275	389	413	551
51029	NM_001297746,NM_016076,NR	DESI2	desumoylating isopeptidase 2	1.021535045	1.151518497	19.03331	19.414536	20.605796	6.25395271	6.28429393	6.45500789	2957	4036	4188
9107	NM_004685	MTMR6	myotubularin related protein 6	1.067267988	1.151463993	3.082934	3.296305	3.339007	4.05872616	4.14719676	4.25079537	615	877	871
23118	NM_001292034,NM_001292035	TAB2	TGF-beta activated kinase 1/MAI	1.178953336	1.151426862	13.369981	15.596071	14.29403	5.76790176	6.00139486	5.96784829	2100	3308	2974
10801	NM_001113491,NM_001113492	SEPT9	septin 9	1.059605896	1.15137361	151.987937	161.098095	166.865625	9.17777633	9.26116435	9.38080577	22698	32135	32143
283248	NM_173587	RCOR2	REST corepressor 2	1.039375241	1.151350778	0.359389	0.375422	0.385319	0.93882015	0.96581954	1.03996283	36	50	51
8933	NM_001078171	RTL8C	retrotransposon Gag like 8C	1.400278579	1.151345636	21.679537	30.404423	23.474903	4.76875136	5.2392989	4.96511882	1031	1929	1460
7565	NM_001330617,NM_006959	ZNF17	zinc finger protein 17	-1.07006626	1.151132639	8.696162	8.193837	9.458143	4.59414465	4.50061221	4.78935776	909	1135	1287
6773	NM_005419,NM_198332	STAT2	signal transducer and activator of	-1.392385349	1.151125517	16.375613	11.77196	17.714677	6.19899112	5.72909676	6.39946319	2845	2730	4028
1417	NM_004076	CRYBB3	crystallin beta B3	-1.14450978	1.151116206	0.710757	0.625501	0.766683	0.68823103	0.61708849	0.76902392	24	28	34
2487	NM_001463	FRZB	frizzled-related protein	1.401524212	1.151116206	0.226633	0.317801	0.243806	0.68823103	0.89363826	0.76902392	24	45	34
55109	NM_018046	AGGF1	angiogenic factor with G-patch ar	1.076957572	1.151024429	9.980642	10.760036	10.795888	5.48575866	5.59042388	5.68445867	1720	2475	2435
8925	NM_003922	HERC1	HECT and RLD domain containir	-1.006982908	1.151011043	2.480191	2.465171	2.682634	5.23664058	5.22686782	5.43452772	1441	1912	2040
51386	NM_001242923,NM_016091	EIF3L	eukaryotic translation initiation fa	1.104697285	1.150892672	126.999161	140.445816	137.361874	8.01990879	8.16303444	8.22193525	10151	14983	14369
84928	NM_001301163,NM_032842	TMEM209	transmembrane protein 209	-1.194540456	1.15081845	11.129911	9.388779	12.01563	5.29490023	5.04555672	5.49275009	1502	1680	2126
4835	NM_000904,NM_001290221,NM	NQO2	N-ribosyldihydronicotinamide:quir	1.005707667	1.15079465	11.326009	11.393039	12.193829	3.89300127	3.90066256	4.08288706	544	731	770
80185	NM_001102401,NM_001265581	TTI2	TELO2 interacting protein 2	1.048163345	1.150615318	4.217843	4.351048	4.612907	3.50674676	3.56878235	3.6924898	407	570	576
79635	NM_001142683,NM_024584	CCDC121	coiled-coil domain containing 121	1.427079503	1.150520125	0.608731	0.911797	0.774499	1.40904982	1.75012126	1.53866543	65	124	92
9943	NM_005109	OXSR1	oxidative stress responsive 1	1.054757674	1.150507891	20.052198	21.17083	21.678364	6.48072286	6.55679785	6.68088498	3467	4886	4906
10109	NM_005731,NM_152862	ARPC2	actin related protein 2/3 complex	1.241195989	1.150478783	197.864833	246.361487	214.480045	8.18178466	8.49255243	8.3833709	11361	18841	16076

8804	NM_003851	CREG1	cellular repressor of E1A stimulat	-1.04788114	1.150468882	24.08935	23.018402	26.046949	5.60002041	5.53396493	5.79835805	1865	2378	2639
8294	NM_003495	HIST1H4I	histone cluster 1 H4 family memb	-1.06824733	1.150416833	0.80647	0.785743	0.865427	0.38477185	0.36283659	0.43515728	12	15	17
115273	NM_001193532,NM_152304	RAB42	RAB42, member RAS oncogene	-1.986550784	1.150416833	0.252871	0.134429	0.275657	0.38477185	0.20477631	0.43515728	12	8	17
100128750	NR_046205	RBPMS-AS1	RBPMS antisense RNA 1	-1.332164478	1.150416833	0.321125	0.256846	0.355025	0.38477185	0.29720487	0.43515728	12	12	17
4233	NM_000245,NM_001127500,NM	MET	MET proto-oncogene, receptor ty	-1.61823992	1.15017082	74.616481	45.080022	79.78565	8.86521261	8.17269327	9.06665792	18269	15084	25844
79862	NM_001142572,NM_024804	ZNF669	zinc finger protein 669	-1.724570582	1.150066984	2.42587	1.384434	2.61612	2.37218925	1.77444288	2.53718654	164	127	232
645	NM_000713	BLVRB	biliverdin reductase B	-1.992174289	1.149937259	122.209473	61.393482	132.06752	6.68452367	5.70399173	6.88425408	3999	2682	5656
27183	NM_013245	VPS4A	vacuolar protein sorting 4 homolo	-1.052962793	1.149929773	30.66828	29.155287	33.141079	6.04388682	5.97058687	6.2425869	2551	3237	3608
10553	NM_001098520,NM_001098521	HTATIP2	HIV-1 Tat interactive protein 2	-1.329317532	1.149873839	91.813744	74.852321	102.570602	7.02841505	6.62135418	7.22845362	5086	5112	7193
55669	NM_033540	MFN1	mitofusin 1	1.175410564	1.149840922	16.104816	18.951857	17.400984	5.80977473	6.0391067	6.00786311	2163	3397	3059
677769	NR_003003	SCARNA17	small Cajal body-specific RNA 17	-1.095347668	1.149760065	2.536083	2.319685	2.735865	1.03161781	0.96581954	1.13825079	41	50	58
79834	NM_024776	PEAK1	pseudopodium enriched atypical	1.037130416	1.149722075	2.731737	2.835907	2.949998	5.01931705	5.07032524	5.21481417	1234	1710	1745
1803	NM_001935	DPP4	dipeptidyl peptidase 4	1.987188051	1.149646606	0.469495	0.930915	0.506354	1.47662927	2.18453503	1.60891398	70	186	99
375035	NM_199344	SFT2D2	SFT2 domain containing 2	1.224468275	1.149599542	3.024149	3.70285	3.263675	1.81624731	2.03149389	1.96321628	99	162	140
58472	NM_001271213,NM_021199	SQOR	sulfide quinone oxidoreductase	-4.460961274	1.149518821	26.600241	5.965615	28.769336	5.72477705	3.65863507	5.92226458	2037	610	2880
90324	NM_001346100,NM_052848	CCDC97	coiled-coil domain containing 97	1.147778424	1.149496739	7.996511	8.819963	8.567883	4.76334931	4.95535656	4.95744636	1027	1575	1452
9897	NM_001330609,NM_014846	WASHC5	WASH complex subunit 5	1.248209568	1.149420507	15.629953	18.964388	16.584751	5.96018767	6.27544509	6.15808745	2405	4011	3400
79095	NM_024112	C9orf16	chromosome 9 open reading fran	1.110939	1.149199307	70.347923	78.250015	75.984711	5.63962535	5.78852012	5.83650316	1918	2847	2711
9870	NM_001039479	AREL1	apoptosis resistant E3 ubiquitin p	1.084613511	1.149195317	14.651301	15.911374	15.824492	6.29805119	6.4138051	6.49629974	3050	4420	4311
7410	NM_001134398,NM_003371	VAV2	vav guanine nucleotide exchange	1.306849108	1.149192971	11.423681	14.94863	12.333077	5.74615826	6.12594312	5.94329559	2068	3611	2923
23001	NM_014991	WDFY3	WD repeat and FYVE domain co	1.101554227	1.149128758	5.250361	5.791352	5.670053	6.21342436	6.35117624	6.41144669	2874	4230	4062
27068	NM_006903,NM_176866,NM_17	PPA2	pyrophosphatase (inorganic) 2	1.14566243	1.149099102	34.168248	39.065941	36.756231	5.79859486	5.99148667	5.99574068	2146	3285	3033
22876	NM_001243194,NM_001243195	INPP5F	inositol polyphosphate-5-phosph	-1.243654138	1.149061887	4.780038	3.648721	4.846428	4.36010717	4.06249189	4.55144767	767	824	1084
302	NM_001002857,NM_001002858	ANXA2	annexin A2	-1.110270467	1.14901172	1229.035068	1109.561512	1336.2971	10.88606575	10.73523842	11.08636068	74260	89366	104945
317662	NM_173348	FAM149B1	family with sequence similarity 14	-1.78841083	1.148957115	5.575766	3.11698	6.023264	4.17110273	3.39405638	4.36103743	668	499	944
66002	NM_023944,NR_117085	CYP4F12	cytochrome P450 family 4 subfar	-1.288731283	1.148784364	16.078801	12.514182	17.612509	4.91233736	4.56011536	5.10624493	1143	1185	1615
9097	NM_001037334,NM_005151	USP14	ubiquitin specific peptidase 14	1.134612832	1.148665643	38.001339	43.378085	41.049564	7.27397346	7.4550702	7.47272887	6037	9152	8529
9501	NM_001190411,NM_001190412	RPH3AL	rabphilin 3A like (without C2 dom	-2.200556353	1.14845228	2.076219	0.915232	2.210376	2.64317467	1.75827411	2.81278744	206	125	291
113277	NM_001291586,NM_001291587	TMEM106A	transmembrane protein 106A	-1.176909604	1.148047574	1.15644	0.993805	1.294057	2.25390578	2.07130288	2.41366048	148	168	209
85444	NM_001349636,NM_001349637	LRRCC1	leucine rich repeat and coiled-coi	-1.34701069	1.148043218	4.368605	3.29196	4.889112	4.05651939	3.65645563	4.24450423	614	609	867
27032	NM_001001485,NM_001001486	ATP2C1	ATPase secretory pathway Ca2+	1.325854785	1.147586765	32.536866	49.445586	37.662815	7.12134394	7.52572479	7.31861777	5427	9614	7660
57187	NM_001081550	THOC2	THO complex 2	-1.104095747	1.14744264	27.934818	25.330452	30.123917	7.26301006	7.12111952	7.46022811	5991	7250	8455
10667	NM_001318872,NM_006567	FARS2	phenylalanyl-tRNA synthetase 2,	1.305568755	1.147350973	3.21478	4.199387	3.468033	2.78321062	3.11812251	2.95442604	231	403	326
100130742	NM_001129890	LRRC69	leucine rich repeat containing 69	1.012548287	1.147350698	0.663868	0.677114	0.715532	0.89996265	0.90836632	0.99569924	34	46	48
11051	NM_007006	NUDT21	nudix hydrolase 21	1.102313335	1.147259741	37.572903	41.464348	40.511234	7.34191562	7.4816266	7.53896902	6330	9323	8932
4683	NM_001024688,NM_002485	NBN	nibrin	-1.188412213	1.147208028	17.739561	14.998998	19.164144	6.34220103	6.09650305	6.53805101	3146	3537	4439
79695	NM_024642	GALNT12	polypeptide N-acetylgalactosamir	-1.088485882	1.147183642	24.761133	22.771294	26.694445	6.06489791	5.94447568	6.26023459	2589	3178	3653
151525	NM_001128212,NM_001128213	WDSUB1	WD repeat, sterile alpha motif an	1.039151859	1.147119182	8.689363	9.38038	9.946539	4.07625981	4.12844917	4.26329596	623	865	879
130589	NM_138801	GALM	galactose mutarotase	-1.052880336	1.147043328	7.206679	6.846885	7.763951	4.20337642	4.13315895	4.39125092	684	868	965
29911	NM_001100176,NM_013312	HOOK2	hook microtubule tethering protei	-1.009885521	1.146961975	36.406128	36.100115	39.236528	6.54549243	6.53145293	6.74133532	3628	4800	5118
11011	NM_001284333,NM_001284363	TLK2	tousled like kinase 2	1.148255957	1.146919491	10.119701	11.414058	10.896326	5.7705968	5.96663422	5.96498133	2104	3228	2968
54431	NM_001271581,NM_018981,NR	DNAJC10	DnaJ heat shock protein family (H	1.01868665	1.146893305	23.666135	23.437161	25.350825	7.11207679	7.13859634	7.30847548	5392	7339	7606
340351	NM_001345850,NM_001345851	AGBL3	ATP/GTP binding protein like 3	1.406260378	1.14686801	0.220943	0.201103	0.187056	0.5190475	0.68708806	0.58209675	17	32	24
10294	NM_005880	DNAJA2	DnaJ heat shock protein family (H	1.199827816	1.146837261	39.571457	47.53735	42.646732	6.88810125	7.14890418	7.08420605	4611	7392	6504
23399	NM_001143775,NM_015343	CTDNEP1	CTD nuclear envelope phosphata	-1.001784771	1.146833459	37.367813	37.346215	40.278553	5.98534723	5.98281518	6.18009291	2448	3265	3453
3800	NM_004522,NR_111932	KIF5C	kinesin family member 5C	2.416340457	1.146688856	0.208022	0.508116	0.224185	1.27880673	2.15396266	1.39836968	56	181	79
4281	NM_000381,NM_001098624,NM	MID1	midline 1	-1.157930892	1.146575136	1.888296	1.67549	2.078966	3.6915876	3.49751855	3.87461432	468	540	660
84303	NM_001320610,NM_032343	CHCHD6	coiled-coil-helix-coiled-coil-helix c	1.44989998	1.146450731	12.677622	18.439394	13.686235	3.76640418	4.2691718	3.95001076	495	959	698
51592	NM_015906,NM_033020	TRIM33	tripartite motif containing 33	1.145893562	1.146404606	8.861297	10.155939	9.540002	6.18491721	6.37887017	6.37950606	2817	4313	3972
9181	NM_001162383,NM_001162384	ARHGEF2	Rho/Rac guanine nucleotide excl	-1.151551622	1.146352276	23.34784	20.287445	25.241037	6.6044607	6.40312071	6.79962248	3781	4387	5331
10105	NM_005729	PPIF	peptidylprolyl isomerase F	-1.058245134	1.146312588	42.296043	40.008163	45.555805	6.51728721	6.43652791	6.71228179	3557	4491	5015
1891	NM_001398	ECH1	enoyl-CoA hydratase 1	-1.371465236	1.146308374	88.383499	64.507957	95.194717	6.7772253	6.32637162	6.97254554	4267	4157	6016
729920	NM_001101417,NM_001101426	ISPD	isoprenoid synthase domain cont	-1.15747235	1.146190005	0.180247	0.159643	0.196768	0.99521185	0.89363826	1.09694451	39	45	55
28964	NM_001085454,NM_014030	GIT1	GIT ArfGAP 1	1.003324143	1.146084271	19.347092	19.453794	20.861224	6.15892439	6.16364545	6.35306899	2766	3708	3899
2034	NM_001430	EPAS1	endothelial PAS domain protein 1	1.598025999	1.145969947	16.781712	26.845715	18.070679	6.41635423	7.08632739	6.61077645	3314	7076	4671
221937	NM_001037165	FOXK1	forkhead box K1	-1.096776022	1.145753205	7.207983	6.580356	7.761886	6.31384256	6.18232282	6.50783665	3084	3757	4346
219333	NM_182488	USP12	ubiquitin specific peptidase 12	1.080438632	1.145438908	6.176967	6.683234	6.652646	4.81256286	4.92036316	5.00194865	1064	1536	1499
60685	NM_021943	ZFAND3	zinc finger AN1-type containing 3	1.047377545	1.145334693	10.997306	11.534193	11.843074	5.1981206	5.26312825	5.38890871	1402	1962	1975
83856	NM_001145313,NM_001287191	FSD1L	fibronectin type III and SPRY dor	1.131057512	1.145158731	1.813086	2.158245	1.97625	3.48064928	3.64330938	3.65976826	399	603	562
50862	NM_016422	RNF141	ring finger protein 141	-1.835461134	1.145112496	10.941357	5.966774	11.780304	5.47670971	4.62731136	5.66809934	1709	1244	2407
386627	NR_037897	SAP30L-AS1	SAP30L antisense RNA 1 (head	1.528318735	1.14492411	0.162072	0.255133	0.177941	0.64188297	0.89363826	0.71544405	22	45	31
10732	NM_001301726,NM_006602	TCFL5	transcription factor like 5	1.077330842	1.144908693	12.165498	12.996282	12.929337	5.03397229	5.13827799	5.22363661	1247	1795	1756
205	NM_001005353,NM_001330616	AK4	adenylate kinase 4	1.423644942	1.144680327	14.850172	20.990283	16.067254	6.63176857	7.13703402	6.82487859	3854	7331	5426
3065	NM_004964	HDAC1	histone deacetylase 1	1.088988095	1.14465367	57.018524	62.163353	61.337711	6.87156413	6.99354792	7.06492191	4558	6632	6417
79682	NM_024629,NR_104593	CENPU	centromere protein U	-1.173670552	1.144451654	28.509908	24.295938	30.531586	6.15583539	5.92830812	6.34794303	2760	3142	3885
57180	NM_001040135,NM_001350940	ACTR3B	ARP3 actin related protein 3 hom	-1.267151621	1.144413526	4.42898	3.486156	4.756546	3.37481244	3.06980877	3.5518139	368	388	518
11264	NM_007238,NM_183397	PXMP4	peroxisomal membrane protein 4	-1.147550007	1.144411003	2.89927	2.528611	3.121128	4.09788139	3.91166916	4.28184606	633	737	891
11216	NM_001330152,NM_007202	AKAP10	A-kinase anchoring protein 10	1.114498771	1.14420117	7.622152	8.504754	8.200723	4.91964056	5.07114358	5.10797864	1149	1711	1617
54893	NM_017762	MTMR10	myotubularin related protein 10	-1.379737708	1.144180992	2.160391	1.565376	2.321488	3.51641288	3.09899072	3.6947989	410	397	577
10277	NM_001105562,NM_006048	UBE4B	ubiquitination factor E4B	1.050053962	1.144036006	6.964016	7.322356	7.494669	5.25888453	5.32755573	5.44827899	1464	2054	2060
102723316	NR_132316	FRG1DP	FSHD region gene 1 family mem	-1.126786721	1.143697196	1.321425	1.167697	1.40659	0.7550755	0.68708806	0.83750224	27	32	38
246777	NM_145658	SPESP1	sperm equatorial segment protei	-2.696259518	1.143623877	16.035749	5.956995	17.241884	4.54619271	3.21608274	4.73205178	878	435	1235
9902	NM_006039	MRC2	mannose receptor C type 2	1.512625969	1.143566597	2.265345	3.431203	2.433542	3.82979934	4.39215006	4.01058243	519	1049	730
1407	NM_004075	CRY1	cryptochrome circadian clock 1	-1.633479379	1.143301759	14.346303	8.790811	15.415554	5.55539568	4.86669962	5.74476254	1807	1478	2541
8349	NM_003528	HIST2H2BE	histone cluster 2 H2B family mem	1.465032459	1.143231255	13.845797	20.310534	14.880655	4.95324168	5.48935666	5.14052934	1177	2304	1655
95681	NM_001257158,NM_001257159	CEP41	centrosomal protein 41	-1.024182692	1.143196695	2.649117	2.548398	2.729542	4.00480921	3.97250183	4.18661421	591	771	831
55252	NM_018263	ASXL2	additional sex combs like 2, trans	1.180917224	1.143146071	3.651613	4.31754	3.922912	5.54834575	5.78353578	5.73750098	1798	2837	2528
80335	NM_025222	WDR82	WD repeat domain 82	1.145849297	1.14309291	11.696207	13.413544	12.561461	5.64036226	5.83310336	5.82969004	1919	2938	2698
84515	NM_001281520,NM_001281521	MCM8	minichromosome maintenance 8	1.286305607	1.143059081	10.744724	13.765375	11.382538	5.31627029	5.67144382	5.50464438	1525	2621	2144
7014	NM_005652	TERF2	telomeric repeat binding factor 2	1.011068459	1.142872753	8.643	8.748456	9.281169	4.70677986	4.72205729	4.8925416	986	1332	1386
5564	NM_006253	PRKAB1	protein kinase AMP-activated nor	-1.162897349	1.142673202	9.210943	7.927267	9.891473	4.49496323	4.28759625	4.67938813	846	972	1189
64844	NM_001282805,NM_001282806	MARCH7	membrane associated ring-CH-ty	1.352952695	1.142649479	26.73739	36.537662	28.617959	6.53403852	6.96609398	6.72448215	3599	6506	5058
399818	NM_001304467,NM_001304468	EEF1AKMT2	EEF1A lysine methyltransferase	-1.249600281	1.142531204	4.555338	3.632387	4.74232	4.04097679	3.741163	4.22226663	607	649	853
148808	NM_181644	MFSD4A	major facilitator superfamily dom	-1.829518186	1.142522186	0.705437	0.385149	0.761997	1.93632435	1.34717796	2.08088	111	81	156
56987	NM_001142568,NM_001276286	BBX	BBX, HMG-box containing	1.537928674	1.14241622	6.809782	10.604216	7.334642	5.92735377	6.54004932	6.11649229	2350	4829	3302
54806	NM_001134830,NM_001134831	AHI1	Abelson helper integration site 1	-1.235071227	1.141987813	2.389931	1.76364	2.403132	3.71696001	3.43785926	3.89484064	477	516	670
23042	NM_001285444,NM_001285445	PDXDC1	pyridoxal dependent decarboxyla	-1.079499537	1.141836744	36.809242	35.514543	38.154706	7.20217381	7.09258773	7.39231617	5742	7107	8064
410	NM_000487,NM_001085425,NM	ARSA	arylsulfatase A	1.277798038	1.14183487	4.286083	5.415946	4.546431	4.19537543	4.53185994	4.37694243	680	1161	955
79846	NM_001039706,NM_001160138	CFAP69	cilia and flagella associated prote	-1.023042246	1.141738752	1.223646	1.200068	1.312288	2.50111114	2.47408961	2.66044767	183	239	257
9150	NM_001202504,NM_001318511	CTDP1	CTD phosphatase subunit 1	-1.020257731	1.141585666	10.058853	9.885442	10.806875	5.21404828	5.18589965	5.40027255	1418	1857	1991
1111	NM_001114121,NM_001114122	CHEK1	checkpoint kinase 1	-1.086892942	1.14154029	9.07571	6.705254	7.592419	4.71239751	4.59694851	4.89655945	990	1217	1390
6553	NM_001323971,NM_001323972	SLC9A5	solute carrier family 9 member A5	-1.135375745	1.141516254	2.824536	2.403899	2.985	3.47073972	3.30503223	3.64551432	396	466	556
147687	NM_001297734,NM_152475	ZNF417	zinc finger protein 417	1.168619649	1.141201575	3.972945	4.654122	4.266665	4.26583542	4.47980886	4.44710663	716	1118	1005
101929511	NR_135639	LINC01970	long intergenic non-protein codin	1.639482556	1.141094187	0.745019	1.226428	0.797191	1.21691895	1.66595383	1.32870978	52	114	73
3925	NM_001145454,NM_005563,NM	STMN1	stathmin 1	-1.237630367	1.140906174	289.410406	233.887103	310.625574	8.76386485	8.45707065	8.95363623	17027	18382	23893
144486	NR_027035	CEP83-AS1	CEP83 antisense RNA 1 (head to	-1.029119065	1.140847049	0.5774	0.560141	0.618237	1.29387258	1.26944952	1.40965954	57	74	80
1106	NM_001042572,NM_001271	CHD2	chromodomain helicase DNA bin	1.031378777	1.140835678	24.674889	25.582879	26.813306	7.74173896	7.78610871	7.93100002	8364	11526	11736
2060	NM_001159969,NM_001981	EPS15	epidermal growth factor receptor	-1.14682961	1.14063172	10.355913	9.1141	11.174357	5.74067079	5.54696516	5.92718294	2060	2400	2890
666	NM_032515	BOK	BOK, BCL2 family apoptosis regu	1.293080465	1.140551558	13.321507	17.24198	14.275902	5.14302819	5.50458181	5.32773407	1348	2329	1891

80205	NM_001308319,NM_001352127	CHD9	chromodomain helicase DNA bin	1.056003244	1.140508338	5.288441	5.742496	5.944566	5.9085127	5.98585613	6.09523617	2319	3272	3253
171546	NM_138288	SPTSSA	serine palmitoyltransferase small	1.036254661	1.140231642	16.186148	16.787983	17.343353	5.35539329	5.40554179	5.54039208	1568	2171	2199
65123	NM_001324475,NM_023015	INTS3	integrator complex subunit 3	1.086718153	1.140224233	18.005527	19.266893	18.747932	6.48974676	6.60844647	6.67709439	3489	5066	4893
203054	NM_174922	ADCK5	aarF domain containing kinase 5	-1.144039596	1.140131136	9.086721	7.951138	9.736549	4.21727226	4.03423402	4.39693481	691	807	969
3280	NM_005524	HES1	hes family bHLH transcription fac	-1.307833624	1.14009857	58.788193	45.008965	62.989021	6.40338905	6.02143287	6.59045771	3284	3355	4605
126133	NM_001145396,NM_153329	ALDH16A1	aldehyde dehydrogenase 16 fam	-1.105973621	1.140091286	18.740686	17.10037	20.163675	5.84982955	5.70715386	6.03591064	2225	2688	3120
286827	NM_173084	TRIM59	tripartite motif containing 59	-1.195523155	1.140015855	15.037274	12.589618	16.107189	5.85173976	5.59896071	6.03773091	2228	2490	3124
23405	NM_001195573,NM_001271282	DICER1	dicer 1, ribonuclease III	1.313663954	1.139967328	13.12068	17.334188	14.144179	7.0513143	7.44231817	7.23897445	5168	9071	7246
1459	NM_001896	CSNK2A2	casein kinase 2 alpha 2	-1.043732482	1.139903229	23.41592	22.463699	25.091383	5.27889898	5.21876673	5.46325552	1485	1901	2082
997	NM_004359	CDC34	cell division cycle 34	1.2263006	1.139893368	41.557915	51.019566	44.51651	5.8943731	6.18421515	6.08030079	2296	3762	3219
5693	NM_001130725,NM_001144932	PSMB5	proteasome subunit beta 5	1.078227609	1.139888806	129.463151	132.358491	132.681344	7.22175453	7.3297168	7.4094644	5821	8386	8161
53820	NM_001317768,NM_001317777	RIPPLY3	ripply transcriptional repressor 3	1.13876058	1.139868323	1.077136	1.263142	1.149241	1.74129091	1.87534115	1.87636985	92	140	129
55663	NM_001304453,NM_017908	ZNF446	zinc finger protein 446	1.415032504	1.139611431	6.933879	9.853897	7.435155	4.01393646	4.48841131	4.19152809	595	1125	834
26190	NM_012164	FBXW2	F-box and WD repeat domain co	-1.001165256	1.139470646	10.00072	10.000484	10.708684	6.49912087	6.49745927	6.68553669	3512	4687	4922
11143	NM_001199155,NM_001199156	KAT7	lysine acetyltransferase 7	-1.090052508	1.139075015	20.49392	18.633321	21.634672	6.1828954	6.06028071	6.36833848	2813	3448	3941
94240	NM_001002264,NM_001330543	EPSTI1	epithelial stromal interaction 1	-1.370641189	1.139017583	1.605035	1.175692	1.711898	2.58923814	2.2203867	2.74755257	197	192	276
677847	NR_002989	SNORA81	small nucleolar RNA, H/ACA box	-1.068863933	1.138905046	33.97216	31.848577	36.412102	2.78853894	2.70672986	2.95056637	232	290	325
8836	NM_003878	GGH	gamma-glutamyl hydrolase	1.029706673	1.13888951	45.669212	47.077606	48.876823	6.07365394	6.11527096	6.25867461	2605	3584	3649
29886	NM_013321	SNX8	sorting nexin 8	-1.40835267	1.138847306	10.757308	7.646053	11.513224	5.66229636	5.17984253	5.84640328	1949	1849	2730
196	NM_001621	AHR	aryl hydrocarbon receptor	1.39076313	1.138773943	5.285769	7.361695	5.657693	5.05180751	5.51544527	5.23399405	1263	2347	1769
7319	NM_001282161,NM_003336,NM	UBE2A	ubiquitin conjugating enzyme E2	1.036330618	1.138696027	42.245603	44.052355	45.715273	6.28679934	6.33763751	6.47193601	3026	4190	4238
387882	NM_001145199	C12orf75	chromosome 12 open reading fra	1.220391444	1.138689053	6.730604	8.234213	7.20472	3.31698997	3.57802367	3.48667429	352	574	493
9969	NM_005121	MED13	mediator complex subunit 13	-1.032357191	1.138645242	14.025473	13.600637	15.007136	7.16910711	7.12348833	7.35520781	5611	7262	7858
91408	NM_001136497,NM_001243767	BTF3L4	basic transcription factor 3 like 4	1.011928916	1.138337916	12.527891	12.691853	13.432964	5.83831485	5.85512633	6.02218491	2207	2984	3090
26974	NM_001291488,NM_001291489	ZNF285	zinc finger protein 285	1.362942458	1.138160627	1.290203	1.806335	1.451711	2.19091668	2.55119137	2.33885915	140	255	196
645426	NM_001207052	TMEM191C	transmembrane protein 191C	1.921257023	1.138108281	1.88609	3.65547	2.020878	1.77389308	2.49862948	1.90855849	95	244	133
728323	NR_130699,NR_130700,NR_13(	LINC01881	long intergenic non-protein codi	1.185675049	1.138033187	1.353844	1.592914	1.451172	1.40904982	1.5673195	1.52834475	65	103	91
100130950	NR_034082	LOC100130950	uncharacterized LOC100130950	1.660111639	1.137961924	0.742266	1.251855	0.799051	1.18495016	1.63971536	1.29257653	50	111	70
120379	NM_001082619,NM_138789	PIH1D2	PIH1 domain containing 2	1.545467345	1.137927638	0.927855	1.442166	1.001764	1.10178443	1.47139626	1.20457043	45	93	63
283551	NR_126496,NR_126497,NR_12(	LINC01588	long intergenic non-protein codi	-1.856525707	1.137683654	3.197248	1.724578	3.029081	3.12395512	2.36620557	3.28994455	303	218	424
101927574	NR_110388	LINC01186	long intergenic non-protein codi	-1.191985994	1.137653964	0.96827	0.834376	1.058709	0.71085839	0.61708849	0.78645063	25	28	35
27161	NM_001164623,NM_012154	AGO2	argonaute 2, RISC catalytic com	1.883036224	1.137627814	6.419571	12.107931	6.893405	6.51929164	7.42497817	6.70342173	3562	8962	4984
9138	NM_004706,NM_198977,NM_19	ARHGEF1	Rho guanine nucleotide exchang	-1.239449773	1.13748674	45.674953	36.751025	49.069714	7.17038321	6.8630731	7.35502532	5616	6054	7857
91750	NM_001024674	LIN52	lin-52 DREAM MuvB core comple	1.255311668	1.137432279	5.895711	7.423872	6.310792	3.50351028	3.80552077	3.67388275	406	681	568
94081	NM_001322977,NM_001322978	SFXN1	sideroflexin 1	1.210096743	1.137327083	24.282107	29.827538	29.029657	6.54745809	6.81990741	6.73124706	3633	5874	5082
101929054	NR_135307	LOC101929054	uncharacterized LOC101929054	-1.00207692	1.137245484	0.326724	0.345709	0.359874	0.46682716	0.46599441	0.52094563	15	20	21
196441	NM_144982	ZFC3H1	zinc finger C3H1-type containing	-1.032827969	1.137120171	12.953135	12.556772	13.844393	6.51246517	6.46638246	6.69594853	3545	4586	4958
84186	NM_001289119,NM_001289120	ZCCHC7	zinc finger CCHC-type containing	1.090480023	1.137054069	5.317244	5.861179	5.645126	3.90040276	4.01734947	4.07404468	547	797	765
8874	NM_001113511,NM_001113512	ARHGEF7	Rho guanine nucleotide exchang	1.127357691	1.136896405	8.612628	9.726345	9.723834	5.44162746	5.61082803	5.62273699	1667	2511	2331
23074	NM_001006947,NM_015054	UHRF1BP1L	UHRF1 binding protein 1 like	-1.185594913	1.136814417	2.38596	2.039138	2.479316	3.65995838	3.43531911	3.83119295	457	515	639
104310353	NR_126356	LINC01399	long intergenic non-protein codi	6.008718263	1.13673828	0.397648	2.429836	0.408329	0.3273552	1.34717796	0.36729312	10	81	14
23305	NM_001009185,NM_001205247	ACSL6	acyl-CoA synthetase long-chain f	1.491607957	1.13673828	0.038142	0.059695	0.039105	0.3273552	0.46599441	0.36729312	10	20	14
283440	NR_146317,NR_146318	ITFG2-AS1	ITFG2 antisense RNA 1	2.084015866	1.13673828	0.457764	0.979905	0.477487	0.3273552	0.61708849	0.36729312	10	28	14
6051	NM_001319182,NM_001319183	RNPEP	arginyl aminopeptidase	-1.195560692	1.136575775	79.316193	66.53431	84.728281	7.54603125	7.28984863	7.72979986	7298	8156	10202
8907	NM_001130524,NM_032493	AP1M1	adaptor related protein complex '	1.199754973	1.136327891	16.774733	20.170679	17.938462	5.30608931	5.56276153	5.48609953	1514	2427	2116
7453	NM_004184,NM_173701,NM_21	WARS	tryptophanyl-tRNA synthetase	1.24808732	1.136078713	46.44388	59.356904	50.638246	6.99711436	7.31459273	7.17982764	4976	8298	6953
8446	NM_003584	DUSP11	dual specificity phosphatase 11	1.244676641	1.136068101	11.876316	14.808822	12.688472	4.32019405	4.62173661	4.49559213	745	1239	1041
7011	NM_001319035,NM_007110	TEP1	telomerase associated protein 1	1.01613328	1.136046125	7.197144	7.28969	7.734562	6.24089332	6.26368089	6.42263507	2930	3978	4094
5163	NM_001278549,NM_002610,NR	PDK1	pyruvate dehydrogenase kinase 1	4.222665554	1.136032611	2.449539	10.131322	2.486021	3.38187998	5.35065612	3.54926401	370	2088	517
27106	NM_001025604,NM_001286826	ARRDC2	arrestin domain containing 2	-1.437070848	1.135653656	11.713142	8.147861	12.504062	4.88896361	4.38690129	5.0666753	1124	1045	1570
836	NM_004346,NM_032991	CASP3	caspase 3	1.069923149	1.135520409	13.047922	14.274755	14.181068	5.09867502	5.19343546	5.27700936	1306	1867	1824
84187	NM_017698,NM_032227	TMEM164	transmembrane protein 164	-1.460872692	1.135520389	8.63117	5.914928	9.209731	5.57941842	5.04638922	5.75917693	1838	1681	2567
375033	NM_001080471	PEAR1	platelet endothelial aggregation r	-4.147779366	1.135356294	15.622849	3.767182	16.666248	6.22918489	4.2359869	6.41004203	2906	936	4058
93624	NM_152293	TADA2B	transcriptional adaptor 2B	-1.193861128	1.135288579	7.640924	6.406989	8.149917	5.04736934	4.80014446	5.22523491	1259	1409	1758
23786	NM_001270726,NM_001270727	BCL2L13	BCL2 like 13	-1.094037757	1.135253927	7.98262	7.309046	8.76844	5.30608931	5.17984253	5.48476573	1514	1849	2114
102724552	NR_125999	LOC102724552	uncharacterized LOC102724552	1.193356706	1.135238914	0.166477	0.22455	0.190775	0.17294258	0.20477631	0.19530369	5	8	7
4583	NM_002457	MUC2	mucin 2, oligomeric mucus/gel-fo	1.779557809	1.135238914	0.01512	0.025355	0.015151	0.17294258	0.29720487	0.19530369	5	12	7
100132354	NR_024478	LINC01512	long intergenic non-protein codi	-1.647024248	1.135238914	0.045857	0.034274	0.052555	0.17294258	0.10601836	0.19530369	5	4	7
8342	NM_003521	HIST1H2BM	histone cluster 1 H2B family mer	1.486457258	1.135238914	0.23463	0.43036	0.272894	0.17294258	0.25173066	0.19530369	5	10	7
100126314	NR_030615	MIR877	microRNA 877	1.339906982	1.135238914	1.361374	1.992658	1.541807	0.17294258	0.2284445	0.19530369	5	9	7
101927655	NM_001289933	ZASP	ZO-2 associated speckle protein	-1.110794961	1.135238914	0.180242	0.166954	0.194216	0.17294258	0.15624221	0.19530369	5	6	7
102465474	NR_106849	MIR6791	microRNA 6791	-3.184155122	1.135238914	1.909808	0.321528	1.843162	0.17294258	0.05398282	0.19530369	5	2	7
102466985	NR_106896	MIR6837	microRNA 6837	-1.326777498	1.135238914	1.784827	1.394917	1.975944	0.17294258	0.13134883	0.19530369	5	5	7
8289	NM_006015,NM_139135	ARID1A	AT-rich interaction domain 1A	-1.083410163	1.135211581	16.695641	15.547835	17.809805	7.13500329	7.02027731	7.31674494	5479	6757	7650
606495	NM_001031672	CYB5RL	cytochrome b5 reductase like	1.819725929	1.135204793	0.821904	1.497946	0.872725	1.93632435	2.61991364	2.07380058	111	270	155
55920	NM_001136204,NM_018715	RCC2	regulator of chromosome conden	1.102846841	1.135175572	69.903519	74.068538	71.632986	8.07708976	8.21782528	8.25937073	10563	15565	14748
51275	NR_015404	MAPKAPK5-AS1	MAPKAPK5 antisense RNA 1	-1.372153874	1.135158567	7.822368	5.7093	8.350319	4.20337642	3.77571017	4.37694243	684	666	955
80219	NM_001320818,NM_001320819	COQ10B	coenzyme Q10B	1.053914217	1.135046739	18.296008	19.414306	19.498354	5.35539329	5.42935176	5.53395803	1568	2208	2189
9552	NM_004890	SPAG7	sperm associated antigen 7	-1.028947034	1.13498975	32.991764	32.097145	35.19643	5.08466566	5.04472373	5.26229407	1293	1679	1805
25786	NR_024157	DGCR11	DiGeorge syndrome critical regio	-1.249191921	1.134901519	1.184086	0.948024	1.269747	1.83696709	1.61299082	1.97085819	101	108	141
6872	NM_001286074,NM_004606,NM	TAF1	TATA-box binding protein associ	1.079621067	1.134785914	9.438953	10.151776	10.121547	6.15428841	6.2633229	6.33430752	2757	3977	3848
55298	NM_001300926,NM_018320,NR	RNF121	ring finger protein 121	1.91331079	1.134570253	7.612225	14.425826	8.019823	4.33118941	5.23273108	4.50483355	751	1920	1048
6594	NM_001282874,NM_001282875	SMARCA1	SWI/SNF related, matrix associa	1.242969352	1.134544942	33.920297	42.25565	36.094392	7.05932659	7.37100709	7.24016067	5197	8631	7252
5359	NM_021105	PLSCR1	phospholipid scramblase 1	-1.324488133	1.134521789	43.168037	32.62804	46.022804	6.55294771	6.15247651	6.7332142	3647	3679	5089
440400	NM_001004333,NR_037715,NR	RNASEK	ribonuclease K	1.029329872	1.134492032	50.65152	52.742278	54.108407	5.38031	5.4210308	5.55825623	1596	2195	2227
644656	NR_036539	LOC644656	uncharacterized LOC644656	-1.003548202	1.134402028	3.917517	3.925995	4.190431	2.4278825	2.42372099	2.57778742	172	229	240
550643	NM_001348129	NBDY	negative regulator of P-body ass	-1.109210992	1.134264979	6.317655	5.702391	6.734032	3.96064828	3.82117143	4.1314271	572	689	798
58508	NM_170606	KMT2C	lysine methyltransferase 2C	-1.602109931	1.134225029	3.745981	2.34022	3.992572	5.966078	5.2998966	6.14505901	2415	2014	3369
8718	NM_001039664,NM_003790,NM	TNFRSF25	TNF receptor superfamily membe	-1.705181391	1.134064785	17.478778	10.325262	20.928957	4.68408793	3.95309996	4.85896034	970	760	1353
22996	NM_001080494,NM_001144832	TTC39A	tetratricopeptide repeat domain 3	1.045422601	1.134047136	4.86586	5.135903	5.355384	3.79050499	3.8500665	3.95964434	504	704	703
401588	NR_015378	ZNF674-AS1	ZNF674 antisense RNA 1 (head '	1.232506472	1.134045563	1.448138	1.783091	1.541031	2.01115992	2.24380223	2.14982678	119	196	166
286262	NM_001128228	TPRN	taperin	-1.22728232	1.13402487	8.405483	6.854751	8.958993	4.49496323	4.21392593	4.66885564	846	921	1180
55833	NM_001282529,NM_001282530	UBAP2	ubiquitin associated protein 2	1.141399947	1.133982638	9.879339	11.615388	10.879261	5.38031	5.56682881	5.55762202	1596	2434	2226
55905	NM_018683	RNF114	ring finger protein 114	-1.151177577	1.133895166	46.132786	40.11882	49.162518	6.80509625	6.60392963	6.98486361	4351	5050	6068
873	NM_001286789,NM_001757	CBR1	carbonyl reductase 1	1.299816441	1.133230535	70.122336	91.985699	75.642786	6.54076391	6.91550292	6.71938758	3616	6280	5040
79955	NM_001195263,NM_001351044	PDZD7	PDZ domain containing 7	-1.259419242	1.133192851	0.662116	0.559408	0.77882	1.81624731	1.58576189	1.94780992	99	105	138
55291	NM_001164160,NM_001164161	PPP6R3	protein phosphatase 6 regulatory	1.25809027	1.133180581	20.234644	25.20957	21.309379	6.63694713	6.96521381	6.81562507	3868	6502	5391
100506801	NR_038404	LOC100506801	uncharacterized LOC100506801	-2.37802806	1.133117563	0.661504	0.276486	0.711192	1.42282133	0.77006602	1.53866543	66	37	92
10982	NM_001143826,NM_001143827	MAPRE2	microtubule associated protein R	-1.045254786	1.132936359	2.818001	2.710126	2.984201	3.66865353	3.60990992	3.83538509	460	588	641
10791	NM_006634	VAMP5	vesicle associated membrane pro	1.012216202	1.132817731	4.78549	4.841671	5.096637	2.03825186	2.05153568	2.1765086	122	165	170
23053	NM_001242487,NM_001242488	ZSWIM8	zinc finger SWIM-type containing	-1.343868451	1.132815958	11.247061	8.381054	11.978987	6.06873521	5.64969525	6.24613375	2596	2581	3617
253461	NM_001080412,NM_001350099	ZBTB38	zinc finger and BTB domain cont	-1.259953195	1.132613702	10.260911	8.196417	10.917887	6.36333314	6.03449873	6.54094167	3193	3386	4448
84975	NM_001170790,NM_032889	MFSD5	major facilitator superfamily dom	-1.192846497	1.132574333	13.568468	11.372643	14.429081	4.60475543	4.36170552	4.77742372	916	1026	1276
63897	NM_022070	HEATR6	HEAT repeat containing 6	-1.080381745	1.13255086	14.379897	13.322428	15.300612	5.80977473	5.70029381	5.98634592	2163	2675	3013
10526	NM_001190995,NM_006390	IPO8	importin 8	-1.128669973	1.132399038	10.101348	8.909318	10.547108	5.71571556	5.54461018	5.89189395	2024	2396	2819
100190939	NR_024458	TPT1-AS1	TPT1 antisense RNA 1	1.002365305	1.132331296	1.906494	1.922758	2.035417	1.94589385	1.94842076	2.08088	112	150	156
6257	NM_001270401,NM_001291989	RXRB	retinoid X receptor beta	1.047356608	1.132300635	11.959492	12.548965	12.730052	5.13154304	5.1964388	5.30599715	1337	1871	1862
100137047	NM_001114632	JMJD7	jumonji domain containing 7	-1.107244421	1.132129727	8.795813	7.956678	9.362498	3.72253824	3.58720616	3.88880246	479	578	667
490	NM_001001323,NM_001682	ATP2B1	ATPase plasma membrane Ca2+	1.790196183	1.132118098	8.263345	14.820587	8.794563	5.79661292	6.6252598	5.97261393	2143	5126	2984

388677	NM_203458	NOTCH2NL	notch 2 N-terminal like	-1.16017128	1.132091566	4.799953	4.140726	5.106308	4.69263929	4.48718552	4.86512445	976	1124	1359
3932	NM_001042771,NM_001330468	LCK	LCK proto-oncogene, Src family t	-2.326361014	1.131935494	0.345292	0.138756	0.382101	0.77668602	0.38406669	0.85412573	28	16	39
10961	NM_001034025,NM_006817	ERP29	endoplasmic reticulum protein 29	-1.031446821	1.131864771	124.01418	120.616156	132.140652	7.47737375	7.43295818	7.65513456	6957	9012	9685
138429	NM_001135219,NM_173492	PIP5KL1	phosphatidylinositol-4-phosphate	-1.300260567	1.131836433	5.660985	4.610032	6.245214	2.90595399	2.58366954	3.06208787	255	262	355
79650	NM_001195302,NM_001204911	USB1	U6 snRNA biogenesis phosphodi	1.482725372	1.131650839	18.530117	27.60933	20.152434	5.46677296	6.02439359	5.64141262	1697	3362	2362
116151	NM_080821	FAM210B	family with sequence similarity 2	1.122622118	1.131645719	3.825301	4.303701	4.069846	3.62166181	3.77571017	3.78642406	444	666	618
81887	NM_001170649,NM_001170650	LAS1L	LAS1 like, ribosome biogenesis f	1.088604905	1.131578078	30.881367	33.784369	32.962772	6.21094615	6.33184486	6.38702237	2869	4173	3993
8073	NM_001195100,NM_001195101	PTP4A2	protein tyrosine phosphatase typ	1.05752932	1.131094279	56.56418	59.44438	61.753595	7.69000425	7.77032284	7.8669158	8068	11400	11224
5110	NM_001252049,NM_001252050	PCMT1	protein-L-isoaspartate (D-asparta	1.123981315	1.130789123	45.368352	50.796579	46.939617	6.02820678	6.19439106	6.20298496	2523	3789	3509
55266	NM_018279	TMEM19	transmembrane protein 19	1.05558853	1.130734774	4.856755	5.133458	5.15858	4.52559963	4.60035389	4.69562066	865	1220	1203
147339	NM_001008239,NM_145055	C18orf25	chromosome 18 open reading fra	-1.117205191	1.130721427	8.440053	7.577172	9.012955	5.48985314	5.33370663	5.66339097	1725	2063	2399
284656	NM_001099439,NM_173641	EPHA10	EPH receptor A10	1.227824078	1.130603156	1.518987	1.865388	1.612496	3.19389071	3.46052158	3.35270793	320	525	445
307	NM_001153,NM_001320698,NM	ANXA4	annexin A4	-1.520601293	1.130557171	38.102065	25.231265	40.980621	6.36912395	5.77351513	6.54414675	3206	2817	4458
8312	NM_003502,NM_181050,NR_13	AXIN1	axin 1	1.130352213	1.130496383	17.625896	20.069045	18.818487	5.97369967	6.14782946	6.14801122	2428	3667	3376
408	NM_004041,NM_020251	ARRB1	arrestin beta 1	1.309692923	1.130309367	1.710596	2.247337	1.820159	3.75828027	4.12214544	3.92268941	492	861	684
151242	NM_001080545,NM_001261424	PPP1R1C	protein phosphatase 1 regulatory	-1.394217901	1.130276563	8.600615	6.475705	10.390266	3.91021271	3.46799726	4.0758175	551	528	766
2595	NM_001301409,NM_001301410	GANC	glucosidase alpha, neutral C	1.075056987	1.130143626	5.220149	5.726387	5.632408	3.98405928	4.08211055	4.15005835	582	836	809
80204	NM_001190274,NM_025133	FBXO11	F-box protein 11	1.09850559	1.13011782	22.399824	24.629578	23.785347	6.48277871	6.61687763	6.65739873	3472	5096	4826
55014	NM_017919	STX17	syntaxin 17	1.072871679	1.130094875	7.912447	8.498583	8.402967	5.76114205	5.86081659	5.93452919	2090	2996	2905
401934	NM_001205252	RNF223	ring finger protein 223	-1.081387667	1.130053069	6.033932	5.585637	6.411053	2.92543304	2.8278811	3.07986458	259	320	360
2176	NM_000136,NM_001243743,NM	FANCC	Fanconi anemia complementatior	1.193591903	1.130018244	5.074288	5.952793	5.64717	4.49984402	4.7448025	4.66885564	849	1354	1180
57555	NM_020795	NLGN2	neuroligin 2	1.395766912	1.129896102	8.755363	12.231451	9.296082	5.3436769	5.81465343	5.51578855	1555	2900	2161
5825	NM_001122674,NM_002858	ABCD3	ATP binding cassette subfamily D	-1.00436042	1.129774273	19.855314	19.335238	16.835914	5.69316485	5.68700902	5.866002	1992	2650	2768
90271	NR_026762	OLMALINC	oligodendrocyte maturation-asso	-2.328149498	1.129674576	10.035811	4.319161	10.67077	3.41670749	2.36620557	3.57706795	380	218	528
441150	NM_001008739	C6orf226	chromosome 6 open reading fran	-1.1132932	1.129559938	4.457346	4.037261	4.753989	1.77389308	1.66595383	1.90057841	95	114	132
84133	NM_001206998,NM_032173	ZNRF3	zinc and ring finger 3	1.097685022	1.129455375	2.172616	2.385906	2.303701	3.9199564	4.04593672	4.08464905	555	814	771
10552	NM_001190996,NM_006409	ARPC1A	actin related protein 2/3 complex	-1.313302545	1.1294461	83.406309	63.570057	88.526971	7.03907211	6.64929626	7.21343331	5124	5213	7118
22826	NM_014280	DNAJC8	DnaJ heat shock protein family (I	1.236450921	1.129431065	50.764053	62.837864	53.876568	6.46250395	6.76558561	6.63622955	3423	5655	4755
55250	NM_001242875,NM_001242876	ELP2	elongator acetyltransferase comp	1.135621915	1.129333422	7.813865	8.882538	8.339904	5.49393602	5.67360076	5.66574708	1730	2625	2403
8030	NM_005436	CCDC6	coiled-coil domain containing 6	-1.489466321	1.129212395	10.725025	7.206842	11.381817	5.95071282	5.38725856	6.12336578	2389	2143	3318
93611	NM_001014765,NM_001304790	FBXO44	F-box protein 44	1.382955809	1.129186941	10.045359	14.437415	10.891286	4.96034122	5.41524189	5.13032929	1183	2186	1643
9147	NM_001301732,NM_004713	NEMF	nuclear export mediator factor	-1.15790818	1.129117589	21.245391	18.371554	22.546243	6.64578166	6.43652791	6.8193336	3892	4491	5405
29998	NM_015711	BICRA	BRD4 interacting chromatin remc	-1.118764387	1.128941356	5.09174	4.553299	5.400571	4.88025587	4.72413992	5.04963668	1117	1334	1551
6613	NM_001005849,NM_006937	SUMO2	small ubiquitin-like modifier 2	-1.139255001	1.128903554	283.954466	249.481534	301.071776	8.20488382	8.01747197	8.37924924	11545	13540	16030
100129250	NM_001349118,NM_001349119	SMIM27	small integral membrane protein	1.41248335	1.128836145	0.923327	1.29934	0.978155	0.93882015	1.19932051	1.02535867	36	68	50
10597	NR_002166	TRAPPC2B	trafficking protein particle comple	-1.165888852	1.128792581	37.441261	32.137718	39.71508	4.79148701	4.57864948	4.96032831	1048	1201	1455
84987	NM_001257133,NM_001257134	COX14	COX14, cytochrome c oxidase as	-1.237554248	1.128539296	20.903284	17.062513	22.148535	4.03201954	3.74527057	4.1964253	603	651	837
11258	NM_001281425,NM_001281426	DCTN3	dynactin subunit 3	1.206465615	1.128468543	20.243643	24.453781	21.455265	4.16497021	4.42196539	4.33017765	665	1072	923
56097	NM_018929,NM_032407	PCDHGC5	protocadherin gamma subfamily	3.271292179	1.128437408	0.178477	0.354805	0.189273	0.54446555	1.32539385	0.60191771	18	79	25
9885	NM_001278649,NM_014835,NM	OSBPL2	oxysterol binding protein like 2	-1.039872393	1.128230449	21.481916	20.68147	22.932394	6.40555802	6.34982807	6.57768983	3289	4226	4564
1107	NM_001005271,NM_001005273	CHD3	chromodomain helicase DNA bin	1.191283493	1.128152796	19.971702	23.868503	21.2091	7.16475992	7.41566573	7.33758305	5594	8904	7762
23170	NM_015140	TTLL12	tubulin tyrosine ligase like 12	1.07601932	1.128129397	9.554737	10.295346	10.127831	5.02158142	5.12415271	5.19047509	1236	1777	1715
151507	NR_024322	MSL3P1	male-specific lethal 3 homolog (D	1.872776189	1.128101234	8.000412	15.00447	8.483202	4.04543465	4.90942294	4.20940382	609	1524	845
56907	NM_001128626,NM_001128627	SPIRE1	spire type actin nucleation factor	-1.404761752	1.128013358	10.517286	7.678732	11.314854	5.8267075	5.34660637	5.99761231	2189	2082	3037
22937	NM_001320044,NM_012235	SCAP	SREBF chaperone	1.459484663	1.127538033	10.789956	15.603302	11.275267	5.45509291	5.99018926	5.62455491	1683	3282	2334
6643	NM_001278199,NM_003100	SNX2	sorting nexin 2	1.204351457	1.127448895	8.694868	10.457478	9.164574	5.82086862	6.08479986	5.99105095	2180	3508	3023
57600	NM_001323916,NM_001346043	FNIP2	folliculin interacting protein 2	-1.105603502	1.127420179	1.221368	1.109109	1.296802	3.23740108	3.10858833	3.39308282	331	400	459
375061	NM_198552	FAM89A	family with sequence similarity 89	1.369403314	1.127398114	1.83842	2.536222	1.949278	1.88750088	2.23214197	2.01587867	106	194	147
646	NM_001301206,NM_001717	BNC1	basonuclin 1	-2.397105166	1.127398114	0.58231	0.241089	0.616954	1.88750088	1.08728891	2.01587867	106	59	147
55631	NM_017768	LRRC40	leucine rich repeat containing 40	1.063212174	1.127391195	8.102217	8.627757	8.586126	4.60021751	4.68511603	4.76648826	913	1297	1266
7905	NM_005669	REEP5	receptor accessory protein 5	-1.33824007	1.127292695	84.694435	63.356041	89.718002	8.01096183	7.59251022	8.1831941	10088	10072	13987
50808	NM_001199852,NM_001199853	AK3	adenylate kinase 3	1.059144906	1.127091778	3.503988	3.663046	3.655148	3.91752664	3.99509991	4.07935661	554	784	768
10809	NM_006645	STARD10	StAR related lipid transfer domai	1.02319195	1.127064593	13.755865	14.09556	14.578925	4.78883075	4.82072735	4.95552185	1046	1430	1450
7323	NM_001300795,NM_003340,NM	UBE2D3	ubiquitin conjugating enzyme E2	1.120339606	1.127047892	47.231154	54.088623	51.717141	7.54426054	7.70736848	7.71594032	7289	10911	10104
100506844	NR_038269	LOC100506844	uncharacterized LOC100506844	1.504760006	1.127022382	3.285769	4.954338	3.463737	1.69664266	2.12902957	1.81825156	88	177	122
10973	NM_001284271,NM_006828,NM	ASCC3	activating signal cointegrator 1 cc	1.08321914	1.127021059	8.210174	8.475657	7.76079	5.7853302	5.89864975	5.95490187	2126	3077	2947
10927	NM_006717	SPIN1	spindlin 1	-1.042673166	1.126604903	31.608316	30.349693	33.467949	7.13500329	7.07515303	7.30583432	5479	7021	7592
85369	NM_001270768,NM_033088,NR	STRIP1	striatin interacting protein 1	-1.027589407	1.126520747	9.715921	9.459846	10.254497	5.00222007	4.96419416	5.16904389	1219	1585	1689
5716	NM_002814,NM_170750	PSMD10	proteasome 26S subunit, non-AT	1.197215653	1.126461338	37.177397	44.573642	39.340621	5.84536252	6.10091747	6.01434863	2218	3548	3073
414060	NM_001001418,NM_001001418	TBC1D3C	TBC1 domain family member 3C	-1.317186515	1.126369708	8.664812	6.584821	9.176197	4.23103553	3.8576751	4.39409566	698	708	967
122961	NM_001272007,NM_194279	ISCA2	iron-sulfur cluster assembly 2	1.113030438	1.126269224	29.488219	32.873992	31.21632	4.98839509	5.13827799	5.15485706	1207	1795	1672
54830	NM_017681,NR_033676	NUP62CL	nucleoporin 62 C-terminal like	1.196638387	1.126059452	5.89971	6.827779	6.262878	3.39591204	3.63226192	3.5518139	374	598	518
5310	NM_000296,NM_001009944	PKD1	polycystin 1, transient receptor p	1.590982013	1.12596906	6.603929	10.520557	6.988387	6.52249295	7.18658531	6.69190841	3570	7589	4944
79034	NM_001303039,NM_024067	C7orf26	chromosome 7 open reading fran	-1.25016136	1.125724066	4.361402	3.496402	4.620749	3.36057263	3.07308051	3.51569869	364	389	504
10957	NM_006813	PNRC1	proline rich nuclear receptor coac	1.383117779	1.125641542	7.740196	10.724965	8.191428	4.09573376	4.54015842	4.2570592	632	1168	875
25828	NM_012473	TXN2	thioredoxin 2	1.295242389	1.125550894	59.086895	76.62661	62.495697	6.28302914	6.65203359	6.4515983	3018	5223	4178
2629	NM_000157,NM_001005741,NM	GBA	glucosylceramidase beta	1.330063628	1.125443483	16.915949	23.053295	17.421602	5.3934833	5.79645942	5.56015719	1611	2863	2230
285116	NR_077058	AHCTF1P1	AT-hook containing transcription	1.098372484	1.125440687	1.276102	1.406333	1.351763	3.58538547	3.70997765	3.74245532	432	634	598
22806	NM_001257408,NM_001257409	IKZF3	IKAROS family zinc finger 3	1.776661526	1.125385452	0.541136	0.962846	0.579872	2.62541894	3.34888182	2.7696269	203	482	281
152302	NR_002786	CIDECP	cell death-inducing DFFA-like effe	1.181860124	1.12529578	5.323671	6.306606	5.642348	2.37218925	2.56983975	2.51121823	164	259	227
11007	NM_006848	CCDC85B	coiled-coil domain containing 85E	1.302392288	1.125231245	40.526516	52.851059	42.861553	5.63297616	6.00739279	5.79996773	1909	3322	2642
57156	NM_020431	TMEM63C	transmembrane protein 63C	4.390294555	1.125222834	0.123038	0.552901	0.132025	0.73313634	1.96964178	0.80366934	26	153	36
7248	NM_000368,NM_001162426,NM	TSC1	tuberous sclerosis 1	1.100285812	1.125151094	10.342327	11.360546	10.929267	6.45162908	6.58800837	6.61991848	3397	4994	4701
6451	NM_003022	SH3BGRL	SH3 domain binding glutamate ri	-1.098440594	1.12512359	23.86198	21.743605	25.225033	5.6120916	5.47952756	5.7789005	1881	2288	2603
7098	NM_003265	TLR3	toll like receptor 3	-1.065233366	1.125116832	3.516181	3.304069	3.719755	3.51319803	3.43022535	3.66919325	409	513	566
6868	NM_003183	ADAM17	ADAM metallopeptidase domain	1.224666866	1.124847041	21.702374	26.610943	22.943617	6.5482436	6.83780992	6.71626534	3635	5948	5029
161247	NM_203402	FITM1	fat storage inducing transmembra	-1.332465618	1.124687495	0.317215	0.248061	0.343175	0.41264541	0.31941552	0.45708734	13	13	18
4976	NM_015560,NM_130831,NM_13	OPA1	OPA1, mitochondrial dynamin like	1.068172471	1.124251379	25.843437	27.68074	27.302565	7.33487669	7.42945242	7.5028562	6299	8990	8710
9028	NM_001278720,NM_001278721	RHBDL1	rhomboid like 1	1.201503884	1.124242542	4.285661	5.155564	4.550909	2.91084846	3.14324318	3.05850609	256	411	354
23438	NM_001278731,NM_001278732	HARS2	histidyl-tRNA synthetase 2, mitoc	1.145684373	1.124195159	19.750858	22.542573	20.833479	5.61434375	5.80681389	5.77998839	1884	2884	2605
10782	NM_001278734,NM_016324,NM	ZNF274	zinc finger protein 274	-1.224295709	1.124166751	23.635146	19.50975	24.987802	6.06818765	5.78103713	6.23467365	2595	2832	3588
23517	NM_015360	SKIV2L2	Ski2 like RNA helicase 2	1.008885176	1.124129855	30.558594	30.863194	32.282627	6.97829416	6.99095565	7.14584247	4911	6620	6790
4738	NM_006156	NEDD8	neural precursor cell expressed,	1.128729105	1.124096984	198.346802	224.128326	209.536667	6.92727522	7.10062566	7.09473626	4739	7147	6552
59342	NM_021626	SCPEP1	serine carboxypeptidase 1	-1.027197413	1.124038409	23.516052	22.922205	24.844685	5.50934646	5.47149182	5.67454836	1749	2275	2418
29926	NM_013335,NM_205847	GMPPA	GDP-mannose pyrophosphorylas	1.213193118	1.123962736	23.179333	25.639096	23.494806	5.26176079	5.53396493	5.4262136	1467	2378	2028
8673	NM_003761	VAMP8	vesicle associated membrane pro	-1.023055169	1.12377156	98.707779	96.577408	104.22699	6.23992124	6.20747612	6.40617212	2928	3824	4047
10098	NM_005723	TSPAN5	tetraspanin 5	1.326364169	1.123753775	3.885338	5.158996	4.102232	3.79315817	4.17486912	3.95001076	505	895	698
79633	NM_001291285,NM_001291303	FAT4	FAT atypical cadherin 4	2.671921329	1.123719333	0.108752	0.295384	0.115479	1.44997615	2.49375478	1.5590879	68	243	94
727957	NM_001099280,NM_001099281	MROH1	maestro heat like repeat family m	1.006840126	1.123675258	12.460254	13.243571	14.58742	5.68747166	5.6971166	5.85262116	1984	2669	2742
10490	NM_006370	VTI1B	vesicle transport through interact	1.164667931	1.123400779	32.919037	38.391254	34.752911	5.44753406	5.6627837	5.61178108	1674	2605	2313
7942	NM_001167827,NM_001271943	TFEB	transcription factor EB	-1.24568558	1.123278925	2.38211	1.922158	2.537537	2.64904502	2.38738651	2.79136857	207	222	286
8036	NM_001269039,NM_001324336	SHOC2	SHOC2, leucine rich repeat scaff	-1.115456662	1.123188358	4.626641	4.086779	4.619628	4.1064401	3.95841731	4.26485094	637	763	880
3054	NM_005334	HCFC1	host cell factor C1	-1.132855097	1.122804992	15.571985	13.761493	16.43013	7.00857515	6.83009554	7.17446007	5016	5916	6927
51409	NM_001317851,NM_016173	HEMK1	HemK methyltransferase family m	1.354240524	1.122759374	2.333496	3.177671	2.491562	3.89793981	4.30995418	4.05439856	546	988	754
124245	NM_001294340,NM_144604	ZC3H18	zinc finger CCCH-type containing	1.21238903	1.122739092	25.866237	31.352789	27.35236	6.57624091	6.85144979	6.74161454	3707	6005	5119
3159	NM_001319077,NM_001319078	HMGA1	high mobility group AT-hook 1	1.341475518	1.122468959	206.915349	279.567012	219.041928	8.65120455	9.07411404	8.81748964	15745	28221	21737
51244	NM_016474,NR_135523	CCDC174	coiled-coil domain containing 174	1.009514541	1.122311608	5.328584	5.384592	5.65121	4.02977152	4.04260277	4.18661421	602	812	831
84962	NM_001289097,NM_032876,NM	AJUBA	ajuba LIM protein	-3.211921729	1.122310018	16.753374	5.599228	17.445377	6.04778035	4.41166458	6.21187981	2558	1064	3531
8243	NM_001281463,NM_006306	SMC1A	structural maintenance of chromc	-2.112783917	1.122286946	6.937748	3.28711	7.316094	6.06818765	5.01270852	6.23229119	2595	1641	3582

22	NM_001271696,NM_001271697	ABCB7	ATP binding cassette subfamily E	1.040921496	1.122284779	10.373925	10.781753	10.907342	4.66538426	4.72101485	4.82563275	957	1331	1321
92912	NM_001145335,NM_001284382	UBE2Q2	ubiquitin conjugating enzyme E2	-1.080282776	1.122077529	8.630727	7.91836	9.029364	4.733271	4.62619813	4.89354711	1005	1243	1387
8847	NR_002612	DLEU2	deleted in lymphocytic leukemia 2	1.548748446	1.12204656	0.664956	1.038313	0.705965	1.48977343	1.92688293	1.59908548	71	147	98
85025	NM_032936	TMEM60	transmembrane protein 60	-1.485372032	1.121909746	28.298433	19.076748	29.834024	4.64790143	4.1046668	4.80760927	945	850	1304
79672	NM_024619,NR_046408	FN3KRP	fructosamine 3 kinase related pro	-1.038432763	1.12188091	31.981682	30.948973	33.777662	5.86125307	5.80779617	6.02448161	2243	2886	3095
55755	NM_001011649,NM_001272039	CDK5RAP2	CDK5 regulatory subunit associa	1.077303358	1.121797083	13.967983	14.903239	14.499469	6.39642635	6.50262483	6.56038277	3268	4704	4509
828	NM_004058,NM_080590	CAPS	calcyphosine	1.263686985	1.121794808	17.422733	22.00352	18.308025	5.21800288	5.54755331	5.37960921	1422	2401	1962
221302	NM_145062	ZUFSP	zinc finger with UFM1 specific pe	-1.189885524	1.121687967	11.83889	9.961881	12.481532	4.7277342	4.48718552	4.88750351	1001	1124	1381
3460	NM_001329128,NM_005534	IFNGR2	interferon gamma receptor 2	-1.10604797	1.121647119	42.627291	38.579987	44.934325	6.54234181	6.39856393	6.70628577	3620	4373	4994
5594	NM_002745,NM_138957	MAPK1	mitogen-activated protein kinase	1.595187623	1.121534941	25.31844	39.415871	28.754068	7.03010299	7.69971592	7.19438431	5092	10853	7024
26289	NM_012093,NM_174858	AK5	adenylate kinase 5	1.189385699	1.121513866	1.484246	1.771234	1.856306	2.74535098	2.96099164	2.88736461	224	356	309
57154	NM_001199847,NM_020429,NM	SMURF1	SMAD specific E3 ubiquitin prote	-1.880990168	1.121396356	23.029951	12.255186	24.275087	7.00457422	6.10291957	7.16865719	5002	3553	6899
55315	NM_001174098,NM_018344,NR	SLC29A3	solute carrier family 29 member 3	-1.018645992	1.121104772	5.139203	5.109069	5.482145	3.62762016	3.60313623	3.77991357	446	585	615
83891	NM_001317781	SNX25	sorting nexin 25	-1.224463579	1.121080803	3.275782	2.682907	3.456494	3.53871843	3.27409343	3.69017701	417	455	575
6715	NM_001047,NM_001324322,NM	SRD5A1	steroid 5 alpha-reductase 1	1.422349952	1.121059096	3.578307	5.087535	3.80002	3.20189991	3.6629841	3.34978031	322	612	444
554223	NR_132323	LOC554223	histocompatibility antigen-related	1.22045128	1.120717756	6.615089	8.083233	6.954489	3.21383096	3.47295962	3.36145532	325	530	448
5868	NM_001292048,NM_004162	RAB5A	RAB5A, member RAS oncogene	-1.065092129	1.120684886	20.259931	19.02882	21.323548	5.67097747	5.58183624	5.83231428	1961	2460	2703
79109	NM_001006617,NM_001006618	MAPKAP1	mitogen-activated protein kinase	1.158904476	1.120615458	28.352694	32.602807	29.168377	6.42920394	6.63967452	6.59169733	3344	5178	4609
113130	NM_080668	CDCA5	cell division cycle associated 5	1.144578684	1.120566874	27.897308	31.959204	29.36994	6.14911979	6.34137334	6.3111659	2747	4201	3786
23102	NM_015079,NM_144572	TBC1D2B	TBC1 domain family member 2B	-1.07601215	1.12047494	6.382907	5.93875	6.72273	5.27510805	5.17223519	5.4352184	1481	1839	2041
6888	NM_006755	TALDO1	transaldolase 1	-1.29268887	1.12035309	89.594498	69.374557	94.322311	6.77387117	6.40733916	6.93641176	4257	4400	5866
27241	NM_001033604,NM_001033605	BBS9	Bardet-Biedl syndrome 9	-1.020495212	1.120343369	1.808322	1.96464	1.991558	3.0724647	3.04669644	3.21791812	291	381	401
83943	NM_001244606,NM_001350959	IMMP2L	inner mitochondrial membrane pe	-1.271666482	1.120324638	6.225922	4.998365	7.056846	3.25675288	2.95035633	3.40441396	336	353	463
85021	NM_001128617,NM_001286611	REPS1	RALBP1 associated Eps domain	1.150473913	1.12030193	8.736762	10.087159	9.20682	5.12523965	5.32206616	5.28469437	1331	2046	1834
8065	NM_003478	CUL5	cullin 5	-1.045431764	1.120281785	4.992435	4.778363	5.255664	4.99417161	4.93212219	5.15317881	1212	1549	1670
23094	NM_015073	SIPA1L3	signal induced proliferation assoc	-1.11649079	1.120280457	10.944495	9.814624	11.522377	6.42963029	6.27260196	6.59169733	3345	4003	4609
10827	NM_001317993,NM_001317994	FAM114A2	family with sequence similarity 1	1.013433553	1.120183505	3.858955	3.915284	4.116833	4.18531139	4.20351422	4.34053776	675	914	930
50813	NM_001164093,NM_001164094	COPS7A	COP9 signalosome subunit 7A	-1.097646622	1.120165137	29.212267	27.251968	31.546618	5.85745527	5.7254627	6.01850255	2237	2723	3082
2921	NM_002090	CXCL3	C-X-C motif chemokine ligand 3	1.122578796	1.120128646	4.073034	4.584815	4.2902	2.48150547	2.61991364	2.6172769	180	270	248
25953	NM_001077399,NM_015488,NM	PNKD	paroxysmal nonkinesigenic dyski	1.128449763	1.120113391	29.649912	33.616519	28.060054	5.14821864	5.31793524	5.30750681	1353	2040	1864
10808	NM_001286503,NM_001286504	HSPH1	heat shock protein family H (Hsp	1.787368049	1.120041338	19.263191	34.589125	20.467799	6.63842332	7.46988406	6.80042684	3872	9247	5334
56922	NM_001293273,NM_020166,NR	MCCC1	methylcrotonoyl-CoA carboxylase	1.079854052	1.119953171	17.649101	19.092052	18.684173	5.48082991	5.58928181	5.64081394	1714	2473	2361
56886	NM_020120,NR_027671	UGGT1	UDP-glucose glycoprotein glucos	1.251135563	1.119928401	9.546301	11.960217	10.134093	6.68130478	7.00172617	6.84320946	3990	6670	5496
253143	NM_173566	PRR14L	proline rich 14 like	-1.138604365	1.119854386	3.968657	3.488523	4.177022	5.42119065	5.23857061	5.5809041	1643	1928	2263
79651	NM_001005498,NM_024599	RHBDF2	rhomboid 5 homolog 2	-1.205759874	1.119574574	21.881647	18.216308	23.063714	6.23748817	5.97146376	6.39840103	2923	3239	4025
8270	NM_006014	LAGE3	L antigen family member 3	1.343400775	1.119574452	10.08823	13.551742	10.610052	3.3677101	3.75752359	3.51569869	366	657	504
23506	NM_001318819,NM_015349	BICRAL	BRD4 interacting chromatin remo	-1.285082178	1.119258688	1.749681	1.363184	1.838893	3.65121051	3.3216324	3.80150191	454	472	625
84196	NM_001032730,NM_001330394	USP48	ubiquitin specific peptidase 48	-1.31470327	1.119204658	22.590352	17.451549	24.719193	6.4436299	6.05408526	6.60434238	3378	3433	4650
106633808	NR_132759	SNORD143	small nucleolar RNA, C/D box 14	1.117628228	1.119091484	26.413483	29.481592	27.826292	1.56621863	1.67459501	1.67590018	77	115	106
11176	NM_001300905,NM_001351156	BAZ2A	bromodomain adjacent to zinc fin	-1.018934752	1.118866146	22.872274	22.478761	24.053698	7.64499974	7.61807417	7.80627344	7819	10253	10760
10577	NM_006432	NPC2	NPC intracellular cholesterol tran	2.327178749	1.11871851	129.784424	302.360474	136.421463	6.83726752	8.04865591	6.99777889	4450	13837	6123
5980	NM_001286431,NM_001286432	REV3L	REV3 like, DNA directed polymer	1.116435394	1.118596034	1.425053	1.593764	1.499838	4.03426408	4.18397654	4.18661421	604	901	831
5720	NM_001281528,NM_001281529	PSME1	proteasome activator subunit 1	-1.165866892	1.118531463	222.27712	191.179053	233.455034	7.78869429	7.56837004	7.94961054	8642	9904	11889
2635	NM_001319179,NM_001319180	GBP3	guanylate binding protein 3	-1.24133102	1.118472685	11.888651	9.436062	12.207635	5.11679207	4.81487642	5.27392385	1323	1424	1820
5467	NM_001171818,NM_001171819	PPARD	peroxisome proliferator activated	-1.263526987	1.118305648	11.827227	9.651904	12.593243	5.43059347	5.10190141	5.58837507	1654	1749	2275
59343	NM_021627	SENP2	SUMO1/sentrin/SMT3 specific pe	-1.030766414	1.118132957	21.562935	20.941738	22.660336	6.08885029	6.04578304	6.24770733	2633	3413	3621
57224	NM_001144060,NM_020464	NHSL1	NHS like 1	-1.249111825	1.118095063	2.056348	1.64777	2.305638	4.00022383	3.70154644	4.15174023	589	630	810
10161	NM_001162497,NM_001162498	LPAR6	lysophosphatidic acid receptor 6	1.796479631	1.117896398	2.869483	4.544372	3.008372	2.98698269	3.74936649	3.12850473	272	653	374
678	NM_006887	ZFP36L2	ZFP36 ring finger protein like 2	-1.122714223	1.117869353	20.272209	18.075346	21.297813	6.20746949	6.04286594	6.366167	2862	3406	3935
348094	NM_182703	ANKDD1A	ankyrin repeat and death domain	1.172045045	1.117847845	1.13861	1.194743	1.15835987	2.15835987	2.33928445	2.28470378	136	213	187
50640	NM_001256007,NM_001256008	PNPLA8	patatin like phospholipase domai	-2.113647368	1.11779529	7.987996	3.783793	8.45119	5.24150548	4.20351422	5.39814864	1446	914	1988
5880	NM_002872	RAC2	ras-related C3 botulinum toxin su	1.264573948	1.117616064	22.075249	27.965075	23.196112	5.06613802	5.39577602	5.22203654	1276	2156	1754
149111	NM_001322302,NM_001322303	CNIH3	cornichon family AMPA receptor	2.372607137	1.117615343	0.442627	1.13165	0.47996	1.01352966	1.77444288	1.09694451	40	127	55
729461	NR_136569	LINC01660	long intergenic non-protein codin	2.031357075	1.117533218	0.43705	0.91157	0.469186	0.86002952	1.41062362	0.93448595	32	87	44
26508	NM_014571	HEYL	hes related family bHLH transcrip	-1.281069912	1.117396599	0.152366	0.118974	0.160286	0.68823103	0.56226574	0.75138414	24	25	33
220594	NR_003554	USP32P2	ubiquitin specific peptidase 32 ps	1.24629254	1.117396599	0.128978	0.160592	0.134458	0.68823103	0.81765451	0.75138414	24	40	33
441687	NR_033777	TEX21P	testis expressed 21, pseudogene	-1.105237558	1.117396599	0.595628	0.542854	0.627163	0.68823103	0.63490936	0.75138414	24	29	33
5428	NM_001126131,NM_002693	POLG	DNA polymerase gamma, catalyt	-1.047148223	1.117383282	22.536786	21.547727	23.647332	6.62023516	6.55445888	6.77882228	3823	4878	5254
3727	NM_001286968,NM_005354	JUND	JunD proto-oncogene, AP-1 trans	-1.177161136	1.11726363	34.03791	28.949621	35.74024	6.04611298	5.81465343	6.20379585	2555	2900	3511
84988	NM_001329442,NM_001329443	PPP1R16A	protein phosphatase 1 regulatory	1.337467473	1.117192995	20.879947	28.049356	22.024886	5.90606357	6.31950074	6.06342327	2315	4137	3181
54680	NM_001170670,NM_017953	ZNHIT6	zinc finger HIT-type containing 6	1.355524668	1.117123586	5.810472	7.889563	6.10716	5.17287613	5.60122869	5.32847781	1377	2494	1892
8805	NM_003852,NM_015905	TRIM24	tripartite motif containing 24	-1.372882559	1.116861069	8.140257	5.956666	8.480964	5.01591383	4.57519242	5.1707038	1231	1198	1691
57480	NM_001029884,NM_001329798	PLEKHG1	pleckstrin homology and RhoGEF	1.468208398	1.116844668	1.547288	1.640515	1.448662	3.06370142	3.5618123	3.20501631	289	567	397
7001	NM_005809	PRDX2	peroxiredoxin 2	1.509594812	1.116715081	60.640343	91.664841	63.660229	5.96136766	6.54771313	6.11821373	2407	4855	3306
101929693	NR_110861	RARA-AS1	RARA antisense RNA 1	1.455210802	1.11668441	1.316893	1.9335	1.385436	1.73025772	2.12902957	1.84344662	91	177	125
79029	NM_001323640,NM_024063,NR	SPATA5L1	spermatogenesis associated 5 lik	-1.062810024	1.116673831	4.971417	4.67403	5.102036	3.7528388	3.67164303	3.90085366	490	616	673
8821	NM_001101669,NM_001331040	INPP4B	inositol polyphosphate-4-phosph	-1.801125972	1.11666975	9.502056	8.483742	12.925177	6.25491539	5.4210308	6.41214851	2959	2195	4064
286333	NR_024366	FAM225A	family with sequence similarity 2	1.259846556	1.116575801	0.710615	0.896019	0.745379	2.32896139	2.6019054	2.45783582	158	266	217
55561	NM_017525	CDC42BPG	CDC42 binding protein kinase ga	1.415322384	1.116464373	6.954047	9.85089	7.29566	5.10616256	5.59498314	5.26073633	1313	2483	1803
2804	NM_001256486,NM_001256487	GOLGB1	golgin B1	1.32886977	1.116444915	9.962557	13.257699	10.47757	6.78057164	7.18752899	6.93811829	4277	7594	5873
10762	NM_007172,NM_153645	NUP50	nucleoporin 50	-1.056450674	1.11634442	15.304863	14.508621	16.07993	6.30085049	6.22265489	6.45772977	3056	3865	4196
730098	NM_001320037,NM_001320038	LOC730098	uncharacterized LOC730098	4.906126483	1.11631497	0.123995	0.710537	0.143831	0.26755841	1.00745609	0.29607837	8	53	11
84206	NM_032246	MEX3B	mex-3 RNA binding family memb	-1.330220287	1.11631497	0.059069	0.044423	0.056624	0.26755841	0.20477631	0.29607837	8	8	11
89781	NM_001349896,NM_001349898	HPS4	HPS4, biogenesis of lysosomal o	1.099571791	1.116217921	31.33594	34.129012	32.965866	7.25340848	7.38949612	7.41104519	5951	8743	8170
54726	NM_001102653,NM_017493	OTUD4	OTU deubiquitinase 4	-1.116077901	1.115988761	15.208982	13.758705	16.309611	6.6873789	6.53056071	6.84424993	4007	4797	5500
83933	NM_001159286,NM_032019	HDAC10	histone deacetylase 10	1.129319475	1.115977077	13.568853	15.359101	14.252629	5.19711926	5.36807488	5.35134557	1401	2114	1923
79576	NM_024528	NKAP	NFKB activating protein	-1.222804415	1.115894131	17.265362	14.143755	18.117231	4.79015949	4.51150549	4.94294958	1047	1144	1437
389856	NM_001145073	USP27X	ubiquitin specific peptidase 27, X	1.437250659	1.115697887	0.49079	0.707343	0.517087	1.2010231	1.52015496	1.29257653	51	98	70
285671	NM_001113561,NM_001323291	RNF180	ring finger protein 180	-4.230024687	1.115697887	0.331298	0.087913	0.292526	1.2010231	0.38406669	1.29257653	51	16	70
8639	NM_001277731,NM_001277732	AOC3	amine oxidase, copper containing	1.583839141	1.115614508	1.2544	2.074463	1.252959	2.48150547	3.04669644	2.61239943	180	381	247
65125	NM_001184985,NM_014823,NM	WNK1	WNK lysine deficient protein kina	-1.046549999	1.115480757	14.950096	13.623938	14.911461	7.1593717	7.0941989	7.31599513	5573	7115	7646
220729	NR_003266	LOC220729	succinate dehydrogenase comple	1.227680212	1.115247522	35.569603	43.731369	37.283791	5.75230681	6.04328303	5.90691023	2077	3407	2849
148156	NM_001304350,NM_144693	ZNF558	zinc finger protein 558	1.116078387	1.11522907	11.632252	12.994218	12.252972	5.32912486	5.48383604	5.48276272	1539	2295	2111
23432	NM_001267609,NM_001267610	GPR161	G protein-coupled receptor 161	-1.37902883	1.115077102	0.899333	0.652969	0.962328	3.05929974	2.65962865	3.19852188	288	279	395
150864	NM_173511	FAM117B	family with sequence similarity 1	1.19210021	1.115010539	1.899798	2.270209	1.992748	3.54817338	3.78172178	3.6924898	420	669	576
6717	NM_001256891,NM_001256892	SRI	sorcin	-1.491422847	1.1149276	38.054659	25.592005	38.010594	5.93458154	5.36940611	6.08910502	2362	2116	3239
131408	NM_001171093,NM_144635	FAM131A	family with sequence similarity 13	-1.193167894	1.114779027	9.762029	8.391384	10.413416	4.6331688	4.38952806	4.78394543	935	1047	1282
65265	NM_023080	C8orf33	chromosome 8 open reading fran	1.155638669	1.11472132	12.01332	13.895562	12.584159	4.98259535	5.1851439	5.13458808	1202	1856	1648
25945	NM_001243286,NM_001243288	NECTIN3	nectin cell adhesion molecule 3	-1.750071196	1.114667473	1.470869	0.844404	1.555551	3.0370876	2.35549733	3.17555833	283	216	388
55051	NM_017970	NRDE2	NRDE-2, necessary for RNA inte	-1.169083636	1.114666847	5.886529	5.041704	6.16788	4.58498718	4.36970972	4.7354205	903	1032	1238
91661	NM_001040185,NM_001350495	ZNF765	zinc finger protein 765	-1.347336814	1.114483163	2.02841	1.815919	2.749195	3.3316643	2.95035633	3.47328584	356	353	488
54790	NM_001127208,NM_017628	TET2	tet methylcytosine dioxygenase 2	1.173996566	1.114345435	2.759882	3.209895	2.886158	4.75384679	4.97734988	4.90456173	1020	1600	1398
23116	NM_001308120,NM_015091,NR	TOGARAM1	TOG array regulator of axonemal	1.231518093	1.114163937	3.156115	3.841909	3.306238	4.34028863	4.62731136	4.48895469	756	1244	1036
101735302	NR_103720	STAG3L5P	stromal antigen 3-like 5 pseudog	-1.265467165	1.114062463	3.872436	3.063526	4.039524	1.90722898	1.66595383	2.02324736	108	114	148
51702	NM_016233	PADI3	peptidyl arginine deiminase 3	1.225685545	1.113910729	9.529113	11.695235	9.978086	4.93533846	5.22024304	5.08615607	1162	1903	1592
81627	NM_001202423,NM_030934	TRMT1L	tRNA methyltransferase 1 like	-2.116128245	1.11376737	9.69342	4.583559	10.142662	5.41258913	4.36837876	5.56458305	1633	1031	2237
1390	NM_001267562,NM_001267563	CREM	cAMP responsive element modul	1.049151213	1.11371015	3.385687	3.459407	3.427781	2.71768687	2.77662633	2.85055786	219	307	300
441054	NM_001114357,NM_001346007	C4orf47	chromosome 4 open reading fran	4.117891549	1.11370872	0.758663	3.081098	0.709118	0.7550755	1.94127715	0.82068496	27	149	37
26157	NM_015660	GIMAP2	GTPase, IMAP family member 2	-1.619244129	1.11339273	6.513645	4.024845	6.822871	3.34619087	2.73592201	3.48667429	360	297	493

83540	NM_031423,NM_145697	NUF2	NUF2, NDC80 kinetochore comp	-1.219468453	1.113286889	47.248621	38.056323	48.297853	6.47371098	6.19100706	6.62688834	3450	3780	4724
728407	NR_029388	PARGP1	poly(ADP-ribose) glycohydrolase	-1.085522988	1.113156728	2.338548	2.155075	2.457112	2.10810423	2.01797632	2.22843599	130	160	178
57590	NM_020830	WDFY1	WD repeat and FYVE domain co	1.011771965	1.113148949	13.325111	13.495859	13.939559	5.94296841	5.95958045	6.09523617	2376	3212	3253
93663	NM_033515	ARHGAP18	Rho GTPase activating protein 1	-1.805947098	1.113114536	13.390293	7.42182	14.001668	5.54283855	4.71474432	5.69430137	1791	1325	2452
80173	NM_001099222,NM_001099223	IFT74	intraflagellar transport 74	1.282958386	1.11307522	2.396398	2.611476	2.3248	3.54817338	3.88026307	3.69017701	420	720	575
51307	NM_001135647,NM_001350194	FAM53C	family with sequence similarity 5	1.531691999	1.112920018	14.703154	22.548794	15.37323	5.99805187	6.60534266	6.15011624	2470	5055	3381
8612	NM_003712,NM_177526,NM_17	PLPP2	phospholipid phosphatase 2	1.117411253	1.112564599	116.559259	130.361979	121.879825	7.23083465	7.38998812	7.38375446	5858	8746	8016
23168	NM_015138	RTF1	RTF1 homolog, Paf1/RNA polym	1.072628178	1.112551477	11.497973	12.345921	12.021964	5.83831485	5.9377612	5.98964105	2207	3163	3020
55588	NM_001317770,NM_017592,NR	MED29	mediator complex subunit 29	-1.006398559	1.112538003	12.115127	12.045638	12.662217	5.46427804	5.45528479	5.61483275	1694	2249	2318
51175	NM_016262	TUBE1	tubulin epsilon 1	-1.166104397	1.11250913	7.256465	6.233479	7.591331	4.10857187	3.90066256	4.25393069	638	731	873
292	NM_001152	SLC25A5	solute carrier family 25 member 5	1.088019229	1.112419421	245.436783	267.339111	256.568787	8.33916542	8.46051002	8.49241727	12675	18426	17342
57524	NM_020764	CASKIN1	CASK interacting protein 1	1.312456269	1.112374947	0.257521	0.343934	0.272685	1.29387258	1.5392062	1.38699077	57	100	78
644192	NR_102743,NR_102744,NR_12	NR2F2-AS1	NR2F2 antisense RNA 1	2.123964196	1.11226476	0.850025	2.283071	1.430079	0.97665845	1.61299082	1.05442063	38	108	52
162681	NM_001288980,NM_001288981	C18orf54	chromosome 18 open reading fra	-1.343635133	1.112168546	2.431224	2.101456	2.90707	3.85794988	3.46550967	4.00128413	530	527	725
105372480	NR_144444	LOC105372480	uncharacterized LOC105372480	-1.123898843	1.112073738	3.943922	3.511239	4.117048	1.88750088	1.76638114	2.0010274	106	126	145
51043	NM_001252406,NM_001256455	ZBTB7B	zinc finger and BTB domain cont	1.131147228	1.111958286	10.302415	11.725534	10.840325	5.28927297	5.46278758	5.43866688	1496	2261	2046
5565	NM_005399,NR_103870,NR_10	PRKAB2	protein kinase AMP-activated no	1.288267733	1.111827439	7.010826	9.041225	7.49225	5.25888453	5.6158843	5.4080336	1464	2520	2002
9882	NM_001286658,NM_001286659	TBC1D4	TBC1 domain family member 4	-1.367988657	1.111686897	3.311808	2.42221	3.564962	4.44184742	4.01394871	4.58793254	814	795	1113
7175	NM_003292	TPR	translocated promoter region, nu	-1.163981103	1.111673127	40.404343	34.751522	42.209915	8.58058789	8.36213639	8.73294301	14991	17208	20497
1073	NM_001243645,NM_021914,NM	CFL2	cofilin 2	-1.342768357	1.111596646	14.563837	10.972499	15.337238	5.55851796	5.14373402	5.70808361	1811	1802	2476
84740	NR_026892	AFAP1-AS1	AFAP1 antisense RNA 1	-1.040498206	1.111575323	13.767157	13.247407	14.381598	6.52488929	6.46824743	6.67592604	3576	4592	4889
1767	NM_001369	DNAH5	dynein axonemal heavy chain 5	2.197524996	1.111495377	0.213392	0.472081	0.223452	2.09095564	3.02995795	2.20918158	128	376	175
79230	NM_001044387,NM_001044388	ZNF557	zinc finger protein 557	1.485903084	1.111446771	1.506343	2.244682	1.57601	3.29469475	3.81727465	3.43235848	346	687	473
5828	NM_000318,NM_001079867,NM	PEX2	peroxisomal biogenesis factor 2	-1.049385551	1.111420152	7.502537	7.151313	7.802321	5.03509348	4.9677141	5.18309257	1248	1589	1706
81554	NM_001281441,NM_030798,NM	RCC1L	RCC1 like	-1.368989642	1.111327387	12.217901	8.913902	12.734954	4.88772286	4.45243375	5.03513062	1123	1096	1535
8844	NM_014238	KSR1	kinase suppressor of ras 1	-1.062980524	1.111303727	0.632099	0.595735	0.663117	1.91699278	1.85267538	2.03057861	109	137	149
10572	NM_006427,NM_021709	SIVA1	SIVA1 apoptosis inducing factor	1.226786881	1.111200618	75.496414	92.669333	78.85905	5.84472325	6.13497237	5.99433536	2217	3634	3030
57721	NM_020961	METTL14	methyltransferase like 14	-1.018065741	1.11112202	10.460954	10.290622	10.924093	5.1981206	5.17299774	5.34621347	1402	1840	1916
3550	NM_006083	IK	IK cytokine, down-regulator of HL	1.031400526	1.111112064	73.008919	75.392441	76.234413	7.11446546	7.15875401	7.26543125	5401	7443	7381
93134	NM_001330365,NM_152289	ZNF561	zinc finger protein 561	1.039843622	1.111080528	6.064037	6.313373	6.338333	4.79810635	4.85249002	4.94489092	1053	1463	1439
253512	NM_001010875,NM_001286806	SLC25A30	solute carrier family 25 member 3	-1.365322253	1.11088235	1.615153	1.16813	1.686256	2.78853894	2.41343275	2.91931156	232	227	317
9747	NM_001206938,NM_001206941	TCAF1	TRPM8 channel associated facto	-1.489713958	1.110803213	13.417622	9.041477	14.016403	6.23553875	5.669824	6.38523632	2919	2618	3988
54862	NM_017721	CC2D1A	coiled-coil and C2 domain contai	1.122520951	1.110772068	21.288944	23.930748	22.227791	6.25683881	6.42152616	6.40652436	2963	4444	4048
7337	NM_000462,NM_130838,NM_13	UBE3A	ubiquitin protein ligase E3A	-1.085366088	1.110655772	20.158401	18.580029	20.556326	6.61986158	6.50292811	6.76981528	3822	4705	5221
6276	NM_002962	S100A5	S100 calcium binding protein A5	1.318679586	1.110433058	6.032606	7.972674	6.288875	2.37218925	2.70251092	2.49540988	164	289	224
157313	NM_001317906,NM_001317907	CDCA2	cell division cycle associated 2	1.048238986	1.110413223	9.354243	9.90487	9.830909	5.17389443	5.24002681	5.32102311	1378	1930	1882
2618	NM_000819,NM_001136005,NM	GART	phosphoribosylglycinamide formy	-1.273451811	1.110235779	50.251946	39.557826	53.85037	7.24930837	6.90314758	7.39923534	5934	6226	8103
83719	NM_001145524,NM_031477	YPEL3	yippee like 3	-1.029824594	1.110080943	22.236811	21.157948	23.942733	5.07161207	5.03048889	5.21802858	1281	1662	1749
79658	NM_024605	ARHGAP10	Rho GTPase activating protein 1	1.13738974	1.110079207	4.986056	5.68258	5.202935	3.98637962	4.16109929	4.1280136	583	886	796
25850	NM_001242472,NM_001242474	ZNF345	zinc finger protein 345	-1.427219191	1.110048108	1.558104	1.184445	1.729929	2.3142594	1.9196316	2.43591723	156	146	213
441307	NR_033963	HRAT92	heart tissue-associated transcript	-3.456357013	1.109890274	0.381657	0.111541	0.40292	1.21691895	0.46599441	1.30472178	52	20	71
55596	NM_001350935,NM_001350936	ZCCHC8	zinc finger CCHC-type containing	-1.533191916	1.109820029	12.816046	8.364552	13.849886	5.81956788	5.21654942	5.96737086	2178	1898	2973
221061	NM_001010924	FAM171A1	family with sequence similarity 1	1.364669009	1.109784159	4.462334	6.098587	4.651462	4.18329013	4.61052206	4.32571472	674	1229	920
728190	NR_024397	NUTM2A-AS1	NUTM2A antisense RNA 1	-1.215047711	1.109698397	6.694094	5.511435	6.999055	2.67229041	2.43901707	2.79997431	211	232	288
10897	NM_001300861,NM_020470	YIF1A	Yip1 interacting factor homolog A	1.01365925	1.109654814	49.111804	50.342537	50.396207	5.7073003	5.72650194	5.85468784	2012	2725	2746
124446	NM_001083613,NM_194280	TMEM219	transmembrane protein 219	-1.070582969	1.109378494	39.869351	37.264118	41.56438	5.31165148	5.21580956	5.45782748	1520	1897	2074
1355	NM_001320974,NM_001320975	COX15	COX15, cytochrome c oxidase as	-1.324924869	1.10919747	9.344494	6.936555	9.197545	5.53334846	5.13749687	5.67980348	1779	1794	2427
84924	NM_001145343,NM_001145344	ZNF566	zinc finger protein 566	-1.494924849	1.109179761	2.200972	1.471608	2.289593	3.61567874	3.09255667	3.75357377	442	395	603
5631	NM_001204402,NM_002764	PRPS1	phosphoribosyl pyrophosphate s	1.433188373	1.109104345	13.192305	18.871609	13.719701	4.83591088	5.33983141	4.9803424	1082	2072	1476
113655	NM_138431,NR_130120	MFSD3	major facilitator superfamily dom	1.233708333	1.109033148	10.681876	13.170133	10.923171	4.11282598	4.39998757	4.25393069	640	1055	873
90627	NM_001243466,NM_001243474	STARD13	StAR related lipid transfer domai	-1.212257209	1.108447303	1.461758	1.146766	1.597854	3.24517298	2.99933041	3.37879258	333	367	454
55659	NM_017879	ZNF416	zinc finger protein 416	1.330487755	1.10842362	5.908487	7.874791	6.152996	3.94879876	4.33742297	4.08816658	567	1008	773
5585	NM_002741,NM_213560	PKN1	protein kinase N1	-1.35630742	1.108351112	60.46898	44.659294	63.100421	7.51060032	7.07372461	7.65824389	7120	7014	9706
55681	NM_001317784,NM_001330253	SCYL2	SCY1 like pseudokinase 2	-1.064594036	1.108248748	9.807559	9.231502	10.285511	5.78732769	5.69870608	5.93306293	2129	2672	2902
23099	NM_001135776,NM_014007	ZBTB43	zinc finger and BTB domain cont	-1.845864909	1.108202082	7.948126	4.258554	8.238091	5.53810131	4.67976064	5.68329628	1785	1292	2433
84265	NM_001330685,NM_032305	POLR3GL	RNA polymerase III subunit G lik	-1.340152478	1.108121259	14.471303	10.803248	15.091644	4.14017606	3.74527057	4.2803093	653	651	890
115330	NM_001303473,NM_001303474	GPR146	G protein-coupled receptor 146	-1.228243141	1.107631023	0.977756	0.848508	1.012636	1.602974	1.41062362	1.70368302	80	87	109
164832	NM_198461	LONRF2	LON peptidase N-terminal domai	1.468176332	1.107631023	0.149117	0.221013	0.156437	1.602974	1.99745948	1.70368302	80	157	109
101927763	NR_133006	OLFM5P	olfactomedin family member 5, p	-1.12914463	1.107609037	0.241514	0.216692	0.242788	0.35634914	0.31941552	0.39027104	11	13	15
339456	NM_178545	TMEM52	transmembrane protein 52	-1.316388626	1.107459963	1.877441	1.420059	1.956106	1.46336426	1.22307764	1.5590879	69	70	94
200844	NM_001351530,NM_001351531	C3orf67	chromosome 3 open reading fran	1.131173692	1.107362227	1.738178	1.916364	1.702858	2.44147625	2.58825016	2.56269567	174	263	237
147991	NM_001172774,NM_207325	DPY19L3	dpy-19 like 3 (C. elegans)	1.005994953	1.107077189	4.955924	5.004398	5.167575	4.91111656	4.91945464	5.05324052	1142	1535	1555
8437	NM_001193520,NM_001193521	RASAL1	RAS protein activator like 1	-1.252021906	1.107037508	6.033818	4.819055	6.2918	4.49984402	4.19152238	4.64038721	849	906	1156
55184	NM_001099407,NM_001351683	DZANK1	double zinc ribbon and ankyrin re	2.121142392	1.107002363	1.644154	3.525393	1.735453	2.74535098	3.71207778	2.87112187	224	635	305
2055	NM_018941	CLN8	CLN8, transmembrane ER and E	-1.07429652	1.107000662	3.187095	2.970933	3.317755	4.53988763	4.44108339	4.68055367	874	1087	1190
126374	NM_001080436	WTIP	WT1 interacting protein	-2.065993873	1.106907879	1.533392	0.742884	1.599975	2.10810423	1.37924953	2.22204636	130	84	177
54972	NM_017870,NM_178031	TMEM132A	transmembrane protein 132A	1.382606584	1.106729005	14.212139	19.685785	14.784671	5.63593514	6.09529677	5.77944455	1913	3534	2604
346157	NM_001076781,NM_001322288	ZNF391	zinc finger protein 391	-1.349996434	1.106708229	1.222013	0.858898	1.269589	2.55212659	2.20257223	2.67455554	191	189	260
3491	NM_001554	CYR61	cysteine rich angiogenic inducer	2.034511157	1.106538515	57.649742	117.418304	59.945972	7.01909594	8.03812673	7.16408135	5053	13736	6877
3064	NM_002111	HTT	huntingtin	-1.027220461	1.106445034	10.039184	9.782814	10.438313	7.05214523	7.01369446	7.19703378	5171	6726	7037
54941	NM_017831	RNF125	ring finger protein 125	-1.613062069	1.106184799	0.540657	0.334993	0.560671	2.03825186	1.5486383	2.14982678	122	101	166
130888	NM_174899	FBXO36	F-box protein 36	1.007504051	1.106161076	1.926152	1.94427	2.007298	2.65489157	2.66397469	2.77836293	208	280	283
23063	NM_001318328,NM_015045	WAPL	WAPL cohesin release factor	-1.396654841	1.106054972	10.441334	7.483545	10.849883	6.02313036	5.5499035	6.16643088	2514	2405	3420
26073	NM_001290145,NM_015584	POLDIP2	DNA polymerase delta interacting	1.250693362	1.105471664	29.577172	37.062555	30.715453	6.31615031	6.63525454	6.45908879	3089	5162	4200
4669	NM_000263	NAGLU	N-acetyl-alpha-glucosaminidase	1.021230925	1.105346963	16.995955	17.378994	17.655001	5.55617688	5.58585016	5.69775929	1808	2467	2458
442247	NM_001013734	RFPL4B	ret finger protein like 4B	-1.112897211	1.10527712	0.343955	0.300408	0.352893	0.71085839	0.65251278	0.76902392	25	30	34
4774	NM_001134673,NM_001145511	NFIA	nuclear factor I A	1.242829925	1.105268393	1.372175	1.719209	1.432443	3.7744826	4.06742162	3.90883222	498	827	677
7106	NM_001025234,NM_001025235	TSPAN4	tetraspanin 4	-1.10169138	1.105251303	24.722942	22.467551	25.81721	5.12418641	4.98865526	5.26462754	1330	1613	1808
2519	NM_032020	FUCA2	fucosidase, alpha-L- 2, plasma	1.41559784	1.105214948	29.598255	41.949509	30.741013	6.21787444	6.71359985	6.36036033	2883	5453	3919
79738	NM_024685	BBS10	Bardet-Biedl syndrome 10	1.049016665	1.105201237	4.486447	4.714143	4.662859	4.05872616	4.12372395	4.19479474	615	862	836
140775	NM_144775	SMCR8	Smith-Magenis syndrome chrom	1.273268356	1.105169062	2.179779	2.780954	2.264079	4.22121804	4.55310322	4.35812668	693	1179	942
55041	NM_001100623,NM_001267062	PLEKHB2	pleckstrin homology domain cont	-1.193690775	1.105114153	33.361064	28.006602	34.6701	7.13996212	6.88650747	7.28318703	5498	6154	7473
3590	NM_001142784,NR_052010	IL11RA	interleukin 11 receptor subunit al	1.373147035	1.105060556	3.066327	4.166077	3.213018	2.64317467	3.03667667	2.76523898	206	378	280
751071	NM_001043229	METTL12	methyltransferase like 12	1.227519829	1.10505704	1.206085	1.481826	1.249361	1.39514558	1.58576189	1.4863075	64	105	87
115704	NM_001159944,NM_145245	EVI5L	ecotropic viral integration site 5 li	1.508349153	1.105002564	4.045456	6.026238	4.139397	3.98405928	4.54605682	4.11944434	582	1173	791
85450	NM_001272012,NM_001272013	ITPRIP	inositol 1,4,5-trisphosphate recep	-3.506625209	1.104696951	2.851119	0.811166	2.962602	4.27725131	2.64211237	4.41385336	722	275	981
84888	NM_032802	SPPL2A	signal peptide peptidase like 2A	-1.061266407	1.104638833	19.260134	18.171633	19.993948	5.26940291	5.18589965	5.40944022	1475	1857	2004
116412	NM_138466,NR_049780	ZNF837	zinc finger protein 837	-1.461250138	1.104540267	2.587412	1.788807	2.694105	2.59533164	2.15396266	2.71606984	198	181	269
8078	NM_001098536,NM_003481	USP5	ubiquitin specific peptidase 5	1.036665912	1.104441395	21.971035	22.755368	22.798263	6.08181474	6.13301429	6.22312179	2620	3629	3559
5442	NM_005035	POLRMT	RNA polymerase mitochondrial	-1.089157038	1.104434301	16.577408	15.234573	17.202745	5.96195729	5.84080171	6.10308098	2408	2954	3271
79814	NM_024758	AGMAT	agmatinase	-1.07286781	1.104411551	0.875453	0.817652	0.906253	1.88750088	1.81408935	1.99354404	106	132	144
7706	NM_005082	TRIM25	tripartite motif containing 25	-1.00580982	1.104332385	32.340275	32.188511	33.560757	7.50676645	7.49845487	7.64919343	7101	9433	9645
23039	NM_015024	XPO7	exportin 7	-1.075440044	1.104254973	15.375515	14.314628	15.957021	6.20597692	6.10251937	6.34720926	2859	3552	3883
6573	NM_001205206,NM_001205207	SLC19A1	solute carrier family 19 member	1.622482509	1.104046101	6.067081	10.577496	6.452031	4.1064401	4.77223569	4.24134834	637	1381	865
1537	NM_001916	CYC1	cytochrome c1	1.029679124	1.103971544	119.199471	122.880272	123.663681	7.17216785	7.21407511	7.31393113	5623	7736	7635
89849	NM_001318766,NM_033388	ATG16L2	autophagy related 16 like 2	-1.07040371	1.103928963	13.461061	12.697131	14.057379	4.89885139	4.80408772	5.03695187	1132	1413	1537
123228	NM_001166340,NM_001172109	SENP8	SUMO/sentrin peptidase family n	-1.311646632	1.103852208	1.67375	1.376025	1.866135	1.91699278	1.63971536	2.02324736	109	111	148
4795	NM_001144961,NM_001144962	NFKBIL1	NFKB inhibitor like 1	-1.054287449	1.103688062	11.040891	10.453475	11.403842	4.07189633	4.00026496	4.20617012	621	787	843
64781	NM_022766	CERK	ceramide kinase	1.442844648	1.103586558	3.832813	5.538813	3.976594	4.13809052	4.64170569	4.27260082	652	1257	885

8899	NM_003913,NR_146783,NR_14(	PRPF4B	pre-mRNA processing factor 4B	1.024778784	1.103498917	17.094267	16.589324	16.861383	6.62471065	6.65967059	6.76542793	3835	5251	5205
5965	NM_002907,NM_032941	RECQL	RecQ like helicase	-1.035932359	1.103447536	15.655955	14.634288	15.830049	5.82151855	5.77150262	5.96114983	2181	2813	2960
261734	NM_001291593,NM_001291594	NPHP4	nephrocystin 4	1.519489955	1.103405524	2.892902	4.466465	3.008371	3.9320442	4.50304006	4.06514778	560	1137	760
1838	NM_001256303,NM_001256304	DTNB	dystrobrevin beta	1.716046563	1.103382653	3.000777	4.878954	2.920061	2.95890107	3.65863507	3.08339379	266	610	361
130827	NM_001321343,NM_001321344	TMEM182	transmembrane protein 182	1.008600136	1.10337104	1.621628	1.636311	1.677509	2.66651408	2.67693481	2.78704637	210	283	285
8563	NM_001002877,NM_001002878	THOC5	THO complex 5	1.176602595	1.103272706	8.67022	10.16795	8.943447	4.47196531	4.69682839	4.60767403	832	1308	1129
642236	NR_033907	FRG1JP	FSHD region gene 1 family mem	-1.647779888	1.103150884	0.731166	0.450972	0.765676	1.04948198	0.72085408	1.12461302	42	34	57
57472	NM_001303241	CNOT6	CCR4-NOT transcription comple:	-1.119467333	1.103101252	14.161982	12.665086	14.680822	6.46583364	6.30496449	6.60587691	3431	4095	4655
6300	NM_001303252,NM_002969	MAPK12	mitogen-activated protein kinase	1.391759903	1.103080065	14.292366	19.927496	14.804229	4.7808325	5.2429348	4.91747151	1040	1934	1411
729438	NM_001145064	CASTOR2	cytosolic arginine sensor for mTC	-1.104590658	1.102956767	0.888378	0.805142	0.922328	2.98698269	2.86232771	3.11132089	272	329	369
79751	NM_001191060,NM_001191061	SLC25A22	solute carrier family 25 member 2	1.339382481	1.10289078	14.896648	19.785566	15.096804	5.33003867	5.74251508	5.46798836	1540	2756	2089
10728	NM_001282601,NM_001282602	PTGES3	prostaglandin E synthase 3	1.008947237	1.102806454	217.242801	220.225004	226.689828	8.75980146	8.77262276	8.90067166	16979	22889	23030
145173	NM_194318	B3GLCT	beta 3-glucosyltransferase	1.152714143	1.102725602	2.154935	2.490435	2.238852	3.30216484	3.48774509	3.42958826	348	536	472
81876	NM_030981	RAB1B	RAB1B, member RAS oncogene	1.146464348	1.102679725	63.698811	73.115562	66.008751	6.93390241	7.12958981	7.07382021	4761	7293	6457
148418	NM_001010971,NM_001134663	SAMD13	sterile alpha motif domain contai	2.713343111	1.102604577	0.233651	0.685981	0.242359	0.43999062	0.97983233	0.47868902	14	51	19
333929	NM_178310	SNAI3	snail family transcriptional repres	-1.434126113	1.102604577	0.209274	0.138107	0.217775	0.43999062	0.31941552	0.47868902	14	13	19
29901	NM_013299	SAC3D1	SAC3 domain containing 1	1.338681236	1.102485104	11.524933	15.452095	11.949615	4.21727226	4.61838139	4.35082401	691	1236	937
27230	NM_014445	SERP1	stress associated endoplasmic re	-1.056211557	1.102338257	45.870098	43.477554	47.518764	7.15654125	7.07820918	7.29617184	5562	7036	7541
7318	NM_003335	UBA7	ubiquitin like modifier activating e	1.026187834	1.10233756	16.511545	16.960558	17.098881	5.76181945	5.79843744	5.89992208	2091	2867	2835
3956	NM_002305	LGALS1	galectin 1	1.047973121	1.102313081	1147.634644	1204.039917	1188.812744	9.28657452	9.35407076	9.42689473	24479	34276	33188
9842	NM_014798,NR_027774,NR_02	PLEKHM1	pleckstrin homology and RUN do	-1.455909087	1.102084323	15.262658	10.395235	15.661441	6.29243623	5.75886043	6.43096978	3038	2788	4118
23389	NM_015335	MED13L	mediator complex subunit 13 like	-1.086166266	1.10206271	5.963424	5.496371	6.176216	5.79330362	5.6762924	5.9311056	2138	2630	2898
25813	NM_015380	SAMM50	SAMM50 sorting and assembly m	1.446846888	1.101924788	17.397804	25.201181	18.009735	4.94252643	5.46091554	5.07821843	1168	2258	1583
533	NM_001039457,NM_001294333	ATP6V0B	ATPase H+ transporting V0 subu	-1.077317807	1.101814668	73.738693	62.24757	65.884909	6.63509978	6.52877463	6.77364331	3863	4791	5235
25930	NM_001304482,NM_015466	PTPN23	protein tyrosine phosphatase, no	-1.024133049	1.101734079	9.732584	9.5287	10.103854	5.67241926	5.63869677	5.80958828	1963	2561	2660
7760	NM_001134655,NM_004220,NR	ZNF213	zinc finger protein 213	1.061316578	1.101694184	3.766963	3.972769	3.913632	3.71416279	3.79367035	3.84373298	476	675	645
6909	NM_005994	TBX2	T-box 2	-1.361080602	1.101691404	0.823278	0.60594	0.856687	1.89739865	1.58576189	2.0010274	107	105	145
55643	NM_017797	BTBD2	BTB domain containing 2	1.288883692	1.101586807	27.508249	35.496933	28.478161	6.15222318	6.51380399	6.28993987	2753	4741	3730
23164	NM_015134,NM_201274	MPRIP	myosin phosphatase Rho interac	1.076198265	1.101583838	63.475046	68.389547	65.863053	9.4119472	9.51774154	9.55133174	26705	38400	36182
637	NM_001196,NM_001244567,NM	BID	BH3 interacting domain death ag	1.427457458	1.101547514	25.832294	36.955444	26.80832	5.79396609	6.29973728	5.9311056	2139	4080	2898
55359	NM_018423	STYK1	serine/threonine/tyrosine kinase 1	-1.430015672	1.101339135	9.032274	6.325498	9.356369	4.77413325	4.28053774	4.90854629	1035	967	1402
51028	NM_001282168,NM_001282169	VPS36	vacuolar protein sorting 36 homo	-1.31888018	1.101322705	7.515784	5.781452	7.801828	5.09545415	4.70949799	5.23081505	1303	1320	1765
55325	NM_018359,NR_028085,NR_14	UFSP2	UFM1 specific peptidase 2	1.182459965	1.101278286	5.663946	6.738936	5.784227	3.82979934	4.0558926	3.95964434	519	820	703
9101	NM_001128610,NM_001283049	USP8	ubiquitin specific peptidase 8	-1.102112422	1.101273994	9.884028	8.999452	10.34472	5.77462994	5.63703974	5.91138481	2110	2558	2858
27293	NM_001009568,NM_001304579	SMPDL3B	sphingomyelin phosphodiesteras	-1.115621933	1.101192337	10.669296	9.624338	11.103365	4.34572074	4.19603101	4.47827086	759	909	1028
101929188	NR_134628	LOC101929188	uncharacterized LOC101929188	1.374859612	1.101186896	0.371683	0.517204	0.387898	0.83964067	1.06116576	0.90287695	31	57	42
56100	NM_018926,NM_032100	PCDHGB6	protocadherin gamma subfamily	-1.4261675	1.101186896	0.321438	0.228746	0.335464	0.83964067	0.63490936	0.90287695	31	29	42
23429	NM_012234	RYBP	RING1 and YY1 binding protein	1.313976012	1.101085226	6.73412	8.860205	6.970753	4.91599358	5.29849962	5.05053848	1146	2012	1552
339263	NM_001113434	C17orf51	chromosome 17 open reading fra	1.586094449	1.100871277	0.689682	1.095942	0.712973	2.66071453	3.23956766	2.77836293	209	443	283
29068	NM_001301098,NM_001301099	ZBTB44	zinc finger and BTB domain cont	-1.814466319	1.100825442	6.663868	3.711046	6.948031	5.84344384	5.00416403	5.979733	2215	1631	2999
6829	NM_001111020,NM_001130824	SUPT5H	SPT5 homolog, DSIF elongation	1.021218777	1.100707613	47.867214	48.891244	49.691121	7.45304323	7.48316458	7.59072316	6840	9333	9260
65059	NM_001329728,NM_203365,NM	RAPH1	Ras association (RalGDS/AF-6) a	1.108018071	1.100520934	4.640687	5.064262	4.841632	5.43824136	5.58298421	5.57339425	1663	2462	2251
138716	NM_148178,NM_148179	RPP25L	ribonuclease P/MRP subunit p25	1.347521877	1.100409509	9.085974	12.638314	9.315796	3.23740108	3.62781914	3.36145532	331	596	448
64755	NM_022744	C16orf58	chromosome 16 open reading fra	1.08204672	1.100359704	27.681479	29.987648	28.627569	6.26355069	6.37589278	6.39981707	2977	4304	4029
55930	NM_018728	MYO5C	myosin VC	-2.097767922	1.100242972	14.45101	6.896288	14.939094	6.63139796	5.57838681	6.76789746	3853	2454	5214
1029	NM_000077,NM_001195132,NM	CDKN2A	cyclin dependent kinase inhibitor	1.327006693	1.100233129	90.47573	119.172309	93.615444	6.69307245	7.09781747	6.82961453	4023	7133	5444
219771	NM_001282852,NM_001282853	CCNY	cyclin Y	1.183529148	1.100225086	9.759495	11.388444	9.957273	5.49148768	5.72961516	5.62637054	1727	2731	2337
6877	NM_006951,NM_139052	TAF5	TATA-box binding protein associ	-1.681016814	1.100196403	3.617952	2.190239	3.750372	3.64828273	2.97505117	3.77555686	453	360	613
5997	NM_002923	RGS2	regulator of G protein signaling 2	-2.382229599	1.100108707	7.177994	3.016795	7.432087	3.38540081	2.31185141	3.51046477	371	208	502
112840	NM_001008726,NM_001258272	WDR89	WD repeat domain 89	-1.09975571	1.100102623	6.855886	6.326943	7.479466	4.59870169	4.46742999	4.73092712	912	1108	1234
752	NM_005892	FMNL1	formin like 1	-2.864998317	1.099934848	5.179994	1.807263	5.354289	4.3919615	2.99588686	4.52314065	785	366	1062
51529	NM_001002244,NM_001002245	ANAPC11	anaphase promoting complex sul	-1.128626481	1.099849767	114.261006	100.48479	116.620664	6.66654934	6.49380183	6.80256961	3949	4675	5342
4782	NM_001245002,NM_001245004	NFIC	nuclear factor I C	1.062912641	1.099755551	8.19058	8.766738	8.436103	6.00779304	6.09449202	6.14294659	2487	3532	3364
55176	NM_001142627,NM_001142628	SEC61A2	Sec61 translocon alpha 2 subuni	1.372779213	1.09973161	9.319052	12.849618	9.653844	4.57884974	5.01950785	4.71053193	899	1649	1216
387036	NR_003504	GUSBP2	glucuronidase, beta pseudogene	1.210041678	1.099681358	0.726821	0.892027	0.751784	0.89996265	1.03456085	0.96541723	34	55	46
51100	NM_001206651,NM_001206652	SH3GLB1	SH3 domain containing GRB2 lik	-1.023554981	1.099672955	10.604368	10.344843	10.957191	6.06379966	6.03071759	6.19892364	2587	3377	3499
9330	NM_001206774,NM_012086	GTF3C3	general transcription factor IIIC s	1.217842021	1.09963189	16.038354	18.896769	16.162266	6.04833571	6.32876871	6.18338553	2559	4164	3461
151987	NM_001318025,NM_001318026	PPP4R2	protein phosphatase 4 regulatory	1.205780927	1.099596951	11.414239	13.411268	11.515494	5.77530104	6.04077869	5.90989482	2111	3401	2855
10943	NM_001193270,NM_001282174	MSL3	male-specific lethal 3 homolog (D	1.004021681	1.099539372	7.387538	7.220784	7.034541	4.30168014	4.30717831	4.43196063	735	986	994
2547	NM_001288976,NM_001288977	XRCC6	X-ray repair cross complementin	1.107720028	1.099535243	127.52859	141.545592	131.889248	8.13575341	8.28284916	8.27218416	11003	16285	14880
114134	NM_052885	SLC2A13	solute carrier family 2 member 1:	-1.095984261	1.099501435	0.782394	0.714418	0.810492	2.66651408	2.55587612	2.78271118	210	256	284
9232	NM_001282382,NM_001282383	PTTG1	pituitary tumor-transforming 1	-1.051441829	1.099418799	185.002043	175.755563	199.922389	7.22716041	7.15528527	7.36303295	5843	7425	7901
100533111	NR_037894	ZNF670-ZNF695	ZNF670-ZNF695 readthrough (N	-1.333318851	1.099354824	0.378237	0.286772	0.40827	0.5190475	0.40498891	0.56199967	17	17	23
5862	NM_001242644,NM_002865	RAB2A	RAB2A, member RAS oncogene	-1.160783247	1.099322474	39.259038	33.87082	40.569035	7.19667331	6.98315078	7.33240167	5720	6584	7734
170082	NM_001297563,NM_001297564	TCEANC	transcription elongation factor A I	-1.606406447	1.099179825	0.561885	0.34804	0.585254	1.48977343	1.08728891	1.57922529	71	59	96
1827	NM_001285389,NM_001285391	RCAN1	regulator of calcineurin 1	-1.095885259	1.099160373	14.474687	13.245964	14.857615	5.15957233	5.03133013	5.29233867	1364	1663	1844
8706	NM_001038628,NM_001349130	B3GALNT1	beta-1,3-N-acetylgalactosaminylt	1.293957442	1.099105664	4.444842	5.721165	4.443639	4.01166006	4.36304264	4.13992569	594	1027	803
80856	NM_001305008,NM_001305009	LNPK	lunapark, ER junction formation f	1.21197149	1.099059461	2.976623	3.614172	3.071376	4.528787	4.79520018	4.65942844	867	1404	1172
23071	NM_015051	ERP44	endoplasmic reticulum protein 44	-1.001769781	1.09891693	33.46711	33.446957	34.561764	7.31747326	7.31493823	7.45274355	6223	8300	8411
339318	NM_001029997,NM_001145665	ZNF181	zinc finger protein 181	-1.04075877	1.098692517	3.929679	3.778233	4.054953	3.62166181	3.56878235	3.74691298	444	570	600
9648	NM_181453,NR_028063	GCC2	GRIP and coiled-coil domain con	-1.002557513	1.098671452	8.068456	8.055612	8.330029	5.8025506	5.79893152	5.93599396	2152	2868	2908
2023	NM_001201483,NM_001428	ENO1	enolase 1	1.670783807	1.098645356	473.240305	792.667135	482.595798	9.98636547	10.72632097	10.12196388	39785	88815	53760
139596	NM_001307944,NM_145052,NR	UPRT	uracil phosphoribosyltransferase	-1.031847853	1.098632739	4.030589	3.907075	4.145704	3.45071403	3.40966853	3.57456234	390	505	527
84668	NM_032581	FAM126A	family with sequence similarity 12	1.062652416	1.098532829	2.782123	2.961573	2.87504	4.14433812	4.22720295	4.27260082	655	930	885
80781	NM_030582,NR_130444,NM_13	COL18A1	collagen type XVIII alpha 1 chain	2.042318315	1.098526401	24.115086	49.237202	24.748433	7.00514646	8.02962666	7.13971124	5004	13655	6761
84629	NM_001080495	TNRC18	trinucleotide repeat containing 18	-1.35313347	1.09845795	17.710205	13.102991	18.280684	7.51783667	7.08430211	7.65261257	7156	7066	9668
11196	NM_007190,NR_037771	SEC23IP	SEC23 interacting protein	1.083324404	1.098395408	5.208787	5.59095	5.314731	5.19511448	5.30755587	5.32698995	1399	2025	1890
1739	NM_001098424,NM_001204386	DLG1	discs large MAGUK scaffold prot	-1.266316676	1.098370482	46.996787	37.163218	48.488089	7.8409168	7.50194883	7.9757195	8962	9456	12107
1466	NM_001300965,NM_001321	CSRP2	cysteine and glycine rich protein	1.337243588	1.098366349	2.639336	3.547061	2.736096	1.76310734	2.07130288	1.86000215	94	168	127
9374	NM_001204103,NM_005155,NM	PPT2	palmitoyl-protein thioesterase 2	1.008264957	1.098344143	3.482598	3.400058	3.555862	2.9541672	2.96451939	3.07278016	265	357	358
100128731	NM_001134693	OST4	oligosaccharyltransferase comple	1.08063266	1.098283165	154.170212	166.833466	159.147751	6.20597692	6.31639813	6.33948207	2859	4128	3862
84327	NM_001329564,NM_032367,NR	ZBED3	zinc finger BED-type containing 3	1.260032976	1.098138897	1.064285	1.277243	1.022589	1.29387258	1.50084877	1.37552141	57	96	77
23150	NM_015123	FRMD4B	FERM domain containing 4B	-1.69309227	1.097730728	3.985838	2.356676	4.114571	4.71379852	3.99164624	4.84343385	991	782	1338
2633	NM_002053	GBP1	guanylate binding protein 1	1.044134801	1.097607991	8.740865	9.142259	9.018336	4.75384679	4.81389696	4.88346032	1020	1423	1377
80311	NM_030624	KLHL15	kelch like family member 15	-1.034798693	1.097586541	1.416146	1.370619	1.460225	3.27204958	3.22787299	3.39308282	340	439	459
8220	NM_022719,NR_134304	DGCR14	DiGeorge syndrome critical regio	1.561397634	1.09705164	2.524071	3.954115	2.603938	3.83495865	4.44108339	3.95964434	521	1087	703
55142	NM_001130447,NM_001323629	HAUS2	HAUS augmin like complex subu	-1.205566925	1.09699083	6.613621	5.039937	6.610424	4.51599508	4.25915261	4.64397663	859	952	1159
113802	NM_001102592,NM_144584	HENMT1	HEN1 methyltransferase homolo(	1.037153824	1.096894402	7.134561	7.077002	7.295313	3.68016614	3.72876963	3.80364308	464	643	626
89853	NM_001011703,NM_033446	MVB12B	multivesicular body subunit 12B	1.257212584	1.096395464	1.107593	1.445181	1.12663	2.44822539	2.72348335	2.5576298	175	294	236
63891	NM_022064,NR_135218	RNF123	ring finger protein 123	1.142890787	1.096314896	8.022792	9.266922	8.320321	5.14406778	5.33165924	5.27315144	1349	2060	1819
4676	NM_005969	NAP1L4	nucleosome assembly protein 1 l	1.136911451	1.09629403	37.067928	42.190933	38.188595	6.52169329	6.70492623	6.65295218	3568	5420	4811
51768	NM_016551	TM7SF3	transmembrane 7 superfamily me	-1.466486176	1.096236739	19.714113	13.458656	20.309965	6.39860581	5.85417577	6.52966751	3273	2982	4413
29927	NM_013336	SEC61A1	Sec61 translocon alpha 1 subuni	1.649998871	1.096173661	65.223915	107.74752	67.189667	7.85749005	8.57750915	7.98942211	9066	19987	12223
152137	NM_174908,NM_178335	CCDC50	coiled-coil domain containing 50	-1.169990796	1.096103907	21.505772	18.407487	21.882941	7.50494685	7.27979552	7.63663685	7092	8099	9561
8859	NM_004197,NM_004197_dup1,N	STK19	serine/threonine kinase 19	1.251891771	1.096100631	9.977987	11.170789	8.600864	3.42357323	3.72044785	3.54415067	382	639	515
84811	NM_001159736,NM_032725	BUD13	BUD13 homolog	-1.188094468	1.096083323	13.891521	11.818524	14.438113	4.94729859	4.7073941	5.07556282	1172	1318	1580
55599	NM_017619	RNPC3	RNA binding region (RNP1, RRM	1.323583489	1.096019566	7.843233	10.405903	8.081943	3.94641713	4.32786827	4.0704925	566	1001	763
219738	NM_145306	C10orf35	chromosome 10 open reading fra	-2.408649876	1.095990286	10.460081	4.351547	10.782251	3.58232086	2.47408961	3.70399851	431	239	581
3838	NM_001320611,NM_002266	KPNA2	karyopherin subunit alpha 2	1.013269491	1.095914963	320.25794	323.793268	329.49859	9.36757712	9.38656652	9.49952237	25895	35058	34904

6726	NM_001130440,NM_003133	SRP9	signal recognition particle 9	-1.015622239	1.095773659	124.915223	124.326409	129.310975	7.5719483	7.54970253	7.70323717	7431	9776	10015
472	NM_000051,NM_001351834,NM	ATM	ATM serine/threonine kinase	-1.159177168	1.095606679	8.306308	5.479077	8.106794	6.24332066	6.03323945	6.37339261	2935	3383	3955
9938	NM_001007231,NM_001166276	ARHGAP25	Rho GTPase activating protein 2	1.235448372	1.095386268	0.193586	0.242638	0.20108	0.66524311	0.7861039	0.71544405	23	38	31
574432	NR_103539,NR_103540	IBA57-AS1	IBA57 antisense RNA 1 (head to	-1.024244919	1.095386268	0.995326	1.03609	1.019762	0.66524311	0.65251278	0.71544405	23	30	31
64946	NM_022909	CENPH	centromere protein H	-1.105219991	1.0953	24.516699	22.208923	25.226822	5.09437892	4.95446982	5.22203654	1302	1574	1754
256356	NM_001039547,NR_033289	GK5	glycerol kinase 5 (putative)	-1.134727823	1.095294472	5.141269	4.438294	5.348976	5.73447233	5.55576227	5.86343838	2051	2415	2763
132001	NM_001284401,NM_001321294	TAMM41	TAM41 mitochondrial translocato	-1.21950882	1.095254309	5.537372	4.53643	5.73427	3.16959281	2.91797176	3.28688662	314	344	423
8867	NM_001160302,NM_001160306	SYNJ1	synaptojanin 1	1.154669429	1.095240318	2.407373	2.846515	2.533467	4.14225859	4.33878278	4.26640425	654	1009	881
4249	NM_002410	MGAT5	mannosyl (alpha-1,6-)-glycoprote	1.689479496	1.095007893	5.962384	10.087774	6.13517	5.59394673	6.33831747	5.72230146	1857	4192	2501
55178	NM_001317947,NM_018146	MRM3	mitochondrial rRNA methyltransf	1.22844973	1.095000121	8.863654	10.955111	9.165086	4.01166006	4.29181484	4.13483255	594	975	800
2288	NM_002014	FKBP4	FK506 binding protein 4	1.225650622	1.094914711	49.440891	60.659908	50.869244	7.50048924	7.79257349	7.6306189	7070	11578	9521
4832	NM_002513	NME3	NME/NM23 nucleoside diphosph	1.112704295	1.094858298	38.840858	43.266964	39.960625	5.29863957	5.44900251	5.4262136	1506	2239	2028
7189	NM_004620,NM_145803	TRAF6	TNF receptor associated factor 6	-1.356988077	1.094664487	2.781715	2.051552	2.86124	4.49006587	4.07233457	4.615008	843	830	1135
127700	NM_001330493,NM_145047,NM	OSCP1	organic solute carrier partner 1	1.696650151	1.094654273	2.559932	4.596219	2.440824	2.0823043	2.69827959	2.18310266	127	288	171
11186	NM_001206957,NM_007182,NM	RASSF1	Ras association domain family m	1.04931641	1.094579335	8.822745	9.153421	8.908239	4.00709644	4.07233457	4.12972136	592	830	797
388152	NR_027001	GOLGA2P7	golgin A2 pseudogene 7	1.29214934	1.094575645	4.816214	6.228215	4.95244	4.06532627	4.41553601	4.18825403	618	1067	832
5589	NM_001001329,NM_001289102	PRKCSH	protein kinase C substrate 80K-H	1.532902403	1.094566744	102.668834	158.016711	105.966765	7.71014936	8.32402467	7.83991564	8182	16758	11015
9818	NM_001008564,NM_014089	NUP58	nucleoporin 58	1.238216554	1.094524477	9.531166	11.824619	9.829237	5.36612462	5.66766141	5.49341346	1580	2614	2127
647087	NM_001130929	C7orf73	chromosome 7 open reading fran	-1.239133168	1.09451376	9.763791	7.892297	10.044204	4.63020414	4.33469951	4.75546927	933	1006	1256
100134229	NR_024451	JHDM1D-AS1	JHDM1D antisense RNA 1 (head	-1.052475096	1.094085906	0.284054	0.271298	0.287792	0.73313634	0.70406985	0.78645063	26	33	35
101928784	NR_120339,NR_120340,NR_12(	RORA-AS1	RORA antisense RNA 1	1.265488634	1.094085906	0.642117	1.042787	0.354541	0.73313634	0.8787583	0.78645063	26	44	35
56940	NM_001286555,NM_020185,NR	DUSP22	dual specificity phosphatase 22	1.19650622	1.094072985	2.74498	3.421972	2.753507	3.2098649	3.44292618	3.32614297	324	518	436
10901	NM_001282987,NM_001282988	DHRS4	dehydrogenase/reductase 4	1.190277567	1.094024429	13.654319	16.780793	13.888816	4.08061013	4.31824987	4.20292915	625	994	841
6429	NM_005626	SRSF4	serine and arginine rich splicing f	-1.100189335	1.093991595	92.041466	83.757065	94.624481	7.83126799	7.69414897	7.96032684	8902	10811	11978
6470	NM_001281786,NM_004169,NM	SHMT1	serine hydroxymethyltransferase	-1.285379191	1.093877978	16.136798	12.644114	16.708959	5.33550944	4.9834484	5.46190042	1546	1607	2080
79943	NM_030895	ZNF696	zinc finger protein 696	1.319316987	1.093819725	2.162263	2.861618	2.223796	3.44397655	3.81140968	3.56196863	388	684	522
117608	NM_058230	ZNF354B	zinc finger protein 354B	1.06957713	1.093655227	6.379241	6.832922	6.55478	3.8730763	3.96371513	3.99380211	536	766	721
128989	NM_001283106,NM_001283116	TANGO2	transport and golgi organization 2	-1.104894448	1.093613321	2.712938	2.453035	2.837041	3.37481244	3.24537964	3.49199507	368	445	495
57175	NM_001018070,NM_020441	CORO1B	coronin 1B	-1.114062349	1.093569486	60.409262	54.265987	62.054156	6.82759419	6.67320833	6.95555497	4420	5301	5945
57728	NM_001317924,NM_025132	WDR19	WD repeat domain 19	1.152632276	1.093482258	3.867168	4.471291	3.976215	4.15675275	4.3509637	4.27877089	661	1018	889
824	NM_001146068,NM_001748	CAPN2	calpain 2	-1.072532632	1.093423428	179.948368	166.909684	184.608978	9.24847605	9.14762607	9.37712614	23840	29699	32061
4952	NM_000276,NM_001318784,NM	OCRL	OCRL, inositol polyphosphate-5-p	-1.14856386	1.093257053	18.616755	16.220722	19.12843	6.55646575	6.35890386	6.68379406	3656	4253	4916
100507547	NR_037169,NR_037170,NR_037	LOC100507547	uncharacterized LOC100507547	-1.548333864	1.093233069	1.525083	1.264005	1.977891	1.30878272	0.96581954	1.38699077	58	50	78
60592	NM_001153446,NM_001153484	SCOC	short coiled-coil protein	-1.062718338	1.093097309	31.253788	29.490232	32.156646	5.92130285	5.8350318	6.04770155	2340	2942	3146
375056	NM_001300867,NM_001324062	MIA3	MIA family member 3, ER export	1.059867324	1.093039064	16.3365	16.180882	15.880888	6.54391798	6.62693041	6.67095	3624	5132	4872
245	NR_002710,NR_120453	ALOX12P2	arachidonate 12-lipoxygenase ps	1.120709448	1.093029072	4.254568	4.537454	4.316918	3.45742019	3.60765556	3.57456234	392	587	527
51016	NM_001346874,NM_001346875	EMC9	ER membrane protein complex s	1.219468336	1.093009518	42.6097	52.051802	43.648341	5.27795218	5.55751527	5.40309959	1484	2418	1995
552889	NM_001136262	ATXN7L3B	ataxin 7 like 3B	-1.070224239	1.093006541	18.302452	17.11701	18.794069	6.05774429	5.96134713	6.18420751	2576	3216	3463
7298	NM_001071	TYMS	thymidylate synthetase	1.020527947	1.09292272	105.182968	107.460136	108.023361	7.34982173	7.37895999	7.47726312	6365	8679	8556
441951	NR_003604,NR_003605,NR_003	ZFAS1	ZNFX1 antisense RNA 1	-1.297912965	1.0928307	169.032245	134.072797	172.983817	7.14152453	6.76836345	7.26872871	5504	5666	7398
55280	NM_001303404,NM_001303405	CWF19L1	CWF19 like 1, cell cycle control (	-1.389299295	1.092711354	12.588001	9.011567	12.995658	5.04068637	4.58324605	5.16488576	1253	1205	1684
10458	NM_001144888,NM_006340,NM	BAIAP2	BAI1 associated protein 2	-1.481489645	1.092632136	48.717308	32.796008	49.828154	7.27539734	6.71281349	7.40241765	6043	5450	8121
90390	NM_001282986,NM_080651	MED30	mediator complex subunit 30	1.117922716	1.092588078	21.422313	23.730542	21.638399	4.40762874	4.56128075	4.52962301	794	1186	1067
54884	NM_017750	RETSAT	retinol saturase	-1.032881396	1.092532886	19.21249	18.620279	19.722971	5.97603671	5.93011349	6.10177647	2432	3146	3268
91304	NM_001033026,NM_033420	TMEM259	transmembrane protein 259	1.214000729	1.092478294	122.862843	149.436565	126.025037	8.34143305	8.62042021	8.46866218	12695	20592	17058
867	NM_005188	CBL	Cbl proto-oncogene	-1.594660911	1.092456196	5.414052	3.399176	5.558677	5.91217861	5.25306686	6.03773091	2325	1948	3124
51247	NM_001033112,NM_016480,NR	PAIP2	poly(A) binding protein interacting	1.047058527	1.092428648	38.81704	40.743401	39.93174	5.8357435	5.90095294	5.96114983	2203	3082	2960
4331	NM_001177963,NM_002431	MNAT1	MNAT1, CDK activating kinase a	1.103092916	1.092129537	31.27093	34.448483	32.175234	5.42376114	5.56217955	5.54807524	1646	2426	2211
7171	NM_001145160,NM_003290	TPM4	tropomyosin 4	-1.223199286	1.0919789	86.029457	70.410909	88.409032	7.79267723	7.50346531	7.91907562	8666	9466	11639
23765	NM_001289905,NM_014339	IL17RA	interleukin 17 receptor A	1.964035597	1.091952379	4.275055	8.420114	4.390812	5.20411411	6.15864937	5.32773407	1408	3695	1891
25788	NM_001205262,NM_001205263	RAD54B	RAD54 homolog B (S. cerevisiae	-1.417450344	1.091935616	11.219002	7.827344	11.363036	5.20710155	4.71997165	5.33070673	1411	1330	1895
55500	NM_001039481,NM_018638	ETNK1	ethanolamine kinase 1	-1.181696361	1.091863687	15.897393	13.672937	15.836782	6.60710179	6.36892155	6.73265244	3788	4283	5087
23600	NM_001167595,NM_014324,NM	AMACR	alpha-methylacyl-CoA racemase	-1.022625631	1.091494854	3.684194	3.634154	3.859995	3.70573844	3.67595308	3.82277197	473	618	635
23657	NM_014331	SLC7A11	solute carrier family 7 member 11	1.338059493	1.091458996	4.370213	5.853905	4.481435	5.39435726	5.80583095	5.51774626	1612	2882	2164
9282	NM_004229	MED14	mediator complex subunit 14	-1.02234923	1.091451179	6.09476	5.969039	6.250163	5.36344926	5.33234202	5.48676597	1577	2061	2117
51534	NM_001286371,NM_001286372	VTA1	vesicle trafficking 1	1.29422294	1.091359439	11.07514	14.290114	11.31466	5.20710155	5.57030594	5.32996414	1411	2440	1894
5728	NM_000314,NM_001304717,NM	PTEN	phosphatase and tensin homolog	-1.472625196	1.0912177	9.096525	6.190486	9.335223	6.28915072	5.7394296	6.41355113	3031	2750	4068
151195	NM_001142300,NM_001330218	CCNYL1	cyclin Y like 1	1.448693987	1.091212721	5.821014	8.503261	5.98336	4.53514067	5.05054456	4.6558773	871	1686	1169
11054	NM_007346	OGFR	opioid growth factor receptor	1.045131691	1.091207359	46.687237	48.853935	47.877068	6.8093619	6.87249258	6.93421469	4364	6094	5857
203102	NM_001313994,NM_145004	ADAM32	ADAM metallopeptidase domain	-1.455561916	1.091063077	2.248019	1.557608	2.285526	2.78321062	2.33383942	2.89139688	231	212	310
4089	NM_005359	SMAD4	SMAD family member 4	1.131379923	1.091055753	2.424671	2.745479	2.484231	4.44015563	4.61052206	4.56033921	813	1229	1091
9879	NM_001300860,NM_014829,NR	DDX46	DEAD-box helicase 46	-1.068957894	1.091002649	31.92263	29.903995	32.724461	7.4849834	7.38933208	7.60996803	6994	8742	9385
55660	NM_017892	PRPF40A	pre-mRNA processing factor 40 h	1.196211611	1.090958009	28.427078	34.041813	29.141115	7.70979835	7.96714328	7.8348209	8180	13074	10976
6238	NM_001042576,NM_004587	RRBP1	ribosome binding protein 1	1.164322533	1.090944067	81.423012	94.926452	83.798283	8.21975165	8.43856113	8.34492647	11665	18147	15652
284018	NM_181655,NM_181656	C17orf58	chromosome 17 open reading fra	-1.530179275	1.090929225	10.595663	6.848637	10.854277	4.00480921	3.43785926	4.12287815	591	516	793
55004	NM_017907	LAMTOR1	late endosomal/lysosomal adapto	1.35482066	1.090882876	24.666019	33.450771	25.284832	4.87400371	5.29919828	4.99540703	1112	2013	1492
79680	NM_024627	RTL10	retrotransposon Gag like 10	1.324950382	1.090805366	9.509848	12.613309	9.745602	5.96431343	6.36458909	6.0877878	2412	4270	3236
6801	NM_003162	STRN	striatin	1.149563507	1.090759572	3.376095	3.884569	3.460276	5.56629422	5.76342439	5.6890989	1821	2797	2443
81789	NM_001243253,NM_030953	TIGD6	tigger transposable element deriv	-1.221180559	1.090751028	2.644878	2.164082	2.708743	3.49701545	3.23665287	3.61169701	404	442	542
5530	NM_000944,NM_001130691,NM	PPP3CA	protein phosphatase 3 catalytic s	1.107071241	1.09066789	16.622404	18.829007	17.365155	6.25298939	6.39791178	6.37663237	2955	4371	3964
9804	NM_014765	TOMM20	translocase of outer mitochondria	1.100571511	1.090401626	61.825016	68.11911	63.351753	7.67785255	7.81546323	7.8021296	8000	11764	10729
221472	NM_173558	FGD2	FYVE, RhoGEF and PH domain c	-4.435729039	1.090275657	1.482935	0.329666	1.518656	2.43469539	0.99371033	2.53718654	173	52	232
51200	NM_001163446,NM_016352	CPA4	carboxypeptidase A4	1.632563264	1.09016991	1.650028	2.670208	1.668587	2.45494311	3.05664709	2.5576298	176	384	236
5639	NM_000951,NM_001316335	PRRG2	proline rich and Gla domain 2	1.174729309	1.089910096	5.990962	6.947734	6.178717	3.2058879	3.41483526	3.31717816	323	507	433
598	NM_001191,NM_001317919,NM	BCL2L1	BCL2 like 1	-1.259828815	1.08986972	87.065249	70.697233	91.716455	7.84171797	7.51011894	7.96535646	8967	9510	12020
6533	NM_001134367,NM_001134368	SLC6A6	solute carrier family 6 member 6	1.147406576	1.089776545	6.179918	7.667952	6.331653	5.17083736	5.36407379	5.29157606	1375	2108	1843
1499	NM_001098209,NM_001098210	CTNNB1	catenin beta 1	2.012618112	1.089580088	62.470212	126.241868	62.560723	7.71487965	8.72050339	7.83808883	8209	22075	11001
9156	NM_001319224,NM_003686,NM	EXO1	exonuclease 1	1.109522159	1.089164946	12.067773	13.219557	12.24607	5.33003867	5.47644219	5.45033042	1540	2283	2063
79616	NM_001308173,NM_024565,NR	CCNJL	cyclin J like	1.069028307	1.089101154	5.610264	5.969339	5.752439	4.23103553	4.3223799	4.34789256	698	997	935
100128537	NR_135298,NR_135299	C1orf132	chromosome 1 open reading fran	1.214008687	1.088993151	0.605363	0.743129	0.623706	1.23264156	1.40024122	1.30472178	53	86	71
9889	NM_014838	ZBED4	zinc finger BED-type containing 4	-1.041735456	1.088911988	3.816539	3.668481	3.905238	4.733271	4.67653783	4.85173544	1005	1289	1346
80143	NM_001102396,NM_025073,NR	SIKE1	suppressor of IKBKE 1	1.058618134	1.088698742	7.505812	7.972032	7.685335	5.3643416	5.44458858	5.48409837	1578	2232	2113
57707	NM_020947	TLDC1	TBC/LysM-associated domain co	1.450986501	1.08869088	20.108164	29.205793	20.570427	6.63657785	7.16911045	6.75799388	3867	7497	5178
2876	NM_000581,NM_001329455,NM	GPX1	glutathione peroxidase 1	-1.096361695	1.088537376	96.897267	88.589023	98.960025	6.78923598	6.65776512	6.91056862	4303	5244	5761
5927	NM_001042603	KDM5A	lysine demethylase 5A	-1.13032986	1.088096186	5.514734	4.885083	5.640381	5.88756138	5.71398144	6.00739874	2285	2701	3058
143	NM_006437	PARP4	poly(ADP-ribose) polymerase fan	1.152222526	1.087999336	17.7941	20.525442	18.193695	6.58123858	6.78367148	6.70170058	3720	5727	4978
6604	NM_001003801,NM_001003802	SMARCD3	SWI/SNF related, matrix associa	-1.593226475	1.087980888	2.072483	1.302268	2.119946	2.15835987	1.66595383	2.2537152	136	114	182
85379	NM_001145206	KIAA1671	KIAA1671	1.207083328	1.087973608	7.776156	9.398362	7.951674	6.33037656	6.59883122	6.45057385	3120	5032	4175
51125	NM_001002296,NM_001174124	GOLGA7	golgin A7	1.080861438	1.087907189	21.765828	23.57662	22.472757	5.40219915	5.5118332	5.52100322	1621	2341	2169
84529	NM_001130010,NM_001290232	C15orf41	chromosome 15 open reading fra	-1.136035252	1.087887524	6.207941	5.462845	6.441055	4.10430518	3.93163239	4.21906166	636	748	851
573	NM_001172415,NM_001349286	BAG1	BCL2 associated athanogene 1	-1.022384873	1.087702241	5.003283	4.899578	5.122723	4.31651022	4.28618731	4.43196063	743	971	994
5859	NM_001272073,NM_005051,NR	QARS	glutaminyl-tRNA synthetase	-1.08100627	1.087695757	38.095736	35.366206	38.928893	6.72225246	6.610981	6.84242861	4106	5075	5493
130074	NM_001009993,NM_001321743	FAM168B	family with sequence similarity 16	1.286619885	1.087638134	21.339558	27.220987	21.499494	6.82110907	7.18185761	6.94128221	4400	7564	5886
123169	NM_001286430,NM_001323903	LEO1	LEO1 homolog, Paf1/RNA polym	-1.111970986	1.087410136	21.234065	19.16493	21.789619	5.53493448	5.38528583	5.65333437	1781	2140	2382
6599	NM_003074	SMARCC1	SWI/SNF related, matrix associa	1.077916695	1.087337217	10.910214	11.772952	11.145734	5.95782478	6.06439629	6.076764	2401	3458	3211
2039	NM_001114135,NM_001114136	DMTN	dematin actin binding protein	1.148296825	1.087274687	3.265821	3.833784	3.391329	3.25290323	3.43277448	3.36145532	335	514	448
254827	NM_207015	NAALADL2	N-acetylated alpha-linked acidic c	-1.056096521	1.087215331	0.437575	0.417969	0.451488	1.63881618	1.58576189	1.72191206	83	105	111
54509	NM_019034	RHOF	ras homolog family member F, fil	-1.401975735	1.08714536	90.787193	64.833664	92.754738	7.75659858	7.27180908	7.87661009	8451	8054	11300
23483	NM_001304430,NM_014305,NR	TGDS	TDP-glucose 4,6-dehydratase	-1.111112257	1.086847516	6.643784	5.970958	6.718245	3.73639016	3.59633059	3.84788889	484	582	647

6749	NM_003146	SSRP1	structure specific recognition prot	1.291491087	1.086837308	74.435036	96.241486	76.018066	7.68161622	8.04907133	7.80119225	8021	13841	10722
9927	NM_001127660,NM_014874	MFN2	mitofusin 2	1.081913407	1.086814328	24.814542	26.907646	25.360303	6.8330837	6.94571301	6.95218097	4437	6414	5931
6733	NM_001278273,NM_001350738	SRPK2	SRSF protein kinase 2	-1.346845517	1.08676643	13.81195	10.345222	14.356822	5.70236863	5.28233643	5.82020306	2005	1989	2680
93487	NM_144578	MAPK1IP1L	mitogen-activated protein kinase	1.077278793	1.086736908	16.409412	17.696642	16.757103	6.70544932	6.81185167	6.82435142	4058	5841	5424
7936	NM_002904	NELFE	negative elongation factor compl	1.159348013	1.086645884	24.595192	28.550018	25.113348	5.26271827	5.47087182	5.37960921	1468	2274	1962
875	NM_000071,NM_001178008,NM	CBS	cystathionine-beta-synthase	1.407395281	1.086582988	22.866779	32.22219	23.385426	5.87133194	6.35722746	5.98917078	2259	4248	3019
25956	NM_015490	SEC31B	SEC31 homolog B, COPII coat c	1.089349139	1.086398417	2.613284	2.847961	2.666595	3.67154031	3.78571564	3.782087	461	671	616
1643	NM_000107,NM_001300734	DDB2	damage specific DNA binding pro	-1.603289891	1.086298663	26.350483	16.061056	26.519555	5.56396574	4.90116298	5.68096869	1818	1515	2429
10096	NM_001277140,NM_005721,NR	ACTR3	ARP3 actin related protein 3 hom	1.204854973	1.086271698	37.420755	44.907706	38.007211	7.69781293	7.96549372	7.81664753	8112	13059	10838
9025	NM_003958,NM_183078,NR_04	RNF8	ring finger protein 8	-1.126734244	1.086262898	1.701982	1.502263	1.725664	3.3677101	3.21312006	3.47597349	366	434	489
53832	NM_001278722,NM_001278723	IL20RA	interleukin 20 receptor subunit al	1.467675066	1.08616738	6.559011	9.54956	6.591678	4.57884974	5.11306995	4.69331288	899	1763	1201
11102	NM_001098783,NM_007042	RPP14	ribonuclease P/MRP subunit p14	-1.023118114	1.085819228	2.425193	2.342558	2.43713	3.12816418	3.09899072	3.23388481	304	397	406
11284	NM_007254	PNKP	polynucleotide kinase 3'-phospha	1.261696269	1.085806417	30.710732	38.802967	31.338631	5.71361634	6.04328303	5.83021527	2021	3407	2699
7316	NM_021009	UBC	ubiquitin C	-1.072335525	1.085790507	845.405701	789.250732	862.598389	11.05577659	10.95506909	11.17446896	83535	104084	111557
57017	NM_020312	COQ9	coenzyme Q9	1.209346929	1.085567918	30.673006	37.146774	31.293262	5.68533091	5.9547109	5.80157562	1981	3201	2645
9584	NM_001242599,NM_001242600	RBM39	RNA binding motif protein 39	1.029912231	1.085532218	143.115306	147.163146	146.169308	8.64937537	8.6917927	8.7674956	15725	21639	20995
2733	NM_001003722,NM_001499	GLE1	GLE1, RNA export mediator	1.013675753	1.085234131	20.337592	20.480607	20.83847	6.04221495	6.06151662	6.15850577	2548	3451	3401
23354	NM_015302	HAUS5	HAUS augmin like complex subu	-1.215747728	1.084919151	11.524579	9.490734	11.75439	5.62555205	5.34998195	5.74085701	1899	2087	2534
9587	NM_001003690,NM_014628	MAD2L1BP	MAD2L1 binding protein	1.220595604	1.0849078	11.963736	15.838318	13.17446	4.10002583	4.37236795	4.21101796	634	1034	846
22995	NM_001194998,NM_014985	CEP152	centrosomal protein 152	-1.271646686	1.084778315	8.879148	7.023828	8.996352	5.62182561	5.28304295	5.73694088	1894	1990	2527
84939	NM_032853,NR_024247	MUM1	melanoma associated antigen (m	1.313076581	1.084665757	8.51381	11.244107	8.719178	5.13886234	5.52204401	5.25292228	1344	2358	1793
10971	NM_006826	YWHAQ	tyrosine 3-monooxygenase/trypto	1.399488352	1.084632961	127.82457	179.10022	130.289062	8.13718986	8.62062984	8.25399802	11014	20595	14693
7975	NM_002360	MAFK	MAF bZIP transcription factor K	-1.210332842	1.084596177	23.894444	19.762817	24.351391	6.30271368	6.03113821	6.41844959	3060	3378	4082
157680	NM_015243,NM_017890,NM_15	VPS13B	vacuolar protein sorting 13 homo	-1.154703881	1.084533711	4.157611	3.449239	3.805794	5.48985314	5.28727486	5.60443065	1725	1996	2301
182	NM_000214	JAG1	jagged 1	1.27144635	1.084475997	11.502857	14.644622	11.723782	6.08885029	6.43080098	6.20420113	2633	4473	3512
81608	NM_001134937,NM_001134938	FIP1L1	factor interacting with PAPOLA a	1.201461474	1.08444826	30.930662	37.118007	31.98366	6.18895235	6.45043212	6.30437724	2825	4535	3768
92675	NM_001318043,NM_080820	DTD1	D-tyrosyl-tRNA deacylase 1	1.292649643	1.084333147	18.287134	23.758923	18.842222	4.70677986	5.06458384	4.8192972	986	1703	1315
79763	NM_001136201,NM_001136202	ISOC2	isochorismatase domain contain	-1.115560881	1.084234691	39.678336	35.691749	40.626581	5.35539329	5.20167964	5.46933775	1568	1878	2091
90135	NM_033271	BTBD6	BTB domain containing 6	1.015730239	1.084055406	9.947188	10.120954	10.139203	4.34028863	4.36170552	4.45120991	756	1026	1008
100533106	NM_001204180	ZHX1-C8orf76	ZHX1-C8orf76 readthrough	-1.455979056	1.084035495	4.971346	3.416376	5.072971	3.0724647	2.60642858	3.17555833	291	267	388
6775	NM_001243835,NM_003151	STAT4	signal transducer and activator o	1.092086901	1.083998115	1.242106	1.366185	1.268714	2.15010456	2.24959721	2.24113096	135	197	180
2959	NM_001514	GTF2B	general transcription factor IIB	-1.46142558	1.083961928	42.091557	28.831709	42.876751	6.09638885	5.55868275	6.21107345	2647	2420	3529
137209	NM_152412	ZNF572	zinc finger protein 572	1.257403087	1.083937173	0.573883	0.723953	0.585251	1.50279892	1.72538257	1.57922529	72	121	96
126823	NM_001007255,NM_152366,NR	KLHDC9	kelch domain containing 9	1.102763096	1.083902846	1.251592	1.32372	1.253417	1.29387258	1.37924953	1.36396013	57	84	76
7127	NM_006291	TNFAIP2	TNF alpha induced protein 2	1.247890264	1.083858107	121.716194	152.060715	123.972809	8.95354542	9.27245989	9.0694968	19425	32388	25895
515	NM_001688	ATP5F1	ATP synthase, H+ transporting, r	1.23133193	1.083757413	65.583969	80.853485	66.80056	7.07875709	7.37697587	7.19397627	5268	8667	7022
387914	NM_001007538	SHISA2	shisa family member 2	2.091722103	1.083754876	0.267988	0.565835	0.276613	0.81895953	1.37924953	0.87055986	30	84	40
100129961	NR_036549	CCNT2-AS1	CCNT2 antisense RNA 1	1.370477878	1.083676992	0.440298	0.603727	0.454824	0.68823103	0.8787583	0.73352601	24	44	32
440823	NR_003491,NR_033319,NR_03	MIAT	myocardial infarction associated	-10.24819433	1.083676992	0.060113	0.004108	0.062139	0.68823103	0.08023518	0.73352601	24	3	32
150244	NR_003950	ZDHHC8P1	zinc finger DHHC-type containing	-1.99528907	1.083676992	0.243485	0.122782	0.245927	0.68823103	0.38406669	0.73352601	24	16	32
30849	NM_014602	PIK3R4	phosphoinositide-3-kinase regula	1.393571872	1.083622835	9.58977	13.384791	9.767521	5.58708313	6.05739281	5.70063458	1848	3441	2463
5813	NM_005859	PURA	purine rich element binding prote	1.018410416	1.083614783	6.00948	6.12958	6.122376	4.99992509	5.0254311	5.11230382	1217	1656	1622
816	NM_001220,NM_001293170,NM	CAMK2B	calcium/calmodulin dependent pr	-2.385213757	1.083547505	0.102457	0.0427	0.101627	0.54446555	0.25173066	0.58209675	18	10	24
55848	NM_018465	PLGRKT	plasminogen receptor with a C-te	-1.034477994	1.083492147	12.765368	12.363308	13.004669	3.74463792	3.69943092	3.85203285	487	629	649
196951	NM_001330293,NM_152647	FAM227B	family with sequence similarity 22	1.146553924	1.083444204	0.196374	0.272934	0.218386	0.46682716	0.52452456	0.49997203	15	23	20
102724354	NR_136540	LINC01669	long intergenic non-protein codin	-1.054423872	1.083444204	0.563094	0.530023	0.575943	0.46682716	0.44594451	0.49997203	15	19	20
24138	NM_012420	IFIT5	interferon induced protein with te	-1.636246801	1.083435545	5.655025	3.457468	5.753824	4.53672473	3.86524379	4.64755715	872	712	1162
2009	NM_001008707,NM_004434	EML1	echinoderm microtubule associat	1.49247703	1.08328973	0.069397	0.103908	0.067865	0.38477185	0.54351856	0.41288872	12	24	16
401546	NM_001012993	C9orf152	chromosome 9 open reading fran	-5.146843863	1.08328973	0.117906	0.02214	0.119741	0.38477185	0.08023518	0.41288872	12	3	16
4921	NM_001014796,NM_006182	DDR2	discoidin domain receptor tyrosin	3.46340288	1.083033542	0.068928	0.253632	0.073878	0.29776659	0.84852988	0.32020933	9	42	12
100286844	NR_027499,NR_027500,NR_02	BCDIN3D-AS1	BCDIN3D antisense RNA 1	3.874313838	1.083033542	0.07608	0.297595	0.077675	0.29776659	0.92294555	0.32020933	9	47	12
10699	NM_001278585,NM_001278586	CORIN	corin, serine peptidase	-1.980810008	1.083033542	0.041783	0.019566	0.043895	0.29776659	0.15624221	0.32020933	9	6	12
9215	NM_004737,NM_133642	LARGE1	LARGE xylosyl- and glucuronyltra	-1.091995207	1.083033542	0.051746	0.04571	0.054345	0.29776659	0.27464693	0.32020933	9	11	12
5409	NM_002686,NR_073461	PNMT	phenylethanolamine N-methyltrar	1.080119318	1.083033542	0.227827	0.246077	0.241383	0.29776659	0.31941552	0.32020933	9	13	12
56130	NM_001303145,NM_018939	PCDHB6	protocadherin beta 6	-1.002067138	1.083033542	0.069244	0.071213	0.06925	0.29776659	0.29720487	0.32020933	9	12	12
124149	NR_026556	ANKRD26P1	ankyrin repeat domain 26 pseudo	1.737576852	1.083033542	0.03803	0.067132	0.036846	0.29776659	0.48576948	0.32020933	9	21	12
9910	NM_001035230,NM_001243763	RABGAP1L	RAB GTPase activating protein 1	-1.903653248	1.082999541	27.117933	15.407908	25.325577	6.87688916	5.95913844	6.99098343	4575	3211	6094
199746	NM_001040425,NM_144987,NR	U2AF1L4	U2 small nuclear RNA auxiliary fa	-1.396364694	1.082984693	14.784592	10.886708	15.216906	3.80372238	3.36231592	3.91081999	509	487	678
389084	NM_001346120,NM_001346121	C2orf82	chromosome 2 open reading fran	1.528379116	1.082958546	4.612657	7.072809	4.682571	3.77179482	4.34691481	3.87868233	497	1015	662
83590	NM_001136044,NM_031434	TMUB1	transmembrane and ubiquitin like	-1.224076302	1.082904715	30.354759	24.254007	31.835704	5.4177562	5.13358491	5.53008379	1639	1789	2183
1848	NM_001946,NM_022652	DUSP6	dual specificity phosphatase 6	-1.063450945	1.082824975	17.122294	16.00979	17.394219	5.8260599	5.73891471	5.93891905	2188	2749	2914
23264	NM_017590	ZC3H7B	zinc finger CCCH-type containing	1.550976378	1.082817364	36.375687	56.483372	37.015171	7.72081433	8.3515662	7.83508261	8243	17082	10978
1033	NM_001130851,NM_001330173	CDKN3	cyclin dependent kinase inhibitor	-1.015121395	1.082738825	71.314804	70.596015	72.845298	5.98882325	5.96751352	6.10177647	2454	3230	3268
116138	NM_057161,NR_040101	KLHDC3	kelch domain containing 3	1.197482649	1.082623378	21.961302	26.374206	22.361345	5.46511016	5.71973349	5.57715406	1695	2712	2257
118813	NM_001002261,NM_001002262	ZFYVE27	zinc finger FYVE-type containing	-1.296330533	1.082604238	16.368558	12.430152	16.53635	5.59470734	5.22906934	5.70694011	1858	1915	2474
56663	NR_026704	VTRNA1-2	vault RNA 1-2	1.488162123	1.082525863	1.495266	2.591348	1.758644	0.20517559	0.29720487	0.22116494	6	12	8
2280	NM_000801,NM_001199786,NM	FKBP1A	FK506 binding protein 1A	1.769318169	1.082446713	99.828492	176.070553	101.027047	7.27088354	8.0900206	7.38446988	6024	14241	8020
9990	NM_001042494,NM_001042495	SLC12A6	solute carrier family 12 member 6	-1.266973963	1.082438866	5.290335	4.20015	5.15933	5.29583597	4.96419416	5.40732977	1503	1585	2001
64754	NM_001167740,NM_022743	SMYD3	SET and MYND domain containi	1.172528653	1.082416011	15.829761	18.585786	16.150592	4.71239751	4.9339228	4.82246845	990	1551	1318
23287	NM_001286715,NM_001286717	AGTPBP1	ATP/GTP binding protein 1	1.014439628	1.082407411	1.911527	1.94043	1.952507	3.24129226	3.25980797	3.34390718	332	450	442
94107	NM_053045	TMEM203	transmembrane protein 203	1.075723559	1.082371424	26.950663	29.024384	27.408634	5.38647251	5.48935666	5.49804855	1603	2304	2134
259295	NM_176889	TAS2R20	taste 2 receptor member 20	-1.175644164	1.082248144	1.708751	1.456585	1.748311	2.29940604	2.11639955	2.39105499	154	175	205
57657	NM_020897,NR_073074	HCN3	hyperpolarization activated cyclic	-1.088516498	1.082114669	6.585684	6.057244	6.699502	4.69263929	4.57519242	4.8022651	976	1198	1299
93210	NM_001291726,NM_001291728	PGAP3	post-GPI attachment to proteins :	1.561711594	1.082026849	9.284534	14.531737	9.355956	4.63168723	5.25378786	4.74101761	934	1949	1243
84254	NM_032294,NM_172206,NM_17	CAMKK1	calcium/calmodulin dependent pr	1.134607027	1.081808286	0.979758	1.044452	0.93301	2.05603457	2.19658485	2.1430785	124	188	165
8480	NM_001015885,NM_003610	RAE1	ribonucleic acid export 1	1.395802368	1.081529225	35.475249	49.477312	36.071447	5.99171354	6.46638246	6.10308098	2459	4586	3271
100506144	NM_001195156	TMEM35B	transmembrane protein 35B	1.167566528	1.081481959	38.621796	45.148659	39.245674	5.16162707	5.3793514	5.27160538	1366	2131	1817
55034	NM_017947	MOCOS	molybdenum cofactor sulfurase	1.008810603	1.081421868	5.325884	5.375742	5.413941	4.06532627	4.07723084	4.17177132	618	833	822
91107	NM_033452	TRIM47	tripartite motif containing 47	-1.664723046	1.081262653	52.52681	31.58148	53.370148	6.86716399	6.14005097	6.97895459	4544	3647	6043
6383	NM_002998	SDC2	syndecan 2	-1.002019576	1.081039405	0.022446	0.021663	0.022942	0.10623215	0.10601836	0.1148164	3	4	4
29065	NR_002765	ASAP1-IT1	ASAP1 intronic transcript 1	-1.320831011	1.081039405	0.044379	0.049877	0.058918	0.10623215	0.08023518	0.1148164	3	3	4
145407	NM_001001872,NM_001320173	C14orf37	chromosome 14 open reading fra	4.129494323	1.081039405	0.021679	0.103114	0.024723	0.10623215	0.40498891	0.1148164	3	17	4
100874372	NR_046816	ARHGAP26-IT1	ARHGAP26 intronic transcript 1	1.238869866	1.081039405	0.153414	0.20685	0.19221	0.10623215	0.13134883	0.1148164	3	5	4
51440	NM_001282396,NM_001282397	HPCAL4	hippocalcin like 4	2.925067466	1.081039405	0.010728	0.04823	0.016353	0.10623215	0.29720487	0.1148164	3	12	4
100287364	NM_001256859,NM_001256859	USP17L18	ubiquitin specific peptidase 17-lik	1.479755237	1.081039405	0.036885	0.065772	0.041109	0.10623215	0.15624221	0.1148164	3	6	4
729020	NM_001143909	RPEL1	ribulose-5-phosphate-3-epimeras	-1.320831011	1.081039405	0.030706	0.021959	0.028153	0.10623215	0.08023518	0.1148164	3	3	4
100616388	NR_039875	MIR4723	microRNA 4723	-1.002019576	1.081039405	0.748287	0.730778	0.767182	0.10623215	0.10601836	0.1148164	3	4	4
57116	NM_001204221,NM_020394,NR	ZNF695	zinc finger protein 695	-1.79422624	1.081022928	4.018312	1.983846	5.078479	2.97768275	2.27254712	3.07632672	270	201	359
701	NM_001211	BUB1B	BUB1 mitotic checkpoint serine/tl	-1.183779666	1.08092761	31.993551	27.058773	32.499321	6.87751435	6.6363608	6.98886797	4577	5166	6085
100302401	NR_027982	RASAL2-AS1	RASAL2 antisense RNA 1	1.394330117	1.080885086	0.980253	1.374632	0.995866	1.69664266	2.04488597	1.77525664	88	164	117
101060684	NM_001351372	NBPF26	NBPF member 26	1.113182616	1.080876769	15.692032	17.485229	15.937428	6.45288807	6.60590749	6.56386083	3400	5057	4520
8568	NM_003683	RRP1	ribosomal RNA processing 1	-1.00046912	1.080799808	46.861988	46.886723	47.591763	6.38283974	6.38217119	6.49364973	3237	4323	4303
57567	NM_020807	ZNF319	zinc finger protein 319	1.246712909	1.08071619	2.52098	3.143921	2.561591	3.50674676	3.79960773	3.60925088	407	678	541
55706	NM_001168551,NM_018087,NR	NDC1	NDC1 transmembrane nucleopor	1.148615767	1.080689532	18.441768	21.096256	18.587845	6.4410946	6.6388468	6.55180999	3372	5175	4482
134728	NM_001010844	IRAK1BP1	interleukin 1 receptor associated	-1.226990814	1.080523004	0.598314	0.488566	0.605442	1.59082596	1.40024122	1.66651905	79	86	105
55568	NM_198321	GALNT10	polypeptide N-acetylgalactosami	1.213503466	1.080335618	16.91489	20.552052	17.174513	6.63065645	6.9072778	6.74105604	3851	6244	5117
57592	NM_001304763,NM_001304764	ZNF687	zinc finger protein 687	1.216693375	1.080236989	8.36814	10.287743	8.559746	5.28173566	5.55809913	5.39033409	1488	2419	1977
25822	NM_001135004,NM_001135005	DNAJB5	DnaJ heat shock protein family (H	1.414241423	1.080232092	0.676995	1.098994	0.790886	1.51570785	1.86027027	1.58918956	73	138	97
284942	NR_026981,NR_026982	RPL23AP82	ribosomal protein L23a pseudoge	-1.150867021	1.080214536	2.559902	2.231084	2.618104	2.21486103	2.05815489	2.30298238	143	166	190

648	NM_005180	BMI1	BMI1 proto-oncogene, polycomb	1.087914271	1.080209683	8.048573	8.770424	8.174084	4.80206342	4.91945464	4.90954071	1056	1535	1403
11147	NM_001031693,NM_001036645	HHLA3	HERV-H LTR-associating 3	1.029753345	1.080202402	13.283524	13.816216	13.364096	3.55756677	3.59633059	3.65976826	423	582	562
11177	NM_013448,NM_182648	BAZ1A	bromodomain adjacent to zinc fin	1.229756439	1.080119192	18.258476	22.521306	18.635151	6.75085396	7.0467285	6.86105334	4189	6883	5565
51701	NM_016231	NLK	nemo like kinase	1.24317493	1.080077089	7.040573	8.764853	7.151099	4.65958016	4.96243097	4.76648826	953	1583	1266
30968	NM_001287031,NM_001287032	STOML2	stomatin like 2	1.103593116	1.079910439	37.236519	41.144472	37.919799	5.71501616	5.85465113	5.82389986	2023	2983	2687
5520	NM_001177591,NM_002717	PPP2R2A	protein phosphatase 2 regulatory	1.242628336	1.079863023	14.989635	18.651644	15.214514	5.87070407	6.17928992	5.979733	2258	3749	2999
4297	NM_001197104,NM_005933	KMT2A	lysine methyltransferase 2A	-1.521399123	1.079781533	6.084756	4.00254	6.173672	6.63362019	6.03575691	6.74328876	3859	3389	5125
284348	NM_001031749,NM_001288763	LYPD5	LY6/PLAUR domain containing 5	-1.042752658	1.079693629	0.655859	0.62909	0.658625	1.39514558	1.3579479	1.46482043	64	82	85
10695	NM_001318842,NM_001318845	CNPY3	canopy FGF signaling regulator 3	1.267738038	1.079568278	23.246807	29.591842	22.545988	5.35539329	5.69020854	5.46325552	1568	2656	2082
151648	NM_001012409,NM_001012410	SGO1	shugoshin 1	-1.243631447	1.079474831	6.75942	4.773096	5.883685	4.00709644	3.71417485	4.11082387	592	636	786
79602	NM_024551	ADIPOR2	adiponectin receptor 2	1.089778068	1.079375042	30.493647	33.269962	30.93251	6.89150985	7.01454555	7.00081478	4622	6730	6136
6156	NM_000989	RPL30	ribosomal protein L30	-1.173259924	1.079324225	1060.359741	904.775696	1075.488403	9.16227482	8.93218717	9.27221859	22455	25572	29809
23468	NM_001127321,NM_001127322	CBX5	chromobox 5	1.36024128	1.079124231	11.67221	15.899031	11.840096	7.04770809	7.48868777	7.15677157	5155	9369	6842
100499177	NR_034075,NR_034076,NR_034	THAP9-AS1	THAP9 antisense RNA 1	3.065421588	1.078992203	19.233225	58.700578	19.611099	5.43314728	7.02661934	5.54039208	1657	6787	2199
133015	NM_001130727,NM_001258345	PACRGL	PARK2 coregulated like	-1.051564128	1.07894555	3.168029	2.400196	2.544615	3.56379523	3.49751855	3.66448845	425	540	564
66004	NM_023946,NM_177457,NM_17	LYNX1	Ly6/neurotoxin 1	1.122525595	1.078771092	0.572921	0.713386	0.583768	1.86749926	1.99055518	1.94780992	104	156	138
105376114	NR_135839,NR_135840	LOC105376114	uncharacterized LOC105376114	1.237307061	1.07868636	1.214559	1.473812	1.077721	1.21691895	1.40024122	1.28032816	52	86	69
9399	NM_001256672,NM_001256673	STOML1	stomatin like 1	1.483509046	1.078462238	4.395324	6.382406	4.358929	3.27204958	3.79168578	3.37014999	340	674	451
164	NM_001030007,NM_001128	AP1G1	adaptor related protein complex 1	-1.002379594	1.078150832	19.973057	19.945122	20.237241	7.06647239	7.06306898	7.17425323	5223	6962	6926
84256	NM_001308068,NM_020912,NM	FLYWCH1	FLYWCH-type zinc finger 1	1.316101236	1.077911299	6.511339	9.154563	7.329243	5.01931705	5.40489279	5.12434579	1234	2170	1636
4048	NM_000895,NM_001256643,NM	LTA4H	leukotriene A4 hydrolase	-1.391025238	1.077872545	74.056873	53.317618	75.744903	7.32528497	6.85264014	7.43282335	6257	6010	8295
5781	NM_001330437,NM_002834,NM	PTPN11	protein tyrosine phosphatase, no	-1.260403984	1.077363092	31.46494	25.073435	31.888235	7.55778083	7.22588171	7.66473701	7358	7800	9750
54495	NM_001350512,NM_001350513	TMX3	thioredoxin related transmembrai	-1.191300309	1.07735794	30.548391	25.353121	30.896815	7.12845243	6.87788106	7.23521165	5454	6117	7227
23644	NM_014329	EDC4	enhancer of mRNA decapping 4	1.136076237	1.077347981	23.032843	26.195749	23.319677	6.76680203	6.94927828	6.87333758	4236	6430	5613
26232	NM_012168	FBXO2	F-box protein 2	-1.0778178	1.07717884	6.615042	6.146307	6.696758	3.59149522	3.49264009	3.69017701	434	538	575
23137	NM_015110	SMC5	structural maintenance of chromo	-1.037957329	1.077174647	16.212622	15.635568	16.410007	6.57238473	6.51921133	6.67855348	3697	4759	4898
10581	NM_006435	IFITM2	interferon induced transmembran	-2.034492388	1.077029749	53.842445	26.478912	54.5158	5.29583597	4.30856691	5.40027255	1503	987	1991
439	NM_004317	ASNA1	arsA arsenite transporter, ATP-bi	1.099451951	1.076921194	40.792599	44.891544	41.27977	5.73309128	5.86742698	5.83807086	2049	3010	2714
988	NM_001253	CDC5L	cell division cycle 5 like	1.101012354	1.076812466	8.511358	9.383034	8.611459	5.76317329	5.89957147	5.86804962	2093	3079	2772
9776	NM_001142673,NM_001205119	ATG13	autophagy related 13	-1.587331782	1.076810097	9.583361	6.091501	9.717697	5.81239274	5.16074844	5.9173294	2167	1824	2870
2729	NM_001197115,NM_001498	GCLC	glutamate-cysteine ligase catalyti	-1.284587776	1.076772182	13.517725	10.557252	13.746034	5.6774542	5.3241272	5.7821617	1970	2049	2609
80331	NM_025219	DNAJC5	DnaJ heat shock protein family (H	1.092962083	1.076750371	23.984371	26.245226	24.268541	6.96129094	7.08855191	7.06715164	4853	7087	6427
9734	NM_001204144,NM_001204145	HDAC9	histone deacetylase 9	-1.047772357	1.076697034	7.4356	6.570467	6.911726	4.90989472	4.84485395	5.01309432	1141	1455	1511
57819	NM_021177	LSM2	LSM2 homolog, U6 small nuclear	-1.231029352	1.076653425	17.396309	14.158169	17.617245	3.99331833	3.71417485	4.09342686	586	636	776
55506	NM_018649	H2AFY2	H2A histone family member Y2	-1.128427313	1.076354466	15.19591	13.478445	15.368409	5.05512721	4.88636082	5.15820771	1266	1499	1676
64756	NM_001042546,NM_001243728	ATPAF1	ATP synthase mitochondrial F1 c	1.387976074	1.076290789	13.648292	18.978375	13.826681	4.64349742	5.10029882	4.74547971	942	1747	1247
55715	NM_001330556,NM_018110	DOK4	docking protein 4	-1.541519459	1.076127055	11.997984	7.823874	12.24642	5.09006999	4.48841131	5.19292756	1298	1125	1718
2990	NM_000181,NM_001284290,NM	GUSB	glucuronidase beta	-2.078219555	1.076063874	29.566166	14.385471	28.729083	5.98011746	4.94913788	6.08426934	2439	1568	3228
8904	NM_001198863,NM_003915,NM	CPNE1	copine 1	1.138763128	1.075976351	75.419302	86.268403	76.218472	7.22372265	7.41001692	7.32868926	5829	8869	7714
25973	NM_152268	PARS2	prolyl-tRNA synthetase 2, mitoch	1.344272409	1.075902635	1.821517	2.457665	1.846326	2.39332696	2.74825434	2.47942639	167	300	221
55421	NM_001114118	NCBP3	nuclear cap binding subunit 3	1.097834821	1.075895574	5.832802	6.409328	5.897378	5.56939302	5.70135133	5.6727924	1825	2677	2415
1292	NM_001849,NM_058174,NM_05	COL6A2	collagen type VI alpha 2 chain	-1.11955207	1.07584284	15.620353	13.902643	15.717936	5.7065968	5.54696516	5.81012087	2011	2400	2661
2774	NM_001142339,NM_001261443	GNAL	G protein subunit alpha L	1.2201614	1.075598496	0.301851	0.373664	0.308369	1.40904982	1.59489542	1.47560397	65	106	86
332	NM_001012270,NM_001012271	BIRC5	baculoviral IAP repeat containing	1.022457231	1.075497477	44.828421	46.056978	45.443885	6.86053853	6.89230706	6.9646734	4523	6179	5983
56893	NM_001304342,NM_020131	UBQLN4	ubiquilin 4	1.167236696	1.075451414	14.57224	17.031113	14.732207	5.70870626	5.92785643	5.81171749	2014	3141	2664
7737	NM_006978	RNF113A	ring finger protein 113A	1.255383329	1.075308366	9.98485	12.563878	10.10285	3.76910202	4.07560059	3.86644371	496	832	656
6804	NM_001165903,NM_004603	STX1A	syntaxin 1A	-1.551471317	1.075219312	19.237757	12.411922	19.440514	5.35089824	4.73657294	5.45306113	1563	1346	2067
221092	NM_001079559	HNRNPUL2	heterogeneous nuclear ribonucle	-1.006989897	1.075133752	35.003845	34.798695	35.366699	7.46286639	7.45287419	7.56681258	6887	9138	9107
5742	NM_000962,NM_001271164,NM	PTGS1	prostaglandin-endoperoxide synt	1.230499676	1.075053957	0.146758	0.196822	0.152219	0.83964067	0.97983233	0.88680889	31	51	41
3556	NM_001167928,NM_001167929	IL1RAP	interleukin 1 receptor accessory p	1.177064129	1.075025476	6.188912	7.524986	7.358372	4.71239751	4.93931117	4.81293371	990	1557	1309
9329	NM_012204,NR_133925	GTF3C4	general transcription factor IIIC su	1.23844645	1.075025416	5.89857	7.312776	5.956297	5.72616608	6.02945501	5.82863901	2039	3374	2696
4841	NM_001145408,NM_001145409	NONO	non-POU domain containing, octa	1.135185017	1.074917772	126.751876	144.190768	128.158536	8.58881916	8.77130155	8.69278504	15077	22868	19933
84302	NM_001303107,NM_001303108	TMEM246	transmembrane protein 246	1.333234692	1.074901091	5.78714	7.759957	5.83818	4.65082999	5.05137419	4.75103801	947	1687	1252
339448	NM_207356	C1orf174	chromosome 1 open reading fran	-1.009380407	1.074850309	18.467558	18.323238	18.660788	4.95324168	4.94020727	5.05414007	1177	1558	1556
10591	NM_006443,NM_199184	DNPH1	2'-deoxynucleoside 5'-phosphate	-2.020901082	1.074793181	15.955679	7.728786	16.31071	3.51641288	2.6243808	3.61169701	410	271	542
79970	NR_027788,NR_027789	ZNF767P	zinc finger family member 767, ps	-1.288472646	1.074760717	6.031192	4.830696	6.052368	4.34391231	3.99854533	4.44299165	758	786	1002
7095	NM_003262	SEC62	SEC62 homolog, preprotein trans	1.452116847	1.074741355	27.896563	40.554501	28.172375	7.48108076	8.01672881	7.58451019	6975	13533	9220
57035	NM_001321772,NM_020317,NR	RSRP1	arginine and serine rich protein 1	-1.026433766	1.074738311	60.494702	59.718187	66.099294	7.25461217	7.21722067	7.35794233	5956	7753	7873
2767	NM_002067	GNA11	G protein subunit alpha 11	-1.043290597	1.07463115	14.142506	13.570205	14.282004	5.85998825	5.79991918	5.96210866	2241	2870	2962
50937	NM_001243597,NM_016952	CDON	cell adhesion associated, oncoge	1.148813916	1.074517833	6.355278	7.313863	6.42902	5.66953424	5.86601302	5.77126223	1959	3007	2589
79703	NM_001302084,NM_024650	C11orf80	chromosome 11 open reading fra	-1.453174153	1.07449371	15.709266	10.922776	15.872139	5.09545415	4.57519242	5.19619105	1303	1198	1722
80230	NM_001040451,NM_001040452	RUFY1	RUN and FYVE domain containin	1.292692743	1.074468112	16.956176	22.09863	17.233243	5.48821674	5.85132032	5.58961648	1723	2976	2277
79716	NM_001204872,NM_001204873	NPEPL1	aminopeptidase-like 1	1.456329382	1.074458154	18.579307	27.098919	18.754942	5.34095955	5.87213021	5.44210713	1552	3020	2051
127281	NM_001195736,NM_001195737	FAM213B	family with sequence similarity 21	1.009048935	1.074379387	8.412191	8.592154	8.546809	4.57422946	4.58668388	4.67354624	896	1208	1184
152185	NM_001331078,NM_001331079	SPICE1	spindle and centriole associated	1.597629589	1.074342498	3.96482	6.340039	3.98299	4.43336854	5.08417426	4.53220782	809	1727	1069
5588	NM_001242413,NM_001282644	PRKCQ	protein kinase C theta	-1.698250288	1.074240066	0.433281	0.253344	0.437343	1.27880673	0.8787583	1.34055586	56	44	74
23070	NM_015050	CMTR1	cap methyltransferase 1	1.000180566	1.074206406	8.370811	8.38061	8.451358	5.11361143	5.11386441	5.21400945	1320	1764	1744
151556	NM_001033045,NM_001267050	GPR155	G protein-coupled receptor 155	5.728833947	1.074092032	0.096599	0.557139	0.095545	0.77668602	2.35011326	0.82068496	28	215	37
100289373	NR_036531,NR_036532	UBAC2-AS1	UBAC2 antisense RNA 1	2.027537721	1.074092032	0.3997	0.816161	0.405806	0.77668602	1.29208824	0.82068496	28	76	37
23269	NM_001080541,NM_001164273	MGA	MGA, MAX dimerization protein	-1.161615545	1.073886537	5.445315	4.707174	5.493971	6.01690149	5.80435527	6.11821373	2503	2879	3306
36	NM_001330174,NM_001609	ACADSB	acyl-CoA dehydrogenase, short/b	-1.113384316	1.07380959	1.183214	1.064825	1.176196	2.9541672	2.8201133	3.04408938	265	318	350
355	NM_000043,NM_001320619,NM	FAS	Fas cell surface death receptor	-1.775326557	1.073762064	7.384305	4.100913	7.274412	4.83204569	4.04260277	4.93124635	1079	812	1425
2673	NM_001244710,NM_002056	GFPT1	glutamine--fructose-6-phosphate	-1.952381821	1.073531215	10.264021	5.24967	10.338217	6.45120918	5.50154961	6.55244677	3396	2324	4484
84058	NM_001320823,NM_001320824	WDR54	WD repeat domain 54	2.016544	1.073459136	21.657353	43.558792	21.870095	4.64936645	5.6320572	4.7477056	946	2549	1249
3420	NM_001258384,NM_001330763	IDH3B	isocitrate dehydrogenase 3 (NAD	1.493489829	1.073429328	58.722562	87.538629	58.432809	6.42664313	6.9997934	6.52772592	3338	6661	4407
84795	NM_032709	PYROXD2	pyridine nucleotide-disulphide ox	-1.317523065	1.073372154	13.490191	10.245152	13.601658	4.81386993	4.43219317	4.91251987	1065	1080	1406
81855	NM_030971	SFXN3	sideroflexin 3	-1.361780721	1.073343086	18.400322	13.529585	18.56496	5.81696286	5.38067227	5.9173294	2174	2133	2870
178	NM_000028,NM_000642,NM_00	AGL	amylo-alpha-1, 6-glucosidase, 4-	-1.081165485	1.073279475	6.062119	5.623729	6.114429	5.47670971	5.36674241	5.5765281	1709	2112	2256
23043	NM_001161560,NM_001161561	TNIK	TRAF2 and NCK interacting kina	2.074811339	1.07321587	2.606684	4.497524	2.018092	4.28292556	5.29710129	4.3798155	725	2010	957
51507	NM_001283035,NM_001283036	RTFDC1	replication termination factor 2 dc	1.103348085	1.073082859	47.11648	51.825593	47.59252	6.26784903	6.40798706	6.36833848	2986	4402	3941
9920	NM_014867	KBTBD11	kelch repeat and BTB domain co	1.226946762	1.073063368	0.194662	0.239004	0.19481	1.18495016	1.3579479	1.24294651	50	82	66
150275	NM_001284263,NM_001284264	CCDC117	coiled-coil domain containing 117	1.316900162	1.072959601	9.754525	13.025502	9.838729	5.28267998	5.67090409	5.38176058	1489	2620	1965
740	NM_004927,NR_037567,NR_03	MRPL49	mitochondrial ribosomal protein L	1.135930956	1.072860933	23.480948	26.981111	23.687251	5.61659238	5.79695418	5.71606286	1887	2864	2490
84920	NM_032834	ALG10	ALG10, alpha-1,2-glucosyltransfe	1.286419418	1.072818163	2.820504	3.640264	2.850553	3.26442151	3.59405489	3.35562963	338	581	446
11278	NM_007249	KLF12	Kruppel like factor 12	-1.101573114	1.072657786	0.854188	0.776853	0.861831	3.33530968	3.21015128	3.42681271	357	433	471
9200	NM_014241	HACD1	3-hydroxyacyl-CoA dehydratase	-1.058652974	1.07252737	20.680286	19.56472	20.848471	4.77278965	4.69364353	4.87024117	1034	1305	1364
8567	NM_001135943,NM_001135944	MADD	MAP kinase activating death dom	-1.405660966	1.072407028	11.831188	8.402802	11.964751	6.08560737	5.60292734	6.18502902	2627	2497	3465
55603	NM_017633	FAM46A	family with sequence similarity 46	1.24301467	1.072362793	1.620506	2.018392	1.633512	3.30216484	3.58720616	3.39308282	348	578	459
10245	NM_001167947,NM_005834	TIMM17B	translocase of inner mitochondria	-1.057939273	1.072276136	25.653973	24.19556	25.7123	4.67835874	4.60035389	4.77524325	966	1220	1274
10289	NM_005875	EIF1B	eukaryotic translation initiation fa	1.279574633	1.072177208	21.812412	27.947247	21.981184	4.49170017	4.83332352	4.58793254	844	1443	1113
613	NM_004327,NM_021574	BCR	BCR, RhoGEF and GTPase activ	1.15548103	1.072036365	11.059439	12.799489	11.146646	6.24040736	6.44633799	6.33948207	2929	4522	3862
4798	NM_001143835,NM_006165	NFRKB	nuclear factor related to kappaB b	-1.759935483	1.071985134	14.416685	8.198076	14.522101	6.16712953	5.36674241	6.2660695	2782	2112	3668
55857	NM_001163022,NM_001163023	KIZ	kizuna centrosomal protein	1.330144344	1.071974413	3.473864	4.710815	3.570751	3.07682646	3.44545299	3.16560381	292	519	385
3021	NM_005324	H3F3B	H3 histone family member 3B	-1.279221149	1.071869065	362.219971	283.468445	364.847412	9.98012101	9.62525302	10.08015418	39613	41375	52223
27445	NM_014510,NM_033026	PCLO	piccolo presynaptic cytomatrix pr	-2.098872646	1.071724551	0.425245	0.202377	0.428293	3.23349937	2.32288731	3.32316088	330	210	435
9985	NM_001048205,NM_005132	REC8	REC8 meiotic recombination prot	-1.586644963	1.071714752	14.975031	9.476036	15.150281	5.11042377	4.46867267	5.20755546	1317	1109	1736
10857	NM_001282621,NM_006667	PGRMC1	progesterone receptor membrane	1.246639008	1.071643701	73.239336	91.137067	73.454949	7.09953794	7.41550464	7.19866181	5345	8903	7045
5087	NM_001204961,NM_001204963	PBX1	PBX homeobox 1	1.717377259	1.071576575	0.994755	1.715649	1.01025	2.93507482	3.63447819	3.02219055	261	599	344
100507398	NR_103812	INTS6-AS1	INTS6 antisense RNA 1	1.207197106	1.071566568	1.443423	1.763448	1.457999	1.08456011	1.23481109	1.13825079	44	71	58

401303	NR_023382	ZNF815P	zinc finger protein 815, pseudoge	1.241216981	1.071220195	0.784772	0.97434	0.790906	1.66222564	1.88281796	1.73094093	85	141	112
56257	NM_001194990,NM_001194991	MEPCE	methylphosphate capping enzym	-1.106897028	1.071066175	19.339466	18.204278	20.508409	5.85364745	5.70978368	5.95104347	2231	2693	2939
29844	NM_001321792,NM_013342	TFPT	TCF3 fusion partner	1.042298407	1.070947217	11.204067	11.851225	11.431196	3.82462152	3.88026307	3.9167669	517	720	681
9487	NM_004278	PIGL	phosphatidylinositol glycan ancho	-1.191870701	1.070932769	8.426439	7.083838	8.492075	3.38891307	3.16180073	3.47865614	372	417	490
55253	NM_018264,NR_134540	TYW1	tRNA-yW synthesizing protein 1 l	-1.177999554	1.070861648	6.563044	5.601448	6.603026	4.48842972	4.26345507	4.58295467	842	955	1109
10675	NM_001206942,NM_001206943	CSPG5	chondroitin sulfate proteoglycan !	1.62302422	1.070654333	0.473376	0.768474	0.459909	1.03161781	1.43116693	1.08290869	41	89	54
51098	NM_001303458,NM_001303459	IFT52	intraflagellar transport 52	1.606700991	1.070622928	6.990497	11.323475	7.090406	3.94403156	4.59239547	4.03630328	565	1213	744
9209	NM_001134369,NM_001282691	LRRFIP2	LRR binding FLII interacting prote	-1.256074739	1.070520548	14.642785	11.901517	14.667327	5.38471448	5.06458384	5.48075692	1601	1703	2108
5281	NM_002643,NM_173074	PIGF	phosphatidylinositol glycan ancho	-1.090374514	1.070423521	23.7037	21.636472	24.069303	4.66103338	4.54134004	4.75546927	954	1169	1256
65057	NM_001082486,NM_001082487	ACD	ACD, shelterin complex subunit a	1.395961889	1.070410179	18.599228	26.030208	18.742434	5.26367512	5.73427243	5.35937359	1469	2740	1934
11261	NM_007236	CHP1	calcineurin like EF-hand protein 1	-1.475421526	1.070407475	40.680019	27.603683	40.92141	7.00971624	6.45388734	7.10714186	5020	4546	6609
6722	NM_001292001,NM_003131	SRF	serum response factor	-1.018389736	1.070375499	6.891177	7.14433	6.982612	4.87023937	4.84485395	4.96511882	1109	1455	1460
149840	NM_001303477,NM_001303478	C20orf196	chromosome 20 open reading fra	2.092242112	1.070245805	2.037646	3.881873	1.721286	1.59082596	2.38212036	1.65707652	79	221	104
29843	NM_001267594,NM_001267595	SENP1	SUMO1/sentrin specific peptidas	1.206578559	1.070151887	4.397407	5.396547	4.457381	4.48186648	4.74172192	4.5754555	838	1351	1103
80198	NM_001350283,NM_025128,NR	MUS81	MUS81 structure-specific endonu	1.061738334	1.06993622	20.642444	21.955167	20.78303	5.6113401	5.69605597	5.70694011	1880	2667	2474
51031	NM_016080	GLOD4	glyoxalase domain containing 4	-1.019966115	1.069872796	33.71685	33.096989	33.896679	5.93818188	5.91012909	6.03408807	2368	3102	3116
642819	NR_026693	ZNF487	zinc finger protein 487	-1.491934023	1.069666795	0.997906	0.659842	0.992571	1.29387258	0.97983233	1.35230545	57	51	75
4726	NM_004553	NDUFS6	NADH:ubiquinone oxidoreductas	-1.116090036	1.069647295	106.778221	95.766136	107.321304	5.85745527	5.7018798	5.95297396	2237	2678	2943
6730	NM_001260502,NM_001260503	SRP68	signal recognition particle 68	-1.067112883	1.069614956	46.553915	43.322354	47.069921	7.01142618	6.91846161	7.10779184	5026	6293	6612
84952	NM_001252335,NM_032866	CGNL1	cingulin like 1	-2.299402476	1.069598484	0.134532	0.056089	0.135936	0.97665845	0.50527715	1.02535867	38	22	50
5049	NM_001184746,NM_001184747	PAFAH1B2	platelet activating factor acetylhy	-1.072055149	1.069556948	21.494862	20.020063	21.670451	6.42107909	6.32190927	6.51700021	3325	4144	4374
4836	NM_021079	NMT1	N-myristoyltransferase 1	-1.156535246	1.069445198	47.925198	41.482468	48.163567	7.84235858	7.63353034	7.93881404	8971	10364	11800
100874392	NR_046228	ANKRD20A12P	ankyrin repeat domain 20 family r	1.611598014	1.069394354	0.277561	0.456585	0.27736	0.56944351	0.83317479	0.60191771	19	41	25
55140	NM_001284220,NM_001284222	ELP3	elongator acetyltransferase comp	1.266545404	1.069362315	6.438347	8.169287	6.471169	4.40589631	4.7324405	4.49823858	793	1342	1043
55245	NM_001184977,NM_018244,NM	UQCC1	ubiquinol-cytochrome c reductase	1.389924538	1.069360636	18.041992	24.693409	17.933991	5.4348473	5.90049259	5.52943707	1659	3081	2182
55690	NM_018026	PACS1	phosphofurin acidic cluster sortin	1.122382143	1.069351727	8.36162	9.400282	8.40423	5.23273681	5.39512262	5.32698995	1437	2155	1890
404281	NM_206923	YY2	YY2 transcription factor	1.503126486	1.069348263	1.055399	1.589634	1.061719	1.93632435	2.39263351	2.00847214	111	223	146
5931	NM_001198719,NM_002893	RBBP7	RB binding protein 7, chromatin r	1.151434244	1.069304001	62.317124	71.846062	62.630465	6.94855447	7.15045371	7.04447148	4810	7400	6326
3597	NM_001560	IL13RA1	interleukin 13 receptor subunit al	1.203691433	1.069300647	19.53632	23.545401	19.630524	6.27070749	6.53501627	6.366167	2992	4812	3935
130814	NM_001282710,NM_001282711	PQLC3	PQ loop repeat containing 3	-1.607002115	1.06928029	8.396753	5.284684	8.653102	3.99562384	3.36498779	4.08640889	587	488	772
23078	NM_001009814,NM_015058	VWA8	von Willebrand factor A domain c	1.021573212	1.069240958	2.463644	2.42392	2.674105	4.07407972	4.10306728	4.16512515	622	849	818
9230	NM_004218	RAB11B	RAB11B, member RAS oncogene	1.154482506	1.069214829	44.5611	51.509624	44.787666	6.16303279	6.36758987	6.25828436	2774	4279	3648
3216	NM_018952	HOXB6	homeobox B6	-2.973159567	1.069160322	74.918907	25.221392	75.268784	6.94557632	5.39642914	7.04129937	4800	2157	6312
23049	NM_015092	SMG1	SMG1, nonsense mediated mRN	1.130571347	1.069033426	12.769161	14.453656	12.828233	7.64921614	7.8254402	7.74506027	7842	11846	10311
6878	NM_001190415,NM_005641,NM	TAF6	TATA-box binding protein associ	-1.44891458	1.068972196	22.555224	15.381709	21.84641	5.7806586	5.25738748	5.87519349	2119	1954	2786
400604	NR_038458	TOB1-AS1	TOB1 antisense RNA 1	-1.460697653	1.068508039	1.938367	1.319987	1.94519	1.87753473	1.50084877	1.94780992	105	96	138
100101267	NM_001099415	POM121C	POM121 transmembrane nucleo	-1.289233113	1.068494329	9.935828	7.713913	9.978787	5.85173976	5.49241457	5.94572127	2228	2309	2928
9869	NM_001145415,NM_001243491	SETDB1	SET domain bifurcated 1	1.025631211	1.068307772	14.86524	14.78883	14.791733	5.91034682	5.94626094	6.00414397	2322	3182	3051
7283	NM_001070	TUBG1	tubulin gamma 1	-1.056792594	1.06814786	82.1772	77.844109	82.490608	7.29565343	7.21648115	7.3901805	6129	7749	8052
10844	NM_001256617,NM_001256618	TUBGCP2	tubulin gamma complex associat	1.295396265	1.068012067	11.644203	15.396315	11.876649	5.61958513	5.98629003	5.71264859	1891	3273	2484
138151	NM_144653	NACC2	NACC family member 2	1.162906443	1.067925817	5.282929	6.153012	5.303022	5.19009033	5.40229387	5.28239316	1394	2166	1831
23585	NM_014313	TMEM50A	transmembrane protein 50A	-1.175349745	1.067830238	54.247387	46.208267	54.438881	6.93780422	6.70677045	7.03174097	4774	5427	6270
6683	NM_014946,NM_199436	SPAST	spastin	-1.001010646	1.067669883	4.675054	4.644259	4.660764	4.61977956	4.61838139	4.71053193	926	1236	1216
130271	NM_172069	PLEKHH2	pleckstrin homology, MyTH4 and	-1.309657514	1.067669036	2.423987	1.85326	2.432573	4.12340669	3.75955568	4.21263029	645	658	847
506	NM_001686	ATP5B	ATP synthase, H+ transporting, r	-1.055490281	1.067626161	354.013977	335.7789	355.177612	9.31344866	9.23566084	9.40771199	24940	31571	32749
123016	NM_001288781,NM_001288782	TTC8	tetratricopeptide repeat domain 8	1.394632859	1.067588424	14.018436	19.662721	14.049419	4.9814326	5.44837277	5.07290231	1201	2238	1577
22885	NM_001301015,NM_001301018	ABLIM3	actin binding LIM protein family n	1.357239353	1.06752259	20.334532	27.631485	20.651435	6.37134502	6.8074386	6.46451209	3211	5823	4216
79174	NM_001135101,NM_001284317	CRELD2	cysteine rich with EGF like doma	1.247201363	1.067507762	26.527492	32.977078	26.512348	5.25215086	5.56334327	5.3440084	1457	2428	1913
2011	NM_001039469,NM_001163296	MARK2	microtubule affinity regulating kin	1.147763383	1.067495407	13.386905	15.437952	13.413051	5.92069636	6.11646066	6.01342391	2339	3587	3071
9724	NM_021645	UTP14C	UTP14, small subunit processom	1.150308158	1.067435625	2.233378	2.573997	2.241846	3.70573844	3.89327789	3.7929053	473	727	621
102724316	NR_003930,NR_110920,NR_11(	SVIL-AS1	SVIL antisense RNA 1	1.231062563	1.06740741	6.805839	8.158034	6.593306	3.83495865	4.11581404	3.92268941	521	857	684
9325	NM_001321924,NM_016213,NR	TRIP4	thyroid hormone receptor interact	1.039084091	1.067395246	11.977163	12.356043	11.968052	4.62127341	4.67438529	4.71167259	927	1287	1217
9690	NM_014671	UBE3C	ubiquitin protein ligase E3C	-1.299575811	1.067272918	17.601982	13.557918	17.650913	6.48851955	6.11527096	6.58143848	3486	3584	4576
55233	NM_001317110,NM_001317111	MOB1A	MOB kinase activator 1A	1.168207709	1.06724833	31.528535	36.912491	31.583339	7.24446976	7.46739973	7.33776775	5914	9231	7763
8409	NM_004182,NM_153477,NR_04	UXT	ubiquitously expressed prefoldin	1.090150789	1.067116247	23.437803	26.211892	24.345157	3.97005831	4.08697381	4.05799054	576	839	756
85236	NM_001312653,NM_080593	HIST1H2BK	histone cluster 1 H2B family men	1.426016021	1.066858241	106.237406	151.619984	106.492403	6.45372678	6.9608049	6.54606638	3402	6482	4464
2820	NM_000408,NM_001083112	GPD2	glycerol-3-phosphate dehydroger	-1.03516286	1.066834686	13.838954	13.242486	13.974067	6.33493591	6.28570472	6.42715568	3130	4040	4107
121268	NM_001303126,NM_144593,NR	RHEBL1	Ras homolog enriched in brain lik	-1.328842663	1.066820808	4.060718	2.978796	4.018096	2.53954085	2.20853486	2.6172769	189	190	248
54442	NM_018992	KCTD5	potassium channel tetramerizatio	1.185907042	1.066750627	24.436651	29.014553	24.498745	5.89745874	6.13966094	5.98917078	2301	3646	3019
8891	NM_001166588,NM_001261418	EIF2B3	eukaryotic translation initiation fa	1.17322408	1.066740661	8.964352	10.694198	9.124449	4.18126603	4.39998757	4.26950586	673	1055	883
133	NM_001124	ADM	adrenomedullin	1.358472928	1.06668658	41.824322	56.87228	41.919968	6.03886537	6.47506509	6.13063316	2542	4614	3335
677839	NR_003017	SNORA71C	small nucleolar RNA, H/ACA box	1.121836775	1.066662213	2.918696	3.393508	3.031654	0.49317359	0.54351856	0.52094563	16	24	21
1024	NM_001260,NM_001318368,NM	CDK8	cyclin dependent kinase 8	1.078176415	1.066613861	5.16866	5.53938	5.15169	4.03874269	4.14097459	4.12630381	606	873	795
6217	NM_001020,NM_001321111	RPS16	ribosomal protein S16	-1.197252616	1.066607354	592.139263	496.378193	591.795549	8.65494712	8.39592343	8.74775342	15786	17617	20709
107133515	NR_015341	LOC107133515	SMURF2P1-LRRC37BP1 readth	-1.096918734	1.066516979	6.679887	6.094721	6.695547	4.94967876	4.82072735	5.03967945	1174	1430	1540
1375	NM_001145134,NM_001145135	CPT1B	carnitine palmitoyltransferase 1B	-1.123098456	1.066105402	15.330321	13.876997	15.512793	5.42290482	5.25954295	5.51317412	1645	1957	2157
9212	NM_001256834,NM_001284526	AURKB	aurora kinase B	-1.055723608	1.066044552	55.929981	54.098507	56.100091	6.11241148	6.03533764	6.20339046	2677	3388	3510
1374	NM_001031847,NM_001876	CPT1A	carnitine palmitoyltransferase 1A	-1.088368177	1.065945949	15.83695	14.749118	15.825841	6.33993461	6.21933662	6.43096978	3141	3856	4118
7286	NM_001126337,NM_001301317	TUFT1	tuftelin 1	1.332753795	1.065876492	19.877966	26.01688	20.575575	5.95664189	6.36525647	6.04724983	2399	4272	3145
50626	NM_001129888,NM_001330618	CYHR1	cysteine and histidine rich 1	1.107987377	1.065818639	23.391769	28.587791	24.497504	5.41345159	5.55809913	5.50332763	1634	2419	2142
55825	NM_018441	PECR	peroxisomal trans-2-enoyl-CoA re	1.063638123	1.065739314	6.196291	6.590591	6.199959	3.62166181	3.70365887	3.70628928	444	631	582
8636	NM_003731	SSNA1	SS nuclear autoantigen 1	1.133072247	1.065638873	40.880833	46.388161	40.950909	5.26558691	5.44142749	5.35500021	1471	2227	1928
11068	NM_001291284,NM_007022,NR	CYB561D2	cytochrome b561 family member	-1.011581899	1.06554334	5.838894	5.962045	6.111755	3.08117507	3.06652959	3.16227031	293	387	384
5747	NM_001199649,NM_001316342	PTK2	protein tyrosine kinase 2	-1.022691183	1.065474881	37.440151	36.649329	37.532045	7.38344285	7.3512678	7.47440988	6516	8513	8539
9797	NM_014760	TATDN2	TatD DNase domain containing 2	1.193549809	1.065472767	4.272948	5.107283	4.278542	4.42141376	4.66574286	4.5087761	802	1279	1051
4588	NM_005961	MUC6	mucin 6, oligomeric mucus/gel-fo	-63.09936704	1.065351781	0.947473	0.013992	0.947405	3.06808971	0.15624221	3.14885876	290	6	380
7417	NM_001184783,NM_001184823	VDAC2	voltage dependent anion channel	-1.405851173	1.065191638	105.570211	76.277392	103.432288	7.28603192	6.79832467	7.37658069	6088	5786	7976
9757	NM_014727	KMT2B	lysine methyltransferase 2B	-1.162816326	1.065021559	11.997677	10.327421	12.006062	6.6432105	6.42792896	6.7332142	3885	4464	5089
126321	NM_001287529,NM_174983	MFSD12	major facilitator superfamily doma	1.287970893	1.064865536	11.597051	14.943971	11.596745	4.64936645	5.00159078	4.73654166	946	1628	1239
56929	NM_020177	FEM1C	fem-1 homolog C	-1.158460314	1.064796239	7.659144	6.61883	7.661897	5.47092159	5.26384425	5.55952381	1702	1963	2229
51366	NM_001282873,NM_015902	UBR5	ubiquitin protein ligase E3 compo	-1.03940199	1.06479087	19.805165	19.0766	19.818683	7.7201174	7.66463251	7.81027231	8239	10591	10790
54993	NM_001007072,NM_017894,NM	ZSCAN2	zinc finger and SCAN domain co	1.42540877	1.064680741	3.633582	5.543252	2.809718	2.89117014	3.34347295	2.96976228	252	480	330
5089	NM_002586	PBX2	PBX homeobox 2	-1.025890291	1.064541115	20.00153	19.519924	20.006931	5.99055812	5.95426739	6.0794174	2457	3200	3217
56267	NM_001008661,NM_001008662	KYAT3	kynurenine aminotransferase 3	-1.176312312	1.064510828	17.659063	14.615846	17.771226	5.09545415	4.8685837	5.18309257	1303	1480	1706
6767	NM_001278589,NM_003932	ST13	ST13, Hsp70 interacting protein	-1.43467461	1.064412437	34.466837	24.089162	34.474994	6.92031409	6.40474465	7.00965244	4716	4392	6174
5562	NM_006251,NM_206907	PRKAA1	protein kinase AMP-activated cat	2.200537399	1.064394453	5.151118	11.380596	5.154621	4.73465189	5.8427199	4.82141214	1006	2958	1317
163590	NM_001199260,NM_001349931	TOR1AIP2	torsin 1A interacting protein 2	1.029056981	1.064387399	14.849839	15.518521	15.018575	6.9184926	6.9594796	7.00779638	4710	6476	6166
8031	NM_001145260,NM_001145261	NCOA4	nuclear receptor coactivator 4	-1.270658678	1.064341869	31.172718	24.529978	31.265876	6.76545159	6.42344998	6.85461363	4232	4450	5540
128061	NM_001300830,NM_152379	C1orf131	chromosome 1 open reading fran	1.297911158	1.064314253	9.194917	11.953505	9.196053	3.80108857	4.15339221	3.88476293	508	881	665
57599	NM_001303402,NM_001303403	WDR48	WD repeat domain 48	1.14030247	1.064286368	6.079566	7.02993	6.100664	4.62871952	4.81096259	4.71508918	932	1420	1220
9721	NM_014696	GPRIN2	G protein regulated inducer of ne	-1.020608098	1.064226628	0.781756	0.771298	0.787879	1.26358189	1.24644988	1.31676564	55	72	72
5318	NM_001005242,NM_004572	PKP2	plakophilin 2	-3.667340926	1.064189705	15.232521	4.151139	15.231407	6.02030239	4.20351422	6.10872026	2509	914	3284
79864	NM_024806,NM_199124	C11orf63	chromosome 11 open reading fra	-2.284538437	1.064113727	2.616106	1.116872	2.457425	2.98698269	2.01116974	3.06566078	272	159	356
55070	NM_001144074,NM_001321594	DET1	de-etiolated homolog 1 (Arabidop	1.074878918	1.064011743	1.755394	1.271751	1.557262	2.26158951	2.34470901	2.3329413	149	214	195
259173	NM_001190707,NM_147129,NR	ALS2CL	ALS2 C-terminal like	1.302297119	1.063980144	29.920801	38.860557	29.387764	7.32299179	7.70196312	7.41192266	6247	10870	8175
9860	NM_001312686,NM_014813	LRIG2	leucine rich repeats and immuno	-1.177503706	1.063900466	7.362612	6.260943	7.357644	5.06723449	4.83910025	5.15401818	1277	1449	1671
6897	NM_001258437,NM_001258438	TARS	threonyl-tRNA synthetase	1.512979166	1.063861642	55.137448	85.803326	54.490065	7.273736	7.86797226	7.36248838	6036	12202	7898
84964	NM_001297701,NM_032878,NM	ALKBH6	alkB homolog 6	1.083146508	1.063627508	15.429772	16.721454	15.465	4.01847853	4.12687582	4.10215159	597	864	781
8631	NM_001075099,NM_003726	SKAP1	src kinase associated phosphopr	-5.68881009	1.063601098	16.040987	2.806956	16.048611	4.67979317	2.41343275	4.76539014	967	227	1265

6574	NM_005415	SLC20A1	solute carrier family 20 member 1	1.275387749	1.06336324	155.652969	198.740829	155.539688	9.00222705	9.35255702	9.09069438	20093	34240	26279
79770	NM_001350735,NM_024715	TXNDC15	thioredoxin domain containing 15	1.282617854	1.063254473	11.541494	14.823019	11.539766	5.40826906	5.7598759	5.4947393	1628	2790	2129
4637	NM_021019,NM_079423	MYL6	myosin light chain 6	-1.052221752	1.063234829	561.217315	532.956543	564.567139	8.749678	8.67641376	8.83793948	16860	21409	22048
5287	NM_002646	PIK3C2B	phosphatidylinositol-4-phosphate	-1.747952929	1.063119294	4.495446	2.572967	4.489323	5.09974705	4.32512669	5.18555761	1307	999	1709
5740	NM_000961	PTGIS	prostaglandin I2 synthase	-2.309703154	1.063084403	0.242056	0.101454	0.240451	1.21691895	0.65251278	1.26797492	52	30	68
6175	NM_001002,NM_053275	RPLP0	ribosomal protein lateral stalk sul	-1.15087704	1.062974258	931.676545	810.366485	928.901932	10.1057936	9.90325688	10.19382291	43222	50179	56508
51380	NM_001244705,NM_001244706	CSAD	cysteine sulfinic acid decarboxyla	-1.274804187	1.062965772	21.291998	17.342243	22.380911	5.50773204	5.16612031	5.59395302	1747	1831	2284
9643	NM_001142418,NM_001142419	MORF4L2	mortality factor 4 like 2	1.05731035	1.062913702	220.239031	234.066215	219.727326	8.72209754	8.80231176	8.80992036	16540	23366	21623
51706	NM_016243	CYB5R1	cytochrome b5 reductase 1	-1.018269454	1.062912843	19.781538	19.457588	19.758238	5.02947882	5.00416403	5.11489271	1243	1631	1625
219988	NM_152716	PATL1	PAT1 homolog 1, processing boc	1.156366828	1.062856555	23.002127	26.628147	22.97513	6.54588578	6.75340116	6.63292185	3629	5607	4744
1571	NM_000773	CYP2E1	cytochrome P450 family 2 subfar	-1.571623368	1.062679866	0.204017	0.125969	0.192317	0.41264541	0.27464693	0.43515728	13	11	17
406962	NR_029707	MIR186	microRNA 186	-1.15648108	1.062679866	3.894373	3.40986	3.723349	0.41264541	0.36283659	0.43515728	13	15	17
2212	NM_001136219,NM_021642	FCGR2A	Fc fragment of IgG receptor IIa	-1.021559294	1.062679866	0.134896	0.132913	0.139689	0.41264541	0.40498891	0.43515728	13	17	17
735301	NR_003142	SNHG9	small nucleolar RNA host gene 9	-1.008719881	1.062610222	12.781038	12.689037	12.698956	1.96484452	1.95552917	2.03057861	114	151	149
55893	NM_018660	ZNF395	zinc finger protein 395	1.312073239	1.062588614	6.529925	8.577106	6.521813	4.97910428	5.36006158	5.06399834	1199	2102	1567
25917	NM_001114092,NM_015453	THUMPD3	THUMP domain containing 3	-1.044027862	1.062398492	8.237826	7.853168	8.184245	5.01022388	4.9500279	5.09492475	1226	1569	1602
7112	NM_001032283,NM_001032284	TMPO	thymopoietin	-1.165415637	1.062324772	39.789394	34.005683	39.736518	7.28485417	7.06553497	7.37153773	6083	6974	7948
353116	NM_001319243,NM_001319244	RILPL1	Rab interacting lysosomal proteir	1.12498421	1.062324392	6.081035	6.609368	5.862968	4.10430518	4.26488638	4.18661421	636	956	831
51090	NM_015993	PLLP	plasmolipin	1.527354816	1.061708131	2.962754	4.542688	2.954938	2.41415945	2.92884759	2.48477392	170	347	222
1155	NM_001281,NM_001300971	TBCB	tubulin folding cofactor B	-1.434502021	1.06154935	47.099549	32.486707	46.734475	6.08398318	5.57261939	6.16892453	2624	2444	3426
29796	NM_001003684,NM_013387	UQCR10	ubiquinol-cytochrome c reductase	-1.148124767	1.061515581	52.706419	46.004308	52.736605	5.60757673	5.41266157	5.69199148	1875	2182	2448
8766	NM_001206836,NM_004663	RAB11A	RAB11A, member RAS oncogen	-1.398955015	1.061515539	43.245857	30.945339	43.140856	7.70417055	7.22257085	7.78989622	8148	7782	10638
84293	NM_001243778,NM_001243779	FAM213A	family with sequence similarity 21	-2.551742864	1.061448091	22.118131	8.573776	22.147112	5.27984516	3.98471399	5.36373375	1486	778	1940
440307	NR_104604	TTLL13P	tubulin tyrosine ligase like 13, ps	-1.298856025	1.061292248	0.510672	0.400879	0.516799	0.93882015	0.77006602	0.98063768	36	37	47
2649	NM_001278546,NM_001489,NM	NR6A1	nuclear receptor subfamily 6 grou	1.244552192	1.061290962	3.617041	4.501094	3.603139	4.69263929	4.99729182	4.77524325	976	1623	1274
54620	NM_001099784,NM_001282351	FBXL19	F-box and leucine rich repeat pro	3.552348893	1.061195488	4.494188	15.979095	4.480202	4.13809052	5.90692407	4.21906166	652	3095	851
55729	NM_001286514,NM_001286515	ATF7IP	activating transcription factor 7 in	-1.098011542	1.060722383	13.055531	11.884149	13.624423	6.78290947	6.64929626	6.86720853	4284	5213	5589
115426	NM_152896,NR_046386	UHRF2	ubiquitin like with PHD and ring fi	1.062664938	1.060703306	14.853495	15.823275	14.820921	5.7441029	5.83020585	5.82758721	2065	2932	2694
989	NM_001011553,NM_001242956	SEPT7	septin 7	-1.039497595	1.060652606	40.8731	38.97468	40.657752	7.42872196	7.37316534	7.51319663	6725	8644	8773
819	NM_001745	CAMLG	calcium modulating ligand	-1.144302261	1.060466899	14.334274	12.544094	14.289444	5.01364054	4.82558505	5.09579869	1229	1435	1603
345462	NM_001136116	ZNF879	zinc finger protein 879	1.062896306	1.060403035	0.688916	0.735161	0.684042	1.46336426	1.52015496	1.51794971	69	98	90
138639	NM_001253829,NM_001253830	PTPDC1	protein tyrosine phosphatase dor	1.441710153	1.06034449	4.210875	6.06911	4.172029	4.27345604	4.77826176	4.35374951	720	1387	939
7776	NM_001306089,NM_007345	ZNF236	zinc finger protein 236	-1.277402175	1.06009122	5.60473	4.392119	5.651196	5.50287791	5.15844008	5.58526687	1741	1821	2270
66036	NM_015458	MTMR9	myotubularin related protein 9	1.274413641	1.060076828	2.11496	2.698658	2.105823	4.02300634	4.35365666	4.10215159	599	1020	781
23011	NM_014999	RAB21	RAB21, member RAS oncogene	-1.024820348	1.060020691	6.200174	6.058163	6.177429	5.7502602	5.71555246	5.83283856	2074	2704	2704
221749	NM_183373	PXDC1	PX domain containing 1	-1.506371963	1.059727576	6.633585	4.410219	6.607582	3.92480368	3.38091595	4.00314859	557	494	726
27112	NM_015686	FAM155B	family with sequence similarity 15	1.177099668	1.059631679	0.579285	0.681944	0.575491	1.70793493	1.87534115	1.76650173	89	140	116
9785	NM_014003	DHX38	DEAH-box helicase 38	-1.017972225	1.059338364	33.599308	33.045303	33.447063	7.19692379	7.17140181	7.27953794	5721	7509	7454
22823	NM_001164391,NM_001164392	MTF2	metal response element binding t	1.171487511	1.059085154	12.096132	14.204593	12.012978	5.60908326	5.83310336	5.69025663	1877	2938	2445
10111	NM_005732	RAD50	RAD50 double strand break repa	-1.037137433	1.058992049	17.015663	16.423529	16.932924	6.78524352	6.7331208	6.86720853	4291	5528	5589
53344	NM_001039840,NM_001300884	CHIC1	cysteine rich hydrophobic domair	1.125734335	1.058978439	2.736103	3.086462	2.725209	4.28103662	4.44361342	4.35958279	724	1089	943
55152	NM_001009996,NM_001276405	DALRD3	DALR anticodon binding domain	-1.085074878	1.058774144	7.914054	7.289718	7.880671	3.8730763	3.76361129	3.95001076	536	660	698
5298	NM_001198773,NM_001198774	PI4KB	phosphatidylinositol 4-kinase bet	1.003660684	1.058728178	9.705152	9.931138	9.683396	5.31072594	5.31586533	5.39104626	1519	2037	1978
23300	NM_001300728,NM_015251	ATMIN	ATM interactor	1.021369791	1.058668702	16.963812	17.465359	16.841238	6.3431066	6.37324105	6.42437545	3148	4296	4099
51660	NM_001270879,NM_016098,NR	MPC1	mitochondrial pyruvate carrier 1	-1.426437182	1.058665747	7.765349	5.402248	7.756566	3.23740108	2.78861701	3.31117052	331	310	431
6809	NM_001178040,NM_004177	STX3	syntaxin 3	1.040991276	1.058606327	5.589926	5.845751	5.625227	5.17998922	5.23638356	5.25995683	1384	1925	1802
83931	NM_001282546,NM_001282547	STK40	serine/threonine kinase 40	1.068754942	1.058514183	12.347563	13.111393	12.114989	5.49312037	5.58699495	5.57339425	1729	2469	2251
51465	NM_016021	UBE2J1	ubiquitin conjugating enzyme E2	1.091980158	1.058456273	7.202699	7.874583	7.166873	4.97560474	5.09869445	5.05503907	1196	1745	1557
54942	NM_017832	FAM206A	family with sequence similarity 20	1.235562956	1.058443474	8.972197	11.105513	8.928965	4.14641466	4.43600998	4.22386645	656	1083	854
63929	NM_001204827,NM_022098	XPNPEP3	X-prolyl aminopeptidase 3	1.024389873	1.058319698	3.893056	3.666617	2.823814	4.26583542	4.29881852	4.34348418	716	980	932
137886	NM_001077619,NM_001330535	UBXN2B	UBX domain protein 2B	1.126573482	1.058209724	7.494972	8.62091	7.193893	5.02271227	5.18967248	5.10190152	1237	1862	1610
92922	NM_033212	CCDC102A	coiled-coil domain containing 102	1.28074853	1.058206651	4.172513	5.357648	4.151739	3.44397655	3.77168848	3.51830855	388	664	505
8793	NM_003840	TNFRSF10D	TNF receptor superfamily membe	1.66495855	1.058134638	6.646038	11.078372	6.607172	4.57884974	5.29008925	4.65706199	899	2000	1170
55791	NM_001006945,NM_018372	LRIF1	ligand dependent nuclear recepto	1.024509686	1.058007932	26.704421	23.493641	25.021144	5.76857599	5.80287809	5.84847888	2101	2876	2734
4257	NM_001260511,NM_001260512	MGST1	microsomal glutathione S-transfe	1.154360075	1.057989003	385.114359	445.669253	383.019375	8.44744762	8.6539897	8.52854636	13666	21078	17783
80095	NM_001348022,NM_001348023	ZNF606	zinc finger protein 606	-1.035105115	1.057921585	4.337383	4.183695	4.294007	4.18329013	4.13629029	4.26018095	674	870	877
29882	NM_013366	ANAPC2	anaphase promoting complex sul	1.256237073	1.057887023	20.463194	25.729187	20.341557	5.77597182	6.09971488	5.85572008	2112	3545	2748
91647	NM_145691	ATPAF2	ATP synthase mitochondrial F1 c	-1.014714739	1.057687335	7.441808	7.340291	7.399388	3.61567874	3.59633059	3.69017701	442	582	575
2972	NM_001242786,NM_001242787	BRF1	BRF1, RNA polymerase III transc	1.22499551	1.057674279	13.423084	16.324705	13.758873	5.57171277	5.85892233	5.65095789	1828	2992	2378
64747	NM_001167903,NM_001289406	MFSD1	major facilitator superfamily dom	1.118547909	1.057579921	26.286293	29.436494	26.121212	5.94177327	6.10091747	6.0212652	2374	3548	3088
2583	NM_001276468,NM_001276469	B4GALNT1	beta-1,4-N-acetyl-galactosaminyl	-2.00243557	1.057574713	1.327288	0.541187	1.371007	2.04717061	1.3579479	2.10885534	123	82	160
83989	NM_001163417,NM_001163418	FAM172A	family with sequence similarity 17	1.118369044	1.057420281	4.046454	4.457863	3.971846	4.23884171	4.39215006	4.31524723	702	1049	913
4756	NM_001172623,NM_001172624	NEO1	neogenin 1	2.342673513	1.057375315	0.675339	1.584017	0.666099	2.50111114	3.57571888	2.56774382	183	573	238
23787	NM_001271641,NM_014341,NR	MTCH1	mitochondrial carrier 1	1.057952985	1.057267555	36.752849	38.670374	36.365915	6.37356266	6.45388734	6.4529631	3216	4546	4182
51428	NM_001321732,NM_001321830	DDX41	DEAD-box helicase 41	1.173228122	1.057158768	33.596821	39.414371	34.641635	6.42920394	6.65722024	6.50849312	3344	5242	4348
4207	NM_005919,NR_027307,NR_02	BORCS8-MEF2B	BORCS8-MEF2B readthrough	1.051137532	1.057152025	4.865354	5.181714	4.851857	3.49375703	3.55948145	3.56701931	403	566	524
30011	NM_001024666,NM_001184960	SH3KBP1	SH3 domain containing kinase bi	-1.307432832	1.05712559	32.140062	24.626602	31.433478	7.02107776	6.63774244	7.1006259	5060	5171	6579
5584	NM_002740	PRKCI	protein kinase C iota	-1.095990773	1.057069433	20.753838	18.957144	20.615108	6.63952946	6.50867837	6.7188204	3875	4724	5038
84938	NM_032852,NM_178221	ATG4C	autophagy related 4C cysteine pe	-1.142814689	1.056655582	5.219103	4.592902	5.232682	3.89300127	3.71417485	3.96730515	544	636	707
100113393	NR_003695	SNORD12B	small nucleolar RNA, C/D box 12	1.419779319	1.056648628	4.828381	7.193982	5.007153	0.59399636	0.7861039	0.62147005	20	38	26
3651	NM_000209	PDX1	pancreatic and duodenal homeot	1.196443995	1.056648628	0.203252	0.243481	0.198673	0.59399636	0.68708806	0.62147005	20	32	26
9761	NM_001303627,NM_001303628	MLEC	malectin	-1.025784984	1.056463767	29.968805	29.25366	29.760539	7.54544126	7.50891147	7.62427292	7295	9502	9479
441208	NR_003502	ZNRF2P1	zinc and ring finger 2 pseudogen	-1.331384061	1.056334602	0.12862	0.091479	0.120812	0.3273552	0.25173066	0.34394329	10	10	13
579	NM_001189	NKX3-2	NK3 homeobox 2	1.491607957	1.056334602	0.116508	0.17459	0.107032	0.3273552	0.46599441	0.34394329	10	20	13
3978	NM_000234,NM_001289063,NM	LIG1	DNA ligase 1	1.087004034	1.056277518	23.148577	27.14887	24.358747	6.46666486	6.58571949	6.54478691	3433	4986	4460
25941	NM_001271949,NM_001271950	TPGS2	tubulin polyglutamylase complex	-1.069967336	1.056222922	11.602957	9.898793	11.623993	4.71659644	4.62285328	4.79259544	993	1240	1290
10422	NM_016172	UBAC1	UBA domain containing 1	1.086328736	1.05604693	19.9242	21.667477	19.766563	5.21800288	5.33438844	5.29462408	1422	2064	1847
4192	NM_001012333,NM_001012334	MDK	midkine (neurite growth-promotin	-1.917149047	1.055940997	121.525343	65.34424	132.359623	6.88654921	5.9586963	6.96443417	4606	3210	5982
5431	NM_000938,NM_001303268,NM	POLR2B	RNA polymerase II subunit B	1.046404942	1.055790214	40.085951	43.633479	40.298442	7.33442139	7.39946725	7.4122735	6297	8804	8177
283149	NM_182557	BCL9L	B-cell CLL/lymphoma 9 like	-1.764796824	1.055786142	31.232506	17.714884	30.985722	7.88448861	7.0696356	7.96248454	9238	6994	11996
9320	NM_001284214,NM_001284215	TRIP12	thyroid hormone receptor interact	1.181750861	1.055733809	21.134296	25.097697	21.024008	7.68751079	7.92736288	7.76538846	8054	12717	10458
6945	NM_170607,NM_198204,NM_19	MLX	MLX, MAX dimerization protein	-1.08946266	1.055721699	28.102812	25.599186	27.620569	6.04500033	5.92333168	6.12207949	2553	3131	3315
1622	NM_001079862,NM_001079863	DBI	diazepam binding inhibitor, acyl-C	-1.192928932	1.055700194	216.312568	181.611671	215.193733	7.06207917	6.80964682	7.13971124	5207	5832	6761
401265	NM_001003760	KLHL31	kelch like family member 31	1.250713852	1.05565953	0.304711	0.381897	0.302421	1.43646263	1.64851465	1.4863075	67	112	87
102724560	NM_001321073	CBSL	cystathionine-beta-synthase like	-1.089909978	1.055604529	1.354281	1.249155	1.348614	2.05603457	1.96260273	2.11576522	124	152	161
266655	NR_015427	LINC00094	long intergenic non-protein codin	1.901737225	1.05540601	2.15444	4.106283	2.139975	3.70573844	4.5798	3.77773686	473	1202	614
84181	NM_032221	CHD6	chromodomain helicase DNA bin	1.253540551	1.055169247	6.465169	8.112336	6.410186	6.11028539	6.43207559	6.18667065	2673	4477	3469
81928	NM_031215	CABLES2	Cdk5 and Abl enzyme substrate 2	1.083779947	1.054760006	4.854564	5.27116	4.81209	4.24078667	4.3509637	4.31374565	703	1018	912
6659	NM_003107	SOX4	SRY-box 4	-1.065422237	1.054752149	1.982123	1.862535	1.9637	3.38187998	3.29945612	3.45160249	370	464	480
4267	NM_001122898_chrX,NM_0011	CD99	CD99 molecule (Xg blood group)	2.236619805	1.054690668	6.901778	15.875314	7.150613	3.34619087	4.42708835	3.4156568	360	1076	467
171022	NR_026690	ABHD11-AS1	ABHD11 antisense RNA 1 (tail to	-4.91565869	1.054685889	4.078099	0.80051	4.025016	1.5285023	0.46599441	1.57922529	74	20	96
339524	NR_026985,NR_026986,NR_02	LINC01140	long intergenic non-protein codin	-1.542467578	1.05460329	0.388535	0.219707	0.375732	0.95786335	0.68708806	0.99569924	37	32	48
93343	NM_001304547,NM_138401	MVB12A	multivesicular body subunit 12A	-1.168674117	1.054431612	19.042601	16.529793	18.911133	4.80863452	4.59239547	4.88244775	1061	1213	1376
283450	NM_001109662	HECTD4	HECT domain E3 ubiquitin protei	-1.182936207	1.054348827	6.314316	5.344759	6.257676	6.58507119	6.34543779	6.66065085	3730	4213	4837
51647	NM_016062,NM_024525,NM_04	FAM96B	family with sequence similarity 96	1.018693092	1.054261293	88.799728	90.82463	88.547834	5.97895271	6.00525353	6.05401081	2437	3317	3160
79074	NM_001286537,NM_024093	C2orf49	chromosome 2 open reading fran	1.237965517	1.054210855	6.234667	7.718741	6.179423	4.77413325	5.07196145	4.84759061	1035	1712	1342
3654	NM_001025242,NM_001025243	IRAK1	interleukin 1 receptor associated	1.360402188	1.054165099	47.293019	64.28106	46.773515	7.36148853	7.80320207	7.43713981	6417	11664	8320
10400	NM_001267551,NM_001267552	PEMT	phosphatidylethanolamine N-met	1.089003997	1.054000876	13.510687	14.729981	13.476152	3.84009957	3.95487459	3.91081999	523	761	678
57688	NM_020928	ZSWIM6	zinc finger SWIM-type containing	1.11160598	1.053992917	6.765015	7.531364	6.704772	5.21997612	5.36874065	5.29386268	1424	2115	1846

55257	NM_018270,NR_136405	MRGBP	MRG domain binding protein	1.164698246	1.053815395	23.955047	27.902052	23.788877	6.08939006	6.30635522	6.16393291	2634	4099	3414
26820	NR_002447	SNORD24	small nucleolar RNA, C/D box 24	1.163583	1.053731766	9.169397	10.883751	9.214393	0.7550755	0.84852988	0.78645063	27	42	35
4065	NM_002349	LY75	lymphocyte antigen 75	1.030472629	1.053536484	3.585831	3.698146	3.5491	4.65521171	4.69682839	4.72754788	950	1308	1231
80021	NM_001347004,NM_001347005	TMEM62	transmembrane protein 62	-1.391851862	1.053375585	9.907043	7.240735	9.610823	4.72356761	4.26774474	4.79582587	998	958	1293
23241	NM_001100913,NM_001243127	PACS2	phosphofurin acidic cluster sortin	1.160895083	1.0533567	8.154407	9.485429	8.086795	5.7502602	5.96178846	5.82389986	2074	3217	2687
23660	NM_001318082,NM_001318083	ZKSCAN5	zinc finger with KRAB and SCAN	-1.345803147	1.053330146	5.304682	4.00432	5.244638	4.46698912	4.0608449	4.53864966	829	823	1074
7368	NM_001128174,NM_001322112	UGT8	UDP glycosyltransferase 8	1.244469558	1.05317992	8.157034	10.16809	8.02339	5.08141332	5.38857221	5.15401818	1290	2145	1671
166785	NM_172250	MMAA	methylmalonic aciduria (cobalam	-1.400392761	1.05281743	0.95389	0.679999	0.944529	2.70647097	2.30630165	2.7696269	217	207	281
7629	NM_001292032,NM_003427	ZNF76	zinc finger protein 76	1.065129016	1.052689538	10.258407	11.020547	10.119023	4.76470172	4.85249002	4.83613057	1028	1463	1331
3219	NM_024017	HOXB9	homeobox B9	-1.587262233	1.052664698	24.917202	15.711846	24.651972	6.05553602	5.40164341	6.12849949	2572	2165	3330
84708	NM_001126328,NM_032622	LNX1	ligand of numb-protein X 1	1.221005411	1.052655889	1.597833	1.951741	1.578518	2.54584744	2.78861701	2.6075054	190	310	246
23484	NM_001128208,NM_015344	LEPROTL1	leptin receptor overlapping transc	-1.036669141	1.052518779	13.970067	13.810457	14.263685	5.20511061	5.15458457	5.27700936	1409	1816	1824
1315	NM_001144061,NM_001144062	COPB1	coatomer protein complex subun	-1.444355385	1.052517646	89.17414	61.724355	88.114627	8.24395347	7.715635	8.31756109	11863	10974	15357
55844	NM_001291310,NM_018461,NR	PPP2R2D	protein phosphatase 2 regulatory	1.122644461	1.052495628	9.279803	10.415915	9.183747	5.56551847	5.72909676	5.6378168	1820	2730	2356
117143	NM_053053	TADA1	transcriptional adaptor 1	-1.186902899	1.052241491	8.427629	7.108541	8.331592	4.28103662	4.04760082	4.35082401	724	815	937
10081	NM_005707	PDCD7	programmed cell death 7	-1.085811952	1.052229593	8.351817	7.701955	8.262979	4.59718428	4.48350191	4.66768061	911	1121	1179
3268	NM_006076	AGFG2	ArfGAP with FG repeats 2	-1.168084023	1.05216441	1.660684	1.422588	1.642238	3.13654562	2.93964205	3.20177275	306	350	396
22907	NM_001330990,NM_014966,NM	DHX30	DExH-box helicase 30	1.172908138	1.05166142	17.294013	19.54783	16.88279	6.05774429	6.28464676	6.12935334	2576	4037	3332
54858	NM_001300927,NM_001308366	PGPEP1	pyroglutamyl-peptidase I	-1.621600681	1.051659471	5.496525	3.618204	5.390052	5.17592889	4.50304006	5.24664042	1380	1137	1785
56474	NM_001144002,NM_019857,NM	CTPS2	CTP synthase 2	1.065528135	1.051403652	3.354764	3.517422	3.237783	3.7744826	3.859571	3.84165053	498	709	644
112483	NM_001320845,NM_001320846	SAT2	spermidine/spermine N1-acetyltra	-1.038055922	1.051319775	13.53402	13.236682	13.580727	3.79315817	3.74321825	3.86028525	505	650	653
11257	NR_015381	TP53TG1	TP53 target 1 (non-protein codin	-1.817401461	1.051266993	7.899822	4.342062	7.833615	2.69516719	2.0043309	2.75642288	215	158	278
10312	NM_001351059,NM_006019,NM	TCIRG1	T-cell immune regulator 1, ATPas	-1.01697555	1.051101895	98.45658	96.743872	97.60153	7.9264809	7.90229065	7.99809592	9512	12497	12297
55125	NM_032142	CEP192	centrosomal protein 192	-1.138756013	1.050951421	10.198461	8.965776	10.073993	6.32580276	6.14083071	6.39662902	3110	3649	4020
25820	NM_005744	ARIH1	ariadne RBR E3 ubiquitin protein	1.040775402	1.050900279	12.088078	12.597071	11.939532	5.97311481	6.02987599	6.04363098	2427	3375	3137
79591	NM_024541	C10orf76	chromosome 10 open reading fra	-1.145913309	1.050897432	4.530449	3.958387	4.477249	4.25625269	4.07069878	4.32422401	711	829	919
3108	NM_006120	HLA-DMA	major histocompatibility complex,	-1.079044321	1.050810873	16.322577	15.148175	16.139198	4.23689412	4.13315895	4.30470323	701	868	906
93100	NM_001286829,NM_145201	NAPRT	nicotinate phosphoribosyltransfer	-1.4884328	1.050792888	110.600337	74.390699	109.209992	7.5250369	6.9550531	7.59613765	7192	6456	9295
79370	NM_030766,NM_138722,NM_13	BCL2L14	BCL2 like 14	-1.705158347	1.050776237	1.413357	0.838171	1.430713	1.88750088	1.36863803	1.9400446	106	83	137
3276	NM_001207042,NM_001536,NM	PRMT1	protein arginine methyltransferas	-1.032014171	1.050695463	217.4722	211.431245	215.642705	8.22835432	8.18304512	8.29946743	11735	15193	15165
57570	NM_001350253,NM_001350254	TRMT5	tRNA methyltransferase 5	1.131524726	1.050689162	6.289472	7.37938	6.466171	5.14302819	5.31655563	5.21239866	1348	2038	1742
79066	NM_024086	METTL16	methyltransferase like 16	1.143077659	1.050586356	3.826942	4.379804	3.77785	4.48842972	4.67330781	4.55653526	842	1286	1088
23005	NM_001128608,NM_001265611	MAPKBP1	mitogen-activated protein kinase	1.193591903	1.050543197	3.11824	3.666094	3.031518	4.49984402	4.7448025	4.56791716	849	1354	1097
84320	NM_032360	ACBD6	acyl-CoA binding domain contain	1.138306745	1.050209519	24.351805	27.759954	24.040983	5.29770564	5.48014385	5.36663322	1505	2289	1944
5190	NM_000287,NM_001316313,NR	PEX6	peroxisomal biogenesis factor 6	-1.45570564	1.050118956	7.678078	5.252198	7.572447	4.75656819	4.23890304	4.82457876	1022	938	1320
79157	NM_001242532,NM_001242533	MFSD11	major facilitator superfamily dom	-1.259935752	1.050072741	15.068791	12.289576	14.995663	5.52618969	5.20093212	5.59518964	1770	1877	2286
131601	NM_001136053,NM_001142646	TPRA1	transmembrane protein adipocyte	1.360273635	1.049975743	15.36084	20.927088	15.064827	4.89515141	5.32618529	4.96320452	1129	2052	1458
89839	NM_001039841	ARHGAP11B	Rho GTPase activating protein 1	-1.041751219	1.049954035	11.644235	11.193496	11.478819	3.64828273	3.59405489	3.71313983	453	581	585
25888	NM_001006656,NM_001308424	ZNF473	zinc finger protein 473	-1.057114029	1.049907198	6.12782	5.803406	6.060882	4.85254141	4.77525187	4.92043435	1095	1384	1414
64430	NM_001330177,NM_022495	PCNX4	pecanex homolog 4 (Drosophila)	1.122540403	1.049887451	21.139012	24.203956	20.493124	6.48647187	6.65148654	6.55594399	3481	5221	4495
440295	NM_001291420,NM_001291420	GOLGA6L9	golgin A6 family-like 9	-1.073764869	1.049827765	8.000403	7.470123	7.911027	5.11573264	5.01611219	5.18391472	1322	1645	1707
9110	NM_004687	MTMR4	myotubularin related protein 4	-1.356808873	1.049819236	19.709438	14.543436	19.446009	6.81850685	6.38283049	6.88804282	4392	4325	5671
2588	NM_000512,NM_001323543,NM	GALNS	galactosamine (N-acetyl)-6-sulfat	1.71937955	1.049675893	14.038842	24.13959	13.82261	5.04959013	5.81318676	5.11747697	1261	2897	1628
114044	NR_002776,NR_110565,NR_11(	MCM3AP-AS1	MCM3AP antisense RNA 1	1.472841342	1.049623582	1.938354	2.939033	1.950948	2.53954085	3.01642592	2.59766727	189	372	244
79818	NM_024762	ZNF552	zinc finger protein 552	1.017427111	1.049597351	4.546672	4.629099	4.486764	3.51319803	3.53596345	3.57706795	409	556	528
10475	NM_006355	TRIM38	tripartite motif containing 38	1.103498058	1.049539577	4.632174	5.117623	4.568448	4.72217607	4.85913858	4.78935776	997	1470	1287
23144	NM_015117	ZC3H3	zinc finger CCCH-type containing	-1.11307725	1.049506316	12.70906	11.427591	12.533543	5.37677671	5.22613324	5.44485344	1592	1911	2055
7867	NM_001243925,NM_001243926	MAPKAPK3	mitogen-activated protein kinase-	-1.076939916	1.049475417	11.228543	10.42261	11.063228	4.85508302	4.75196505	4.92240621	1097	1361	1416
85406	NM_032364	DNAJC14	DnaJ heat shock protein family (I	-1.02209503	1.049473898	11.941173	11.693016	11.771996	5.31165148	5.28092234	5.37960921	1520	1987	1962
57406	NM_001320126,NM_020676	ABHD6	abhydrolase domain containing 6	1.316155389	1.049430657	1.479179	1.945782	1.483588	2.19894236	2.51796519	2.2537152	141	248	182
79969	NM_001031722,NM_001190724	ATAT1	alpha tubulin acetyltransferase 1	-1.116813851	1.049408869	8.216589	6.842545	7.488859	4.14848821	3.99854533	4.21424082	657	786	848
22908	NM_001319072,NM_001319073	SACM1L	SAC1 suppressor of actin mutatic	-1.001296347	1.04940602	8.872238	8.786995	8.683313	5.04736934	5.04555672	5.11489271	1259	1680	1625
54960	NM_001042479,NM_001042480	GEMIN8	gem nuclear organelle associate	1.125500525	1.049381452	2.116187	2.404167	2.114177	2.92058788	3.06980877	2.98115841	258	388	333
84309	NM_001193452,NM_032349	NUDT16L1	nudix hydrolase 16 like 1	1.263229932	1.049130113	12.001749	15.37176	12.122208	4.12129674	4.44108339	4.18661421	644	1087	831
51149	NM_001017987,NM_016175	MRNIP	MRN complex interacting protein	1.110561974	1.049089486	14.16511	17.395346	13.977745	4.13600195	4.27912189	4.20130594	651	966	840
84263	NM_001195822,NM_032303,NR	HSDL2	hydroxysteroid dehydrogenase lil	1.060707621	1.049027644	23.97816	25.30863	23.544594	6.30410949	6.38809405	6.37231107	3063	4341	3952
261729	NM_001040665,NM_001040666	STEAP2	STEAP2 metalloreductase	-1.537341802	1.048580739	5.320434	3.565346	5.361234	5.12313239	4.52470876	5.18965667	1329	1155	1714
55591	NM_001352088,NM_001352089	VEZT	vezatin, adherens junctions trans	-1.492373338	1.048496213	30.753454	20.475354	29.776732	7.14074354	6.56814636	7.2085936	5501	4925	7094
5430	NM_000937	POLR2A	RNA polymerase II subunit A	1.175944268	1.048386053	27.779648	32.704174	27.368176	7.51763615	7.75028061	7.58544384	7155	11242	9226
221	NM_000694,NM_001030010,NM	ALDH3B1	aldehyde dehydrogenase 3 famil	-2.025740059	1.0482481	59.695455	29.607889	58.396163	7.34259498	6.33320993	7.41016719	6333	4177	8165
26284	NM_001317985,NM_001317986	ERAL1	Era like 12S mitochondrial rRNA	1.191646755	1.048115561	33.779697	40.627824	33.112811	5.97778702	6.22706742	6.04453654	2435	3877	3139
9049	NM_001302959,NM_001302960	AIP	aryl hydrocarbon receptor interac	1.053859093	1.048070953	45.997158	48.530979	45.267542	5.81826596	5.8926442	5.8848325	2176	3064	2805
23312	NM_001174116,NM_001174117	DMXL2	Dmx like 2	-1.579569151	1.047964952	8.058244	4.930716	7.681944	6.34536805	5.69605597	6.41214851	3153	2667	4064
89845	NM_001198934,NM_001350518	ABCC10	ATP binding cassette subfamily (	1.194395611	1.047864273	4.536001	5.574828	4.478153	4.54304361	4.7892446	4.60767403	876	1398	1129
80155	NM_057175	NAA15	N(alpha)-acetyltransferase 15, N	1.137979503	1.047760381	17.299971	19.710907	17.036034	6.57739575	6.76204243	6.64401774	3710	5641	4781
7468	NM_001042424,NM_007331,NM	NSD2	nuclear receptor binding SET dor	-1.160914959	1.047533678	54.846043	46.875952	53.901199	8.64681062	8.43212583	8.71364438	15697	18066	20224
64116	NM_001135146,NM_001135147	SLC39A8	solute carrier family 39 member 8	-1.369663243	1.047418239	11.262494	8.069753	10.924403	5.14094677	4.70212092	5.20593745	1346	1313	1734
387841	NR_003932	RPL13AP20	ribosomal protein L13a pseudoge	-2.529511215	1.047362846	20.463488	8.082669	20.163904	3.82462152	2.63327382	3.88678411	517	273	666
5498	NM_000309,NM_001122764,NM	PPOX	protoporphyrinogen oxidase	-1.081454091	1.047266955	5.31421	4.988394	5.15691	3.34979977	3.24827687	3.41004634	361	446	465
11044	NM_001171805,NM_001171806	PAPD7	poly(A) RNA polymerase D7, nor	1.263023357	1.047062309	12.07073	15.157276	11.857538	5.4065374	5.73633751	5.47135948	1626	2744	2094
9880	NM_014830	ZBTB39	zinc finger and BTB domain cont	-1.230701657	1.04703409	2.30232	1.871157	2.265401	3.90531607	3.62781914	3.96730515	549	596	707
79575	NM_024527	ABHD8	abhydrolase domain containing 8	1.217528612	1.04684381	7.744774	9.437824	7.617395	4.06751962	4.33606188	4.12972136	619	1007	797
4259	NM_004528	MGST3	microsomal glutathione S-transfe	-1.218700498	1.046805504	34.335331	28.207609	33.785027	5.69387492	5.41459724	5.75862518	1993	2185	2566
125488	NM_001135993,NM_001243425	TTC39C	tetratricopeptide repeat domain 3	1.157069781	1.046430249	7.833177	7.600565	7.147414	4.11494833	4.31410799	4.17673594	641	991	825
90121	NM_001346789,NM_001346790	TSR2	TSR2, ribosome maturation facto	1.152998655	1.046104541	4.882807	5.601696	4.763476	4.35472906	4.55075824	4.41665393	764	1177	983
23031	NM_015016	MAST3	microtubule associated serine/thr	-1.094703593	1.046029923	3.76404	3.442663	3.700369	4.49821892	4.37369523	4.56033921	848	1035	1091
50619	NM_022047	DEF6	DEF6, guanine nucleotide excha	-1.389565151	1.045970451	12.725668	9.165837	12.509214	4.88025587	4.42452914	4.94294958	1117	1074	1437
9898	NM_001127320,NM_001287815	UBAP2L	ubiquitin associated protein 2 like	1.252716096	1.045821264	44.037563	55.440259	42.340104	7.36059442	7.68388676	7.42484717	6413	10734	8249
203228	NM_001256054,NM_018325,NM	C9orf72	chromosome 9 open reading fran	-2.129662723	1.045672057	2.901649	1.380543	3.001488	3.29469475	2.36086138	3.35270793	346	217	445
140459	NM_001202403,NM_017873,NM	ASB6	ankyrin repeat and SOCS box co	1.207138315	1.045494868	8.99569	10.834819	8.79675	5.3670153	5.63261167	5.42968364	1581	2550	2033
222166	NM_152793	MTURN	maturin, neural progenitor differe	-1.697205046	1.045451301	4.98977	2.941942	4.902013	4.87023937	4.14097459	4.93222525	1109	873	1426
8881	NM_001078645,NM_001318517	CDC16	cell division cycle 16	-1.01955061	1.045413519	19.889159	19.504546	19.58802	5.53493448	5.50760765	5.5976597	1781	2334	2290
57418	NM_024100	WDR18	WD repeat domain 18	1.197868468	1.045347875	26.085621	31.280058	25.626667	5.33641922	5.59099458	5.39885696	1547	2476	1989
55274	NM_018288,NM_133325	PHF10	PHD finger protein 10	1.344647091	1.045341851	8.529639	11.50399	8.382086	3.89793981	4.30021519	3.95772276	546	981	702
311	NM_001157,NM_001278407,NM	ANXA11	annexin A11	-2.327800179	1.045321574	82.856163	35.843057	81.534903	7.68197415	6.47227994	7.74561722	8023	4605	10315
286046	NM_173683,NR_138152,NR_13	XKR6	XK related 6	1.18354793	1.045299339	2.078751	2.395911	1.886142	1.80577475	1.98361768	1.85174813	98	155	126
8153	NM_005440	RND2	Rho family GTPase 2	-2.707469867	1.0452706	0.391653	0.144575	0.387132	1.38110603	0.669904	1.42086173	63	31	81
55150	NM_018111	C19orf73	chromosome 19 open reading fra	-1.128476299	1.045247636	1.70175	1.52704	1.683519	1.16869613	1.07428646	1.20457043	49	58	63
81545	NM_001271723,NM_030793	FBXO38	F-box protein 38	1.082233198	1.045227916	12.921871	13.562658	12.339476	5.64551013	5.7573359	5.70808361	1926	2785	2476
693221	NR_030366	MIR636	microRNA 636	-1.039082097	1.045206358	9.229584	8.887136	8.98931	0.91952222	0.89363826	0.95003448	35	45	45
388799	NM_001013646	FAM209B	family with sequence similarity 20	-1.436500499	1.045170301	1.024861	0.718668	1.033989	0.77668602	0.58077243	0.80366934	28	26	36
10902	NM_001164326,NM_001300961	BRD8	bromodomain containing 8	-1.021157863	1.045141725	46.799299	46.256788	45.648115	7.15860031	7.12860745	7.22186394	5570	7288	7160
51	NM_001185039,NM_004035,NM	ACOX1	acyl-CoA oxidase 1	1.754246206	1.045081081	7.456456	13.072074	7.292014	5.81369997	6.61351108	5.87621116	2169	5084	2788
22850	NM_014913	ADNP2	ADNP homeobox 2	-1.255003944	1.045059481	15.554839	12.404417	15.275251	6.36778967	6.04453358	6.43062346	3203	3410	4117
79992	NR_024277	AGPAT4-IT1	AGPAT4 intronic transcript 1	1.599207805	1.044990878	0.185416	0.312135	0.183694	0.43999062	0.65251278	0.45708734	14	30	18
65989	NM_001286655,NM_001286656	DLK2	delta like non-canonical Notch lig	1.599207805	1.044990878	0.172936	0.279316	0.181769	0.43999062	0.65251278	0.45708734	14	30	18
255031	NR_015401,NR_072981,NR_07	LINC00957	long intergenic non-protein codin	-2.061547509	1.044990878	0.20106	0.101956	0.199638	0.43999062	0.2284445	0.45708734	14	9	18
11128	NM_007055	POLR3A	RNA polymerase III subunit A	-1.247844789	1.044905697	5.183631	4.158347	5.089882	5.10829474	4.79915696	5.16987408	1315	1408	1690
102723859	NM_001291466,NM_001291466	TBC1D3E	TBC1 domain family member 3E	-1.023713338	1.044884051	34.812401	34.047001	34.190044	6.14860191	6.11527096	6.21107345	2746	3584	3529

152225	NR_026934	LINC02085	long intergenic non-protein codin	2.331861258	1.044636274	0.076785	0.208119	0.087035	0.23670415	0.50527715	0.24657076	7	22	9
2066	NM_001042599,NM_005235	ERBB4	erb-b2 receptor tyrosine kinase 4	2.542479768	1.044636274	0.015338	0.039342	0.015088	0.23670415	0.54351856	0.24657076	7	24	9
100128233	NR_103769	LINC02324	long intergenic non-protein codin	-2.292028919	1.044636274	0.117297	0.04078	0.114881	0.23670415	0.10601836	0.24657076	7	4	9
677800	NR_002954	SNORA12	small nucleolar RNA, H/ACA box	-1.329394012	1.044636274	1.244013	0.8034	1.141648	0.23670415	0.18071335	0.24657076	7	7	9
339175	NM_181725	METTL2A	methyltransferase like 2A	-1.05269739	1.044453439	12.147633	11.551917	11.926168	4.94491448	4.87328317	5.00567345	1170	1485	1503
10741	NM_006606	RBBP9	RB binding protein 9, serine hydr	1.208737452	1.044372256	4.85857	5.881788	4.772468	4.26774435	4.52828878	4.3272039	717	1158	921
255189	NM_213600,NR_033151	PLA2G4F	phospholipase A2 group IVF	-1.273673789	1.044366413	0.916073	0.723226	0.903552	2.04717061	1.79043244	2.09493548	123	129	158
10437	NM_006332	IFI30	IFI30, lysosomal thiol reductase	-1.071579673	1.044335699	46.697128	43.612698	45.812187	5.57711104	5.47952756	5.63841672	1835	2288	2357
79886	NM_001167575,NM_024828	CAAP1	caspase activity and apoptosis in	1.013473727	1.044276456	4.983898	5.074639	4.860683	3.89547266	3.91349545	3.95387191	545	738	700
9173	NM_001282408,NM_003856,NM	IL1RL1	interleukin 1 receptor like 1	7.021575781	1.044246752	2.162681	15.119944	2.105562	3.19389071	5.86412557	3.24967672	320	3003	411
101928741	NR_146488	LINC02065	long intergenic non-protein codin	-1.999702853	1.044047144	4.29428	2.149062	4.214388	2.98234021	2.15396266	3.03682664	271	181	348
9169	NM_004719	SCAF11	SR-related CTD associated facto	-1.119126294	1.044023664	14.776408	13.218298	14.498429	6.78524352	6.62442377	6.84684785	4291	5123	5510
55975	NM_001031710,NM_001172428	KLHL7	kelch like family member 7	1.011628184	1.044015038	12.449725	10.22739	12.15236	4.53830704	4.55427428	4.59783705	873	1180	1121
3397	NM_002165,NM_181353	ID1	inhibitor of DNA binding 1, HLH p	-1.095362928	1.043918061	148.698612	138.446857	147.025787	7.2654005	7.13488306	7.32701555	6001	7320	7705
4817	NM_001185092,NM_001185093	NIT1	nitrilase 1	1.120830516	1.043848485	17.145052	19.819191	17.493193	5.02609951	5.18589965	5.08615607	1240	1857	1592
6618	NM_003083,NR_030717	SNAPC2	small nuclear RNA activating con	-1.046848889	1.043813637	8.971455	8.714019	8.875099	3.86300966	3.80158144	3.92071794	532	679	683
11319	NM_001135752,NM_001135753	ECD	ecdysoneless cell cycle regulator	-1.423276855	1.043635452	20.635737	14.64316	20.449246	5.5205971	5.02458641	5.5809041	1763	1655	2263
1855	NM_001330311,NM_004421	DVL1	dishevelled segment polarity prot	1.149021773	1.043589118	69.098028	79.89302	67.978001	7.65013112	7.84960801	7.71138576	7847	12047	10072
51304	NM_001135179,NM_001330761	ZDHHC3	zinc finger DHHC-type containing	-1.006945175	1.04351141	8.084503	8.124668	8.535053	6.48401081	6.47413731	6.54478691	3475	4611	4460
116150	NM_138459	NUS1	NUS1 dehydrodolichyl diphospha	1.03053473	1.043492623	9.205695	9.498621	9.026297	5.46094475	5.50336969	5.52100322	1690	2327	2169
64412	NM_001317012,NM_001317019	GZF1	GDNF inducible zinc finger protei	1.354805291	1.043435656	2.351805	3.153021	2.287847	3.56999691	3.97600158	3.62628732	427	773	548
440193	NM_001080414	CCDC88C	coiled-coil domain containing 88C	1.260397407	1.043407818	8.50669	10.733561	8.340263	5.98824449	6.31743308	6.04860456	2453	4131	3148
2885	NM_002086,NM_203506	GRB2	growth factor receptor bound pro	-1.194911597	1.043405724	48.71983	40.844866	47.791452	7.3010229	7.04589619	7.36194361	6152	6879	7895
7416	NM_003374,NR_036624,NR_03	VDAC1	voltage dependent anion channel	1.347385732	1.043133403	164.678363	222.329436	161.500522	8.38032972	8.809379	8.44107486	13043	23481	16734
1230	NM_001295	CCR1	C-C motif chemokine receptor 1	-1.683731466	1.043068546	0.517367	0.305255	0.502376	1.23264156	0.84852988	1.26797492	53	42	68
79872	NM_001284291,NM_024814,NR	CBLL1	Cbl proto-oncogene like 1	-1.325957961	1.042980701	9.260851	6.98375	9.065723	5.3571874	4.96154856	5.41645284	1570	1582	2014
146434	NM_152457	ZNF597	zinc finger protein 597	1.136246094	1.042725959	2.735914	3.11687	2.682693	3.97240126	4.14564373	4.02900114	577	876	740
9728	NM_001193489,NM_014701	SECISBP2L	SECIS binding protein 2 like	-1.279604706	1.042551044	5.447377	4.31148	5.404893	5.28833296	4.94289226	5.34694775	1495	1561	1917
3613	NM_014214	IMPA2	inositol monophosphatase 2	-1.110167573	1.042544988	7.773035	7.00736	7.612527	3.65995838	3.5216664	3.71541614	457	550	586
252969	NM_001135746,NM_001135747	NEIL2	nei like DNA glycosylase 2	1.188963511	1.042521855	2.615001	3.156937	2.399437	2.87121968	3.08932884	2.92325564	248	394	318
55082	NM_018011	ARGLU1	arginine and glutamate rich 1	1.029397986	1.042511626	55.626606	57.330391	54.503796	6.58277284	6.62414499	6.64222419	3724	5122	4775
10093	NM_001024959,NM_001024960	ARPC4	actin related protein 2/3 complex	1.082590162	1.042489559	23.445597	25.635297	22.994224	5.50773204	5.61980474	5.5664757	1747	2527	2240
126308	NM_130807	MOB3A	MOB kinase activator 3A	1.236764402	1.042435899	7.206363	8.925198	7.062384	4.6077728	4.90300262	4.66532766	918	1517	1177
127396	NM_152373	ZNF684	zinc finger protein 684	-1.10279227	1.042357732	3.887067	3.530748	3.806317	3.16140171	3.03667667	3.21470347	312	378	400
105371248	NR_146572	LINC02178	long intergenic non-protein codin	-1.576555549	1.042251495	2.362285	1.520182	2.327347	0.99521185	0.70406985	1.02535867	39	33	50
8569	NM_001135553,NM_003684,NM	MKNK1	MAP kinase interacting serine/thr	-1.004456007	1.042212939	15.563219	15.641502	15.310283	5.37766084	5.37140066	5.43590876	1593	2119	2042
2827	NM_005281	GPR3	G protein-coupled receptor 3	-1.360388234	1.042140481	1.352088	0.996451	1.328407	1.92669094	1.61299082	1.97085819	110	108	141
10668	NM_006568	CGRRF1	cell growth regulator with ring fing	1.108512009	1.041873886	12.569739	13.947026	12.309067	4.10430518	4.24471769	4.16012035	636	942	815
23030	NM_015015	KDM4B	lysine demethylase 4B	1.641815037	1.041862584	4.047996	6.650659	3.961816	4.528787	5.21950507	4.58544575	867	1902	1111
8693	NM_003774	GALNT4	polypeptide N-acetylgalactosamir	-1.940752451	1.04182969	3.204311	1.650952	3.136776	4.15675275	3.27409343	4.21263029	661	455	847
8452	NM_001257197,NM_001257198	CUL3	cullin 3	-1.113472735	1.04160989	17.707806	16.208318	17.355037	6.88344013	6.72975603	6.94176835	4596	5515	5888
27246	NM_014455	RNF115	ring finger protein 115	1.101880731	1.041566816	7.008552	7.730174	6.866392	3.84009957	3.97074877	3.89484064	523	770	670
6940	NM_001324339,NM_005649	ZNF354A	zinc finger protein 354A	-1.221653827	1.041505078	7.432223	6.089678	7.293083	4.28481203	4.01224533	4.34053776	726	794	930
26057	NM_001286771,NM_015574,NM	ANKRD17	ankyrin repeat domain 17	1.032005522	1.041445057	14.655175	15.152808	14.313168	7.1487935	7.19392972	7.20697674	5532	7628	7086
5954	NM_002901	RCN1	reticulocalbin 1	1.206880191	1.041434683	182.560806	220.579208	178.669632	8.80806873	9.07880085	8.86651339	17558	28313	22490
83596	NM_001040668,NM_001282516	BCL2L12	BCL2 like 12	-1.190031667	1.041401093	20.739051	17.839081	20.690551	5.23761487	4.99384339	5.29462408	1442	1619	1847
7227	NM_001282902,NM_001282903	TRPS1	transcriptional repressor GATA b	1.360859298	1.041231174	1.11265	1.506741	1.098957	3.56068436	3.97250183	3.614139	424	771	543
7071	NM_001032282,NM_005655,NR	KLF10	Kruppel like factor 10	2.107956931	1.041208013	13.072009	27.621291	12.713836	5.33277665	6.38973499	5.38962158	1543	4346	1976
5229	NM_005023	PGGT1B	protein geranylgeranyltransferase	1.008817887	1.041115629	10.525462	10.633904	10.299623	4.85635215	4.8685837	4.91251987	1098	1480	1406
6901	NM_000116,NM_001303465,NM	TAZ	tafazzin	-1.2976242	1.041043805	22.617044	17.344489	22.10023	5.40046018	5.03469021	5.45714754	1619	1667	2073
55971	NM_018842	BAIAP2L1	BAI1 associated protein 2 like 1	-1.478485252	1.041030258	41.971287	28.414368	41.059746	7.2381552	6.67858806	7.2957916	5888	5321	7539
26088	NM_001001560,NM_001172687	GGA1	golgi associated, gamma adaptin	1.007135591	1.040977037	37.107967	37.402588	35.908064	6.82403098	6.83419894	6.88146934	4409	5933	5645
10600	NM_001001992,NM_001032410	USP16	ubiquitin specific peptidase 16	1.039550086	1.040780954	18.305485	19.08987	17.945353	5.75775028	5.81269754	5.8143746	2085	2896	2669
25833	NM_001244682,NM_014352	POU2F3	POU class 2 homeobox 3	-1.589284412	1.0406444	0.643153	0.407272	0.636725	1.54118428	1.1381576	1.57922529	75	63	96
64582	NM_022571	GPR135	G protein-coupled receptor 135	-1.077399032	1.0406444	1.074801	0.993943	1.044431	1.54118428	1.47139626	1.57922529	75	93	96
26024	NM_015545	PTCD1	pentatricopeptide repeat domain	1.442587904	1.040588576	1.510081	2.182138	1.476452	3.18583679	3.66515371	3.23705705	318	613	407
22903	NM_001282550,NM_001282551	BTBD3	BTB domain containing 3	1.063509028	1.040581977	4.54718	4.813175	4.416023	4.46698912	4.55193121	4.52184068	829	1178	1061
25959	NM_001136191,NM_001329451	KANK2	KN motif and ankyrin repeat dom	-1.086629544	1.040495064	10.324004	9.484913	10.114513	5.72269098	5.6051891	5.7789005	2034	2501	2603
84451	NM_032435	MAP3K21	mitogen-activated protein kinase	1.063178676	1.040471125	6.967125	7.418084	6.816312	5.33732843	5.42359621	5.39318065	1548	2199	1981
51562	NM_001144891,NM_001308110	MBIP	MAP3K12 binding inhibitory prote	1.380197804	1.040431978	15.541837	21.492828	15.11359	4.68265777	5.13201715	4.73766195	969	1787	1240
28998	NM_014078	MRPL13	mitochondrial ribosomal protein L	1.110957292	1.04035314	46.018852	51.192467	44.989819	5.66519587	5.81416471	5.72116918	1953	2899	2499
403313	NM_203453	PLPP6	phospholipid phosphatase 6	1.046188062	1.040345729	4.074975	4.27181	3.99109	3.68302999	3.74321825	3.73574288	465	650	595
692223	NR_004403	SNORD97	small nucleolar RNA, C/D box 97	-1.261374274	1.040160678	12.502397	9.819944	12.111144	1.44997615	1.24644988	1.4863075	68	72	87
64324	NM_022455,NM_172349	NSD1	nuclear receptor binding SET dor	1.211426002	1.040160105	7.005887	8.481152	6.945401	6.48154555	6.75543907	6.53772946	3469	5615	4438
7161	NM_001126240,NM_001126241	TP73	tumor protein p73	1.62570043	1.040022034	0.999783	1.635776	0.975677	2.58923814	3.19521507	2.63662357	197	428	252
492303	NR_002830	GEMIN8P4	gem nuclear organelle associate	-1.248597577	1.03989682	1.30881	1.058065	1.280676	1.67378945	1.4614436	1.71282633	86	92	110
57095	NM_020362	PITHD1	PITH domain containing 1	1.040571061	1.039734646	21.691938	22.587656	21.185631	5.13990493	5.19568855	5.19456023	1345	1870	1720
169611	NM_001282715,NM_182487	OLFML2A	olfactomedin like 2A	1.272622789	1.039314108	1.689795	2.156439	1.650506	3.56068436	3.88212953	3.61169701	424	721	542
109617006	NR_145766	SNORA63B	small nucleolar RNA, H/ACA box	1.293290199	1.039166964	66.943855	86.579384	65.33194	3.19389071	3.52883263	3.24338068	320	553	409
91603	NM_052857	ZNF830	zinc finger protein 830	1.063895555	1.0389372	16.590418	17.671875	16.191557	4.79546226	4.88170383	4.84862794	1051	1494	1343
1211	NM_001076677,NM_001184760	CLTA	clathrin light chain A	1.088246305	1.038896934	69.05252	75.333492	67.617928	6.28585772	6.40636677	6.34021978	3024	4397	3864
64224	NM_022373	HERPUD2	HERPUD family member 2	-1.204675236	1.038678105	9.388455	7.801366	9.167834	4.78750079	4.52948015	4.8403084	1045	1159	1335
83451	NM_001145364,NM_001301058	ABHD11	abhydrolase domain containing 1	-2.015152517	1.038452568	43.360533	21.524117	41.4389	6.09477676	5.10510125	6.14843247	2644	1753	3377
23113	NM_015089	CUL9	cullin 9	-1.013149071	1.038425853	4.855863	4.799652	4.739703	5.23858851	5.22024304	5.29157606	1443	1903	1843
84299	NM_001330206,NM_032339	MIEN1	migration and invasion enhancer	-1.233322506	1.038148828	26.111916	21.222671	25.522251	5.24732179	4.95358253	5.29994267	1452	1573	1854
6477	NM_001006610,NM_003031	SIAH1	siah E3 ubiquitin protein ligase 1	-1.079284284	1.038105326	20.886581	19.335474	22.2111	5.57171277	5.46403427	5.62455491	1828	2263	2334
54704	NM_001161779,NM_001161780	PDP1	pyruvate dehyrogenase phospha	-1.140783742	1.038097413	19.055107	16.795013	18.592539	6.32442779	6.1369278	6.37771067	3107	3639	3967
203259	NM_001184940,NM_001184941	FAM219A	family with sequence similarity 21	1.700835868	1.038068491	3.547612	6.067253	3.502809	3.76640418	4.48841131	3.81642381	495	1125	632
715	NM_001733	C1R	complement C1r	-1.04876922	1.038020434	4.207303	4.021513	4.105419	3.36414578	3.30224686	3.4128543	365	465	466
57498	NM_001348729,NM_001348731	KIDINS220	kinase D interacting substrate 22	1.300361575	1.038019574	8.713917	11.378196	8.314305	5.99920131	6.37290924	6.05221098	2472	4295	3156
64760	NM_022749	FAM160B2	family with sequence similarity 16	-1.169375779	1.037927046	14.693594	12.584118	14.334277	5.76587717	5.54461018	5.81861581	2097	2396	2677
26136	NM_015641,NM_152829	TES	testin LIM domain protein	-1.215408633	1.037907346	17.607123	14.500686	17.209566	5.60606863	5.33097613	5.65866718	1873	2059	2391
1660	NM_001357,NR_033302	DHX9	DExH-box helicase 9	1.21984561	1.037826407	55.570749	68.310656	54.624981	7.97869335	8.26436359	8.03205063	9864	16077	12591
55901	NM_018676,NM_199263	THSD1	thrombospondin type 1 domain c	-1.563575097	1.037742201	1.573347	1.038057	1.562539	2.60139951	2.08433212	2.64620047	199	170	254
56123	NM_018933	PCDHB13	protocadherin beta 13	1.959207845	1.037567707	0.779472	1.536331	0.760399	1.87753473	2.64211237	1.91649468	105	275	134
27229	NM_001286414,NM_014444	TUBGCP4	tubulin gamma complex associat	1.033986124	1.037566421	7.651127	7.920213	7.46278	5.00794161	5.05468796	5.05952566	1224	1691	1562
23559	NM_012477	WBP1	WW domain binding protein 1	1.034782741	1.037550771	53.834457	55.772221	52.48135	6.02482449	6.07340943	6.07720657	2517	3480	3212
342909	NM_001037813	ZNF284	zinc finger protein 284	1.052813937	1.037534332	1.610988	1.694384	1.567673	2.96832247	3.03332122	3.01481644	268	377	342
677822	NR_002973	SNORA40	small nucleolar RNA, H/ACA box	-1.497948087	1.037534332	55.049427	36.883503	53.806408	2.96832247	2.47408961	3.01481644	268	239	342
55161	NM_018126	TMEM33	transmembrane protein 33	1.097251683	1.037447143	19.528828	21.451021	19.037804	7.20690743	7.3399385	7.25959375	5761	8446	7351
8396	NM_003559	PIP4K2B	phosphatidylinositol-5-phosphate	1.10488052	1.037422445	12.300926	13.609236	11.992355	6.12246788	6.26439661	6.17472633	2696	3980	3440
5822	NM_005049	PWP2	PWP2 periodic tryptophan protei	1.220322539	1.037235815	17.46209	21.328829	17.017738	5.81826596	6.10091747	5.87009434	2176	3548	2776
246269	NM_001323005,NM_145315,NR	AFG1L	AFG1 like ATPase	-1.217495259	1.037166737	0.966325	0.798236	0.940615	2.51403512	2.28388661	2.5576298	185	203	236
101927612	NR_108047,NR_108048	RNF139-AS1	RNF139 antisense RNA 1 (head	1.343356771	1.03708731	2.06315	2.18365	1.469387	2.0823043	2.41858604	2.12264217	127	228	162
2738	NM_138465	GLI4	GLI family zinc finger 4	-1.19158539	1.036990775	18.951654	15.913176	18.467846	4.68836995	4.44613902	4.73878137	973	1091	1241
119385	NM_133447	AGAP11	ArfGAP with GTPase domain, an	-1.401246439	1.03687841	4.093744	2.924598	3.990386	3.76370129	3.31887893	3.81217614	494	471	630
23352	NM_020765	UBR4	ubiquitin protein ligase E3 compo	1.046370625	1.036842003	23.159948	24.260151	22.56562	8.48833737	8.55355376	8.54039109	14060	19657	17930
57674	NM_001256071,NM_020954	RNF213	ring finger protein 213	-2.51669269	1.036661216	41.632666	17.056787	41.394716	9.63962984	8.31083389	9.69151055	31277	16605	39879
27243	NM_014453,NM_198426	CHMP2A	charged multivesicular body prot	-1.287329022	1.036642574	173.851814	135.180425	169.37034	7.28155132	6.91982512	7.33314301	6069	6299	7738

729533	NM_001123168,NM_001317901	FAM72A	family with sequence similarity 72	-1.826552388	1.036568039	22.479936	13.578081	22.508407	5.72477705	4.87796738	5.77563191	2037	1490	2597
4802	NM_001142587,NM_001142588	NFYC	nuclear transcription factor Y sub	1.059979329	1.036456492	17.833984	18.899204	17.543492	5.2579245	5.33983141	5.30826105	1463	2072	1865
114987	NM_001006615,NM_001012361	WDR31	WD repeat domain 31	-7.739291366	1.03629001	1.314249	0.159917	1.259103	2.85098947	0.84852988	2.89541791	244	42	311
4644	NM_000259,NM_001142495	MYO5A	myosin VA	-1.230393834	1.036259564	1.395827	1.135549	1.36129	4.13809052	3.8576751	4.18661421	652	708	831
843	NM_001206524,NM_001206542	CASP10	caspase 10	1.005249579	1.036236922	4.686262	4.91132	4.717187	4.6077728	4.61501835	4.65706199	918	1233	1170
92092	NM_080660	ZC3HAV1L	zinc finger CCCH-type containing	1.452368731	1.036201557	1.520298	2.208231	1.479028	1.84721644	2.255369	1.88448459	102	198	130
55168	NM_001193343,NM_018135	MRPS18A	mitochondrial ribosomal protein S	1.257954012	1.03604297	18.171561	22.954091	17.710704	4.45865714	4.77625587	4.50746311	824	1385	1050
57680	NM_001170629,NM_020920	CHD8	chromodomain helicase DNA bin	1.165167237	1.035964995	18.235543	21.321427	17.698122	7.18358814	7.40272153	7.23421981	5668	8824	7222
51313	NM_001031700,NM_001128424	FAM198B	family with sequence similarity 19	-2.114973509	1.035917868	2.460541	1.165304	2.396928	3.65704831	2.69827959	3.70399851	456	288	581
79796	NM_001077690,NM_001077691	ALG9	ALG9, alpha-1,2-mannosyltransfe	-1.691701387	1.035836491	5.288404	3.195733	5.168388	5.05954162	4.33060465	5.10884471	1270	1003	1618
55728	NM_001318359,NM_018177	N4BP2	NEDD4 binding protein 2	1.056603158	1.035779115	1.594912	1.686648	1.551618	4.01393646	4.08859126	4.0615736	595	840	758
84176	NR_002147	MYH16	myosin heavy chain 16 pseudoge	-2.705971331	1.035771103	1.678676	0.618696	1.635607	2.23841445	1.24644988	2.27855908	146	72	186
100506714	NR_038956,NR_038957	NUP50-AS1	NUP50 antisense RNA 1 (head to	-1.12915414	1.035619152	8.770617	7.769424	8.550236	3.61567874	3.45551618	3.66213029	442	523	563
100289230	NR_036530	LOC100289230	uncharacterized LOC100289230	-1.035328358	1.035595616	2.482944	2.408253	2.426082	2.47491059	2.43393638	2.51644942	179	231	228
153443	NM_152546	SRFBP1	serum response factor binding pr	1.364115409	1.035590513	5.923907	8.098236	5.770817	4.11918371	4.54487907	4.16678956	643	1172	819
63941	NM_031231,NM_031232	NECAB3	N-terminal EF-hand calcium bind	1.296929259	1.03555032	14.139618	18.256776	13.734963	4.81648053	5.17984253	4.86512445	1067	1849	1359
92421	NM_152284	CHMP4C	charged multivesicular body prot	-1.331723444	1.035109489	13.020761	9.789081	12.667546	4.60927912	4.21540721	4.65706199	919	922	1170
1892	NM_004092	ECHS1	enoyl-CoA hydratase, short chair	1.30113605	1.034924969	43.196438	56.279751	42.015354	5.830587	6.20449569	5.87925987	2195	3816	2794
3880	NM_002276	KRT19	keratin 19	-1.111747949	1.034912728	1796.870605	1618.042969	1747.49939	11.33006165	11.17729435	11.37955191	101035	121426	128605
346389	NM_182762	MACC1	MACC1, MET transcriptional regu	-6.876383394	1.034841195	8.364914	1.217428	8.134829	6.24040736	3.56646274	6.28917599	2929	569	3728
230	NM_005165	ALDOC	aldolase, fructose-bisphosphate	-1.259684102	1.034782211	6.273969	4.977854	6.092112	3.47073972	3.17099075	3.51569869	396	420	504
768096	NR_003244	HAR1A	highly accelerated region 1A (nor	-2.037178574	1.034721283	0.511963	0.24726	0.496601	1.26358189	0.75384785	1.29257653	55	36	70
26502	NM_001083618,NM_001083608	NARF	nuclear prelamin A recognition fa	-1.127194597	1.034655398	26.04763	23.313066	25.484065	5.42803514	5.25954295	5.47606585	1651	1957	2101
9751	NM_001318234,NM_014723	SNPH	syntaphilin	1.5438025	1.034503115	2.722939	4.169227	2.642262	3.84522223	4.43600998	3.890818	525	1083	668
3090	NM_001098202,NM_006497	HIC1	HIC ZBTB transcriptional repress	1.896385267	1.034441039	0.082868	0.173789	0.088577	0.35634914	0.61708849	0.36729312	11	28	14
54345	NM_018419	SOX18	SRY-box 18	-2.415817531	1.034441039	0.163921	0.068752	0.15604	0.35634914	0.15624221	0.36729312	11	6	14
51368	NM_001129884,NM_001243725	TEX264	testis expressed 264	1.138244685	1.034381948	24.112884	27.370271	23.283372	5.06064312	5.24220835	5.10797864	1271	1933	1617
967	NM_001257389,NM_001257390	CD63	CD63 molecule	-1.050487479	1.034367944	514.864738	491.507367	499.375741	8.99921415	8.92829713	9.04787021	20051	25503	25509
4170	NM_001197320,NM_021960,NM	MCL1	MCL1, BCL2 family apoptosis reg	-1.007095251	1.034365973	100.842274	98.783853	96.983126	8.62379985	8.61362559	8.6724254	15448	20495	19653
261726	NM_001031800,NM_152902	TIPRL	TOR signaling pathway regulator	-1.215981396	1.034324879	18.450921	14.486063	17.463721	5.7441029	5.46776786	5.79190132	2065	2269	2627
958	NM_001250,NM_001302753,NM	CD40	CD40 molecule	-1.073437935	1.034194249	57.818109	53.979615	56.729386	6.53126011	6.43016324	6.57925295	3592	4471	4569
84687	NM_032595	PPP1R9B	protein phosphatase 1 regulatory	-1.05042078	1.034188765	30.103617	28.692793	29.258837	6.90383733	6.83347566	6.95193967	4662	5930	5930
114790	NM_052902	STK11IP	serine/threonine kinase 11 intera	1.343361457	1.033977976	8.551781	11.502246	8.310232	4.96269999	5.37670602	5.00938866	1185	2127	1507
84816	NM_001318746,NM_032730	RTN4IP1	reticulon 4 interacting protein 1	1.059081493	1.03394094	2.391773	2.539457	2.365481	2.9541672	3.02658682	2.99621456	265	375	337
9817	NM_012289,NM_203500	KEAP1	kelch like ECH associated protein	1.154423791	1.033925735	23.319871	26.935244	22.690481	5.90176757	6.10571785	5.9491104	2308	3560	2935
100132341	NR_024034	CLUHP3	clustered mitochondria homolog	2.100456679	1.033904097	3.736794	7.864573	3.62593	2.90104285	3.86712977	2.94281592	254	713	323
84969	NM_001098796,NM_001098797	TOX2	TOX high mobility group box fam	1.544566278	1.033709154	1.433777	2.282379	1.438267	2.19091668	2.70251092	2.22843599	140	289	178
6147	NM_000984	RPL23A	ribosomal protein L23a	-1.020388414	1.033703108	657.270752	644.867676	638.484924	9.28020558	9.25113433	9.32795203	24371	31912	30985
10575	NM_001256721,NM_006430	CCT4	chaperonin containing TCP1 sub	1.599930041	1.033629525	99.94025	160.09403	97.516788	7.84187815	8.51753353	7.88939331	8968	19171	11401
51085	NM_032951,NM_032952,NM_03	MLXIPL	MLX interacting protein like	-2.801305178	1.033566728	2.926956	1.034828	2.83808	3.36414578	2.11004283	3.4072329	365	174	464
10776	NM_001306191,NM_001306195	ARPP19	cAMP regulated phosphoprotein	-9.171520245	1.033186376	24.790659	2.702164	24.181361	7.06125395	3.94954414	7.10800844	5204	758	6613
55503	NM_018646	TRPV6	transient receptor potential catior	-1.110552914	1.033096933	1.194073	1.075734	1.154509	2.13345087	2.01797632	2.16988427	133	160	169
85365	NM_033087,NR_024532	ALG2	ALG2, alpha-1,3/1,6-mannosyltra	1.267691491	1.033061149	27.652881	35.235108	26.576731	6.29055972	6.628877	6.33689711	3034	5139	3855
8558	NM_001098533,NM_001160367	CDK10	cyclin dependent kinase 10	1.067767516	1.032993359	39.564906	42.419704	38.216297	6.08831031	6.18156519	6.13446583	2632	3755	3344
5601	NM_001135044,NM_001308244	MAPK9	mitogen-activated protein kinase	-1.118693413	1.032945729	15.960083	14.239418	15.432227	5.98302523	5.82390789	6.02906409	2444	2919	3105
5256	NM_000292	PHKA2	phosphorylase kinase regulatory	1.456111644	1.032883427	3.433953	5.003147	3.330244	4.23299104	4.750944	4.27723085	699	1360	888
9772	NM_001321148,NM_001321149	TMEM94	transmembrane protein 94	-1.114426069	1.032865111	20.218943	18.124687	19.568865	6.7875738	6.63276234	6.83381125	4298	5153	5460
34	NM_000016,NM_001127328,NM	ACADM	acyl-CoA dehydrogenase, C-4 to	1.186317186	1.032814741	8.959144	10.649468	8.726473	4.57884974	4.81585323	4.62351744	899	1425	1142
3094	NM_005340,NR_024610,NR_02	HINT1	histidine triad nucleotide binding	-1.103704147	1.032769078	258.306869	234.121737	253.810251	7.50454218	7.36301011	7.55080851	7090	8583	9006
57060	NM_001174100,NM_020418,NM	PCBP4	poly(rC) binding protein 4	-1.102920474	1.032619192	8.462346	7.709673	8.106468	4.12971807	3.99682365	4.17342809	648	785	823
23673	NM_177424	STX12	syntaxin 12	-1.055719238	1.032614867	9.539068	9.039068	9.249599	4.88648104	4.81096259	4.93124635	1122	1420	1425
4938	NM_001032409,NM_001320151	OAS1	2'-5'-oligoadenylate synthetase 1	-1.600329112	1.032492614	46.496382	29.025315	45.054711	6.37356266	5.70557366	6.41914802	3216	2685	4084
98	NM_001320586,NM_001320587	ACYP2	acylphosphatase 2	1.619277341	1.032426357	3.993363	6.486009	3.837539	2.53954085	3.1370039	2.57778742	189	409	240
79585	NM_001201472,NM_001201473	CORO7	coronin 7	-1.003549974	1.032381344	5.856953	5.881202	5.688916	4.51760029	4.5127108	4.56160497	860	1145	1092
314	NM_001158,NM_009590	AOC2	amine oxidase, copper containing	1.926246794	1.032179437	2.526104	4.847641	2.443706	2.91084846	3.76158491	2.95056637	256	659	325
55367	NM_145886,NM_145887	PIDD1	p53-induced death domain protei	-1.01865532	1.03214782	12.22784	12.003141	11.854445	5.19511448	5.16918099	5.23954048	1399	1835	1776
26995	NM_001329861,NM_001329862	TRUB2	TruB pseudouridine synthase fan	1.195924952	1.031992959	19.433852	23.445008	19.109539	5.14302819	5.39446891	5.18719863	1348	2154	1711
11191	NR_023917	PTENP1	phosphatase and tensin homolog	-1.679361377	1.031903368	0.515576	0.307563	0.501908	1.57857476	1.12560759	1.60891398	78	62	99
8541	NM_003660,NR_103842	PPFIA3	PTPRF interacting protein alpha	-1.501845337	1.031850521	1.953554	1.295469	1.886148	3.32067256	2.8044511	3.36145532	353	314	448
85437	NM_033114	ZCRB1	zinc finger CCHC-type and RNA	-1.28595107	1.031825287	25.465328	19.823164	24.691372	5.5459881	5.19193145	5.59023678	1795	1865	2278
9220	NM_004740	TIAF1	TGFB1-induced anti-apoptotic fa	-1.173192682	1.031668193	7.118163	6.076596	6.906064	3.95355025	3.73910482	3.99567625	569	648	722
100130899	NR_039988	LOC100130899	uncharacterized LOC100130899	2.155449267	1.031574911	0.570691	1.235071	0.553767	1.75224037	2.61093762	1.78395874	93	268	118
51116	NM_016034,NR_051967,NR_05	MRPS2	mitochondrial ribosomal protein S	1.22308821	1.031571475	47.665023	58.391483	46.564134	6.18844858	6.47537422	6.23268854	2824	4615	3583
642946	NR_027285,NR_027286	FLVCR1-AS1	FLVCR1 antisense RNA 1 (head	-1.193418267	1.031456171	3.770746	3.111594	3.589156	2.14180175	1.94842076	2.1765086	134	150	170
10951	NM_001127228,NM_006807	CBX1	chromobox 1	-1.08422579	1.031436851	69.125872	63.831817	66.741792	7.2476167	7.13174867	7.29198365	5927	7304	7519
162427	NM_178126,NR_026697	RETREG3	reticulophagy regulator family me	-1.040198164	1.031324627	17.573072	16.91116	17.069109	6.04833571	5.99235095	6.09217385	2559	3287	3246
730051	NM_001144989	ZNF814	zinc finger protein 814	-1.491906586	1.031323107	2.310492	1.54853	2.237931	3.9199564	3.38881456	3.96156336	555	497	704
5245	NM_001281496,NM_001281497	PHB	prohibitin	-1.074493256	1.03125475	110.012112	102.396074	106.830253	7.64058685	7.53746743	7.68476911	7795	9693	9887
284233	NR_026756	CYP4F35P	cytochrome P450 family 4 subfar	1.277452819	1.031143931	0.7455	0.95155	0.720394	1.62696745	1.87534115	1.65707652	82	140	104
10612	NM_001248006,NM_001248007	TRIM3	tripartite motif containing 3	-1.1772863	1.031119612	5.871335	4.866009	5.612641	4.12340669	3.90250283	4.16512515	645	732	818
4591	NM_001005207,NM_001320987	TRIM37	tripartite motif containing 37	-1.155973645	1.031115265	15.511425	13.383502	15.1293	6.04555676	5.83984166	6.08910502	2554	2952	3239
94122	NM_001163334,NM_001163335	SYTL5	synaptotagmin like 5	3.425514877	1.031101467	0.2195	0.766333	0.212283	1.03161781	2.19658485	1.05442063	41	188	52
59307	NM_001135053,NM_001135054	SIGIRR	single Ig and TIR domain contain	-1.32964176	1.031021093	32.540305	24.394604	31.485076	5.73998338	5.3377927	5.78324714	2059	2069	2611
747	NM_006133	DAGLA	diacylglycerol lipase alpha	-2.257278518	1.030855347	7.934242	3.517356	7.68595	5.50773204	4.37236795	5.55062723	1747	1034	2215
30844	NM_139265	EHD4	EH domain containing 4	-1.091892154	1.030813996	15.966639	14.640185	15.463845	5.52299658	5.39903863	5.56584509	1766	2161	2239
4909	NM_006179	NTF4	neurotrophin 4	-1.845984456	1.030801563	2.909661	1.581744	2.82394	1.94589385	1.34717796	1.97845985	112	81	142
10221	NM_001282985,NM_025195	TRIB1	tribbles pseudokinase 1	-1.255250241	1.030583804	22.59985	17.554482	20.460725	6.24283552	5.91970164	6.28573352	2934	3123	3719
114904	NM_031910,NM_182486	C1QTNF6	C1q and TNF related 6	1.000580772	1.030441911	13.862347	14.450804	14.516852	5.28927297	5.29008925	5.33144894	1496	2000	1896
221491	NM_001008703,NM_001008704	SMIM29	small integral membrane protein	-1.951459833	1.03043507	9.209426	4.92655	9.292327	3.39591204	2.55587612	3.43512339	374	256	474
7918	NM_001199237,NM_001199238	GPANK1	G-patch domain and ankyrin repe	-1.029101299	1.030373805	3.944272	3.84825	3.83388	3.54502861	3.50722624	3.58455875	419	544	531
64283	NM_001080479,NM_001177693	ARHGEF28	Rho guanine nucleotide exchang	-1.099380282	1.030279106	13.593152	12.655508	13.097061	6.39293236	6.25794232	6.43546439	3260	3962	4131
51324	NM_001127889,NM_001127890	SPG21	SPG21, maspardin	1.118038851	1.03017085	30.198193	33.922998	29.423116	5.77597182	5.93416733	5.81808634	2112	3155	2676
7773	NM_006300	ZNF230	zinc finger protein 230	-1.086149542	1.030162844	2.225324	2.053151	2.156057	3.31698997	3.21015128	3.35562963	352	433	446
6050	NM_002939,NM_203383,NM_20	RNH1	ribonuclease/angiogenin inhibitor	-1.187789549	1.030064029	42.698935	36.126049	41.495872	6.26067799	6.01591823	6.30286431	2971	3342	3764
7695	NM_001348013,NM_001348014	ZNF136	zinc finger protein 136	-1.314666082	1.02998244	3.115181	2.323094	3.023386	3.58232086	3.22493445	3.62144026	431	438	546
175	NM_000027,NM_001171988,NR	AGA	aspartylglucosaminidase	1.059962913	1.02996984	12.470506	13.201571	12.041763	4.72217607	4.80310291	4.76319139	997	1412	1263
8835	NM_001270467,NM_001270468	SOCS2	suppressor of cytokine signaling	-1.25662765	1.029852734	2.921052	2.336526	2.783726	3.04601354	2.76048215	3.08339379	285	303	361
131616	NM_144638	TMEM42	transmembrane protein 42	-1.046890529	1.029752534	13.655643	13.069026	13.231959	3.81681978	3.75548863	3.85616495	514	656	651
3835	NM_001256269,NM_001256270	KIF22	kinesin family member 22	1.147723801	1.029636873	67.759873	77.799253	65.513719	7.13839801	7.33585851	7.1802397	5492	8422	6955
51136	NM_016125	RNFT1	ring finger protein, transmembrar	1.026174142	1.029547992	4.572137	4.702465	4.426209	3.32067256	3.35427048	3.35854542	353	484	447
94134	NM_001270695,NM_001270696	ARHGAP12	Rho GTPase activating protein 1	-1.01200989	1.029486425	16.508893	16.349207	15.9096	6.34988034	6.33286878	6.39129989	3163	4176	4005
64927	NM_001288615,NM_001288616	TTC23	tetratricopeptide repeat domain 2	1.71945846	1.029457608	3.661414	6.842836	3.688912	3.88556162	4.62619813	3.92465818	541	1243	685
9825	NM_001135773,NM_006038	SPATA2	spermatogenesis associated 2	-1.041194611	1.029342637	6.60069	6.474101	6.510648	4.78750079	4.73140553	4.82773844	1045	1341	1323
28232	NM_001145044,NM_013272,NR	SLCO3A1	solute carrier organic anion trans	-2.240649811	1.029325408	21.960457	9.863068	22.065165	6.62172853	5.47582432	6.66301144	3827	2282	4845
285381	NM_001047434,NM_206831	DPH3	diphthamide biosynthesis 3	-1.077500157	1.02931587	3.244132	3.004597	3.121958	3.77984319	3.68025029	3.81854297	500	620	633
221393	NM_001347855,NM_153838	ADGRF4	adhesion G protein-coupled rece	-1.141715884	1.029276207	2.320009	2.031028	2.249484	3.03260384	2.86610484	3.06922486	282	330	357
3306	NM_021979	HSPA2	heat shock protein family A (Hsp	1.405105389	1.029164562	2.232327	3.145859	2.158929	2.81488929	3.24537964	2.85055786	237	445	300
399665	NM_001035254,NM_203305	FAM102A	family with sequence similarity 10	-1.601411224	1.029164393	11.530611	7.142238	11.00573	5.47588426	4.81585323	5.51644141	1708	1425	2162
65109	NM_023010,NM_080632	UPF3B	UPF3 regulator of nonsense tran	1.001388223	1.029107638	21.295887	21.396221	20.640322	5.65429253	5.65625432	5.69487827	1938	2593	2453

57332	NM_020649	CBX8	chromobox 8	1.137409685	1.028936612	5.984325	6.816743	5.788617	3.32434579	3.49264009	3.36145532	354	538	448
51110	NM_016027	LACTB2	lactamase beta 2	-1.502845223	1.028917256	15.469631	10.307449	14.959051	4.60927912	4.05092325	4.64874869	919	817	1163
6911	NM_004608	TBX6	T-box 6	-1.929383835	1.028870263	10.331454	5.354951	9.983808	4.25817433	3.37827344	4.29712434	712	493	901
7770	NM_001289166,NM_001289167	ZNF227	zinc finger protein 227	-1.4018123	1.028826536	5.882271	4.200359	5.760492	4.19938147	3.74321825	4.23818553	682	650	863
3600	NM_000585,NM_172175,NR_03	IL15	interleukin 15	-2.68012881	1.028687286	3.094039	1.148631	3.381311	3.01906833	1.86782539	3.0549154	279	139	353
143888	NM_153705	KDELC2	KDEL motif containing 2	-1.011238232	1.028539896	13.915057	13.78136	13.454785	5.893137	5.87728612	5.93306293	2294	3031	2902
5599	NM_001278547,NM_001278548	MAPK8	mitogen-activated protein kinase	-1.267424899	1.028426707	7.351303	5.925006	6.978236	5.43654533	5.10350222	5.47606585	1661	1751	2101
91433	NM_001017919,NM_033544	RCCD1	RCC1 domain containing 1	-1.672719993	1.028243534	18.279693	11.005993	17.733513	5.63223546	4.90942294	5.67162056	1908	1524	2413
9588	NM_004905	PRDX6	peroxiredoxin 6	-1.223687954	1.028195924	272.787903	223.175064	263.572571	8.79621446	8.50570251	8.83624091	17414	19014	22022
8975	NM_003940	USP13	ubiquitin specific peptidase 13 (is	1.210048239	1.027973031	3.216183	3.897216	3.107034	4.69263929	4.95801353	4.73092712	976	1578	1234
54901	NM_017774	CDKAL1	CDK5 regulatory subunit associa	-1.153747487	1.027849235	3.231486	2.809763	3.124953	3.50026652	3.31335619	3.53644652	405	469	512
22827	NM_001136033,NM_001271096	PUF60	poly(U) binding splicing factor 60	1.137645753	1.027824252	72.754952	82.884081	71.063462	7.09147705	7.27625145	7.13078507	5315	8079	6719
286053	NM_001349485,NM_001349486	NSMCE2	NSE2/MMS21 homolog, SMC5-S	-1.153999661	1.027783736	39.168164	34.007014	38.636192	5.56939302	5.3674088	5.60811055	1825	2113	2307
8209	NM_001320383,NM_001320384	C21orf33	chromosome 21 open reading fra	1.220243932	1.027687834	31.213302	37.82633	29.228638	5.62182561	5.90371192	5.66044042	1894	3088	2394
51490	NM_016390	SPOUT1	SPOUT domain containing methy	-1.223206699	1.027582895	18.710297	15.312962	18.069218	6.30550396	6.01889027	6.34427044	3066	3349	3875
1979	NM_004096	EIF4EBP2	eukaryotic translation initiation fa	-1.353957099	1.027552872	6.84647	5.061733	6.611152	5.67817204	5.25090169	5.71663112	1971	1945	2491
7170	NM_001043351,NM_001043352	TPM3	tropomyosin 3	-1.00797168	1.02750664	84.633689	84.386972	83.002104	8.09749203	8.0860788	8.13649914	10714	14202	13540
64777	NM_001288794,NM_001288795	RMND5B	required for meiotic nuclear divisi	1.010943582	1.027433634	10.67057	10.715508	10.300843	4.42483953	4.43981671	4.46209528	804	1086	1016
400931	NR_027033,NR_110479	MIRLET7BHG	MIRLET7B host gene	1.410603179	1.027280138	0.822799	1.168414	0.790484	2.25390578	2.65962865	2.28470378	148	279	187
54965	NM_001166304,NM_017861	PIGX	phosphatidylinositol glycan ancho	1.338361978	1.027266244	15.73466	21.159268	15.254169	5.5786497	5.99148667	5.61666065	1837	3285	2321
5611	NM_006260	DNAJC3	DnaJ heat shock protein family (H	-1.342121594	1.027163884	10.428357	7.776347	10.066785	5.85237594	5.43635529	5.89038369	2229	2219	2816
3344	NM_002158	FOXN2	forkhead box N2	-1.312143517	1.027083386	5.508508	4.204257	5.317544	4.92206677	4.54487907	4.9593683	1151	1172	1454
5493	NM_002705	PPL	periplakin	-1.032496375	1.02700854	61.448418	59.582691	59.305077	8.54777426	8.50176247	8.58612134	14653	18962	18509
282969	NM_001098483,NM_001301827	FUOM	fucose mutarotase	-1.450034292	1.026985129	3.958143	2.641315	3.906529	1.96484452	1.58576189	1.99354404	114	105	144
9080	NM_020982	CLDN9	claudin 9	1.511226161	1.026948563	9.254685	14.01433	8.934527	4.28481203	4.85534316	4.32123795	726	1466	917
56946	NM_001300942,NM_001300943	EMSY	EMSY, BRCA2 interacting transc	1.213624939	1.026938137	4.586593	5.574355	4.41815	4.98607799	5.25738748	5.02323609	1205	1954	1522
9530	NM_001204878,NM_004874	BAG4	BCL2 associated athanogene 4	1.000458238	1.026911212	5.191918	5.227412	5.018696	4.55247036	4.55310322	4.58917433	882	1179	1114
7738	NM_001318891,NM_001318892	ZNF184	zinc finger protein 184	-1.359549781	1.026879281	2.703498	2.024332	2.614308	3.29469475	2.90334193	3.3291189	346	340	437
26777	NR_002911	SNORA71A	small nucleolar RNA, H/ACA box	1.650768677	1.026853218	3.600038	5.950241	3.421725	0.56944351	0.84852988	0.58209675	19	42	24
51634	NM_016024	RBMX2	RNA binding motif protein, X-link	-1.031562844	1.026765235	22.135359	21.483141	21.360405	5.36166293	5.31793524	5.39885696	1575	2040	1989
92106	NM_001330670,NM_001330671	OXNAD1	oxidoreductase NAD binding dom	1.045667679	1.026394193	2.049329	2.306534	2.163759	3.03260384	3.08932884	3.06566078	282	394	356
144100	NM_001329630,NM_001329631	PLEKHA7	pleckstrin homology domain cont	-2.412357218	1.026295867	29.489949	12.223989	28.790613	7.21731642	5.96046406	7.2545154	5803	3214	7325
1635	NM_001012732,NM_001351743	DCTD	dCMP deaminase	1.046610685	1.026109307	50.090139	53.64822	49.627155	6.73023514	6.79535639	6.76707475	4129	5774	5211
594	NM_000056,NM_001318975,NM	BCKDHB	branched chain keto acid dehydr	1.195751152	1.025950553	5.026782	5.869248	4.238839	3.85032677	4.09182073	3.88476293	527	842	665
29058	NM_001009923,NM_001009924	TMEM230	transmembrane protein 230	1.187014869	1.025688647	75.991261	90.191701	73.608078	6.82143402	7.06676638	6.85770828	4401	6980	5552
10309	NM_021147,NR_125346,NR_12	CCNO	cyclin O	-2.663426262	1.025576569	11.689976	4.650509	11.357464	4.15055879	2.86610484	4.18497252	658	330	830
81556	NM_001136043,NM_001207058	INTS14	integrator complex subunit 14	-1.000819711	1.025509116	20.415948	20.192518	19.181773	5.67529852	5.67413949	5.71093841	1967	2626	2481
28989	NM_001286796,NM_001286797	NTMT1	N-terminal Xaa-Pro-Lys N-methy	1.165144639	1.025508868	37.667108	44.039843	35.810556	5.81173868	6.02861267	5.84744145	2166	3372	2732
27	NM_001136000,NM_001136001	ABL2	ABL proto-oncogene 2, non-rece	-1.392984132	1.025349471	12.228947	9.163412	12.076371	7.14334521	6.66916033	7.17920932	5511	5286	6950
9659	NM_001002810,NM_001002811	PDE4DIP	phosphodiesterase 4D interacting	-1.35735053	1.025272523	6.196736	4.662449	5.916861	5.48903517	5.05964438	5.52425285	1724	1697	2174
2628	NM_001321015,NM_001482	GATM	glycine amidinotransferase	-1.098704812	1.025221987	9.68715	8.812499	9.337132	4.66683165	4.53660777	4.70137402	958	1165	1208
80174	NM_025104,NM_145663,NR_03	DBF4B	DBF4 zinc finger B	1.03670422	1.025190004	20.962281	21.684121	20.025772	6.10121435	6.15247651	6.1365907	2656	3679	3349
89970	NM_001305163,NM_001305164	RSPRY1	ring finger and SPRY domain cor	1.018344122	1.025172624	11.66929	11.519451	11.475163	5.30608931	5.33165924	5.34106306	1514	2060	1909
57559	NM_020799	STAMBPL1	STAM binding protein like 1	-1.390713863	1.025145302	6.028211	4.339912	5.807724	3.7336304	3.29945612	3.76680378	483	464	609
11159	NM_001306158,NM_001306159	RABL2A	RAB, member of RAS oncogene	2.262728666	1.025137524	2.874143	6.804665	2.737858	2.64904502	3.69306567	2.67922768	207	626	261
79041	NM_024074	TMEM38A	transmembrane protein 38A	1.647720585	1.02511034	1.097937	1.832789	1.070019	1.46336426	1.96260273	1.4863075	69	152	87
10521	NM_001098504,NM_006386	DDX17	DEAD-box helicase 17	1.522462454	1.025108325	243.798237	371.593766	234.681397	10.15271063	10.75868348	10.18845605	44652	90831	56298
3482	NM_000876	IGF2R	insulin like growth factor 2 recept	1.306763024	1.025078164	5.484999	7.177728	5.285247	6.24816313	6.62971046	6.28343397	2945	5142	3713
9702	NM_001243776,NM_001243777	CEP57	centrosomal protein 57	-1.475646878	1.024881564	21.762553	14.724529	20.985536	6.08885029	5.53752208	6.12379429	2633	2384	3319
65979	NM_001048183,NM_001350158	PHACTR4	phosphatase and actin regulator	-1.188243869	1.024709864	8.407094	7.111873	8.103934	5.70518879	5.46153982	5.7397392	2009	2259	2532
54927	NM_001317177,NM_001317178	CHCHD3	coiled-coil-helix-coiled-coil-helix c	-1.17233466	1.024690285	20.486476	17.513876	19.71283	5.10295837	4.88077062	5.13713733	1310	1493	1651
57396	NM_020666	CLK4	CDC like kinase 4	-1.013953294	1.024655478	12.700832	12.547695	12.230948	5.00107303	4.98170859	5.03513062	1218	1605	1535
57232	NM_001037735,NM_001190255	ZNF630	zinc finger protein 630	-1.18930973	1.024647965	1.340268	1.124243	1.296778	2.23841445	2.04488597	2.26619062	146	164	184
275	NM_000481,NM_001164710,NM	AMT	aminomethyltransferase	-1.219556228	1.024570743	6.189067	5.148105	5.988259	3.75828027	3.49508138	3.79074812	492	539	620
2189	NM_004629	FANCG	Fanconi anemia complementation	-1.005429654	1.024569025	12.717751	12.666791	12.253098	5.07051892	5.06293923	5.10450913	1280	1701	1613
5331	NM_000932,NM_001184883,NM	PLCB3	phospholipase C beta 3	1.195054918	1.02454175	15.548493	18.63741	14.030243	6.3381189	6.59229025	6.37267168	3137	5009	3953
6827	NM_003168,NR_073470	SUPT4H1	SPT4 homolog, DSIF elongation	1.027010011	1.0243314	39.915682	41.029269	38.418469	5.91644377	5.95426739	5.95056045	2332	3200	2938
4615	NM_001172566,NM_001172567	MYD88	myeloid differentiation primary re	-1.165476988	1.024303296	20.397105	17.61779	19.910351	5.8421633	5.62538694	5.87621116	2213	2537	2788
2669	NM_005261,NM_181702	GEM	GTP binding protein overexpress	1.619700896	1.024221201	2.762037	4.566396	2.699903	2.78321062	3.39667017	2.81278744	231	500	291
152815	NM_001317791,NM_144721,NR	THAP6	THAP domain containing 6	1.150933646	1.024205813	5.276047	5.41083	4.386052	3.75011035	3.9388238	3.782087	489	752	616
51162	NM_016215,NM_201446,NR_04	EGFL7	EGF like domain multiple 7	1.078368176	1.02418731	38.373928	41.420101	36.930454	5.63371647	5.74045883	5.66751164	1910	2752	2406
154	NM_000024	ADRB2	adrenoceptor beta 2	1.715739065	1.024186774	6.604263	11.353508	6.356005	3.82202563	4.55778177	3.85410038	516	1183	650
54583	NM_022051	EGLN1	egl-9 family hypoxia inducible fac	1.595157537	1.024147749	4.683172	7.479848	4.506901	5.06064312	5.71816702	5.09405028	1271	2709	1601
114885	NM_022776	OSBPL11	oxysterol binding protein like 11	1.256086789	1.024077333	3.537322	4.454027	3.407929	4.0871111	4.39868427	4.11944434	628	1054	791
56993	NM_020243	TOMM22	translocase of outer mitochondria	1.361465742	1.024064554	56.209957	76.603851	54.09269	6.25683881	6.69699562	6.29070334	2963	5390	3732
1528	NM_001190807,NM_001914,NM	CYB5A	cytochrome b5 type A	-1.108845792	1.023826537	66.761023	60.228928	64.273696	5.79264085	5.6464045	5.82600807	2137	2575	2691
4793	NM_001243116,NM_002503,NR	NFKBIB	NFKB inhibitor beta	-1.113191603	1.023782939	25.881641	21.767913	21.956925	5.3643416	5.2135877	5.39743997	1578	1894	1987
132660	NM_001115007,NM_001115008	LIN54	lin-54 DREAM MuvB core comple	1.088924074	1.023771921	3.919003	4.532753	3.863517	4.57884974	4.69682839	4.61134568	899	1308	1132
80714	NM_025245,NR_038198	PBX4	PBX homeobox 4	-1.488177163	1.02371163	11.246681	7.580567	10.817042	4.26200996	3.72461474	4.2940816	714	641	899
54414	NM_001199922,NM_170601	SIAE	sialic acid acetylesterase	-2.052491764	1.023702585	9.891171	4.615509	9.522222	4.85888706	3.87277297	4.89153539	1100	716	1385
29890	NM_013286	RBM15B	RNA binding motif protein 15B	1.227243732	1.023514554	8.004725	9.836742	7.700651	5.72129859	6.01166181	5.75420361	2032	3332	2558
29928	NM_013337	TIMM22	translocase of inner mitochondria	1.167993444	1.023428963	5.026513	5.87928	4.835718	3.38540081	3.58949269	3.4156568	371	579	467
57798	NM_021167,NR_052016	GATAD1	GATA zinc finger domain contain	-1.798159831	1.023427111	12.906921	7.191258	12.349689	5.87007592	5.04305632	5.90292115	2257	1677	2841
1373	NM_001122633,NM_001122634	CPS1	carbamoyl-phosphate synthase 1	1.496198717	1.023109547	5.426422	8.131755	5.221717	4.97560474	5.54166104	5.00753225	1196	2391	1505
1534	NM_001017916,NM_001017917	CYB561	cytochrome b561	-1.122208004	1.023102334	55.042532	49.348813	53.238346	7.36595076	7.20067615	7.39870427	6437	7664	8100
642587	NR_145433,NR_145434,NR_14	MIR205HG	MIR205 host gene	2.424620765	1.023078883	62.038125	150.248085	60.021109	5.86756061	7.13076777	5.89992208	2253	7299	2835
65250	NM_023073	C5orf42	chromosome 5 open reading fran	1.075274169	1.02286413	0.940395	1.012932	0.90483	3.49375703	3.58949269	3.52351414	403	579	507
100507437	NR_125384,NR_125385	LOC100507437	uncharacterized LOC100507437	2.096659293	1.022788078	0.53206	1.065943	0.482841	0.83964067	1.41062362	0.85412573	31	87	39
892	NM_001013399,NM_005190	CCNC	cyclin C	1.0468183	1.022634459	24.059288	25.210333	23.121099	5.78266256	5.84750424	5.8143746	2122	2968	2669
54554	NM_019069	WDR5B	WD repeat domain 5B	1.254084408	1.022535142	3.466865	4.356189	3.334053	3.9199564	4.22720295	3.95001076	555	930	698
84726	NM_013318	PRRC2B	proline rich coiled-coil 2B	1.123682337	1.022444931	29.170813	32.815742	28.027821	8.29971037	8.46744197	8.33163329	12332	18515	15508
58509	NM_001080543,NM_021231	CACTIN	cactin, spliceosome C complex s	1.186872327	1.022379836	14.245793	17.018063	13.911791	5.60833019	5.85084386	5.63961583	1876	2975	2359
3566	NM_000418,NM_001257406,NM	IL4R	interleukin 4 receptor	-2.081903157	1.021975344	27.273838	13.265998	26.298807	6.6269432	5.58470447	6.65799057	3841	2465	4828
64743	NM_001166426,NM_001347217	WDR13	WD repeat domain 13	1.00634015	1.021804463	10.157806	10.617771	10.724071	4.5618359	4.57057009	4.59165471	888	1194	1116
51574	NM_001267039,NM_015454,NM	LARP7	La ribonucleoprotein domain fam	-1.030616289	1.021757077	25.950897	25.889525	25.098385	5.85935542	5.81660668	5.88987992	2240	2904	2815
317671	NM_001131065,NM_001131066	RFESD	Rieske Fe-S domain containing	-1.063351893	1.021494599	0.917884	0.983943	1.128668	1.57857476	1.52015496	1.59908548	78	98	98
51573	NM_001324066,NM_001324067	GDE1	glycerophosphodiester phosphod	1.509626467	1.021492624	40.178693	60.743134	38.574843	6.85737282	7.44736949	6.88779054	4513	9103	5670
4650	NM_001130065,NM_004145	MYO9B	myosin IXB	-1.018064166	1.021404328	12.484027	12.268886	11.97475	6.54745809	6.52190742	6.57768983	3633	4768	4564
3770	NM_013348	KCNJ14	potassium voltage-gated channel	-1.569356008	1.021346608	3.326331	2.123622	3.195707	3.35699061	2.78463118	3.38452567	363	309	456
375616	NM_001135914,NM_199349	KCP	kielin/chordin-like protein	-1.68714324	1.021317157	4.611216	2.716621	4.407184	4.43846184	3.72876963	4.46750731	812	643	1020
84295	NM_001015877,NM_032335,NM	PHF6	PHD finger protein 6	1.108418218	1.021247455	12.709977	14.006688	12.380487	5.82281754	5.96883147	5.85262116	2183	3233	2742
51073	NM_015956,NM_146387,NM_14	MRPL4	mitochondrial ribosomal protein L	1.196751037	1.021095122	33.558191	41.088868	33.643027	5.7268601	5.98150995	5.75641609	2040	3262	2562
170622	NM_001287392,NM_001287393	COMMD6	COMM domain containing 6	1.157512521	1.020903827	10.76887	12.519616	10.398287	4.25047234	4.451177	4.27877089	708	1095	889
3150	NM_004965	HMGN1	high mobility group nucleosome b	-1.06818371	1.020808352	222.257263	208.304688	213.212585	8.14564895	8.05083553	8.17525744	11079	13858	13910
23552	NM_001039803,NM_001170639	CDK20	cyclin dependent kinase 20	1.431869306	1.020703207	2.50902	3.669444	2.571668	2.78321062	3.23665287	2.80852903	231	442	290
200539	NM_144994	ANKRD23	ankyrin repeat domain 23	1.253753144	1.020703207	2.330534	2.931753	2.239237	2.78321062	3.06652959	2.80852903	231	387	290
116986	NM_001122772,NM_014770	AGAP2	ArfGAP with GTPase domain, an	-2.126008462	1.020613782	0.49402	0.194889	0.405572	1.76310734	1.08728891	1.78395874	94	59	118
5026	NM_001204519,NM_001204520	P2RX5	purinergic receptor P2X 5	1.208653194	1.02061071	8.061275	9.790016	7.763206	4.2524017	4.5127108	4.2803093	709	1145	890
1200	NM_000391	TPP1	tripeptidyl peptidase 1	-1.295021312	1.020601159	41.763954	32.288708	40.057934	7.17674682	6.80670178	7.20596529	5641	5820	7081

84513	NM_001102559,NM_001102560	PLPP5	phospholipid phosphatase 5	-1.190721043	1.020597405	18.755216	15.098203	14.741036	4.58192172	4.34149855	4.61012283	901	1011	1131
51027	NM_001321025,NM_001321026	BOLA1	bolA family member 1	-1.177131358	1.020597	13.874023	11.874545	12.923715	3.70855202	3.49264009	3.73574288	474	538	595
85007	NM_001278346,NM_153373,NR	PHYKPL	5-phosphohydroxy-L-lysine phos	1.196772734	1.020575193	11.107632	11.811469	10.173947	4.51438809	4.76314913	4.542501	858	1372	1077
7314	NM_001281716,NM_001281717	UBB	ubiquitin B	1.158935939	1.020508782	344.458272	399.302806	330.170124	8.67764974	8.88996862	8.70686773	16037	24833	20129
353274	NM_181489	ZNF445	zinc finger protein 445	1.289708537	1.020493626	2.480293	3.20288	2.379265	4.68694402	5.04138697	4.71508918	972	1675	1220
285315	NM_001308229,NM_173657	C3orf33	chromosome 3 open reading fran	1.176063211	1.020455997	5.614473	6.504935	5.383469	3.45742019	3.67164303	3.48400652	392	616	492
2059	NM_004447	EPS8	epidermal growth factor receptor	1.17252824	1.020280756	22.979021	26.976627	22.032791	6.52887435	6.75620254	6.55753084	3586	5618	4500
23443	NM_001271684,NM_001271685	SLC35A3	solute carrier family 35 member A	-1.195281575	1.020207049	6.369548	5.254974	6.052757	5.20511061	4.95535656	5.23319995	1409	1575	1768
4204	NM_001110792,NM_001316337	MECP2	methyl-CpG binding protein 2	1.232149027	1.020096534	8.959439	10.250675	9.819391	6.11718378	6.4144501	6.14548112	2686	4422	3370
83642	NM_031454	SELENOO	selenoprotein O	1.158394875	1.019995291	13.71376	15.907858	13.149861	4.99647575	5.20242678	5.02415454	1214	1879	1523
8382	NM_003551	NME5	NME/NM23 family member 5	2.691073648	1.019968611	1.158841	3.157288	1.109778	1.26358189	2.255369	1.28032816	55	198	69
51657	NM_001317785,NM_001317786	STYXL1	serine/threonine/tyrosine interact	-1.262651534	1.01979821	31.649982	24.374148	29.403676	5.13573004	4.80998247	5.16321914	1341	1419	1682
64718	NM_001037125,NM_001193388	UNKL	unkempt family like zinc finger	-1.156657316	1.019757797	14.133641	12.835404	14.309077	5.89745874	5.69127348	5.92521761	2301	2658	2886
124044	NM_152339	SPATA2L	spermatogenesis associated 2 lik	1.237563605	1.019714093	3.996189	4.958507	3.834964	3.33894587	3.61889232	3.36435937	358	592	449
23295	NM_001142289,NM_001142290	MGRN1	mahogunin ring finger 1	1.197198671	1.019644416	9.35616	12.155196	8.907487	5.37146049	5.62538694	5.39885696	1586	2537	1989
85360	NM_001300910,NM_033025	SYDE1	synapse defective Rho GTPase h	1.240805747	1.019616933	2.430864	3.015837	2.327809	3.14071816	3.41998355	3.16560381	307	509	385
54665	NM_018364,NR_130896	RSBN1	round spermatid basic protein 1	1.017349775	1.019536861	2.735952	2.769629	2.607349	4.22121804	4.24471769	4.24765323	693	942	869
23262	NM_001276277,NM_001281471	PPIP5K2	diphosphoinositol pentakisphospl	-1.274076863	1.019531168	6.041837	4.738549	5.798067	5.47340506	5.13280125	5.5006905	1705	1788	2138
404734	NM_020690	ANKHD1-EIF4EBP3	ANKHD1-EIF4EBP3 readthrough	1.013747033	1.019472682	14.512292	14.728711	13.904081	6.89460161	6.91413532	6.92219377	4632	6274	5808
7260	NM_001330530,NM_001330531	TSSC1	tumor suppressing subtransferab	1.259042541	1.0194219	16.054524	20.164587	15.373003	4.87275002	5.19493791	4.89956551	1111	1869	1393
348327	NM_001321981,NM_020880,NR	ZNF530	zinc finger protein 530	1.118493495	1.019381875	6.705823	6.822755	5.983143	4.71659644	4.8723445	4.74325038	993	1484	1245
8398	NM_001004426,NM_001199562	PLA2G6	phospholipase A2 group VI	-1.244970035	1.01928469	4.741068	4.172331	4.79489	4.05872616	3.76361129	4.08464905	615	660	771
11329	NM_001305102,NM_007271	STK38	serine/threonine kinase 38	-1.480808566	1.019208496	25.29613	17.10026	24.231585	6.49830814	5.93955479	6.52545742	3510	3167	4400
607	NM_004326	BCL9	B-cell CLL/lymphoma 9	1.356187369	1.019188454	3.27884	4.455962	3.142783	4.37789078	4.79915696	4.40400834	777	1408	974
92	NM_001278579,NM_001278580	ACVR2A	activin A receptor type 2A	1.976040154	1.019118774	2.159754	4.272634	2.067496	3.59149522	4.51391509	3.61657686	434	1146	544
80000	NM_001142966	GREB1L	growth regulation by estrogen in	-1.015476322	1.019113449	4.677363	4.609697	4.479599	5.44331753	5.42167258	5.47001198	1669	2196	2092
100506649	NR_038924	PXN-AS1	PXN antisense RNA 1	-1.065954748	1.018995404	2.565443	2.396564	2.451229	1.54118428	1.48128072	1.5590879	75	94	94
8914	NM_001330295,NM_003920,NR	TIMELESS	timeless circadian clock	-1.21002709	1.018932106	18.053834	14.921262	17.262641	6.52249295	6.25073686	6.5492601	3570	3942	4474
222161	NR_002186	DKFZP586I1420	uncharacterized protein DKFZp58	-1.431849191	1.01886062	4.547096	3.179656	4.357412	3.71975182	3.24827687	3.74468587	478	446	599
147372	NM_133459	CCBE1	collagen and calcium binding EG	-2.633004277	1.01885981	0.329405	0.122429	0.314622	1.59082596	0.81765451	1.60891398	79	40	99
81488	NM_001018102,NM_015532	POLR2M	RNA polymerase II subunit M	-1.289073086	1.018783308	7.992803	6.236797	7.648987	5.10935964	4.75502386	5.13543833	1316	1364	1649
340252	NM_001130022,NM_178558	ZNF680	zinc finger protein 680	-1.666841978	1.018749603	7.936739	4.7862	7.78344	4.6062649	3.90800961	4.63197698	917	735	1149
83461	NM_001297602,NM_001297603	CDCA3	cell division cycle associated 3	-1.065142182	1.018561074	36.648233	34.346595	35.095721	5.98128127	5.89171805	6.00739874	2441	3062	3058
10531	NM_001242307,NM_001242309	PITRM1	pitrilysin metallopeptidase 1	1.166150482	1.018538883	17.437385	20.039946	16.994818	5.94177327	6.16018847	5.96784829	2374	3699	2974
65080	NM_022915	MRPL44	mitochondrial ribosomal protein L	1.201526137	1.018509662	22.148088	26.640963	21.191196	5.27415876	5.53277726	5.29994267	1480	2376	1854
162968	NM_001207009,NM_198458	ZNF497	zinc finger protein 497	1.19094711	1.018488106	1.395801	1.66777	1.335123	2.52045395	2.73178769	2.54232458	186	296	233
64375	NM_001351089,NM_001351090	IKZF4	IKAROS family zinc finger 4	-1.16112348	1.018476137	3.285368	2.90347	3.385889	4.14848821	3.94597954	4.17342809	657	756	823
55033	NM_017946,NR_046478,NR_04	FKBP14	FK506 binding protein 14	-1.157634866	1.018344542	6.105034	5.321171	5.900647	4.98026891	4.77625587	5.00567345	1200	1385	1503
10048	NM_005493	RANBP9	RAN binding protein 9	-1.246140352	1.018226308	13.38638	10.749459	12.809285	5.37766084	5.06868717	5.40309959	1593	1708	1995
5118	NM_002593	PCOLCE	procollagen C-endopeptidase enl	-3.3946764	1.018042273	3.7659	1.105384	3.613052	2.7991369	1.4614436	2.82126674	234	92	293
6991	NM_174910,NR_138070	TCTE3	t-complex-associated-testis-expre	1.247175695	1.017949811	1.551013	1.931023	1.481605	2.04717061	2.29513766	2.06668624	123	205	154
51172	NM_016256	NAGPA	N-acetylglucosamine-1-phosphod	1.229084027	1.017552146	9.693295	11.933575	9.269038	4.45027676	4.73554094	4.47424393	819	1345	1025
55904	NM_018682,NM_182931	KMT2E	lysine methyltransferase 2E	-1.321809236	1.017490524	9.866669	7.471188	9.434707	6.06489791	5.66928366	6.08954382	2589	2617	3240
54788	NM_001002762,NM_017626	DNAJB12	DnaJ heat shock protein family (H	-1.128718767	1.017318483	20.011577	18.064893	18.774924	6.1914686	6.01931435	6.21590487	2830	3350	3541
55540	NM_018725	IL17RB	interleukin 17 receptor B	-1.118670038	1.017267784	2.694596	2.415383	2.583418	2.67229041	2.53704518	2.69315397	211	252	264
64428	NM_001304799,NM_022493	NARFL	nuclear prelamin A recognition fa	-1.082195187	1.017226406	16.254267	15.031182	15.452355	5.12839479	5.01781102	5.15233896	1334	1647	1669
284129	NM_001166347,NM_001166348	SLC26A11	solute carrier family 26 member 1	-1.90910438	1.017142192	2.168306	1.138339	2.065296	2.84588727	2.08433212	2.86703245	243	170	304
284403	NM_001083961,NM_173636	WDR62	WD repeat domain 62	-1.107438509	1.017104939	16.986755	15.356083	16.24285	6.3119937	6.16671138	6.3361577	3080	3716	3853
200576	NM_001178000,NM_015040,NM	PIKFYVE	phosphoinositide kinase, FYVE-ty	1.134019253	1.017045689	5.333586	6.111497	4.933146	5.6564798	5.83454993	5.6803862	1941	2941	2428
81611	NM_001136478,NM_001136479	ANP32E	acidic nuclear phosphoprotein 32	-1.049468829	1.016967342	40.244353	38.229157	38.440653	7.08282885	7.01369446	7.10692514	5283	6726	6608
9442	NM_001253881,NM_001253882	MED27	mediator complex subunit 27	1.286716237	1.016884423	20.082138	25.868596	19.142314	4.81386993	5.16612031	4.83717616	1065	1831	1332
55039	NM_017956	TRMT12	tRNA methyltransferase 12 homo	1.018254633	1.016835432	13.729128	13.998306	13.120143	4.93893692	4.96419416	4.96224642	1165	1585	1457
5605	NM_030662	MAP2K2	mitogen-activated protein kinase	1.084051085	1.01671554	68.630974	74.488144	65.581696	6.86810801	6.9835855	6.89182163	4547	6586	5686
255919	NM_001281789,NM_153261,NR	CNEP1R1	CTD nuclear envelope phosphata	-2.282259423	1.016495574	13.840856	5.919014	12.913343	4.86772434	3.73910482	4.89052847	1107	648	1384
284273	NM_001306093,NM_175907	ZADH2	zinc binding alcohol dehydrogena	-1.416046282	1.016470117	8.749914	6.200339	8.369876	6.06325022	5.57030594	6.08646939	2586	2440	3233
25782	NM_012414	RAB3GAP2	RAB3 GTPase activating non-cat	1.12817827	1.016463178	9.659024	10.908341	9.224268	6.12773269	6.29938812	6.15095739	2706	4079	3383
347862	NM_001318818,NM_001318820	GATD1	glutamine amidotransferase like c	1.311413068	1.016312814	13.498789	17.50436	12.83319	5.86188507	6.24712059	5.8848325	2244	3932	2805
2952	NM_000853,NM_001293807,NM	GSTT1	glutathione S-transferase theta 1	-1.303796409	1.016311366	20.374322	15.53447	19.527422	4.43166676	4.06906113	4.45393896	808	828	1010
84314	NM_001351278,NM_001351279	TMEM107	transmembrane protein 107	1.165109429	1.016306704	3.168068	3.730074	3.023404	2.94941774	3.14324318	2.96976228	264	411	330
7099	NM_003266,NM_138554,NM_13	TLR4	toll like receptor 4	8.908768173	1.016291885	0.37731	3.523938	0.360239	1.65056839	4.32786827	1.66651905	84	1001	105
285966	NM_001130025,NM_001130026	TCAF2	TRPM8 channel associated facto	2.109534038	1.016281035	0.300463	0.640926	0.288444	1.27880673	2.0043309	1.29257653	56	158	70
402573	NM_001004323	C7orf61	chromosome 7 open reading fran	-1.175286408	1.016272168	1.136706	0.966318	1.081768	1.08456011	0.96581954	1.09694451	44	50	55
619498	NR_002579	SNORD74	small nucleolar RNA, C/D box 74	-1.039051175	1.01624857	9.821271	9.574389	9.713768	0.77668602	0.75384785	0.78645063	28	36	35
282679	NM_173039	AQP11	aquaporin 11	-1.391779045	1.016237778	0.289952	0.20229	0.276863	0.68823103	0.52452456	0.69713258	24	23	30
57491	NM_001242412,NM_020731	AHRR	aryl-hydrocarbon receptor repres	-1.06824733	1.016162628	0.055466	0.049684	0.049476	0.38477185	0.36283659	0.39027104	12	15	15
693175	NR_030321	MIR590	microRNA 590	1.059749296	1.016162628	2.998786	3.327293	2.988956	0.38477185	0.40498891	0.39027104	12	17	15
7940	NM_001166538,NM_007161,NM	LST1	leukocyte specific transcript 1	-1.986550784	1.016162628	0.397985	0.20206	0.390481	0.38477185	0.20477631	0.39027104	12	8	15
693158	NR_030299	MIR573	microRNA 573	-1.002073241	1.016162628	2.91989	2.983665	2.828622	0.38477185	0.38406669	0.39027104	12	16	15
728920	NR_146087,NR_146087_dup1	PDE4DIPP1	phosphodiesterase 4D interacting	-1.184729479	1.016088172	0.095937	0.080758	0.088854	0.26755841	0.2284445	0.27153692	8	9	10
619564	NR_002583	SNORD72	small nucleolar RNA, C/D box 72	-1.067926637	1.016088172	2.551372	2.304646	2.461426	0.26755841	0.25173066	0.27153692	8	10	10
23513	NM_015356,NM_182706	SCRIB	scribbled planar cell polarity prote	-1.009366791	1.016060089	28.49824	28.276441	27.153371	7.17114833	7.1577913	7.19397627	5619	7438	7022
9340	NM_004246	GLP2R	glucagon like peptide 2 receptor	2.577321332	1.015868861	0.02277	0.058779	0.017484	0.13997292	0.3412894	0.14214747	4	14	5
26785	NR_002913	SNORD63	small nucleolar RNA, C/D box 63	1.483922171	1.015868861	1.340823	2.243284	1.289284	0.13997292	0.20477631	0.14214747	4	8	5
102467147	NR_104664	LINC01948	long intergenic non-protein codin	1.666155364	1.015868861	0.155842	0.268747	0.151245	0.13997292	0.2284445	0.14214747	4	9	5
56964	NM_001284395,NM_001284396	WDR93	WD repeat domain 93	4.035186879	1.015868861	0.051349	0.194099	0.041384	0.13997292	0.50527715	0.14214747	4	22	5
644990	NR_046244	THRB-AS1	THRB antisense RNA 1	1.483922171	1.015868861	0.091668	0.159881	0.111778	0.13997292	0.20477631	0.14214747	4	8	5
100847079	NR_049825	MIR5193	microRNA 5193	-1.324521912	1.015868861	0.728015	0.668965	0.735644	0.13997292	0.10601836	0.14214747	4	4	5
100616380	NR_039818	MIR4671	microRNA 4671	-1.06698239	1.015868861	1.167375	1.302698	1.228087	0.13997292	0.13134883	0.14214747	4	5	5
9541	NM_004882	CIR1	corepressor interacting with RBP	1.158579748	1.015749759	31.602121	36.650814	30.157845	5.81173868	6.02058584	5.83388654	2166	3353	2706
378805	NR_015431,NR_024153,NR_03	LINC-PINT	long intergenic non-protein codin	-1.548439963	1.015746851	10.893846	7.084392	10.809981	4.83204569	4.22867066	4.85380339	1079	931	1348
8322	NM_012193	FZD4	frizzled class receptor 4	-1.150846095	1.015528175	0.919943	0.801835	0.879967	2.93507482	2.76048215	2.95442604	261	303	326
9265	NM_004227	CYTH3	cytohesin 3	-2.335864373	1.01546492	16.842985	7.214375	16.072701	6.21441445	5.01611219	6.23625978	2876	1645	3592
114112	NM_001173513,NM_052883	TXNRD3	thioredoxin reductase 3	1.2009698	1.015040602	5.801765	6.962556	5.524266	4.11282598	4.36304264	4.13313083	640	1027	799
4054	NM_001130144,NM_001164266	LTBP3	latent transforming growth factor	1.401818069	1.014802054	67.865112	95.302554	64.565121	8.25674505	8.7426952	8.27787479	11969	22418	14939
7157	NM_000546,NM_001126112,NM	TP53	tumor protein p53	1.142693854	1.014721777	17.071396	19.375199	15.916391	5.43654533	5.62482969	5.45714754	1661	2536	2073
26030	NM_001308147	PLEKHG3	pleckstrin homology and RhoGEF	1.029632556	1.014482989	20.66807	21.301199	19.702307	6.55997524	6.60166589	6.58050223	3665	5042	4573
60437	NM_001348204,NM_021810,NM	CDH26	cadherin 26	-1.158580035	1.014475123	1.248051	1.0459	1.143296	2.73434891	2.55587612	2.75199454	222	256	277
5297	NM_058004	PI4KA	phosphatidylinositol 4-kinase alpl	1.485340785	1.01445127	21.146074	31.444895	20.157801	7.12950257	7.69693513	7.15005577	5458	10832	6810
9645	NM_001282663,NM_001282664	MICAL2	microtubule associated monooxy	-1.26061903	1.014403164	4.183081	3.343922	4.131794	4.50470835	4.18699961	4.52443946	852	903	1063
4522	NM_005956	MTHFD1	methylenetetrahydrofolate dehyd	1.241390017	1.014376757	68.483109	85.107719	65.277931	7.85113824	8.16188254	7.87164353	9026	14971	11261
26751	NM_001159597,NM_001282682	SH3YL1	SH3 and SYLF domain containin	-1.121493114	1.014295066	32.163175	29.476915	33.369514	6.23651379	6.07340943	6.25672228	2921	3480	3644
9881	NM_001329998,NM_014831	TRANK1	tetratricopeptide repeat and anky	1.006642556	1.014277	0.985372	0.992999	0.939424	3.4839374	3.49264009	3.50257812	400	538	499
253769	NM_001202550,NM_001350623	WDR27	WD repeat domain 27	1.107780103	1.013789841	7.238798	7.933576	7.45394	4.42825718	4.56941219	4.44710663	806	1193	1005
9534	NM_001278661,NM_001278662	ZNF254	zinc finger protein 254	-1.342725207	1.013789841	5.560731	3.935992	4.942631	4.42825718	4.02581645	4.44710663	806	802	1005
7529	NM_003404,NM_139323	YWHAB	tyrosine 3-monooxygenase/trypto	1.050428371	1.013759464	117.735065	123.485851	112.002193	8.49914316	8.56993004	8.51880458	14166	19882	17663
10920	NM_006710,NM_198189	COPS8	COP9 signalosome subunit 8	1.372675848	1.013545292	22.922031	31.505085	21.874365	5.68675843	6.13614594	5.70579569	1983	3637	2472
55612	NM_017671	FERMT1	fermitin family member 1	1.209566258	1.013522259	9.246817	11.197126	8.808133	5.56629422	5.83551351	5.58526687	1821	2943	2270
6651	NM_001291411,NM_001291412	SON	SON DNA binding protein	-1.289652238	1.01350124	34.541435	27.070184	33.682127	8.05584255	7.6904257	8.07511838	10408	10783	12974
284889	NR_038911	MIF-AS1	MIF antisense RNA 1	1.572353186	1.013356941	1.642184	2.59198	1.560812	2.17473011	2.70672986	2.18966672	138	290	172

7503	NR_001564	XIST	X inactive specific transcript (non	1.209348258	1.013344049	107.832375	130.550598	102.683746	10.98382266	11.25792943	11.00293738	79469	128409	99046
7569	NM_001007088,NM_001178099	ZNF182	zinc finger protein 182	1.089776911	1.01326368	3.491781	3.766653	3.326244	3.6887407	3.80355245	3.70628928	467	680	582
8581	NM_003695	LY6D	lymphocyte antigen 6 family mem	2.22644183	1.013075602	4.078798	9.145261	3.906992	2.07360076	3.02658682	2.08792485	126	375	157
79142	NM_001284517,NM_001284518	PHF23	PHD finger protein 23	1.422505582	1.01284632	12.938604	18.400108	12.230013	4.72217607	5.2143287	4.73989992	997	1895	1242
54455	NM_018994	FBXO42	F-box protein 42	1.278124567	1.012731356	4.04431	5.178078	3.85065	4.67692288	5.01865969	4.69446723	965	1648	1202
170959	NM_001319124,NM_001319126	ZNF431	zinc finger protein 431	1.223701846	1.012490398	5.659476	6.662145	5.824991	4.94491448	5.22760203	4.96224642	1170	1913	1457
23095	NM_015074,NM_183416	KIF1B	kinesin family member 1B	-1.116627578	1.012317445	4.745751	4.383003	4.256857	5.40826906	5.25306686	5.42551859	1628	1948	2027
132160	NM_001122870,NM_144641	PPM1M	protein phosphatase, Mg2+/Mn2-	1.186232488	1.012090935	2.998777	3.531304	2.776289	2.83562844	3.05002095	2.85055786	241	382	300
4939	NM_001032731,NM_002535,NM	OAS2	2'-5'-oligoadenylate synthetase 2	-3.370609505	1.012012339	13.044448	3.991668	11.747863	5.21404828	3.55012021	5.23081505	1418	562	1765
8605	NM_001159322,NM_001159323	PLA2G4C	phospholipase A2 group IVC	2.91989346	1.011974608	0.971386	2.815523	0.906101	1.76310734	2.99933041	1.77525664	94	367	117
54973	NM_001256456,NM_001256460	INTS11	integrator complex subunit 11	1.105291156	1.011954044	48.600128	53.748183	46.355133	6.68844815	6.83154513	6.70542715	4010	5922	4991
8560	NM_001321541,NM_001321542	DEGS1	delta 4-desaturase, sphingolipid	1.3582812	1.011864525	50.198652	68.371206	47.848864	6.67592393	7.11398966	6.6927751	3975	7214	4947
402483	NR_024368	LINC01000	long intergenic non-protein codin	-1.294844499	1.011835043	11.914993	9.212749	11.33015	6.90782119	6.53857084	6.92465517	4675	4824	5818
124454	NM_001083614,NM_001308211	EARS2	glutamyl-tRNA synthetase 2, mitc	1.212095725	1.011795789	10.030216	12.0509	9.368023	5.27889898	5.5499035	5.29538508	1485	2405	1848
222699	NR_002936	TOB2P1	transducer of ERBB2, 2 pseudog	2.889453207	1.011767363	0.325509	0.944443	0.308866	1.10178443	2.11004283	1.11084509	45	174	56
7581	NM_001278170,NM_001278171	ZNF33A	zinc finger protein 33A	1.108971435	1.011636878	6.365942	7.120415	6.052628	5.2722583	5.41781761	5.28852158	1478	2190	1839
10989	NM_001100169,NM_001100170	IMMT	inner membrane mitochondrial pr	1.16007054	1.011570616	52.801083	61.273475	50.039898	7.26516164	7.47808301	7.2816517	6000	9300	7465
100506314	NR_038920	LOC100506314	uncharacterized LOC100506314	-1.46312954	1.011550639	4.473777	3.061414	4.261627	3.10699502	2.63327382	3.12165574	299	273	372
55326	NM_018361	AGPAT5	1-acylglycerol-3-phosphate O-ac	1.207334229	1.011418978	8.854778	10.704989	8.419098	5.60304769	5.86978051	5.61909426	1869	3015	2325
100379571	NR_037803	BACE1-AS	BACE1 antisense RNA	-1.043388851	1.011340497	2.569817	2.474692	2.455632	1.62696745	1.58576189	1.63800401	82	105	102
7326	NM_003342	UBE2G1	ubiquitin conjugating enzyme E2	-1.05634931	1.011292812	11.825778	11.211444	11.240676	5.62852628	5.55107715	5.6444023	1903	2407	2367
3455	NM_000874,NM_001289125,NM	IFNAR2	interferon alpha and beta recepto	-1.497729824	1.011242646	4.194031	2.673348	3.931297	3.34979977	2.83560729	3.36435937	361	322	449
26003	NM_001201428,NM_015530	GORASP2	golgi reassembly stacking protein	1.294578581	1.011211773	39.694241	51.538736	37.716512	6.95182336	7.3216589	6.96777967	4821	8339	5996
152579	NM_152540	SCFD2	sec1 family domain containing 2	1.059175588	1.011160283	4.221724	4.481956	4.015373	3.8142098	3.8914258	3.82909231	513	726	638
31	NM_198834,NM_198836,NM_19	ACACA	acetyl-CoA carboxylase alpha	-1.031994585	1.011121693	14.095768	13.708655	13.430323	7.0513143	7.00622592	7.06715164	5168	6691	6427
8790	NM_001199328,NM_001199329	FPGT	fucose-1-phosphate guanylyltran	1.025023966	1.01100844	2.548222	2.672632	2.453742	3.66865353	3.70154644	3.68321628	460	630	572
201161	NM_181716	CENPV	centromere protein V	1.13235228	1.011005758	5.154574	5.848523	4.906445	2.77786255	2.93245472	2.79136857	230	348	286
4926	NM_001286561,NM_006185,NR	NUMA1	nuclear mitotic apparatus protein	1.071122968	1.011005051	38.513235	41.148136	36.31757	8.09332695	8.19210122	8.10905982	10683	15289	13284
5861	NM_004161,NM_015543	RAB1A	RAB1A, member RAS oncogene	1.076406363	1.010868643	66.956925	72.173436	63.958215	7.43553089	7.54116363	7.45103708	6757	9718	8401
404217	NM_206833	CTXN1	cortexin 1	4.031094224	1.010803674	1.522142	6.189056	1.455367	1.5285023	3.10539622	1.53866543	74	399	92
28972	NM_014041	SPCS1	signal peptidase complex subunit	1.019496527	1.010723842	71.967041	73.448334	68.337532	6.26594024	6.29343951	6.28113075	2982	4062	3707
91272	NM_001159651,NM_138369	BOD1	biorientation of chromosomes in	1.392594297	1.01055096	27.742774	38.736088	26.311427	5.73101721	6.20113534	5.74587647	2046	3807	2543
5296	NM_005027,NR_073517	PIK3R2	phosphoinositide-3-kinase regula	-1.054880238	1.010527043	6.90364	6.595328	6.578961	4.79942658	4.72518011	4.81399624	1054	1335	1310
6228	NM_001025	RPS23	ribosomal protein S23	-1.005690933	1.010503395	171.494965	170.714401	162.849472	9.11467889	9.10650665	9.12972608	21725	28863	27001
28982	NM_014053	FLVCR1	feline leukemia virus subgroup C	-1.058965692	1.010490233	6.762324	6.395994	6.422737	5.32637993	5.24583694	5.34106306	1536	1938	1909
148223	NM_152482	C19orf25	chromosome 19 open reading fra	1.252190452	1.010293145	10.17441	12.755408	9.654137	4.54619271	4.85819066	4.56033921	878	1469	1091
10152	NM_001282925,NM_001282926	ABI2	abl interactor 2	1.366366135	1.010241772	12.69568	17.521503	11.690995	6.3119937	6.75747409	6.32651073	3080	5623	3827
114897	NM_030968,NM_153372,NM_19	C1QTNF1	C1q and TNF related 1	-1.916412111	1.010150321	2.676843	1.354122	2.494519	3.09843961	2.30630165	3.11132089	297	207	369
101410543	NR_102710,NR_102711	XXYLT1-AS2	XXYLT1 antisense RNA 2	-2.886858339	1.010145536	4.084453	1.40213	3.861527	2.12505137	1.11294744	2.1362985	132	61	164
283687	NR_028330	ST20-AS1	ST20 antisense RNA 1	-1.571795699	1.010119773	0.40489	0.261965	0.392673	0.88013425	0.61708849	0.88680889	33	28	41
6472	NM_001166356,NM_001166357	SHMT2	serine hydroxymethyltransferase	-1.029945088	1.010086241	54.145201	52.716591	51.349795	6.92546251	6.88324948	6.9398228	4733	6140	5880
6498	NM_001145097,NM_001145098	SKIL	SKI like proto-oncogene	1.028684482	1.01004534	14.881024	15.387743	14.122872	6.71527492	6.7556936	6.72955881	4086	5616	5076
126695	NM_152365	KDF1	keratinocyte differentiation factor	1.015462162	1.009979696	14.436521	14.676217	13.691956	4.71938895	4.74069359	4.73317556	995	1350	1236
51547	NM_016538	SIRT7	sirtuin 7	-1.080728987	1.00995426	54.090996	50.117592	51.339836	6.52648463	6.41573924	6.54062077	3580	4426	4447
23593	NM_001326380,NM_001326381	HEBP2	heme binding protein 2	-1.3629761	1.009873682	34.535986	25.205627	32.537419	5.44669174	5.01185634	5.46054405	1673	1640	2078
26996	NM_014373	GPR160	G protein-coupled receptor 160	-1.387288522	1.009849784	12.671259	9.137454	12.023259	4.69121758	4.2403589	4.70481505	975	939	1211
8934	NM_001135662,NM_001135663	RAB29	RAB29, member RAS oncogene	1.318193698	1.009821036	4.551335	6.205274	4.76637	4.01393646	4.39083965	4.02716981	595	1048	739
6132	NM_000973,NM_001317771,NM	RPL8	ribosomal protein L8	-1.010799041	1.009763871	1369.488976	1369.823864	1325.321216	10.30666781	10.29118388	10.32067479	49685	65676	61706
1655	NM_001320595,NM_001320596	DDX5	DEAD-box helicase 5	1.149177778	1.009713511	259.66287	299.561334	246.41675	9.94005716	10.14046902	9.95398908	38527	59156	47846
4800	NM_002505,NM_021705	NFYA	nuclear transcription factor Y sub	-1.234421525	1.009532865	6.207815	5.034219	5.940831	5.26367512	4.96859275	5.27700936	1469	1590	1824
10360	NM_006993	NPM3	nucleophosmin/nucleoplasmin 3	-1.652272359	1.009504937	37.379581	22.656147	35.464577	5.04180235	4.34556265	5.05503907	1254	1014	1557
79819	NM_024763,NM_207014	WDR78	WD repeat domain 78	1.906453281	1.009268738	0.196423	0.372745	0.182778	0.79797761	1.26944952	0.80366934	29	74	36
79768	NM_024713	KATNBL1	katanin regulatory subunit B1 like	-1.470008737	1.009178172	12.777184	8.703258	12.117358	5.13363806	4.59694851	5.14644623	1339	1217	1662
199870	NM_001143912,NM_001143913	FAM76A	family with sequence similarity 76	1.007015813	1.008892856	3.246405	3.248426	3.050089	3.51962058	3.52883263	3.53128747	411	553	510
401261	NR_134654	LOC401261	uncharacterized LOC401261	-1.423626643	1.008779304	1.535545	1.082151	1.458729	2.3579234	1.96260273	2.36808966	162	152	201
7597	NM_001304507,NM_001304508	ZBTB25	zinc finger and BTB domain conta	-1.142075641	1.008725988	10.428155	10.068914	10.766331	4.37789078	4.19603101	4.38982644	777	909	964
50810	NM_016073	HDGFL3	HDGF like 3	1.01403596	1.008649642	17.95822	18.230652	17.020321	5.44500562	5.4646572	5.45714754	1671	2264	2073
55620	NM_001013841,NM_017720	STAP2	signal transducing adaptor family	-1.249668712	1.008439837	45.841912	36.771628	42.774931	6.0321428	5.71607575	6.04408383	2530	2705	3138
7718	NM_003447	ZNF165	zinc finger protein 165	-1.549277197	1.008357413	8.151364	5.268695	7.726191	4.31281697	3.72044785	4.32422401	741	639	919
7379	NM_006760	UPK2	uroplakin 2	-5.934330966	1.007976993	26.618031	4.477034	25.22212	4.65666933	2.34470901	4.66768061	951	214	1179
5627	NM_000313,NM_001314077	PROS1	protein S (alpha)	1.385727368	1.007808817	1.87192	2.664201	1.817756	2.94465259	3.36231592	2.95442604	263	487	326
11180	NM_001320546,NM_001320547	WDR6	WD repeat domain 6	1.053976287	1.007790713	40.806647	42.765192	38.897886	7.4788165	7.55424579	7.48995022	6964	9807	8632
3658	NM_001320941,NM_001320942	IREB2	iron responsive element binding p	-1.202010873	1.00775358	13.985235	11.359013	13.241898	6.358415	6.09650305	6.369423	3182	3537	3944
51559	NM_001031701	NT5DC3	5'-nucleotidase domain containin	-2.06641632	1.007654944	7.149565	3.462258	6.769036	5.67888953	4.66140215	5.68967788	1972	1275	2444
118426	NM_001300742,NM_001330356	BORCS5	BLOC-1 related complex subunit	-1.165391314	1.007599327	1.140604	0.985878	1.079062	3.02359429	2.83174937	3.03318152	280	321	347
100507507	NM_001322263	LOC100507507	uncharacterized LOC100507507	-1.375222827	1.007599327	4.267302	3.101133	4.036541	3.02359429	2.62883416	3.03318152	280	272	347
55916	NM_001242617,NM_001242618	NXT2	nuclear transport factor 2 like exp	-1.086193427	1.007581947	3.278561	3.022611	3.098462	3.21778616	3.11177339	3.22751932	326	401	404
4946	NM_001301020,NM_004152	OAZ1	ornithine decarboxylase antizyme	-1.078293754	1.007457287	375.279265	348.40439	355.29891	8.75632156	8.64783177	8.76701561	16938	20988	20988
5029	NM_002564,NM_176071,NM_17	P2RY2	purinergic receptor P2Y2	1.435219625	1.007333601	4.487346	6.481293	4.271828	5.28739233	5.79744877	5.29766568	1494	2865	1851
29997	NM_015710	NOP53	NOP53 ribosome biogenesis fact	-1.218976231	1.007333168	99.978127	82.10498	94.648415	7.22076946	6.93721004	7.23124017	5817	6376	7207
9180	NM_001168355,NM_001323504	OSMR	oncostatin M receptor	1.074584524	1.007307137	9.859456	10.256988	10.773637	5.53651876	5.63814463	5.54679755	1783	2560	2209
2152	NM_001178096,NM_001993	F3	coagulation factor III, tissue facto	1.585792947	1.007142196	393.91787	625.019013	372.251295	9.83574741	10.5003701	9.84600364	35837	75932	44392
9524	NM_001321170,NM_138501,NR	TECR	trans-2,3-enoyl-CoA reductase	-1.137279571	1.006990611	155.725581	137.001921	147.247025	7.47696127	7.29248258	7.48695545	6955	8171	8614
79685	NM_001131062,NM_001131063	SAP30L	SAP30 like	-1.184105534	1.006851798	2.987931	2.52252	2.81897	4.2524017	4.02243562	4.26173929	709	800	878
7051	NM_000359	TGM1	transglutaminase 1	-1.055783008	1.006728651	3.787167	3.59735	3.590629	3.49375703	3.42255083	3.50257812	403	510	499
8548	NM_001320972,NM_001320973	BLZF1	basic leucine zipper nuclear facto	-1.125107467	1.006684214	15.179613	13.320793	14.738199	5.33095191	5.16535412	5.34032578	1541	1830	1908
84223	NM_001134435,NM_001323027	IQCG	IQ motif containing G	2.050243867	1.006631728	3.672765	7.553898	3.244219	2.99161027	3.93163239	2.99995417	273	748	338
55721	NM_001160042,NM_018134	IQCC	IQ motif containing C	-1.125751306	1.006631728	3.449422	2.767427	3.24865	2.99161027	2.84329233	2.99995417	273	324	338
100507173	NR_038292,NR_038293,NR_038	LINC01012	long intergenic non-protein codin	-2.332920025	1.006622666	1.168454	0.694323	1.328033	1.38110603	0.75384785	1.38699077	63	36	78
5923	NM_001145648,NM_002891,NM	RASGRF1	Ras protein specific guanine nucl	2.274448555	1.006599221	0.083654	0.194189	0.079298	0.61813834	1.15059938	0.62147005	21	64	26
2617	NM_001316772,NM_002047	GARS	glycyl-tRNA synthetase	-1.172923995	1.006581539	79.199099	67.607066	74.924492	7.55250536	7.32372052	7.5619193	7331	8351	9076
55810	NM_018416	FOXJ2	forkhead box J2	1.115094554	1.006477604	4.731104	5.283015	4.476116	4.71519816	4.86669962	4.72416071	992	1478	1228
10737	NR_001450,NR_002596,NR_104	RFPL3S	RFPL3 antisense	1.149747982	1.006475408	5.194354	5.986891	4.938312	2.87121968	3.04669644	2.8792661	248	381	307
1471	NM_000099,NM_001288614	CST3	cystatin C	1.475890919	1.006366758	154.720806	228.76243	145.917657	7.10194744	7.66015249	7.11103736	5354	10558	6627
53339	NM_001011885,NM_025238	BTBD1	BTB domain containing 1	-1.068129785	1.00623842	15.60267	14.616895	14.717403	5.66808957	5.57492912	5.67688633	1957	2448	2422
400818	NM_001037675,NM_001277444	NBPF9	NBPF member 9	-1.02144336	1.006162096	17.964549	17.657643	17.016014	6.47122805	6.44096653	6.47999149	3444	4505	4262
6045	NM_007212	RNF2	ring finger protein 2	1.195099492	1.00616203	6.043227	7.233983	5.713958	4.45363476	4.70000624	4.46209528	821	1311	1016
23272	NM_001112736,NM_015224	FAM208A	family with sequence similarity 20	-1.113352835	1.00604075	7.697475	7.04455	7.252721	5.96549005	5.81318676	5.97404055	2414	2897	2987
79091	NM_024109,NR_134608	METTL22	methyltransferase like 22	-1.032304048	1.006020777	10.489189	10.164575	9.897102	4.09573376	4.05258161	4.10389022	632	818	782
9231	NM_004747	DLG5	discs large MAGUK scaffold prot	-1.598209348	1.005980519	20.091187	12.58378	18.992638	7.20267282	6.53204743	7.21121713	5744	4802	7107
57448	NM_016252	BIRC6	baculoviral IAP repeat containing	1.058463766	1.005887625	8.120489	8.603684	7.675389	6.96747045	7.04880718	6.9758723	4874	6893	6030
25998	NM_001300906,NM_015525	IBTK	inhibitor of Bruton tyrosine kinase	-3.718189191	1.005841489	13.12858	3.533955	12.447231	6.28774036	4.44234896	6.29603638	3028	1088	3746
10280	NM_001282205,NM_001282206	SIGMAR1	sigma non-opioid intracellular rec	1.542188156	1.005629104	48.17586	74.486028	45.549551	6.33037656	6.94905571	6.3383748	3120	6429	3859
8704	NM_001005417,NM_003780,NM	B4GALT2	beta-1,4-galactosyltransferase 2	1.071886879	1.005412831	28.108706	29.167649	25.067562	5.84600152	5.94447568	5.85365487	2219	3178	2744
6499	NM_006929	SKIV2L	Ski2 like RNA helicase	-1.10068342	1.005337702	14.982869	13.627516	14.156252	5.95071282	5.81465343	5.95826952	2389	2900	2954
83959	NM_001174089,NM_001174090	SLC4A11	solute carrier family 4 member 11	-3.034725697	1.005315047	12.554807	4.128836	11.888883	5.29021237	3.76158491	5.29766568	1497	659	1851
9296	NM_001198909,NM_004231	ATP6V1F	ATPase H+ transporting V1 subu	-1.705233669	1.005069006	66.760381	39.335462	63.247581	5.6113401	4.86197862	5.61848624	1880	1473	2324
10106	NM_005730	CTDSP2	CTD small phosphatase 2	1.015454121	1.004941684	17.044508	17.328962	16.094336	6.39773442	6.41959976	6.40476231	3271	4438	4043
64332	NM_001005474,NM_031419	NFKBIZ	NFKB inhibitor zeta	1.572178588	1.004786189	14.117043	22.218683	13.313354	5.77865184	6.42184697	5.78541555	2116	4445	2615

55824	NM_018440	PAG1	phosphoprotein membrane ancho	4.498624289	1.004694856	0.339287	1.536092	0.320951	2.19091668	4.09182073	2.19620105	140	842	173
23283	NM_015235	CSTF2T	cleavage stimulation factor subur	-1.108779796	1.004657147	8.102112	7.313018	7.646976	5.06284359	4.91854554	5.06934731	1273	1534	1573
8502	NM_001005476,NM_001304969	PKP4	plakophilin 4	1.090624171	1.004492557	24.344232	26.685733	23.138723	6.7647759	6.88883011	6.7711836	4230	6164	5226
535	NM_001130020,NM_001130021	ATP6V0A1	ATPase H+ transporting V0 subu	-1.113350734	1.004397423	14.885937	13.37506	14.074719	5.92976705	5.77753174	5.93599396	2354	2825	2908
57097	NM_001286521,NM_001286522	PARP11	poly(ADP-ribose) polymerase fan	1.26645235	1.004354789	1.263881	1.641055	1.190606	2.72881625	3.0232078	2.73414401	221	374	273
56144	NM_018907,NM_031500	PCDHA4	protocadherin alpha 4	-1.430323707	1.004353409	0.697484	0.522476	0.73886	2.15835987	1.77444288	2.16322937	136	127	168
352961	NR_002812	HCG26	HLA complex group 26 (non-prot	-2.062942541	1.004352897	2.630615	1.2633	2.47564	2.01115992	1.30327576	2.01587867	119	77	147
101927221	NR_125415	UBR5-AS1	UBR5 antisense RNA 1	-1.999978186	1.004347441	1.7898	0.889926	1.6935	1.2010231	0.72085408	1.20457043	51	34	63
387804	NM_001144871	VSTM5	V-set and transmembrane domai	-1.032248592	1.004328453	0.717043	0.683287	0.689873	0.5190475	0.50527715	0.52094563	17	22	21
84461	NM_001005408,NM_032442	NEURL4	neuralized E3 ubiquitin protein lig	1.29456629	1.004286776	6.903762	8.948919	6.515495	5.1647037	5.52801678	5.1707038	1369	2368	1691
51447	NM_001005909,NM_001005910	IP6K2	inositol hexakisphosphate kinase	1.312078851	1.004282315	38.893837	50.106861	36.201017	6.05332436	6.44001655	6.05939688	2568	4502	3172
3066	NM_001527,NR_033441,NR_07	HDAC2	histone deacetylase 2	1.231387269	1.004281826	17.529202	21.606497	16.559762	6.8404776	7.13840114	6.84658827	4460	7338	5509
10915	NM_001040006,NM_006706	TCERG1	transcription elongation regulator	-1.077479548	1.004231097	52.790227	48.874358	49.755164	7.74122382	7.63408424	7.7472868	8361	10368	10327
4253	NM_001247988,NM_001247989	MIA2	melanoma inhibitory activity 2	-1.006758959	1.004222958	20.438818	20.545864	18.972758	6.20547905	6.19589251	6.21147669	2858	3793	3530
57142	NM_001321859,NM_001321860	RTN4	reticulon 4	1.109893477	1.004149331	223.056622	249.390534	210.121202	9.01478455	9.16493035	9.0207469	20269	30058	25033
55135	NM_001143990,NM_001143991	WRAP53	WD repeat containing antisense t	1.393886441	1.004143877	6.162213	8.713452	5.89431	3.64240927	4.08859126	3.64789978	451	840	557
91966	NM_001171907,NM_001171908	CXorf40A	chromosome X open reading fran	-1.050893836	1.004094815	3.680336	3.581097	3.889337	3.90531607	3.83857782	3.91081999	549	698	678
89866	NM_033127	SEC16B	SEC16 homolog B, endoplasmic	-1.262844578	1.003953928	6.946104	5.508021	6.552503	4.81908641	4.49574422	4.82457876	1069	1131	1320
9986	NM_001032279,NM_005133	RCE1	Ras converting CAAX endopeptic	1.341272647	1.003879058	28.501884	38.279929	26.894176	5.38559376	5.80041275	5.39104626	1602	2871	1978
79047	NM_001129994,NM_001129995	KCTD15	potassium channel tetramerizatio	1.415837264	1.00381179	5.581681	7.909873	5.044001	4.54619271	5.02964715	4.55144767	878	1661	1084
10825	NM_006656	NEU3	neuraminidase 3	1.703076116	1.003607602	1.8256	3.114318	1.71819	2.55837852	3.22198991	2.56269567	192	437	237
135932	NM_001242773,NM_001242774	TMEM139	transmembrane protein 139	-1.783012336	1.003508083	28.647986	15.905027	26.417931	5.86818985	5.05303203	5.87315599	2254	1689	2782
51091	NM_016955	SEPSECS	Sep (O-phosphoserine) tRNA:Se	-1.237960667	1.003371572	7.419998	6.00061	6.997742	5.38559376	5.08579485	5.39033409	1602	1729	1977
79676	NM_001304833,NM_001304834	OGFOD2	2-oxoglutarate and iron depender	-1.264761358	1.003309977	15.106394	11.865091	14.451294	4.96623093	4.63950051	4.9708465	1188	1255	1466
85378	NM_020461	TUBGCP6	tubulin gamma complex associat	1.115009935	1.003254283	11.496419	12.834756	10.84115	6.09585169	6.25073686	6.10047078	2646	3942	3265
2585	NM_001001556,NM_001289030	GALK2	galactokinase 2	-1.466344558	1.003229299	8.6931	5.954165	8.276769	4.8626811	4.33333585	4.86717332	1103	1005	1361
140545	NM_001184996,NM_001184997	RNF32	ring finger protein 32	-1.018601602	1.003110222	1.029786	1.02899	0.960346	1.56621863	1.5486383	1.56919173	77	101	95
10450	NM_001195007,NM_001319293	PPIE	peptidylprolyl isomerase E	1.125774266	1.003015901	20.970289	17.983694	17.704764	5.72199495	5.88986396	5.72625748	2033	3058	2508
11152	NM_001029896,NM_007075	WDR45	WD repeat domain 45	1.137380378	1.002960119	10.916617	12.503239	10.329855	4.20536975	4.38163336	4.20940382	685	1041	845
101927541	NR_109943	LINC01273	long intergenic non-protein codin	-2.352176484	1.002757628	2.206651	0.922065	2.088234	1.33814853	0.72085408	1.34055586	60	34	74
284697	NM_183242	BTBD8	BTB domain containing 8	-4.940038022	1.002752494	0.53564	0.096971	0.503746	0.81895953	0.20477631	0.82068496	30	8	37
1431	NM_004077	CS	citrate synthase	-1.016384648	1.002729274	49.850792	49.100151	46.972652	7.18434629	7.16106187	7.18825152	5671	7455	6994
80305	NM_001320484,NM_001320485	TRABD	TraB domain containing	1.042989293	1.002628468	22.943998	24.057314	21.848146	5.71291592	5.77250923	5.71663112	2020	2815	2491
23325	NM_001293640,NM_015275	WASHC4	WASH complex subunit 4	-1.415222576	1.002591149	12.799464	9.050771	12.058665	6.19849084	5.70557366	6.20217361	2844	2685	3507
79719	NM_001271885,NM_001271886	AAGAB	alpha- and gamma-adaptin bindir	1.021044408	1.002568256	15.801381	16.225045	14.99416	5.66447154	5.69393237	5.66809934	1952	2663	2407
5558	NM_000947,NM_001282487,NM	PRIM2	primase (DNA) subunit 2	1.013364887	1.002522525	8.25786	8.442423	7.530045	4.19135823	4.20947297	4.19479474	678	918	836
25801	NM_001330265,NM_001330266	GCA	grancalcin	-1.065548911	1.002185365	12.580775	11.800327	11.839996	5.53731025	5.44774276	5.54039208	1784	2237	2199
79609	NM_001040662,NM_024558,NR	VCPKMT	valosin containing protein lysine r	-1.030994711	1.002171525	14.099753	13.552101	13.155811	4.67404687	4.6317557	4.67705421	963	1248	1187
8683	NM_003769	SRSF9	serine and arginine rich splicing f	1.15103037	1.00216564	115.374092	132.941696	108.638344	7.04046851	7.24195961	7.04356587	5129	7888	6322
80347	NM_001042529,NM_001042532	COASY	Coenzyme A synthase	-1.111695504	1.002162635	52.160678	47.290657	49.117733	7.00771875	6.85620532	7.01081128	5013	6025	6179
57127	NM_001256395,NM_001256396	RHBG	Rh family B glycoprotein (gene/ps	-1.550662833	1.002126963	0.537001	0.28915	0.433093	1.06712766	0.77006602	1.06873498	43	37	53
23270	NM_021648	TSPYL4	TSPY like 4	1.300256261	1.001941151	3.758806	4.895907	3.541042	4.01166006	4.36970972	4.01428503	594	1032	732
84640	NM_001290325,NM_001290326	USP38	ubiquitin specific peptidase 38	1.239392917	1.001810109	3.883743	4.648418	3.705448	4.63907992	4.93751728	4.64158467	939	1555	1157
140691	NM_001301144,NM_001301145	TRIM69	tripartite motif containing 69	-2.261891528	1.001791277	0.861414	0.475316	0.712822	1.27880673	0.70406985	1.28032816	56	33	69
10388	NM_014258	SYCP2	synaptonemal complex protein 2	-2.042333132	1.001685496	4.362599	2.138695	4.106682	4.62723337	3.65645563	4.62956502	931	609	1147
80862	NR_026751,NR_145416,NR_14	ZNRD1ASP	zinc ribbon domain containing 1 a	1.382597798	1.001505293	1.612626	2.190722	1.429743	2.27683528	2.65962865	2.27855908	151	279	186
11282	NM_014275,NM_054013	MGAT4B	mannosyl (alpha-1,3-)-glycoprote	-1.049778988	1.001360136	94.065556	84.368436	91.691099	7.95741376	7.88761625	7.95936683	9719	12370	11970
8214	NM_005675	DGCR6	DiGeorge syndrome critical regio	2.419932802	1.001256805	9.298568	22.54944	8.744194	3.58538547	4.78824961	3.58704707	432	1397	532
55756	NM_001145159,NM_001172562	INTS9	integrator complex subunit 9	1.196618062	1.001144529	5.066706	6.077355	4.781427	3.8730763	4.11581404	3.87461432	536	857	660
1616	NM_001141969,NM_001141970	DAXX	death domain associated protein	-1.024273073	1.000874277	15.929936	15.575693	14.995745	5.37766084	5.3439002	5.37889138	1593	2078	1961
285368	NM_001318871,NM_207351,NR	PRRT3	proline rich transmembrane prote	1.366954573	1.000677305	0.723359	0.988235	0.733393	1.86749926	2.20853486	1.86820921	104	190	128
285540	NR_037934	SEPSECS-AS1	SEPSECS antisense RNA 1 (hea	1.435710285	1.000672237	0.280299	0.42558	0.285547	0.41264541	0.56226574	0.41288872	13	25	16
23126	NM_001194937,NM_001194938	POGZ	pogo transposable element deriv	1.317224533	1.000520627	12.89876	17.37734	11.83615	6.35707077	6.75033891	6.35781255	3179	5595	3912
1939	NM_001201478,NM_006893	EIF2D	eukaryotic translation initiation fa	-1.120762355	1.000446908	27.007452	24.250248	25.389233	5.81108433	5.64969525	5.81171749	2165	2581	2664
9394	NM_004807	HS6ST1	heparan sulfate 6-O-sulfotransfer	2.200170323	1.00038972	2.871958	6.327415	2.697864	3.59149522	4.66248855	3.59201087	434	1276	534
79713	NM_001346003,NM_001346004	IGFLR1	IGF like family receptor 1	-1.720234031	1.000266375	2.614732	1.511162	2.362067	2.28439817	1.70021226	2.28470378	152	118	187
2975	NM_001286242,NM_001520	GTF3C1	general transcription factor IIIC su	1.410273005	1.000257574	13.206195	18.639922	12.420611	6.52249295	7.01390728	6.52286048	3570	6727	4392
7084	NM_001172643,NM_001172644	TK2	thymidine kinase 2, mitochondria	-1.05967748	1.000198151	5.924214	5.143588	5.423504	4.94848917	4.86764197	4.94876578	1173	1479	1443
11230	NM_007213	PRAF2	PRA1 domain family member 2	-1.414220083	1.000181505	2.50283	1.774383	2.354298	2.07360076	1.70865125	2.07380058	126	119	155
23341	NM_001287811,NM_015291,NR	DNAJC16	DnaJ heat shock protein family (H	1.198948805	1.000165797	5.414407	6.463291	5.082234	5.00451141	5.25882482	5.00474315	1221	1956	1502
58505	NM_001267817,NM_001267818	OSTC	oligosaccharyltransferase comple	-1.012003069	-1.000019464	85.174655	84.065112	79.765326	6.49301423	6.47599228	6.49298646	3497	4617	4301
64210	NM_001289403,NM_001289404	MMS19	MMS19 homolog, cytosolic iron-s	-1.714400634	-1.000081184	26.468096	15.737574	25.123078	6.57624091	5.80926833	6.57612501	3707	2889	4559
84163	NM_001281447,NM_173537,NM	GTF2IRD2	GTF2I repeat domain containing	-1.379811387	-1.000205917	6.596975	2.756209	6.687949	3.0370876	2.63769986	3.03682664	283	274	348
80264	NM_001172671,NM_025189	ZNF430	zinc finger protein 430	-1.225996734	-1.000235946	3.193622	2.606085	2.997306	3.72253824	3.45300696	3.72222359	479	522	589
57646	NM_001301029,NM_001346252	USP28	ubiquitin specific peptidase 28	-1.011676407	-1.000476956	6.035066	6.073909	5.67462	4.76605286	4.74992223	4.76539014	1029	1359	1265
100616408	NR_039969	MIR5047	microRNA 5047	1.048796073	-1.000647738	394.789185	414.706238	370.924805	5.30236925	5.36940611	5.30145868	1510	2116	1856
79754	NM_024701,NR_024581,NR_03	ASB13	ankyrin repeat and SOCS box co	1.103538407	-1.000788976	6.748145	7.673366	6.521274	4.28292556	4.4181112	4.28184606	725	1069	891
9968	NM_005120	MED12	mediator complex subunit 12	1.047011557	-1.001037996	8.484261	8.894096	7.965258	5.87258686	5.9377612	5.87111561	2261	3163	2778
8161	NM_004645	COIL	coilin	-1.092886065	-1.001074828	26.24696	24.038965	24.638334	6.09370103	5.96751352	6.09217385	2642	3230	3246
339674	NR_024355	LINC00634	long intergenic non-protein codin	1.495354316	-1.001106573	0.607826	0.898569	0.564406	0.91952222	1.22307764	0.91876801	35	70	43
441389	NR_038977	LINC01239	long intergenic non-protein codin	2.61704845	-1.001108344	0.769796	2.040961	0.726771	1.47662927	2.50348775	1.47560397	70	245	86
6493	NM_005069,NM_009586	SIM2	single-minded family bHLH trans	1.12001652	-1.001147787	11.194088	12.938506	10.833102	5.58937463	5.74968897	5.58775396	1851	2770	2274
90379	NM_138353	DCAF15	DDB1 and CUL4 associated fact	1.042098676	-1.001217946	31.964071	33.351395	30.00705	6.17935039	6.23804005	6.17761851	2806	3907	3447
340371	NM_178564	NRBP2	nuclear receptor binding protein 2	1.26946145	-1.001256418	13.412885	17.045509	12.586336	5.63297616	5.97102538	5.63120109	1909	3238	2345
1138	NM_000745,NM_001307945	CHRNA5	cholinergic receptor nicotinic alph	-1.082829481	-1.001271167	10.855845	9.705819	9.995788	5.24053381	5.12887652	5.23874944	1445	1783	1775
157777	NM_001136160,NM_001136161	MCMDC2	minichromosome maintenance do	-1.165037516	-1.001362415	0.942078	0.815579	0.951156	2.5075876	2.32837375	2.50596801	184	211	226
84441	NM_032427	MAML2	mastermind like transcriptional co	-1.111713888	-1.001457613	5.175241	4.661004	4.854887	5.20311692	5.05468796	5.20107248	1407	1691	1728
28955	NM_014015	DEXI	Dexi homolog	1.075681262	-1.001464027	9.159319	9.870178	8.597095	3.84777676	3.94597954	3.84581243	526	756	646
83723	NM_001318504,NM_001352173	FAM57B	family with sequence similarity 57	1.050387014	-1.001517243	1.397158	1.50392	1.315842	1.96484452	2.01797632	1.96321628	114	160	140
89765	NM_001286506,NM_080860	RSPH1	radial spoke head 1 homolog	-1.180559159	-1.001517243	2.242855	1.830808	2.008724	1.96484452	1.79043244	1.96321628	114	129	140
6223	NM_001022,NM_001321483,NM	RPS19	ribosomal protein S19	-1.268986235	-1.001615879	973.868622	801.694218	922.062348	9.60286328	9.25968447	9.60053693	30489	32102	37439
84936	NM_001077268,NM_001258420	ZFYVE19	zinc finger FYVE-type containing	1.187333262	-1.001741753	17.961534	21.187084	16.854518	5.26940291	5.51123031	5.26695724	1475	2340	1811
7268	NM_001291333,NM_004623	TTC4	tetratricopeptide repeat domain 4	1.122339074	-1.00189978	13.652276	15.29141	12.699474	5.02497132	5.18665501	5.02231705	1239	1858	1521
57523	NM_025081	NYNRIN	NYN domain and retroviral integr	1.097521693	-1.001958157	2.926111	3.214237	2.743044	4.54776468	4.67653783	4.54506286	879	1289	1079
11243	NM_001199653,NM_001199654	PMF1	polyamine modulated factor 1	1.058126355	-1.002047427	28.476886	30.189289	26.664712	5.00107303	5.0801148	4.99821421	1218	1722	1495
57403	NM_020673	RAB22A	RAB22A, member RAS oncogen	1.023270365	-1.002065027	7.524438	7.710488	7.058314	6.00893474	6.04161395	6.00600474	2489	3403	3055
23560	NM_012341	GTPBP4	GTP binding protein 4	1.148935402	-1.002152836	46.45565	53.43832	43.562222	6.84368056	7.04235351	6.84060498	4470	6862	5486
128497	NM_080608	SPATA25	spermatogenesis associated 25	1.765304197	-1.002158474	0.76554	1.397235	0.734161	0.64188297	0.99371033	0.64076093	22	52	27
100507530	NR_125386	MCPH1-AS1	MCPH1 antisense RNA 1	5.692491845	-1.002158474	0.13506	0.79634	0.126971	0.64188297	2.07130288	0.64076093	22	168	27
64838	NM_022823	FNDC4	fibronectin type III domain contai	-4.80835891	-1.002158474	0.334513	0.062825	0.313436	0.64188297	0.15624221	0.64076093	22	6	27
79947	NM_001243564,NM_001243565	DHDDS	dehydrodolichyl diphosphate syn	-1.042618427	-1.002401501	8.871018	8.500247	8.333115	4.88274916	4.82461482	4.87940578	1119	1434	1373
79647	NM_001136275,NM_024595	AKIRIN1	akirin 1	1.387811002	-1.002467049	27.605404	38.354158	25.946708	6.21094615	6.67831955	6.20743924	2869	5320	3520
9989	NM_001042388,NM_005134,NR	PPP4R1	protein phosphatase 4 regulatory	-1.174674832	-1.00257209	28.222446	24.057027	26.436263	6.76882532	6.53886665	6.76515328	4242	4825	5204
51006	NM_001281457,NM_001281458	SLC35C2	solute carrier family 35 member C	-1.104682986	-1.002627836	29.789905	27.637628	27.715809	6.11028539	5.96883147	6.10655391	2673	3233	3279
84893	NM_001258452,NM_001258453	FBXO18	F-box protein, helicase, 18	1.174806185	-1.002634654	18.137459	21.651569	17.403932	6.04555676	6.27473483	6.04181814	2554	4009	3133
25890	NM_001349329,NM_001349330	ABI3BP	ABI family member 3 binding pro	1.321186044	-1.002747379	0.525246	0.758327	0.487335	2.09095564	2.40826098	2.08792485	128	226	157
256643	NM_198279	CXorf23	chromosome X open reading fran	-1.044168485	-1.002775225	1.973111	1.892086	1.849569	3.7528388	3.69519054	3.74913666	490	627	601
727800	NM_031297	RNF208	ring finger protein 208	-1.250824001	-1.002838757	12.106478	9.695662	11.352721	3.77716539	3.48037129	3.77337356	499	533	612
728855	NR_024510,NR_024510_dup1,N	LINC00623	long intergenic non-protein codin	1.309604001	-1.002868794	1.638625	2.178037	1.544112	2.6780437	3.01302298	2.67455554	212	371	260

6908	NM_001172085,NM_003194	TBP	TATA-box binding protein	1.194602924	-1.003032715	6.52031	7.767315	6.115708	3.70573844	3.94419393	3.7017041	473	755	580
3814	NM_002256	KISS1	KiSS-1 metastasis-suppressor	-3.291371945	-1.003049942	3.075983	0.926131	2.871543	1.65056839	0.72085408	1.64757178	84	34	103
4152	NM_001204136,NM_001204137	MBD1	methyl-CpG binding domain prote	1.195218036	-1.003058614	17.302899	21.038377	16.262904	5.73170889	5.98455365	5.72738578	2047	3269	2510
51571	NM_001256763,NM_001330612	FAM49B	family with sequence similarity 49	-1.629648552	-1.003289588	35.754417	21.866034	33.294984	7.10728754	6.40928199	7.10258378	5374	4406	6588
692057	NR_003030	SNORD12	small nucleolar RNA, C/D box 12	1.44703958	-1.003356086	8.780864	13.039715	8.327344	0.83964067	1.10017522	0.83750224	31	60	38
101927621	NR_110707,NR_110708	LINC01534	long intergenic non-protein codin	-1.378854087	-1.003356086	0.901869	0.647947	0.844913	0.83964067	0.65251278	0.83750224	31	30	38
51177	NM_001304722,NM_001304723	PLEKHO1	pleckstrin homology domain cont	-1.035452584	-1.003364195	1.166478	1.129354	1.065617	1.75224037	1.71704117	1.74883089	93	120	114
6992	NM_021959	PPP1R11	protein phosphatase 1 regulatory	-1.18270071	-1.0033891	19.65127	16.637007	18.412539	5.03621379	4.80211744	5.0314812	1249	1411	1531
9550	NM_004888	ATP6V1G1	ATPase H+ transporting V1 subu	-1.016451412	-1.003676392	52.869858	52.072815	49.503296	6.39118219	6.36792291	6.38595101	3256	4280	3990
8496	NM_001198915,NM_001198916	PPFIBP1	PPFIA binding protein 1	-1.027639405	-1.003729386	4.770855	4.644664	4.463988	4.85381277	4.81585323	4.84862794	1096	1425	1343
84268	NM_001033002,NM_001160243	RPAIN	RPA interacting protein	-1.082790661	-1.003827714	13.594309	12.500946	12.193915	4.60927912	4.49939675	4.60399302	919	1134	1126
54880	NM_001123383,NM_001123384	BCOR	BCL6 corepressor	1.051050463	-1.003833645	3.023547	3.179719	2.825239	4.30540198	4.37369523	4.30016067	737	1035	903
401207	NM_001164478,NM_001164479	C5orf63	chromosome 5 open reading fran	1.750263494	-1.004164775	2.516059	4.977004	2.786142	3.10272366	3.83665412	3.09742492	298	697	365
440567	NM_001089591	UQCRHL	ubiquinol-cytochrome c reductase	1.084707628	-1.004253711	4.327576	4.677912	4.045989	1.70793493	1.79043244	1.70368302	89	129	109
195828	NM_153695	ZNF367	zinc finger protein 367	-1.169894556	-1.004364562	12.929297	11.063559	12.099062	5.57325719	5.35200352	5.56710603	1830	2090	2241
63906	NM_022078	GPATCH3	G-patch domain containing 3	1.197769738	-1.004553384	5.74698	6.890636	5.371703	3.6915876	3.93343361	3.68554025	468	749	573
51202	NM_016355,NM_201224	DDX47	DEAD-box helicase 47	-1.649240887	-1.004662247	100.004942	60.801365	93.39752	7.47096705	6.75442047	7.46429429	6926	5611	8479
55322	NM_018356,NR_134298	C5orf22	chromosome 5 open reading fran	-1.122396509	-1.004684576	4.180903	3.713516	3.842119	3.93685111	3.78172178	3.93054845	562	669	688
6134	NM_001256577,NM_001256580	RPL10	ribosomal protein L10	-1.021355379	-1.00470045	299.749311	294.624803	280.450491	9.35005934	9.31962149	9.34330428	25582	33466	31317
58155	NM_001300985,NM_001300986	PTBP2	polypyrimidine tract binding prote	2.528910177	-1.004734953	4.390625	10.959215	4.068661	3.97940737	5.2616952	3.97302418	580	1960	710
10992	NM_006842	SF3B2	splicing factor 3b subunit 2	1.132015425	-1.004837963	96.438721	109.294106	90.190956	8.091442	8.26972032	8.08450464	10669	16137	13059
100506637	NR_109996,NR_109997	PRKAR2A-AS1	PRKAR2A antisense RNA 1	1.230652931	-1.005100848	0.569684	0.703663	0.532494	1.55375576	1.75827411	1.549128	76	125	93
7994	NM_001305878,NM_006766	KAT6A	lysine acetyltransferase 6A	-1.196585883	-1.005126638	8.392647	7.134903	7.865155	6.24235022	5.98715744	6.23507035	2933	3275	3589
90826	NM_001304458,NM_001350141	PRMT9	protein arginine methyltransferas	1.011355103	-1.00521511	3.798533	3.824909	3.456076	3.78251604	3.79763131	3.77555686	501	677	613
9716	NM_014691	AQR	aquarius intron-binding spliceoso	-1.000124563	-1.005303788	13.088335	13.100109	12.233189	6.03886537	6.0386884	6.03134989	2542	3396	3110
57658	NM_001143682,NM_020898,NR	CALCOCO1	calcium binding and coiled-coil d	1.190342901	-1.005422755	9.728718	11.6282	9.346446	4.90008261	5.14373402	4.8925416	1133	1802	1386
84933	NM_032847	C8orf76	chromosome 8 open reading fran	1.023130082	-1.005528305	25.06901	25.672203	23.424395	5.07706542	5.10909111	5.06934731	1286	1758	1573
10049	NM_005494,NM_058246	DNAJB6	DnaJ heat shock protein family (H	-1.64994055	-1.005637547	67.257171	43.431647	71.225725	7.01142618	6.29624191	7.00337862	5026	4070	6147
387893	NM_001324504,NM_001324505	KMT5A	lysine methyltransferase 5A	-1.28744204	-1.005650722	26.972705	20.981276	25.218192	6.23114291	5.87213021	6.22312179	2910	3020	3559
80210	NM_001271466,NM_001291656	ARMC9	armadillo repeat containing 9	1.395992785	-1.005703792	2.266416	2.882121	4.573248	3.64828273	4.09665142	3.64073154	453	845	554
100506730	NR_037847	LOC100506730	uncharacterized LOC100506730	1.224210734	-1.005810189	1.927794	2.363979	1.804817	2.5075876	2.75234181	2.5006986	184	301	225
6648	NM_000636,NM_001024465,NM	SOD2	superoxide dismutase 2	1.000477882	-1.00584	9.886492	9.515068	8.987046	4.94133092	4.94199782	4.93320349	1167	1560	1427
27034	NM_014384	ACAD8	acyl-CoA dehydrogenase family r	-1.553756389	-1.005849137	11.788232	7.594337	11.010939	4.71519816	4.10945474	4.70710452	992	853	1213
23392	NM_001080398	KIAA0368	KIAA0368	1.043147123	-1.005986021	21.938641	22.913498	20.495817	7.31193353	7.3725016	7.30337747	6199	8640	7579
9877	NM_001319238,NM_001319239	ZC3H11A	zinc finger CCCH-type containing	-1.023112358	-1.006036329	77.161989	78.769795	74.754242	8.58173926	8.54886146	8.57307952	15003	19593	18342
22950	NM_018158	SLC4A1AP	solute carrier family 4 member 1	1.131729732	-1.006103723	15.592196	17.6691	14.564041	5.52698687	5.7018798	5.51839824	1771	2678	2165
100500872	NR_037473	MIR3911	microRNA 3911	-1.199655349	-1.006232566	2.022277	1.730654	1.995377	0.29776659	0.25173066	0.29607837	9	10	11
692148	NR_004387	SCARNA10	small Cajal body-specific RNA 10	-5.678101318	-1.006232566	0.670319	0.098654	0.618899	0.29776659	0.05398282	0.29607837	9	2	11
101929552	NR_136400	LOC101929552	uncharacterized LOC101929552	-1.980810008	-1.006232566	0.246592	0.129964	0.236778	0.29776659	0.15624221	0.29607837	9	6	11
101928489	NR_110889	LOC101928489	uncharacterized LOC101928489	1.493932334	-1.006271386	0.235917	0.352454	0.222779	0.54446555	0.75384785	0.54161868	18	36	22
101410535	NR_102697	LAMTOR5-AS1	LAMTOR5 antisense RNA 1	1.163260734	-1.006271386	0.843646	1.011523	0.820576	0.54446555	0.61708849	0.54161868	18	28	22
102465909	NR_107062_chr1,NR_107062_c	MIR6859-2	microRNA 6859-2	-1.263814783	-1.006271386	6.644193	5.427584	6.322013	0.54446555	0.44594451	0.54161868	18	19	22
102465910	NR_107063_chr1,NR_107063_c	MIR6859-3	microRNA 6859-3	-1.263814783	-1.006271386	6.656244	5.436581	6.333004	0.54446555	0.44594451	0.54161868	18	19	22
102466751	NR_106918_chr1,NR_106918_c	MIR6859-1	microRNA 6859-1	-1.263814783	-1.006271386	6.632164	5.418603	6.311041	0.54446555	0.44594451	0.54161868	18	19	22
54905	NM_017781	CYP2W1	cytochrome P450 family 2 subfar	-4.704113901	-1.006271386	0.201219	0.041696	0.185106	0.54446555	0.13134883	0.54161868	18	5	22
359821	NR_002208	MRPL42P5	mitochondrial ribosomal protein L	1.493932334	-1.006271386	0.673913	1.053945	0.634273	0.54446555	0.75384785	0.54161868	18	36	22
102723508	NR_110456,NR_145973,NR_14	KANTR	KDM5C adjacent transcript	-1.023341858	-1.006290979	0.192329	0.141299	0.171572	0.93882015	0.92294555	0.93448595	36	47	44
6095	NM_002943,NM_134260,NM_13	RORA	RAR related orphan receptor A	2.025485766	-1.006300821	0.17094	0.353003	0.158352	1.50279892	2.23798388	1.4969322	72	195	88
55349	NM_018397	CHDH	choline dehydrogenase	-1.258812019	-1.006307967	1.841202	1.464259	1.71593	2.93507482	2.65089709	2.92718897	261	277	319
10793	NM_021148,NR_003099	ZNF273	zinc finger protein 273	-1.766201813	-1.006308143	1.657086	0.937705	1.561044	3.01906833	2.32837375	3.0111152	279	211	341
5709	NM_002809	PSMD3	proteasome 26S subunit, non-AT	1.074894954	-1.006370745	109.191513	117.502365	101.970253	7.8674378	7.97120413	7.85831515	9129	13111	11157
11132	NM_023083,NM_023085	CAPN10	calpain 10	1.080761444	-1.00647654	11.012363	11.759755	10.273165	4.85762016	4.96595521	4.84862794	1099	1587	1343
4040	NM_002336	LRP6	LDL receptor related protein 6	1.103053374	-1.006501933	4.955003	5.471511	4.62572	5.63371647	5.77250923	5.62455491	1910	2815	2334
8462	NM_001177716,NM_001177718	KLF11	Kruppel like factor 11	1.120130844	-1.006611237	1.955039	2.196616	1.8267	3.11973375	3.26553914	3.11132089	302	452	369
80339	NM_025225	PNPLA3	patatin like phospholipase domai	1.153540198	-1.006651909	2.479791	2.858976	2.313771	2.95890107	3.14012691	2.95056637	266	410	325
84316	NM_001320924,NM_001320925	NAA38	N(alpha)-acetyltransferase 38, Na	1.248410311	-1.006697414	20.773794	26.526352	19.692237	4.25047234	4.55544439	4.24134834	708	1181	865
84809	NR_026752	CROCCP2	ciliary rootlet coiled-coil, rootletin	-1.009113711	-1.006790997	28.209167	27.994036	26.332224	5.62926888	5.61644502	5.61970202	1904	2521	2326
5884	NM_001278622,NM_002873,NM	RAD17	RAD17 checkpoint clamp loader	-1.125964592	-1.006795583	16.300309	14.223453	15.251883	5.61584323	5.44837277	5.60627178	1886	2238	2304
64766	NM_001256121,NM_022753,NR	S100PBP	S100P binding protein	1.13475397	-1.007135987	11.532128	13.093948	10.671386	5.61509368	5.79398308	5.60504462	1885	2858	2302
92906	NM_001142650,NM_138394	HNRNPLL	heterogeneous nuclear ribonucle	1.063560032	-1.007388283	13.050517	13.820876	12.126382	5.23956149	5.32618529	5.22922293	1444	2052	1763
7709	NM_001242884,NM_001287603	ZBTB17	zinc finger and BTB domain cont	1.072119025	-1.007469444	13.552712	14.591607	12.669699	5.21800288	5.31586533	5.20755546	1422	2037	1736
57634	NM_015409	EP400	E1A binding protein p400	-1.427607799	-1.007610175	13.023427	9.13318	12.146156	7.29635493	6.78666387	7.28548698	6132	5739	7485
22882	NM_014943	ZHX2	zinc fingers and homeoboxes 2	1.393595676	-1.007686081	2.392527	3.337839	2.232556	3.48064928	3.92259242	3.47059318	399	743	487
10307	NM_006051,NM_133172,NM_13	APBB3	amyloid beta precursor protein bi	1.315814203	-1.007687169	9.673742	12.893415	9.118647	4.41453771	4.79420929	4.40400834	798	1403	974
27124	NM_001002837,NM_001284285	INPP5J	inositol polyphosphate-5-phospha	1.490562346	-1.007746541	0.941493	1.424546	0.886158	2.0823043	2.541776	2.07380058	127	253	155
1786	NM_001130823,NM_001318730	DNMT1	DNA methyltransferase 1	1.028671732	-1.007793706	43.713452	45.478926	40.756649	7.85002379	7.89063263	7.83887203	9019	12396	11007
56852	NM_020165	RAD18	RAD18, E3 ubiquitin protein ligas	1.123944012	-1.007826293	5.229376	5.887276	4.877277	4.91842592	5.08173995	4.90755118	1148	1724	1401
2035	NM_001166005,NM_001166006	EPB41	erythrocyte membrane protein ba	-1.321083065	-1.00807072	5.121069	3.987576	4.98769	4.91477587	4.52828878	4.90356387	1145	1158	1397
9774	NM_001077440,NM_001077441	BCLAF1	BCL2 associated transcription fac	1.081316584	-1.008072909	22.04509	23.891747	20.600692	7.28508979	7.39718488	7.27356427	6084	8790	7423
121536	NM_001114176,NM_001267043	AEBP2	AE binding protein 2	1.195943941	-1.008083181	8.244639	9.747283	7.470344	5.43059347	5.68326725	5.41924838	1654	2643	2018
55449	NR_023921,NR_023922,NR_02	DHRS4-AS1	DHRS4 antisense RNA 1	-1.304620132	-1.008241849	5.840726	4.594635	5.662063	4.16292023	3.80355245	4.15174023	664	680	810
2107	NM_001256302,NM_001282185	ETF1	eukaryotic translation termination	1.219960801	-1.008251635	44.739284	54.490565	41.756432	7.36260538	7.64786307	7.35082176	6422	10468	7834
51239	NM_016466	ANKRD39	ankyrin repeat domain 39	1.067778229	-1.00828214	6.731125	7.206942	6.291043	2.78853894	2.86987209	2.77836293	232	331	283
115825	NM_052950	WDFY2	WD repeat and FYVE domain co	-1.306005016	-1.00841157	2.091221	1.605121	1.947291	3.13236099	2.79655578	3.12165574	305	312	372
9919	NM_001276418,NM_014866	SEC16A	SEC16 homolog A, endoplasmic	1.015407611	-1.008422034	25.279867	25.687637	23.580805	7.80275131	7.82471251	7.79070602	8727	11840	10644
79026	NM_001346445,NM_001346446	AHNAK	AHNAK nucleoprotein	1.067927637	-1.008487293	63.927428	68.682237	59.588412	10.10839243	10.20312342	10.09621059	43300	61784	52808
1760	NM_001081560,NM_001081562	DMPK	dystrophia myotonica protein kina	1.107959765	-1.008521807	21.109717	23.812914	19.874133	5.9841867	6.12987583	5.97213807	2446	3621	2983
26779	NR_002584	SNORA69	small nucleolar RNA, H/ACA box	1.095021906	-1.008523434	7.933546	8.770151	7.517339	1.03161781	1.10017522	1.02535867	41	60	50
9491	NM_001323407,NM_001323408	PSMF1	proteasome inhibitor subunit 1	1.723439707	-1.008735632	12.290765	20.724126	11.104064	6.54627901	7.3250933	6.53386535	3630	8359	4426
11339	NM_001317860,NM_007280	OIP5	Opa interacting protein 5	-1.062531993	-1.008746376	12.848917	12.109498	11.93412	4.02977152	3.94776294	4.01797815	602	757	734
100529209	NR_037717	RNASEK-C17orf49	RNASEK-C17orf49 readthrough	-1.597565397	-1.00880968	0.99819	0.621199	0.92857	1.51570785	1.11294744	1.50747923	73	61	89
116985	NM_001040118,NM_001135190	ARAP1	ArfGAP with RhoGAP domain, ar	1.410394959	-1.008882861	18.973066	26.707057	17.738426	6.53483138	7.02640839	6.52221051	3601	6786	4390
9837	NM_021067,NR_134574	GINS1	GINS complex subunit 1	1.167263344	-1.009064638	17.977957	20.943698	16.935658	5.89560815	6.11527096	5.88280858	2298	3584	2801
101928111	NR_135575,NR_135576,NR_13	LOC101928111	uncharacterized LOC101928111	2.054667696	-1.009145341	1.54742	4.102672	1.413545	0.86002952	1.42093184	0.85412573	32	88	39
527	NM_001198569,NM_001694	ATP6V0C	ATPase H+ transporting V0 subu	1.231099072	-1.009146508	166.952147	207.292869	156.286931	7.58616924	7.88470993	7.57310207	7505	12345	9147
101060691	NR_120611,NR_120612,NR_12	NUTM2B-AS1	NUTM2B antisense RNA 1	-1.374351883	-1.009295599	6.164329	4.414989	5.331643	3.57924973	3.16487058	3.56701931	430	418	524
4131	NM_001324255,NM_005909	MAP1B	microtubule associated protein 1B	2.323669562	-1.00934207	0.328472	0.765888	0.305875	2.27683528	3.31335619	2.26619062	151	469	184
26515	NM_012192	TIMM10B	translocase of inner mitochondria	-1.252779745	-1.009474312	11.203681	8.955946	10.432069	5.00107303	4.68725264	4.98789438	1218	1299	1484
26298	NM_001206615,NM_001206616	EHF	ETS homologous factor	-1.498100896	-1.009681904	55.978445	37.458332	52.127075	8.21827174	7.6375413	8.20441766	11653	10393	14195
10243	NM_001024218,NM_020806	GPHN	gephyrin	-1.077288927	-1.009694858	5.770507	5.356246	5.3684	4.65375261	4.55075824	4.64038721	949	1177	1156
10963	NM_001282652,NM_001282653	STIP1	stress induced phosphoprotein 1	1.5183707	-1.009711712	112.976009	171.402467	103.842377	7.92466719	8.52516826	7.91078125	9500	19273	11572
153339	NM_174909	TMEM167A	transmembrane protein 167A	-1.220138183	-1.009719805	32.396336	26.582348	30.150539	7.18687058	6.9019982	7.17301154	5681	6221	6920
6390	NM_003000	SDHB	succinate dehydrogenase comple	-1.122299137	-1.010044391	101.686363	90.705528	94.613457	6.83758885	6.67266925	6.82329648	4451	5299	5420
55672	NM_017940	NBPF1	NBPF member 1	-1.022967077	-1.01023422	6.89543	6.748181	6.415481	5.35539329	5.32344051	5.34106306	1568	2048	1909
55227	NM_018214	LRRC1	leucine rich repeat containing 1	-1.279113872	-1.010239955	9.413882	7.368581	8.759444	4.91477587	4.57288311	4.90056614	1145	1196	1394
105371907	NR_136503	LOC105371907	uncharacterized LOC105371907	-1.097033567	-1.010253495	0.844898	0.778801	0.786711	0.66524311	0.61708849	0.65979727	23	28	28
2348	NM_000802,NM_016724,NM_01	FOLR1	folate receptor 1	-1.658580037	-1.010278681	1.246261	0.634658	1.150712	1.11880553	0.77006602	1.11084509	46	37	56
80821	NM_001160147,NM_001160148	DDHD1	DDHD domain containing 1	1.19520152	-1.010352011	1.469502	1.760029	1.369884	4.28669603	4.53185994	4.27260082	727	1161	885
11316	NM_001330469,NM_007263,NM	COPE	coatomer protein complex subun	-1.115898592	-1.010522745	104.345201	93.232455	96.143289	6.83694612	6.68019807	6.82197673	4449	5327	5415

84330	NM_001146175,NM_032370	ZNF414	zinc finger protein 414	1.266328961	-1.010618001	6.910587	8.957713	6.356272	3.27584855	3.58491601	3.26218696	341	577	415
5708	NM_001278708,NM_001278709	PSMD2	proteasome 26S subunit, non-AT	1.284815989	-1.010668198	138.599822	178.112059	128.52156	8.68784408	9.04863221	8.67257185	16151	27726	19655
80262	NM_001320540,NM_001320541	C16orf70	chromosome 16 open reading fra	-1.078463408	-1.01086384	12.405746	11.499919	11.548297	5.14718204	5.04138697	5.13203431	1352	1675	1645
30008	NM_016938,NR_037718	EFEMP2	EGF containing fibulin like extrac	1.498683695	-1.010866349	4.988406	7.493211	4.631086	3.42699388	3.96547676	3.4128543	383	767	466
201514	NM_001318002,NM_173548,NR	ZNF584	zinc finger protein 584	1.293167715	-1.010927574	6.518109	8.49442	6.103545	3.97240126	4.3223799	3.95772276	577	997	702
26000	NM_015527	TBC1D10B	TBC1 domain family member 10E	1.138966548	-1.01106167	14.768617	16.843422	13.730014	5.59394673	5.77803304	5.57840516	1857	2826	2259
81621	NM_001319303,NM_030929,NR	KAZALD1	Kazal type serine peptidase inhib	-6.510914882	-1.011062515	6.634616	1.307239	6.463817	3.8375314	1.60397149	3.82277197	522	107	635
223	NM_000696	ALDH9A1	aldehyde dehydrogenase 9 family	1.019673292	-1.011138746	21.704069	22.159988	20.177858	5.75094273	5.77853415	5.73525928	2075	2827	2524
84312	NM_032352	BRMS1L	breast cancer metastasis-suppre	1.320504682	-1.011229283	4.727962	6.246817	4.388995	3.6887407	4.06249189	3.67388275	467	824	568
23198	NM_014614	PSME4	proteasome activator subunit 4	1.196552834	-1.011463211	13.546779	16.22723	12.585443	6.56425305	6.82063753	6.54798346	3676	5877	4470
64788	NM_001352017,NM_001352018	LMF1	lipase maturation factor 1	-1.359889795	-1.011632774	5.394989	3.976028	5.005529	3.95592014	3.5454167	3.94031242	570	560	693
2177	NM_001018115,NM_001319984	FANCD2	Fanconi anemia complementatio	1.058051749	-1.011803193	6.914467	7.331553	6.424185	5.1687957	5.24800972	5.15233896	1373	1941	1669
54477	NM_001143821,NM_001190860	PLEKHA5	pleckstrin homology domain cont	-1.338370741	-1.011817542	7.394476	5.31249	6.566615	4.94372095	4.53897585	4.92732408	1169	1167	1421
79724	NM_024671	ZNF768	zinc finger protein 768	1.105741662	-1.011839165	29.511225	32.669476	27.411829	6.07746806	6.22044356	6.06074026	2612	3859	3175
22902	NM_001037442,NM_001130709	RUFY3	RUN and FYVE domain containir	-1.152916193	-1.011843	11.828543	10.505001	12.681557	5.69529401	5.49424621	5.67863733	1995	2312	2425
7784	NM_001110354,NM_007155	ZP3	zona pellucida glycoprotein 3	-1.010399138	-1.011906652	11.774256	11.235279	10.224723	4.04320744	4.02918937	4.02716981	608	804	739
100507424	NR_038933	LOC100507424	uncharacterized LOC100507424	1.344820136	-1.012116847	2.345019	3.151539	2.174256	1.59082596	1.89025622	1.57922529	79	142	96
191	NM_000687,NM_001161766,NM	AHCY	adenosylhomocysteinase	1.119248087	-1.012166026	89.681928	99.601054	82.768774	7.66342415	7.82519768	7.64606452	7920	11844	9624
23528	NM_001281293,NM_001281294	ZNF281	zinc finger protein 281	1.113104829	-1.012172734	7.135149	7.951204	6.627662	5.12839479	5.2787986	5.11143982	1334	1984	1621
5871	NM_001307990,NM_004579	MAP4K2	mitogen-activated protein kinase	1.243380411	-1.012174422	6.552121	8.166627	6.090886	4.33483599	4.63508	4.3182457	753	1251	915
153579	NM_001308245,NM_152547	BTNL9	butyrophilin like 9	-1.010226967	-1.012232703	0.694552	0.683849	0.642565	1.75224037	1.74192207	1.73991364	93	123	113
256281	NM_001318380,NM_177533	NUDT14	nudix hydrolase 14	-1.223760761	-1.012233457	21.642494	17.777412	19.990044	4.29233333	4.01734947	4.27568916	730	797	887
3750	NM_004979	KCND1	potassium voltage-gated channel	-1.591979298	-1.012590357	1.136022	0.712394	1.05434	2.63728034	2.09724475	2.62213795	205	172	249
79171	NM_001319113,NM_024321	RBM42	RNA binding motif protein 42	1.005840431	-1.012659333	69.444198	70.226431	64.46207	6.8330837	6.84141188	6.8150945	4437	5963	5389
26219	NM_001004452	OR1J4	olfactory receptor family 1 subfan	1.227829369	-1.012784193	0.38056	0.47839	0.352953	0.43999062	0.52452456	0.43515728	14	23	17
100129827	NR_034093,NR_034094,NR_04(	MRVI1-AS1	MRVI1 antisense RNA 1	-3.664933469	-1.012784193	0.551354	0.108898	0.484419	0.43999062	0.13134883	0.43515728	14	5	17
101927354	NR_108073	LOC101927354	uncharacterized LOC101927354	-1.332724173	-1.012784193	0.462564	0.335952	0.420253	0.43999062	0.3412894	0.43515728	14	14	17
100131733	NR_038996	USP30-AS1	USP30 antisense RNA 1	-2.061547509	-1.012784193	0.348012	0.162126	0.329435	0.43999062	0.2284445	0.43515728	14	9	17
84328	NM_001316973,NM_001316974	LZIC	leucine zipper and CTNNBIP1 dc	-1.027391402	-1.01278805	6.711789	6.579161	6.662446	4.98955224	4.95180631	4.97179891	1208	1571	1467
115908	NM_001256099,NM_138455	CTHRC1	collagen triple helix repeat contai	2.959518785	-1.012835831	0.572971	1.684167	0.524181	0.77668602	1.63971536	0.76902392	28	111	34
728963	NR_026768	RPS15AP10	ribosomal protein S15a pseudoge	-1.622712497	-1.012835831	0.782785	0.49033	0.744596	0.77668602	0.52452456	0.76902392	28	23	34
64282	NM_001040284,NM_001040285	PAPD5	poly(A) RNA polymerase D5, nor	1.146079277	-1.012886054	2.582471	2.959026	2.396771	4.41453771	4.60261969	4.39693481	798	1222	969
9603	NM_004289	NFE2L3	nuclear factor, erythroid 2 like 3	-1.415958885	-1.01295487	87.662628	61.977989	81.325508	8.31132516	7.81142856	8.29281393	12432	11731	15095
4171	NM_004526,NR_073375	MCM2	minichromosome maintenance co	1.971607381	-1.012969087	27.377357	54.055724	25.406549	6.55451234	7.52632166	6.53612067	3651	9618	4433
9113	NM_001270519,NM_001350339	LATS1	large tumor suppressor kinase 1	1.128856857	-1.013061194	4.490966	4.880856	3.721087	4.75792697	4.92670685	4.73989992	1023	1543	1242
79087	NM_024105	ALG12	ALG12, alpha-1,6-mannosyltrans	1.145351169	-1.013076698	10.346274	11.86743	9.597659	4.63907992	4.82752356	4.62109129	939	1437	1140
11333	NM_014891	PDAP1	PDGFA associated protein 1	-1.418032866	-1.013085728	46.844246	33.069061	43.456394	6.94914936	6.4501176	6.93054544	4812	4534	5842
5469	NM_004774	MED1	mediator complex subunit 1	1.144891364	-1.013182651	10.367672	11.88475	9.617404	6.379754	6.57277645	6.36108745	3230	4941	3921
641339	NM_001039891,NM_001146291	ZNF674	zinc finger protein 674	1.111767009	-1.013223724	1.102254	1.228035	1.020271	2.43469539	2.5605457	2.41925694	173	257	210
4067	NM_001111097,NM_002350	LYN	LYN proto-oncogene, Src family t	-1.479546541	-1.013238984	3.190288	2.147852	2.968053	3.79315817	3.27693362	3.77555686	505	456	613
154807	NM_001284342,NM_173517	VKORC1L1	vitamin K epoxide reductase com	-1.80377676	-1.013242788	9.436216	5.216938	8.728873	5.82021839	4.98952124	5.80157562	2179	1614	2645
8812	NM_001099402	CCNK	cyclin K	1.131301055	-1.013334474	32.123268	36.381737	29.78339	6.36645416	6.54241176	6.34757619	3200	4837	3884
27335	NM_001300992,NM_001308393	EIF3K	eukaryotic translation initiation fa	-1.245840903	-1.01335663	160.866632	129.333017	148.992129	7.13133848	6.81673931	7.11233353	5465	5861	6633
7041	NM_001042454,NM_001164719	TGFB1I1	transforming growth factor beta 1	-1.009785102	-1.013610299	4.751068	4.704317	4.402917	3.22566415	3.21312006	3.20825259	328	434	398
283742	NM_173611	FAM98B	family with sequence similarity 98	1.074379436	-1.013676708	12.15508	13.073556	11.266118	5.72616608	5.82778681	5.70694011	2039	2927	2474
9733	NM_014706	SART3	squamous cell carcinoma antiger	-1.092396403	-1.013709767	19.53134	17.898466	18.108168	6.3942436	6.26832673	6.3748334	3263	3991	3959
171425	NM_206808,NR_104592	CLYBL	citrate lyase beta like	1.300031863	-1.01383979	1.107828	1.389081	1.022574	1.35261029	1.59489542	1.34055586	61	106	74
158399	NM_001007169,NM_133464	ZNF483	zinc finger protein 483	-1.131730948	-1.01383979	0.447848	0.376417	0.401839	1.35261029	1.24644988	1.34055586	61	72	74
9883	NM_001257190,NM_172020	POM121	POM121 transmembrane nucleop	-1.356443545	-1.01387577	11.855768	8.693805	11.01707	6.03438714	5.60236135	6.01481077	2534	2496	3074
26071	NM_001078172,NM_001134321	RTL8A	retrotransposon Gag like 8A	1.117365493	-1.014048567	40.792635	44.423784	34.961333	5.28267998	5.43889362	5.26307232	1489	2223	1806
161497	NM_153700	STRC	stereocilin	1.892939181	-1.014123006	0.220885	0.421983	0.205032	1.13562816	1.70865125	1.12461302	47	119	57
3425	NM_000203,NR_110313	IDUA	iduronidase, alpha-L-	1.11350111	-1.014173449	24.638451	27.47553	22.835476	5.75910795	5.91150049	5.73917997	2087	3105	2531
79815	NM_001321635,NM_001321636	NIPAL2	NIPA like domain containing 2	-1.103163799	-1.014391337	4.398216	3.908591	3.993736	4.33483599	4.20052559	4.31524723	753	912	913
84619	NM_001083113,NM_001195653	ZGPAT	zinc finger CCCH-type and G-pat	-1.017959917	-1.014403144	35.60816	35.095136	33.144315	6.06982971	6.04453358	6.04950701	2598	3410	3150
7167	NM_000365,NM_001159287,NM	TPI1	triosephosphate isomerase 1	1.249234656	-1.014431111	415.768505	519.892704	366.061142	9.09955264	9.42007378	9.07891958	21498	35883	26065
5092	NM_000281,NM_001289797,NM	PCBD1	pterin-4 alpha-carbinolamine deh	-1.790457668	-1.014453849	65.575969	36.670579	60.688254	6.02313036	5.2001842	6.00274682	2514	1876	3048
388569	NM_207395	ZNF324B	zinc finger protein 324B	1.313916733	-1.014805767	1.887477	2.491985	1.753611	2.70647097	3.04669644	2.68852679	217	381	263
9117	NM_001201572,NM_001201584	SEC22C	SEC22 homolog C, vesicle traffic	1.062130812	-1.014883682	6.044105	6.769517	7.712086	4.82817011	4.91216578	4.80760927	1076	1527	1304
55317	NM_001204446,NM_001204447	AP5S1	adaptor related protein complex 5	1.461930257	-1.014888923	5.41546	7.943407	5.059606	3.44059597	3.94597954	3.42124554	387	756	469
79631	NM_001040610,NM_001322844	EFL1	elongation factor like GTPase 1	-1.030010509	-1.014924186	7.048502	6.857456	6.567274	4.72078319	4.67976064	4.70022518	996	1292	1207
10555	NM_001012727,NM_006412	AGPAT2	1-acylglycerol-3-phosphate O-acy	1.104841461	-1.015018634	105.764889	117.336314	97.667232	7.31678196	7.45976472	7.29541125	6220	9182	7537
51652	NM_001005753,NM_001193517	CHMP3	charged multivesicular body prote	1.043185092	-1.015079076	24.02041	25.127127	22.192624	6.2335867	6.29379011	6.21228282	2915	4063	3532
554202	NR_027054	MIR31HG	MIR31 host gene	1.415692155	-1.015139757	4.826163	6.845632	4.470797	3.47405048	3.9370293	3.45433079	397	751	481
2710	NM_000167,NM_001128127,NM	GK	glycerol kinase	-1.215169117	-1.015474094	0.919139	0.758719	0.865687	2.35073722	2.12902957	2.3329413	161	177	195
80149	NM_001323550,NM_001323551	ZC3H12A	zinc finger CCCH-type containing	1.575089505	-1.015484124	18.521257	29.14105	16.999478	5.64990801	6.29484139	5.62818389	1932	4066	2340
6668	NM_003110	SP2	Sp2 transcription factor	1.179640386	-1.015857776	10.648223	12.57711	9.849711	5.05623208	5.28797897	5.03421913	1267	1997	1534
10888	NM_001330345,NM_016540	GPR83	G protein-coupled receptor 83	1.200868564	-1.015985584	0.229745	0.275069	0.21163	0.97665845	1.11294744	0.96541723	38	61	46
55529	NM_018710	TMEM55A	transmembrane protein 55A	-1.098626436	-1.016155521	3.771834	3.441952	3.486784	3.29843463	3.17708507	3.27767373	347	422	420
1912	NM_001330488,NM_004427,NM	PHC2	polyhomeotic homolog 2	1.552815718	-1.016203564	41.997537	65.099474	38.934324	6.73610712	7.36618088	6.71313634	4146	8602	5018
6760	NM_001007559,NM_001308201	SS18	SS18, nBAF chromatin remodelir	1.046159003	-1.016218637	58.894868	61.772386	54.448862	7.60931277	7.67408977	7.58622142	7627	10661	9231
55677	NM_017969	IWS1	IWS1, SUPT6H interacting protei	1.156017271	-1.016314496	35.590706	41.191452	32.910202	6.71387535	6.92118734	6.690752	4082	6305	4940
283349	NM_178169,NR_040718	RASSF3	Ras association domain family m	-2.115437263	-1.01633141	21.250279	9.965041	19.561808	6.1823895	5.1233639	6.15934204	2812	1776	3403
729234	NR_003698	FAHD2CP	fumarylacetoacetate hydrolase do	1.182331307	-1.016456694	3.048898	3.613972	2.832893	1.82664439	2.0043309	1.80975447	100	158	121
27153	NM_015694	ZNF777	zinc finger protein 777	-1.195200214	-1.016518396	4.621428	3.869885	4.272965	3.89052565	3.65208684	3.8684907	543	607	657
55330	NM_018366	BLOC1S4	biogenesis of lysosomal organell	1.103271129	-1.016649061	12.083418	13.352038	11.17409	4.26392396	4.39868427	4.24134834	715	1054	865
81562	NM_001142292,NM_001322346	LMAN2L	lectin, mannose binding 2 like	1.841468602	-1.016722644	13.210618	24.243596	12.221482	4.99877622	5.85892233	4.97560227	1216	2992	1471
3939	NM_001135239,NM_001165414	LDHA	lactate dehydrogenase A	-1.676621783	-1.016768163	854.285092	519.493434	788.952583	10.8395975	10.09457136	10.8156199	71905	57302	86980
117584	NM_001017368,NR_037713	RFFL	ring finger and FYVE like domain	-1.025495432	-1.016895745	11.911411	11.622404	11.038038	5.62257167	5.58699495	5.59889315	1895	2469	2292
7766	NM_013361	ZNF223	zinc finger protein 223	-1.260151958	-1.016954424	4.123748	3.278423	3.813673	3.42357323	3.1244438	3.40158951	382	405	462
27005	NM_001014443,NM_001319847	USP21	ubiquitin specific peptidase 21	-1.011175447	-1.016979444	16.505061	16.457229	15.122253	5.24247649	5.22686782	5.21883106	1447	1912	1750
222229	NM_001317721,NM_152892	LRWD1	leucine rich repeats and WD repe	-1.141756814	-1.01712893	9.786904	8.562851	9.027595	4.49496323	4.31272471	4.47155306	846	990	1023
9529	NM_001015048,NM_001015049	BAG5	BCL2 associated athanogene 5	1.084379237	-1.017200428	12.854828	13.779903	11.729046	5.94892933	6.06398526	5.92472586	2386	3457	2885
51000	NM_001142541,NM_015948,NR	SLC35B3	solute carrier family 35 member E	1.010221978	-1.01731456	8.08942	8.577758	7.784092	4.21331566	4.22720295	4.18989199	689	930	833
5860	NM_000320,NM_001306140	QDPR	quinoid dihydropteridine reductas	1.043724454	-1.017523668	22.132085	23.166552	20.41964	5.19009033	5.25017924	5.16571834	1394	1944	1685
121665	NM_139015	SPPL3	signal peptide peptidase like 3	-1.043545555	-1.017588162	13.871069	13.310996	12.81133	5.82735481	5.76696418	5.80264655	2190	2804	2647
440926	NR_002315	H3F3AP4	H3 histone, family 3A, pseudoger	-1.091276298	-1.017618488	309.720703	284.12973	286.013275	8.30029334	8.17469345	8.27517695	12337	15105	14911
10233	NM_001135217,NM_006992,NM	LRRC23	leucine rich repeat containing 23	-1.362583787	-1.017756567	5.177202	3.701534	4.737285	3.14071816	2.75234181	3.11821901	307	301	371
55693	NM_018039	KDM4D	lysine demethylase 4D	1.1225151	-1.017856257	0.839262	0.947718	0.777421	1.78459878	1.90501868	1.76650173	96	144	116
80004	NM_024939	ESRP2	epithelial splicing regulatory prote	-1.007868421	-1.017929692	30.358494	30.155758	28.030848	6.90537089	6.89415802	6.87994812	4667	6187	5639
5093	NM_006196	PCBP1	poly(rC) binding protein 1	1.127490281	-1.018187212	145.635788	164.378906	134.405045	7.96081016	8.13327201	7.93491232	9742	14676	11768
8501	NM_001198810,NM_003627	SLC43A1	solute carrier family 43 member 1	-1.482191341	-1.018204543	1.034455	0.699867	0.961559	1.83696709	1.45142181	1.81825156	101	91	122
9694	NM_001329493,NM_001329494	EMC2	ER membrane protein complex s	-1.364857597	-1.018376217	28.111342	20.850329	26.067119	6.6108664	6.16747684	6.58486621	3798	3718	4587
79158	NM_024312	GNPTAB	N-acetylglucosamine-1-phosphat	-1.14856751	-1.018430817	6.173615	5.38125	5.696215	5.12313239	4.92941706	5.09754499	1329	1546	1605
8896	NM_003910	BUD31	BUD31 homolog	-1.333416807	-1.018459097	49.884476	37.445526	46.026596	5.69103253	5.28516046	5.66515841	1989	1993	2402
6376	NM_001304392,NM_002996	CX3CL1	C-X3-C motif chemokine ligand 1	-1.767618176	-1.018500304	0.425284	0.242683	0.388202	1.23264156	0.81765451	1.21747621	53	40	64
113444	NM_001164824,NM_001164825	SMIM12	small integral membrane protein	-1.063094541	-1.018524306	5.100719	4.804068	4.747431	4.90499701	4.81975384	4.87940578	1137	1429	1373
84747	NM_001080533	UNC119B	unc-119 lipid binding chaperone I	1.093812579	-1.018538499	7.552499	8.272923	6.967116	5.06174377	5.18740997	5.03604153	1272	1859	1536
91289	NM_033200	LMF2	lipase maturation factor 2	1.214794082	-1.018609269	27.508131	33.451981	25.374285	6.14185247	6.41895706	6.11563079	2733	4436	3300
26061	NM_001284413,NM_001284415	HACL1	2-hydroxyacyl-CoA lyase 1	-1.065880244	-1.018792082	5.738187	5.419054	5.229417	3.77179482	3.68667221	3.74691298	497	623	600

170384	NM_001284194,NM_173540	FUT11	fucosyltransferase 11	1.954449097	-1.018807642	1.364014	2.730576	1.281139	1.93632435	2.70672986	1.91649468	111	290	134
7546	NM_007129	ZIC2	Zic family member 2	1.179877511	-1.018817314	1.933903	2.283282	1.78133	2.73434891	2.93964205	2.71151569	222	350	268
27095	NM_001270894,NM_001270895	TRAPPC3	trafficking protein particle comple	-1.887060743	-1.018856675	68.721709	36.781575	63.520141	6.49993314	5.59782538	6.47328171	3514	2488	4242
79813	NM_001145527,NM_024757	EHMT1	euchromatic histone lysine methy	-1.120227207	-1.019185131	25.793423	23.054513	24.839093	6.95626902	6.79386996	6.92907512	4836	5768	5836
51291	NM_001288998,NM_001288999	GMIP	GEM interacting protein	1.098926357	-1.019326892	5.392793	5.939648	4.979632	4.28481203	4.41424668	4.25862092	726	1066	876
147650	NM_001316972,NM_001316994	SPACA6	sperm acrosome associated 6	-1.055502257	-1.019382517	6.940646	7.172451	6.283149	3.75011035	3.67810329	3.72448562	489	619	590
9775	NM_014740	EIF4A3	eukaryotic translation initiation fa	1.150771061	-1.019486239	115.031754	132.529037	106.029999	7.60893635	7.81057126	7.58123763	7625	11724	9199
51194	NM_001134779,NM_016338	IPO11	importin 11	1.088900555	-1.019560705	7.331625	7.988953	6.758811	5.01591383	5.13515097	4.9888356	1231	1791	1485
1396	NM_001311	CRIP1	cysteine rich protein 1	-1.459375572	-1.01957009	351.194275	240.880402	323.698395	7.36148853	6.82015082	7.33369876	6417	5875	7741
201799	NM_152680	TMEM154	transmembrane protein 154	1.035787991	-1.01958365	2.831005	2.941414	2.612315	3.30588543	3.35157866	3.28075123	349	483	421
26262	NM_001006616,NM_012171,NM	TSPAN17	tetraspanin 17	-1.031533481	-1.019675969	20.651807	20.074395	18.992257	5.72129859	5.67736765	5.69372425	2032	2632	2451
84545	NM_001308396,NM_032112,NM	MRPL43	mitochondrial ribosomal protein L	-1.621515719	-1.019758332	55.812679	34.82812	52.165455	5.87258686	5.19042586	5.84484461	2261	1863	2727
29801	NM_001185024,NM_013373	ZDHHC8	zinc finger DHHC-type containing	1.219414515	-1.019785299	9.107843	11.069086	8.236396	5.48000681	5.76038336	5.45237894	1713	2791	2066
26276	NM_001289148,NM_001289149	VPS33B	VPS33B, late endosome and lyso	-1.362483456	-1.019792851	7.783625	5.708111	7.158409	4.46032739	4.03758735	4.43334413	825	809	995
92482	NM_001195304,NM_001195305	BBIP1	BBSome interacting protein 1	1.081294249	-1.019865306	3.99475	4.28052	3.68692	3.25290323	3.35427048	3.22751932	335	484	404
22878	NM_014939	TRAPPC8	trafficking protein particle comple	-1.208982803	-1.020057522	5.52769	4.577949	5.091604	5.11573264	4.85058478	5.08791408	1322	1461	1594
146880	NR_027487	ARHGAP27P1	Rho GTPase activating protein 27	-1.776829844	-1.020342257	24.546944	13.830473	22.606377	6.12299522	5.30963773	6.09436188	2697	2028	3251
9873	NM_014824	FCHSD2	FCH and double SH3 domains 2	-1.123615467	-1.02058083	3.635602	3.235873	3.346511	4.07843661	3.92077761	4.05079762	624	742	752
102606463	NR_110124,NR_110132	LINC01152	long intergenic non-protein codin	-1.143069062	-1.020817587	0.60593	0.526087	0.55809	1.63881618	1.51053416	1.61867597	83	97	100
84464	NM_032444	SLX4	SLX4 structure-specific endonucl	-1.094557638	-1.020882935	6.742875	6.167529	6.205211	5.61509368	5.4875188	5.58588904	1885	2301	2271
140809	NM_080725	SRXN1	sulfiredoxin 1	1.206887304	-1.020920261	23.85165	28.821394	21.954792	5.98766551	6.25506445	5.95826952	2452	3954	2954
60560	NM_001321881,NM_001321882	NAA35	N(alpha)-acetyltransferase 35, Na	1.07813757	-1.021196119	15.36144	16.238261	11.918129	5.87822053	5.98498794	5.84847888	2270	3270	2734
79989	NM_001144920,NM_001144923	TTC26	tetratricopeptide repeat domain 2	-1.043747414	-1.02137464	2.935044	2.494776	2.171334	3.42357323	3.36765473	3.39592396	382	489	460
89932	NM_173462	PAPLN	papilin, proteoglycan like sulfated	1.282123638	-1.02142061	1.733193	2.227388	1.596206	3.44059597	3.76967342	3.4128543	387	663	466
100861548	NR_046507	PINK1-AS	PINK1 antisense RNA	1.294227863	-1.021597789	1.500105	1.942306	1.378834	2.90595399	3.23373217	2.8792661	255	441	307
102157402	NM_001015891,NM_016283	AK6	adenylate kinase 6	-1.272640048	-1.021748918	50.959318	38.138723	46.572053	5.60002041	5.26026072	5.56962461	1865	1958	2245
55787	NM_001168683,NM_018360	TXLNG	taxilin gamma	-1.207157356	-1.021832095	20.82954	17.319878	19.160729	6.4946452	6.22633294	6.46383529	3501	3875	4214
134266	NM_152407	GRPEL2	GrpE like 2, mitochondrial	-1.019352218	-1.02221392	12.322532	12.104327	11.327849	5.651371	5.62427223	5.62030952	1934	2535	2327
134957	NM_001127715,NM_139244	STXBP5	syntaxin binding protein 5	1.134301048	-1.022275901	0.893094	1.017342	0.821746	3.19389071	3.35695728	3.16560381	320	485	385
7728	NM_007147,NR_136208	ZNF175	zinc finger protein 175	1.096823738	-1.022329846	4.339203	4.994998	3.986772	3.8375314	3.96195135	3.80791592	522	765	628
5917	NM_002887	RARS	arginyl-tRNA synthetase	1.05138281	-1.022344985	55.311001	58.226746	50.842205	6.85990595	6.93158852	6.82830055	4521	6351	5439
23491	NM_001185176,NM_001185177	CES3	carboxylesterase 3	-1.056221771	-1.022360961	12.030945	10.826853	10.120955	5.30887308	5.23199948	5.2777797	1517	1919	1825
51218	NM_016417	GLRX5	glutaredoxin 5	-1.001863404	-1.022537505	98.024559	97.951584	90.087379	6.81720397	6.81454192	6.78533769	4388	5852	5278
106635540	NR_132788_chr2,NR_132788_c	SNORA105B	small nucleolar RNA, H/ACA box	1.849629473	-1.0226078	16.633963	30.83913	15.262885	1.5285023	2.16626946	1.50747923	74	183	89
7422	NM_001025366,NM_001025367	VEGFA	vascular endothelial growth facto	2.030325096	-1.022645984	21.109543	42.684301	19.414242	6.18491721	7.19655708	6.15305813	2817	7642	3388
10477	NM_001278554,NM_001278555	UBE2E3	ubiquitin conjugating enzyme E2	1.98666545	-1.022829635	50.847968	101.264019	46.813165	6.30364437	7.28491901	6.27149421	3062	8128	3682
667	NM_001144769,NM_001144770	DST	dystonin	-1.308531681	-1.022898564	11.783396	8.959996	11.334228	7.58425566	7.19861809	7.55176422	7495	7653	9012
2962	NM_002096	GTF2F1	general transcription factor IIF su	1.211281244	-1.022921463	36.273537	43.992733	33.330505	6.50964486	6.78342183	6.47731132	3538	5726	4254
100505771	NR_132417,NR_132418	MHENCR	melanoma highly expressed com	-1.141050524	-1.022931212	16.938719	18.924376	15.613592	3.80372238	3.62781914	3.77337356	509	596	612
55701	NM_001278529,NM_001278530	ARHGEF40	Rho guanine nucleotide exchang	1.131710723	-1.023104037	3.267498	3.801098	3.002326	4.31466478	4.48473082	4.28338119	742	1122	892
9128	NM_001244926,NM_001322266	PRPF4	pre-mRNA processing factor 4	1.121794085	-1.023159653	19.671508	22.098009	18.045765	5.83187785	5.99494072	5.79943137	2197	3293	2641
9921	NM_001330474,NM_014868	RNF10	ring finger protein 10	-1.320939057	-1.023406174	24.842023	18.835392	22.828215	6.55685612	6.16018847	6.52383488	3657	3699	4395
57805	NM_021174,NR_033902	CCAR2	cell cycle and apoptosis regulator	1.120959441	-1.023451074	25.788997	28.975021	23.711815	6.59916399	6.7622958	6.56606979	3767	5642	4527
54187	NM_018946	NANS	N-acetylneuraminate synthase	-1.283591164	-1.023520988	101.049858	78.806206	92.784073	6.942592	6.58571949	6.90932637	4790	4986	5756
100287896	NM_001319240	LOC100287896	uncharacterized LOC100287896	-1.245444192	-1.023548518	1.307843	1.045842	1.197821	2.05603457	1.82188956	2.03057861	124	133	149
171392	NM_138330	ZNF675	zinc finger protein 675	-1.040280547	-1.02357587	8.863753	8.533265	8.140298	4.32753359	4.27344453	4.29560377	749	962	900
80169	NM_025099,NR_046431	CTC1	CST telomere replication comple	1.075059361	-1.023656223	3.438206	3.692099	3.162669	4.62276571	4.72309898	4.59041505	928	1333	1115
88745	NM_001329704,NM_033112,NR	RRP36	ribosomal RNA processing 36	1.272907836	-1.023764839	18.767148	23.956082	17.222465	4.53196733	4.86669962	4.49955999	869	1478	1044
5379	NR_003613	PMS2P1	PMS1 homolog 2, mismatch repa	-1.960357107	-1.023807494	7.775095	3.96484	7.145469	3.64534899	2.78063431	3.614139	452	308	543
51665	NM_001040445,NM_001330196	ASB1	ankyrin repeat and SOCS box co	1.393811376	-1.023818473	8.051015	11.284651	7.390163	5.78199488	6.25362336	5.74865753	2121	3950	2548
5771	NM_001207013,NM_001308287	PTPN2	protein tyrosine phosphatase, no	-1.134896821	-1.023840026	57.254994	53.613197	46.765198	6.74367085	6.56291967	6.71000053	4168	4907	5007
124411	NM_001130913	ZNF720	zinc finger protein 720	1.066542887	-1.023889219	4.59074	4.901769	4.208091	3.74463792	3.83086756	3.71313983	487	694	585
6813	NM_001127396,NM_001272034	STXBP2	syntaxin binding protein 2	1.086216203	-1.023954904	42.783808	46.607029	39.305213	6.32213325	6.44001655	6.28841171	3102	4502	3726
7186	NM_021138	TRAF2	TNF receptor associated factor 2	-1.094569925	-1.024117945	35.548912	32.51825	32.625458	6.31891475	6.19025398	6.28496741	3095	3778	3717
5209	NM_001145443,NM_001282630	PFKFB3	6-phosphofructo-2-kinase/fructos	2.495453183	-1.024163483	4.981256	12.705677	4.757979	4.54304361	5.8248786	4.5100879	876	2921	1052
473	NM_001042681,NM_001042682	RERE	arginine-glutamic acid dipeptide r	1.136132291	-1.02428605	10.146657	12.525237	9.313578	6.32488626	6.50686498	6.29070334	3108	4718	3732
148423	NM_198077,NR_024113	C1orf52	chromosome 1 open reading fran	1.090852398	-1.0242997	12.380982	13.542564	11.357173	5.37412105	5.49668479	5.34032578	1589	2316	1908
26154	NM_015657,NM_173076,NR_10	ABCA12	ATP binding cassette subfamily A	21.8569456	-1.024385657	0.011399	0.317737	0.011836	0.17294258	1.94127715	0.16897038	5	149	6
131368	NM_001329788,NM_175056	ZPLD1	zona pellucida like domain contai	9.546722428	-1.024385657	0.028032	0.337461	0.026772	0.17294258	1.16293478	0.16897038	5	65	6
197	NM_001622	AHSG	alpha 2-HS glycoprotein	1.486457258	-1.024385657	0.082828	0.112152	0.069616	0.17294258	0.25173066	0.16897038	5	10	6
106633804	NR_132756	SNORD135	small nucleolar RNA, C/D box 13	2.658859464	-1.024385657	1.630115	4.562067	1.562119	0.17294258	0.42561204	0.16897038	5	18	6
100287441	NM_001256861,NM_001256861	USP17L20	ubiquitin specific peptidase 17-lik	1.193356706	-1.024385657	0.074727	0.097791	0.065906	0.17294258	0.20477631	0.16897038	5	8	6
114655	NR_145963	SNORD73B	small nucleolar RNA, C/D box U7	-1.647024248	-1.024385657	1.471248	0.931562	1.554203	0.17294258	0.10601836	0.16897038	5	4	6
107985781	NR_144453,NR_144454	FSIP2-AS1	FSIP2 antisense RNA 1	2.219208637	-1.024385657	0.171099	0.468194	0.165404	0.17294258	0.36283659	0.16897038	5	15	6
26812	NR_002602	SNORD37	small nucleolar RNA, C/D box 37	1.339906982	-1.024385657	1.769987	2.387716	1.715164	0.17294258	0.2284445	0.16897038	5	9	6
27443	NM_001290046,NM_001290047	CECR2	CECR2, histone acetyl-lysine rea	3.831261671	-1.024385657	0.010836	0.052734	0.012357	0.17294258	0.58077243	0.16897038	5	26	6
100129291	NR_136576	LOC100129291	chromosome X open reading fran	-1.326777498	-1.024385657	0.057643	0.048056	0.055842	0.17294258	0.13134883	0.16897038	5	5	6
23435	NM_007375	TARDBP	TAR DNA binding protein	1.122164249	-1.024398565	71.607903	80.445946	65.686783	8.20463472	8.37038755	8.16997655	11543	17307	13859
170954	NM_001134870,NM_133471	PPP1R18	protein phosphatase 1 regulatory	-1.022466762	-1.024409601	13.705785	14.176603	13.076632	5.85491784	5.82342229	5.82073175	2233	2918	2681
727936	NM_001080393,NR_138564	GXYLT2	glucoside xylosyltransferase 2	-1.331384061	-1.024662684	0.169438	0.117629	0.154965	0.3273552	0.25173066	0.32020933	10	10	12
286133	NM_173833	SCARA5	scavenger receptor class A mem	2.084015866	-1.024662684	0.059315	0.13208	0.057736	0.3273552	0.61708849	0.32020933	10	28	12
101928153	NR_110531,NR_110532	ITGA9-AS1	ITGA9 antisense RNA 1	-1.890555353	-1.024662684	0.43047	0.203543	0.346331	0.3273552	0.18071335	0.32020933	10	7	12
29935	NM_013347	RPA4	replication protein A4	1.491607957	-1.024662684	0.166251	0.251142	0.154324	0.3273552	0.46599441	0.32020933	10	20	12
103021294	NR_131907	ABALON	apoptotic BCL2L1-antisense long	-1.112099584	-1.024662684	0.137065	0.123401	0.125419	0.3273552	0.29720487	0.32020933	10	12	12
60509	NM_001035507,NM_021831,NR	AGBL5	ATP/GTP binding protein like 5	1.448961694	-1.024736629	8.079017	11.29983	6.818709	4.46698912	4.98170859	4.43334413	829	1605	995
644660	NR_033886	RAD21-AS1	RAD21 antisense RNA 1	2.137397517	-1.02475643	0.184823	0.414522	0.173905	0.46682716	0.86372326	0.45708734	15	43	18
83849	NM_031912,NM_181519	SYT15	synaptotagmin 15	-1.33294858	-1.02475643	0.070187	0.053258	0.064055	0.46682716	0.36283659	0.45708734	15	15	18
2273	NM_001159699,NM_001159700	FHL1	four and a half LIM domains 1	4.323138527	-1.024803571	0.181083	0.783459	0.161585	0.59399636	1.68318474	0.58209675	20	116	24
101927780	NR_110416,NR_110417	LOC101927780	uncharacterized LOC101927780	1.717559751	-1.024803571	0.098296	0.173213	0.514796	0.59399636	0.90836632	0.58209675	20	46	24
55835	NM_018451,NR_047594,NR_04	CENPJ	centromere protein J	-1.2275018	-1.024820148	7.059758	5.717027	6.426244	5.17491202	4.88821942	5.14052934	1379	1501	1655
29798	NM_013310	C2orf27A	chromosome 2 open reading fran	3.074669104	-1.024831942	0.524531	1.650278	0.476657	0.71085839	1.5673195	0.69713258	25	103	30
400658	NM_001272093	C18orf65	chromosome 18 open reading fra	1.673609712	-1.024831942	0.312523	0.525415	0.281086	0.71085839	1.04792463	0.69713258	25	56	30
170850	NM_133329,NM_172344	KCNG3	potassium voltage-gated channel	-1.666184016	-1.024850891	0.202598	0.122972	0.183881	0.81895953	0.54351856	0.80366934	30	24	36
554210	NR_029957	MIR429	microRNA 429	-1.054696234	-1.024882539	14.183824	13.402896	12.837506	1.10178443	1.06116576	1.08290869	45	57	54
221895	NM_175061	JAZF1	JAZF zinc finger 1	2.2306345	-1.024898393	0.489301	1.093411	0.448763	1.33814853	2.14154997	1.31676564	60	179	72
100289092	NR_046287,NR_046288,NR_046	ATP2A1-AS1	ATP2A1 antisense RNA 1	1.162684149	-1.024902055	3.09391	3.578537	2.86477	1.40904982	1.5486383	1.38699077	65	101	78
286122	NM_173687,NR_126398,NR_12	C8orf31	chromosome 8 open reading fran	-1.253487235	-1.024939087	5.458013	4.361439	4.831096	3.51641288	3.22198991	3.48400652	410	437	492
64925	NM_022903	CCDC71	coiled-coil domain containing 71	1.622315144	-1.024939489	6.267894	10.196565	5.757771	3.59454042	4.2461677	3.56196863	435	943	522
64223	NM_001199173,NM_001199174	MLST8	MTOR associated protein, LST8	1.317213051	-1.02494341	14.590987	19.258793	13.433764	4.82687594	5.21210456	4.79259544	1075	1892	1290
5355	NM_002668	PLP2	proteolipid protein 2	-1.314731635	-1.024944993	66.006386	50.265076	60.536877	6.09531432	5.70715386	6.0602926	2645	2688	3174
4925	NM_001330227,NM_005013	NUCB2	nucleobindin 2	-1.73064686	-1.024978526	83.586188	48.36037	76.544001	7.05352904	6.27010962	7.01820583	5176	3996	6211
9295	NM_001190987,NM_001350605	SRSF11	serine and arginine rich splicing f	-1.1576516	-1.025058998	76.611129	66.414953	72.056299	8.26978144	8.05931503	8.23419128	12078	13940	14492
440456	NR_024386	PLEKHM1P1	pleckstrin homology and RUN do	-1.572712429	-1.025109994	18.502573	11.773925	16.957918	5.74547346	5.10749649	5.7103679	2067	1756	2480
9015	NM_001201536,NM_005681,NM	TAF1A	TATA-box binding protein associ	1.100711016	-1.025310451	6.280594	6.940251	5.768259	3.67154031	3.79960773	3.63833419	461	678	553
84286	NM_001297423,NM_001297424	TMEM175	transmembrane protein 175	-1.086062736	-1.025415973	26.06801	24.941827	24.630711	5.81826596	5.70135133	5.78270452	2176	2677	2610
81576	NM_001320561,NM_001320564	CCDC130	coiled-coil domain containing 130	1.017087982	-1.025439698	19.237407	19.61793	17.627251	5.17491202	5.19868721	5.13968208	1379	1874	1654
9262	NM_004226	STK17B	serine/threonine kinase 17b	-1.515444205	-1.025475475	4.743817	3.13428	4.349329	4.67404687	4.10306728	4.63918874	963	849	1155
118433	NR_000029,NR_024528,NR_024	RPL23AP7	ribosomal protein L23a pseudoge	1.056315625	-1.025477676	6.921772	7.202078	6.085045	3.17773766	3.24827687	3.1454863	316	446	379
7266	NM_001144766,NM_003315,NR	DNAJC7	DnaJ heat shock protein family (H	1.520419584	-1.025524899	52.906691	80.391411	48.487912	6.73023514	7.33005869	6.69421843	4129	8388	4952

11313	NM_007260	LYPLA2	lysophospholipase II	1.19047106	-1.025724816	38.838871	46.281971	35.578724	5.96372475	6.21156417	5.92767386	2411	3835	2891
56998	NM_001012329,NM_020248	CTNNBIP1	catenin beta interacting protein 1	-1.047364743	-1.025874637	14.592858	13.941857	13.364376	5.43059347	5.36540872	5.39460182	1654	2110	1983
162655	NM_145287,NR_033354	ZNF519	zinc finger protein 519	1.460615188	-1.02602182	4.718072	6.811889	4.881458	4.65666933	5.1851439	4.62109129	951	1856	1140
5706	NM_002806	PSMC6	proteasome 26S subunit, ATPase	-1.019511232	-1.02611077	79.526505	78.095482	72.833122	6.95833899	6.93068704	6.92145453	4843	6347	5805
10939	NM_006796	AFG3L2	AFG3 like matrix AAA peptidase	1.251182339	-1.026114687	26.219156	32.846951	24.018305	6.37400578	6.69381113	6.33726668	3217	5378	3856
55827	NM_001017977,NM_001198956	DCAF6	DDB1 and CUL4 associated facto	1.293308038	-1.026143439	16.629504	21.487224	15.262699	5.79462825	6.15980385	5.75807323	2140	3698	2565
55861	NM_001048221,NM_001048222	DBNDD2	dysbindin domain containing 2	-1.841191614	-1.026340202	43.00223	23.393709	40.825161	5.67097747	4.81389896	5.63421199	1961	1423	2350
23012	NM_015000	STK38L	serine/threonine kinase 38 like	-1.419278393	-1.026401836	4.814611	3.393769	4.406676	4.63760441	4.15647998	4.60153379	938	883	1124
387680	NM_001005751,NM_001291398	WASHC2A	WASH complex subunit 2A	-1.408268988	-1.026514541	7.27442	5.158637	6.683645	5.09974705	4.62285328	5.06310491	1307	1240	1566
4605	NM_001278610,NM_002466	MYBL2	MYB proto-oncogene like 2	1.491269514	-1.026555795	24.028375	35.876126	22.003489	6.04277245	6.61210602	6.00553977	2549	5079	3054
84171	NM_032211	LOXL4	lysyl oxidase like 4	1.597239604	-1.026578194	40.480366	64.731697	37.053562	7.18131131	7.85318067	7.14373119	5659	12077	6780
84065	NM_032125,NR_037576,NR_03	TMEM222	transmembrane protein 222	1.165509855	-1.026623809	19.100615	22.371796	17.706589	5.00565571	5.22024304	4.9689398	1222	1903	1464
115992	NM_001171815,NM_001171816	RNF166	ring finger protein 166	-1.017993903	-1.026722183	6.620565	6.480203	5.992551	3.72531928	3.70154644	3.69017701	480	630	575
54784	NM_017621	ALKBH4	alkB homolog 4, lysine demethyla	-1.621514088	-1.026792824	4.545045	2.808558	4.165214	3.37835054	2.76453517	3.34390718	369	304	442
10488	NM_006368	CREB3	cAMP responsive element bindin	1.134287146	-1.026807232	9.066278	10.295016	8.303451	4.11706758	4.28900382	4.08112291	642	973	769
7570	NM_006963	ZNF22	zinc finger protein 22	1.443433031	-1.026963402	3.000736	4.331475	2.746047	2.89611494	3.36498779	2.86293141	253	488	303
9382	NM_018714	COG1	component of oligomeric golgi co	-1.098662167	-1.026976955	24.692003	22.496878	22.593592	6.20846367	6.07463414	6.17058457	2864	3483	3430
92270	NM_001017971,NM_001349371	ATP6AP1L	ATPase H+ transporting accesso	1.570411543	-1.027040148	1.023443	1.595451	0.962064	2.3579234	2.90334193	2.32699908	162	340	194
51181	NM_001195218,NM_016286	DCXR	dicarbonyl and L-xylulose reducta	-1.393596614	-1.027252759	77.288417	55.497002	70.682704	5.98534723	5.51544527	5.94717473	2448	2347	2931
9093	NM_001135110,NM_001286516	DNAJA3	DnaJ heat shock protein family (H	1.164280763	-1.027276395	29.090096	34.724186	26.876404	6.27972211	6.49654578	6.24140268	3011	4684	3605
8792	NM_001270949,NM_001270950	TNFRSF11A	TNF receptor superfamily membe	-1.48574092	-1.027287858	1.238154	0.834594	1.230217	2.71208982	2.24380223	2.67922768	218	196	261
64780	NM_001159291,NM_001286613	MICAL1	microtubule associated monooxy	-1.235091602	-1.02747369	7.49018	6.000753	6.778546	4.74702075	4.45494398	4.70939036	1015	1098	1215
8535	NM_003655	CBX4	chromobox 4	1.011912017	-1.027700286	26.38979	26.73801	24.137856	6.11028539	6.12712406	6.0714425	2673	3614	3199
29105	NM_013242,NR_136632	CFAP20	cilia and flagella associated prote	1.337373316	-1.027745037	37.866348	50.931674	34.859724	5.62331734	6.03533764	5.58464442	1896	3388	2269
79169	NM_024319,NR_130975	C1orf35	chromosome 1 open reading fran	1.002885864	-1.027805524	33.324218	33.813741	30.749	5.59318572	5.59725738	5.55444677	1856	2487	2221
147138	NM_152468	TMC8	transmembrane channel like 8	-4.882009683	-1.027824932	9.149491	1.872838	8.366858	5.33550944	3.1801226	5.29690588	1546	423	1850
22906	NM_001042646,NM_001265608	TRAK1	trafficking kinesin protein 1	-1.526623369	-1.0278828	16.254052	10.605551	15.282819	6.34355917	5.74251508	6.30437724	3149	2756	3768
2146	NM_001203247,NM_001203248	EZH2	enhancer of zeste 2 polycomb re	-1.517717257	-1.028715348	37.883158	24.977826	34.613477	6.65273749	6.05821852	6.61230415	3911	3443	4676
90843	NM_001006684,NM_153333	TCEAL8	transcription elongation factor A l	1.50445378	-1.028785188	32.3394	48.422242	29.361802	5.26749617	5.84415687	5.22762904	1473	2961	1761
475	NM_004045	ATOX1	antioxidant 1 copper chaperone	-1.159550719	-1.028829092	101.537354	87.659584	92.739281	5.62480753	5.41588625	5.58464442	1898	2187	2269
79648	NM_001172574,NM_001172575	MCPH1	microcephalin 1	-1.019378268	-1.028897078	3.641185	3.095728	3.191966	4.30540198	4.27912189	4.26640425	737	966	881
205251	NR_027063	LINC00116	long intergenic non-protein codin	1.203263211	-1.02892902	10.410498	12.546576	9.514653	4.12340669	4.37634613	4.08464905	645	1037	771
5681	NM_006742	PSKH1	protein serine kinase H1	1.14518332	-1.029198039	4.234174	4.858983	3.869098	3.94164205	4.12530075	3.90285244	564	863	674
7022	NM_003222	TFAP2C	transcription factor AP-2 gamma	1.00438454	-1.029258985	11.609543	11.674333	10.602902	5.06174377	5.06786744	5.02139743	1272	1707	1520
23318	NM_001009881,NM_015269	ZCCHC11	zinc finger CCHC-type containing	-1.08945642	-1.029276944	10.091	9.276314	9.214491	5.87007592	5.74866631	5.82916462	2257	2768	2697
90381	NM_001308025,NM_152259	TICRR	TOPBP1 interacting checkpoint a	-1.087898862	-1.029448812	8.216905	7.561908	7.502329	5.76452587	5.64530591	5.72343286	2095	2573	2503
55333	NM_018373	SYNJ2BP	synaptojanin 2 binding protein	1.118200395	-1.029482551	9.66548	10.822061	8.82463	6.07583467	6.23475702	6.03454393	2609	3898	3117
1875	NM_001083588,NM_001083589	E2F5	E2F transcription factor 5	1.202850126	-1.02957769	8.364658	10.082423	7.633141	3.9199564	4.17029377	3.88071204	555	892	663
4237	NM_001135247,NM_001135248	MFAP2	microfibrillar associated protein 2	4.184420863	-1.029665703	1.689194	7.154973	1.526146	1.50279892	3.11812251	1.47560397	72	403	86
10654	NM_001323011,NM_001323012	PMVK	phosphomevalonate kinase	-1.038264513	-1.029668907	12.529588	12.063512	11.384267	4.06312959	4.01224533	4.02350017	617	794	737
9973	NM_005125	CCS	copper chaperone for superoxide	1.528636927	-1.029810297	17.859629	27.343761	16.301926	4.24466875	4.83042646	4.20455055	705	1440	842
2121	NM_001306090,NM_001306092	EVC	EvC ciliary complex subunit 1	-1.208969858	-1.02985484	1.403282	1.195803	1.161159	3.16959281	2.92884759	3.13191707	314	347	375
5711	NM_001270427,NM_005047	PSMD5	proteasome 26S subunit, non-AT	1.098607806	-1.029916547	20.245678	22.315219	18.489947	6.12088468	6.25470432	6.07897551	2693	3953	3216
10609	NM_006455	P3H4	prolyl 3-hydroxylase family memb	1.667958317	-1.029963483	7.555504	12.62454	6.894072	4.33301385	5.04222188	4.29255782	752	1676	898
55656	NM_017864,NR_073444,NR_07	INTS8	integrator complex subunit 8	-1.227215134	-1.029968439	24.470732	19.770572	22.203612	6.35662242	6.06521799	6.31454829	3178	3460	3795
56916	NM_001128429,NM_001128430	SMARCAD1	SWI/SNF-related, matrix-associa	-1.201121803	-1.030035507	9.818409	8.339567	9.195775	5.64257074	5.38396917	5.60074135	1922	2138	2295
29086	NM_001033549,NM_001288756	BABAM1	BRISC and BRCA1 A complex m	-1.094159043	-1.030102171	49.939282	45.446198	45.186204	6.15738072	6.02945501	6.11519985	2763	3374	3299
64417	NM_022483	TMEM267	transmembrane protein 267	-1.250470724	-1.030162109	4.981657	3.98779	4.540422	3.84777676	3.55012021	3.80791592	526	562	628
26155	NM_015658	NOC2L	NOC2 like nucleolar associated t	1.198981307	-1.030291626	72.318367	86.810898	65.96727	7.63060821	7.89121199	7.58777534	7741	12401	9241
84503	NM_032453	ZNF527	zinc finger protein 527	1.070852522	-1.030362392	1.289332	1.38244	1.17472	2.89611494	2.98202989	2.85881867	253	362	302
22834	NM_001145365,NM_014897,NR	ZNF652	zinc finger protein 652	-1.40137318	-1.030436703	6.967521	4.981912	6.045503	6.21490924	5.73582152	6.1722427	2877	2743	3434
146547	NM_001258290,NM_001258291	PRSS36	protease, serine 36	-1.591983775	-1.030448501	0.855143	0.537183	0.802626	1.75224037	1.31437719	1.72191206	93	78	111
4736	NM_007104	RPL10A	ribosomal protein L10a	1.07050337	-1.030457347	424.032562	454.417542	386.693268	8.21839513	8.31636628	8.17525744	11654	16669	13910
55319	NM_018352	TMA16	translation machinery associated	1.042128622	-1.030641388	14.384933	15.006495	13.117098	4.8007456	4.85819066	4.75878381	1055	1469	1259
84945	NM_032859	ABHD13	abhydrolase domain containing 1	1.032014247	-1.030642626	2.523784	2.609367	2.301833	3.81159508	3.85387581	3.77118696	512	706	611
5899	NM_002881	RALB	RAS like proto-oncogene B	1.182996538	-1.030655887	39.665844	46.98703	36.173382	6.46500194	6.70492623	6.42193834	3429	5420	4092
1122	NM_001821	CHML	CHM like, Rab escort protein 2	1.171401193	-1.030676255	6.987083	8.197064	6.371594	5.61734115	5.8412815	5.57464861	1888	2955	2253
2109	NM_001014763,NM_001985	ETFB	electron transfer flavoprotein beta	-1.049401606	-1.030744283	124.28859	118.502207	113.849411	6.82565171	6.75671129	6.78235512	4414	5620	5267
3326	NM_001271969,NM_001271970	HSP90AB1	heat shock protein 90 alpha famil	1.048206736	-1.030814504	505.44477	530.594966	460.924031	10.37779714	10.44567069	10.33404571	52198	73105	62281
150135	NR_027272	LINC00479	long intergenic non-protein codin	1.548361089	-1.030912487	0.733364	1.130817	0.66069	1.29387258	1.70021226	1.26797492	57	118	68
22985	NM_001164814,NM_001164815	ACIN1	apoptotic chromatin condensation	1.131297198	-1.030939645	105.852447	116.53414	84.27969	8.41163754	8.58912508	8.36780836	13330	20149	15903
146059	NM_138477	CDAN1	codanin 1	-1.206706236	-1.030948246	7.068835	5.864541	6.444319	5.04736934	4.78526051	5.00474315	1259	1394	1502
220074	NM_001145307,NM_001145308	LRTOMT	leucine rich transmembrane and	1.332255246	-1.031095047	2.259573	2.935569	1.955812	2.73986043	3.09899072	2.70236404	223	397	266
92312	NM_001093725	MEX3A	mex-3 RNA binding family memb	1.034521946	-1.031242391	1.395016	1.445361	1.269699	3.22173053	3.26553914	3.18215673	327	452	390
7707	NM_001348424,NM_001348425	ZNF148	zinc finger protein 148	1.559094781	-1.031343632	4.722965	7.620275	4.308805	5.48493837	6.11408027	5.44141974	1719	3581	2050
79866	NM_001286746,NM_001286747	BORA	bora, aurora kinase A activator	-1.213738844	-1.03135375	8.801126	7.346834	8.11103	4.71519816	4.44740017	4.67237502	992	1092	1183
84561	NM_001195483,NM_024628	SLC12A8	solute carrier family 12 member 8	1.367254617	-1.031402919	6.569367	8.998373	5.969319	4.54619271	4.9808379	4.50351696	878	1604	1047
154313	NM_001031743	CFAP206	cilia and flagella associated prote	-3.292881657	-1.031488601	0.608089	0.178389	0.551515	1.21691895	0.48576948	1.19154816	52	21	62
94234	NM_033260	FOXQ1	forkhead box Q1	-1.102673522	-1.031737286	39.695141	36.03759	36.155003	6.50196181	6.36258509	6.45738982	3519	4264	4195
5128	NM_001170464,NM_002595	CDK17	cyclin dependent kinase 17	-1.209782112	-1.031812625	8.305381	6.946872	7.519444	5.22293093	4.95624277	5.17897479	1427	1576	1701
23225	NM_024923	NUP210	nucleoporin 210	1.321832245	-1.031853013	14.724953	19.489693	13.412145	6.70544932	7.10462789	6.66065085	4058	7167	4837
80194	NM_001078650,NM_001078651	TMEM134	transmembrane protein 134	-1.037204051	-1.032023508	25.13387	24.278981	22.904111	4.61528873	4.56477126	4.57169125	923	1189	1100
6666	NM_006943	SOX12	SRY-box 12	1.894247735	-1.03216711	6.415062	12.172465	5.843722	4.91233736	5.81122887	4.86819666	1143	2893	1362
6170	NM_001000	RPL39	ribosomal protein L39	1.090135638	-1.032212096	1008.153442	1100.253052	917.816895	8.6874876	8.81170668	8.64186055	16147	23519	19240
5016	NM_002557	OVGP1	oviductal glycoprotein 1	1.428947145	-1.032247269	2.1842	3.138666	1.989444	2.53320657	2.97154911	2.49540988	188	359	224
57179	NM_001079684,NM_001079685	KIAA1191	KIAA1191	-1.06446726	-1.032605199	40.347424	37.753923	35.550244	6.73196468	6.64270541	6.6861171	4134	5189	4924
26056	NM_015470	RAB11FIP5	RAB11 family interacting protein	1.301860931	-1.032661948	4.158797	5.42401	3.78861	4.21727226	4.5798	4.17342809	691	1202	823
23	NM_001025091,NM_001090	ABCF1	ATP binding cassette subfamily F	1.038563377	-1.03290397	23.488811	24.325243	21.246645	6.31799386	6.37191335	6.27188091	3093	4292	3683
55609	NM_017666	ZNF280C	zinc finger protein 280C	1.51889893	-1.033139342	3.388175	5.156874	3.085611	4.04543465	4.61838139	4.00128413	609	1236	725
91300	NM_138774	R3HDM4	R3H domain containing 4	1.049183041	-1.033290484	40.881599	42.934551	37.174591	6.18995938	6.25830165	6.14336932	2827	3963	3365
85476	NM_001308164,NM_001308166	GFM1	G elongation factor mitochondrial	1.380412123	-1.033481575	24.407696	33.142327	22.085794	6.3610997	6.82136727	6.31417286	3188	5880	3794
11083	NM_001193369,NM_001193370	DIDO1	death inducer-obliterator 1	1.174970821	-1.033634397	29.599791	32.861021	26.583408	7.22372265	7.45491345	7.17632034	5829	9151	6936
8079	NM_005439,NR_026581	MLF2	myeloid leukemia factor 2	-1.041594264	-1.033710432	110.520617	103.604801	98.268153	7.6055441	7.54705793	7.55796097	7607	9758	9051
137964	NM_178819	GPAT4	glycerol-3-phosphate acyltransfer	-1.427467719	-1.033793228	16.385145	11.490998	14.896272	6.66799555	6.16057299	6.62052589	3953	3700	4703
59344	NM_001165960,NM_021628	ALOXE3	arachidonate lipoxygenase 3	1.965122186	-1.033836112	1.335505	2.634805	1.213929	2.44147625	3.27976824	2.40240201	174	457	207
9689	NM_001207067,NM_001207068	BZW1	basic leucine zipper and W2 dom	1.357478807	-1.033950309	105.379517	147.48582	98.025416	8.30552945	8.74526108	8.25751696	12382	22458	14729
246721	NM_032959	POLR2J2	RNA polymerase II subunit J2	-1.541366254	-1.034002565	3.745667	2.430933	3.405725	3.04601354	2.5131555	3.00368412	285	247	339
152518	NM_001278623,NM_001278624	NFXL1	nuclear transcription factor, X-bo	-1.444793994	-1.034147272	11.16068	7.926224	10.304155	5.42547226	4.90942294	5.37817319	1648	1524	1960
140680	NM_080571,NM_153269	C20orf96	chromosome 20 open reading fra	1.230972119	-1.034154665	14.610986	18.034036	13.275296	4.57731129	4.86575665	4.53091599	898	1477	1068
871	NM_001207014,NM_001235	SERPINH1	serpin family H member 1	2.750914858	-1.03415507	72.448529	199.525462	65.835683	7.33669648	8.79093167	7.28854788	6307	23182	7501
4735	NM_001008491,NM_001008492	SEPT2	septin 2	1.179022051	-1.034374465	107.638768	126.216834	98.082918	8.45814498	8.69511436	8.40952701	13768	21689	16371
90987	NM_138367	ZNF251	zinc finger protein 251	1.139663955	-1.034396273	7.451872	8.506435	6.770416	4.5127793	4.69364353	4.46615621	857	1305	1019
103910	NM_001144944,NM_001144945	MYL12B	myosin light chain 12B	-1.246186434	-1.034442679	590.573034	474.416248	536.447876	9.17860095	8.86169215	9.12983275	22711	24350	27003
84693	NM_032601	MCEE	methylmalonyl-CoA epimerase	-1.408407752	-1.034549311	6.988845	4.9603	6.343493	2.79384765	2.38212036	2.75199454	233	221	277
10645	NM_001270485,NM_001270486	CAMKK2	calcium/calmodulin dependent pr	-1.11774312	-1.034581415	10.784407	9.287491	9.598343	5.68889706	5.53158861	5.64081394	1986	2374	2361
353088	NM_001001415,NM_001346912	ZNF429	zinc finger protein 429	1.023356799	-1.034603201	1.445611	1.481999	1.164203	2.5831188	2.61093762	2.54232458	196	268	233
101927415	NR_110049	LOC101927415	uncharacterized LOC101927415	1.270380483	-1.034631669	0.594279	0.761278	0.543637	1.23264156	1.44132992	1.20457043	53	90	63
202	NM_001624	CRYBG1	crystallin beta-gamma domain co	-1.496050444	-1.034677884	13.94997	9.333994	12.668338	6.69271726	6.11844132	6.64401774	4022	3592	4781
10248	NM_005837	POP7	POP7 homolog, ribonuclease P/M	-1.322111378	-1.034757604	34.507248	26.137712	31.351694	5.01477763	4.62619813	4.96703057	1230	1243	1462

22828	NM_001286188,NM_001286189	SCAF8	SR-related CTD associated facto	1.107860615	-1.034759651	10.716536	11.888278	9.733421	5.7853302	5.93056448	5.73694088	2126	3147	2527
153129	NM_001258286,NM_001258287	SLC38A9	solute carrier family 38 member 9	1.178707542	-1.034787071	10.032796	11.684058	9.21628	4.6997269	4.92851423	4.6523174	981	1545	1166
10224	NM_005815	ZNF443	zinc finger protein 443	-1.181115792	-1.034825549	2.388925	2.023574	2.173857	2.85098947	2.64651142	2.80852903	244	276	290
1069	NM_004344	CETN2	centrin 2	1.086815304	-1.034904112	31.86001	34.672119	28.938454	5.10935964	5.22613324	5.06131639	1316	1911	1564
90417	NM_001142761,NM_001142762	KNSTRN	kinetochore localized astrin/SPA(	1.000031412	-1.034927301	73.901219	74.395988	67.746604	7.02192628	7.02197125	6.97278342	5063	6765	6017
81559	NM_145214	TRIM11	tripartite motif containing 11	1.03229198	-1.035056674	25.03615	25.878311	22.736446	6.05774429	6.10291957	6.0087914	2576	3553	3061
51192	NM_001040138,NM_016326,NM	CKLF	chemokine like factor	-1.196771132	-1.035072556	98.608884	83.089271	89.051731	6.29758411	6.0420314	6.24849348	3049	3404	3623
85026	NR_122035,NR_122036	ARRDC1-AS1	ARRDC1 antisense RNA 1	1.624918795	-1.035234991	14.324484	22.161655	12.288686	4.36724694	5.04055157	4.3197426	771	1674	916
145508	NM_152446	CEP128	centrosomal protein 128	1.114695134	-1.035398484	3.868253	4.319932	3.514	4.15469105	4.30300447	4.10736121	660	983	784
26025	NM_003735,NM_032094	PCDHGA12	protocadherin gamma subfamily .	-1.772294266	-1.035477511	0.149686	0.089295	0.139179	0.49317359	0.29720487	0.47868902	16	12	19
388533	NM_001244847,NM_207392	KRTDAP	keratinocyte differentiation assoc	1.261235873	-1.035477511	0.821037	1.064703	0.713851	0.49317359	0.59904473	0.47868902	16	27	19
54916	NM_017799	TMEM260	transmembrane protein 260	1.056948988	-1.035488185	5.521105	5.842295	5.01205	4.58038655	4.65704834	4.53220782	900	1271	1069
5827	NM_018663	PXMP2	peroxisomal membrane protein 2	1.376890077	-1.035600797	11.922615	16.448486	10.82135	3.60061161	4.02918937	3.55435929	437	804	519
100289098	NR_134570,NR_134571,NR_134	GS1-124K5.4	uncharacterized LOC100289098	-2.028989776	-1.035605781	1.216724	0.689788	1.277073	0.86002952	0.48576948	0.83750224	32	21	38
1154	NM_013324,NM_145071	CISH	cytokine inducible SH2 containing	-1.051254828	-1.035648745	0.549681	0.560897	0.526119	1.15225689	1.11294744	1.12461302	48	61	57
23466	NM_001303494,NM_014292	CBX6	chromobox 6	1.040901382	-1.035706761	17.106334	17.76354	15.437761	6.68844815	6.7457333	6.63833051	4010	5577	4762
116987	NM_001037131,NM_001244888	AGAP1	ArfGAP with GTPase domain, an	1.075483397	-1.035709402	5.820027	6.320354	5.222191	5.89251855	5.99580294	5.84276377	2293	3295	2723
220929	NM_001143766,NM_001143767	ZNF438	zinc finger protein 438	1.111799425	-1.035715045	2.176918	2.094563	1.683154	2.65489157	2.78463118	2.61239943	208	309	247
64376	NM_001271840,NR_073475,NR_	IKZF5	IKAROS family zinc finger 5	-1.004505671	-1.035728334	3.53192	3.552014	3.220046	4.07843661	4.07233457	4.03083014	624	830	741
23076	NM_015056	RRP1B	ribosomal RNA processing 1B	-1.408559066	-1.03595793	14.991585	10.656885	13.600029	6.23407496	5.74764293	6.18379658	2916	2766	3462
84932	NM_001163380,NM_032846,NR	RAB2B	RAB2B, member RAS oncogene	-1.010073455	-1.035964463	14.989446	14.844229	13.5906	5.42290482	5.40878241	5.37313581	1645	2176	1953
100630918	NR_038942	PARTICL	promoter of MAT2A antisense ra	1.183388338	-1.035982998	3.731931	4.42123	3.379134	2.62541894	2.83174937	2.58278312	203	321	241
3914	NM_000228,NM_001017402,NM	LAMB3	laminin subunit beta 3	1.172061924	-1.03600487	280.615897	329.282722	254.572959	10.11913776	10.34799663	10.06815355	43624	68316	51790
8314	NM_004656	BAP1	BRCA1 associated protein 1	1.073171871	-1.036005592	15.552302	16.71419	14.111901	5.8344561	5.93461705	5.78433175	2201	3156	2613
5422	NM_001330360,NM_016937	POLA1	DNA polymerase alpha 1, catalyt	1.178280031	-1.036009247	3.727318	4.318954	3.322212	4.38318339	4.6093958	4.33462681	780	1228	926
55094	NM_018025,NR_135270	GPATCH1	G-patch domain containing 1	-1.221314827	-1.036046208	9.336979	7.692861	8.418647	4.99532414	4.71683753	4.94586061	1213	1327	1440
56912	NM_001168618,NM_020153	IFT46	intraflagellar transport 46	-1.531211736	-1.036192513	10.692985	6.811938	9.489735	4.24660587	3.67164303	4.19805401	706	616	838
57594	NM_020834	HOMEZ	homeobox and leucine zipper en	1.165520114	-1.036208496	3.959285	4.622067	3.593054	3.94879876	4.15647998	3.90085366	567	883	673
25771	NM_001284303,NM_001284304	TBC1D22A	TBC1 domain family member 22A	1.368669637	-1.036216842	3.020415	4.123014	2.752647	3.62166181	4.04260277	3.57456234	444	812	527
9730	NM_001171904,NM_001349168	DCAF1	DDB1 and CUL4 associated facto	1.080214173	-1.036260892	8.643926	9.36843	7.271207	5.51417891	5.62315665	5.46393259	1755	2533	2083
54627	NM_019090	MAP10	microtubule associated protein 10	1.714399525	-1.036311186	0.870705	1.496462	0.78951	2.49460548	3.16180073	2.45238731	182	417	216
9531	NM_004281	BAG3	BCL2 associated athanogene 3	1.069813461	-1.03635033	27.905266	29.887087	25.304777	6.1460097	6.24204253	6.09523617	2741	3918	3253
5763	NM_001330333,NM_002824	PTMS	parathymosin	-1.789263884	-1.036387307	158.48751	88.808782	143.873434	7.55661017	6.72326319	7.50532494	7352	5490	8725
124989	NM_001195192,NM_152347	EFCAB13	EF-hand calcium binding domain	-1.162390862	-1.036396457	1.713411	1.504935	1.538705	2.83562844	2.65089709	2.79136857	241	277	286
85395	NM_001316983,NM_001316984	FAM207A	family with sequence similarity 20	1.0891422	-1.036401885	18.572785	20.358288	16.772506	4.12971807	4.2461677	4.08112291	648	943	769
64792	NM_001130820,NM_001130821	IFT22	intraflagellar transport 22	1.163324329	-1.036572008	4.151899	4.849212	3.774479	3.43720745	3.63669106	3.39023607	386	600	458
5830	NM_000319,NM_001131023,NM	PEX5	peroxisomal biogenesis factor 5	-1.039967318	-1.036711756	8.180177	8.343009	7.684678	4.80338004	4.74889974	4.75325534	1057	1358	1254
347240	NM_194313	KIF24	kinesin family member 24	1.432126276	-1.036717367	1.830537	2.627862	1.659538	3.60967076	4.09182073	3.56196863	440	842	522
5116	NM_001315529,NM_006031	PCNT	pericentrin	1.128239744	-1.036757284	7.801809	8.804189	7.069896	6.34174803	6.51380399	6.29032166	3145	4741	3731
10011	NM_001035235,NM_001253764	SRA1	steroid receptor RNA activator 1	2.290480827	-1.036774266	18.047057	41.470786	16.20785	5.04403172	6.21490035	4.99353253	1256	3844	1490
84284	NM_001329452,NM_001329453	NTPCR	nucleoside-triphosphatase, canc	1.104576366	-1.036860361	23.274615	25.517859	20.855341	4.93293448	5.07196145	4.88244775	1160	1712	1376
2241	NM_001308028,NM_001308031	FER	FER tyrosine kinase	1.255436366	-1.036896354	3.76444	4.386859	3.184798	5.33641922	5.6573446	5.28546063	1547	2595	1835
692073	NR_003035	SNORA16A	small nucleolar RNA, H/ACA box	-1.248212407	-1.036904487	8.248128	6.642015	7.502614	1.06712766	0.90836632	1.03996283	43	46	51
3934	NM_005564	LCN2	lipocalin 2	-1.536079304	-1.037052756	1464.333008	954.333313	1326.907104	10.23569411	9.61708096	10.18324903	47298	41141	56095
23531	NM_012329	MMD	monocyte to macrophage differer	1.294581815	-1.037109425	14.466832	18.749805	13.104082	5.29863957	5.6627837	5.24742715	1506	2605	1786
1593	NM_000784	CYP27A1	cytochrome P450 family 27 subfa	-1.465519857	-1.037165117	2.567407	1.756386	2.333674	2.75626978	2.30073045	2.71151569	226	206	268
55020	NM_017931	TTC38	tetratricopeptide repeat domain 3	1.071854576	-1.037207525	4.79846	5.149803	4.346297	3.72253824	3.81532231	3.67388275	479	686	568
1946	NM_001962	EFNA5	ephrin A5	1.116474084	-1.037396544	15.45289	17.273024	13.997925	6.34491604	6.50201808	6.29261026	3152	4702	3737
1611	NM_001291963,NM_004394	DAP	death associated protein	-1.084445329	-1.037425943	23.081832	21.309967	20.895756	5.74341713	5.62872593	5.69141343	2064	2543	2447
6122	NM_000967,NM_001033853	RPL3	ribosomal protein L3	1.145633411	-1.037476036	1131.371429	1297.638584	1024.390796	10.52082059	10.71684157	10.46777927	57642	88233	68335
23334	NM_015284	SZT2	seizure threshold 2 homolog (mo	1.152963181	-1.037492865	5.981851	6.905684	5.418464	6.36511741	6.56814636	6.31267016	3197	4925	3790
90634	NM_001079691,NM_001286459	N4BP2L1	NEDD4 binding protein 2 like 1	-1.534306562	-1.037717271	0.925635	0.598125	0.863417	1.90722898	1.48128072	1.86820921	108	94	128
642423	NR_049748,NR_049748_dup1	LOC642423	golgin A2 pseudogene	1.237993601	-1.037730805	6.887213	8.575644	6.247894	2.3579234	2.61093762	2.3150407	162	268	192
54677	NM_001143935,NM_001243745	CROT	carnitine O-octanoyltransferase	-1.35670168	-1.037748334	4.854896	3.43382	4.359404	3.66576097	3.26553914	3.61657686	459	452	544
493856	NM_001008388	CISD2	CDGSH iron sulfur domain 2	1.078932657	-1.037769517	10.716715	11.579099	9.706285	5.96549005	6.07340943	5.91287325	2414	3480	2861
138199	NM_001320497,NM_152420,NR	CARNMT1	carnosine N-methyltransferase 1	-6.176382885	-1.037803779	7.841075	1.286099	7.283077	5.1114871	2.68551066	5.05952566	1318	285	1562
150946	NM_001168241,NM_001191033	GAREM2	GRB2 associated regulator of MA	-1.100641798	-1.037919887	0.873944	0.797172	0.959489	2.43469539	2.32288731	2.39105499	173	210	205
10168	NM_001024855,NM_001323293	ZNF197	zinc finger protein 197	1.050102854	-1.038250215	2.643815	3.319529	2.624628	4.13391036	4.20052559	4.08288706	650	912	770
4860	NM_000270	PNP	purine nucleoside phosphorylase	1.133632163	-1.038389015	42.702881	48.457779	38.646709	6.66763414	6.84691748	6.61383023	3952	5986	4681
120071	NM_001300721,NM_001300722	LARGE2	LARGE xylosyl- and glucuronyltra	-2.432429726	-1.038416766	7.617462	3.110726	6.840064	4.29607932	3.11495144	4.24450423	732	402	867
396	NM_001185077,NM_001185078	ARHGDIA	Rho GDP dissociation inhibitor al	-1.039268596	-1.038509712	173.81197	168.499591	158.812488	8.36458453	8.30918737	8.31023789	12901	16586	15279
7016	NM_001318230,NM_006285	TESK1	testis-specific kinase 1	1.104462817	-1.038520219	8.450229	9.273224	7.582235	4.42312766	4.56011536	4.37117906	803	1185	951
2794	NM_005275	GNL1	G protein nucleolar 1 (putative)	1.153122758	-1.038609208	5.278538	6.092641	4.776073	5.38031	5.5812619	5.32698995	1596	2459	1890
10172	NM_005773	ZNF256	zinc finger protein 256	-1.05436481	-1.038716326	9.96264	9.46166	9.012047	4.48842972	4.41553601	4.43610715	842	1067	997
205717	NM_001009899,NR_111981	USF3	upstream transcription factor fam	1.361096759	-1.038818429	1.451344	1.978077	1.313185	4.34933082	4.77525187	4.29712434	761	1384	901
63916	NM_001318253,NM_133171,NM	ELMO2	engulfment and cell motility 2	1.004632774	-1.038821399	9.941813	9.607033	8.750448	5.31350078	5.32000218	5.25995683	1522	2043	1802
123283	NM_152334	TARSL2	threonyl-tRNA synthetase like 2	1.456149117	-1.038961902	1.465339	2.13974	1.325907	2.51403512	2.97505117	2.46867149	185	360	219
316	NM_001159	AOX1	aldehyde oxidase 1	5.382212206	-1.038990568	0.254758	1.402153	0.234853	1.16869613	2.95035633	1.13825079	49	353	58
26220	NR_002733,NR_045121,NR_110	DGCR5	DiGeorge syndrome critical regio	-1.453447983	-1.038990568	0.379469	0.261276	0.346053	1.16869613	0.89363826	1.13825079	49	45	58
114625	NM_001017922,NM_018538	ERMAP	erythroblast membrane associate	1.228391476	-1.039187032	3.584514	4.545165	3.280114	3.70855202	3.98471399	3.65740237	474	778	561
79966	NM_001037582,NM_024906	SCD5	stearoyl-CoA desaturase 5	1.272304279	-1.039204366	7.244896	9.268759	6.48768	4.50146728	4.83525167	4.44847569	850	1445	1006
10352	NM_015836,NM_201263	WARS2	tryptophanyl tRNA synthetase 2,	1.037977947	-1.039437784	4.104462	4.267038	3.7058	3.61567874	3.66515371	3.56449618	442	613	523
55884	NM_001278557,NM_001278558	WSB2	WD repeat and SOCS box contai	-1.511644581	-1.039474908	28.131088	18.948744	25.516291	6.32121441	5.73427243	6.2660695	3100	2740	3668
80028	NM_001321213,NM_024963	FBXL18	F-box and leucine rich repeat pro	-1.386870199	-1.039584914	5.905434	3.797911	4.676329	5.21602693	4.75909222	5.1615506	1420	1368	1680
4296	NM_002419	MAP3K11	mitogen-activated protein kinase	1.22921121	-1.039634077	18.995167	23.377834	17.17308	6.06818765	6.36191648	6.01296133	2595	4262	3070
123879	NM_173475	DCUN1D3	defective in cullin neddylation 1 d	1.060501086	-1.039642571	3.513795	3.734228	3.176682	4.46032739	4.54134004	4.40682806	825	1169	976
157657	NM_177965	C8orf37	chromosome 8 open reading fran	-1.24842459	-1.039700651	3.094028	2.478274	2.793856	3.48064928	3.19220918	3.42958826	399	427	472
6293	NM_001289174,NM_001289175	VPS52	VPS52, GARP complex subunit	-1.271788258	-1.039712122	15.369757	11.889129	13.847514	5.51819362	5.17984253	5.46325552	1760	1849	2082
55657	NM_001136036,NM_001193328	ZNF692	zinc finger protein 692	1.200519009	-1.039855814	42.473196	50.656775	37.149459	6.43558612	6.69646536	6.37986487	3359	5388	3973
84269	NM_001304353,NM_001304354	CHCHD5	coiled-coil-helix-coiled-coil-helix c	-1.008333939	-1.039964251	24.551898	24.43978	22.258101	4.12551355	4.11422685	4.07226969	646	856	764
55723	NM_018154	ASF1B	anti-silencing function 1B histone	-1.003973004	-1.040004196	44.346878	44.230255	40.075886	6.24332066	6.23767564	6.18749076	2935	3906	3471
28973	NM_014046	MRPS18B	mitochondrial ribosomal protein S	-1.012726329	-1.040026646	39.783783	39.319668	35.945885	5.91705205	5.89911068	5.8613842	2333	3078	2759
57506	NM_001206491,NM_020746,NR	MAVS	mitochondrial antiviral signaling p	1.469154622	-1.04004365	13.33038	19.573134	12.01535	7.2579772	7.8099586	7.20170942	5970	11719	7060
160335	NM_001320321,NM_001320322	TMTC2	transmembrane and tetratricopep	-1.946179868	-1.040083107	3.649074	2.028109	3.759497	4.11494833	3.23080555	4.0615736	641	440	758
92960	NM_001270539,NM_001300881	PEX11G	peroxisomal biogenesis factor 11	1.234460544	-1.040096334	3.330655	4.113237	2.972753	2.16656821	2.40826098	2.12264217	137	226	162
105369635	NR_135032	MIR200CHG	MIR200C and MIR141 host gene	-1.245147768	-1.040273228	14.273544	11.500214	12.913071	2.55837852	2.30073045	2.51121823	192	206	227
26083	NM_015594	TBC1D29	TBC1 domain family member 29	1.492081616	-1.040287158	0.429199	0.629637	0.362755	0.35634914	0.50527715	0.34394329	11	22	13
441518	NM_001078173	RTL8B	retrotransposon Gag like 8B	1.020394024	-1.040287158	0.128683	0.137084	0.125138	0.35634914	0.36283659	0.34394329	11	15	13
9868	NM_014820	TOMM70	translocase of outer mitochondria	1.431526775	-1.040337327	24.143581	34.608025	21.81123	6.70051131	7.21388986	6.64401774	4044	7735	4781
55906	NM_001178032,NM_001178033	ZC4H2	zinc finger C4H2-type containing	1.363781661	-1.040476334	6.495453	8.845024	5.744064	4.2192465	4.64610598	4.16512515	692	1261	818
101928222	NR_110235	LOC101928222	uncharacterized LOC101928222	1.460608604	-1.040499691	0.497359	0.72753	0.451909	0.64188297	0.86372326	0.62147005	22	43	26
101927126	NR_135079	GTF2IP7	general transcription factor IIi pse	-5.743294332	-1.040499691	0.738085	0.107347	0.673804	0.64188297	0.13134883	0.62147005	22	5	26
6861	NM_001297774,NM_003180	SYT5	synaptotagmin 5	-1.108939755	-1.0406068	0.622152	0.565097	0.56403	1.08456011	1.00745609	1.05442063	44	53	52
51569	NM_001286703,NM_001286704	UFM1	ubiquitin fold modifier 1	-1.278159503	-1.040668716	17.474138	13.445125	15.488589	5.59090027	5.24511195	5.53460273	1853	1937	2190
201164	NM_178836	PLD6	phospholipase D family member	-1.101314072	-1.040699592	3.229289	2.943214	2.919097	3.18986937	3.06652959	3.13871764	319	387	377
23032	NM_015017,NM_201624,NM_20	USP33	ubiquitin specific peptidase 33	1.055574111	-1.04071273	16.815234	17.595839	15.675016	6.13350189	6.2104504	6.076764	2717	3832	3211
147694	NM_001172773,NM_152909	ZNF548	zinc finger protein 548	-1.034873785	-1.040799141	26.858927	25.986248	24.259903	6.90690282	6.85786605	6.84970016	4672	6032	5521
123803	NM_001270766,NM_001270767	NTAN1	N-terminal asparagine amidase	1.192150279	-1.040817347	15.692356	18.810752	14.254548	4.29607932	4.5377923	4.24134834	732	1166	865
4651	NM_012334	MYO10	myosin X	1.100133091	-1.040893538	4.032575	4.441131	3.639922	5.51979639	5.65461734	5.46325552	1762	2590	2082

9874	NM_001136554,NM_001136555	TLK1	tousled like kinase 1	1.060765989	-1.04095293	15.240027	16.245404	13.754798	6.42664313	6.51079113	6.369423	3338	4731	3944
56242	NM_001331133,NM_001331134	ZNF253	zinc finger protein 253	-1.497241005	-1.041083546	2.305774	1.529433	2.058792	2.63136183	2.16012918	2.58278312	204	182	241
10807	NM_001039707,NM_001039708	SDCCAG3	serologically defined colon cance	1.069606027	-1.04124741	41.566148	44.50626	38.570056	6.53364194	6.62971046	6.47596937	3598	5142	4250
55666	NM_017921,NR_130139,NR_13	NPLOC4	NPL4 homolog, ubiquitin recognit	-1.272275884	-1.041282638	78.450241	48.433712	69.06102	8.03847681	7.69255446	7.98034095	10283	10799	12146
126295	NM_001319083,NM_173480	ZNF57	zinc finger protein 57	1.116891947	-1.0413152	5.104613	5.71946	4.619414	3.45742019	3.60313623	3.40441396	392	585	463
8996	NM_001185057,NM_001276307	NOL3	nucleolar protein 3	-1.311064135	-1.041323645	31.878773	24.409675	24.861761	5.57557074	5.19418688	5.51839824	1833	1868	2165
57470	NM_020710	LRRC47	leucine rich repeat containing 47	1.277645267	-1.04137302	15.544531	19.888113	14.025746	5.36076893	5.70662732	5.3037297	1574	2687	1859
9903	NM_001324309,NM_014851	KLHL21	kelch like family member 21	-1.154820303	-1.041382648	10.244868	9.08708	9.598123	5.63445641	5.43126519	5.57715406	1911	2211	2257
10188	NM_001010938,NM_001308046	TNK2	tyrosine kinase non receptor 2	-1.251093059	-1.041436215	59.467537	47.759879	53.609026	8.01181631	7.6900262	7.95347276	10094	10780	11921
84444	NM_032482	DOT1L	DOT1 like histone lysine methyltr	-1.056286671	-1.041452963	9.13535	8.657968	8.243393	6.06928257	5.99148667	6.01157269	2597	3285	3067
8526	NM_003647	DGKE	diacylglycerol kinase epsilon	-1.132865187	-1.041512821	6.736454	5.953067	6.079447	5.69954294	5.52324054	5.64201105	2001	2360	2363
55696	NM_018047	RBM22	RNA binding motif protein 22	-1.009334368	-1.041553742	36.125908	35.833847	32.596519	6.38416018	6.37091677	6.3261384	3240	4289	3826
54955	NM_001303030,NM_001303031	C1orf109	chromosome 1 open reading fran	1.15605288	-1.041592507	9.26467	10.435075	8.176012	4.59109862	4.79222547	4.53478801	907	1401	1071
585	NM_001252678,NM_001320665	BBS4	Bardet-Biedl syndrome 4	-1.431461744	-1.041656936	7.07049	4.955623	6.391829	4.18531139	3.70154644	4.12972136	675	630	797
3151	NM_005517	HMGN2	high mobility group nucleosomal	1.062442382	-1.041839876	139.973679	148.875244	126.245171	8.07327464	8.16034525	8.01436453	10535	14955	12437
10885	NM_006784	WDR3	WD repeat domain 3	1.344165099	-1.042143333	12.465918	16.775658	11.243649	5.58708313	6.00610962	5.52879006	1848	3319	2181
9909	NM_014856	DENND4B	DENN domain containing 4B	1.097631107	-1.042212318	13.27435	14.590837	11.969408	6.21984785	6.35252315	6.16101314	2887	4234	3407
151636	NM_138287	DTX3L	deltex E3 ubiquitin ligase 3L	1.49509475	-1.042309215	11.613254	17.387537	10.471905	6.04944579	6.62247115	5.99058114	2561	5116	3022
23014	NM_015002,NM_033624	FBXO21	F-box protein 21	-1.17780972	-1.04235453	8.171778	6.918988	7.360861	5.11042377	4.88170383	5.0523404	1317	1494	1554
64772	NM_001042573	ENGASE	endo-beta-N-acetylglucosaminida	-1.151266257	-1.042396015	14.138053	12.291812	12.746066	5.97428428	5.77452033	5.91535059	2429	2819	2866
100874260	NR_144562	UBE2CP5	ubiquitin conjugating enzyme E2	-1.092774174	-1.042764479	15.482802	14.172002	13.949359	4.31281697	4.19152238	4.25549579	741	906	874
55196	NM_018169	KIAA1551	KIAA1551	-1.564337276	-1.042790022	19.130636	12.244411	17.240065	6.87156413	6.23292987	6.81164103	4558	3893	5376
51606	NM_015941,NM_213619,NM_21	ATP6V1H	ATPase H+ transporting V1 subu	-1.316414215	-1.042880611	17.045017	13.002574	15.479091	5.20112046	4.81682937	5.14222236	1405	1426	1657
51115	NM_001286707,NM_001286719	RMDN1	regulator of microtubule dynamic	-1.102354732	-1.043041809	15.987622	14.865729	14.336796	5.56939302	5.43190244	5.50989942	1825	2212	2152
80142	NM_001256335,NM_025072,NM	PTGES2	prostaglandin E synthase 2	1.338927868	-1.043085255	39.675345	61.156979	36.032088	6.47371098	6.89068553	6.41355113	3450	6172	4068
11321	NM_001145047,NM_001145048	GPN1	GPN-loop GTPase 1	1.174133285	-1.043357707	22.946756	26.43183	20.258101	5.36344926	5.58985296	5.3037297	1577	2474	1859
126147	NM_145807	NTN5	netrin 5	1.175440748	-1.043400114	0.380289	0.448792	0.3414	0.77668602	0.8787583	0.75138414	28	44	33
60487	NM_001031712,NM_001350580	TRMT11	tRNA methyltransferase 11 homo	-1.112013215	-1.043456399	13.189302	11.531772	11.94011	4.79678491	4.64939741	4.73766195	1052	1264	1240
4723	NM_001166102,NM_007103	NDUFV1	NADH:ubiquinone oxidoreductas	1.116478647	-1.043467599	72.049242	80.472663	64.838197	6.84942803	7.00708143	6.78858439	4488	6695	5290
55278	NM_018292	QRSL1	glutaminyl-tRNA synthase (glutar	-1.203095087	-1.043493631	4.941242	4.112298	4.448361	4.37965714	4.12687582	4.32123795	778	864	917
79071	NM_001130721,NM_024090	ELOVL6	ELOVL fatty acid elongase 6	1.345813975	-1.043718723	27.919825	37.69761	25.152731	6.46083622	6.88511209	6.39981707	3419	6148	4029
9794	NM_014757	MAML1	mastermind like transcriptional co	1.331954348	-1.043791967	8.814913	11.757113	7.935683	5.64771074	6.05408526	5.58713259	1929	3433	2273
10052	NM_001080383,NM_005497	GJC1	gap junction protein gamma 1	-1.471712745	-1.043812861	14.894059	10.129542	13.406674	6.80542482	6.25398377	6.74412514	4352	3951	5128
148304	NM_152485	C1orf74	chromosome 1 open reading fran	1.036594742	-1.043854239	3.017999	3.133269	2.718963	3.93444966	3.98297572	3.87664976	561	777	661
197131	NM_174916	UBR1	ubiquitin protein ligase E3 compo	1.005435872	-1.043903163	4.925108	4.960213	4.435072	5.25407798	5.2616952	5.19374412	1459	1960	1719
5700	NM_001330212,NM_002802	PSMC1	proteasome 26S subunit, ATPas	1.227120061	-1.043934709	113.476897	139.402091	102.153503	7.46161609	7.75538098	7.39994313	6881	11282	8107
51512	NM_016426	GTSE1	G2 and S-phase expressed 1	1.184456598	-1.043986584	12.363091	14.658588	11.128057	5.26558691	5.50397588	5.20512776	1471	2328	1733
202243	NM_001297696,NM_001297697	CCDC125	coiled-coil domain containing 125	-1.035362003	-1.044083497	6.020367	5.754513	5.338796	4.43336854	4.38558611	4.37406362	809	1044	953
7756	NM_001032293,NM_001098507	ZNF207	zinc finger protein 207	1.112427866	-1.04421138	137.4821	153.437745	123.05359	8.24806879	8.40130223	8.18586419	11897	17683	14013
29079	NM_001270629,NM_014166	MED4	mediator complex subunit 4	1.083727462	-1.044349365	6.181784	6.70668	5.539543	3.85541331	3.96371513	3.79721001	529	766	623
7458	NM_022170,NM_031992	EIF4H	eukaryotic translation initiation fa	-1.402142694	-1.044592686	141.464184	100.770619	127.753402	8.43507865	7.9491162	8.37232348	13549	12911	15953
54914	NM_017794	FOCAD	focadhesin	-1.095481486	-1.044779837	5.759363	5.265992	5.182446	5.137819	5.01015048	5.07644857	1343	1638	1581
896	NM_001136017,NM_001136125	CCND3	cyclin D3	-1.173159783	-1.044789478	13.314372	11.253805	11.911082	4.78616959	4.56477126	4.72529065	1044	1189	1229
8905	NM_001272071,NM_003916	AP1S2	adaptor related protein complex '	-1.671325564	-1.04480731	2.849262	1.704935	2.560076	2.90104285	2.28388661	2.84640965	254	203	299
1911	NM_004426	PHC1	polyhomeotic homolog 1	-1.296300433	-1.044897896	9.217568	7.116487	8.28901	5.56861895	5.20317354	5.50661727	1824	1880	2147
84108	NM_001011663,NM_032154	PCGF6	polycomb group ring finger 6	-1.568790554	-1.044907501	6.952065	4.438802	6.250056	4.00938005	3.40966853	3.95001076	593	505	698
26292	NM_012333,NR_037632	MYCBP	MYC binding protein	-1.208082888	-1.044915856	15.212408	12.61513	13.689073	5.28456677	5.01950785	5.2228368	1491	1649	1755
284323	NM_001010880,NM_001142577	ZNF780A	zinc finger protein 780A	1.053098498	-1.044974845	4.497581	4.72013	4.099823	4.19135823	4.26202234	4.1314271	678	954	798
83606	NM_001284251,NM_001284252	GUCD1	guanylyl cyclase domain containi	1.214835985	-1.045100312	45.982949	55.615827	40.842086	7.31931512	7.59848268	7.25607987	6231	10114	7333
27154	NM_015695	BRPF3	bromodomain and PHD finger co	1.323919062	-1.045119768	7.045387	9.342075	6.336858	5.40393602	5.80041275	5.34179996	1623	2871	1910
3298	NM_001135564,NM_001243094	HSF2	heat shock transcription factor 2	-1.19222487	-1.045126515	8.455675	7.04669	7.415212	4.480221	4.23890304	4.41944907	837	938	985
100874054	NR_051960	FALEC	focally amplified long non-coding	-1.032248592	-1.045133825	0.748694	0.739955	0.676439	0.5190475	0.50527715	0.49997203	17	22	20
11315	NM_001123377,NM_007262	PARK7	Parkinsonism associated deglyca	1.034094438	-1.045137227	211.555672	219.708054	189.85305	7.58291465	7.63103516	7.5195609	7488	10346	8812
10296	NM_001017405,NM_001297430	MAEA	macrophage erythroblast attache	1.078700296	-1.045204076	61.816981	66.394211	55.635569	7.06784253	7.176354	7.0045425	5228	7535	6152
136	NM_000676	ADORA2B	adenosine A2b receptor	1.477189791	-1.04531709	23.249781	34.39439	20.900301	5.44921723	6.00139486	5.38676798	1676	3308	1972
7423	NM_001243733,NM_003377	VEGFB	vascular endothelial growth facto	1.178122316	-1.045543724	30.22291	35.709545	27.103828	5.74341713	5.97584027	5.6803862	2064	3249	2428
25980	NM_001271874,NM_015511	AAR2	AAR2 splicing factor homolog	1.165647756	-1.04554506	15.869111	18.190547	13.686089	5.30608931	5.52204401	5.2434892	1514	2358	1781
55017	NM_017924	C14orf119	chromosome 14 open reading fra	1.141549181	-1.045572946	28.661348	32.757462	25.75885	6.37842952	6.56727656	6.31492362	3227	4922	3796
115939	NM_001001410	TSR3	TSR3, acp transferase ribosome	1.30656115	-1.04561769	25.621178	33.535156	23.037113	4.95679582	5.33165924	4.89455192	1180	2060	1388
6611	NM_001258423,NM_004595	SMS	spermine synthase	1.074839358	-1.045622377	62.457882	67.247668	56.403545	6.81818124	6.92141425	6.75440081	4391	6306	5165
84321	NM_032361	THOC3	THO complex 3	1.078977613	-1.045811441	48.720745	52.627522	43.774738	6.26594024	6.37423602	6.20217361	2982	4299	3507
8871	NM_001178088,NM_003898	SYNJ2	synaptojanin 2	2.002208834	-1.046016769	6.425953	12.882335	5.774307	5.56551847	6.55182299	5.50200967	1820	4869	2140
5566	NM_001304349,NM_002730,NM	PRKACA	protein kinase cAMP-activated ca	1.113302988	-1.046195365	23.110791	25.76276	20.512589	5.93518222	6.08763347	5.87111561	2363	3515	2778
22800	NM_001102669,NM_001177314	RRAS2	related RAS viral (r-ras) oncogen	-1.227529995	-1.046316549	31.587834	25.718465	28.252174	6.18390666	5.8926442	6.11950347	2815	3064	3309
222068	NM_001303058,NM_001303059	TMED4	transmembrane p24 trafficking pr	-1.854181001	-1.046326504	49.952775	27.866873	44.388935	6.8356598	5.95559749	6.77091004	4445	3203	5225
79036	NM_001171948,NM_001171949	KXD1	KxDL motif containing 1	-1.18551251	-1.046354706	45.557637	38.772793	41.020295	6.06105037	5.8195316	5.9966768	2582	2910	3035
11285	NM_007255	B4GALT7	beta-1,4-galactosyltransferase 7	1.34158316	-1.046357248	14.666493	19.708996	13.174341	4.68265777	5.09225907	4.61987668	969	1737	1139
159013	NM_001330455,NM_144970	CXorf38	chromosome X open reading fran	-1.02400143	-1.046422388	6.139034	5.973933	5.378924	4.66683165	4.63397275	4.60399302	958	1250	1126
3213	NM_001330322,NM_001330323	HOXB3	homeobox B3	-2.217018959	-1.046516397	107.084666	49.372312	94.943406	8.50330115	7.35949744	8.43789063	14207	8562	16697
56970	NM_001098833,NM_020218	ATXN7L3	ataxin 7 like 3	1.028470184	-1.046574273	33.680896	34.678015	30.239667	6.89367478	6.93383976	6.82856345	4629	6361	5440
90410	NM_001267774,NM_001267775	IFT20	intraflagellar transport 20	-1.072541217	-1.046584251	21.692063	20.791834	19.508768	4.45531083	4.35902755	4.39267399	822	1024	966
79155	NM_001161527,NM_001292016	TNIP2	TNFAIP3 interacting protein 2	1.103819205	-1.046886716	22.320776	24.521554	19.508464	5.40999864	5.54931631	5.34547883	1630	2404	1915
192683	NM_001178111,NM_001178112	SCAMP5	secretory carrier membrane prote	1.386814205	-1.046889964	2.142317	3.18062	1.92233	3.05045588	3.47295962	2.99246523	286	530	336
100310756	NR_147504	LOC100310756	uncharacterized LOC100310756	-1.973163915	-1.04693838	8.125072	4.119528	7.29828	3.64534899	2.77260719	3.58455875	452	306	531
23251	NM_015206	KIAA1024	KIAA1024	-1.475573835	-1.047019458	0.244092	0.16339	0.218187	1.38110603	1.06116576	1.34055586	63	57	74
91373	NM_207309	UAP1L1	UDP-N-acetylglucosamine pyrop	1.019970445	-1.047094537	3.428473	3.496934	3.071123	3.61267788	3.63890054	3.5518139	441	601	518
26015	NM_015540	RPAP1	RNA polymerase II associated pr	1.06434118	-1.047133685	10.719091	11.425166	9.618615	5.64036226	5.72857817	5.57527538	1919	2729	2254
80704	NM_025243	SLC19A3	solute carrier family 19 member 3	-2.185853538	-1.047164886	1.029077	0.47151	0.928366	2.26158951	1.45142181	2.20918158	149	91	175
5188	NM_004564	GATB	glutamyl-tRNA amidotransferase	1.393556459	-1.047167293	9.938366	13.859887	8.914574	4.49821892	4.9588981	4.4347263	848	1579	996
79641	NM_024589,NR_046480	ROGDI	rogdi homolog	1.142752627	-1.047252618	12.670323	14.539897	11.343502	4.480221	4.6646589	4.41665393	837	1278	983
7178	NM_001286272,NM_001286273	TPT1	tumor protein, translationally-con	-1.055431153	-1.047292463	205.955455	194.634935	184.837397	9.88453602	9.80678792	9.81794361	37071	46930	43536
23317	NM_001329126,NM_015268	DNAJC13	DnaJ heat shock protein family (H	1.214539591	-1.047367418	14.056454	17.092941	12.612463	6.70474492	6.98271594	6.6386304	4056	6582	4763
92691	NM_001142310,NM_001142311	TMEM169	transmembrane protein 169	1.105888677	-1.047480175	0.168983	0.189589	0.155744	0.66524311	0.72085408	0.64076093	23	34	27
339240	NR_001443	KRT17P5	keratin 17 pseudogene 5	-1.460238429	-1.047480175	0.182868	0.129394	0.16811	0.66524311	0.48576948	0.64076093	23	21	27
83475	NM_001145165,NM_031304	DOHH	deoxyhypusine hydroxylase	1.186565589	-1.047932207	9.569698	11.122718	8.312863	4.26200996	4.49696276	4.19805401	714	1132	838
10051	NM_001002800,NM_001288753	SMC4	structural maintenance of chromo	1.199140227	-1.047972039	124.754652	149.80015	111.878778	9.32787299	9.58950152	9.26038015	25191	40361	29565
134218	NM_001012339,NM_001348420	DNAJC21	DnaJ heat shock protein family (H	1.009151746	-1.047972379	3.898458	3.942394	3.481679	4.59566527	4.60826865	4.53091599	910	1227	1068
5939	NM_002898	RBMS2	RNA binding motif single strande	-2.066630918	-1.048119081	34.369366	16.648903	30.815746	8.15470361	7.11279792	8.08714386	11149	7208	13083
79596	NM_024546	RNF219	ring finger protein 219	1.436726909	-1.048212092	4.606189	6.631899	4.133016	4.07625981	4.57288311	4.01243492	623	1196	731
5886	NM_001270362,NM_001270363	RAD23A	RAD23 homolog A, nucleotide ex	-1.080811954	-1.048256768	88.97674	82.431849	79.672045	7.30474643	7.19336609	7.2371933	6168	7625	7237
149951	NM_001099339,NM_053041	COMMD7	COMM domain containing 7	-1.087727236	-1.048326654	10.428082	9.591703	9.34196	4.35113248	4.2359869	4.28644656	762	936	894
51575	NM_001276380,NM_016649	ESF1	ESF1 nucleolar pre-rRNA proces	-1.089405466	-1.048444589	18.804734	17.282366	16.861328	5.8999225	5.77853415	5.83283856	2305	2827	2704
51715	NM_001278666,NM_001278667	RAB23	RAB23, member RAS oncogene	-1.009680751	-1.048584364	2.15346	2.215055	1.933018	3.48064928	3.46799726	3.41845388	399	528	468
55032	NM_001348905,NM_001348906	SLC35A5	solute carrier family 35 member A	1.525063573	-1.048738268	5.265264	7.916349	4.827814	4.53988763	5.12730363	4.47424393	874	1781	1025
106479524	NR_144555	RN7SL832P	RNA, 7SL, cytoplasmic 832, pseu	1.983592876	-1.048864283	0.363327	0.719735	0.322281	0.79797761	1.30327576	0.76902392	29	77	34
399821	NR_033847	FLJ37035	uncharacterized LOC399821	1.057917735	-1.048864283	0.293088	0.314889	0.262104	0.79797761	0.83317479	0.76902392	29	41	34
9914	NM_001286527,NM_001291454	ATP2C2	ATPase secretory pathway Ca2+	-1.243830466	-1.048882323	10.899019	8.65982	9.705205	5.1981206	4.89285546	5.13118205	1402	1506	1644
11209	NR_027504	MST1P2	macrophage stimulating 1 pseud	-1.440849049	-1.048896126	5.861547	4.072884	5.254181	4.00480921	3.51686905	3.94031242	591	548	693
55794	NM_018380	DDX28	DEAD-box helicase 28	1.319842971	-1.049052982	6.135559	8.112413	5.494305	4.04543465	4.42452914	3.98061446	609	1074	714

55623	NM_001304550,NM_017736	THUMPD1	THUMP domain containing 1	1.065716724	-1.049111678	19.233263	20.556621	17.283373	6.28255717	6.37324105	6.21429619	3017	4296	3537
23367	NM_015315	LARP1	La ribonucleoprotein domain fam	1.057963205	-1.049128435	68.291267	72.329277	61.170341	8.77975413	8.86086422	8.71072362	17216	24336	20183
55466	NM_001130182,NM_001130183	DNAJA4	DnaJ heat shock protein family (H	-1.239067106	-1.049342156	39.399866	32.061168	35.060335	6.92273916	6.61631708	6.85383893	4724	5094	5537
9354	NM_001204077,NM_004788	UBE4A	ubiquitination factor E4A	-1.861417242	-1.049386766	14.187028	7.630187	12.701903	6.41592393	5.53396493	6.34720926	3313	2378	3883
81790	NM_001160223,NM_001160224	RNF170	ring finger protein 170	-1.285930197	-1.049427028	6.958455	5.343405	6.137629	4.72634668	4.37899217	4.65942844	1000	1039	1172
81622	NM_030930	UNC93B1	unc-93 homolog B1 (C. elegans)	-1.094608482	-1.049453405	57.673473	52.745544	51.642406	7.03711487	6.90773598	6.96801834	5117	6246	5997
25791	NM_001114090,NM_019850	NGEF	neuronal guanine nucleotide excl	1.1557488	-1.049469842	11.121584	12.890796	9.963053	4.97793871	5.18060106	4.91053445	1198	1850	1404
23054	NM_001242539,NM_001318240	NCOA6	nuclear receptor coactivator 6	1.069496965	-1.049537655	15.503833	11.644362	9.435818	6.16661807	6.26224839	6.09785585	2781	3974	3259
4846	NM_000603,NM_001160109,NM	NOS3	nitric oxide synthase 3	-4.412768537	-1.049572828	1.750457	0.39354	1.571105	3.0724647	1.42093184	3.0111152	291	88	341
81691	NM_001144924,NM_001199053	REXO5	RNA exonuclease 5	-1.271697706	-1.049621686	6.174535	4.726141	5.527175	4.13809052	3.81336732	4.07226969	652	685	764
152002	NM_001308069,NM_152531	XXYLT1	xyloside xylosyltransferase 1	1.334940311	-1.049660131	8.829387	11.813633	7.984452	4.61078387	5.01270852	4.5437825	920	1641	1078
22818	NM_001271734,NM_001271735	COPZ1	coatomer protein complex subun	-1.124473882	-1.049664307	48.651805	43.265757	43.579542	6.52688319	6.35957387	6.45772977	3581	4255	4196
23598	NM_014323,NM_032050,NM_03	PATZ1	POZ/BTB and AT hook containing	-1.18580214	-1.049695182	10.352685	8.880489	9.12829	5.1523576	4.91399145	5.08439592	1357	1529	1590
130340	NM_001039569,NR_110905,NR	AP1S3	adaptor related protein complex 1	-1.36170322	-1.049744418	13.578496	9.971846	12.132858	5.7502602	5.31448374	5.68155094	2074	2035	2430
728978	NR_038453	UNC5B-AS1	UNC5B antisense RNA 1	-3.316645489	-1.049777353	1.412814	0.423247	1.244621	0.91952222	0.3412894	0.88680889	35	14	41
55750	NM_018238	AGK	acylglycerol kinase	-1.438158029	-1.049780413	11.458758	7.979287	10.257294	5.07270439	4.56709359	5.00474315	1282	1191	1502
23240	NM_001131007,NM_015196	TMEM131L	transmembrane 131 like	1.43071759	-1.049804878	2.12582	3.041922	1.89886	3.509976	3.98818428	3.44613034	408	780	478
10062	NM_001130101,NM_001130102	NR1H3	nuclear receptor subfamily 1 grou	-1.589644739	-1.049974208	46.123012	29.208889	40.806735	6.40121682	5.74251508	6.33171327	3279	2756	3841
151056	NM_001170585,NM_153021,NR	PLB1	phospholipase B1	-1.267276625	-1.050122355	0.558822	0.442724	0.50187	1.93632435	1.69172362	1.88448459	111	117	130
90853	NM_001281987,NM_001281988	SPOCD1	SPOC domain containing 1	1.976323238	-1.050126893	6.296374	12.496012	5.638858	4.65812548	5.61251543	4.59041505	952	2514	1115
9377	NM_004255	COX5A	cytochrome c oxidase subunit 5A	-1.033765175	-1.050147779	106.307701	102.93119	95.108162	6.33265804	6.28535216	6.26296048	3125	4039	3660
57092	NM_001320395,NM_001320397	PCNP	PEST proteolytic signal containin	1.400178428	-1.050157113	42.245578	59.486933	37.589888	6.62359308	7.1050275	6.55371946	3832	7169	4488
5688	NM_002792	PSMA7	proteasome subunit alpha 7	1.12816385	-1.050179727	256.356384	289.529419	229.394119	8.01451885	8.18786325	7.94416173	10113	15244	11844
8337	NM_003516,NM_003516_dup1	HIST2H2AA3	histone cluster 2 H2A family men	1.489851042	-1.050242675	36.925484	55.088852	33.038975	4.33665583	4.88821942	4.26950586	754	1501	883
3840	NM_002268	KPNA4	karyopherin subunit alpha 4	1.268263647	-1.050287826	14.305407	18.162283	12.797748	6.97829416	7.31873322	6.90808305	4911	8322	5751
64105	NM_001267038,NM_001349367	CENPK	centromere protein K	-1.099830238	-1.050331567	45.926432	41.695038	40.998926	6.25779957	6.1223945	6.18790064	2965	3602	3472
10155	NM_005762	TRIM28	tripartite motif containing 28	1.022779138	-1.050351081	241.795914	247.576904	216.327957	9.44627553	9.47872423	9.37550773	27349	37374	32025
81894	NM_031212	SLC25A28	solute carrier family 25 member 2	-1.958881921	-1.050422453	33.160713	16.943972	29.675488	5.67888953	4.73554094	5.6093351	1972	1345	2309
64326	NM_001001740,NM_001286644	RFWD2	ring finger and WD repeat domai	-1.069697459	-1.050601775	17.620922	16.455433	15.728049	5.59850439	5.50336969	5.52879006	1863	2327	2181
11344	NM_007284	TWF2	twinfilin actin binding protein 2	-1.028031526	-1.05070208	20.56041	20.023989	18.40053	5.06833013	5.02964715	4.99914873	1278	1661	1496
23301	NM_001142614,NM_001142615	EHBP1	EH domain binding protein 1	-1.158807745	-1.050733406	17.695222	15.306046	15.840999	6.41333943	6.20337644	6.34279879	3307	3813	3871
1500	NM_001085458,NM_001085459	CTNND1	catenin delta 1	-1.097784294	-1.050767357	87.631097	80.100174	78.781518	8.95650718	8.82219574	8.88521089	19465	23691	22784
55173	NM_018141	MRPS10	mitochondrial ribosomal protein S	-1.0992423	-1.050802662	20.363918	18.543999	18.207365	5.44078168	5.30755587	5.37097154	1666	2025	1950
4947	NM_001301302,NM_002537	OAZ2	ornithine decarboxylase antizyme	2.978978701	-1.050929249	10.482875	31.263247	9.374829	4.37435155	5.90233309	4.30621424	775	3085	907
9849	NM_001278524,NM_001278525	ZNF518A	zinc finger protein 518A	-5.911974677	-1.050933346	10.241678	1.849651	9.223837	6.38195877	3.90066256	6.3111659	3235	731	3786
8882	NM_001317086,NM_003904	ZPR1	ZPR1 zinc finger	1.02725141	-1.051069328	18.396168	18.95031	16.49314	5.31350078	5.35132998	5.2434892	1522	2089	1781
83877	NM_001024380,NM_001024381	TM2D2	TM2 domain containing 2	1.233867075	-1.051135574	17.604817	21.788969	15.440676	5.85555262	6.1540222	5.78487375	2234	3683	2614
134510	NM_145049	UBLCP1	ubiquitin like domain containing C	1.118417932	-1.051211686	17.325603	19.400434	15.493361	5.37854443	5.53633734	5.30826105	1594	2382	1865
163786	NM_001304829,NM_194292	SASS6	SAS-6 centriolar assembly protei	-1.274413017	-1.051253076	11.796219	9.24891	10.610172	5.56396574	5.22245468	5.49341346	1818	1906	2127
400927	NR_002821	LOC400927	TPTE and PTEN homologous inc	-1.387433095	-1.051282245	2.789217	1.997257	2.484241	1.23264156	0.97983233	1.19154816	53	51	62
374291	NM_024407	NDUFS7	NADH:ubiquinone oxidoreductase	-1.119943719	-1.051297444	64.805725	57.944977	57.948624	5.66157057	5.50154961	5.59085682	1948	2324	2279
340481	NM_178566	ZDHHC21	zinc finger DHHC-type containing	1.1770771	-1.051448044	2.468693	2.909894	2.206348	4.52719419	4.75298538	4.45802288	866	1362	1013
7917	NM_001098534,NM_001199697	BAG6	BCL2 associated athanogene 6	-1.124060593	-1.051571261	36.826422	32.741054	32.725513	7.06015292	6.89277003	6.98816212	5200	6181	6082
23759	NM_001317996,NM_014337,NM	PPIL2	peptidylprolyl isomerase like 2	-1.108445955	-1.051577885	13.829833	12.699747	13.285404	5.64697758	5.50154961	5.57590188	1928	2324	2255
6834	NM_001280787,NM_003172	SURF1	SURF1, cytochrome c oxidase as	1.182691052	-1.051594097	35.774728	42.459171	32.064256	5.22293093	5.45904106	5.15233896	1427	2255	1669
23479	NM_001301140,NM_001301141	ISCU	iron-sulfur cluster assembly enzy	-1.711867865	-1.051719265	21.18576	12.162491	20.435241	5.68247162	4.92670685	5.61116998	1977	1543	2312
200894	NM_001174150,NM_001174151	ARL13B	ADP ribosylation factor like GTPa	-1.433987555	-1.051758758	4.845468	3.462322	4.402126	4.29607932	3.80748641	4.22706077	732	682	856
51499	NM_016399	TRIAP1	TP53 regulated inhibitor of apopt	1.052025885	-1.051765329	14.892262	15.676107	13.303158	4.18126603	4.250509	4.11255209	673	946	787
11062	NM_001270419,NM_181581,NR	DUS4L	dihydrouridine synthase 4 like	-1.661855987	-1.052175218	5.883569	3.626058	5.36398	3.81942505	3.15255179	3.75135692	515	414	602
54454	NM_001242338,NM_017552,NR	ATAD2B	ATPase family, AAA domain cont	-1.319562975	-1.052186217	3.092709	2.347267	2.764631	4.66827758	4.28618731	4.59783705	959	971	1121
23142	NM_001040402,NM_001287755	DCUN1D4	defective in cullin neddylation 1 d	1.2316725	-1.052204227	13.795243	16.98916	12.221604	5.89869115	6.19476657	5.82653464	2303	3790	2692
10072	NM_001256670,NM_005700,NM	DPP3	dipeptidyl peptidase 3	1.30194345	-1.05229285	16.562549	21.512144	14.741932	5.65793614	6.03197907	5.58588904	1943	3380	2271
8556	NM_001319210,NM_001319211	CDC14A	cell division cycle 14A	-1.264743922	-1.052312478	0.949494	0.753467	0.927972	2.34351507	2.07783221	2.28470378	160	169	187
55011	NM_017916	PIH1D1	PIH1 domain containing 1	1.014513444	-1.052445709	46.063461	46.77813	41.116695	5.82994114	5.85036724	5.75752106	2194	2974	2564
5306	NM_006224	PITPNA	phosphatidylinositol transfer prote	1.140767755	-1.052475942	7.411523	8.467307	6.618275	4.77009871	4.95358253	4.69907543	1032	1573	1206
124976	NM_001124758	SPNS2	sphingolipid transporter 2	-2.775982094	-1.052524315	44.881256	16.182589	40.070667	7.24325756	5.78702662	7.16990263	5909	2844	6905
8372	NM_001200029,NM_001200030	HYAL3	hyaluronoglucosaminidase 3	-1.39839799	-1.052658556	4.331862	4.270355	3.979097	3.0724647	2.65526948	3.00740444	291	278	340
126299	NM_182498	ZNF428	zinc finger protein 428	-1.260294065	-1.052769146	7.798992	6.19423	6.963536	3.53555296	3.23373217	3.46789548	416	441	486
4351	NM_001289155,NM_001289156	MPI	mannose phosphate isomerase	1.3645823	-1.052930635	7.107094	9.66677	6.263318	4.44353723	4.87422123	4.37262206	815	1486	952
100216545	NR_024586	KMT2E-AS1	KMT2E antisense RNA 1 (head t	-1.373821431	-1.052969828	2.632316	1.918005	2.348896	3.35699061	2.95035633	3.28994455	363	353	424
7703	NM_007144	PCGF2	polycomb group ring finger 2	-1.005272849	-1.053016691	34.912762	34.771328	31.156883	6.48442128	6.47691888	6.41074453	3476	4620	4060
8195	NM_018848,NM_170784,NR_07	MKKS	McKusick-Kaufman syndrome	1.307417187	-1.053070006	17.700216	21.0115	13.68166	5.4617788	5.84080171	5.38890871	1691	2954	1975
9862	NM_001079518,NM_001267797	MED24	mediator complex subunit 24	-1.017484307	-1.053090368	23.162109	22.687818	20.625211	6.4124769	6.38776564	6.33874398	3305	4340	3860
57827	NM_021184	C6orf47	chromosome 6 open reading fran	-1.032451982	-1.053178172	6.106653	5.92083	5.443429	3.97474042	3.93163239	3.90484845	578	748	675
84645	NM_001207062,NM_032561	C22orf23	chromosome 22 open reading fra	3.449013912	-1.053182635	0.078827	0.274322	0.060327	0.20517559	0.61708849	0.19530369	6	28	7
132228	NM_001304385,NM_153215	LSMEM2	leucine rich single-pass membrar	-1.328298723	-1.053182635	0.094496	0.076372	0.084839	0.20517559	0.15624221	0.19530369	6	6	7
8344	NM_003523	HIST1H2BE	histone cluster 1 H2B family men	2.346034781	-1.053182635	0.355486	0.814448	0.289121	0.20517559	0.44594451	0.19530369	6	19	7
101930094	NR_135518	LOC101930094	uncharacterized LOC101930094	-1.969526583	-1.053182635	0.310688	0.151703	0.277971	0.20517559	0.10601836	0.19530369	6	4	7
100507290	NM_001195605	ZNF865	zinc finger protein 865	1.115636393	-1.053286371	6.772272	7.565883	6.042318	5.07270439	5.22613324	5.00008264	1282	1911	1497
79188	NM_024334	TMEM43	transmembrane protein 43	1.335564269	-1.05337268	19.284004	25.787615	17.206602	5.99459805	6.40636677	5.9207858	2464	4397	2877
387032	NM_001304506,NM_019110	ZKSCAN4	zinc finger with KRAB and SCAN	-1.04818054	-1.053557125	1.134314	1.082228	1.010397	2.79384765	2.73592201	2.72964665	233	297	272
91614	NM_001077242,NM_139160	DEPDC7	DEP domain containing 7	-2.176613466	-1.053605347	3.286494	1.714554	3.329234	2.88620833	1.97664665	2.82126674	251	154	293
7159	NM_001031685,NM_005426	TP53BP2	tumor protein p53 binding protein	1.015661295	-1.05363253	12.753172	12.89842	11.400154	5.88258712	5.9046304	5.80852249	2277	3090	2658
2073	NM_000123	ERCC5	ERCC excision repair 5, endonuc	-1.362150805	-1.05365262	20.091526	14.766054	17.922342	6.33857304	5.89911068	6.26412715	3138	3078	3663
10542	NM_006402	LAMTOR5	late endosomal/lysosomal adapto	-1.378552125	-1.053663853	74.130226	53.826065	66.101913	5.95900671	5.50458181	5.8848325	2403	2329	2805
100616114	NR_039814	MIR4668	microRNA 4668	1.616113525	-1.053701334	4.240569	7.080981	3.887392	0.38477185	0.58077243	0.36729312	12	26	14
84909	NM_001193329,NM_001193331	C9orf3	chromosome 9 open reading fran	1.102706686	-1.053775466	5.719769	6.160156	5.147523	4.18732982	4.32100453	4.11600233	676	996	789
23677	NM_014521	SH3BP4	SH3 domain binding protein 4	1.432880861	-1.0538637	10.450734	14.994715	9.318462	5.74821069	6.25902004	5.67396328	2071	3965	2417
724033	NR_030386	MIR663A	microRNA 663a	3.312626129	-1.053876489	4.932569	16.659185	4.379415	0.54446555	1.33632702	0.52094563	18	80	21
199720	NM_152657	GGN	gametogenetin	-1.84076809	-1.053876489	0.215371	0.110372	0.193853	0.54446555	0.31941552	0.52094563	18	13	21
221468	NM_001162900,NM_001286401	TMEM217	transmembrane protein 217	-1.45463322	-1.053964496	0.302176	0.206302	0.287023	0.68823103	0.50527715	0.65979727	24	22	28
85014	NM_032928	TMEM141	transmembrane protein 141	-1.068893034	-1.054040877	49.50581	46.362587	44.141003	5.40480367	5.31102396	5.33070673	1624	2030	1895
440278	NR_002318	CATSPER2P1	cation channel sperm associated	1.122339954	-1.054078071	0.668547	0.754975	0.594401	1.04948198	1.1381576	1.01060517	42	63	49
55328	NM_001031709,NM_018363	RNLS	renalase, FAD dependent amine	-1.05640632	-1.054167485	1.098916	1.046812	0.979525	1.84721644	1.79043244	1.79260867	102	129	119
331	NM_001167,NM_001204401,NR	XIAP	X-linked inhibitor of apoptosis	1.215437342	-1.054226554	5.977175	6.837025	5.131778	5.49719403	5.77301226	5.42273519	1734	2816	2023
23310	NM_015261	NCAPD3	non-SMC condensin II complex s	-1.684230688	-1.054339991	18.943922	11.260356	16.883156	6.70580139	5.96311165	6.63020989	4059	3220	4735
347902	NM_001143668,NM_181847	AMIGO2	adhesion molecule with Ig like do	1.383154293	-1.054392549	44.700395	62.894298	40.622885	7.37039922	7.83595062	7.29445996	6457	11933	7532
285464	NM_175918	CRIPAK	cysteine rich PAK1 inhibitor	-1.49210553	-1.054407431	9.79225	6.570249	8.729995	5.43908863	4.87796738	5.36445917	1664	1490	1941
8864	NM_022817	PER2	period circadian clock 2	1.616565623	-1.054466121	5.154678	8.344447	4.592785	5.03733324	5.71345739	4.96320452	1250	2700	1458
80119	NM_001286496,NM_001286497	PIF1	PIF1 5'-to-3' DNA helicase	-1.016071718	-1.055025502	14.811523	14.685896	13.27656	5.30050562	5.27808999	5.22523491	1508	1983	1758
55425	NM_001316951,NM_001316952	GPALPP1	GPALPP motifs containing 1	1.110021073	-1.055067873	11.314936	11.713938	9.637087	4.25817433	4.40128969	4.18497252	712	1056	830
10318	NM_001252385,NM_001252386	TNIP1	TNFAIP3 interacting protein 1	-1.12971238	-1.055153763	47.739612	42.158566	42.441047	7.064552	6.88999002	6.98769137	5216	6169	6080
79884	NM_001039580	MAP9	microtubule associated protein 9	-1.203367817	-1.055343925	2.392911	1.989662	2.130507	4.1772093	3.92621521	4.10389022	671	745	782
54778	NM_001270528,NM_001270529	RNF111	ring finger protein 111	-1.064091959	-1.055358906	5.843313	5.500091	5.208385	5.12418641	5.03720514	5.04873431	1330	1670	1550
80349	NM_001303247,NM_001303248	WDR61	WD repeat domain 61	1.165653826	-1.055381387	30.986322	36.290545	27.611233	5.35359695	5.569727	5.2777797	1566	2439	1825
221154	NM_152726	MICU2	mitochondrial calcium uptake 2	-1.251702402	-1.055402672	19.065155	15.249479	16.975582	5.20511061	4.89100283	5.12947602	1409	1504	1642
54543	NM_019059	TOMM7	translocase of outer mitochondria	-1.183087522	-1.055426641	80.213524	67.880074	71.41684	5.95071282	5.71240871	5.8741751	2389	2698	2784
4150	NM_001042539,NM_001276275	MAZ	MYC associated zinc finger prote	1.298380979	-1.055469062	49.698198	65.333409	46.029932	6.99970098	7.37382876	6.9224401	4985	8648	5809
35	NM_000017,NM_001302554	ACADS	acyl-CoA dehydrogenase, C-2 to	-1.799656722	-1.055768149	5.201471	2.894588	4.62993	3.44397655	2.69827959	3.37303661	388	288	452

51616	NM_015975	TAF9B	TATA-box binding protein associ	-1.007609195	-1.055776654	7.544212	7.496155	6.718383	4.37612225	4.36571317	4.30167645	776	1029	904
9683	NM_153029	N4BP1	NEDD4 binding protein 1	1.014927704	-1.055846848	6.851739	6.965103	6.099359	5.59774578	5.6186857	5.52100322	1862	2525	2169
23353	NM_001130965,NM_001171944	SUN1	Sad1 and UNC84 domain contair	-1.201718448	-1.055880881	73.730722	61.85141	65.077461	8.1303867	7.86632313	8.05222647	10962	12188	12769
9796	NM_001099335,NM_014759	PHYHIP	phytanoyl-CoA 2-hydroxylase inte	-1.110924271	-1.056056071	0.619024	0.556753	0.549647	1.59082596	1.49109792	1.53866543	79	95	92
27258	NM_014463	LSM3	LSM3 homolog, U6 small nuclear	1.072688375	-1.056078241	45.940166	49.356312	40.887169	5.00451141	5.10270203	4.92830565	1221	1750	1422
200030	NM_001101663,NM_001101663	NBPF11	NBPF member 11	1.269663788	-1.056260318	5.902496	7.967938	5.280438	4.96387793	5.29849962	4.88750351	1186	2012	1381
27297	NM_001040647,NM_001040648	CRCP	CGRP receptor component	-1.333785613	-1.056302325	10.737434	8.049019	9.767323	4.93533846	4.53542228	4.85896034	1162	1164	1353
6189	NM_001006,NM_001267699	RPS3A	ribosomal protein S3A	1.03208626	-1.056396123	851.821659	883.136036	763.455759	9.63344341	9.67895064	9.55439464	31143	42946	36259
79733	NM_001256371,NM_001256372	E2F8	E2F transcription factor 8	-1.277214313	-1.056402068	17.275316	13.470423	15.261528	5.97428428	5.62761379	5.89641527	2429	2541	2828
9590	NM_005100,NM_144497	AKAP12	A-kinase anchoring protein 12	-1.179780076	-1.056488591	3.814335	3.236108	3.390187	4.98026891	4.74992223	4.90356387	1200	1359	1397
114825	NM_001130864,NM_001267035	PWWP2A	PWWP domain containing 2A	1.124012709	-1.056547436	9.920695	10.65729	8.609066	5.16060006	5.32481356	5.08351504	1365	2050	1589
81847	NM_001242844,NM_001242845	RNF146	ring finger protein 146	-1.17946488	-1.056675705	8.4446	6.823857	7.235479	4.3072593	4.08211055	4.23183903	738	836	859
6461	NM_003028	SHB	SH2 domain containing adaptor p	1.616450653	-1.056700381	6.357037	10.292768	5.656276	5.25888453	5.93731246	5.18144687	1464	3162	1704
100130581	NR_027412,NR_027413	LINC00910	long intergenic non-protein codin	-1.532890791	-1.056839685	1.503645	1.027048	1.348543	2.42103729	1.94127715	2.35646843	171	149	199
10669	NM_001166239,NM_001166240	CGREF1	cell growth regulator with EF-han	1.003505437	-1.05692349	6.246806	6.424727	5.484701	3.62762016	3.63226192	3.55435929	446	598	519
984	NM_001291345,NM_001787,NM	CDK11B	cyclin dependent kinase 11B	-1.051947618	-1.057066965	32.471991	30.799798	28.298341	6.58277284	6.51048949	6.50356227	3724	4730	4333
375690	NR_033266	WASH5P	WAS protein family homolog 5 ps	1.241417722	-1.057077032	9.688934	12.051376	8.623781	3.55444243	3.84241755	3.48133381	422	700	491
128272	NM_153213	ARHGEF19	Rho guanine nucleotide exchang	1.068027078	-1.057158969	20.169874	21.565229	17.925114	6.05774429	6.15131615	5.97878582	2576	3676	2997
105370941	NR_135665	LOC105370941	uncharacterized LOC105370941	1.602472317	-1.057174484	0.47247	0.755112	0.417999	1.16869613	1.58576189	1.12461302	49	105	57
440590	NM_001004339,NM_001307931	ZYG11A	zyg-11 family member A, cell cyc	-1.421949198	-1.057174484	0.294894	0.20937	0.26661	1.16869613	0.90836632	1.12461302	49	46	57
283232	NM_001042463,NM_001276253	TMEM80	transmembrane protein 80	1.557835429	-1.0573654	9.158148	14.31014	8.163756	3.97005831	4.5763457	3.89484064	576	1199	670
6205	NM_001015	RPS11	ribosomal protein S11	-1.266376382	-1.057429326	990.46521	782.998962	880.221313	9.28533831	8.94524614	9.20490947	24458	25805	28448
10926	NM_001318060,NM_001318061	DBF4	DBF4 zinc finger	-1.64182607	-1.057449937	15.252242	9.271057	13.510779	5.86567124	5.16612031	5.78649854	2250	1831	2617
11112	NM_152740	HIBADH	3-hydroxyisobutyrate dehydroger	-1.844027866	-1.057584511	28.276175	15.351096	25.12377	5.80780809	4.94646449	5.7285132	2160	1565	2512
56126	NM_018930	PCDHB10	protocadherin beta 10	1.443548672	-1.057585543	0.341596	0.497167	0.301188	1.06712766	1.36863803	1.02535867	43	83	50
6150	NM_021134	MRPL23	mitochondrial ribosomal protein L	1.25977234	-1.05773886	43.955627	55.439014	39.037552	5.03397229	5.35805128	4.95552185	1247	2099	1450
11277	NM_007248,NM_016381,NM_03	TREX1	three prime repair exonuclease 1	-1.115605707	-1.058098734	12.336298	11.002312	11.401052	4.63464885	4.48350191	4.55653526	936	1121	1088
8106	NM_004643	PABPN1	poly(A) binding protein nuclear 1	1.216440184	-1.058180815	80.016579	97.440689	71.058769	7.9228512	8.20446127	7.84160989	9488	15421	11028
9205	NM_001039649,NM_001039650	ZMYM5	zinc finger MYM-type containing	-1.153691575	-1.058404573	8.997562	7.96114	8.397459	4.34572074	4.15029781	4.26795589	759	879	882
200035	NM_001012758	NUDT17	nudix hydrolase 17	-1.163840173	-1.058440928	3.393646	2.922956	3.012452	2.20692365	2.03820547	2.1430785	142	163	165
26036	NM_001031623,NM_001257273	ZNF451	zinc finger protein 451	-1.081453221	-1.058496321	8.514931	7.676772	8.009802	5.60908326	5.49851102	5.52879006	1877	2319	2181
8428	NM_001032296,NM_001286649	STK24	serine/threonine kinase 24	-2.085996878	-1.058521206	19.520927	9.34806	17.326561	6.47453768	5.43126519	6.39343391	3452	2211	4011
7469	NM_005663	NELFA	negative elongation factor comple	-1.085742026	-1.058540754	34.563618	31.871773	30.680927	6.39336957	6.276155	6.31229424	3261	4013	3789
1906	NM_001168319,NM_001955	EDN1	endothelin 1	-2.040044156	-1.058627314	1.694065	0.827039	1.491907	2.15835987	1.43116693	2.09493548	136	89	158
54429	NM_018980	TAS2R5	taste 2 receptor member 5	1.218846121	-1.058997957	1.403262	1.704658	1.250666	1.36692851	1.5486383	1.31676564	62	101	72
379	NM_001661	ARL4D	ADP ribosylation factor like GTPa	1.015728013	-1.059014585	6.231412	6.338737	5.534184	3.40980893	3.43022535	3.33505242	378	513	439
9790	NM_014753	BMS1	BMS1, ribosome biogenesis facto	-1.247199244	-1.059035851	11.763866	9.440471	10.437416	6.54194749	6.22706742	6.46010721	3619	3877	4203
5216	NM_005022	PFN1	profilin 1	1.007168797	-1.059071329	401.87793	405.205536	356.593292	9.06367459	9.07396095	8.98103364	20969	28218	24352
90355	NM_001316968,NM_001316969	C5orf30	chromosome 5 open reading fran	1.094471196	-1.059101514	6.313774	6.99938	5.670283	4.31835331	4.44234896	4.2397678	744	1088	864
26173	NM_001080453	INTS1	integrator complex subunit 1	-1.146100846	-1.059201862	17.917919	15.651903	15.896693	6.93870314	6.74368163	6.85641965	4777	5569	5547
10170	NM_001142270,NM_001142271	DHRS9	dehydrogenase/reductase 9	2.20898697	-1.059242862	21.630617	46.607626	18.622907	5.30050562	6.42377037	5.2196331	1508	4451	1751
59283	NM_031895	CACNG8	calcium voltage-gated channel au	-2.405665234	-1.059261411	2.783239	1.157561	2.468144	4.62723337	3.44039495	4.54762018	931	517	1081
54535	NM_001105563,NM_001105564	CCHCR1	coiled-coil alpha-helical rod prote	-1.119781402	-1.059279761	15.187846	12.616894	13.475525	5.31811369	5.15920994	5.23716605	1527	1822	1773
54819	NM_001300816,NM_001300817	ZCCHC10	zinc finger CCHC-type containing	1.038736798	-1.059291466	9.240594	9.550532	8.278549	4.37612225	4.42836625	4.29712434	776	1077	901
79016	NM_024050	DDA1	DET1 and DDB1 associated 1	-1.208249432	-1.059518217	19.823832	16.426128	17.584297	6.31661142	6.04744731	6.23427685	3090	3417	3587
54935	NM_001319658,NM_001319659	DUSP23	dual specificity phosphatase 23	1.007343179	-1.05965398	18.603067	18.851023	16.770695	3.85541331	3.86524379	3.77773686	529	712	614
10494	NM_001271977,NM_001271978	STK25	serine/threonine kinase 25	1.506565807	-1.059661608	37.387243	56.121534	32.856966	6.54313011	7.12919695	6.46044653	3622	7291	4204
79661	NM_001256552,NM_024608,NR	NEIL1	nei like DNA glycosylase 1	-1.006213409	-1.05966677	4.27648	4.275219	3.776645	3.13236099	3.1244438	3.05850609	305	405	354
100652758	NR_132345,NR_132346	LOC100652758	uncharacterized LOC100652758	1.293203033	-1.05975021	1.783177	2.321638	1.586766	1.61502061	1.87534115	1.5590879	81	140	94
79850	NM_001318006,NM_001318007	FAM57A	family with sequence similarity 57	1.279524813	-1.05993677	11.011987	14.208698	9.773325	4.66103338	5.00416403	4.58045927	954	1631	1107
128977	NM_001166242,NM_173793	C22orf39	chromosome 22 open reading fra	1.007384784	-1.059965563	7.505669	7.431767	6.44362	3.70855202	3.71835988	3.63111814	474	638	550
402415	NM_212559	XKRX	XK related, X-linked	-2.371921053	-1.060003739	0.224439	0.095846	0.203179	0.71085839	0.3412894	0.6785857	25	14	29
160418	NM_181783	TMTC3	transmembrane and tetratricopep	-1.129106181	-1.060151411	5.011006	4.441427	4.44057	5.17592889	5.00587698	5.09405028	1380	1633	1601
102724312	NR_125994,NR_125995,NR_12	LINC01770	long intergenic non-protein codin	-1.672221755	-1.060493058	3.348619	1.872434	2.903445	2.22275497	1.67459501	2.15654364	144	115	167
3984	NM_001204426,NM_002314	LIMK1	LIM domain kinase 1	-1.572818709	-1.0605232	25.132358	15.995558	22.266352	6.38811426	5.74456841	6.30437724	3249	2760	3768
283659	NM_173814	PRTG	protogenin	-1.012662922	-1.060540924	0.87883	0.868244	0.778503	3.50674676	3.49019467	3.42958826	407	537	472
55722	NM_018140	CEP72	centrosomal protein 72	1.184544093	-1.060541786	7.523457	8.927471	6.67273	4.22515306	4.45744984	4.14500092	695	1100	806
3281	NM_001537	HSBP1	heat shock factor binding protein	1.003115262	-1.060547636	51.70599	51.932396	45.821152	6.66038669	6.6648299	6.57643811	3932	5270	4560
127703	NM_001348691,NM_152374	C1orf216	chromosome 1 open reading fran	1.06882047	-1.060570811	7.868969	8.461508	6.981469	4.55403551	4.64610598	4.47289912	883	1261	1024
23172	NM_032182	ABRAXAS2	abraxas 2, BRISC complex subui	-1.093408215	-1.060703547	7.556584	6.924348	6.697139	4.52559963	4.40259064	4.44436461	865	1057	1003
23433	NM_012249	RHOQ	ras homolog family member Q	1.12805525	-1.060808708	3.970357	4.485975	3.519129	4.21529532	4.38031337	4.13483255	690	1040	800
5192	NM_002617,NM_153818	PEX10	peroxisomal biogenesis factor 10	1.122637829	-1.060937447	12.230269	13.80803	10.889015	4.65229204	4.81292083	4.57043432	948	1422	1099
658	NM_001203,NM_001256792,NM	BMPR1B	bone morphogenetic protein rece	-3.89172733	-1.060955837	0.202373	0.052568	0.181245	1.08456011	0.36283659	1.03996283	44	15	51
55711	NM_001271783,NM_001271784	FAR2	fatty acyl-CoA reductase 2	-1.090541409	-1.061242367	3.106606	2.848806	2.75142	3.64828273	3.53359042	3.56953804	453	555	525
10949	NM_006805	HNRNPA0	heterogeneous nuclear ribonucle	1.228380486	-1.061425778	63.536644	78.129982	56.246845	7.52782731	7.82313459	7.44230257	7206	11827	8350
10045	NM_005490	SH2D3A	SH2 domain containing 3A	1.032414172	-1.061440381	53.222317	55.012283	47.12286	6.94378647	6.98944134	6.85848091	4794	6613	5555
4052	NM_000627,NM_001166264,NM	LTBP1	latent transforming growth factor	1.620902492	-1.061477666	1.146316	1.908809	1.026749	2.98234021	3.60765556	2.9074142	271	587	314
50628	NM_015721	GEMIN4	gem nuclear organelle associated	1.258944715	-1.061661074	7.982113	10.061006	7.065598	4.91111656	5.23346232	4.82773844	1142	1921	1323
653121	NM_001040441,NM_001291496	ZBTB8A	zinc finger and BTB domain conta	-1.128998415	-1.061834533	1.415187	1.266892	1.259016	3.47735365	3.31887893	3.39875952	398	471	461
5795	NM_001098503,NM_002843	PTPRJ	protein tyrosine phosphatase, rec	-1.231459532	-1.061854376	10.814848	8.985234	9.871033	6.31338057	6.01719271	6.2279131	3083	3345	3571
80017	NM_001102366,NM_001102367	C14orf159	chromosome 14 open reading fra	1.034952822	-1.061927864	23.058354	22.261277	19.20689	5.93578265	5.98455365	5.85055151	2364	3269	2738
155368	NM_152559	METTL27	methyltransferase like 27	-1.46332166	-1.062087129	1.579545	1.084924	1.398306	1.29387258	0.99371033	1.24294651	57	52	66
113235	NM_001242366,NM_080669	SLC46A1	solute carrier family 46 member 1	1.491675906	-1.062114828	4.119491	6.150662	3.646776	4.75248416	5.31171658	4.66885564	1019	2031	1180
158228	NR_027294	FAM201A	family with sequence similarity 20	-3.617706094	-1.062151918	1.721218	0.465572	1.525552	1.96484452	0.84852988	1.90057841	114	42	132
84245	NM_001031727,NM_001329572	MRI1	methylthioribose-1-phosphate isc	1.120290009	-1.062242548	13.05425	14.646735	11.600644	5.37500681	5.53515162	5.29004963	1590	2380	1841
283373	NM_173595	ANKRD52	ankyrin repeat domain 52	1.166023632	-1.062251779	11.88797	13.877675	10.516932	6.66111308	6.88068447	6.57487194	3934	6129	4555
10587	NM_001282512,NM_006440	TXNRD2	thioredoxin reductase 2	1.537487285	-1.062326401	26.418037	40.665132	23.370005	5.79197777	6.40344564	5.70636801	2136	4388	2473
56928	NM_001077238,NM_152988	SPPL2B	signal peptide peptidase like 2B	1.124462873	-1.062331044	17.116987	19.263132	15.155781	5.99920131	6.16594551	5.91336906	2472	3714	2862
523	NM_001690	ATP6V1A	ATPase H+ transporting V1 subu	1.540363345	-1.062744561	22.33552	34.448532	19.750097	6.65674927	7.27500897	6.56984871	3922	8072	4539
5694	NM_001270481,NM_002798	PSMB6	proteasome subunit beta 6	-1.148503139	-1.062904715	98.808438	86.132783	87.346237	6.37533433	6.17815094	6.28841171	3220	3746	3726
435	NM_000048,NM_001024943,NM	ASL	argininosuccinate lyase	-1.847693062	-1.062954097	15.992375	8.944057	14.611046	5.02044968	4.1718205	4.93515799	1235	893	1429
7188	NM_001033910,NM_001319207	TRAF5	TNF receptor associated factor 5	-1.089205577	-1.063015547	12.197463	11.212136	10.803803	5.60077783	5.48014385	5.51448193	1866	2289	2159
55851	NM_001281532,NM_172341	PSENEN	presenilin enhancer gamma-secr	-1.360734105	-1.063130094	50.853175	37.317484	44.819324	5.90912433	5.4733502	5.82231668	2320	2278	2684
3014	NM_002105	H2AFX	H2A histone family member X	-1.865542604	-1.063236044	134.59758	72.217384	118.957962	7.71260404	6.81893335	7.62457574	8196	5870	9481
283464	NM_001099650,NM_173601	GXYLT1	glucoside xylosyltransferase 1	-1.169906958	-1.063322305	5.077248	4.313148	4.468709	5.23175921	5.01185634	5.14560245	1436	1640	1661
2264	NM_001291980,NM_002011,NM	FGFR4	fibroblast growth factor receptor 4	1.909260605	-1.063324104	4.773298	8.394122	3.892845	3.82462152	4.70844643	3.74245532	517	1319	598
84304	NM_001128612,NM_001128613	NUDT22	nudix hydrolase 22	-1.010312926	-1.063340069	23.736702	23.580389	21.253424	4.70255223	4.68831976	4.61744439	983	1300	1137
23339	NM_001301138,NM_015289	VPS39	VPS39, HOPS complex subunit	-1.018995463	-1.063401098	16.236531	15.94585	14.341942	6.28114034	6.25434409	6.19362678	3014	3952	3486
10681	NM_006578,NM_016194	GNB5	G protein subunit beta 5	1.236499856	-1.063464725	5.046896	6.25074	4.457514	3.96771155	4.25627716	3.88476293	575	950	665
51123	NM_001042510,NM_001267708	ZNF706	zinc finger protein 706	-1.061019218	-1.063522209	18.695615	17.638354	16.525473	5.69458464	5.61082803	5.60749789	1994	2511	2306
839	NM_001226,NM_032992,NR_13	CASP6	caspase 6	1.237700138	-1.063562943	9.124054	11.249606	8.018047	3.95355025	4.24326622	3.8705348	569	941	658
286077	NM_198488	FAM83H	family with sequence similarity 83	-1.006077229	-1.063566972	43.656212	43.440666	38.570099	7.90026394	7.89155948	7.81173579	9340	12404	10801
4686	NM_001351504,NM_001351505	NCBP1	nuclear cap binding protein subu	1.271263371	-1.06358503	21.824774	27.793282	19.294358	6.84751475	7.19110937	6.75937345	4482	7613	5183
84073	NM_032133	MYCBPAP	MYCBP associated protein	-1.985370643	-1.063609293	0.521381	0.261428	0.461649	1.39514558	0.86372326	1.34055586	64	43	74
57148	NM_001282917,NM_001282918	RALGAPB	Ral GTPase activating protein no	1.006175997	-1.063670654	11.028151	11.109604	9.77046	6.55529403	6.5640828	6.46721612	3653	4911	4224
55758	NM_001136223,NM_001136224	RCOR3	REST corepressor 3	-1.010579994	-1.063736702	15.649172	15.756123	13.965733	5.94595195	5.93101533	5.85829744	2381	3148	2753
79613	NM_024562	TANGO6	transport and golgi organization 6	1.24731127	-1.063791315	1.806283	2.256358	1.597174	3.24517298	3.53359042	3.16560381	333	555	385
92345	NM_001128931,NM_138386	NAF1	nuclear assembly factor 1 ribonu	1.492390394	-1.063872009	4.758168	7.150296	4.234849	3.29469475	3.82311587	3.21470347	346	690	400
51070	NM_001270960,NM_015953	NOSIP	nitric oxide synthase interacting p	-1.093908417	-1.063947035	43.09473	38.826701	37.693275	5.74341713	5.61644502	5.65570694	2064	2521	2386

1479	NM_001033505,NM_001033506	CSTF3	cleavage stimulation factor subur	1.176062757	-1.06407992	49.570264	45.909231	39.179578	6.06654372	6.29729141	5.97831199	2592	4073	2996
81550	NM_001146070,NM_001146071	TDRD3	tudor domain containing 3	-1.570781363	-1.064351567	4.749077	2.838499	4.278273	3.76640418	3.17404113	3.68321628	495	421	572
37	NM_000018,NM_001033859,NM	ACADVL	acyl-CoA dehydrogenase, very lo	1.020211208	-1.064461002	149.639553	152.780191	132.438007	8.39895634	8.42773975	8.30910794	13213	18011	15267
389668	NM_001011720,NM_001287258	XKR9	XK related 9	-1.239516717	-1.064491113	0.833653	0.679555	0.742165	1.8573935	1.63971536	1.79260867	103	111	119
5058	NM_001128620,NM_002576	PAK1	p21 (RAC1) activated kinase 1	-1.057333321	-1.064511587	21.122332	20.00899	18.689983	6.16610643	6.08682444	6.07720657	2780	3513	3212
8514	NM_001199860,NM_001199861	KCNAB2	potassium voltage-gated channel	-2.141174093	-1.064747044	1.857333	0.86909	1.63808	3.12395512	2.20257223	3.04408938	303	189	350
171391	NR_126342,NR_126343	LOC171391	uncharacterized LOC171391	-1.117178235	-1.06475342	7.902719	7.210916	7.122396	2.85607368	2.71931318	2.77836293	245	293	283
30832	NM_014594	ZNF354C	zinc finger protein 354C	1.100380465	-1.06495121	3.229437	3.560825	2.850441	4.27914521	4.41037179	4.19316234	723	1063	835
26100	NM_001033518,NM_001033519	WIPI2	WD repeat domain, phosphoinos	-1.171889686	-1.064958847	12.32577	10.579385	10.955862	5.76655235	5.54225135	5.67747023	2098	2392	2423
6458	NR_033412	SH3GL1P1	SH3 domain containing GRB2 lik	-1.079936991	-1.065131628	1.012058	0.937915	0.890553	1.79522562	1.71704117	1.73094093	97	120	112
9076	NM_021101	CLDN1	claudin 1	-1.015749649	-1.065165077	82.989777	81.791893	73.215714	8.12894345	8.10647953	8.03820126	10951	14405	12645
338692	NM_001347901,NM_207354	ANKRD13D	ankyrin repeat domain 13D	1.007832525	-1.065207992	24.644489	24.826125	21.4835	5.72477705	5.73582152	5.63541459	2037	2743	2352
55096	NM_018029	EBLN2	endogenous Bornavirus-like nucl	1.599789216	-1.065303073	1.922253	3.100069	1.698137	2.04717061	2.58825016	1.97845985	123	263	142
374786	NM_001145053,NM_198529,NR	EFCAB5	EF-hand calcium binding domain	1.892524869	-1.065343245	0.06127	0.164513	0.063104	0.41264541	0.70406985	0.39027104	13	33	15
80736	NM_001178044,NM_001178045	SLC44A4	solute carrier family 44 member 4	-2.783126455	-1.065493367	6.432178	2.307647	5.815741	4.12971807	2.79259185	4.04356865	648	311	748
2744	NM_001256310,NM_014905	GLS	glutaminase	1.661216511	-1.065512551	35.135086	58.382591	31.042676	7.29143725	8.02001707	7.20049115	6111	13564	7054
202781	NR_028090	PAXIP1-AS1	PAXIP1 antisense RNA 1 (head t	-1.764951782	-1.065637015	8.294082	4.701309	7.306759	4.27345604	3.50964299	4.18661421	720	545	831
94	NM_000020,NM_001077401	ACVRL1	activin A receptor like type 1	6.282378874	-1.065642208	0.156011	1.039146	0.139596	0.73313634	2.37153003	0.69713258	26	219	30
100134259	NR_024452	LINC01119	long intergenic non-protein codin	1.437496299	-1.065642208	0.561715	0.805553	0.492142	0.73313634	0.96581954	0.69713258	26	50	30
93621	NM_001272053,NM_001272054	MRFAP1	Morf4 family associated protein 1	1.126345373	-1.065722371	155.19754	174.758758	137.339918	8.36034172	8.53149982	8.26879773	12863	19358	14845
219654	NM_153367	ZCCHC24	zinc finger CCHC-type containing	2.043086213	-1.065893442	0.824869	1.693152	0.729004	2.3142594	3.18919702	2.24113096	156	426	180
23398	NM_001278926,NM_001278927	PPWD1	peptidylprolyl isomerase domain	-1.084555694	-1.065965957	36.21902	31.936118	30.4142	6.20995367	6.09449202	6.11907368	2867	3532	3308
6633	NM_004597,NM_177542	SNRPD2	small nuclear ribonucleoprotein D	-1.334199368	-1.065973406	256.656815	192.684439	226.188909	7.47861048	7.06532963	7.38697106	6963	6973	8034
28992	NM_014067	MACROD1	MACRO domain containing 1	-1.410298809	-1.066118217	24.508228	17.389206	21.61017	4.95442736	4.47734157	4.86512445	1178	1116	1359
9697	NM_012288	TRAM2	translocation associated membra	1.23021768	-1.066146115	5.064538	6.240884	4.464259	5.16265334	5.45403052	5.07290231	1367	2247	1577
4176	NM_001278595,NM_005916,NM	MCM7	minichromosome maintenance co	-1.137084824	-1.066149602	64.865153	57.667923	57.61479	7.59740797	7.41308616	7.50548937	7564	8888	8726
10950	NM_001130914,NM_006806	BTG3	BTG anti-proliferation factor 3	-1.292370941	-1.066178876	47.357557	36.628175	41.590877	6.11877104	5.75479142	6.02769088	2689	2780	3102
692196	NR_003942	SNORD76	small nucleolar RNA, C/D box 76	1.140272127	-1.066192618	81.892693	93.467697	72.207947	2.90104285	3.06652959	2.82126674	254	387	293
8834	NM_003876,NR_024547	TMEM11	transmembrane protein 11	1.00173876	-1.066371954	17.531142	17.188947	14.704811	4.69547851	4.69788845	4.60644807	978	1309	1128
7869	NM_001005914,NM_001290060	SEMA3B	semaphorin 3B	-2.195415541	-1.066517048	17.372599	8.473432	14.887161	5.58631849	4.48718552	5.49540175	1847	1124	2130
240	NM_000698,NM_001256153,NM	ALOX5	arachidonate 5-lipoxygenase	-1.827441549	-1.066532603	20.063481	10.986259	17.356707	5.64036226	4.79420929	5.5493518	1919	1403	2213
9448	NM_001242559,NM_001242560	MAP4K4	mitogen-activated protein kinase	1.319379813	-1.066764488	58.031888	76.947474	50.760427	8.67648264	9.07549109	8.58347566	16024	28248	18475
286151	NM_001029860,NR_036491	FBXO43	F-box protein 43	1.288541574	-1.066820809	0.558005	0.727281	0.491522	1.50279892	1.75012126	1.4430085	72	124	83
152992	NM_001350233,NM_152544	TRMT44	tRNA methyltransferase 44 homo	-1.107097769	-1.066870475	7.0126	6.334603	6.152437	4.34572074	4.20649667	4.2570592	759	916	875
3572	NM_001190981,NM_002184,NM	IL6ST	interleukin 6 signal transducer	1.410073328	-1.066898543	10.813069	15.168908	9.555734	6.5800868	7.07147709	6.48766934	3717	7003	4285
8621	NM_003718,NM_031267	CDK13	cyclin dependent kinase 13	-1.234862212	-1.067060645	10.320274	8.38159	9.117462	6.14133197	5.84176112	6.04905586	2732	2956	3149
9100	NM_001272075,NM_005153,NR	USP10	ubiquitin specific peptidase 10	1.272667699	-1.067071465	38.547504	48.767448	33.606198	6.97916821	7.32457866	6.88627598	4914	8356	5664
22980	NM_014972	TCF25	transcription factor 25	1.207257479	-1.067215397	40.099823	48.463009	35.305492	6.47907607	6.74803794	6.38630822	3463	5586	3991
51390	NM_001286587,NM_001286588	AIG1	androgen induced 1	1.337032363	-1.06725178	68.378723	93.770719	55.648886	6.71107214	7.1266407	6.6180947	4074	7278	4695
84083	NM_001286568,NM_001286569	ZRANB3	zinc finger RANBP2-type contain	1.420694538	-1.067273666	1.183699	1.739696	1.055462	3.17367098	3.63226192	3.09042641	315	598	363
284996	NM_173647	RNF149	ring finger protein 149	1.592717399	-1.067612127	36.054054	57.490917	31.735302	6.71702247	7.38341431	6.62355913	4091	8706	4713
56897	NM_020135,NM_130395	WRNIP1	Werner helicase interacting prote	1.012526723	-1.067699356	21.616944	21.911658	19.004079	5.82411536	5.84176112	5.7313279	2185	2956	2517
55785	NM_018351	FGD6	FYVE, RhoGEF and PH domain c	-1.879722545	-1.06777644	18.804928	10.014415	16.550203	7.41844793	6.51530806	7.32440814	6677	4746	7691
115196	NM_001102651	ZNF554	zinc finger protein 554	-1.056416655	-1.067851057	4.190589	3.972248	3.688282	4.00938005	3.93523257	3.92071794	593	750	683
400793	NM_001085375,NM_001135240	C1orf226	chromosome 1 open reading fran	1.122616774	-1.068005295	2.383306	2.785245	2.096119	3.40287721	3.55480842	3.31717816	376	564	433
65999	NM_001142928,NM_023942	LRRC61	leucine rich repeat containing 61	1.576827339	-1.068153785	5.099432	8.054873	4.485748	3.42699388	4.03423402	3.34096163	383	807	441
90990	NM_145754	KIFC2	kinesin family member C2	-1.141712269	-1.068261838	55.837711	48.956097	49.11451	7.4802578	7.2902001	7.38554235	6971	8158	8026
27107	NM_014415	ZBTB11	zinc finger and BTB domain conta	1.27491179	-1.068343992	12.872796	16.42832	11.3211	6.0259528	6.37158123	5.93208459	2519	4291	2900
286128	NM_001271156,NM_173832	ZFP41	ZFP41 zinc finger protein	1.250340245	-1.068618202	3.646215	4.365464	3.14824	3.98637962	4.29041001	3.89684777	583	974	671
7133	NM_001066	TNFRSF1B	TNF receptor superfamily membe	1.366966748	-1.068641846	6.931292	9.485194	6.093762	4.68979447	5.12572903	4.59783705	974	1779	1121
57583	NM_020823	TMEM181	transmembrane protein 181	1.007344886	-1.068685111	13.717684	13.830811	12.060436	6.1914686	6.20188276	6.09698315	2830	3809	3257
10560	NM_001319667,NM_006996	SLC19A2	solute carrier family 19 member 2	-1.244658709	-1.068727197	7.343958	6.029476	6.471039	4.75520813	4.45243375	4.66297087	1021	1096	1175
54069	NM_018944	MIS18A	MIS18 kinetochore protein A	-1.150786334	-1.068735938	21.196732	18.439268	18.627317	5.06284359	4.86669962	4.96989347	1273	1478	1465
11020	NM_001177701,NM_006860,NR	IFT27	intraflagellar transport 27	1.293504211	-1.068969713	29.319135	38.005192	25.839247	5.00565571	5.36674241	4.91251987	1222	2112	1406
56666	NM_001160300,NM_052839,NR	PANX2	pannexin 2	-1.124597835	-1.069097164	1.155844	1.031172	0.998112	2.13345087	2.0043309	2.05953665	133	158	153
10293	NM_005879	TRAIP	TRAF interacting protein	1.124547952	-1.069097501	7.551197	8.50806	6.645024	3.99100913	4.15029781	3.90085366	585	879	673
23192	NM_013325,NM_178326	ATG4B	autophagy related 4B cysteine pe	1.264628224	-1.0691657	28.519282	36.107535	25.074615	6.33902704	6.67401657	6.24377015	3139	5304	3611
26263	NM_012170,NM_147188,NR_03	FBXO22	F-box protein 22	-1.107859224	-1.069219505	15.587966	13.926643	13.700422	5.66736669	5.5226424	5.57276666	1956	2359	2250
51205	NM_001323625,NM_016361,NR	ACP6	acid phosphatase 6, lysophospha	1.173838539	-1.069288647	25.204387	31.582352	23.608844	5.6535627	5.88055759	5.55889016	1937	3038	2228
53637	NM_001166215,NM_030760	S1PR5	sphingosine-1-phosphate recepto	2.540555563	-1.069301213	0.425379	1.081119	0.372529	1.01352966	1.84504029	0.96541723	40	136	46
56624	NM_001143974,NM_019893	ASAH2	N-acylsphingosine amidohydrolas	-3.319576843	-1.069301213	0.428608	0.126412	0.374651	1.01352966	0.38406669	0.96541723	40	16	46
51295	NM_001142464,NM_001142465	ECSIT	ECSIT signalling integrator	-1.16890332	-1.069322171	24.444683	20.721537	21.117541	5.32821046	5.10909111	5.23399405	1538	1758	1769
155400	NR_033322,NR_104013	NSUN5P1	NOP2/Sun RNA methyltransferas	-1.167519378	-1.06936937	19.572836	16.810706	17.246162	5.18807576	4.97122547	5.09405028	1392	1593	1601
51463	NM_001350180,NM_001350181	GPR89B	G protein-coupled receptor 89B	1.095163974	-1.06941952	7.281531	8.036435	6.424435	4.05430925	4.17791132	3.96347983	613	897	705
101927045	NR_110032	LOC101927045	uncharacterized LOC101927045	1.396723842	-1.069439566	1.478705	2.072645	1.294604	2.02024721	2.3978615	1.94780992	120	224	138
55565	NM_001201552,NM_001201553	ZNF821	zinc finger protein 821	-1.116709172	-1.069479197	3.78381	3.66261	3.310088	3.02359429	2.88484352	2.93892502	280	335	322
653689	NM_001080843,NM_001080843	GSTT2B	glutathione S-transferase theta 2	1.695599002	-1.069487024	9.08669	15.41407	7.9776	3.41670749	4.12214544	3.3291189	380	861	437
26750	NM_001136138,NM_001287218	RPS6KC1	ribosomal protein S6 kinase C1	-1.102223257	-1.069562739	7.39059	7.107834	6.619765	5.36076893	5.22392723	5.26618109	1574	1908	1810
400569	NM_001001683,NM_001305000	MED11	mediator complex subunit 11	-1.378125989	-1.069796898	8.574433	6.383533	7.681782	3.08551061	2.68551066	2.99995417	294	285	338
118672	NM_153336	PSTK	phosphoseryl-tRNA kinase	1.031280952	-1.069809289	1.932724	2.001218	1.708282	2.0823043	2.11639955	2.00847214	127	175	146
738	NM_013265,NR_073519	VPS51	VPS51, GARP complex subunit	1.188245238	-1.069849371	19.19772	22.846057	16.860155	5.71641462	5.96090566	5.62091677	2025	3215	2328
7625	NM_001256523,NM_001256524	ZNF74	zinc finger protein 74	1.037080685	-1.070069921	8.85688	9.185398	7.711419	5.10509529	5.15612801	5.01031597	1312	1818	1508
3421	NM_004135,NM_174869	IDH3G	isocitrate dehydrogenase 3 (NAD	1.121694786	-1.070142695	32.171724	36.21673	28.906433	5.67673599	5.8393614	5.5809041	1969	2951	2263
129642	NM_001321265,NM_001321266	MBOAT2	membrane bound O-acyltransfera	1.103703481	-1.070254735	11.474769	12.825174	10.058718	6.43516152	6.57595105	6.3383748	3358	4952	3859
377007	NM_198582	KLHL30	kelch like family member 30	-1.448685363	-1.07043928	0.997147	0.686513	0.876845	2.19894236	1.79836126	2.12264217	141	130	162
26993	NM_001291478,NM_014371,NR	AKAP8L	A-kinase anchoring protein 8 like	1.007974878	-1.070506679	49.438975	49.879664	43.460489	6.75119512	6.76254913	6.65384259	4190	5643	4814
2926	NM_001098477,NM_002092	GRSF1	G-rich RNA sequence binding fac	1.17241111	-1.070543691	18.598375	22.086557	16.336117	6.92001067	7.14774095	6.82250478	4715	7386	5417
54936	NM_017825	ADPRHL2	ADP-ribosylhydrolase like 2	1.067951568	-1.070682571	36.087311	38.587048	31.676222	5.91705205	6.01038243	5.82020306	2333	3329	2680
4155	NM_001025081,NM_001025090	MBP	myelin basic protein	-1.557926036	-1.070689997	33.231572	21.173038	29.227031	7.26133439	6.62693041	7.16345625	5984	5132	6874
6203	NM_001013,NM_001321701,NM	RPS9	ribosomal protein S9	-1.105078425	-1.070731979	576.218466	525.009534	500.866035	8.67252543	8.52874714	8.57417739	15980	19321	18356
6646	NM_001252511,NM_001252512	SOAT1	sterol O-acyltransferase 1	1.227676395	-1.070790333	8.893231	10.936476	7.810048	5.93036974	6.22191816	5.83336264	2355	3863	2705
102724668	NR_132360	DPY19L1P2	DPY19L1 pseudogene 2	-1.385392244	-1.070918516	0.599684	0.42821	0.518776	0.7550755	0.58077243	0.71544405	27	26	31
129285	NM_001135629,NM_001193475	PPP1R21	protein phosphatase 1 regulatory	1.139696542	-1.070991129	8.498349	9.74983	7.463808	4.76875136	4.95091738	4.67354624	1031	1570	1184
55769	NM_001105549,NM_001105550	ZNF83	zinc finger protein 83	-1.635027751	-1.0710849	42.279028	25.894855	36.730447	6.9133192	6.21156417	6.8150945	4693	3835	5389
11079	NM_007033	RER1	retention in endoplasmic reticulur	1.014165704	-1.071100942	61.831779	62.778351	54.25037	7.55367935	7.57386578	7.45512921	7337	9942	8425
56882	NM_001038707,NM_020239	CDC42SE1	CDC42 small effector 1	1.136679119	-1.071170229	29.031669	32.976641	25.37954	6.44742452	6.63026582	6.34940945	3387	5144	3889
692205	NR_003070	SNORD89	small nucleolar RNA, C/D box 89	1.140808771	-1.071659596	13.875882	15.931211	12.214472	1.35261029	1.47139626	1.29257653	61	93	70
84274	NM_032314	COQ5	coenzyme Q5, methyltransferase	-1.258960009	-1.07180473	29.133085	23.172466	25.538483	5.45341658	5.12966232	5.35573003	1681	1784	1929
5339	NM_000445,NM_201378,NM_20	PLEC	plectin	1.111576022	-1.071815185	150.606977	167.83124	132.203553	11.08673874	11.23927896	10.98673008	85348	126759	97939
28976	NM_014049,NR_033426	ACAD9	acyl-CoA dehydrogenase family r	1.742867036	-1.07183364	16.203128	27.664411	14.114343	5.38383466	6.17053465	5.28622648	1600	3726	1836
115110	NR_037844	LOC115110	uncharacterized LOC115110	-1.310633157	-1.07184357	0.880746	0.673396	0.771562	2.3142594	2.01116974	2.23479744	156	159	179
9462	NM_004841,NM_170692	RASAL2	RAS protein activator like 2	-1.132187136	-1.071909486	13.548426	11.778099	11.547296	7.11764422	6.93990061	7.01820583	5413	6388	6211
83892	NM_001317395,NM_001317399	KCTD10	potassium channel tetramerizatio	-1.092499615	-1.071962464	14.525437	13.389129	12.73551	5.90545064	5.78003646	5.80692233	2314	2830	2655
107303344	NM_001320727,NR_135324	SETDB2-PHF11	SETDB2-PHF11 readthrough	1.846409271	-1.071996727	0.223881	0.417041	0.195936	0.89996265	1.37924953	0.85412573	34	84	39
55343	NM_001145265,NM_001145266	SLC35C1	solute carrier family 35 member C	-1.168962124	-1.072005588	8.750819	7.540424	7.583685	4.92569844	4.70844643	4.82879013	1154	1319	1324
115416	NM_138446	MALSU1	mitochondrial assembly of riboso	-1.429309975	-1.072063876	37.164585	26.031174	32.569427	4.8242841	4.33060465	4.72754788	1073	1003	1231
29035	NM_014117	C16orf72	chromosome 16 open reading fra	1.411279751	-1.07210851	7.992332	11.293519	7.006851	4.99877622	5.48260636	4.90156607	1216	2293	1395
80777	NM_030579	CYB5B	cytochrome b5 type B	-1.120038005	-1.072259513	34.322754	30.681444	30.080399	7.17242262	7.01007171	7.07248896	5624	6709	6451
26160	NM_015662	IFT172	intraflagellar transport 172	-1.014198044	-1.072294631	5.110637	5.044286	4.475184	4.81778406	4.79816878	4.72076557	1068	1407	1225

53407	NM_001346281,NM_001346282	STX18	syntaxin 18	-1.112970697	-1.072313573	19.604547	17.635957	17.179867	5.4065374	5.25594871	5.30826105	1626	1952	1865
3965	NM_001330163,NM_002308,NM	LGALS9	galectin 9	-1.364554694	-1.072339356	17.417171	12.906836	15.260966	4.92085417	4.48963605	4.82352399	1150	1126	1319
7518	NM_001318012,NM_001318013	XRCC4	X-ray repair cross complementing	-1.1981157	-1.072402613	13.363981	11.580427	11.867781	4.50308873	4.25483728	4.40682806	851	949	976
101928891	NR_110537	LINC01637	long intergenic non-protein codin	1.661012828	-1.072423683	4.139823	6.900869	3.627559	1.91699278	2.48886356	1.84344662	109	242	125
3489	NM_002178	IGFBP6	insulin like growth factor binding	-1.444624401	-1.072503556	196.85289	136.414032	172.483505	7.54031781	7.01305581	7.43989558	7269	6723	8336
161253	NM_173527	REM2	RRAD and GEM like GTPase 2	1.025912693	-1.07308004	1.247739	1.282944	1.089666	1.70793493	1.73367602	1.63800401	89	122	102
84335	NM_001098632,NM_001098633	AKT1S1	AKT1 substrate 1	1.052521923	-1.073170926	30.222435	29.105068	25.817504	5.98302523	6.05573998	5.88280858	2444	3437	2801
8100	NM_001318491,NM_001318493	IFT88	intraflagellar transport 88	1.332813046	-1.073184282	2.588643	3.339151	2.131019	3.17367098	3.54777037	3.08339379	315	561	361
100133220	NM_001310153	GOLGA6L3	golgin A6 family-like 3	-1.830699553	-1.073306181	1.491016	0.8095	1.306137	1.78459878	1.22307764	1.71282633	96	70	110
55249	NM_001198899,NM_001198900	YY1AP1	YY1 associated protein 1	-1.051528698	-1.073360657	36.069865	33.203918	31.381189	6.64394559	6.5721985	6.54286557	3887	4939	4454
83855	NM_031918	KLF16	Kruppel like factor 16	1.242464822	-1.073365603	13.352625	16.607599	11.688411	5.26844986	5.57435204	5.16904389	1474	2447	1689
6155	NM_000988,NM_001349921,NM	RPL27	ribosomal protein L27	-1.014995428	-1.073423238	971.611698	961.815752	876.468559	9.03734005	9.01590789	8.93532204	20589	27103	23591
64225	NM_001135673,NM_001308076	ATL2	atlastin GTPase 2	1.669544729	-1.073503582	18.922437	31.675961	16.425085	6.38679744	7.11934035	6.28573352	3246	7241	3719
22821	NM_001320821,NM_001320822	RASA3	RAS p21 protein activator 3	1.021040537	-1.07351757	5.07316	5.756447	4.931312	4.58957318	4.61838139	4.49161333	906	1236	1038
4141	NM_004990	MARS	methionyl-tRNA synthetase	-1.24374147	-1.073527056	84.944496	68.367149	74.355988	7.91936414	7.6061254	7.81744245	9465	10168	10844
1763	NM_001080449,NR_102264	DNA2	DNA replication helicase/nucleas	-1.732823951	-1.073567277	8.189995	4.758295	7.144166	5.12629213	4.36304264	5.0269064	1332	1027	1526
3570	NM_000565,NM_001206866,NM	IL6R	interleukin 6 receptor	1.0184268	-1.07358911	0.745817	0.710899	0.630645	2.32896139	2.35011326	2.2474368	158	215	181
392862	NM_001145118	GRID2IP	Grid2 interacting protein	-3.655352696	-1.073591961	0.307971	0.081898	0.269365	1.26358189	0.46599441	1.20457043	55	20	63
5606	NM_001316332,NM_002756,NM	MAP2K3	mitogen-activated protein kinase	1.290432015	-1.073605201	29.345289	37.686476	25.62868	6.06873521	6.43175704	5.96784829	2596	4476	2974
84068	NM_001029998,NM_001300842	SLC10A7	solute carrier family 10 member 7	-1.03289772	-1.073609869	2.863678	2.886776	2.594522	3.54502861	3.50238056	3.45160249	419	542	480
9126	NM_005445	SMC3	structural maintenance of chromo	1.017893057	-1.073681898	25.181059	25.659328	22.039543	6.66980129	6.69513886	6.56827536	3958	5383	4534
216	NM_000689	ALDH1A1	aldehyde dehydrogenase 1 family	-1.007793396	-1.073891444	98.564186	97.916359	86.254364	7.83513526	7.82398446	7.73275252	8926	11834	10223
57405	NM_020675	SPC25	SPC25, NDC80 kinetochore com	1.068290646	-1.073939671	18.592371	19.879421	16.268179	4.66538426	4.75705948	4.56665693	957	1366	1096
7336	NM_003350	UBE2V2	ubiquitin conjugating enzyme E2	-1.077877386	-1.073986991	54.371738	50.501575	47.579987	6.3521312	6.24530905	6.25045698	3168	3927	3628
64135	NM_022168	IFIH1	interferon induced with helicase C	-1.175114289	-1.07398736	12.410272	10.573256	10.855865	5.47588426	5.24873326	5.37529684	1708	1942	1956
51399	NM_001318486,NM_001318488	TRAPPC4	trafficking protein particle comple	-1.614171915	-1.074083005	24.939875	15.838743	22.132276	4.99532414	4.33197089	4.89555604	1213	1004	1389
116828	NR_026928	N4BPL2L2-IT2	N4BPL2 intronic transcript 2	-1.169063271	-1.074228772	2.581909	2.209087	2.258287	3.7336304	3.52644784	3.63833419	483	552	553
51111	NM_001300907,NM_001300908	KMT5B	lysine methyltransferase 5B	1.169351266	-1.074282838	14.198277	16.711237	15.515359	5.72894015	5.95071445	5.6275797	2043	3192	2339
10953	NM_006809	TOMM34	translocase of outer mitochondria	1.168669615	-1.074295818	26.260067	30.720987	22.968943	5.73860759	5.95958045	5.63721662	2057	3212	2355
100130557	NR_024567	NFYC-AS1	NFYC antisense RNA 1	1.547071402	-1.074354497	2.437351	3.778603	2.13502	3.09843961	3.66732006	3.00740444	297	614	340
7329	NM_003345,NM_194259,NM_19	UBE2I	ubiquitin conjugating enzyme E2	1.128347602	-1.074727729	55.520423	62.748194	48.793042	7.32115463	7.49434244	7.2178555	6239	9406	7140
10273	NM_001293197,NM_005861	STUB1	STIP1 homology and U-box cont	1.230361185	-1.074792486	38.150755	47.718353	33.580688	5.95959731	6.25434409	5.85726705	2404	3952	2751
102466740	NR_106866	MIR6808	microRNA 6808	-1.03859314	-1.074902353	2.855833	2.737889	2.601817	0.23670415	0.2284445	0.22116494	7	9	8
56125	NM_018931	PCDHB11	protocadherin beta 11	2.226552004	-1.074902353	0.051577	0.125227	0.046719	0.23670415	0.48576948	0.22116494	7	21	8
80254	NM_001042383,NM_001042384	CEP63	centrosomal protein 63	1.386737918	-1.075188236	5.730534	8.468123	5.870595	4.64790143	5.10350222	4.54762018	945	1751	1081
149345	NM_198149,NR_030775	SHISA4	shisa family member 4	1.122569693	-1.075296525	3.004445	2.83963	2.201873	2.34351507	2.47903109	2.25996639	160	240	183
134549	NM_001172700,NM_133456	SHROOM1	shroom family member 1	-1.087822127	-1.075340124	10.710572	9.787461	9.348847	5.37146049	5.25306686	5.26928318	1586	1948	1814
56164	NM_001260504,NM_001260505	STK31	serine/threonine kinase 31	-1.168345145	-1.075517004	3.163043	2.710361	2.607875	3.51962058	3.3161202	3.42403181	411	470	470
374897	NM_001166034,NM_001166035	SBSN	suprabasin	2.554180924	-1.075542351	0.183898	0.621279	0.15356	0.43999062	0.93737891	0.41288872	14	48	16
100506874	NR_038273,NR_038274	LINC00933	long intergenic non-protein codin	-1.858298909	-1.075542351	0.130715	0.072896	0.115327	0.43999062	0.25173066	0.41288872	14	10	16
8674	NM_001185127,NM_003762,NR	VAMP4	vesicle associated membrane pro	-1.209037216	-1.075575408	5.347303	4.381603	4.500284	4.55716073	4.29602113	4.45666286	885	978	1012
3898	NM_005558	LAD1	ladinin 1	1.053824344	-1.075616795	36.669437	38.689423	32.036236	6.67771978	6.75263621	6.57361777	3980	5604	4551
196528	NM_001347839,NM_152641	ARID2	AT-rich interaction domain 2	-1.484219268	-1.075677974	5.17279	3.431837	4.689924	5.36879502	4.81585323	5.26618109	1583	1425	1810
101928063	NR_110727	LOC101928063	uncharacterized LOC101928063	-6.807061619	-1.075758121	0.244191	0.031429	0.213587	0.61813834	0.10601836	0.58209675	21	4	24
7515	NM_006297	XRCC1	X-ray repair cross complementing	-1.077853684	-1.075794363	25.446926	23.637688	22.227579	5.73032519	5.62427223	5.62697525	2045	2535	2338
54957	NM_001142317,NM_001142318	TXNL4B	thioredoxin like 4B	1.415213788	-1.075886454	5.103618	7.263859	4.738468	3.6915876	4.15956116	3.59448638	468	885	535
2043	NM_001304536,NM_001304537	EPHA4	EPH receptor A4	5.652492646	-1.075931627	0.13858	0.787961	0.12134	0.91952222	2.597368	0.87055986	35	265	40
5143	NM_000923,NM_001098818,NM	PDE4C	phosphodiesterase 4C	-1.559026041	-1.076006552	3.919243	2.450813	3.204432	4.36368147	3.76158491	4.26329596	769	659	879
8999	NM_001330724,NM_003948	CDKL2	cyclin dependent kinase like 2	-1.507355175	-1.076062278	0.365374	0.244464	0.320085	1.47662927	1.12560759	1.40965954	70	62	80
256355	NR_026676	RPS2P32	ribosomal protein S2 pseudogen	-1.094339706	-1.076074178	1.958716	1.792121	1.707184	1.56621863	1.48128072	1.4969322	77	94	88
5814	NM_033224	PURB	purine rich element binding prote	-1.048235761	-1.076081022	7.123615	6.801206	6.219725	6.00149745	5.93461705	5.89741808	2476	3156	2830
84662	NM_001318918,NM_032575	GLIS2	GLIS family zinc finger 2	-1.308509437	-1.076098721	10.702975	8.194514	9.352253	5.30979981	4.93302278	5.20674668	1518	1550	1735
100288911	NR_037631	LOC100288911	uncharacterized LOC100288911	-1.458101458	-1.076116207	4.338627	2.958566	3.785321	2.01115992	1.62195412	1.93223726	119	109	136
23087	NM_001304495,NM_171982	TRIM35	tripartite motif containing 35	1.360903487	-1.076157903	1.595083	2.177294	1.413953	2.92543304	3.31887893	2.83389297	259	471	296
80212	NM_001304957,NM_001304958	CCDC92	coiled-coil domain containing 92	-1.130542983	-1.07636867	9.020236	7.594487	7.670049	4.26392396	4.09665142	4.16345881	715	845	817
54963	NM_001193379,NM_017859,NR	UCKL1	uridine-cytidine kinase 1 like 1	1.072799195	-1.076441503	29.689008	32.072005	25.919256	5.78932242	5.88893602	5.68503952	2132	3056	2436
81502	NM_030789,NM_178580,NM_17	HM13	histocompatibility minor 13	1.229453623	-1.076560048	134.2599	165.26557	116.925652	7.75063896	8.04740893	7.64472148	8416	13825	9615
7385	NM_003366	UQCRC2	ubiquinol-cytochrome c reductase	1.067618848	-1.07658479	97.346779	104.052994	84.975372	7.7181991	7.81216297	7.61226026	8228	11737	9400
55740	NM_001008493,NM_018212	ENAH	enabled homolog (Drosophila)	1.542406877	-1.076732893	5.420001	8.38489	4.737545	6.13872666	6.75671129	6.03363206	2727	5620	3115
58485	NM_001166621,NM_021210,NR	TRAPPC1	trafficking protein particle comple	1.081391776	-1.076801949	50.653592	54.200009	43.430926	5.32362978	5.43381248	5.2196331	1533	2215	1751
26086	NM_001145638,NM_001145639	GPSM1	G protein signaling modulator 1	1.105719154	-1.076863145	10.439095	11.980968	9.637727	5.18908339	5.3302927	5.08527627	1393	2058	1591
55973	NM_001008405,NM_018844,NR	BCAP29	B-cell receptor associated protei	-1.582085694	-1.076910195	8.191421	5.156625	7.141255	5.60002041	4.95535656	5.49540175	1865	1575	2130
10040	NM_001321173,NM_001321174	TOM1L1	target of myb1 like 1 membrane t	-1.316687241	-1.077101925	28.939611	22.169414	25.539336	6.02030239	5.63039253	5.91485546	2509	2546	2865
2671	NM_005262	GFER	growth factor, augmenter of liver	-1.177443412	-1.077142293	16.84313	14.313687	14.69005	5.34005262	5.11068396	5.23558092	1551	1760	1771
147804	NR_003148	TPM3P9	tropomyosin 3 pseudogene 9	-1.014934992	-1.077192453	3.518931	3.473733	3.074535	3.46742135	3.44797538	3.37014999	395	520	451
79847	NM_024789	MFSD13A	major facilitator superfamily doma	-1.566524574	-1.077289654	3.320314	2.122864	2.897954	3.34619087	2.77662633	3.24967672	360	307	411
5269	NM_001195291,NM_001271822	SERPINB6	serpin family B member 6	-1.093668725	-1.077320183	43.346321	38.927116	38.239196	6.32075477	6.19326394	6.21469853	3099	3786	3538
51377	NM_001199261,NM_001199262	UCHL5	ubiquitin C-terminal hydrolase L5	1.243338231	-1.077384794	18.201228	21.081106	12.998637	6.06654372	6.37655495	5.96067018	2592	4306	2959
8853	NM_001135191,NM_003887	ASAP2	ArfGAP with SH3 domain, ankyri	-1.036173124	-1.077666026	34.329106	33.122951	29.941495	7.56653064	7.51554009	7.45920977	7403	9546	8449
7391	NM_001276373,NM_007122,NM	USF1	upstream transcription factor 1	1.021658783	-1.07773857	27.887366	28.517778	24.345891	5.63519596	5.66549558	5.52943707	1912	2610	2182
4088	NM_001145102,NM_001145103	SMAD3	SMAD family member 3	-1.512306927	-1.077788318	94.391115	62.617152	84.684693	9.16278782	8.56732229	9.0549092	22463	19846	25634
205564	NM_001308045,NM_152699	SENP5	SUMO1/sentrin specific peptidas	1.253522636	-1.077848608	15.930463	19.91021	13.945886	6.61911412	6.94213892	6.5120984	3820	6398	4359
1968	NM_001415	EIF2S3	eukaryotic translation initiation fa	-1.068360006	-1.077869938	66.089119	61.931965	57.62096	7.80718797	7.71222916	7.69950493	8754	10948	9989
7705	NM_001099638,NM_001099639	ZNF146	zinc finger protein 146	-1.286087752	-1.078150775	47.970777	37.279161	41.711615	7.48989801	7.12919695	7.38196436	7018	7291	8006
3308	NM_002154	HSPA4	heat shock protein family A (Hsp7	1.070035008	-1.078162498	93.05574	99.684853	81.104706	8.26536943	8.36272136	8.15716018	12041	17215	13736
51061	NM_001303447,NM_001324022	TXNDC11	thioredoxin domain containing 11	1.06200922	-1.078188447	13.896612	14.444055	12.061654	5.33823707	5.42295529	5.23240543	1549	2198	1767
83605	NM_001029835,NM_001167934	CCM2	CCM2 scaffolding protein	-1.210845691	-1.078212028	25.003324	20.757215	22.062459	5.54834575	5.2787986	5.44210713	1798	1984	2051
2483	NM_004477	FRG1	FSHD region gene 1	-1.066208429	-1.07835479	35.8522678	33.662388	31.251945	5.2258797	5.13593336	5.12005661	1430	1792	1631
79868	NM_001039210,NM_001099922	ALG13	ALG13, UDP-N-acetylglucosamir	-1.05695626	-1.078523038	26.697165	25.611818	20.680282	6.30410949	6.22523051	6.19648136	3063	3872	3493
339500	NM_178549,NR_033184,NR_10	ZNF678	zinc finger protein 678	-1.754601143	-1.078632001	2.056235	1.333594	1.934974	3.90531607	3.16487058	3.80364308	549	418	626
64708	NM_001282949,NM_001282950	COPS7B	COP9 signalosome subunit 7B	1.228345935	-1.078732398	23.940986	29.577478	21.00631	5.91705205	6.20933576	5.80958828	2333	3829	2660
51340	NM_001278625,NM_001278626	CRNKL1	crooked neck pre-mRNA splicing	1.273629176	-1.078755463	12.217079	15.573927	10.642819	5.61958513	5.96222966	5.51251978	1891	3218	2156
8648	NM_003743,NM_147223,NM_14	NCOA1	nuclear receptor coactivator 1	1.275723205	-1.078798638	2.718264	3.476198	2.36775	4.27535493	4.61052206	4.17177132	721	1229	822
653390	NR_003369	RRN3P2	RRN3 homolog, RNA polymerase	1.399836619	-1.078991416	1.011556	1.415847	0.879402	1.75224037	2.11004283	1.67590018	93	174	106
54979	NM_017878	HRASLS2	HRAS like suppressor 2	-1.909681668	-1.078991416	3.244065	1.711107	2.830128	1.75224037	1.16293478	1.67590018	93	65	106
27304	NM_014484	MOCS3	molybdenum cofactor synthesis 3	1.573722582	-1.079015232	3.245215	5.119416	2.826471	3.41326233	4.01734947	3.31417746	379	797	432
23476	NM_001330384,NM_014299,NM	BRD4	bromodomain containing 4	1.085648821	-1.079100925	16.427146	17.897384	14.303903	6.32351041	6.44064994	6.21510076	3105	4504	3539
5499	NM_001008709,NM_002708,NM	PPP1CA	protein phosphatase 1 catalytic s	1.10251032	-1.079118244	106.176563	117.296785	92.303074	7.25220377	7.39211817	7.14309721	5946	8759	6777
219541	NM_001317078,NM_153450	MED19	mediator complex subunit 19	1.071139921	-1.079394181	11.525395	12.46428	10.118845	3.89547266	3.98818428	3.7929053	545	780	621
10018	NM_001204106,NM_001204107	BCL2L11	BCL2 like 11	1.359883542	-1.079533377	9.72305	12.476305	8.072999	5.46427804	5.89911068	5.35645948	1694	3078	1930
374659	NM_001286451,NM_198527,NR	HDDC3	HD domain containing 3	-1.408432486	-1.079534227	16.229566	11.563831	14.389465	4.03201954	3.57341041	3.9285877	603	572	687
23221	NM_001160036,NM_001160037	RHOBTB2	Rho related BTB domain contain	1.182691284	-1.079568038	2.481808	2.9356	2.160051	3.82462152	4.05092325	3.72222359	517	817	589
319089	NM_001310135	TTC6	tetratricopeptide repeat domain 6	-2.997350578	-1.079808978	0.271999	0.090726	0.236651	1.50279892	0.68708806	1.43197761	72	32	82
2700	NM_021954	GJA3	gap junction protein alpha 3	-1.296426681	-1.079821145	1.480241	1.142528	1.288103	3.08983317	2.76453517	2.99246523	295	304	336
1639	NM_001135040,NM_001135041	DCTN1	dynactin subunit 1	1.3238222	-1.079909466	29.601835	39.27206	25.863586	6.99653892	7.39848953	6.88652852	4974	8798	5665
83549	NM_001135954,NM_001261450	UCK1	uridine-cytidine kinase 1	1.160650261	-1.079958709	12.974374	15.023387	11.230029	4.8242841	5.03217088	4.71736241	1073	1664	1222
201294	NM_199242	UNC13D	unc-13 homolog D	-1.517055358	-1.080113852	124.732864	82.30822	108.520859	9.06140661	8.46152463	8.95043892	20936	18439	23840
23461	NM_018672,NM_172232	ABCA5	ATP binding cassette subfamily A	-1.860943904	-1.080166366	3.54776	1.9084	3.087121	5.04291747	4.18397654	4.93515799	1255	901	1429
55585	NM_017582	UBE2Q1	ubiquitin conjugating enzyme E2	1.051196108	-1.080196579	20.782345	21.870783	18.079773	6.04944579	6.12041926	5.93989276	2561	3597	2916

54896	NM_001040125,NM_001040126	PQLC2	PQ loop repeat containing 2	1.280395743	-1.080283538	10.00638	13.091905	9.582697	4.45698495	4.79915696	4.35082401	823	1408	937
91689	NM_033318,NR_146715,NR_14	SMDT1	single-pass membrane protein wi	1.087839187	-1.080458903	7.408956	8.057154	6.409354	3.61267788	3.72461474	3.51046477	441	641	502
116984	NM_015230,NR_146893,NR_14	ARAP2	ArfGAP with RhoGAP domain, ar	-1.356172421	-1.080462844	6.924767	5.095299	6.037343	5.66953424	5.24002681	5.56015719	1959	1930	2230
55742	NM_018222	PARVA	parvin alpha	-1.194426723	-1.080720105	6.169365	5.172982	5.366345	5.73860759	5.4875188	5.62878784	2057	2301	2341
23417	NM_012213	MLYCD	malonyl-CoA decarboxylase	-1.135070072	-1.080741462	4.569771	4.035886	3.977276	3.43040645	3.26553914	3.3291189	384	452	437
7508	NM_004628,NR_027299	XPC	XPC complex subunit, DNA dama	-1.083707589	-1.080946734	6.576839	6.076674	5.718536	4.63464885	4.52351345	4.52703356	936	1154	1065
29098	NM_001177801,NM_001177802	RANGRF	RAN guanine nucleotide release	1.303963448	-1.080989611	4.279179	5.611458	3.689099	2.39332696	2.71093651	2.30298238	167	291	190
84146	NM_016620,NM_032186,NM_20	ZNF644	zinc finger protein 644	1.259813011	-1.081000073	6.136569	8.08351	5.826358	5.05180751	5.37604391	4.94294958	1263	2126	1437
51075	NM_001144012,NM_001347890	TMX2	thioredoxin related transmembrai	1.196157009	-1.081294994	73.469271	86.832275	63.264042	6.91970718	7.17616385	6.80791265	4714	7534	5362
54505	NM_001345964,NM_001345965	DHX29	DExH-box helicase 29	-1.244281611	-1.081308984	14.250333	11.522153	12.418363	6.04944579	5.7394296	5.93843194	2561	2750	2913
1389	NM_001310	CREBL2	cAMP responsive element bindin	-1.509352387	-1.08135707	6.990959	4.634855	6.072028	4.75248416	4.18548887	4.64397663	1019	902	1159
57610	NM_001320238,NM_001320239	RANBP10	RAN binding protein 10	1.213381806	-1.081539324	4.883924	6.148487	4.395635	4.77413325	5.04389026	4.66532766	1035	1678	1177
85015	NM_001080481,NM_001346021	USP45	ubiquitin specific peptidase 45	-1.04257456	-1.081595976	5.04945	4.448885	3.964281	4.45865714	4.40128969	4.35082401	824	1056	937
83593	NM_182663,NM_182664,NM_18	RASSF5	Ras association domain family m	-2.476895348	-1.081735131	11.055442	4.431548	9.497632	5.30330016	4.04760082	5.19292756	1511	815	1718
5352	NM_000935,NM_182943	PLOD2	procollagen-lysine,2-oxoglutarate	1.940782767	-1.081865031	26.011264	50.493918	22.580959	6.68630885	7.63615947	6.57393141	4004	10383	4552
677825	NR_002976	SNORA44	small nucleolar RNA, H/ACA box	-1.049557354	-1.08189138	4.281129	4.032029	3.669581	0.64188297	0.61708849	0.60191771	22	28	25
124930	NM_152345	ANKRD13B	ankyrin repeat domain 13B	-1.108615112	-1.081915046	6.021845	5.438914	5.234185	4.29420754	4.15339221	4.18661421	731	881	831
6584	NM_001308122,NM_003060	SLC22A5	solute carrier family 22 member 5	-1.287881446	-1.08197177	10.434818	8.053088	9.0365	5.10188873	4.74889974	4.99165559	1309	1358	1488
30	NM_001130410,NM_001607,NR	ACAA1	acetyl-CoA acyltransferase 1	-1.164773623	-1.082119159	47.330307	40.116852	41.093954	6.42364978	6.20635918	6.3111659	3331	3821	3786
116841	NM_001323930,NM_001323931	SNAP47	synaptosome associated protein	1.010787648	-1.082146163	11.286677	10.35384	8.960589	4.62574568	4.64060352	4.51662904	930	1256	1057
284900	NR_026962,NR_026963	TTC28-AS1	TTC28 antisense RNA 1	-1.131469877	-1.082146549	5.940856	5.550041	5.229178	2.98234021	2.8278811	2.88332104	271	320	308
80007	NM_024942	C10orf88	chromosome 10 open reading fra	-1.073295768	-1.082243488	4.525846	4.22826	3.930441	3.87056622	3.77571017	3.76460718	535	666	608
10024	NM_001100620,NM_001278324	TROAP	trophinin associated protein	-1.022653122	-1.082351925	52.242661	44.879589	40.986136	6.59460842	6.56262875	6.48166407	3755	4906	4267
8574	NM_001320979,NM_003689	AKR7A2	aldo-keto reductase family 7 mem	-1.407782207	-1.082385476	7.938034	5.597079	6.825998	4.3919615	3.92621521	4.28338119	785	745	892
55969	NM_001199534,NM_018840,NM	C20orf24	chromosome 20 open reading fra	1.096969302	-1.082499209	88.90897	97.554844	77.376037	6.61836627	6.75059434	6.50520776	3818	5596	4338
80208	NM_001160227,NM_025137	SPG11	SPG11, spatacsin vesicle traffick	-1.499024989	-1.082499477	10.108408	6.686855	8.691598	6.26546264	5.69074111	6.15263823	2981	2657	3387
10763	NM_006617	NES	nestin	-3.051097974	-1.08251859	3.210992	1.052453	2.789205	4.20138033	2.74415527	4.09342686	683	299	776
5345	NM_000934,NM_001165920,NM	SERPINF2	serpin family F member 2	-1.414881127	-1.0826093	1.420541	1.009549	1.235858	2.0648444	1.70021226	1.97845985	125	118	142
144233	NM_181708	BCDIN3D	BCDIN3 domain containing RNA	-1.325333618	-1.082649389	2.009119	1.515628	1.745385	2.88122939	2.53704518	2.78271118	250	252	284
2157	NM_000132,NM_019863	F8	coagulation factor VIII	1.460059135	-1.082666122	0.296722	0.435386	0.258709	1.8573935	2.27254712	1.77525664	103	201	117
9564	NM_001170714,NM_001170715	BCAR1	BCAR1, Cas family scaffolding pr	1.780465306	-1.082830039	41.189959	73.383875	35.765695	7.04297863	7.87044244	6.92907512	5138	12223	5836
4711	NM_001199957,NM_001199958	NDUFB5	NADH:ubiquinone oxidoreductase	-1.022710139	-1.082870769	78.129305	76.118083	68.206841	6.32121441	6.28922568	6.2078435	3100	4050	3521
6259	NM_001005861,NM_002958	RYK	receptor-like tyrosine kinase	1.441675879	-1.082914566	40.356478	58.260719	35.024575	6.8627504	7.38670496	6.74885548	4530	8726	5145
64108	NM_022147	RTP4	receptor transporter protein 4	-1.485469241	-1.082988065	16.95022	11.426647	14.717297	4.14641466	3.61440811	4.03812306	656	590	745
80279	NM_001278197,NM_001278198	CDK5RAP3	CDK5 regulatory subunit associa	-1.502430215	-1.083085613	187.150771	127.230568	166.698903	9.01627562	8.43037299	8.90135914	20290	18044	23041
255967	NM_175854	PAN3	PAN3 poly(A) specific ribonuclea	-1.003664341	-1.083173812	9.630707	9.606107	8.356859	5.7502602	5.74508128	5.63721662	2074	2761	2355
7750	NM_001190964,NM_001190965	ZMYM2	zinc finger MYM-type containing	1.046057402	-1.083201473	13.798095	14.507217	11.981721	7.11127968	7.17578346	6.99684349	5389	7532	6119
84159	NM_001244638,NM_032199	ARID5B	AT-rich interaction domain 5B	1.901311202	-1.083244472	0.437804	0.83201	0.377854	2.13345087	2.89597101	2.04513029	133	338	151
29097	NM_001277197,NM_001277198	CNIH4	cornichon family AMPA receptor	-1.148604266	-1.083325283	90.214898	78.932785	77.90794	7.10675442	6.90842298	6.99215735	5372	6249	6099
38	NM_000019	ACAT1	acetyl-CoA acetyltransferase 1	-1.11174277	-1.083492642	20.224939	18.211897	17.536634	5.42718135	5.27808999	5.31428079	1650	1983	1873
9526	NM_001330073,NM_004870,NR	MPDU1	mannose-P-dolichol utilization de	1.229903872	-1.083695209	22.526595	27.984018	19.423671	5.16675115	5.45779006	5.05414007	1371	2253	1556
3104	NM_001278647,NM_001278648	ZBTB48	zinc finger and BTB domain conta	1.144333564	-1.08371614	9.695272	10.94	8.351789	4.49333262	4.67976064	4.38268287	845	1292	959
5438	NM_006233	POLR2I	RNA polymerase II subunit I	-1.487130027	-1.083742288	36.004578	24.252974	31.241648	4.9814326	4.43091866	4.86921928	1201	1079	1363
55860	NM_018477	ACTR10	actin related protein 10 homolog	-1.000293474	-1.08379979	23.845848	23.863503	20.673548	5.84982955	5.84941354	5.73582003	2225	2972	2525
100505746	NR_038311,NR_038312,NR_03	ITGB2-AS1	ITGB2 antisense RNA 1	-3.144171732	-1.083814882	0.565091	0.179489	0.485132	1.21691895	0.50527715	1.15176086	52	22	59
81671	NM_001329394,NM_001329395	VMP1	vacuole membrane protein 1	1.021851544	-1.083821803	154.990213	158.032787	135.084315	9.18436015	9.21549289	9.06843983	22802	31132	25876
80006	NM_001093755,NM_001093756	TRAPPC13	trafficking protein particle comple	-1.070376271	-1.083838959	18.036366	16.865081	14.533791	5.60908326	5.51303823	5.49540175	1877	2343	2130
306	NM_005139	ANXA3	annexin A3	-1.647142584	-1.084303672	288.55957	175.375885	250.076447	8.83156967	8.1136465	8.71506699	17847	14477	20244
81888	NM_001190880,NM_001243526	HYI	hydroxypyruvate isomerase (puta	-1.894818298	-1.084349632	19.380448	10.169665	16.685701	4.64936645	3.77771682	4.53736359	946	667	1073
10263	NM_001271849,NM_005851,NR	CDK2AP2	cyclin dependent kinase 2 associ	-1.236541997	-1.084404155	47.40541	38.165229	41.138533	5.90299631	5.60236135	5.7881215	2310	2496	2620
79797	NM_001184751,NM_024741	ZNF408	zinc finger protein 408	-1.064126497	-1.084405428	6.547302	6.235894	5.67683	4.08278037	3.99854533	3.97302418	626	786	710
9219	NM_001330292,NM_004739	MTA2	metastasis associated 1 family m	1.246459284	-1.084406977	32.555766	40.915247	28.452094	6.60332733	6.91823424	6.48766934	3778	6292	4285
4603	NM_001080416,NM_001144755	MYBL1	MYB proto-oncogene like 1	1.236777368	-1.084435363	12.395725	15.32995	10.699052	5.95723345	6.25937911	5.84224309	2400	3966	2722
101929224	NR_110787	LOC101929224	uncharacterized LOC101929224	-2.825824255	-1.084551217	0.531067	0.176045	0.450392	0.46682716	0.18071335	0.43515728	15	7	17
286967	NR_027402,NR_027402_dup1	FAM223B	family with sequence similarity 22	-1.33294858	-1.084551217	0.550696	0.413435	0.473436	0.46682716	0.36283659	0.43515728	15	15	17
29104	NM_013240,NM_182749,NR_04	N6AMT1	N-6 adenine-specific DNA methyl	-1.105719202	-1.084636713	8.467008	7.517648	7.194784	3.25675288	3.12759409	3.15222336	336	406	381
595	NM_053056	CCND1	cyclin D1	1.500126656	-1.084808246	72.764725	109.279732	63.035564	8.25205189	8.83556199	8.13501196	11930	23912	13526
100271836	NR_027155	SMG1P3	SMG1P3, nonsense mediated ml	1.274713754	-1.084886551	5.805498	7.412933	5.029654	4.88648104	5.22613324	4.77305948	1122	1911	1272
81605	NM_001135947,NM_001265582	URM1	ubiquitin related modifier 1	1.054215071	-1.085071033	17.18322	18.168013	15.351579	5.68818454	5.762918	5.57276666	1985	2796	2250
10164	NM_001166395,NM_005769	CHST4	carbohydrate sulfotransferase 4	1.261380246	-1.08513837	1.255089	1.585961	1.085578	1.87753473	2.12902957	1.79260867	105	177	119
54903	NM_001165927,NM_001321268	MKS1	Meckel syndrome, type 1	1.132828278	-1.085227286	11.815894	13.437421	10.20546	4.81386993	4.98778875	4.70022518	1065	1612	1207
54621	NM_019086	VSIG10	V-set and immunoglobulin domai	-1.501728323	-1.085364491	10.046999	6.698751	8.70139	5.64257074	5.07032524	5.52684729	1922	1710	2178
51398	NM_016145	WDR83OS	WD repeat domain 83 opposite s	-1.129880988	-1.085556727	77.891937	69.008812	67.417435	6.05110931	5.87775393	5.93452919	2564	3032	2905
23545	NM_012463	ATP6V0A2	ATPase H+ transporting V0 subu	-1.060202005	-1.085649813	3.60808	3.409598	3.125764	4.58498718	4.50425245	4.47155306	903	1138	1023
54497	NM_019024	HEATR5B	HEAT repeat containing 5B	-1.111787635	-1.085796088	6.515758	5.86696	5.63925	5.46511016	5.31586533	5.34914834	1695	2037	1920
11120	NM_001197233,NM_001197234	BTN2A1	butyrophilin subfamily 2 member	-1.260646476	-1.085923803	16.391615	13.086846	14.293571	5.65429253	5.32755573	5.5378219	1938	2054	2195
6874	NM_003185	TAF4	TATA-box binding protein associ	1.052084207	-1.08593006	9.29067	9.78588	8.039984	5.42204799	5.49363592	5.30599715	1644	2311	1862
131544	NM_153605	CRYBG3	crystallin beta-gamma domain co	1.226505351	-1.085966547	2.73354	3.357548	2.365125	4.88896361	5.17452161	4.77415178	1124	1842	1273
8831	NM_001130066,NM_006772	SYNGAP1	synaptic Ras GTPase activating	1.230605841	-1.085969748	2.607269	3.196054	2.255815	4.01620928	4.29881852	3.90484845	596	980	675
402	NM_001199745,NM_001667	ARL2	ADP ribosylation factor like GTPa	-1.412108612	-1.086047114	31.32377	22.305673	27.134483	4.94491448	4.46618624	4.82984105	1170	1107	1325
91056	NM_138368	AP5B1	adaptor related protein complex 5	-1.085179567	-1.086127192	6.500424	5.995033	5.625083	5.4177562	5.3026865	5.30145868	1639	2018	1856
58488	NM_001102402,NM_001330377	PCTP	phosphatidylcholine transfer prot	-1.25663189	-1.086168906	16.133386	12.306876	15.121266	5.15545404	4.83621478	5.03967945	1360	1446	1540
90624	NM_001293735,NM_181705,NR	LYRM7	LYR motif containing 7	-1.041194611	-1.086450093	4.377467	4.213847	3.786757	4.78750079	4.73140553	4.67237502	1045	1341	1183
378884	NM_198586	NHLRC1	NHL repeat containing E3 ubiquit	1.175904415	-1.086526025	1.114622	1.306326	0.960955	1.73025772	1.89765633	1.64757178	91	143	103
11124	NM_007051	FAF1	Fas associated factor 1	-1.179535491	-1.086646526	22.882034	19.419807	19.787186	5.87195953	5.63814463	5.75420361	2260	2560	2558
55063	NM_001258008,NM_017984	ZCWPW1	zinc finger CW-type and PWWP	-1.108864443	-1.086744714	2.256245	2.05827	1.955199	2.63728034	2.5131555	2.53718654	205	247	232
6882	NM_001270488,NM_005643	TAF11	TATA-box binding protein associ	-1.180682921	-1.086859505	17.21177	14.447063	15.114116	4.75112025	4.52111984	4.63558737	1018	1152	1152
11334	NM_007275	TUSC2	tumor suppressor candidate 2	1.009908633	-1.086940329	10.98031	11.104309	9.492558	4.2485404	4.26202234	4.13483255	707	954	800
126070	NM_152357	ZNF440	zinc finger protein 440	-1.131871977	-1.086980658	4.091434	3.617664	3.534626	4.14225859	3.97425277	4.02900114	654	772	740
9949	NM_001025580,NM_001171689	AMMECR1	Alport syndrome, mental retardat	-1.240876002	-1.08705536	5.715105	4.61022	5.16613	5.00908319	4.70844643	4.8925416	1225	1319	1386
147015	NM_144683	DHRS13	dehydrogenase/reductase 13	-1.223534479	-1.08707172	10.351463	8.472944	8.955627	4.34933082	4.07396851	4.23501577	761	831	861
112939	NM_052876	NACC1	nucleus accumbens associated 1	1.108010013	-1.087071926	28.528481	31.647535	24.663582	6.97741958	7.12427707	6.85796587	4908	7266	5553
29952	NM_013379	DPP7	dipeptidyl peptidase 7	-1.036640911	-1.087261151	61.765385	59.646927	53.377781	6.61836627	6.56698651	6.49894489	3818	4921	4319
79913	NM_024855	ACTR5	ARP5 actin related protein 5 hom	1.027122097	-1.087321634	9.003724	9.26176	7.781908	4.50794215	4.54487907	4.39267399	854	1172	966
643866	NM_001039771	CBLN3	cerebellin 3 precursor	1.314015226	-1.087458261	1.872504	2.459062	1.614975	2.4003046	2.7276415	2.30298238	168	295	190
9416	NM_004818	DDX23	DEAD-box helicase 23	-1.160208148	-1.087859238	46.473419	40.102604	40.142445	7.21756334	7.00472756	7.09692041	5804	6684	6562
122809	NM_080867,NM_199421	SOCS4	suppressor of cytokine signaling	1.180666462	-1.087923016	11.8752	13.973758	10.267155	6.33993461	6.57681563	6.21991873	3141	4955	3551
53	NM_001302489,NM_001302490	ACP2	acid phosphatase 2, lysosomal	1.05082426	-1.088243359	20.012946	20.992211	17.309467	5.41517498	5.48506467	5.29614568	1636	2297	1849
9589	NM_001270531,NM_001270532	WTAP	WT1 associated protein	1.211707684	-1.088362257	28.35017	34.955736	24.142558	5.8130465	6.08561003	5.69314689	2168	3510	2450
155061	NM_001163474,NM_152557	ZNF746	zinc finger protein 746	-1.188392975	-1.088367055	4.758617	4.009311	4.106861	4.2192465	3.98471399	4.10389022	692	778	782
158293	NM_001322224,NM_198841,NR	FAM120AOS	family with sequence similarity 12	1.223368205	-1.088393723	19.405699	24.570133	16.754409	5.32821046	5.61251543	5.20917167	1538	2514	1738
57679	NM_001135745,NM_020919	ALS2	ALS2, alsin Rho guanine nucleot	-1.031514949	-1.088516028	10.820189	10.013259	9.457989	5.92130285	5.87728612	5.80103986	2340	3031	2644
51460	NM_016329	SFMBT1	Scm-like with four mbt domains 1	1.084571594	-1.088526932	3.663197	3.979344	3.16242	4.11070049	4.22131714	3.99567625	639	926	722
4507	NM_002451	MTAP	methylthioadenosine phosphoryla	-1.06144449	-1.0885318	20.745489	19.568275	17.911442	6.6498128	6.56466401	6.52869704	3903	4913	4410
8533	NM_001199125,NM_001316354	COPS3	COP9 signalosome subunit 3	1.228022997	-1.088636146	29.61683	36.296263	25.386407	5.7597863	6.05118491	5.63961583	2088	3426	2359
200728	NM_198276	TMEM17	transmembrane protein 17	1.252421316	-1.088831479	2.614311	3.283656	2.260316	2.56460348	2.83945493	2.46326383	193	323	218
126328	NM_001193375,NM_175614,NR	NDUFA11	NADH:ubiquinone oxidoreductase	1.062135024	-1.089044545	72.855801	77.79089	59.161623	5.9085127	5.99407798	5.78758072	2319	3291	2619
113026	NM_133373	PLCD3	phospholipase C delta 3	-1.94382725	-1.089091145	59.789322	30.789782	51.585869	7.6645113	6.71228901	7.54201817	7926	5448	8951
64087	NM_022132	MCCC2	methylcrotonoyl-CoA carboxylase	1.007412941	-1.089138417	34.597206	34.896427	29.84993	6.96276465	6.97333495	6.84060498	4858	6539	5486

27249	NM_015702	MMADHC	methylmalonic aciduria and homo	1.236216516	-1.089171747	71.966621	89.079674	62.095947	6.66835688	6.97158289	6.54638607	3954	6531	4465
10019	NM_001291424,NM_005475	SH2B3	SH2B adaptor protein 3	1.018772314	-1.089534454	7.060183	7.199208	6.089032	5.25407798	5.28021477	5.13373733	1459	1986	1647
154007	NM_152551	SNRNP48	small nuclear ribonucleoprotein U	1.119835081	-1.08964125	3.357827	3.763473	2.893219	3.8730763	4.02581645	3.75799726	536	802	605
9972	NM_001278209,NM_001278210	NUP153	nucleoporin 153	-1.086627189	-1.089673026	13.923839	12.819758	12.02257	6.34942974	6.23110042	6.22711565	3162	3888	3569
8602	NM_001291978,NM_001291979	NOP14	NOP14 nucleolar protein	1.272649929	-1.089811331	17.061351	21.840562	14.713984	5.90299631	6.24567154	5.78107545	2310	3928	2607
9412	NM_001271811,NM_004264,NR	MED21	mediator complex subunit 21	-1.333479086	-1.089845691	9.937054	7.446624	8.536123	4.76334931	4.36571317	4.64397663	1027	1029	1159
100101467	NM_001112734,NM_001166012	ZSCAN30	zinc finger and SCAN domain co	1.439638245	-1.089872705	3.227396	4.654729	2.785215	3.87558202	4.37103944	3.76020394	537	1033	606
27031	NM_153240	NPHP3	nephrocystin 3	1.380454309	-1.089882929	5.203513	7.190714	4.487435	4.8255806	5.27667173	4.70596024	1074	1981	1212
11342	NM_007282,NM_183381	RNF13	ring finger protein 13	1.011650332	-1.089998819	26.864822	27.314085	23.272991	6.25539649	6.2718903	6.13276368	2960	4001	3340
90233	NM_001270938,NM_138347,NR	ZNF551	zinc finger protein 551	1.303422381	-1.090148186	3.559071	4.737267	3.073544	3.79844995	4.15647998	3.68321628	507	883	572
51497	NM_198976	NELFCD	negative elongation factor compl	1.069125962	-1.090247562	56.337456	60.293095	48.559063	6.99365829	7.08935998	6.87002091	4964	7091	5600
7862	NM_001003694,NM_001319049	BRPF1	bromodomain and PHD finger co	1.201684014	-1.090314426	4.359308	5.260975	3.720076	4.38494329	4.63839665	4.26640425	781	1254	881
51141	NM_001321329,NM_001321330	INSIG2	insulin induced gene 2	1.069217856	-1.090353281	39.090006	41.415191	33.448689	6.63362019	6.72923768	6.510133	3859	5513	4353
58986	NM_021259	TMEM8A	transmembrane protein 8A	1.150158429	-1.090385463	21.023264	24.206877	18.113266	6.24477511	6.44412863	6.12165047	2938	4515	3314
1021	NM_001145306,NM_001259	CDK6	cyclin dependent kinase 6	-1.50363185	-1.090410415	4.742576	3.14788	4.092011	5.77998999	5.20466589	5.65748382	2118	1882	2389
84792	NM_001037163	FAM220A	family with sequence similarity 22	-1.360190571	-1.090458419	6.650702	4.891332	5.725617	3.93924856	3.52883263	3.82277197	563	553	635
84883	NM_001198696,NM_032797	AIFM2	apoptosis inducing factor, mitoch	-1.933240793	-1.090469243	16.369629	8.475025	14.111288	5.72199495	4.7961904	5.59950948	2033	1405	2293
728233	NR_003563	PI4KAP1	phosphatidylinositol 4-kinase alpl	1.560148153	-1.090537424	23.587734	36.840389	20.326712	5.78666216	6.41895706	5.66398036	2128	4436	2400
79863	NM_001171967,NM_024805	RBFA	ribosome binding factor A (putativ	-1.094870797	-1.090582802	7.075709	6.465899	6.088031	5.32454708	5.19718866	5.20269596	1534	1872	1730
5424	NM_001256849,NM_001308632	POLD1	DNA polymerase delta 1, catalyti	-1.010639746	-1.090680658	23.660914	23.417502	20.40036	6.33630091	6.32122153	6.21268572	3133	4142	3533
93973	NM_022899	ACTR8	ARP8 actin related protein 8 hom	1.257047739	-1.090808098	4.633776	5.831946	3.993575	4.11918371	4.43219317	4.00128413	643	1080	725
143282	NM_152429	FGFBP3	fibroblast growth factor binding pi	-1.957820072	-1.09097047	1.112363	0.570721	0.963584	1.92669094	1.28081328	1.83509706	110	75	124
23543	NM_001031695,NM_001082576	RBFOX2	RNA binding protein, fox-1 homo	1.557179497	-1.091057157	21.467397	33.647898	18.457464	7.17471352	7.81008115	7.04989321	5633	11720	6350
9380	NM_012203	GRHPR	glyoxylate and hydroxypyruvate r	1.311358897	-1.091234341	46.648384	61.261047	40.174763	5.8357435	6.22081235	5.71207875	2203	3860	2483
54987	NM_001304759,NM_001304760	C1orf123	chromosome 1 open reading fran	-1.2125156	-1.091341697	29.806048	24.690528	26.29171	4.9767722	4.70844643	4.85483626	1197	1319	1349
114822	NM_052924	RHPN1	rhophilin Rho GTPase binding pr	-1.120542624	-1.091358109	34.770866	31.064129	29.937038	6.98614161	6.82331145	6.86105334	4938	5888	5565
514	NM_006886	ATP5E	ATP synthase, H+ transporting, r	-1.09139969	-1.091428899	485.543457	445.350067	418.059052	7.70417055	7.57862147	7.57858298	8148	9975	9182
439990	NR_038464	LINC00857	long intergenic non-protein codin	-1.891283446	-1.091624622	6.305779	3.329997	5.427376	3.83495865	3.00276575	3.71768886	521	368	587
79961	NM_001271833,NM_024901	DENND2D	DENN domain containing 2D	-1.027235029	-1.091638352	17.353916	16.980309	14.814672	5.80452441	5.76645903	5.6803862	2155	2803	2428
84126	NM_001271022,NM_001271023	ATRIP	ATR interacting protein	1.18607608	-1.091711897	3.985025	4.754014	3.42507	3.46742135	3.69306567	3.35270793	395	626	445
23195	NM_014611	MDN1	midasin AAA ATPase 1	-1.226335427	-1.091714439	4.028547	3.28801	3.467837	6.17833593	5.88847182	6.05356106	2804	3055	3159
2622	NM_001286205,NM_001286208	GAS8	growth arrest specific 8	1.156115455	-1.091846902	13.154244	15.359017	11.266208	5.40219915	5.6068831	5.27854964	1621	2504	1826
150197	NR_026919	LINC00896	long intergenic non-protein codin	2.044226257	-1.0918683	0.094404	0.194955	0.072487	0.26755841	0.50527715	0.24657076	8	22	9
257203	NR_026719	DSCR9	Down syndrome critical region 9	1.76726817	-1.0918683	0.111392	0.219574	0.106017	0.26755841	0.44594451	0.24657076	8	19	9
109616976	NR_145735	SNORA79B	small nucleolar RNA, H/ACA box	1.305671359	-1.0918683	1.328365	1.724386	1.10719	0.26755841	0.3412894	0.24657076	8	14	9
51729	NM_016312	WBP11	WW domain binding protein 11	-1.08489158	-1.091905466	45.11758	41.63596	38.828598	6.88841145	6.77189115	6.76267904	4612	5680	5195
54617	NM_017553,NR_104038	INO80	INO80 complex subunit	-2.591390893	-1.091974434	8.894436	3.437156	7.655399	5.80320883	4.46991427	5.67863733	2153	1110	2425
58517	NM_021239	RBM25	RNA binding motif protein 25	-1.118226594	-1.092284834	83.459267	74.717415	71.804306	8.45395938	8.29323182	8.32698881	13728	16403	15458
374899	NM_001037232,NM_001171979	ZNF829	zinc finger protein 829	-5.287162299	-1.092293015	0.699958	0.130746	0.600939	2.15010456	0.72085408	2.05235145	135	34	152
9674	NM_001162893,NM_001162894	KIAA0040	KIAA0040	-2.399730796	-1.092344394	4.3985	1.877322	3.887739	4.40762874	3.23665287	4.28644656	794	442	894
94137	NM_178857	RP1L1	RP1 like 1	1.632966799	-1.092566707	0.052441	0.084691	0.043987	0.49317359	0.73744528	0.45708734	16	35	18
102466194	NR_106831	MIR6773	microRNA 6773	-1.42118233	-1.092566707	5.449975	3.960188	4.62934	0.49317359	0.36283659	0.45708734	16	15	18
10396	NM_001105529,NM_006095	ATP8A1	ATPase phospholipid transportin	-1.333145184	-1.092566707	0.050626	0.037815	0.042408	0.49317359	0.38406669	0.45708734	16	16	18
158376	NM_001348107	SPAAR	small regulatory polypeptide of ai	-1.637447101	-1.092566707	0.269745	0.157981	0.224274	0.49317359	0.31941552	0.45708734	16	13	18
79809	NM_024753	TTC21B	tetratricopeptide repeat domain 2	1.163917961	-1.092692479	4.067368	4.74071	3.499681	4.50470835	4.71474432	4.38268287	852	1325	959
23184	NM_015154,NR_126327,NR_12	MESDC2	mesoderm development candida	1.132974623	-1.092727994	31.438046	35.737256	24.96475	6.90782119	7.08652976	6.78099736	4675	7077	5262
136288	NM_001100159,NM_001267865	C7orf57	chromosome 7 open reading fran	-1.391779045	-1.092801876	0.296328	0.21248	0.246774	0.68823103	0.52452456	0.64076093	24	23	27
79630	NM_001301039,NM_001301040	C1orf54	chromosome 1 open reading fran	1.060014533	-1.092801876	1.091962	1.12607	0.874205	0.68823103	0.72085408	0.64076093	24	34	27
1388	NM_001136153,NM_004381	ATF6B	activating transcription factor 6 b	-1.558018207	-1.092818591	24.158246	15.525878	20.780632	5.966078	5.33915216	5.84015848	2415	2071	2718
8841	NM_003883	HDAC3	histone deacetylase 3	1.453982566	-1.092864938	32.172199	46.826527	27.660194	5.9524941	6.48523156	5.82653464	2392	4647	2692
8927	NM_003458	BSN	bassoon presynaptic cytomatrix p	1.611765902	-1.092919908	0.052246	0.084238	0.045077	0.86002952	1.21124797	0.80366934	32	69	36
100506674	NR_109862,NR_109863,NR_10	BRCAT54	breast cancer-associated transcr	-1.334925126	-1.092990872	0.752268	0.555	0.661879	1.01352966	0.81765451	0.95003448	40	40	45
400657	NR_024484	LINC00909	long intergenic non-protein codin	-1.110594234	-1.093051018	4.443124	4.011004	3.825567	4.00480921	3.86335533	3.88476293	591	711	665
202018	NM_153365	TAPT1	transmembrane anterior posterio	1.079876807	-1.09306211	6.783619	7.333497	5.830564	4.9685801	5.07604389	4.84447416	1190	1717	1339
51429	NM_016224	SNX9	sorting nexin 9	-1.185131656	-1.093108429	13.684854	11.559272	11.763596	5.86251679	5.62204021	5.73638056	2245	2531	2526
619505	NR_002576	SNORA21	small nucleolar RNA, H/ACA box	-1.200686444	-1.093133126	15.568617	13.116873	13.384533	1.602974	1.43116693	1.51794971	80	89	90
8490	NM_001195303,NM_001254748	RGS5	regulator of G protein signaling 5	-1.133534929	-1.09317385	1.226677	0.864271	0.633693	1.94589385	1.81408935	1.85174813	112	132	126
22916	NM_001042540,NM_001308036	NCBP2	nuclear cap binding protein subu	1.007142399	-1.093198835	78.112788	78.753467	66.628005	7.37106531	7.38127136	7.24331916	6460	8693	7268
55122	NM_018064	AKIRIN2	akirin 2	1.107169897	-1.093453101	17.674601	19.596497	15.194275	5.09867502	5.24148154	4.97370184	1306	1932	1469
51082	NM_001206559,NM_015972,NM	POLR1D	RNA polymerase I subunit D	-1.103898972	-1.093526634	43.423193	40.897124	34.003368	5.62033234	5.48075987	5.49407653	1892	2290	2128
55766	NM_177925,NR_027716	H2AFJ	H2A histone family member J	-1.128140307	-1.093721383	46.654575	41.273659	40.008677	7.38432291	7.21147948	7.25588441	6520	7722	7332
23165	NM_001329434,NM_015135	NUP205	nucleoporin 205	-1.504563227	-1.093797558	30.045916	19.990006	25.817964	7.52643278	6.9410202	7.39781873	7199	6393	8095
2562	NM_000814,NM_001191320,NM	GABRB3	gamma-aminobutyric acid type A	-1.357407833	-1.094345211	2.116045	1.575658	1.902292	3.62166181	3.22198991	3.50257812	444	437	499
51374	NM_001170795,NM_016085,NM	ATRAID	all-trans retinoic acid induced diff	1.194706413	-1.094367122	133.963632	159.032166	114.012822	6.97274618	7.22753429	6.84372979	4892	7809	5498
8745	NM_003812	ADAM23	ADAM metallopeptidase domain	1.118304733	-1.094512088	0.372285	0.417114	0.318299	1.70793493	1.82188956	1.61867597	89	133	100
57461	NM_001199469,NM_020701	ISY1	ISY1 splicing factor homolog	-1.899929081	-1.0945157	10.792547	5.639122	9.188604	5.34819447	4.45368941	5.22123584	1560	1097	1753
5328	NM_001145031,NM_001319191	PLAU	plasminogen activator, urokinase	-1.296844378	-1.094828833	783.473185	605.456764	673.236145	10.82844517	10.45367465	10.69781485	71351	73512	80156
643387	NR_026923	LOC643387	TAR DNA binding protein pseudc	1.025234173	-1.094862635	1.431888	1.470711	1.22992	2.23841445	2.26684378	2.1362985	146	200	164
57168	NM_020437	ASPHD2	aspartate beta-hydroxylase doma	1.61276477	-1.094889206	1.722621	2.786708	1.483232	2.71768687	3.3216324	2.6075054	219	472	246
80154	NR_026811,NR_026811_dup1,N	GOLGA2P10	golgin A2 pseudogene 10	1.036289459	-1.094914109	7.148518	7.29834	6.113499	4.55559896	4.60488193	4.4305758	884	1224	993
221955	NM_001142936,NM_139179	DAGLB	diacylglycerol lipase beta	-1.428572559	-1.095097757	15.757884	10.897935	13.340237	5.49393602	4.99298	5.3659089	1730	1618	1943
57019	NM_001308347,NM_001308358	CIAPIN1	cytokine induced apoptosis inhibi	1.15258831	-1.095190502	22.178493	25.551724	19.096984	5.5786497	5.77953587	5.45033042	1837	2829	2063
55761	NM_001307943,NM_018259	TTC17	tetratricopeptide repeat domain 1	-1.243837884	-1.095371219	30.044671	24.844373	25.727767	6.87938828	6.56756655	6.74913326	4583	4923	5146
50717	NM_015726,NR_028103,NR_02	DCAF8	DDB1 and CUL4 associated facto	-1.053524768	-1.095695974	35.209182	34.341095	30.91738	7.12160784	7.04693651	6.99074853	5428	6884	6093
81620	NM_030928	CDT1	chromatin licensing and DNA rep	1.066259832	-1.09574738	23.559044	25.148624	20.202816	5.96254669	6.05367128	5.83283856	2409	3432	2704
10443	NM_001278432,NM_001320836	N4BP2L2	NEDD4 binding protein 2 like 2	-1.008648193	-1.095777869	24.523949	23.610228	20.608747	6.16046641	6.14821729	6.030436	2769	3668	3108
23119	NM_015094	HIC2	HIC ZBTB transcriptional repress	1.64653035	-1.095778181	10.468491	17.256983	8.977396	6.13507131	6.84643957	6.00507466	2720	5984	3053
207	NM_001014431,NM_001014432	AKT1	AKT serine/threonine kinase 1	-1.054630671	-1.095979102	70.067045	66.5187	60.683323	7.67227866	7.59592608	7.54073511	7969	10096	8943
5423	NM_002690	POLB	DNA polymerase beta	-1.158029872	-1.096164027	19.759108	17.08584	16.945827	4.7374097	4.53423581	4.61012283	1008	1163	1131
53371	NM_001278603,NM_017426,NM	NUP54	nucleoporin 54	1.103372222	-1.096313558	22.147594	24.220568	18.731897	5.67097747	5.81024893	5.54103391	1961	2891	2200
23563	NM_024533	CHST5	carbohydrate sulfotransferase 5	1.246591083	-1.096609278	0.258667	0.32664	0.222105	0.88013425	1.03456085	0.82068496	33	55	37
1748	NM_001934,NM_138281	DLX4	distal-less homeobox 4	-1.062351329	-1.096788961	0.974677	0.906208	0.786812	1.42282133	1.36863803	1.34055586	66	83	74
10201	NM_001308426,NM_001308427	NME6	NME/NM23 nucleoside diphosph	1.068909164	-1.096866448	3.401403	3.712143	2.923164	3.49701545	3.58491601	3.37591747	404	577	453
1039	NM_001802	CDR2	cerebellar degeneration related p	-1.004130059	-1.096945202	16.684521	16.640135	14.2985	5.48903517	5.48322133	5.35864561	1724	2294	1933
79725	NM_001317776,NM_024672	THAP9	THAP domain containing 9	-1.302105502	-1.096996492	4.357345	3.348968	3.734406	4.13809052	3.78172178	4.01243492	652	669	731
8773	NM_003825,NM_130798	SNAP23	synaptosome associated protein	-1.375231254	-1.097078475	17.88288	12.961374	15.260264	5.54362658	5.09548035	5.41295079	1792	1741	2009
7596	NM_003425	ZNF45	zinc finger protein 45	-1.125542298	-1.097116032	4.869864	4.334054	4.169109	4.28669603	4.12530075	4.16012035	727	863	815
5433	NM_004805	POLR2D	RNA polymerase II subunit D	1.249311173	-1.097134314	17.377964	21.734863	14.881587	5.33459909	5.64859917	5.20431761	1545	2579	1732
90701	NM_001307941,NM_033280	SEC11C	SEC11 homolog C, signal peptid	-1.049940732	-1.097226165	35.574395	33.789659	30.281759	4.86898241	4.80113128	4.73989992	1108	1410	1242
23609	NM_001271707,NM_014160	MKRN2	makorin ring finger protein 2	1.090488873	-1.097500624	10.049968	11.022745	8.604572	4.85126892	4.97210198	4.72189817	1094	1594	1226
25940	NM_001304538,NM_015475	FAM98A	family with sequence similarity 98	-1.066933125	-1.097525073	15.3853	14.365014	12.99994	5.41258913	5.32137849	5.28162528	1633	2045	1830
54809	NM_001193307,NM_017654	SAMD9	sterile alpha motif domain contair	-2.288265617	-1.097563155	11.203951	4.885889	9.615616	6.23943495	5.06950644	6.10698744	2927	1709	3280
4670	NM_001297418,NM_005968,NM	HNRNPM	heterogeneous nuclear ribonucle	1.279785079	-1.09769025	102.51015	130.820871	87.734422	7.95386115	8.30849354	7.81995683	9695	16578	10863
996	NM_001114091,NM_001256,NM	CDC27	cell division cycle 27	-1.002528574	-1.097738389	28.304529	28.189964	24.211689	7.33760551	7.33398465	7.20394025	6311	8411	7071
29941	NM_001317926,NM_013355	PKN3	protein kinase N3	-1.552225652	-1.097742566	15.563914	10.023174	13.313006	5.71151407	5.09225907	5.57965517	2018	1737	2261
55260	NM_001303538,NM_001303539	TMEM143	transmembrane protein 143	-1.007887856	-1.09778824	5.875967	5.970825	5.091659	3.94403156	3.93343361	3.81854297	565	749	633
1801	NM_001346574,NM_001346575	DPH1	diphthamide biosynthesis 1	1.019878741	-1.097838357	13.180486	13.708711	11.342682	4.91721027	4.94467948	4.78719527	1147	1563	1285
440993	NR_122105	LINC00969	long intergenic non-protein codin	1.219774591	-1.097990024	8.559637	10.457006	7.326009	3.14487866	3.40188358	3.02586351	308	502	345
663	NM_001320674,NM_001320675	BNIP2	BCL2 interacting protein 2	-1.076090307	-1.098210475	14.065643	13.32524	12.047569	6.45707676	6.35252315	6.3235294	3410	4234	3819

348995	NM_198887,NR_104456	NUP43	nucleoporin 43	1.047720675	-1.098238409	12.439642	13.06108	10.713384	5.52459402	5.59042388	5.39246953	1768	2475	1980
23067	NM_015048	SETD1B	SET domain containing 1B	-1.141577846	-1.098250398	6.256048	5.48671	5.35316	5.67025604	5.48322133	5.5378219	1960	2294	2195
4828	NM_021077,NM_205858	NMB	neuromedin B	1.551916913	-1.098309385	11.023649	17.119964	9.419	3.59149522	4.18246262	3.46789548	434	900	486
196394	NM_001113402,NM_001278411	AMN1	antagonist of mitotic exit network	-2.668721842	-1.098318659	11.099248	4.045563	9.388639	4.56959435	3.2511683	4.44024179	893	447	1000
9806	NM_001134434,NM_001244950	SPOCK2	SPARC/osteonectin, cwcv and ka	-1.932527113	-1.098412657	1.328302	0.677325	1.06296	2.92543304	2.14154997	2.80852903	259	179	290
54537	NM_001330112,NM_019054	FAM35A	family with sequence similarity 35	-1.776067931	-1.098419608	10.689477	5.900626	9.018827	5.24053381	4.44108339	5.10884471	1445	1087	1618
65258	NM_001242904,NM_001319154	MPPE1	metallophosphoesterase 1	1.283367794	-1.098445943	10.148711	13.133316	8.769201	4.95442736	5.30407942	4.82352399	1178	2020	1319
55102	NM_018036	ATG2B	autophagy related 2B	1.110099304	-1.098595538	3.128706	3.47694	2.677094	5.3670153	5.51424225	5.2347877	1581	2345	1770
3212	NM_002145	HOXB2	homeobox B2	-2.160016415	-1.09869458	54.398617	25.212851	46.528469	6.42407778	5.33234202	6.28993987	3332	2061	3730
29980	NM_017613	DONSON	downstream neighbor of SON	-1.666922597	-1.098695089	28.483612	17.105701	24.36162	6.14860191	5.42487721	6.01481077	2746	2201	3074
54883	NM_017748,NR_073428	CWC25	CWC25 spliceosome associated	-1.136947842	-1.098737417	21.149676	18.708732	18.34728	6.04109929	5.85892233	5.90740809	2546	2992	2850
644128	NR_003572	RPL23AP53	ribosomal protein L23a pseudoge	1.309572053	-1.098756315	0.792971	1.040196	0.67887	2.45494311	2.78063431	2.34475282	176	308	197
5982	NM_001278791,NM_001278792	RFC2	replication factor C subunit 2	-1.380179592	-1.098832819	32.278716	23.238966	27.381321	5.78732769	5.33234202	5.65392788	2129	2061	2383
80010	NM_024945	RMI1	RecQ mediated genome instabilit	1.102535824	-1.098868632	11.419466	12.608349	9.770925	5.31719229	5.45465779	5.1847364	1526	2248	1708
8438	NM_001142548,NM_003579	RAD54L	RAD54 like (S. cerevisiae)	1.057087983	-1.098883049	19.171705	20.292915	16.420803	5.90053779	5.97933194	5.76687928	2306	3257	2581
84080	NM_032140,NR_138150	ENKD1	enkurin domain containing 1	-1.270002451	-1.098924736	18.088256	14.314399	15.324155	4.80338004	4.47239429	4.67237502	1057	1112	1183
54943	NM_001040192,NM_001320746	DNAJC28	DnaJ heat shock protein family (H	-1.95598518	-1.098994302	1.952823	1.020801	1.883666	2.07360076	1.40024122	1.97085819	126	86	141
8846	NM_006020	ALKBH1	alkB homolog 1, histone H2A dio	1.174511034	-1.099033059	6.199005	7.292057	5.299453	4.05430925	4.27344453	3.92662428	613	962	686
25909	NM_001323342,NM_001323343	AHCTF1	AT-hook containing transcription	-1.01198572	-1.099135022	36.522418	36.171889	31.28654	8.31963028	8.30249532	8.18370797	12504	16509	13992
2582	NM_000403,NM_001008216,NM	GALE	UDP-galactose-4-epimerase	1.077128252	-1.099285451	100.274375	108.027928	85.877742	7.28296775	7.38949612	7.14731852	6075	8743	6797
55311	NM_001253792,NM_018337	ZNF444	zinc finger protein 444	1.18029109	-1.099290778	12.571237	14.871313	10.754522	4.71659644	4.94735617	4.58544575	993	1566	1111
100134934	NM_001113324	TEN1	TEN1, CST complex subunit	-1.119952206	-1.099350977	24.736822	22.110445	21.138783	4.64496692	4.48841131	4.51401614	943	1125	1055
83636	NM_001031726,NM_001256046	C19orf12	chromosome 19 open reading fra	1.013584081	-1.099437147	3.37135	3.200335	2.768735	3.96300655	3.98123534	3.83538509	573	776	641
171024	NM_001128933,NM_001128934	SYNPO2	synaptopodin 2	-1.103592251	-1.099545364	0.271563	0.247747	0.234251	1.55375576	1.4614436	1.46482043	76	92	85
109617000	NR_145776	SNORA11F	small nucleolar RNA, H/ACA box	-2.806963906	-1.099744541	3.434072	1.152323	2.888156	0.5190475	0.20477631	0.47868902	17	8	19
5977	NM_001330308,NM_006268	DPF2	double PHD fingers 2	1.16823358	-1.099771505	20.298294	23.711609	17.476633	5.7065968	5.92695261	5.57213879	2011	3139	2249
55172	NM_001083908,NM_018139	DNAAF2	dynein axonemal assembly facto	1.083208455	-1.099832746	8.440021	9.092263	7.206142	4.62574568	4.73657294	4.49426709	930	1346	1040
64682	NM_022662	ANAPC1	anaphase promoting complex sul	1.058965662	-1.099845436	10.748149	11.395953	9.182986	6.35707077	6.43874893	6.22152115	3179	4498	3555
6430	NM_001039465,NM_001320214	SRSF5	serine and arginine rich splicing f	1.097209286	-1.099894599	264.634139	290.56759	225.214133	8.67989151	8.8134194	8.54287659	16062	23547	17961
9056	NM_001126105,NM_001126106	SLC7A7	solute carrier family 7 member 7	-2.089628467	-1.099980719	1.584917	0.761506	1.265632	2.22275497	1.4614436	2.11576522	144	92	161
6474	NM_001163678,NM_003030,NM	SHOX2	short stature homeobox 2	1.027335949	-1.100032159	1.32653	1.371418	1.127308	2.35073722	2.38212036	2.24113096	161	221	180
6206	NM_001016	RPS12	ribosomal protein S12	-1.15787196	-1.100108317	687.482788	594.421143	587.25238	8.39960938	8.1888061	8.26239018	13219	15254	14779
23051	NM_015035	ZHX3	zinc fingers and homeoboxes 3	1.026694876	-1.100155639	2.040421	2.097767	1.742844	4.38318339	4.41939707	4.25236388	780	1070	872
27303	NM_001003792,NM_001003793	RBMS3	RNA binding motif single strande	1.144361532	-1.100225962	0.160325	0.184359	0.137449	1.2010231	1.31437719	1.12461302	51	78	57
904	NM_001240,NM_001277842	CCNT1	cyclin T1	-1.229108861	-1.100380205	12.726003	10.47236	10.861262	6.46250395	6.16862428	6.3261384	3423	3721	3826
100533464	NR_037918	PRH1-PRR4	PRH1-PRR4 readthrough	1.330319091	-1.10038747	2.431226	3.231429	2.073882	2.29192162	2.62883416	2.18310266	153	272	171
338440	NM_001012302,NM_001347882	ANO9	anoctamin 9	1.048792846	-1.100461605	16.323601	17.108619	13.942752	5.56474231	5.6320572	5.42968364	1819	2549	2033
55239	NM_001324357,NM_001324358	OGFOD1	2-oxoglutarate and iron depende	1.076464284	-1.100785769	17.043621	18.377325	14.503308	5.67673599	5.78103713	5.54103391	1969	2832	2200
441250	NM_001145440	TYW1B	tRNA-yW synthesizing protein 1 l	1.069164957	-1.100847308	2.254042	2.420012	1.9258	2.99161027	3.07634485	2.87112187	273	390	305
55177	NM_001304802,NM_001323894	RMDN3	regulator of microtubule dynamic	-1.018501884	-1.100912992	17.301831	16.939334	14.79392	5.32637993	5.30059458	5.19129304	1536	2015	1716
1677	NM_001282669,NM_001320132	DFFB	DNA fragmentation factor subuni	1.005872438	-1.101040698	2.310012	2.413048	2.022376	3.03260384	3.04002433	2.9113909	282	379	315
79415	NM_001033046,NM_001100407	C17orf62	chromosome 17 open reading fra	1.237945728	-1.101063264	31.120179	40.366954	28.645735	6.02199984	6.325686	5.88533804	2512	4155	2806
84000	NM_001077263,NM_001206789	TMPRSS13	transmembrane protease, serine	-1.910508914	-1.101079382	3.951585	2.053732	3.369007	3.34257292	2.53229878	3.21791812	359	251	401
5928	NM_001135255,NM_001135256	RBBP4	RB binding protein 4, chromatin r	1.117987523	-1.101221168	29.865352	33.427993	25.489056	7.85796531	8.01821476	7.71948858	9069	13547	10129
255043	NM_173804	TMEM86B	transmembrane protein 86B	-1.420095929	-1.101283083	9.333539	6.573627	7.965649	4.08061013	3.60990992	3.95001076	625	588	698
79760	NM_001007269,NM_001007270	GEMIN7	gem nuclear organelle associate	-1.191462333	-1.10132511	15.75414	13.623712	13.559379	4.52559963	4.28477699	4.39267399	865	970	966
219293	NM_001039211	ATAD3C	ATPase family, AAA domain cont	-1.903063161	-1.101443041	3.133216	1.650136	2.675983	3.67729659	2.84711955	3.54926401	463	325	517
101927957	NR_126330	LINC01572	long intergenic non-protein codin	-1.092122466	-1.101472549	1.547719	1.419599	1.319935	1.8573935	1.76638114	1.75769337	103	126	115
7289	NM_001160408,NM_003324	TULP3	tubby like protein 3	-1.371915581	-1.101531323	13.220905	9.882932	11.625769	5.3571874	4.91399145	5.22123584	1570	1529	1753
79858	NM_001146003,NM_001321220	NEK11	NIMA related kinase 11	2.636862598	-1.101616851	2.845893	6.913276	2.351805	3.07682646	4.36571317	2.95442604	292	1029	326
2475	NM_004958	MTOR	mechanistic target of rapamycin	1.144729958	-1.101656525	11.380254	13.041802	9.707106	6.61011426	6.80325827	6.47193601	3796	5806	4238
83658	NM_001281727,NM_001281728	DYNLRB1	dynein light chain roadblock-type	-1.154856072	-1.101788274	162.659917	140.550708	138.937459	6.83018009	6.62442377	6.69161939	4428	5123	4943
284306	NM_173631	ZNF547	zinc finger protein 547	1.045241202	-1.101834769	5.346856	5.58962	4.558148	3.99100913	4.05092325	3.86028525	585	817	653
84318	NM_001130146,NM_001130147	CCDC77	coiled-coil domain containing 77	-1.308807127	-1.101853577	10.111957	7.814165	8.647908	4.59870169	4.22867066	4.46480383	912	931	1018
2825	NM_001098199,NM_001261452	GPR1	G protein-coupled receptor 1	-1.492484777	-1.102167594	0.959806	0.645356	0.821913	1.77389308	1.38978355	1.67590018	95	85	106
388284	NM_001012984	C16orf86	chromosome 16 open reading fra	-1.731727759	-1.102214597	0.534921	0.296303	0.453821	0.73313634	0.46599441	0.6785857	26	20	29
3313	NM_004134	HSPA9	heat shock protein family A (Hsp7	1.101082373	-1.102480806	179.821747	198.214478	153.270645	9.2640396	9.40274716	9.12352583	24099	35454	26885
829	NM_006135	CAPZA1	capping actin protein of muscle Z	-1.144501383	-1.102542451	136.173203	119.115448	116.065102	8.50896106	8.31481244	8.36853173	14263	16651	15911
51703	NM_016234,NM_203379,NM_20	ACSL5	acyl-CoA synthetase long-chain f	1.005752795	-1.102591077	49.517276	49.944212	42.296566	7.34959644	7.35782173	7.20960321	6364	8552	7099
84910	NM_001329914,NM_032824	TMEM87B	transmembrane protein 87B	1.066195418	-1.102718175	18.093626	19.312793	15.419728	6.5368116	6.6283211	6.39733809	3606	5137	4022
55324	NM_001351298,NM_001351299	ABCF3	ATP binding cassette subfamily F	1.39699826	-1.102836727	24.586389	34.199419	20.95806	5.9696007	6.44539154	5.83074031	2421	4519	2700
54496	NM_001184824,NM_001290018	PRMT7	protein arginine methyltransferas	1.280077059	-1.102975643	10.065707	13.044615	8.605287	4.80732269	5.15226631	4.67120285	1060	1813	1182
4436	NM_000251,NM_001258281	MSH2	mutS homolog 2	1.428648307	-1.103011809	26.564058	37.999466	22.630238	6.39249502	6.9019982	6.25280965	3259	6221	3634
84188	NM_032228	FAR1	fatty acyl-CoA reductase 1	-1.326661177	-1.103090013	47.311314	35.701435	40.303684	7.93070405	7.52482903	7.78976121	9540	9608	10637
54534	NM_019051	MRPL50	mitochondrial ribosomal protein L	-1.018161219	-1.103337659	37.734581	37.103573	32.135788	5.26749617	5.24220835	5.12947602	1473	1933	1642
5336	NM_002661	PLCG2	phospholipase C gamma 2	-2.008183598	-1.103607808	2.098538	1.04546	1.78744	4.23299104	3.30224686	4.09866803	699	465	779
57117	NM_001142471,NM_020395	INTS12	integrator complex subunit 12	-2.366761212	-1.103616625	14.778265	5.975622	11.698627	4.58038655	3.4174117	4.44436461	900	508	1003
55308	NM_001320522,NM_001320525	DDX19A	DEAD-box helicase 19A	1.144049484	-1.103665906	17.000933	19.532539	14.527808	5.66881209	5.85939613	5.52943707	1958	2993	2182
1487	NM_001012614,NM_001328	CTBP1	C-terminal binding protein 1	-1.062918535	-1.103675667	94.425607	88.840119	80.213155	7.83497433	7.74733971	7.69331111	8925	11219	9946
2030	NM_001078175,NM_001078177	SLC29A1	solute carrier family 29 member 1	1.044233843	-1.103878496	9.146582	9.611786	7.763838	4.41281357	4.47239429	4.27723085	797	1112	888
84311	NM_001278279,NM_032351	MRPL45	mitochondrial ribosomal protein L	1.106978652	-1.103919639	34.972784	38.668515	29.746123	5.87759565	6.0218562	5.73750098	2269	3356	2528
58525	NM_001330395,NM_021241	WIZ	widely interspaced zinc finger mo	2.540626065	-1.103952761	8.071123	20.540609	6.873035	5.08899074	6.4083109	4.95069932	1297	4403	1445
25911	NM_001329742,NM_001329743	DPCD	deleted in primary ciliary dyskine	-1.034408956	-1.104008172	13.650999	12.891829	11.750517	3.65704831	3.61216077	3.5261099	456	589	508
23412	NM_012071	COMMD3	COMM domain containing 3	1.059435333	-1.104068099	26.791275	28.410326	22.792055	4.67692288	4.75705948	4.53993458	965	1366	1075
1606	NM_001345,NM_001351033,NM	DGKA	diacylglycerol kinase alpha	-1.957059593	-1.104101786	35.269847	17.572755	29.810493	6.55763653	5.60349311	6.41635229	3659	2498	4076
1965	NM_004094	EIF2S1	eukaryotic translation initiation fa	1.210726328	-1.104231738	35.093079	42.534927	29.863943	7.16296608	7.43709041	7.02096905	5587	9038	6223
4232	NM_001253900,NM_001253901	MEST	mesoderm specific transcript	-1.204902681	-1.104381585	26.834611	22.340872	23.417398	6.01916965	5.75479142	5.87824435	2507	2780	2792
115004	NM_138441	MB21D1	Mab-21 domain containing 1	1.109004225	-1.104548779	6.778283	7.518	5.760236	3.6887407	3.82699692	3.5569002	467	692	520
84255	NM_001287498,NM_032295,NM	SLC37A3	solute carrier family 37 member 3	-1.807890805	-1.104646363	19.503282	10.632987	16.604949	5.9922909	5.15612801	5.8510692	2460	1818	2739
4940	NM_006187	OAS3	2'-5'-oligoadenylate synthetase 3	-1.343622051	-1.104937632	20.445065	15.232086	17.389734	7.05712075	6.63470109	6.91428896	5189	5160	5776
57213	NM_001127482,NM_020456,NR	SPRYD7	SPRY domain containing 7	-1.391938893	-1.104965627	3.238093	2.336291	2.77348	3.47735365	3.05002095	3.34684674	398	382	443
646719	NR_046262	NIPBL-AS1	NIPBL antisense RNA 1 (head to	1.506351318	-1.105117235	1.519269	2.289755	1.291085	3.15316384	3.68880651	3.02586351	310	624	345
51426	NM_001345921,NM_001345922	POLK	DNA polymerase kappa	-1.135952082	-1.105306146	4.37485	3.838943	3.712855	5.5221972	5.34254521	5.38104381	1765	2076	1964
10746	NM_006609	MAP3K2	mitogen-activated protein kinase	1.243926378	-1.105406178	6.429908	8.006764	5.466217	6.10975337	6.42056328	5.96737086	2672	4441	2973
5194	NM_002618	PEX13	peroxisomal biogenesis factor 13	1.04714602	-1.1054196	11.828493	12.401368	10.054906	5.72824713	5.7934873	5.58651095	2042	2857	2272
8615	NM_001290049,NM_003715	USO1	USO1 vesicle transport factor	-1.500708924	-1.105437934	46.035546	30.708701	39.136337	7.51442403	6.93271458	7.37063535	7139	6356	7943
66035	NM_001024938,NM_001024939	SLC2A11	solute carrier family 2 member 11	-1.216578783	-1.105463127	8.544754	6.545219	7.085745	4.58957318	4.31962786	4.45120991	906	995	1008
642517	NM_001190810,NM_001190810	AGAP9	ArfGAP with GTPase domain, an	-1.815121828	-1.105465188	11.582721	6.387534	9.840813	4.80338004	3.98471399	4.66414974	1057	778	1176
91807	NM_001308301,NM_182493	MYLK3	myosin light chain kinase 3	1.326665894	-1.105487209	0.027755	0.037506	0.024719	0.29776659	0.38406669	0.27153692	9	16	10
64493	NR_024121	LINC00235	long intergenic non-protein codin	1.573212469	-1.105487209	0.396614	0.646918	0.327017	0.29776659	0.44594451	0.27153692	9	19	10
692199	NR_003065	SNORD84	small nucleolar RNA, C/D box 84	1.901941236	-1.105487209	2.806077	5.573449	2.33171	0.29776659	0.52452456	0.27153692	9	23	10
101929464	NR_125978	LOC101929464	uncharacterized LOC101929464	1.080119318	-1.105487209	0.431927	0.504608	0.394704	0.29776659	0.31941552	0.27153692	9	13	10
54776	NM_001271618,NM_017607	PPP1R12C	protein phosphatase 1 regulatory	1.027292797	-1.105498151	33.899663	34.860068	28.81611	6.62582736	6.66428769	6.48266668	3838	5268	4270
10535	NM_006397	RNASEH2A	ribonuclease H2 subunit A	1.150075509	-1.105573345	40.915581	47.121136	34.782856	5.53968212	5.73736895	5.39814864	1787	2746	1988
1841	NM_001165031,NM_001320902	DTYMK	deoxythymidylate kinase	1.090064167	-1.105738685	42.619265	46.2774	35.629411	5.6564798	5.77853415	5.51448193	1941	2827	2159
79035	NM_024068	NABP2	nucleic acid binding protein 2	-1.066895743	-1.10589457	17.974705	16.868839	15.277071	4.67404687	4.5843929	4.53478801	963	1206	1071
65084	NM_001168724,NM_022918,NR	TMEM135	transmembrane protein 135	-1.424298561	-1.106106483	3.865076	2.715596	3.282964	5.13468443	4.64170569	4.99353253	1340	1257	1490
79573	NM_001122835,NM_024525	TTC13	tetratricopeptide repeat domain 1	-1.001173184	-1.106121875	13.136018	13.200893	11.116534	5.41431354	5.41266157	5.27237862	1635	2182	1818
84896	NM_001321967,NM_001321968	ATAD1	ATPase family, AAA domain cont	-3.254484932	-1.106168427	21.114371	6.525097	17.949633	6.53165735	4.86386887	6.38773618	3593	1475	3995

5715	NM_001261400,NM_002813,NR	PSMD9	proteasome 26S subunit, non-AT	-1.133709231	-1.106184362	19.68417	17.62931	16.818452	5.52618969	5.34930747	5.38390874	1770	2086	1968
389610	NM_001289973,NM_207411	XKR5	XK related 5	2.444613181	-1.106209506	0.093586	0.227715	0.079374	0.54446555	1.08728891	0.49997203	18	59	20
9915	NM_014862	ARNT2	aryl hydrocarbon receptor nuclea	-1.333473374	-1.106209506	0.067817	0.05244	0.060868	0.54446555	0.42561204	0.49997203	18	18	20
8672	NM_001198801,NM_001198802	EIF4G3	eukaryotic translation initiation fa	-1.027681005	-1.106303604	19.51619	18.717844	16.190811	6.86211879	6.82306857	6.71768538	4528	5887	5034
7554	NM_021089	ZNF8	zinc finger protein 8	1.102386942	-1.106339496	6.779525	7.484007	5.757966	3.96300655	4.09504299	3.8269886	573	844	637
375133	NR_003700	PI4KAP2	phosphatidylinositol 4-kinase alpl	1.602072257	-1.106529635	31.276785	50.177036	26.563688	6.13350189	6.80571875	5.98964105	2717	5816	3020
145282	NM_001195296,NM_001195297	MIPOL1	mirror-image polydactyly 1	-1.092576707	-1.106578466	1.429753	1.302601	1.218441	3.27584855	3.16180073	3.1454863	341	417	379
100287227	NR_027954	TIPARP-AS1	TIPARP antisense RNA 1	-1.55733367	-1.106731518	0.640681	0.411507	0.543826	1.38110603	1.02107213	1.29257653	63	54	70
256714	NM_001168465,NM_001168466	MAP7D2	MAP7 domain containing 2	1.471360608	-1.106794505	0.564123	0.842926	0.474547	1.7191395	2.12902957	1.61867597	90	177	100
159371	NM_001134658,NM_001345993	SLC35G1	solute carrier family 35 member (	1.849454569	-1.10681902	0.855487	1.686713	0.728258	1.90722898	2.60642858	1.80120704	108	267	120
6236	NM_001128850,NM_004165	RRAD	RRAD, Ras related glycolysis inh	5.224604364	-1.106843546	2.385522	12.574479	2.014614	2.15010456	4.2461677	2.0378728	135	943	150
7322	NM_003339,NM_181838	UBE2D2	ubiquitin conjugating enzyme E2	1.08706731	-1.106867643	44.223892	48.820583	37.802361	6.90291642	7.02239442	6.7577178	4659	6767	5177
1455	NM_001319	CSNK1G2	casein kinase 1 gamma 2	1.142501958	-1.106910594	35.820431	40.972794	30.413824	6.67664254	6.86708389	6.53160649	3977	6071	4419
79979	NM_001167970,NM_001167971	TRMT2B	tRNA methyltransferase 2 homol	-1.050370248	-1.1072039	5.832792	5.582785	5.246497	4.40762874	4.3401413	4.26795589	794	1010	882
23108	NM_001100398,NM_001330058	RAP1GAP2	RAP1 GTPase activating protein	-1.067993029	-1.107267062	6.554993	6.126895	5.567658	5.43654533	5.3439002	5.29310088	1661	2078	1845
81930	NM_031217	KIF18A	kinesin family member 18A	-1.243963327	-1.107325105	19.369968	15.593703	16.438744	6.05166339	5.74200129	5.90691023	2565	2755	2849
55337	NM_001308277,NM_018381	C19orf66	chromosome 19 open reading fra	-1.075744747	-1.107447046	21.277306	19.692074	18.038366	5.49148768	5.38857221	5.34768165	1727	2145	1918
57128	NM_001164840,NM_001164841	LYRM4	LYR motif containing 4	1.049262825	-1.107723499	7.722838	8.095614	6.33264	3.509976	3.57341041	3.37591747	408	572	453
1351	NM_004074	COX8A	cytochrome c oxidase subunit 8A	-1.031474107	-1.107794671	307.440094	298.4216	260.813934	7.25533391	7.21092267	7.10865803	5959	7719	6616
92610	NM_052864	TIFA	TRAF interacting protein with fork	1.220679054	-1.108089728	5.650282	6.902656	4.79232	4.16292023	4.43600998	4.02350017	664	1083	737
147645	NM_001163922	VSIG10L	V-set and immunoglobulin domai	-1.464555461	-1.108260531	2.917731	1.993443	2.473494	3.51641288	3.0232078	3.38166197	410	374	455
8690	NM_001261833,NM_003772	JRKL	JRK-like	-1.103172157	-1.108347833	8.139843	7.531167	6.88362	4.85126892	4.71474432	4.7082479	1094	1325	1214
63893	NM_022066	UBE2O	ubiquitin conjugating enzyme E2	1.095017229	-1.108352165	16.075623	17.621908	13.629275	6.4064247	6.53590573	6.25984475	3291	4815	3652
6018	NM_012421	RLF	rearranged L-myc fusion	1.048533852	-1.10840585	9.46632	9.937098	8.02427	5.87321391	5.94045074	5.72738578	2262	3169	2510
5863	NM_001243738,NM_004761	RGL2	ral guanine nucleotide dissociatic	1.084676804	-1.108438039	14.778963	15.818716	12.455425	5.58325587	5.69817645	5.43797784	1843	2671	2045
7035	NM_001032281,NM_001318941	TFPI	tissue factor pathway inhibitor	1.104974631	-1.108518751	43.915342	47.870347	36.04901	6.97858557	7.12151459	6.83118973	4912	7252	5450
23175	NM_001261427,NM_001261428	LPIN1	lipin 1	-1.727524078	-1.108572673	8.743792	5.061412	7.41538	5.57093993	4.80408772	5.42551859	1827	1413	2027
55255	NM_018268	WDR41	WD repeat domain 41	-1.419693173	-1.108597171	12.061938	8.509203	10.229654	5.54991538	5.0571683	5.40451103	1800	1694	1997
23518	NM_001282798,NM_001282799	R3HDM1	R3H domain containing 1	1.183677017	-1.108611044	8.731194	10.27619	7.40964	5.2482889	5.48567859	5.10364045	1453	2298	1612
128	NM_000671	ADH5	alcohol dehydrogenase 5 (class I	1.076422249	-1.109057619	56.310776	60.688805	47.718323	7.19341313	7.29895917	7.04515031	5707	8208	6329
90668	NM_138360	CARMIL3	capping protein regulator and my	-2.661913032	-1.109058538	0.259218	0.097287	0.217367	1.11880553	0.52452456	1.03996283	46	23	51
79777	NM_001135705,NM_001135706	ACBD4	acyl-CoA binding domain contain	-1.491350966	-1.109125544	19.614884	13.559766	17.761818	5.60606863	5.04389026	5.45986539	1873	1678	2077
79723	NM_001193424,NM_001193425	SUV39H2	suppressor of variegation 3-9 hor	1.203216047	-1.109174662	3.834872	4.662732	3.298952	3.61267788	3.859571	3.47597349	441	709	489
79574	NM_001319952,NM_024526,NM	EPS8L3	EPS8 like 3	-39.15584699	-1.10920577	1.035159	0.025336	0.864683	1.74129091	0.08023518	1.63800401	92	3	102
7284	NM_003321	TUFM	Tu translation elongation factor, r	1.249973176	-1.109430982	153.628281	192.24118	130.131165	8.27974807	8.60071908	8.13043461	12162	20312	13483
9183	NM_004724	ZW10	zw10 kinetochore protein	-1.052252973	-1.109511487	11.443295	10.885868	9.690163	5.08141332	5.01015048	4.93613424	1290	1638	1430
51308	NM_001271803,NM_016606,NR	REEP2	receptor accessory protein 2	-1.123157218	-1.109573094	0.424598	0.384776	0.35703	0.95786335	0.8787583	0.88680889	37	44	41
445	NM_000050,NM_054012	ASS1	argininosuccinate synthase 1	-2.766953084	-1.109627522	42.12288	15.287529	35.701751	6.21984785	4.78526051	6.07188671	2887	1394	3200
5147	NM_001291018,NM_002601,NR	PDE6D	phosphodiesterase 6D	1.137543274	-1.109632162	31.615719	34.071422	25.262179	5.15132397	5.33234202	5.00567345	1356	2061	1503
80270	NM_001142777,NM_001142778	HSD3B7	hydroxy-delta-5-steroid dehydrog	-1.106052856	-1.109873814	7.231722	6.544497	6.080541	4.02751998	3.8914258	3.88678411	601	726	666
10513	NM_001282476,NM_006380	APPBP2	amyloid beta precursor protein bi	-1.938765433	-1.110209577	9.553258	4.932549	8.087691	5.94177327	5.0084426	5.79351821	2374	1636	2630
130399	NM_001111031,NM_001111032	ACVR1C	activin A receptor type 1C	-1.205870632	-1.110418152	0.082177	0.068088	0.068383	0.77668602	0.669904	0.71544405	28	31	31
358	NM_001329872,NM_198098	AQP1	aquaporin 1 (Colton blood group)	-2.069913576	-1.110418152	0.249334	0.120881	0.205782	0.77668602	0.42561204	0.71544405	28	18	31
139285	NM_152424	AMER1	APC membrane recruitment prote	1.372406324	-1.1105144	0.949361	1.305521	0.804131	3.14071816	3.55246622	3.00740444	307	563	340
91869	NM_052859	RFT1	RFT1 homolog	1.364319587	-1.110599971	8.547313	11.674317	7.231761	5.44078168	5.88009069	5.29310088	1666	3037	1845
10523	NM_006387	CHERP	calcium homeostasis endoplasmi	1.037663076	-1.110613847	28.185198	29.280085	23.846901	6.82500364	6.87788106	6.67504916	4412	6117	4886
342897	NM_001001414	NCCRP1	non-specific cytotoxic cell recept	-1.557149749	-1.110663179	0.742146	0.467641	0.61969	1.27880673	0.93737891	1.19154816	56	48	62
2787	NM_005274	GNG5	G protein subunit gamma 5	-1.151853352	-1.110770568	137.35762	119.371567	116.189316	6.76545159	6.56350135	6.61535471	4232	4909	4686
10780	NM_001144824,NM_006630	ZNF234	zinc finger protein 234	1.073525441	-1.11081755	3.153436	3.336182	2.624758	3.88556162	3.98123534	3.74468587	541	776	599
23327	NM_001144964,NM_001144965	NEDD4L	neural precursor cell expressed,	1.338814553	-1.110854873	21.48214	28.721507	18.082905	7.45262374	7.87149979	7.30186348	6838	12232	7571
163702	NM_170743,NM_173064,NM_17	IFNLR1	interferon lambda receptor 1	-1.484397166	-1.110876417	2.41221	1.622569	2.037569	3.54817338	3.03667667	3.41004634	420	378	465
64744	NM_001198978,NM_001198979	SMAP2	small ArfGAP2	1.403752434	-1.110891028	6.99998	9.483699	6.031183	4.34028863	4.80900168	4.1964253	756	1418	837
5371	NM_002675,NM_033238,NM_03	PML	promyelocytic leukemia	-1.20678194	-1.111144498	27.303337	23.827804	25.078823	6.59308669	6.32500006	6.44269547	3751	4153	4152
25979	NM_001330159,NM_015510	DHRS7B	dehydrogenase/reductase 7B	-1.007468765	-1.111275571	11.971315	11.796341	9.745757	4.49006587	4.47980886	4.34495514	843	1118	933
79637	NM_001304271,NM_001304272	ARMC7	armadillo repeat containing 7	-1.463901467	-1.111322026	20.245876	13.764783	16.8056	5.23858851	4.706341	5.09054707	1443	1317	1597
7514	NM_003400	XPO1	exportin 1	1.013910244	-1.111467739	93.753189	95.166145	79.269905	8.78759205	8.80747733	8.6354888	17310	23450	19155
23451	NM_001005526,NM_001308824	SF3B1	splicing factor 3b subunit 1	1.149947508	-1.111493465	140.061921	160.488347	117.52593	9.13749916	9.33873402	8.98528445	22072	33913	24424
6288	NM_000331,NM_001178006,NM	SAA1	serum amyloid A1	-2.934926123	-1.11162704	34.909466	12.080374	29.987511	4.38318339	2.95745524	4.23818553	780	355	863
11260	NM_007235	XPOT	exportin for tRNA	-1.463793548	-1.111648728	58.055244	39.704292	49.073929	7.80800808	7.26127053	7.65602362	8759	7995	9691
79912	NM_001350912,NM_001350913	PYROXD1	pyridine nucleotide-disulphide ox	-1.111444471	-1.111651136	4.177928	3.798977	3.534075	4.15055879	4.00712306	4.0068703	658	791	728
10598	NM_001321441,NM_012111	AHSA1	activator of HSP90 ATPase activ	1.123475532	-1.111681057	152.467941	171.53054	128.965562	7.70628355	7.8734949	7.5543097	8160	12249	9028
83930	NM_032016	STARD3NL	STARD3 N-terminal like	-1.144978699	-1.11196164	19.224327	16.810448	16.248522	5.07270439	4.88356843	4.92437537	1282	1496	1418
100270804	NR_026885	LOC100270804	uncharacterized LOC100270804	1.724355438	-1.1120047	1.954291	3.372795	1.646221	2.27683528	2.93245472	2.15654364	151	348	167
57091	NM_001164114,NM_001164115	CASS4	Cas scaffolding protein family me	1.650768677	-1.112062777	0.155967	0.257726	0.132557	0.56944351	0.84852988	0.52094563	19	42	21
23369	NM_001282752,NM_001282790	PUM2	pumilio RNA binding family meml	1.021893684	-1.112285565	26.804638	26.703435	22.071293	7.34078263	7.37183757	7.18825152	6325	8636	6994
9669	NM_015904	EIF5B	eukaryotic translation initiation fa	1.100465537	-1.112323165	34.285286	37.769024	28.965174	7.30218753	7.43946901	7.149635	6157	9053	6808
8458	NM_003594	TTF2	transcription termination factor 2	-1.114350249	-1.112344759	10.85768	9.75385	9.17196	5.70940872	5.55634684	5.55889016	2015	2416	2228
7424	NM_005429	VEGFC	vascular endothelial growth facto	2.18629858	-1.112409096	10.667351	23.351994	9.009684	4.50470835	5.59839316	4.35812668	852	2489	942
9039	NM_003968,NM_198195	UBA3	ubiquitin like modifier activating e	-1.062270149	-1.112652811	28.10853	26.560495	23.761028	5.88258712	5.79695418	5.7313279	2277	2864	2517
10938	NM_001282444,NM_001282445	EHD1	EH domain containing 1	1.170720153	-1.11310193	37.364681	43.795013	31.547359	7.01000137	7.23576892	6.85667747	5021	7854	5548
801	NM_006888	CALM1	calmodulin 1	-1.105768223	-1.113230388	72.996338	66.087677	61.622589	8.24552836	8.10098066	8.09131278	11876	14350	13121
90411	NM_001171506,NM_001171507	MCFD2	multiple coagulation factor deficie	1.130472916	-1.113259022	45.126988	51.094129	38.057891	7.53120843	7.70723688	7.37729967	7223	10910	7980
7644	NM_001300951,NM_003430	ZNF91	zinc finger protein 91	-1.138708449	-1.113276567	2.508189	2.196015	2.108006	3.84522223	3.67164303	3.7017041	525	616	580
92609	NM_001001563,NM_001329559	TIMM50	translocase of inner mitochondria	-1.188713178	-1.113349849	17.368619	14.630693	14.659883	6.54588578	6.29938812	6.39272292	3629	4079	4009
79717	NM_001077447,NM_001287506	PPCS	phosphopantothenoylcysteine sy	-1.115892892	-1.113446117	27.187217	23.50352	22.379128	5.8267075	5.67144382	5.67454836	2189	2621	2418
57645	NM_001314014,NM_017542	POGK	pogo transposable element deriv	-1.05737183	-1.113496201	12.233527	11.582497	10.32534	6.13297837	6.05367128	5.98020636	2716	3432	3000
28966	NM_014035,NR_146145	SNX24	sorting nexin 24	-1.179234169	-1.113537484	9.872413	8.495829	8.498357	4.48842972	4.26202234	4.34053776	842	954	930
27235	NM_015697	COQ2	coenzyme Q2, polyprenyltransfer	1.075189589	-1.113590512	30.658182	33.007458	25.881714	5.64404118	5.74661881	5.49208641	1924	2764	2125
266812	NM_153757	NAP1L5	nucleosome assembly protein 1 l	-1.105316636	-1.113597596	1.289872	1.164536	1.085531	1.97422731	1.86782539	1.86000215	115	139	127
57212	NR_033708,NR_033709,NR_03	TP73-AS1	TP73 antisense RNA 1	-1.831540938	-1.113635592	2.960488	1.774973	2.437691	4.19537543	3.38618649	4.04899377	680	496	751
116113	NM_001012426,NM_001012427	FOXP4	forkhead box P4	1.219207799	-1.113676178	3.12461	3.812959	2.634697	4.2524017	4.52470876	4.10562676	709	1155	783
990	NM_001254	CDC6	cell division cycle 6	-1.190119919	-1.113738657	31.293242	26.32373	26.403572	6.55490324	6.3067027	6.40123173	3652	4100	4033
201292	NM_001256124,NM_173547	TRIM65	tripartite motif containing 65	-1.159669142	-1.113761327	22.158066	19.137253	18.687538	6.22869497	6.01804174	6.07543546	2905	3347	3208
105378828	NR_135837,NR_135838	LOC105378828	uncharacterized LOC105378828	1.340762917	-1.114137038	0.761204	0.962486	0.593807	0.79797761	0.99371033	0.73352601	29	52	32
260293	NM_001320289,NM_001320290	CYP4X1	cytochrome P450 family 4 subfar	3.912082751	-1.114137038	0.319959	1.262643	0.266335	0.79797761	1.96260273	0.73352601	29	152	32
105379252	NR_136310,NR_136310_dup1,N	LOC105379252	family with sequence similarity 95	-1.487608754	-1.114137038	0.478983	0.319356	0.401661	0.79797761	0.58077243	0.73352601	29	26	32
10525	NM_001130991,NM_006389	HYOU1	hypoxia up-regulated 1	-1.572135086	-1.114182275	25.624399	16.293906	21.58497	6.84559893	6.20001348	6.69104119	4476	3804	4941
80209	NM_025138	PROSER1	proline and serine rich 1	1.082315519	-1.114210168	7.745495	8.394636	6.532293	5.32179342	5.43317608	5.16987408	1531	2214	1690
59284	NM_031896	CACNG7	calcium voltage-gated channel au	-1.972837527	-1.114329409	1.822777	0.920863	1.537909	2.37218925	1.63971536	2.2474368	164	111	181
23023	NM_001017395,NM_001128224	TMCC1	transmembrane and coiled-coil d	1.234433995	-1.114348268	6.271413	7.481518	4.991186	5.08358236	5.3793514	4.93222525	1292	2131	1426
23683	NM_005813	PRKD3	protein kinase D3	1.38884369	-1.114482387	3.590503	4.995239	3.028509	4.43676607	4.89192945	4.2879768	811	1505	895
10907	NM_001303471,NM_001305557	TXNL4A	thioredoxin like 4A	-1.391832591	-1.114596047	26.906443	19.345142	22.531666	5.29115116	4.82849184	5.13883433	1498	1438	1653
8550	NM_003668,NM_139078	MAPKAPK5	mitogen-activated protein kinase-	-1.079728782	-1.114696402	15.246613	14.152519	12.858012	5.12944497	5.02204937	4.97750019	1335	1652	1473
3623	NM_002191	INHA	inhibin alpha subunit	1.086377466	-1.114761087	2.823726	3.079846	2.383141	2.30685184	2.40307061	2.18310266	155	225	171
54875	NM_001114395,NM_001286984	CNTLN	centlein	-1.046101084	-1.1150834	2.874083	2.54446	2.398708	3.79315817	3.7329126	3.64789978	505	645	557
5757	NM_001099285,NM_002823	PTMA	prothymosin, alpha	-1.030592607	-1.115124537	1133.945923	1101.494171	955.611481	10.37801806	10.33457699	10.22093767	52206	67683	57581
203274	NR_146625,NR_146626	LINC00537	long intergenic non-protein codin	1.901183153	-1.115246344	0.891402	1.697326	0.751204	2.23841445	3.01302298	2.11576522	146	371	161
6416	NM_001281435,NM_003010	MAP2K4	mitogen-activated protein kinase	1.195319867	-1.115294633	6.623543	7.924665	5.58418	4.68836995	4.9366195	4.53736359	973	1554	1073
7692	NM_001083330,NM_001282995	ZNF133	zinc finger protein 133	1.534201468	-1.115337984	9.844838	15.562519	8.840299	4.80863452	5.40813487	4.65706199	1061	2175	1170

54881	NM_001161584,NM_017746	TEX10	testis expressed 10	1.422495345	-1.115356326	9.871844	14.060728	8.318211	4.95442736	5.44900251	4.8022651	1178	2239	1299
125476	NM_001098817,NM_001308064	INO80C	INO80 complex subunit C	-2.48247979	-1.115412916	15.34609	6.292894	13.545289	4.03426408	2.84711955	3.88678411	604	325	666
1147	NM_001278,NM_001320928	CHUK	conserved helix-loop-helix ubiqui	-4.112733488	-1.11541811	8.172038	1.987439	6.893271	4.89638579	2.99933041	4.74436548	1130	367	1246
7884	NM_001306074,NM_001306075	SLBP	stem-loop binding protein	-1.011104525	-1.115627062	85.943091	85.109829	72.979697	7.55035055	7.53450363	7.39338282	7320	9673	8070
84100	NM_001278293,NM_001323513	ARL6	ADP ribosylation factor like GTPa	1.205995137	-1.115635443	2.386411	2.774364	1.699332	3.24129226	3.48529134	3.10091148	332	535	366
400027	NR_028408	LINC00938	long intergenic non-protein codin	-1.025887926	-1.115665615	7.422019	7.23993	6.246276	4.08061013	4.04593672	3.93250653	625	814	689
11060	NM_001270453,NM_001270454	WWP2	WW domain containing E3 ubiqu	1.123286011	-1.115736964	11.44403	12.560373	9.894902	5.50934646	5.67360076	5.35500021	1749	2625	1928
641298	NR_027154	SMG1P1	SMG1P1, nonsense mediated m	1.19487425	-1.115759195	5.405407	6.47023	4.55329	4.77547559	5.02374123	4.62351744	1036	1654	1142
4552	NM_002454,NM_024010,NR_13	MTRR	5-methyltetrahydrofolate-homocy	1.055345683	-1.115891872	5.953009	6.312088	4.977311	4.29981562	4.37369523	4.15005835	734	1035	809
90075	NM_001099437,NM_001099438	ZNF30	zinc finger protein 30	-1.704530355	-1.116278375	3.857739	2.328002	3.271518	3.46076162	2.78063431	3.31717816	393	308	433
5908	NM_001010942,NM_001251917	RAP1B	RAP1B, member of RAS oncoge	-1.066619934	-1.116293681	40.066177	37.600223	33.770818	6.4020861	6.31017284	6.24534631	3281	4110	3615
83740	NM_080720,NM_080720_dup1,N	H2AFB3	H2A histone family member B3	1.639709934	-1.116660608	0.486439	0.811404	0.39418	0.3273552	0.50527715	0.29607837	10	22	11
401247	NR_130726	LINC00243	long intergenic non-protein codin	-1.658385536	-1.116660608	0.12472	0.066867	0.10686	0.3273552	0.20477631	0.29607837	10	8	11
84173	NM_001135021,NM_001135022	ELMOD3	ELMO domain containing 3	1.022857151	-1.116749843	7.829204	8.029758	6.698576	4.32936266	4.36036716	4.17838702	750	1025	826
221443	NM_001329684,NM_001329685	OARD1	O-acyl-ADP-ribose deacylase 1	-1.537139725	-1.116835755	17.239701	11.22389	14.083652	4.54776468	3.96018541	4.39551594	879	764	968
55223	NM_001330483,NM_018207	TRIM62	tripartite motif containing 62	1.273984306	-1.1169068	2.359879	3.003972	1.978582	3.29469475	3.61216077	3.15222336	346	589	381
6449	NM_003021	SGTA	small glutamine rich tetratricopep	1.192455903	-1.116964718	46.701336	55.749493	39.2934	6.73161893	6.98336816	6.57361777	4133	6585	4551
10947	NM_001134296,NM_006803	AP3M2	adaptor related protein complex 3	1.075517705	-1.117458961	13.451505	14.487542	11.313321	5.59470734	5.69764662	5.43797784	1858	2670	2045
6885	NM_003188,NM_145331,NM_14	MAP3K7	mitogen-activated protein kinase	1.097149243	-1.117476352	9.758445	10.74203	8.151547	5.63223546	5.76342439	5.47539445	1908	2797	2100
64172	NM_022353	OSGEPL1	O-sialoglycoprotein endopeptidas	1.321651863	-1.117798802	4.050526	5.365482	3.408112	3.34257292	3.70997765	3.19852188	359	634	395
79657	NM_001146075,NM_001146076	RPAP3	RNA polymerase II associated pr	-1.167618447	-1.117858238	14.372889	12.289372	12.017697	5.68103985	5.46216384	5.52360351	1975	2260	2173
7297	NM_003331	TYK2	tyrosine kinase 2	1.044532032	-1.117870114	27.579706	28.840652	23.18638	6.84655716	6.9088808	6.68727722	4479	6251	4928
266675	NM_153274	BEST4	bestrophin 4	1.25940199	-1.117876721	0.747285	0.942732	0.627146	1.33814853	1.5486383	1.24294651	60	101	66
9812	NM_001142603,NM_014773	KIAA0141	KIAA0141	1.185727877	-1.11803812	20.235693	24.745029	17.494513	6.59916399	6.84261054	6.43994504	3767	5968	4144
23753	NM_022044	SDF2L1	stromal cell derived factor 2 like 1	-1.263438774	-1.11848412	15.613962	12.363316	13.10783	3.8375314	3.52644784	3.68786049	522	552	574
144348	NM_001204298,NM_152437	ZNF664	zinc finger protein 664	-1.122904097	-1.118596732	32.339817	28.842809	27.240178	7.09685598	6.93091246	6.93641176	5335	6348	5866
7073	NM_001033925,NM_001323964	TIAL1	TIA1 cytotoxic granule associate	-1.148820651	-1.118801912	29.784669	25.889241	24.949913	6.93720463	6.73879719	6.77664393	4772	5550	5246
8851	NM_003885	CDK5R1	cyclin dependent kinase 5 regula	-1.063996678	-1.118842567	1.773261	1.669431	1.489155	2.93987166	2.86232771	2.79997431	262	329	288
57605	NM_001300801,NM_020845	PITPNM2	phosphatidylinositol transfer prot	-1.902674175	-1.118849808	4.794364	2.513577	4.024717	5.02497132	4.13629029	4.86819666	1239	870	1362
152195	NR_002949,NR_027766	NUDT16P1	nudix hydrolase 16 pseudogene	1.658878164	-1.118880818	4.321745	7.181764	3.606438	3.42699388	4.10306728	3.28075123	383	849	421
100874291	NR_046762	ALMS1-IT1	ALMS1 intronic transcript 1	-1.96103148	-1.118935638	3.445786	1.747807	2.886367	2.78853894	2.0043309	2.65096518	232	158	255
581	NM_001291428,NM_001291429	BAX	BCL2 associated X, apoptosis re	-1.123087766	-1.119109302	68.429699	59.844108	57.156546	5.80189206	5.63759229	5.64260924	2151	2559	2364
25832	NM_015383	NBPF14	NBPF member 14	1.057832412	-1.119156475	2.513416	2.664359	2.110655	4.68408793	4.76213597	4.52832887	970	1371	1066
528	NM_001695	ATP6V1C1	ATPase H+ transporting V1 subu	1.106839424	-1.119186104	16.172901	17.921545	13.582213	6.50520177	6.65011801	6.34463812	3527	5216	3876
727677	NR_024393,NR_117100	CASC8	cancer susceptibility 8 (non-prote	-1.369037476	-1.119247497	19.484518	14.242208	16.354834	4.93653894	4.50061221	4.7796009	1163	1135	1278
51290	NM_016570	ERGIC2	ERGIC and golgi 2	1.079586569	-1.119339398	37.988564	41.057339	31.895884	6.06049988	6.16938873	5.90042236	2581	3723	2836
64599	NM_022574	GIGYF1	GRB10 interacting GYF protein 1	-1.334963669	-1.119383232	23.239248	17.427027	19.509659	7.21706946	6.80350451	7.05551926	5802	5807	6375
84131	NM_001098802,NM_001330691	CEP78	centrosomal protein 78	1.051850759	-1.119405733	7.305365	7.569552	7.429866	5.56007657	5.63150252	5.40097983	1813	2548	1992
11098	NM_001293178,NM_001293179	PRSS23	protease, serine 23	-3.856657232	-1.119428698	21.550189	5.697356	18.054466	6.39380665	4.49452466	6.23308578	3262	1130	3584
8394	NM_001135636,NM_001135637	PIP5K1A	phosphatidylinositol-4-phosphate	-1.018782024	-1.11963575	33.072647	32.372907	27.604563	6.91545167	6.88883011	6.75384724	4700	6164	5163
7097	NM_001318787,NM_001318789	TLR2	toll like receptor 2	1.049097805	-1.11980677	0.806173	0.802127	0.639436	1.94589385	1.99745948	1.8266989	112	157	123
285352	NR_033373	KIF9-AS1	KIF9 antisense RNA 1	1.144361532	-1.119824596	0.521247	0.597591	0.440351	1.2010231	1.31437719	1.11084509	51	78	56
83746	NM_031488	L3MBTL2	L3MBTL2 polycomb repressive c	1.151986097	-1.120104854	8.358752	9.640874	7.012559	4.75384679	4.95091738	4.5966027	1020	1570	1120
84826	NM_032740	SFT2D3	SFT2 domain containing 3	1.280841435	-1.120128564	5.126177	6.573709	4.300866	3.91021271	4.2461677	3.75799726	551	943	605
54938	NM_001145901,NM_017827	SARS2	seryl-tRNA synthetase 2, mitocho	-1.274695035	-1.120144514	22.936994	18.010654	19.2354	5.55461405	5.21284632	5.39460182	1806	1893	1983
128308	NM_001321284,NM_181441,NM	MRPL55	mitochondrial ribosomal protein L	-1.087758909	-1.120301229	49.003595	45.944324	41.258077	5.14406778	5.0262753	4.98506702	1349	1657	1481
55166	NM_018132	CENPQ	centromere protein Q	-1.066152037	-1.120383975	9.434237	8.859634	7.917862	4.07843661	3.99164624	3.92465818	624	782	685
84140	NM_001201543,NM_032180,NR	FAM161A	family with sequence similarity 16	1.0869098	-1.120403574	3.043622	3.317434	2.574525	3.60967076	3.72044785	3.45977198	440	639	483
10402	NM_001271142,NM_001271145	ST3GAL6	ST3 beta-galactoside alpha-2,3-s	2.799716818	-1.12042321	0.58041	1.778139	0.51374	1.62696745	2.77662633	1.51794971	82	307	90
100874058	NR_049718	COX10-AS1	COX10 antisense RNA 1	1.259424987	-1.120533842	2.750503	3.47847	2.313274	2.63728034	2.92160616	2.5006986	205	345	225
23774	NM_001304808,NM_001304809	BRD1	bromodomain containing 1	1.232529886	-1.120721814	13.038541	15.860851	11.27269	5.8769705	6.17396696	5.71549438	2268	3735	2489
257068	NM_001185106,NM_153268	PLCXD2	phosphatidylinositol specific phos	-1.145000914	-1.120742935	0.871846	0.762106	0.745443	1.50279892	1.37924953	1.39836968	72	84	79
85363	NM_033034,NM_033092,NM_03	TRIM5	tripartite motif containing 5	-1.518662463	-1.12083572	13.081026	8.290155	10.5458	5.35897928	4.77424718	5.19863383	1572	1383	1725
9667	NM_014649	SAFB2	scaffold attachment factor B2	1.161319719	-1.120842865	35.401428	41.153419	29.675877	6.85006522	7.06409699	6.68698728	4490	6967	4927
81846	NM_030962	SBF2	SET binding factor 2	-1.600924721	-1.121192724	10.83915	6.777973	9.086859	6.31338057	5.64530591	6.15053688	3083	2573	3382
50865	NM_015987	HEBP1	heme binding protein 1	-1.060017995	-1.121271947	27.934765	26.375622	23.399643	5.08141332	4.99987273	4.92142062	1290	1626	1415
84220	NM_005054,NM_005054_dup1,N	RGPD5	RANBP2-like and GRIP domain c	-1.372238761	-1.121298098	14.281685	10.417994	11.971354	6.65310266	6.20188276	6.48966556	3912	3809	4291
340061	NM_001301738,NM_198282	TMEM173	transmembrane protein 173	-1.46060618	-1.121380665	19.560962	14.269218	16.93516	5.34819447	4.81780485	5.18719863	1560	1427	1711
4688	NM_000433,NM_001127651,NM	NCF2	neutrophil cytosolic factor 2	-1.312524821	-1.121710421	3.120037	2.361663	2.634891	3.02359429	2.68551066	2.8792661	280	285	307
4848	NM_001199302,NM_001199303	CNOT2	CCR4-NOT transcription complex	-1.111402894	-1.122158015	28.328412	25.521211	23.730658	6.51889098	6.36825587	6.35453021	3561	4281	3903
11275	NM_001161521,NM_001161522	KLHL2	kelch like family member 2	1.005577572	-1.122288565	8.979187	9.064354	7.526335	4.8255806	4.83332352	4.66532766	1074	1443	1177
7700	NM_001348277,NM_001348278	ZNF141	zinc finger protein 141	-1.423797623	-1.122322247	4.506054	3.68126	3.728879	5.70800345	5.20987698	5.5448789	2013	1889	2206
5717	NM_001270482,NM_002815	PSMD11	proteasome 26S subunit, non-AT	-1.087197715	-1.122327074	58.267699	53.610693	48.788371	7.79978594	7.67973452	7.63408229	8709	10703	9544
57038	NM_001318785,NM_001350505	RARS2	arginyl-tRNA synthetase 2, mitoc	1.110015647	-1.122436595	9.962448	11.113199	8.541879	4.60927912	4.75400498	4.44984345	919	1363	1007
84062	NM_001271667,NM_001271668	DTNBP1	dystrobrevin binding protein 1	-1.27122315	-1.12262434	5.633233	4.467558	4.611839	3.16959281	2.86610484	3.02219055	314	330	344
8813	NM_001317034,NM_001317035	DPM1	dolichyl-phosphate mannosyltran	-1.032659994	-1.122853356	81.784245	79.49231	68.284439	6.48236777	6.43652791	6.31717356	3471	4491	3802
283643	NM_001134875,NM_001134876	C14orf80	chromosome 14 open reading fra	-1.033115986	-1.123005701	24.331511	23.599181	20.297559	5.35269794	5.30686125	5.18965667	1565	2024	1714
8924	NM_004667	HERC2	HECT and RLD domain containir	-1.150583707	-1.123115148	8.713409	7.582431	7.290719	7.03515498	6.83443995	6.86899886	5110	5934	5596
100132832	NR_028058	PMS2P9	PMS1 homolog 2, mismatch repa	-2.237496775	-1.123309119	2.835673	1.263951	2.367645	2.89611494	1.95552917	2.75199454	253	151	277
116064	NM_001099678	LRRC58	leucine rich repeat containing 58	1.43158806	-1.123372517	8.005009	11.471396	6.69547	5.92795747	6.43843185	5.76303325	2351	4497	2574
4701	NM_005001,NR_135539	NDUFA7	NADH:ubiquinone oxidoreductas	-1.563053681	-1.12347945	85.398151	55.514127	75.69004	5.65720815	5.02880493	5.49275009	1942	1660	2126
83479	NM_001031725,NM_001320181	DDX59	DEAD-box helicase 59	-1.405266198	-1.123524771	12.102995	8.92761	10.518408	4.80206342	4.33197089	4.64038721	1056	1004	1156
1497	NM_001031681,NM_004937	CTNS	cystinosin, lysosomal cystine trar	1.104908817	-1.123551732	1.88975	2.110659	1.570357	3.18179291	3.31058687	3.03318152	317	468	347
8737	NM_001317061,NM_003804	RIPK1	receptor interacting serine/threon	-1.013935058	-1.123593989	5.117887	5.053144	4.566577	4.51116872	4.49208242	4.35082401	856	1128	937
57226	NM_020466,NR_028493,NR_02	LYRM2	LYR motif containing 2	-1.020475416	-1.123621205	3.206914	3.159623	2.684505	4.16701728	4.13941485	4.00872756	666	872	729
57670	NM_001164665,NM_020910	KIAA1549	KIAA1549	-1.477819721	-1.123766813	1.403103	0.950932	1.172857	4.16906145	3.64330938	4.01058243	667	603	730
126003	NM_001042461,NM_001042462	TRAPPC5	trafficking protein particle comple	-1.13218237	-1.123780782	66.396482	58.467958	56.255897	5.56629422	5.39119595	5.40168676	1821	2149	1993
26097	NM_001206612,NM_001244664	CHTOP	chromatin target of PRMT1	-1.079710307	-1.123854104	44.186265	40.886995	36.848614	6.54785089	6.43843185	6.38129921	3634	4497	3977
284739	NM_001348090	C20orf204	chromosome 20 open reading fra	-1.1869219	-1.124023652	0.311622	0.259689	0.259065	0.86002952	0.75384785	0.78645063	32	36	35
26353	NM_014365	HSPB8	heat shock protein family B (sma	-1.937358509	-1.124023652	0.41027	0.214724	0.339461	0.86002952	0.50527715	0.78645063	32	22	35
51567	NM_016614	TDP2	tyrosyl-DNA phosphodiesterase 2	-1.129772989	-1.124239435	15.170379	13.447462	12.680326	4.88025587	4.71054878	4.71736241	1117	1321	1222
10733	NM_001190799,NM_001190801	PLK4	polo like kinase 4	-1.080074227	-1.124431942	20.249666	18.773925	17.044659	6.27213461	6.16249404	6.10525254	2995	3705	3276
91151	NM_033208	TIGD7	tigger transposable element deriv	-1.116299022	-1.124463232	2.435831	2.1839	2.034888	3.19389071	3.05333783	3.04408938	320	383	350
5326	NM_002657	PLAGL2	PLAG1 like zinc finger 2	1.210992837	-1.124501367	6.658106	8.074822	5.56394	5.23566562	5.50518749	5.0711259	1440	2330	1575
4350	NM_001015052,NM_001015054	MPG	N-methylpurine DNA glycosylase	1.134943804	-1.124664625	42.808071	48.286177	35.451348	5.5459881	5.72494281	5.38032669	1795	2722	1963
79832	NM_001076786	QSER1	glutamine and serine rich 1	-1.174988123	-1.124721974	8.806279	7.501743	7.356727	6.3408416	6.11129818	6.17348505	3143	3574	3437
113675	NM_001304993,NM_138432	SDSL	serine dehydratase like	1.027993636	-1.124826185	5.178452	5.324347	4.324026	2.91572639	2.95035633	2.7696269	257	353	281
283951	NM_001272051	C16orf91	chromosome 16 open reading fra	1.244601288	-1.124836609	14.593987	18.184353	12.18402	3.89793981	4.19452969	3.74022131	546	908	597
29780	NM_001003828,NM_001243385	PARVB	parvin beta	1.012708307	-1.125012547	5.394992	5.485177	4.493722	3.32434579	3.3407609	3.17224778	354	479	387
521	NM_007100,NR_033743	ATP5I	ATP synthase, H+ transporting, r	-1.293932398	-1.12513873	293.003779	227.580966	244.779028	6.71317506	6.34543779	6.54478691	4080	4213	4460
8742	NM_003809,NR_037146	TNFSF12	TNF superfamily member 12	-2.14819489	-1.125318045	2.425626	1.222493	2.089022	2.19091668	1.41062362	2.05953665	140	87	153
85451	NM_001080419,NR_038131	UNK	unkempt family zinc finger	-1.013203716	-1.125376728	19.083824	18.557149	15.330774	5.91217861	5.89356976	5.74476254	2325	3066	2541
115399	NM_198075	LRRC56	leucine rich repeat containing 56	1.006292759	-1.125730484	1.117607	1.127082	0.933229	2.01115992	2.01797632	1.88448459	119	160	130
6654	NM_005633	SOS1	SOS Ras/Rac guanine nucleotide	1.038497292	-1.125752549	6.32086	6.571709	5.275947	5.70589297	5.75936825	5.53846488	2010	2789	2196
4175	NM_005915	MCM6	minichromosome maintenance co	1.460396625	-1.125958652	28.497267	41.67041	23.788254	6.72919642	7.27127508	6.5597495	4126	8051	4507
23586	NM_014314	DDX58	DExD/H-box helicase 58	-1.635322924	-1.12597115	6.731563	4.120839	5.618095	5.00794161	4.32649813	4.84239278	1224	1000	1337
144402	NM_153634	CPNE8	copine 8	-1.262940641	-1.125982706	8.351997	6.620658	6.968253	4.86520493	4.54134004	4.70022518	1105	1169	1207
23331	NM_001145418	TTC28	tetratricopeptide repeat domain 2	1.829001325	-1.12599293	0.02218	0.044771	0.020302	0.35634914	0.59904473	0.32020933	11	27	12
653604	NM_001123375	HIST2H3D	histone cluster 2 H3 family memb	1.896385267	-1.12599293	0.583982	1.165444	0.513922	0.35634914	0.61708849	0.32020933	11	28	12

2826	NM_016602	CCR10	C-C motif chemokine receptor 10	-1.82246816	-1.12599293	0.227397	0.111158	0.176745	0.35634914	0.20477631	0.32020933	11	8	12
574406	NR_104133	ADAMTSL4-AS1	ADAMTSL4 antisense RNA 1	1.289929791	-1.12599293	0.257774	0.346944	0.22043	0.35634914	0.44594451	0.32020933	11	19	12
493900	NR_073431	TMEM9B-AS1	TMEM9B antisense RNA 1	1.063475363	-1.12599649	1.869278	1.999018	1.558703	1.55375576	1.61299082	1.4430085	76	108	83
4486	NM_001244937,NM_001318913	MST1R	macrophage stimulating 1 recept	1.118069335	-1.125997089	28.409016	31.948733	23.633523	6.95182336	7.11160519	6.78208367	4821	7202	5266
3720	NM_001267040,NM_004973	JARID2	jumonji and AT-rich interaction do	-1.453297965	-1.12606558	2.974999	2.046267	2.483695	4.22318689	3.71835988	4.0615736	694	638	758
5994	NM_000538	RFXAP	regulatory factor X associated pro	1.505160903	-1.126270155	1.608324	2.428038	1.341541	2.44147625	2.93964205	2.30298238	174	350	190
22796	NM_001145036,NM_007357	COG2	component of oligomeric golgi co	-1.010939398	-1.126402041	12.112765	11.994035	10.106571	5.16675115	5.15149273	5.00008264	1371	1812	1497
28960	NM_001350236,NM_014026	DCPS	decapping enzyme, scavenger	-1.853551132	-1.12642643	6.395748	3.457183	5.339413	5.2001212	4.34285452	5.03330706	1404	1012	1533
27440	NM_017829,NM_033070	HDHD5	haloacid dehalogenase like hydro	1.727733233	-1.126522962	28.483849	49.350123	23.775025	5.67097747	6.44791403	5.5026688	1961	4527	2141
84971	NM_001281504,NM_032885,NR	ATG4D	autophagy related 4D cysteine pe	-1.354667789	-1.126541321	15.268062	11.481324	12.864671	4.94013441	4.51872225	4.77415178	1166	1150	1273
220979	NM_001039380,NR_126421	C10orf25	chromosome 10 open reading fra	2.002289658	-1.126712672	0.181903	0.367657	0.152513	0.64188297	1.08728891	0.58209675	22	59	24
9828	NM_014786	ARHGEF17	Rho guanine nucleotide exchang	-1.015254864	-1.126749378	8.260756	8.148623	6.891519	5.99690152	5.97540322	5.82758721	2468	3248	2694
10204	NM_001322038,NM_001322039	NUTF2	nuclear transport factor 2	1.071525853	-1.126787062	39.301393	42.610673	32.750136	6.54076391	6.63939866	6.37050671	3616	5177	3947
51714	NM_016275	SELENOT	selenoprotein T	1.107453481	-1.126921515	46.119526	51.129135	38.455475	7.30776474	7.45412946	7.13652966	6181	9146	6746
51807	NM_001193414,NM_018943	TUBA8	tubulin alpha 8	2.324373871	-1.127148478	0.671946	1.569507	0.560541	1.26358189	2.09080288	1.16514558	55	171	60
5376	NM_000304,NM_001281455,NM	PMP22	peripheral myelin protein 22	-1.415895189	-1.127257898	1.230392	0.928439	1.094725	1.69664266	1.36863803	1.57922529	88	83	96
9263	NM_004760	STK17A	serine/threonine kinase 17a	-1.289933208	-1.127309037	15.046784	11.678969	12.546903	5.87384069	5.51364037	5.70407737	2263	2344	2469
100507062	NR_038286	PSMD6-AS2	PSMD6 antisense RNA 2	1.167014049	-1.127328138	1.466439	1.708762	1.219206	2.21486103	2.39263351	2.08088	143	223	156
11162	NM_007083,NM_198041	NUDT6	nudix hydrolase 6	-1.221515316	-1.127394939	7.985638	6.58297	6.750587	3.31698997	3.05994879	3.16227031	352	385	384
83448	NM_001098614,NM_001098615	PUS7L	pseudouridylate synthase 7 like	-1.354023738	-1.127404978	2.831355	2.192115	2.364231	3.64240927	3.24537964	3.48400652	451	445	492
84246	NM_032286	MED10	mediator complex subunit 10	1.050325146	-1.127541645	19.609617	20.617495	16.338406	4.36724694	4.43473883	4.20292915	771	1082	841
25948	NM_015483	KBTBD2	kelch repeat and BTB domain co	-1.041952325	-1.127667985	19.899876	19.122448	16.58515	6.18794463	6.12948304	6.01711924	2823	3620	3079
54762	NM_001172105,NM_017577	GRAMD1C	GRAM domain containing 1C	1.00694559	-1.127810876	5.364325	5.242176	4.361792	4.26965076	4.27912189	4.10562676	718	966	783
89891	NM_052844	WDR34	WD repeat domain 34	-1.128539933	-1.127980709	77.20459	68.488518	64.319778	7.10515387	6.93203905	6.93274811	5366	6353	5851
60489	NM_001349436,NM_001349437	APOBEC3G	apolipoprotein B mRNA editing e	1.372287682	-1.127994764	18.450223	25.609218	15.415935	5.1220776	5.56740891	4.95359477	1328	2435	1448
4704	NM_005002	NDUFA9	NADH:ubiquinone oxidoreductas	-1.119964433	-1.128129847	50.626106	45.252022	42.172661	6.32763402	6.16632849	6.15599403	3114	3715	3395
23640	NM_001130106,NM_001297600	HSPBP1	HSPA (Hsp70) binding protein 1	-1.18493021	-1.128284772	31.907617	27.233663	26.840221	5.90912433	5.66874311	5.73806086	2320	2616	2529
1937	NM_001404	EEF1G	eukaryotic translation elongation	1.10443237	-1.12834567	660.671204	730.477173	550.230652	9.95175578	10.09492348	9.7777331	38841	57316	42338
23173	NM_015143	METAP1	methionyl aminopeptidase 1	-1.19663062	-1.12839802	36.941704	30.905882	30.767187	6.65310266	6.39693301	6.48066075	3912	4368	4264
51228	NM_016433	GLTP	glycolipid transfer protein	-1.230696781	-1.128402772	12.832059	10.437033	10.681881	4.92811449	4.63950051	4.75988697	1156	1255	1260
29923	NM_001098786,NM_013332	HILPDA	hypoxia inducible lipid droplet ass	1.054318248	-1.128421361	6.792348	7.389592	5.723835	3.37126564	3.44039495	3.21470347	367	517	400
100130691	NR_026966	LOC100130691	uncharacterized LOC100130691	1.290370466	-1.128542241	0.782254	1.010718	0.652342	2.3142594	2.61543261	2.1765086	156	269	170
55847	NM_018464	CISD1	CDGSH iron sulfur domain 1	-1.662381967	-1.128561117	8.142866	4.903074	6.788995	4.14848821	3.46799726	3.98439467	657	528	716
23510	NM_015353,NR_110834,NR_11	KCTD2	potassium channel tetramerizatio	-1.442376067	-1.128701148	16.784254	11.64298	13.973675	5.94237097	5.42423685	5.77071509	2375	2200	2588
4833	NM_001286433,NM_001286435	NME4	NME/NM23 nucleoside diphosph	-1.098067302	-1.128703691	71.601504	63.311611	58.558077	6.2582797	6.12515529	6.08602965	2966	3609	3232
5990	NM_000635,NM_134433	RFX2	regulatory factor X2	1.102704563	-1.128712471	2.266317	2.504526	1.90533	3.33530968	3.46301778	3.1788613	357	526	389
197370	NM_145080	NSMCE1	NSE1 homolog, SMC5-SMC6 co	1.069093437	-1.12877541	55.239601	59.123207	45.992374	5.86125307	5.95604059	5.68967788	2243	3204	2444
5936	NM_001198843,NM_001198844	RBM4	RNA binding motif protein 4	1.482062598	-1.128848202	58.832907	91.543641	49.830405	6.75426196	7.31752681	6.58112647	4199	8315	4575
57210	NM_001080431,NM_001286646	SLC45A4	solute carrier family 45 member 4	1.017165568	-1.128895665	3.718753	3.797943	3.247705	4.57114104	4.59467379	4.40400834	894	1215	974
79019	NM_001002876,NM_001110215	CENPM	centromere protein M	-1.044743389	-1.129010206	17.685426	17.317713	14.899423	4.13181574	4.07233457	3.96730515	649	830	707
407043	NR_029605	MIR7-1	microRNA 7-1	-1.019030255	-1.129168921	10.697631	10.379507	8.750937	1.10178443	1.08728891	1.01060517	45	59	49
23140	NM_015113	ZZEF1	zinc finger ZZ-type and EF-hand	-1.127691986	-1.129293418	3.477602	3.086718	2.893066	5.30701783	5.13827799	5.13628808	1515	1795	1650
107984974	NM_001350575,NM_001350578	LOC107984974	uncharacterized LOC107984974	-1.035777811	-1.129571727	1.698211	1.640062	1.429842	2.40724866	2.36620557	2.26619062	169	218	184
126208	NM_001002836,NM_001351682	ZNF787	zinc finger protein 787	1.080109679	-1.129786492	20.014487	21.634655	16.079958	5.2722583	5.38067227	5.10103126	1478	2133	1609
10413	NM_001130145,NM_001195044	YAP1	Yes associated protein 1	-1.863207477	-1.129910577	19.11464	10.293248	15.930074	6.65602068	5.77049531	6.48166407	3920	2811	4267
6382	NM_001006946,NM_002997	SDC1	syndecan 1	1.195181525	-1.129959983	108.775209	129.551225	89.679497	8.41239272	8.66893268	8.23666967	13337	21298	14517
8772	NM_003824	FADD	Fas associated via death domain	1.214241871	-1.130133191	19.654121	23.901548	16.346752	5.18908339	5.46216384	5.01771306	1393	2260	1516
3614	NM_000883,NM_001102605,NM	IMPDH1	inosine monophosphate dehydro	-1.128202671	-1.130365202	15.917911	14.378264	13.071872	5.27035534	5.10110034	5.09841735	1476	1748	1606
9704	NM_014681	DHX34	DExH-box helicase 34	1.063383711	-1.13041766	14.544246	15.48373	12.088958	5.97545281	6.06275147	5.80157562	2431	3454	2645
100506060	NR_033959	SMG1P7	SMG1P7, nonsense mediated m	-1.419855437	-1.130464588	12.423471	8.753916	10.319908	4.57731129	4.09665142	4.40823586	898	845	977
60558	NM_001345867,NM_001345868	GUF1	GUF1 homolog, GTPase	1.159324685	-1.130807908	13.198684	15.342293	10.986717	5.78266256	5.99235095	5.60872295	2122	3287	2308
8721	NM_001281297,NM_001281298	EDF1	endothelial differentiation related	-1.058404666	-1.130829926	348.37055	328.055191	288.406434	7.92194234	7.84039747	7.74533877	9482	11970	10313
25873	NM_015414,NM_033643	RPL36	ribosomal protein L36	-1.239673567	-1.130880456	1036.883663	836.688332	862.130512	8.7921584	8.48297578	8.61513662	17365	18716	18886
84310	NM_001134395,NM_001134396	C7orf50	chromosome 7 open reading fran	-1.14948674	-1.13099065	28.400641	24.673886	24.215373	5.22489744	5.02964715	5.0523404	1429	1661	1554
7775	NM_001320952,NM_001320953	ZNF232	zinc finger protein 232	-1.054534131	-1.131020553	2.849619	2.716172	2.367234	2.64317467	2.57907432	2.49540988	206	261	224
8711	NM_001251902,NM_003985	TNK1	tyrosine kinase non receptor 1	1.468756664	-1.131129625	4.3144	6.361778	3.58725	3.72809498	4.24761625	3.56449618	481	944	523
253725	NM_001169106,NM_001169107	WASHC2C	WASH complex subunit 2C	-1.483757846	-1.131283205	7.265739	4.855779	6.022626	5.08791068	4.53897585	4.91549289	1296	1167	1409
51142	NM_001320327,NM_016139	CHCHD2	coiled-coil-helix-coiled-coil-helix c	-1.302867823	-1.131355063	254.634063	195.670507	211.51227	7.90410426	7.52423154	7.72684115	9365	9604	10181
50618	NM_001348181,NM_001348182	ITSN2	intersectin 2	-1.234607268	-1.131467174	8.369736	6.819098	6.969591	5.75435052	5.45653796	5.57965517	2080	2251	2261
7525	NM_005433	YES1	YES proto-oncogene 1, Src famil	1.132538814	-1.131501346	21.756348	24.668833	18.068434	6.64761539	6.82549553	6.47126268	3897	5897	4236
80139	NM_025069	ZNF703	zinc finger protein 703	1.241729989	-1.131572717	6.91495	8.603528	5.742573	4.5587208	4.85913858	4.38840055	886	1470	963
25988	NM_001243259,NM_001351957	HINFP	histone H4 transcription factor	-1.589773079	-1.131620634	10.328391	6.424413	8.762678	4.61228705	3.97774828	4.44161737	921	774	1001
64426	NM_022491	SUDS3	SDS3 homolog, SIN3A corepress	-1.284250896	-1.131652556	28.223923	21.998604	23.437643	7.03263119	6.67482436	6.85564592	5101	5307	5544
64127	NM_001293557,NM_022162	NOD2	nucleotide binding oligomerizatio	-3.014536448	-1.131814996	0.606043	0.19692	0.501713	1.86749926	0.90836632	1.73991364	104	46	113
7799	NM_001007257,NM_001135610	PRDM2	PR/SET domain 2	-1.008920529	-1.131857294	7.420888	6.900502	6.341586	5.4373936	5.42487721	5.26307232	1662	2201	1806
51608	NM_015949	GET4	golgi to ER traffic protein 4	-1.231384632	-1.131869263	63.190857	51.370434	52.464489	7.01654388	6.71883131	6.83930098	5044	5473	5481
100289361	NR_034032	LOC100289361	uncharacterized LOC100289361	-1.011441822	-1.131892864	3.394733	3.36252	2.80877	1.61502061	1.60397149	1.4969322	81	107	88
55107	NM_018043,NR_030691	ANO1	anoctamin 1	-1.566493757	-1.131919704	67.124982	43.002913	56.066336	8.29046811	7.64552993	8.11230249	12253	10451	13314
29102	NM_001100412,NM_013235	DROSHA	drosha ribonuclease III	1.307745688	-1.13203956	8.635261	11.269681	7.149535	5.53493448	5.91469537	5.3601012	1781	3112	1935
390	NM_001254738,NM_005168	RND3	Rho family GTPase 3	-1.169532804	-1.132231446	32.251928	27.055561	26.681991	6.44616075	6.22302312	6.26917183	3384	3866	3676
100506668	NR_038854	NRAV	negative regulator of antiviral res	1.464410969	-1.132424279	2.561688	3.750965	2.126446	2.52045395	2.98897501	2.37386535	186	364	202
22913	NM_007367,NM_016732	RALY	RALY heterogeneous nuclear rib	1.118554547	-1.132462443	37.100512	41.584952	30.828819	7.43659189	7.59734697	7.25822826	6762	10106	7344
283927	NM_001105663,NM_001243657	NUDT7	nudix hydrolase 7	-1.105446026	-1.132549014	3.964697	3.634404	3.299479	2.36507395	2.24959721	2.22204636	163	197	177
55605	NM_001173463,NM_001173464	KIF21A	kinesin family member 21A	-1.362809028	-1.132662518	5.919915	4.349718	4.910904	5.27320884	4.8400608	5.09841735	1479	1450	1606
10636	NM_006480	RGS14	regulator of G protein signaling 14	-1.52125938	-1.132755087	6.33595	4.163584	5.253962	3.98637962	3.42767171	3.81854297	583	512	633
253152	NM_173567	EPHX4	epoxide hydrolase 4	1.334816423	-1.132766314	4.432463	5.934566	3.67212	2.85607368	3.22198991	2.70236404	245	437	266
8877	NM_001142601,NM_001142602	SPHK1	sphingosine kinase 1	-1.21890002	-1.132791332	5.210034	4.409019	4.268223	3.43381096	3.17708507	3.27149895	385	422	418
157697	NM_001303100,NM_207332	ERICH1	glutamate rich 1	1.211041043	-1.132908334	8.181168	9.221284	6.936595	3.98869623	4.24906335	3.82065902	584	945	634
8928	NM_003923	FOXH1	forkhead box H1	1.0860304	-1.132992761	0.97366	1.068348	0.814097	1.62696745	1.70865125	1.50747923	82	119	89
92797	NM_033647,NR_135080,NR_13	HELB	DNA helicase B	-1.172347898	-1.133166361	0.963692	0.791156	0.795441	2.09955541	1.92688293	1.96321628	129	147	140
221710	NM_001135575	SMIM13	small integral membrane protein	-1.073106264	-1.133189643	2.547819	2.375904	2.113194	3.65995838	3.56646274	3.49464812	457	569	496
8347	NM_003526	HIST1H2BC	histone cluster 1 H2B family men	1.100145054	-1.133496086	11.93751	13.145288	9.868974	2.60744196	2.72348335	2.45783582	200	294	217
138050	NM_152419	HGSNAT	heparan-alpha-glucosaminide N-	-1.455561702	-1.133525384	18.523092	12.739957	15.357306	6.57045277	6.03575691	6.39165578	3692	3389	4006
100272147	NM_001018024	CMC4	C-X9-C motif containing 4	-1.031745954	-1.133538487	10.302048	10.004662	8.553807	3.4839374	3.44292618	3.32017262	400	518	434
266977	NM_025048,NM_153840	ADGRF1	adhesion G protein-coupled rece	-1.083424261	-1.133576792	46.554095	43.128859	39.6066	7.87669215	7.76160445	7.69662739	9188	11331	9969
284565	NM_001170755,NM_173638	NBPF15	NBPF member 15	1.095775077	-1.133636112	14.19828	15.272946	11.582524	5.97895271	6.10890925	5.80103986	2437	3568	2644
100170841	NM_001130677	EPOP	elongin BC and polycomb repres	-1.440187931	-1.133652777	5.146358	3.576001	4.26832	4.10430518	3.61440811	3.93446197	636	590	690
55911	NM_018690	APOBR	apolipoprotein B receptor	-1.176397135	-1.133702565	0.811426	0.688681	0.673927	2.00201503	1.82964782	1.86820921	118	134	128
124739	NM_001267576,NM_153210	USP43	ubiquitin specific peptidase 43	-1.379947464	-1.133713485	4.147942	3.007848	3.431351	4.15469105	3.72044785	3.98439467	660	639	716
25803	NM_001252294,NM_012391	SPDEF	SAM pointed domain containing E	-7.548560965	-1.133904578	0.163885	0.020482	0.125318	0.38477185	0.05398282	0.34394329	12	2	13
280	NM_020978	AMY2B	amylase, alpha 2B (pancreatic)	1.608413567	-1.133980534	4.690701	7.552139	3.880577	3.49049124	4.12687582	3.32614297	402	864	436
329	NM_001166,NM_001256163,NM	BIRC2	baculoviral IAP repeat containing	-1.408174272	-1.133989296	16.284464	11.572827	13.488632	5.93578265	5.4515187	5.75752106	2364	2243	2564
8454	NM_003592	CUL1	cullin 1	-1.183697241	-1.134022361	21.394449	18.095947	17.73118	6.08452478	5.84511405	5.90591399	2625	2963	2847
89894	NM_001193453,NM_001193531	TMEM116	transmembrane protein 116	-1.342932348	-1.134316431	3.269749	2.37553	2.681312	2.54584744	2.20257223	2.39673966	190	189	206
11214	NM_001270546,NM_006738,NM	AKAP13	A-kinase anchoring protein 13	-1.127360424	-1.134340413	5.429461	5.390766	5.778724	6.15841002	5.98802432	5.97925949	2765	3277	2998
468	NM_001675,NM_182810	ATF4	activating transcription factor 4	1.207418562	-1.134364285	808.812835	994.158967	698.057968	10.25906251	10.53078669	10.07733622	48071	77551	52121
9406	NM_005455,NM_203350	ZRANB2	zinc finger RANBP2-type contain	1.217710213	-1.134576543	43.995949	53.754014	36.68574	7.07957236	7.36184017	6.89884903	5271	8576	5714
100132057	NR_111930,NR_111930_dup1	LOC100132057	phosphodiesterase 4D interacting	-1.45463322	-1.134610985	0.415364	0.283545	0.33918	0.68823103	0.50527715	0.62147005	24	22	26
64848	NM_001345975,NM_001345976	YTHDC2	YTH domain containing 2	-1.007230477	-1.134692736	14.484602	14.502296	12.067664	6.48565198	6.47537422	6.30551091	3479	4615	3771

163087	NM_001345947,NM_001345948	ZNF383	zinc finger protein 383	-1.470695788	-1.134822925	4.653489	3.122555	3.859615	3.42357323	2.92884759	3.25906955	382	347	414
197322	NM_001127214,NM_001243279	ACSF3	acyl-CoA synthetase family mem	1.379901933	-1.134935648	6.065612	8.092565	4.172415	4.26774435	4.71159881	4.09517603	717	1322	777
8701	NM_001277115	DNAH11	dynein axonemal heavy chain 11	-1.345729723	-1.134944959	0.517485	0.3839	0.427996	3.00082115	2.63327382	2.84224948	275	273	298
102723166	NR_110370	STAM-AS1	STAM antisense RNA 1 (head to	-1.027735586	-1.135038418	0.595506	0.579428	0.492168	1.33814853	1.31437719	1.23026757	60	78	65
4665	NM_001330305,NM_005967	NAB2	NGFI-A binding protein 2	1.167453036	-1.135184201	7.545672	8.689733	5.995713	4.31651022	4.52948015	4.14331116	743	1159	805
5356	NM_001201564,NM_002669	PLRG1	pleiotropic regulator 1	1.2168532	-1.135250111	20.004694	24.364816	16.550912	6.05221726	6.33150339	5.87213616	2566	4172	2780
79892	NM_001256378,NM_001256379	MCMBP	minichromosome maintenance co	1.093632649	-1.135318644	16.80997	18.39972	13.910352	6.15583539	6.28323493	5.97546577	2760	4033	2990
79668	NM_001178055,NM_001178056	PARP8	poly(ADP-ribose) polymerase fan	1.250300996	-1.135461562	6.523412	8.171598	5.406891	5.54283855	5.85892233	5.36373375	1791	2992	1940
64750	NM_022739	SMURF2	SMAD specific E3 ubiquitin prote	-2.057957038	-1.135737204	38.426151	18.690588	31.793724	7.18636607	6.15556624	7.00407706	5679	3687	6150
55012	NM_001305155,NM_001305156	PPP2R3C	protein phosphatase 2 regulatory	-1.039448342	-1.135858281	5.88621	5.75793	4.988487	3.66576097	3.61440811	3.4972963	459	590	497
100288142	NM_001278267	NBPF20	NBPF member 20	-2.772186328	-1.135920591	0.608864	0.219873	0.504159	3.51962058	2.255369	3.35270793	411	198	445
800	NM_004342,NM_033138,NM_03	CALD1	caldesmon 1	1.238240379	-1.135951902	8.333689	10.260614	6.96677	5.16162707	5.46216384	4.98317903	1366	2260	1479
29964	NM_013397	PRICKLE4	prickle planar cell polarity protein	-2.002482486	-1.136156278	11.353086	5.675784	9.389841	4.26392396	3.33532144	4.08992214	715	477	774
55626	NM_001267782,NM_001267783	AMBRA1	autophagy and beclin 1 regulator	-1.357940715	-1.136441776	11.266291	8.212471	9.408029	5.81826596	5.3859437	5.63721662	2176	2141	2355
8416	NM_003568	ANXA9	annexin A9	-1.576150543	-1.136452459	15.344387	9.737502	12.680588	4.82038759	4.19302682	4.64278115	1070	907	1158
7436	NM_001018056,NM_001322225	VLDLR	very low density lipoprotein recep	2.341105923	-1.136473252	0.301568	0.71036	0.24619	1.35261029	2.21447295	1.24294651	61	191	66
7342	NM_001128160,NM_001128161	UBP1	upstream binding protein 1 (LBP-	1.144231599	-1.136606024	15.156826	17.445559	12.478318	5.94237097	6.13379784	5.7608309	2375	3631	2570
10478	NM_001282726,NM_001282727	SLC25A17	solute carrier family 25 member 1	1.123851059	-1.136805838	25.036712	28.266995	21.072018	5.81631087	5.98194516	5.63481342	2173	3263	2351
51103	NM_016013,NR_045620	NDUFAF1	NADH:ubiquinone oxidoreductas	-1.120549716	-1.136852079	12.699346	11.459825	10.553277	4.29794868	4.14253265	4.12287815	733	874	793
755	NM_001271440,NM_001271441	C21orf2	chromosome 21 open reading fra	-1.669880837	-1.136875325	4.86216	3.000654	4.085701	3.60967076	2.94679374	3.44063736	440	352	476
134145	NM_001258388,NM_001258389	FAM173B	family with sequence similarity 17	1.005641206	-1.137000359	3.467697	3.500549	2.881281	3.32800968	3.33532144	3.16227031	355	477	384
134430	NM_139281	WDR36	WD repeat domain 36	-1.019050201	-1.137087778	10.789455	10.598281	8.915464	6.13454836	6.1077133	5.95200904	2719	3565	2941
157574	NM_001258211,NM_172366	FBXO16	F-box protein 16	-1.262296904	-1.137156159	1.671331	1.314646	1.366807	1.67378945	1.45142181	1.5489128	86	91	93
100507266	NR_037888	STX18-AS1	STX18 antisense RNA 1 (head to	1.49543595	-1.137217126	0.389075	0.584459	0.322272	0.95786335	1.26944952	0.87055986	37	74	40
26147	NM_001009936,NM_001286840	PHF19	PHD finger protein 19	-1.408800818	-1.137487529	54.674691	57.236038	58.779673	6.63989799	6.15131615	6.4560292	3876	3676	4191
1070	NM_001297765,NM_001297768	CETN3	centrin 3	-1.379454365	-1.137757915	43.267166	31.387913	35.66675	5.87133194	5.41653033	5.68851969	2259	2188	2442
56901	NM_020142	NDUFA4L2	NDUFA4, mitochondrial complex	-1.077162126	-1.138123576	0.542715	0.498254	0.440447	0.71085839	0.669904	0.64076093	25	31	27
10061	NM_005692,NM_007189	ABCF2	ATP binding cassette subfamily F	-1.053745697	-1.138264719	12.655227	12.097373	10.64361	5.83252285	5.75835243	5.64917295	2198	2787	2375
79058	NM_001251888,NM_001330528	ASPSCR1	ASPSCR1, UBX domain containi	1.017155454	-1.138327849	25.508225	25.901687	20.942789	5.5514833	5.57550598	5.36880401	1802	2449	1947
138428	NM_001002913,NM_001345977	PTRH1	peptidyl-tRNA hydrolase 1 homol	-1.111011177	-1.138365466	7.43716	6.618301	6.129249	3.15728865	3.0232078	2.99246523	311	374	336
389641	NR_033928	LOC389641	uncharacterized LOC389641	1.066132671	-1.138546628	2.813353	3.001077	2.31939	2.6780437	2.75641772	2.52166171	212	302	229
127002	NM_001350174,NM_001350175	ATXN7L2	ataxin 7 like 2	1.129679645	-1.138588327	4.167315	4.958816	3.584892	3.56068436	3.7225328	3.39023607	424	640	458
7039	NM_001099691,NM_001308158	TGFA	transforming growth factor alpha	1.560509404	-1.138792012	37.301348	58.504699	30.859465	7.30869218	7.9474459	7.12244674	6185	12896	6680
483	NM_001679	ATP1B3	ATPase Na+/K+ transporting sub	1.230842971	-1.139042661	102.302551	126.066101	84.406578	7.56962894	7.86785453	7.38285969	7419	12201	8011
101927181	NR_108066	LOC101927181	uncharacterized LOC101927181	-1.202097309	-1.139216573	0.792307	0.654617	0.650658	1.38110603	1.22307764	1.26797492	63	70	68
25929	NM_001252156,NM_015465	GEMIN5	gem nuclear organelle associated	1.167532333	-1.139222661	9.118409	10.658118	7.521194	5.61434375	5.83358571	5.43037665	1884	2939	2034
126074	NM_175871	SWSAP1	SWIM-type zinc finger 7 associat	1.169618398	-1.139270094	2.362373	2.776961	1.949624	2.27683528	2.45916281	2.1294865	151	236	163
375248	NM_001164315	ANKRD36	ankyrin repeat domain 36	-1.272289529	-1.139334272	6.820925	5.366833	5.62729	5.41431354	5.07604389	5.23081505	1635	1717	1765
8570	NM_003685	KHSRP	KH-type splicing regulatory protei	1.193832353	-1.139461722	43.793369	52.341599	36.11742	7.1250342	7.37895999	6.93811829	5441	8679	5873
5578	NM_002737	PRKCA	protein kinase C alpha	-1.071258459	-1.139508815	9.734729	9.097347	8.029485	6.39468041	6.29659183	6.20865166	3264	4071	3523
140461	NM_001319296,NM_001319297	ASB8	ankyrin repeat and SOCS box co	-1.090245904	-1.139523277	6.38583	5.875223	5.237934	4.11918371	4.00198255	3.94225731	643	788	694
23132	NM_001322253,NM_001322256	RAD54L2	RAD54-like 2 (S. cerevisiae)	1.115819853	-1.139533652	2.430465	2.715397	2.003314	4.63020414	4.7822652	4.44984345	933	1391	1007
10456	NM_001018837,NM_006118	HAX1	HCLS1 associated protein X-1	-1.099081149	-1.139601516	73.025458	66.237217	59.814185	6.39074431	6.25614433	6.20460629	3255	3957	3513
283254	NM_173811	HARBI1	harbinger transposase derived 1	1.115977587	-1.139783323	1.942499	2.186473	1.612355	1.96484452	2.08433212	1.8266989	114	170	123
646851	NM_001013647,NM_001291030	FAM227A	family with sequence similarity 22	2.323599423	-1.139834463	1.756898	4.095562	1.448175	4.22711655	5.39903863	4.04899377	696	2161	751
6130	NM_000972	RPL7A	ribosomal protein L7a	-1.02178363	-1.139880888	1888.250366	1850.019531	1556.65979	10.67702634	10.64595576	10.48826617	64238	84000	69313
79882	NM_001160103,NM_001160104	ZC3H14	zinc finger CCCH-type containing	1.252271136	-1.139972988	23.75839	29.666364	19.388134	6.56386469	6.88534475	6.37699189	3675	6149	3965
7782	NM_001321036,NM_013309	SLC30A4	solute carrier family 30 member 4	-1.098946554	-1.139981167	0.968321	0.961436	0.725827	2.83562844	2.71931318	2.67455554	241	293	260
56942	NM_001351967,NM_001351968	CMC2	C-X9-C motif containing 2	-1.367744299	-1.140051493	73.744987	54.709575	63.276331	5.91766008	5.47458779	5.73189018	2334	2280	2518
10256	NM_001297647,NM_001297648	CNKSR1	connector enhancer of kinase su	-1.05370431	-1.140213267	20.261028	19.259046	16.700325	5.6774542	5.60349311	5.49208641	1970	2498	2125
9946	NM_145858	CRYZL1	crystallin zeta like 1	-1.61568415	-1.140323525	11.478411	7.111389	9.460429	4.33118941	3.68239411	4.15174023	751	621	810
4084	NM_001202513,NM_001202514	MXD1	MAX dimerization protein 1	-1.006889454	-1.140457084	6.351614	6.316351	5.234233	5.15957233	5.14994432	4.97560227	1364	1810	1471
3227	NM_014212	HOXC11	homeobox C11	-1.707605179	-1.140616724	2.296234	1.340521	1.889945	2.47491059	1.87534115	2.32103228	179	140	193
153743	NM_206858	PPP1R2P3	protein phosphatase 1 regulatory	1.207302992	-1.140697015	0.154868	0.200583	0.133825	0.41264541	0.48576948	0.36729312	13	21	14
389860	NM_001015038	PAGE2B	PAGE family member 2B	-2.150633409	-1.140697015	0.649001	0.308687	0.512136	0.41264541	0.20477631	0.36729312	13	8	14
56124	NM_018932	PCDHB12	protocadherin beta 12	-1.021559294	-1.140697015	0.096723	0.097403	0.08086	0.41264541	0.40498891	0.36729312	13	17	14
29907	NM_013306,NM_147777	SNX15	sorting nexin 15	-1.297909401	-1.140792996	14.999727	11.443486	12.191213	4.84361034	4.48227194	4.66061022	1088	1120	1173
8554	NM_001320687,NM_016166	PIAS1	protein inhibitor of activated STA	-1.050332867	-1.140799844	8.454503	8.059448	6.966554	5.59318572	5.52383844	5.40732977	1856	2361	2001
56834	NM_001170726,NM_001170880	GPR137	G protein-coupled receptor 137	1.032852688	-1.140845813	12.085523	12.495432	9.918814	4.53514067	4.5798	4.35374951	871	1202	939
92979	NM_138396	MARCH9	membrane associated ring-CH-ty	-1.539631255	-1.140996476	10.008075	6.504226	8.242072	4.91111656	4.31410799	4.72754788	1142	991	1231
80728	NM_001308207,NM_001308208	ARHGAP39	Rho GTPase activating protein 3	1.356555586	-1.141015272	2.651746	3.59458	2.187767	3.71136013	4.12214544	3.53644652	475	861	512
7343	NM_001076683,NM_001076684	UBTF	upstream binding transcription fa	1.50956928	-1.141031746	16.966129	26.390138	14.239173	6.32991983	6.91800682	6.14210075	3119	6291	3362
84287	NM_001287803,NM_001287804	ZDHHC16	zinc finger DHHC-type containing	-1.185555565	-1.141083406	19.527436	15.552422	15.842329	5.11679207	4.8789024	4.93222525	1323	1491	1426
81688	NM_030939	C6orf62	chromosome 6 open reading fran	-1.013760098	-1.141104852	31.942919	31.548054	26.307329	6.28350096	6.2640388	6.09567311	3019	3979	3254
28956	NM_001145264,NM_014017	LAMTOR2	late endosomal/lysosomal adapto	-1.334404064	-1.14114333	60.85974	45.870176	50.099082	5.3670153	4.96243097	5.18144687	1581	1583	1704
10237	NM_001278784,NM_005827	SLC35B1	solute carrier family 35 member E	1.108381706	-1.141165591	42.368891	46.964333	34.941123	6.06928257	6.21564068	5.88179555	2597	3846	2799
9466	NM_004843	IL27RA	interleukin 27 receptor subunit al	-1.275983237	-1.141272967	4.180014	3.277544	3.441927	3.56379523	3.24537964	3.39023607	425	445	458
64940	NR_040585,NR_040586	STAG3L4	stromal antigen 3-like 4 (pseudog	-1.633947999	-1.141361171	5.807691	3.557766	4.786966	3.71696001	3.07634485	3.54158719	477	390	514
493911	NM_001008489,NM_001199285	PHOSPHO2	phosphatase, orphan 2	1.775629614	-1.141381154	3.012986	5.428055	2.530672	2.20692365	2.89227137	2.05953665	142	337	153
100129196	NR_034182	MATN1-AS1	MATN1 antisense RNA 1	1.043558869	-1.141381154	1.808609	1.891904	1.491325	2.20692365	2.255369	2.05953665	142	198	153
23163	NM_001172703,NM_001172704	GGA3	golgi associated, gamma adaptin	-1.253166433	-1.141528959	35.427695	28.398863	29.057954	7.09631899	6.77340039	6.90683866	5333	5686	5746
55757	NM_020121	UGGT2	UDP-glucose glycoprotein glucos	1.126475396	-1.141609898	5.690552	6.420632	4.687072	4.8242841	4.99038671	4.64038721	1073	1615	1156
100506215	NR_038879,NR_038880	PAXBP1-AS1	PAXBP1 antisense RNA 1	2.299309955	-1.141617829	0.294826	0.670143	0.235825	0.99521185	1.71704117	0.90287695	39	120	42
144097	NM_138471	C11orf84	chromosome 11 open reading fra	1.408397316	-1.141641928	14.763014	20.831398	12.154661	4.92206677	5.40229387	4.73766195	1151	2166	1240
9726	NM_014699	ZNF646	zinc finger protein 646	1.135199689	-1.141649537	4.139546	4.704759	3.407543	4.70255223	4.8789024	4.51923721	983	1491	1059
148327	NM_001255978,NM_001255979	CREB3L4	cAMP responsive element bindin	1.128783894	-1.141650573	9.017315	10.303368	7.522312	4.04320744	4.20798559	3.86439381	608	917	655
285761	NM_173674	DCBLD1	discoidin, CUB and LCCL domair	-1.145175408	-1.141677532	3.161325	2.765552	2.601297	2.86114003	2.69403583	2.69776636	246	287	265
441072	NR_102755	FLJ31104	uncharacterized LOC441072	1.194216829	-1.141733779	0.587065	0.707031	0.484072	1.21691895	1.36863803	1.11084509	52	83	56
730394	NM_001042490	GTF2H2C_2	GTF2H2 family member C, copy	1.024833846	-1.14174182	14.653138	15.034617	12.052809	4.74016226	4.77424718	4.55653526	1010	1383	1088
6944	NM_001271087,NM_001271088	VPS72	vacuolar protein sorting 72 homo	-1.163258892	-1.141818168	32.666349	28.117747	26.905189	5.63962535	5.42615707	5.45237894	1918	2203	2066
80184	NM_025114	CEP290	centrosomal protein 290	-1.383409074	-1.141916859	6.824387	4.939884	5.617279	5.75162493	5.29359953	5.56395161	2076	2005	2236
10401	NM_006099	PIAS3	protein inhibitor of activated STA	1.148554199	-1.14198544	24.341604	27.997253	20.032522	6.12510269	6.32225302	5.93648189	2701	4145	2909
54475	NM_001014445,NM_018096	NLE1	notchless homolog 1	1.457709943	-1.142147711	9.556565	14.230078	8.016131	4.69263929	5.21876673	4.5087761	976	1901	1051
11234	NM_007216,NM_181507,NM_18	HPS5	HPS5, biogenesis of lysosomal o	-1.550144259	-1.142166864	15.054614	9.49945	11.99879	6.1214126	5.50033494	5.93257384	2694	2322	2901
94239	NM_012412,NM_138635,NM_20	H2AFV	H2A histone family member V	-1.326062386	-1.142388151	48.223203	37.631942	39.444043	6.93059262	6.52728454	6.74021788	4750	4786	5114
7844	NM_001198951,NM_001198952	RNF103	ring finger protein 103	1.102125838	-1.142765405	15.355568	16.815926	12.822824	5.70307419	5.84080171	5.51448193	2006	2954	2159
8193	NM_001135155,NM_001135156	DPF1	double PHD fingers 1	1.805068404	-1.142771316	1.019001	1.84784	0.841319	1.74129091	2.38738651	1.60891398	92	222	99
54986	NM_001322500,NM_001322501	ULK4	unc-51 like kinase 4	-1.302597523	-1.142884539	0.439256	0.33755	0.360843	1.59082596	1.34717796	1.46482043	79	81	85
56674	NM_001286094,NM_001286095	TMEM9B	TMEM9 domain family member E	-5.42210608	-1.143165755	33.763056	6.167249	26.322822	6.08289937	3.73497962	5.89289991	2622	646	2821
93664	NM_001009571,NM_001167940	CADPS2	calcium dependent secretion acti	-4.350965335	-1.143177447	2.551243	0.584284	2.096823	3.97940737	2.13530335	3.79935756	580	178	624
51335	NM_001033088,NR_028052	NGRN	neugrin, neurite outgrowth assoc	-1.019413251	-1.143237884	60.854423	60.095593	49.32117	6.66437729	6.63691362	6.47328171	3943	5168	4242
284390	NM_001012753	ZNF763	zinc finger protein 763	1.298566381	-1.14327744	0.524928	0.684175	0.430218	1.23264156	1.4614436	1.12461302	53	92	57
692072	NR_003033	SNORD5	small nucleolar RNA, C/D box 5	-1.753475647	-1.143400524	61.469948	34.941555	50.454578	2.4278825	1.80624674	2.27238811	172	131	185
51004	NM_182476,NM_182480	COQ6	coenzyme Q6, monooxygenase	1.035370142	-1.143454909	15.336021	15.910355	12.606044	4.63907992	4.68725264	4.45393896	939	1299	1010
23197	NM_014613	FAF2	Fas associated factor family men	1.105457889	-1.143479102	48.051201	53.174438	39.488361	7.7294977	7.8734949	7.53703992	8293	12249	8920
26273	NM_012175,NM_033406	FBXO3	F-box protein 3	-1.114233122	-1.143581952	11.51058	10.159315	9.870216	4.60173173	4.45243375	4.41665393	914	1096	983
84993	NM_001286739,NM_001286740	UBL7	ubiquitin like 7	1.072725932	-1.143644017	19.616411	20.870378	16.104543	4.84232994	4.94020727	4.6558773	1087	1558	1169
64858	NM_001319946,NM_001319947	DCLRE1B	DNA cross-link repair 1B	1.060269338	-1.143667116	3.562053	3.723789	2.877871	3.79050499	3.86901329	3.61169701	504	714	542
57664	NM_001161354,NM_020904	PLEKHA4	pleckstrin homology domain cont	-1.260904517	-1.143817864	2.711947	2.22456	2.249409	3.18986937	2.89597101	3.0185082	319	338	343

118924	NM_001347712,NM_001347713	FRA10AC1	FRA10A associated CGG repeat	-1.623340703	-1.143870027	7.557508	4.688604	6.247652	4.58651747	3.92440495	4.40118309	904	744	972
159195	NM_001320437,NM_001320441	USP54	ubiquitin specific peptidase 54	-2.612925242	-1.143881365	8.999235	3.318444	7.375816	5.72269098	4.38031337	5.53266778	2034	1040	2187
54878	NM_001320875,NM_001320876	DPP8	dipeptidyl peptidase 8	-1.39768588	-1.143998787	5.69316	4.142026	4.729046	5.3934833	4.92399154	5.20431761	1611	1540	1732
3189	NM_001322434,NM_001322436	HNRNPH3	heterogeneous nuclear ribonucle	-1.317649572	-1.144022102	152.091249	114.792762	124.051916	8.45824946	8.06158156	8.2647235	13769	13962	14803
24145	NM_015368	PANX1	pannexin 1	-1.010848295	-1.144150412	13.361279	13.235342	10.971281	5.21008282	5.19493791	5.02139743	1414	1869	1520
148113	NM_153221	CILP2	cartilage intermediate layer prote	-1.06683236	-1.144201673	0.665707	0.62253	0.547051	1.89739865	1.82964782	1.75769337	107	134	115
8517	NM_001099856,NM_001099857	IKBKG	inhibitor of nuclear factor kappa E	1.010722604	-1.144348927	12.982074	13.163469	10.69258	4.87149524	4.88636082	4.68404466	1110	1499	1193
552891	NM_004125	DNAJC25-GNG10	DNAJC25-GNG10 readthrough	-1.798059653	-1.144425455	0.461938	0.255348	0.376861	0.7550755	0.46599441	0.6785857	27	20	29
2967	NM_001271866,NM_001271867	GTF2H3	general transcription factor IIH su	-1.026892572	-1.144481572	12.231842	11.931252	10.050995	5.37854443	5.34118895	5.18883779	1594	2074	1713
51808	NM_032177	PHAX	phosphorylated adaptor for RNA	1.020087209	-1.14449444	12.603784	12.873582	10.349989	5.51417891	5.54225135	5.32400962	1755	2392	1886
25937	NM_001168278,NM_001168280	WWTR1	WW domain containing transcript	1.204768168	-1.144514503	30.450405	36.439007	24.836721	7.22691512	7.49403735	7.03356651	5842	9404	6278
2356	NM_001018078,NM_001288803	FPGS	folylpolyglutamate synthase	1.034804656	-1.145247224	31.620032	32.737213	25.903011	6.16917354	6.21785938	5.97641514	2786	3852	2992
57730	NM_025190	ANKRD36B	ankyrin repeat domain 36B	-1.339206365	-1.145373861	5.621076	4.203502	4.613911	5.07815362	4.67115044	4.88851253	1287	1284	1382
374395	NM_199337	TMEM179B	transmembrane protein 179B	1.031125802	-1.145384452	34.658607	35.779659	28.431807	5.17796048	5.22098063	4.98789438	1382	1904	1484
580	NM_000465,NM_001282543,NM	BARD1	BRCA1 associated RING domain	1.035883445	-1.145554532	8.966627	9.367813	7.621667	5.57557074	5.62538694	5.38390874	1833	2537	1968
402055	NM_001013694	SRRD	SRR1 domain containing	1.494207565	-1.145569989	28.838425	43.151817	23.666529	5.22194666	5.78852012	5.0314812	1426	2847	1531
3305	NM_005527	HSPA1L	heat shock protein family A (Hsp7	-1.439950273	-1.145619718	0.422379	0.292273	0.343387	1.03161781	0.7861039	0.93448595	41	38	44
23177	NM_001319100,NM_001319101	CEP68	centrosomal protein 68	1.641201615	-1.145658757	2.42712	4.011169	2.017533	3.89300127	4.56941219	3.71085992	544	1193	584
6158	NM_000991,NM_001136134,NM	RPL28	ribosomal protein L28	-1.375257377	-1.146099637	152.633424	120.526722	157.401713	8.84313812	8.38461163	8.64686323	17991	17479	19307
54520	NM_019044	CCDC93	coiled-coil domain containing 93	1.25514041	-1.146191751	10.439329	13.114908	8.558081	6.15841002	6.48215837	5.96450295	2765	4637	2967
9470	NM_001276336,NM_001276337	EIF4E2	eukaryotic translation initiation fa	1.254719719	-1.146193703	66.711288	81.201033	57.120648	6.34942974	6.67320833	6.15515581	3162	5301	3393
11091	NM_017588,NM_052821	WDR5	WD repeat domain 5	1.045660206	-1.146240991	32.257766	33.354226	26.135356	6.62471065	6.68848798	6.42993057	3835	5358	4115
8552	NR_024616	INE1	inactivation escape 1 (non-protei	-1.207068412	-1.1463642	5.346194	4.414639	4.369978	2.64317467	2.41858604	2.47942639	206	228	221
23248	NM_001297673,NM_001297674	RPRD2	regulation of nuclear pre-mRNA c	-2.751543591	-1.146398895	5.716508	2.123892	4.720275	5.54834575	4.14097459	5.35573003	1798	873	1929
8641	NM_003736,NM_032098	PCDHGB4	protocadherin gamma subfamily	-1.858298909	-1.146592043	0.148179	0.079081	0.118275	0.43999062	0.25173066	0.39027104	14	10	15
162514	NM_001258205,NM_145068	TRPV3	transient receptor potential cation	3.933586542	-1.146592043	0.055663	0.23538	0.047796	0.43999062	1.26944952	0.39027104	14	74	15
440804	NM_001128635,NM_001128636	RIMBP3B	RIMS binding protein 3B	2.129748427	-1.146592043	0.066916	0.14364	0.051693	0.43999062	0.81765451	0.39027104	14	40	15
728558	NR_038444,NR_134320,NR_134	ENTPD1-AS1	ENTPD1 antisense RNA 1	-1.038897293	-1.146592043	0.195341	0.289147	0.132501	0.43999062	0.42561204	0.39027104	14	18	15
677776	NR_003009	SCARNA8	small Cajal body-specific RNA 8	1.01561312	-1.146592043	2.65943	2.703723	2.202622	0.43999062	0.44594451	0.39027104	14	19	15
109623469	NR_145805	SNORD175	small nucleolar RNA, C/D box 17	-1.552229372	-1.146592043	4.83149	3.256155	3.969067	0.43999062	0.29720487	0.39027104	14	12	15
643155	NM_001048249	SMIM15	small integral membrane protein	-1.025221236	-1.146682274	35.90361	35.061039	29.428131	6.67304593	6.63746622	6.47764661	3967	5170	4255
23016	NM_015004,NR_023353	EXOSC7	exosome component 7	-1.09851279	-1.146959785	34.764967	31.737624	28.76855	5.21997612	5.08822232	5.02782252	1424	1732	1527
11187	NM_001303029,NM_007183	PKP3	plakophilin 3	1.100375064	-1.147092957	32.7814	35.92889	26.720295	6.51246517	6.64902225	6.31679881	3545	5212	3801
5279	NM_002642,NM_153747	PIGC	phosphatidylinositol glycan ancho	1.052064819	-1.147242295	19.713477	20.843025	16.228833	4.88896361	4.95978213	4.69792476	1124	1580	1205
102465978	NR_106909	MIR6850	microRNA 6850	1.015672566	-1.147262742	11.715461	11.988136	9.784874	0.77668602	0.7861039	0.69713258	28	38	30
729732	NR_047662	LOC729732	uncharacterized LOC729732	2.080793782	-1.147262742	0.181272	0.379833	0.145755	0.77668602	1.31437719	0.69713258	28	78	30
114789	NM_001006641,NM_001006642	SLC25A25	solute carrier family 25 member 2	-1.12549806	-1.147416947	18.379239	16.382159	14.894301	5.94416257	5.77652864	5.7492131	2378	2823	2549
100616487	NR_039742	MIR4517	microRNA 4517	1.460056948	-1.147487674	13.797048	20.210869	11.313072	1.04948198	1.3579479	0.95003448	42	82	45
28512	NM_020345	NKIRAS1	NFKB inhibitor interacting Ras lik	1.191769113	-1.147579235	3.471941	4.145529	2.837313	2.9541672	3.17708507	2.78271118	265	422	284
56270	NM_019613	WDR45B	WD repeat domain 45B	-1.161015761	-1.147772629	75.297012	64.925781	61.647209	7.57484227	7.36066928	7.37711996	7446	8569	7979
57620	NM_001169117,NM_001169118	STIM2	stromal interaction molecule 2	1.105727246	-1.147777595	5.36306	5.930515	4.397621	4.80994515	4.9500279	4.61866105	1062	1569	1138
65062	NM_001044385,NM_152388	TMEM237	transmembrane protein 237	1.465958323	-1.147913458	3.42659	5.058318	2.790134	4.28857758	4.81682937	4.10041086	728	1426	780
4771	NM_000268,NM_016418,NM_18	NF2	neurofibromin 2	-1.335432125	-1.14803192	18.139773	13.350833	15.149119	6.66292744	6.25037564	6.46586474	3939	3941	4220
9338	NM_001006639,NM_001006640	TCEAL1	transcription elongation factor A l	1.271411523	-1.148071748	9.4568	12.048649	7.735126	3.59454042	3.91531944	3.4128543	435	739	466
54850	NM_001316936,NM_001316937	FBXL12	F-box and leucine rich repeat pro	-1.005372635	-1.148226952	20.705241	20.703938	17.112359	5.32271189	5.3151747	5.12862225	1532	2036	1641
5094	NM_001098620,NM_001128911	PCBP2	poly(rC) binding protein 2	-1.058836687	-1.148410029	138.530659	130.932681	113.617462	8.70959998	8.62732208	8.51047202	16397	20691	17561
10180	NM_001167582,NM_001349190	RBM6	RNA binding motif protein 6	-1.195424938	-1.148541961	29.280401	24.222447	24.275886	6.69768198	6.44286462	6.49993558	4036	4511	4322
226	NM_000034,NM_001127617,NM	ALDOA	aldolase, fructose-bisphosphate A	1.748147287	-1.148558315	877.858257	1356.967306	701.331767	10.40815913	11.21353266	10.20849229	53309	124516	57086
80724	NM_001136538,NM_025247	ACAD10	acyl-CoA dehydrogenase family r	-1.279664498	-1.148868282	7.344079	5.737597	5.999934	4.90131278	4.55894904	4.7082479	1134	1184	1214
27301	NM_001271748,NM_014481	APEX2	apurinic/apyrimidinic endodeoxyr	1.024171025	-1.148929425	13.334826	13.732311	10.857871	4.81256286	4.84581067	4.61987668	1064	1456	1139
3816	NM_002257	KLK1	kallikrein 1	-2.618192999	-1.148947106	1.693034	0.636284	1.379626	1.29387258	0.63490936	1.17840727	57	29	61
55731	NM_001077498,NM_001288631	FAM222B	family with sequence similarity 22	-1.224410136	-1.149031466	12.372691	10.253729	10.280068	5.71081263	5.42487721	5.51448193	2017	2201	2159
3615	NM_000884	IMPDH2	inosine monophosphate dehydro	-1.091003229	-1.149048797	90.542076	83.08329	74.054039	7.22961095	7.10482771	7.03059884	5853	7168	6265
91039	NM_139159	DPP9	dipeptidyl peptidase 9	-1.455799421	-1.149073658	53.296833	36.6493	43.5858	7.79780563	7.2589381	7.59829777	8697	7982	9309
2768	NM_001282440,NM_001282441	GNA12	G protein subunit alpha 12	1.188368693	-1.149087954	12.632928	15.047807	10.337399	5.77998999	6.02481605	5.58339873	2118	3363	2267
2186	NM_004459,NM_182641	BPTF	bromodomain PHD finger transcr	-1.305624101	-1.149402003	16.469577	12.621364	13.469228	7.45534827	7.073112	7.25568891	6851	7011	7331
10574	NM_001009570,NM_001166284	CCT7	chaperonin containing TCP1 sub	1.353581983	-1.149564575	203.882522	276.405426	166.937949	8.58461368	9.02041665	8.38408651	15033	27188	16084
4055	NM_001270987,NM_002342	LTBR	lymphotoxin beta receptor	-1.117749731	-1.149825553	141.488361	123.506645	109.965284	8.2185185	8.05848995	8.01782692	11655	13932	12467
10818	NM_001042555,NM_001278351	FRS2	fibroblast growth factor receptor s	1.02846693	-1.14985271	4.548938	4.733772	3.737915	4.97092545	5.01015048	4.7763339	1192	1638	1275
101928725	NR_135474	LOC101928725	uncharacterized LOC101928725	-1.334473231	-1.149917788	0.53236	0.402829	0.434651	0.79797761	0.63490936	0.71544405	29	29	31
26984	NM_012430	SEC22A	SEC22 homolog A, vesicle traffic	1.395504813	-1.14997293	6.273941	8.767938	5.129011	4.45195674	4.91399145	4.26018095	820	1529	877
41	NM_001095,NM_001256830,NM	ASIC1	acid sensing ion channel subunit	-1.133037533	-1.150099767	2.274834	2.000881	1.888402	3.29469475	3.13387411	3.11477407	346	408	370
4277	NM_001289160,NM_001289161	MICB	MHC class I polypeptide-related s	-2.588004804	-1.150229391	1.648574	0.638378	1.400545	2.33625658	1.3579479	2.1765086	159	82	170
106707243	NM_001316893	PRH1-TAS2R14	PRH1-TAS2R14 readthrough	-1.335295767	-1.150250468	0.879953	0.65235	0.722213	1.30878272	1.07428646	1.19154816	58	58	62
54958	NM_017854	TMEM160	transmembrane protein 160	-1.400600187	-1.150310604	19.016998	13.604095	15.561419	3.71696001	3.27409343	3.53128747	477	455	510
112479	NM_001142725,NM_080663	ERI2	ERI1 exoribonuclease family mer	1.088932817	-1.150364385	8.386028	9.24876	7.09571	4.85762016	4.97647655	4.66297087	1099	1599	1175
9696	NM_014675	CROCC	ciliary rootlet coiled-coil, rootletin	1.162918741	-1.150507684	4.782584	5.566782	3.906124	4.99992509	5.21136241	4.80440514	1217	1891	1301
283459	NM_176818,NR_033684	GATC	glutamyl-tRNA amidotransferase	-1.156315734	-1.150640677	10.061838	8.701149	8.189313	5.41861558	5.2143287	5.22123584	1640	1895	1753
51119	NM_016038	SBDS	SBDS, ribosome maturation facto	-2.049464201	-1.150645	51.01918	24.926998	41.676708	6.43897844	5.4210308	6.23903131	3367	2195	3599
283267	NR_015451	LINC00294	long intergenic non-protein codin	-1.537798165	-1.15077612	3.797717	2.472656	3.098855	3.72531928	3.16180073	3.53901915	480	417	513
2037	NM_001135554,NM_001135555	EPB41L2	erythrocyte membrane protein ba	-1.336510546	-1.150869589	19.83336	15.200651	16.545098	6.41850381	6.00568164	6.21831452	3319	3318	3547
10904	NM_001167820,NM_001167821	BLCAP	bladder cancer associated protei	1.102391431	-1.15087227	39.565991	44.161119	32.557426	6.34807711	6.48707234	6.14801122	3159	4653	3376
26953	NM_001243202,NM_001243203	RANBP6	RAN binding protein 6	-1.216756794	-1.151315787	6.352384	4.840591	4.803422	4.7808325	4.50909186	4.58544575	1040	1142	1111
222194	NM_198467	RSBN1L	round spermatid basic protein 1 l	-1.718469432	-1.151336771	8.212865	4.782332	6.697992	4.74153657	3.99854533	4.54634208	1011	786	1080
80152	NM_025082	CENPT	centromere protein T	1.199654884	-1.151346051	32.357876	38.865082	26.407681	6.22673363	6.48615224	6.02631635	2901	4650	3099
285074	NR_026846	LOC285074	anaphase promoting complex sul	-1.021652024	-1.151505299	8.373224	8.204312	6.837602	4.24466875	4.21540721	4.05259921	705	922	753
283693	NR_036446	ACTG1P17	actin gamma 1 pseudogene 17	-1.010637156	-1.151512544	0.921161	0.914575	0.760228	1.32354034	1.31437719	1.20457043	59	78	63
64061	NM_022117	TSPYL2	TSPY like 2	1.246172573	-1.151535076	15.355557	19.15167	12.52769	5.43569657	5.74661881	5.23716605	1660	2764	1773
54532	NM_019050	USP53	ubiquitin specific peptidase 53	-1.223127757	-1.151580583	7.169148	5.868377	5.850161	5.55617688	5.27240856	5.35718856	1808	1975	1931
1059	NM_001810	CENPB	centromere protein B	1.409533692	-1.151731397	41.922035	59.155361	34.200043	6.86936574	7.36100392	6.66742714	4551	8571	4860
23523	NM_001199281,NM_001201429	CABIN1	calcineurin binding protein 1	1.395686201	-1.151753026	18.670797	26.026191	14.890981	7.06839023	7.54632246	6.86618449	5230	9753	5585
5207	NM_001271804,NM_001271805	PFKFB1	6-phosphofructo-2-kinase/fructos	1.988770978	-1.151756488	0.220523	0.445454	0.181967	0.46682716	0.81765451	0.41288872	15	40	16
9398	NM_001256106,NM_001256109	CD101	CD101 molecule	-1.054423872	-1.151756488	0.110065	0.102563	0.086968	0.46682716	0.44594451	0.41288872	15	19	16
11158	NM_001003789,NM_001130919	RABL2B	RAB, member of RAS oncogene	1.381650333	-1.151841736	6.314454	8.499122	4.827436	3.89793981	4.33742297	3.70857641	546	1008	583
2889	NM_001304275,NM_005312,NM	RAPGEF1	Rap guanine nucleotide exchang	-1.15394169	-1.151901961	8.005478	6.945697	6.529858	5.60757673	5.40554179	5.4080336	1875	2171	2002
23013	NM_015001	SPEN	spen family transcriptional repres	-1.072341874	-1.151984548	9.454693	8.826368	7.712592	6.82727062	6.72742198	6.62507336	4419	5506	4718
54891	NM_017759	INO80D	INO80 complex subunit D	1.242530762	-1.152309471	3.049748	3.793589	2.485965	5.42547226	5.73220439	5.2260334	1648	2736	1759
222	NM_000695,NM_001031615	ALDH3B2	aldehyde dehydrogenase 3 famil	1.1002303	-1.152379843	1.320526	1.456811	1.074722	2.14180175	2.24959721	1.98602166	134	197	143
150356	NM_138481	CHADL	chondroadherin like	-2.217201616	-1.152407607	0.306051	0.134104	0.252067	0.81895953	0.42561204	0.73352601	30	18	32
11174	NM_197941,NR_135689	ADAMTS6	ADAM metallopeptidase with thro	-1.012989257	-1.152583441	2.768744	2.792436	2.29844	4.40068652	4.38295215	4.20617012	790	1042	843
109617002	NR_145761	SNORA21B	small nucleolar RNA, H/ACA box	-1.365301669	-1.152625889	8.636003	6.311046	6.969139	1.10178443	0.8787583	0.99569924	45	44	48
64795	NM_022780	RMND5A	required for meiotic nuclear divisi	1.026055774	-1.152854457	9.111599	9.358718	7.426746	5.80386677	5.84032177	5.60258718	2154	2953	2298
122769	NM_152329,NM_203467,NR_03	LRR1	leucine rich repeat protein 1	-1.034569184	-1.152956014	27.666667	25.678894	23.569164	5.30516019	5.25738748	5.10537729	1513	1954	1614
29085	NM_001135861,NM_001287342	PHPT1	phosphohistidine phosphatase 1	1.051156346	-1.152970029	50.450193	53.595706	40.659519	6.0163339	6.08722901	5.8143746	2502	3514	2669
100616387	NR_039891	MIR3064	microRNA 3064	-1.12098694	-1.153145502	607.211609	542.235046	494.800995	5.32271189	5.1622853	5.12263165	1532	1826	1634
5501	NM_001244974,NM_002710	PPP1CC	protein phosphatase 1 catalytic s	-1.102428878	-1.153174888	101.585347	92.501714	82.271779	7.93971223	7.79962676	7.73499812	9600	11635	10239
124923	NM_001174103	SGK494	uncharacterized serine/threonine	-1.020006378	-1.153220353	5.517234	5.41435	4.493549	4.41281357	4.38558611	4.21745651	797	1044	850
10113	NM_001330484,NM_001330485	PREB	prolactin regulatory element bind	1.061078864	-1.153220986	21.08342	22.555312	16.987791	5.48082991	5.56450605	5.28008828	1714	2430	1828
23649	NM_002689	POLA2	DNA polymerase alpha 2, access	1.109140453	-1.153277692	16.264771	18.061682	13.254001	5.87946948	6.02650467	5.67747023	2272	3367	2423

3267	NM_001135187,NM_001135188	AGFG1	ArfGAP with FG repeats 1	1.163229627	-1.153384629	9.325768	10.861294	7.600134	6.31615031	6.53175021	6.11304322	3089	4801	3294
10607	NM_006453	TBL3	transducin beta like 3	1.078364501	-1.153584449	35.955997	38.818382	29.296021	6.52089319	6.62859908	6.31717356	3566	5138	3802
54625	NM_017554	PARP14	poly(ADP-ribose) polymerase fan	-1.036428796	-1.15359969	18.15616	17.537605	14.791061	7.10087705	7.04963781	6.89634317	5350	6897	5704
30851	NM_001204698,NM_014604	TAX1BP3	Tax1 binding protein 3	-1.080887651	-1.153631569	42.674085	39.706128	34.722897	5.86756061	5.7573359	5.66515841	2253	2785	2402
220002	NM_001161452,NM_001161454	CYB561A3	cytochrome b561 family member	-1.011537413	-1.153666897	11.070743	11.240614	9.510827	4.78616959	4.7702214	4.58793254	1044	1379	1113
8723	NM_003794,NR_073435	SNX4	sorting nexin 4	1.426725023	-1.153754194	11.58189	16.554518	9.4366	4.88523815	5.38331039	4.6863673	1121	2137	1195
126626	NM_001323906,NM_001323907	GABPB2	GA binding protein transcription f	1.359985531	-1.154160308	6.996791	9.036055	5.849794	4.11282598	4.53423581	3.91874377	640	1163	682
29995	NM_001278233,NM_001278234	LMCD1	LIM and cysteine rich domains 1	-1.030508302	-1.154239375	4.242723	4.115676	3.471988	3.06808971	3.02995795	2.88736461	290	376	309
7879	NM_004637	RAB7A	RAB7A, member RAS oncogene	1.436361205	-1.154342377	132.80661	190.970673	108.110909	8.17798317	8.69889175	7.97167843	11331	21746	12073
58473	NM_001130033,NM_001130034	PLEKHB1	pleckstrin homology domain cont	1.079491099	-1.154350718	4.071037	4.465881	3.333203	3.19790087	3.29665996	3.01481644	321	463	342
3954	NM_012318	LETM1	leucine zipper and EF-hand cont	1.062527338	-1.154415247	21.669689	23.051983	17.63949	6.86117084	6.94794234	6.65591807	4525	6424	4821
146909	NM_001264573,NM_001265577	KIF18B	kinesin family member 18B	-1.056362985	-1.154497304	53.032138	50.240018	43.234975	7.78952498	7.7107858	7.58326438	8647	10937	9212
10838	NM_001080485	ZNF275	zinc finger protein 275	1.085398182	-1.154562054	2.537296	2.756062	2.062259	4.05430925	4.16570385	3.86028525	613	889	653
8420	NR_002909,NR_036473	SNHG3	small nucleolar RNA host gene 3	1.029976182	-1.15464163	37.260489	38.334846	29.997767	6.36199349	6.40409529	6.15725044	3190	4390	3398
8857	NM_003890	FCGBP	Fc fragment of IgG binding protei	1.711699463	-1.154747155	0.049126	0.084367	0.039949	0.83964067	1.23481109	0.75138414	31	71	33
169714	NM_181701	QSOX2	quiescin sulfhydryl oxidase 2	1.266547102	-1.154988211	24.22121	30.713547	19.709597	6.75187721	7.08996573	6.54606638	4192	7094	4464
64895	NM_022894	PAPOLG	poly(A) polymerase gamma	1.075208129	-1.155079103	3.77031	4.060042	3.068149	4.81386993	4.91490342	4.61378826	1065	1530	1134
9644	NM_014631	SH3PXD2A	SH3 and PX domains 2A	-1.465081413	-1.155094937	3.697776	2.525444	3.007679	5.37500681	4.8400608	5.17236179	1590	1450	1693
6392	NM_001276503,NM_001276504	SDHD	succinate dehydrogenase comple	-1.70228761	-1.155132257	69.07814	40.597544	56.389904	6.57006607	5.81318676	6.36435492	3691	2897	3930
6139	NM_000985,NM_001035006,NM	RPL17	ribosomal protein L17	-1.091227624	-1.155213872	612.163899	556.011134	486.990374	8.8909851	8.76530948	8.68329539	18599	22773	19802
118424	NM_058167,NM_194315,NM_19	UBE2J2	ubiquitin conjugating enzyme E2	1.015731653	-1.155215105	24.028928	24.399997	19.604396	5.74889418	5.77099905	5.5448789	2072	2812	2206
80013	NM_001318330,NM_024948	MINDY3	MINDY lysine 48 deubiquitinase :	1.027481192	-1.155221202	10.132972	10.439321	8.301902	4.62276571	4.66031492	4.42363166	928	1274	988
10300	NM_005886	KATNB1	katanin regulatory subunit B1	-1.179048669	-1.155302806	9.789407	8.31884	7.966705	4.71379852	4.4859587	4.51401614	991	1123	1055
86	NM_004301,NM_177989,NM_17	ACTL6A	actin like 6A	1.115046386	-1.155366413	81.500887	90.61719	66.381651	7.23059	7.38670496	7.02372698	5857	8726	6235
2063	NM_005234	NR2F6	nuclear receptor subfamily 2 grou	-1.284724708	-1.155441623	68.144753	53.107334	55.426773	6.91545167	6.557382	6.70885854	4700	4888	5003
220359	NM_145719	TIGD3	tigger transposable element deriv	-1.318632711	-1.15558433	1.033799	0.781596	0.838825	1.57857476	1.32539385	1.4430085	78	79	83
130916	NM_001330179,NM_001330180	MTERF4	mitochondrial transcription termir	1.294032978	-1.155727204	17.608892	24.471232	15.716928	5.40132993	5.76544819	5.19782003	1620	2801	1724
8829	NM_001024628,NM_001024629	NRP1	neuropilin 1	-1.485377598	-1.155861945	26.39094	17.312354	22.189096	7.13865882	6.57277645	6.93128004	5493	4941	5845
114984	NM_001142499,NM_001142500	FLYWCH2	FLYWCH family member 2	1.245621695	-1.156061973	21.894799	27.379898	17.838003	4.51920371	4.82364393	4.3197426	861	1433	916
348180	NM_001012759,NM_001012762	CTU2	cytosolic thiouridylase subunit 2	1.03368883	-1.156096587	15.254522	15.823896	12.297191	4.75112025	4.79717993	4.55017297	1018	1406	1083
144481	NR_038263	SOCS2-AS1	SOCS2 antisense RNA 1	-2.642881282	-1.15631824	0.697195	0.267795	0.586684	0.49317359	0.20477631	0.43515728	16	8	17
6079	NR_000005	SNORD15A	small nucleolar RNA, C/D box 15	-1.333145184	-1.15631824	2.674233	2.112537	2.250485	0.49317359	0.38406669	0.43515728	16	16	17
101929467	NR_125775	LURAP1L-AS1	LURAP1L antisense RNA 1	1.354168604	-1.15631824	0.421872	0.58919	0.341929	0.49317359	0.63490936	0.43515728	16	29	17
57456	NM_001320334,NM_020696	KIAA1143	KIAA1143	-1.507375052	-1.156359201	4.850705	3.225845	3.960853	4.66827758	4.1046668	4.46750731	959	850	1020
11269	NM_001014449,NM_001014451	DDX19B	DEAD-box helicase 19B	-1.105593787	-1.156460557	28.198745	25.513987	22.958604	5.66447154	5.5226424	5.45918641	1952	2359	2076
143666	NR_026967	LOC143666	uncharacterized LOC143666	1.931404472	-1.15646801	0.986853	1.918464	0.807329	1.59082596	2.2895231	1.45395569	79	204	84
147841	NM_001317031,NM_001317032	SPC24	SPC24, NDC80 kinetochore com	-1.281281388	-1.156607706	31.886233	27.400503	26.246928	6.02876973	5.67736765	5.82231668	2524	2632	2684
10206	NM_001007278,NM_005798,NM	TRIM13	tripartite motif containing 13	-1.402716508	-1.156630709	3.539898	2.505164	2.87731	4.65082999	4.18548887	4.44984345	947	902	1007
91368	NM_080656	CDKN2AIPNL	CDKN2A interacting protein N-te	-1.124239803	-1.156715845	15.161699	13.496522	12.315316	4.28669603	4.12687582	4.08816658	727	864	773
4904	NM_004559,NR_132737	YBX1	Y-box binding protein 1	1.344957224	-1.156792811	232.480335	313.76586	188.975324	9.48391736	9.9109622	9.27410183	28073	50448	29848
7769	NM_001032372,NM_001032373	ZNF226	zinc finger protein 226	-1.469862171	-1.156824839	21.434121	14.654054	20.703389	5.48985314	4.94913788	5.28469437	1725	1568	1834
7003	NM_021961	TEAD1	TEA domain transcription factor 1	-1.155188228	-1.157039121	22.225912	19.26145	18.051853	7.67785255	7.47081459	7.46851774	8000	9253	8504
55695	NM_001168347,NM_001168348	NSUN5	NOP2/Sun RNA methyltransferas	-1.406213048	-1.157068619	13.378708	9.534957	10.766273	5.00679911	4.53304836	4.80333552	1223	1162	1300
1212	NM_001834,NM_007097,NR_04	CLTB	clathrin light chain B	-1.287099805	-1.15718914	62.978737	49.623297	59.483021	6.41420145	6.05491286	6.2062258	3309	3435	3517
100131205	NR_026911	RPL21P28	ribosomal protein L21 pseudoger	-1.04474155	-1.157190846	515.387878	493.862183	418.540466	8.14474035	8.0818217	7.93491232	11072	14160	11768
11232	NM_007215	POLG2	DNA polymerase gamma 2, acce	-1.027369516	-1.157208401	15.67346	15.263483	12.727144	4.66248513	4.62508404	4.46073909	955	1242	1015
653519	NM_001097612,NM_001097613	GPR89A	G protein-coupled receptor 89A	-1.028542459	-1.157271611	15.195383	14.823085	12.364077	4.91721027	4.87796738	4.71395121	1147	1490	1219
79000	NM_001287490,NM_024037	AUNIP	aurora kinase A and ninein intera	1.167083619	-1.157327784	7.738837	8.861306	6.022702	4.03874269	4.24906335	3.84165053	606	945	644
9814	NM_001007467,NM_001258325	SFI1	SFI1 centrin binding protein	1.021151483	-1.157400935	7.406861	7.546964	5.991854	4.99532414	5.02458641	4.79151702	1213	1655	1289
10890	NM_016131	RAB10	RAB10, member RAS oncogene	1.030800232	-1.157830453	37.955456	39.167446	30.80512	7.13839801	7.18185761	6.92858469	5492	7564	5834
10643	NM_006547	IGF2BP3	insulin like growth factor 2 mRNA	-1.318270467	-1.158015952	31.600908	23.996758	25.642061	7.01085643	6.61575632	6.80096283	5024	5092	5336
84960	NM_001039374	CCDC183	coiled-coil domain containing 183	-1.270247327	-1.158076954	1.477559	1.16196	1.189545	1.79522562	1.55800914	1.64757178	97	102	103
677792	NR_003026	SNORA1	small nucleolar RNA, H/ACA box	1.099523058	-1.158557039	9.783081	10.796565	7.905488	1.16869613	1.24644988	1.05442063	49	72	52
728642	NM_001313896,NM_001313982	CDK11A	cyclin dependent kinase 11A	-1.146735547	-1.15856134	34.609324	30.279846	28.050943	6.74504182	6.54947593	6.53483235	4172	4861	4429
29915	NM_013320	HCFC2	host cell factor C2	-1.108864192	-1.158661283	2.222219	2.008909	1.803252	3.74737673	3.60990992	3.5518139	488	588	518
7520	NM_021141	XRCC5	X-ray repair cross complementing	1.230642874	-1.158771089	148.16217	182.537766	120.1539	8.95361953	9.25248773	8.74148454	19426	31942	20619
7024	NM_001173452,NM_001173453	TFCP2	transcription factor CP2	-1.023861261	-1.158816023	11.598507	11.332996	9.414763	5.42376114	5.39054046	5.21642227	1646	2148	1747
3015	NM_002106	H2AFZ	H2A histone family member Z	1.023634139	-1.158940453	530.774353	543.937866	430.390289	8.93782067	8.97145309	8.72548039	19214	26279	20391
2086	NM_001007253,NR_145414,NR	ERV3-1	endogenous retrovirus group 3 m	1.055910169	-1.158980338	5.830367	5.830992	5.907902	3.96064828	4.03423402	3.76240724	572	807	607
170960	NM_133474	ZNF721	zinc finger protein 721	-1.124641816	-1.159008694	16.219276	14.436376	13.14778	6.23065365	6.06357411	6.02080512	2909	3456	3087
4359	NM_000530,NM_001315491	MPZ	myelin protein zero	2.083032382	-1.159026759	0.432947	0.908233	0.352899	0.88013425	1.4614436	0.78645063	33	92	35
84679	NM_001257291,NM_032591	SLC9A7	solute carrier family 9 member A7	-1.011463316	-1.159125748	11.406166	11.287767	9.246146	6.81165358	6.79535639	6.60065285	4371	5774	4638
2968	NM_001517	GTF2H4	general transcription factor IIH su	1.090977627	-1.159165227	10.983299	11.999879	8.900127	4.27535493	4.39476729	4.07404468	721	1051	765
100506365	NR_110438,NR_110439	OTUD6B-AS1	OTUD6B antisense RNA 1 (head	-1.120391554	-1.159307556	11.189994	10.214994	9.056163	4.75112025	4.59353508	4.54634208	1018	1214	1080
100128398	NR_036508	LOC100128398	uncharacterized LOC100128398	1.439456765	-1.159339401	0.903139	1.30983	0.729228	1.42282133	1.77444288	1.29257653	66	127	70
80196	NM_001256858,NM_025126,NM	RNF34	ring finger protein 34	-1.306271865	-1.159355759	15.245583	11.828661	12.650493	4.96034122	4.58897123	4.75436273	1183	1210	1255
22994	NM_001009811,NM_001319228	CEP131	centrosomal protein 131	1.076765941	-1.159496441	19.12313	20.657548	15.527332	6.10709035	6.21230621	5.89691676	2667	3837	2829
6161	NM_000994,NM_001007073,NM	RPL32	ribosomal protein L32	-1.001717649	-1.159526272	180.053131	179.405727	145.780551	8.18418709	8.18171967	7.97144037	11380	15179	12071
57697	NM_001308133,NM_001308134	FANCM	Fanconi anemia complementatio	1.05390109	-1.159530864	4.612643	5.04829	3.737806	5.03956952	5.11306995	4.83298924	1252	1763	1328
81858	NM_030974,NR_038270	SHARPIN	SHANK associated RH domain ir	-1.160976327	-1.15993183	23.443073	20.226282	18.809429	5.37589203	5.16612031	5.16738207	1591	1831	1687
11099	NM_007039	PTPN21	protein tyrosine phosphatase, no	-1.123975721	-1.159935205	2.16066	1.925609	1.751254	3.81681978	3.66081122	3.61901061	514	611	545
79003	NM_001258217,NM_001258218	MIS12	MIS12, kinetochore complex com	-1.594395561	-1.160317996	6.420941	4.202004	4.972436	4.06312959	3.44039495	3.86234099	617	517	654
6468	NM_001323541,NM_022039,NR	FBXW4	F-box and WD repeat domain co	-1.6060619	-1.160357546	19.352483	12.359195	15.54132	5.39958992	4.73657294	5.19047509	1618	1346	1715
3299	NM_001040667,NM_001538	HSF4	heat shock transcription factor 4	-1.441691993	-1.160452944	9.898089	6.861398	7.999192	4.68265777	4.17943002	4.4769298	969	898	1027
2256	NM_001303460,NM_004112,NR	FGF11	fibroblast growth factor 11	1.803420514	-1.160487322	1.20795	2.302986	0.927264	2.14180175	2.83945493	1.97845985	134	323	142
56260	NM_019607	C8orf44	chromosome 8 open reading fran	1.469832287	-1.160494841	3.482429	5.124015	2.818877	2.88620833	3.37827344	2.70236404	251	493	266
2071	NM_000122,NM_001303416,NM	ERCC3	ERCC excision repair 3, TFIIH cc	1.10082004	-1.160500014	31.377038	34.939757	25.259109	6.42877745	6.56582572	6.21670854	3343	4917	3543
378708	NM_199294,NR_036462	CENPS	centromere protein S	-1.068880455	-1.160560777	9.032879	8.172841	7.342015	3.64534899	3.55714683	3.44886901	452	565	479
51204	NM_016360	TACO1	translational activator of cytochro	1.043521245	-1.160764637	30.478788	31.842514	24.671534	5.47918324	5.53929737	5.26928318	1712	2387	1814
30827	NM_001101654,NM_014593	CXXC1	CXXC finger protein 1	1.14817775	-1.160792459	14.263457	16.399052	11.550853	5.39435726	5.58928181	5.1847364	1612	2473	1708
25875	NM_001243689,NM_001300765	LETMD1	LETM1 domain containing 1	-1.339021107	-1.16081925	21.526203	16.197014	17.442842	5.57402879	5.16305312	5.36373375	1831	1827	1940
10635	NM_001130862,NM_006479	RAD51AP1	RAD51 associated protein 1	-1.345189242	-1.160898878	26.142955	19.44526	21.111712	5.84664023	5.4274358	5.63541459	2220	2205	2352
54463	NM_001034850,NM_019000	RETREG1	reticulophagy regulator 1	-1.887552228	-1.160988925	0.270301	0.142006	0.219523	0.89996265	0.54351856	0.80366934	34	24	36
79697	NM_024644	RIOX1	ribosomal oxygenase 1	1.154586831	-1.161132384	7.697179	8.894745	6.228469	4.28481203	4.48227194	4.08112291	726	1120	769
114041	NR_026542,NR_026543	B3GALT5-AS1	B3GALT5 antisense RNA 1	-1.513049366	-1.16129665	1.269103	0.859166	1.013879	1.44997615	1.10017522	1.31676564	68	60	72
64410	NM_022480	KLHL25	kelch like family member 25	1.01991841	-1.161536806	2.171063	2.220137	1.756294	3.13654562	3.16180073	2.94669635	306	417	324
11129	NM_001278439,NM_007056,NR	CLASRP	CLK4 associating serine/arginine	-1.045709005	-1.161740137	43.085883	41.233887	34.870299	6.56580548	6.50201808	6.3519721	3680	4702	3896
8607	NM_001319084,NM_001319086	RUVBL1	RuvB like AAA ATPase 1	1.814420634	-1.161852546	60.364941	109.775505	48.756953	6.71876791	7.57213254	6.50454979	4096	9930	4336
54801	NM_001270890,NM_017645	HAUS6	HAUS augmin like complex subu	1.236384551	-1.161853576	8.204625	10.157858	6.636596	5.69671172	5.99752584	5.48476573	1997	3299	2114
55661	NM_001348187,NM_017895	DDX27	DEAD-box helicase 27	1.163046818	-1.161931654	51.14878	59.439455	41.304775	7.08201541	7.29843513	6.86720853	5280	8205	5589
8540	NM_003659	AGPS	alkylglycerone phosphate syntha	1.362202522	-1.161954047	9.274375	12.64983	7.50127	6.13402522	6.57450891	5.9207858	2718	4947	2877
84218	NM_032258,NM_032258_dup1,N	TBC1D3F	TBC1 domain family member 3F	-1.285953883	-1.161990496	7.743622	6.029226	6.266932	4.06092955	3.7225328	3.85822657	616	640	652
8771	NM_003823	TNFRSF6B	TNF receptor superfamily membe	1.037107759	-1.162142631	67.170868	69.74044	54.310226	6.2582797	6.31017284	6.04453654	2966	4110	3139
29789	NM_001011708,NM_001328688	OLA1	Obg like ATPase 1	1.41857448	-1.162463735	18.814736	26.861218	15.280673	6.33766462	6.83684787	6.12336578	3136	5944	3318
4796	NM_013432	TONSL	tonsoku like, DNA repair protein	1.109102413	-1.16270441	23.045532	25.590681	18.627134	6.67700171	6.82501047	6.46180299	3978	5895	4208
162239	NM_001318469,NM_001318471	ZFP1	ZFP1 zinc finger protein	-1.453057544	-1.162822399	6.431745	4.477702	5.229202	4.47196531	3.96195135	4.26485094	832	765	880
100302240	NR_031639	MIR1304	microRNA 1304	-1.271795514	-1.162847284	39.806923	31.278139	32.262638	2.18284609	1.9196316	2.01587867	139	146	147
56731	NM_020062	SLC2A4RG	SLC2A4 regulator	-1.044354299	-1.16303127	79.416367	76.131363	64.166664	7.46951643	7.40726522	7.25294923	6919	8852	7317
79607	NM_001330446,NM_024556	FAM118B	family with sequence similarity 11	-1.340989683	-1.163238609	6.758381	5.048422	5.460104	3.88556162	3.49508138	3.68321628	541	539	572

159091	NM_001170779,NM_001170780	FAM122C	family with sequence similarity 12	-1.947101133	-1.163300159	1.832817	0.958727	1.585073	2.19091668	1.50084877	2.02324736	140	96	148
79736	NM_024683	TEFM	transcription elongation factor, m	1.264465552	-1.163366109	11.956144	15.125273	9.655007	4.05209571	4.37236795	3.84788889	612	1034	647
10658	NM_001025596,NM_001172639	CELF1	CUGBP Elav-like family member	-1.06746943	-1.163388409	28.162702	28.49395	24.214589	7.43000109	7.33636914	7.21303062	6731	8425	7116
57215	NM_020457	THAP11	THAP domain containing 11	1.242768368	-1.163592582	16.538162	20.580742	13.354611	4.9720967	5.27667173	4.76098929	1193	1981	1261
55186	NM_001104647,NM_018155	SLC25A36	solute carrier family 25 member 3	1.152383353	-1.163596145	34.753741	40.091469	28.064775	7.31031377	7.51373531	7.09320538	6192	9534	6545
11017	NM_006857,NR_037862	SNRNP27	small nuclear ribonucleoprotein U	1.1020885	-1.16368795	27.815867	30.648271	22.314724	5.30330016	5.44016111	5.09054707	1511	2225	1597
3672	NM_181501	ITGA1	integrin subunit alpha 1	-1.82848565	-1.163822695	1.536575	0.838922	1.239516	3.03260384	2.30073045	2.84224948	282	206	298
9129	NM_001350529,NM_004698,NR	PRPF3	pre-mRNA processing factor 3	1.047505996	-1.163826382	41.306718	43.321282	33.577606	6.67736079	6.74368163	6.46078576	3979	5569	4205
100616403	NR_039840	MIR4691	microRNA 4691	1.121926784	-1.164011571	5.274918	6.109951	4.386475	0.54446555	0.59904473	0.47868902	18	27	19
115811	NM_001330452,NM_138451	IQCD	IQ motif containing D	-1.157872384	-1.16414926	3.079562	2.695823	2.448275	2.57697339	2.40307061	2.39673966	195	225	206
84334	NM_001302652,NM_001302653	APOPT1	apoptogenic 1, mitochondrial	-1.07911091	-1.16431128	15.040746	13.778219	12.692521	4.38494329	4.28053774	4.17673594	781	967	825
1948	NM_004093	EFNB2	ephrin B2	1.551734733	-1.164465689	13.574503	21.089987	10.956877	5.88258712	6.50777196	5.66692369	2277	4721	2405
6508	NM_001326559,NM_005070,NM	SLC4A3	solute carrier family 4 member 3	-1.050172471	-1.164559525	3.747582	3.532216	2.995434	4.03874269	3.97250183	3.83329054	606	771	640
51222	NM_001101672,NM_001102454	ZNF219	zinc finger protein 219	1.297855989	-1.164577651	7.097568	9.271033	5.871351	4.45195674	4.81292083	4.24292715	820	1422	866
729966	NR_036575	LOC729966	uncharacterized LOC729966	-2.539019101	-1.164580791	8.811667	3.466661	7.118625	2.78853894	1.73367602	2.60259472	232	122	245
124565	NM_001037984,NM_138570	SLC38A10	solute carrier family 38 member 1	1.10173157	-1.164590495	60.370648	68.534176	49.616227	7.94866452	8.08789943	7.72979986	9660	14220	10202
79092	NM_001257970,NM_024110,NM	CARD14	caspase recruitment domain fam	-1.264803734	-1.164604561	0.223404	0.175532	0.177841	0.93882015	0.7861039	0.83750224	36	38	38
8799	NM_001184795,NM_003846,NR	PEX11B	peroxisomal biogenesis factor 11	-1.138761396	-1.16483313	14.851021	13.270786	12.148848	4.80206342	4.62173661	4.59041505	1056	1239	1115
79706	NM_024653	PRKRIP1	PRKR interacting protein 1 (IL11	-1.558959223	-1.164961924	13.331774	8.55762	10.759235	4.87400371	4.26058819	4.66179103	1112	953	1174
221078	NM_001351115,NM_001351116	NSUN6	NOP2/Sun RNA methyltransferas	-1.122729875	-1.165388327	6.38504	5.721516	5.198818	3.88804577	3.7329126	3.68321628	542	645	572
254863	NM_152766	TMEM256	transmembrane protein 256	1.010315556	-1.165500947	29.06082	29.425993	23.431301	3.89052565	3.90434076	3.68554025	543	733	573
388753	NM_001012985,NM_001206641	COA6	cytochrome c oxidase assembly f	-1.356969732	-1.165504112	61.924101	45.908489	50.475565	5.51417891	5.08498478	5.29842508	1755	1728	1852
1030	NM_004936,NM_078487	CDKN2B	cyclin dependent kinase inhibitor	1.795326336	-1.165810693	7.25191	13.057945	5.805155	4.8242841	5.64585531	4.61134568	1073	2574	1132
55347	NM_001272069,NM_018394,NR	ABHD10	abhydrolase domain containing 1	1.929889634	-1.16587779	15.44805	30.898809	12.304683	5.38559376	6.31743308	5.16987408	1602	4131	1690
4713	NM_004146	NDUFB7	NADH:ubiquinone oxidoreductas	-1.133577443	-1.165901363	120.382149	106.319565	97.019539	6.02256521	5.84463554	5.80478602	2513	2962	2651
128240	NM_144772	NAXE	NAD(P)HX epimerase	1.066766654	-1.16624265	62.808578	67.091858	50.61248	6.12035656	6.21230621	5.90192215	2692	3837	2839
79968	NM_001167941,NM_024908	WDR76	WD repeat domain 76	1.000821879	-1.166433609	6.67791	6.701665	5.425612	4.76605286	4.76719467	4.55272125	1029	1376	1085
1513	NM_000396	CTSK	cathepsin K	-1.253045622	-1.166655214	11.995462	9.58218	9.652885	4.46864976	4.15956116	4.2570592	830	885	875
100505633	NR_038849	LINC01133	long intergenic non-protein codin	-1.473554383	-1.166940534	11.076286	7.511641	8.907577	3.73086534	3.22198991	3.5261099	482	437	508
8425	NM_001042544,NM_001042545	LTBP4	latent transforming growth factor	-5.396138377	-1.167184866	7.189689	1.340172	6.003722	5.21008282	2.93964205	4.99353253	1414	350	1490
6732	NM_003137,NR_034069	SRPK1	SRSF protein kinase 1	-1.206942455	-1.167456742	31.071575	25.83939	25.064504	7.05877545	6.78965007	6.83721211	5195	5751	5473
55633	NM_017772,NR_130108	TBC1D22B	TBC1 domain family member 22B	-1.227540241	-1.167604111	4.797022	3.959689	3.907784	4.14641466	3.86901329	3.93641476	656	714	691
56122	NM_018934	PCDHB14	protocadherin beta 14	1.359289388	-1.16776822	1.36453	1.857119	1.09806	2.64904502	3.02995795	2.46326383	207	376	218
400073	NM_207435	C12orf76	chromosome 12 open reading fra	-1.058327605	-1.167783556	4.032613	3.817851	3.248966	3.1155	3.04336424	2.91931156	301	380	317
133308	NM_001300754,NM_001300756	SLC9B2	solute carrier family 9 member B2	-1.19342291	-1.167824516	1.886581	1.5891	1.641308	2.75626978	2.541776	2.56774382	226	253	238
3434	NM_001270927,NM_001270928	IFIT1	interferon induced protein with te	-2.732626555	-1.168039408	21.211383	7.771639	17.108791	6.52129329	5.09789159	6.29983366	3567	1744	3756
9852	NM_014805	EPM2AIP1	EPM2A interacting protein 1	1.201378977	-1.168263274	5.129788	6.170864	4.125355	5.25407798	5.51243584	5.03604153	1459	2342	1536
146754	NM_001303270,NM_020877	DNAH2	dynein axonemal heavy chain 2	-1.164135753	-1.168275725	1.491764	1.282371	1.199508	4.50632616	4.29742051	4.29255782	853	979	898
9167	NM_001319036,NM_001319037	COX7A2L	cytochrome c oxidase subunit 7A	1.008160958	-1.168647681	42.009748	42.31942	33.723192	5.5886112	5.60009515	5.36880401	1850	2492	1947
8440	NM_001004720,NM_001004722	NCK2	NCK adaptor protein 2	1.61447834	-1.168660371	11.338813	17.516671	8.774465	4.66103338	5.3302927	4.44573627	954	2058	1004
26037	NM_001284245,NM_001284246	SIPA1L1	signal induced proliferation assoc	-1.055139375	-1.168734799	9.671075	9.185613	7.77879	6.21836805	6.14199953	5.9966768	2884	3652	3035
80772	NM_001029885	CPTP	ceramide-1-phosphate transfer p	1.308706631	-1.168745272	17.319193	22.687138	13.921568	5.24732179	5.6265008	5.02873806	1452	2539	1528
84231	NM_032271	TRAF7	TNF receptor associated factor 7	-1.099692325	-1.168812437	45.294754	41.23431	36.414265	7.34621288	7.20999417	7.12266115	6349	7714	6681
5937	NM_002897,NM_016836	RBMS1	RNA binding motif single strande	-1.0559299	-1.16884242	12.036389	11.416054	9.677447	5.67817204	5.60122869	5.45782748	1971	2494	2074
150786	NM_001077637	WTH3DI	RAB6C-like	-1.534403133	-1.168910205	0.546394	0.357807	0.437066	1.56621863	1.18729361	1.42086173	77	67	81
116988	NM_001042535,NM_001281300	AGAP3	ArfGAP with GTPase domain, an	-1.479337141	-1.169012659	18.679096	12.355119	14.59059	5.89869115	5.34525392	5.67747023	2303	2080	2423
102723927	NR_110592	LINC01238	long intergenic non-protein codin	1.032263848	-1.169064505	0.690017	0.714599	0.557393	1.30878272	1.33632702	1.17840727	58	80	61
114049	NM_001202560,NM_017528,NR	BUD23	BUD23, rRNA methyltransferase	-1.544451548	-1.169395758	80.319741	52.096921	64.591425	6.62098204	6.00182411	6.39769249	3825	3309	4023
64222	NM_022371	TOR3A	torsin family 3 member A	1.009335896	-1.169466785	33.374969	33.725883	26.818506	6.12878335	6.14199953	5.9064122	2708	3652	2848
5037	NM_002567	PEBP1	phosphatidylethanolamine bindin	-1.146244689	-1.169565719	122.841606	107.290276	98.703682	7.65360276	7.4577323	7.42884077	7866	9169	8272
9191	NM_001039711,NM_001039712	DEDD	death effector domain containing	1.090739093	-1.169622066	16.988704	18.463553	13.453192	5.30516019	5.4274358	5.08527627	1513	2205	1591
286076	NR_015445	BREA2	breast cancer estrogen-induced a	-1.426801612	-1.169653164	2.022951	1.408693	1.626611	1.57857476	1.25799553	1.43197761	78	73	82
221504	NM_152735	ZBTB9	zinc finger and BTB domain conta	1.023394642	-1.17011889	4.906236	5.026704	3.94318	3.80372238	3.83472784	3.59448638	509	696	535
388115	NM_207380	C15orf52	chromosome 15 open reading fra	1.21090829	-1.17016012	99.406471	120.507698	79.829834	9.0128652	9.28846963	8.78663303	20242	32750	21276
55704	NM_001135597,NM_001254943	CCDC88A	coiled-coil domain containing 88A	1.366610748	-1.170222029	3.840061	5.249512	3.083489	5.22686128	5.66712026	5.00660315	1431	2613	1504
23332	NM_001142273,NM_001142274	CLASP1	cytoplasmic linker associated pro	-1.101312787	-1.170345982	14.842323	13.489382	11.805743	6.8526112	6.71464766	6.62779498	4498	5457	4727
84777	NR_119377	DLGAP1-AS2	DLGAP1 antisense RNA 2	1.01216436	-1.170411236	8.920983	9.03807	7.14988	3.17367098	3.18919702	2.973571	315	426	331
7711	NM_001260486,NM_001260487	ZNF155	zinc finger protein 155	-1.198442102	-1.170697146	9.358224	7.796688	7.412461	4.65375261	4.40389042	4.43610715	949	1058	997
10058	NM_001349828,NM_005689	ABCB6	ATP binding cassette subfamily B	-1.589077724	-1.17073499	5.608114	3.536656	4.565097	4.14017606	3.51926972	3.92662428	653	549	686
6522	NM_001199692,NM_001199693	SLC4A2	solute carrier family 4 member 2	-1.49823257	-1.170895794	31.646393	21.037818	25.355762	7.00914581	6.43143843	6.7834404	5018	4475	5271
27330	NM_001330512,NM_014496	RPS6KA6	ribosomal protein S6 kinase A6	1.115646919	-1.17090897	1.705804	1.858194	1.334219	3.8730763	4.02074222	3.66213029	536	799	563
683	NM_004334	BST1	bone marrow stromal cell antigen	-19.38465271	-1.17099269	1.043355	0.050486	0.848044	1.33814853	0.10601836	1.20457043	60	4	63
272	NM_000480,NM_001025389,NM	AMPD3	adenosine monophosphate deam	-1.363277169	-1.171121361	8.768792	6.342488	7.013425	5.24925536	4.81585323	5.02782252	1454	1425	1527
8241	NM_001204466,NM_001204467	RBM10	RNA binding motif protein 10	1.140064333	-1.171155974	21.594574	24.605157	17.097562	6.15067232	6.33729741	5.92620061	2750	4189	2888
643699	NM_001282494	GOLGA8N	golgin A8 family member N	-1.107241363	-1.171291963	1.472328	1.329237	1.177992	3.11125378	2.98202989	2.9113909	300	362	315
672	NM_007294,NM_007297,NM_00	BRCA1	BRCA1, DNA repair associated	1.100748353	-1.17129708	15.037626	14.545766	11.253357	6.13350189	6.27010962	5.90890064	2717	3996	2853
25851	NM_015395	TECPR1	tectonin beta-propeller repeat co	-1.6900607	-1.171617515	5.671805	3.359774	4.549326	5.03285024	4.30578837	4.81187039	1246	985	1308
27175	NM_001320509,NM_016437	TUBG2	tubulin gamma 2	-1.047787102	-1.171668177	33.543598	32.047278	26.869215	5.91705205	5.85084386	5.6925693	2333	2975	2449
57465	NM_001199107,NM_020705	TBC1D24	TBC1 domain family member 24	1.310911849	-1.17169699	3.760257	4.936058	3.014666	4.64936645	5.0262753	4.4305758	946	1657	993
102466747	NR_106899	MIR6840	microRNA 6840	-1.76942278	-1.171911688	22.210268	12.685973	17.805752	1.35261029	0.90836632	1.21747621	61	46	64
347344	NM_007137	ZNF81	zinc finger protein 81	1.164122947	-1.17191654	0.537392	0.626144	0.432157	2.3579234	2.53704518	2.1765086	162	252	170
79048	NM_001282688,NM_001282689	SECISBP2	SECIS binding protein 2	-1.224263553	-1.171931241	13.746936	11.225397	10.997774	5.58018673	5.29500126	5.35645948	1839	2007	1930
27345	NM_014505	KCNMB4	potassium calcium-activated char	1.759955935	-1.171986234	1.790901	3.160781	1.43722	3.2098649	3.95664704	3.00740444	324	762	340
100132774	NR_033827	KDM4A-AS1	KDM4A antisense RNA 1	1.22247074	-1.172176876	0.674918	0.840316	0.548603	1.03161781	1.18729361	0.91876801	41	67	43
102157401	NR_109828	PCBP2-OT1	PCBP2 overlapping transcript 1	-1.519631033	-1.172176876	1.793033	1.167436	1.435161	1.03161781	0.75384785	0.91876801	41	36	43
11157	NM_007080	LSM6	LSM6 homolog, U6 small nuclear	-1.068411097	-1.172215783	29.688032	27.84021	23.812681	4.57114104	4.47980886	4.3522875	894	1118	938
58477	NM_021203	SRPRB	SRP receptor beta subunit	1.961016617	-1.172378701	29.402847	57.734425	23.567402	6.35662242	7.31959433	6.13020668	3178	8327	3334
1861	NM_000113	TOR1A	torsin family 1 member A	1.35732488	-1.172456774	29.640862	40.286869	23.759521	5.94952407	6.38414818	5.72399823	2387	4329	2504
26528	NM_001352033,NM_001352034	DAZAP1	DAZ associated protein 1	1.169308528	-1.17252802	80.151564	94.014447	65.144794	7.43637976	7.66083217	7.20818955	6761	10563	7092
28958	NM_001040431	COA3	cytochrome c oxidase assembly f	1.059298347	-1.172777807	39.198635	41.572498	31.400051	4.99301815	5.07359581	4.7708724	1211	1714	1270
29066	NM_014153	ZC3H7A	zinc finger CCCH-type containing	1.068812466	-1.172855633	19.440746	20.801105	15.575136	6.19448232	6.28922568	5.96784829	2836	4050	2974
9675	NM_001303457,NM_014657	TTI1	TELO2 interacting protein 1	-1.04377516	-1.172879581	16.185523	15.626314	12.975631	5.94833435	5.88754299	5.72230146	2385	3053	2501
340591	NR_026551	CA5BP1	carbonic anhydrase 5B pseudoge	-1.189725305	-1.172920251	8.106687	6.837348	6.514891	3.1490272	2.92884759	2.94669635	309	347	324
399744	NR_024497	LINC00999	long intergenic non-protein codin	1.009280253	-1.173253131	1.856924	1.874838	1.484451	3.3924168	3.40448323	3.18544464	373	503	391
84900	NM_001109903,NM_032814	RNFT2	ring finger protein, transmembrar	-1.115349945	-1.173336726	1.684468	1.51238	1.345707	2.52684435	2.3978615	2.33885915	187	224	196
653464	NM_001271872,NM_001329984	SRGAP2C	SLIT-ROBO Rho GTPase activat	1.198816562	-1.173488647	1.908246	2.292681	1.530189	3.60061161	3.84241755	3.39023607	437	700	458
728819	NM_001101330	C1GALT1C1L	C1GALT1-specific chaperone 1 li	2.421088092	-1.173666314	1.399607	3.411083	1.1223	1.38110603	2.2895231	1.24294651	63	204	66
116349	NR_126522	EXOC3-AS1	EXOC3 antisense RNA 1	1.448587257	-1.173666314	1.243113	1.791792	0.9937	1.38110603	1.73367602	1.24294651	63	122	66
92703	NM_001322955,NM_001322956	TMEM183A	transmembrane protein 183A	-1.309231267	-1.173760029	7.44321	5.615057	5.943433	4.54619271	4.17639102	4.32571472	878	896	920
3097	NM_006734	HIVEP2	human immunodeficiency virus ty	1.316362089	-1.174227747	4.027502	5.306973	3.222122	5.29021237	5.67790497	5.06489121	1497	2633	1568
90459	NM_153332	ERI1	exoribonuclease 1	-1.164685742	-1.174228601	6.932207	5.956328	5.546447	5.00679911	4.79420929	4.78286053	1223	1403	1281
1105	NM_001270	CHD1	chromodomain helicase DNA bin	-1.005842965	-1.174296642	24.712322	24.595976	19.77705	7.28885462	7.28050329	7.05866029	6100	8103	6389
283638	NM_001112726,NM_015005	CEP170B	centrosomal protein 170B	1.168845433	-1.174432835	36.454565	42.661511	29.196173	7.89903287	8.12324606	7.66811955	9332	14574	9773
22863	NM_014924	ATG14	autophagy related 14	1.013487864	-1.174465235	10.50931	10.665352	8.409568	5.63075293	5.64969525	5.40380548	1906	2581	1996
388969	NM_001013649	C2orf68	chromosome 2 open reading fran	-1.04090566	-1.174505038	12.089642	11.62728	9.669762	5.67817204	5.62148167	5.45101358	1971	2530	2064
161742	NM_152594	SPRED1	sprouty related EVH1 domain co	1.1927006	-1.174516125	4.967021	5.931125	3.975271	5.17389443	5.42167258	4.94876578	1378	2196	1443
5160	NM_000284,NM_001173454,NM	PDHA1	pyruvate dehydrogenase (lipoam	1.087771264	-1.174605901	23.399267	25.306668	18.729032	6.28161277	6.40149494	6.05266115	3015	4382	3157
9819	NM_001303264,NM_014779,NR	TSC22D2	TSC22 domain family member 2	1.570331882	-1.174606108	10.876132	17.102621	8.751636	5.69600304	6.33695723	5.46866322	1996	4188	2090
4733	NM_004147	DRG1	developmentally regulated GTP b	1.071891576	-1.174613318	25.671276	27.545061	20.527315	5.21898983	5.31655563	4.99353253	1423	2038	1490

8539	NM_001142930,NM_001142931	API5	apoptosis inhibitor 5	-1.169811243	-1.174800708	19.982805	17.104547	16.050678	6.21490924	5.99191887	5.98587457	2877	3286	3012
10527	NM_006391	IPO7	importin 7	-1.658560747	-1.174955834	68.804283	41.528584	55.028873	8.69973552	7.97208065	8.46773417	16285	13119	17047
26259	NM_012174,NM_153348	FBXW8	F-box and WD repeat domain co	1.06976849	-1.175096571	3.948161	4.349368	3.199094	4.29045667	4.38295215	4.0704925	729	1042	763
54998	NM_001127229,NM_001127230	AURKAIP1	aurora kinase A interacting protei	1.05265991	-1.175139365	102.497491	108.988365	82.03253	6.58583649	6.65912642	6.35562515	3732	5249	3906
1207	NM_001293,NM_001311199,NM	CLNS1A	chloride nucleotide-sensitive cha	1.125569267	-1.175202019	71.52051	80.294408	57.131865	6.57931844	6.74829378	6.34904298	3715	5587	3888
54810	NM_001304725,NM_017655	GIPC2	GIPC PDZ domain containing fa	-1.755216363	-1.175222691	3.214669	1.8302	2.569194	3.70573844	2.97505117	3.49199507	473	360	495
199223	NM_001105513,NM_145755	TTC21A	tetratricopeptide repeat domain 2	1.350992186	-1.175299466	0.665965	0.908617	0.529376	1.90722898	2.23798388	1.73991364	108	195	113
55611	NM_017670,NR_003089	OTUB1	OTU deubiquitinase, ubiquitin ald	1.323768513	-1.175320012	41.892717	55.533737	33.963315	6.62322036	7.02429717	6.39272292	3831	6776	4009
59277	NM_001329700,NM_001329701	NTN4	netrin 4	-2.216730701	-1.175386575	15.094734	6.847028	11.971028	5.74684273	4.6306459	5.51839824	2069	1247	2165
112970	NM_138417	KTI12	KTI12 chromatin associated hom	1.021872179	-1.175455872	8.982991	9.188379	7.185894	3.98637962	4.01565009	3.76899703	583	796	610
7673	NM_001129996,NM_013360	ZNF222	zinc finger protein 222	1.074458802	-1.175540027	4.804005	5.158114	3.831029	3.12395512	3.21608274	2.91931156	303	435	317
23277	NM_015229	CLUH	clustered mitochondria homolog	1.223147032	-1.175565847	23.604919	28.906191	18.86742	6.91970718	7.20813537	6.68843641	4714	7704	4932
54974	NM_001317824,NM_001317825	THG1L	tRNA-histidine guanylyltransfera	-1.231545617	-1.175595976	16.712979	13.581647	13.538763	5.59698677	5.30338313	5.36880401	1861	2019	1947
92579	NM_001319945,NM_138387,NR	G6PC3	glucose-6-phosphatase catalytic	-1.250198803	-1.17575384	64.744288	52.219537	51.29169	6.6108664	6.29238721	6.37986487	3798	4059	3973
291	NM_001151	SLC25A4	solute carrier family 25 member 4	1.059933547	-1.175818511	5.401499	5.732822	4.314567	4.60021751	4.68083331	4.37694243	913	1293	955
161779	NM_152595	PGBD4	piggyBac transposable element c	-1.128900168	-1.175884391	2.459373	2.175018	1.963924	2.71208982	2.56520021	2.51644942	218	258	228
100302285	NR_036052_chr12,NR_036052_	MIR1244-1	microRNA 1244-1	-1.199202058	-1.175932471	13.925621	11.637492	11.074837	1.08456011	0.95166931	0.96541723	44	49	46
7462	NM_014146,NM_032463,NM_03	LAT2	linker for activation of T-cells fam	-5.10427366	-1.175983833	4.694352	0.916788	3.603669	3.38540081	1.51053416	3.17555833	371	97	388
79154	NM_024308	DHRS11	dehydrogenase/reductase 11	1.009580402	-1.176007654	17.500071	17.682484	13.97753	4.81908641	4.83235848	4.59413082	1069	1442	1118
51626	NM_001193464,NM_001348912	DYNC2LI1	dynein cytoplasmic 2 light interm	1.178692164	-1.176255398	11.582735	13.67002	9.627778	4.05651939	4.28053774	3.83747659	614	967	642
55268	NM_001198961,NM_001198962	ECHDC2	enoyl-CoA hydratase domain cor	-1.169676447	-1.176270829	28.558239	25.409971	22.834669	5.71081263	5.48935666	5.48142583	2017	2304	2109
23729	NM_013276	SHPK	sedoheptulokinase	-1.033859167	-1.176490712	6.151641	5.956638	4.911619	4.58498718	4.53897585	4.36103743	903	1167	944
377677	NM_198584	CA13	carbonic anhydrase 13	-1.786306467	-1.176630145	3.146099	1.763192	2.512555	3.68016614	2.92884759	3.46519272	464	347	485
471	NM_004044	ATIC	5-aminoimidazole-4-carboxamide	1.444713481	-1.176633409	83.41478	120.645126	66.619019	7.4115577	7.93973748	7.17838449	6645	12827	6946
54915	NM_017798	YTHDF1	YTH N6-methyladenosine RNA b	-1.050338544	-1.176665833	33.047554	31.503328	26.393658	6.72329619	6.65312707	6.49099484	4109	5227	4295
148413	NR_015434	LOC148413	uncharacterized LOC148413	1.128035707	-1.176997359	9.319624	10.529037	7.438482	4.07625981	4.2403589	3.85616495	623	939	651
57147	NM_020423,NM_181093	SCYL3	SCY1 like pseudokinase 3	1.048640685	-1.177073083	6.303361	6.584589	5.030597	4.26009342	4.32512669	4.03812306	713	999	745
5515	NM_002715	PPP2CA	protein phosphatase 2 catalytic s	1.165960705	-1.177092859	121.268913	141.550522	96.812225	8.29035074	8.51121563	8.05593612	12252	19087	12802
153733	NM_001040440,NM_152549	CCDC112	coiled-coil domain containing 112	-1.266708717	-1.177311232	7.269753	5.739499	5.786912	4.22318689	3.90250283	4.00128413	694	732	725
84166	NM_001330552,NM_032206	NLRC5	NLR family CARD domain contai	-1.57578684	-1.177336554	5.571004	3.534036	4.44566	5.22882245	4.59467379	5.00008264	1433	1215	1497
4343	NM_001130079,NM_001286072	MOV10	Mov10 RISC complex RNA helic	1.173187958	-1.177450971	45.527954	52.941062	36.117883	7.25749696	7.48654238	7.02349736	5968	9355	6234
81932	NM_001304509,NM_001304510	HDHD3	haloacid dehalogenase like hydro	-1.101276885	-1.177455134	25.446	22.85471	21.197211	5.35808361	5.22245468	5.12862225	1571	1906	1641
10336	NM_001317836,NM_006315	PCGF3	polycomb group ring finger 3	-1.11558728	-1.17764059	15.194465	13.657073	12.110966	6.50883804	6.35285968	6.27574222	3536	4235	3693
79667	NR_026804	KLF3-AS1	KLF3 antisense RNA 1	-1.097033567	-1.177667222	0.250984	0.225643	0.198792	0.66524311	0.61708849	0.58209675	23	28	24
55008	NM_001165136,NM_017912	HERC6	HECT and RLD domain containir	-1.871222934	-1.177770087	5.998079	3.166364	4.761119	4.5509035	3.69943092	4.32571472	881	629	920
103344931	NR_120684	LOC103344931	uncharacterized LOC103344931	1.089585511	-1.17785106	1.256782	1.370117	1.004231	2.63136183	2.73592201	2.43591723	204	297	213
2597	NM_001256799,NM_001289745	GAPDH	glyceraldehyde-3-phosphate deh	1.000192813	-1.178017834	3956.606074	3960.246739	3155.247219	12.39848064	12.39875874	12.1621667	211927	283215	221267
65005	NM_001300733,NM_031420,NR	MRPL9	mitochondrial ribosomal protein L	1.038084757	-1.178044306	35.909837	37.679763	28.596538	5.86441029	5.91742821	5.63240621	2248	3118	2347
150209	NM_001018060,NM_001146288	AIFM3	apoptosis inducing factor, mitoch	2.055154302	-1.178066781	6.651487	13.772555	5.241134	4.00480921	4.99729182	3.78425716	591	1623	617
55314	NM_018342	TMEM144	transmembrane protein 144	-1.486643427	-1.178079858	4.12287	2.776571	3.287687	3.84009957	3.3161202	3.62144026	523	470	546
125150	NM_001042697,NM_001042698	ZSWIM7	zinc finger SWIM-type containing	-1.430255936	-1.178083922	4.842112	3.396382	3.885649	3.36057263	2.90334193	3.14885876	364	340	380
1140	NM_000747	CHRNB1	cholinergic receptor nicotinic beta	1.268459951	-1.178176143	0.492961	0.62093	0.393567	1.1880553	1.31437719	0.99569924	46	78	48
55080	NM_001351355,NM_018009,NR	TAPBPL	TAP binding protein like	1.160058102	-1.17836008	15.711054	18.081609	12.388852	4.88399419	5.09145262	4.6558773	1120	1736	1169
79714	NM_001256964,NM_001256965	CCDC51	coiled-coil domain containing 51	-1.034735151	-1.178411488	10.300438	10.12661	8.25729	4.06532627	4.01904684	3.84373298	618	798	645
286527	NM_001350211,NM_001350212	TMSB15B	thymosin beta 15B	1.675057121	-1.178431699	2.825503	4.632543	1.662044	1.74129091	2.30073045	1.57922529	92	206	96
1564	NM_001348386,NR_002570,NR	CYP2D7	cytochrome P450 family 2 subfar	-1.035630275	-1.178845305	1.797843	1.74549	1.440621	2.3579234	2.31737991	2.16988427	162	209	169
23408	NM_001193267,NM_001242827	SIRT5	sirtuin 5	1.210049172	-1.178901638	5.299858	6.286476	4.897822	4.43336854	4.69682839	4.20778788	809	1308	844
7251	NM_006292	TSG101	tumor susceptibility 101	-1.549764472	-1.179234464	79.787788	51.541073	63.581585	6.92213328	6.29659183	6.68640722	4722	4071	4925
7263	NM_001270483,NM_003312	TST	thiosulfate sulfurtransferase	1.229415656	-1.179424794	59.103611	72.74279	47.082439	6.05387759	6.34780345	5.81967417	2569	4220	2679
6502	NM_001243120,NM_005983,NM	SKP2	S-phase kinase associated prote	1.097835406	-1.179521623	6.398002	7.273179	5.807314	4.19336823	4.32100453	3.96921401	679	996	708
64841	NM_198066	GNPNAT1	glucosamine-phosphate N-acetyl	1.032927328	-1.179526801	22.443855	23.208763	17.876976	6.44024851	6.48645901	6.20501134	3370	4651	3514
58492	NM_021217	ZNF77	zinc finger protein 77	-1.094462681	-1.179555807	6.910949	6.314111	5.498519	3.88058045	3.75955568	3.65976826	539	658	562
29123	NM_001256182,NM_001256183	ANKRD11	ankyrin repeat domain 11	-1.016288622	-1.179616816	38.611386	36.734785	28.991898	8.22676055	8.2035286	7.98930454	11722	15411	12222
9701	NM_001242898,NM_001242899	PPP6R2	protein phosphatase 6 regulatory	1.018009888	-1.179646426	12.249014	12.564356	9.782371	5.64404118	5.66928366	5.41084548	1924	2617	2006
65260	NM_023077	COA7	cytochrome c oxidase assembly f	1.120868962	-1.179885507	7.872251	8.840347	6.269538	4.1064401	4.26202234	3.88273891	637	954	664
64434	NM_138400	NOM1	nucleolar protein with MIF4G dor	-1.490339335	-1.179960581	8.233822	5.531357	6.5571	5.63593514	5.0744123	5.40239335	1913	1715	1994
3843	NM_002271	IPO5	importin 5	-1.049000755	-1.179978769	21.001051	20.0439	16.725603	6.95122956	6.88278346	6.71455945	4819	6138	5023
7389	NM_000374,NR_036510	UROD	uroporphyrinogen decarboxylase	1.11564154	-1.180226991	31.54502	35.313408	25.027366	5.47753469	5.6320572	5.24427765	1710	2549	1782
25840	NM_014033	METTL7A	methyltransferase like 7A	-1.002496221	-1.180317956	4.775183	4.764867	3.800275	4.06751962	4.06413701	3.84373298	619	825	645
223082	NM_147128	ZNRF2	zinc and ring finger 2	-1.94032052	-1.180850545	4.726562	2.439236	3.765173	4.08278037	3.20419534	3.85822657	626	431	652
84292	NM_001099737,NM_032332,NR	WDR83	WD repeat domain 83	1.000091425	-1.180891867	9.242317	9.04292	6.750021	3.66286259	3.6629841	3.44338646	458	612	477
3902	NM_002286	LAG3	lymphocyte activating 3	-2.220212969	-1.181002994	3.491138	1.571228	2.776402	2.95890107	2.01797632	2.75199454	266	160	277
7915	NM_001080,NM_170740	ALDH5A1	aldehyde dehydrogenase 5 famil	-1.283043331	-1.181086282	5.143511	4.035097	4.110046	4.74838854	4.40389042	4.51793371	1016	1058	1058
7849	NM_003466,NM_013952,NM_01	PAX8	paired box 8	2.379757536	-1.181212589	0.312552	0.808615	0.248356	1.16869613	1.99055518	1.03996283	49	156	51
8448	NM_001282062,NM_001282063	DOC2A	double C2 domain alpha	-1.130915425	-1.181310695	12.106825	10.813034	9.628794	4.65229204	4.48227194	4.42223881	948	1120	987
51596	NM_001014433,NM_001014837	CUTA	cutA divalent cation tolerance ho	-1.138966294	-1.181334966	61.884587	54.875	49.95497	5.91766008	5.73323878	5.68155094	2334	2738	2430
9050	NM_024430	PSTPIP2	proline-serine-threonine phospha	-1.002090244	-1.181538926	1.266441	1.26701	1.009544	2.24618091	2.24380223	2.05953665	147	196	153
9646	NM_001346279,NM_014633	CTR9	CTR9 homolog, Paf1/RNA polym	-1.100094165	-1.181653444	21.61416	19.661548	17.168058	6.60936173	6.47320892	6.37122872	3794	4608	3949
109	NM_001320613,NM_004036	ADCY3	adenylate cyclase 3	-1.027982672	-1.181711806	13.279875	12.937276	10.56328	5.95841587	5.91924724	5.72173543	2402	3122	2500
6621	NM_003086	SNAPC4	small nuclear RNA activating con	1.389343073	-1.181849436	13.250576	18.433765	10.537092	5.93337942	6.40116956	5.69660757	2360	4381	2456
2302	NM_001454	FOXJ1	forkhead box J1	1.539797094	-1.181965081	1.709549	2.64097	1.357214	2.43469539	2.96099164	2.24113096	173	356	180
56984	NM_020232,NM_147163	PSMG2	proteasome assembly chaperone	-1.201673203	-1.181995554	80.966666	67.478143	64.381723	6.48483163	6.22302312	6.24652731	3477	3866	3618
388228	NM_001024401	SBK1	SH3 domain binding kinase 1	1.206973713	-1.182068767	0.648306	0.784473	0.516325	2.05603457	2.26684378	1.87636985	124	200	129
55689	NM_001351369,NM_001351370	YEATS2	YEATS domain containing 2	1.368670715	-1.182111747	16.02843	21.979957	12.482373	6.65091025	7.09982388	6.41214851	3906	7143	4064
3038	NM_001199280,NM_005329,NM	HAS3	hyaluronan synthase 3	-1.336059709	-1.182124381	4.675517	3.505077	4.474048	4.36010717	3.96547676	4.1314271	767	767	798
101928504	NR_135252	LOC101928504	uncharacterized LOC101928504	1.46593428	-1.182147087	0.631526	0.928409	0.505768	1.18495016	1.52015496	1.05442063	50	98	52
56910	NM_020151	STARD7	StAR related lipid transfer domai	1.098721319	-1.18215962	32.567963	35.826298	25.890993	6.75698258	6.89161234	6.51797857	4207	6176	4377
5305	NM_001330062,NM_005028	PIP4K2A	phosphatidylinositol-5-phosphate	1.187761167	-1.182248641	6.936912	8.251524	5.514142	4.74975504	4.98952124	4.51793371	1017	1614	1058
57410	NM_001048218,NM_020680	SCYL1	SCY1 like pseudokinase 1	1.124133059	-1.182275594	32.563535	36.620842	25.827708	6.39947667	6.56640623	6.16101314	3275	4919	3407
29062	NM_014149	WDR91	WD repeat domain 91	-1.610349094	-1.182455477	2.259031	1.401721	1.795057	3.48064928	2.86987209	3.26218696	399	331	415
26272	NM_001297437,NM_012176,NM	FBXO4	F-box protein 4	1.061392236	-1.182542079	4.826856	5.049425	3.826431	3.00082115	3.07634485	2.79136857	275	390	286
51441	NM_001172828,NM_001173128	YTHDF2	YTH N6-methyladenosine RNA b	1.070352976	-1.182706225	47.350383	46.34531	36.792139	7.02079481	7.11815303	6.78072565	5059	7235	5261
8893	NM_003907	EIF2B5	eukaryotic translation initiation fa	-1.034751739	-1.182717078	30.530216	29.535965	24.251188	6.44531763	6.3966066	6.2062258	3382	4367	3517
55262	NM_001303470,NM_018275	C7orf43	chromosome 7 open reading fran	-1.497925468	-1.182769798	11.566689	7.759883	9.177862	4.89515141	4.33606188	4.66179103	1129	1007	1174
101929580	NR_135738	GTF2IP23	general transcription factor IIi pse	-1.253198711	-1.182909632	1.918126	1.525158	1.52053	1.55375576	1.34717796	1.39836968	76	81	79
100500804	NR_037427_chr11,NR_037427_	MIR3654	microRNA 3654	1.100428163	-1.183046411	23.513235	26.194986	18.753134	1.2010231	1.28081328	1.06873498	51	75	53
10714	NM_006591,NR_046409,NR_04	POLD3	DNA polymerase delta 3, access	1.088126014	-1.183054121	12.069973	13.100271	9.273785	5.52379552	5.64310623	5.28699192	1767	2569	1837
200014	NM_001330585,NM_032449	CC2D1B	coiled-coil and C2 domain contai	1.009683492	-1.183102593	9.472474	9.503995	7.537961	5.67817204	5.69180565	5.44073202	1971	2659	2049
85359	NM_033257	DGCR6L	DiGeorge syndrome critical regio	1.215790561	-1.183121687	10.861599	13.223682	8.630598	3.79580649	4.05919602	3.57205238	506	822	526
100527963	NM_001199661,NM_001199662	PMF1-BGLAP	PMF1-BGLAP readthrough	1.154933635	-1.183155507	3.364853	4.016321	2.730392	2.0823043	2.24380223	1.90057841	127	196	132
8479	NM_001197323,NM_003609	HIRIP3	HIRA interacting protein 3	1.312362725	-1.183282686	13.383023	17.745148	10.308669	5.29021237	5.67360076	5.05414007	1497	2625	1556
285830	NR_026972,NR_026973	HLA-F-AS1	HLA-F antisense RNA 1	1.589929989	-1.18350687	1.748336	2.793743	1.387228	2.09095564	2.62883416	1.90855849	128	272	133
83743	NM_031485	GRWD1	glutamate rich WD repeat contair	1.352103988	-1.183709208	17.732075	24.000875	14.074002	5.4399354	5.86648449	5.20269596	1665	3008	1730
10425	NM_001317333,NM_001317334	ARIH2	ariadne RBR E3 ubiquitin protein	-1.034331335	-1.183743073	25.626355	24.690581	19.058101	6.50924151	6.46108525	6.2687844	3537	4569	3675
10965	NM_006821,NR_046028	ACOT2	acyl-CoA thioesterase 2	-1.107710748	-1.183801726	12.928032	11.883399	10.332394	4.53830704	4.39737979	4.30621424	873	1053	907
100506737	NR_131244	LINC01589	long intergenic non-protein codin	-1.133795024	-1.184505519	7.139816	6.306775	5.66094	2.32162912	2.17847216	2.1294865	157	185	163
55332	NM_018370	DRAM1	DNA damage regulated autophag	-2.081981583	-1.184770834	6.198373	2.979411	4.916729	4.48515183	3.49508138	4.25236388	840	539	872
145553	NM_001199821,NM_001199822	MDP1	magnesium dependent phosphat	-1.168733009	-1.184818084	15.962843	13.897172	12.516362	3.63059014	3.42511354	3.4072329	447	511	464
23256	NM_001257376,NM_001283031	SCFD1	sec1 family domain containing 1	1.010356584	-1.184896854	44.88475	45.007801	35.33895	6.60747869	6.62219199	6.36544244	3789	5115	3933

79078	NM_024097,NR_040733	C1orf50	chromosome 1 open reading fran	1.07894667	-1.184996895	8.298256	8.866535	6.47096	3.18179291	3.27976824	2.96594348	317	457	329
728441	NM_001351304	GGT2	gamma-glutamyltransferase 2	1.053141559	-1.185097498	0.269849	0.2848	0.209614	0.7550755	0.7861039	0.65979727	27	38	28
6218	NM_001021,NM_001021_dup1,N	RPS17	ribosomal protein S17	-1.079411923	-1.185163365	460.740143	459.23476	398.408071	8.31248151	8.20259533	8.06823321	12442	15401	12912
202051	NM_194296	SPATA24	spermatogenesis associated 24	-1.174339129	-1.18517217	3.066246	2.603416	2.443146	1.602974	1.45142181	1.4430085	80	91	83
54989	NM_014106	ZNF770	zinc finger protein 770	-1.014625474	-1.185227727	11.076495	10.929176	8.781211	5.90115281	5.88055759	5.66044042	2307	3038	2394
9666	NM_014648	DZIP3	DAZ interacting zinc finger protei	1.220859375	-1.185320434	10.033641	12.263366	7.955138	5.7853302	6.06850015	5.5448789	2126	3468	2206
5440	NM_005034	POLR2K	RNA polymerase II subunit K	-1.448553818	-1.185385	75.993637	52.506802	60.248295	6.18693619	5.66115412	5.94523646	2821	2602	2927
761	NM_005181	CA3	carbonic anhydrase 3	-1.50164333	-1.185552145	0.792263	0.535658	0.637989	1.24819466	0.93737891	1.11084509	54	48	56
151176	NM_001291832	ERFE	erythroferrone	1.055026927	-1.185574878	5.779451	6.111972	4.581244	4.11706758	4.19001636	3.88678411	642	905	666
112936	NM_052875	VPS26B	VPS26, retromer complex compc	-1.977263546	-1.18562133	15.760734	7.977328	12.489781	5.86441029	4.90483992	5.62334321	2248	1519	2332
5993	NM_000449,NM_001025603	RFX5	regulatory factor X5	-1.155537285	-1.185637788	21.111044	18.300641	16.743776	6.23505098	6.02945501	5.99292867	2918	3374	3027
9577	NM_001261840,NM_001329112	BABAM2	BRISC and BRCA1 A complex m	1.078042222	-1.185809152	16.626327	18.127872	13.490869	4.96034122	5.06540544	4.72302988	1183	1704	1227
169792	NM_001042413,NM_152629	GLIS3	GLIS family zinc finger 3	-1.024846396	-1.185826007	0.459306	0.450199	0.364401	2.15010456	2.12272838	1.96321628	135	176	140
1072	NM_005507	CFL1	cofilin 1	1.150203811	-1.185900717	655.784424	755.136475	519.658691	9.65032408	9.85197978	9.40467361	31510	48425	32680
27436	NM_001145076,NM_019063	EML4	echinoderm microtubule associat	1.093731005	-1.186207906	22.463135	24.594893	17.792303	6.93810392	7.06635603	6.69392988	4775	6978	4951
25864	NM_015407	ABHD14A	abhydrolase domain containing 1	-1.636564627	-1.186277622	8.994252	5.484768	7.119893	3.42014445	2.79259185	3.19852188	381	311	395
339541	NM_001145636	C1orf228	chromosome 1 open reading fran	-1.165427729	-1.18637464	2.542078	2.181822	2.011256	2.37218925	2.19658485	2.1765086	164	188	170
10195	NM_001006941,NM_005787,NR	ALG3	ALG3, alpha-1,3- mannosyltransf	1.246338814	-1.186531755	48.890387	61.43454	38.85539	6.21193794	6.52579291	5.96880267	2871	4781	2976
10325	NM_006064,NM_016656	RRAGB	Ras related GTP binding B	1.16954475	-1.186594874	3.030052	3.555714	2.403011	2.86618866	3.06324294	2.65571422	247	386	256
85465	NM_033505,NR_137633	SELENOI	selenoprotein I	-1.000025599	-1.186701965	10.513785	10.531675	8.324195	6.39599007	6.39595357	6.1522182	3267	4365	3386
1465	NM_001193570,NM_001193571	CSRP1	cysteine and glycine rich protein	1.07621397	-1.18696123	18.182936	19.50592	14.351923	5.1492545	5.2523455	4.90954071	1354	1947	1403
26240	NM_012135	FAM50B	family with sequence similarity 5(	-1.079286994	-1.186993095	10.497228	9.736529	8.311473	4.16292023	4.05919602	3.93054845	664	822	688
113115	NM_001099286,NM_001318738	MTFR2	mitochondrial fission regulator 2	-1.202473934	-1.187034343	10.934017	9.719651	9.068913	4.27345604	4.02243562	4.03994054	720	800	746
100533105	NM_001204173	C8orf44-SGK3	C8orf44-SGK3 readthrough	-1.21136004	-1.187471296	2.724397	2.25247	2.158976	3.71416279	3.46052158	3.48667429	476	525	493
11093	NM_139025,NM_139026,NM_13	ADAMTS13	ADAM metallopeptidase with thrc	1.216062466	-1.187543387	4.955355	6.171457	3.632061	4.44015563	4.71054878	4.20455055	813	1321	842
55748	NM_001168499,NM_018235	CNDP2	carnosine dipeptidase 2	-1.382482248	-1.187699305	28.249079	20.428735	22.280914	7.1289776	6.66564285	6.8827358	5456	5273	5650
23307	NM_015258	FKBP15	FK506 binding protein 15	1.051067457	-1.187748494	20.213488	21.272608	15.993267	6.4321858	6.50323133	6.18708076	3351	4706	3470
101926917	NR_136286	ANKRD18CP	ankyrin repeat domain 18C, pseu	1.430408679	-1.187803654	0.379066	0.540797	0.298953	1.29387258	1.62195412	1.15176086	57	109	59
619568	NR_002588	SNORA4	small nucleolar RNA, H/ACA box	-1.045764119	-1.188478715	158.707077	152.088562	125.61245	4.47196531	4.41037179	4.23501577	832	1063	861
283345	NR_002803	RPL13P5	ribosomal protein L13 pseudoger	-2.492955691	-1.188504238	2.398034	0.95918	1.910848	1.30878272	0.669904	1.16514558	58	31	60
129025	NM_080740	ZNF280A	zinc finger protein 280A	1.276989111	-1.188504238	0.701689	0.892796	0.553818	1.30878272	1.52971203	1.16514558	58	99	60
11194	NM_001282291,NM_001282292	ABCB8	ATP binding cassette subfamily E	-1.57053795	-1.188607065	9.78528	6.331648	7.79903	5.51417891	4.88077062	5.27083173	1755	1493	1816
8667	NM_003756	EIF3H	eukaryotic translation initiation fa	-1.108401877	-1.18866265	392.379852	354.411072	310.203094	8.93106093	8.78289936	8.68227753	19124	23053	19788
22948	NM_001306153,NM_001306154	CCT5	chaperonin containing TCP1 sub	1.024902942	-1.188721436	100.81077	103.228581	78.729452	8.46917842	8.50456706	8.22053363	13874	18999	14355
84632	NM_001001936,NM_001287824	AFAP1L2	actin filament associated protein	2.008767205	-1.188854583	5.167752	10.324849	4.065499	4.38670105	5.35805128	4.15005835	782	2099	809
64429	NM_001303134,NM_001351082	ZDHHC6	zinc finger DHHC-type containing	-1.202844914	-1.188943377	28.497684	23.765362	22.698013	5.97895271	5.71712177	5.73357571	2437	2707	2521
79915	NM_024857	ATAD5	ATPase family, AAA domain cont	-1.200229058	-1.18915495	10.666974	8.897248	8.4315	6.17630487	5.9169731	5.93012593	2800	3117	2896
79582	NM_001025436,NM_024532,NR	SPAG16	sperm associated antigen 16	1.289810495	-1.189214923	21.200315	27.385894	16.883614	4.79678491	5.15226631	4.55653526	1052	1813	1088
51400	NM_001271593,NM_016147	PPME1	protein phosphatase methylester	1.37060641	-1.189295275	27.093918	37.171299	21.401636	6.18137717	6.63082098	5.93501761	2810	5146	2906
2017	NM_001184740,NM_005231,NM	CTTN	cortactin	-1.001605135	-1.189400736	119.319217	119.157179	93.77006	8.52351383	8.52120624	8.27401919	14408	19220	14899
65980	NM_001009877,NM_001317951	BRD9	bromodomain containing 9	-1.1763244	-1.189528543	21.039387	17.919015	16.713051	5.85808894	5.62816997	5.61239193	2238	2542	2314
112755	NM_052874	STX1B	syntaxin 1B	1.117509715	-1.18957048	0.82814	0.926218	0.650198	2.25390578	2.38212036	2.05953665	148	221	153
2580	NM_001318134,NM_005255	GAK	cyclin G associated kinase	-1.10369295	-1.189865657	42.552298	38.575747	33.607232	7.5591454	7.41759738	7.30979425	7365	8916	7613
2135	NM_001033025,NM_001261440	EXTL2	exostosin like glycosyltransferase	1.307167978	-1.189870073	7.79589	10.200575	6.116795	4.65666933	5.02964715	4.41665393	951	1661	983
8824	NM_003869,NM_198061,NR_03	CES2	carboxylesterase 2	1.137950423	-1.189951412	6.195533	6.819215	4.736357	4.65958016	4.83910025	4.41944907	953	1449	985
5534	NM_000945	PPP3R1	protein phosphatase 3 regulatory	1.137410583	-1.189963832	26.647491	30.33997	21.041172	6.30596848	6.48952306	6.05850059	3067	4661	3170
10726	NM_006600	NUDC	nuclear distribution C, dynein cor	1.209652717	-1.190013884	93.768845	113.561913	74.053558	7.3796966	7.6527914	7.13035863	6499	10504	6717
3633	NM_001297434,NM_001350227	INPP5B	inositol polyphosphate-5-phosph	1.017050847	-1.190034486	7.497151	7.109178	5.908295	4.90131278	4.92489722	4.65942844	1134	1541	1172
9187	NM_001254740,NM_001301031	SLC24A1	solute carrier family 24 member '	-1.153706766	-1.190066185	1.72696	2.006522	1.248392	3.22958708	3.04669644	3.00740444	329	381	340
3931	NM_000229	LCAT	lecithin-cholesterol acyltransferas	-1.252380671	-1.190126845	9.252154	7.393457	7.302317	3.72253824	3.42511354	3.49199507	479	511	495
100996939	NM_032906	PYURF	PIGY upstream reading frame	-1.06002375	-1.190193876	41.031384	38.747841	32.39901	5.78599633	5.70346404	5.53974996	2127	2681	2198
10440	NM_006335	TIMM17A	translocase of inner mitochondria	1.01254845	-1.190275114	53.483776	54.211391	42.221226	6.44952833	6.46731524	6.20136181	3392	4589	3505
54148	NM_017446,NM_080794	MRPL39	mitochondrial ribosomal protein L	-1.10380866	-1.190522713	36.50725	33.102475	28.809751	5.30330016	5.16458753	5.05862945	1511	1829	1561
284359	NM_001321864,NM_001321865	IZUMO1	izumo sperm-egg fusion 1	-1.4261675	-1.190678175	0.694932	0.379668	0.530783	0.83964067	0.63490936	0.73352601	31	29	32
5437	NM_001278698,NM_001278699	POLR2H	RNA polymerase II subunit H	1.087434176	-1.19099011	123.933003	142.654065	104.800556	7.54229052	7.66259786	7.2916023	7279	10576	7517
252983	NM_178509	STXBP4	syntaxin binding protein 4	1.285244214	-1.191002819	1.193786	1.533714	0.93924	3.00540462	3.32712361	2.78704637	276	474	285
1936	NM_001130053,NM_001130055	EEF1D	eukaryotic translation elongation	-1.006912455	-1.191315232	326.145172	323.635335	268.020342	8.8983199	8.88840249	8.64634137	18694	24806	19300
5902	NM_001278639,NM_001278640	RANBP1	RAN binding protein 1	1.075993947	-1.191330883	76.305841	79.491648	58.789161	6.47660236	6.58113081	6.22711565	3457	4970	3569
7771	NM_001083335,NM_001348281	ZNF112	zinc finger protein 112	1.281512413	-1.191431074	2.599123	3.374867	2.068137	3.38891307	3.71626888	3.16227031	372	637	384
23218	NM_015175	NBEAL2	neurobeachin like 2	-1.351249565	-1.191529065	23.811829	17.641199	18.779875	7.68429855	7.25245928	7.43282335	8036	7946	8295
84727	NM_001146316,NM_001319670	SPSB2	splA/ryanodine receptor domain a	-1.365264143	-1.191531049	6.917041	5.046849	5.400815	3.3924168	2.99243508	3.16560381	373	365	385
554251	NM_001024680,NR_133923	FBXO48	F-box protein 48	1.202042342	-1.191623656	1.336652	2.518115	1.645824	1.76310734	1.95552917	1.58918956	94	151	97
9063	NM_001324046,NM_001324047	PIAS2	protein inhibitor of activated STA	1.360897833	-1.191636421	2.683944	4.015448	2.660385	4.58498718	5.01356019	4.34348418	903	1642	932
84950	NM_032864	PRPF38A	pre-mRNA processing factor 38A	1.103843759	-1.191642643	22.914383	25.321548	18.06608	5.97369967	6.11408027	5.7251283	2428	3581	2506
377	NM_001659	ARF3	ADP ribosylation factor 3	-1.087572375	-1.191823922	58.055721	53.445126	45.775387	7.65067983	7.53019529	7.39888132	7850	9644	8101
60561	NM_001346599,NM_001346600	RINT1	RAD50 interactor 1	-1.998836021	-1.191837574	17.117978	8.681224	13.304395	5.63297616	4.66248855	5.38533907	1909	1276	1970
140738	NM_183240	TMEM37	transmembrane protein 37	-1.220695978	-1.191862835	0.491636	0.405522	0.386892	0.86002952	0.73744528	0.75138414	32	35	33
144193	NM_152435	AMDHD1	amidohydrolase domain containir	1.23879597	-1.191862835	0.381027	0.47855	0.308159	0.86002952	1.00745609	0.75138414	32	53	33
29802	NM_013378	VPREB3	V-set pre-B cell surrogate light cl	-1.377481973	-1.191862835	1.421137	1.023458	1.104031	0.86002952	0.669904	0.75138414	32	31	33
168850	NM_176814	ZNF800	zinc finger protein 800	-1.7968928	-1.191865751	4.926795	2.74304	3.883471	4.49496323	3.69943092	4.25393069	846	629	873
9783	NM_014747	RIMS3	regulating synaptic membrane ex	-1.847960208	-1.191891209	0.793084	0.428877	0.626417	2.72326229	2.01116974	2.51121823	220	159	227
5001	NM_002553,NM_181747	ORC5	origin recognition complex subun	-1.16146957	-1.192081017	10.115844	8.448441	7.92924	4.28292556	4.07885924	4.04356865	725	834	748
390916	NM_001105570	NUDT19	nudix hydrolase 19	-1.253275233	-1.192454838	16.275555	12.995468	12.824581	5.58555344	5.26741889	5.3373729	1846	1968	1904
94056	NM_032796,NR_033181	SYAP1	synapse associated protein 1	-1.439771696	-1.192607353	26.79936	18.633198	21.114655	6.53878911	6.0197383	6.28764702	3611	3351	3724
572	NM_004322,NM_032989	BAD	BCL2 associated agonist of cell c	1.334395971	-1.192720302	21.428395	28.334544	17.114032	4.47527325	4.87515868	4.23342827	834	1487	860
126661	NR_033296,NR_033297,NR_03:	CCDC163	coiled-coil domain containing 163	-1.480794235	-1.192904236	2.406176	1.608478	1.880215	2.57080169	2.11639955	2.36229074	194	175	200
7425	NM_003378	VGF	VGF nerve growth factor inducibl	1.016381639	-1.192951565	1.650926	1.687384	1.302691	2.3579234	2.37683491	2.15654364	162	220	167
440435	NM_001004334	GPR179	G protein-coupled receptor 179	2.19606499	-1.19297809	0.104907	0.238119	0.084667	0.88013425	1.51053416	0.76902392	33	97	34
224	NM_000382,NM_001031806	ALDH3A2	aldehyde dehydrogenase 3 famil	-1.075992895	-1.193112852	9.744484	9.073067	7.687037	5.19009033	5.08741362	4.94294958	1394	1731	1437
10775	NM_006627,NR_027368	POP4	POP4 homolog, ribonuclease P/M	-1.287130724	-1.193115967	19.763702	15.406199	15.517146	5.65939101	5.30338313	5.41014302	1945	2019	2005
2167	NM_001442	FABP4	fatty acid binding protein 4	11.77988958	-1.19313888	3.039033	36.092472	2.407434	1.80577475	4.92761082	1.62837236	98	1544	101
399473	NM_001042522,NR_073032	SPRED3	sprouty related EVH1 domain cor	1.102571541	-1.193295333	0.677067	0.747419	0.528381	2.11660268	2.22627631	1.92438744	131	193	135
84866	NM_001144034,NM_001144035	TMEM25	transmembrane protein 25	1.476329951	-1.193314587	3.648711	5.35472	2.711386	3.08551061	3.59177559	2.86293141	294	580	303
54662	NM_001286772,NM_018201	TBC1D13	TBC1 domain family member 13	1.153232644	-1.193331935	9.182325	10.620775	7.229617	5.16572779	5.36607572	4.9184598	1370	2111	1412
9202	NM_001350138,NM_001350139	ZMYM4	zinc finger MYM-type containing	1.053414219	-1.193506667	9.579969	10.261069	7.621086	6.06489791	6.13888057	5.81384357	2589	3644	2668
5987	NM_006510	TRIM27	tripartite motif containing 27	-1.213720373	-1.193523515	17.910332	14.771305	14.10196	5.72199495	5.44837277	5.47203276	2033	2238	2095
8774	NM_003826	NAPG	NSF attachment protein gamma	-1.198961556	-1.193567162	22.650562	18.913424	17.835083	6.37400578	6.11566763	6.12207949	3217	3585	3315
9166	NM_001278938,NM_004215,NM	EBAG9	estrogen receptor binding site as	-1.03031801	-1.193587281	21.428371	20.713855	16.977807	5.67817204	5.63593399	5.42829662	1971	2556	2031
53373	NM_001143819,NM_001301214	TPCN1	two pore segment channel 1	-1.314060321	-1.193865772	23.47535	17.882804	18.3698	6.93570456	6.54535937	6.68234025	4767	4847	4911
100506161	NR_110613,NR_110614	TAF1A-AS1	TAF1A antisense RNA 1	1.206102454	-1.19389751	1.906372	2.299773	1.486975	1.43646263	1.61299082	1.28032816	67	108	69
1196	NM_001294338,NM_001294339	CLK2	CDC like kinase 2	-1.071316636	-1.194170093	43.081311	40.487938	33.790102	6.51045123	6.41219133	6.25750353	3540	4415	3646
54976	NM_001039140,NM_001258429	C20orf27	chromosome 20 open reading fra	1.600420932	-1.194279467	31.169461	50.04984	24.50839	5.36166293	6.02692652	5.11230382	1575	3368	1622
25895	NM_015433,NM_206914	EEF1AKMT3	EEF1A lysine methyltransferase	1.168793238	-1.194303295	4.538625	5.172102	3.405603	3.6972646	3.90617635	3.4624849	470	734	484
122509	NM_145249,NM_206949	IFI27L1	interferon alpha inducible protein	-1.396719487	-1.194344277	16.978553	12.075667	13.320907	3.60061161	3.16487058	3.36725759	437	418	450
125228	NM_001098801,NM_152352	FAM210A	family with sequence similarity 2'	1.139858577	-1.194361078	9.102214	10.371623	7.112774	5.2722583	5.45653796	5.02323609	1478	2251	1522
78999	NM_024036	LRFN4	leucine rich repeat and fibronecti	1.168001607	-1.194373945	19.123611	22.361437	15.040438	5.58708313	5.80681389	5.33663373	1848	2884	1903
100289274	NR_132117	DNAJC3-AS1	DNAJC3 antisense RNA 1 (head	-1.075077093	-1.194872305	0.924401	0.859277	0.722876	1.8573935	1.78245981	1.67590018	103	128	106
200315	NM_001270406,NM_145699	APOBEC3A	apolipoprotein B mRNA editing e	-1.239516717	-1.194872305	1.849134	1.5019	1.462691	1.8573935	1.63971536	1.67590018	103	111	106
100288798	NR_125377,NR_125378,NR_12!	LOC100288798	uncharacterized LOC100288798	-1.917941475	-1.194910691	3.237218	1.65377	2.452118	1.46336426	0.93737891	1.30472178	69	48	71

27030	NM_001040108,NM_014381	MLH3	mutL homolog 3	1.004268487	-1.19503414	6.263536	6.296624	4.938841	5.61434375	5.62036393	5.36300797	1884	2528	1939
7419	NM_001135694,NM_005662	VDAC3	voltage dependent anion channel	-1.186128726	-1.195034458	128.791625	108.688035	101.26689	7.59816676	7.35328757	7.34256181	7568	8525	7789
23129	NM_015103	PLXND1	plexin D1	1.409753648	-1.195147003	3.348767	4.728868	2.634002	4.57114104	5.04888386	4.32571472	894	1684	920
375260	NR_024077	WASH2P	WAS protein family homolog 2 ps	1.019396961	-1.195173308	24.351786	24.85006	19.148897	5.56784445	5.59498314	5.31653173	1823	2483	1876
8473	NM_181672,NM_181673	OGT	O-linked N-acetylglucosamine (G	1.143571424	-1.195202077	105.843601	121.154858	83.18158	9.14108329	9.3343098	8.88432601	22127	33809	22770
79134	NM_024121	TMEM185B	transmembrane protein 185B	1.350866913	-1.19529124	16.418444	22.202974	12.908062	6.58392248	7.01390728	6.32948591	3727	6727	3835
902	NM_001199189,NM_001239	CCNH	cyclin H	1.139706568	-1.195369732	28.163205	32.426725	22.359376	5.34548562	5.5298038	5.09492475	1557	2371	1602
105371807	NR_131239,NR_131240	THRA1/BTR	uncharacterized LOC105371807	-5.464132985	-1.195396214	2.586875	0.449503	2.028942	1.47662927	0.40498891	1.31676564	70	17	72
388886	NM_207644	LRRC75B	leucine rich repeat containing 75I	-1.05902064	-1.195417825	9.510094	8.987572	7.47541	3.64240927	3.56646274	3.4072329	451	569	464
26512	NM_001039937,NM_001039938	INTS6	integrator complex subunit 6	1.210161969	-1.195428188	6.886851	7.684053	5.061652	5.40913411	5.6784421	5.15820771	1629	2634	1676
23162	NM_001040439,NM_001318852	MAPK8IP3	mitogen-activated protein kinase	-1.132900721	-1.195551002	45.746646	40.401591	35.952758	7.97021914	7.79096	7.71366484	9806	11565	10088
23219	NM_001136115,NM_015176,NR	FBXO28	F-box protein 28	-1.100777212	-1.195653502	16.149753	14.668653	12.686694	6.43133447	6.29449105	6.17679276	3349	4065	3445
5724	NM_000952,NM_001164721,NM	PTAFR	platelet activating factor receptor	-2.141252703	-1.195707955	2.544414	1.185982	2.071775	3.54817338	2.58366954	3.31417746	420	262	432
6535	NM_001142805,NM_001142806	SLC6A8	solute carrier family 6 member 8	1.237682361	-1.195955965	15.068781	18.364592	11.570984	5.7037794	6.00610962	5.45101358	2007	3319	2064
85446	NM_033400	ZFHX2	zinc finger homeobox 2	2.275965735	-1.196053273	0.402046	0.921953	0.317806	2.20692365	3.20717639	2.00847214	142	432	146
7874	NM_001286457,NM_001286458	USP7	ubiquitin specific peptidase 7	1.16426575	-1.196057185	32.713005	37.988555	25.712116	7.43276864	7.65101367	7.17611376	6744	10491	6935
3437	NM_001031683,NM_001289758	IFIT3	interferon induced protein with te	-2.66237129	-1.196095406	25.389238	9.677555	19.985385	6.00092376	4.62508404	5.74698953	2475	1242	2545
84705	NM_001128855,NM_001195422	GTPBP3	GTP binding protein 3 (mitochon	-1.045972729	-1.196119323	17.215842	16.584752	13.569092	5.49719403	5.43381248	5.24506567	1734	2215	1783
200895	NM_001195643,NM_176815	DHFR2	dihydrofolate reductase 2	1.251204835	-1.196207412	2.127784	2.637919	1.669754	3.19389071	3.48529134	2.96594348	320	535	329
203069	NM_001136108,NM_001301650	R3HCC1	R3H domain and coiled-coil conta	1.113501635	-1.196219807	8.771393	9.844469	6.892499	3.86553291	4.01053992	3.62628732	533	793	548
9303	NR_002565	SNORD25	small nucleolar RNA, C/D box 25	1.599926444	-1.196327967	27.781132	44.874874	21.878689	1.50279892	1.97664665	1.34055586	72	154	74
80765	NM_181900,NR_135013	STARD5	StAR related lipid transfer domain	1.132820827	-1.196748369	2.158193	2.500791	1.690151	1.91699278	2.05153568	1.73094093	109	165	112
143570	NM_001270380,NM_001270381	XRRA1	X-ray radiation resistance associ	-1.545181998	-1.196796614	3.803005	2.470734	2.984247	4.20934818	3.62336263	3.96539376	687	594	706
7156	NM_001320759,NM_004618	TOP3A	topoisomerase (DNA) III alpha	1.155322498	-1.196843239	4.382072	5.072195	3.440929	4.88648104	5.08822232	4.63678882	1122	1732	1153
348801	NM_001085451	LNP1	leukemia NUP98 fusion partner 1	1.515605571	-1.196854256	0.434859	0.658386	0.339659	0.95786335	1.28081328	0.83750224	37	75	38
6626	NM_004596	SNRPA	small nuclear ribonucleoprotein p	-1.1146951	-1.196972495	89.819473	80.667427	70.50972	7.16450379	7.00900446	6.90708762	5593	6704	5747
55049	NM_001100418,NM_001100419	C19orf60	chromosome 19 open reading fra	-1.453195898	-1.197042909	33.938416	23.433993	27.011427	4.89515141	4.37766975	4.64517113	1129	1038	1160
122786	NM_001042481,NM_001267046	FRMD6	FERM domain containing 6	1.024355639	-1.197253248	6.767587	7.114248	5.445712	5.07379588	5.10749649	4.82246845	1283	1756	1318
203522	NM_001351601,NM_001351603	INTS6L	integrator complex subunit 6 like	1.107234013	-1.197746622	3.800267	4.420873	3.023242	3.92963473	4.06742162	3.68786049	559	827	574
10399	NM_006098	RACK1	receptor for activated C kinase 1	1.065761549	-1.19794621	1166.112061	1244.174561	914.748718	10.29933772	10.39115195	10.03900055	49433	70392	50753
140823	NM_080748	ROMO1	reactive oxygen species modulat	-1.110635454	-1.198033987	142.824814	128.711365	112.001411	6.02482449	5.87588179	5.76852445	2517	3028	2584
114926	NM_001135674,NM_001135675	SMIM19	small integral membrane protein	-1.410550147	-1.198262082	21.479184	15.107595	16.13903	4.53514067	4.06413701	4.28644656	871	825	894
57654	NM_001317934,NM_001317935	UVSSA	UV stimulated scaffold protein A	-1.340958171	-1.198273314	11.058985	8.24982	8.691425	5.64917596	5.2356538	5.39389141	1931	1924	1982
23503	NM_015346	ZFYVE26	zinc finger FYVE-type containing	1.121784909	-1.19827737	4.708213	5.289433	3.692237	5.50368806	5.66603734	5.24899932	1742	2611	1788
401321	NR_036501	LINC00997	long intergenic non-protein codin	-2.118348156	-1.198578063	3.018151	1.426804	2.368231	3.8730763	2.89597101	3.63111814	536	338	550
54853	NM_017706	WDR55	WD repeat domain 55	1.106111051	-1.198592256	16.287899	18.04014	12.768388	5.38998216	5.53218306	5.13543833	1607	2375	1649
153364	NM_203406	MBLAC2	metallo-beta-lactamase domain c	-1.302187088	-1.198728316	2.347846	1.80472	1.840319	3.43381096	3.09255667	3.19852188	385	395	395
58490	NM_021215	RPRD1B	regulation of nuclear pre-mRNA c	-1.012236077	-1.198865346	27.638849	27.33539	21.665611	6.71702247	6.69964401	6.45806964	4091	5400	4197
6294	NM_001201338,NM_001201339	SAFB	scaffold attachment factor B	1.109688676	-1.198988982	49.641812	55.146591	38.848283	7.23864191	7.38785491	6.97871772	5890	8733	6042
54494	NM_001271562,NM_019021	C11orf71	chromosome 11 open reading fra	-1.57630698	-1.199015213	6.544011	4.153522	5.130678	3.79315817	3.19521507	3.5518139	505	428	518
51691	NM_016200	LSM8	LSM8 homolog, U6 small nuclear	-1.624089363	-1.199073815	3.816051	2.351793	2.990684	5.57093993	4.89007563	5.3150315	1827	1503	1874
5366	NM_021127	PMAIP1	phorbol-12-myristate-13-acetate-	-1.470192183	-1.199177101	36.883751	25.109612	28.900845	6.14029041	5.59384468	5.88230216	2730	2481	2800
100128071	NM_001167676	FAM229A	family with sequence similarity 22	-1.024758507	-1.199227368	2.939294	2.865875	2.299815	1.59082596	1.5673195	1.42086173	79	103	81
152503	NM_001009555,NM_001128923	SH3D19	SH3 domain containing 19	1.082720176	-1.199356551	7.316955	6.8567	5.906275	5.04403172	5.1553565	4.7904378	1256	1817	1288
132320	NM_001300897,NM_001300898	SCLT1	sodium channel and clathrin linke	1.014031947	-1.199412623	9.258364	9.357046	7.308683	4.64643492	4.66574286	4.39551594	944	1279	968
56943	NM_001193557,NM_020189,NR	ENY2	ENY2, transcription and export co	1.115244077	-1.199460362	33.542135	37.359433	26.179845	6.58889365	6.74470783	6.32948591	3740	5573	3835
57139	NM_001035223,NM_001161616	RGL3	ral guanine nucleotide dissociatic	1.062055112	-1.199526521	56.333506	59.840703	44.13081	7.1363099	7.22257085	6.87588368	5484	7782	5623
57862	NM_001242924,NM_001242926	ZNF410	zinc finger protein 410	-1.048663141	-1.199560775	21.077493	20.233837	16.49009	5.76384974	5.69658638	5.50661727	2094	2668	2147
100131017	NM_001278559	ZNF316	zinc finger protein 316	-1.195515905	-1.199628972	9.587663	8.028501	7.508143	6.09960764	5.8460706	5.84120116	2653	2965	2720
4548	NM_000254,NM_001291939,NM	MTR	5-methyltetrahydrofolate-homocy	-1.074418354	-1.199806395	8.290303	7.815126	6.506707	6.43005653	6.32774187	6.17058457	3346	4161	3430
51522	NM_001165258,NM_016462	TMEM14C	transmembrane protein 14C	-1.131970564	-1.199883701	48.079753	42.499108	37.640848	5.80583878	5.63039253	5.54807524	2157	2546	2211
50636	NM_001001666,NM_001001891	ANO7	anoctamin 7	1.531989338	-1.199994522	0.25566	0.398446	0.20075	1.03161781	1.37924953	0.90287695	41	84	42
80023	NM_001323679,NM_001323680	NRSN2	neurensin 2	1.531989338	-1.199994522	0.430142	0.657765	0.37239	1.03161781	1.37924953	0.90287695	41	84	42
23080	NM_015060	AVL9	AVL9 cell migration associated	-1.447264095	-1.200076909	10.121162	7.002512	7.926439	6.14237278	5.61812585	5.88331483	2734	2524	2802
81929	NM_001013437,NM_031216	SEH1L	SEH1 like nucleoporin	-1.032051382	-1.20015776	20.970648	20.190686	17.908244	6.10388823	6.05904375	5.84484461	2661	3445	2727
54758	NM_001184854,NM_001184856	KLHDC4	kelch domain containing 4	1.051623952	-1.200520679	26.572339	27.989638	20.87999	5.67025604	5.74148732	5.41224936	1960	2754	2008
29984	NM_001300886,NM_014578	RHOD	ras homolog family member D	1.158370704	-1.200611054	114.636991	133.212583	90.028813	6.99106079	7.20161067	6.72955881	4955	7669	5076
135293	NM_001010853	PM20D2	peptidase M20 domain containing	-1.295237766	-1.200653379	3.873244	2.992148	3.032688	4.23103553	3.88026307	3.9825058	698	720	715
100132352	NR_034006,NR_034006_dup1	FRG1HP	FSHD region gene 1 family mem	-1.031305457	-1.200657505	4.993958	4.863311	3.919641	2.86618866	2.8278811	2.64141996	247	320	253
9583	NM_001128930,NM_004901	ENTPD4	ectonucleoside triphosphate diph	-1.387839064	-1.200735031	13.699732	9.879677	10.730402	6.31430441	5.8484592	6.05401081	3085	2970	3160
146439	NM_001103175	BICDL2	BICD family like cargo adaptor 2	-1.082123383	-1.20078526	30.946043	28.630411	24.222591	5.79661292	5.68487205	5.5378219	2143	2646	2195
9521	NM_001135650,NM_004280	EEF1E1	eukaryotic translation elongation	1.0511044	-1.200893023	23.330865	24.162268	18.127192	4.65812548	4.72725823	4.40541889	952	1337	975
79836	NM_001031855,NM_001289109	LONRF3	LON peptidase N-terminal domai	1.373133685	-1.200999228	2.765655	3.828773	2.195045	3.24517298	3.66081122	3.0111152	333	611	341
80775	NM_001105198,NM_001105199	TMEM177	transmembrane protein 177	1.607606412	-1.201090576	8.114375	12.85951	6.174097	3.66576097	4.30717831	3.42403181	459	986	470
23413	NM_001128826,NM_014286	NCS1	neuronal calcium sensor 1	1.357453466	-1.20116563	7.003221	9.522063	5.48214	5.13154304	5.56159734	4.8753398	1337	2425	1369
151050	NM_001307976,NM_152519	KANSL1L	KAT8 regulatory NSL complex su	1.19027021	-1.201203105	1.151278	1.413686	0.905189	2.66651408	2.8811152	2.44691815	210	334	215
23648	NM_001009955,NM_018070,NM	SSBP3	single stranded DNA binding prot	1.535441452	-1.201222729	4.108649	6.303937	3.22111	3.78784692	4.36970972	3.54415067	503	1032	515
140467	NM_018083	ZNF358	zinc finger protein 358	1.01771078	-1.201308237	15.181534	15.469094	11.880196	4.90131278	4.92580232	4.64636463	1134	1542	1161
51385	NM_016089	ZNF589	zinc finger protein 589	-1.263143569	-1.201333541	4.574473	3.624838	3.57809	4.00022383	3.68667221	3.75357377	589	623	603
91695	NR_002184	RRP7BP	ribosomal RNA processing 7 hom	1.30123938	-1.201334098	4.753737	6.188631	3.716639	3.55131131	3.90250283	3.31117052	421	732	431
84456	NM_001007102,NM_001346550	L3MBTL3	l(3)mbt-like 3 (Drosophila)	1.222224953	-1.201417212	1.993556	2.442112	1.560641	3.26442151	3.52644784	3.02952715	338	552	346
835	NM_001224,NM_032982,NM_03	CASP2	caspase 2	-1.373218567	-1.201462937	18.568984	13.998214	14.757842	6.27308523	5.8224506	6.01203572	2997	2916	3068
57665	NM_020905	RDH14	retinol dehydrogenase 14 (all-tra	1.619394945	-1.201530794	20.762558	33.654625	16.23262	5.05954162	5.73839964	4.80333552	1270	2748	1300
729288	NM_001145045	ZNF286B	zinc finger protein 286B	-1.074765019	-1.201554434	1.250324	1.165279	0.978401	2.90595399	2.81621366	2.67922768	255	317	261
28971	NM_001316957,NM_001316958	AAMDC	adipogenesis associated Mth938	-1.519088227	-1.201581044	15.49151	10.163949	12.293378	3.27204958	2.74415527	3.03682664	340	299	348
9718	NM_001037324,NM_001100120	ECE2	endothelin converting enzyme 2	1.132216753	-1.201632388	12.363224	13.97799	9.636692	3.6915876	3.8576751	3.44886901	468	708	479
55230	NM_018218	USP40	ubiquitin specific peptidase 40	1.083649183	-1.201803874	17.113817	18.567703	13.38161	6.56386469	6.67858806	6.30172856	3675	5321	3761
100129460	NR_036680	DPY19L1P1	DPY19L1 pseudogene 1	-1.740206115	-1.201824096	2.358181	1.353066	1.846042	2.4278825	1.81408935	2.21562831	172	132	176
7541	NM_001143823,NM_001243702	ZBTB14	zinc finger and BTB domain cont	-1.11630934	-1.201876214	5.489489	4.915166	4.551457	4.36902637	4.2183652	4.11772436	772	924	790
23309	NM_001297595,NM_001297597	SIN3B	SIN3 transcription regulator famil	-1.064623756	-1.201886645	25.389807	23.305849	19.110666	6.78357673	6.69407677	6.5209097	4286	5379	4386
11311	NM_001279353,NM_001279354	VPS45	vacuolar protein sorting 45 homo	1.071393132	-1.20195338	19.852746	18.965802	13.917743	5.61958513	5.71712177	5.3601012	1891	2707	1935
102465694	NR_106981	MIR7161	microRNA 7161	2.124920087	-1.20207179	0.23127	0.644608	0.148428	0.03628685	0.08023518	0.02957205	1	3	1
109623480	NR_145795	SNORD173	small nucleolar RNA, C/D box 17	-1.294074149	-1.20207179	0.12453	0.063448	0.173166	0.03628685	0.02724389	0.02957205	1	1	1
693122	NR_030398	MIR421	microRNA 421	5.505337187	-1.20207179	0.074581	1.659916	0.204888	0.03628685	0.20477631	0.02957205	1	8	1
10638	NM_006542	SPHAR	S-phase response (cyclin related)	3.477086927	-1.20207179	0.019518	0.078891	0.010151	0.03628685	0.13134883	0.02957205	1	5	1
100302275	NR_031630	MIR548L	microRNA 548l	2.124920087	-1.20207179	0.280106	0.60705	0.214012	0.03628685	0.08023518	0.02957205	1	3	1
100302258	NR_031715	MIR1469	microRNA 1469	2.124920087	-1.20207179	0.518045	1.024058	0.265275	0.03628685	0.08023518	0.02957205	1	3	1
100874273	NR_046798	TGFA-IT1	TGFA intronic transcript 1	11.59008797	-1.20207179	0.020006	0.647932	0.034191	0.03628685	0.40498891	0.02957205	1	17	1
102465512	NR_106910	MIR6851	microRNA 6851	2.801003507	-1.20207179	0.09665	1.092036	0.168366	0.03628685	0.10601836	0.02957205	1	4	1
102466731	NR_106827	MIR6769A	microRNA 6769a	2.124920087	-1.20207179	0.262571	0.776302	0.249413	0.03628685	0.08023518	0.02957205	1	3	1
105379511	NR_135512	LOC105379511	nuclear receptor corepressor 1 ps	4.153170347	-1.20207179	0.088984	0.364387	0.04712	0.03628685	0.15624221	0.02957205	1	6	1
677824	NR_002975	SNORA17B	small nucleolar RNA, H/ACA box	-1.294074149	-1.20207179	0.100085	0.073797	0.112661	0.03628685	0.02724389	0.02957205	1	1	1
728640	NR_027508	FAM133CP	family with sequence similarity 13	1.448836667	-1.20207179	0.009769	0.015101	0.000746	0.03628685	0.05398282	0.02957205	1	2	1
100302159	NR_031644_chr1,NR_031644_c	MIR548F3	microRNA 548f-3	1.448836667	-1.20207179	0.06325	0.337037	0.077184	0.03628685	0.05398282	0.02957205	1	2	1
100302280	NR_031602	MIR1237	microRNA 1237	-1.294074149	-1.20207179	0.017776	0.156973	0.105743	0.03628685	0.02724389	0.02957205	1	1	1
100500840	NR_037429	MIR3656	microRNA 3656	-1.294074149	-1.20207179	0.086974	0.166578	0.164866	0.03628685	0.02724389	0.02957205	1	1	1
106635539	NR_132787_chr2,NR_132787_c	SNORA105A	small nucleolar RNA, H/ACA box	2.124920087	-1.20207179	0.142212	0.35097	0.15227	0.03628685	0.08023518	0.02957205	1	3	1
8944	NR_000007	SNORD73A	small nucleolar RNA, C/D box 73	-1.294074149	-1.20207179	0.073077	0.047125	0.037724	0.03628685	0.02724389	0.02957205	1	1	1
26787	NR_002735	SNORD61	small nucleolar RNA, C/D box 61	1.448836667	-1.20207179	0.031934	0.327004	0.21417	0.03628685	0.05398282	0.02957205	1	2	1

26790	NR_002572	SNORD58B	small nucleolar RNA, C/D box 58	-1.294074149	-1.20207179	0.188922	0.150636	0.16174	0.03628685	0.02724389	0.02957205	1	1	1
391627	NR_046416	USP17L9P	ubiquitin specific peptidase 17-lik	2.124920087	-1.20207179	0.004927	0.026282	0.005019	0.03628685	0.08023518	0.02957205	1	3	1
406883	NR_029478	MIRLET7A3	microRNA let-7a-3	-1.294074149	-1.20207179	0.122511	0.145557	0.045221	0.03628685	0.02724389	0.02957205	1	1	1
406884	NR_029479	MIRLET7B	microRNA let-7b	-1.294074149	-1.20207179	0.168716	0.116182	0.123392	0.03628685	0.02724389	0.02957205	1	1	1
406975	NR_029584	MIR198	microRNA 198	-1.294074149	-1.20207179	0.182677	0.147122	0.160986	0.03628685	0.02724389	0.02957205	1	1	1
406991	NR_029493	MIR21	microRNA 21	-1.294074149	-1.20207179	0.161856	0.122402	0.147487	0.03628685	0.02724389	0.02957205	1	1	1
407004	NR_029494	MIR22	microRNA 22	-1.294074149	-1.20207179	0.129278	0.126181	0.125795	0.03628685	0.02724389	0.02957205	1	1	1
407025	NR_029518	MIR29B2	microRNA 29b-2	-1.294074149	-1.20207179	0.186776	0.147154	0.115222	0.03628685	0.02724389	0.02957205	1	1	1
407026	NR_029832	MIR29C	microRNA 29c	-1.294074149	-1.20207179	0.139451	0.158832	0.167564	0.03628685	0.02724389	0.02957205	1	1	1
407040	NR_029610	MIR34A	microRNA 34a	-1.294074149	-1.20207179	0.009419	0.028425	0.026352	0.03628685	0.02724389	0.02957205	1	1	1
677804	NR_002958	SNORA17A	small nucleolar RNA, H/ACA box	-1.294074149	-1.20207179	0.120841	0.09193	0.106312	0.03628685	0.02724389	0.02957205	1	1	1
677835	NR_002984	SNORA56	small nucleolar RNA, H/ACA box	-1.294074149	-1.20207179	0.068525	0.055135	0.085278	0.03628685	0.02724389	0.02957205	1	1	1
677838	NR_002987	SNORA61	small nucleolar RNA, H/ACA box	-1.294074149	-1.20207179	0.154259	0.119826	0.130685	0.03628685	0.02724389	0.02957205	1	1	1
693140	NR_030282	MIR555	microRNA 555	-1.294074149	-1.20207179	0.103429	0.132158	0.093269	0.03628685	0.02724389	0.02957205	1	1	1
693149	NR_030290	MIR564	microRNA 564	-1.294074149	-1.20207179	0.06163	0.028943	0.040757	0.03628685	0.02724389	0.02957205	1	1	1
693153	NR_030293	MIR568	microRNA 568	-1.294074149	-1.20207179	0.067482	0.034146	0.111735	0.03628685	0.02724389	0.02957205	1	1	1
693185	NR_030331	MIR600	microRNA 600	-1.294074149	-1.20207179	0.071741	0.128865	0.124699	0.03628685	0.02724389	0.02957205	1	1	1
693197	NR_030343	MIR612	microRNA 612	-1.294074149	-1.20207179	0.196371	0.145071	0.156935	0.03628685	0.02724389	0.02957205	1	1	1
693206	NR_030352	MIR621	microRNA 621	-1.294074149	-1.20207179	0.117562	0.130966	0.105995	0.03628685	0.02724389	0.02957205	1	1	1
693217	NR_030362	MIR632	microRNA 632	-1.294074149	-1.20207179	0.015906	0.017935	0.015718	0.03628685	0.02724389	0.02957205	1	1	1
693224	NR_030369	MIR639	microRNA 639	-1.294074149	-1.20207179	0.007292	0.000159	0.028272	0.03628685	0.02724389	0.02957205	1	1	1
693230	NR_030375	MIR645	microRNA 645	1.448836667	-1.20207179	0.256267	0.394345	0.202114	0.03628685	0.05398282	0.02957205	1	2	1
723790	NM_001040874,NM_001040874	HIST2H2AA4	histone cluster 2 H2A family men	1.448836667	-1.20207179	0.030784	0.038001	0.022367	0.03628685	0.05398282	0.02957205	1	2	1
724028	NR_030395	MIR658	microRNA 658	-1.294074149	-1.20207179	0.02288	0.023403	0.058762	0.03628685	0.02724389	0.02957205	1	1	1
768213	NR_030407	MIR671	microRNA 671	-1.294074149	-1.20207179	0.126456	0.112368	0.109127	0.03628685	0.02724389	0.02957205	1	1	1
100126311	NR_030599	MIR147B	microRNA 147b	-1.294074149	-1.20207179	0.229663	0.15986	0.175818	0.03628685	0.02724389	0.02957205	1	1	1
100126321	NR_030627	MIR922	microRNA 922	-1.294074149	-1.20207179	0.199624	0.164572	0.186411	0.03628685	0.02724389	0.02957205	1	1	1
100126325	NR_030632	MIR935	microRNA 935	-1.294074149	-1.20207179	0.072583	0.03359	0.061328	0.03628685	0.02724389	0.02957205	1	1	1
100302132	NR_031593	MIR1182	microRNA 1182	-1.294074149	-1.20207179	0.149272	0.135653	0.137206	0.03628685	0.02724389	0.02957205	1	1	1
100302160	NR_036050,NR_036050_dup1	MIR1233-1	microRNA 1233-1	-1.294074149	-1.20207179	0.0792	0.230385	0.192164	0.03628685	0.02724389	0.02957205	1	1	1
100302183	NR_031726	MIR1825	microRNA 1825	-1.294074149	-1.20207179	0.222401	0.154746	0.167637	0.03628685	0.02724389	0.02957205	1	1	1
100302197	NR_031706	MIR1306	microRNA 1306	-1.294074149	-1.20207179	0.145956	0.116531	0.140861	0.03628685	0.02724389	0.02957205	1	1	1
100302213	NR_031592	MIR1181	microRNA 1181	-1.294074149	-1.20207179	0.1413	0.10392	0.109901	0.03628685	0.02724389	0.02957205	1	1	1
100302224	NR_031747	MIR2110	microRNA 2110	-1.294074149	-1.20207179	0.002345	0.194815	0.089189	0.03628685	0.02724389	0.02957205	1	1	1
100313843	NR_031756	MIR711	microRNA 711	-1.294074149	-1.20207179	0.091534	0.083726	0.073284	0.03628685	0.02724389	0.02957205	1	1	1
100313887	NR_031754	MIR2277	microRNA 2277	-1.294074149	-1.20207179	0.077479	0.065261	0.058423	0.03628685	0.02724389	0.02957205	1	1	1
100313892	NR_031580	MIR761	microRNA 761	-1.294074149	-1.20207179	0.118328	0.092669	0.058847	0.03628685	0.02724389	0.02957205	1	1	1
100422845	NR_036261,NR_036261_dup1	MIR1233-2	microRNA 1233-2	-1.294074149	-1.20207179	0.0819	0.23587	0.202839	0.03628685	0.02724389	0.02957205	1	1	1
100422961	NR_036271,NR_036271_dup1	MIR4315-2	microRNA 4315-2	-1.294074149	-1.20207179	0.121316	0.086785	0.090814	0.03628685	0.02724389	0.02957205	1	1	1
100423004	NR_036199,NR_036199_dup1	MIR4315-1	microRNA 4315-1	-1.294074149	-1.20207179	0.119993	0.084248	0.090183	0.03628685	0.02724389	0.02957205	1	1	1
100463482	NM_001190487	MTRNR2L6	MT-RNR2-like 6	-1.294074149	-1.20207179	0.010445	0.001356	0.006526	0.03628685	0.02724389	0.02957205	1	1	1
100500808	NR_037481	MIR3917	microRNA 3917	-1.294074149	-1.20207179	0.043814	0.034368	0.033692	0.03628685	0.02724389	0.02957205	1	1	1
100500820	NR_037428	MIR3655	microRNA 3655	-1.294074149	-1.20207179	0.159134	0.120772	0.11931	0.03628685	0.02724389	0.02957205	1	1	1
100500827	NR_037408	MIR3614	microRNA 3614	-1.294074149	-1.20207179	0.167434	0.126858	0.087292	0.03628685	0.02724389	0.02957205	1	1	1
100500832	NR_037431	MIR3658	microRNA 3658	-1.294074149	-1.20207179	0.197118	0.147129	0.12167	0.03628685	0.02724389	0.02957205	1	1	1
100500847	NR_037409	MIR3615	microRNA 3615	-1.294074149	-1.20207179	0.074432	0.052943	0.088026	0.03628685	0.02724389	0.02957205	1	1	1
100500860	NR_037412	MIR3618	microRNA 3618	-1.294074149	-1.20207179	0.1405	0.10153	0.124901	0.03628685	0.02724389	0.02957205	1	1	1
100500914	NR_037404	MIR3610	microRNA 3610	-1.294074149	-1.20207179	0.075696	0.095443	0.093191	0.03628685	0.02724389	0.02957205	1	1	1
100616113	NR_039828	MIR4680	microRNA 4680	-1.294074149	-1.20207179	0.141114	0.09279	0.140286	0.03628685	0.02724389	0.02957205	1	1	1
100616119	NR_039846	MIR4697	microRNA 4697	-1.294074149	-1.20207179	0.116228	0.083768	0.119793	0.03628685	0.02724389	0.02957205	1	1	1
100616124	NR_039790	MIR4647	microRNA 4647	-1.294074149	-1.20207179	0.196107	0.145038	0.142713	0.03628685	0.02724389	0.02957205	1	1	1
100616139	NR_039895	MIR4741	microRNA 4741	-1.294074149	-1.20207179	0.070032	0.040718	0.096508	0.03628685	0.02724389	0.02957205	1	1	1
100616143	NR_039920	MIR4763	microRNA 4763	-1.294074149	-1.20207179	0.232651	0.109528	0.157391	0.03628685	0.02724389	0.02957205	1	1	1
100616149	NR_039737	MIR4512	microRNA 4512	2.801003507	-1.20207179	0.201836	0.937611	0.125939	0.03628685	0.10601836	0.02957205	1	4	1
100616193	NR_039658	MIR4453	microRNA 4453	-1.294074149	-1.20207179	0.112777	0.082621	0.134808	0.03628685	0.02724389	0.02957205	1	1	1
100616211	NR_039858	MIR4709	microRNA 4709	-1.294074149	-1.20207179	0.217861	0.162967	0.16865	0.03628685	0.02724389	0.02957205	1	1	1
100616248	NR_039877	MIR4724	microRNA 4724	-1.294074149	-1.20207179	0.056752	0.046883	0.102065	0.03628685	0.02724389	0.02957205	1	1	1
100616313	NR_039904	MIR4749	microRNA 4749	-1.294074149	-1.20207179	0.303608	0.014317	0.326054	0.03628685	0.02724389	0.02957205	1	1	1
100616329	NR_039849	MIR4700	microRNA 4700	-1.294074149	-1.20207179	0.131317	0.115752	0.120305	0.03628685	0.02724389	0.02957205	1	1	1
100616340	NR_039915	MIR4758	microRNA 4758	-1.294074149	-1.20207179	0.003231	0.002424	0.002412	0.03628685	0.02724389	0.02957205	1	1	1
100616358	NR_039964	MIR4800	microRNA 4800	-1.294074149	-1.20207179	0.150017	0.116597	0.15152	0.03628685	0.02724389	0.02957205	1	1	1
100616361	NR_039934	MIR4775	microRNA 4775	-1.294074149	-1.20207179	0.097133	0.098185	0.137716	0.03628685	0.02724389	0.02957205	1	1	1
100616367	NR_039677	MIR4467	microRNA 4467	-1.294074149	-1.20207179	0.172682	0.286492	0.021989	0.03628685	0.02724389	0.02957205	1	1	1
100616393	NR_039801	MIR4657	microRNA 4657	-1.294074149	-1.20207179	0.205625	0.167271	0.178167	0.03628685	0.02724389	0.02957205	1	1	1
100616400	NR_039851	MIR3198-2	microRNA 3198-2	-1.294074149	-1.20207179	0.169009	0.132946	0.143256	0.03628685	0.02724389	0.02957205	1	1	1
100616453	NR_039835	MIR4687	microRNA 4687	-1.294074149	-1.20207179	0.178078	0.114944	0.131649	0.03628685	0.02724389	0.02957205	1	1	1
100616483	NR_039906	MIR4751	microRNA 4751	-1.294074149	-1.20207179	0.14944	0.142159	0.124328	0.03628685	0.02724389	0.02957205	1	1	1
100616499	NR_039634,NR_039634_dup1	MIR4435-1	microRNA 4435-1	-1.294074149	-1.20207179	0.024781	0.025006	0.047417	0.03628685	0.02724389	0.02957205	1	1	1
100847012	NR_049800	MIR5004	microRNA 5004	-1.294074149	-1.20207179	0.026386	0.028819	0.020217	0.03628685	0.02724389	0.02957205	1	1	1
100847037	NR_049797	MIR5001	microRNA 5001	-1.294074149	-1.20207179	0.152936	0.102028	0.0784	0.03628685	0.02724389	0.02957205	1	1	1
100847070	NR_049828	MIR5196	microRNA 5196	-1.294074149	-1.20207179	0.039321	0.020437	0.021341	0.03628685	0.02724389	0.02957205	1	1	1
101060376	NM_001291459,NM_001291459	TBC1D3L	TBC1 domain family member 3L	-1.294074149	-1.20207179	0.004368	0.001574	0.001363	0.03628685	0.02724389	0.02957205	1	1	1
102464833	NR_106732	MIR6084	microRNA 6084	-1.294074149	-1.20207179	0.103945	0.091745	0.088247	0.03628685	0.02724389	0.02957205	1	1	1
102465133	NR_106740	MIR6125	microRNA 6125	-1.294074149	-1.20207179	0.027696	0.01688	0.032027	0.03628685	0.02724389	0.02957205	1	1	1
102465248	NR_106756	MIR6501	microRNA 6501	-1.294074149	-1.20207179	0.174664	0.119011	0.164624	0.03628685	0.02724389	0.02957205	1	1	1
102465429	NR_106775,NR_106775_dup1	MIR6511B1	microRNA 6511b-1	-1.294074149	-1.20207179	0.048177	0.018084	0.102384	0.03628685	0.02724389	0.02957205	1	1	1
102465457	NR_106820	MIR6762	microRNA 6762	-1.294074149	-1.20207179	0.109934	0.055289	0.205958	0.03628685	0.02724389	0.02957205	1	1	1
102465461	NR_106828,NR_106828_dup1,N	MIR6770-1	microRNA 6770-1	-1.294074149	-1.20207179	0.051329	0.283451	0.00566	0.03628685	0.02724389	0.02957205	1	1	1
102465483	NR_106863	MIR6805	microRNA 6805	-1.294074149	-1.20207179	0.023117	0.028591	0.037504	0.03628685	0.02724389	0.02957205	1	1	1
102465495	NR_106882	MIR6824	microRNA 6824	-1.294074149	-1.20207179	0.173915	0.088016	0.109463	0.03628685	0.02724389	0.02957205	1	1	1
102465500	NR_106891	MIR6833	microRNA 6833	-1.294074149	-1.20207179	0.259023	0.198848	0.20277	0.03628685	0.02724389	0.02957205	1	1	1
102465504	NR_106897	MIR6838	microRNA 6838	-1.294074149	-1.20207179	0.351192	0.223911	0.180131	0.03628685	0.02724389	0.02957205	1	1	1
102465519	NR_106921	MIR6861	microRNA 6861	-1.294074149	-1.20207179	0.257305	0.012311	0.014265	0.03628685	0.02724389	0.02957205	1	1	1
102465526	NR_106932	MIR6872	microRNA 6872	-1.294074149	-1.20207179	0.154299	0.126867	0.163082	0.03628685	0.02724389	0.02957205	1	1	1
102465803	NR_106993	MIR7706	microRNA 7706	-1.294074149	-1.20207179	0.025405	0.01738	0.056927	0.03628685	0.02724389	0.02957205	1	1	1
102465834	NR_106998	MIR7844	microRNA 7844	-1.294074149	-1.20207179	0.166666	0.097875	0.113302	0.03628685	0.02724389	0.02957205	1	1	1
102465907	NR_107058,NR_107058_dup1	MIR6862-2	microRNA 6862-2	-1.294074149	-1.20207179	0.072949	0.107296	0.194228	0.03628685	0.02724389	0.02957205	1	1	1
102465908	NR_107060,NR_107060_dup1,N	MIR6770-2	microRNA 6770-2	-1.294074149	-1.20207179	0.052939	0.295124	0.005496	0.03628685	0.02724389	0.02957205	1	1	1
102466259	NR_107061,NR_107061_dup1,N	MIR6770-3	microRNA 6770-3	-1.294074149	-1.20207179	0.054599	0.289229	0.005672	0.03628685	0.02724389	0.02957205	1	1	1
102466738	NR_106856	MIR6798	microRNA 6798	1.448836667	-1.20207179	0.0683	0.392979	0.199873	0.03628685	0.05398282	0.02957205	1	2	1
102466741	NR_106871	MIR6813	microRNA 6813	3.477086927	-1.20207179	0.425537	1.445169	0.236778	0.03628685	0.13134883	0.02957205	1	5	1
102466854	NR_106992	MIR7705	microRNA 7705	-1.294074149	-1.20207179	0.211206	0.173924	0.172425	0.03628685	0.02724389	0.02957205	1	1	1
102466877	NR_107039	MIR8072	microRNA 8072	-1.294074149	-1.20207179	0.062905	0.034267	0.048769	0.03628685	0.02724389	0.02957205	1	1	1
103504729	NR_128709,NR_128709_dup1,N	MIR3179-4	microRNA 3179-4	-1.294074149	-1.20207179	0.027054	0.201902	0.033919	0.03628685	0.02724389	0.02957205	1	1	1
103504732	NR_128719,NR_128719_dup1,N	MIR941-5	microRNA 941-5	-1.294074149	-1.20207179	0.050365	0.074128	0.148301	0.03628685	0.02724389	0.02957205	1	1	1
106633814	NR_132768	SNORA86	small nucleolar RNA, H/ACA box	-1.294074149	-1.20207179	0.172327	0.130953	0.125836	0.03628685	0.02724389	0.02957205	1	1	1
106635532	NR_132782	SNORA100	small nucleolar RNA, H/ACA box	-1.294074149	-1.20207179	0.113246	0.097422	0.091166	0.03628685	0.02724389	0.02957205	1	1	1
109136578	NR_144569	MASCRNA	MALAT1-associated small cytopl	-1.294074149	-1.20207179	0.154396	0.099911	0.109796	0.03628685	0.02724389	0.02957205	1	1	1
109616966	NR_145985	SNORA31B	small nucleolar RNA, H/ACA box	-1.294074149	-1.20207179	0.063574	0.009897	0.084816	0.03628685	0.02724389	0.02957205	1	1	1

109616967	NR_145723	SNORA40B	small nucleolar RNA, H/ACA box	1.448836667	-1.20207179	0.149747	0.288081	0.077306	0.03628685	0.05398282	0.02957205	1	2	1
1975	NM_001300821,NM_001330654	EIF4B	eukaryotic translation initiation fa	-1.391214605	-1.20210828	83.837421	60.346794	65.567497	8.37602242	7.90137067	8.11133046	13004	12489	13305
100507178	NR_037868	SLFNL1-AS1	SLFNL1 antisense RNA 1	1.496463332	-1.202191368	0.709459	1.06906	0.557919	2.11660268	2.58366954	1.91649468	131	262	134
898	NM_001238,NM_001322259,NM	CCNE1	cyclin E1	-1.068021112	-1.202472259	16.755827	15.607985	13.150944	5.01136367	4.91945464	4.75436273	1227	1535	1255
126789	NM_001346116,NM_153339,NR	PUSL1	pseudouridylate synthase-like 1	1.289151907	-1.202495159	15.170374	19.524969	11.949372	4.32203244	4.67222953	4.0704925	746	1285	763
7137	NM_000363	TNNI3	troponin I3, cardiac type	-2.139053085	-1.202598953	4.130193	1.93322	3.213051	2.13345087	1.36863803	1.93223726	133	83	136
92595	NM_001172679,NM_033410	ZNF764	zinc finger protein 764	1.189642299	-1.202602056	4.126436	4.90802	3.222395	3.61267788	3.8443336	3.37014999	441	701	451
6339	NM_001130413,NR_037668	SCNN1D	sodium channel epithelial 1 delta	-1.15866564	-1.202602279	4.595507	3.932038	3.626435	3.85541331	3.65863507	3.60925088	529	610	541
7301	NM_001330264,NM_006293	TYRO3	TYRO3 protein tyrosine kinase	1.208909333	-1.202770102	5.251386	6.360276	4.105441	4.41453771	4.67653783	4.16179054	798	1289	816
1368	NM_001005502,NM_001874,NM	CPM	carboxypeptidase M	-1.017260954	-1.202798288	0.521569	0.515198	0.409762	2.14180175	2.12272838	1.9400446	134	176	137
79156	NM_024310	PLEKHF1	pleckstrin homology and FYVE d	-2.025586936	-1.202898578	2.690915	1.329512	2.101602	2.47491059	1.70021226	2.25996639	179	118	183
26580	NM_001122955,NM_001130702	BSCL2	BSCL2, seipin lipid droplet bioger	-1.429523746	-1.202929403	34.74313	23.986316	30.963786	6.03774712	5.53158861	5.77563191	2540	2374	2597
50485	NM_001127207,NM_014140	SMARCAL1	SWI/SNF related, matrix associat	1.22856606	-1.202956645	8.324836	9.836595	6.507198	4.70114026	4.98778875	4.44573627	982	1612	1004
26146	NM_001139490,NM_015650	TRAF3IP1	TRAF3 interacting protein 1	1.475568677	-1.202961547	4.399356	6.503556	3.440412	4.17517665	4.71054878	3.92465818	670	1321	685
10270	NM_005858	AKAP8	A-kinase anchoring protein 8	-1.124282548	-1.203024554	20.141029	17.936176	15.731631	6.1392481	5.97277811	5.87671972	2728	3242	2789
57622	NM_020862	LRFN1	leucine rich repeat and fibronecti	1.956638319	-1.203096102	2.251313	4.414039	1.75927	2.97768275	3.85387581	2.74755257	270	706	276
55813	NM_018428	UTP6	UTP6, small subunit processome	1.084350456	-1.203221371	41.085922	44.605762	32.090233	6.38591889	6.50141107	6.12250838	3244	4700	3316
92181	NM_152277	UBTD2	ubiquitin domain containing 2	1.220862632	-1.20327287	5.87891	7.186841	4.590904	4.19135823	4.46494141	3.94031242	678	1106	693
54840	NM_001195248,NM_001195249	APTX	aprataxin	-1.071734121	-1.20328298	11.870678	11.340304	9.338494	4.58651747	4.49085976	4.33166223	904	1127	924
10921	NM_001286625,NM_001286626	RNPS1	RNA binding protein with serine r	1.154156043	-1.203296859	90.714009	104.428154	70.905441	7.55895054	7.76476938	7.29350803	7364	11356	7527
112703	NM_001308429,NM_138411	FAM71E1	family with sequence similarity 71	1.370929573	-1.203379223	2.851393	3.868749	2.191452	2.16656821	2.53229878	1.96321628	137	251	140
55256	NM_001306077,NM_018269	ADI1	acireductone dioxygenase 1	-1.113384241	-1.203385109	40.804209	39.729777	36.858043	6.64834823	6.49502201	6.38416363	3899	4679	3985
8503	NM_001114172,NM_001303428	PIK3R3	phosphoinositide-3-kinase regula	1.646118998	-1.203456107	2.368752	3.821749	1.80303	3.78251604	4.45995136	3.53644652	501	1102	512
152485	NM_001306215,NM_178835	ZNF827	zinc finger protein 827	1.470032919	-1.203473262	4.123204	6.070788	3.219385	5.01364054	5.55517746	4.75546927	1229	2414	1256
94162	NR_001456	SNORD38A	small nucleolar RNA, C/D box 38	1.034989251	-1.203726397	17.223173	17.936945	13.485319	1.13562816	1.16293478	0.99569924	47	65	48
113510	NM_001297755,NM_001297756	HELQ	helicase, POLQ-like	1.137843767	-1.203798599	6.03394	6.482262	4.524006	4.3072593	4.48473082	4.05439856	738	1122	754
651302	NR_103448	ZNF192P1	zinc finger protein 192 pseudoge	-1.064290754	-1.204018599	1.489419	1.401142	1.156098	1.76310734	1.70021226	1.57922529	94	118	96
115098	NM_001136203,NM_138442	CCDC124	coiled-coil domain containing 124	-1.178477159	-1.204086462	73.457558	62.543173	57.541855	6.17427094	5.94089851	5.91039165	2796	3170	2856
26229	NM_001288721,NM_001288722	B3GAT3	beta-1,3-glucuronyltransferase 3	1.073970084	-1.204116047	41.773447	44.422241	33.102429	6.12773269	6.22926863	5.86395147	2706	3883	2764
55072	NM_001310332,NM_017999	RNF31	ring finger protein 31	1.195835034	-1.20437445	17.366171	20.972612	13.541991	5.93157437	6.18572723	5.66809934	2357	3766	2407
80789	NM_030628	INTS5	integrator complex subunit 5	1.40066389	-1.204517656	3.778417	5.304256	2.950963	3.71136013	4.16570385	3.46519272	475	889	485
9112	NM_001203258,NM_004689	MTA1	metastasis associated 1	1.120626939	-1.204608946	61.843307	69.337767	48.051209	7.41479159	7.57818978	7.14795066	6660	9972	6800
55739	NM_001242881,NM_001242882	NAXD	NAD(P)HX dehydratase	-1.134796378	-1.204669609	19.420866	16.889547	14.924317	5.66736669	5.4887443	5.40451103	1956	2303	1997
10153	NM_005760	CEBPZ	CCAAT/enhancer binding protein	1.151309484	-1.204675177	29.253973	33.718872	22.819859	6.5816223	6.78292241	6.31604903	3721	5724	3799
26017	NM_014077	FAM32A	family with sequence similarity 32	-1.136408897	-1.204683981	52.589138	46.322258	41.010822	6.25491539	6.07300096	5.99011117	2959	3479	3021
51021	NM_016065	MRPS16	mitochondrial ribosomal protein S	-1.684116	-1.204720945	34.645935	20.593807	27.02519	6.48974676	5.74866631	6.22432111	3489	2768	3562
284349	NM_001297752,NM_181845	ZNF283	zinc finger protein 283	-1.461922399	-1.204908716	3.008147	2.058381	2.342449	3.06808971	2.597368	2.83389297	290	265	296
9853	NM_001135999,NM_001330740	RUSC2	RUN and SH3 domain containing	-1.172848556	-1.20492862	7.357317	4.411418	3.754053	4.74428126	4.52351345	4.48629114	1013	1154	1034
23587	NM_015362,NM_203413,NM_20	ELP5	elongator acetyltransferase comp	1.123183388	-1.205047315	10.741156	11.5189	8.330165	4.19336823	4.35231081	3.94031242	679	1019	693
317762	NM_001144995	CCDC85C	coiled-coil domain containing 85C	-1.095856633	-1.205078662	19.461473	17.777702	15.176983	6.76105391	6.63026582	6.49464405	4219	5144	4306
4255	NM_002412	MGMT	O-6-methylguanine-DNA methylt	-1.123363312	-1.205265955	13.980987	12.460859	10.907345	4.29981562	4.14097459	4.04537929	734	873	749
2639	NM_000159,NM_013976,NR_10	GCDH	glutaryl-CoA dehydrogenase	-1.135289887	-1.205289408	8.08262	7.14338	6.284897	3.99792567	3.82699692	3.74691298	588	692	600
150962	NM_001322123,NM_001322124	PUS10	pseudouridylate synthase 10	-1.6731628	-1.205299854	0.67999	0.401165	0.52468	1.81624731	1.32539385	1.62837236	99	79	101
1161	NM_000082,NM_001007233,NM	ERCC8	ERCC excision repair 8, CSA ubi	1.033062255	-1.205330406	9.067271	8.499876	7.099009	4.00022383	4.04427071	3.74913666	589	813	601
55818	NM_001146688,NM_018433	KDM3A	lysine demethylase 3A	1.470964959	-1.205400749	15.771912	23.231983	12.345602	6.25009557	6.80079358	5.98445961	2949	5796	3009
7174	NM_001330588,NM_003291	TPP2	tripeptidyl peptidase 2	-1.108334985	-1.205569343	12.209856	11.035845	9.5209	6.05664057	5.91058637	5.79136195	2574	3103	2626
51296	NM_016582,NR_027391	SLC15A3	solute carrier family 15 member 3	1.172233161	-1.2055745	9.150327	10.671774	6.971643	4.22318689	4.44108339	3.96921401	694	1087	708
463	NM_001164766,NM_006885	ZFHX3	zinc finger homeobox 3	-1.079959637	-1.205672173	1.620608	1.501565	1.262928	4.71938895	4.61277196	4.46073909	995	1231	1015
1615	NM_001293312,NM_001349	DARS	aspartyl-tRNA synthetase	1.421375948	-1.205992581	36.023854	51.087418	28.041503	6.79916906	7.30262219	6.53160649	4333	8229	4419
5600	NM_002751,NR_110887	MAPK11	mitogen-activated protein kinase	1.00086144	-1.206151051	13.939646	13.86571	10.635993	5.05843928	5.05964438	4.79690107	1269	1697	1294
219736	NM_001130159,NM_001130160	STOX1	storkhead box 1	-2.769016786	-1.206192518	0.395237	0.209769	0.332303	1.21691895	0.56226574	1.06873498	52	25	53
84678	NM_001005366,NM_032590	KDM2B	lysine demethylase 2B	1.13600476	-1.206410683	6.07409	6.918381	4.734474	5.01704913	5.19568855	4.75546927	1232	1870	1256
22856	NM_014918	CHSY1	chondroitin sulfate synthase 1	1.742341319	-1.206479594	7.991158	13.936919	6.224373	5.18605837	5.97014822	4.92339112	1390	3236	1417
55795	NM_001127202,NM_001127203	PCID2	PCI domain containing 2	1.202325519	-1.207331545	17.425835	21.082282	12.501917	5.18605837	5.44521997	4.92240621	1390	2233	1416
23760	NM_001284277,NM_001284278	PITPNB	phosphatidylinositol transfer prot	1.055896924	-1.207332975	21.264451	22.612356	16.514593	5.96018767	6.03743278	5.69314689	2405	3393	2450
200845	NM_001128214,NM_153331	KCTD6	potassium channel tetramerizatic	1.329612492	-1.207340173	3.193263	4.391421	2.565667	2.69516719	3.05002095	2.46867149	215	382	219
2621	NM_000820	GAS6	growth arrest specific 6	-2.140162732	-1.207429369	19.389292	9.066846	15.088608	5.61058821	4.54605682	5.3447438	1879	1173	1914
116842	NM_052971	LEAP2	liver enriched antimicrobial peptic	1.509485477	-1.207461329	2.011363	3.063482	1.564194	1.26358189	1.63971536	1.11084509	55	111	56
51537	NM_001003704,NM_016498	MTFP1	mitochondrial fission process 1	1.851224415	-1.207510421	48.356992	85.516776	35.57973	5.83959881	6.7164795	5.57276666	2209	5464	2250
51079	NM_015965	NDUFA13	NADH:ubiquinone oxidoreductas	-1.17073581	-1.207775148	255.234039	218.251816	198.587143	7.08418355	6.85857721	6.81403278	5288	6035	5385
80153	NM_001142443,NM_001142444	EDC3	enhancer of mRNA decapping 3	1.098876672	-1.207880271	9.80717	11.28195	7.970535	5.27415876	5.40683891	5.00938866	1480	2173	1507
64601	NM_022575,NM_080413	VPS16	VPS16, CORVET/HOPS core su	1.135431535	-1.2079102	21.110267	23.939276	16.202835	5.830587	6.01080901	5.5633199	2195	3330	2235
81926	NM_001130111,NM_031213	ABHD17A	abhydrolase domain containing 1	1.052862199	-1.207939848	25.731442	27.111408	19.995951	5.32912486	5.40164341	5.06399834	1539	2165	1567
55571	NM_017546	CNOT11	CCR4-NOT transcription complex	-1.131579609	-1.208192058	36.378361	32.182381	28.292002	6.49505265	6.31881185	6.22551943	3502	4135	3565
9984	NM_005131	THOC1	THO complex 1	1.002889275	-1.208234666	43.292416	43.468487	33.67094	6.49790161	6.50201808	6.22831165	3509	4702	3572
85462	NM_033393	FHDC1	FH2 domain containing 1	-1.23325209	-1.208273507	1.355712	1.099461	1.0546	3.25675288	2.98897501	3.01481644	336	364	342
7745	NM_001278119,NM_001278121	ZKSCAN8	zinc finger with KRAB and SCAN	1.170065031	-1.208341222	6.085511	7.164914	4.761121	5.49882028	5.72077686	5.23240543	1736	2714	1767
8886	NM_006773	DDX18	DEAD-box helicase 18	1.393823881	-1.208423859	28.108213	39.225147	21.859823	6.70121777	7.176354	6.43096978	4046	7535	4118
22943	NM_012242	DKK1	dickkopf WNT signaling pathway	-1.30344808	-1.208769154	157.281631	120.796318	122.268188	8.1158888	7.73512861	7.84343225	10852	11124	11042
6838	NM_001278942,NM_006753,NR	SURF6	surfeit 6	1.228708502	-1.208797945	29.616288	36.433138	23.027781	6.08831031	6.38151159	5.81914509	2632	4321	2678
23406	NM_021149	COTL1	coactosin like F-actin binding pro	1.316533963	-1.2092452	346.461456	456.641815	269.239777	9.29408666	9.69028026	9.02045926	24607	43285	25028
116285	NM_001318890,NM_052956,NR	ACSM1	acyl-CoA synthetase medium-cha	-1.381255649	-1.209299302	0.784784	0.539168	0.548081	1.33814853	1.07428646	1.17840727	60	58	61
284004	NM_001330542,NM_173620	HEXDC	hexosaminidase D	-1.28664366	-1.209374975	13.242489	10.291061	10.303657	4.88274916	4.53304836	4.61866105	1119	1162	1138
9784	NM_001267059,NM_001267060	SNX17	sorting nexin 17	1.387752181	-1.20955776	23.758563	33.072296	18.415406	5.87007592	6.3359362	5.60074135	2257	4185	2295
1964	NM_001412	EIF1AX	eukaryotic translation initiation fa	1.156874537	-1.209614191	29.17359	33.7892	22.665123	6.98730058	7.19599448	6.71512831	4942	7639	5025
2717	NM_000169	GLA	galactosidase alpha	-1.359230317	-1.20976254	53.119705	39.130154	41.270439	6.2163926	5.78053688	5.94572127	2880	2831	2928
116844	NM_052972	LRG1	leucine rich alpha-2-glycoprotein	-1.060108249	-1.209818736	3.545844	3.354888	2.755307	2.84076697	2.76857683	2.6075054	242	305	246
84455	NM_032437	EFCAB7	EF-hand calcium binding domain	1.028577785	-1.210019778	3.978616	4.09732	3.090411	3.3677101	3.40448323	3.12165574	366	503	372
56339	NM_019852	METTL3	methyltransferase like 3	1.02755837	-1.210032182	51.922596	53.421814	40.324306	6.68987259	6.72871914	6.41775083	4014	5511	4080
126432	NM_001195833,NM_198445	RINL	Ras and Rab interactor like	-1.552548496	-1.210152056	4.716538	3.036837	3.664302	3.91265481	3.3298614	3.65740237	552	475	561
3183	NM_001077442,NM_001077443	HNRNPC	heterogeneous nuclear ribonucle	-1.375158806	-1.210213137	282.151279	205.457326	219.34417	9.79065475	9.33166582	9.515735	34733	33747	35299
643707	NM_001267536	GOLGA6L4	golgin A6 family-like 4	-1.352343617	-1.210219244	1.413398	1.045983	1.099081	2.86618866	2.49862948	2.63181117	247	244	251
55676	NM_001193513,NM_001193514	SLC30A6	solute carrier family 30 member 6	1.199141086	-1.210536899	8.455781	9.973934	6.436777	5.40480367	5.66115412	5.13628808	1624	2602	1650
51096	NM_016001	UTP18	UTP18, small subunit processom	1.001981486	-1.210592149	40.853378	40.991947	31.721243	6.24719593	6.25001433	5.97546577	2943	3940	2990
5425	NM_001127218,NM_001256879	POLD2	DNA polymerase delta 2, access	-1.069497621	-1.210740787	44.370974	41.479723	34.70437	6.19648797	6.10091747	5.92472586	2840	3548	2885
102724532	NR_135480	LOC102724532	uncharacterized LOC102724532	1.093711447	-1.210859375	3.235562	3.537349	2.476622	1.40904982	1.49109792	1.24294651	65	95	66
3187	NM_001257293,NM_005520	HNRNPH1	heterogeneous nuclear ribonucle	-1.01561932	-1.211093666	334.169403	329.128994	259.235356	9.53345247	9.51112305	9.25755174	29055	38224	29507
100533975	NR_037929,NR_037930	SLMO2-ATP5E	SLMO2-ATP5E readthrough	-1.896840023	-1.211125907	22.982153	12.216537	17.936732	4.58498718	3.71417485	4.32123795	903	636	917
79039	NM_001111322,NM_024072	DDX54	DEAD-box helicase 54	-1.080325034	-1.211421595	21.111835	19.562063	16.378173	6.50843446	6.39823789	6.23507035	3535	4372	3589
5130	NM_001312673,NM_005017	PCYT1A	phosphate cytidylyltransferase 1,	-1.086127702	-1.211439418	27.296971	25.16925	21.170219	5.893137	5.77602682	5.62152376	2294	2822	2329
10625	NM_006469	IVNS1ABP	influenza virus NS1A binding pro	-1.173386953	-1.211478945	34.783714	29.675758	26.982513	7.16142673	6.93248943	6.88678101	5581	6355	5666
401115	NM_001141936,NM_001168243	C4orf48	chromosome 4 open reading fran	-1.203828431	-1.211523633	31.667171	26.358742	24.50716	3.85287228	3.60539766	3.59695765	528	586	536
6832	NM_001301683,NM_001323584	SUPV3L1	Suv3 like RNA helicase	-1.420367487	-1.211592889	17.903562	12.644899	13.970463	5.48493837	4.99211609	5.21481417	1719	1617	1745
113189	NM_130468	CHST14	carbohydrate sulfotransferase 14	1.115989727	-1.211958699	16.066351	17.944914	12.455951	5.14510661	5.29919828	4.87635737	1350	2013	1370
79902	NM_001303276,NM_001330472	NUP85	nucleoporin 85	-1.205337229	-1.212111151	69.82783	57.601032	54.50482	7.32551408	7.05791793	7.04989321	6258	6937	6350
254225	NM_001098638	RNF169	ring finger protein 169	-1.073546965	-1.212118911	9.103866	8.490534	7.057871	6.13663902	6.03575691	5.86343838	2723	3389	2763
7543	NM_001178084,NM_001178085	ZFX	zinc finger protein, X-linked	1.318329312	-1.212463713	4.915115	6.69244	3.978675	5.25407798	5.64365646	4.98412333	1459	2570	1480
374650	NR_003246	GOLGA6L5P	golgin A6 family-like 5, pseudoge	-1.428335468	-1.212726368	2.362589	1.658432	1.831258	3.76640418	3.29665996	3.51046477	495	463	502
22869	NM_001314059,NM_001314060	ZNF510	zinc finger protein 510	1.135693701	-1.212786856	4.593054	5.367204	3.677285	4.98491804	5.16305312	4.71622624	1204	1827	1221

28978	NM_014051	TMEM14A	transmembrane protein 14A	1.034178501	-1.213029229	20.388573	21.123121	15.792473	4.4024252	4.44866021	4.13822997	791	1093	802
79758	NM_001031719,NM_001270424	DHRS12	dehydrogenase/reductase 12	-1.595265485	-1.213044106	5.675509	3.519921	4.381123	3.06370142	2.48886356	2.82126674	289	242	293
65985	NM_001319839,NM_001319840	AACS	acetoacetyl-CoA synthetase	1.291040854	-1.21319287	11.536076	14.866704	8.855649	5.24635403	5.60631865	4.97560227	1451	2503	1471
51542	NM_001005739,NM_016516	VPS54	VPS54, GARP complex subunit	-1.069519049	-1.213585429	11.221183	10.560975	8.700294	5.47670971	5.38199194	5.20431761	1709	2135	1732
126917	NM_001136265	IFFO2	intermediate filament family orpha	1.299395071	-1.213613421	3.007569	3.91329	2.328106	4.1772093	4.53660777	3.91478732	671	1165	680
57336	NM_001346167,NM_001346168	ZNF287	zinc finger protein 287	1.392503816	-1.213665144	0.511291	0.719614	0.39338	2.27683528	2.66830768	2.05953665	151	281	153
2040	NM_001270526,NM_001270527	STOM	stomatin	-7.955401494	-1.213764502	1.855275	0.2237	1.4318	2.76169834	0.7861039	2.52685524	227	38	230
106635533	NR_132783	SNORA103	small nucleolar RNA, H/ACA box	1.132065794	-1.213992981	23.450642	26.822374	18.26552	1.57857476	1.70021226	1.39836968	78	118	79
3508	NM_002180	IGHMBP2	immunoglobulin mu binding prote	-1.077762578	-1.214134606	9.162873	8.508421	7.089844	5.18100252	5.07604389	4.90954071	1385	1717	1403
100505761	NR_038937,NR_038938	RPARP-AS1	RPARP antisense RNA 1	-1.160932141	-1.214171157	8.335151	7.868771	6.476283	3.68016614	3.48283341	3.42403181	464	534	470
23041	NM_001278469,NM_001278470	MON2	MON2 homolog, regulator of end	-1.174489625	-1.214243072	6.250238	5.320736	4.832174	6.00264414	5.77452033	5.72738578	2478	2819	2510
84698	NM_001286547,NM_001286548	CAPS2	calcyphosine 2	-1.398701904	-1.214272991	3.150521	2.119356	2.425631	3.16140171	2.74004451	2.91535666	312	298	316
6194	NM_001010	RPS6	ribosomal protein S6	-1.126009664	-1.214449896	1192.957886	1060.636719	923.095886	9.91271616	9.74168503	9.63273322	37803	44857	38285
57580	NM_020820	PREX1	phosphatidylinositol-3,4,5-trispho	-1.282941088	-1.214753994	0.954259	0.742846	0.736194	2.84076697	2.53704518	2.60259472	242	252	245
100303755	NM_001164811	PET117	PET117 homolog	1.194304234	-1.214789137	18.143677	21.715206	14.034154	4.42483953	4.67007054	4.15844821	804	1283	814
55839	NM_001100624,NM_001100625	CENPN	centromere protein N	-1.005726678	-1.214799389	24.573251	22.384387	18.453523	6.00950526	6.00139486	5.73357571	2490	3308	2521
84142	NM_001345962,NM_139076	ABRAXAS1	abraxas 1, BRCA1 A complex su	-1.107715216	-1.214891607	2.999379	2.719242	2.320222	3.21383096	3.08285145	2.96594348	325	392	329
23135	NM_001080424,NM_001348716	KDM6B	lysine demethylase 6B	1.589711286	-1.215043468	7.07852	11.262382	5.452243	5.54756029	6.20486858	5.27315144	1797	3817	1819
2139	NM_005244,NM_172110	EYA2	EYA transcriptional coactivator ar	5.023740936	-1.215104974	0.019364	0.096032	0.014795	0.07168335	0.3412894	0.0585501	2	14	2
26786	NR_002914,NR_002914_dup1	SNORD62A	small nucleolar RNA, C/D box 62	1.471633007	-1.215104974	0.452913	0.83868	0.444113	0.07168335	0.10601836	0.0585501	2	4	2
30813	NM_001256271,NM_001256272	VSX1	visual system homeobox 1	21.00822662	-1.215104974	0.023718	0.495848	0.018122	0.07168335	1.08728891	0.0585501	2	59	2
57576	NM_001122819,NM_001287212	KIF17	kinesin family member 17	4.313319351	-1.215104974	0.011757	0.055411	0.010075	0.07168335	0.29720487	0.0585501	2	12	2
102465524	NR_106929	MIR6869	microRNA 6869	2.892476179	-1.215104974	0.655912	2.25691	0.395807	0.07168335	0.20477631	0.0585501	2	8	2
109617012	NR_145755	SNORD53B	small nucleolar RNA, C/D box 53	-1.313695475	-1.215104974	0.564536	0.455331	0.398344	0.07168335	0.05398282	0.0585501	2	2	2
80094	NR_027715	BIN3-IT1	BIN3 intronic transcript 1	2.182054593	-1.215104974	0.02817	0.063848	0.021738	0.07168335	0.15624221	0.0585501	2	6	2
100462953	NR_051980	MINOS1P1	mitochondrial inner membrane or	2.182054593	-1.215104974	0.022958	0.050925	0.017541	0.07168335	0.15624221	0.0585501	2	6	2
100302221	NR_031623	MIR1291	microRNA 1291	1.471633007	-1.215104974	0.588482	0.765557	0.38463	0.07168335	0.10601836	0.0585501	2	4	2
100507589	NR_038913	NAGPA-AS1	NAGPA antisense RNA 1	2.182054593	-1.215104974	0.042298	0.079231	0.032317	0.07168335	0.15624221	0.0585501	2	6	2
100847028	NR_049852	MIR5587	microRNA 5587	1.8268438	-1.215104974	0.615789	1.640487	0.522764	0.07168335	0.13134883	0.0585501	2	5	2
3020	NM_002107	H3F3A	H3 histone family member 3A	1.471633007	-1.215104974	0.036017	0.060655	0.03192	0.07168335	0.10601836	0.0585501	2	4	2
692200	NR_003066	SNORD103C	small nucleolar RNA, C/D box 10	-2.463050374	-1.215104974	0.452426	0.1889	0.456498	0.07168335	0.02724389	0.0585501	2	1	2
693226	NR_030371	MIR641	microRNA 641	-1.313695475	-1.215104974	0.306117	0.245045	0.325841	0.07168335	0.05398282	0.0585501	2	2	2
102466912	NR_106931	MIR6871	microRNA 6871	2.182054593	-1.215104974	0.823322	1.867976	0.537167	0.07168335	0.15624221	0.0585501	2	6	2
162515	NM_153357	SLC16A11	solute carrier family 16 member 1	1.8268438	-1.215104974	0.020185	0.043487	0.01172	0.07168335	0.13134883	0.0585501	2	5	2
653505	NM_001143883,NM_001143883	PPIAL4A	peptidylprolyl isomerase A like 4A	2.182054593	-1.215104974	0.050716	0.134328	0.02632	0.07168335	0.15624221	0.0585501	2	6	2
101954268	NR_125392	RNVU1-20	RNA, variant U1 small nuclear 20	-1.313695475	-1.215104974	0.286037	0.22533	0.182727	0.07168335	0.05398282	0.0585501	2	2	2
406891	NR_029661	MIRLET7I	microRNA let-7i	3.247686972	-1.215104974	0.437871	2.058586	0.336907	0.07168335	0.2284445	0.0585501	2	9	2
100287327	NM_001256857	USP17L17	ubiquitin specific peptidase 17-lik	1.116422214	-1.215104974	0.018195	0.036786	0.023542	0.07168335	0.08023518	0.0585501	2	3	2
100287513	NM_001256863,NM_001256863	USP17L22	ubiquitin specific peptidase 17-lik	-1.313695475	-1.215104974	0.018723	0.018136	0.017567	0.07168335	0.05398282	0.0585501	2	2	2
100616237	NR_039783	MIR4640	microRNA 4640	1.116422214	-1.215104974	0.411558	0.463625	0.36942	0.07168335	0.08023518	0.0585501	2	3	2
101060389	NM_001291465,NM_001291465	TBC1D3D	TBC1 domain family member 3D	-2.463050374	-1.215104974	0.021647	0.008739	0.009691	0.07168335	0.02724389	0.0585501	2	1	2
102466719	NR_106773	MIR6716	microRNA 6716	-2.463050374	-1.215104974	0.480265	0.077474	0.357975	0.07168335	0.02724389	0.0585501	2	1	2
102466733	NR_106836	MIR6778	microRNA 6778	-2.463050374	-1.215104974	0.544657	0.170591	0.292789	0.07168335	0.02724389	0.0585501	2	1	2
102724862	NM_001291463,NM_001291463	TBC1D3I	TBC1 domain family member 3I	-2.463050374	-1.215104974	0.021165	0.008667	0.009682	0.07168335	0.02724389	0.0585501	2	1	2
106635524	NR_132773	SNORA91	small nucleolar RNA, H/ACA box	-2.463050374	-1.215104974	0.225553	0.011993	0.180114	0.07168335	0.02724389	0.0585501	2	1	2
106635541	NR_132789	SNORA105C	small nucleolar RNA, H/ACA box	-1.313695475	-1.215104974	0.285818	0.242594	0.237143	0.07168335	0.05398282	0.0585501	2	2	2
4975	NM_006189	OMP	olfactory marker protein	-1.313695475	-1.215104974	0.106347	0.056803	0.065163	0.07168335	0.05398282	0.0585501	2	2	2
723972	NR_003144	ANP32AP1	acidic nuclear phosphoprotein 32	-1.313695475	-1.215104974	0.043171	0.052069	0.04961	0.07168335	0.05398282	0.0585501	2	2	2
100126326	NR_030760	MIR936	microRNA 936	2.892476179	-1.215104974	0.392349	1.53062	0.37754	0.07168335	0.20477631	0.0585501	2	8	2
102466754	NR_106933	MIR6873	microRNA 6873	1.471633007	-1.215104974	0.670169	1.118244	0.527743	0.07168335	0.10601836	0.0585501	2	4	2
55327	NM_018362	LIN7C	lin-7 homolog C, crumbs cell pola	-1.229100942	-1.215108284	9.288671	7.565803	7.181795	5.48821674	5.19793813	5.21400945	1723	1873	1744
109951030	NR_146231	PKD1P3-NPIPA1	PKD1P3-NPIPA1 readthrough	1.177383565	-1.215416041	15.468839	18.231524	11.959979	6.8064101	7.04005655	6.52772592	4355	6851	4407
80063	NM_001256160,NM_001352120	ATF7IP2	activating transcription factor 7 in	-1.026844505	-1.215464335	4.850222	4.682232	3.613536	4.18329013	4.14719676	3.91874377	674	877	682
84264	NM_001290137,NM_001290139	HAGHL	hydroxyacylglutathione hydrolase	-1.315637415	-1.215741116	9.729631	7.453863	7.509651	3.82202563	3.45802105	3.56196863	516	524	522
118460	NM_058219	EXOSC6	exosome component 6	1.242088414	-1.215877316	15.992089	19.89963	12.363387	4.7808325	5.08336328	4.5100879	1040	1726	1052
1104	NM_001048194,NM_001048195	RCC1	regulator of chromosome conden	-1.064870936	-1.21592112	54.970522	52.057142	42.583991	7.15473716	7.06471344	6.87486578	5555	6970	5619
84282	NM_001184992,NM_032322,NM	RNF135	ring finger protein 135	-1.551795658	-1.216069627	15.286941	9.770684	11.723263	5.01477763	4.40518902	4.74213443	1230	1059	1244
7690	NM_001297548,NM_001330704	ZNF131	zinc finger protein 131	-1.026360627	-1.216205709	6.949432	6.618096	5.453366	4.5618359	4.52590309	4.29255782	888	1156	898
6232	NM_001030,NM_001349946,NM	RPS27	ribosomal protein S27	-1.258808436	-1.216467471	1075.319409	855.488592	820.541899	8.58106774	8.24998098	8.29918291	14996	15917	15162
6120	NM_001278282,NM_001278283	RPE	ribulose-5-phosphate-3-epimeras	1.274217958	-1.216523363	27.609637	35.258466	21.329843	6.47329746	6.81942046	6.19403493	3449	5872	3487
27257	NM_014462,NR_045492,NR_04	LSM1	LSM1 homolog, mRNA degradati	-1.030969951	-1.216562666	18.968123	18.502282	14.735804	4.45363476	4.41166458	4.18497252	821	1064	830
2161	NM_000505	F12	coagulation factor XII	-1.305247825	-1.216632809	7.016386	5.389709	5.418573	3.91021271	3.55480842	3.64789978	551	564	557
317749	NM_001193635,NM_001193636	DHRS4L2	dehydrogenase/reductase 4 like 2	1.046533666	-1.216642729	15.640326	16.443876	12.047682	4.46199571	4.52470876	4.19316234	826	1155	835
79161	NM_001329472,NM_001329473	TMEM243	transmembrane protein 243	-1.301849406	-1.216725153	13.138865	10.101875	10.248433	4.12761734	3.77168848	3.86234099	647	664	654
8764	NM_001297605,NM_003820	TNFRSF14	TNF receptor superfamily membe	-1.17256974	-1.216748939	6.923664	5.912085	5.333477	4.61828417	4.39868427	4.34789256	925	1054	935
100507634	NR_038320	LOC100507634	uncharacterized LOC100507634	-1.1132932	-1.216812049	1.206706	1.089874	0.930959	1.77389308	1.66595383	1.57922529	95	114	96
55119	NM_001349757,NM_001349758	PRPF38B	pre-mRNA processing factor 38B	1.093507678	-1.217042221	32.822652	35.860697	25.44875	7.29775691	7.42593811	7.01636074	6138	8968	6203
58500	NM_001109689,NM_021061	ZNF250	zinc finger protein 250	-1.1238626	-1.217064231	1.172826	1.042863	0.905057	3.05045588	2.90334193	2.80425801	286	340	289
64745	NM_001029991,NM_022734	METTL17	methyltransferase like 17	1.026143905	-1.217122252	52.017816	53.658134	40.540455	6.50115068	6.53797902	6.22112071	3517	4822	3554
60678	NM_021937	EEFSEC	eukaryotic elongation factor, sele	1.79533576	-1.217482182	6.902386	12.422047	5.331411	4.00022383	4.80408772	3.73574288	589	1413	595
8888	NM_003906	MCM3AP	minichromosome maintenance co	1.0127761	-1.217601707	21.692595	21.992876	16.739595	7.0424212	7.06059877	6.7607517	5136	6950	5188
23092	NM_001135608,NM_001349547	ARHGAP26	Rho GTPase activating protein 2(	1.053791416	-1.217629894	20.233577	21.120973	15.150691	7.4715883	7.54676379	7.18927546	6929	9756	6999
29063	NM_001318148,NM_024936	ZCCHC4	zinc finger CCHC-type containing	-1.06035716	-1.217660065	12.382241	11.774171	9.520728	5.08358236	5.00159078	4.80867573	1292	1628	1305
50861	NM_001276310,NM_015894,NR	STMN3	stathmin 3	-1.040516404	-1.218046795	13.176693	12.682637	10.015534	5.01704913	4.96154856	4.74213443	1232	1582	1244
23230	NM_001018037,NM_001018038	VPS13A	vacuolar protein sorting 13 homo	-1.095640853	-1.21823719	11.625989	10.594937	9.048483	6.94348794	6.81283053	6.66124136	4793	5845	4839
200186	NM_181715	CRTC2	CREB regulated transcription coa	-1.137265309	-1.218420702	25.865273	22.766071	19.948618	6.09692582	5.91423939	5.81649676	2648	3111	2673
127253	NM_001162916,NM_138467,NR	TYW3	tRNA-yW synthesizing protein 3 h	-1.171378959	-1.219028636	10.240296	8.749076	7.906247	5.13886234	4.91763587	4.86204569	1344	1533	1356
6159	NM_000992	RPL29	ribosomal protein L29	-1.153183747	-1.21909623	736.736389	639.596191	567.907593	8.96447371	8.75929243	8.67929251	19573	22678	19747
5394	NM_001001998,NM_002685	EXOSC10	exosome component 10	1.03405446	-1.219125496	50.937367	52.734914	39.275761	7.14801643	7.19599448	6.86439066	5529	7639	5578
9284	NM_006985	NPIPA1	nuclear pore complex interacting	1.035174247	-1.21916892	13.665239	14.172649	10.538432	3.95355025	4.00026496	3.68786049	569	787	574
79568	NM_024520	MAIP1	matrix AAA peptidase interacting	1.141815286	-1.219265328	15.866886	18.139748	12.22739	4.6062649	4.79023891	4.33314528	917	1399	925
56851	NM_020154	EMC7	ER membrane protein complex s	-1.039773425	-1.219362305	106.075516	102.124504	81.747345	6.77823001	6.72248209	6.49497534	4270	5487	4307
92745	NM_033518	SLC38A5	solute carrier family 38 member 5	1.164866517	-1.21938093	28.801594	33.591637	22.194283	5.83252285	6.04910966	5.55190154	2198	3421	2217
8761	NM_001135653,NM_001135654	PABPC4	poly(A) binding protein cytoplasm	-1.072361446	-1.219446273	63.288986	59.030473	48.556765	7.61850462	7.51824305	7.33388396	7676	9564	7742
54741	NM_001198681,NM_001198683	LEPROT	leptin receptor overlapping transc	-1.212074138	-1.219455672	17.202745	14.672905	13.800876	6.23212092	5.9586963	5.95007726	2912	3210	2937
114915	NR_015370	EPB41L4A-AS1	EPB41L4A antisense RNA 1	-1.127250844	-1.219591641	25.887175	22.984936	19.94376	4.95916039	4.79222547	4.68288194	1182	1401	1192
654364	NM_001018136,NR_037149	NME1-NME2	NME1-NME2 readthrough	-1.272691453	-1.219605648	2.515673	2.106188	2.066028	2.02927762	1.77444288	1.81825156	121	127	122
4072	NM_002354	EPCAM	epithelial cell adhesion molecule	-1.10898671	-1.219682813	715.604614	645.9953	551.34613	10.22639924	10.07728805	9.94015701	46994	56619	47389
83939	NM_001319043,NM_001319044	EIF2A	eukaryotic translation initiation fa	1.022674896	-1.219747189	42.533197	43.514087	32.75989	7.37284005	7.40499516	7.08816389	6468	8838	6522
474384	NM_001007524,NM_001007524	F8A3	coagulation factor VIII-associatec	-1.320831011	-1.219831182	0.061959	0.040781	0.045139	0.10623215	0.08023518	0.08695753	3	3	3
137835	NM_001145153,NM_144649	TMEM71	transmembrane protein 71	-1.002019576	-1.219831182	0.025143	0.037677	0.01921	0.10623215	0.10601836	0.08695753	3	4	3
53358	NM_016848	SHC3	SHC adaptor protein 3	3.64772358	-1.219831182	0.005357	0.028829	0.005876	0.10623215	0.36283659	0.08695753	3	15	3
474383	NM_001007523,NM_001007523	F8A2	coagulation factor VIII-associatec	-1.320831011	-1.219831182	0.060654	0.040372	0.044072	0.10623215	0.08023518	0.08695753	3	3	3
100287846	NR_037168	LOC100287846	patched 1 pseudogene	2.684182094	-1.219831182	0.014907	0.059715	0.01325	0.10623215	0.27464693	0.08695753	3	11	3
3117	NM_002122	HLA-DQA1	major histocompatibility complex,	3.165952837	-1.219831182	0.050309	0.155625	0.032406	0.10623215	0.31941552	0.08695753	3	13	3
641451	NR_002917	SNORA19	small nucleolar RNA, H/ACA box	1.479755237	-1.219831182	0.417314	0.833292	0.408373	0.10623215	0.15624221	0.08695753	3	6	3
100616439	NR_039802	MIR4658	microRNA 4658	1.479755237	-1.219831182	1.167015	1.666096	0.780953	0.10623215	0.15624221	0.08695753	3	6	3
102465522	NR_106925	MIR6865	microRNA 6865	-3.632026599	-1.219831182	0.864737	0.194075	0.910357	0.10623215	0.02724389	0.08695753	3	1	3
51078	NM_001164356,NM_015963	THAP4	THAP domain containing 4	1.20640471	-1.219873198	26.814926	30.58343	20.076738	5.74341713	6.00952888	5.46257813	2064	3327	2081

90019	NM_001290332,NM_001290333	SYT8	synaptotagmin 8	1.620336579	-1.219989056	12.274432	19.982327	9.466441	4.30911424	4.97734988	4.03812306	739	1600	745
5411	NM_002687	PNN	pinin, desmosome associated pro	1.345165751	-1.220088558	205.879715	277.245148	158.56601	9.49948537	9.92675919	9.21293686	28378	51004	28607
54103	NM_001350896,NM_001350897	GSAP	gamma-secretase activating prot	-2.59071147	-1.220147351	11.955767	4.573325	9.42947	5.29208934	3.97600158	5.01309432	1499	773	1511
55073	NR_002940	LRRC37A4P	leucine rich repeat containing 37	-1.589777097	-1.220287337	3.667218	2.309245	2.82394	4.74702075	4.10945474	4.47155306	1015	853	1023
9939	NM_005105	RBM8A	RNA binding motif protein 8A	1.075826083	-1.220310174	31.451727	33.871136	24.219286	7.25605529	7.36083661	6.97087928	5962	8570	6009
80380	NM_025239	PDCD1LG2	programmed cell death 1 ligand 2	-1.564101338	-1.220314572	1.40568	0.897017	1.081513	2.10810423	1.63971536	1.89255394	130	111	131
79070	NM_001318732,NM_024089	KDELC1	KDEL motif containing 1	1.136988259	-1.220430462	3.213181	3.663531	2.478398	2.93026199	3.09255667	2.68388473	260	395	262
84450	NM_001271286,NM_001271287	ZNF512	zinc finger protein 512	1.226663445	-1.22047806	14.173718	17.479946	10.91615	5.65502199	5.94447568	5.37385651	1939	3178	1954
54952	NM_017846,NR_003109	TRNAU1AP	tRNA selenocysteine 1 associate	-1.208046926	-1.220881561	16.474974	13.672898	12.874274	4.99992509	4.73657294	4.72189817	1217	1346	1226
339803	NR_036496	LOC339803	uncharacterized LOC339803	1.412227589	-1.221121128	1.443314	2.04838	1.116836	2.20692365	2.61093762	1.98602166	142	268	143
55765	NM_001142569,NM_018265	C1orf106	chromosome 1 open reading fran	-2.694317819	-1.221142947	11.313008	4.177814	8.661829	5.55695766	4.17791132	5.27546743	1809	897	1822
100128191	NR_027157	TMPO-AS1	TMPO antisense RNA 1	-1.670578972	-1.221156639	5.779988	3.46358	4.449977	4.23299104	3.54305919	3.96156336	699	559	704
29101	NM_014188	SSU72	SSU72 homolog, RNA polymeras	-1.117613789	-1.22151452	67.055428	60.056599	51.579487	6.42749724	6.26904015	6.14252373	3340	3993	3363
64318	NM_022451	NOC3L	NOC3 like DNA replication regula	-1.161689285	-1.221517666	13.069734	11.261211	10.054576	5.50611581	5.29500126	5.22443598	1745	2007	1757
5930	NM_006910,NM_018703,NM_03	RBBP6	RB binding protein 6, ubiquitin lig	-1.00696406	-1.22167351	29.3972	29.626645	21.444505	7.38212176	7.37216962	7.09517335	6510	8638	6554
11072	NM_007026	DUSP14	dual specificity phosphatase 14	-1.215523799	-1.221740057	41.436825	34.129158	31.876062	5.95071282	5.67413949	5.66692369	2389	2626	2405
[truncated: 1,040,973 more chars]
