# Supplementary material for: Direct cell-to-cell transfer in stressed tumor microenvironment aggravates tumorigenic or metastatic potential in pancreatic cancer
Source: NPJ Genom Med. 2022 Oct 27;7:63. doi: 10.1038/s41525-022-00333-w (PMC9613679; doi:10.1038/s41525-022-00333-w)
Supplement: Supplementary file 2 — Reporting Summary [file 41525_2022_333_MOESM2_ESM.pdf]

nature portfolio

Corresponding author(s): Sung-Yup Cho and Charles Lee

Last updated by author(s): May 5, 2022

Reporting Summary

Nature Portfolio wishes to improve the reproducibility of the work that we publish. This form provides structure for consistency and transparency in reporting. For further information on Nature Portfolio policies, see our Editorial Policies and the Editorial Policy Checklist.

Statistics

For all statistical analyses, confirm that the following items are present in the figure legend, table legend, main text, or Methods section.

n/a	Confirmed	
☐	☒	The exact sample size (*n*) for each experimental group/condition, given as a discrete number and unit of measurement
☐	☒	A statement on whether measurements were taken from distinct samples or whether the same sample was measured repeatedly
☐	☒	The statistical test(s) used AND whether they are one- or two-sided<br>*Only common tests should be described solely by name; describe more complex techniques in the Methods section.*
☒	☐	A description of all covariates tested
☐	☒	A description of any assumptions or corrections, such as tests of normality and adjustment for multiple comparisons
☐	☒	A full description of the statistical parameters including central tendency (e.g. means) or other basic estimates (e.g. regression coefficient) AND variation (e.g. standard deviation) or associated estimates of uncertainty (e.g. confidence intervals)
☐	☒	For null hypothesis testing, the test statistic (e.g. *F*, *t*, *r*) with confidence intervals, effect sizes, degrees of freedom and *P* value noted<br>*Give P values as exact values whenever suitable.*
☒	☐	For Bayesian analysis, information on the choice of priors and Markov chain Monte Carlo settings
☐	☒	For hierarchical and complex designs, identification of the appropriate level for tests and full reporting of outcomes
☒	☐	Estimates of effect sizes (e.g. Cohen's *d*, Pearson's *r*), indicating how they were calculated

Our web collection on statistics for biologists contains articles on many of the points above.

Software and code

Policy information about availability of computer code

Data collection	FACS data : BD FACSDiva software v8.0.2<br>QPCR : CFX96 (Bio-Rad)<br>Illumina sequencing : Illumina NovaSeq 6000 for RNA sequencing, Illumina HiSeq X sequencer for Whole Genome Sequencing<br>Transcript assembly : StringTie program<br>Confocal microscopy : LSM 880 AiryScan<br>Short tandem repeat (STR) analysis : 3530xL DNA analyzer (Applied Biosystems)<br>Aldefluor assay : LSRFortessa cell analyzer (BD Biosciences)<br>Nano LC-MS/MS : Easy n-LC (Thermo Fisher Scientific), LTQ Orbitrap XL mass spectrometer (Thermo Fisher Scientific)
Data analysis	Short tandem repeat (STR) analysis : GeneMapper v5 software (Applied Biosystems)<br>Image analysis : ImageJ<br>Statistical calculation : Prism 8.0.1 (GraphPad)<br>Difference between two variables : Student’s t test (unpaired)<br>Difference between multiple variables : ANOVA with Tukey's or Dunnett's multiple comparison test<br>Fusion genes : SOAPfuse, Defuse, FusionCatcher and STAR-Fusion<br>Whole genome sequencing : IVC (Isaac Variant Caller 2.0.13), SnpEff 3.3, Control-FREEC 6.4, Manta 0.20.2 and Breakdancer<br>Gene set enrichment analysis (GSEA) : javaGSEA desktop application (GSEA v4.0.3)<br>Peptide sequence : Mascot algorithm (Matrixscience)<br>Protein-protein network analysis : STRING database

For manuscripts utilizing custom algorithms or software that are central to the research but not yet described in published literature, software must be made available to editors and reviewers. We strongly encourage code deposition in a community repository (e.g. GitHub). See the Nature Portfolio guidelines for submitting code & software for further information.

Data

Policy information about availability of data

All manuscripts must include a data availability statement. This statement should provide the following information, where applicable:

- Accession codes, unique identifiers, or web links for publicly available datasets
- A description of any restrictions on data availability
- For clinical datasets or third party data, please ensure that the statement adheres to our policy

Sequencing data (.fastq.gz files) are available from the Gene Expression Omnibus (accession number GSE201876).

Field-specific reporting

Please select the one below that is the best fit for your research. If you are not sure, read the appropriate sections before making your selection.

☒ Life sciences ☐ Behavioural & social sciences ☐ Ecological, evolutionary & environmental sciences

For a reference copy of the document with all sections, see nature.com/documents/nr-reporting-summary-flat.pdf

Life sciences study design

All studies must disclose on these points even when the disclosure is negative.

Sample size	GSEA was performed using data from the TCGA Pancreatic Cancer (PAAD) database (n = 182, http://xena.ucsc.edu/).<br>For Kaplan-Meier plot for overall survival of the pancreatic cancer patients (TCGA Pancreatic Cancer (PAAD) database (n = 182, http://xena.ucsc.edu/) or GSE84219 from Gene Expression Omnibus (n = 30, https://www.ncbi.nlm.nih.gov/geo/)), each was divided into two groups (median cutoff) with either high or low expression of CD24 (left), CD44 (middle), or MCT1 (right).
Data exclusions	no data exclusions
Replication	Experimental findings were reliably reproduced as described in manuscript and figure legends.
Randomization	Randomization was not performed.
Blinding	Blinding was not performed.

Reporting for specific materials, systems and methods

We require information from authors about some types of materials, experimental systems and methods used in many studies. Here, indicate whether each material, system or method listed is relevant to your study. If you are not sure if a list item applies to your research, read the appropriate section before selecting a response.

Materials & experimental systems

n/a	Involved in the study	
☐	☒	Antibodies
☐	☒	Eukaryotic cell lines
☒	☐	Palaeontology and archaeology
☒	☐	Animals and other organisms
☒	☐	Human research participants
☒	☐	Clinical data
☒	☐	Dual use research of concern

Methods

n/a	Involved in the study	
☒	☐	ChIP-seq
☐	☒	Flow cytometry
☒	☐	MRI-based neuroimaging

Antibodies

Antibodies used	anti-human CD24 monoclonal antibody (clone ML5, BioLegend, USA), anti-human CD44 monoclonal antibody (clone BJ18, BioLegend, USA), anti-MCT1 polyclonal antibody (Immunogen : MCT1 fusion protein Ag14098, Proteintech, USA)
Validation	For Western Blotting :<br>Anti-human CD24 monoclonal antibody (clone ML5, BioLegend, USA) : https://www.biolegend.com/it-it/products/purified-anti-human-cd24-antibody-1806<br>Anti-human CD44 monoclonal antibody (clone BJ18, BioLegend, USA) : https://www.biolegend.com/fr-fr/products/purified-anti-human-cd44-antibody-5581<br>Anti-MCT1 polyclonal antibody (Immunogen : MCT1 fusion protein Ag14098, Proteintech, USA) : https://www.ptglab.com/products/MCT1-Antibody-20139-1-AP.htm

For flow cytometry :
PE anti-human CD24 monoclonal antibody (clone ML5, BioLegend, USA) : biolegend.com/nl-be/products/pe-anti-human-cd24-antibody-1805
APC anti-human CD44 monoclonal antibody (clone BJ18, BioLegend, USA) : https://www.biolegend.com/de-at/products/apc-anti-human-cd44-antibody-5583

Eukaryotic cell lines

Policy information about cell lines

Cell line source(s)	U937, Panc0203, Panc0327, Human pancreatic fibroblasts, Human pancreatic cancer-associated fibroblasts (CAFs)
Authentication	U937 : ATCC (CRL-1593.2)<br>Panc0203 : ATCC (CRL-2553)<br>Panc0327 : ATCC (CRL-2549)<br>Human pancreatic fibroblasts : Neuromics (SC00A5)<br>Human pancreatic CAFs : Neuromics (CAF08)
Mycoplasma contamination	U937, Panc0203, PanC0327, fibroblasts and CAFs cells were negative for mycoplasma contamination.
Commonly misidentified lines (See ICLAC register)	Not applicable

Flow Cytometry

Plots

Confirm that:

☒ The axis labels state the marker and fluorochrome used (e.g. CD4-FITC).

☒ The axis scales are clearly visible. Include numbers along axes only for bottom left plot of group (a 'group' is an analysis of identical markers).

☒ All plots are contour plots with outliers or pseudocolor plots.

☒ A numerical value for number of cells or percentage (with statistics) is provided.

Methodology

Sample preparation	CMTMR-stained pancreatic cancer cells were co-cultured with CMFDA-labled macrophages, then, to isolate DFC (CMFDA- and CMTMR-positive) populations, FACS was performed using a FACSAria IIu cell sorter (BD Biosciences) according to the manufacturer's instructions.
Instrument	FACSAria IIu cell sorter (BD Biosciences)
Software	BD FACSDiva software v8.0.2 was used for data collection.
Cell population abundance	N/A
Gating strategy	1) SSC vs. FSC gating to exclude debris. 2) CMTMR & CMFDA double fluorescence positive cells (DFCs) gating to exclude single fluorescence positive cells. and to quantify DFCs.

☒ Tick this box to confirm that a figure exemplifying the gating strategy is provided in the Supplementary Information.
